# Transcriptional and Functional Profiling of Human Embryonic Stem Cell-Derived Cardiomyocytes

**DOI:** 10.1371/journal.pone.0003474

**Published:** 2008-10-22

**Authors:** Feng Cao, Roger A. Wagner, Kitchener D. Wilson, Xiaoyan Xie, Ji-Dong Fu, Micha Drukker, Andrew Lee, Ronald A. Li, Sanjiv S. Gambhir, Irving L. Weissman, Robert C. Robbins, Joseph C. Wu

**Affiliations:** 1 Department of Radiology, Stanford University School of Medicine, Stanford, California, United States of America; 2 Department of Medicine (Division of Cardiology), Stanford University School of Medicine, Stanford, California, United States of America; 3 Department of Bioengineering, Stanford University School of Medicine, Stanford, California, United States of America; 4 Department of Developmental Biology, Stanford University School of Medicine, Stanford, California, United States of America; 5 Department of Cardiothoracic Surgery, Stanford University School of Medicine, Stanford, California, United States of America; 6 Stem Cell Program and Department of Cell Biology and Human Anatomy, University of California Davis, Davis, California, United States of America; California Institute for Regenerative Medicine, United States of America

## Abstract

Human embryonic stem cells (hESCs) can serve as a potentially limitless source of cells that may enable regeneration of diseased tissue and organs. Here we investigate the use of human embryonic stem cell-derived cardiomyocytes (hESC-CMs) in promoting recovery from cardiac ischemia reperfusion injury in a mouse model. Using microarrays, we have described the hESC-CM transcriptome within the spectrum of changes that occur between undifferentiated hESCs and fetal heart cells. The hESC-CMs expressed cardiomyocyte genes at levels similar to those found in 20-week fetal heart cells, making this population a good source of potential replacement cells *in vivo*. Echocardiographic studies showed significant improvement in heart function by 8 weeks after transplantation. Finally, we demonstrate long-term engraftment of hESC-CMs by using molecular imaging to track cellular localization, survival, and proliferation *in vivo*. Taken together, global gene expression profiling of hESC differentiation enables a systems-based analysis of the biological processes, networks, and genes that drive hESC fate decisions, and studies such as this will serve as the foundation for future clinical applications of stem cell therapies.

## Introduction

Myocardial infarction is a major cause of morbidity and mortality worldwide. The limited ability of the surviving cardiac cells to proliferate following an ischemic attack renders the damaged heart susceptible to unfavorable remodeling processes and heart failure [1]. Currently, pharmaceutical and implantable device management of heart failure seek only to preserve existing viable myocardium after an ischemic attack, and thus merely slows the progression of cardiac dysfunction. Ultimately, heart transplantation is the only viable treatment option for end-stage heart failure patients. To “regenerate” the heart and not only preserve cardiac function but also recover lost or diseased muscle, stem cell therapy has emerged as a promising therapy for heart disease because it can provide a virtually unlimited source of cardiomyocytes, endothelial cells, and other differentiated cell types. The hope is to use these cells to replace diseased myocardium that would otherwise progress to outright failure and regenerate the heart to its former, healthy self.

Recently, human embryonic stem cells (hESCs) have generated much interest because of their capacity for self-renewal and pluripotency. In practical terms, hESCs can be cultured indefinitely *ex vivo*, and can differentiate into virtually any cell type in the adult body [2], [3]. hESCs are thus an attractive source for the derivation of large numbers of cells to be used in various tissue repair and cell replacement therapies. However, upon transplantation into living organisms, undifferentiated hESCs can spontaneously differentiate into rapidly proliferating teratomas, which are disordered amalgams of all three germs layers [2], [3]. Nevertheless, under the appropriate conditions, *ex vivo* hESCs can be directed to differentiate into beating cardiomyocytes via an embryoid body (EB) intermediate [4]. Subsequently, the cardiomyocyte sub-population is enriched several-fold using discontinuous density gradient separation [5]. Therefore, coaxing hESCs into cardiomyocytes for therapeutic applications is an innovative and feasible strategy that can minimize the risk of cellular misbehavior and teratoma formation [6].

In order to define at a molecular level the changes occurring at each stage of hESC differentiation into cardiomyocytes, we performed transcriptional profiling of the cells using whole human genome microarrays. This allowed us to examine the activation of specific genes as well as broader developmental processes during the progression from hESC to fetal cardiomyocyte, and to identify novel genes that are potentially important in mediating differentiation and development as well as potential novel markers of each stage. In the future, such genes may prove vital in efforts to more closely direct and assess differentiation of potential therapeutic pre-cardiomyocytes or cardiomyocytes in the repair of injured cardiac tissues. To monitor cell survival after transplantation, we then employ molecular imaging techniques that allow repetitive, noninvasive assessment of transplanted ES cell engraftment, viability, and proliferation in small animal models. Using these genomic and imaging tools, we investigate the molecular networks governing our differentiating cardiomyocytes, with an eye toward transplantation and assessment of cell survival and proliferation *in vivo* in a myocardial ischemia reperfusion model.

## Results

### Differentiation of hESCs to cardiomyocytes

**Figure 1:**
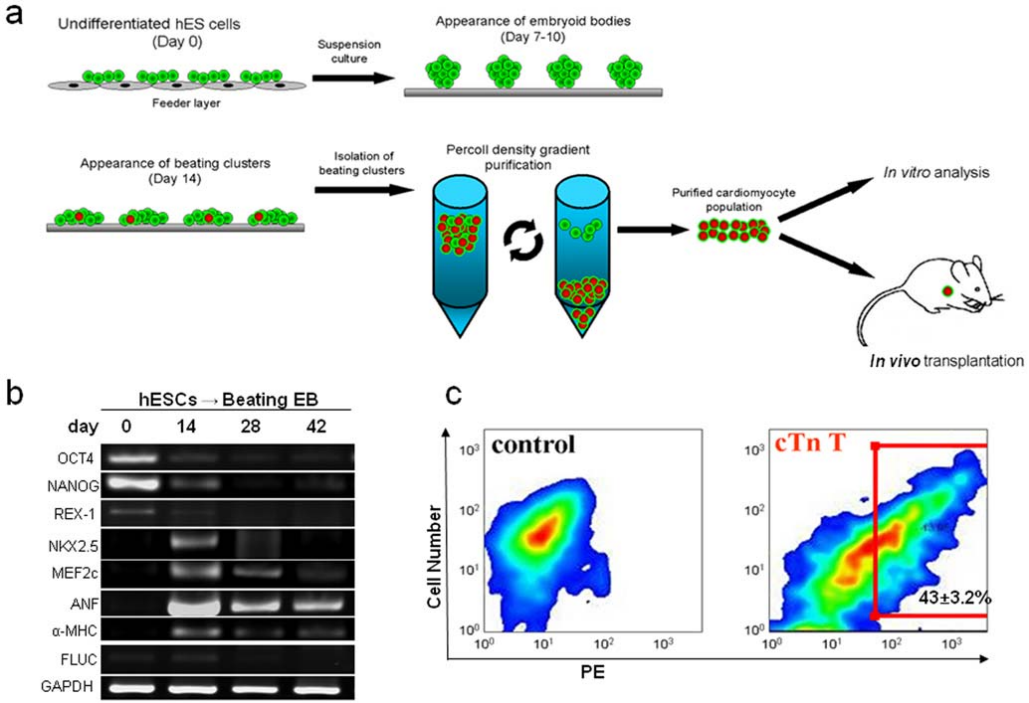
Differentiation of hESCs to cardiomyocytes that express lineage-specific genes. (a) Schematic highlighting the experimental design. hESCs are maintained in an undifferentiated state on mouse embryonic fibroblasts (MEFs), then transferred to suspension culture and allowed to form embryoid bodies for 7 to 10 days. Upon appearance of beating clusters, the whole embryoid bodies are dissociated and separated by Percoll density gradient enrichment to obtain cardiomyocytes (hESC-CMs). The hESC-CMs are then used for either *in vitro* analysis or *in vivo* transplantation into the heart. (b) RT-PCR analysis of embryoid bodies over the course of 6 weeks shows expression of endodermal, (AFP), mesodermal (αMHC), and endodermal (NeuroD) germ layer markers. GAPDH is used as loading control. (c) FACS analysis shows approximately 43.0±3.2% cells isolated by Percoll density gradient separation are positive for cardiac troponin-T. Control population is cells prior to Percoll separation.

We differentiated hESCs into cardiomyocytes as shown in **Figure 1a**. To understand the time course of transcriptional changes occurring in these cells, we performed RT-PCR analysis of hESC-derived EBs as they differentiated over the course of 42 days into beating clusters (**Figure 1b**). Expression of stem cell markers (Oct4, NANOG, Rex1) decreased substantially by day 28, while early stage cardiac transcriptional factors (Nkx2.5, MEF2C) appeared between days 14–28. As expected, cardiac specific markers (αMHC, ANF) appeared by day 14 and persisted through terminal differentiation into beating EBs. Before enrichment, only 2–5% of the cells within beating EBs expressed cardiac marker troponin-T as determined by FACS analysis. However, by utilizing Percoll density gradient separation, we were able to achieve cardiomyocyte-enriched populations ranging from 40–45%, a ten-fold increase (**Fig 1c** **&** **Movies S1**, **S2** and **S3**).

### Major changes in gene expression between stages highlight developmental progression

**Figure 2:**
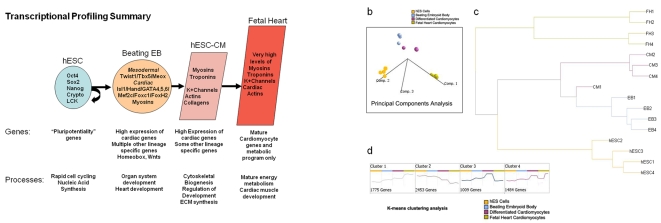
Major themes in gene expression profiles at each stage of differentiation. (a) hESCs express high levels of pluripotency-associated genes including Oct4, Sox2, NANOG, Crypto, and LCK. At the beating EB stage, the cells express high levels of mesodermal master regulators such as Twist1, Tbx5, and Meox as well as very enriched levels of cardiogenic specific master regulators including Isl1, Hand, GATA4, 5, and 6, and MEF2C, along with high levels of cardiac specific myosins. This population also expresses genes from other cell layers, and many developmental genes from Wnt and homeobox families. Cardiomyocytes downregulate early mesodermal genes and express more cardiac specific and structural genes, while fetal heart cells have the highest levels of mature cardiac gene expression with very few other developmental lineages represented. (b) Principal Components Analysis (PCA) shows that replicate experiments of each cell type are very similar while differentiation groups separate significantly along components 1 and 2. (c) Hierarchical Clustering Analysis - Cells from each developmental stage cluster relatively close to each other, with the most distance between hESCs and fetal heart cardiomyocytes. (d) K-means clustering analysis identifies major trends in gene expression across the timecourse.

cRNA derived from four independent biological replicates at the three stages of differentiation, and from cells isolated from four individual human fetal hearts (19, 19, 20, and 21 weeks), was hybridized into individual whole human genome microarrays. Because fetal and adult hearts are composed of numerous cell types, including cardiomyocytes, endothelial cells, smooth muscle, fibroblasts, and many others, we isolated only primary ventricular cardiomyocytes for microarray analysis (see **Methods S1**). Doing so prevented non-cardiac cell types from contaminating our gene expression data. The resulting data were analyzed using the SAM algorithm [7] to identify genes which had changed expression significantly between stages. A summary of our major findings is shown in **Figure 2a**. To obtain an overview of the transcriptional landscape, we looked at the data using principal components analysis (PCA), a dimensional reduction technique which identifies “principal components” or major trends in gene expression in the overall data (**Figure 2b**). PCA demonstrates that each of the four replicates from each stage has very similar transcriptional profiles to one another, but distinctly different between stages, as expected. “Adjacent” stages show a progression of gene expression changes primarily along component one, a pattern of continuously decreasing gene expression across time, a pattern that we also identified as prominent in clustering analyses. A hierarchical clustering overview of the microarray experiments as a whole (**Figure 2c**) likewise shows that the overall gene expressions among replicates of each stage are very similar, with progressive differences between more distantly separated stages.

### hESCs exhibit unique biologic processes and molecular signature

To better understand which cellular processes are important in the undifferentiated hESC stage, we performed statistical Gene Ontology (GO) biological process overrepresentation analysis and found that the most highly upregulated processes involved almost exclusively cell cycling and mitosis, as well as nucleic acid synthesis and metabolism (**Table S2 (A7)**). This was not surprising given that hESCs' primary mission is to self renew. hESCs are also characterized by a network of genes important for pluripotency, including the unique homeobox transcription factor NANOG, which is the main downstream effector of this network. When we compared expression patterns in hESCs to EB cells, we found that there were 2,219 genes expressed much more highly in hESCs. The most dramatically elevated transcript in hESCs was NANOG, which is expressed at a level 250 times higher in hESCs than in EB [8], [9] (**Table S1 (A1)**). POU5F1 (also known as Oct4), upstream of NANOG, is one of the critical regulators of pluripotency in the mammalian embryo, and our results show that it is expressed 106–160 fold more highly in the undifferentiated hESCs when compared to EB. SOX2, another key pluripotency gene, is expressed at 7.4 times the level in hESCs as in EB [10]. Other known markers of ES cell status are also clearly present at high levels: TDGF1 and 3 (Crypto1 and 3) [11], expressed at ∼100 fold higher levels in hESCs; the SRC family kinase LCK (40 fold higher), whose repression is associated with ES differentiation [12]; the ES cell markers such as developmental pluripotency-associated 4 (Dppa4) (15.7 fold) [13] and homeobox expressed in ES cells 1 (Hesx1, 10 fold) [14]. Further discussion can be found in **Supplemental Results S1**.

### Beating EB cells express many mesodermal and cardiac specific gene programs

Differentiation to the beating EB stage is a very exciting and complex time in the life of the cell population. Our microarray results showed significant upregulation of master cardiac transcriptional regulators, as well as cardiac-specific structural and functional genes (**Figure 2a**, also see **Supplemental Results S1**). Although this population of cells had clearly differentiated with a significant bias toward mesodermal and cardiac lineages, we could still see expression of genes characteristic of all three cell layers. Analysis of the biological processes in the beating EBs confirms these observations, with overexpression of embryonic and organ system developmental categories including nervous system development, kidney development, skeletal muscle development, and heart development (**Table S2 (A8)**).

### hESC-CMs downregulate early mesodermal genes and upregulate cardiovascular and structural genes

Differentiation of beating EBs to cardiomyocytes is marked by a transcriptional downregulation of 2,389 genes, including early mesodermal genes such as TWIST1, whose expression goes down substantially by 5 fold between the EB and CM stages, and MEOX2, with a 15 fold reduction (**Table S1 (A3)**). There are also considerable reductions in the expression of a number of homeobox genes (HOXB3, 4, IRX, HHEX, HESX1). At the same time, we observe substantial increases in expression of 1,012 genes, including cardiac structural genes such as tropomyosin 1 and 2 (TPM1, 2, ∼3 fold upregulated) [15], the heart and muscle gene LMO7 (3.5 fold) [16], and a number of actins and actin-regulatory genes such as beta actin (ACTNB, 3.4 fold), alpha actinin (5 fold), coronin (2.5 fold), transgelin (4.6 fold) [17], and caldesmon 1 (5 fold) [18] (**Table S1 (A4)**). Cells in this population also exhibit evidence of maturation with the increased expression of extracellular structural components such as vascular collagens COL8A1 (18 fold) [19], COL4A3 and 4 (11 fold and 16 fold) [20], COL6A3 (4 fold) [21], and COL2A1 (2.4 fold) [22]. Nevertheless, there is still transcriptional evidence for the presence of other mesodermal derivatives such as hematopoietic derivatives with the increased expression of IL1A (10–20 fold), toll like receptor 3 (TLR 3, 3 fold), skeletogenic genes such as RUNX1 (4 fold), sclerostosis (SOST, 8.7 fold), osteoprotogerin (TNFSFR11, 7 fold). Some neuroectodermal genes are also upregulated, including neuralized (NEURL, 2.7 fold) and neurofilament light peptide (NEFL, 2.3 fold).

### Significant changes in energy metabolism between CM and fetal heart cardiomyocytes

One of our goals in this study is to compare our CM population to a cell population that would likely be optimal for cell transplantation into the damaged heart. An optimal cell type would be committed to the cardiomyocyte lineage but would still retain the capacity to undergo mitosis and thereby regenerate damaged heart muscle. We therefore chose primary fetal heart (FH) cardiomyocytes as the gold standard for comparison since they retain some proliferative capacity while also maintaining a cardiac phenotype [23]. In general, we found that expression of cardiac structural and force generating protein genes in the FH cells was not significantly higher than in the CM populations. This suggests that the CM population, while still somewhat heterogeneous, is composed of differentiated cardiomyocytes that are capable of contraction but have not yet faced the biomechanical stresses *in vivo* required for cardiac development. This is further corroborated when we looked at the GO processes that are more active in the FH cells and found a pattern suggesting increased metabolic activity but not structural protein biogenesis (**Table S2 (A11 and A12)**). Specifically, many of the increased processes in FH cells include the TCA cycle, cellular respiration, mitochondrial biogenesis, and lipid metabolism. These energy-related pathways are necessary for the mature cardiomyocyte to contract forcefully, and their expression timing may correlate with the necessity for active cardiac contraction in the fetus that continues up to and beyond birth, with an interesting shift to a lipid-based metabolism in the postnatal heart.

### Electrophysiological recordings of hESC-CMs

**Figure 3:**
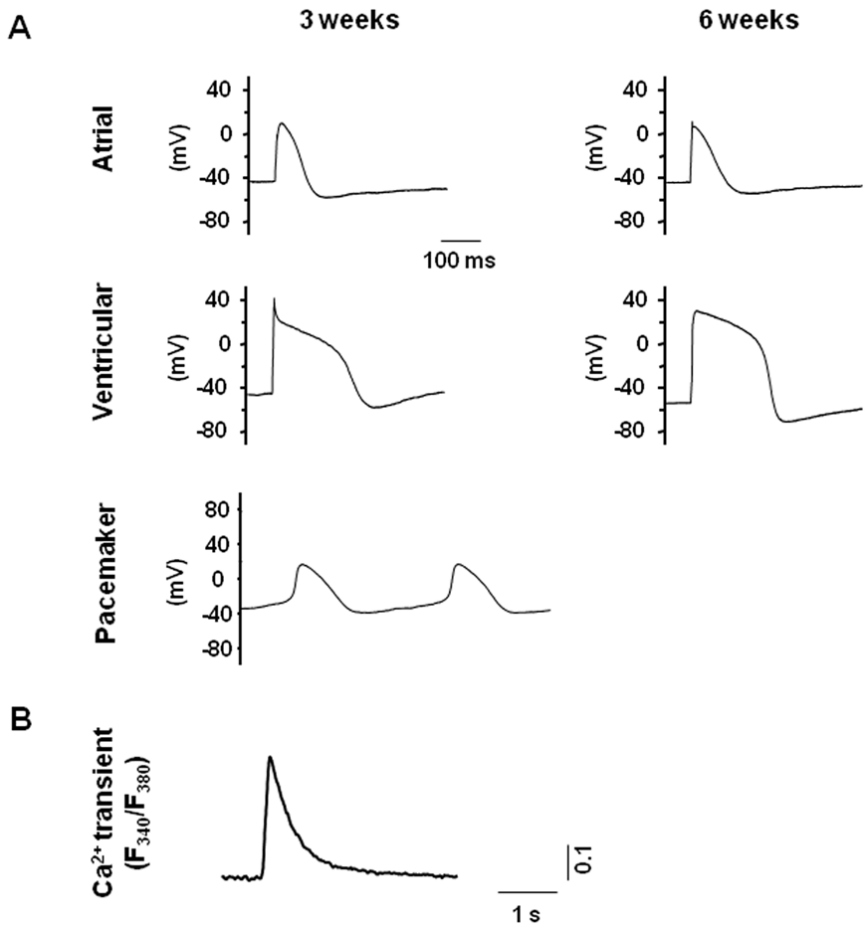
Electrophysiological recordings of hESC-CMs. Action potential (AP) recordings from single cells were done using the whole-cell patch-clamp technique. hESC-CMs were categorized into pacemaker-, atrial- or ventricular-like phenotypes, based on such common electrophysiological characteristics as the AP amplitude (mV), upstroke velocity (mV/ms), APD50 and APD90 (ms), as well as the resting membrane potential (RMP, mV). (a) Representative ventricular-, atrial- and pacemaker-like action potentials, demonstrating electrophysiological heterogeneity in our hESC-CM population, and (b) Ca2+ transients recorded from hESC-derived cardiomyocytes, confirming calcium influx of these cells. See Materials and Methods for description of experimental parameters.

Previous groups have studied the electrophysiology of hESC-CMs and have reported significant heterogeneity within the population [24], [25], [26]. To understand the electrophysiological properties of our hESC-CMs, we took action potential (AP) recordings from single cells using whole-cell patch-clamps. hESC-CMs were categorized into pacemaker-, atrial-, or ventricular-like phenotypes, based on such common electrophysiological characteristics as the AP amplitude, upstroke velocity, as well as the resting membrane potential. Ca^2+^ transients that are crucial for excitation-contraction coupling were also recorded. At 3 and 6 weeks post-differentiation, ventricular-, atrial-, and pacemaker-like derivatives were readily observed (**Figure 3**). Noticeably, ventricular-like hESC-CMs were most similar to fetal rather than adult ventricular cells as indicated by their depolarized resting membrane potential. Nonetheless, the AP profiles did not appear to change significantly over the time course of our experiments. Ideally, we would like to isolate only the ventricular-like hESC-CMs and use those cells for transplantation studies in ischemic left ventricles. Given the lack of specific cellular markers for identifying ventricular/atrial/pacemaker CM types, we were limited to using the whole population for transplantation studies.

### hESC-CM transplantation improves left ventricular function in a mouse myocardial infarction model

**Figure 4:**
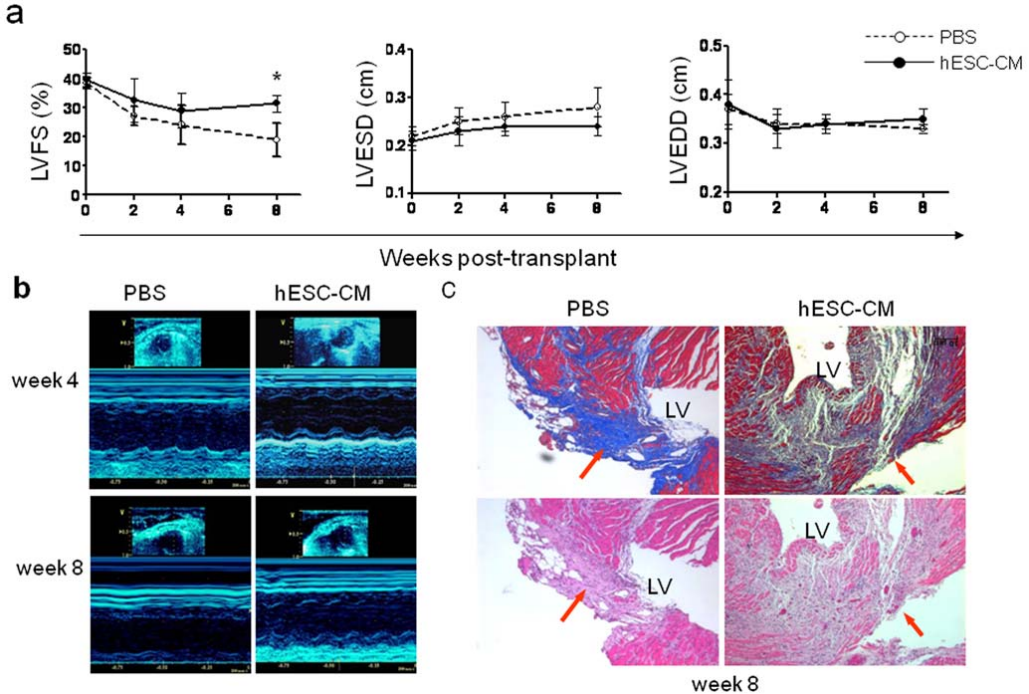
Assessment of myocardial function after ischemic injury and hESC-CM transplantation. (a) Echocardiography demonstrates improved cardiac contractility (left ventricular fractional shortening, LVFS) following delivery of one million hESC-CMs compared to PBS injection alone at eight weeks post-transplant (*P* = 0.03). This was primarily due to improvement in left ventricular end systolic dimension (LVESD, middle) without significant changes in left ventricular end diastolic dimension (LVEDD, right). (b) Representative M-mode echocardiographic images from a mouse receiving hESC-CM transplantation (right panels) versus a mouse receiving a control PBS injection (left panels) at 4 (top panels) and 8 weeks (bottom panels). (c) Histological evaluation of infarct fibrosis reveals attenuation of scar in a representative animal treated with hESC-CMs (right panels) as compared with a representative animal receiving PBS alone (left panels) at 8 weeks post-transplantation. Masson's Trichrome stain (top panels) produces blue connective tissue and red muscle fibers to allow easy identification of the fibrotic scar resulting from ischemia reperfusion injury. Scale bars = 10 µm.

After analyzing the molecular changes underlying hESC differentiation as well as their electrophysiological phenotypes, we then assessed the effect of hESC-CM transplantation on myocardial function. Using SCID-Beige mice, one million hESC-CMs were transplanted by direct injection into ischemic regions of the left ventricle after 30 minutes of temporary left anterior descending (LAD) coronary artery occlusion. To characterize potential functional improvements, we performed echocardiography on post-transplant animals that received either hESC-CMs (n = 21) or PBS (n = 12) as a control. Left ventricular fractional shortening (LVFS), which is a common method for quantifying cardiac contractility or ability of the ventricle to eject blood, was used as the metric for comparison of the two groups' outcomes. Expressed as a percentage of the ventricle's volume, diminished LVFS is associated with a failing heart. Animals receiving hESC-CMs showed a 12.5±4.2% improvement over controls at 8 weeks as measured by LVFS (*P* = 0.03, **Figure 4**). This was primarily due to improvements in the left ventricular end systolic dimension (LVESD), as the left ventricular end diastolic dimension (LVEDD) remained constant between the two groups.

We also noted evidence of increased angiogenesis in the ischemic regions of explanted mouse hearts (**Figure S1**), and histology confirmed that the fibrotic scar was attenuated at 8 weeks post-transplantation in animals that underwent hESC-CM transplantation (**Figure 4c** and **Figure S2**). Using NIH Image J software, the quantified infarct sizes (percent of LV) in hESC-CM-treated mice and PBS controls were 21%±3% (n = 6) and 25%±2% (n = 6) (*P* = 0.041), respectively. However, improved LVFS was not sustained at later time point (16 weeks) (data not shown). Although the reasons for this are unclear, we suspect that acute donor cell death within the first month is responsible for attenuation of the positive remodeling processes initiated by hESC-CM paracrine effectors. To confirm this hypothesis, we decided to use molecular imaging to track hESC-CM fate noninvasively in living mice.

### Lentiviral transduction with reporter genes does not alter hESC characteristics

**Figure 5:**
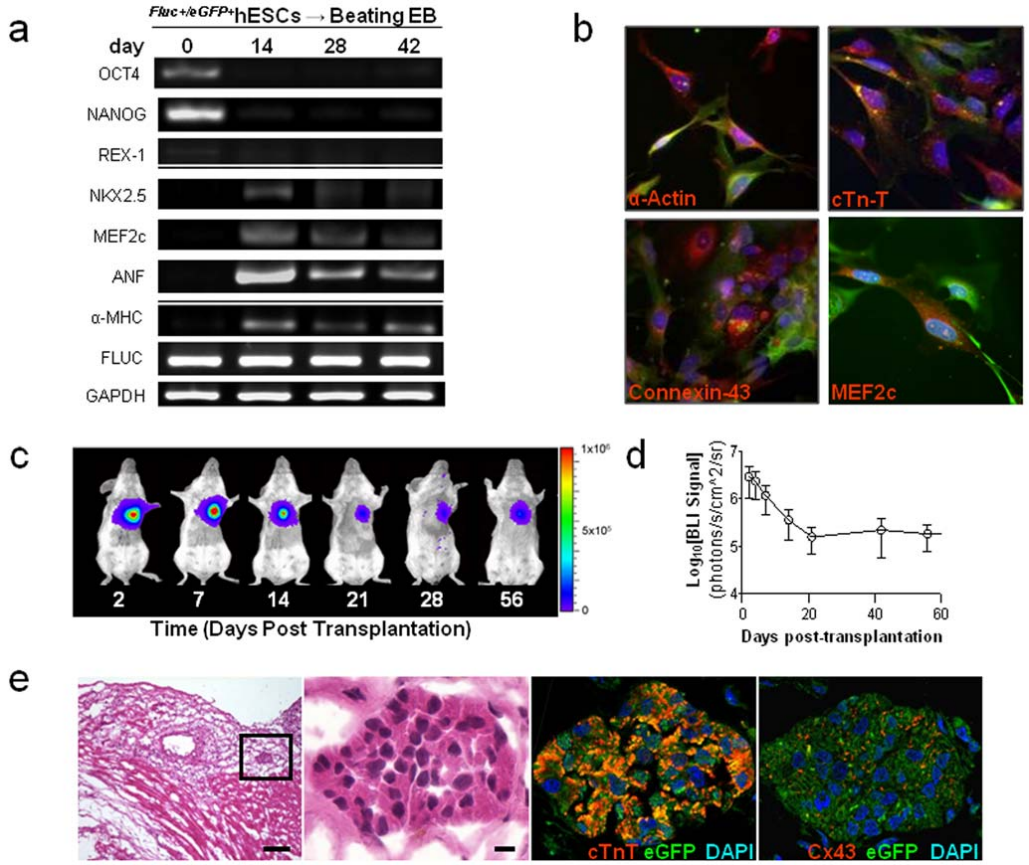
Survival and fate of *^Fluc+/eGFP+^*hESC-CMs *in vivo*. (a) RT-PCR analysis of various hESC and cardiac specific markers revealed no significant differences between *^Fluc+/eGFP+^*hESCs and control non-transduced hESCs (see Figure 1b for comparison), other than the presence of Fluc. (b) *^Fluc+/eGFP+^*hESC-CMs express cardiac specific markers such as α-actin, troponin-T, connexin-43, and MEF2C (all in red) and GFP (green, scale bars = 50 µm). (c) A representative animal imaged for 2 months following transplantation of 1 million *^Fluc+/eGFP+^*hESC-CMs into the heart. (d) *In vivo* bioluminescence imaging (BLI) signal measured from animals in which *^Fluc+/eGFP+^*hESC-CMs were transplanted into the ischemic hearts (n = 15). Signal activity falls drastically within the first 3 weeks of transplantation and remains stable thereafter, with no evidence of tumorigenesis (left). From 21 days post-transplantation onwards, BLI signal is reduced to <10% of the signal obtained at two days post-transplantation. (e) Histopathological evaluation of hearts following *^Fluc+/eGFP+^*hESC-CM delivery. H&E staining (left panels) demonstrates cluster of cells within the infarcted region of the heart (scale bars = 200, 20 µm for low and high magnification images, respectively). GFP positive cells within this cluster also express cardiac troponin-T (red, near right panel) and connexin-43 (red, far right panel). Scale bars = 20 µm.

In order to follow the fate of transplanted hESC-CMs noninvasively and longitudinally, we next employed reporter gene imaging. Undifferentiated hESCs were stably transduced with a firefly luciferase (Fluc) and enhanced green fluorescent protein (eGFP) double fusion reporter gene driven by a constitutive human ubiquitin promoter (pUB) using a lentiviral vector. The double fusion reporter gene can be stably expressed and does not alter hESC and hESC-CM characteristics (**Figure 5a and 5b**, **Figure S3**).

### Reporter gene imaging for tracking transplanted hESC-CM fate *in vivo*

To understand the fate of transplanted cells *in vivo*, one million *^Fluc+/eGFP+^*hESC-CMs were injected into peri-infarct regions of the myocardium. *^Fluc+/eGFP+^*hESC-CMs engrafted successfully, emitting a robust and stable bioluminescent signal for 8 weeks following transplantation (**Figure 5c**). Quantitative imaging analysis revealed that signal intensity declined logarithmically during the first 3 weeks post-injection and remained constant thereafter (**Figure 5d**). This initial drop in bioluminescence correlates with the death of roughly 90% of the administered cell population. Importantly, *^Fluc+/eGFP+^*hESC-CMs remained localized to the heart throughout our studies. Imaging did not reveal any cellular misbehavior, and no histological evidence of teratoma formation was observed in any animal within this group (n = 15) (**Figures 5d and 5e**, **Figure S4**). In contrast, injection of one million undifferentiated *^Fluc+/eGFP+^*hESCs into the heart led to both intra-cardiac and extra-cardiac teratoma formation in 7 out of 7 mice (**Figure S5**). We also performed spike-in studies to mimic clinically relevant scenarios in which contaminating undifferentiated hESCs are injected along with hESC-CMs. Our results show teratoma formation with 100 k hESCs (+400 k hESC-CMs), but not with 1 k hESCs (+499 k hESC-CMs) or 10 k hESCs (+490 k hESC-CMs) (**Figure S6**). Thus, these data suggest there is likely a threshold for the number of contaminating hESCs within an injected hESC-CM population that can lead to teratoma formation in the heart.

### Histologic evaluation of transplanted hESC-CMs

Importantly, our histologic studies revealed minimal integration of the *^Fluc+/eGFP+^*hESC-CMs into infarcted areas of the left ventricle. Though small clusters of injected cells appeared to engraft and then persist for many weeks after transplantation (emiting measurable bioluminescent signal), they did not exhibit functional organization with the surrounding host myocardium. These findings confirm previous reports which have also found minimal organized integration of hESC-CMs with host myocardium [27], [28], [29]. It is therefore difficult to explain the transient improvement in cardiac contractility at 8 weeks given the underwhelming evidence for robust integration of transplanted cells (**Figure 5e** and **Figure S4**). Such findings have led us and others to hypothesize that paracrine factors may be involved by helping to increase angiogenesis or preventing apoptosis of ischemic host myocardial tissues [30], [31], [32].

## Discussion

In this study, we have described the hESC-CM transcriptome within the spectrum of changes that occur between undifferentiated hESCs and fetal heart cells, and used molecular imaging to follow their survival and engraftment in the heart. Global gene expression profiling of hESC differentiation thus enables a systems-based analysis of the biological processes, networks, and genes that drive hESC fate decisions. This systems biology approach has obvious benefits over traditional PCR-based methods, which measure only a limited number of transcripts and so cannot define the complex regulatory networks of genes and pathways important for hESC differentiation.

Previous studies have also analyzed the transcriptional profiles of hESC-CMs [33], [34], and we found a high degree of similarity in the significant gene lists between our results and theirs. For example, we also noted upregulation of cardiac markers (e.g. MYH6, MYL4, TNNT2), cardiac transcription factors (e.g. TBX5, MEF2C, GATA4), as well as phospholamban (PLN). Interestingly, the two previous studies compared only spontaneously beating clusters of non-purified cardiomyocytes (what we refer to as “beating embryoid bodies” in this study). In our experience, only 2–5% of the cells within these clusters actually express the cardiac-specific marker cardiac troponin-T. Because of concern that non-cardiac and non-mesodermal cell types will obscure the hESC-CM molecular signatures, we used Percoll density gradient separation to achieve cardiac troponin-T-enriched populations ranging from 40–45%. Analysis of the expression differences between purified and non-purified CMs revealed considerable downregulation of early mesodermal and homeobox genes, and upregulation of cardiovascular and structural genes such as actins and extracellular collagens. Given this purified population of CMs, we were also able to perform robust bioinformatics analysis of these cells and compare them with fetal heart cardiomyocytes. This is important since we would like to establish how closely our derived cells compare with a gold standard. When looking at the GO processes that are more active in the FH cells, we found a pattern suggesting increased metabolic activity but not structural protein biogenesis. We believe these energy-related pathways are likely necessary for the cardiomyocyte to contract forcefully in the *in vivo* environment. This finding suggests that derived hESC-CMs have not adequately matured, at least as far as energy metabolism, and so may benefit from exogenous mechanical or electrical stimulation in order to upregulate energy-related pathways prior to transplantation.

Using purified hESC-CMs, our comprehensive, systems-based approach to transcriptional analyses supports the case that each of the stages of differentiation and selection results in a significant enrichment in cells of the cardiomyocyte lineage, expresses appropriate stage specific genes, and turns on appropriate biological processes corresponding to these stages. Given the robustness of our differentiation method, we believe the hESC-CM population would be an ideal source of replacement cells in the *in vivo* setting. We also demonstrate that hESC-CMs can successfully engraft in the ischemic heart for an extended duration that result in improved cardiac function, though only transiently. This latter finding may be partly attributed to the activation of paracrine signaling mechanisms by transplanted cells on host cells and themselves, which then attenuates after acute donor cell death. Lastly, we show that cardiac differentiation prior to transplantation can prevent teratoma formation, which remains a major safety concern for investigators exploring the therapeutic uses of hESCs.

In our microarray analysis, we observed high expression of pluripotency-related genes involved in the core hESC regulatory circuitry, including OCT4, SOX2, and NANOG, as well as CRYPTO 1 and 3, LCK, and HESX1. Differentiation into beating EBs was accompanied by mesodermal differentiation and dramatic activation of TWIST1, TBX5, and MEOX transcription, as well as the very clear induction of nearly all of the early cardiogenic genes, including FOXC1, ISL2, HAND1, GATA4, 5, and 6, FOXH1, and MEF2C. While it is clear that other developmental lineages are still present in the EB population, it is also clear from the high levels of cardiac gene expression that this population is significantly enriched for the cardiac lineage even at an early stage. The transcriptional analysis of the final differentiation and selection of the hESC-derived CMs indicates that this enrichment continues, with the CM population expressing differentiated cardiomyocyte genes at levels similar to our more advanced FH cells. Importantly, because of the cell type heterogeneity in the fetal heart, we specifically isolated cardiomyocytes from the fetal left ventricles for microarray analysis.

We now briefly discuss the four major trends in the microarray data seen in the K-means clustering analysis (**Figure 2d**), which will allow us to explore the major themes within an enormous amount expression data. Cluster 1 is composed of 1775 genes whose expression increases at each stage from hESC to EB to hESC-CM to FH (**Figure 2d** and **Table S3 (B1)**). Overrepresentation analysis of this cluster of genes shows that the GO processes to which these genes contribute include many basic differentiated cell functions such as the establishment of cellular transport and secretory processes, regulation of cell localization, response to cellular stresses and hypoxia, cytoskeletal biogenesis, control of apoptosis, and interestingly, cardioblast cell fate commitment (**Table S4 (B5)**). This cluster of genes has a substantial overlap with the component genes of principal component 1 from the PCA analysis (**Figure 2b**), demonstrating how two analytic approaches can result in similar significant findings.

The converse expression pattern is seen in the 2,453 genes composing cluster 2, which are sequentially downregulated across the groups from hESC to EB to hESC-CM to FH (**Table S3 (B2)**). The processes overrepresented in this cluster primarily involve nucleic acid synthesis, DNA replication and chromatin maintenance, cell cycle, and transcription in general (**Table S4 (B6)**). This theme is consistent with patterns seen in normal embryonic development in both drosophila and mouse [35], and reflects the fact that earlier undifferentiated cells are undergoing rapid replication and production of broad ranges of transcripts, while cell cycling slows dramatically later in development as cells begin to express a more limited number of genes that are appropriate for the differentiated state.

Cluster 3 is comprised of 1,009 genes whose expression increases at each stage from hESC to EB to hESC-CM, but which are expressed significantly less in the FH cells (**Table S3 (B3)**). The overrepresented processes in this cluster correspond to non-cardiac cell differentiation pathways, particularly neuroectodermal differentiation, that compose a portion of the hESC-CM population which we have differentiated and purified it from hESC precursors, but are not present in the harvested fetal heart cells (**Table S4 (B7)**). The final interesting cluster of genes is cluster 4 (**Table S3 (B4)**), representing genes which generally increase in expression across all stages from hESC to EB to hESC-CM to FH, but which are expressed at considerably higher levels in the two older FH samples, 3 and 4 (at 20 and 21 weeks, respectively), than in FH1 and 2 (19 weeks each). The processes overrepresented in this gene group are heavily weighted toward cardiac muscle contraction, muscle development, heart development and other cardiac specific processes, and the genes contributing to these processes include dozens of cardiac structural proteins such as cardiac myosin heavy and light chains, cardiomyocyte potassium channels such as KCNE1 [36], KCNQ1 [37] and KCNH2 [38], cardiomyocyte troponins including T2 and C1, as well as cardiac phospholamban and cardiac actin 1 (**Table S4 (B8)**). Thus the hESC-CM population's expression of terminal cardiac differentiation markers at a level intermediate between younger and older FH cardiomyocytes suggests that this population is sufficiently advanced developmentally to serve as a potential replacement population for cells lost to ischemia.

With these very interesting and detailed gene expression studies, we began focusing on cellular transplantation to the ischemic heart. We observed significant improvements in echocardiographic metrics when comparing treated and control animals. Histologic analysis revealed reduced scar formation, but there was underwhelming evidence of functional myocardium regeneration, confirming previous reports [28], [29], [39]. To explain this disparity, the improvement in cardiac function may be due to paracrine factors, as suggested by Dzau and colleagues [30], [31], [32]. Our own studies indicate some increased cytokine signaling in hypoxic hESC-CMs (**Figure S7**). However, if paracrine signaling is the primary mechanism of improvement, then long-term generation of these factors from sufficient numbers of transplanted hESC-CMs may be required for a sustained improvement in cardiac function. Until now no studies have analyzed the overall survival and growth kinetics of transplanted hESC-CMs in ischemic myocardium.

To address this lack of understanding, we employed molecular imaging technology for understanding the fate of cells following transplantation [40]. Longitudinal imaging of transplanted hESC-CMs exposes the limitations of cardiac stem cell therapy, as ∼90% of cells die within the first three weeks of delivery. Though we did not address the specific mechanism of death in this study, poor cell survival is likely due to widespread apoptosis and anoikis of cells injected into an inhospitable environment. Improving cell survival by subjecting hESC-CMs to the appropriate anti-apoptotic and pro-survival cues may alleviate some of the survival issues, and efforts to this end have been reported since completion of this work [41]. Other methods that take advantage of tissue engineering technologies in which biopolymers and synthetic tissue constructs are used to organize and support transplanted cells may offer another means for increasing cell survival [42]. Delivery techniques other than intra-myocardial injection, such as intracoronary or retrograde coronary venous, may also improve cell survival [43]. Another confounding factor is the host immune response, which we did not address in this study (as SCID mice were used). With a functioning host immune system, we would expect to see a further reduction in cell survival. Nevertheless, it is important to note that even in our SCID mice, transplantation of *^Fluc+/eGFP+^*hESC-CMs did *not* form teratomas in the post-transplantation period. The lack of teratoma formation emphasizes the robustness of our hESC-CM purification protocol in removing undifferentiated cell contaminants.

In summary, hESC-CMs hold potential promise for treatment of cardiovascular disease. The molecular processes that control stem cell pluripotency, differentiation, and proliferation are complex, justifying the need for a broad investigation that integrates systems biological tools for transcriptome analysis with molecular imaging tools for confirmation of survival, engraftment and functional benefit in the *in vivo* setting. We found that the enriched hESC-CMs expresses cardiomyocyte genes at levels similar to 20-week fetal heart cells, making this population a good source of potential replacement cells in the *in vivo* setting. Beyond a characterization of the overall transcriptional characteristics of our differentiated cells, we have also identified a large number of potentially important new genes that are expressed at high levels at distinct stages and that may play roles in the cardiogenic developmental program. These genes may also act as specific markers of cell differentiation in addition to being inducers of cardiogenic differentiation, thus opening new avenues of investigation into the basic biology of cardiovascular development. However, understanding the molecular networks of differentiation is not enough to predict the fate of differentiated cells once transplanted in a living host. To address this lack of knowledge, we have shown molecular imaging to be a powerful method for assessing cellular localization, engraftment, survival, and proliferation *in vivo*. Taken together, gene expression and molecular imaging studies such as this will serve as a crucial foundation for future clinical applications of stem cell therapies.

## Materials and Methods

### Culture of undifferentiated hESCs

hESCs (H9 line) from passage 35–40 were initially maintained on top of murine embryonic fibroblasts (MEF) feeder layers, seeded onto 0.1% gelatin coated plastic dishes, and inactivated by 10 µg/ml of mitomycin C. hESCs were maintained in ES medium containing 80% Dulbecco's modified Eagle's medium/F12, 1 mM L-glutamine, 0.1 mM β-mercaptoethanol, 0.1 mN non-essential amino acids, 20% Knockout Serum Replacement, and 8 ng/ml hbFGF. The ES cell culture medium was changed daily and hESCs were passaged every 4–5 days.

### Lentiviral production and generation of stable hESC lines

SIN lentivirus was prepared by transient transfection of 293T cells [44]. hESCs were stably transduced with LV-pUB-Fluc-eGFP at a multiplicity of infection (MOI) of 10. The infectivity was determined by eGFP expression as analyzed on FACScan (BD Bioscience). The eGFP positive cell populations were isolated by fluorescence activated cell sorting (FACS) Vantage SE cell sorter (Becton Dickinson) followed by plating on the feeder layer cells for long-term culturing. Flow cytometry data were analyzed with FlowJo (Treestar, San Carlos, CA) analysis software.

### Embryoid body formation and cardiac specific differentiation

hESC colonies were dispersed into cell aggregates containing approximately 500 to 1,000 cells using 1 mg/mL collagenase IV. The cell aggregates were then suspension-cultured in ultra-low attachment cell culture dishes with hESC differentiated medium (without basic fibroblast growth factor) for 9 days with the media changed every two days. To promote cardiac differentiation, 9-day old EBs were transferred to 10 cm dishes coated with 0.1% gelatin and grown in media consisting of DMEM supplemented with 20% FBS and 2 mmol/L L-glutamine. During differentiation, the media was changed every two days. Spontaneously contracting cells appeared as clusters in outgrowths from the EBs at day 10 after differentiation. These beating EBs were maintained in long-term cultures for up to 103 days.

### Isolation of hESC-CMs

Differentiated cultures containing beating cardiomyocytes were washed with phosphate buffered saline (PBS) and incubated with 0.56 units/ml Liberase Blendzyme IV (Roche, Indianapolis, IN) at 37°C for 25 min. After dissociation, the cell suspension was separated by Percoll density (58.5% and 40.5%) centrifugation at 1500 g for 30 minutes at room temperature.

### Cell samples collection and RNA preparation

The undifferentiated hESC, day 10 beating whole embryoid bodies (Beating EBs), day 14 Percoll-enriched cardiomyocytes derived from human hESCs (hESC-CM) and human fetus heart-derived left ventricular cardiomyocytes (FH-CM) at 19, 19, 20, and 21 weeks were collected at chosen time points. Four samples from each group (for a total of 16 unique samples) were harvested for RNA isolation. Total RNA was isolated as described previously in Trizol (Invitrogen) followed by purification over a Qiagen RNeasy column (Qiagen).

### Microarray hybridization and data acquisition

A full description of RNA quality control, and labeling reaction and hybridization is included in **Methods S1**. Using Agilent Low RNA Input Fluorescent Linear Amplification Kits, cDNA was reverse transcribed from each of 16 RNA samples representing four biological quadruplicates, as well as the pooled reference control, and cRNA was then transcribed and fluorescently labeled with Cy5/Cy3. cRNA was purified using an RNeasy kit (Qiagen, Valencia, CA, USA). 825 ng of Cy3- and Cy5- labeled and amplified cRNA was hybridized to Agilent 4×44 K whole human genome microarrays (G4112F) and processed according to the manufacturer's instructions. The array was scanned using Agilent G2505B DNA microarray scanner. The image files were extracted using Agilent Feature Extraction software version 9.5.1 applying LOWESS background subtraction and dye-normalization.

### Microarray data analysis

The data were analyzed using the SAM algorithm [7] with multiple testing correction to identify genes which had statistically significantly changed expression between each stage, and K-means clustering to identify clusters of genes having unique temporal expression profiles. For hierarchical clustering, we used positive correlation for distance determination and required complete linkage. Gene Ontology overrepresentation analysis was performed using Fisher's Exact test and High Throughput GOMiner software.

### Electrophysiology analysis

Action potential (AP) recordings from single cells were done using the whole-cell patch-clamp technique. Patch pipettes were prepared from 1.5 mm thin-walled borosilicate glass tubes using a Sutter Micropipette Puller (P-97) and typically had resistances of 4–6 MΏ when filled with an internal solution containing (mM): 110 K^+^ aspartate, 20 KCl, 1 MgCl_2_, 0.1 Na-GTP, 5 Mg-ATP, 5 Na_2_-phospocreatine, 1 EGTA, 10 HEPES, pH adjusted to 7.3 with KOH. The external Tyrode's bath solution consisted of (mM): 140 NaCl, 5 KCl, 1 CaCl_2_, 1 MgCl_2_, 10 glucose, 10 HEPES, pH adjusted to 7.4 with NaOH. Upon seal formation and following membrane rupture, APs were recorded using the current-clamp mode. Data were filtered at 10 KHz. Axopatch 200B, Digitize 1322 and pClamp8 (Axon Burlingame, CA, USA) were used for data amplification and acquisition. hESC-CMs were categorized into pacemaker-, atrial- or ventricular-like phenotypes, based on such common electrophysiological characteristics as the AP amplitude (mV), upstroke velocity (mV/ms), APD50 and APD90 (ms), as well as the resting membrane potential (RMP, mV). We primarily used the AP profiles as signatures of different chamber-specific CM types. Nodal-like AP phenotype was defined as those that exhibited: **a)** prominent phase-4 depolarization, **b)** slow upstroke (dV/dt), **c)** small action potential amplitude (APA), **d)** relatively depolarized MDP, and **e)** spontaneous firing. By contrast, like others, we defined the ventricular-like phenotype as those that displayed: **i)** a significant plateau phase, **ii)** longer APD (vs. those of atrial and nodal), **iii)** rapid upstroke, and **iv)** a flat phase 4. Atrial APs were those that displayed a triangular shape. Of note, in comparison to neonatal and adult human ventricular and atrial CMs, the AP parameters of hESC-CMs exhibit MDP and upstroke velocities that were positive (∼−40 vs. ∼−80 mV) and slow (∼10 V/s vs. 100–300 V/s), respectively. (See **Methods S1** for further Electrophysiology Methods).

### Measurements of cytosolic Ca^2+^

A spectrofluorometric method with Fura-2/AM as the Ca^2+^ indicator was used for measuring [Ca^2+^]_i_. FLV- or hESC-CMs were incubated with 5 µM Fura-2/AM and 0.2% pluronic F-127 for 30 min at 37°C. Fluorescent signals obtained upon excitation at 340 nm (F340) and 380 nm (F380) were recorded from cells perfused with Tyrode solution containing (mM): 140 NaCl, 5.0 KCl, 1.0 CaCl_2_, 1.0 MaCl_2_, 10.0 glucose and 10 HEPES (pH 7.4) unless otherwise indicated. Data were analyzed using the Ionwizard software (Version 5, IonOptix) to generate the Ca^2+^ transient parameters. The F340/F380 ratio was used to represent cytosolic [Ca^2+^]. To induce cytoplasmic Ca^2+^ transients, hESC-CMs were electrically stimulated. Ca^2+^ transients were recorded and analyzed after a series of depolarizations that enabled each transient to fully decay so as to establish steady-state SR content.

### Effect of reporter genes on hESC proliferation and differentiation

Reverse transcription polymerase chain reaction (RT-PCR) was used to compare the expression of embryonic markers (Oct4, NANOG, Rex1), cardiac transcription factors (Nkx2.5, MEF2C), ventricular specific proteins (αMHC, ANF), and Fluc reporter gene between control non-transduced hES and *^Fluc+/eGFP+^*hESCs. RNA was isolated from hES and *^Fluc+/eGFP+^*hESCs using Trizol reagent. Two µg of total RNA extracted from EBs was reverse-transcribed using ThermoScript RT-PCR system (Invitrogen, Carlsbad, CA). One µl of cDNA sample was PCR amplified with gene-specific primers (see **Methods S1**) using optimized PCR cycles to obtain amplified reactions in a linear range. PCR products were separated on 1% agarose gel by electrophoresis and quantified by using Labworks 4.6 Image Acquisition and analysis software (UVP Bio-imaging systems, Upland, CA).

### Transplantation of hESC-CMs into ischemic myocardium

A total of 50 adult female SCID Beige mice (Charles River Laboratories) weighing 20–25 gm (8 weeks old) were used. All procedures were performed in accordance with protocols approved by the Stanford Animal Research Committee guidelines. Following induction of anesthesia with isoflurane (3–4%), animals were orotracheally intubated and ventilated with a mixture of oxygen and 2–3% isoflurane with a volume-cycled rodent ventilator as described [45]. A lateral thoracotomy was performed followed by ligation of the mid left anterior descending (LAD) artery for 30 minutes. Myocardial infarction was confirmed by blanching and EKG changes. Subsequently, 3 groups received direct myocardial injection of: (1) 1×10^6^ *^Fluc+/eGFP+^*hESC-derived cardiomyocytes in 40 µl of PBS (n = 16), (2) 1×10^6^ non-transduced hESC-derived cardiomyocytes (n = 6), and (3) 40 µl of PBS as control (n = 12). Another set of 16 animals were used to evaluate the potential for teratoma formation following intramyocardial injection of undifferentiated *^Fluc+/eGFP+^*hESCs. Animals were injected with 1×10^6^ undifferentiated *^Fluc+/eGFP+^*hESCs (n = 7), 1×10^5^ undifferentiated *^Fluc+/eGFP+^*hESCs spiked with 4×10^5^ non-transduced hESC-CMs (n = 3), 1×10^4^ undifferentiated *^Fluc+/eGFP+^*hESCs spiked with 4.9×10^5^ non-transduced hESC-CMs (n = 3), and 1×10^3^ undifferentiated *^Fluc+/eGFP+^*hESCs spiked with 4.99×10^5^ non-transduced hESC-CMs (n = 3). Post-operative analgesia was provided by a one-time, subcutaneous injection of buprenorphine (0.1 mg/kg body weight). Animals were recovered in a warmed, humidified chamber.

### Bioluminescence imaging (BLI) of hESC and hESC-CM transplantation

Cardiac BLI was performed by an independent, blinded operator using the Xenogen In Vivo Imaging System. Mice were anesthetized with 2% isoflurane and D-Luciferin was administered intraperitoneally at a dose of 375 mg/kg body weight. At the time of imaging, animals were placed in a light-tight chamber, and photons emitted from luciferase expressing hESCs transplanted into the animals were collected with integration times of 1–10 min, depending on the intensity of the bioluminescence emission. Ventral images were obtained to better determine the origin of photon emission. The same mice were scanned repetitively over 12 months as per the study design. Bioluminescence was quantified in units of maximum photons per second per centimeter square per steridian (p/s/cm^2^/sr).

### Assessment of left ventricular contractility

Echocardiography was performed by an independent, blinded operator using the Siemens-Acuson Sequioa C512 system equipped with a multi-frequency (8–14 MHz) 15L8 transducer. Mice were assessed pre-operatively, and 2, 4, 8, and 16 weeks post-transplant. Animals received continuous inhaled isoflurane (1.5–2%) for the duration of the imaging session (10–15 minutes). Animals were imaged in the supine position resting on a specialized platform allowing for continual inhaled anesthesia while maintaining optimal exposure of the left chest. M-mode short axis views of the LV were obtained and archived. Analysis of the M-Mode images was performed using the Siemens built-in analysis software. Left ventricular end diastolic diameter (LVEDD) and end-systolic diameter (LVESD) were measured and used to calculate fractional shortening (FS) by the following formula: FS = [LVEDD-LVESD]/LVEDD [46].

### Cell and tissue immunohistochemical analysis

To confirm their undifferentiated state, cultured hESCs were plated onto 8 chamber slides and fixed with acetone on ice for 20 minutes, then stained for immunofluorescence with the appropriate antibodies. Microscopy was performed using a ZEISS Axiovert microscropy (Sutter Instrument). Hearts were excised two months after transplantation and prepared in 10-micron thick frozen sections. Immunofluorescent labeling was analyzed using a Zeiss LSM 510 Confocal Laser Scanning Microscope equipped with a Coherent Mira 900 tunable Ti:Sapphire laser for two-photon excitation (Zeiss, Minneapolis, MN).

### Statistical analysis

Unless otherwise noted, non-microarray data are presented as mean±S.D. Data were compared using standard or repeated measures, using ANOVA where appropriate. Pairwise comparisons were performed using a two-tailed Student's t–test. For electrophysiology data, data are expressed as mean±SEM. One-way ANOVA followed by Newman-Keuls multiple comparison tests or paired *t* test was carried out to test for differences between the mean values within the same study. For all data, differences were considered significant for *P*-values<0.05.

## Supporting Information

Supplemental Results S1 Document contains additional analysis of microarray data.
(0.07 MB PDF)

Methods S1 Document contains supplemental methods.
(0.11 MB PDF)

Figure S1 Quantitative analysis of the endothelial cell marker CD31 (mouse) shows upregulation of capillary density in ischemic hearts at week 8. The hESC-CM-treated group showed significant augmentation of CD31 positive capillary density (*P*<0.05). Capillary densities were examined by counting the number of capillaries stained with anti-CD31 in five random fields on two different sections (approximately 3 mm apart) from each mouse. Images were analyzed using Image J software.
(2.08 MB TIF)

Figure S2 Ventricular scar formation after hESC-CM transplantation. (a) Histological evaluation of infarct fibrosis reveals attenuation of scar in a representative animal treated with hESC-CMs (right panels) as compared with a representative animal receiving PBS alone (left panels) at 8 weeks post-transplantation. Masson's Trichrome stain (bottom panels) produces blue connective tissue and red muscle fibers to allow easy identification of the fibrotic scar resulting from ischemia reperfusion injury. (b) The quantified infarct sizes (percent of LV) in hESC-CM-treated mice and PBS controls were 21%±3% (n = 6) and 25%±2% (n = 6) (*P* = 0.041), respectively. Scale bars = 10 µm.
(40.89 MB TIF)

Figure S3 Stable lentiviral transduction of hESCs with double fusion (DF) reporter gene. (a) Schema of the DF reporter gene containing Fluc and eGFP with brightfield (left) and fluorescent (right) images of ^Fluc+/eGFP+^hESCs (scale bars = 200 µm). (b) Stably transduced ^Fluc+/eGFP+^hESCs (collected by FACS) show robust correlation between cell number and reporter gene activity. Raw bioluminescence images of increasing numbers of ^Fluc+/eGFP+^hESCs in vitro are shown below graph. (c) ^Fluc+/eGFP+^hESCs maintain firefly luciferase activity over successive passages. (d) ^Fluc+/eGFP+^hESCs maintain pluripotent stem cell markers such as SSEA-4, Oct-4, and AKP, but remain negative for differentiation marker SSEA-1. Scale bars = 50 µm. (e) RT-PCR analysis of embryoid bodies over the course of 7 weeks shows expression of endodermal (AFP), mesodermal (αMHC), and ectodermal (NeuroD) germ layer markers for both control non-transduced hESCs and ^Fluc+/eGFP+^hESCs. GAPDH is used as loading control.
(3.70 MB TIF)

Figure S4 Histopathological evaluation demonstrates that hESC-CMs do not integrate into host myocardium but continue to express cardiac markers. Representative histopathological images of explanted hearts taken two months after ^Fluc+/eGFP+^hESC-CM delivery. GFP positive cells (transplanted ^Fluc+/eGFP+^hESC-CMs) express cardiac troponin-T and connexin-43, but do not appear to be well integrated with the surrounding host myocardium.
(5.26 MB TIF)

Figure S5 Bioluminescence imaging and histological fate of undifferentiated ^Fluc+/eGFP+^hES cells transplanted into the heart. (a) Representative images from a single animal receiving one million undifferentiated ^Fluc+/eGFP+^hES cells. Undifferentiated hES cells rapidly form teratomas with extra-cardiac spread within 3 to 4 weeks of transplantation. (b) Quantification of imaging signals from animals receiving undifferentiated ^Fluc+/eGFP+^hESCs (n = 6) or ^Fluc+/eGFP+^hESC-CMs (n = 15) shows logarithmic increases in BLI signals in the undifferentiated group (*P<0.001) vs. the hESC-CM group due to teratoma formation. (c) Histology demonstrating typical teratoma formation in the heart following transplantation of undifferentiated ^Fluc+/eGFP+^hES cells. Histological features of low-power field of teratoma (I), respiratory epithelium (II), and cartilage formation (III) can be identified (scale bars = 50 µm). The border of the graft area shows that only host myocardium stains positive for cardiac markers such as cardiac troponin-T (cTnT), while cardiac markers are absent from the eGFP+ region (IV).
(3.84 MB TIF)

Figure S6 Bioluminescence imaging of undifferentiated ^Fluc+/eGFP+^hESCs mixed with non-transduced hESC-derived cardiomyocytes after transplantation to SCID mouse heart. This study represents a clinically relevant scenario in which undifferentiated hESC contaminants are mixed in with the hESC-CM population. We observed teratoma formation in the 100 k hESC contaminant group, but not in the 10 k or 1 k hESC groups. Data presented as mean±SEM.
(2.41 MB TIF)

Figure S7 ^Fluc+/eGFP+^hESC-CMs upregulate secretion of angiogenic growth factors under hypoxic conditions. (a) Culture media from hESC-CMs under hypoxia (1% O2/5% CO2/94% N2) or normoxia (20% O2/5% CO2) was washed over an antibody array to assess angiogenic protein secretion levels. (b) Hypoxia induces significant up-regulation of multiple cytokines by ^Fluc+/eGFP+^hESC-CMs. Following 12 hours of hypoxia *in vitro*, media from ^Fluc+/eGFP+^hESC-CMs had increased levels of FGF, IL-6, IL-8 and VEGF as compared to cells maintained in normoxic conditions.
(0.81 MB TIF)

Table S1 Microarray data tables of differentially-regulated genes.
(13.12 MB PDF)

Table S2 Gene Ontology analysis of differentially-regulated genes.
(0.30 MB PDF)

Table S3 K-means clustering analysis of microarray data.
(3.37 MB PDF)

Table S4 Gene Ontology analysis of K-means clustering data.
(0.20 MB PDF)

Movie S1 Beating embryoid body.
(1.78 MB MOV)

Movie S3 Beating left ventricular fetal cardiomyocytes.
(0.93 MB MOV)

Movie S2 Beating hESC-CMs after Percoll purification.
(0.39 MB MOV)
