# Supplementary material for: Transcriptional and Functional Profiling of Human Embryonic Stem Cell-Derived Cardiomyocytes
Source: PLoS One. 2008 Oct 22;3(10):e3474. doi: 10.1371/journal.pone.0003474 (PMC2565131; doi:10.1371/journal.pone.0003474)
Supplement: Supplemental Results S1 — Document contains additional analysis of microarray data. (0.07 MB PDF) [file pone.0003474.s001.pdf]

SUPPLEMENTAL RESULTS S1

Microarray Analysis of Novel hESC Markers. In addition to these known ES markers, however, we have also identified a significant number of novel genes with elevated expression in hESCs that may play important roles in the developmental or pluripotentiality. Of particular interest is Fzd5, the receptor for Wnt5a, which is 15-19 fold more highly expressed in hESCs, suggesting some canonical Wnt signaling occurring in this population, although there also appears to be a significant upregulation of a number of Wnt ligands at the EB stage. PIM2, an anti-apoptotic kinase whose relatives PIM1 and PIM3 are involved in ESC self-renewal, is also expressed 20 fold higher in hESCs than in EB, suggesting that it too plays a role in self-renewal, a role it may reprise in several hematopoietic malignancies [1,2]. Another gene that appears prominently in our experiments is phorbol-12-myristate-13-acetate-induced protein 1 (PMAIP1), whose RNA is 10 fold more highly expressed in hESCs [3,4]. PMAIP1, a BH3-only member of the Bcl2 family, appears to be a mediator of p53 dependent apoptosis, and is highly expressed in adult T-cell leukemia cell lines.

Microarray Analysis of Embryoid Bodies. Between the hESC and EB stages, we identified 4,407 genes that are significantly upregulated (**Table S1 (A2)**). Importantly, the most significantly upregulated gene is TWIST1, a bHLH family member which is one of the master regulators of mesodermal differentiation, and which is upregulated by 22 fold in the EB cells [5] while MEOX1, a gene necessary for somitogenesis, is 4 fold upregulated [6]. Almost as statistically significant, and much more highly upregulated at 46 fold, is Tbx2, a gene involved in regulating mesodermal genes and which is important later in regulating angiogenesis and cardiac development. Others include the closely related Tbx5 gene, which at 222 fold is one of the most

highly upregulated genes in EB, and the cardiac regulator Tbx1 (22 fold) [7,8]. Other early regulators of cardiac and vascular differentiation are also highly upregulated, including the forkhead box gene Foxc1 (50 fold induction) [9,10,11,12], the second heart field regulator Isl1 (35 fold induction) [13], the master muscle regulator Mef2C (28 fold induction) [14], and the cardiac morphogenetic gene Hand2 (6.4 fold induction) and FoxH1 (5.2 fold induction). GATA4, a highly dosage sensitive regulator of cardiac development, is upregulated at a comparatively modest but still substantial level (6.7 fold induction) [15], and so are its relatives GATA5 (16 fold induction) and GATA6 (12 fold induction) [16,17], as well as Irx5 (8 fold induction) [18].

The elevated expression levels of these cardiac master transcriptional regulators are reflected in very significantly upregulated *cardiac specific* structural and functional genes, including the following: Myoz2 (Calsarcin1), a calcineurin binding Z-disc protein implicated in cardiomyopathy, which is the most highly upregulated gene at 250 fold [19]; phospholamban (PLN), a major mediator of cardiomyocyte SERCA pump activity (upregulated 218 fold) [20]; cardiac muscle myosin alpha heavy chain 6 (MYH6, 100 fold); ventricular myosin light chain 3 (MYL3, 30 fold); cardiac troponin T 2 (TNNT2, 27 fold), atrial myosin light chain 4 (MYL4, 19 fold); cardiac myosin binding protein C (MYBPC3, 13 fold); cardiac myosin heavy chain (MYH7, 8.4 fold); cardiac myosin heavy chain 7B (MYH7B, 6.4 fold); myosin light chain 3 (MYL3, 6 fold); cardiac alpha actin 1 (ACTC1, 4.7 fold); and ATPA2 (SERCA2, 2.6 fold).

Clearly, this population of cells has differentiated with a significant bias toward mesodermal and cardiac lineages, but we do still see at this stage expression of genes characteristic of all three cell layers, endoderm, mesoderm and ectoderm. These include endodermal genes such as Foxa1 (HNF3A, 20 fold) [21,22], liver genes such as complement

factor I (CFI, 120-170 fold) [23] and liver arginase (ARG1, 89 fold), skeletal muscle genes such as fast skeletal myosin light chain 2 (MYLPF, 25 fold) and skeletal myosin light chain 1 (MYL1, 7 fold), and neuroectodermal genes such as ectodermal-neural cortex (ENC1, 6 fold) and neural cell adhesion molecule (NCAM, 3 fold), demonstrating that beating EBs still contain cells from each lineage. Many developmental regulatory genes are also very much active in the beating EBs population, including Wnts (Wnt1, 2, 2b, 3a, 5a, 10b, and 11, and FZD1, 3, 4, and 10, all significantly upregulated) [24], and homeobox genes (HOXA2, A3, A4, B2, B3, B4, B5, C5, D1, D2, D3, MSX1, 2, MEOX1, and a number of others) [25], demonstrating the multiplicity of developmental lineages represented.

Microarray Analysis of hESC-CM Biological Processes. Biological process analysis of the hESC-CMs shows that cellular maturation processes such as cytoskeletal organization and biogenesis, cell localization, and developmental processes such as muscle development and angiogenesis predominate in the CMs when compared to the beating EB populations. This maturation of the CMs, which are still somewhat heterogeneous, is a necessary intermediate step between undifferentiated, rapidly cycling cells and the stable cells possessing a solid cytoskeleton that are beginning to become firmly anchored into place with ECM and ready to contract with significant force (**Table S2 (A9 and A10)**). We can also see that the cells in this population are continuing to slow the rampant cell cycling, nucleotide synthesis, transcription, and mRNA processing seen in the hESC populations, as well as to downregulate non-cardiac developmental programs such as mesenchymal cell differentiation, kidney development, and neural development, among others.

Microarray Analysis of Fetal Heart Cells. Comparing the transcriptional profile of the CM cells to cultured primary fetal cardiomyocytes (FH), we find that 1,451 genes are expressed at lower levels in the FH population, and that many of these genes are associated with differentiation programs other than the cardiomyocyte program, highlighting the fact that the FH cells are less heterogeneous than our differentiated CM cell populations (**Table S1 (A5)**). For example, the early pan-mesodermal gene Dkk2 [26] is expressed 92 fold less in FH, the skeletogenic gene sclerostosis (SOST) is expressed 406 fold less, and a significant number of neurogenic genes including synaptotagmins-4 (141 fold) and -13 (88 fold), neurofilament light (23 fold), medium (86 fold), and heavy chains (4.8 fold), as well as regulatory genes such as EPHB1 (5 fold) and DCX (72 fold) [27] are also expressed at much lower levels in FH than CM.

Compared to the CM cell population, we find that a total of 634 genes are expressed more highly in the transcriptional program of the FH cells (**Table S1 (A6)**). Among the most differentially elevated transcripts are several encoding tumor suppressor genes, including tumor suppressor candidate 1 (TUSC1, 42 fold more highly expressed) [28], cervical cancer suppressor gene 5 (ZNF434, 33 fold) [29], TSPYL5 (56 fold) [30], XAF1 (BIRC4BP, 20 fold) [31], and OSGIN1 (4.6 fold) [32], as well as the serum deprivation response gene (SDPR, 42 fold) [33], consistent with a terminally differentiated cell population. We also see much higher expression of the later cardiac developmental genes CSRP1 (Heart Lim Protein, 26 fold) [34], FHL2 (5 fold) [35], and natriuretic peptide receptor 1 (NPR1, 5.2 fold) [36] in the FH population, consistent with their known developmental expression patterns. Other very interesting differences between our CM population and the primary FH cardiomyocytes are revealed when we perform GO process analysis. As might be expected, we see that processes that are less active in the FH cells include many having to do with developmental processes of other tissues, particularly neural developmental processes, body plan regulatory processes, and diverse signaling families such as

Wnt pathways. This suggests, as would be expected, that the FH cells are a somewhat more pure cardiomyocyte population than our differentiated CMs.

SUPPLEMENTAL REFERENCES

1. Aksoy I, Sakabedoyan C, Bourillot PY, Malashicheva AB, Mancip J, et al. (2007) Self-renewal of murine embryonic stem cells is supported by the serine/threonine kinases pim-1 and pim-3. Stem Cells 25: 2996-3004.
2. Kelly BD, Hackett SF, Hirota K, Oshima Y, Cai Z, et al. (2003) Cell type-specific regulation of angiogenic growth factor gene expression and induction of angiogenesis in nonischemic tissue by a constitutively active form of hypoxia-inducible factor 1. Circ Res 93: 1074-1081.
3. Oda E, Ohki R, Murasawa H, Nemoto J, Shibue T, et al. (2000) Noxa, a BH3-only member of the Bcl-2 family and candidate mediator of p53-induced apoptosis. Science 288: 1053-1058.
4. Yakovlev AG, Di Giovanni S, Wang G, Liu W, Stoica B, et al. (2004) BOK and NOXA are essential mediators of p53-dependent apoptosis. J Biol Chem 279: 28367-28374.
5. Chen ZF, Behringer RR (1995) twist is required in head mesenchyme for cranial neural tube morphogenesis. Genes Dev 9: 686-699.
6. Mankoo BS, Skuntz S, Harrigan I, Grigorieva E, Candia A, et al. (2003) The concerted action of Meox homeobox genes is required upstream of genetic pathways essential for the formation, patterning and differentiation of somites. Development 130: 4655-4664.
7. Chapman DL, Garvey N, Hancock S, Alexiou M, Agulnik SI, et al. (1996) Expression of the T-box family genes, Tbx1-Tbx5, during early mouse development. Dev Dyn 206: 379-390.
8. Harrelson Z, Kelly RG, Goldin SN, Gibson-Brown JJ, Bollag RJ, et al. (2004) Tbx2 is essential for patterning the atrioventricular canal and for morphogenesis of the outflow tract during heart development. Development 131: 5041-5052.
9. Kume T, Jiang H, Topczewska JM, Hogan BL (2001) The murine winged helix transcription factors, Foxc1 and Foxc2, are both required for cardiovascular development and somitogenesis. Genes Dev 15: 2470-2482.
10. Seo S, Fujita H, Nakano A, Kang M, Duarte A, et al. (2006) The forkhead transcription factors, Foxc1 and Foxc2, are required for arterial specification and lymphatic sprouting during vascular development. Dev Biol 294: 458-470.
11. Seo S, Kume T (2006) Forkhead transcription factors, Foxc1 and Foxc2, are required for the morphogenesis of the cardiac outflow tract. Dev Biol 296: 421-436.
12. Yamagishi H, Maeda J, Hu T, McAnally J, Conway SJ, et al. (2003) Tbx1 is regulated by tissue-specific forkhead proteins through a common Sonic hedgehog-responsive enhancer. Genes Dev 17: 269-281.
13. Moretti A, Caron L, Nakano A, Lam JT, Bernshausen A, et al. (2006) Multipotent embryonic isl1+ progenitor cells lead to cardiac, smooth muscle, and endothelial cell diversification. Cell 127: 1151-1165.
14. Srivastava D (2006) Making or breaking the heart: from lineage determination to morphogenesis. Cell 126: 1037-1048.
15. Pu WT, Ishiwata T, Juraszek AL, Ma Q, Izumo S (2004) GATA4 is a dosage-sensitive regulator of cardiac morphogenesis. Dev Biol 275: 235-244.
16. Alexandrovich A, Arno M, Patient RK, Shah AM, Pizzey JA, et al. (2006) Wnt2 is a direct downstream target of GATA6 during early cardiogenesis. Mech Dev 123: 297-311.
17. Stennard FA, Costa MW, Elliott DA, Rankin S, Haast SJ, et al. (2003) Cardiac T-box factor Tbx20 directly interacts with Nkx2-5, GATA4, and GATA5 in regulation of gene expression in the developing heart. Dev Biol 262: 206-224.

18. Costantini DL, Arruda EP, Agarwal P, Kim KH, Zhu Y, et al. (2005) The homeodomain transcription factor Irx5 establishes the mouse cardiac ventricular repolarization gradient. Cell 123: 347-358.
19. Frey N, Barrientos T, Shelton JM, Frank D, Rutten H, et al. (2004) Mice lacking calsarcin-1 are sensitized to calcineurin signaling and show accelerated cardiomyopathy in response to pathological biomechanical stress. Nat Med 10: 1336-1343.
20. Schmitt JP, Kamisago M, Asahi M, Li GH, Ahmad F, et al. (2003) Dilated cardiomyopathy and heart failure caused by a mutation in phospholamban. Science 299: 1410-1413.
21. Kaestner KH (2005) The making of the liver: developmental competence in foregut endoderm and induction of the hepatogenic program. Cell Cycle 4: 1146-1148.
22. Lee CS, Friedman JR, Fulmer JT, Kaestner KH (2005) The initiation of liver development is dependent on Foxa transcription factors. Nature 435: 944-947.
23. Minta JO, Fung M, Turner S, Eren R, Zemach L, et al. (1998) Cloning and characterization of the promoter for the human complement factor I (C3b/C4b inactivator) gene. Gene 208: 17-24.
24. Matushansky I, Maki RG, Cordon-Cardo C (2008) A context dependent role for Wnt signaling in tumorigenesis and stem cells. Cell Cycle 7: 720-724.
25. Pearson JC, Lemons D, McGinnis W (2005) Modulating Hox gene functions during animal body patterning. Nat Rev Genet 6: 893-904.
26. Monaghan AP, Kioschis P, Wu W, Zuniga A, Bock D, et al. (1999) Dickkopf genes are co-ordinately expressed in mesodermal lineages. Mech Dev 87: 45-56.
27. Corbo JC, Deuel TA, Long JM, LaPorte P, Tsai E, et al. (2002) Doublecortin is required in mice for lamination of the hippocampus but not the neocortex. J Neurosci 22: 7548-7557.
28. Shan Z, Parker T, Wiest JS (2004) Identifying novel homozygous deletions by microsatellite analysis and characterization of tumor suppressor candidate 1 gene, TUSC1, on chromosome 9p in human lung cancer. Oncogene 23: 6612-6620.
29. Ma X, Wang X, Gao X, Wang L, Lu Y, et al. (2007) Identification of five human novel genes associated with cell proliferation by cell-based screening from an expressed cDNA ORF library. Life Sci 81: 1141-1151.
30. Jung Y, Park J, Bang YJ, Kim TY (2008) Gene silencing of TSPYL5 mediated by aberrant promoter methylation in gastric cancers. Lab Invest 88: 153-160.
31. Plenchette S, Cheung HH, Fong WG, LaCasse EC, Korneluk RG (2007) The role of XAF1 in cancer. Curr Opin Investig Drugs 8: 469-476.
32. Ong CK, Leong C, Tan PH, Van T, Huynh H (2007) The role of 5' untranslated region in translational suppression of OKL38 mRNA in hepatocellular carcinoma. Oncogene 26: 1155-1165.
33. Gustincich S, Vatta P, Goruppi S, Wolf M, Saccone S, et al. (1999) The human serum deprivation response gene (SDPR) maps to 2q32-q33 and codes for a phosphatidylserine-binding protein. Genomics 57: 120-129.
34. Yu TS, Moctezuma-Anaya M, Kubo A, Keller G, Robertson S (2002) The heart LIM protein gene (Hlp), expressed in the developing and adult heart, defines a new tissue-specific LIM-only protein family. Mech Dev 116: 187-192.
35. Kong Y, Shelton JM, Rothermel B, Li X, Richardson JA, et al. (2001) Cardiac-specific LIM protein FHL2 modifies the hypertrophic response to beta-adrenergic stimulation. Circulation 103: 2731-2738.
36. Molkentin JD (2003) A friend within the heart: natriuretic peptide receptor signaling. J Clin Invest 111: 1275-1277.
