# Supplementary material for: Transcriptional and Functional Profiling of Human Embryonic Stem Cell-Derived Cardiomyocytes
Source: PLoS One. 2008 Oct 22;3(10):e3474. doi: 10.1371/journal.pone.0003474 (PMC2565131; doi:10.1371/journal.pone.0003474)
Supplement: Methods S1 — Document contains supplemental methods. (0.11 MB PDF) [file pone.0003474.s002.pdf]

SUPPLEMENTAL METHODS S1

Isolation of human fetal cardiomyocytes from 20-week hearts. Hearts were placed in 10 cm dishes and great vessels, atria, and right ventricle removed. The left ventricle was then submersed in buffer (116 mM NaCl, 20 mM Hepes, 0.8 mM Na2HPO4, 5.6 mM glucose, 5.4 mM KCl, 0.8 mM MgSO4) and minced with forceps and micro-scissors. The resulting cell/tissue suspension was transferred to sterilized 15 ml centrifuge tube containing pre-warmed 10 ml of 0.4 mg/ml Collagenase Type II and agitated at approximately 80 rpms in a 37ºC incubator. After 10 minutes, the suspension is discarded since most cells at this point are erythrocytes. The minced tissue is resuspended in Collagenase Type II followed by collection of the suspension after another 10 mins (repeat this step twice). After each digestion repeat, the cell suspension is placed immediately in 2 ml of neonatal calf serum and centrifuged at 660 rpm for 5 minutes. The resulting pellet is then resuspended in 4 ml of cell medium (85% DMEM + 5% FBS + 10% Horse Serum + 10.000 Units/ml Penicilin/10 mg/ml Streptomycin) and kept at 37ºC. The collected cell-suspensions can be pooled and centrifuged again for 5 minutes at 660 rpm. The pellet is then re-suspended in media and pre-plated for 60 minutes. After pre-plating, fibroblasts will adhere to the plate while cardiomyocytes remain in suspension. The cardiomyocyte-enriched fraction is removed and plated onto 10 cm^2 gelatin-coated plates (4-8 million cells per plate). Cytosine arabinoside · HCl (Sigma Chemical Co., St Louis, MO, U.S.A.) at a final concentration of 10 M in the serum-free medium was added to prevent the growth of any residual fibroblasts as described [1].

RNA quality control. RNA concentration was measured by spectrophotometry, and RNA integrity assessed with an Agilent 2100 bioanalyzer with 6000 Nano Chips. RNA was judged as

suitable for array hybridization only if samples exhibited intact bands corresponding to 18S and 28S ribosomal RNA subunits and had an RNA Integrity Number (RIN) greater than six. A pool of all samples was used as microarray reference controls.

Labeling reaction and hybridization. All sample processing was performed at the same time to negate any potential technical variability. Using Low RNA Input Fluorescent Linear Amplification Kits (Agilent Technologies, Santa Clara, CA, USA), cDNA was reverse transcribed from each RNA sample, and cRNA was then transcribed and fluorescently labeled from each cDNA sample. The 16 cRNA samples representing four biological quadruplicates, as well as the pooled reference control, were labeled with Cy5/Cy3. The resulting cRNA was purified using an RNeasy kit (Qiagen, Valencia, CA, USA) followed by quantification of the cRNA by spectroscopy using an ND-1000 spectrophotometer (NanoDrop Technologies, Wilmington, DE, USA). 825 ng of Cy3- and Cy5- labeled and amplified cRNA was mixed and fragmented according to the Agilent technology protocol. cRNA was hybridized to 4x44K whole human genome microarray slides from Agilent (Part G4112F) according to the manufacturer's instructions. The hybridization was carried in a rotating hybridization chamber in the dark at 65°C for 17 h.

RT-PCR primer sets used for transcriptional analysis. The primer sets used in the amplification reaction are as follows:

Human Oct-4 forward primer: 5'-GGCGTTCTCTTTGCAAAGGTGTTC-3'

Human Oct-4 reverse primer: 5'-CTCGAACCACATCCTTCTCT -3'

Human Nanog forward primer: 5'-GGCGTTCTCTTTGCAAAGGTGTTC-3'

Human Nanog reverse primer: 5'-CTCGAACCACATCCTTCTCT -3'

Human Rex-1 forward primer: 5'-CGTACGCAAATTAAAGTCCAGA-3'

Human Rex-1 reverse primer: 5'-CAGCATCCTAAACAGCTCGCAGAAT-3'

Human NeuroD forward primer: 5'-AAGCCATGAACGCAGAGGAGGACT-3'

Human NeuroD reverse primer: 5'-AGCTGTCCATGGTACCGTAA-3'

Human AFP forward primer: 5'-AGAACCTGTCACAAGCTGTG-3'

Human AFP reverse primer: 5'-GACAGCAAGCTGAGGATGTC-3'

Human Nkx2.5 forward primer: 5'-CTTCAAGCCAGAGGCCTACG-3'

Human Nkx2.5 reverse primer: 5'-CCGCCTCTGTCTTCTTCAGC-3'

Human α-MHC forward primer: 5'-GTCATTGCTGAAACCGAGAATG-3'

Human α-MHC reverse primer: 5'-GCAAAGTACTGGATGACACGCT-3'

Human ANP forward primer: 5'-GAACCAGAGGGGAGAGAC AGAG-3'

Human ANP reverse primer: 5'-CCCTCAGCTTGCTTTTTAGGAG-3'

Fluc forward primer: 5'-ATCTACTGGTCTGCCTAAAG-3'

Fluc reverse primer: 5'-CAGCTCTTCTTCAAATCTATAC-3'

Human GAPDH forward primer: 5'-TGAAGGTCGGAGTCAACGGATTTGGT-3'

Human GAPDH reverse primer: 5'-CATGTGGGCCATGAGGTCCACCAC-3'

Quantitation of scar formation. Hearts subjected to I/R injury were perfusion fixed in 10% neutral buffered formalin at 2, 4, 6, 8 weeks after I/R injury. Two 5-μm sections from each paraffin-embedded slice (i.e., basal and apical) were obtained (one just below the ligature and the other from the basal side of the apical part of LV) and were stained with hematoxylin/eosin. Scar and spared myocardium area were assessed and calculated using NIH Image J software. Infarct

area was calculated as the ratio between scar area and the area of the whole-LV section and expressed in percentages. Measurements obtained in the two representative sections of each heart were averaged.

Quantitative analysis of the endothelial cell marker (mouse CD31) positive capillary density in ischemic hearts at week 8. Capillary densities were examined by counting the number of capillaries stained with anti-CD31 in five random fields on two different sections (approximately 2mm apart) from each mouse. Images were analyzed using Image J software as described [2].

Induction of hypoxia and angiogenesis antibody array. The effect of angiogenesis induction was analyzed by comparing the expression of angiogenesis inhibitors and activators in media from cultured hESC-CMs exposed to hypoxia versus normoxia. Plates with hESC-CMs were placed in a humidified Billups-Rothenberg modular incubation chamber (model MIC-101, Billups-Rothenberg, Del Mar, CA), charged with a gas mixture of 1% O_2/5% CO_2/94% N_2 and sealed prior to placement into a tissue culture incubator set at 37ºC. Hypoxic exposure was carried out for 12 hours and control hESCs were kept alongside at ambient oxygen concentrations. Culture medium was harvested following hypoxic exposure, and total cell protein was analyzed with the Bio-Rad protein assay. Cytokines were measured by the TranSignal™ Human Angiogenesis Antibody Array (Panomics, Fremont, CA). The following angiogenic growth factors were evaluated: angiogenin (ang), basic fibroblast growth factor (bFGF), FGF-α, interleukin 6 (IL-6), IL-8, hepatocyte growth factor (HGF), tumor necrosis factor alpha (TNF-α), and vascular endothelial growth factor (VEGF).

Electrophysiology methods. Cells were grown on mouse embryonic fibroblasts (MEFs) pre-treated with 10 µg/ml mitomycin C (Sigma; St Louis, MO, USA) for 3 hr, as previously described [3,4]. Culture medium consisted of DMEM (Invitrogen, Carlsbad, CA, USA) containing 2mM l-glutamine (Invitrogen, Carlsbad, CA, USA), insulin-transferrin-selenium (Invitrogen, Carlsbad, CA, USA), non-essential amino acids (Invitrogen, Carlsbad, CA, USA), β-mercaptoethanol (Gibco, Carlsbad, CA, USA), 20 U/ml penicillin (Invitrogen, Carlsbad, CA, USA), 20 µg/mL streptomycin (Invitrogen, Carlsbad, CA, USA), and 20% fetal calf serum (FCS) (Hyclone, Logan, UT). The HES2 line was passaged manually ("cut-and-paste"), by cutting colony pieces and removing them from the MEFs using dispase (10 mg/mL) for 2 min. The resulting HES2 colony pieces were washed twice in PBS then placed on fresh MEFs. For differentiation to cardiomyocytes, co-culture of the HES2 and END2 cells was carried out as described previously [3,4]. In brief, END2 cells were grown to 100% confluence and treated with 10 µg/mL mitomycin C (Sigma; St Louis, MO.) for 3 hr. Undifferentiated HES2 cells were removed from the MEFs using 10mg/mL dispase (Invitrogen, Carlsbad, CA). Colonies were then washed twice with PBS, resuspended in hESC media and broken into pieces by repeated pipeting. These pieces were then transferred to the END2 cell layer and incubated at 37ºC for 2 - 3 weeks in hESC medium lacking serum. The co-cultures were refreshed with medium lacking serum every 4–5 days. Areas of beating cardiomyocytes were scored by visual examination from day 7 onwards.

SUPPLEMENTAL REFERENCES

1. Haddad J, Decker ML, Hsieh LC, Lesch M, Samarel AM, et al. (1988) Attachment and maintenance of adult rabbit cardiac myocytes in primary cell culture. American Journal of Physiology 255: C19-27.
2. Yamahara K, Sone M, Itoh H, Yamashita JK, Yurugi-Kobayashi T, et al. (2008) Augmentation of neovascularizaiton in hindlimb ischemia by combined transplantation of human embryonic stem cells-derived endothelial and mural cells. PloS ONE 3: e1666.
3. Moore JC, van Laake LW, Braam SR, Xue T, Tsang SY, et al. (2005) Human embryonic stem cells: genetic manipulation on the way to cardiac cell therapies. Reprod Toxicol 20: 377-391.
4. Mummery C, Ward-van Oostwaard D, Doevendans P, Spijker R, van den Brink S, et al. (2003) Differentiation of human embryonic stem cells to cardiomyocytes: role of coculture with visceral endoderm-like cells. Circulation 107: 2733-2740.
