# Supplementary figures and images for: Transcriptional and Functional Profiling of Human Embryonic Stem Cell-Derived Cardiomyocytes
Source: PLoS One. 2008 Oct 22;3(10):e3474. doi: 10.1371/journal.pone.0003474 (PMC2565131; doi:10.1371/journal.pone.0003474)

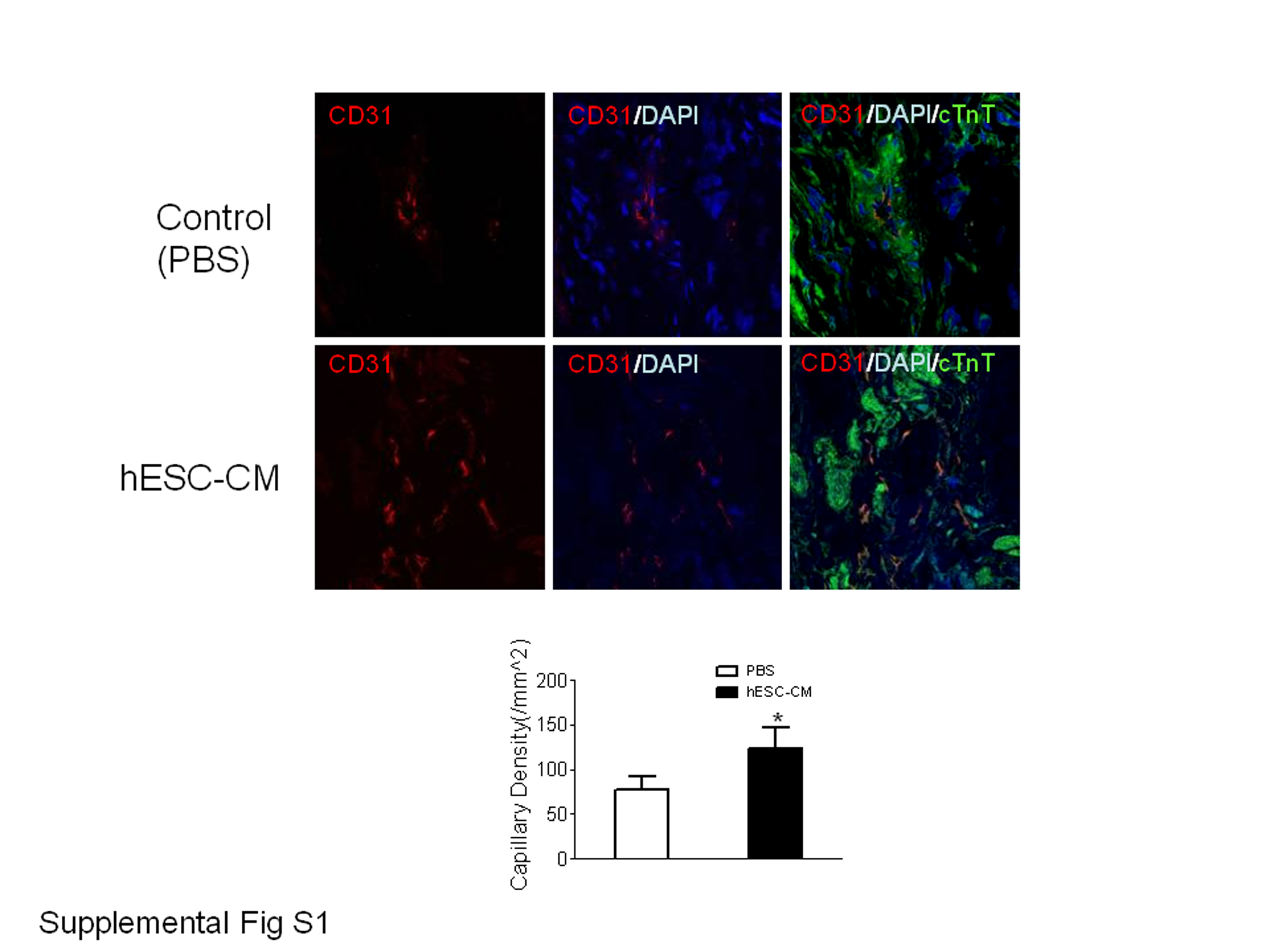

Supplement: Figure S1 — Quantitative analysis of the endothelial cell marker CD31 (mouse) shows upregulation of capillary density in ischemic hearts at week 8. The hESC-CM-treated group showed significant augmentation of CD31 positive capillary density (P<0.05). Capillary densities were examined by counting the number of capillaries stained with anti-CD31 in five random fields on two different sections (approximately 3 mm apart) from each mouse. Images were analyzed using Image J software. (2.08 MB TIF) [file pone.0003474.s003.tif]

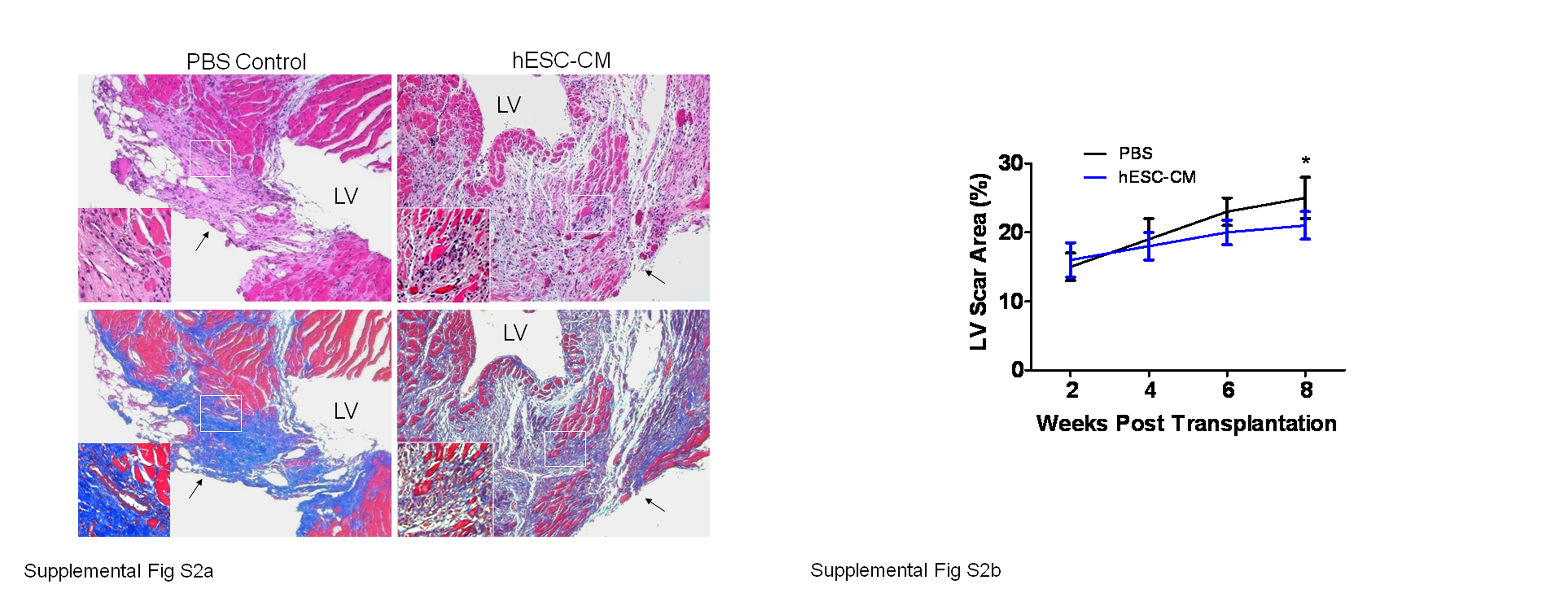

Supplement: Figure S2 — Ventricular scar formation after hESC-CM transplantation. (a) Histological evaluation of infarct fibrosis reveals attenuation of scar in a representative animal treated with hESC-CMs (right panels) as compared with a representative animal receiving PBS alone (left panels) at 8 weeks post-transplantation. Masson's Trichrome stain (bottom panels) produces blue connective tissue and red muscle fibers to allow easy identification of the fibrotic scar resulting from ischemia reperfusion injury. (b) The quantified infarct sizes (percent of LV) in hESC-CM-treated mice and PBS controls were 21%±3% (n = 6) and 25%±2% (n = 6) (P = 0.041), respectively. Scale bars = 10 µm. (40.89 MB TIF) [file pone.0003474.s004.tif]

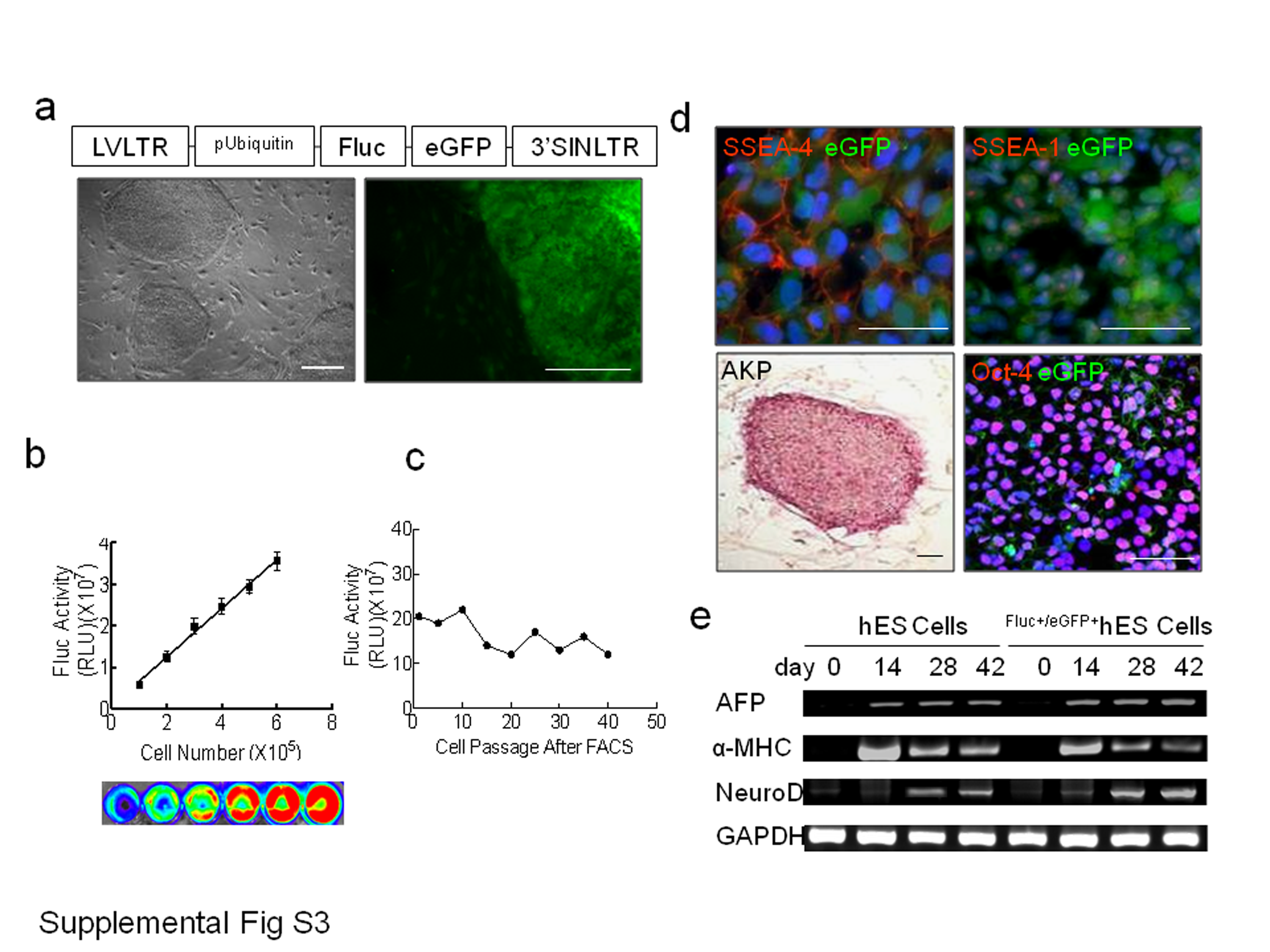

Supplement: Figure S3 — Stable lentiviral transduction of hESCs with double fusion (DF) reporter gene. (a) Schema of the DF reporter gene containing Fluc and eGFP with brightfield (left) and fluorescent (right) images of Fluc+/eGFP+hESCs (scale bars = 200 µm). (b) Stably transduced Fluc+/eGFP+hESCs (collected by FACS) show robust correlation between cell number and reporter gene activity. Raw bioluminescence images of increasing numbers of Fluc+/eGFP+hESCs in vitro are shown below graph. (c) Fluc+/eGFP+hESCs maintain firefly luciferase activity over successive passages. (d) Fluc+/eGFP+hESCs maintain pluripotent stem cell markers such as SSEA-4, Oct-4, and AKP, but remain negative for differentiation marker SSEA-1. Scale bars = 50 µm. (e) RT-PCR analysis of embryoid bodies over the course of 7 weeks shows expression of endodermal (AFP), mesodermal (αMHC), and ectodermal (NeuroD) germ layer markers for both control non-transduced hESCs and Fluc+/eGFP+hESCs. GAPDH is used as loading control. (3.70 MB TIF) [file pone.0003474.s005.tif]

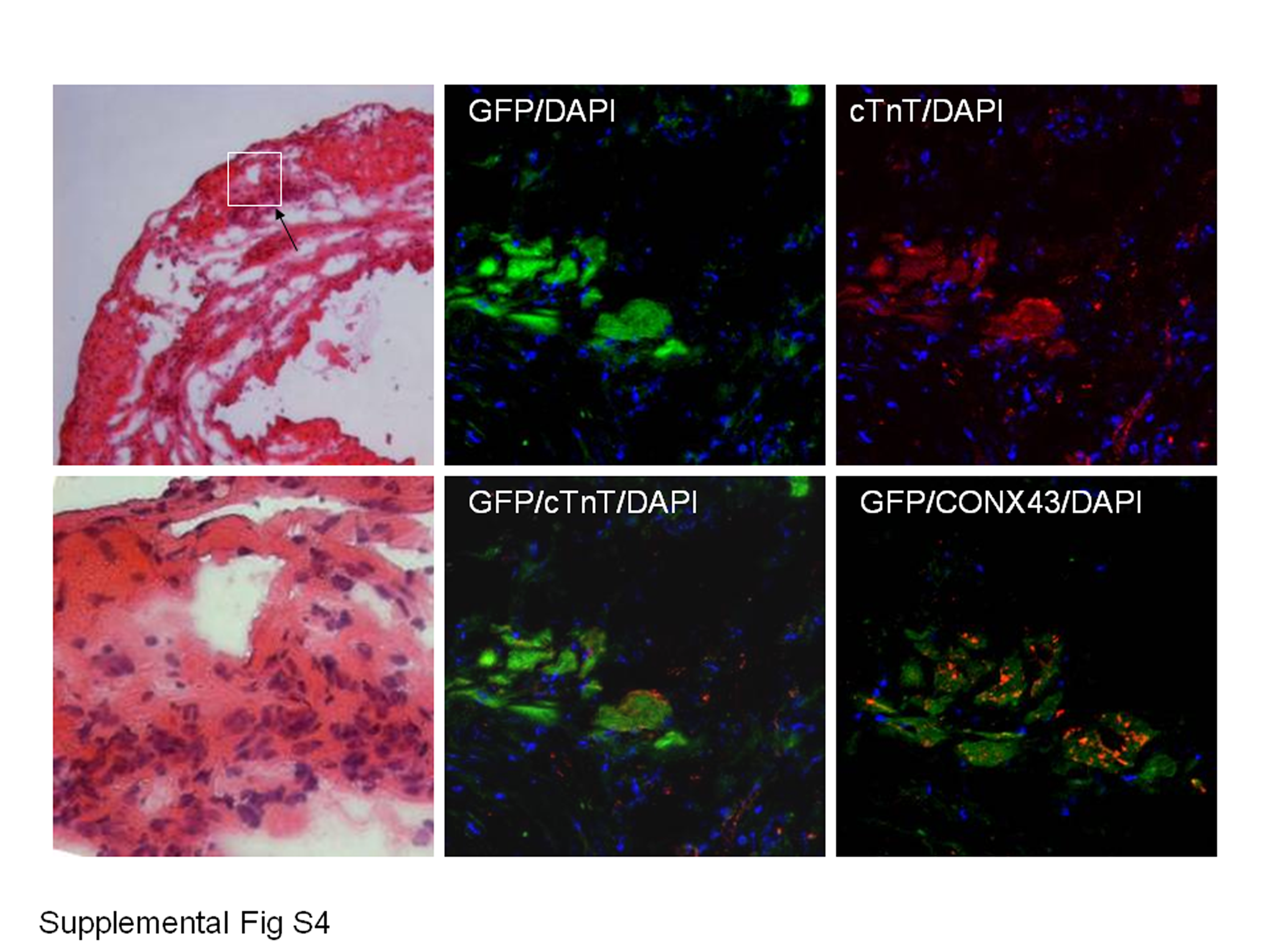

Supplement: Figure S4 — Histopathological evaluation demonstrates that hESC-CMs do not integrate into host myocardium but continue to express cardiac markers. Representative histopathological images of explanted hearts taken two months after Fluc+/eGFP+hESC-CM delivery. GFP positive cells (transplanted Fluc+/eGFP+hESC-CMs) express cardiac troponin-T and connexin-43, but do not appear to be well integrated with the surrounding host myocardium. (5.26 MB TIF) [file pone.0003474.s006.tif]

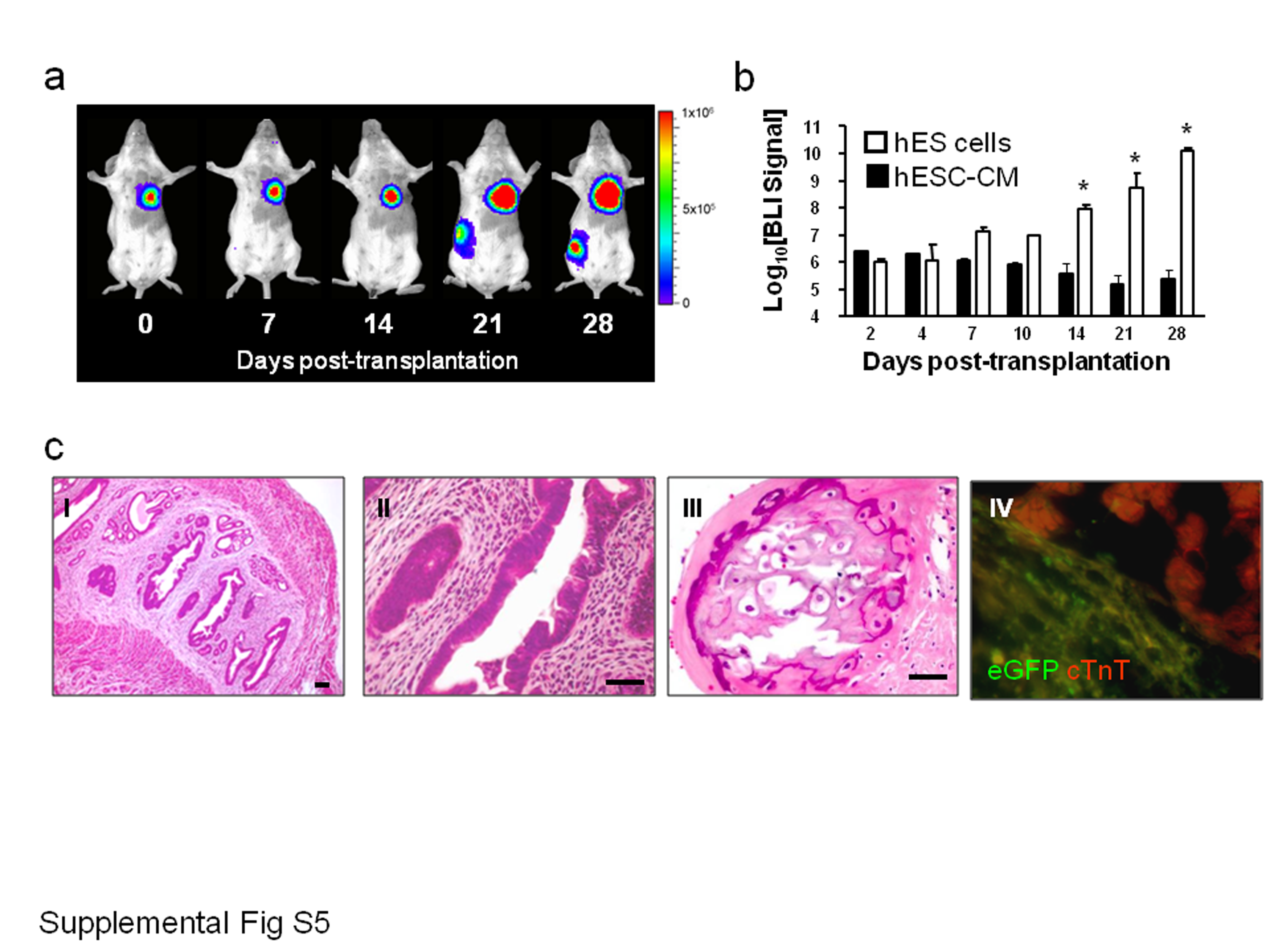

Supplement: Figure S5 — Bioluminescence imaging and histological fate of undifferentiated Fluc+/eGFP+hES cells transplanted into the heart. (a) Representative images from a single animal receiving one million undifferentiated Fluc+/eGFP+hES cells. Undifferentiated hES cells rapidly form teratomas with extra-cardiac spread within 3 to 4 weeks of transplantation. (b) Quantification of imaging signals from animals receiving undifferentiated Fluc+/eGFP+hESCs (n = 6) or Fluc+/eGFP+hESC-CMs (n = 15) shows logarithmic increases in BLI signals in the undifferentiated group (*P<0.001) vs. the hESC-CM group due to teratoma formation. (c) Histology demonstrating typical teratoma formation in the heart following transplantation of undifferentiated Fluc+/eGFP+hES cells. Histological features of low-power field of teratoma (I), respiratory epithelium (II), and cartilage formation (III) can be identified (scale bars = 50 µm). The border of the graft area shows that only host myocardium stains positive for cardiac markers such as cardiac troponin-T (cTnT), while cardiac markers are absent from the eGFP+ region (IV). (3.84 MB TIF) [file pone.0003474.s007.tif]

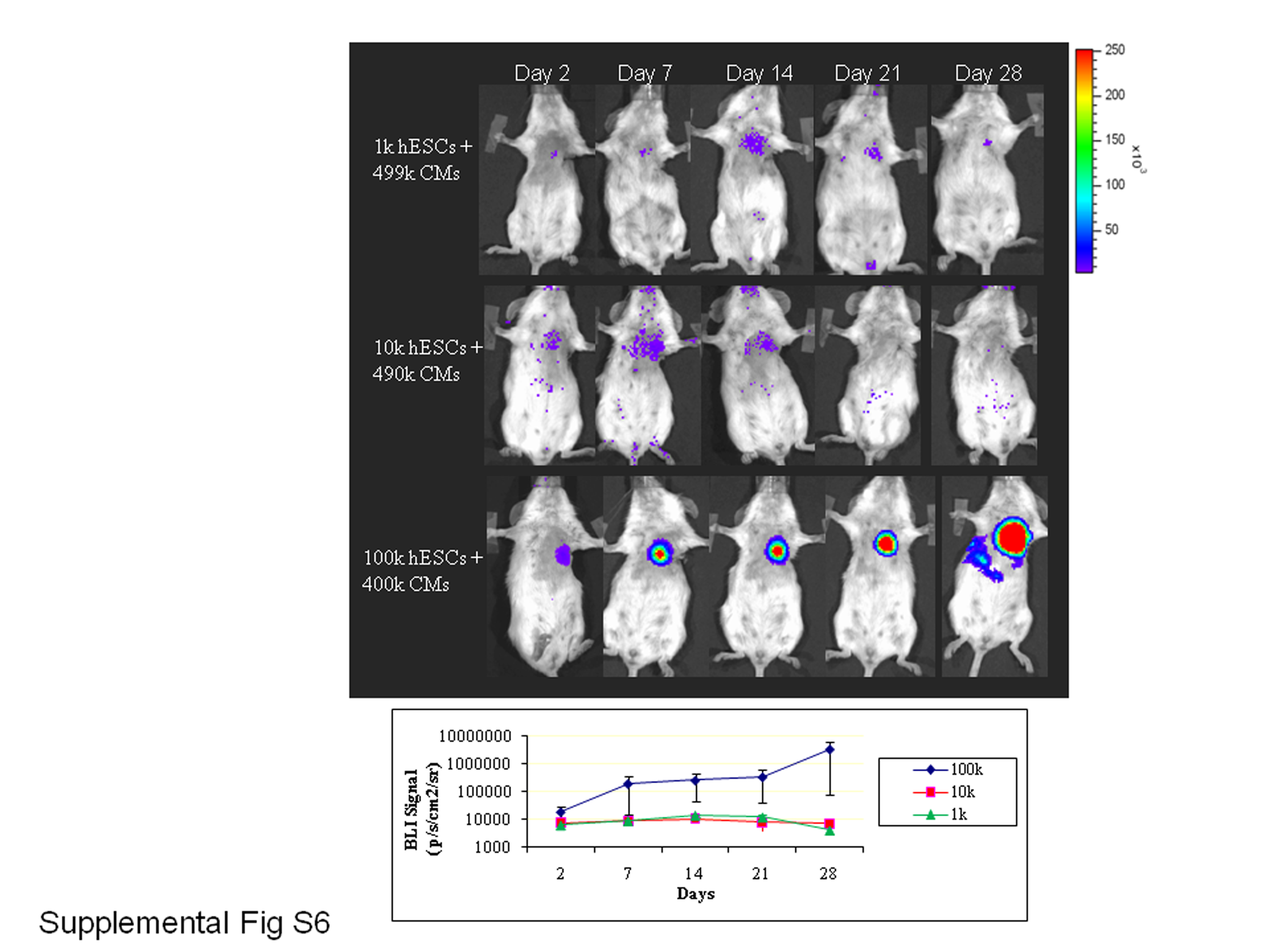

Supplement: Figure S6 — Bioluminescence imaging of undifferentiated Fluc+/eGFP+hESCs mixed with non-transduced hESC-derived cardiomyocytes after transplantation to SCID mouse heart. This study represents a clinically relevant scenario in which undifferentiated hESC contaminants are mixed in with the hESC-CM population. We observed teratoma formation in the 100 k hESC contaminant group, but not in the 10 k or 1 k hESC groups. Data presented as mean±SEM. (2.41 MB TIF) [file pone.0003474.s008.tif]

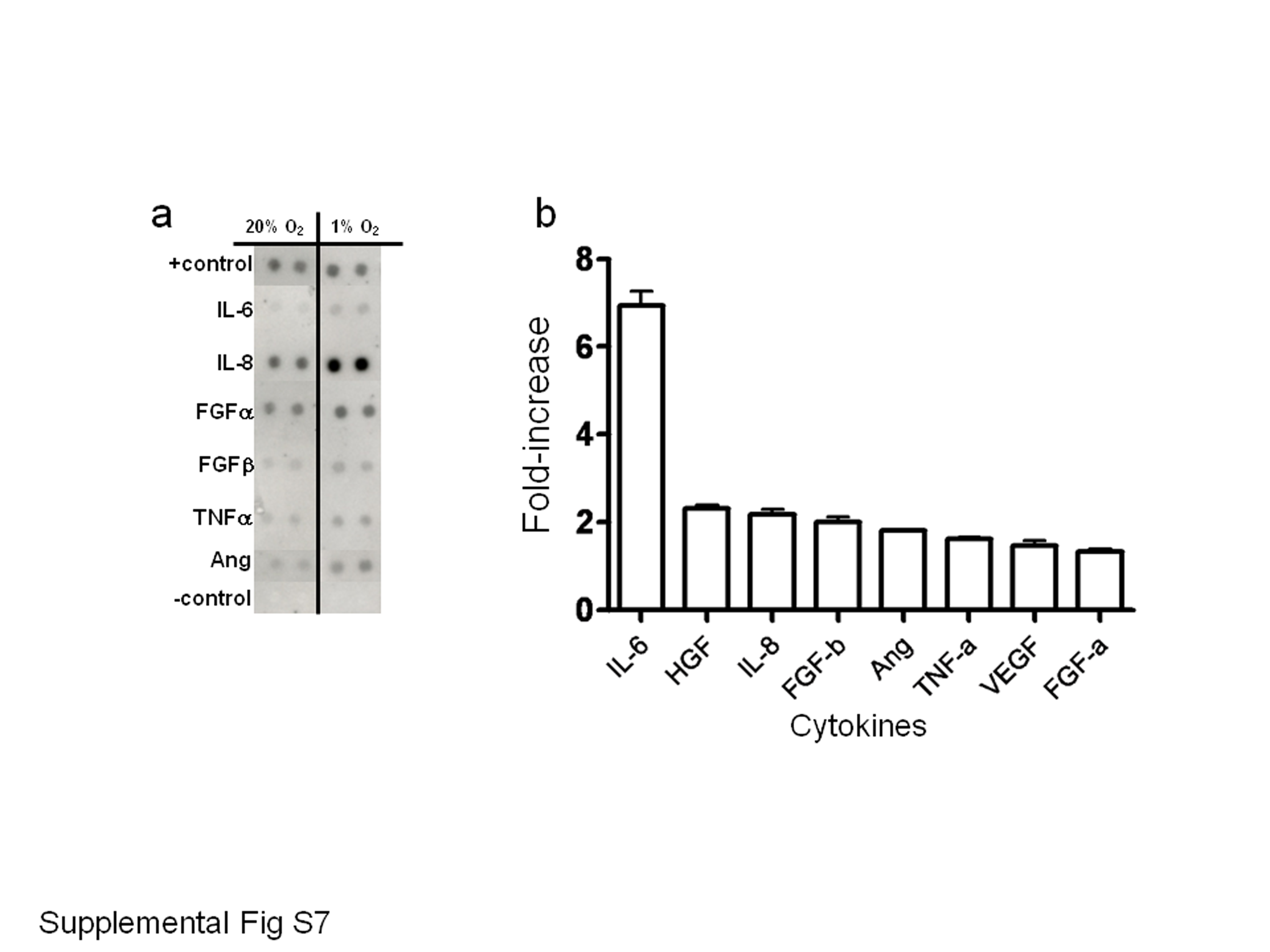

Supplement: Figure S7 — Fluc+/eGFP+hESC-CMs upregulate secretion of angiogenic growth factors under hypoxic conditions. (a) Culture media from hESC-CMs under hypoxia (1% O2/5% CO2/94% N2) or normoxia (20% O2/5% CO2) was washed over an antibody array to assess angiogenic protein secretion levels. (b) Hypoxia induces significant up-regulation of multiple cytokines by Fluc+/eGFP+hESC-CMs. Following 12 hours of hypoxia in vitro, media from Fluc+/eGFP+hESC-CMs had increased levels of FGF, IL-6, IL-8 and VEGF as compared to cells maintained in normoxic conditions. (0.81 MB TIF) [file pone.0003474.s009.tif]
