# Supplementary material for: Transcriptional and Functional Profiling of Human Embryonic Stem Cell-Derived Cardiomyocytes
Source: PLoS One. 2008 Oct 22;3(10):e3474. doi: 10.1371/journal.pone.0003474 (PMC2565131; doi:10.1371/journal.pone.0003474)
Supplement: Table S1 — Microarray data tables of differentially-regulated genes. (13.12 MB PDF) [file pone.0003474.s010.pdf]

Supplemental Table A1

Genes Downregulated in EB vs ES (2219) (High expression in hESC)

SAM FDR 0.1%

		Fold				
**Gene Name**	**Score(d)**	**Down EB**	**Description**	**Acc**	**UGCluster**	**UGRepAcc**
L1TD1	-31.6	32.2	LINE-1 type transposase domain containing 1 (L1TD1), mRNA	NM_019079	Hs.562195	NM_019079
THC2537217	-25.1	19.5	THC2537217	THC2537217	Unknown	
AL832758	-19.8	6.1	mRNA; cDNA DKFZp686C0927 (from clone DKFZp686C0927).	AL832758	Hs.555970	BX648788
BICD1	-18.2	3.2	bicaudal D homolog 1 (Drosophila), mRNA (cDNA clone IMAGE:3050215), with apparent retained intron.	BC010091	Unknown	
DB362335	-17.2	15.9	DB362335 NT2RM4 cDNA clone NT2RM4000005 3', mRNA sequence	DB362335	Hs.653872	BX383015
CECR2	-17.1	3.2	cDNA FLJ34435 fis, clone HLUNG2000955.	AK091754	Hs.658723	NM_031413
THC2626182	-15.6	2.6	THC2626182	THC2626182	Unknown	
CLDN10	-15.1	12.9	claudin 10 (CLDN10), transcript variant 1, mRNA	NM_182848	Hs.534377	NM_182848
PHC1	-15.1	6.3	polyhomeotic homolog 1 (Drosophila) (PHC1), mRNA	NM_004426	Hs.305985	NM_004426
MAP7	-14.9	8.3	microtubule-associated protein 7 (MAP7), mRNA	NM_003980	Hs.486548	NM_003980
HTATIP2	-14.1	9.3	HIV-1 Tat interactive protein 2, 30kDa (HTATIP2), mRNA	NM_006410	Hs.90753	NM_001098522
C1orf38	-14.0	8.3	chromosome 1 open reading frame 38, mRNA (cDNA clone IMAGE:5170350), complete cds.	BC031655	Hs.10649	AB050854
PYCARD	-14.0	5.6	PYD and CARD domain containing (PYCARD), transcript variant 1, mRNA	NM_013258	Hs.499094	BM918855
A_24_P158193	-14.0	29.6	A_24_P158193	A_24_P158193	Unknown	
BC038245	-13.7	3.9	Homo sapiens, clone IMAGE:5241654, mRNA.	BC038245	Hs.335413	BC038245
B3GAT1	-13.7	3.3	beta-1,3-glucuronyltransferase 1 (glucuronosyltransferase P) (B3GAT1), transcript variant 2, mRNA	NM_054025	Hs.381050	BC010466
GPRC5B	-13.7	4.1	G protein-coupled receptor, family C, group 5, member B (GPRC5B), mRNA	NM_016235	Hs.148685	NM_016235
KLHL7	-13.5	4.5	kelch-like 7 (Drosophila), mRNA (cDNA clone IMAGE:3899090), complete cds.	BC009555	Hs.654817	NM_018846
THC2710780	-13.2	12.7	THC2710780	THC2710780	Unknown	
SCG3	-13.2	14.0	secretogranin III (SCG3), mRNA	NM_013243	Hs.232618	NM_013243
MARVELD3	-13.0	12.1	MARVEL domain containing 3 (MARVELD3), transcript variant 1, mRNA	NM_001017967	Hs.513706	NM_001017967
ZNF398	-13.0	2.9	zinc finger protein 398 (ZNF398), transcript variant 2, mRNA	NM_020781	Hs.654721	NM_020781
LARP2	-12.7	3.3	La ribonucleoprotein domain family, member 2 (LARP2), transcript variant 2, mRNA	NM_178043	Hs.657067	NM_018078
TNFRSF8	-12.7	22.3	tumor necrosis factor receptor superfamily, member 8 (TNFRSF8), transcript variant 1, mRNA	NM_001243	Hs.1314	NM_001243
CRIP3	-12.7	7.8	cysteine-rich protein 3 (CRIP3), mRNA	NM_206922	Hs.653165	AK124657
CLDN6	-12.5	6.1	claudin 6 (CLDN6), mRNA	NM_021195	Hs.533779	NM_021195
FAM124A	-12.2	16.2	family with sequence similarity 124A, mRNA (cDNA clone IMAGE:4836906), complete cds.	BC034497	Hs.71913	BC051771
PHF17	-12.1	3.3	PHD finger protein 17 (PHF17), transcript variant L, mRNA	NM_199320	Hs.12420	NM_199320
MTAC2D1	-12.1	5.6	membrane targeting (tandem) C2 domain containing 1 (MTAC2D1), mRNA	NM_152332	Hs.510262	BC040503
SLC25A21	-12.0	2.7	solute carrier family 25 (mitochondrial oxodicarboxylate carrier), member 21 (SLC25A21), mRNA	NM_030631	Hs.693866	AJ278148
FLJ44186	-12.0	29.4	FLJ44186 protein, mRNA (cDNA clone MGC:131846 IMAGE:6139360), complete cds.	BC110858	Hs.17572	BC110858
ACSBG1	-12.0	12.9	acyl-CoA synthetase bubblegum family member 1 (ACSBG1), mRNA	NM_015162	Hs.655760	NM_015162
THC2645710	-12.0	86.6	THC2645710	THC2645710	Unknown	
KRTCAP3	-12.0	13.4	clone DNA129535 MRV222 (UNQ3066) mRNA, complete cds.	AY358993	Hs.59509	AY358993
ACOXL	-11.9	13.3	acyl-Coenzyme A oxidase-like (ACOXL), mRNA	NM_018308	Hs.253320	BC022268
EDG7	-11.8	6.4	Lysophosphatidic acid receptor Edg-7 (LPA receptor 3) (LPA-3).	ENST00000370	Unknown	
PTPRZ1	-11.7	5.5	protein tyrosine phosphatase, receptor-type, Z polypeptide 1 (PTPRZ1), mRNA	NM_002851	Hs.489824	NM_002851
STYX	-11.7	3.8	serine/threonine/tyrosine interacting protein (STYX), mRNA	NM_145251	Hs.364980	BX647636
ABHD9	-11.6	4.4	abhydrolase domain containing 9 (ABHD9), mRNA	NM_024794	Hs.156457	AK026061
A_32_P232647	-11.5	5.8	A_32_P232647	A_32_P232647	Unknown	
BC013077	-11.5	3.7	Homo sapiens, clone IMAGE:3459334, mRNA.	BC013077	Hs.594275	BQ214382
GPR114	-11.5	8.6	G protein-coupled receptor 114 (GPR114), mRNA	NM_153837	Hs.187884	AK160368
LOC388494	-11.5	3.8	full-length cDNA clone CS0DF014YD20 of Fetal brain of (human).	CR593500	Hs.65750	NM_015174
PIF1	-11.4	4.4	PIF1 5'-to-3' DNA helicase homolog (S. cerevisiae) (PIF1), mRNA	NM_025049	Hs.112160	AB185926
RBM35B	-11.4	5.0	RNA binding motif protein 35B (RBM35B), mRNA	NM_024939	Hs.592053	NM_024939
OGFOD1	-11.4	3.0	2-oxoglutarate and iron-dependent oxygenase domain containing 1 (OGFOD1), mRNA	NM_018233	Hs.231883	NM_018233
CAMKV	-11.4	5.8	CaM kinase-like vesicle-associated (CAMKV), mRNA	NM_024046	Hs.145156	AL833943
CYP26A1	-11.3	6.8	cytochrome P450, family 26, subfamily A, polypeptide 1 (CYP26A1), transcript variant 2, mRNA	NM_057157	Hs.150595	AK027560
MICB	-11.3	3.6	MHC class I polypeptide-related sequence B (MICB), mRNA	NM_005931	Hs.211580	BC044218
MTL5	-11.1	5.9	metallothionein-like 5, testis-specific (tesmin) (MTL5), transcript variant 2, mRNA	NM_001039656	Hs.145932	AK128308

IGSF21	-11.1	10.2	immunoglobin superfamily, member 21 (IGSF21), mRNA	NM_032880	Hs.212511	AK092618
LOC91664	-11.1	2.8	Homo sapiens, Similar to zinc finger protein 268, clone IMAGE:3352268, mRNA, partial cds.	BC007307	Hs.660790	BC007307
THC2529323	-11.0	28.9	THC2529323	THC2529323	Unknown	
PPP2R2B	-10.9	4.8	protein phosphatase 2 (formerly 2A), regulatory subunit B (PR 52), beta isoform (PPP2R2B), transcript variant 1, mRNA	NM_004576	Hs.655213	M64930
DPPA4	-10.9	15.7	developmental pluripotency associated 4 (DPPA4), mRNA	NM_018189	Hs.317659	NM_018189
ZNF165	-10.9	2.9	zinc finger protein 165 (ZNF165), mRNA	NM_003447	Hs.535177	NM_003447
RAB42	-10.8	3.0	RAB42, member RAS oncogene family (RAB42), mRNA	NM_152304	Hs.652321	BU162527
CKMT1A	-10.7	6.6	creatine kinase, mitochondrial 1A (CKMT1A), nuclear gene encoding mitochondrial protein, mRNA	NM_001015001	Hs.425633	AK094322
USP32	-10.7	3.4	ubiquitin specific peptidase 32 (USP32), mRNA	NM_032582	Hs.132868	NM_032582
BM928667	-10.7	3.7	AGENCOURT_6726860 NIH_MGC_100 cDNA clone IMAGE:5798808 5', mRNA sequence	BM928667	Hs.113170	BM928667
TACSTD1	-10.6	3.0	tumor-associated calcium signal transducer 1 (TACSTD1), mRNA	NM_002354	Hs.699160	AK026585
FOXA3	-10.6	5.8	forkhead box A3 (FOXA3), mRNA	NM_004497	Hs.36137	NM_004497
HLA-DPB2	-10.6	46.6	major histocompatibility complex, class II, DP beta 2 (pseudogene), mRNA (cDNA clone MGC:24119 IMAGE:4663904), complete cds.	BC017967	Hs.665450	BC017967
AF268617	-10.4	160.3	POU 5 domain protein (POU5FLC12) mRNA, complete cds.	AF268617	Hs.646545	XR_019128
HPS3	-10.4	2.4	Hermansky-Pudlak syndrome 3 (HPS3), mRNA	NM_032383	Hs.591311	NM_032383
C1orf182	-10.3	2.6	chromosome 1 open reading frame 182 (C1orf182), mRNA	NM_144627	Hs.534539	BQ072165
PCOLCE2	-10.3	3.2	procollagen C-endopeptidase enhancer 2 (PCOLCE2), mRNA	NM_013363	Hs.8944	AY358557
C1orf38	-10.3	9.1	chromosome 1 open reading frame 38 (C1orf38), transcript variant 2, mRNA	NM_001039477	Hs.10649	AB050854
FLJ14712	-10.3	3.2	cDNA FLJ14712 fis, clone NT2RP3000825, weakly similar to NEUROGENIC LOCUS NOTCH 3 PROTEIN.	AK027618	Hs.669526	AK027618
KIF5C	-10.2	1.9	kinesin family member 5C (KIF5C), mRNA	NM_004522	Hs.660699	NM_004522
TIMM8A	-10.2	3.4	translocase of inner mitochondrial membrane 8 homolog A (yeast) (TIMM8A), nuclear gene encoding mitochondrial protein, mRNA	NM_004085	Hs.447877	CB959370
PRDM14	-10.2	153.4	PR domain containing 14 (PRDM14), mRNA	NM_024504	Hs.287532	NM_024504
TMEM102	-10.2	3.4	transmembrane protein 102 (TMEM102), mRNA	NM_178518	Hs.655662	BC046189
POU5F1	-10.1	148.0	POU domain, class 5, transcription factor 1 (POU5F1), transcript variant 1, mRNA	NM_002701	Hs.249184	DQ486514
GTF2E1	-10.1	2.3	general transcription factor IIE, polypeptide 1 (alpha subunit, 56kD) (GTF2E1), mRNA	NM_005513	Hs.445272	NM_005513
HPS3	-10.1	2.5	Hermansky-Pudlak syndrome 3 (HPS3), mRNA	NM_032383	Hs.591311	NM_032383
SEMA6A	-10.0	5.5	sema domain, transmembrane domain (TM), and cytoplasmic domain, (semaphorin) 6A (SEMA6A), mRNA	NM_020796	Hs.156967	BC032619
TNFRSF11A	-10.0	11.0	tumor necrosis factor receptor superfamily, member 11a, NFKB activator (TNFRSF11A), mRNA	NM_003839	Hs.204044	AK125052
GRPEL2	-9.9	2.6	GrpE-like 2, mitochondrial (E. coli) (GRPEL2), nuclear gene encoding mitochondrial protein, mRNA	NM_152407	Hs.511816	NM_152407
LRRC2	-9.9	16.2	leucine rich repeat containing 2 (LRRC2), mRNA	NM_024750	Hs.657345	NM_024512
GYLTL1B	-9.8	5.3	glycosyltransferase-like 1B (GYLTL1B), mRNA	NM_152312	Hs.86543	NM_152312
ATPBD4	-9.8	5.2	ATP binding domain 4 (ATPBD4), mRNA	NM_080650	Hs.107196	BX537862
CDC42BPG	-9.7	4.7	CDC42 binding protein kinase gamma (DMPK-like) (CDC42BPG), mRNA	NM_017525	Hs.293590	AY648038
CHST6	-9.7	6.2	carbohydrate (N-acetylglucosamine 6-O) sulfotransferase 6 (CHST6), mRNA	NM_021615	Hs.655622	NM_021615
FRAT2	-9.7	5.1	frequently rearranged in advanced T-cell lymphomas 2 (FRAT2), mRNA	NM_012083	Hs.140720	NM_012083
SCGB3A2	-9.7	36.8	secretoglobin, family 3A, member 2 (SCGB3A2), mRNA	NM_054023	Hs.483765	BI518636
AK096991	-9.7	18.3	cDNA FLJ39672 fis, clone SMINT2009233.	AK096991	Hs.488591	AK126044
THC2648753	-9.6	4.6	THC2648753	THC2648753	Unknown	
MARVELD2	-9.6	4.1	MARVEL domain containing 2 (MARVELD2), transcript variant 2, mRNA	NM_144724	Hs.657687	AK055094
TPMT	-9.5	2.6	thiopurine S-methyltransferase (TPMT), mRNA	NM_000367	Hs.444319	NM_000367
PYDC1	-9.5	6.3	PYD (pyrin domain) containing 1 (PYDC1), mRNA	NM_152901	Hs.58314	CA489551
LARS2	-9.4	2.3	leucyl-tRNA synthetase 2, mitochondrial (LARS2), nuclear gene encoding mitochondrial protein, mRNA	NM_015340	Hs.526975	BC025989
A_23_P99731	-9.4	3.0	A_23_P99731	A_23_P99731	Unknown	
L2HGDH	-9.4	3.1	L-2-hydroxyglutarate dehydrogenase (L2HGDH), nuclear gene encoding mitochondrial protein, mRNA	NM_024884	Hs.256034	NM_024884
ENST00000270[illegible]	-9.4	2.9	Nucleolar preribosomal-associated protein 1 (Fragment).	ENST00000270[illegible]	Unknown	
C9orf135	-9.4	25.4	chromosome 9 open reading frame 135 (C9orf135), mRNA	NM_001010940	Hs.444459	NM_001010940
C12orf56	-9.4	6.5	C12orf56 protein (Fragment).	ENST00000333[illegible]	Unknown	
TMEM92	-9.4	3.8	transmembrane protein 92 (TMEM92), mRNA	NM_153229	Hs.224630	AK090637
CEP27	-9.3	2.2	centrosomal protein 27kDa (CEP27), mRNA	NM_018097	Hs.14347	AK022939
ENST00000335[illegible]	-9.3	3.8	Full-length cDNA clone CS0DK001YK16 of HeLa cells of (human) (Fragment).	ENST00000335[illegible]	Unknown	
ALOX12	-9.3	17.2	arachidonate 12-lipoxygenase (ALOX12), mRNA	NM_000697	Hs.654431	NM_000697
LECT1	-9.3	34.2	leukocyte cell derived chemotaxin 1 (LECT1), transcript variant 1, mRNA	NM_007015	Hs.421391	NM_007015
FLJ40432	-9.3	4.3	hypothetical protein FLJ40432 (FLJ40432), mRNA	NM_152523	Hs.471234	BC067253

LOC153346	-9.3	7.5	cDNA FLJ14284 fis, clone PLACE1005898.	AK024346	Hs.483816	BX648997
SMPDL3B	-9.3	9.4	sphingomyelin phosphodiesterase, acid-like 3B (SMPDL3B), transcript variant 2, mRNA	NM_001009568	Hs.123659	NM_014474
MUC3B	-9.2	13.6	Intestinal mucin 3 (Fragment).	ENST00000305[illegible]	Unknown	
ATXN3	-9.2	2.1	ataxin 3 (ATXN3), transcript variant 1, mRNA	NM_004993	Hs.532632	BC095402
AF268613	-9.2	131.6	POU 5 domain protein (POU5FLC1) mRNA, complete cds.	AF268613	Hs.632482	XR_019318
PKMYT1	-9.2	2.7	protein kinase, membrane associated tyrosine/threonine 1 (PKMYT1), transcript variant 2, mRNA	NM_182687	Hs.77783	NM_182687
THC2627432	-9.1	4.4	Q3LEG7_9CHLO (Q3LEG7) Proton-translocating inorganic pyrophosphatase (Fragment) , partial (5%)	THC2627432	Unknown	
LOC440132	-9.1	20.6	AGENCOURT_13976018 NIH_MGC_173 cDNA 5', mRNA sequence	CD050206	Hs.147880	CD050206
THC2623129	-9.1	3.1	ALU2_HUMAN (P39189) Alu subfamily SB sequence contamination warning entry, partial (32%)	THC2623129	Unknown	
PIF1	-9.1	4.2	PIF1 5'-to-3' DNA helicase homolog (S. cerevisiae) (PIF1), mRNA	NM_025049	Hs.112160	AB185926
ELL3	-9.1	4.1	elongation factor RNA polymerase II-like 3 (ELL3), mRNA	NM_025165	Hs.699876	AK126384
IMPA2	-9.1	3.1	inositol(myo)-1(or 4)-monophosphatase 2 (IMPA2), mRNA	NM_014214	Hs.367992	BM924855
LOC730125	-9.1	17.8	cDNA FLJ34228 fis, clone FCBBF3025417.	AK091547	Hs.455955	AK091547
STEAP3	-9.1	5.2	STEAP family member 3 (STEAP3), transcript variant 1, mRNA	NM_182915	Hs.647822	AL833624
EPB41L4B	-9.1	2.9	erythrocyte membrane protein band 4.1 like 4B (EPB41L4B), transcript variant 1, mRNA	NM_018424	Hs.591901	AF153416
CCKBR	-9.1	7.0	cholecystokinin B receptor (CCKBR), mRNA	NM_176875	Hs.203	AF239668
ATP10A	-9.0	6.1	ATPase, Class V, type 10A (ATP10A), mRNA	NM_024490	Hs.659258	AB051358
ZIC5	-9.0	2.8	Zic family member 5 (odd-paired homolog, Drosophila) (ZIC5), mRNA	NM_033132	Hs.508570	NM_033132
MMP25	-9.0	8.9	matrix metallopeptidase 25 (MMP25), mRNA	NM_022468	Hs.654979	AJ272137
ENST00000338[illegible]	-9.0	5.3	AY659951 Fxh variant 1 {Mus musculus} (exp=-1; wgp=0; cg=0), partial (34%)	ENST00000338[illegible]	Unknown	
FLJ20674	-9.0	4.6	CDNA FLJ20674 fis, clone KAIA4450.	ENST00000359[illegible]	Unknown	
P2RX5	-9.0	4.5	purinergic receptor P2X, ligand-gated ion channel, 5 (P2RX5), transcript variant 1, mRNA	NM_002561	Hs.408615	NM_002561
PAICS	-9.0	2.3	phosphoribosylaminoimidazole carboxylase, phosphoribosylaminoimidazole succinocarboxamide synthetase (PAICS), transcript variant 1, mRNA	NM_001079525	Hs.518774	NM_006452
HPS3	-8.9	2.4	Hermansky-Pudlak syndrome 3 (HPS3), mRNA	NM_032383	Hs.591311	NM_032383
SLC15A3	-8.9	3.3	solute carrier family 15, member 3 (SLC15A3), mRNA	NM_016582	Hs.237856	AK127216
ZNF215	-8.9	2.2	zinc finger protein 215 (ZNF215), mRNA	NM_013250	Hs.523457	NM_013250
POU5F1	-8.9	107.4	POU domain, class 5, transcription factor 1 (POU5F1), transcript variant 1, mRNA	NM_002701	Hs.249184	DQ486514
RP11-125A7.3	-8.9	2.0	KIAA0564 protein (KIAA0564), transcript variant 2, mRNA	NM_001009814	Hs.368282	NM_015058
SMCR5	-8.8	9.0	Smith-Magenis syndrome chromosome region candidate 5 protein (SMCR5) mRNA, complete cds.	AF467442	Hs.352643	AF467442
A_24_P715434	-8.8	8.2	A_24_P715434	A_24_P715434	Unknown	
IGF2BP1	-8.8	3.7	insulin-like growth factor 2 mRNA binding protein 1 (IGF2BP1), mRNA	NM_006546	Hs.144936	NM_006546
C13orf25	-8.8	2.1	C13orf25 v_2 mRNA, complete cds, miR-91-precursor-13 micro RNA, microRNA miR-91, microRNA miR-17, miR-18-precursor-13 micro RNA, microRNA miR-18, miR-19a-precursor-13 micro RNA, microRNA miR-19a, microRNA miR-20, miR-19b-precursor-13...	AB176708	Hs.24115	AB176708
WDR62	-8.8	2.5	WD repeat domain 62 (WDR62), mRNA	NM_173636	Hs.116244	NM_001083961
UGT3A2	-8.8	5.3	UDP glycosyltransferase 3 family, polypeptide A2 (UGT3A2), mRNA	NM_174914	Hs.348941	NM_174914
ARG2	-8.8	1.9	arginase, type II (ARG2), nuclear gene encoding mitochondrial protein, mRNA	NM_001172	Hs.699233	BC029050
KCMF1	-8.8	2.0	potassium channel modulatory factor 1 (KCMF1), mRNA	NM_020122	Hs.654968	NM_020122
KIAA1155	-8.8	3.3	cDNA FLJ37016 fis, clone BRACE2010632.	ENST00000244[illegible]	Unknown	
KIAA0241	-8.7	3.5	KIAA0241 (KIAA0241), mRNA	NM_015060	Hs.128056	D87682
OR1D2	-8.7	14.4	olfactory receptor, family 1, subfamily D, member 2 (OR1D2), mRNA	NM_002548	Hs.532771	NM_002548
HLA-DPB2	-8.7	12.3	major histocompatibility complex, class II, DP beta 2 (pseudogene), mRNA (cDNA clone MGC:24119 IMAGE:4663904), complete cds.	BC017967	Hs.665450	BC017967
MOCOS	-8.7	4.5	molybdenum cofactor sulfurase (MOCOS), mRNA	NM_017947	Hs.405028	AK222886
TKT	-8.6	3.5	transketolase (Wernicke-Korsakoff syndrome) (TKT), mRNA	NM_001064	Hs.89643	BX649193
FUT1	-8.6	5.7	fucosyltransferase 1 (galactoside 2-alpha-L-fucosyltransferase, H blood group) (FUT1), mRNA	NM_000148	Hs.69747	NM_000148
NOM1	-8.6	2.9	nucleolar protein with MIF4G domain 1 (NOM1), mRNA	NM_138400	Hs.15825	AY576779
DLAT	-8.6	2.2	dihydrolipoamide S-acetyltransferase (E2 component of pyruvate dehydrogenase complex) (DLAT), mRNA	NM_001931	Hs.335551	NM_001931
THC2507805	-8.6	7.8	THC2507805	THC2507805	Unknown	
THC2678806	-8.6	7.9	HESX1_HUMAN (Q9UBX0) Homeobox expressed in ES cells 1 (Homeobox protein ANF) (hAnf), partial (70%)	THC2678806	Unknown	
A_32_P233713	-8.6	3.0	A_32_P233713	A_32_P233713	Unknown	
PVRL1	-8.5	3.2	poliovirus receptor-related 1 (herpesvirus entry mediator C; nectin) (PVRL1), transcript variant 3, mRNA	NM_203286	Hs.334846	NM_002855
PIAS2	-8.5	2.2	protein inhibitor of activated STAT, 2 (PIAS2), transcript variant beta, mRNA	NM_004671	Hs.658013	CR749597
FAM124A	-8.5	8.0	family with sequence similarity 124A (FAM124A), mRNA	NM_145019	Hs.71913	BC051771

A_24_P50328	-8.5	2.8	A_24_P50328	A_24_P50328	Unknown	
WDR12	-8.5	1.9	WD repeat domain 12 (WDR12), mRNA	NM_018256	Hs.73291	AK056092
MBD2	-8.5	3.8	methyl-CpG binding domain protein 2 (MBD2), transcript variant testis-specific, mRNA	NM_015832	Hs.25674	NM_003927
PLS1	-8.5	2.9	plastin 1 (I isoform) (PLS1), mRNA	NM_002670	Hs.203637	NM_002670
SEPHS1	-8.5	4.7	selenophosphate synthetase 1 (SEPHS1), mRNA	NM_012247	Hs.124027	AK125066
BM667833	-8.5	3.8	BM667833 UI-E-DW0-agj-g-19-0-UI.s1 UI-E-DW0 cDNA clone UI-E-DW0-agj-g-19-0-UI 3', mRNA sequence	BM667833	Hs.516217	BX537559
AJ276555	-8.5	9.9	mRNA for hypothetical protein (ORF1), clone 00275.	AJ276555	Hs.152129	CD246916
GLDC	-8.5	8.2	glycine dehydrogenase (decarboxylating) (GLDC), mRNA	NM_000170	Hs.584238	NM_000170
KIAA0258	-8.5	3.0	KIAA0258, mRNA (cDNA clone MGC:755 IMAGE:3533857), complete cds.	BC001725	Hs.493804	D87447
FCHO1	-8.5	3.1	FCH domain only 1 (FCHO1), mRNA	NM_015122	Hs.96485	NM_015122
THC2522889	-8.5	3.1	GPR27_HUMAN (Q9NS67) Probable G-protein coupled receptor 27 (Super conserved receptor expressed in brain 1), complete	THC2522889	Unknown	
MOCOS	-8.4	4.9	molybdenum cofactor sulfurase (MOCOS), mRNA	NM_017947	Hs.405028	AK222886
ENST00000332	-8.4	3.2	ENST00000332498	ENST00000332	Unknown	
PARD6A	-8.4	2.5	par-6 partitioning defective 6 homolog alpha (C. elegans) (PARD6A), transcript variant 1, mRNA	NM_016948	Hs.112933	AF252292
DENR	-8.4	2.7	density-regulated protein (DENR), mRNA	NM_003677	Hs.22393	NM_003677
TNPO3	-8.4	3.7	transportin 3 (TNPO3), mRNA	NM_012470	Hs.193613	NM_012470
TERF1	-8.4	9.4	telomeric repeat binding factor (NIMA-interacting) 1 (TERF1), transcript variant 1, mRNA	NM_017489	Hs.442707	AK128828
LARP2	-8.4	3.4	La ribonucleoprotein domain family, member 2 (LARP2), transcript variant 3, mRNA	NM_032239	Hs.657067	NM_018078
IFI30	-8.4	7.3	interferon, gamma-inducible protein 30 (IFI30), mRNA	NM_006332	Hs.14623	AK123477
BCAT1	-8.4	2.8	branched chain aminotransferase 1, cytosolic (BCAT1), mRNA	NM_005504	Hs.438993	NM_005504
HPS3	-8.4	2.5	Hermansky-Pudlak syndrome 3 (HPS3), mRNA	NM_032383	Hs.591311	NM_032383
ITPR3	-8.4	4.5	inositol 1,4,5-triphosphate receptor, type 3 (ITPR3), mRNA	NM_002224	Hs.65758	NM_002224
VASH2	-8.4	4.7	vasohibin 2 (VASH2), mRNA	NM_024749	Hs.96885	BC051856
TDRKH	-8.4	3.4	cDNA FLJ31840 fis, clone NT2RP7000109, highly similar to putative RNA binding protein mRNA.	AK056402	Hs.144439	AK225160
QSER1	-8.3	2.9	glutamine and serine rich 1 (QSER1), mRNA	NM_001076786	Hs.369368	NM_001076786
RBM35A	-8.3	6.5	RNA binding motif protein 35A (RBM35A), transcript variant 1, mRNA	NM_017697	Hs.487471	BX647570
ZNF137	-8.3	1.7	zinc finger protein 137 (ZNF137), mRNA	NM_003438	Hs.373648	NM_003438
MOCOS	-8.3	4.7	molybdenum cofactor sulfurase (MOCOS), mRNA	NM_017947	Hs.405028	AK222886
BRAF	-8.3	2.5	v-raf murine sarcoma viral oncogene homolog B1 (BRAF), mRNA	NM_004333	Hs.550061	M95712
ITGA6	-8.3	3.4	integrin, alpha 6 (ITGA6), transcript variant 2, mRNA	NM_000210	Hs.133397	NM_000210
GRB14	-8.2	2.6	growth factor receptor-bound protein 14 (GRB14), mRNA	NM_004490	Hs.411881	NM_004490
COX6A1	-8.2	106.8	cytochrome C oxidase subunit VIa homolog mRNA, complete cds.	AF020589	Hs.497118	BQ433343
PHC1	-8.2	8.1	polyhomeotic homolog 1 (Drosophila) (PHC1), mRNA	NM_004426	Hs.305985	NM_004426
WAPAL	-8.2	2.2	wings apart-like homolog (Drosophila) (WAPAL), mRNA	NM_015045	Hs.203099	AB065003
RASGEF1A	-8.2	6.2	RasGEF domain family, member 1A (RASGEF1A), mRNA	NM_145313	Hs.125293	AK127432
FAM46B	-8.2	8.0	family with sequence similarity 46, member B (FAM46B), mRNA	NM_052943	Hs.632378	BC012790
LOC199882	-8.2	42.6	PREDICTED: similar to phosphodiesterase 4D interacting protein isoform 1 (LOC199882), mRNA	XM_114047	Hs.438876	BG218865
ENST00000331	-8.2	14.3	ENST00000331096	ENST00000331	Unknown	
MOCOS	-8.2	4.7	molybdenum cofactor sulfurase (MOCOS), mRNA	NM_017947	Hs.405028	AK222886
CBS	-8.1	3.0	cystathionine-beta-synthase (CBS), mRNA	NM_000071	Hs.533013	L00972
TRIM14	-8.1	7.3	tripartite motif-containing 14 (TRIM14), transcript variant 1, mRNA	NM_014788	Hs.575631	NM_014788
DPYSL3	-8.1	3.2	dihydropyrimidinase-like 3 (DPYSL3), mRNA	NM_001387	Hs.519659	BC077077
ZIC5	-8.1	5.2	Zic family member 5 (odd-paired homolog, Drosophila) (ZIC5), mRNA	NM_033132	Hs.508570	NM_033132
VDP	-8.1	2.4	vesicle docking protein p115 (VDP), mRNA	NM_003715	Hs.292689	AL832010
IVD	-8.1	3.2	Isovaleryl-CoA dehydrogenase, mitochondrial precursor (EC 1.3.99.10) (IVD).	ENST00000249	Unknown	
EPB41L5	-8.1	4.2	erythrocyte membrane protein band 4.1 like 5, mRNA (cDNA clone MGC:26029 IMAGE:4827274), complete cds.	BC032822	Hs.654802	BC054508
MCM3	-8.1	1.8	MCM3 minichromosome maintenance deficient 3 (S. cerevisiae) (MCM3), mRNA	NM_002388	Hs.179565	NM_002388
ENST00000321	-8.1	10.2	fetal Alzheimer antigen isoform 1	ENST00000321	Unknown	
MREG	-8.1	3.2	melanoregulin (MREG), mRNA	NM_018000	Hs.281680	NM_018000
FAM104A	-8.0	2.6	family with sequence similarity 104, member A (FAM104A), mRNA	NM_032837	Hs.103555	NM_001098832
ZBTB3	-8.0	3.4	zinc finger and BTB domain containing 3 (ZBTB3), mRNA	NM_024784	Hs.147554	NM_024784
NOLC1	-8.0	2.4	nucleolar and coiled-body phosphoprotein 1 (NOLC1), mRNA	NM_004741	Hs.523238	NM_004741
POU5F1	-8.0	106.2	POU domain, class 5, transcription factor 1 (POU5F1), transcript variant 1, mRNA	NM_002701	Hs.249184	DQ486514
POU5F1	-8.0	157.4	POU domain, class 5, transcription factor 1 (POU5F1), transcript variant 1, mRNA	NM_002701	Hs.249184	DQ486514

SLC29A1	-8.0	2.3	solute carrier family 29 (nucleoside transporters), member 1 (SLC29A1), nuclear gene encoding mitochondrial protein, transcript variant 1, mRNA	NM_001078177	Hs.25450	NM_001078177
PIGL	-8.0	3.4	phosphatidylinositol glycan anchor biosynthesis, class L (PIGL), mRNA	NM_004278	Hs.499793	AK023469
MOCOS	-8.0	4.6	molybdenum cofactor sulfurase (MOCOS), mRNA	NM_017947	Hs.405028	AK222886
ZNF589	-8.0	3.3	zinc finger protein 589 (ZNF589), mRNA	NM_016089	Hs.172602	AB209387
ITPK1	-8.0	2.7	inositol 1,3,4-triphosphate 5/6 kinase (ITPK1), mRNA	NM_014216	Hs.308122	NM_014216
FBXO27	-7.9	3.2	F-box protein 27 (FBXO27), mRNA	NM_178820	Hs.187461	NM_178820
MOCOS	-7.9	4.9	molybdenum cofactor sulfurase (MOCOS), mRNA	NM_017947	Hs.405028	AK222886
ARMC4	-7.9	2.5	armadillo repeat containing 4 (ARMC4), mRNA	NM_018076	Hs.127530	BC085611
ISG20L1	-7.9	4.0	interferon stimulated exonuclease gene 20kDa-like 1 (ISG20L1), mRNA	NM_022767	Hs.436102	AB209323
SCNN1A	-7.9	8.6	sodium channel, nonvoltage-gated 1 alpha (SCNN1A), mRNA	NM_001038	Hs.591047	AK172792
EIF4E3	-7.9	5.0	eukaryotic translation initiation factor 4E family member 3 (EIF4E3), mRNA	NM_173359	Hs.655608	AL161983
A_24_P341731	-7.9	6.2	A_24_P341731	A_24_P341731	Unknown	
NARG1	-7.9	3.2	NMDA receptor regulated 1 (NARG1), mRNA	NM_057175	Hs.555985	NM_057175
ARFGEF1	-7.9	2.3	ADP-ribosylation factor guanine nucleotide-exchange factor 1(brefeldin A-inhibited) (ARFGEF1), mRNA	NM_006421	Hs.656902	NM_006421
TRIM14	-7.9	7.9	tripartite motif-containing 14 (TRIM14), transcript variant 1, mRNA	NM_014788	Hs.575631	NM_014788
PRPH2	-7.9	5.8	peripherin 2 (retinal degeneration, slow) (PRPH2), mRNA	NM_000322	Hs.654489	BX640679
KIAA1576	-7.8	4.7	KIAA1576 protein (KIAA1576), mRNA	NM_020927	Hs.461405	BC033537
MOCOS	-7.8	4.9	molybdenum cofactor sulfurase (MOCOS), mRNA	NM_017947	Hs.405028	AK222886
MFSD7	-7.8	2.4	major facilitator superfamily domain containing 7 (MFSD7), mRNA	NM_032219	Hs.567612	NM_032219
CYCS	-7.8	2.2	cytochrome c, somatic (CYCS), nuclear gene encoding mitochondrial protein, mRNA	NM_018947	Hs.437060	NM_018947
ZNF544	-7.8	2.3	zinc finger protein 544 (ZNF544), mRNA	NM_014480	Hs.438994	AK226080
CRSP2	-7.8	3.3	cofactor required for Sp1 transcriptional activation, subunit 2, 150kDa (CRSP2), mRNA	NM_004229	Hs.407604	AB006651
CD24	-7.8	3.1	CD24 signal transducer mRNA, complete cds and 3' region.	L33930	Hs.694721	AK125531
TSPAN33	-7.8	4.7	tetraspanin 33 (TSPAN33), mRNA	NM_178562	Hs.27267	BC044244
SCLY	-7.8	2.1	selenocysteine lyase (SCLY), mRNA	NM_016510	Hs.512606	AB209458
TSPAN33	-7.8	2.7	tetraspanin 33 (TSPAN33), mRNA	NM_178562	Hs.27267	BC044244
ENST00000372(	-7.8	2.8	Chordin-like protein 1 precursor (Neuralin-1) (Ventroptin) (Neurogenesin-1).	ENST00000372(	Unknown	
MGST1	-7.7	2.6	microsomal glutathione S-transferase 1 (MGST1), transcript variant 1c, mRNA	NM_145791	Hs.389700	AK123482
DPYSL3	-7.7	5.6	dihydropyrimidinase-like 3 (DPYSL3), mRNA	NM_001387	Hs.519659	BC077077
HPS3	-7.7	2.4	Hermansky-Pudlak syndrome 3 (HPS3), mRNA	NM_032383	Hs.591311	NM_032383
C12orf11	-7.7	2.1	chromosome 12 open reading frame 11 (C12orf11), mRNA	NM_018164	Hs.505077	NM_018164
NLK	-7.7	2.7	nemo-like kinase (NLK), mRNA	NM_016231	Hs.208759	BC064663
CEBPZ	-7.7	3.5	CCAAT/enhancer binding protein zeta (CEBPZ), mRNA	NM_005760	Hs.135406	AK125997
FUT2	-7.7	15.5	fucosyltransferase 2 (secretor status included) (FUT2), mRNA	NM_000511	Hs.579928	NM_000511
FAM57B	-7.7	3.4	family with sequence similarity 57, member B (FAM57B), mRNA	NM_031478	Hs.558560	NM_031478
ZNRF2	-7.7	2.1	zinc and ring finger 2 (ZNRF2), mRNA	NM_147128	Hs.487869	AF513707
MOCOS	-7.7	5.0	molybdenum cofactor sulfurase (MOCOS), mRNA	NM_017947	Hs.405028	AK222886
MOCOS	-7.7	4.8	molybdenum cofactor sulfurase (MOCOS), mRNA	NM_017947	Hs.405028	AK222886
TERF1	-7.6	9.3	telomeric repeat binding factor (NIMA-interacting) 1 (TERF1), transcript variant 1, mRNA	NM_017489	Hs.442707	AK128828
PRSS12	-7.6	5.3	protease, serine, 12 (neurotrypsin, motopsin) (PRSS12), mRNA	NM_003619	Hs.654823	NM_003619
MRPS17	-7.6	1.9	mitochondrial ribosomal protein S17 (MRPS17), nuclear gene encoding mitochondrial protein, mRNA	NM_015969	Hs.44298	AK026553
CDC25A	-7.6	4.2	cell division cycle 25 homolog A (S. cerevisiae) (CDC25A), transcript variant 1, mRNA	NM_001789	Hs.437705	NM_001789
FAM54A	-7.6	2.4	family with sequence similarity 54, member A (FAM54A), mRNA	NM_138419	Hs.121536	NM_001099286
A_32_P82580	-7.6	16.2	A_32_P82580	A_32_P82580	Unknown	
BC073935	-7.6	2.7	cDNA clone IMAGE:5219247, partial cds.	BC073935	Hs.434403	AK074886
MT1F	-7.6	4.1	metallothionein 1F (functional) (MT1F), mRNA	NM_005949	Hs.513626	BC108722
BC032118	-7.6	2.5	cDNA clone IMAGE:5016307, partial cds.	BC032118	Hs.16004	NM_024541
CYP2S1	-7.6	5.2	cytochrome P450, family 2, subfamily S, polypeptide 1 (CYP2S1), mRNA	NM_030622	Hs.98370	NM_030622
PDK3	-7.6	2.6	pyruvate dehydrogenase kinase, isozyme 3 (PDK3), mRNA	NM_005391	Hs.658190	CR606226
A_24_P127063	-7.6	3.2	A_24_P127063	A_24_P127063	Unknown	
MRPL42	-7.6	1.9	mitochondrial ribosomal protein L42 (MRPL42), nuclear gene encoding mitochondrial protein, transcript variant 3, mRNA	NM_172178	Hs.199579	CR749344
C9orf135	-7.6	28.1	chromosome 9 open reading frame 135 (C9orf135), mRNA	NM_001010940	Hs.444459	NM_001010940
MYCL1	-7.6	2.5	v-myc myelocytomatosis viral oncogene homolog 1, lung carcinoma derived (avian) (MYCL1), transcript variant 3, mRNA	NM_005376	Hs.437922	NM_001033081
HPS3	-7.6	2.5	Hermansky-Pudlak syndrome 3 (HPS3), mRNA	NM_032383	Hs.591311	NM_032383
RAB39B	-7.6	1.5	RAB39B, member RAS oncogene family (RAB39B), mRNA	NM_171998	Hs.632832	NM_171998

FAM104A	-7.5	2.4	family with sequence similarity 104, member A (FAM104A), mRNA	NM_032837	Hs.103555	NM_001098832
C9orf61	-7.5	3.2	chromosome 9 open reading frame 61 (C9orf61), mRNA	NM_004816	Hs.118003	AK126127
CR600638	-7.5	6.1	full-length cDNA clone CS0DI053YD12 of Placenta Cot 25-normalized of (human).	CR600638	Hs.624952	CR600638
DCC1	-7.5	2.6	defective in sister chromatid cohesion homolog 1 (S. cerevisiae) (DCC1), mRNA	NM_024094	Hs.315167	BC001316
FKBP4	-7.5	2.4	FK506 binding protein 4, 59kDa (FKBP4), mRNA	NM_002014	Hs.524183	NM_002014
TERF1	-7.5	10.1	telomeric repeat binding factor (NIMA-interacting) 1 (TERF1), transcript variant 1, mRNA	NM_017489	Hs.442707	AK128828
A_32_P128399	-7.5	1.9	A_32_P128399	A_32_P128399	Unknown	
A_32_P89415	-7.5	5.5	A_32_P89415	A_32_P89415	Unknown	
B3GALT5	-7.5	4.0	UDP-Gal:betaGlcNAc beta 1,3-galactosyltransferase, polypeptide 5 (B3GALT5), transcript variant 5, mRNA	NM_033173	Hs.655094	NM_033173
TDGF1	-7.4	14.2	teratocarcinoma-derived growth factor 1 (TDGF1), mRNA	NM_003212	Hs.385870	NM_003212
PASK	-7.4	2.1	mRNA; cDNA DKFZp434O051 (from clone DKFZp434O051).	AL117400	Unknown	
TTTY22	-7.4	2.5	testis-specific transcript, Y-linked 22 (TTTY22) on chromosome Y	NR_001539	Unknown	
SOX2	-7.4	3.4	SRY (sex determining region Y)-box 2 (SOX2), mRNA	NM_003106	Hs.518438	NM_003106
NLE1	-7.4	2.5	notchless homolog 1 (Drosophila) (NLE1), transcript variant 2, mRNA	NM_001014445	Hs.85570	AB209111
CHST4	-7.4	8.6	carbohydrate (N-acetylglucosamine 6-O) sulfotransferase 4 (CHST4), mRNA	NM_005769	Hs.251383	BC035282
BOLA3	-7.4	2.4	bolA homolog 3 (E. coli) (BOLA3), transcript variant 1, mRNA	NM_212552	Hs.61472	CD357422
NOL11	-7.4	1.7	nucleolar protein 11 (NOL11), mRNA	NM_015462	Hs.463936	BC064404
MAL2	-7.4	3.7	mal, T-cell differentiation protein 2 (MAL2), mRNA	NM_052886	Hs.201083	NM_052886
PTPRZ1	-7.4	5.5	protein tyrosine phosphatase, receptor-type, Z polypeptide 1 (PTPRZ1), mRNA	NM_002851	Hs.489824	NM_002851
HPS3	-7.4	2.3	Hermansky-Pudlak syndrome 3 (HPS3), mRNA	NM_032383	Hs.591311	NM_032383
TDGF1	-7.4	99.9	teratocarcinoma-derived growth factor 1 (TDGF1), mRNA	NM_003212	Hs.385870	NM_003212
FBXO28	-7.4	1.8	F-box protein 28 (FBXO28), mRNA	NM_015176	Hs.64691	AB007952
PIM2	-7.4	20.1	pim-2 oncogene (PIM2), mRNA	NM_006875	Hs.496096	NM_006875
UNC5D	-7.4	3.7	unc-5 homolog D (C. elegans) (UNC5D), mRNA	NM_080872	Hs.238889	NM_080872
LOC642123	-7.4	3.1	cDNA FLJ46881 fis, clone UTERU3015647, moderately similar to Embigin precursor.	AK128714	Hs.697682	AK128714
PLA2G3	-7.4	9.0	phospholipase A2, group III (PLA2G3), mRNA	NM_015715	Hs.149623	AF220490
RALGPS1	-7.4	2.8	Ral GEF with PH domain and SH3 binding motif 1 (RALGPS1), mRNA	NM_014636	Hs.696056	BX648170
A_32_P132169	-7.3	3.1	A_32_P132169	A_32_P132169	Unknown	
C6orf120	-7.3	2.0	chromosome 6 open reading frame 120 (C6orf120), mRNA	NM_001029863	Hs.591375	BC051700
CA941346	-7.3	15.9	CA941346 ir34a05.x1 HR85 islet cDNA clone IMAGE:6546921 3', mRNA sequence	CA941346	Unknown	
DCC1	-7.3	2.7	defective in sister chromatid cohesion homolog 1 (S. cerevisiae) (DCC1), mRNA	NM_024094	Hs.315167	BC001316
OSBPL10	-7.3	3.5	oxysterol binding protein-like 10 (OSBPL10), mRNA	NM_017784	Hs.150122	AB209872
LOC283174	-7.3	5.7	cDNA FLJ41855 fis, clone NT2RI3005724.	AK123849	Hs.504370	BC106032
NCL	-7.3	2.5	nucleolin (NCL), mRNA	NM_005381	Hs.79110	AK128584
MOCOS	-7.3	4.4	molybdenum cofactor sulfurase (MOCOS), mRNA	NM_017947	Hs.405028	AK222886
RBPMS2	-7.3	3.2	RNA binding protein with multiple splicing 2 (RBPMS2), mRNA	NM_194272	Hs.436518	AY369207
BC042080	-7.3	10.7	Homo sapiens, clone IMAGE:5744974, mRNA.	BC042080	Hs.663835	BC042080
HPS3	-7.3	2.5	Hermansky-Pudlak syndrome 3 (HPS3), mRNA	NM_032383	Hs.591311	NM_032383
A_24_P230416	-7.3	1.7	A_24_P230416	A_24_P230416	Unknown	
LHPP	-7.3	3.1	phospholysine phosphohistidine inorganic pyrophosphate phosphatase (LHPP), mRNA	NM_022126	Hs.527748	BC110344
PPP1R1B	-7.3	4.7	protein phosphatase 1, regulatory (inhibitor) subunit 1B (dopamine and cAMP regulated phosphoprotein, DARPP-32) (PPP1R1B), transcript variant 1, mRNA	NM_032192	Hs.286192	AK123950
LHFPL4	-7.2	26.1	lipoma HMGIC fusion partner-like 4 (LHFPL4), mRNA	NM_198560	Hs.56782	NM_198560
HSPE1	-7.2	2.4	heat shock 10kDa protein 1 (chaperonin 10) (HSPE1), mRNA	NM_002157	Hs.1197	BU517060
SCG3	-7.2	6.3	secretogranin III (SCG3), mRNA	NM_013243	Hs.232618	NM_013243
CENTB1	-7.2	3.6	centaurin, beta 1 (CENTB1), mRNA	NM_014716	Hs.337242	AK126509
THC2576067	-7.2	3.3	THC2576067	THC2576067	Unknown	
ITGB1BP3	-7.2	9.2	integrin beta 1 binding protein 3 (ITGB1BP3), transcript variant 1, mRNA	NM_014446	Unknown	
BTBD3	-7.2	1.7	BTB (POZ) domain containing 3 (BTBD3), transcript variant 1, mRNA	NM_014962	Hs.244590	NM_014962
MYO10	-7.2	3.9	myosin X (MYO10), mRNA	NM_012334	Hs.481720	NM_012334
ENPP5	-7.2	3.5	ectonucleotide pyrophosphatase/phosphodiesterase 5 (putative function) (ENPP5), mRNA	NM_021572	Hs.35198	BX647968
CCDC112	-7.2	1.8	coiled-coil domain containing 112 (CCDC112), transcript variant 1, mRNA	NM_001040440	Hs.436121	NM_152549
COX17	-7.2	1.9	COX17 cytochrome c oxidase assembly homolog (S. cerevisiae) (COX17), nuclear gene encoding mitochondrial protein, mRNA	NM_005694	Hs.534383	BU506590
AL359605	-7.1	2.5	mRNA; cDNA DKFZp547G036 (from clone DKFZp547G036).	AL359605	Hs.283851	AK124873
TCOF1	-7.1	2.1	Treacher Collins-Franceschetti syndrome 1 (TCOF1), transcript variant 1, mRNA	NM_001008656	Hs.519672	AB209317
THC2707492	-7.1	3.1	THC2707492	THC2707492	Unknown	
HLA-DOA	-7.1	5.0	major histocompatibility complex, class II, DO alpha (HLA-DOA), mRNA	NM_002119	Hs.631991	NM_002119

CR621447	-7.1	3.7	full-length cDNA clone CS0DD008YK01 of Neuroblastoma Cot 50-normalized of (human).	CR621447	Hs.675860	CR621447
C9orf85	-7.1	2.2	chromosome 9 open reading frame 85 (C9orf85), mRNA	NM_182505	Hs.534190	NM_182505
A_23_P213350	-7.1	5.7	A_23_P213350	A_23_P213350	Unknown	
THC2550720	-7.1	4.1	ALU1_HUMAN (P39188) Alu subfamily J sequence contamination warning entry, partial (30%)	THC2550720	Unknown	
UGT8	-7.1	3.0	UDP glycosyltransferase 8 (UDP-galactose ceramide galactosyltransferase) (UGT8), mRNA	NM_003360	Hs.144197	NM_003360
PSIP1	-7.1	2.9	PC4 and SFRS1 interacting protein 1 (PSIP1), transcript variant 1, mRNA	NM_021144	Hs.658434	BX649155
ELOVL6	-7.1	2.6	ELOVL family member 6, elongation of long chain fatty acids (FEN1/Elo2, SUR4/Elo3-like, yeast) (ELOVL6), mRNA	NM_024090	Hs.412939	AK027031
KCTD8	-7.1	3.8	potassium channel tetramerisation domain containing 8 (KCTD8), mRNA	NM_198353	Hs.479644	AK123347
FAM119A	-7.1	2.5	family with sequence similarity 119, member A (FAM119A), mRNA	NM_145280	Hs.668241	BC033720
JPH3	-7.1	3.4	junctophilin 3, mRNA (cDNA clone IMAGE:3867947), complete cds.	BC008690	Hs.592068	AK126663
ALOX12P2	-7.1	4.4	arachidonate 12-lipoxygenase pseudogene 2 (ALOX12P2) on chromosome 17	NR_002710	Unknown	
C17orf39	-7.1	2.9	chromosome 17 open reading frame 39 (C17orf39), mRNA	NM_024052	Hs.187422	NM_024052
LYAR	-7.1	2.3	hypothetical protein FLJ20425 (LYAR), mRNA	NM_017816	Hs.425427	AK000432
WDR4	-7.0	3.1	WD repeat domain 4 (WDR4), transcript variant 2, mRNA	NM_033661	Hs.248815	AK092786
AL157455	-7.0	10.8	mRNA; cDNA DKFZp761I1912 (from clone DKFZp761I1912).	AL157455	Hs.592473	BC043194
EDG7	-7.0	4.7	Lysophosphatidic acid receptor Edg-7 (LPA receptor 3) (LPA-3).	ENST00000370	Unknown	
DCC1	-7.0	2.7	defective in sister chromatid cohesion homolog 1 (S. cerevisiae) (DCC1), mRNA	NM_024094	Hs.315167	BC001316
SNRPN	-7.0	6.5	small nuclear ribonucleoprotein polypeptide N (SNRPN), transcript variant 4, mRNA	NM_022807	Hs.564847	U81001
KRTCAP3	-7.0	11.1	keratinocyte associated protein 3 (KRTCAP3), mRNA	NM_173853	Hs.59509	AY358993
DHTKD1	-7.0	3.1	dehydrogenase E1 and transketolase domain containing 1 (DHTKD1), mRNA	NM_018706	Hs.104980	NM_018706
U88048	-7.0	3.6	Human clone KiSS-16 unknown product mRNA, complete cds.	U88048	Unknown	
FAM83D	-7.0	2.6	family with sequence similarity 83, member D (FAM83D), mRNA	NM_030919	Hs.472716	AL832274
MKKS	-7.0	2.8	McKusick-Kaufman syndrome (MKKS), transcript variant 2, mRNA	NM_170784	Hs.472119	NM_018848
KRT75	-7.0	2.5	keratin 75 (KRT75), mRNA	NM_004693	Hs.697046	NM_004693
DDX18	-7.0	2.2	DEAD (Asp-Glu-Ala-Asp) box polypeptide 18 (DDX18), mRNA	NM_006773	Hs.363492	AB209392
AL566369	-7.0	2.0	AL566369 FETAL BRAIN cDNA clone CS0DF038YD15 3-PRIME, mRNA sequence	AL566369	Hs.693667	BM466238
UPP1	-7.0	4.0	uridine phosphorylase 1, mRNA (cDNA clone MGC:54255 IMAGE:5549432), complete cds.	BC047030	Hs.488240	BC007348
BUB1	-7.0	3.8	BUB1 budding uninhibited by benzimidazoles 1 homolog (yeast) (BUB1), mRNA	NM_004336	Hs.469649	AF053305
THC2614148	-7.0	3.8	Q59G86_HUMAN (Q59G86) Androgen-regulated short-chain dehydrogenase/reductase 1 variant (Fragment), partial (7%)	THC2614148	Unknown	
N4BP3	-7.0	5.0	Nedd4 binding protein 3 (N4BP3), mRNA	NM_015111	Hs.101761	NM_015111
HRSP12	-7.0	1.6	heat-responsive protein 12 (HRSP12), mRNA	NM_005836	Hs.18426	BF217184
GLS2	-7.0	7.0	glutaminase 2 (liver, mitochondrial) (GLS2), nuclear gene encoding mitochondrial protein, mRNA	NM_013267	Hs.212606	BC048344
AW275876	-7.0	2.9	xq40c08.x1 NCI_CGAP_Lu28 cDNA clone IMAGE:2753102 3' similar to gb:X57352 INTERFERON-INDUCIBLE PROTEIN 1-8U (HUMAN);, mRNA sequence	AW275876	Hs.545434	AW275876
TEAD4	-7.0	3.6	TEA domain family member 4 (TEAD4), transcript variant 1, mRNA	NM_003213	Hs.94865	BM423370
NSUN7	-7.0	5.0	NOL1/NOP2/Sun domain family, member 7 (NSUN7), mRNA	NM_024677	Hs.590923	BC042401
USP44	-7.0	35.6	ubiquitin specific peptidase 44 (USP44), transcript variant 1, mRNA	NM_032147	Hs.646421	NM_032147
EIF2AK4	-7.0	2.2	eukaryotic translation initiation factor 2 alpha kinase 4 (EIF2AK4), mRNA	NM_001013703	Hs.656673	NM_001013703
ENST00000324	-7.0	4.5	chromosome 1 open reading frame 211, mRNA (cDNA clone MGC:40168 IMAGE:5141008), complete cds.	ENST00000324	Unknown	
CCDC109B	-7.0	2.4	coiled-coil domain containing 109B (CCDC109B), mRNA	NM_017918	Hs.234149	AK095936
THC2497326	-6.9	2.3	THC2497326	THC2497326	Unknown	
TSPAN33	-6.9	4.5	tetraspanin 33 (TSPAN33), mRNA	NM_178562	Hs.27267	BC044244
DCC1	-6.9	2.6	defective in sister chromatid cohesion homolog 1 (S. cerevisiae) (DCC1), mRNA	NM_024094	Hs.315167	BC001316
ZFHX2	-6.9	2.2	zinc finger homeobox 2 (ZFHX2), mRNA	NM_033400	Unknown	
U52054	-6.9	3.1	Human S6 H-8 mRNA expressed in chromosome 6-suppressed melanoma cells.	U52054	Hs.561411	BM564371
SYT6	-6.9	5.9	synaptotagmin VI (SYT6), mRNA	NM_205848	Hs.370963	NM_205848
CNOT6	-6.9	3.9	CCR4-NOT transcription complex, subunit 6 (CNOT6), mRNA	NM_015455	Hs.654984	AB033020
DCC1	-6.9	2.6	defective in sister chromatid cohesion homolog 1 (S. cerevisiae) (DCC1), mRNA	NM_024094	Hs.315167	BC001316
PVRL1	-6.9	2.5	poliovirus receptor-related 1 (herpesvirus entry mediator C; nectin) (PVRL1), transcript variant 1, mRNA	NM_002855	Hs.334846	NM_002855
FKBP11	-6.9	2.5	FK506 binding protein 11, 19 kDa (FKBP11), mRNA	NM_016594	Hs.655103	AB209018
LOC647195	-6.9	2.6	PREDICTED: hypothetical LOC647195 (LOC647195), mRNA	XR_019590	Hs.632457	XR_019590
ACVR1C	-6.9	3.5	activin A receptor, type IC (ACVR1C), mRNA	NM_145259	Hs.352338	BC022530
C18orf25	-6.9	2.1	chromosome 18 open reading frame 25 (C18orf25), transcript variant 1, mRNA	NM_145055	Hs.696077	NM_145055
SIPA1L1	-6.9	2.2	signal-induced proliferation-associated 1 like 1 (SIPA1L1), mRNA	NM_015556	Hs.654657	AK122930
ENST00000366	-6.9	2.5	Novel protein (MGC27277).	ENST00000366	Unknown	

A_32_P36412	-6.9	1.9	A_32_P36412	A_32_P36412	Unknown	
DUSP13	-6.9	5.7	dual specificity phosphatase 13 (DUSP13), transcript variant 1, mRNA	NM_001007271	Hs.178170	NM_001007271
ARHGEF16	-6.9	2.2	Rho guanine exchange factor (GEF) 16 (ARHGEF16), mRNA	NM_014448	Hs.87435	CR609468
FABP6	-6.9	6.7	fatty acid binding protein 6, ileal (gastrotropin) (FABP6), transcript variant 1, mRNA	NM_001040442	Hs.519719	BG192623
ATP6V0A2	-6.9	2.7	ATPase, H+ transporting, lysosomal V0 subunit a2 (ATP6V0A2), mRNA	NM_012463	Hs.201939	NM_012463
OXNAD1	-6.9	2.1	oxidoreductase NAD-binding domain containing 1 (OXNAD1), mRNA	NM_138381	Hs.655449	NM_138381
A_24_P367397	-6.8	2.6	A_24_P367397	A_24_P367397	Unknown	
DYDC2	-6.8	2.7	DPY30 domain containing 2 (DYDC2), mRNA	NM_032372	Hs.512782	BC018606
DCC1	-6.8	2.6	defective in sister chromatid cohesion homolog 1 (S. cerevisiae) (DCC1), mRNA	NM_024094	Hs.315167	BC001316
RTN4IP1	-6.8	2.3	reticulon 4 interacting protein 1 (RTN4IP1), nuclear gene encoding mitochondrial protein, mRNA	NM_032730	Hs.155839	NM_032730
DQ786238	-6.8	2.1	clone HLS_IMAGE_1881469 mRNA sequence.	DQ786238	Hs.654872	BM557567
ENPP1	-6.8	4.3	Ectonucleotide pyrophosphatase/phosphodiesterase 1 (E-NPP 1) (Phosphodiesterase I/nucleotide pyrophosphatase 1) (Plasma-cell membrane glycoprotein PC-1) [Includes: Alkaline phosphodiesterase I (EC 3.1.4.1); Nucleotide pyrophosphatase (EC 3.6.1.9)...	ENST00000367\[illegible]	Unknown	
ALPL	-6.8	4.2	alkaline phosphatase, liver/bone/kidney (ALPL), mRNA	NM_000478	Hs.75431	NM_000478
ENST00000375[illegible]	-6.8	58.8	OTTHUMP00000021675.	ENST00000375[illegible]	Unknown	
NOLC1	-6.8	2.7	nucleolar and coiled-body phosphoprotein 1 (NOLC1), mRNA	NM_004741	Hs.523238	NM_004741
BAG4	-6.8	1.8	BAG family molecular chaperone regulator 4 (BCL2-associated athanogene 4) (BAG-4) (Silencer of death domains).	ENST00000287[illegible]	Unknown	
CXXC6	-6.8	3.3	CXXC-type zinc finger protein 6 (Leukemia-associated protein with a CXXC domain).	ENST00000373[illegible]	Unknown	
AF086442	-6.8	3.7	full length insert cDNA clone ZD81B04.	AF086442	Hs.655734	AK022764
DOCK3	-6.8	4.3	dedicator of cytokinesis 3 (DOCK3), mRNA	NM_004947	Hs.476284	NM_004947
GABRA5	-6.8	14.7	gamma-aminobutyric acid (GABA) A receptor, alpha 5 (GABRA5), mRNA	NM_000810	Hs.612087	NM_000810
ZNF101	-6.8	3.2	zinc finger protein 101 (ZNF101), mRNA	NM_033204	Hs.631642	AK097169
ASPHD2	-6.8	3.7	aspartate beta-hydroxylase domain containing 2 (ASPHD2), mRNA	NM_020437	Hs.567547	AK097157
TGIF	-6.8	2.9	TGFB-induced factor (TALE family homeobox) (TGIF), transcript variant 1, mRNA	NM_170695	Hs.373550	NM_170695
LCK	-6.8	30.0	lymphocyte-specific protein tyrosine kinase (LCK), transcript variant 2, mRNA	NM_005356	Hs.470627	BC013200
PPCDC	-6.7	1.7	phosphopantothenoylcysteine decarboxylase (PPCDC), mRNA	NM_021823	Hs.458922	NM_021823
CCBL1	-6.7	2.2	cysteine conjugate-beta lyase; cytoplasmic (glutamine transaminase K, kyneurenine aminotransferase) (CCBL1), mRNA	NM_004059	Hs.495250	AK094505
HESX1	-6.7	9.8	homeobox, ES cell expressed 1 (HESX1), mRNA	NM_003865	Hs.171980	NM_003865
MYBBP1A	-6.7	4.0	MYB binding protein (P160) 1a (MYBBP1A), mRNA	NM_014520	Hs.22824	AF147709
OSBPL10	-6.7	4.9	oxysterol binding protein-like 10 (OSBPL10), mRNA	NM_017784	Hs.150122	AB209872
A_24_P213325	-6.7	2.8	A_24_P213325	A_24_P213325	Unknown	
KIF1A	-6.7	4.4	kinesin family member 1A (KIF1A), mRNA	NM_004321	Hs.516802	NM_004321
LCK	-6.7	31.0	lymphocyte-specific protein tyrosine kinase (LCK), transcript variant 2, mRNA	NM_005356	Hs.470627	BC013200
PROM1	-6.7	1.9	prominin 1 (PROM1), mRNA	NM_006017	Hs.614734	AF117225
PTCD3	-6.7	1.6	Pentatricopeptide repeat domain 3 (PTCD3), mRNA	NM_017952	Hs.323489	NM_017952
PAK6	-6.7	2.5	p21(CDKN1A)-activated kinase 6 (PAK6), mRNA	NM_020168	Hs.513645	AK131522
LRRC14	-6.7	2.7	leucine rich repeat containing 14 (LRRC14), mRNA	NM_014665	Hs.459391	NM_014665
JARID2	-6.7	2.9	jumonji, AT rich interactive domain 2 (JARID2), mRNA	NM_004973	Hs.696068	NM_004973
BRAF	-6.7	2.5	v-raf murine sarcoma viral oncogene homolog B1 (BRAF), mRNA	NM_004333	Hs.550061	M95712
JPH3	-6.7	3.3	junctophilin 3 (JPH3), mRNA	NM_020655	Hs.592068	AK126663
PCDH1	-6.7	4.0	protocadherin 1 (cadherin-like 1) (PCDH1), transcript variant 1, mRNA	NM_002587	Hs.79769	NM_032420
CKS2	-6.7	2.6	CDC28 protein kinase regulatory subunit 2 (CKS2), mRNA	NM_001827	Hs.83758	BQ898943
A_24_P407742	-6.7	2.6	A_24_P407742	A_24_P407742	Unknown	
ADRA2B	-6.7	4.1	adrenergic, alpha-2B-, receptor (ADRA2B), mRNA	NM_000682	Hs.673848	NM_000682
A_24_P761727	-6.7	2.1	A_24_P761727	A_24_P761727	Unknown	
LOC728996	-6.7	2.6	PREDICTED: similar to methyltransferase like 2 (LOC728996), mRNA	XR_015425	Hs.693326	XR_015453
SGOL1	-6.7	2.0	shugoshin-like 1 (S. pombe) (SGOL1), transcript variant A1, mRNA	NM_001012409	Hs.105153	AB187578
EARS2	-6.7	2.1	glutamyl-tRNA synthetase 2 (mitochondrial)(putative) (EARS2), mRNA	NM_133451	Unknown	
ZCCHC2	-6.7	5.1	zinc finger, CCHC domain containing 2, mRNA (cDNA clone IMAGE:4079754), complete cds.	BC006340	Hs.699275	NM_017742
PRIM2A	-6.7	2.1	primase, polypeptide 2A, 58kDa (PRIM2A), mRNA	NM_000947	Hs.654580	NM_000947
AK096500	-6.7	2.0	cDNA FLJ39181 fis, clone OCBBF2004235.	AK096500	Hs.200260	AK096500
C14orf156	-6.7	1.9	chromosome 14 open reading frame 156 (C14orf156), mRNA	NM_031210	Hs.655105	BQ278491
CHKA	-6.7	2.5	choline kinase alpha (CHKA), transcript variant 1, mRNA	NM_001277	Hs.569019	AK054792
HCK	-6.7	3.0	hemopoietic cell kinase (HCK), mRNA	NM_002110	Hs.655210	AK225819
SEPHS1	-6.7	3.6	selenophosphate synthetase 1 (SEPHS1), mRNA	NM_012247	Hs.124027	AK125066
HDAC6	-6.6	2.8	histone deacetylase 6, mRNA (cDNA clone IMAGE:4179066), complete cds.	BC011498	Hs.6764	BC069243

HPS3	-6.6	2.3	Hermansky-Pudlak syndrome 3 (HPS3), mRNA	NM_032383	Hs.591311	NM_032383
LCK	-6.6	34.4	lymphocyte-specific protein tyrosine kinase (LCK), transcript variant 2, mRNA	NM_005356	Hs.470627	BC013200
PRKCA	-6.6	5.1	protein kinase C, alpha (PRKCA), mRNA	NM_002737	Hs.531704	NM_002737
EAF2	-6.6	1.9	ELL associated factor 2 (EAF2), mRNA	NM_018456	Hs.477325	BF965153
FAM126A	-6.6	4.1	family with sequence similarity 126, member A (FAM126A), mRNA	NM_032581	Hs.85603	AL833296
GPR176	-6.6	5.0	G protein-coupled receptor 176 (GPR176), mRNA	NM_007223	Hs.37196	BC067106
IL20RA	-6.6	2.9	interleukin 20 receptor, alpha (IL20RA), mRNA	NM_014432	Hs.445868	NM_014432
CCNF	-6.6	3.0	cyclin F (CCNF), mRNA	NM_001761	Hs.1973	NM_001761
THC2672083	-6.6	12.0	Q9NY18_HUMAN (Q9NY18) Calcium channel alpha2-delta3 subunit, partial (32%)	THC2672083	Unknown	
DMKN	-6.6	2.4	dermokine (DMKN), transcript variant 1, mRNA	NM_001035516	Hs.417795	AK125695
CXXC6	-6.6	3.3	CXXC-type zinc finger protein 6 (Leukemia-associated protein with a CXXC domain).	ENST00000373	Unknown	
ENST00000335	-6.6	3.9	ENST00000335078	ENST00000335	Unknown	
PHTF2	-6.6	2.0	putative homeodomain transcription factor 2 (PHTF2), mRNA	NM_020432	Hs.203965	AK225854
WDR33	-6.6	2.2	WD repeat domain 33 (WDR33), transcript variant 1, mRNA	NM_018383	Hs.620490	NM_018383
PURB	-6.6	2.5	purine-rich element binding protein B (PURB), mRNA	NM_033224	Hs.349150	NM_033224
METTL5	-6.6	1.5	methyltransferase like 5 (METTL5), mRNA	NM_014168	Hs.470553	BM465751
BRI3BP	-6.6	2.6	BRI3 binding protein (BRI3BP), mRNA	NM_080626	Hs.632740	AK025766
PPAP2C	-6.6	5.6	phosphatidic acid phosphatase type 2C (PPAP2C), transcript variant 3, mRNA	NM_177543	Hs.465506	AB209696
CARKL	-6.6	2.6	carbohydrate kinase-like (CARKL), mRNA	NM_013276	Hs.579217	AF163573
DDX18	-6.6	2.2	DEAD (Asp-Glu-Ala-Asp) box polypeptide 18 (DDX18), mRNA	NM_006773	Hs.363492	AB209392
NME3	-6.6	2.0	non-metastatic cells 3, protein expressed in (NME3), mRNA	NM_002513	Hs.514065	BU196357
DDX18	-6.6	2.2	DEAD (Asp-Glu-Ala-Asp) box polypeptide 18 (DDX18), mRNA	NM_006773	Hs.363492	AB209392
PRKCA	-6.6	4.6	protein kinase C, alpha (PRKCA), mRNA	NM_002737	Hs.531704	NM_002737
ARHGAP26	-6.6	1.9	Rho GTPase activating protein 26 (ARHGAP26), mRNA	NM_015071	Hs.654668	NM_015071
TERT	-6.6	4.1	telomerase reverse transcriptase (TERT), transcript variant 1, mRNA	NM_198253	Hs.492203	AF018167
DCC1	-6.6	2.7	defective in sister chromatid cohesion homolog 1 (S. cerevisiae) (DCC1), mRNA	NM_024094	Hs.315167	BC001316
RNF44	-6.6	1.9	ring finger protein 44 (RNF44), mRNA	NM_014901	Hs.650059	BC063297
OTX2	-6.5	5.1	orthodenticle homolog 2 (Drosophila) (OTX2), transcript variant 1, mRNA	NM_021728	Hs.288655	BC032579
EIF5A2	-6.5	2.9	eukaryotic translation initiation factor 5A2 (EIF5A2), mRNA	NM_020390	Hs.164144	AY205261
LRP8	-6.5	5.2	low density lipoprotein receptor-related protein 8, apolipoprotein e receptor (LRP8), transcript variant 2, mRNA	NM_033300	Hs.576154	NM_004631
TGFA	-6.5	7.5	transforming growth factor, alpha (TGFA), mRNA	NM_003236	Hs.170009	NM_003236
TERT	-6.5	4.3	telomerase reverse transcriptase (TERT), transcript variant 1, mRNA	NM_198253	Hs.492203	AF018167
ASXL2	-6.5	1.5	additional sex combs like 2	ENST00000336	Unknown	
FUT4	-6.5	2.7	fucosyltransferase 4 (alpha (1,3) fucosyltransferase, myeloid-specific) (FUT4), mRNA	NM_002033	Hs.390420	NM_002033
ZIC2	-6.5	5.7	Zic family member 2 (odd-paired homolog, Drosophila) (ZIC2), mRNA	NM_007129	Hs.591205	NM_007129
DDX18	-6.5	2.1	DEAD (Asp-Glu-Ala-Asp) box polypeptide 18 (DDX18), mRNA	NM_006773	Hs.363492	AB209392
C9orf52	-6.5	3.7	chromosome 9 open reading frame 52 (C9orf52), mRNA	NM_152574	Hs.563630	BC142985
LCK	-6.5	34.4	lymphocyte-specific protein tyrosine kinase (LCK), transcript variant 2, mRNA	NM_005356	Hs.470627	BC013200
USP28	-6.5	3.6	ubiquitin specific peptidase 28 (USP28), mRNA	NM_020886	Hs.503891	NM_020886
ST6GAL1	-6.5	2.9	ST6 beta-galactosamide alpha-2,6-sialyltranferase 1 (ST6GAL1), transcript variant 1, mRNA	NM_173216	Hs.207459	AK128726
TPST2	-6.5	5.0	tyrosylprotein sulfotransferase 2 (TPST2), transcript variant 1, mRNA	NM_001008566	Hs.694819	AK057171
ALDH3A2	-6.5	2.4	aldehyde dehydrogenase 3 family, member A2 (ALDH3A2), transcript variant 1, mRNA	NM_001031806	Hs.499886	U46689
SYN2	-6.5	4.3	synapsin II (SYN2), transcript variant IIa, mRNA	NM_133625	Hs.445503	NM_003178
TDRKH	-6.5	2.2	tudor and KH domain containing (TDRKH), mRNA	NM_006862	Hs.144439	AK225160
ADAM22	-6.5	2.4	ADAM metallopeptidase domain 22 (ADAM22), transcript variant 1, mRNA	NM_021723	Hs.592282	NM_021723
FLJ40432	-6.5	3.6	hypothetical protein FLJ40432 (FLJ40432), mRNA	NM_152523	Hs.471234	BC067253
PRSS12	-6.5	6.8	protease, serine, 12 (neurotrypsin, motopsin) (PRSS12), mRNA	NM_003619	Hs.654823	NM_003619
FAM33A	-6.5	1.9	family with sequence similarity 33, member A (FAM33A), mRNA	NM_182620	Hs.463607	NM_182620
BCAT1	-6.5	2.8	branched chain aminotransferase 1, cytosolic (BCAT1), mRNA	NM_005504	Hs.438993	NM_005504
C9orf126	-6.5	2.2	chromosome 9 open reading frame 126 (C9orf126), mRNA	NM_173690	Hs.59504	NM_173690
PRO1853	-6.4	4.4	hypothetical protein PRO1853 (PRO1853), transcript variant 2, mRNA	NM_018607	Unknown	
DLGAP1	-6.4	2.3	discs, large (Drosophila) homolog-associated protein 1 (DLGAP1), transcript variant alpha, mRNA	NM_004746	Hs.654793	NM_004746
EEF1E1	-6.4	2.1	eukaryotic translation elongation factor 1 epsilon 1 (EEF1E1), mRNA	NM_004280	Hs.631818	BC005291
MCART1	-6.4	2.3	mitochondrial carrier triple repeat 1 (MCART1), mRNA	NM_033412	Hs.645492	AK091407
LOC729608	-6.4	2.0	PREDICTED: similar to Putative Brix domain containing protein 1P (LOC729608), mRNA	XM_001130778	Hs.567693	XM_001130778
LOC732215	-6.4	4.4	PREDICTED: similar to 3-oxoacid CoA transferase 2 (LOC732215), mRNA	XR_015772	Hs.700547	XR_015772

C20orf160	-6.4	3.2	chromosome 20 open reading frame 160 (C20orf160), mRNA	NM_080625	Hs.382151	NM_080625
FLJ20105	-6.4	2.4	FLJ20105 protein (FLJ20105), transcript variant 2, mRNA	NM_001009954	Unknown	
CLDN7	-6.4	4.3	claudin 7 (CLDN7), mRNA	NM_001307	Hs.513915	NM_001307
SNHG3-RCC1	-6.4	3.3	regulator of chromosome condensation 1 (SNHG3-RCC1), transcript variant 1, mRNA	NM_001048197	Hs.469723	NM_001048197
ITGA7	-6.4	2.7	integrin, alpha 7 (ITGA7), mRNA	NM_002206	Hs.524484	BC050280
DCC1	-6.4	2.6	defective in sister chromatid cohesion homolog 1 (S. cerevisiae) (DCC1), mRNA	NM_024094	Hs.315167	BC001316
CABYR	-6.4	3.2	calcium binding tyrosine-(Y)-phosphorylation regulated (fibrousheathin 2) (CABYR), transcript variant 1, mRNA	NM_012189	Hs.511983	NM_012189
OVOL2	-6.4	6.1	ovo-like 2 (Drosophila) (OVOL2), mRNA	NM_021220	Hs.697036	NM_021220
CDC14C	-6.4	1.9	CDC14 cell division cycle 14 homolog C (S. cerevisiae), mRNA (cDNA clone IMAGE:4826219), partial cds.	BC028690	Hs.567757	BC068452
MRPL16	-6.4	1.6	mitochondrial ribosomal protein L16 (MRPL16), nuclear gene encoding mitochondrial protein, mRNA	NM_017840	Hs.530734	BQ058130
CDCA2	-6.4	2.1	cell division cycle associated 2 (CDCA2), mRNA	NM_152562	Hs.33366	NM_152562
ELL2	-6.4	2.2	elongation factor, RNA polymerase II, 2 (ELL2), mRNA	NM_012081	Hs.592742	NM_012081
C14orf130	-6.4	2.0	chromosome 14 open reading frame 130 (C14orf130), transcript variant 2, mRNA	NM_175748	Hs.648806	NM_175748
A_24_P620521	-6.4	2.6	A_24_P620521	A_24_P620521	Unknown	
LCK	-6.4	39.8	lymphocyte-specific protein tyrosine kinase (LCK), transcript variant 2, mRNA	NM_005356	Hs.470627	BC013200
RAD54B	-6.4	1.9	fibrinogen silencer binding protein mRNA, complete cds.	AF007866	Hs.30561	NM_012415
CDS1	-6.4	2.6	CDP-diacylglycerol synthase (phosphatidate cytidylyltransferase) 1 (CDS1), mRNA	NM_001263	Hs.654899	NM_001263
BC037528	-6.4	8.0	cDNA clone IMAGE:4794876.	BC037528	Hs.593150	AK127092
AIM1L	-6.4	2.4	absent in melanoma 1-like (AIM1L), mRNA	NM_017977	Hs.128738	NM_001039775
PIGQ	-6.4	3.1	phosphatidylinositol glycan, class Q, mRNA (cDNA clone IMAGE:3357878), partial cds.	BC010094	Unknown	
A_24_P67063	-6.4	2.9	A_24_P67063	A_24_P67063	Unknown	
RRAS2	-6.4	3.7	related RAS viral (r-ras) oncogene homolog 2 (RRAS2), mRNA	NM_012250	Hs.502004	CR592913
ERBB2	-6.3	3.1	v-erb-b2 erythroblastic leukemia viral oncogene homolog 2, neuro/glioblastoma derived oncogene homolog (avian) (ERBB2), transcript variant 2, mRNA	NM_001005862	Hs.446352	NM_001005862
ERBB2	-6.3	2.9	v-erb-b2 erythroblastic leukemia viral oncogene homolog 2, neuro/glioblastoma derived oncogene homolog (avian) (ERBB2), transcript variant 2, mRNA	NM_001005862	Hs.446352	NM_001005862
CD3EAP	-6.3	2.7	CD3e molecule, epsilon associated protein (CD3EAP), mRNA	NM_012099	Hs.699903	NM_012099
CXADR	-6.3	3.3	coxsackie virus and adenovirus receptor (CXADR), mRNA	NM_001338	Hs.634837	BC003684
LCK	-6.3	40.7	lymphocyte-specific protein tyrosine kinase (LCK), transcript variant 2, mRNA	NM_005356	Hs.470627	BC013200
MPP6	-6.3	3.2	membrane protein, palmitoylated 6 (MAGUK p55 subfamily member 6) (MPP6), mRNA	NM_016447	Hs.533355	NM_016447
RAB39B	-6.3	6.8	RAB39B, member RAS oncogene family (RAB39B), mRNA	NM_171998	Hs.632832	NM_171998
UNC93B1	-6.3	2.7	unc-93 homolog B1 (C. elegans) (UNC93B1), mRNA	NM_030930	Hs.502989	NM_030930
AGPS	-6.3	2.0	alkylglycerone phosphate synthase (AGPS), mRNA	NM_003659	Hs.591631	NM_003659
BXDC1	-6.3	2.0	brix domain containing 1 (BXDC1), mRNA	NM_032194	Hs.372265	AL833613
LOC389641	-6.3	3.4	cDNA FLJ42301 fis, clone TOVAR2002514.	AK124295	Hs.591835	AK124295
PITPNC1	-6.3	3.9	phosphatidylinositol transfer protein, cytoplasmic 1 (PITPNC1), transcript variant 2, mRNA	NM_181671	Hs.591185	NM_181671
LCK	-6.3	36.7	lymphocyte-specific protein tyrosine kinase (LCK), transcript variant 2, mRNA	NM_005356	Hs.470627	BC013200
ARL11	-6.3	3.1	ADP-ribosylation factor-like 11 (ARL11), mRNA	NM_138450	Hs.558599	AF441378
HPS3	-6.3	2.4	Hermansky-Pudlak syndrome 3 (HPS3), mRNA	NM_032383	Hs.591311	NM_032383
CXADR	-6.3	3.2	coxsackie virus and adenovirus receptor (CXADR), mRNA	NM_001338	Hs.634837	BC003684
PSMD12	-6.3	2.1	proteasome (prosome, macropain) 26S subunit, non-ATPase, 12 (PSMD12), mRNA	NM_002816	Hs.646575	AB003103
SPIB	-6.3	4.7	Spi-B transcription factor (Spi-1/PU.1 related) (SPIB), mRNA	NM_003121	Hs.437905	AK225225
ZNF134	-6.3	1.6	zinc finger protein 134 (ZNF134), mRNA	NM_003435	Hs.469694	BC042636
CAPG	-6.3	3.2	capping protein (actin filament), gelsolin-like (CAPG), mRNA	NM_001747	Hs.516155	AK130130
A_24_P912871	-6.3	2.0	A_24_P912871	A_24_P912871	Unknown	
DAZAP1	-6.3	2.6	DAZ associated protein 1 (DAZAP1), transcript variant 1, mRNA	NM_170711	Hs.222510	AK124583
PRSS16	-6.3	2.6	protease, serine, 16 (thymus) (PRSS16), mRNA	NM_005865	Hs.274407	AK126160
MAP7	-6.3	2.9	microtubule-associated protein 7 (MAP7), mRNA	NM_003980	Hs.486548	NM_003980
ALG1	-6.3	2.4	asparagine-linked glycosylation 1 homolog (S. cerevisiae, beta-1,4-mannosyltransferase) (ALG1), mRNA	NM_019109	Hs.592086	NM_019109
NCR1	-6.3	9.7	natural cytotoxicity triggering receptor 1, mRNA (cDNA clone MGC:65100 IMAGE:5218848), complete cds.	BC064806	Hs.97084	BC064806
SOX2	-6.3	7.4	SRY (sex determining region Y)-box 2 (SOX2), mRNA	NM_003106	Hs.518438	NM_003106
CDR2	-6.3	2.3	cerebellar degeneration-related protein 2, 62kDa (CDR2), mRNA	NM_001802	Hs.513430	BC017503
TMEM177	-6.3	1.8	transmembrane protein 177 (TMEM177), mRNA	NM_030577	Hs.439991	AK057313
SPECC1	-6.3	2.5	sperm antigen with calponin homology and coiled-coil domains 1 (SPECC1), transcript variant NSP5beta3beta, mRNA	NM_001033553	Hs.431045	BC033618
BRAF	-6.2	2.5	v-raf murine sarcoma viral oncogene homolog B1 (BRAF), mRNA	NM_004333	Hs.550061	M95712
KIAA1804	-6.2	8.2	mixed lineage kinase 4 (KIAA1804), mRNA	NM_032435	Hs.547779	NM_032435

ZFAND5	-6.2	1.9	zinc finger, AN1-type domain 5 (ZFAND5), mRNA	NM_006007	Hs.406096	BX648551
PTPN2	-6.2	2.0	protein tyrosine phosphatase, non-receptor type 2 (PTPN2), transcript variant 1, mRNA	NM_002828	Hs.654527	AB209569
THC2691500	-6.2	3.5	Q206M1_9ARAC (Q206M1) Major ampullate spidroin 2 (Fragment), partial (3%)	THC2691500	Unknown	
BM045853	-6.2	6.1	603624848F1 NIH_MGC_40 cDNA clone IMAGE:5451514 5', mRNA sequence	BM045853	Hs.569458	BM045853
LCK	-6.2	37.2	lymphocyte-specific protein tyrosine kinase (LCK), transcript variant 2, mRNA	NM_005356	Hs.470627	BC013200
ELL3	-6.2	4.3	elongation factor RNA polymerase II-like 3 (ELL3), mRNA	NM_025165	Hs.699876	AK126384
GABRB3	-6.2	19.2	gamma-aminobutyric acid (GABA) A receptor, beta 3 (GABRB3), transcript variant 1, mRNA	NM_000814	Hs.302352	NM_000814
GRPR	-6.2	4.3	Human GRP/bombesin receptor mRNA, partial cds.	U57365	Hs.567282	NM_005314
AKR1CL2	-6.2	2.1	mRNA for aldo-keto reductase related protein 1, complete cds.	AB040820	Hs.657944	CR598545
THC2545456	-6.2	2.7	THC2545456	THC2545456	Unknown	
WDR51A	-6.2	2.0	WD repeat domain 51A (WDR51A), mRNA	NM_015426	Hs.476306	BC110877
FAM60A	-6.2	1.7	family with sequence similarity 60, member A (FAM60A), mRNA	NM_021238	Hs.505154	BX648630
TUBA1	-6.2	8.8	tubulin, alpha 1 (TUBA1), mRNA	NM_006000	Hs.75318	AK054731
TRIM37	-6.2	2.0	tripartite motif-containing 37 (TRIM37), transcript variant 1, mRNA	NM_015294	Hs.579079	NM_015294
GNAO1	-6.2	6.9	Guanine nucleotide-binding protein G(o) subunit alpha 2.	ENST00000262[illegible]	Unknown	
KIAA1553	-6.2	2.7	OTTHUMP00000016928.	ENST00000369[illegible]	Unknown	
PDCD2L	-6.2	2.2	programmed cell death 2-like (PDCD2L), mRNA	NM_032346	Hs.515344	CR603540
GRB7	-6.2	2.8	growth factor receptor-bound protein 7 (GRB7), transcript variant 1, mRNA	NM_005310	Hs.86859	NM_005310
C17orf63	-6.2	2.4	chromosome 17 open reading frame 63 (C17orf63), transcript variant 2, mRNA	NM_018182	Hs.564533	NM_018182
LCK	-6.2	34.6	lymphocyte-specific protein tyrosine kinase (LCK), transcript variant 2, mRNA	NM_005356	Hs.470627	BC013200
TERT	-6.2	4.3	telomerase reverse transcriptase (TERT), transcript variant 1, mRNA	NM_198253	Hs.492203	AF018167
A_32_P169353	-6.2	7.3	A_32_P169353	A_32_P169353	Unknown	
HMBS	-6.2	1.8	hydroxymethylbilane synthase (HMBS), transcript variant 1, mRNA	NM_000190	Hs.82609	BU168137
KIAA1804	-6.2	4.4	mixed lineage kinase 4 (KIAA1804), mRNA	NM_032435	Hs.547779	NM_032435
FGF2	-6.2	5.2	fibroblast growth factor 2 (basic) (FGF2), mRNA	NM_002006	Hs.284244	NM_002006
SLC25A19	-6.2	2.4	solute carrier family 25 (mitochondrial deoxynucleotide carrier), member 19 (SLC25A19), mRNA	NM_021734	Hs.514470	AK097882
PSMD12	-6.2	2.1	proteasome (prosome, macropain) 26S subunit, non-ATPase, 12 (PSMD12), mRNA	NM_002816	Hs.646575	AB003103
LOC441795	-6.2	2.9	PREDICTED: similar to high-mobility group box 3 (LOC441795), mRNA	ENST00000332[illegible]	Unknown	
BRAF	-6.2	2.4	v-raf murine sarcoma viral oncogene homolog B1 (BRAF), mRNA	NM_004333	Hs.550061	M95712
LOC645360	-6.2	2.9	PREDICTED: similar to high-mobility group box 3 (LOC645360), mRNA	XR_018797	Hs.693337	XR_018797
TERT	-6.2	4.2	telomerase reverse transcriptase (TERT), transcript variant 1, mRNA	NM_198253	Hs.492203	AF018167
POLR1B	-6.2	2.3	polymerase (RNA) I polypeptide B, 128kDa (POLR1B), mRNA	NM_019014	Hs.86337	BX647683
HCP5	-6.2	2.8	P5-1 mRNA, complete cds.	L06175	Hs.654480	L06175
LOC345041	-6.1	2.0	PREDICTED: similar to 60 kDa heat shock protein, mitochondrial precursor (Hsp60) (60 kDa chaperonin) (CPN60) (Heat shock protein 60) (HSP-60) (Mitochondrial matrix protein P1) (P60 lymphocyte protein) (HuCHA60) (LOC345041), mRNA	XR_018747	Hs.647976	XR_018747
ENST00000292[illegible]	-6.1	5.9	mRNA for KIAA1653 protein, partial cds.	ENST00000292[illegible]	Unknown	
LOC388564	-6.1	1.8	hypothetical gene supported by BC052596, mRNA (cDNA clone IMAGE:6728287).	ENST00000376[illegible]	Unknown	
C10orf95	-6.1	2.4	chromosome 10 open reading frame 95 (C10orf95), mRNA	NM_024886	Hs.225084	AK024342
SS18L2	-6.1	1.8	synovial sarcoma translocation gene on chromosome 18-like 2 (SS18L2), mRNA	NM_016305	Hs.534454	BQ278408
LOC200810	-6.1	2.1	similar to beta-1,4-mannosyltransferase; beta-1,4 mannosyltransferase (LOC200810), mRNA	NM_001015050	Hs.591299	BX382902
EXOSC3	-6.1	1.7	exosome component 3 (EXOSC3), transcript variant 1, mRNA	NM_016042	Hs.591076	NM_016042
C13orf34	-6.1	1.5	chromosome 13 open reading frame 34 (C13orf34), mRNA	NM_024808	Hs.643464	NM_024808
ERBB2	-6.1	3.0	v-erb-b2 erythroblastic leukemia viral oncogene homolog 2, neuro/glioblastoma derived oncogene homolog (avian) (ERBB2), transcript variant 2, mRNA	NM_001005862	Hs.446352	NM_001005862
COCH	-6.1	5.0	coagulation factor C homolog, cochlin (Limulus polyphemus) (COCH), mRNA	NM_004086	Hs.21016	AK123362
MTHFD1	-6.1	2.3	methylenetetrahydrofolate dehydrogenase (NADP+ dependent) 1, methenyltetrahydrofolate cyclohydrolase, formyltetrahydrofolate synthetase (MTHFD1), mRNA	NM_005956	Hs.652308	BC050420
CRYGD	-6.1	6.3	crystallin, gamma D (CRYGD), mRNA	NM_006891	Hs.546247	CD049752
BC073929	-6.1	2.1	cDNA clone IMAGE:5196961, partial cds.	BC073929	Hs.389638	AL832779
SOLH	-6.1	3.0	small optic lobes homolog (Drosophila), mRNA (cDNA clone IMAGE:5518093), partial cds.	BC032648	Hs.632219	NM_005632
FZD5	-6.1	15.1	frizzled homolog 5 (Drosophila) (FZD5), mRNA	NM_003468	Hs.17631	NM_003468
C21orf88	-6.1	1.9	chromosome 21 open reading frame 88 (C21orf88), mRNA	NM_153754	Hs.375120	BC080530
TERT	-6.1	4.5	telomerase reverse transcriptase (TERT), transcript variant 1, mRNA	NM_198253	Hs.492203	AF018167
ISOC2	-6.1	2.0	isochorismatase domain containing 2 (ISOC2), mRNA	NM_024710	Hs.467306	AK097280
ANAPC1	-6.1	2.3	anaphase promoting complex subunit 1 (ANAPC1), mRNA	NM_022662	Hs.436527	NM_022662
DHX33	-6.1	3.3	DEAH (Asp-Glu-Ala-His) box polypeptide 33 (DHX33), mRNA	NM_020162	Hs.250456	NM_020162

HMBS	-6.1	1.8	hydroxymethylbilane synthase (HMBS), transcript variant 1, mRNA	NM_000190	Hs.82609	BU168137
ERBB2	-6.1	3.0	v-erb-b2 erythroblastic leukemia viral oncogene homolog 2, neuro/glioblastoma derived oncogene homolog (avian) (ERBB2), transcript variant 2, mRNA	NM_001005862	Hs.446352	NM_001005862
LOC200810	-6.1	2.2	similar to beta-1,4-mannosyltransferase; beta-1,4 mannosyltransferase (LOC200810), mRNA	NM_001015050	Hs.591299	BX382902
RGL3	-6.1	3.2	ral guanine nucleotide dissociation stimulator-like 3	ENST00000380[illegible]	Unknown	
GEMIN7	-6.1	1.8	gem (nuclear organelle) associated protein 7 (GEMIN7), transcript variant 1, mRNA	NM_024707	Hs.466919	NM_024707
LCK	-6.1	38.5	lymphocyte-specific protein tyrosine kinase (LCK), transcript variant 2, mRNA	NM_005356	Hs.470627	BC013200
LOC645808	-6.1	2.4	PREDICTED: similar to 60 kDa heat shock protein, mitochondrial precursor (Hsp60) (60 kDa chaperonin) (CPN60) (Heat shock protein 60) (HSP-60) (Mitochondrial matrix protein P1) (P60 lymphocyte protein) (HuCHA60) (LOC645808), mRNA	XR_016831	Hs.646762	XR_016831
ERBB2	-6.1	3.0	v-erb-b2 erythroblastic leukemia viral oncogene homolog 2, neuro/glioblastoma derived oncogene homolog (avian) (ERBB2), transcript variant 2, mRNA	NM_001005862	Hs.446352	NM_001005862
CHAC2	-6.1	2.2	ChaC, cation transport regulator homolog 2 (E. coli) (CHAC2), mRNA	NM_001008708	Hs.585944	AK097260
C15orf42	-6.1	3.2	chromosome 15 open reading frame 42 (C15orf42), mRNA	NM_152259	Hs.441708	NM_152259
A_23_P118135	-6.1	2.0	A_23_P118135	A_23_P118135	Unknown	
USHBP1	-6.1	3.6	Usher syndrome 1C binding protein 1 (USHBP1), mRNA	NM_031941	Hs.512773	BC027910
ERBB2	-6.1	3.0	v-erb-b2 erythroblastic leukemia viral oncogene homolog 2, neuro/glioblastoma derived oncogene homolog (avian) (ERBB2), transcript variant 2, mRNA	NM_001005862	Hs.446352	NM_001005862
LOC389023	-6.1	2.3	hypothetical gene supported by BC032913; BC048425, mRNA (cDNA clone IMAGE:5265535).	BC032913	Hs.97540	BU569564
DDX18	-6.1	2.2	DEAD (Asp-Glu-Ala-Asp) box polypeptide 18 (DDX18), mRNA	NM_006773	Hs.363492	AB209392
RTP1	-6.1	12.7	receptor (chemosensory) transporter protein 1 (RTP1), mRNA	NM_153708	Hs.518480	BC065202
FLJ38973	-6.1	1.9	hypothetical protein FLJ38973 (FLJ38973), mRNA	NM_153689	Hs.471040	BC036456
PIF1	-6.1	3.8	PIF1 5'-to-3' DNA helicase homolog (S. cerevisiae) (PIF1), mRNA	NM_025049	Hs.112160	AB185926
CB305813	-6.1	2.4	CB305813 UI-CF-EN1-aeg-d-07-0-UI.s1 UI-CF-EN1 cDNA clone UI-CF-EN1-aeg-d-07-0-UI 3', mRNA sequence	CB305813	Hs.473152	NM_003222
C6orf115	-6.1	1.9	chromosome 6 open reading frame 115, mRNA (cDNA clone IMAGE:4849571), complete cds.	BC014953	Hs.600861	BM542613
RMND5B	-6.1	1.8	cDNA FLJ36746 fis, clone UTERU2016757.	AK094065	Hs.519804	AK122692
CD048206	-6.1	61.4	AGENCOURT_13966160 NIH_MGC_172 cDNA 5', mRNA sequence	CD048206	Hs.642978	CD247471
SNAI3	-6.1	3.7	snail homolog 3 (Drosophila) (SNAI3), mRNA	NM_178310	Hs.499548	BX640980
NSD1	-6.0	2.2	nuclear receptor binding SET domain protein 1 (NSD1), transcript variant 2, mRNA	NM_022455	Hs.654666	NM_022455
CDYL	-6.0	1.9	chromodomain protein, Y-like (CDYL), transcript variant 3, mRNA	NM_170752	Hs.269092	NM_170751
TERT	-6.0	4.4	telomerase reverse transcriptase (TERT), transcript variant 1, mRNA	NM_198253	Hs.492203	AF018167
AK055696	-6.0	4.3	cDNA FLJ31134 fis, clone IMR322000984.	AK055696	Hs.231895	AK095590
CPT1A	-6.0	4.4	carnitine palmitoyltransferase 1A (liver) (CPT1A), nuclear gene encoding mitochondrial protein, transcript variant 2, mRNA	NM_001031847	Hs.503043	AK172798
AL117636	-6.0	3.8	mRNA; cDNA DKFZp434H205 (from clone DKFZp434H205).	AL117636	Hs.592750	AL117636
HPS3	-6.0	2.3	Hermansky-Pudlak syndrome 3 (HPS3), mRNA	NM_032383	Hs.591311	NM_032383
TARS	-6.0	1.9	threonyl-tRNA synthetase (TARS), mRNA	NM_152295	Hs.481860	NM_152295
ZNF770	-6.0	1.9	zinc finger protein 770 (ZNF770), mRNA	NM_014106	Hs.5327	BC071603
DNMT3B	-6.0	34.4	DNA (cytosine-5-)-methyltransferase 3 beta (DNMT3B), transcript variant 6, mRNA	NM_175850	Hs.655708	DQ321787
VCL	-6.0	2.2	vinculin (VCL), transcript variant 1, mRNA	NM_014000	Hs.699180	NM_014000
TAP1	-6.0	6.3	transporter 1, ATP-binding cassette, sub-family B (MDR/TAP) (TAP1), mRNA	NM_000593	Hs.352018	BX648013
WDR75	-6.0	1.7	WD repeat domain 75 (WDR75), mRNA	NM_032168	Hs.399984	NM_032168
SLC2A13	-6.0	2.8	solute carrier family 2 (facilitated glucose transporter), member 13 (SLC2A13), mRNA	NM_052885	Hs.558595	NM_052885
MTHFD1L	-6.0	1.9	truncated C1-tetrahydrofolate synthase mRNA, complete cds; nuclear gene for mitochondrial product; alternatively spliced.	AY374131	Hs.591343	AK127089
KIAA0020	-6.0	1.7	KIAA0020 (KIAA0020), mRNA	NM_014878	Hs.493309	NM_014878
ZNF532	-6.0	2.3	zinc finger protein 532 (ZNF532), mRNA	NM_018181	Hs.529023	NM_018181
FXYD5	-6.0	4.4	FXYD domain containing ion transport regulator 5 (FXYD5), transcript variant 1, mRNA	NM_144779	Hs.333418	BX648809
DCC1	-6.0	2.7	defective in sister chromatid cohesion homolog 1 (S. cerevisiae) (DCC1), mRNA	NM_024094	Hs.315167	BC001316
PHF15	-6.0	5.0	PHD finger protein 15 (PHF15), mRNA	NM_015288	Hs.483419	D87076
C16orf53	-6.0	2.0	chromosome 16 open reading frame 53 (C16orf53), mRNA	NM_024516	Hs.676031	CR609250
IFITM1	-6.0	5.6	interferon induced transmembrane protein 1 (9-27) (IFITM1), mRNA	NM_003641	Hs.458414	BF210063
FLJ12949	-6.0	1.9	hypothetical protein FLJ12949 (FLJ12949), transcript variant 1, mRNA	NM_023008	Hs.699549	NM_023008
TTLL12	-6.0	2.4	tubulin tyrosine ligase-like family, member 12 (TTLL12), mRNA	NM_015140	Hs.517670	D63487
BRAF	-6.0	2.5	v-raf murine sarcoma viral oncogene homolog B1 (BRAF), mRNA	NM_004333	Hs.550061	M95712
STRBP	-6.0	2.1	spermatid perinuclear RNA binding protein (STRBP), mRNA	NM_018387	Hs.696159	NM_018387
UBE2D2	-6.0	2.2	ubiquitin-conjugating enzyme E2D 2 (UBC4/5 homolog, yeast) (UBE2D2), transcript variant 2, mRNA	NM_181838	Hs.108332	NM_181838
THC2655527	-6.0	3.3	THC2655527	THC2655527	Unknown	
GNG4	-6.0	2.2	guanine nucleotide binding protein (G protein), gamma 4 (GNG4), mRNA	NM_004485	Hs.159711	NM_001098722
BRUNOL4	-6.0	1.9	bruno-like 4, RNA binding protein (Drosophila) (BRUNOL4), mRNA	NM_020180	Hs.435976	NM_020180

LOC91431	-6.0	1.8	prematurely terminated mRNA decay factor-like (LOC91431), mRNA	NM_138698	Unknown	
TJP2	-6.0	2.7	tight junction protein 2 (zona occludens 2) (TJP2), transcript variant 1, mRNA	NM_004817	Hs.50382	AB209630
A_24_P307466	-6.0	2.0	A_24_P307466	A_24_P307466	Unknown	
SNURF	-6.0	6.5	SNRPN upstream reading frame (SNURF), transcript variant 2, mRNA	NM_022804	Hs.564847	U81001
FABP5	-6.0	2.2	fatty acid binding protein 5 (psoriasis-associated) (FABP5), mRNA	NM_001444	Hs.408061	BM563703
GNPNAT1	-6.0	2.2	glucosamine-phosphate N-acetyltransferase 1 (GNPNAT1), mRNA	NM_198066	Hs.478025	NM_198066
LOC441461	-5.9	2.5	hypothetical gene supported by BC030123, mRNA (cDNA clone IMAGE:4815474).	BC030123	Hs.163155	XM_499157
UGP2	-5.9	5.3	UDP-glucose pyrophosphorylase 2 (UGP2), transcript variant 1, mRNA	NM_006759	Hs.516217	BX537559
AK024566	-5.9	2.5	cDNA: FLJ20913 fis, clone ADSE00630.	AK024566	Hs.655063	AK024566
ARNTL2	-5.9	3.2	cycle-like factor CLIF mRNA, complete cds.	AF256215	Unknown	
PRR13	-5.9	2.1	proline rich 13 (PRR13), transcript variant 3, mRNA	NM_001005354	Hs.426359	BE906094
DHX37	-5.9	2.2	DEAH (Asp-Glu-Ala-His) box polypeptide 37 (DHX37), mRNA	NM_032656	Hs.107382	NM_032656
ZBTB7A	-5.9	1.9	zinc finger and BTB domain containing 7A (ZBTB7A), mRNA	NM_015898	Hs.591384	NM_015898
CDCA5	-5.9	1.9	cell division cycle associated 5 (CDCA5), mRNA	NM_080668	Hs.434886	BC011000
THC2696143	-5.9	2.5	THC2696143	THC2696143	Unknown	
DDX18	-5.9	2.2	DEAD (Asp-Glu-Ala-Asp) box polypeptide 18 (DDX18), mRNA	NM_006773	Hs.363492	AB209392
MICB	-5.9	3.6	MHC class I polypeptide-related sequence B (MICB), mRNA	NM_005931	Hs.211580	BC044218
TCF15	-5.9	1.7	transcription factor 15 (basic helix-loop-helix) (TCF15), mRNA	NM_004609	Hs.437	NM_004609
A_24_P230009	-5.9	1.5	A_24_P230009	A_24_P230009	Unknown	
HACL1	-5.9	2.3	2-hydroxyacyl-CoA lyase 1 (HACL1), mRNA	NM_012260	Hs.63290	NM_012260
ARHGEF5	-5.9	2.7	Rho guanine nucleotide exchange factor (GEF) 5 (ARHGEF5), mRNA	NM_005435	Hs.334	AK160365
A_24_P178523	-5.9	4.1	A_24_P178523	A_24_P178523	Unknown	
PMAIP1	-5.9	10.4	phorbol-12-myristate-13-acetate-induced protein 1 (PMAIP1), mRNA	NM_021127	Hs.96	NM_021127
GRB10	-5.9	3.3	growth factor receptor-bound protein 10 (GRB10), transcript variant 4, mRNA	NM_001001555	Hs.164060	D86962
PLA2G3	-5.9	8.4	phospholipase A2, group III (PLA2G3), mRNA	NM_015715	Hs.149623	AF220490
ERBB2	-5.9	2.9	v-erb-b2 erythroblastic leukemia viral oncogene homolog 2, neuro/glioblastoma derived oncogene homolog (avian) (ERBB2), transcript variant 2, mRNA	NM_001005862	Hs.446352	NM_001005862
PIK3CD	-5.9	4.1	phosphoinositide-3-kinase, catalytic, delta polypeptide (PIK3CD), mRNA	NM_005026	Hs.518451	NM_005026
KIAA1333	-5.9	2.1	KIAA1333 (KIAA1333), mRNA	NM_017769	Hs.509008	AB037754
TXNDC10	-5.9	2.2	thioredoxin domain containing 10 (TXNDC10), mRNA	NM_019022	Hs.440534	BX647846
UPP1	-5.9	5.2	uridine phosphorylase 1 (UPP1), transcript variant 2, mRNA	NM_181597	Hs.488240	BC007348
PKIB	-5.9	5.0	protein kinase (cAMP-dependent, catalytic) inhibitor beta (PKIB), transcript variant 1, mRNA	NM_181795	Hs.486354	CR749456
TCL1B	-5.9	6.4	T-cell leukemia/lymphoma 1B (TCL1B), transcript variant 2, mRNA	NM_199206	Hs.632346	NM_199206
MYO1E	-5.9	4.6	myosin IE (MYO1E), mRNA	NM_004998	Hs.654506	BC098392
TERT	-5.9	4.5	telomerase reverse transcriptase (TERT), transcript variant 1, mRNA	NM_198253	Hs.492203	AF018167
WDR4	-5.9	3.7	WD repeat domain 4 (WDR4), transcript variant 2, mRNA	NM_033661	Hs.248815	AK092786
HERC5	-5.9	3.5	hect domain and RLD 5 (HERC5), mRNA	NM_016323	Hs.26663	AY337518
UTP15	-5.9	2.4	UTP15, U3 small nucleolar ribonucleoprotein, homolog (S. cerevisiae) (UTP15), mRNA	NM_032175	Hs.406703	AL831972
HMBS	-5.9	1.8	hydroxymethylbilane synthase (HMBS), transcript variant 1, mRNA	NM_000190	Hs.82609	BU168137
BAG4	-5.9	2.1	BCL2-associated athanogene 4 (BAG4), mRNA	NM_004874	Hs.194726	BC038505
GSR	-5.9	2.5	glutathione reductase (GSR), mRNA	NM_000637	Hs.271510	BC035691
A_23_P98042	-5.9	2.9	A_23_P98042	A_23_P98042	Unknown	
TERT	-5.8	4.5	telomerase reverse transcriptase (TERT), transcript variant 1, mRNA	NM_198253	Hs.492203	AF018167
POLR3E	-5.8	1.8	polymerase (RNA) III (DNA directed) polypeptide E (80kD) (POLR3E), mRNA	NM_018119	Hs.460298	AB040885
DB352368	-5.8	3.0	DB352368 UTERU3 cDNA clone UTERU3005422 3', mRNA sequence	DB352368	Hs.657491	BG176988
CLTCL1	-5.8	1.9	clathrin, heavy chain-like 1 (CLTCL1), mRNA	NM_007098	Hs.368266	U41763
RRS1	-5.8	2.5	RRS1 ribosome biogenesis regulator homolog (S. cerevisiae) (RRS1), mRNA	NM_015169	Hs.71827	NM_015169
HMBS	-5.8	1.8	hydroxymethylbilane synthase (HMBS), transcript variant 1, mRNA	NM_000190	Hs.82609	BU168137
USP10	-5.8	1.8	ubiquitin specific peptidase 10 (USP10), mRNA	NM_005153	Hs.136778	NM_005153
MPHOSPH1	-5.8	1.9	M-phase phosphoprotein 1 (MPHOSPH1), mRNA	NM_016195	Hs.240	AB033337
ERBB2	-5.8	3.0	v-erb-b2 erythroblastic leukemia viral oncogene homolog 2, neuro/glioblastoma derived oncogene homolog (avian) (ERBB2), transcript variant 2, mRNA	NM_001005862	Hs.446352	NM_001005862
HSPA2	-5.8	6.8	heat shock 70kDa protein 2 (HSPA2), mRNA	NM_021979	Hs.432648	NM_021979
KIAA1622	-5.8	3.2	KIAA1622 (KIAA1622), transcript variant 1, mRNA	NM_058237	Hs.259599	NM_058237
BX102076	-5.8	4.6	BX102076 Soares adult brain N2b5HB55Y cDNA clone IMAGp998J06323, mRNA sequence	BX102076	Hs.356239	BX102076
CDK9	-5.8	2.3	cyclin-dependent kinase 9 (CDC2-related kinase) (CDK9), mRNA	NM_001261	Hs.557646	NM_001261
CDH26	-5.8	5.0	cadherin-like 26 (CDH26), transcript variant b, mRNA	NM_021810	Hs.54973	NM_177980
UNC13D	-5.8	2.4	unc-13 homolog D (C. elegans) (UNC13D), mRNA	NM_199242	Hs.41045	NM_199242

TIMM44	-5.8	2.8	translocase of inner mitochondrial membrane 44 homolog (yeast) (TIMM44), mRNA	NM_006351	Hs.465784	CR627419
BRAF	-5.8	2.5	v-raf murine sarcoma viral oncogene homolog B1 (BRAF), mRNA	NM_004333	Hs.550061	M95712
THC2618461	-5.8	2.5	CHKEN2A01 engrailed protein {Gallus gallus} (exp=-1; wgp=0; cg=0), partial (12%)	THC2618461	Unknown	
RFFL	-5.8	1.7	ring finger and FYVE-like domain containing 1 (RFFL), transcript variant 1, mRNA	NM_057178	Hs.13680	NM_057178
FGF2	-5.8	5.4	fibroblast growth factor 2 (basic) (FGF2), mRNA	NM_002006	Hs.284244	NM_002006
ZNF275	-5.8	2.3	Zinc finger protein 275.	ENST00000095[illegible]	Unknown	
THC2582438	-5.8	2.0	Q6DGI9_BRARE (Q6DGI9) Zgc:92899, partial (5%)	THC2582438	Unknown	
DCC1	-5.8	2.6	defective in sister chromatid cohesion homolog 1 (S. cerevisiae) (DCC1), mRNA	NM_024094	Hs.315167	BC001316
ADD2	-5.8	15.7	adducin 2 (beta) (ADD2), transcript variant beta-2, mRNA	NM_017482	Hs.188528	NM_017488
HMBS	-5.8	1.8	hydroxymethylbilane synthase (HMBS), transcript variant 1, mRNA	NM_000190	Hs.82609	BU168137
ERBB2	-5.8	3.0	v-erb-b2 erythroblastic leukemia viral oncogene homolog 2, neuro/glioblastoma derived oncogene homolog (avian) (ERBB2), transcript variant 2, mRNA	NM_001005862	Hs.446352	NM_001005862
BC032716	-5.8	11.5	cDNA clone IMAGE:5518697, **** WARNING: chimeric clone ****.	BC032716	Unknown	
WDR5	-5.8	2.4	WD repeat domain 5 (WDR5), transcript variant 1, mRNA	NM_017588	Hs.397638	NM_017588
ATP4A	-5.8	2.7	ATPase, H+/K+ exchanging, alpha polypeptide (ATP4A), mRNA	NM_000704	Hs.36992	AL832971
DSCR1L2	-5.8	3.2	Calcipressin-3 (Down syndrome candidate region 1-like protein 2) (Myocyte-enriched calcineurin-interacting protein 3) (MCIP3).	ENST00000374[illegible]	Unknown	
CTAGE6	-5.8	3.1	CTAGE family, member 6, mRNA (cDNA clone MGC:41943 IMAGE:5296763), complete cds.	BC043153	Hs.676333	NM_178561
OTUD7B	-5.8	2.1	OTU domain containing 7B (OTUD7B), mRNA	NM_020205	Hs.98322	NM_020205
COL8A1	-5.8	6.5	collagen, type VIII, alpha 1 (COL8A1), transcript variant 1, mRNA	NM_001850	Hs.654548	AF170702
MCM4	-5.8	2.5	MCM4 minichromosome maintenance deficient 4 (S. cerevisiae) (MCM4), transcript variant 1, mRNA	NM_005914	Hs.460184	NM_005914
HDAC4	-5.8	3.7	histone deacetylase 4 (HDAC4), mRNA	NM_006037	Hs.20516	NM_006037
QTRTD1	-5.8	1.7	queuine tRNA-ribosyltransferase domain containing 1 (QTRTD1), mRNA	NM_024638	Hs.477162	NM_024638
ARNTL2	-5.8	5.6	aryl hydrocarbon receptor nuclear translocator-like 2 (ARNTL2), mRNA	NM_020183	Hs.663740	AF231338
MRPS30	-5.8	2.2	mitochondrial ribosomal protein S30 (MRPS30), nuclear gene encoding mitochondrial protein, mRNA	NM_016640	Hs.591747	NM_016640
THC2686343	-5.8	55.9	THC2686343	THC2686343	Unknown	
TMEM180	-5.8	1.7	transmembrane protein 180 (TMEM180), mRNA	NM_024789	Hs.309069	NM_024789
FOXH1	-5.7	5.2	forkhead box H1 (FOXH1), mRNA	NM_003923	Hs.652162	AF076292
KLK10	-5.7	3.2	kallikrein-related peptidase 10 (KLK10), transcript variant 1, mRNA	NM_002776	Hs.275464	NM_002776
LRPPRC	-5.7	1.5	leucine-rich PPR-motif containing (LRPPRC), mRNA	NM_133259	Hs.368084	AY289212
PACSIN1	-5.7	2.7	protein kinase C and casein kinase substrate in neurons 1 (PACSIN1), mRNA	NM_020804	Hs.520087	BC040228
RTP1	-5.7	27.5	receptor (chemosensory) transporter protein 1 (RTP1), mRNA	NM_153708	Hs.518480	BC065202
TOMM7	-5.7	1.6	translocase of outer mitochondrial membrane 7 homolog (yeast) (TOMM7), mRNA	NM_019059	Hs.112318	CD048567
TMEM28	-5.7	4.0	transmembrane protein 28 (TMEM28), mRNA	NM_015686	Hs.87619	NM_015686
LOC439949	-5.7	3.2	clone CDABP0095 mRNA sequence.	AY007155	Hs.590987	XM_001128367
NETO1	-5.7	4.1	neuropilin (NRP) and tolloid (TLL)-like 1 (NETO1), transcript variant 1, mRNA	NM_138999	Hs.465407	AF448838
PPARGC1B	-5.7	6.7	peroxisome proliferator-activated receptor gamma, coactivator 1 beta (PPARGC1B), mRNA	NM_133263	Hs.591261	AY188950
SARS2	-5.7	1.9	seryl-tRNA synthetase 2 (SARS2), mRNA	NM_017827	Hs.696180	NM_017827
HSD17B12	-5.7	1.6	hydroxysteroid (17-beta) dehydrogenase 12 (HSD17B12), mRNA	NM_016142	Hs.132513	BX537496
POLQ	-5.7	1.9	polymerase (DNA directed), theta (POLQ), mRNA	NM_199420	Hs.241517	AY032677
DOT1L	-5.7	2.0	DOT1-like, histone H3 methyltransferase (S. cerevisiae) (DOT1L), mRNA	NM_032482	Hs.591379	AB058717
AF247042	-5.7	3.7	tandem pore domain potassium channel TRAAK (KCNK4) mRNA, complete cds.	AF247042	Unknown	
GPR64	-5.7	7.0	G protein-coupled receptor 64 (GPR64), transcript variant 1, mRNA	NM_001079858	Hs.146978	BC063315
PMAIP1	-5.7	10.4	phorbol-12-myristate-13-acetate-induced protein 1 (PMAIP1), mRNA	NM_021127	Hs.96	NM_021127
RP3-510O8.5	-5.7	2.1	AAAL3045 (UNQ3045), mRNA	NM_207409	Hs.259563	CD513822
CLDN23	-5.7	2.4	claudin 23 (CLDN23), mRNA	NM_194284	Hs.183617	NM_194284
LOC284242	-5.7	8.8	Homo sapiens, clone IMAGE:5745916, mRNA.	BC035844	Hs.701425	BC035844
ASPHD2	-5.7	2.3	cDNA FLJ39838 fis, clone SPLEN2014119, weakly similar to Aspartate beta-hydroxylase, isoform a.	AK097157	Hs.567547	AK097157
ENST00000254[illegible]	-5.7	2.2	CDNA FLJ46156 fis, clone TESTI4001569.	ENST00000254[illegible]	Unknown	
ENST00000328[illegible]	-5.7	2.6	CSL-type zinc finger-containing protein 1.	ENST00000328[illegible]	Unknown	
RAB10	-5.7	1.8	RAB10, member RAS oncogene family (RAB10), mRNA	NM_016131	Hs.467960	AK023223
SCAMP5	-5.7	1.7	secretory carrier membrane protein 5 (SCAMP5), mRNA	NM_138967	Hs.374180	AL390185
DHFR	-5.7	1.9	dihydrofolate reductase (DHFR), mRNA	NM_000791	Hs.648635	NM_000791
TPMT	-5.7	2.2	thiopurine S-methyltransferase (TPMT), mRNA	NM_000367	Hs.444319	NM_000367
LQK1	-5.7	4.7	LQK1 hypothetical protein long isoform (LQK1) mRNA, complete cds, alternatively spliced.	AY030238	Hs.552649	AK092887
TERT	-5.7	4.5	telomerase reverse transcriptase (TERT), transcript variant 1, mRNA	NM_198253	Hs.492203	AF018167

CELSR3	-5.7	2.9	cadherin, EGF LAG seven-pass G-type receptor 3 (flamingo homolog, Drosophila) (CELSR3), mRNA	NM_001407	Hs.631926	NM_001407
SYNGR3	-5.7	2.4	synaptogyrin 3 (SYNGR3), mRNA	NM_004209	Hs.435277	NM_004209
HMBS	-5.7	1.8	hydroxymethylbilane synthase (HMBS), transcript variant 1, mRNA	NM_000190	Hs.82609	BU168137
TGS1	-5.7	1.9	trimethylguanosine synthase homolog (S. cerevisiae) (TGS1), mRNA	NM_024831	Hs.335068	AF286340
METAP1	-5.7	2.1	methionyl aminopeptidase 1 (METAP1), mRNA	NM_015143	Hs.480364	CR936632
PMAIP1	-5.7	10.5	phorbol-12-myristate-13-acetate-induced protein 1 (PMAIP1), mRNA	NM_021127	Hs.96	NM_021127
PMAIP1	-5.6	10.3	phorbol-12-myristate-13-acetate-induced protein 1 (PMAIP1), mRNA	NM_021127	Hs.96	NM_021127
PMAIP1	-5.6	10.7	phorbol-12-myristate-13-acetate-induced protein 1 (PMAIP1), mRNA	NM_021127	Hs.96	NM_021127
FLVCR	-5.6	5.9	feline leukemia virus subgroup C cellular receptor (FLVCR), mRNA	NM_014053	Hs.7055	AK001419
HAPLN3	-5.6	2.9	hyaluronan and proteoglycan link protein 3 (HAPLN3), mRNA	NM_178232	Hs.447530	BC053689
DDX18	-5.6	2.1	DEAD (Asp-Glu-Ala-Asp) box polypeptide 18 (DDX18), mRNA	NM_006773	Hs.363492	AB209392
ME2	-5.6	1.7	malic enzyme 2, NAD(+)-dependent, mitochondrial (ME2), nuclear gene encoding mitochondrial protein, mRNA	NM_002396	Hs.699163	NM_002396
DDX18	-5.6	2.2	DEAD (Asp-Glu-Ala-Asp) box polypeptide 18 (DDX18), mRNA	NM_006773	Hs.363492	AB209392
HMBS	-5.6	1.8	hydroxymethylbilane synthase (HMBS), transcript variant 1, mRNA	NM_000190	Hs.82609	BU168137
LARP1	-5.6	3.0	La ribonucleoprotein domain family, member 1 (LARP1), transcript variant 2, mRNA	NM_033551	Hs.292078	NM_033551
PPIL2	-5.6	2.4	peptidylprolyl isomerase (cyclophilin)-like 2 (PPIL2), transcript variant 1, mRNA	NM_014337	Hs.438587	AK127259
CTAGE5	-5.6	4.1	CTAGE family, member 5 (CTAGE5), transcript variant 4, mRNA	NM_203356	Hs.540038	AF338234
RASD2	-5.6	1.8	RASD family, member 2 (RASD2), mRNA	NM_014310	Hs.474711	BC013419
PRR15	-5.6	3.9	proline rich 15 (PRR15), mRNA	NM_175887	Hs.91109	NM_175887
PRKCA	-5.6	4.1	protein kinase C, alpha (PRKCA), mRNA	NM_002737	Hs.531704	NM_002737
ARID3B	-5.6	2.2	AT rich interactive domain 3B (BRIGHT-like) (ARID3B), mRNA	NM_006465	Hs.696207	AB208830
THC2714968	-5.6	2.4	Q2NY92_XANOM (Q2NY92) Methyltransferase, partial (3%)	THC2714968	Unknown	
ERBB2	-5.6	3.0	v-erb-b2 erythroblastic leukemia viral oncogene homolog 2, neuro/glioblastoma derived oncogene homolog (avian) (ERBB2), transcript variant 2, mRNA	NM_001005862	Hs.446352	NM_001005862
FLJ23834	-5.6	5.9	cDNA FLJ43271 fis, clone KIDNE2002882, highly similar to Cadherin.	AK125261	Hs.202120	AK126338
KCNH6	-5.6	5.0	potassium voltage-gated channel, subfamily H (eag-related), member 6 (KCNH6), transcript variant 2, mRNA	NM_173092	Hs.591177	NM_030779
ASNS	-5.6	4.5	asparagine synthetase, mRNA (cDNA clone IMAGE:5266877), **** WARNING: chimeric clone ****.	BC030024	Unknown	
PYGL	-5.6	2.4	phosphorylase, glycogen; liver (Hers disease, glycogen storage disease type VI) (PYGL), mRNA	NM_002863	Hs.282417	BC110791
NLN	-5.6	3.8	mRNA for KIAA1226 protein, partial cds.	AB033052	Hs.247460	AB033052
ECAT8	-5.6	8.0	cDNA FLJ13072 fis, clone NT2RP3001844.	AK023134	Hs.646351	NM_001015890
EIF2C2	-5.6	3.7	eukaryotic translation initiation factor 2C, 2 (EIF2C2), mRNA	NM_012154	Hs.696322	BC054491
BLM	-5.6	1.6	Bloom syndrome (BLM), mRNA	NM_000057	Hs.169348	NM_000057
C10orf12	-5.6	2.2	chromosome 10 open reading frame 12 (C10orf12), mRNA	NM_015652	Hs.427927	NM_015652
MCM5	-5.6	2.2	MCM5 minichromosome maintenance deficient 5, cell division cycle 46 (S. cerevisiae) (MCM5), mRNA	NM_006739	Hs.517582	NM_006739
NEK8	-5.6	3.9	NIMA (never in mitosis gene a)- related kinase 8 (NEK8), mRNA	NM_178170	Hs.448468	NM_178170
SALL2	-5.6	2.2	sal-like 2 (Drosophila) (SALL2), mRNA	NM_005407	Hs.416358	NM_005407
FGF2	-5.6	5.4	fibroblast growth factor 2 (basic) (FGF2), mRNA	NM_002006	Hs.284244	NM_002006
FOXA3	-5.6	3.2	forkhead box A3 (FOXA3), mRNA	NM_004497	Hs.36137	NM_004497
MGC4172	-5.6	3.0	short-chain dehydrogenase/reductase (MGC4172), mRNA	NM_024308	Hs.462859	CR592298
A_24_P490011	-5.6	2.0	A_24_P490011	A_24_P490011	Unknown	
CD048206	-5.6	73.4	AGENCOURT_13966160 NIH_MGC_172 cDNA 5', mRNA sequence	CD048206	Hs.642978	CD247471
HMBS	-5.6	1.7	hydroxymethylbilane synthase (HMBS), transcript variant 1, mRNA	NM_000190	Hs.82609	BU168137
VENTX	-5.6	20.8	VENT homeobox homolog (Xenopus laevis) (VENTX), mRNA	NM_014468	Hs.125231	AF068006
YTHDF1	-5.6	1.8	YTH domain family, member 1 (YTHDF1), mRNA	NM_017798	Hs.11747	BC050284
TDGF3	-5.6	44.8	Human (clone CR-3) teratocarcinoma-derived growth factor 3 (TDGF3) mRNA, complete cds.	M96956	Hs.592361	M96956
MYOHD1	-5.6	2.2	myosin head domain containing 1 (MYOHD1), transcript variant 1, mRNA	NM_025109	Hs.302051	AB209035
SCO1	-5.6	2.3	SCO cytochrome oxidase deficient homolog 1 (yeast) (SCO1), nuclear gene encoding mitochondrial protein, mRNA	NM_004589	Hs.14511	AF026852
ESCO2	-5.6	1.7	establishment of cohesion 1 homolog 2 (S. cerevisiae) (ESCO2), mRNA	NM_001017420	Hs.99480	AL832666
FANCD2	-5.6	2.6	Fanconi anemia, complementation group D2 (FANCD2), transcript variant 2, mRNA	NM_001018115	Hs.208388	BC038666
KIF26A	-5.6	2.2	kinesin family member 26A, mRNA (cDNA clone IMAGE:3502885), complete cds.	BC009415	Hs.134970	NM_015656
RCCD1	-5.6	2.1	RCC1 domain containing 1 (RCCD1), transcript variant 1, mRNA	NM_033544	Hs.655895	BC094739
GNB5	-5.6	31.4	guanine nucleotide binding protein (G protein), beta 5, mRNA (cDNA clone IMAGE:4131809), complete cds.	BC011671	Hs.155090	NM_016194
GMPS	-5.5	2.0	guanine monphosphate synthetase (GMPS), mRNA	NM_003875	Hs.591314	NM_003875
DDX18	-5.5	2.2	DEAD (Asp-Glu-Ala-Asp) box polypeptide 18 (DDX18), mRNA	NM_006773	Hs.363492	AB209392

ACADSB	-5.5	3.2	acyl-Coenzyme A dehydrogenase, short/branched chain (ACADSB), nuclear gene encoding mitochondrial protein, mRNA	NM_001609	Hs.81934	NM_001609
SERF1B	-5.5	2.2	small EDRK-rich factor 1B (centromeric) (SERF1B), mRNA	NM_022978	Hs.559428	NM_022978
XPO1	-5.5	1.8	exportin 1 (CRM1 homolog, yeast) (XPO1), mRNA	NM_003400	Hs.370770	AL833550
WRN	-5.5	1.4	Werner syndrome (WRN), mRNA	NM_000553	Hs.632050	NM_000553
GINS4	-5.5	3.4	GINS complex subunit 4 (Sld5 homolog) (GINS4), mRNA	NM_032336	Hs.656996	AK095334
CTBP2	-5.5	2.2	C-terminal binding protein 2 (CTBP2), transcript variant 1, mRNA	NM_001329	Hs.501345	NM_022802
FRMD1	-5.5	1.7	FERM domain containing 1 (FRMD1), mRNA	NM_024919	Hs.266746	AK074110
TERT	-5.5	4.6	telomerase reverse transcriptase (TERT), transcript variant 1, mRNA	NM_198253	Hs.492203	AF018167
APRT	-5.5	2.3	adenine phosphoribosyltransferase (APRT), transcript variant 1, mRNA	NM_000485	Hs.28914	CR622599
RET	-5.5	9.2	ret proto-oncogene (multiple endocrine neoplasia and medullary thyroid carcinoma 1, Hirschsprung disease) (RET), transcript variant 4, mRNA	NM_020630	Hs.350321	NM_020975
NUS1	-5.5	2.7	nuclear undecaprenyl pyrophosphate synthase 1 homolog (S. cerevisiae) (NUS1), [illegible]	NM_138459	Hs.289008	NM_138459
C14orf162	-5.5	3.5	chromosome 14 open reading frame 162 (C14orf162), mRNA	NM_020181	Unknown	
ZC3HAV1	-5.5	3.1	zinc finger CCCH-type, antiviral 1 (ZC3HAV1), transcript variant 2, mRNA	NM_024625	Hs.133512	NM_020119
ORC1L	-5.5	2.9	origin recognition complex, subunit 1-like (yeast) (ORC1L), mRNA	NM_004153	Hs.17908	NM_004153
DUSP16	-5.5	2.6	dual specificity phosphatase 16 (DUSP16), mRNA	NM_030640	Hs.536535	AL833073
ZFAND5	-5.5	1.8	zinc finger, AN1-type domain 5 (ZFAND5), mRNA	NM_006007	Hs.406096	BX648551
PIGL	-5.5	2.2	phosphatidylinositol glycan anchor biosynthesis, class L (PIGL), mRNA	NM_004278	Hs.499793	AK023469
AY358101	-5.5	2.0	clone DNA108695 Wpep3002 (UNQ3002) mRNA, complete cds.	AY358101	Hs.369998	NM_145663
BTBD7	-5.5	2.4	BTB (POZ) domain containing 7 (BTBD7), transcript variant 2, mRNA	NM_018167	Hs.525549	NM_001002860
RAB15	-5.5	2.7	RAB15, member RAS onocogene family (RAB15), mRNA	NM_198686	Hs.512492	NM_198686
LOC388494	-5.5	1.8	full-length cDNA clone CS0DF014YD20 of Fetal brain of (human).	CR593500	Hs.65750	NM_015174
LOC442293	-5.5	4.8	PREDICTED: similar to Glycine cleavage system H protein, mitochondrial precursor (LOC731353), mRNA	ENST000003298	Unknown	
DDX18	-5.5	2.2	DEAD (Asp-Glu-Ala-Asp) box polypeptide 18 (DDX18), mRNA	NM_006773	Hs.363492	AB209392
PRKCD	-5.5	2.3	protein kinase C, delta (PRKCD), transcript variant 1, mRNA	NM_006254	Hs.155342	NM_006254
DPPA2	-5.5	5.6	developmental pluripotency associated 2 (DPPA2), mRNA	NM_138815	Hs.351113	AY283672
NSUN5	-5.5	2.0	NOL1/NOP2/Sun domain family, member 5 (NSUN5), transcript variant 1, mRNA	NM_148956	Hs.647060	AK126375
QRSL1	-5.5	2.2	glutaminyl-tRNA synthase (glutamine-hydrolyzing)-like 1 (QRSL1), mRNA	NM_018292	Hs.406917	AL136679
LOC91431	-5.5	1.5	prematurely terminated mRNA decay factor-like (LOC91431), mRNA	NM_138698	Unknown	
TRIM36	-5.5	2.4	tripartite motif-containing 36 (TRIM36), transcript variant 1, mRNA	NM_018700	Hs.519514	NM_018700
GCNT2	-5.5	3.0	glucosaminyl (N-acetyl) transferase 2, I-branching enzyme (I blood group) (GCNT2), transcript variant 2, mRNA	NM_001491	Hs.519884	NM_001491
SWAP70	-5.5	2.1	SWAP-70 protein (SWAP70), mRNA	NM_015055	Hs.153026	NM_015055
CYB561	-5.5	1.8	cytochrome b-561 (CYB561), transcript variant 2, mRNA	NM_001017916	Hs.355264	AK095244
FAM77C	-5.5	2.2	family with sequence similarity 77, member C (FAM77C), mRNA	NM_024522	Hs.470259	AK022712
BC041899	-5.5	2.1	cDNA clone IMAGE:5298862.	BC041899	Hs.639379	BC041899
THC2578949	-5.5	4.8	AY918495 mitochondrial cytochrome c somatic {Macaca sylvanus} (exp=-1; wgp=0; cg=0), complete	THC2578949	Unknown	
ZFP64	-5.5	3.0	zinc finger protein 64 homolog (mouse) (ZFP64), transcript variant 4, mRNA	NM_199427	Hs.473082	NM_018197
A_23_P64184	-5.5	3.2	A_23_P64184	A_23_P64184	Unknown	
PFAS	-5.5	2.2	phosphoribosylformylglycinamidine synthase (FGAR amidotransferase) (PFAS), mRNA	NM_012393	Hs.573976	BC146768
FAM83H	-5.5	2.7	family with sequence similarity 83, member H (FAM83H), mRNA	NM_198488	Hs.67776	NM_198488
LSR	-5.5	2.6	lipolysis stimulated lipoprotein receptor (LSR), transcript variant 2, mRNA	NM_205834	Hs.466507	AK126834
A_24_P912502	-5.5	2.7	A_24_P912502	A_24_P912502	Unknown	
CR598940	-5.5	3.0	full-length cDNA clone CS0DI032YF23 of Placenta Cot 25-normalized of (human).	CR598940	Hs.561513	CR598940
THC2700053	-5.5	2.6	THC2700053	THC2700053	Unknown	
THC2693923	-5.5	1.7	AY175578 antibody variable domain {Oryctolagus cuniculus} (exp=-1; wgp=0; cg=0), partial (11%)	THC2693923	Unknown	
KCNH6	-5.5	6.7	cDNA FLJ33650 fis, clone BRAMY2024514, highly similar to Rattus norvegicus potassium channel (erg2) mRNA.	AK090969	Hs.591177	NM_030779
LIMK2	-5.5	2.7	LIM domain kinase 2 (LIMK2), transcript variant 1, mRNA	NM_001031801	Hs.474596	NM_016733
C6orf60	-5.5	1.4	chromosome 6 open reading frame 60 (C6orf60), mRNA	NM_024581	Hs.443789	BC060769
ANKHD1	-5.4	3.9	ankyrin repeat and KH domain containing 1 (ANKHD1), transcript variant 3, mRNA	NM_024668	Hs.653135	NM_020690
MANEAL	-5.4	3.2	mannosidase, endo-alpha-like (MANEAL), transcript variant 1, mRNA	NM_001031740	Hs.534562	AK055996
THC2579173	-5.4	1.7	ALU1_HUMAN (P39188) Alu subfamily J sequence contamination warning entry, partial (11%)	THC2579173	Unknown	
SOX13	-5.4	1.6	SRY (sex determining region Y)-box 13 (SOX13), mRNA	NM_005686	Hs.201671	NM_005686
CENTD3	-5.4	2.0	centaurin, delta 3 (CENTD3), mRNA	NM_022481	Hs.25277	NM_022481
UBE2D1	-5.4	1.9	ubiquitin-conjugating enzyme E2D 1 (UBC4/5 homolog, yeast) (UBE2D1), mRNA	NM_003338	Hs.129683	NM_003338
GNPTAB	-5.4	5.9	N-acetylglucosamine-1-phosphate transferase, alpha and beta subunits (GNPTAB), mRNA	NM_024312	Hs.46850	AM085438

HMBS	-5.4	1.7	hydroxymethylbilane synthase (HMBS), transcript variant 1, mRNA	NM_000190	Hs.82609	BU168137
MAPKAPK5	-5.4	1.6	mitogen-activated protein kinase-activated protein kinase 5 (MAPKAPK5), transcript variant 2, mRNA	NM_139078	Hs.413901	AK122767
LIN28	-5.4	12.4	lin-28 homolog (C. elegans) (LIN28), mRNA	NM_024674	Hs.86154	NM_024674
Z25424	-5.4	2.2	H.sapiens protein-serine/threonine kinase gene, complete CDS.	Z25424	Hs.515032	AB209074
DHX33	-5.4	3.5	DEAH (Asp-Glu-Ala-His) box polypeptide 33 (DHX33), mRNA	NM_020162	Hs.250456	NM_020162
BC062473	-5.4	4.6	cDNA clone IMAGE:30374677, partial cds.	BC062473	Hs.389700	AK123482
SOCS2	-5.4	2.3	suppressor of cytokine signaling 2 (SOCS2), mRNA	NM_003877	Hs.485572	BC070039
PPP1R14B	-5.4	2.4	protein phosphatase 1, regulatory (inhibitor) subunit 14B (PPP1R14B), mRNA	NM_138689	Hs.523760	BM911684
SLC7A6OS	-5.4	2.0	solute carrier family 7, member 6 opposite strand (SLC7A6OS), mRNA	NM_032178	Hs.653193	AK091674
BC039479	-5.4	3.9	Homo sapiens, clone IMAGE:5534210, mRNA.	BC039479	Hs.569831	BF665266
AL832582	-5.4	3.2	mRNA; cDNA DKFZp451G0416 (from clone DKFZp451G0416).	AL832582	Unknown	
CHST7	-5.4	5.6	carbohydrate (N-acetylglucosamine 6-O) sulfotransferase 7 (CHST7), mRNA	NM_019886	Hs.129955	NM_019886
FGF2	-5.4	5.2	fibroblast growth factor 2 (basic) (FGF2), mRNA	NM_002006	Hs.284244	NM_002006
ENST00000263	-5.4	1.9	PREDICTED: hypothetical protein LOC729440 (LOC729440), mRNA	ENST00000263	Unknown	
STAT3	-5.4	2.4	signal transducer and activator of transcription 3 (acute-phase response factor) (STAT3), transcript variant 3, mRNA	NM_213662	Hs.463059	NM_139276
GART	-5.4	2.1	phosphoribosylglycinamide formyltransferase, phosphoribosylglycinamide synthetase, phosphoribosylaminoimidazole synthetase (GART), transcript variant 1, mRNA	NM_000819	Hs.473648	BC068438
FGF2	-5.4	5.4	fibroblast growth factor 2 (basic) (FGF2), mRNA	NM_002006	Hs.284244	NM_002006
C21orf63	-5.4	2.4	chromosome 21 open reading frame 63 (C21orf63), mRNA	NM_058187	Hs.208358	NM_058187
NCAPH	-5.4	1.8	non-SMC condensin I complex, subunit H (NCAPH), mRNA	NM_015341	Hs.308045	NM_015341
FGD6	-5.4	2.1	FYVE, RhoGEF and PH domain containing 6 (FGD6), mRNA	NM_018351	Hs.506381	NM_018351
ENST00000354	-5.4	1.8	Serine/threonine/tyrosine-interacting protein (Protein tyrosine phosphatase-like protein).	ENST00000354	Unknown	
CTSC	-5.4	3.6	cathepsin C (CTSC), transcript variant 1, mRNA	NM_001814	Hs.128065	BX537913
BRAF	-5.4	2.4	v-raf murine sarcoma viral oncogene homolog B1 (BRAF), mRNA	NM_004333	Hs.550061	M95712
RFWD3	-5.4	3.6	ring finger and WD repeat domain 3 (RFWD3), mRNA	NM_018124	Hs.567525	NM_018124
A_32_P227400	-5.4	2.3	A_32_P227400	A_32_P227400	Unknown	
KIAA0241	-5.4	3.3	KIAA0241 (KIAA0241), mRNA	NM_015060	Hs.128056	D87682
REPS2	-5.4	2.7	RALBP1 associated Eps domain containing 2 (REPS2), mRNA	NM_004726	Hs.186810	NM_004726
PHF15	-5.4	5.0	PHD finger protein 15 (PHF15), mRNA	NM_015288	Hs.483419	D87076
TMEM155	-5.4	3.3	transmembrane protein 155 (TMEM155), mRNA	NM_152399	Hs.27524	BC059365
ZNF551	-5.4	2.5	zinc finger protein 551 (ZNF551), mRNA	NM_138347	Hs.656485	AK126625
SV2A	-5.4	2.8	synaptic vesicle glycoprotein 2A (SV2A), mRNA	NM_014849	Hs.516153	BC034038
SOD2	-5.4	2.4	superoxide dismutase 2, mitochondrial (SOD2), nuclear gene encoding mitochondrial protein, transcript variant 2, mRNA	NM_001024465	Hs.487046	AK097395
A_24_P799580	-5.4	2.1	A_24_P799580	A_24_P799580	Unknown	
GFER	-5.4	2.2	growth factor, augmenter of liver regeneration (ERV1 homolog, S. cerevisiae) (GFER), mRNA	NM_005262	Hs.27184	NM_005262
ZDHHC3	-5.4	2.0	zinc finger, DHHC-type containing 3 (ZDHHC3), mRNA	NM_016598	Hs.61430	AK127837
AF086052	-5.4	3.4	full length insert cDNA clone YY74A12.	AF086052	Hs.504370	BC106032
THC2539425	-5.4	2.4	XM_799236 mucin-associated surface protein (MASP) {Trypanosoma cruzi strain CL Brener} (exp=-1; wgp=0; cg=0), partial (6%)	THC2539425	Unknown	
CHRM3	-5.4	2.5	cholinergic receptor, muscarinic 3 (CHRM3), mRNA	NM_000740	Hs.7138	NM_000740
CORO2A	-5.4	5.5	coronin, actin binding protein, 2A (CORO2A), transcript variant 1, mRNA	NM_003389	Hs.113094	BX648086
GLT1D1	-5.4	3.2	glycosyltransferase 1 domain containing 1 (GLT1D1), mRNA	NM_144669	Hs.655668	BC043528
ARIH2	-5.4	2.2	ariadne homolog 2 (Drosophila) (ARIH2), mRNA	NM_006321	Hs.649132	NM_006321
DDX51	-5.4	2.0	DEAD (Asp-Glu-Ala-Asp) box polypeptide 51 (DDX51), mRNA	NM_175066	Hs.445168	NM_175066
SALL3	-5.4	7.9	sal-like 3 (Drosophila) (SALL3), mRNA	NM_171999	Hs.699525	NM_171999
RTEL1	-5.4	2.3	regulator of telomere elongation helicase 1 (RTEL1), transcript variant 1, mRNA	NM_016434	Hs.434878	AF217795
ENST00000330	-5.4	3.8	KIAA1244 (KIAA1244), mRNA	ENST00000330	Unknown	
BQ424374	-5.4	2.4	AGENCOURT_7892842 NIH_MGC_72 cDNA clone IMAGE:6157378 5', mRNA sequence	BQ424374	Hs.643605	BQ424374
LLGL2	-5.4	3.5	lethal giant larvae homolog 2 (Drosophila) (LLGL2), transcript variant 2, mRNA	NM_001015002	Hs.514477	NM_001031803
SLC6A15	-5.4	7.1	solute carrier family 6, member 15 (SLC6A15), transcript variant 2, mRNA	NM_018057	Hs.44424	NM_182767
PRR11	-5.4	1.5	cDNA FLJ11029 fis, clone PLACE1004156.	AK001891	Hs.631750	AK000296
C18orf54	-5.4	2.4	chromosome 18 open reading frame 54 (C18orf54), mRNA	NM_173529	Hs.208701	NM_173529
PMAIP1	-5.4	10.4	phorbol-12-myristate-13-acetate-induced protein 1 (PMAIP1), mRNA	NM_021127	Hs.96	NM_021127
BRAF	-5.4	2.5	v-raf murine sarcoma viral oncogene homolog B1 (BRAF), mRNA	NM_004333	Hs.550061	M95712
PLK1	-5.4	3.3	polo-like kinase 1 (Drosophila) (PLK1), mRNA	NM_005030	Hs.592049	AB209179
PIP5K1A	-5.4	2.8	phosphatidylinositol-4-phosphate 5-kinase, type I, alpha (PIP5K1A), mRNA	NM_003557	Hs.655131	NM_003557

C2orf37	-5.4	1.8	chromosome 2 open reading frame 37 (C2orf37), mRNA	NM_025000	Hs.659439	CR933646
FGD6	-5.4	2.3	FYVE, RhoGEF and PH domain containing 6 (FGD6), mRNA	NM_018351	Hs.506381	NM_018351
RTN4R	-5.4	2.1	reticulon 4 receptor (RTN4R), mRNA	NM_023004	Hs.30868	AK054602
A_32_P146320	-5.4	181.3	A_32_P146320	A_32_P146320	Unknown	
CAPN13	-5.3	4.7	cDNA FLJ23838 fis, clone KAT02256.	AK074418	Hs.660911	BX647678
GSTO2	-5.3	6.9	glutathione S-transferase omega 2 (GSTO2), mRNA	NM_183239	Hs.203634	AK094412
COCH	-5.3	5.3	coagulation factor C homolog, cochlin (Limulus polyphemus) (COCH), mRNA	NM_004086	Hs.21016	AK123362
AU146963	-5.3	2.4	AU146963 AU146963 HEMBB1 cDNA clone HEMBB1001996 3', mRNA sequence	AU146963	Hs.655425	BX648816
ARID1A	-5.3	2.0	AT rich interactive domain 1A (SWI-like) (ARID1A), transcript variant 1, mRNA	NM_006015	Hs.468972	NM_006015
A_24_P383751	-5.3	2.5	A_24_P383751	A_24_P383751	Unknown	
AK125299	-5.3	2.5	cDNA FLJ43309 fis, clone NT2RI2004618, highly similar to Cytosolic acyl coenzyme A thioester hydrolase (EC 3.1.2.2).	AK125299	Hs.534633	XR_000194
DHCR24	-5.3	3.5	24-dehydrocholesterol reductase (DHCR24), mRNA	NM_014762	Hs.498727	NM_014762
FGF2	-5.3	5.3	fibroblast growth factor 2 (basic) (FGF2), mRNA	NM_002006	Hs.284244	NM_002006
GFPT2	-5.3	3.2	glutamine-fructose-6-phosphate transaminase 2 (GFPT2), mRNA	NM_005110	Hs.696497	BC000012
FLJ20273	-5.3	3.7	RNA-binding protein (FLJ20273), mRNA	NM_019027	Hs.518727	NM_001098634
NSUN5B	-5.3	1.9	NOL1/NOP2/Sun domain family, member 5B (NSUN5B), transcript variant 1, mRNA	NM_001039575	Hs.699272	AK126486
TMEM102	-5.3	3.3	transmembrane protein 102 (TMEM102), mRNA	NM_178518	Hs.655662	BC046189
IL27RA	-5.3	2.3	interleukin 27 receptor, alpha (IL27RA), mRNA	NM_004843	Hs.132781	NM_004843
EIF4E3	-5.3	3.1	eukaryotic translation initiation factor 4E family member 3 (EIF4E3), mRNA	NM_173359	Hs.655608	AL161983
FGF2	-5.3	5.3	fibroblast growth factor 2 (basic) (FGF2), mRNA	NM_002006	Hs.284244	NM_002006
PMAIP1	-5.3	10.0	phorbol-12-myristate-13-acetate-induced protein 1 (PMAIP1), mRNA	NM_021127	Hs.96	NM_021127
ASMT	-5.3	6.3	acetylserotonin O-methyltransferase (ASMT), mRNA	NM_004043	Hs.522572	L03426
A_32_P89049	-5.3	191.6	A_32_P89049	A_32_P89049	Unknown	
AURKA	-5.3	2.5	aurora kinase A (AURKA), transcript variant 1, mRNA	NM_198433	Hs.250822	NM_198433
FAM101B	-5.3	2.8	family with sequence similarity 101, member B (FAM101B), mRNA	NM_182705	Hs.591203	NM_182705
PLCB3	-5.3	3.8	phospholipase C, beta 3 (phosphatidylinositol-specific) (PLCB3), mRNA	NM_000932	Hs.591953	BC146645
C6orf166	-5.3	1.7	chromosome 6 open reading frame 166 (C6orf166), mRNA	NM_018064	Hs.485915	CR599380
NLRP12	-5.3	20.2	NLR family, pyrin domain containing 12 (NLRP12), transcript variant 1, mRNA	NM_033297	Hs.631573	AY154467
KIAA0953	-5.3	3.0	mRNA for KIAA0953 protein, partial cds.	ENST00000334[illegible]	Unknown	
SPBC25	-5.3	2.1	spindle pole body component 25 homolog (S. cerevisiae) (SPBC25), mRNA	NM_020675	Hs.421956	BC022255
A_24_P734892	-5.3	1.8	A_24_P734892	A_24_P734892	Unknown	
A_24_P249187	-5.3	1.9	A_24_P249187	A_24_P249187	Unknown	
GART	-5.3	3.4	phosphoribosylglycinamide formyltransferase, phosphoribosylglycinamide synthetase, phosphoribosylaminoimidazole synthetase (GART), transcript variant 1, mRNA	NM_000819	Hs.473648	BC068438
AK125099	-5.3	1.5	cDNA FLJ43109 fis, clone CTONG2025516, moderately similar to general transcription factor II, i (GTF2I).	AK125099	Hs.588355	AK125099
C1orf172	-5.3	3.9	chromosome 1 open reading frame 172 (C1orf172), mRNA	NM_152365	Hs.188881	AK091952
ARHGEF10	-5.3	3.1	Rho guanine nucleotide exchange factor (GEF) 10 (ARHGEF10), mRNA	NM_014629	Hs.98594	CR749570
FLJ40432	-5.3	3.2	hypothetical protein FLJ40432 (FLJ40432), mRNA	NM_152523	Hs.471234	BC067253
LOC153346	-5.3	7.2	cDNA FLJ14284 fis, clone PLACE1005898.	AK024346	Hs.483816	BX648997
NCBP2	-5.3	2.4	nuclear cap binding protein subunit 2, 20kDa (NCBP2), transcript variant 1, mRNA	NM_007362	Hs.591671	AK093216
DDX21	-5.3	2.8	DEAD (Asp-Glu-Ala-Asp) box polypeptide 21 (DDX21), mRNA	NM_004728	Hs.696064	BX648405
DDX55	-5.3	2.3	DEAD (Asp-Glu-Ala-Asp) box polypeptide 55 (DDX55), mRNA	NM_020936	Hs.286173	AB046815
RABGAP1L	-5.3	3.1	mRNA for KIAA0471 protein, partial cds.	AB007940	Hs.585378	AB007940
USP31	-5.3	3.1	mRNA for KIAA1203 protein, partial cds.	AB033029	Hs.183817	NM_020718
FAM83F	-5.3	9.5	family with sequence similarity 83, member F (FAM83F), mRNA	NM_138435	Hs.197680	BC011204
KIAA1787	-5.3	1.8	KIAA1787 protein (KIAA1787), transcript variant 1, mRNA	NM_032442	Hs.654794	NM_032442
GJE1	-5.3	4.7	gap junction protein, epsilon 1, 29kDa, mRNA (cDNA clone IMAGE:5392714), with apparent retained intron.	BC038207	Hs.647524	NM_181538
TCN2	-5.3	4.0	transcobalamin II; macrocytic anemia (TCN2), mRNA	NM_000355	Hs.417948	AK225445
AK057719	-5.3	3.9	cDNA FLJ33157 fis, clone UTERU2000393.	AK057719	Hs.655511	AK057719
CNNM2	-5.3	2.5	cyclin M2 (CNNM2), transcript variant 1, mRNA	NM_017649	Hs.696431	NM_017649
FGF2	-5.3	5.4	fibroblast growth factor 2 (basic) (FGF2), mRNA	NM_002006	Hs.284244	NM_002006
FGF2	-5.3	5.3	fibroblast growth factor 2 (basic) (FGF2), mRNA	NM_002006	Hs.284244	NM_002006
THC2512741	-5.3	2.1	THC2512741	THC2512741	Unknown	
LLGL2	-5.3	3.2	lethal giant larvae homolog 2 (Drosophila) (LLGL2), transcript variant 1, mRNA	NM_004524	Hs.514477	NM_001031803
THC2621073	-5.3	3.1	Q5VW09_HUMAN (Q5VW09) OTTHUMP00000018325, partial (41%)	THC2621073	Unknown	
NANOG	-5.3	250.5	Nanog homeobox (NANOG), mRNA	NM_024865	Hs.661360	AB093576
SNCB	-5.3	2.8	synuclein, beta (SNCB), transcript variant 1, mRNA	NM_001001502	Hs.90297	AB209029

HMBS	-5.3	1.8	hydroxymethylbilane synthase (HMBS), transcript variant 1, mRNA	NM_000190	Hs.82609	BU168137
A_24_P910471	-5.3	67.5	A_24_P910471	A_24_P910471	Unknown	
PCBD2	-5.3	3.1	pterin-4 alpha-carbinolamine dehydratase/dimerization cofactor of hepatocyte nuclear factor 1 alpha (TCF1) 2 (PCBD2), mRNA	NM_032151	Hs.631804	NM_032151
L48692	-5.3	2.2	(clone p5-23-3) mRNA.	L48692	Hs.262858	NM_020143
A_32_P152696	-5.3	3.6	A_32_P152696	A_32_P152696	Unknown	
BC029655	-5.3	2.4	Homo sapiens, clone IMAGE:5167625, mRNA.	BC029655	Hs.530588	AK127982
RHOV	-5.3	2.8	ras homolog gene family, member V	ENST00000220[illegible]	Unknown	
AGPAT5	-5.3	1.9	1-acylglycerol-3-phosphate O-acyltransferase 5 (lysophosphatidic acid acyltransferase, epsilon) (AGPAT5), mRNA	NM_018361	Hs.624002	BX640918
WDR3	-5.3	1.4	WD repeat domain 3 (WDR3), mRNA	NM_006784	Hs.310809	NM_006784
MATK	-5.3	4.2	megakaryocyte-associated tyrosine kinase (MATK), transcript variant 1, mRNA	NM_139355	Hs.631845	BC000114
USHBP1	-5.3	3.3	Usher syndrome 1C binding protein 1 (USHBP1), mRNA	NM_031941	Hs.512773	BC027910
BC022928	-5.3	2.3	cDNA clone IMAGE:3457540.	BC022928	Hs.676211	BC022928
MCART2	-5.3	2.5	mitochondrial carrier triple repeat 2 (MCART2), mRNA	NM_001034172	Hs.567708	NM_001034172
TOM1L1	-5.3	2.1	target of myb1-like 1 (chicken) (TOM1L1), mRNA	NM_005486	Hs.153504	AK225351
UBE2G1	-5.3	2.4	ubiquitin-conjugating enzyme E2G 1 (UBC7 homolog, yeast) (UBE2G1), mRNA	NM_003342	Hs.462035	NM_003342
LAS1L	-5.3	1.9	LAS1-like (S. cerevisiae) (LAS1L), mRNA	NM_031206	Hs.522675	AK074087
CCNC	-5.2	1.9	cyclin C (CCNC), transcript variant 1, mRNA	NM_005190	Hs.430646	BC041123
BOP1	-5.2	2.6	block of proliferation 1 (BOP1), mRNA	NM_015201	Hs.535901	NM_015201
FASTKD1	-5.2	1.9	FAST kinase domains 1 (FASTKD1), mRNA	NM_024622	Hs.529276	AB058703
AF161353	-5.2	1.9	HSPC090 mRNA, partial cds.	AF161353	Hs.669325	AF150232
HSD11B2	-5.2	3.8	hydroxysteroid (11-beta) dehydrogenase 2 (HSD11B2), mRNA	NM_000196	Hs.1376	BC036780
CHURC1	-5.2	1.6	churchill domain containing 1 (CHURC1), mRNA	NM_145165	Hs.325531	NM_145165
ANKRD39	-5.2	1.8	ankyrin repeat domain 39 (ANKRD39), mRNA	NM_016466	Unknown	
MCCC2	-5.2	2.0	methylcrotonoyl-Coenzyme A carboxylase 2 (beta) (MCCC2), mRNA	NM_022132	Hs.604789	AK094987
LSM12	-5.2	2.7	LSM12 homolog (S. cerevisiae) (LSM12), mRNA	NM_152344	Hs.355570	BC044587
NUP35	-5.2	1.8	nucleoporin 35kDa (NUP35), transcript variant 1, mRNA	NM_138285	Hs.180591	BC111016
C14orf115	-5.2	130.6	chromosome 14 open reading frame 115 (C14orf115), mRNA	NM_018228	Hs.578167	NM_018228
PRSS8	-5.2	2.8	protease, serine, 8 (prostasin) (PRSS8), mRNA	NM_002773	Hs.75799	NM_002773
PROK2	-5.2	14.5	prokineticin 2 (PROK2), mRNA	NM_021935	Hs.528665	AF333025
SNTG2	-5.2	1.9	syntrophin, gamma 2 (SNTG2), mRNA	NM_018968	Hs.657453	AJ003029
SLC13A3	-5.2	2.4	solute carrier family 13 (sodium-dependent dicarboxylate transporter), member 3 (SLC13A3), transcript variant 2, mRNA	NM_001011554	Hs.655498	NM_001011554
ORC3L	-5.2	1.5	origin recognition complex, subunit 3-like (yeast) (ORC3L), transcript variant 1, mRNA	NM_181837	Hs.410228	NM_181837
WIBG	-5.2	2.1	within bgcn homolog (Drosophila) (WIBG), mRNA	NM_032345	Hs.505687	BC006135
FANCD2	-5.2	2.7	Fanconi anemia, complementation group D2 (FANCD2), transcript variant 2, mRNA	NM_001018115	Hs.208388	BC038666
LOC650186	-5.2	2.2	PREDICTED: similar to ATP-dependent RNA helicase DDX18 (DEAD box protein 18) (Myc-regulated DEAD box protein) (MrDb) (LOC650186), mRNA	XR_019017	Hs.646977	XR_019017
ALPL	-5.2	4.5	alkaline phosphatase, liver/bone/kidney (ALPL), mRNA	NM_000478	Hs.75431	NM_000478
JPH1	-5.2	3.1	junctophilin 1 (JPH1), mRNA	NM_020647	Hs.657367	NM_020647
GPR114	-5.2	3.8	G protein-coupled receptor 114 (GPR114), mRNA	NM_153837	Hs.187884	AK160368
HMG4L	-5.2	3.0	high-mobility group (nonhistone chromosomal) protein 4-like (HMG4L) on chromosome 20	NR_002165	Unknown	
BC089388	-5.2	3.3	cDNA clone IMAGE:30390722, containing frame-shift errors.	BC089388	Hs.526423	XR_016766
AA971667	-5.2	3.6	AA971667 op85c06.s1 Soares_NFL_T_GBC_S1 cDNA clone IMAGE:1583626 3', mRNA sequence	AA971667	Hs.655057	NM_014494
PMAIP1	-5.2	10.3	phorbol-12-myristate-13-acetate-induced protein 1 (PMAIP1), mRNA	NM_021127	Hs.96	NM_021127
OR5L2	-5.2	1.9	olfactory receptor, family 5, subfamily L, member 2 (OR5L2), mRNA	NM_001004739	Hs.528356	NM_001004739
SLC38A1	-5.2	1.9	solute carrier family 38, member 1 (SLC38A1), transcript variant 1, mRNA	NM_030674	Hs.699239	NM_030674
2'-PDE	-5.2	1.8	2'-phosphodiesterase (2'-PDE), mRNA	NM_177966	Hs.572993	NM_177966
A_23_P15226	-5.2	3.3	A_23_P15226	A_23_P15226	Unknown	
MGC70870	-5.2	2.4	hypothetical LOC403340 (MGC70870), mRNA	NM_203481	Unknown	
ZNF508	-5.2	1.5	zinc finger protein 508 (ZNF508), mRNA	NM_014913	Hs.131915	BC071589
LIN7A	-5.2	3.0	lin-7 homolog A (C. elegans) (LIN7A), mRNA	NM_004664	Hs.144333	AK127428
CHDH	-5.2	3.0	choline dehydrogenase (CHDH), mRNA	NM_018397	Hs.126688	AK055402
FAM62C	-5.2	4.4	chromosome 3 cDNA.	AJ697972	Hs.477711	AJ697972
TNKS1BP1	-5.2	2.3	tankyrase 1 binding protein 1, 182kDa (TNKS1BP1), mRNA	NM_033396	Hs.530730	NM_033396
RHOV	-5.2	2.4	ras homolog gene family, member V (RHOV), mRNA	NM_133639	Hs.447901	CR599960
CHAF1A	-5.2	2.1	chromatin assembly factor 1, subunit A (p150) (CHAF1A), mRNA	NM_005483	Hs.79018	NM_005483
OGDHL	-5.2	2.5	oxoglutarate dehydrogenase-like (OGDHL), mRNA	NM_018245	Hs.17860	BC026320

CR603272	-5.2	1.8	full-length cDNA clone CS0DC013YI10 of Neuroblastoma Cot 25-normalized of (human).	CR603272	Hs.696487	AK097068
LOC203547	-5.2	2.4	hypothetical protein LOC203547 (LOC203547), mRNA	NM_001017980	Hs.58633	AL833596
SLC26A6	-5.2	1.9	solute carrier family 26, member 6 (SLC26A6), transcript variant 4, mRNA	NM_001040454	Hs.631925	NM_001040454
LRIG1	-5.2	2.2	leucine-rich repeats and immunoglobulin-like domains 1 (LRIG1), mRNA	NM_015541	Hs.518055	BC071561
AK026140	-5.2	21.7	cDNA: FLJ22487 fis, clone HRC10931.	AK026140	Hs.632006	AK125281
PFAS	-5.2	3.1	phosphoribosylformylglycinamidine synthase (FGAR amidotransferase) (PFAS), mRNA	NM_012393	Hs.573976	BC146768
POMC	-5.2	3.2	proopiomelanocortin (adrenocorticotropin/ beta-lipotropin/ alpha-melanocyte stimulating hormone/ beta-melanocyte stimulating hormone/ beta-endorphin) (POMC), transcript variant 1, mRNA	NM_001035256	Hs.1897	CD518878
VPS53	-5.2	2.6	vacuolar protein sorting 53 homolog (S. cerevisiae) (VPS53), mRNA	NM_018289	Hs.461819	AK092532
GTSE1	-5.2	2.2	G-2 and S-phase expressed 1 (GTSE1), mRNA	NM_016426	Hs.386189	NM_016426
SFXN1	-5.2	1.8	sideroflexin 1 (SFXN1), mRNA	NM_022754	Hs.369440	BC063241
GRTP1	-5.2	3.4	growth hormone regulated TBC protein 1 (GRTP1), mRNA	NM_024719	Hs.170904	AK026127
B3GNT3	-5.2	3.1	UDP-GlcNAc:betaGal beta-1,3-N-acetylglucosaminyltransferase 3 (B3GNT3), mRNA	NM_014256	Hs.69009	NM_014256
MTHFD1L	-5.2	2.3	methylenetetrahydrofolate dehydrogenase (NADP+ dependent) 1-like (MTHFD1L), mRNA	NM_015440	Hs.591343	AK127089
FLJ10781	-5.2	2.1	hypothetical protein FLJ10781 (FLJ10781), mRNA	NM_018215	Hs.8395	BC032508
MREG	-5.2	2.2	melanoregulin (MREG), mRNA	NM_018000	Hs.281680	NM_018000
UGT8	-5.2	4.3	Human ceramide UDPgalactosyltransferase mRNA, complete cds.	U62899	Hs.144197	NM_003360
FGFR1	-5.2	10.9	fibroblast growth factor receptor 1 (fms-related tyrosine kinase 2, Pfeiffer syndrome) (FGFR1), transcript variant 5, mRNA	NM_023107	Hs.264887	NM_023110
ARF3	-5.2	2.5	ADP-ribosylation factor 3 (ARF3), mRNA	NM_001659	Hs.119177	NM_001659
PMAIP1	-5.2	10.2	phorbol-12-myristate-13-acetate-induced protein 1 (PMAIP1), mRNA	NM_021127	Hs.96	NM_021127
NBEAL2	-5.2	2.8	mRNA for FLJ00341 protein.	AK131104	Hs.437043	NM_015175
CSDA	-5.2	2.9	cold shock domain protein A (CSDA), mRNA	NM_003651	Hs.221889	AB209896
C17orf41	-5.2	2.1	chromosome 17 open reading frame 41 (C17orf41), mRNA	NM_024857	Hs.528902	AY557611
CENPH	-5.2	1.9	centromere protein H (CENPH), mRNA	NM_022909	Hs.631967	NM_022909
POLR3G	-5.2	18.2	polymerase (RNA) III (DNA directed) polypeptide G (32kD) (POLR3G), mRNA	NM_006467	Hs.282387	NM_006467
PPIL5	-5.2	1.8	peptidylprolyl isomerase (cyclophilin)-like 5 (PPIL5), transcript variant 3, mRNA	NM_203467	Hs.700904	BX648029
BRAF	-5.2	2.4	v-raf murine sarcoma viral oncogene homolog B1 (BRAF), mRNA	NM_004333	Hs.550061	M95712
CSDA	-5.2	3.0	cold shock domain protein A (CSDA), mRNA	NM_003651	Hs.221889	AB209896
VCL	-5.2	3.0	vinculin (VCL), transcript variant 1, mRNA	NM_014000	Hs.699180	NM_014000
CUTL2	-5.2	2.4	cut-like 2 (Drosophila) (CUTL2), mRNA	NM_015267	Hs.124953	BC151245
HOMER1	-5.2	1.6	homer homolog 1 (Drosophila) (HOMER1), mRNA	NM_004272	Hs.591761	NM_004272
GSG2	-5.2	3.4	germ cell associated 2 (haspin) (GSG2), mRNA	NM_031965	Hs.534059	AK056691
FAM29A	-5.1	1.9	family with sequence similarity 29, member A (FAM29A), mRNA	NM_017645	Hs.533468	NM_017645
LOC387924	-5.1	2.5	PREDICTED: hypothetical LOC387924 (LOC387924), mRNA	XR_019271	Hs.646629	XR_019271
TRAF2	-5.1	2.5	TNF receptor-associated factor 2 (TRAF2), mRNA	NM_021138	Hs.522506	BC064662
YWHAH	-5.1	2.2	tyrosine 3-monooxygenase/tryptophan 5-monooxygenase activation protein, eta polypeptide (YWHAH), mRNA	NM_003405	Hs.226755	CR622695
C16orf59	-5.1	1.8	chromosome 16 open reading frame 59 (C16orf59), mRNA	NM_025108	Hs.534491	CR612076
CHCHD7	-5.1	2.6	coiled-coil-helix-coiled-coil-helix domain containing 7 (CHCHD7), transcript variant 1, mRNA	NM_001011667	Hs.436913	AK098285
EIF5A	-5.1	4.1	eukaryotic translation initiation factor 5A (EIF5A), mRNA	NM_001970	Hs.534314	CR622789
HSPB9	-5.1	1.5	heat shock protein, alpha-crystallin-related, B9, mRNA (cDNA clone MGC:121026 IMAGE:7939836), complete cds.	BC093991	Hs.620611	AK093488
A_32_P46700	-5.1	2.7	A_32_P46700	A_32_P46700	Unknown	
CR626252	-5.1	8.5	full-length cDNA clone CS0DD001YO10 of Neuroblastoma Cot 50-normalized of (human).	CR626252	Hs.299329	AK126207
FKSG24	-5.1	2.0	hypothetical protein MGC12972 (FKSG24), mRNA	NM_032683	Hs.515254	BC028146
CCDC88	-5.1	5.7	cDNA FLJ37970 fis, clone CTONG2009955.	AK095289	Hs.98564	BC151218
CPXM2	-5.1	4.3	carboxypeptidase X (M14 family), member 2 (CPXM2), mRNA	NM_198148	Hs.656887	AY358565
KIAA0664	-5.1	2.7	KIAA0664 (KIAA0664), mRNA	NM_015229	Hs.22616	NM_015229
RECK	-5.1	2.4	cDNA clone MGC:71628 IMAGE:30336414, complete cds.	BC060806	Hs.388918	BX648668
TFAP2C	-5.1	2.4	transcription factor AP-2 gamma (activating enhancer binding protein 2 gamma) (TFAP2C), mRNA	NM_003222	Hs.473152	NM_003222
SOCS2	-5.1	3.4	suppressor of cytokine signaling 2 (SOCS2), mRNA	NM_003877	Hs.485572	BC070039
ZNF808	-5.1	2.4	zinc finger protein 808 (ZNF808), mRNA	NM_001039886	Hs.697780	BC033230
RASGEF1A	-5.1	3.7	RasGEF domain family, member 1A, mRNA (cDNA clone MGC:26821 IMAGE:4814750), complete cds.	BC022548	Hs.125293	AK127432
PPM1J	-5.1	4.9	protein phosphatase 1J (PP2C domain containing) (PPM1J), mRNA	NM_005167	Hs.655231	BC073828
TTC9	-5.1	2.8	Tetratricopeptide repeat protein 9 (TPR repeat protein 9) (Fragment).	ENST0000256	Unknown	
BC035392	-5.1	3.4	cDNA clone IMAGE:4822878.	BC035392	Hs.668794	BC035392

ZNF485	-5.1	1.9	zinc finger protein 485 (ZNF485), mRNA	NM_145312	Hs.147440	BC014161
ATP1B3	-5.1	1.7	ATPase, Na+/K+ transporting, beta 3 polypeptide (ATP1B3), mRNA	NM_001679	Hs.477789	AK094673
MAN2A1	-5.1	4.4	mannosidase, alpha, class 2A, member 1 (MAN2A1), mRNA	NM_002372	Hs.432822	CR936691
METAP1	-5.1	1.4	methionyl aminopeptidase 1 (METAP1), mRNA	NM_015143	Hs.480364	CR936632
LRRC8E	-5.1	3.5	leucine rich repeat containing 8 family, member E (LRRC8E), mRNA	NM_025061	Hs.501511	NM_025061
UNC13A	-5.1	14.5	Unc-13 homolog A (Munc13-1).	ENST00000252	Unknown	
FGF2	-5.1	5.4	fibroblast growth factor 2 (basic) (FGF2), mRNA	NM_002006	Hs.284244	NM_002006
TTF2	-5.1	2.2	transcription termination factor, RNA polymerase II (TTF2), mRNA	NM_003594	Hs.486818	AB209845
A_23_P20793	-5.1	2.1	A_23_P20793	A_23_P20793	Unknown	
C1orf67	-5.1	2.6	cDNA clone IMAGE:5270407.	BC042869	Hs.133977	BC042869
PMAIP1	-5.1	10.5	phorbol-12-myristate-13-acetate-induced protein 1 (PMAIP1), mRNA	NM_021127	Hs.96	NM_021127
FLJ25801	-5.1	16.2	hypothetical protein FLJ25801 (FLJ25801), mRNA	NM_173553	Hs.276429	NM_173553
IGF1R	-5.1	2.3	insulin-like growth factor 1 receptor (IGF1R), mRNA	NM_000875	Hs.643120	NM_000875
BRAF	-5.1	2.5	v-raf murine sarcoma viral oncogene homolog B1 (BRAF), mRNA	NM_004333	Hs.550061	M95712
APEH	-5.1	2.0	N-acylaminoacyl-peptide hydrolase (APEH), mRNA	NM_001640	Hs.517969	BC000362
EPHA1	-5.1	9.3	EPH receptor A1 (EPHA1), mRNA	NM_005232	Hs.89839	M18391
PHF17	-5.1	2.8	PHD finger protein 17 (PHF17), transcript variant S, mRNA	NM_024900	Hs.12420	NM_199320
LOC341346	-5.1	1.9	PREDICTED: hypothetical LOC341346 (LOC341346), mRNA	XM_292021	Hs.282121	XM_292021
SYAP1	-5.1	2.5	synapse associated protein 1, SAP47 homolog (Drosophila) (SYAP1), mRNA	NM_032796	Hs.659466	AK126173
LARP1	-5.1	1.8	La ribonucleoprotein domain family, member 1 (LARP1), transcript variant 2, mRNA	NM_033551	Hs.292078	NM_033551
PCTK3	-5.1	2.4	PCTAIRE protein kinase 3 (PCTK3), transcript variant 1, mRNA	NM_212503	Hs.700583	AK126879
THC2536711	-5.1	13.3	THC2536711	THC2536711	Unknown	
EIF4A1	-5.1	3.1	eukaryotic translation initiation factor 4A, isoform 1 (EIF4A1), mRNA	NM_001416	Hs.129673	BG033657
SUGT1	-5.1	1.5	SGT1, suppressor of G2 allele of SKP1 (S. cerevisiae) (SUGT1), mRNA	NM_006704	Hs.281902	CR616065
DPH3	-5.1	2.1	DPH3, KTI11 homolog (S. cerevisiae) (DPH3), transcript variant 1, mRNA	NM_206831	Hs.388087	AK092040
LHX6	-5.1	2.3	LIM homeobox 6 (LHX6), transcript variant 1, mRNA	NM_014368	Hs.103137	AK126982
ASRGL1	-5.1	2.7	asparaginase like 1 (ASRGL1), mRNA	NM_025080	Hs.535326	BX640832
KIAA1727	-5.1	3.9	KIAA1727 protein (KIAA1727), mRNA	NM_033393	Hs.132629	NM_033393
DLG7	-5.1	1.9	discs, large homolog 7 (Drosophila) (DLG7), mRNA	NM_014750	Hs.77695	NM_014750
TLCD1	-5.1	3.5	TLC domain containing 1 (TLCD1), mRNA	NM_138463	Hs.696686	BQ683306
C14orf172	-5.1	2.1	chromosome 14 open reading frame 172 (C14orf172), mRNA	NM_152307	Hs.525610	NM_152307
GUCA1A	-5.1	7.7	guanylate cyclase activator 1A (retina) (GUCA1A), mRNA	NM_000409	Hs.92858	BX647537
ENST00000335	-5.1	2.0	cDNA FLJ46467 fis, clone THYMU3022668.	ENST00000335	Unknown	
STEAP3	-5.1	3.9	STEAP family member 3 (STEAP3), transcript variant 1, mRNA	NM_182915	Hs.647822	AL833624
Magmas	-5.1	1.7	mitochondria-associated protein involved in granulocyte-macrophage colony-stimulating factor signal transduction (Magmas), nuclear gene encoding mitochondrial protein, mRNA	NM_016069	Hs.644614	BQ218379
ST6GAL2	-5.1	2.7	ST6 beta-galactosamide alpha-2,6-sialyltranferase 2 (ST6GAL2), mRNA	NM_032528	Hs.98265	AB058780
SPAG5	-5.1	1.6	sperm associated antigen 5 (SPAG5), mRNA	NM_006461	Hs.514033	AF345347
THC2635964	-5.1	5.1	Q4WTS6_ASPFU (Q4WTS6) Peptidyl-tRNA hydrolase, partial (6%)	THC2635964	Unknown	
BM979049	-5.1	2.1	BM979049 UI-CF-DU1-adl-i-11-0-UI.s1 UI-CF-DU1 cDNA clone UI-CF-DU1-adl-i-11-0-UI 3', mRNA sequence	BM979049	Hs.512908	AL833077
WDFY2	-5.1	2.3	WD repeat and FYVE domain containing 2 (WDFY2), mRNA	NM_052950	Hs.208550	NM_052950
EXDL2	-5.1	1.9	exonuclease 3'-5' domain-like 2 (EXDL2), mRNA	NM_018199	Hs.649365	AK056025
BC062780	-5.1	2.0	cDNA clone IMAGE:4700531, partial cds.	BC062780	Hs.287168	BC035072
MARVELD3	-5.1	6.2	MARVEL domain containing 3 (MARVELD3), transcript variant 2, mRNA	NM_052858	Hs.513706	NM_001017967
C4orf23	-5.1	3.3	cDNA FLJ12891 fis, clone NT2RP2004142.	AK022953	Hs.566191	AK022953
C9orf61	-5.1	2.4	chromosome 9 open reading frame 61 (C9orf61), mRNA	NM_004816	Hs.118003	AK126127
LOC553158	-5.0	3.8	PRR5-ARHGAP8 fusion (LOC553158), mRNA	NM_181334	Hs.102336	BC059382
CSF2RA	-5.0	1.9	colony stimulating factor 2 receptor, alpha, low-affinity (granulocyte-macrophage) (CSF2RA), transcript variant 4, mRNA	NM_172247	Hs.520937	BC071835
MGC3196	-5.0	1.7	MGC3196 protein (Fragment).	ENST00000307	Unknown	
SLCO1A2	-5.0	3.5	solute carrier organic anion transporter family, member 1A2 (SLCO1A2), transcript variant 1, mRNA	NM_134431	Hs.46440	NM_134431
SYT12	-5.0	8.7	synaptotagmin XII (SYT12), mRNA	NM_177963	Hs.287636	AK024381
C11orf48	-5.0	2.0	chromosome 11 open reading frame 48 (C11orf48), mRNA	NM_024099	Hs.9061	CR612336
ENST00000264	-5.0	2.2	Extracellular matrix protein FRAS1 precursor.	ENST00000264	Unknown	
CD300A	-5.0	1.7	CD300a molecule (CD300A), mRNA	NM_007261	Hs.9688	NM_007261
VILL	-5.0	2.5	villin-like (VILL), mRNA	NM_015873	Hs.103665	BC004300
C19orf24	-5.0	1.8	chromosome 19 open reading frame 24 (C19orf24), mRNA	NM_017914	Hs.591383	BC012080

KIAA0133	-5.0	1.8	KIAA0133 (KIAA0133), mRNA	NM_014777	Hs.533628	NM_014777
ANKRD28	-5.0	2.0	ankyrin repeat domain 28 (ANKRD28), mRNA	NM_015199	Hs.335239	NM_015199
ZNF721	-5.0	2.2	zinc finger protein 721 (ZNF721), mRNA	NM_133474	Hs.428360	NM_133474
NLN	-5.0	3.7	neurolysin (metallopeptidase M3 family) (NLN), mRNA	NM_020726	Hs.247460	AB033052
CST11	-5.0	2.4	cystatin 11 (CST11), transcript variant 1, mRNA	NM_130794	Hs.128100	AF335480
LIG3	-5.0	3.1	ligase III, DNA, ATP-dependent (LIG3), nuclear gene encoding mitochondrial protein, transcript variant beta, mRNA	NM_002311	Hs.100299	NM_013975
ASB5	-5.0	14.7	ankyrin repeat and SOCS box-containing 5 (ASB5), mRNA	NM_080874	Hs.591712	BX647857
UGCGL1	-5.0	2.7	UDP-glucose ceramide glucosyltransferase-like 1 (UGCGL1), transcript variant 2,	NM_001025777	Hs.34180	BC041098
PMM2	-5.0	2.1	phosphomannomutase 2 (PMM2), mRNA	NM_000303	Hs.699546	AB209659
STAT3	-5.0	2.3	signal transducer and activator of transcription 3 (acute-phase response factor) (STAT3), transcript variant 3, mRNA	NM_213662	Hs.463059	NM_139276
RRM2	-5.0	2.5	ribonucleotide reductase M2 polypeptide (RRM2), mRNA	NM_001034	Hs.226390	AK123010
BRI3BP	-5.0	2.5	BRI3 binding protein (BRI3BP), mRNA	NM_080626	Hs.632740	AK025766
DKFZP564J102	-5.0	1.7	DKFZP564J102 protein (DKFZP564J102), transcript variant 1, mRNA	NM_015398	Hs.357025	NM_015398
CTAGE4	-5.0	3.0	CTAGE-4 protein mRNA, complete cds.	AF338232	Hs.591829	AF338232
PRKCA	-5.0	4.9	protein kinase C, alpha (PRKCA), mRNA	NM_002737	Hs.531704	NM_002737
KPNA2	-5.0	1.8	karyopherin alpha 2 (RAG cohort 1, importin alpha 1) (KPNA2), mRNA	NM_002266	Hs.594238	BC067848
FOXO1A	-5.0	4.1	forkhead box O1A (rhabdomyosarcoma) (FOXO1A), mRNA	NM_002015	Hs.370666	NM_002015
ZNF8	-5.0	1.5	zinc finger protein 8 (ZNF8), mRNA	NM_021089	Hs.590941	AK225825
ARRB2	-5.0	2.0	arrestin, beta 2 (ARRB2), transcript variant 1, mRNA	NM_004313	Hs.435811	NM_004313
KCND2	-5.0	5.2	potassium voltage-gated channel, Shal-related subfamily, member 2 (KCND2), mRNA	NM_012281	Hs.654739	AB028967
THC2668034	-5.0	10.3	ALU7_HUMAN (P39194) Alu subfamily SQ sequence contamination warning entry, partial (4%)	THC2668034	Unknown	
SLC35F2	-5.0	2.2	solute carrier family 35, member F2 (SLC35F2), mRNA	NM_017515	Hs.524014	AK128062
C11orf75	-5.0	1.9	chromosome 11 open reading frame 75 (C11orf75), mRNA	NM_020179	Hs.438064	BM911813
USP45	-5.0	1.9	ubiquitin specific peptidase 45, mRNA (cDNA clone IMAGE:4047601), complete cds.	BC005991	Hs.143410	NM_001080481
A_24_P638453	-5.0	2.8	A_24_P638453	A_24_P638453	Unknown	
PERQ1	-5.0	2.0	PERQ amino acid rich, with GYF domain 1 (PERQ1), mRNA	NM_022574	Hs.696531	NM_022574
EIF4A1	-5.0	3.1	eukaryotic translation initiation factor 4A, isoform 1 (EIF4A1), mRNA	NM_001416	Hs.129673	BG033657
A_32_P162443	-5.0	7.5	A_32_P162443	A_32_P162443	Unknown	
TTC27	-5.0	1.5	tetratricopeptide repeat domain 27 (TTC27), mRNA	NM_017735	Hs.468125	BC063791
ROD1	-5.0	2.5	ROD1 regulator of differentiation 1 (S. pombe) (ROD1), mRNA	NM_005156	Hs.269988	NM_005156
RHBDF2	-5.0	3.5	rhomboid 5 homolog 2 (Drosophila) (RHBDF2), transcript variant 1, mRNA	NM_024599	Hs.464157	NM_024599
METTL8	-5.0	2.6	methyltransferase like 8 (METTL8), mRNA	NM_024770	Hs.135146	AK024046
DARS2	-5.0	2.0	aspartyl-tRNA synthetase 2 (mitochondrial) (DARS2), mRNA	NM_018122	Hs.647707	NM_018122
THC2713078	-5.0	2.5	THC2713078	THC2713078	Unknown	
SEMA4D	-5.0	2.8	sema domain, immunoglobulin domain (Ig), transmembrane domain (TM) and short cytoplasmic domain, (semaphorin) 4D (SEMA4D), mRNA	NM_006378	Hs.655281	AB210030
ZNF786	-5.0	1.5	zinc finger protein 786 (ZNF786), mRNA	NM_152411	Hs.632029	AL834510
MYOHD1	-5.0	2.1	myosin head domain containing 1 (MYOHD1), transcript variant 3, mRNA	NM_001033580	Hs.302051	AB209035
TFE3	-5.0	2.0	transcription factor binding to IGHM enhancer 3 (TFE3), mRNA	NM_006521	Hs.274184	NM_006521
A_23_P64962	-5.0	4.8	A_23_P64962	A_23_P64962	Unknown	
C5orf16	-5.0	1.9	chromosome 5 open reading frame 16 (C5orf16), mRNA	NM_173828	Hs.120094	BC027919
INPP4A	-5.0	2.7	inositol polyphosphate-4-phosphatase, type I, 107kDa (INPP4A), transcript variant a, mRNA	NM_004027	Hs.580527	BC028361
ASS1	-5.0	3.7	argininosuccinate synthetase 1 (ASS1), transcript variant 1, mRNA	NM_000050	Hs.160786	AK027126
BC010126	-5.0	7.5	cDNA clone IMAGE:3948082, partial cds.	BC010126	Hs.654410	NM_000620
UNQ2446	-5.0	5.2	MRCC2446 (UNQ2446), mRNA	NM_198443	Hs.435464	CD049026
LOC442229	-5.0	2.2	cDNA clone MGC:33544 IMAGE:4821649, complete cds.	BC024198	Hs.390414	BC024198
COMTD1	-5.0	2.5	catechol-O-methyltransferase domain containing 1 (COMTD1), mRNA	NM_144589	Hs.355333	CR625911
RGMB	-5.0	2.4	RGM domain family, member B (RGMB), transcript variant 2, mRNA	NM_173670	Hs.526902	NM_001012761
AYTL2	-5.0	3.2	acyltransferase like 2 (AYTL2), mRNA	NM_024830	Hs.368853	AK090444
A_32_P159289	-5.0	2.4	A_32_P159289	A_32_P159289	Unknown	
TLK1	-5.0	2.1	tousled-like kinase 1 (TLK1), mRNA	NM_012290	Hs.655640	D50927
ENST00000370	-5.0	2.1	CSL-type zinc finger-containing protein 1.	ENST00000370	Unknown	
PLEKHF1	-5.0	3.1	pleckstrin homology domain containing, family F (with FYVE domain) member 1 (PLEKHF1), mRNA	NM_024310	Hs.466383	AY037145
PRKCD	-5.0	2.3	protein kinase C, delta (PRKCD), transcript variant 1, mRNA	NM_006254	Hs.155342	NM_006254
LOC400684	-5.0	2.3	hypothetical gene supported by BC000922, mRNA (cDNA clone IMAGE:3447073), partial cds.	BC000922	Hs.438766	BC000922
MST1R	-5.0	2.2	macrophage stimulating 1 receptor (c-met-related tyrosine kinase) (MST1R), mRNA	NM_002447	Hs.517973	NM_002447

IGF1R	-5.0	2.2	insulin-like growth factor 1 receptor (IGF1R), mRNA	NM_000875	Hs.643120	NM_000875
A_32_P130217	-5.0	4.6	A_32_P130217	A_32_P130217	Unknown	
C14orf169	-5.0	2.0	chromosome 14 open reading frame 169 (C14orf169), mRNA	NM_024644	Hs.509916	AK025455
NLRX1	-5.0	1.6	NLR family member X1 (NLRX1), transcript variant 2, mRNA	NM_170722	Hs.524082	BC110890
TMC5	-5.0	14.2	transmembrane channel-like 5 (TMC5), mRNA	NM_024780	Hs.115838	AY358155
FLJ38020	-5.0	2.3	similar to absent in melanoma 1 (FLJ38020), mRNA	NM_001039775	Hs.128738	NM_001039775
CAMK2G	-5.0	2.9	calcium/calmodulin-dependent protein kinase (CaM kinase) II gamma (CAMK2G), transcript variant 1, mRNA	NM_172171	Hs.699281	AB209474
RBPMS	-5.0	4.0	RNA binding protein with multiple splicing (RBPMS), transcript variant 3, mRNA	NM_001008712	Hs.334587	AK124859
LRFN4	-5.0	1.8	leucine rich repeat and fibronectin type III domain containing 4 (LRFN4), mRNA	NM_024036	Hs.696061	NM_024036
THC2680948	-5.0	12.0	THC2680948	THC2680948	Unknown	
BE181768	-5.0	3.2	BE181768 QV1-HT0639-150500-198-g05 HT0639 cDNA, mRNA sequence	BE181768	Hs.667992	BQ218507
AOX1	-5.0	2.9	aldehyde oxidase 1 (AOX1), mRNA	NM_001159	Hs.406238	L11005
PUS1	-5.0	5.4	pseudouridylate synthase 1 (PUS1), transcript variant 1, mRNA	NM_025215	Hs.592004	BC035964
FLJ40125	-5.0	8.4	hypothetical protein FLJ40125, mRNA (cDNA clone MGC:40208 IMAGE:5240955), complete cds.	BC028228	Unknown	
CCNE1	-5.0	1.8	cyclin E1 (CCNE1), transcript variant 1, mRNA	NM_001238	Hs.244723	BC035498
PTRH2	-5.0	1.8	peptidyl-tRNA hydrolase 2 (PTRH2), nuclear gene encoding mitochondrial protein, transcript variant 1, mRNA	NM_001015509	Unknown	
HRASLS2	-4.9	6.3	HRAS-like suppressor 2 (HRASLS2), mRNA	NM_017878	Hs.272805	AK025029
NOLC1	-4.9	1.8	nucleolar and coiled-body phosphoprotein 1, mRNA (cDNA clone MGC:5049 IMAGE:2900024), complete cds.	BC006769	Hs.523238	NM_004741
DIDO1	-4.9	2.4	death inducer-obliterator 1 (DIDO1), transcript variant 1, mRNA	NM_022105	Hs.517172	NM_033081
FLJ37953	-4.9	1.6	hypothetical protein FLJ37953 (FLJ37953), mRNA	NM_001039693	Hs.204619	NM_001039693
SAV1	-4.9	2.3	salvador homolog 1 (Drosophila) (SAV1), mRNA	NM_021818	Hs.642842	AL833378
EIF4E	-4.9	1.9	eukaryotic translation initiation factor 4E (EIF4E), mRNA	NM_001968	Hs.249718	NM_001968
PRDM5	-4.9	2.6	PR domain containing 5 (PRDM5), mRNA	NM_018699	Hs.660435	NM_018699
C16orf28	-4.9	2.1	chromosome 16 open reading frame 28 (C16orf28), mRNA	NM_023076	Hs.700916	NM_023076
HSPD1	-4.9	3.1	heat shock 60kDa protein 1 (chaperonin) (HSPD1), nuclear gene encoding mitochondrial protein, transcript variant 1, mRNA	NM_002156	Hs.632539	NM_002156
PIP5KL1	-4.9	2.3	phosphatidylinositol-4-phosphate 5-kinase-like 1 (PIP5KL1), mRNA	NM_173492	Hs.445486	BQ050076
LOC647055	-4.9	10.3	PREDICTED: similar to DNA-directed RNA polymerase III 32 kDa polypeptide (RNA polymerase III C32 subunit) (LOC647055), mRNA	XR_017398	Hs.646448	XR_017398
GJB7	-4.9	9.1	gap junction protein, beta 7 (GJB7), mRNA	NM_198568	Hs.146727	BC057813
CN277024	-4.9	3.1	17000600173672 GRN_PREHEP cDNA 5', mRNA sequence	CN277024	Hs.321664	CN277024
TMPRSS2	-4.9	2.7	transmembrane protease, serine 2 (TMPRSS2), mRNA	NM_005656	Hs.439309	NM_005656
C15orf42	-4.9	2.8	chromosome 15 open reading frame 42 (C15orf42), mRNA	NM_152259	Hs.441708	NM_152259
RYR1	-4.9	3.5	ryanodine receptor 1 (skeletal) (RYR1), transcript variant 1, mRNA	NM_000540	Hs.466664	NM_000540
NCAPD3	-4.9	2.0	non-SMC condensin II complex, subunit D3 (NCAPD3), mRNA	NM_015261	Hs.438550	NM_015261
LOC285074	-4.9	1.6	hypothetical protein LOC285074 (LOC285074), mRNA	NM_001012626	Unknown	
OIP5	-4.9	2.0	Opa interacting protein 5 (OIP5), mRNA	NM_007280	Hs.661645	BQ218042
NFKBIL2	-4.9	2.4	nuclear factor of kappa light polypeptide gene enhancer in B-cells inhibitor-like 2 (NFKBIL2), mRNA	NM_013432	Hs.675285	AB209126
PTGES3	-4.9	2.4	prostaglandin E synthase 3 (cytosolic) (PTGES3), mRNA	NM_006601	Hs.50425	AK098214
KCNS3	-4.9	1.8	potassium voltage-gated channel, delayed-rectifier, subfamily S, member 3 (KCNS3), mRNA	NM_002252	Hs.414489	AK225833
MST101	-4.9	2.2	cDNA FLJ31359 fis, clone MESAN2000501, weakly similar to DNA cytosine methyltransferase 3 alpha (DNMT3A) mRNA.	AK055921	Hs.699426	AK055921
COBL	-4.9	10.9	cordon-bleu homolog (mouse) (COBL), mRNA	NM_015198	Hs.99141	BC150263
LOC646561	-4.9	3.0	PREDICTED: similar to WW45 protein (LOC646561), mRNA	XM_929496	Unknown	
OR7E13P	-4.9	1.6	olfactory-like receptor PJCG2 (PJCG2) mRNA, partial cds.	AF238487	Hs.684491	AF238487
C6orf167	-4.9	3.7	chromosome 6 open reading frame 167 (C6orf167), mRNA	NM_198468	Hs.444292	NM_198468
EP400NL	-4.9	1.6	EP400 N-terminal like (EP400NL), mRNA	NM_182613	Unknown	
STAT3	-4.9	2.4	signal transducer and activator of transcription 3 (acute-phase response factor) (STAT3), transcript variant 3, mRNA	NM_213662	Hs.463059	NM_139276
LOC144097	-4.9	1.9	hypothetical protein BC007540 (LOC144097), mRNA	NM_138471	Hs.502793	AK091493
FLJ13769	-4.9	3.0	cDNA FLJ13769 fis, clone PLACE4000222.	AK023831	Hs.677248	AK023831
GTPBP3	-4.9	2.7	GTP binding protein 3 (mitochondrial) (GTPBP3), transcript variant IV, mRNA	NM_133644	Hs.334885	AK091035
AK092090	-4.9	1.8	cDNA FLJ34771 fis, clone NT2NE2003150.	AK092090	Hs.433010	AK092090
BC030102	-4.9	2.3	cDNA clone IMAGE:4796690.	BC030102	Hs.503348	BC008757
MPHOSPH9	-4.9	1.9	M-phase phosphoprotein 9 (MPHOSPH9), mRNA	NM_022782	Hs.577404	AK096541
GNB1L	-4.9	1.9	guanine nucleotide binding protein (G protein), beta polypeptide 1-like (GNB1L), mRNA	NM_053004	Hs.105642	NM_024627
ME2	-4.9	1.9	malic enzyme 2, NAD(+)-dependent, mitochondrial (ME2), nuclear gene encoding mitochondrial protein, mRNA	NM_002396	Hs.699163	NM_002396

ACLY	-4.9	2.1	Human ATP:citrate lyase mRNA, complete cds.	U18197	Hs.387567	NM_001096
KIAA0368	-4.9	1.5	KIAA0368, mRNA (cDNA clone IMAGE:4335177), complete cds.	BC021127	Hs.368255	NM_001080398
ENST00000370:	-4.9	4.4	Gamma-aminobutyric-acid receptor subunit theta precursor (GABA(A) receptor subunit theta).	ENST00000370:	Unknown	
SHROOM1	-4.9	1.8	shroom family member 1 (SHROOM1), mRNA	NM_133456	Hs.519574	AB075840
CXCL3	-4.9	3.9	chemokine (C-X-C motif) ligand 3 (CXCL3), mRNA	NM_002090	Hs.89690	NM_002090
CN431194	-4.9	4.6	CN431194 328775669 GRN_ES cDNA 5', mRNA sequence	CN431194	Hs.591205	NM_007129
STEAP2	-4.9	2.6	six transmembrane epithelial antigen of the prostate 2 (STEAP2), transcript variant 1, mRNA	NM_152999	Hs.489051	NM_152999
CGN	-4.9	2.6	cingulin (CGN), mRNA	NM_020770	Hs.591464	BC146657
CD72	-4.9	2.8	CD72 molecule (CD72), mRNA	NM_001782	Hs.116481	AB209587
LOH12CR1	-4.9	1.8	loss of heterozygosity, 12, chromosomal region 1 (LOH12CR1), mRNA	NM_058169	Hs.504805	NM_058169
GTPBP1	-4.9	2.4	GTP binding protein 1 (GTPBP1), mRNA	NM_004286	Hs.276925	NM_004286
FAM109B	-4.9	3.0	family with sequence similarity 109, member B (FAM109B), mRNA	NM_001002034	Hs.368312	BX648402
PCCB	-4.9	2.4	propionyl Coenzyme A carboxylase, beta polypeptide (PCCB), mRNA	NM_000532	Hs.63788	AB209009
BC013295	-4.9	2.2	cDNA clone IMAGE:2960340.	BC013295	Unknown	
THC2762149	-4.9	1.7	BC037545 poly(ADP-ribosyl)transferase {Homo sapiens} (exp=-1; wgp=0; cg=0), partial (24%)	THC2762149	Unknown	
PCDHGA3	-4.9	4.0	protocadherin gamma subfamily A, 3 (PCDHGA3), transcript variant 2, mRNA	NM_032011	Hs.368160	AB002325
LOC220147	-4.9	1.8	PREDICTED: similar to C-terminal binding protein 2 isoform 2 (LOC220147), mRNA	XR_018866	Hs.646464	XR_018866
AF023203	-4.9	2.5	homeobox protein Og12 (OGL12) mRNA, partial cds.	AF023203	Unknown	
BC015434	-4.9	3.2	Homo sapiens, clone IMAGE:4414697, mRNA.	BC015434	Hs.592734	BC027349
PUS1	-4.9	3.6	pseudouridylate synthase 1 (PUS1), transcript variant 1, mRNA	NM_025215	Hs.592004	BC035964
NOC3L	-4.9	1.9	nucleolar complex associated 3 homolog (S. cerevisiae) (NOC3L), mRNA	NM_022451	Hs.74899	BC049850
SLC6A6	-4.9	5.0	solute carrier family 6 (neurotransmitter transporter, taurine), member 6 (SLC6A6), mRNA	NM_003043	Hs.529488	NM_003043
HTR3A	-4.9	3.8	5-hydroxytryptamine (serotonin) receptor 3A (HTR3A), transcript variant 1, mRNA	NM_213621	Hs.413899	NM_213621
WBP2	-4.9	3.2	WW domain binding protein 2 (WBP2), mRNA	NM_012478	Hs.514489	NM_012478
PRKCD	-4.9	2.2	protein kinase C, delta (PRKCD), transcript variant 1, mRNA	NM_006254	Hs.155342	NM_006254
FUT2	-4.9	2.5	fucosyltransferase 2 (secretor status included) (FUT2), mRNA	NM_000511	Hs.579928	NM_000511
GCDH	-4.9	2.9	glutaryl-Coenzyme A dehydrogenase (GCDH), nuclear gene encoding mitochondrial protein, transcript variant 2, mRNA	NM_013976	Hs.532699	BC002579
MAPK1	-4.9	2.5	mitogen-activated protein kinase 1 (MAPK1), transcript variant 2, mRNA	NM_138957	Hs.431850	AL157438
STAT3	-4.9	2.3	signal transducer and activator of transcription 3 (acute-phase response factor) (STAT3), transcript variant 3, mRNA	NM_213662	Hs.463059	NM_139276
LGP2	-4.9	8.4	likely ortholog of mouse D11lgp2 (LGP2), mRNA	NM_024119	Hs.55918	NM_024119
HELLS	-4.9	2.1	helicase, lymphoid-specific (HELLS), mRNA	NM_018063	Hs.655830	NM_018063
FLJ37078	-4.9	3.3	hypothetical protein FLJ37078 (FLJ37078), mRNA	NM_001039212	Hs.694857	NM_001039212
DGKZ	-4.8	3.0	diacylglycerol kinase, zeta 104kDa (DGKZ), transcript variant 2, mRNA	NM_003646	Hs.502461	AB209635
GSG2	-4.8	2.0	cDNA FLJ32129 fis, clone PEBLM2000213, weakly similar to Mus musculus genes for integrin aM290, hapsin.	AK056691	Hs.534059	AK056691
BX095032	-4.8	2.2	BX095032 Soares breast 2NbHBst cDNA clone IMAGp998E17244, mRNA sequence	BX095032	Hs.562147	BX095032
SUPT16H	-4.8	1.6	suppressor of Ty 16 homolog (S. cerevisiae) (SUPT16H), mRNA	NM_007192	Hs.213724	NM_007192
C16orf69	-4.8	2.2	chromosome 16 open reading frame 69 (C16orf69), mRNA	NM_153261	Hs.59134	AK095521
FAM120C	-4.8	2.0	family with sequence similarity 120C, mRNA (cDNA clone IMAGE:3921622), complete cds.	BC016138	Hs.675423	AY150025
PRKCD	-4.8	2.3	protein kinase C, delta (PRKCD), transcript variant 1, mRNA	NM_006254	Hs.155342	NM_006254
MAN2A1	-4.8	4.4	mannosidase, alpha, class 2A, member 1 (MAN2A1), mRNA	NM_002372	Hs.432822	CR936691
ENST00000330!	-4.8	3.9	HSPC088 mRNA, partial cds.	ENST00000330!	Unknown	
PEX5L	-4.8	1.7	peroxisomal biogenesis factor 5-like (PEX5L), mRNA	NM_016559	Hs.478393	AB032593
LOC85391	-4.8	5.2	RNA, small nucleolar (LOC85391) on chromosome 11	NR_003125	Hs.701607	DB363693
COMMD2	-4.8	1.8	COMM domain containing 2 (COMMD2), mRNA	NM_016094	Hs.591315	NM_016094
CCDC88	-4.8	3.2	coiled-coil domain containing 88 (CCDC88), mRNA	NM_032251	Hs.98564	BC151218
CHES1	-4.8	2.4	checkpoint suppressor 1 (CHES1), mRNA	NM_005197	Hs.434286	NM_005197
MAFG	-4.8	2.1	v-maf musculoaponeurotic fibrosarcoma oncogene homolog G (avian) (MAFG), transcript variant 1, mRNA	NM_002359	Hs.252229	NM_002359
ENST00000329:	-4.8	3.2	PREDICTED: similar to high-mobility group box 3 (LOC729595), mRNA	ENST00000329:	Unknown	
MOBKL2B	-4.8	2.1	MOB1, Mps One Binder kinase activator-like 2B (yeast) (MOBKL2B), mRNA	NM_024761	Hs.699322	NM_024761
CCDC88	-4.8	2.7	coiled-coil domain containing 88 (CCDC88), mRNA	NM_032251	Hs.98564	BC151218
NCAPG2	-4.8	2.3	non-SMC condensin II complex, subunit G2 (NCAPG2), mRNA	NM_017760	Hs.18616	BX537845
CDV3	-4.8	2.0	CDV3 homolog (mouse) (CDV3), mRNA	NM_017548	Hs.699298	AK096865
BQ017638	-4.8	22.0	BQ017638 UI-H-DI0-auv-p-03-0-UI.s1 NCI_CGAP_DI0 cDNA clone IMAGE:5875058 3', mRNA sequence	BQ017638	Hs.17631	NM_003468
CXorf15	-4.8	5.7	chromosome X open reading frame 15 (CXorf15), mRNA	NM_018360	Hs.555961	AY739713

FLJ35767	-4.8	3.6	FLJ35767 protein (FLJ35767), mRNA	NM_207459	Hs.231897	BC050391
RANBP5	-4.8	1.9	RAN binding protein 5 (RANBP5), mRNA	NM_002271	Hs.699240	NM_002271
KLHL23	-4.8	1.8	kelch-like 23 (Drosophila) (KLHL23), mRNA	NM_144711	Hs.655150	BC010437
IDE	-4.8	2.5	insulin-degrading enzyme (IDE), mRNA	NM_004969	Hs.500546	BX648462
PNO1	-4.8	2.6	partner of NOB1 homolog (S. cerevisiae) (PNO1), mRNA	NM_020143	Hs.262858	NM_020143
ANKRD9	-4.8	2.0	ankyrin repeat domain 9 (ANKRD9), mRNA	NM_152326	Hs.432945	BU849491
JAKMIP1	-4.8	7.1	janus kinase and microtubule interacting protein 1 (JAKMIP1), mRNA	NM_144720	Hs.479066	NM_001099433
PRKD3	-4.8	1.9	protein kinase D3 (PRKD3), mRNA	NM_005813	Hs.696257	NM_005813
STAG3	-4.8	3.4	stromal antigen 3 (STAG3), mRNA	NM_012447	Hs.592283	AJ007798
HSPC111	-4.8	2.4	hypothetical protein HSPC111 (HSPC111), mRNA	NM_016391	Hs.696283	AF151875
MTHFS	-4.8	1.7	5,10-methenyltetrahydrofolate synthetase (5-formyltetrahydrofolate cyclo-ligase) (MTHFS), mRNA	NM_006441	Hs.459049	AK054972
MRS2L	-4.8	1.8	MRS2-like, magnesium homeostasis factor (S. cerevisiae) (MRS2L), mRNA	NM_020662	Hs.533291	NM_020662
KHSRP	-4.8	3.0	KH-type splicing regulatory protein (FUSE binding protein 2) (KHSRP), mRNA	NM_003685	Hs.699378	NM_003685
WNK2	-4.8	2.8	WNK lysine deficient protein kinase 2 (WNK2), mRNA	NM_006648	Hs.654856	AB051547
SPINT1	-4.8	3.8	serine peptidase inhibitor, Kunitz type 1 (SPINT1), transcript variant 1, mRNA	NM_181642	Hs.233950	BC018702
RET	-4.8	5.4	ret proto-oncogene (multiple endocrine neoplasia and medullary thyroid carcinoma 1, Hirschsprung disease) (RET), transcript variant 4, mRNA	NM_020630	Hs.350321	NM_020975
GPR62	-4.8	1.7	G protein-coupled receptor 62 (GPR62), mRNA	NM_080865	Hs.232213	NM_080865
TRMT1	-4.8	1.9	TRM1 tRNA methyltransferase 1 homolog (S. cerevisiae) (TRMT1), mRNA	NM_017722	Hs.515169	AL390133
THC2755690	-4.8	4.9	THC2755690	THC2755690	Unknown	
THC2744021	-4.8	2.3	THC2744021	THC2744021	Unknown	
AK124080	-4.8	2.7	cDNA FLJ42086 fis, clone TESOP1000127.	AK124080	Hs.446041	AK124080
CDR2L	-4.8	2.2	cerebellar degeneration-related protein 2-like (CDR2L), mRNA	NM_014603	Hs.78358	NM_014603
C9orf140	-4.8	3.8	chromosome 9 open reading frame 140 (C9orf140), mRNA	NM_178448	Hs.19322	DQ150361
SEC22C	-4.8	1.6	SEC22 vesicle trafficking protein homolog C (S. cerevisiae) (SEC22C), transcript variant 1, mRNA	NM_032970	Hs.445892	CR749670
BUB3	-4.8	1.5	BUB3 budding uninhibited by benzimidazoles 3 homolog (yeast) (BUB3), transcript variant 2, mRNA	NM_001007793	Hs.418533	AK226060
THC2642537	-4.8	4.4	Q2Q5T5_MOUSE (Q2Q5T5) Embryonic stem cell-and germ cell-specific protein ESGP, complete	THC2642537	Unknown	
PAQR9	-4.8	1.5	progestin and adipoQ receptor family member IX (PAQR9), mRNA	NM_198504	Hs.656111	NM_198504
DNA2L	-4.8	1.9	DNA2 DNA replication helicase 2-like (yeast), mRNA (cDNA clone MGC:133297 IMAGE:40038149), complete cds.	BC111740	Hs.532446	NM_001080449
STYX	-4.8	2.0	serine/threonine/tyrosine interacting protein (STYX), mRNA	NM_145251	Hs.364980	BX647636
EPS8L1	-4.8	2.3	EPS8-like 1 (EPS8L1), transcript variant 1, mRNA	NM_133180	Hs.438862	NM_133180
PPRC1	-4.8	2.0	peroxisome proliferator-activated receptor gamma, coactivator-related 1 (PPRC1), mRNA	NM_015062	Hs.533551	AF325193
THC2543120	-4.8	1.5	THC2543120	THC2543120	Unknown	
SYT14	-4.8	2.5	synaptotagmin XIV (SYT14), mRNA	NM_153262	Hs.658866	AJ617624
BM479752	-4.8	1.9	AGENCOURT_6465050 NIH_MGC_92 cDNA clone IMAGE:5577282 5', mRNA sequence	BM479752	Hs.642655	BM479752
IL12RB2	-4.8	4.6	interleukin 12 receptor, beta 2 (IL12RB2), mRNA	NM_001559	Hs.479347	NM_001559
RUVBL1	-4.8	2.2	RuvB-like 1 (E. coli) (RUVBL1), mRNA	NM_003707	Hs.272822	BX647488
SYT3	-4.8	3.1	synaptotagmin III (SYT3), mRNA	NM_032298	Hs.515554	AL136594
EIF5A	-4.8	3.7	eukaryotic translation initiation factor 5A (EIF5A), mRNA	NM_001970	Hs.534314	CR622789
FLJ37953	-4.8	2.2	hypothetical protein FLJ37953 (FLJ37953), mRNA	NM_001039693	Hs.204619	NM_001039693
ITSN1	-4.7	2.4	intersectin 1 (SH3 domain protein) (ITSN1), transcript variant 2, mRNA	NM_001001132	Hs.160324	AF064244
KLK1	-4.7	3.2	kallikrein 1 (KLK1), mRNA	NM_002257	Hs.123107	BM924634
GNAL	-4.7	4.3	guanine nucleotide binding protein (G protein), alpha activating activity polypeptide, olfactory type (GNAL), transcript variant 2, mRNA	NM_002071	Hs.136295	AK090868
ADAMTS2	-4.7	1.9	ADAM metallopeptidase with thrombospondin type 1 motif, 2 (ADAMTS2), transcript variant 2, mRNA	NM_021599	Hs.591725	NM_014244
THC2578493	-4.7	1.8	Q74CT8_GEOSL (Q74CT8) Dethiobiotin synthase , partial (5%)	THC2578493	Unknown	
C19orf52	-4.7	1.6	chromosome 19 open reading frame 52 (C19orf52), mRNA	NM_138358	Hs.662044	BM803846
PTPN6	-4.7	2.9	protein tyrosine phosphatase, non-receptor type 6 (PTPN6), transcript variant 1, mRNA	NM_002831	Hs.63489	NM_002831
NOC3L	-4.7	1.9	nucleolar complex associated 3 homolog (S. cerevisiae) (NOC3L), mRNA	NM_022451	Hs.74899	BC049850
ND6	-4.7	2.7	NADH-ubiquinone oxidoreductase chain 6 (EC 1.6.5.3) (NADH dehydrogenase subunit 6).	ENST0000036[illegible]	Unknown	
A_24_P840868	-4.7	2.3	A_24_P840868	A_24_P840868	Unknown	
POLR1D	-4.7	1.9	polymerase (RNA) I polypeptide D, 16kDa (POLR1D), transcript variant 1, mRNA	NM_015972	Hs.507584	AK097973
STAT1	-4.7	1.7	signal transducer and activator of transcription 1, 91kDa (STAT1), transcript variant beta, mRNA	NM_139266	Hs.699271	NM_007315
THC2697412	-4.7	2.1	Q6YAI6_9HIV1 (Q6YAI6) Gag-pol fusion polyprotein (Fragment), partial (5%)	THC2697412	Unknown	
GSR	-4.7	6.1	Homo sapiens, clone IMAGE:5756011, mRNA.	BC035691	Hs.271510	BC035691

A_24_P187094	-4.7	3.5	A_24_P187094	A_24_P187094	Unknown	
CR2	-4.7	2.9	Human CR2/CD21/C3d/Epstein-Barr virus receptor mRNA, complete cds.	M26004	Hs.445757	NM_001006658
TMTC3	-4.7	2.1	transmembrane and tetratricopeptide repeat containing 3 (TMTC3), mRNA	NM_181783	Hs.331268	CR749309
C14orf58	-4.7	2.8	chromosome 14 open reading frame 58 (C14orf58), mRNA	NM_017791	Hs.509966	AY260577
ZAR1	-4.7	2.2	zygote arrest 1 (ZAR1), mRNA	NM_175619	Hs.276457	NM_175619
THC2616783	-4.7	2.6	THC2616783	THC2616783	Unknown	
SLC43A1	-4.7	2.8	solute carrier family 43, member 1 (SLC43A1), mRNA	NM_003627	Hs.591952	NM_003627
PCCB	-4.7	2.5	propionyl Coenzyme A carboxylase, beta polypeptide (PCCB), mRNA	NM_000532	Hs.63788	AB209009
SRD5A2L	-4.7	1.8	steroid 5 alpha-reductase 2-like (SRD5A2L), mRNA	NM_024592	Hs.590906	AK023414
ATP8A2	-4.7	6.4	ATPase, aminophospholipid transporter-like, Class I, type 8A, member 2 (ATP8A2), mRNA	NM_016529	Hs.444957	AL390129
SLC35B4	-4.7	2.0	solute carrier family 35, member B4 (SLC35B4), mRNA	NM_032826	Hs.490181	NM_032826
THC2659040	-4.7	3.6	THC2659040	THC2659040	Unknown	
STAT3	-4.7	2.3	signal transducer and activator of transcription 3 (acute-phase response factor) (STAT3), transcript variant 3, mRNA	NM_213662	Hs.463059	NM_139276
ZNF507	-4.7	1.6	zinc finger protein 507 (ZNF507), mRNA	NM_014910	Hs.205392	BX640881
PEO1	-4.7	1.8	progressive external ophthalmoplegia 1 (PEO1), mRNA	NM_021830	Hs.22678	BX640829
LOC442157	-4.7	2.5	PREDICTED: similar to heterogeneous nuclear ribonucleoprotein L (LOC442157), mRNA	XR_016510	Hs.646710	XR_016510
PCDH21	-4.7	9.2	protocadherin 21 (PCDH21), mRNA	NM_033100	Hs.137556	NM_033100
ABCC1	-4.7	2.0	ATP-binding cassette, sub-family C (CFTR/MRP), member 1 (ABCC1), transcript variant 2, mRNA	NM_019862	Hs.700568	NM_004996
SLC37A1	-4.7	2.8	solute carrier family 37 (glycerol-3-phosphate transporter), member 1 (SLC37A1), mRNA	NM_018964	Hs.547009	AK127597
KIAA0251	-4.7	1.7	KIAA0251 protein (KIAA0251), mRNA	NM_015027	Hs.370781	BC036520
LOC400506	-4.7	1.7	similar to TSG118.1 (LOC400506), mRNA	NM_001012991	Hs.585209	NM_001012991
TLL2	-4.7	3.8	tolloid-like 2 (TLL2), mRNA	NM_012465	Hs.154296	NM_012465
PPM1B	-4.7	2.5	protein phosphatase 1B (formerly 2C), magnesium-dependent, beta isoform (PPM1B), transcript variant 2, mRNA	NM_177968	Hs.416769	NM_177968
SETD5	-4.7	2.3	SET domain containing 5, mRNA (cDNA clone MGC:8816 IMAGE:3851178), complete cds.	BC020956	Hs.288164	XM_931376
ANKRD35	-4.7	2.4	ankyrin repeat domain 35 (ANKRD35), mRNA	NM_144698	Hs.435761	NM_144698
AF086427	-4.7	1.5	full length insert cDNA clone ZD79D11.	AF086427	Hs.597703	BX106238
PWP2	-4.7	2.6	PWP2 periodic tryptophan protein homolog (yeast) (PWP2), mRNA	NM_005049	Hs.449076	AB209438
GPR98	-4.7	1.4	G protein-coupled receptor 98 (GPR98), transcript variant 1, mRNA	NM_032119	Hs.591777	AF435925
HMGA1	-4.7	2.1	high mobility group AT-hook 1 (HMGA1), transcript variant 6, mRNA	NM_145904	Hs.518805	BC078664
PRKCQ	-4.7	4.3	protein kinase C, theta (PRKCQ), mRNA	NM_006257	Hs.498570	BX647657
USHBP1	-4.7	5.3	Usher syndrome 1C binding protein 1 (USHBP1), mRNA	NM_031941	Hs.512773	BC027910
C16orf68	-4.7	1.9	chromosome 16 open reading frame 68 (C16orf68), mRNA	NM_024109	Hs.306380	AK000114
BE968596	-4.7	3.1	601649770F1 NIH_MGC_74 cDNA clone IMAGE:3933472 5', mRNA sequence	BE968596	Hs.597383	BE968596
ACOT7	-4.7	2.7	acyl-CoA thioesterase 7 (ACOT7), transcript variant hBACHa, mRNA	NM_007274	Hs.126137	BC017365
PGAM5	-4.7	4.0	phosphoglycerate mutase family member 5 (PGAM5), mRNA	NM_138575	Hs.102558	AK097688
RAB5A	-4.7	1.6	RAB5A, member RAS oncogene family (RAB5A), mRNA	NM_004162	Hs.475663	NM_004162
DHTKD1	-4.7	2.0	dehydrogenase E1 and transketolase domain containing 1 (DHTKD1), mRNA	NM_018706	Hs.104980	NM_018706
RPS26	-4.7	2.8	ribosomal protein S26 (RPS26), mRNA	NM_001029	Hs.632726	BG254525
BX648855	-4.7	4.5	mRNA; cDNA DKFZp686L05231 (from clone DKFZp686L05231).	BX648855	Hs.418285	BC062295
PBEF1	-4.7	1.8	pre-B-cell colony enhancing factor 1 (PBEF1), mRNA	NM_005746	Hs.489615	NM_005746
TNPO1	-4.7	3.3	transportin 1 (TNPO1), transcript variant 1, mRNA	NM_002270	Hs.482497	NM_002270
LOC283953	-4.7	2.4	PREDICTED: hypothetical LOC283953 (LOC283953), mRNA	XM_208930	Hs.150849	BU737015
A_24_P75748	-4.7	2.4	A_24_P75748	A_24_P75748	Unknown	
FGFR1	-4.7	5.0	fibroblast growth factor receptor 1 (fms-related tyrosine kinase 2, Pfeiffer syndrome) (FGFR1), transcript variant 9, mRNA	NM_023111	Hs.264887	NM_023110
PRPS2	-4.7	1.8	phosphoribosyl pyrophosphate synthetase 2 (PRPS2), transcript variant 1, mRNA	NM_001039091	Hs.654581	NM_001039091
PDCD10	-4.7	1.4	programmed cell death 10 (PDCD10), transcript variant 1, mRNA	NM_007217	Hs.478150	BC002506
WRN	-4.7	1.4	Werner syndrome (WRN), mRNA	NM_000553	Hs.632050	NM_000553
RPS6KA1	-4.7	2.1	ribosomal protein S6 kinase, 90kDa, polypeptide 1 (RPS6KA1), transcript variant 1, mRNA	NM_002953	Hs.149957	BC014966
BOLA3	-4.7	1.8	bolA homolog 3 (E. coli) (BOLA3), transcript variant 1, mRNA	NM_212552	Hs.61472	CD357422
PCDH1	-4.7	2.2	protocadherin 1 (cadherin-like 1) (PCDH1), transcript variant 2, mRNA	NM_032420	Hs.79769	NM_032420
MRPS12	-4.7	2.1	mitochondrial ribosomal protein S12 (MRPS12), nuclear gene encoding mitochondrial protein, transcript variant 1, mRNA	NM_021107	Hs.411125	BU149479
LOC286382	-4.7	3.1	cDNA FLJ37037 fis, clone BRACE2011611.	AK094356	Hs.668691	AK094356
BX538051	-4.7	5.1	mRNA; cDNA DKFZp686F09156 (from clone DKFZp686F09156).	BX538051	Hs.149540	AK090411
PRKAR1B	-4.7	3.6	protein kinase, cAMP-dependent, regulatory, type I, beta (PRKAR1B), mRNA	NM_002735	Hs.520851	AL833563

NCAPD2	-4.7	1.9	non-SMC condensin I complex, subunit D2 (NCAPD2), mRNA	NM_014865	Hs.5719	D63880
ENST00000262[illegible]	-4.7	2.7	F20887_1, partial CDS (Fragment).	ENST00000262[illegible]	Unknown	
SLC27A3	-4.7	1.9	solute carrier family 27 (fatty acid transporter), member 3 (SLC27A3), mRNA	NM_024330	Hs.438723	AK074134
TPD52	-4.7	4.0	tumor protein D52 (TPD52), transcript variant 1, mRNA	NM_001025252	Hs.368433	NM_001025252
TMEM52	-4.7	2.5	transmembrane protein 52 (TMEM52), mRNA	NM_178545	Hs.123423	BM458237
CDT1	-4.7	2.3	chromatin licensing and DNA replication factor 1 (CDT1), mRNA	NM_030928	Hs.122908	AB053172
KIAA0652	-4.6	1.9	KIAA0652 (KIAA0652), mRNA	NM_014741	Hs.410092	NM_014741
MRPL30	-4.6	2.2	mitochondrial ribosomal protein L30 (MRPL30), nuclear gene encoding mitochondrial protein, transcript variant 3, mRNA	NM_145213	Hs.590896	NM_145212
SEPN1	-4.6	1.8	selenoprotein N, 1 (SEPN1), transcript variant 1, mRNA	NM_020451	Hs.323396	NM_020451
TMEM74	-4.6	2.8	transmembrane protein 74 (TMEM74), mRNA	NM_153015	Hs.99439	AK055230
TBC1D16	-4.6	2.6	mRNA; cDNA DKFZp451F1311 (from clone DKFZp451F1311).	CR936670	Hs.369819	CR936670
MARVELD2	-4.6	4.3	MARVEL domain containing 2 (MARVELD2), transcript variant 1, mRNA	NM_001038603	Hs.657687	AK055094
AL133018	-4.6	2.5	mRNA; cDNA DKFZp434F0327 (from clone DKFZp434F0327).	AL133018	Hs.567920	XR_017516
SALL4	-4.6	9.4	sal-like 4 (Drosophila) (SALL4), mRNA	NM_020436	Hs.517113	NM_020436
TOP1MT	-4.6	2.4	topoisomerase (DNA) I, mitochondrial (TOP1MT), nuclear gene encoding mitochondrial protein, mRNA	NM_052963	Hs.528574	BC039358
C1orf94	-4.6	8.6	chromosome 1 open reading frame 94 (C1orf94), mRNA	NM_032884	Hs.194610	BC064845
DUSP16	-4.6	2.6	dual specificity phosphatase 16 (DUSP16), mRNA	NM_030640	Hs.536535	AL833073
VKORC1L1	-4.6	2.0	vitamin K epoxide reductase complex, subunit 1-like 1 (VKORC1L1), mRNA	NM_173517	Hs.427232	CR627471
TMCO7	-4.6	2.3	mRNA for KIAA1746 protein, partial cds.	AB051533	Hs.13526	NM_024562
A_24_P195454	-4.6	2.7	A_24_P195454	A_24_P195454	Unknown	
EMG1	-4.6	2.1	EMG1 nucleolar protein homolog (S. cerevisiae) (EMG1), mRNA	NM_006331	Hs.558447	BM810908
AKAP1	-4.6	2.0	A kinase (PRKA) anchor protein 1 (AKAP1), nuclear gene encoding mitochondrial protein, mRNA	NM_003488	Hs.463506	NM_003488
CNDP2	-4.6	2.6	CNDP dipeptidase 2 (metallopeptidase M20 family) (CNDP2), mRNA	NM_018235	Hs.149185	AK024471
PPAT	-4.6	2.0	mRNA; cDNA DKFZp686L04109 (from clone DKFZp686L04109)	BX538303	Hs.699385	BX538303
HFE	-4.6	2.0	hemochromatosis (HFE), transcript variant 2, mRNA	NM_139002	Unknown	
A_23_P13232	-4.6	3.1	A_23_P13232	A_23_P13232	Unknown	
PCSK9	-4.6	3.2	proprotein convertase subtilisin/kexin type 9 (PCSK9), mRNA	NM_174936	Hs.18844	AK124635
ZNF468	-4.6	1.9	zinc finger protein 468 (ZNF468), transcript variant 1, mRNA	NM_199132	Hs.467223	CR936695
GGA2	-4.6	2.0	golgi associated, gamma adaptin ear containing, ARF binding protein 2 (GGA2), mRNA	NM_015044	Hs.460336	NM_015044
ZNF142	-4.6	1.8	zinc finger protein 142 (ZNF142), mRNA	NM_005081	Hs.657969	BC033631
MRPS25	-4.6	1.7	mitochondrial ribosomal protein S25 (MRPS25), nuclear gene encoding mitochondrial protein, mRNA	NM_022497	Hs.555973	NM_022497
RAD54L	-4.6	1.7	RAD54-like (S. cerevisiae) (RAD54L), mRNA	NM_003579	Hs.696005	NM_003579
THC2512199	-4.6	1.9	THC2512199	THC2512199	Unknown	
EME2	-4.6	2.7	mRNA for FLJ00151 protein.	AK074080	Unknown	
GPR160	-4.6	3.7	G protein-coupled receptor 160 (GPR160), mRNA	NM_014373	Hs.231320	AJ249248
THRB	-4.6	5.5	thyroid hormone receptor, beta (erythroblastic leukemia viral (v-erb-a) oncogene homolog 2, avian) (THRB), mRNA	NM_000461	Hs.187861	NM_000461
RAC3	-4.6	2.7	ras-related C3 botulinum toxin substrate 3 (rho family, small GTP binding protein Rac3) (RAC3), mRNA	NM_005052	Hs.45002	BM561442
KPTN	-4.6	1.8	kaptin (actin binding protein) (KPTN), mRNA	NM_007059	Hs.25441	NM_007059
ZNF342	-4.6	2.4	zinc finger protein 342 (ZNF342), mRNA	NM_145288	Hs.192237	AF447583
C9orf41	-4.6	1.7	mRNA; cDNA DKFZp686J16172 (from clone DKFZp686J16172).	ENST00000376[illegible]	Unknown	
SLC27A2	-4.6	1.8	solute carrier family 27 (fatty acid transporter), member 2 (SLC27A2), mRNA	NM_003645	Hs.11729	AK223145
SH2D2A	-4.6	2.0	SH2 domain protein 2A (SH2D2A), mRNA	NM_003975	Hs.103527	NM_003975
PRKCD	-4.6	2.2	protein kinase C, delta (PRKCD), transcript variant 1, mRNA	NM_006254	Hs.155342	NM_006254
TXNDC1	-4.6	1.8	thioredoxin domain containing 1 (TXNDC1), mRNA	NM_030755	Hs.125221	NM_030755
TPM3	-4.6	2.0	tropomyosin 3 (TPM3), transcript variant 3, mRNA	NM_001043352	Hs.699198	NM_152263
LOC644540	-4.6	1.5	PREDICTED: similar to transcription elongation factor B (SIII), polypeptide 1 (LOC644540), mRNA	XM_927664	Unknown	
MYO5C	-4.6	2.5	myosin VC (MYO5C), mRNA	NM_018728	Hs.487036	NM_018728
PH-4	-4.6	1.7	hypoxia-inducible factor prolyl 4-hydroxylase, mRNA (cDNA clone IMAGE:5017414), partial cds.	BC018731	Unknown	
ANK1	-4.6	2.9	ankyrin 1, erythrocytic (ANK1), transcript variant 3, mRNA	NM_000037	Hs.654438	NM_000037
LOC400039	-4.6	3.0	PREDICTED: similar to eukaryotic translation initiation factor 4A, isoform 1 (LOC400039), mRNA	XR_019265	Hs.614792	XR_019265
A_32_P9707	-4.6	1.7	A_32_P9707	A_32_P9707	Unknown	
SF3A1	-4.6	1.9	splicing factor 3a, subunit 1, 120kDa (SF3A1), transcript variant 1, mRNA	NM_005877	Hs.406277	NM_005877
RDH11	-4.6	1.8	retinol dehydrogenase 11 (all-trans/9-cis/11-cis) (RDH11), mRNA	NM_016026	Hs.226007	AB209223
THOC4	-4.6	2.3	THO complex 4 (THOC4), mRNA	NM_005782	Hs.534385	BU943861

DPH2	-4.6	2.0	DPH2 homolog (S. cerevisiae) (DPH2), transcript variant 1, mRNA	NM_001384	Hs.632398	NM_001384
LOC643233	-4.6	3.0	PREDICTED: similar to CG5327-PA (LOC643233), mRNA	ENST00000333	Unknown	
CA11	-4.6	1.9	carbonic anhydrase XI (CA11), mRNA	NM_001217	Hs.428446	NM_001217
C12orf52	-4.6	1.7	chromosome 12 open reading frame 52 (C12orf52), mRNA	NM_032848	Hs.524762	CR608011
BCL2L13	-4.6	2.4	BCL2-like 13 (apoptosis facilitator) (BCL2L13), nuclear gene encoding mitochondrial protein, mRNA	NM_015367	Hs.699302	AL831982
DHODH	-4.6	2.0	dihydroorotate dehydrogenase (DHODH), nuclear gene encoding mitochondrial protein, transcript variant 1, mRNA	NM_001361	Hs.654427	NM_001361
LOC649898	-4.6	3.4	cDNA: FLJ22734 fis, clone HUV00109.	AK026387	Hs.306842	AK026387
ARF3	-4.6	2.5	ADP-ribosylation factor 3 (ARF3), mRNA	NM_001659	Hs.119177	NM_001659
C16orf44	-4.6	1.8	mRNA for FLJ00127 protein.	AK074056	Hs.578546	AK074056
NSD1	-4.6	2.0	nuclear receptor binding SET domain protein 1 (NSD1), transcript variant 2, mRNA	NM_022455	Hs.654666	NM_022455
NAP1L2	-4.6	1.9	nucleosome assembly protein 1-like 2 (NAP1L2), mRNA	NM_021963	Hs.66180	NM_021963
NPFFR2	-4.6	8.9	neuropeptide FF receptor 2 (NPFFR2), transcript variant 2, mRNA	NM_053036	Hs.99231	NM_004885
NR6A1	-4.6	3.9	Orphan nuclear receptor NR6A1 (Germ cell nuclear factor) (GCNF) (Retinoid receptor-related testis-specific receptor) (RTR).	ENST00000344	Unknown	
RASA2	-4.6	2.4	RAS p21 protein activator 2 (RASA2), mRNA	NM_006506	Hs.655941	AF115573
CCDC112	-4.6	1.6	coiled-coil domain containing 112 (CCDC112), transcript variant 1, mRNA	NM_001040440	Hs.436121	NM_152549
A_24_P306585	-4.6	5.1	A_24_P306585	A_24_P306585	Unknown	
ECT2	-4.6	3.9	epithelial cell transforming sequence 2 oncogene (ECT2), mRNA	NM_018098	Hs.518299	AY376439
OSCAR	-4.6	2.7	osteoclast-associated receptor (OSCAR), transcript variant 1, mRNA	NM_206818	Hs.347655	BC035023
SLC35E1	-4.6	1.5	solute carrier family 35, member E1 (SLC35E1), mRNA	NM_024881	Hs.585896	BX640756
HSPA4	-4.6	1.9	heat shock 70kDa protein 4 (HSPA4), transcript variant 1, mRNA	NM_002154	Hs.90093	NM_002154
PAQR5	-4.6	3.6	progestin and adipoQ receptor family member V (PAQR5), mRNA	NM_017705	Hs.591096	NM_017705
DLG3	-4.6	2.6	discs, large homolog 3 (neuroendocrine-dlg, Drosophila) (DLG3), transcript variant 1, mRNA	NM_021120	Hs.522680	NM_021120
GPIAP1	-4.6	1.5	GPI-anchored membrane protein 1 (GPIAP1), transcript variant 2, mRNA	NM_203364	Hs.471818	NM_005898
PRKCD	-4.6	2.1	protein kinase C, delta (PRKCD), transcript variant 1, mRNA	NM_006254	Hs.155342	NM_006254
ITGA2B	-4.6	1.9	integrin, alpha 2b (platelet glycoprotein IIb of IIb/IIIa complex, antigen CD41) (ITGA2B), mRNA	NM_000419	Hs.411312	NM_000419
PTPN23	-4.6	2.4	protein tyrosine phosphatase, non-receptor type 23 (PTPN23), mRNA	NM_015466	Hs.25524	NM_015466
YBX2	-4.6	4.0	Y box binding protein 2 (YBX2), mRNA	NM_015982	Hs.567494	AK092747
CYB561	-4.6	2.7	cytochrome b-561 (CYB561), transcript variant 2, mRNA	NM_001017916	Hs.355264	AK095244
HCG18	-4.6	1.9	full-length cDNA clone CS0DI044YA04 of Placenta Cot 25-normalized of (human).	CR606587	Hs.485041	NM_003449
THC2680667	-4.6	2.3	Q9BVX4_HUMAN (Q9BVX4) MGC5566 protein, partial (23%)	THC2680667	Unknown	
C1orf190	-4.6	2.3	chromosome 1 open reading frame 190 (C1orf190), mRNA	NM_001013615	Hs.568642	NM_001013615
A_23_P113811	-4.5	17.2	A_23_P113811	A_23_P113811	Unknown	
RNASE6	-4.5	2.5	ribonuclease, RNase A family, k6 (RNASE6), mRNA	NM_005615	Hs.23262	BC020848
PURB	-4.5	5.2	purine-rich element binding protein B (PURB), mRNA	NM_033224	Hs.349150	NM_033224
A_24_P195749	-4.5	1.8	A_24_P195749	A_24_P195749	Unknown	
WDFY2	-4.5	2.5	WD repeat and FYVE domain containing 2 (WDFY2), mRNA	NM_052950	Hs.208550	NM_052950
NPFFR2	-4.5	10.9	neuropeptide FF receptor 2 (NPFFR2), transcript variant 2, mRNA	NM_053036	Hs.99231	NM_004885
MFAP3L	-4.5	4.2	microfibrillar-associated protein 3-like (MFAP3L), transcript variant 1, mRNA	NM_021647	Hs.593942	NM_021647
ATCAY	-4.5	2.2	ataxia, cerebellar, Cayman type (caytaxin) (ATCAY), mRNA	NM_033064	Hs.418055	NM_033064
SEPHS1	-4.5	2.5	Selenide, water dikinase 1 (EC 2.7.9.3) (Selenophosphate synthetase 1) (Selenium donor protein 1).	ENST00000327	Unknown	
HIATL1	-4.5	3.2	hippocampus abundant transcript-like 1 (HIATL1), mRNA	NM_032558	Hs.699244	NM_032558
ZFAND5	-4.5	1.8	zinc finger, AN1-type domain 5 (ZFAND5), mRNA	NM_006007	Hs.406096	BX648551
ST14	-4.5	2.3	suppression of tumorigenicity 14 (colon carcinoma) (ST14), mRNA	NM_021978	Hs.504315	NM_021978
TERF1	-4.5	11.6	telomeric repeat binding factor (NIMA-interacting) 1 (TERF1), transcript variant 1, mRNA	NM_017489	Hs.442707	AK128828
HOMER2	-4.5	2.2	homer homolog 2 (Drosophila) (HOMER2), transcript variant 2, mRNA	NM_199330	Hs.578443	AK128296
GEMIN5	-4.5	2.2	gem (nuclear organelle) associated protein 5 (GEMIN5), mRNA	NM_015465	Hs.483921	AK074066
HMGA1	-4.5	2.5	high mobility group AT-hook 1 (HMGA1), transcript variant 2, mRNA	NM_002131	Hs.518805	BC078664
PRKCD	-4.5	2.1	protein kinase C, delta (PRKCD), transcript variant 1, mRNA	NM_006254	Hs.155342	NM_006254
XPNPEP3	-4.5	1.6	X-prolyl aminopeptidase (aminopeptidase P) 3, putative (XPNPEP3), mRNA	NM_022098	Hs.529163	BX648018
RNF125	-4.5	5.2	cDNA: FLJ23481 fis, clone KAIA03003.	AK027134	Hs.633703	NM_017831
MPHOSPH9	-4.5	1.9	M-phase phosphoprotein 9 (MPHOSPH9), mRNA	NM_022782	Hs.577404	AK096541
EGF	-4.5	4.6	epidermal growth factor (beta-urogastrone) (EGF), mRNA	NM_001963	Hs.419815	NM_001963
GALNT14	-4.5	2.1	UDP-N-acetyl-alpha-D-galactosamine:polypeptide N-acetylgalactosaminyltransferase 14 (GalNAc-T14) (GALNT14), mRNA	NM_024572	Hs.468058	NM_024572
C7orf47	-4.5	2.0	chromosome 7 open reading frame 47 (C7orf47), mRNA	NM_145030	Hs.413359	BU197074

ALS2CL	-4.5	2.4	ALS2 C-terminal like (ALS2CL), transcript variant 1, mRNA	NM_147129	Hs.517937	CR627258
EXOSC6	-4.5	2.0	exosome component 6 (EXOSC6), mRNA	NM_058219	Hs.660633	CR604283
ZDHHC22	-4.5	12.4	zinc finger, DHHC-type containing 22 (ZDHHC22), mRNA	NM_174976	Hs.525485	NM_174976
JMJD2C	-4.5	1.7	jumonji domain containing 2C (JMJD2C), mRNA	NM_015061	Hs.157106	NM_015061
HTATIP2	-4.5	5.1	alternatively spliced product of metastasis-suppressor gene CC3 (TC3) mRNA, complete cds.	AF092095	Hs.90753	NM_001098522
MRPS12	-4.5	2.0	mitochondrial ribosomal protein S12 (MRPS12), nuclear gene encoding mitochondrial protein, transcript variant 1, mRNA	NM_021107	Hs.411125	BU149479
LOC401127	-4.5	2.0	hypothetical LOC401127, mRNA (cDNA clone MGC:18091 IMAGE:4151462), complete cds.	BC010526	Hs.383197	BC010526
TMEM61	-4.5	2.3	transmembrane protein 61 (TMEM61), mRNA	NM_182532	Hs.663950	BM808032
PLEKHG6	-4.5	2.2	pleckstrin homology domain containing, family G (with RhoGef domain) member 6 (PLEKHG6), mRNA	NM_018173	Hs.631660	AK095373
LOC649459	-4.5	1.9	PREDICTED: similar to Poly polymerase 1 (PARP-1) (ADPRT) (NAD(+) ADP-ribosyltransferase 1) (Poly synthetase 1) (LOC649459), mRNA	XR_019461	Hs.650809	XR_019461
ANKRD58	-4.5	2.9	full-length cDNA clone CS0DI054YC18 of Placenta Cot 25-normalized of (human).	ENST00000343[illegible]	Unknown	
C16orf44	-4.5	1.9	chromosome 16 open reading frame 44 (C16orf44), mRNA	NM_024731	Hs.578546	AK074056
PPP2R1B	-4.5	3.3	protein phosphatase 2 (formerly 2A), regulatory subunit A (PR 65), beta isoform (PPP2R1B), transcript variant 2, mRNA	NM_181699	Hs.584790	NM_002716
MAPK1	-4.5	2.6	mitogen-activated protein kinase 1 (MAPK1), transcript variant 2, mRNA	NM_138957	Hs.431850	AL157438
THC2577283	-4.5	1.7	BM994983 UI-H-ED0-awz-n-15-0-UI.s1 NCI_CGAP_ED0 cDNA clone IMAGE:5825870 3', mRNA sequence	THC2577283	Unknown	
IGF1R	-4.5	2.4	insulin-like growth factor 1 receptor (IGF1R), mRNA	NM_000875	Hs.643120	NM_000875
RET	-4.5	8.4	ret proto-oncogene (multiple endocrine neoplasia and medullary thyroid carcinoma 1, Hirschsprung disease) (RET), transcript variant 4, mRNA	NM_020630	Hs.350321	NM_020975
CYB561	-4.5	2.4	cytochrome b-561 (CYB561), transcript variant 2, mRNA	NM_001017916	Hs.355264	AK095244
TRIM65	-4.5	2.1	tripartite motif-containing 65 (TRIM65), mRNA	NM_173547	Hs.189823	NM_173547
C1orf135	-4.5	1.8	chromosome 1 open reading frame 135 (C1orf135), mRNA	NM_024037	Hs.149305	AK024326
USP9X	-4.5	3.6	ubiquitin specific peptidase 9, X-linked (USP9X), transcript variant 3, mRNA	NM_001039590	Hs.77578	NM_001039590
CSE1L	-4.5	1.5	CSE1 chromosome segregation 1-like (yeast) (CSE1L), mRNA	NM_001316	Hs.90073	NM_001316
STAT3	-4.5	2.3	signal transducer and activator of transcription 3 (acute-phase response factor) (STAT3), transcript variant 3, mRNA	NM_213662	Hs.463059	NM_139276
GTPBP1	-4.5	2.1	GTP binding protein 1 (GTPBP1), mRNA	NM_004286	Hs.276925	NM_004286
SNTB1	-4.5	2.4	syntrophin, beta 1 (dystrophin-associated protein A1, 59kDa, basic component 1) (SNTB1), mRNA	NM_021021	Hs.655236	AK026095
CNOT1	-4.5	2.2	CCR4-NOT transcription complex, subunit 1 (CNOT1), transcript variant 2, mRNA	NM_206999	Hs.460923	NM_016284
IGF1R	-4.5	2.1	insulin-like growth factor 1 receptor (IGF1R), mRNA	NM_000875	Hs.643120	NM_000875
CNNM3	-4.5	1.7	cyclin M3 (CNNM3), transcript variant 1, mRNA	NM_017623	Hs.643430	NM_017623
TMPRSS11E	-4.5	11.0	transmembrane protease, serine 11E (TMPRSS11E), mRNA	NM_014058	Hs.201877	AY359017
KIF1A	-4.5	2.4	kinesin family member 1A (KIF1A), mRNA	NM_004321	Hs.516802	NM_004321
THC2641484	-4.5	3.4	THC2641484	THC2641484	Unknown	
TSR1	-4.5	2.0	TSR1, 20S rRNA accumulation, homolog (S. cerevisiae) (TSR1), mRNA	NM_018128	Hs.388170	NM_018128
CPSF4	-4.5	1.8	cleavage and polyadenylation specific factor 4, 30kDa (CPSF4), mRNA	NM_006693	Hs.489287	NM_006693
ARMC8	-4.5	1.6	armadillo repeat containing 8 (ARMC8), transcript variant 3, mRNA	NM_213654	Hs.266826	AL096748
FSD1	-4.5	2.0	fibronectin type III and SPRY domain containing 1 (FSD1), mRNA	NM_024333	Hs.28144	AY032617
ENST00000278[illegible]	-4.5	2.8	OTTHUMP00000030191.	ENST00000278[illegible]	Unknown	
A_32_P100430	-4.5	1.7	A_32_P100430	A_32_P100430	Unknown	
AF087980	-4.5	1.6	full length insert cDNA clone YW27H10.	AF087980	Hs.643584	NM_178822
CPT2	-4.5	1.6	carnitine palmitoyltransferase II (CPT2), nuclear gene encoding mitochondrial protein, mRNA	NM_000098	Hs.699182	NM_000098
TRDMT1	-4.5	1.8	tRNA aspartic acid methyltransferase 1 (TRDMT1), transcript variant a, mRNA	NM_004412	Hs.351665	BX537961
RP5-1077B9.4	-4.5	2.2	invasion inhibitory protein 45 (IIP45), transcript variant 1, mRNA	NM_021933	Hs.8595	CR617109
AK022793	-4.5	2.1	cDNA FLJ12731 fis, clone NT2RP2000108.	AK022793	Hs.162105	BC043213
ARMC9	-4.5	1.7	armadillo repeat containing 9 (ARMC9), mRNA	NM_025139	Unknown	
RBM15	-4.5	1.4	RNA binding motif protein 15 (RBM15), mRNA	NM_022768	Hs.435947	BC042587
ATCAY	-4.5	4.2	ataxia, cerebellar, Cayman type (caytaxin) (ATCAY), mRNA	NM_033064	Hs.418055	NM_033064
A_24_P272515	-4.5	2.4	A_24_P272515	A_24_P272515	Unknown	
C9orf37	-4.5	1.9	chromosome 9 open reading frame 37 (C9orf37), mRNA	NM_032937	Hs.696059	CR605436
A_32_P91156	-4.5	1.8	A_32_P91156	A_32_P91156	Unknown	
PPL	-4.5	2.3	periplakin (PPL), mRNA	NM_002705	Hs.192233	NM_002705
CLDN19	-4.5	2.5	claudin 19 (CLDN19), mRNA	NM_148960	Hs.496270	AK096063
EMG1	-4.5	2.3	EMG1 nucleolar protein homolog (S. cerevisiae) (EMG1), mRNA	NM_006331	Hs.558447	BM810908
DNAH11	-4.5	1.7	dynein, axonemal, heavy chain 11 (DNAH11), mRNA	NM_003777	Hs.655326	NM_003777
ZNF195	-4.5	1.6	zinc finger protein 195 (ZNF195), mRNA	NM_007152	Hs.386294	AL833722

UTP20	-4.5	3.0	UTP20, small subunit (SSU) processome component, homolog (yeast) (UTP20), mRNA	NM_014503	Hs.295732	NM_014503
HELB	-4.5	2.7	helicase (DNA) B (HELB), mRNA	NM_033647	Hs.505941	NM_033647
LOC389634	-4.5	2.7	hypothetical LOC389634, mRNA (cDNA clone IMAGE:4157715).	BC037255	Hs.696467	AK124896
NUP210	-4.5	2.5	nucleoporin 210kDa (NUP210), mRNA	NM_024923	Hs.475525	NM_024923
MDH2	-4.5	1.6	malate dehydrogenase 2, NAD (mitochondrial) (MDH2), mRNA	NM_005918	Hs.520967	AK095803
IQGAP2	-4.5	2.5	IQ motif containing GTPase activating protein 2 (IQGAP2), mRNA	NM_006633	Hs.291030	NM_006633
MRM1	-4.5	3.3	mitochondrial rRNA methyltransferase 1 homolog (S. cerevisiae) (MRM1), mRNA	NM_024864	Hs.194864	NM_024864
STAT3	-4.5	2.4	signal transducer and activator of transcription 3 (acute-phase response factor) (STAT3), transcript variant 3, mRNA	NM_213662	Hs.463059	NM_139276
WDR21A	-4.5	1.4	WD repeat domain 21A (WDR21A), transcript variant 2, mRNA	NM_181340	Hs.331491	AL080157
UCHL1	-4.5	2.2	ubiquitin carboxyl-terminal esterase L1 (ubiquitin thiolesterase) (UCHL1), mRNA	NM_004181	Hs.518731	AB209038
CDCP1	-4.5	3.8	CUB domain containing protein 1 (CDCP1), transcript variant 1, mRNA	NM_022842	Hs.476093	NM_022842
THC2674649	-4.5	1.5	Q6JHZ7_HUMAN (Q6JHZ7) HCV-NS5ATP5 binding protein 1, partial (24%)	THC2674649	Unknown	
C1orf108	-4.5	2.2	chromosome 1 open reading frame 108 (C1orf108), mRNA	NM_024595	Hs.293563	AK223276
CCT5	-4.5	1.6	chaperonin containing TCP1, subunit 5 (epsilon) (CCT5), mRNA	NM_012073	Hs.1600	NM_012073
FAM20C	-4.5	2.6	family with sequence similarity 20, member C (FAM20C), mRNA	NM_020223	Hs.632006	AK125281
DNAJB6	-4.5	2.4	DnaJ (Hsp40) homolog, subfamily B, member 6 (DNAJB6), transcript variant 1, mRNA	NM_058246	Hs.490745	AL832124
CD40	-4.5	2.4	CD40 molecule, TNF receptor superfamily member 5 (CD40), transcript variant 1, mRNA	NM_001250	Hs.472860	AB209660
MAPK1	-4.5	2.6	mitogen-activated protein kinase 1 (MAPK1), transcript variant 2, mRNA	NM_138957	Hs.431850	AL157438
GPR19	-4.5	2.9	G protein-coupled receptor 19 (GPR19), mRNA	NM_006143	Hs.657862	AK096388
SPINT2	-4.5	1.6	serine peptidase inhibitor, Kunitz type, 2 (SPINT2), mRNA	NM_021102	Hs.31439	AK127479
POLD1	-4.5	1.6	polymerase (DNA directed), delta 1, catalytic subunit 125kDa (POLD1), mRNA	NM_002691	Hs.279413	AB209560
CTAGE4	-4.5	2.9	CTAGE-4 protein mRNA, complete cds.	AF338232	Hs.591829	AF338232
POR	-4.5	2.1	P450 (cytochrome) oxidoreductase (POR), mRNA	NM_000941	Hs.354056	CD014011
A_24_P306814	-4.5	3.5	A_24_P306814	A_24_P306814	Unknown	
A_24_P674924	-4.5	2.0	A_24_P674924	A_24_P674924	Unknown	
ACSL6	-4.4	5.2	acyl-CoA synthetase long-chain family member 6 (ACSL6), transcript variant 2, mRNA	NM_001009185	Hs.14945	AB020644
TIMP2	-4.4	3.9	TIMP metallopeptidase inhibitor 2 (TIMP2), mRNA	NM_003255	Hs.633514	NM_003255
OR5V1	-4.4	1.5	olfactory receptor, family 5, subfamily V, member 1 (OR5V1), mRNA	NM_030876	Hs.666316	AJ459859
POP1	-4.4	1.8	processing of precursor 1, ribonuclease P/MRP subunit (S. cerevisiae) (POP1), mRNA	NM_015029	Hs.252828	D31765
PAPSS2	-4.4	4.5	3'-phosphoadenosine 5'-phosphosulfate synthase 2 (PAPSS2), transcript variant 2, mRNA	NM_001015880	Hs.524491	NM_001015880
LOC285708	-4.4	2.3	cDNA FLJ34759 fis, clone NT2NE2001874.	AK092078	Hs.533011	AK092078
KIAA1822	-4.4	1.7	cDNA FLJ38284 fis, clone FCBBF3007152.	AK095603	Hs.288522	AB058725
LOH12CR1	-4.4	1.7	loss of heterozygosity, 12, chromosomal region 1 (LOH12CR1), mRNA	NM_058169	Hs.504805	NM_058169
EIF4A1	-4.4	2.9	eukaryotic translation initiation factor 4A, isoform 1 (EIF4A1), mRNA	NM_001416	Hs.129673	BG033657
EIF1AX	-4.4	1.6	eukaryotic translation initiation factor 1A, X-linked (EIF1AX), mRNA	NM_001412	Hs.522590	NM_001412
TFB1M	-4.4	2.2	transcription factor B1, mitochondrial (TFB1M), mRNA	NM_016020	Hs.279908	BM465245
MAD2L2	-4.4	2.5	MAD2 mitotic arrest deficient-like 2 (yeast) (MAD2L2), mRNA	NM_006341	Hs.19400	AK094316
NOC3L	-4.4	1.9	nucleolar complex associated 3 homolog (S. cerevisiae) (NOC3L), mRNA	NM_022451	Hs.74899	BC049850
PNPT1	-4.4	1.8	polyribonucleotide nucleotidyltransferase 1 (PNPT1), mRNA	NM_033109	Hs.388733	BC053660
NUS1	-4.4	1.8	nuclear undecaprenyl pyrophosphate synthase 1 homolog (S. cerevisiae) (NUS1), mRNA	NM_138459	Hs.289008	NM_138459
SURF5	-4.4	2.0	surfeit 5 (SURF5), transcript variant b, mRNA	NM_133640	Hs.78354	AK124518
RAPH1	-4.4	2.3	Ras association (RalGDS/AF-6) and pleckstrin homology domains 1 (RAPH1), transcript variant 1, mRNA	NM_213589	Hs.471162	NM_213589
A_32_P885123	-4.4	3.8	A_32_P885123	A_32_P885123	Unknown	
BTBD14B	-4.4	2.5	BTB (POZ) domain containing 14B (BTBD14B), mRNA	NM_052876	Hs.531614	NM_052876
SLC35B4	-4.4	1.8	solute carrier family 35, member B4 (SLC35B4), mRNA	NM_032826	Hs.490181	NM_032826
ZNF589	-4.4	2.7	zinc finger protein 589 (ZNF589), mRNA	NM_016089	Hs.172602	AB209387
C5orf16	-4.4	2.2	chromosome 5 open reading frame 16 (C5orf16), mRNA	NM_173828	Hs.120094	BC027919
ICOSLG	-4.4	2.0	inducible T-cell co-stimulator ligand (ICOSLG), mRNA	NM_015259	Hs.14155	NM_015259
LOC402093	-4.4	2.2	PREDICTED: similar to Adenosylhomocysteinase (S-adenosyl-L-homocysteine hydrolase) (AdoHcyase) (Liver copper-binding protein) (CUBP) (LOC402093), mRNA	XR_018505	Hs.651048	XR_018505
BHLHB8	-4.4	2.8	mRNA; cDNA DKFZp779C0742 (from clone DKFZp779C0742).	BX648200	Hs.511979	BX648200
ZNF57	-4.4	6.1	zinc finger protein 57 (ZNF57), mRNA	NM_173480	Hs.591378	BX537601
DBNDD1	-4.4	3.0	dysbindin (dystrobrevin binding protein 1) domain containing 1 (DBNDD1), transcript variant 1, mRNA	NM_001042610	Hs.301394	AK096507
LARP4	-4.4	2.0	La ribonucleoprotein domain family, member 4 (LARP4), transcript variant 2, mRNA	NM_199188	Hs.696260	NM_199188
PRODH	-4.4	5.9	proline dehydrogenase (oxidase) 1 (PRODH), nuclear gene encoding mitochondrial protein, mRNA	NM_016335	Hs.517352	AB209472
BG196763	-4.4	2.7	RST15990 Athersys RAGE Library cDNA, mRNA sequence	BG196763	Hs.610002	BQ061729

PDZD4	-4.4	2.9	PDZ domain containing 4 (PDZD4), mRNA	NM_032512	Hs.92732	AK127016
KCNK5	-4.4	3.4	potassium channel, subfamily K, member 5 (KCNK5), mRNA	NM_003740	Hs.444448	NM_003740
NAGS	-4.4	2.5	N-acetylglutamate synthase (NAGS), mRNA	NM_153006	Hs.8876	NM_153006
KIAA1244	-4.4	5.3	KIAA1244 (KIAA1244), mRNA	NM_020340	Hs.656215	NM_020340
SLC24A2	-4.4	2.0	solute carrier family 24 (sodium/potassium/calcium exchanger), member 2 (SLC24A2), mRNA	NM_020344	Hs.283014	NM_020344
RIMS4	-4.4	4.4	regulating synaptic membrane exocytosis 4 (RIMS4), mRNA	NM_182970	Hs.517065	NM_182970
ERBB3	-4.4	1.7	v-erb-b2 erythroblastic leukemia viral oncogene homolog 3 (avian) (ERBB3), transcript variant 1, mRNA	NM_001982	Hs.118681	NM_001982
PTAFR	-4.4	3.4	platelet-activating factor receptor (PTAFR), mRNA	NM_000952	Hs.433540	CD013879
A_32_P143803	-4.4	2.8	A_32_P143803	A_32_P143803	Unknown	
TYRO3	-4.4	1.7	TYRO3 protein tyrosine kinase (TYRO3), mRNA	NM_006293	Hs.381282	NM_006293
ENST00000310[illegible]	-4.4	3.2	RNA-binding protein 14 (RNA-binding motif protein 14) (RRM-containing coactivator activator/modulator) (Synaptotagmin-interacting protein) (SYT-interacting protein).	ENST00000310[illegible]	Unknown	
MAP3K9	-4.4	1.5	cDNA FLJ41436 fis, clone BRHIP2007741.	AK123430	Hs.445496	AK123430
HAS3	-4.4	4.1	hyaluronan synthase 3 (HAS3), transcript variant 2, mRNA	NM_138612	Hs.592069	NM_005329
C13orf23	-4.4	2.5	chromosome 13 open reading frame 23 (C13orf23), transcript variant 1, mRNA	NM_025138	Hs.318526	NM_025138
A_24_P298946	-4.4	2.4	A_24_P298946	A_24_P298946	Unknown	
CPO	-4.4	4.1	carboxypeptidase O (CPO), mRNA	NM_173077	Hs.684103	BC112078
BE161325	-4.4	1.8	BE161325 IL2-HT0435-130100-034-C09 HT0435 cDNA, mRNA sequence	BE161325	Unknown	
DCP2	-4.4	1.8	DCP2 decapping enzyme homolog (S. cerevisiae) (DCP2), mRNA	NM_152624	Hs.443875	NM_152624
RHOF	-4.4	2.3	ras homolog gene family, member F (in filopodia) (RHOF), mRNA	NM_019034	Hs.524804	NM_019034
PRKY	-4.4	3.3	protein kinase, Y-linked (PRKY), mRNA	NM_002760	Hs.632287	NM_002760
THC2741040	-4.4	2.7	CB243285 UI-CF-FN0-agc-l-12-0-UI.s1 UI-CF-FN0 cDNA clone UI-CF-FN0-agc-l-12-0-UI 3', mRNA sequence	THC2741040	Unknown	
NUDT21	-4.4	2.7	nudix (nucleoside diphosphate linked moiety X)-type motif 21 (NUDT21), mRNA	NM_007006	Hs.528834	BX537360
SNX21	-4.4	2.2	sorting nexin family member 21 (SNX21), transcript variant 2, mRNA	NM_152897	Hs.472854	NM_001042633
COBL	-4.4	2.0	cordon-bleu homolog (mouse) (COBL), mRNA	NM_015198	Hs.99141	BC150263
HSH2D	-4.4	1.7	hematopoietic SH2 domain containing (HSH2D), mRNA	NM_032855	Hs.631617	AK131222
LOC731514	-4.4	5.7	PREDICTED: similar to beta-1,4-mannosyltransferase (LOC731514), mRNA	XR_015505	Hs.678802	XR_015505
BC036645	-4.4	1.9	cDNA clone IMAGE:4814437.	BC036645	Hs.648455	BC036645
TALDO1	-4.4	1.6	transaldolase 1 (TALDO1), mRNA	NM_006755	Hs.438678	AK130060
SRFBP1	-4.4	1.5	serum response factor binding protein 1 (SRFBP1), mRNA	NM_152546	Hs.107622	NM_152546
UBE2G2	-4.4	2.4	ubiquitin-conjugating enzyme E2G 2 (UBC7 homolog, yeast) (UBE2G2), transcript variant 2, mRNA	NM_182688	Hs.529420	AK122700
W05707	-4.4	2.1	W05707 za87h03.r1 Soares_fetal_lung_NbHL19W cDNA clone IMAGE:299573 5' similar to SW:TCPD_MOUSE P80315 T-COMPLEX PROTEIN 1, DELTA SUBUNIT ;, mRNA sequence	W05707	Hs.421509	NM_006430
THC2680689	-4.4	235.1	ATPO_MOUSE (Q9DB20) ATP synthase O subunit, mitochondrial precursor (Oligomycin sensitivity conferral protein) (OSCP) , partial (54%)	THC2680689	Unknown	
CDC6	-4.4	2.3	cell division cycle 6 homolog (S. cerevisiae) (CDC6), mRNA	NM_001254	Hs.405958	NM_001254
KLKB1	-4.4	11.4	kallikrein B, plasma (Fletcher factor) 1 (KLKB1), mRNA	NM_000892	Hs.646885	NM_000892
C9orf100	-4.4	2.9	chromosome 9 open reading frame 100 (C9orf100), mRNA	NM_032818	Hs.534579	NM_032818
C6orf57	-4.4	2.1	chromosome 6 open reading frame 57 (C6orf57), mRNA	NM_145267	Hs.418495	BU598152
MAPK1	-4.4	2.4	mitogen-activated protein kinase 1 (MAPK1), transcript variant 2, mRNA	NM_138957	Hs.431850	AL157438
TSPYL1	-4.4	2.4	TSPY-like 1 (TSPYL1), mRNA	NM_003309	Hs.458358	NM_003309
FLJ44186	-4.4	27.4	CDNA FLJ44186 fis, clone THYMU2038797, weakly similar to B locus C type Lectin (FLJ44186 protein).	ENST00000340[illegible]	Unknown	
DDX18	-4.4	2.0	DEAD (Asp-Glu-Ala-Asp) box polypeptide 18 (DDX18), mRNA	NM_006773	Hs.363492	AB209392
TBRG4	-4.4	2.2	transforming growth factor beta regulator 4 (TBRG4), transcript variant 2, mRNA	NM_030900	Hs.231411	AB023165
UBE2S	-4.4	2.0	ubiquitin-conjugating enzyme E2S (UBE2S), mRNA	NM_014501	Hs.396393	BM479313
SH2D5	-4.4	4.3	cDNA FLJ42879 fis, clone BRHIP3001283.	AK124869	Hs.591522	XM_375698
GRB2	-4.4	2.2	growth factor receptor-bound protein 2 (GRB2), transcript variant 1, mRNA	NM_002086	Hs.444356	NM_002086
NP	-4.4	2.3	nucleoside phosphorylase (NP), mRNA	NM_000270	Hs.75514	AK126154
ZNF295	-4.4	2.9	zinc finger protein 295 (ZNF295), mRNA	NM_020727	Hs.434947	NM_001098402
PIPOX	-4.4	2.0	pipecolic acid oxidase (PIPOX), mRNA	NM_016518	Hs.462585	NM_016518
PHF20	-4.4	2.3	PHD finger protein 20 (PHF20), mRNA	NM_016436	Hs.517044	BC150178
WDR43	-4.4	2.2	Human mRNA for KIAA0007 gene, partial cds.	D87716	Hs.169863	D26488
C12orf53	-4.4	1.5	chromosome 12 open reading frame 53 (C12orf53), mRNA	NM_153685	Hs.44067	AK091656
KIAA1509	-4.4	1.7	mRNA for KIAA1509 protein, partial cds.	AB040942	Hs.525536	NM_001080414
WRN	-4.4	1.4	Werner syndrome (WRN), mRNA	NM_000553	Hs.632050	NM_000553
THC2562592	-4.4	1.9	1QIN_A Chain A, Human Glyoxalase I Complexed With S-(N-Hydroxy-N-P-Iodophenylcarbamoyl) Glutathione. {Homo sapiens} (exp=-1; wgp=0; cg=0), partial	THC2562592	Unknown	

MAPK1	-4.4	2.5	mitogen-activated protein kinase 1 (MAPK1), transcript variant 2, mRNA	NM_138957	Hs.431850	AL157438
WDR23	-4.4	1.9	WD repeat domain 23 (WDR23), transcript variant 1, mRNA	NM_025230	Hs.525251	NM_025230
SCLY	-4.4	3.8	putative selenocysteine lyase (SCLY) mRNA, complete cds.	AF175767	Hs.512606	AB209458
ADRA2C	-4.4	2.3	adrenergic, alpha-2C-, receptor (ADRA2C), mRNA	NM_000683	Hs.123022	BC142625
CASP10	-4.4	4.6	caspase 10, apoptosis-related cysteine peptidase (CASP10), transcript variant B, mRNA	NM_032974	Hs.5353	NM_032977
VRK1	-4.4	1.4	vaccinia related kinase 1 (VRK1), mRNA	NM_003384	Hs.422662	NM_003384
TRIM36	-4.4	3.9	tripartite motif-containing 36 (TRIM36), transcript variant 1, mRNA	NM_018700	Hs.519514	NM_018700
LOC647813	-4.4	3.0	PREDICTED: similar to Adenosylhomocysteinase (S-adenosyl-L-homocysteine hydrolase) (AdoHcyase) (LOC647813), mRNA	XR_018215	Hs.648168	XR_018215
LOC554203	-4.4	2.1	cDNA FLJ31610 fis, clone NT2RI2002865.	AK056172	Hs.648327	AK056172
CABLES1	-4.4	2.7	Cdk5 and Abl enzyme substrate 1 (CABLES1), mRNA	NM_138375	Hs.11108	AK025627
TFCP2L1	-4.4	8.7	transcription factor CP2-like 1 (TFCP2L1), mRNA	NM_014553	Hs.156471	BC064698
DHFR	-4.4	2.1	dihydrofolate reductase (DHFR), mRNA	NM_000791	Hs.648635	NM_000791
PRKCA	-4.4	4.0	protein kinase C, alpha (PRKCA), mRNA	NM_002737	Hs.531704	NM_002737
SSSCA1	-4.4	1.8	Sjogren's syndrome/scleroderma autoantigen 1 (SSSCA1), mRNA	NM_006396	Hs.654840	BQ711192
AK023018	-4.4	1.7	cDNA FLJ12956 fis, clone NT2RP2005501.	AK023018	Hs.606550	AK023018
SLC2A4	-4.4	1.7	solute carrier family 2 (facilitated glucose transporter), member 4 (SLC2A4), mRNA	NM_001042	Hs.380691	NM_001042
PRKCD	-4.4	2.1	protein kinase C, delta (PRKCD), transcript variant 1, mRNA	NM_006254	Hs.155342	NM_006254
ESRRA	-4.4	2.1	estrogen-related receptor alpha (ESRRA), mRNA	NM_004451	Hs.110849	BC092470
RILP	-4.4	2.5	Rab interacting lysosomal protein (RILP), mRNA	NM_031430	Hs.534497	AJ404317
NOC3L	-4.4	1.9	nucleolar complex associated 3 homolog (S. cerevisiae) (NOC3L), mRNA	NM_022451	Hs.74899	BC049850
DEPDC4	-4.4	1.9	DEP domain containing 4 (DEPDC4), mRNA	NM_152317	Hs.653118	AK090824
RALGPS1	-4.4	4.4	Ral GEF with PH domain and SH3 binding motif 1 (RALGPS1), mRNA	NM_014636	Hs.696056	BX648170
AF116624	-4.4	2.6	PRO1146 mRNA, complete cds.	AF116624	Unknown	
KIF5A	-4.4	2.3	mRNA for KIF5A variant protein, partial cds, clone: ph00435.	AB210045	Hs.151219	AB210045
DSG2	-4.4	2.3	desmoglein 2 (DSG2), mRNA	NM_001943	Hs.412597	Z26317
ZNF195	-4.4	1.5	zinc finger protein 195 (ZNF195), mRNA	NM_007152	Hs.386294	AL833722
ZNF761	-4.4	2.7	zinc finger protein 761 (ZNF761), mRNA	NM_001008401	Hs.699590	BX647981
SORL1	-4.4	4.3	sortilin-related receptor, L(DLR class) A repeats-containing (SORL1), mRNA	NM_003105	Hs.368592	NM_003105
HRASLS3	-4.4	18.8	HRAS-like suppressor 3 (HRASLS3), mRNA	NM_007069	Hs.502775	BQ931456
MRPL45	-4.4	1.6	mitochondrial ribosomal protein L45 (MRPL45), nuclear gene encoding mitochondrial protein, mRNA	NM_032351	Hs.462913	NM_032351
KLHDC4	-4.4	1.7	unknown mRNA.	AY251274	Hs.123450	AY251274
SLC7A8	-4.4	2.8	solute carrier family 7 (cationic amino acid transporter, y+ system), member 8 (SLC7A8), transcript variant 2, mRNA	NM_182728	Hs.632348	Y18483
RNF135	-4.3	2.0	ring finger protein 135 (RNF135), transcript variant 2, mRNA	NM_197939	Hs.29874	NM_032322
C19orf25	-4.3	2.0	chromosome 19 open reading frame 25 (C19orf25), mRNA	NM_152482	Hs.532840	AK056004
THC2742069	-4.3	5.8	Q4TA84_TETNG (Q4TA84) Chromosome 3 SCAF7413, whole genome shotgun sequence. (Fragment), partial (6%)	THC2742069	Unknown	
THC2675062	-4.3	2.8	THC2675062	THC2675062	Unknown	
UBE2S	-4.3	2.4	ubiquitin-conjugating enzyme E2S (UBE2S), mRNA	NM_014501	Hs.396393	BM479313
A_24_P50139	-4.3	10.5	A_24_P50139	A_24_P50139	Unknown	
C19orf46	-4.3	2.4	chromosome 19 open reading frame 46 (C19orf46), mRNA	NM_001039876	Hs.436743	NM_001039876
QRSL1	-4.3	2.2	glutaminyl-tRNA synthase (glutamine-hydrolyzing)-like 1 (QRSL1), mRNA	NM_018292	Hs.406917	AL136679
SOX11	-4.3	4.1	SRY (sex determining region Y)-box 11 (SOX11), mRNA	NM_003108	Hs.432638	AB028641
BC039399	-4.3	2.2	cDNA clone IMAGE:5300185.	BC039399	Hs.460579	BC039399
BDH1	-4.3	2.7	3-hydroxybutyrate dehydrogenase, type 1 (BDH1), nuclear gene encoding mitochondrial protein, transcript variant 3, mRNA	NM_203314	Hs.274539	NM_203314
RAB43	-4.3	2.7	RAB43, member RAS oncogene family (RAB43), mRNA	NM_198490	Hs.546542	NM_198490
PGAM5	-4.3	3.8	phosphoglycerate mutase family member 5 (PGAM5), mRNA	NM_138575	Hs.102558	AK097688
THC2753400	-4.3	2.0	THC2753400	THC2753400	Unknown	
CXorf38	-4.3	2.3	chromosome X open reading frame 38 (CXorf38), mRNA	NM_144970	Hs.495961	AK095710
LOC643513	-4.3	2.3	PREDICTED: similar to Importin alpha-2 subunit (Karyopherin alpha-2 subunit) (SRP1-alpha) (RAG cohort protein 1) (LOC643513), mRNA	XR_019062	Hs.591681	XR_019062
TNFSF11	-4.3	8.3	tumor necrosis factor (ligand) superfamily, member 11 (TNFSF11), transcript variant 1, mRNA	NM_003701	Hs.333791	AF053712
PRR13	-4.3	2.0	proline rich 13 (PRR13), transcript variant 3, mRNA	NM_001005354	Hs.426359	BE906094
SLCO4C1	-4.3	3.5	solute carrier organic anion transporter family, member 4C1 (SLCO4C1), mRNA	NM_180991	Hs.127648	NM_180991
SH3D19	-4.3	2.5	SH3 domain protein D19 (SH3D19), mRNA	NM_001009555	Hs.567725	BX647422
SH2B1	-4.3	2.0	SH2B adaptor protein 1 (SH2B1), mRNA	NM_015503	Hs.15744	AB037720
GDPD2	-4.3	6.5	glycerophosphodiester phosphodiesterase domain containing 2 (GDPD2), mRNA	NM_017711	Hs.438712	NM_017711

C18orf56	-4.3	1.8	chromosome 18 open reading frame 56 (C18orf56), mRNA	NM_001012716	Hs.274959	BI754401
TMEM92	-4.3	2.9	transmembrane protein 92 (TMEM92), mRNA	NM_153229	Hs.224630	AK090637
ZNF544	-4.3	2.5	zinc finger protein 544 (ZNF544), mRNA	NM_014480	Hs.438994	AK226080
TXNL5	-4.3	1.6	thioredoxin-like 5 (TXNL5), mRNA	NM_032731	Hs.408236	BC050406
NAT12	-4.3	2.1	N-acetyltransferase 12 (NAT12), mRNA	NM_001011713	Hs.165465	BX648700
BMP8A	-4.3	2.1	bone morphogenetic protein 8a (BMP8A), mRNA	NM_181809	Hs.472497	NM_181809
SLC7A3	-4.3	3.8	solute carrier family 7 (cationic amino acid transporter, y+ system), member 3 (SLC7A3), transcript variant 1, mRNA	NM_032803	Hs.175220	NM_032803
CR605719	-4.3	1.9	full-length cDNA clone CS0DK002YG10 of HeLa cells Cot 25-normalized of (human).	CR605719	Hs.593076	CR605719
CR936791	-4.3	4.7	mRNA; cDNA DKFZp781C2356 (from clone DKFZp781C2356).	CR936791	Hs.696414	CR936791
RIMS2	-4.3	3.0	regulating synaptic membrane exocytosis 2 (RIMS2), mRNA	NM_014677	Hs.655271	NM_001100117
GRPR	-4.3	8.2	gastrin-releasing peptide receptor (GRPR), mRNA	NM_005314	Hs.567282	NM_005314
ENST00000332[illegible]	-4.3	3.9	PREDICTED: similar to unc-93 homolog B1 (LOC643384), mRNA	ENST00000332[illegible]	Unknown	
BIRC5	-4.3	2.0	baculoviral IAP repeat-containing 5 (survivin) (BIRC5), transcript variant 3, mRNA	NM_001012271	Hs.514527	NM_001012271
SMARCD2	-4.3	3.0	SWI/SNF related, matrix associated, actin dependent regulator of chromatin, subfamily d, member 2 (SMARCD2), mRNA	NM_003077	Hs.250581	NM_001098426
MMP12	-4.3	2.2	matrix metallopeptidase 12 (macrophage elastase) (MMP12), mRNA	NM_002426	Hs.1695	NM_002426
KHK	-4.3	2.3	ketohexokinase (fructokinase) (KHK), transcript variant a, mRNA	NM_000221	Hs.567297	NM_000221
C19orf23	-4.3	1.7	chromosome 19 open reading frame 23 (C19orf23), mRNA	NM_152480	Hs.438829	BC026041
WRN	-4.3	1.4	Werner syndrome (WRN), mRNA	NM_000553	Hs.632050	NM_000553
ALDH1A3	-4.3	4.7	aldehyde dehydrogenase 1 family, member A3 (ALDH1A3), mRNA	NM_000693	Hs.459538	NM_000693
AL109706	-4.3	2.6	mRNA full length insert cDNA clone EUROIMAGE 362430.	AL109706	Hs.32769	AL109706
ZNF195	-4.3	1.5	zinc finger protein 195 (ZNF195), mRNA	NM_007152	Hs.386294	AL833722
A_24_P933372	-4.3	3.1	A_24_P933372	A_24_P933372	Unknown	
CNTNAP2	-4.3	6.1	contactin associated protein-like 2 (CNTNAP2), mRNA	NM_014141	Hs.655684	NM_014141
GINS4	-4.3	2.6	GINS complex subunit 4 (Sld5 homolog) (GINS4), mRNA	NM_032336	Hs.656996	AK095334
C18orf17	-4.3	2.4	chromosome 18 open reading frame 17, mRNA (cDNA clone IMAGE:5582870), partial cds.	BC032684	Hs.128576	AK091080
NOC3L	-4.3	1.9	nucleolar complex associated 3 homolog (S. cerevisiae) (NOC3L), mRNA	NM_022451	Hs.74899	BC049850
TCOF1	-4.3	2.0	Treacher Collins-Franceschetti syndrome 1 (TCOF1), transcript variant 3, mRNA	NM_001008657	Hs.519672	AB209317
BX648958	-4.3	3.4	mRNA; cDNA DKFZp686L0948 (from clone DKFZp686L0948).	BX648958	Hs.375836	NM_173598
BQ632351	-4.3	1.6	BQ632351 il25h07.x1 HR85 islet cDNA clone IMAGE:6031332 3' similar to TR:Q9Z2X1 Q9Z2X1 RIBONUCLEOPROTEIN F. ;, mRNA sequence	BQ632351	Hs.808	NM_001098208
BIRC5	-4.3	1.9	baculoviral IAP repeat-containing 5 (survivin) (BIRC5), transcript variant 3, mRNA	NM_001012271	Hs.514527	NM_001012271
PTPN6	-4.3	2.8	protein tyrosine phosphatase, non-receptor type 6 (PTPN6), transcript variant 1, mRNA	NM_002831	Hs.63489	NM_002831
CD40	-4.3	2.5	CD40 molecule, TNF receptor superfamily member 5 (CD40), transcript variant 1, mRNA	NM_001250	Hs.472860	AB209660
IL28RA	-4.3	1.6	interleukin 28 receptor, alpha (interferon, lambda receptor) (IL28RA), transcript variant 1, mRNA	NM_170743	Hs.221375	NM_170743
STAT3	-4.3	2.1	signal transducer and activator of transcription 3 (acute-phase response factor) (STAT3), transcript variant 3, mRNA	NM_213662	Hs.463059	NM_139276
ATG5	-4.3	1.8	ATG5 autophagy related 5 homolog (S. cerevisiae) (ATG5), mRNA	NM_004849	Hs.486063	Y11588
THC2612620	-4.3	1.7	THC2612620	THC2612620	Unknown	
FABP5	-4.3	2.0	fatty acid binding protein 5 (psoriasis-associated) (FABP5), mRNA	NM_001444	Hs.408061	BM563703
CHFR	-4.3	2.8	checkpoint with forkhead and ring finger domains (CHFR), mRNA	NM_018223	Hs.656770	BC012072
CR936791	-4.3	2.7	mRNA; cDNA DKFZp781C2356 (from clone DKFZp781C2356).	CR936791	Hs.696414	CR936791
C9orf58	-4.3	2.1	chromosome 9 open reading frame 58 (C9orf58), transcript variant 2, mRNA	NM_001002260	Unknown	
A_24_P213321	-4.3	3.0	A_24_P213321	A_24_P213321	Unknown	
BIRC5	-4.3	1.9	baculoviral IAP repeat-containing 5 (survivin) (BIRC5), transcript variant 3, mRNA	NM_001012271	Hs.514527	NM_001012271
PLEKHH1	-4.3	2.1	pleckstrin homology domain containing, family H (with MyTH4 domain) member 1 (PLEKHH1), mRNA	NM_020715	Hs.594236	NM_020715
MNAB	-4.3	2.0	mRNA; cDNA DKFZp667B165 (from clone DKFZp667B165).	AL833177	Unknown	
SLC24A6	-4.3	2.0	solute carrier family 24 (sodium/potassium/calcium exchanger), member 6 (SLC24A6), mRNA	NM_024959	Hs.286194	NM_024959
DCPS	-4.3	2.2	decapping enzyme, scavenger (DCPS), mRNA	NM_014026	Hs.504249	AK090828
TJP2	-4.3	5.1	tight junction protein 2 (zona occludens 2) (TJP2), transcript variant 2, mRNA	NM_201629	Hs.50382	AB209630
KLHL23	-4.3	2.0	kelch-like 23 (Drosophila) (KLHL23), mRNA	NM_144711	Hs.655150	BC010437
RHPN1	-4.3	1.5	rhophilin, Rho GTPase binding protein 1 (RHPN1), mRNA	NM_052924	Hs.521912	NM_052924
PRMT3	-4.3	2.2	protein arginine methyltransferase 3 (PRMT3), mRNA	NM_005788	Hs.152337	AK125039
LAD1	-4.3	2.4	ladinin 1 (LAD1), mRNA	NM_005558	Hs.519035	NM_005558
SART3	-4.3	2.0	squamous cell carcinoma antigen recognized by T cells 3 (SART3), mRNA	NM_014706	Hs.584842	CR933631
ZNF506	-4.3	2.2	mRNA; cDNA DKFZp761G18121 (from clone DKFZp761G18121).	AL136548	Unknown	
C1QBP	-4.3	2.0	complement component 1, q subcomponent binding protein (C1QBP), nuclear gene encoding mitochondrial protein, mRNA	NM_001212	Hs.555866	BF972145

CHRM2	-4.3	2.0	cholinergic receptor, muscarinic 2 (CHRM2), transcript variant 1, mRNA	NM_001006630	Hs.535891	AL832585
NAT13	-4.3	1.5	N-acetyltransferase 13 (NAT13), mRNA	NM_025146	Hs.654706	BC012731
KIAA0179	-4.3	2.1	KIAA0179 (KIAA0179), mRNA	NM_015056	Hs.654727	NM_015056
SULT4A1	-4.3	1.9	sulfotransferase family 4A, member 1 (SULT4A1), mRNA	NM_014351	Hs.189810	BC030665
FZD5	-4.3	19.1	frizzled homolog 5 (Drosophila) (FZD5), mRNA	NM_003468	Hs.17631	NM_003468
SLC2A10	-4.3	2.3	solute carrier family 2 (facilitated glucose transporter), member 10 (SLC2A10), mRNA	NM_030777	Hs.305971	AF248053
NPAS1	-4.3	4.2	neuronal PAS domain protein 1 (NPAS1), mRNA	NM_002517	Hs.79564	BC039016
ARL2	-4.3	2.1	ADP-ribosylation factor-like 2 (ARL2), mRNA	NM_001667	Hs.502836	BM916077
GNA14	-4.3	40.7	guanine nucleotide binding protein (G protein), alpha 14 (GNA14), mRNA	NM_004297	Hs.657795	NM_004297
SLCO1A2	-4.3	2.7	solute carrier organic anion transporter family, member 1A2 (SLCO1A2), transcript variant 3, mRNA	NM_005075	Hs.46440	NM_134431
NAGS	-4.3	2.2	N-acetylglutamate synthase (NAGS), mRNA	NM_153006	Hs.8876	NM_153006
FLJ22659	-4.3	2.5	cDNA: FLJ22659 fis, clone HSI07953.	AK026312	Hs.677375	AK026312
LOC344167	-4.3	1.9	PREDICTED: similar to forkhead box I2 (LOC344167), mRNA	XM_001126001	Unknown	
NUP210	-4.3	2.9	nucleoporin 210kDa (NUP210), mRNA	NM_024923	Hs.475525	NM_024923
FAM29A	-4.3	1.5	family with sequence similarity 29, member A (FAM29A), mRNA	NM_017645	Hs.533468	NM_017645
SEMA6A	-4.3	3.4	sema domain, transmembrane domain (TM), and cytoplasmic domain, (semaphorin) 6A (SEMA6A), mRNA	NM_020796	Hs.156967	BC032619
BC034299	-4.3	1.5	Homo sapiens, clone IMAGE:5165176, mRNA.	BC034299	Hs.568685	BC034299
STAT3	-4.3	2.2	signal transducer and activator of transcription 3 (acute-phase response factor) (STAT3), transcript variant 3, mRNA	NM_213662	Hs.463059	NM_139276
SIRT5	-4.3	2.1	sirtuin (silent mating type information regulation 2 homolog) 5 (S. cerevisiae) (SIRT5), transcript variant 1, mRNA	NM_012241	Hs.567431	BC035196
RFFL	-4.3	2.5	ring finger and FYVE-like domain containing 1 (RFFL), transcript variant 1, mRNA	NM_057178	Hs.13680	NM_057178
PNPO	-4.3	2.6	pyridoxamine 5'-phosphate oxidase (PNPO), mRNA	NM_018129	Hs.631742	NM_018129
LDLR	-4.3	2.4	low density lipoprotein receptor (familial hypercholesterolemia) (LDLR), mRNA	NM_000527	Hs.213289	BX648281
TRDMT1	-4.3	2.1	tRNA aspartic acid methyltransferase 1 (TRDMT1), transcript variant a, mRNA	NM_004412	Hs.351665	BX537961
RABGAP1L	-4.3	8.3	RAB GTPase activating protein 1-like (RABGAP1L), transcript variant 1, mRNA	NM_014857	Hs.585378	AB007940
BG216262	-4.3	2.5	RST35951 Athersys RAGE Library cDNA, mRNA sequence	BG216262	Hs.637431	BG216262
LIN7C	-4.3	1.6	lin-7 homolog C (C. elegans) (LIN7C), mRNA	NM_018362	Hs.91393	BC053907
PRKCD	-4.3	2.3	protein kinase C, delta (PRKCD), transcript variant 1, mRNA	NM_006254	Hs.155342	NM_006254
SNRPD3	-4.3	1.4	small nuclear ribonucleoprotein D3 polypeptide 18kDa (SNRPD3), mRNA	NM_004175	Hs.356549	NM_004175
INHBB	-4.3	5.2	inhibin, beta B (activin AB beta polypeptide) (INHBB), mRNA	NM_002193	Hs.1735	NM_002193
STAT1	-4.3	1.7	signal transducer and activator of transcription 1, 91kDa (STAT1), transcript variant beta, mRNA	NM_139266	Hs.699271	NM_007315
A_24_P910246	-4.3	20.9	A_24_P910246	A_24_P910246	Unknown	
C6orf211	-4.3	1.6	chromosome 6 open reading frame 211 (C6orf211), mRNA	NM_024573	Hs.15929	AK022972
SCN8A	-4.3	5.9	sodium channel, voltage gated, type VIII, alpha (SCN8A), mRNA	NM_014191	Hs.436550	NM_014191
EPB41L4B	-4.3	3.2	cDNA: FLJ21596 fis, clone COL07110.	AK025249	Unknown	
MAPK1	-4.3	2.5	mitogen-activated protein kinase 1 (MAPK1), transcript variant 2, mRNA	NM_138957	Hs.431850	AL157438
NLRP7	-4.3	16.6	NLR family, pyrin domain containing 7 (NLRP7), transcript variant 1, mRNA	NM_139176	Hs.351118	NM_139176
HIPK1	-4.3	2.8	homeodomain interacting protein kinase 1 (HIPK1), transcript variant 2, mRNA	NM_152696	Hs.532363	NM_198268
PNPT1	-4.3	1.5	mRNA; cDNA DKFZp762K1914 (from clone DKFZp762K1914).	CR749867	Hs.388733	BC053660
THC2686753	-4.3	3.0	THC2686753	THC2686753	Unknown	
B4GALT6	-4.3	1.7	UDP-Gal:betaGlcNAc beta 1,4- galactosyltransferase, polypeptide 6 (B4GALT6), mRNA	NM_004775	Hs.591063	AB209194
PARP14	-4.3	3.0	poly (ADP-ribose) polymerase family, member 14 (PARP14), mRNA	NM_017554	Hs.687055	BX648758
OSBPL1A	-4.3	1.9	oxysterol binding protein-like 1A, mRNA (cDNA clone IMAGE:5922907), complete cds.	BC041563	Hs.370725	BX647893
UAP1L1	-4.2	2.5	UDP-N-acteylglucosamine pyrophosphorylase 1-like 1 (UAP1L1), mRNA	NM_207309	Hs.142076	AK074143
CKMT1A	-4.2	13.2	creatine kinase, mitochondrial 1A (CKMT1A), nuclear gene encoding mitochondrial protein, mRNA	NM_001015001	Hs.425633	AK094322
LOC389842	-4.2	2.5	PREDICTED: similar to Ran-specific GTPase-activating protein (Ran-binding protein 1) (RanBP1) (LOC389842), mRNA	XM_372200	Hs.567989	BQ069017
CCRN4L	-4.2	7.4	CCR4 carbon catabolite repression 4-like (S. cerevisiae) (CCRN4L), mRNA	NM_012118	Hs.656047	NM_012118
CEACAM5	-4.2	2.1	carcinoembryonic antigen-related cell adhesion molecule 5 (CEACAM5), mRNA	NM_004363	Hs.466814	NM_004363
ZNF195	-4.2	1.5	zinc finger protein 195 (ZNF195), mRNA	NM_007152	Hs.386294	AL833722
HAB1	-4.2	1.8	H.sapiens B1 mRNA for mucin.	X83412	Unknown	
STXBP1	-4.2	2.0	syntaxin binding protein 1 (STXBP1), transcript variant 2, mRNA	NM_001032221	Hs.288229	NM_003165
BSPRY	-4.2	4.9	B-box and SPRY domain containing (BSPRY), mRNA	NM_017688	Hs.632677	NM_017688
DIAPH2	-4.2	3.1	diaphanous homolog 2 (Drosophila) (DIAPH2), transcript variant 156, mRNA	NM_006729	Hs.696382	Y15909
POLR3D	-4.2	1.6	Human BN51 mRNA, complete cds.	M17754	Hs.148342	AK026588
CXorf45	-4.2	1.9	chromosome X open reading frame 45 (CXorf45), mRNA	NM_001039210	Unknown	

THC2538841	-4.2	3.9	ALU1_HUMAN (P39188) Alu subfamily J sequence contamination warning entry, partial (8%)	THC2538841	Unknown	
MAPK1	-4.2	2.4	mitogen-activated protein kinase 1 (MAPK1), transcript variant 2, mRNA	NM_138957	Hs.431850	AL157438
IRF3	-4.2	2.1	interferon regulatory factor 3 (IRF3), mRNA	NM_001571	Hs.75254	AK057577
FKBP1B	-4.2	2.8	FK506 binding protein 1B, 12.6 kDa (FKBP1B), transcript variant 2, mRNA	NM_054033	Hs.407482	BX647146
FOXO1A	-4.2	2.2	forkhead box O1A (rhabdomyosarcoma) (FOXO1A), mRNA	NM_002015	Hs.370666	NM_002015
JTV1	-4.2	1.8	JTV1 gene (JTV1), mRNA	NM_006303	Hs.301613	BQ065837
NOC3L	-4.2	1.9	nucleolar complex associated 3 homolog (S. cerevisiae) (NOC3L), mRNA	NM_022451	Hs.74899	BC049850
CTDP1	-4.2	2.4	CTD (carboxy-terminal domain, RNA polymerase II, polypeptide A) phosphatase, subunit 1 (CTDP1), transcript variant FCP1b, mRNA	NM_048368	Hs.465490	NM_004715
BM975266	-4.2	3.0	UI-CF-EC1-acc-p-22-0-UI.s1 UI-CF-EC1 cDNA clone UI-CF-EC1-acc-p-22-0-UI 3', mRNA sequence	BM975266	Hs.633884	BM975266
C10orf125	-4.2	1.8	chromosome 10 open reading frame 125 (C10orf125), mRNA	NM_198472	Hs.155823	BQ277643
MYBPC2	-4.2	2.5	myosin binding protein C, fast type (MYBPC2), mRNA	NM_004533	Hs.85937	NM_004533
CD40	-4.2	2.4	CD40 molecule, TNF receptor superfamily member 5 (CD40), transcript variant 1, mRNA	NM_001250	Hs.472860	AB209660
DDR1	-4.2	2.0	discoidin domain receptor family, member 1 (DDR1), transcript variant 1, mRNA	NM_013993	Hs.631988	NM_013993
A_24_P594094	-4.2	3.0	A_24_P594094	A_24_P594094	Unknown	
PROCA1	-4.2	2.4	proline-rich cyclin A1-interacting protein (PROCA1), mRNA	NM_152465	Hs.207471	AL137531
LETM1	-4.2	2.7	leucine zipper-EF-hand containing transmembrane protein 1 (LETM1), mRNA	NM_012318	Hs.120165	BX537709
AA130949	-4.2	3.3	AA130949 zo14f05.r1 Stratagene colon (#937204) cDNA clone IMAGE:586881 5', mRNA sequence	AA130949	Unknown	
MCM6	-4.2	1.7	minichromosome maintenance deficient 6 homolog (S. cerevisiae) (MCM6), mRNA	NM_005915	Hs.444118	NM_005915
A_24_P894763	-4.2	2.4	A_24_P894763	A_24_P894763	Unknown	
AA507111	-4.2	1.6	AA507111 nh41e06.s1 NCI_CGAP_Pr5 cDNA clone IMAGE:954946, mRNA sequence	AA507111	Hs.529023	NM_018181
MMS19L	-4.2	1.7	MMS19-like (MET18 homolog, S. cerevisiae) (MMS19L), mRNA	NM_022362	Hs.500721	AF319947
STAT1	-4.2	1.7	signal transducer and activator of transcription 1, 91kDa (STAT1), transcript variant beta, mRNA	NM_139266	Hs.699271	NM_007315
AK098160	-4.2	1.9	cDNA FLJ40841 fis, clone TRACH2014082.	AK098160	Unknown	
BC042649	-4.2	1.6	cDNA clone IMAGE:4826012.	BC042649	Hs.538511	AK126039
A_32_P196669	-4.2	2.7	A_32_P196669	A_32_P196669	Unknown	
DKFZP564J0863	-4.2	2.5	DKFZP564J0863 protein (DKFZP564J0863), mRNA	NM_015459	Hs.356719	CR936784
PLCG2	-4.2	2.3	phospholipase C, gamma 2 (phosphatidylinositol-specific) (PLCG2), mRNA	NM_002661	Hs.413111	NM_002661
THC2526402	-4.2	2.0	Q504T5_HUMAN (Q504T5) UNC84B protein, partial (12%)	THC2526402	Unknown	
T35358	-4.2	2.8	T35358 EST83346 Human Platelet cDNA 3' end similar to similar to nucleolar protein B23.2, mRNA sequence	T35358	Hs.557550	BM541948
PPM1B	-4.2	2.2	protein phosphatase 1B (formerly 2C), magnesium-dependent, beta isoform (PPM1B), transcript variant 5, mRNA	NM_001033557	Hs.416769	NM_177968
TTLL6	-4.2	4.2	tubulin tyrosine ligase-like family, member 6 (TTLL6), mRNA	NM_173623	Hs.91930	BC041368
ARHGAP11A	-4.2	1.9	Rho GTPase activating protein 11A (ARHGAP11A), transcript variant 1, mRNA	NM_014783	Hs.591130	NM_014783
IVD	-4.2	3.3	isovaleryl Coenzyme A dehydrogenase (IVD), nuclear gene encoding mitochondrial protein, mRNA	NM_002225	Hs.513646	AK122922
ARF6	-4.2	2.4	ADP-ribosylation factor 6 (ARF6), mRNA	NM_001663	Hs.525330	NM_001663
C9orf24	-4.2	2.2	chromosome 9 open reading frame 24 (C9orf24), transcript variant 1, mRNA	NM_032596	Hs.50334	BM805429
AGPAT5	-4.2	2.2	1-acylglycerol-3-phosphate O-acyltransferase 5 (lysophosphatidic acid acyltransferase, epsilon) (AGPAT5), mRNA	NM_018361	Hs.624002	BX640918
PPP1CC	-4.2	1.5	protein phosphatase 1, catalytic subunit, gamma isoform (PPP1CC), mRNA	NM_002710	Hs.79081	NM_002710
ZNF195	-4.2	1.5	zinc finger protein 195 (ZNF195), mRNA	NM_007152	Hs.386294	AL833722
IGF1R	-4.2	2.3	insulin-like growth factor 1 receptor (IGF1R), mRNA	NM_000875	Hs.643120	NM_000875
BAK1	-4.2	2.3	BCL2-antagonist/killer 1 (BAK1), mRNA	NM_001188	Hs.485139	CR627020
TUBA1	-4.2	3.7	tubulin, alpha 1 (TUBA1), mRNA	NM_006000	Hs.75318	AK054731
SIRT5	-4.2	1.7	sirtuin (silent mating type information regulation 2 homolog) 5 (S. cerevisiae) (SIRT5), transcript variant 2, mRNA	NM_031244	Hs.567431	BC035196
HIST1H2BJ	-4.2	3.9	histone cluster 1, H2bj (HIST1H2BJ), mRNA	NM_021058	Hs.656567	BF983642
ELAC2	-4.2	1.6	elaC homolog 2 (E. coli) (ELAC2), mRNA	NM_018127	Hs.434232	AK125030
THC2556482	-4.2	1.8	ALU1_HUMAN (P39188) Alu subfamily J sequence contamination warning entry, partial (6%)	THC2556482	Unknown	
HAP1	-4.2	1.5	huntingtin-associated protein 1 (neuroan 1) (HAP1), transcript variant 1, mRNA	NM_003949	Hs.158300	AB209105
NOC3L	-4.2	2.0	nucleolar complex associated 3 homolog (S. cerevisiae) (NOC3L), mRNA	NM_022451	Hs.74899	BC049850
KCTD14	-4.2	4.5	potassium channel tetramerisation domain containing 14 (KCTD14), mRNA	NM_023930	Hs.407860	NM_004549
FCRL5	-4.2	3.5	Fc receptor-like 5 (FCRL5), mRNA	NM_031281	Hs.415950	AF369794
RAB3B	-4.2	3.7	RAB3B, member RAS oncogene family (RAB3B), mRNA	NM_002867	Hs.123072	AK002107
PODXL	-4.2	2.8	podocalyxin-like (PODXL), transcript variant 1, mRNA	NM_001018111	Hs.16426	BX641124
AK094972	-4.2	11.4	cDNA FLJ37653 fis, clone BRHIP2010217.	AK094972	Hs.130661	XM_496115
TNPO1	-4.2	2.8	transportin 1 (TNPO1), transcript variant 1, mRNA	NM_002270	Hs.482497	NM_002270

ACACA	-4.2	1.8	acetyl-Coenzyme A carboxylase alpha (ACACA), transcript variant 2, mRNA	NM_198839	Hs.160556	NM_198839
SLC27A5	-4.2	1.9	solute carrier family 27 (fatty acid transporter), member 5 (SLC27A5), mRNA	NM_012254	Hs.292177	AB208931
PRKCD	-4.2	2.1	protein kinase C, delta (PRKCD), transcript variant 1, mRNA	NM_006254	Hs.155342	NM_006254
BIRC5	-4.2	1.9	baculoviral IAP repeat-containing 5 (survivin) (BIRC5), transcript variant 3, mRNA	NM_001012271	Hs.514527	NM_001012271
ARL4A	-4.2	1.6	ADP-ribosylation factor-like 4A (ARL4A), transcript variant 1, mRNA	NM_005738	Hs.245540	NM_005738
MGC12935	-4.2	5.2	hypothetical protein MGC12935, mRNA (cDNA clone IMAGE:4309284), partial cds.	BC004565	Hs.247812	AW976335
G3BP2	-4.2	2.1	GTPase activating protein (SH3 domain) binding protein 2 (G3BP2), transcript variant 1, mRNA	NM_203505	Hs.303676	NM_203505
LILRB4	-4.2	1.6	leukocyte immunoglobulin-like receptor, subfamily B (with TM and ITIM domains), member 4 (LILRB4), mRNA	NM_006847	Hs.67846	BC026309
FRAG1	-4.2	1.5	FGF receptor activating protein 1 (FRAG1), mRNA	NM_014489	Hs.133968	AL049261
MASTL	-4.2	1.9	microtubule associated serine/threonine kinase-like (MASTL), mRNA	NM_032844	Hs.276905	AK123004
CNKSR1	-4.2	1.9	connector enhancer of kinase suppressor of Ras 1 (CNKSR1), mRNA	NM_006314	Hs.16232	BC012797
TMEM142A	-4.2	1.9	transmembrane protein 142A (TMEM142A), mRNA	NM_032790	Hs.55148	BM558579
PRKCA	-4.2	5.2	protein kinase C, alpha (PRKCA), mRNA	NM_002737	Hs.531704	NM_002737
HLA-DQA1	-4.2	2.4	major histocompatibility complex, class II, DQ alpha 1 (HLA-DQA1), mRNA	NM_002122	Hs.387679	AB209628
BX648591	-4.2	1.6	mRNA; cDNA DKFZp686G14198 (from clone DKFZp686G14198).	BX648591	Hs.143434	NM_001843
MAPK1	-4.2	2.5	mitogen-activated protein kinase 1 (MAPK1), transcript variant 2, mRNA	NM_138957	Hs.431850	AL157438
CCDC117	-4.2	2.2	coiled-coil domain containing 117 (CCDC117), mRNA	NM_173510	Hs.406460	NM_173510
CCNA2	-4.2	2.1	cyclin A2 (CCNA2), mRNA	NM_001237	Hs.58974	CR604810
NOC3L	-4.2	1.9	nucleolar complex associated 3 homolog (S. cerevisiae) (NOC3L), mRNA	NM_022451	Hs.74899	BC049850
LOC647298	-4.2	2.3	PREDICTED: similar to 60 kDa heat shock protein, mitochondrial precursor (Hsp60) (60 kDa chaperonin) (CPN60) (Heat shock protein 60) (HSP-60) (Mitochondrial matrix protein P1) (P60 lymphocyte protein) (HuCHA60) (LOC647298), mRNA	XR_018387	Hs.646624	XR_018387
ASB16	-4.2	1.5	ankyrin repeat and SOCS box-containing 16 (ASB16), mRNA	NM_080863	Hs.534517	AK054727
CCDC95	-4.2	1.6	coiled-coil domain containing 95 (CCDC95), mRNA	NM_173618	Hs.434864	AK074137
METT5D1	-4.2	1.7	methyltransferase 5 domain containing 1 (METT5D1), mRNA	NM_152636	Hs.243326	AK091298
M74509	-4.2	2.1	Human endogenous retrovirus type C oncovirus sequence.	M74509	Unknown	
C9orf122	-4.2	2.5	cDNA clone IMAGE:5288595.	BC036230	Hs.632652	BC036230
SLC16A1	-4.2	2.4	solute carrier family 16, member 1 (monocarboxylic acid transporter 1) (SLC16A1), mRNA	NM_003051	Hs.75231	NM_003051
CEP72	-4.2	1.8	centrosomal protein 72kDa (CEP72), mRNA	NM_018140	Hs.591741	NM_018140
THC2732721	-4.2	4.4	ARHG5_HUMAN (Q12774) Rho guanine nucleotide exchange factor 5 (Guanine nucleotide regulatory protein TIM) (Oncogene TIM) (p60 TIM) (Transforming immortalized mammary oncogene), partial (8%)	THC2732721	Unknown	
LRP8	-4.2	7.3	low density lipoprotein receptor-related protein 8, apolipoprotein e receptor (LRP8), transcript variant 2, mRNA	NM_033300	Hs.576154	NM_004631
C1orf163	-4.2	1.9	chromosome 1 open reading frame 163 (C1orf163), mRNA	NM_023077	Hs.584966	AK023237
ASRGL1	-4.2	3.2	asparaginase like 1 (ASRGL1), mRNA	NM_025080	Hs.535326	BX640832
NOC3L	-4.2	1.9	nucleolar complex associated 3 homolog (S. cerevisiae) (NOC3L), mRNA	NM_022451	Hs.74899	BC049850
PPP2R5A	-4.2	1.8	protein phosphatase 2, regulatory subunit B (B56), alpha isoform (PPP2R5A), mRNA	NM_006243	Hs.497684	NM_006243
DEFB4	-4.2	1.8	defensin, beta 4 (DEFB4), mRNA	NM_004942	Hs.105924	BF088093
AKNA	-4.2	2.5	AT-hook transcription factor (AKNA), mRNA	NM_030767	Hs.494895	NM_030767
RPGRIP1	-4.2	4.4	retinitis pigmentosa GTPase regulator interacting protein 1 (RPGRIP1), mRNA	NM_020366	Hs.126035	NM_020366
LHX6	-4.2	3.1	LIM homeobox 6 (LHX6), transcript variant 1, mRNA	NM_014368	Hs.103137	AK126982
ECT2	-4.2	1.6	epithelial cell transforming sequence 2 oncogene (ECT2), mRNA	NM_018098	Hs.518299	AY376439
DIP2B	-4.2	1.9	DIP2 disco-interacting protein 2 homolog B (Drosophila) (DIP2B), mRNA	NM_173602	Hs.505516	NM_173602
C12orf45	-4.2	1.8	chromosome 12 open reading frame 45 (C12orf45), mRNA	NM_152318	Hs.295563	BX447742
PUS7	-4.2	2.1	pseudouridylate synthase 7 homolog (S. cerevisiae) (PUS7), mRNA	NM_019042	Hs.520619	NM_019042
CD40	-4.2	2.5	CD40 molecule, TNF receptor superfamily member 5 (CD40), transcript variant 1, mRNA	NM_001250	Hs.472860	AB209660
FADS3	-4.2	2.4	fatty acid desaturase 3 (FADS3), mRNA	NM_021727	Hs.21765	AB209356
FGF12	-4.2	2.9	fibroblast growth factor 12 (FGF12), transcript variant 2, mRNA	NM_004113	Hs.584758	AK125307
AW972815	-4.2	37.6	EST384910 MAGE resequences, MAGL cDNA, mRNA sequence	AW972815	Hs.290255	AW972815
THC2661011	-4.2	8.0	Q3H5V5_9ACTO (Q3H5V5) Thiolase, partial (3%)	THC2661011	Unknown	
SIRT1	-4.2	2.1	sirtuin (silent mating type information regulation 2 homolog) 1 (S. cerevisiae) (SIRT1), mRNA	NM_012238	Hs.369779	NM_012238
HUWE1	-4.2	1.7	HECT, UBA and WWE domain containing 1 (HUWE1), mRNA	NM_031407	Hs.136905	DQ097177
MDH2	-4.2	2.0	cDNA FLJ46193 fis, clone TESTI4006234.	AK128072	Hs.689470	AK128072
PQLC3	-4.2	2.2	PQ loop repeat containing 3 (PQLC3), mRNA	NM_152391	Hs.274415	NM_152391
AA601031	-4.2	15.8	nk67d10.s1 NCI_CGAP_Sch1 cDNA clone IMAGE:1018579 3', mRNA sequence	AA601031	Hs.496631	AA601031
CYP2A13	-4.2	2.3	cytochrome P450, family 2, subfamily A, polypeptide 13 (CYP2A13), mRNA	NM_000766	Hs.567252	NM_000766
AK125299	-4.2	2.7	cDNA FLJ43309 fis, clone NT2RI2004618, highly similar to Cytosolic acyl coenzyme A thioester hydrolase (EC 3.1.2.2).	AK125299	Hs.534633	XR_000194

A_32_P7193	-4.2	2.7	A_32_P7193	A_32_P7193	Unknown	
PCCA	-4.2	1.6	propionyl Coenzyme A carboxylase, alpha polypeptide (PCCA), mRNA	NM_000282	Hs.80741	AL122056
ENST00000355	-4.2	9.4	Endogenous retrovirus H D1 leader region/integrase-derived ORF1, ORF2, and putative envelope protein (Endogenous retrovirus H protease/integrase-derived ORF1, ORF2, and putative envelope protein).	ENST00000355	Unknown	
TUBG1	-4.1	2.2	tubulin, gamma 1 (TUBG1), mRNA	NM_001070	Hs.279669	NM_001070
PPP6C	-4.1	2.0	protein phosphatase 6, catalytic subunit (PPP6C), mRNA	NM_002721	Hs.584019	BF664863
ADD2	-4.1	18.6	adducin 2 (beta) (ADD2), transcript variant beta-4, mRNA	NM_017488	Hs.188528	NM_017488
C16orf68	-4.1	2.2	chromosome 16 open reading frame 68 (C16orf68), mRNA	NM_024109	Hs.306380	AK000114
MYO1E	-4.1	5.3	Human myosin-IC mRNA, complete cds.	U14391	Hs.654506	BC098392
BIRC5	-4.1	1.9	baculoviral IAP repeat-containing 5 (survivin) (BIRC5), transcript variant 3, mRNA	NM_001012271	Hs.514527	NM_001012271
PTPRN	-4.1	2.9	protein tyrosine phosphatase, receptor type, N (PTPRN), mRNA	NM_002846	Hs.89655	AB209368
PDPN	-4.1	2.6	podoplanin (PDPN), transcript variant 2, mRNA	NM_198389	Hs.468675	NM_006474
FXYD5	-4.1	2.8	FXYD domain containing ion transport regulator 5 (FXYD5), transcript variant 1, mRNA	NM_144779	Hs.333418	BX648809
C3orf42	-4.1	1.9	chromosome 3 open reading frame 42 (C3orf42), mRNA	NM_001039102	Unknown	
HLA-DPA1	-4.1	1.8	major histocompatibility complex, class II, DP alpha 1 (HLA-DPA1), mRNA	NM_033554	Hs.347270	AB209058
MRPS10	-4.1	1.9	mitochondrial ribosomal protein S10 (MRPS10), nuclear gene encoding mitochondrial protein, mRNA	NM_018141	Hs.380887	AF113220
GART	-4.1	2.4	phosphoribosylglycinamide formyltransferase, phosphoribosylglycinamide synthetase, phosphoribosylaminoimidazole synthetase (GART), transcript variant 2, mRNA	NM_175085	Hs.473648	BC068438
CDK5R2	-4.1	1.4	cyclin-dependent kinase 5, regulatory subunit 2 (p39) (CDK5R2), mRNA	NM_003936	Hs.158460	BC041771
BCL2L12	-4.1	1.8	BCL2-like 12 (proline rich) (BCL2L12), transcript variant 1, mRNA	NM_138639	Hs.289052	NM_138639
LOC390203	-4.1	1.8	PREDICTED: similar to steroid 5 alpha-reductase 2-like (LOC390203), mRNA	XR_018470	Hs.647254	XR_018470
TTLL4	-4.1	2.5	tubulin tyrosine ligase-like family, member 4 (TTLL4), mRNA	NM_014640	Hs.471405	D79995
RNF39	-4.1	2.5	ring finger protein 39 (RNF39), transcript variant 1, mRNA	NM_025236	Hs.121178	NM_025236
ARHGEF10	-4.1	3.6	Rho guanine nucleotide exchange factor (GEF) 10, mRNA (cDNA clone IMAGE:4250879), complete cds.	BC040474	Hs.98594	CR749570
NOC3L	-4.1	1.9	nucleolar complex associated 3 homolog (S. cerevisiae) (NOC3L), mRNA	NM_022451	Hs.74899	BC049850
ZNF551	-4.1	1.7	zinc finger protein 551 (ZNF551), mRNA	NM_138347	Hs.656485	AK126625
GIT2	-4.1	2.0	G protein-coupled receptor kinase interactor 2 (GIT2), transcript variant 4, mRNA	NM_139201	Hs.434996	NM_057169
ADCY2	-4.1	2.6	adenylate cyclase 2 (brain) (ADCY2), mRNA	NM_020546	Hs.481545	CR749634
SIN3A	-4.1	1.3	SIN3 homolog A, transcription regulator (yeast) (SIN3A), mRNA	NM_015477	Hs.513039	NM_015477
RHO	-4.1	3.9	rhodopsin (opsin 2, rod pigment) (retinitis pigmentosa 4, autosomal dominant) (RHO), mRNA	NM_000539	Hs.247565	NM_000539
SYCP1	-4.1	1.6	synaptonemal complex protein 1 (SYCP1), mRNA	NM_003176	Hs.112743	NM_003176
ERICH1	-4.1	1.9	glutamate-rich 1 (ERICH1), mRNA	NM_207332	Hs.655310	BX647093
HTR1B	-4.1	1.6	5-hydroxytryptamine (serotonin) receptor 1B (HTR1B), mRNA	NM_000863	Hs.123016	M81590
NSUN5C	-4.1	2.6	NOL1/NOP2/Sun domain family, member 5C (NSUN5C), transcript variant 2, mRNA	NM_148936	Hs.510927	AL117583
NP186315	-4.1	1.9	GB|AF111850.1|AAF16689.1 PRO0593	NP186315	Unknown	
ZNF195	-4.1	1.5	zinc finger protein 195 (ZNF195), mRNA	NM_007152	Hs.386294	AL833722
RBM28	-4.1	1.6	RNA binding motif protein 28 (RBM28), mRNA	NM_018077	Hs.274263	AK001239
BC039021	-4.1	1.5	cDNA clone IMAGE:6043059, partial cds.	BC039021	Hs.55028	NM_001100624
MKLN1	-4.1	1.8	muskelin 1, intracellular mediator containing kelch motifs (MKLN1), mRNA	NM_013255	Hs.44693	NM_013255
PTPRJ	-4.1	3.9	protein tyrosine phosphatase, receptor type, J (PTPRJ), mRNA	NM_002843	Hs.318547	NM_002843
MCM10	-4.1	2.0	MCM10 minichromosome maintenance deficient 10 (S. cerevisiae) (MCM10), transcript variant 1, mRNA	NM_182751	Hs.198363	AL136840
POP7	-4.1	1.6	processing of precursor 7, ribonuclease P subunit (S. cerevisiae) (POP7), mRNA	NM_005837	Hs.416994	BU944802
A_32_P125808	-4.1	2.8	A_32_P125808	A_32_P125808	Unknown	
BIRC5	-4.1	1.9	baculoviral IAP repeat-containing 5 (survivin) (BIRC5), transcript variant 3, mRNA	NM_001012271	Hs.514527	NM_001012271
FAM126A	-4.1	4.0	family with sequence similarity 126, member A (FAM126A), mRNA	NM_032581	Hs.85603	AL833296
PFDN4	-4.1	2.1	prefoldin subunit 4 (PFDN4), mRNA	NM_002623	Hs.91161	NM_002623
ARNTL2	-4.1	8.0	cycle-like factor CLIF mRNA, complete cds.	AF256215	Unknown	
DBNDD2	-4.1	1.8	dysbindin (dystrobrevin binding protein 1) domain containing 2 (DBNDD2), transcript variant 6, mRNA	NM_001048226	Hs.655055	NM_001099791
BOLA2B	-4.1	1.9	bolA homolog 2B (E. coli) (BOLA2B), mRNA	NM_001039182	Hs.647333	NM_001039182
LOC388969	-4.1	3.5	hypothetical LOC388969 (LOC388969), mRNA	NM_001013649	Hs.699599	NM_001013649
TRA16	-4.1	1.8	TR4 orphan receptor associated protein TRA16 (TRA16), mRNA	NM_176880	Hs.694846	CR594611
HAS3	-4.1	19.1	hyaluronan synthase 3 (HAS3), transcript variant 1, mRNA	NM_005329	Hs.592069	NM_005329
POU2F3	-4.1	2.4	POU domain, class 2, transcription factor 3 (POU2F3), mRNA	NM_014352	Hs.227115	AF162715
MYO5A	-4.1	2.0	myosin VA (heavy chain 12, myoxin) (MYO5A), mRNA	NM_000259	Hs.21213	NM_000259
PTTG1	-4.1	1.7	pituitary tumor-transforming 1 (PTTG1), mRNA	NM_004219	Hs.350966	BE904476
MAPK1	-4.1	2.5	mitogen-activated protein kinase 1 (MAPK1), transcript variant 2, mRNA	NM_138957	Hs.431850	AL157438

KIAA0828	-4.1	1.9	adenosylhomocysteinase 3 (KIAA0828), mRNA	NM_015328	Hs.600789	BC024325
KNDC1	-4.1	4.3	mRNA for FLJ00252 protein.	AK074179	Unknown	
SULT4A1	-4.1	2.5	sulfotransferase family 4A, member 1 (SULT4A1), mRNA	NM_014351	Hs.189810	BC030665
C6orf107	-4.1	2.8	UHRF1-binding protein 1 (Ubiquitin-like containing PHD and RING finger domains 1-binding protein 1) (ICBP90-binding protein 1).	ENST00000192	Unknown	
TMCO3	-4.1	1.8	transmembrane and coiled-coil domains 3 (TMCO3), mRNA	NM_017905	Hs.317593	BC068515
TMEM48	-4.1	2.2	transmembrane protein 48 (TMEM48), mRNA	NM_018087	Hs.476525	AL354613
ALOX12	-4.1	2.1	arachidonate 12-lipoxygenase (ALOX12), mRNA	NM_000697	Hs.654431	NM_000697
C1orf115	-4.1	1.9	chromosome 1 open reading frame 115 (C1orf115), mRNA	NM_024709	Hs.519839	AK125403
CDCP1	-4.1	3.7	CUB domain containing protein 1 (CDCP1), transcript variant 2, mRNA	NM_178181	Hs.476093	NM_022842
CRB3	-4.1	2.2	crumbs homolog 3 (Drosophila) (CRB3), transcript variant 3, mRNA	NM_174881	Hs.150319	AY103469
THC2578835	-4.1	2.0	THC2578835	THC2578835	Unknown	
KLHL26	-4.1	1.8	kelch-like 26 (Drosophila) (KLHL26), mRNA	NM_018316	Hs.250632	BC026319
LOC595101	-4.1	4.0	mRNA; cDNA DKFZp686H21113 (from clone DKFZp686H21113).	CR627362	Hs.654650	CR627362
ANK2	-4.1	2.5	ankyrin 2, neuronal (ANK2), transcript variant 1, mRNA	NM_001148	Hs.620557	NM_001148
CHST8	-4.1	2.5	carbohydrate (N-acetylgalactosamine 4-0) sulfotransferase 8 (CHST8), mRNA	NM_022467	Hs.165724	NM_022467
APEH	-4.1	2.6	N-acylaminoacyl-peptide hydrolase (APEH), mRNA	NM_001640	Hs.517969	BC000362
RNF10	-4.1	1.8	ring finger protein 10 (RNF10), mRNA	NM_014868	Hs.442798	NM_014868
ATXN7L3	-4.1	1.9	ataxin 7-like 3, mRNA (cDNA clone IMAGE:5499446), partial cds.	BC037418	Hs.512651	NM_020218
GMFB	-4.1	2.4	glia maturation factor, beta (GMFB), mRNA	NM_004124	Hs.151413	BX647679
YARS2	-4.1	1.7	tyrosyl-tRNA synthetase 2 (mitochondrial) (YARS2), mRNA	NM_001040436	Hs.505231	NM_001040436
P18SRP	-4.1	1.5	P18SRP protein (P18SRP), mRNA	NM_173829	Hs.656180	NM_173829
KIAA0406	-4.1	2.7	KIAA0406 (KIAA0406), mRNA	NM_014657	Hs.655481	BC013121
LOC220594	-4.1	2.2	TL132 protein (LOC220594), mRNA	NM_145809	Unknown	
CDCA4	-4.1	2.0	cell division cycle associated 4 (CDCA4), transcript variant 13, mRNA	NM_017955	Hs.34045	BG354577
CRLF3	-4.1	2.3	cytokine receptor-like factor 3 (CRLF3), mRNA	NM_015986	Unknown	
LOC644051	-4.1	2.1	PREDICTED: similar to Isocitrate dehydrogenase cytoplasmic (Cytosolic NADP-isocitrate dehydrogenase) (Oxalosuccinate decarboxylase) (IDH) (NADP(+)-specific ICDH) (IDP) (LOC649122), mRNA	XR_018565	Hs.646711	XR_018565
C9orf61	-4.1	2.3	chromosome 9 open reading frame 61 (C9orf61), mRNA	NM_004816	Hs.118003	AK126127
STAT3	-4.1	2.3	signal transducer and activator of transcription 3 (acute-phase response factor) (STAT3), transcript variant 3, mRNA	NM_213662	Hs.463059	NM_139276
TDP1	-4.1	1.9	tyrosyl-DNA phosphodiesterase 1 (TDP1), transcript variant 1, mRNA	NM_018319	Hs.209945	NM_018319
C20orf54	-4.1	12.4	chromosome 20 open reading frame 54 (C20orf54), mRNA	NM_033409	Hs.283865	AK074650
C19orf50	-4.1	1.9	chromosome 19 open reading frame 50 (C19orf50), mRNA	NM_024069	Hs.696088	AK091145
SLC6A6	-4.1	3.7	solute carrier family 6 (neurotransmitter transporter, taurine), member 6 (SLC6A6), mRNA	NM_003043	Hs.529488	NM_003043
RAB11FIP4	-4.1	2.8	RAB11 family interacting protein 4 (class II) (RAB11FIP4), mRNA	NM_032932	Hs.406788	NM_032932
CPSF2	-4.1	1.7	cleavage and polyadenylation specific factor 2, 100kDa (CPSF2), mRNA	NM_017437	Hs.657632	NM_017437
EDA	-4.1	2.1	ectodysplasin A (EDA), transcript variant 1, mRNA	NM_001399	Hs.105407	AF040628
THC2555723	-4.1	1.6	Q53FV0_HUMAN (Q53FV0) Prohibitin variant (Fragment), partial (81%)	THC2555723	Unknown	
C17orf27	-4.1	1.8	chromosome 17 open reading frame 27 (C17orf27), mRNA	NM_020914	Hs.195642	NM_020914
STRN4	-4.1	1.6	striatin, calmodulin binding protein 4 (STRN4), transcript variant 2, mRNA	NM_001039877	Hs.631590	NM_001039877
UNC5A	-4.1	1.8	unc-5 homolog A (C. elegans) (UNC5A), mRNA	NM_133369	Hs.33191	NM_133369
CRYGD	-4.1	5.9	crystallin, gamma D (CRYGD), mRNA	NM_006891	Hs.546247	CD049752
GAL3ST1	-4.1	2.3	galactose-3-O-sulfotransferase 1 (GAL3ST1), mRNA	NM_004861	Hs.17958	NM_004861
SLC2A14	-4.1	3.0	solute carrier family 2 (facilitated glucose transporter), member 14, mRNA (cDNA clone MGC:71510 IMAGE:5297510), complete cds.	BC060766	Hs.655169	AK126026
GNL3L	-4.1	2.9	guanine nucleotide binding protein-like 3 (nucleolar)-like (GNL3L), mRNA	NM_019067	Hs.654677	NM_019067
NUP37	-4.1	1.6	nucleoporin 37kDa (NUP37), mRNA	NM_024057	Hs.444276	BU535474
STAT1	-4.1	1.6	signal transducer and activator of transcription 1, 91kDa (STAT1), transcript variant beta, mRNA	NM_139266	Hs.699271	NM_007315
WRN	-4.1	1.4	Werner syndrome (WRN), mRNA	NM_000553	Hs.632050	NM_000553
FRMD5	-4.1	2.9	FERM domain containing 5 (FRMD5), transcript variant 2, mRNA	NM_032892	Hs.578544	BC007796
GPC4	-4.1	5.3	glypican 4 (GPC4), mRNA	NM_001448	Hs.58367	AF030186
ALDH1B1	-4.1	2.5	aldehyde dehydrogenase 1 family, member B1 (ALDH1B1), nuclear gene encoding mitochondrial protein, mRNA	NM_000692	Hs.436219	NM_000692
AK094156	-4.1	1.7	cDNA FLJ36837 fis, clone ASTRO2011422.	AK094156	Hs.655890	AK094156
THC2646867	-4.1	2.1	Q99309_HAEIN (Q99309) Lipopolysaccharide core (lic1) locus. (Fragment), partial (45%)	THC2646867	Unknown	
FABP5	-4.1	1.9	fatty acid binding protein 5 (psoriasis-associated) (FABP5), mRNA	NM_001444	Hs.408061	BM563703
EHD4	-4.1	2.7	EH-domain containing 4 (EHD4), mRNA	NM_139265	Hs.143703	BC006287
CCNA2	-4.1	2.0	cyclin A2 (CCNA2), mRNA	NM_001237	Hs.58974	CR604810

A_32_P55438	-4.1	4.5	A_32_P55438	A_32_P55438	Unknown	
BIRC5	-4.1	1.9	baculoviral IAP repeat-containing 5 (survivin) (BIRC5), transcript variant 3, mRNA	NM_001012271	Hs.514527	NM_001012271
FLJ35024	-4.1	5.7	unknown mRNA.	AF424541	Unknown	
ATE1	-4.1	1.6	arginyltransferase 1 (ATE1), transcript variant 1, mRNA	NM_001001976	Hs.632080	AK124669
A_24_P853366	-4.1	3.0	A_24_P853366	A_24_P853366	Unknown	
ST8SIA3	-4.1	4.7	ST8 alpha-N-acetyl-neuraminide alpha-2,8-sialyltransferase 3 (ST8SIA3), mRNA	NM_015879	Hs.23172	NM_015879
CHEK1	-4.1	1.8	CHK1 checkpoint homolog (S. pombe) (CHEK1), mRNA	NM_001274	Hs.24529	BC017575
POLR2D	-4.1	2.5	polymerase (RNA) II (DNA directed) polypeptide D (POLR2D), mRNA	NM_004805	Hs.700670	BC017205
FAM33A	-4.1	1.4	family with sequence similarity 33, member A (FAM33A), mRNA	NM_182620	Hs.463607	NM_182620
FAM100A	-4.1	1.9	family with sequence similarity 100, member A (FAM100A), mRNA	NM_145253	Hs.513313	AK123636
OXCT2	-4.1	2.7	mRNA for FLJ00030 protein, partial cds.	AK024440	Hs.472491	AK024440
THC2524570	-4.1	3.7	Q86TZ0_HUMAN (Q86TZ0) Full-length cDNA clone CS0DC023YN15 of Neuroblastoma of (human) (Fragment), partial (85%)	THC2524570	Unknown	
C9orf86	-4.1	1.5	pp8875 mRNA, complete cds.	AF318367	Unknown	
CHORDC1	-4.1	1.7	cysteine and histidine-rich domain (CHORD)-containing 1 (CHORDC1), mRNA	NM_012124	Hs.22857	BX537692
A_23_P40290	-4.1	7.4	A_23_P40290	A_23_P40290	Unknown	
ENST00000333[illegible]	-4.1	1.6	C21orf86 protein (C21orf86) mRNA, complete cds.	ENST00000333[illegible]	Unknown	
MARK2	-4.1	1.7	MAP/microtubule affinity-regulating kinase 2 (MARK2), transcript variant 1, mRNA	NM_017490	Hs.567261	AB188493
MTMR12	-4.1	3.3	myotubularin related protein 12 (MTMR12), mRNA	NM_001040446	Hs.481836	NM_001040446
PPOX	-4.1	1.8	protoporphyrinogen oxidase (PPOX), nuclear gene encoding mitochondrial protein, mRNA	NM_000309	Hs.517373	AK094855
CCNB1	-4.1	1.7	cyclin B1 (CCNB1), mRNA	NM_031966	Hs.23960	NM_031966
AP3S2	-4.1	1.4	adaptor-related protein complex 3, sigma 2 subunit (AP3S2), mRNA	NM_005829	Hs.632161	NM_005829
15E1.2	-4.1	2.2	hypothetical protein LOC283459 (15E1.2), mRNA	NM_176818	Hs.369624	BC034962
CCNA2	-4.1	2.1	cyclin A2 (CCNA2), mRNA	NM_001237	Hs.58974	CR604810
CREB3L2	-4.1	2.7	cAMP responsive element binding protein 3-like 2, mRNA (cDNA clone IMAGE:4185677), complete cds.	BC063666	Hs.490273	NM_194071
BUB1B	-4.1	2.4	BUB1 budding uninhibited by benzimidazoles 1 homolog beta (yeast) (BUB1B), mRNA	NM_001211	Hs.631699	AF053306
A_24_P340866	-4.1	1.8	A_24_P340866	A_24_P340866	Unknown	
HLA-DMA	-4.1	2.1	major histocompatibility complex, class II, DM alpha (HLA-DMA), mRNA	NM_006120	Hs.351279	AK055186
AY358690	-4.1	2.7	clone DNA62876 LPPA601 (UNQ601) mRNA, complete cds.	AY358690	Hs.686554	AY358690
THC2530030	-4.1	2.2	GCSH_HUMAN (P23434) Glycine cleavage system H protein, mitochondrial precursor, partial (80%)	THC2530030	Unknown	
NFKB2	-4.1	2.1	nuclear factor of kappa light polypeptide gene enhancer in B-cells 2 (p49/p100) (NFKB2), transcript variant 3, mRNA	NM_001077493	Hs.73090	NM_001077494
ECHDC3	-4.1	6.2	enoyl Coenzyme A hydratase domain containing 3 (ECHDC3), mRNA	NM_024693	Hs.22242	AF289604
PKMYT1	-4.1	2.8	protein kinase, membrane associated tyrosine/threonine 1 (PKMYT1), transcript variant 1, mRNA	NM_004203	Hs.77783	NM_182687
NAV2	-4.1	1.9	partial mRNA for steerin2 protein (STEERIN2 gene), alternative exon 1b.	AJ488203	Unknown	
SLC6A6	-4.1	9.1	solute carrier family 6 (neurotransmitter transporter, taurine), member 6 (SLC6A6), mRNA	NM_003043	Hs.529488	NM_003043
TAS2R3	-4.1	2.6	taste receptor, type 2, member 3 (TAS2R3), mRNA	NM_016943	Hs.676011	BC095523
CB852269	-4.1	1.7	CB852269 UI-CF-FN0-afm-c-12-0-UI.s1 UI-CF-FN0 cDNA clone UI-CF-FN0-afm-c-12-0-UI 3', mRNA sequence	CB852269	Hs.661088	AK023844
PPAPDC1B	-4.1	3.3	phosphatidic acid phosphatase type 2 domain containing 1B (PPAPDC1B), mRNA	NM_032483	Hs.567619	BC033025
WFDC10B	-4.0	4.8	WAP four-disulfide core domain 10B (WFDC10B), transcript variant 1, mRNA	NM_172006	Hs.237392	NM_172006
FLJ14712	-4.0	7.7	cDNA PSEC0224 fis, clone HEMBA1005703, weakly similar to UROMODULIN PRECURSOR.	AK075525	Hs.669526	AK027618
LOC392473	-4.0	2.3	PREDICTED: similar to Phosphoglycerate mutase 1 (Phosphoglycerate mutase isozyme B) (PGAM-B) (BPG-dependent PGAM 1) (LOC392473), mRNA	XR_018640	Hs.646613	XR_018640
SNX21	-4.0	1.7	sorting nexin family member 21 (SNX21), transcript variant 4, mRNA	NM_001042633	Hs.472854	NM_001042633
PTPN6	-4.0	2.6	protein tyrosine phosphatase, non-receptor type 6 (PTPN6), transcript variant 1, mRNA	NM_002831	Hs.63489	NM_002831
ZNF331	-4.0	2.7	zinc finger protein 331 (ZNF331), mRNA	NM_018555	Hs.185674	CR749560
NOXO1	-4.0	5.3	NADPH oxidase organizer 1 (NOXO1), transcript variant a, mRNA	NM_144603	Hs.191762	AK096738
CHDH	-4.0	2.7	cDNA FLJ30840 fis, clone FEBRA2002442, highly similar to partial mRNA for choline dehydrogenase.	AK055402	Hs.126688	AK055402
NFKBIB	-4.0	2.3	nuclear factor of kappa light polypeptide gene enhancer in B-cells inhibitor, beta (NFKBIB), transcript variant 1, mRNA	NM_002503	Hs.9731	NM_001001716
LOC440895	-4.0	2.3	cDNA FLJ13403 fis, clone PLACE1001517, moderately similar to gene for glycosylphosphatidylinositol anchor attachment 1 (GPAA1).	AK023465	Hs.372946	AL133596
PAPD1	-4.0	1.6	PAP associated domain containing 1 (PAPD1), mRNA	NM_018109	Hs.173946	NM_018109
TMEM110	-4.0	3.3	transmembrane protein 110 (TMEM110), mRNA	NM_198563	Hs.700760	BX648562
LOC595101	-4.0	3.7	mRNA; cDNA DKFZp686H21113 (from clone DKFZp686H21113).	CR627362	Hs.654650	CR627362
CR749856	-4.0	1.9	mRNA; cDNA DKFZp781I1252 (from clone DKFZp781I1252).	CR749856	Unknown	
OAZ1	-4.0	1.6	ornithine decarboxylase antizyme 1 (OAZ1), mRNA	NM_004152	Hs.446427	BC035151
MOSPD2	-4.0	3.1	motile sperm domain containing 2 (MOSPD2), mRNA	NM_152581	Hs.190043	AL834345

GRID2	-4.0	6.6	mRNA for glutamate receptor, ionotropic, delta 2 variant protein.	AB209318	Hs.480281	AB209318
TSR1	-4.0	2.8	TSR1, 20S rRNA accumulation, homolog (S. cerevisiae) (TSR1), mRNA	NM_018128	Hs.388170	NM_018128
KIAA1754L	-4.0	1.6	KIAA1754-like (KIAA1754L), transcript variant 1, mRNA	NM_178495	Hs.65009	BC034503
HCP5	-4.0	1.9	HLA complex P5 (HCP5), mRNA	NM_006674	Hs.654480	L06175
MYH2	-4.0	3.9	myosin, heavy chain 2, skeletal muscle, adult (MYH2), mRNA	NM_017534	Hs.440895	NM_017534
LONP1	-4.0	1.7	lon peptidase 1, mitochondrial (LONP1), nuclear gene encoding mitochondrial protein, mRNA	NM_004793	Hs.350265	U02389
BC030138	-4.0	2.0	cDNA clone IMAGE:4335164, partial cds.	BC030138	Hs.356467	AK128435
SLC25A38	-4.0	2.1	solute carrier family 25, member 38 (SLC25A38), mRNA	NM_017875	Hs.369615	AK000558
FAM73B	-4.0	1.9	family with sequence similarity 73, member B (FAM73B), mRNA	NM_032809	Hs.632693	AK074127
DKFZp434N035	-4.0	2.9	hypothetical protein DKFZp434N035 (DKFZp434N035), mRNA	NM_032262	Unknown	
MFGE8	-4.0	4.1	milk fat globule-EGF factor 8 protein (MFGE8), mRNA	NM_005928	Hs.3745	BF345974
BEST2	-4.0	2.3	bestrophin 2 (BEST2), mRNA	NM_017682	Hs.435611	NM_017682
ANKRD43	-4.0	1.6	ankyrin repeat domain 43 (ANKRD43), mRNA	NM_175873	Hs.13308	NM_175873
TOX	-4.0	2.5	thymus high mobility group box protein TOX (TOX), mRNA	NM_014729	Hs.491805	BX647462
CECR6	-4.0	2.1	cat eye syndrome chromosome region, candidate 6 (CECR6), mRNA	NM_031890	Hs.209577	AF307451
CCNA2	-4.0	2.1	cyclin A2 (CCNA2), mRNA	NM_001237	Hs.58974	CR604810
AK093229	-4.0	2.7	cDNA FLJ35910 fis, clone TESTI2009987.	AK093229	Hs.586723	AK093229
SLC12A9	-4.0	2.0	solute carrier family 12 (potassium/chloride transporters), member 9 (SLC12A9), mRNA	NM_020246	Hs.521087	AK024466
BIRC5	-4.0	1.9	baculoviral IAP repeat-containing 5 (survivin) (BIRC5), transcript variant 3, mRNA	NM_001012271	Hs.514527	NM_001012271
ACADSB	-4.0	3.8	acyl-Coenzyme A dehydrogenase, short/branched chain (ACADSB), nuclear gene encoding mitochondrial protein, mRNA	NM_001609	Hs.81934	NM_001609
G3BP2	-4.0	1.4	GTPase activating protein (SH3 domain) binding protein 2 (G3BP2), transcript variant 1, mRNA	NM_203505	Hs.303676	NM_203505
PECR	-4.0	1.7	peroxisomal trans-2-enoyl-CoA reductase (PECR), mRNA	NM_018441	Hs.281680	NM_018000
VGF	-4.0	2.0	VGF nerve growth factor inducible (VGF), mRNA	NM_003378	Hs.587325	BC063835
A_32_P205792	-4.0	2.1	A_32_P205792	A_32_P205792	Unknown	
BC013025	-4.0	2.3	cDNA clone IMAGE:3528660, **** WARNING: chimeric clone ****.	BC013025	Unknown	
SLC4A11	-4.0	11.1	solute carrier family 4, sodium bicarbonate transporter-like, member 11 (SLC4A11), mRNA	NM_032034	Hs.105607	AF336127
THC2541607	-4.0	1.5	ALU8_HUMAN (P39195) Alu subfamily SX sequence contamination warning entry, partial (8%)	THC2541607	Unknown	
C13orf33	-4.0	5.3	chromosome 13 open reading frame 33 (C13orf33), mRNA	NM_032849	Hs.646647	NM_032849
AQP5	-4.0	2.2	aquaporin 5, mRNA (cDNA clone MGC:33163 IMAGE:5269384), complete cds.	BC032946	Hs.298023	BM559624
TUSC2	-4.0	2.1	tumor suppressor candidate 2 (TUSC2), mRNA	NM_007275	Hs.517981	AF055479
THC2628099	-4.0	1.6	Q4J4M9_AZOVI (Q4J4M9) Phosphotransferase system PTS, fructose-specific IIB subunit:PTS fructose IIC component, partial (3%)	THC2628099	Unknown	
C7orf43	-4.0	1.7	chromosome 7 open reading frame 43 (C7orf43), mRNA	NM_018275	Hs.533139	NM_018275
BTBD12	-4.0	1.9	BTB (POZ) domain containing 12 (BTBD12), mRNA	NM_032444	Hs.143681	NM_032444
NFKBIB	-4.0	2.8	nuclear factor of kappa light polypeptide gene enhancer in B-cells inhibitor, beta (NFKBIB), transcript variant 2, mRNA	NM_001001716	Hs.9731	NM_001001716
GNA12	-4.0	2.4	guanine nucleotide binding protein (G protein) alpha 12 (GNA12), mRNA	NM_007353	Hs.487341	NM_007353
ANKRD27	-4.0	1.8	ankyrin repeat domain 27 (VPS9 domain) (ANKRD27), mRNA	NM_032139	Hs.59236	AL136784
CENPN	-4.0	1.7	centromere protein N (CENPN), mRNA	NM_018455	Hs.55028	NM_001100624
AK092577	-4.0	1.6	cDNA FLJ35258 fis, clone PROST2004146.	AK092577	Hs.585907	AK092577
THC2671036	-4.0	1.8	Q6C1M3_YARLI (Q6C1M3) Similarity, partial (6%)	THC2671036	Unknown	
A_24_P273014	-4.0	2.2	A_24_P273014	A_24_P273014	Unknown	
SEMA6A	-4.0	4.5	sema domain, transmembrane domain (TM), and cytoplasmic domain, (semaphorin) 6A (SEMA6A), mRNA	NM_020796	Hs.156967	BC032619
DOLPP1	-4.0	1.7	dolichyl pyrophosphate phosphatase 1 (DOLPP1), mRNA	NM_020438	Hs.21701	NM_020438
DDR1	-4.0	2.8	discoidin domain receptor family, member 1 (DDR1), transcript variant 1, mRNA	NM_013993	Hs.631988	NM_013993
PRB3	-4.0	1.6	proline-rich protein BstNI subfamily 3 (PRB3), mRNA	NM_006249	Hs.73031	NM_006249
MFSD3	-4.0	4.3	major facilitator superfamily domain containing 3 (MFSD3), mRNA	NM_138431	Hs.7678	BM548439
LOC120449	-4.0	2.9	PREDICTED: similar to C-terminal-binding protein 2 (CtBP2) (LOC120449), mRNA	XR_019486	Hs.647303	XR_019486
GPR120	-4.0	1.6	G protein-coupled receptor 120 (GPR120), mRNA	NM_181745	Hs.677835	BX647721
WWC2	-4.0	3.3	WW and C2 domain containing 2 (WWC2), mRNA	NM_024949	Hs.333179	BX647378
MFSD2	-4.0	2.0	cDNA FLJ14490 fis, clone MAMMA1002886.	AK027396	Unknown	
TOB2	-4.0	2.6	transducer of ERBB2, 2 (TOB2), mRNA	NM_016272	Hs.474978	NM_016272
CALN1	-4.0	6.5	calneuron 1 (CALN1), transcript variant 1, mRNA	NM_031468	Hs.333274	NM_031468
ZNF462	-4.0	3.2	mRNA; cDNA DKFZp762N2316 (from clone DKFZp762N2316).	AL359561	Hs.370379	BX648965
CCNA2	-4.0	2.1	cyclin A2 (CCNA2), mRNA	NM_001237	Hs.58974	CR604810
SNHG7	-4.0	2.7	cDNA FLJ33266 fis, clone ASTRO2007047.	AK090585	Hs.636619	AK054908

LOC146439	-4.0	5.7	mRNA; cDNA DKFZp666L166 (from clone DKFZp666L166).	AL833749	Hs.513285	XM_085463
PRR12	-4.0	2.1	mRNA for KIAA1205 protein, partial cds.	AB033031	Hs.590971	NM_020719
EPB41L4B	-4.0	6.7	erythrocyte membrane protein band 4.1 like 4B (EPB41L4B), transcript variant 1, mRNA	NM_018424	Hs.591901	AF153416
FLJ20273	-4.0	2.9	RNA-binding protein (FLJ20273), mRNA	NM_019027	Hs.518727	NM_001098634
THC2776730	-4.0	3.7	Q44ZN8_9BURK (Q44ZN8) Acyl-CoA dehydrogenase, C-terminal:Acyl-CoA dehydrogenase, central region, partial (3%)	THC2776730	Unknown	
TRIM14	-4.0	2.0	tripartite motif-containing 14 (TRIM14), transcript variant 2, mRNA	NM_033219	Hs.575631	NM_014788
ING5	-4.0	2.6	inhibitor of growth family, member 5 (ING5), mRNA	NM_032329	Hs.645460	NM_032329
NFS1	-4.0	1.6	NFS1 nitrogen fixation 1 homolog (S. cerevisiae) (NFS1), nuclear gene encoding mitochondrial protein, mRNA	NM_021100	Hs.194692	AK056242
PPP2R3B	-4.0	1.7	protein phosphatase 2 (formerly 2A), regulatory subunit B'', beta (PPP2R3B), transcript variant 1, mRNA	NM_013239	Hs.124942	AB209137
ACAD10	-4.0	2.4	acyl-Coenzyme A dehydrogenase family, member 10 (ACAD10), mRNA	NM_025247	Hs.331141	AL832043
AK022594	-4.0	2.4	cDNA FLJ12532 fis, clone NT2RM4000200.	AK022594	Hs.666219	BX640973
LRRC8E	-4.0	2.6	leucine rich repeat containing 8 family, member E (LRRC8E), mRNA	NM_025061	Hs.501511	NM_025061
PTPN6	-4.0	2.7	protein tyrosine phosphatase, non-receptor type 6 (PTPN6), transcript variant 1, mRNA	NM_002831	Hs.63489	NM_002831
WRN	-4.0	1.4	Werner syndrome (WRN), mRNA	NM_000553	Hs.632050	NM_000553
SERINC5	-4.0	2.8	serine incorporator 5 (SERINC5), mRNA	NM_178276	Hs.655558	CR936687
MAPK1	-4.0	2.4	mitogen-activated protein kinase 1 (MAPK1), transcript variant 2, mRNA	NM_138957	Hs.431850	AL157438
CHES1	-4.0	2.4	checkpoint suppressor 1 (CHES1), mRNA	NM_005197	Hs.434286	NM_005197
BFAR	-4.0	2.0	bifunctional apoptosis regulator (BFAR), mRNA	NM_016561	Hs.435556	AF173003
GNPTAB	-4.0	4.1	N-acetylglucosamine-1-phosphate transferase, alpha and beta subunits (GNPTAB), mRNA	NM_024312	Hs.46850	AM085438
POLD1	-4.0	1.5	polymerase (DNA directed), delta 1, catalytic subunit 125kDa (POLD1), mRNA	NM_002691	Hs.279413	AB209560
PISD	-4.0	2.1	phosphatidylserine decarboxylase (PISD), mRNA	NM_014338	Hs.420559	NM_014338
HUWE1	-4.0	1.6	HECT, UBA and WWE domain containing 1 (HUWE1), mRNA	NM_031407	Hs.136905	DQ097177
BE702584	-4.0	2.0	BE702584 RC5-NN1065-140600-033-G11 NN1065 cDNA, mRNA sequence	BE702584	Hs.650822	AB018345
BIRC5	-4.0	1.9	baculoviral IAP repeat-containing 5 (survivin) (BIRC5), transcript variant 3, mRNA	NM_001012271	Hs.514527	NM_001012271
LOC646359	-4.0	14.5	PREDICTED: similar to telomeric repeat binding factor 1 isoform 2 (LOC646359), mRNA	ENST00000342	Unknown	
OXCT2	-4.0	7.5	3-oxoacid CoA transferase 2 (OXCT2), mRNA	NM_022120	Hs.472491	AK024440
PGM2L1	-4.0	2.1	phosphoglucomutase 2-like 1 (PGM2L1), mRNA	NM_173582	Hs.26612	NM_173582
PIK3CD	-4.0	2.5	phosphoinositide-3-kinase, catalytic, delta polypeptide (PIK3CD), mRNA	NM_005026	Hs.518451	NM_005026
MTMR9	-4.0	1.8	myotubularin related protein 9 (MTMR9), mRNA	NM_015458	Hs.591395	NM_015458
PCCB	-4.0	2.5	propionyl Coenzyme A carboxylase, beta polypeptide (PCCB), mRNA	NM_000532	Hs.63788	AB209009
ENST00000366	-4.0	4.6	Vasohibin-2 (Vasohibin-like protein).	ENST00000366	Unknown	
THC2651548	-4.0	2.0	PEBP_MOUSE (P70296) Phosphatidylethanolamine-binding protein (PEBP) (HCNPpp) , complete	THC2651548	Unknown	
FLYWCH1	-4.0	2.7	FLYWCH-type zinc finger 1 (FLYWCH1), transcript variant 2, mRNA	NM_020912	Hs.655321	NM_032296
NFE2L1	-4.0	2.9	nuclear factor (erythroid-derived 2)-like 1 (NFE2L1), mRNA	NM_003204	Hs.514284	AK090459
RASGRF2	-4.0	9.3	Ras protein-specific guanine nucleotide-releasing factor 2 (RASGRF2), mRNA	NM_006909	Hs.162129	CR749239
BCCIP	-4.0	1.7	BRCA2 and CDKN1A interacting protein (BCCIP), transcript variant B, mRNA	NM_078468	Hs.370292	AK092054
FGFR2	-4.0	1.8	fibroblast growth factor receptor 2 (bacteria-expressed kinase, keratinocyte growth factor receptor, craniofacial dysostosis 1, Crouzon syndrome, Pfeiffer syndrome, Jackson-Weiss syndrome) (FGFR2), transcript variant 2, mRNA	NM_022970	Hs.533683	NM_022970
TMEM34	-4.0	1.8	transmembrane protein 34 (TMEM34), mRNA	NM_018241	Hs.203896	AK001708
PFN1	-4.0	2.1	profilin 1 (PFN1), mRNA	NM_005022	Hs.494691	BM807538
PRKCBP1	-4.0	1.9	protein kinase C binding protein 1 (PRKCBP1), transcript variant 2, mRNA	NM_012408	Hs.446240	NM_183047
ENST00000265	-4.0	2.8	RBM27 protein (Fragment).	ENST00000265	Unknown	
PRKCDBP	-4.0	5.3	protein kinase C, delta binding protein (PRKCDBP), mRNA	NM_145040	Hs.434044	BQ073453
A_23_P254288	-4.0	1.7	A_23_P254288	A_23_P254288	Unknown	
DKFZp762E131	-4.0	2.5	hypothetical protein DKFZp762E1312 (DKFZp762E1312), mRNA	NM_018410	Hs.532968	NM_018410
ZDHHC23	-4.0	3.6	zinc finger, DHHC-type containing 23 (ZDHHC23), mRNA	NM_173570	Hs.21902	AK127260
C14orf122	-4.0	1.7	chromosome 14 open reading frame 122 (C14orf122), mRNA	NM_016049	Hs.271614	BQ054986
PTEN	-4.0	1.7	phosphatase and tensin homolog (mutated in multiple advanced cancers 1) (PTEN), mRNA	NM_000314	Hs.500466	NM_000314
A_24_P76008	-4.0	2.1	A_24_P76008	A_24_P76008	Unknown	
F11R	-4.0	2.9	F11 receptor (F11R), transcript variant 4, mRNA	NM_144503	Hs.517293	NM_144503
TCL1A	-4.0	2.2	T-cell leukemia/lymphoma 1A (TCL1A), mRNA	NM_021966	Hs.2484	BQ057554
DPP3	-4.0	3.5	dipeptidyl-peptidase 3 (DPP3), transcript variant 2, mRNA	NM_130443	Hs.502914	AL833475
STYK1	-4.0	2.9	serine/threonine/tyrosine kinase 1 (STYK1), mRNA	NM_018423	Hs.24979	NM_018423
DLEU7	-4.0	1.4	deleted in lymphocytic leukemia 7 (DLEU7) mRNA, complete cds.	AY357595	Hs.673860	BC035481

R38716	-4.0	2.9	yd03c01.s1 Soares infant brain 1NIB cDNA clone IMAGE:24639 3' similar to contains Alu repetitive element;, mRNA sequence	R38716	Hs.594548	R38716
PHF20	-4.0	1.7	PHD finger protein 20 (PHF20), mRNA	NM_016436	Hs.517044	BC150178
BI496440	-4.0	2.2	df125c12.w1 Morton Fetal Cochlea cDNA clone IMAGE:2538238 3', mRNA sequence	BI496440	Hs.700791	BI496440
AL137342	-4.0	2.0	mRNA; cDNA DKFZp761G1111 (from clone DKFZp761G1111).	AL137342	Hs.144197	NM_003360
A_24_P714707	-4.0	2.8	A_24_P714707	A_24_P714707	Unknown	
ZC3HAV1	-4.0	2.3	zinc finger CCCH-type, antiviral 1 (ZC3HAV1), transcript variant 1, mRNA	NM_020119	Hs.133512	NM_020119
ZYG11A	-4.0	8.0	ZYG-11A early embryogenesis protein.	ENST00000371[illegible]	Unknown	
ZNF195	-4.0	1.5	zinc finger protein 195 (ZNF195), mRNA	NM_007152	Hs.386294	AL833722
AA604115	-4.0	2.1	no72d07.s1 NCI_CGAP_AA1 cDNA clone IMAGE:1112365 3' similar to gb:L12693 CELLULAR NUCLEIC ACID BINDING PROTEIN (HUMAN);, mRNA sequence	AA604115	Hs.701027	AA604115
ABHD11	-4.0	1.5	abhydrolase domain containing 11 (ABHD11), transcript variant 4, mRNA	NM_031295	Unknown	
C9orf114	-4.0	2.4	chromosome 9 open reading frame 114 (C9orf114), mRNA	NM_016390	Hs.224137	AL110193

Supplemental Table A2

Genes Upregulated in EB vs ES (4407)

SAM analysis, FDR 0.1%

Gene Name	Score(d)	Fold Upreg. EB	Description	Acc	UGCluster	UGRepAcc
TWIST1	47.2	22.4	twist homolog 1 (acrocephalosyndactyly 3; Saethre-Chotzen syndrome) (Drosophila) (TWIST1), mRNA	NM_000474	Hs.66744	NM_000474
DNAJC12	34.9	12.4	DnaJ (Hsp40) homolog, subfamily C, member 12 (DNAJC12), transcript variant 1, mRNA	NM_021800	Hs.260720	NM_021800
FOXA1	32.2	17.3	forkhead box A1 (FOXA1), mRNA	NM_004496	Hs.163484	NM_004496
CR615016	30.8	36.1	full-length cDNA clone CS0DB006YM19 of Neuroblastoma Cot 10-normalized of (human).	CR615016	Hs.19193	BC009385
TBX2	27.5	45.7	T-box 2 (TBX2), mRNA	NM_005994	Hs.699297	AB209378
FOXA1	27.5	15.1	forkhead box A1 (FOXA1), mRNA	NM_004496	Hs.163484	NM_004496
LOC399959	27.4	31.4	cDNA FLJ38472 fis, clone FEBRA2022148.	AK095791	Hs.411391	AK095791
FGF9	26.4	11.8	fibroblast growth factor 9 (glia-activating factor) (FGF9), mRNA	NM_002010	Hs.111	D14838
FOXC1	26.4	52.0	forkhead box C1 (FOXC1), mRNA	NM_001453	Hs.348883	NM_001453
BCL2	26.0	7.7	B-cell CLL/lymphoma 2 (BCL2), nuclear gene encoding mitochondrial protein, transcript variant alpha, mRNA	NM_000633	Hs.150749	NM_000633
GAS2	25.2	14.0	growth arrest-specific 2 (GAS2), transcript variant 1, mRNA	NM_005256	Hs.632151	BC013326
AK021804	25.2	28.0	cDNA FLJ11742 fis, clone HEMBA1005508.	AK021804	Hs.411391	AK095791
ENST00000377548	24.1	7.9	Novel protein.	ENST00000377548	Unknown	
FOXA1	23.7	15.3	forkhead box A1 (FOXA1), mRNA	NM_004496	Hs.163484	NM_004496
FOXA1	23.5	19.6	forkhead box A1 (FOXA1), mRNA	NM_004496	Hs.163484	NM_004496
VWC2	23.2	26.4	von Willebrand factor C domain containing 2 (VWC2), mRNA	NM_198570	Hs.677488	AY358393
THC2665223	22.9	10.5	THC2665223	THC2665223	Unknown	
THC2609205	22.9	20.7	THC2609205	THC2609205	Unknown	
FOXA1	22.6	16.6	forkhead box A1 (FOXA1), mRNA	NM_004496	Hs.163484	NM_004496
FOXA1	22.1	15.2	forkhead box A1 (FOXA1), mRNA	NM_004496	Hs.163484	NM_004496
LIX1	21.6	20.5	Lix1 homolog (mouse) (LIX1), mRNA	NM_153234	Hs.656702	BC036467
EVI1	21.5	32.9	ecotropic viral integration site 1 (EVI1), mRNA	NM_005241	Hs.656395	BX640908
ZFHX4	21.5	10.4	zinc finger homeodomain 4 (ZFHX4), mRNA	NM_024721	Hs.458973	NM_024721
THC2652817	21.4	9.4	THC2652817	THC2652817	Unknown	
HOXB3	21.4	73.0	homeobox B3 (HOXB3), mRNA	NM_002146	Hs.654560	NM_002146
ENST00000379426	21.1	3.9	Novel protein.	ENST00000379426	Unknown	
FOXA1	21.1	16.6	forkhead box A1 (FOXA1), mRNA	NM_004496	Hs.163484	NM_004496
EVI1	20.3	25.7	ecotropic viral integration site 1 (EVI1), mRNA	NM_005241	Hs.656395	BX640908
GJB2	20.0	11.1	gap junction protein, beta 2, 26kDa (connexin 26) (GJB2), mRNA	NM_004004	Hs.591234	NM_004004
EBF3	20.0	26.4	early B-cell factor 3 (EBF3), mRNA	NM_001005463	Hs.699395	NM_001005463
BACH2	19.9	4.9	BTB and CNC homology 1, basic leucine zipper transcription factor 2 (BACH2), mRNA	NM_021813	Hs.269764	AJ271878
SEMA3D	19.7	9.4	sema domain, immunoglobulin domain (Ig), short basic domain, secreted, (semaphorin) 3D (SEMA3D), mRNA	NM_152754	Hs.201340	NM_152754
MPPED2	19.7	7.6	metallophosphoesterase domain containing 2 (MPPED2), mRNA	NM_001584	Hs.289795	AB209163
SERPINA1	19.6	12.9	serpin peptidase inhibitor, clade A (alpha-1 antiproteinase, antitrypsin), member 1 (SERPINA1), transcript variant 2, mRNA	NM_001002236	Hs.525557	CR594071
FLJ37644	19.4	13.3	cDNA FLJ37644 fis, clone BRHIP2000239.	AK094963	Hs.657374	AK094963
BC042520	19.2	10.3	cDNA clone IMAGE:4828750.	BC042520	Hs.375762	AK056686
THC2692669	19.2	16.3	ALU1_HUMAN (P39188) Alu subfamily J sequence contamination warning entry, partial (10%)	THC2692669	Unknown	
AK091573	18.9	12.6	cDNA FLJ34254 fis, clone FCBBF5000325.	AK091573	Hs.26409	BC041405
DKFZp586C0721	18.8	14.0	mRNA; cDNA DKFZp586C0721 (from clone DKFZp586C0721).	AL137734	Hs.437437	CR933654
KLF12	18.8	3.5	Kruppel-like factor 12 (KLF12), mRNA	NM_007249	Hs.373857	NM_007249
FOXA1	18.8	16.7	forkhead box A1 (FOXA1), mRNA	NM_004496	Hs.163484	NM_004496
LIX1	18.7	10.9	Lix1 homolog (mouse) (LIX1), mRNA	NM_153234	Hs.656702	BC036467
DACT3	18.7	14.6	dapper, antagonist of beta-catenin, homolog 3 (Xenopus laevis) (DACT3), mRNA	NM_145056	Hs.515490	BC034052
ENST00000383706	18.7	9.3	ADAMTS-9 precursor (EC 3.4.24.-) (A disintegrin and metalloproteinase with thrombospondin motifs 9) (ADAM-TS 9) (ADAM-TS9).	ENST00000383706	Unknown	
BCL2	18.4	7.7	B-cell CLL/lymphoma 2 (BCL2), nuclear gene encoding mitochondrial protein, transcript variant alpha, mRNA	NM_000633	Hs.150749	NM_000633
ZHX1	18.0	9.6	zinc fingers and homeoboxes 1 (ZHX1), transcript variant 1, mRNA	NM_001017926	Hs.612084	NM_001017926

FOXA1	17.9	20.8	forkhead box A1 (FOXA1), mRNA	NM_004496	Hs.163484	NM_004496
NPR3	17.8	11.8	natriuretic peptide receptor C/guanylate cyclase C (atrionatriuretic peptide receptor C) (NPR3), mRNA	NM_000908	Hs.237028	NM_000908
LRRN3	17.8	7.3	leucine rich repeat neuronal 3 (LRRN3), mRNA	NM_018334	Hs.3781	NM_001099660
CBFA2T3	17.5	8.8	core-binding factor, runt domain, alpha subunit 2; translocated to, 3 (CBFA2T3), transcript variant 1, mRNA	NM_005187	Hs.513811	NM_005187
DQ786266	17.3	6.5	clone HLS_IMAGE_298862 mRNA sequence.	DQ786266	Hs.645697	DQ786266
BCL2	17.3	7.7	B-cell CLL/lymphoma 2 (BCL2), nuclear gene encoding mitochondrial protein, transcript variant alpha, mRNA	NM_000633	Hs.150749	NM_000633
DACH1	17.2	30.5	dachshund homolog 1 (Drosophila) (DACH1), transcript variant 1, mRNA	NM_080759	Hs.129452	NM_080759
LOC255480	17.1	41.3	cDNA FLJ34447 fis, clone HLUNG2002059.	AK091766	Hs.567915	AK091766
FOXA1	16.8	14.7	forkhead box A1 (FOXA1), mRNA	NM_004496	Hs.163484	NM_004496
TMEM117	16.8	8.0	transmembrane protein 117 (TMEM117), mRNA	NM_032256	Hs.444668	BC060798
DACH2	16.7	8.5	dachshund homolog 2 (Drosophila) (DACH2), mRNA	NM_053281	Hs.86603	BC048423
ENST00000375284	16.7	7.3	Protein patched homolog 1 (PTC1) (PTC).	ENST00000375284	Unknown	
TSHZ3	16.6	12.7	teashirt family zinc finger 3 (TSHZ3), mRNA	NM_020856	Hs.278436	NM_020856
ITGA8	16.5	15.4	integrin, alpha 8 (ITGA8), mRNA	NM_003638	Hs.171311	NM_003638
TTC3	16.5	4.1	tetratricopeptide repeat domain 3 (TTC3), transcript variant 1, mRNA	NM_003316	Hs.368214	D84294
THC2714296	16.5	9.1	THC2714296	THC2714296	Unknown	
MGC16121	16.5	11.1	hypothetical protein MGC16121, mRNA (cDNA clone IMAGE:3627113), complete cds.	BC007360	Hs.416379	XM_001128419
SLC8A1	16.3	11.8	solute carrier family 8 (sodium/calcium exchanger), member 1 (SLC8A1), mRNA	NM_021097	Hs.468274	NM_021097
EBF3	16.3	61.2	early B-cell factor 3 (EBF3), mRNA	NM_001005463	Hs.699395	NM_001005463
KIAA1713	16.3	36.1	mRNA for KIAA1713 protein, partial cds.	AB051500	Hs.464876	NM_030632
BCL2	16.1	8.6	B-cell CLL/lymphoma 2 (BCL2), nuclear gene encoding mitochondrial protein, transcript variant alpha, mRNA	NM_000633	Hs.150749	NM_000633
BX337332	16.1	33.7	BX337332 PLACENTA COT 25-NORMALIZED cDNA clone CS0DI040YE21 5-PRIME, mRNA sequence	BX337332	Hs.635297	BX337332
IL17D	16.1	10.1	interleukin 17D (IL17D), mRNA	NM_138284	Hs.655142	AK055408
U92981	16.0	7.6	clone DT1P1B6 mRNA, CAG repeat region.	U92981	Hs.596282	U92981
CXCR7	16.0	9.7	chemokine (C-X-C motif) receptor 7 (CXCR7), transcript variant 1, mRNA	NM_001047841	Unknown	
PROS1	15.9	16.7	protein S (alpha) (PROS1), mRNA	NM_000313	Hs.64016	M14338
ZIC1	15.9	25.8	Zic family member 1 (odd-paired homolog, Drosophila) (ZIC1), mRNA	NM_003412	Hs.647962	NM_003412
CDH11	15.8	10.5	cadherin 11, type 2, OB-cadherin (osteoblast) (CDH11), mRNA	NM_001797	Hs.116471	D21255
AK000872	15.7	22.5	cDNA FLJ10010 fis, clone HEMBA1000302.	AK000872	Hs.58690	AK128633
DA834198	15.7	3.6	DA834198 PLACE3 cDNA clone PLACE3000419 5', mRNA sequence	DA834198	Hs.491872	DA834198
FOXA1	15.5	15.7	forkhead box A1 (FOXA1), mRNA	NM_004496	Hs.163484	NM_004496
CR590573	15.4	6.4	full-length cDNA clone CS0DI042YD07 of Placenta Cot 25-normalized of (human).	CR590573	Hs.484967	CR590573
ZNF25	15.4	13.1	zinc finger protein 25 (ZNF25), mRNA	NM_145011	Hs.499429	AL834125
FLJ13197	15.3	5.7	hypothetical protein FLJ13197 (FLJ13197), mRNA	NM_024614	Unknown	
ANKH	15.3	6.8	Progressive ankylosis protein homolog (ANK).	ENST00000382327	Unknown	
SST	15.3	20.9	somatostatin (SST), mRNA	NM_001048	Hs.12409	BI918626
C20orf82	15.1	16.8	cDNA FLJ23893 fis, clone LNG14589.	AK074473	Unknown	
FLJ21986	15.1	28.1	hypothetical protein FLJ21986 (FLJ21986), mRNA	NM_024913	Hs.189652	NM_024913
ID2	15.0	7.3	inhibitor of DNA binding 2, dominant negative helix-loop-helix protein (ID2), mRNA	NM_002166	Hs.180919	CR623038
MEIS1	15.0	23.3	Meis1, myeloid ecotropic viral integration site 1 homolog (mouse) (MEIS1), mRNA	NM_002398	Hs.526754	CR749827
ZNF184	15.0	2.9	zinc finger protein 184 (ZNF184), mRNA	NM_007149	Hs.158174	AK123011
ID2	15.0	12.3	inhibitor of DNA binding 2, dominant negative helix-loop-helix protein (ID2), mRNA	NM_002166	Hs.180919	CR623038
CXXC4	15.0	9.6	CXXC finger 4	ENST00000305749	Unknown	
APOB	15.0	21.2	apolipoprotein B (including Ag(x) antigen) (APOB), mRNA	NM_000384	Hs.120759	NM_000384
SMARCE1	14.9	2.5	SWI/SNF related, matrix associated, actin dependent regulator of chromatin, subfamily e, member 1 (SMARCE1), mRNA	NM_003079	Hs.696086	BC069196
SCRG1	14.9	45.6	scrapie responsive protein 1 (SCRG1), mRNA	NM_007281	Hs.7122	BC067829
NLRP1	14.8	8.3	NLR family, pyrin domain containing 1, mRNA (cDNA clone MGC:57544 IMAGE:5756099), complete cds.	BC051787	Unknown	
IRX5	14.8	8.3	iroquois homeobox protein 5 (IRX5), mRNA	NM_005853	Hs.435730	NM_005853
AU146383	14.7	8.4	AU146383 HEMBB1 cDNA clone HEMBB1000334 3', mRNA sequence	AU146383	Hs.653898	BX405503
C8orf57	14.7	3.1	mRNA; cDNA DKFZp761D112 (from clone DKFZp761D112).	AL136588	Hs.492187	AL136588
ZNF624	14.7	4.4	zinc finger protein 624 (ZNF624), mRNA	NM_020787	Hs.128078	AK131401

TSHZ2	14.6	25.4	teashirt family zinc finger 2 (TSHZ2), mRNA	NM_173485	Hs.473117	NM_173485
ZNF606	14.6	2.7	zinc finger protein 606 (ZNF606), mRNA	NM_025027	Hs.654967	AF455357
TTC18	14.5	14.3	tetratricopeptide repeat domain 18 (TTC18), mRNA	NM_145170	Hs.591367	AL833537
ISL1	14.4	35.6	ISL1 transcription factor, LIM/homeodomain, (islet-1) (ISL1), mRNA	NM_002202	Hs.505	NM_002202
APOB	14.4	21.3	apolipoprotein B (including Ag(x) antigen) (APOB), mRNA	NM_000384	Hs.120759	NM_000384
RAI1	14.4	10.0	retinoic acid induced 1 (RAI1), mRNA	NM_030665	Hs.655395	NM_030665
AK096580	14.4	25.8	cDNA FLJ39261 fis, clone OCBBF2009391.	AK096580	Hs.13281	AK096580
RBM20	14.4	13.4	Novel protein (Fragment).	ENST00000369519	Unknown	
EDG2	14.3	9.5	endothelial differentiation, lysophosphatidic acid G-protein-coupled receptor, 2 (EDG2), transcript variant 2, mRNA	NM_057159	Hs.126667	BC036034
HOXB4	14.3	26.1	homeobox B4 (HOXB4), mRNA	NM_024015	Hs.664706	NM_024015
C10orf63	14.3	22.0	chromosome 10 open reading frame 63 (C10orf63), mRNA	NM_145010	Hs.534486	AK124767
APOM	14.3	6.0	apolipoprotein M (APOM), mRNA	NM_019101	Hs.534468	BG567934
ANKRD6	14.2	3.9	mRNA for KIAA0957 protein, partial cds.	AB023174	Hs.656539	NM_014942
PHOX2B	14.2	34.1	paired-like homeobox 2b (PHOX2B), mRNA	NM_003924	Hs.87202	NM_003924
JAG1	14.1	4.5	jagged 1 (Alagille syndrome) (JAG1), mRNA	NM_000214	Hs.224012	AF003837
SENP7	14.1	4.3	SUMO1/sentrin specific peptidase 7 (SENP7), transcript variant 1, mRNA	NM_020654	Hs.529551	NM_020654
HS3ST3B1	14.1	5.8	Heparan sulfate glucosamine 3-O-sulfotransferase 3B1 (EC 2.8.2.30) (Heparan sulfate D-glucosaminyl 3-O-sulfotransferase 3B1) (Heparan sulfate 3-O-sulfotransferase 3B1) (h3-OST-3B).	ENST00000360954	Unknown	
ELMOD1	14.1	10.9	ELMO/CED-12 domain containing 1 (ELMOD1), mRNA	NM_018712	Hs.495779	NM_018712
NBLA00301	14.0	11.4	neuroblastoma cDNA, clone:Nbla00301, full insert sequence.	AB075501	Hs.61435	DQ831956
LNPEP	14.0	7.9	Leucyl-cystinyl aminopeptidase (EC 3.4.11.3) (Cystinyl aminopeptidase) (Oxytocinase) (OTase) (Insulin-regulated membrane aminopeptidase) (Insulin-responsive aminopeptidase) (IRAP) (Placental leucine aminopeptidase) (P-LAP)....	ENST00000379870	Unknown	
PRND	14.0	64.7	prion protein 2 (dublet) (PRND), mRNA	NM_012409	Hs.406696	NM_012409
ST7OT1	13.9	4.8	ST7 overlapping transcript 1 (antisense non-coding RNA) (ST7OT1) on chromosome 7	NR_002330	Unknown	
PARD6G	13.9	2.9	par-6 partitioning defective 6 homolog gamma (C. elegans) (PARD6G), mRNA	NM_032510	Hs.654920	NM_032510
C5orf23	13.9	80.0	chromosome 5 open reading frame 23 (C5orf23), mRNA	NM_024563	Hs.13528	BC022250
MYL4	13.8	18.9	myosin, light chain 4, alkali; atrial, embryonic (MYL4), transcript variant 2, mRNA	NM_002476	Hs.463300	BM919855
MBNL2	13.8	13.8	muscleblind-like 2 (Drosophila) (MBNL2), transcript variant 1, mRNA	NM_144778	Hs.657347	NM_144778
MDGA1	13.8	5.5	MAM domain containing glycosylphosphatidylinositol anchor 1 (MDGA1), mRNA	NM_153487	Hs.437993	AF478693
APCDD1	13.8	9.4	adenomatosis polyposis coli down-regulated 1 (APCDD1), mRNA	NM_153000	Hs.293274	AB104887
TANC1	13.7	5.7	tetratricopeptide repeat, ankyrin repeat and coiled-coil containing 1 (TANC1), mRNA	NM_033394	Hs.158728	NM_033394
C9orf9	13.7	4.6	chromosome 9 open reading frame 9 (C9orf9), mRNA	NM_018956	Hs.62595	AK130389
RKHD3	13.6	4.5	ring finger and KH domain containing 3 (RKHD3), mRNA	NM_032246	Hs.104744	AK131424
BDH2	13.6	4.0	3-hydroxybutyrate dehydrogenase, type 2 (BDH2), mRNA	NM_020139	Hs.124696	NM_020139
HMGA2	13.6	3.5	high mobility group AT-hook 2 (HMGA2), transcript variant 1, mRNA	NM_003483	Hs.505924	AB209853
PGM5	13.6	13.6	phosphoglucomutase 5 (PGM5), mRNA	NM_021965	Hs.307835	AL137698
KIAA1505	13.5	6.7	KIAA1505 protein (KIAA1505), mRNA	NM_020879	Hs.113940	BX649000
LCORL	13.5	3.9	cDNA FLJ30696 fis, clone FCBBF2000808.	AK055258	Hs.661849	AK055258
MLLT3	13.5	7.8	myeloid/lymphoid or mixed-lineage leukemia (trithorax homolog, Drosophila); translocated to, 3 (MLLT3), mRNA	NM_004529	Hs.591085	NM_004529
POSTN	13.4	21.9	periostin, osteoblast specific factor (POSTN), mRNA	NM_006475	Hs.136348	D13666
FZD1	13.4	9.4	frizzled homolog 1 (Drosophila) (FZD1), mRNA	NM_003505	Hs.94234	AB017363
NR2F2	13.4	26.2	nuclear receptor subfamily 2, group F, member 2 (NR2F2), mRNA	NM_021005	Hs.347991	BC042897
C14orf132	13.4	3.5	chromosome 14 open reading frame 132 (C14orf132), mRNA	NM_020215	Unknown	
EDNRA	13.4	22.6	endothelin receptor type A (EDNRA), mRNA	NM_001957	Hs.183713	NM_001957
THC2660361	13.4	4.7	THC2660361	THC2660361	Unknown	
GJB2	13.4	6.6	gap junction protein, beta 2, 26kDa (connexin 26) (GJB2), mRNA	NM_004004	Hs.591234	NM_004004
EBF3	13.3	50.7	early B-cell factor 3 (EBF3), mRNA	NM_001005463	Hs.699395	NM_001005463
LCOR	13.2	2.7	ligand dependent nuclear receptor corepressor (LCOR), mRNA	NM_032440	Hs.500695	AL834245
TSGA14	13.2	4.6	testis specific, 14 (TSGA14), mRNA	NM_018718	Hs.368315	AF429308
BCL2	13.2	7.9	B-cell CLL/lymphoma 2 (BCL2), nuclear gene encoding mitochondrial protein, transcript variant alpha, mRNA	NM_000633	Hs.150749	NM_000633
IFT88	13.2	5.3	intraflagellar transport 88 homolog (Chlamydomonas) (IFT88), transcript variant 1, mRNA	NM_175605	Hs.187376	AK126668
RBP1	13.2	5.2	retinol binding protein 1, cellular (RBP1), mRNA	NM_002899	Hs.529571	BM926478

GHR	13.1	16.2	growth hormone receptor (GHR), mRNA	NM_000163	Hs.125180	X06562
ITGB8	13.1	5.9	integrin, beta 8 (ITGB8), mRNA	NM_002214	Hs.592171	NM_002214
TANC1	13.1	6.3	tetratricopeptide repeat, ankyrin repeat and coiled-coil containing 1 (TANC1), mRNA	NM_033394	Hs.158728	NM_033394
SOX11	13.1	3.1	SRY (sex determining region Y)-box 11 (SOX11), mRNA	NM_003108	Hs.432638	AB028641
APOB	13.1	17.4	apolipoprotein B (including Ag(x) antigen) (APOB), mRNA	NM_000384	Hs.120759	NM_000384
KPNA5	13.1	2.3	Importin alpha-6 subunit (Karyopherin alpha-5 subunit).	ENST00000368564	Unknown	
THC2610890	13.1	3.3	Q54TC3_DICDI (Q54TC3) FVYE domain-containing protein, partial (3%)	THC2610890	Unknown	
APOB	13.1	21.3	apolipoprotein B (including Ag(x) antigen) (APOB), mRNA	NM_000384	Hs.120759	NM_000384
MEIS2	13.1	54.6	Meis1, myeloid ecotropic viral integration site 1 homolog 2 (mouse) (MEIS2), transcript variant a, mRNA	NM_170677	Hs.510989	NM_170677
CR624880	13.0	3.2	full-length cDNA clone CS0DB005YI23 of Neuroblastoma Cot 10-normalized of (human).	CR624880	Hs.505983	AK056140
APOB	13.0	19.0	apolipoprotein B (including Ag(x) antigen) (APOB), mRNA	NM_000384	Hs.120759	NM_000384
AK021785	13.0	36.0	cDNA FLJ11723 fis, clone HEMBA1005314.	AK021785	Hs.586722	AK021785
COL2A1	12.9	10.8	collagen, type II, alpha 1 (primary osteoarthritis, spondyloepiphyseal dysplasia, congenital) (COL2A1), transcript variant 1, mRNA	NM_001844	Hs.408182	NM_001844
EFCAB1	12.9	12.3	EF-hand calcium binding domain 1 (EFCAB1), mRNA	NM_024593	Hs.23245	NM_024593
C8orf72	12.9	7.4	chromosome 8 open reading frame 72 (C8orf72), mRNA	NM_147189	Hs.154652	NM_147189
FKBP7	12.8	6.2	FK506 binding protein 7 (FKBP7), mRNA	NM_181342	Hs.410378	NM_181342
COL3A1	12.8	38.2	collagen, type III, alpha 1 (Ehlers-Danlos syndrome type IV, autosomal dominant) (COL3A1), mRNA	NM_000090	Hs.443625	NM_000090
PCDH17	12.8	17.9	protocadherin 17 (PCDH17), mRNA	NM_001040429	Hs.106511	NM_001040429
CK820941	12.8	4.5	ie09h05.y5 HR85 islet cDNA clone IMAGE:5086712 5', mRNA sequence	CK820941	Hs.596906	CK820941
CXCL12	12.7	5.1	chemokine (C-X-C motif) ligand 12 (stromal cell-derived factor 1) (CXCL12), transcript variant 2, mRNA	NM_000609	Hs.522891	AK090482
SRGAP3	12.7	5.4	SLIT-ROBO Rho GTPase activating protein 3 (SRGAP3), transcript variant 1, mRNA	NM_014850	Hs.654743	NM_014850
AK055302	12.7	4.3	cDNA FLJ30740 fis, clone FEBRA2000319.	AK055302	Hs.609017	AK055302
PLN	12.7	127.2	phospholamban (PLN), mRNA	NM_002667	Hs.170839	AK129844
EDNRA	12.7	5.8	endothelin receptor type A (EDNRA), mRNA	NM_001957	Hs.183713	NM_001957
DYX1C1	12.7	4.8	dyslexia susceptibility 1 candidate 1 (DYX1C1), transcript variant 1, mRNA	NM_130810	Hs.126403	AF337549
GRIN3A	12.7	9.0	glutamate receptor, ionotropic, N-methyl-D-aspartate 3A (GRIN3A), mRNA	NM_133445	Hs.654783	AB075853
HS6ST2	12.6	3.3	heparan sulfate 6-O-sulfotransferase 2 (HS6ST2), transcript variant L, mRNA	NM_001077188	Hs.385956	NM_001077188
ALG9	12.6	3.5	asparagine-linked glycosylation 9 homolog (S. cerevisiae, alpha- 1,2-mannosyltransferase) (ALG9), transcript variant 3, mRNA	NM_001077691	Hs.503850	NM_001077691
LRRC49	12.6	2.7	leucine rich repeat containing 49 (LRRC49), mRNA	NM_017691	Hs.12692	NM_017691
BCL2	12.6	8.0	B-cell CLL/lymphoma 2 (BCL2), nuclear gene encoding mitochondrial protein, transcript variant alpha, mRNA	NM_000633	Hs.150749	NM_000633
GPR155	12.5	11.4	G protein-coupled receptor 155 (GPR155), transcript variant 9, mRNA	NM_001033045	Hs.516604	NM_001033045
THC2610705	12.5	9.8	THC2610705	THC2610705	Unknown	
SMOC1	12.5	6.6	SPARC related modular calcium binding 1 (SMOC1), transcript variant 1, mRNA	NM_001034852	Hs.497349	NM_001034852
GAS1	12.5	9.0	growth arrest-specific 1 (GAS1), mRNA	NM_002048	Hs.65029	NM_002048
NAIP	12.5	4.9	NLR family, apoptosis inhibitory protein (NAIP), transcript variant 1, mRNA	NM_004536	Hs.654500	NM_004536
FOXF2	12.4	37.2	forkhead box F2 (FOXF2), mRNA	NM_001452	Hs.484423	NM_001452
CAMK2N1	12.4	5.1	calcium/calmodulin-dependent protein kinase II inhibitor 1 (CAMK2N1), mRNA	NM_018584	Hs.197922	NM_018584
IGF2AS	12.4	5.7	insulin-like growth factor 2 antisense (IGF2AS), mRNA	NM_016412	Hs.272259	NM_016412
RIN2	12.4	12.5	Ras and Rab interactor 2 (RIN2), mRNA	NM_018993	Hs.472270	NM_018993
APOB	12.4	20.6	apolipoprotein B (including Ag(x) antigen) (APOB), mRNA	NM_000384	Hs.120759	NM_000384
A_32_P122285	12.4	5.1	A_32_P122285	A_32_P122285	Unknown	
ANKRD38	12.3	10.9	ankyrin repeat domain 38 (ANKRD38), mRNA	NM_181712	Hs.283398	NM_181712
THC2679528	12.3	9.3	THC2679528	THC2679528	Unknown	
HHIP	12.3	17.9	cDNA FLJ90230 fis, clone NT2RM2000410.	AK074711	Hs.507991	AY009951
AK124698	12.3	4.6	cDNA FLJ42708 fis, clone BRAMY3007311.	AK124698	Hs.411472	CR749843
F2	12.3	17.8	coagulation factor II (thrombin) (F2), mRNA	NM_000506	Hs.655207	BC051332
APOB	12.3	21.7	apolipoprotein B (including Ag(x) antigen) (APOB), mRNA	NM_000384	Hs.120759	NM_000384
APOB	12.3	18.7	apolipoprotein B (including Ag(x) antigen) (APOB), mRNA	NM_000384	Hs.120759	NM_000384
GRIK2	12.2	7.2	glutamate receptor, ionotropic, kainate 2 (GRIK2), transcript variant 2, mRNA	NM_175768	Hs.654523	NM_175768
HOXB2	12.1	25.3	homeobox B2 (HOXB2), mRNA	NM_002145	Hs.514289	NM_002145
THC2609891	12.1	15.9	THC2609891	THC2609891	Unknown	
ARID5B	12.1	18.7	AT rich interactive domain 5B (MRF1-like) (ARID5B), mRNA	NM_032199	Hs.535297	NM_032199

LOC729464	12.1	11.1	PREDICTED: hypothetical protein LOC729464 (LOC729464), mRNA	XM_001133802	Hs.111902	XM_001133802
FLJ21986	12.0	11.3	hypothetical protein FLJ21986 (FLJ21986), mRNA	NM_024913	Hs.189652	NM_024913
NPR3	12.0	14.0	natriuretic peptide receptor C/guanylate cyclase C (atrionatriuretic peptide receptor C) (NPR3), mRNA	NM_000908	Hs.237028	NM_000908
LIPC	12.0	53.9	lipase, hepatic (LIPC), mRNA	NM_000236	Hs.654472	BC146659
MGC24039	12.0	4.8	hypothetical protein MGC24039 (MGC24039), mRNA	NM_144973	Hs.118166	NM_144973
ZBTB26	12.0	3.3	Zinc finger and BTB domain-containing protein 26 (Zinc finger protein 481) (Zinc finger protein Bioref).	ENST00000373656	Unknown	
HEY1	12.0	9.7	hairy/enhancer-of-split related with YRPW motif 1 (HEY1), transcript variant 2, mRNA	NM_001040708	Hs.234434	NM_001040708
MAPK4	12.0	2.5	H.sapiens 63 kDa protein kinase related to rat ERK3.	X59727	Hs.433728	BC050299
F2	12.0	18.8	coagulation factor II (thrombin) (F2), mRNA	NM_000506	Hs.655207	BC051332
NME5	11.9	4.0	non-metastatic cells 5, protein expressed in (nucleoside-diphosphate kinase) (NME5), mRNA	NM_003551	Hs.519602	NM_003551
EMP2	11.9	3.0	epithelial membrane protein 2 (EMP2), mRNA	NM_001424	Hs.655130	AK096403
MSX2	11.9	17.2	msh homeobox 2 (MSX2), mRNA	NM_002449	Hs.89404	NM_002449
BCL2	11.8	7.9	B-cell CLL/lymphoma 2 (BCL2), nuclear gene encoding mitochondrial protein, transcript variant alpha, mRNA	NM_000633	Hs.150749	NM_000633
C21orf62	11.8	4.5	chromosome 21 open reading frame 62 (C21orf62), mRNA	NM_019596	Hs.517235	NM_019596
A_23_P10091	11.8	6.7	A_23_P10091	A_23_P10091	Unknown	
RIMBP2	11.7	9.7	RIMS binding protein 2 (RIMBP2), mRNA	NM_015347	Hs.657441	NM_015347
REV3L	11.7	4.5	REV3-like, catalytic subunit of DNA polymerase zeta (yeast) (REV3L), mRNA	NM_002912	Hs.232021	AF078695
ZMIZ1	11.7	4.4	zinc finger, MIZ-type containing 1 (ZMIZ1), mRNA	NM_020338	Hs.193118	NM_020338
RSNL2	11.7	11.9	restin-like 2 (RSNL2), mRNA	NM_024692	Hs.122927	AB209042
C9orf9	11.7	5.8	chromosome 9 open reading frame 9 (C9orf9), mRNA	NM_018956	Hs.62595	AK130389
ASGR2	11.7	12.2	asialoglycoprotein receptor 2 (ASGR2), transcript variant H2', mRNA	NM_080912	Hs.654440	NM_080912
FLJ33630	11.6	6.2	full length insert cDNA clone ZD74E10.	AF088062	Hs.340623	BX648677
CR622072	11.6	4.3	full-length cDNA clone CS0DF032YA11 of Fetal brain of (human).	CR622072	Hs.444785	CR622072
RASGRP3	11.6	24.5	RAS guanyl releasing protein 3 (calcium and DAG-regulated) (RASGRP3), mRNA	NM_170672	Hs.143674	BC027849
CR620977	11.6	2.5	full-length cDNA clone CS0CAP004YK15 of Thymus of (human).	CR620977	Hs.377961	CR620977
EFNB2	11.6	4.0	ephrin-B2 (EFNB2), mRNA	NM_004093	Hs.149239	NM_004093
AK026826	11.6	10.2	cDNA: FLJ23173 fis, clone LNG10019.	AK026826	Hs.113157	BX647350
AK095738	11.5	6.6	cDNA FLJ38419 fis, clone FEBRA2009846.	AK095738	Hs.586950	AK095738
ENST00000380676	11.5	6.0	cDNA FLJ27352 fis, clone TST05165.	ENST00000380676	Unknown	
PIWIL4	11.5	4.1	piwi-like 4 (Drosophila) (PIWIL4), mRNA	NM_152431	Hs.660188	CR749642
PRSS35	11.5	15.2	protease, serine, 35 (PRSS35), mRNA	NM_153362	Hs.98381	AY358661
IFIT5	11.5	5.4	interferon-induced protein with tetratricopeptide repeats 5 (IFIT5), mRNA	NM_012420	Hs.252839	NM_012420
ACSS1	11.5	4.5	acyl-CoA synthetase short-chain family member 1 (ACSS1), nuclear gene encoding mitochondrial protein, mRNA	NM_032501	Hs.529353	AK125058
ZNRF1	11.5	2.3	zinc and ring finger 1 (ZNRF1), mRNA	NM_032268	Hs.427284	NM_032268
F2	11.5	14.6	coagulation factor II (thrombin) (F2), mRNA	NM_000506	Hs.655207	BC051332
APOM	11.5	5.5	apolipoprotein M (APOM), mRNA	NM_019101	Hs.534468	BG567934
LOC728923	11.4	6.3	cDNA clone IMAGE:5286843.	BC036622	Hs.666502	BC036622
CBFA2T2	11.4	2.8	core-binding factor, runt domain, alpha subunit 2; translocated to, 2 (CBFA2T2), transcript variant 2, mRNA	NM_005093	Hs.153934	NM_005093
AW389821	11.3	11.6	AW389821 RC5-ST0171-201099-012-c05 ST0171 cDNA, mRNA sequence	AW389821	Hs.526754	CR749827
HOXB3	11.3	13.2	homeobox B3 (HOXB3), mRNA	NM_002146	Hs.654560	NM_002146
ZNF291	11.3	2.6	zinc finger protein 291 (ZNF291), mRNA	NM_020843	Hs.458986	AB040887
CD242823	11.3	3.0	AGENCOURT_14126724 NIH_MGC_179 cDNA clone IMAGE:30385216 5', mRNA sequence	CD242823	Hs.597139	CD242823
KIAA1715	11.3	2.8	Protein lunapark.	ENST00000272748	Unknown	
RGL1	11.3	7.7	ral guanine nucleotide dissociation stimulator-like 1 (RGL1), mRNA	NM_015149	Hs.497148	NM_015149
CYP26B1	11.3	18.7	cytochrome P450, family 26, subfamily B, polypeptide 1 (CYP26B1), mRNA	NM_019885	Hs.91546	NM_019885
MYL7	11.3	23.9	myosin, light chain 7, regulatory (MYL7), mRNA	NM_021223	Hs.75636	BI836837
FLRT2	11.3	5.8	fibronectin leucine rich transmembrane protein 2 (FLRT2), mRNA	NM_013231	Hs.533710	NM_013231
HOXA2	11.3	13.3	homeobox A2 (HOXA2), mRNA	NM_006735	Hs.592177	NM_006735
SULF1	11.3	10.5	sulfatase 1 (SULF1), mRNA	NM_015170	Hs.409602	AF545571
HSF2	11.2	2.9	heat shock transcription factor 2 (HSF2), mRNA	NM_004506	Hs.158195	BC112323
TOM1L2	11.2	5.2	cDNA FLJ31397 fis, clone NT2NE1000163.	AK055959	Hs.462379	NM_001082968
AK123972	11.2	7.8	cDNA FLJ41978 fis, clone SKNSH2000482.	AK123972	Hs.435458	BC146776

LRRC39	11.2	11.3	leucine rich repeat containing 39 (LRRC39), mRNA	NM_144620	Hs.44277	AL832694
HIVEP3	11.2	7.2	human immunodeficiency virus type I enhancer binding protein 3	ENST00000372583	Unknown	
PAK7	11.2	2.2	p21(CDKN1A)-activated kinase 7 (PAK7), transcript variant 1, mRNA	NM_020341	Hs.32539	AB033090
A_24_P845631	11.2	16.4	A_24_P845631	A_24_P845631	Unknown	
C21orf62	11.2	4.5	chromosome 21 open reading frame 62 (C21orf62), mRNA	NM_019596	Hs.517235	NM_019596
TIFA	11.2	7.1	TRAF-interacting protein with a forkhead-associated domain (TIFA), mRNA	NM_052864	Hs.310640	NM_052864
LOC51136	11.2	4.1	PTD016 protein (LOC51136), mRNA	NM_016125	Hs.531701	NM_016125
APOB	11.2	18.9	apolipoprotein B (including Ag(x) antigen) (APOB), mRNA	NM_000384	Hs.120759	NM_000384
HEYL	11.2	4.9	hairy/enhancer-of-split related with YRPW motif-like (HEYL), mRNA	NM_014571	Hs.472566	NM_014571
RP5-1022P6.2	11.1	3.0	hypothetical protein KIAA1434 (KIAA1434), mRNA	NM_019593	Hs.636359	NM_019593
ADAMTS9	11.1	14.8	ADAM metallopeptidase with thrombospondin type 1 motif, 9 (ADAMTS9), mRNA	NM_182920	Hs.656071	NM_182920
RARB	11.1	23.6	retinoic acid receptor, beta (RARB), transcript variant 1, mRNA	NM_000965	Hs.654490	BC030234
C12orf23	11.1	4.5	chromosome 12 open reading frame 23 (C12orf23), mRNA	NM_152261	Hs.257664	AK001731
BMI1	11.0	4.1	Human prot-oncogene (BMI-1) mRNA, complete cds.	L13689	Hs.496613	NM_005180
SULT1E1	11.0	27.1	sulfotransferase family 1E, estrogen-preferring, member 1 (SULT1E1), mRNA	NM_005420	Hs.479898	BC027956
ITPR1	11.0	11.7	inositol 1,4,5-triphosphate receptor, type 1 (ITPR1), mRNA	NM_002222	Hs.699169	NM_001099952
FAM3B	11.0	2.6	family with sequence similarity 3, member B (FAM3B), transcript variant 1, mRNA	NM_058186	Hs.473877	BC036314
AK055712	11.0	5.9	cDNA FLJ31150 fis, clone IMR322001534.	AK055712	Hs.129828	XM_001128243
BC030083	11.0	10.6	cDNA clone IMAGE:4791783.	BC030083	Hs.648482	AK001884
SMARCD3	11.0	11.7	SWI/SNF related, matrix associated, actin dependent regulator of chromatin, subfamily d, member 3 (SMARCD3), transcript variant 2, mRNA	NM_003078	Hs.647067	BX648385
RP11-564C4.1	11.0	15.8	mRNA; cDNA DKFZp779G2222 (from clone DKFZp779G2222).	CR749831	Hs.282795	CR749831
MAFB	11.0	5.4	v-maf musculoaponeurotic fibrosarcoma oncogene homolog B (avian) (MAFB), mRNA	NM_005461	Hs.651210	NM_005461
WNT2	11.0	9.8	wingless-type MMTV integration site family member 2 (WNT2), mRNA	NM_003391	Hs.567356	BC078170
GJA4	11.0	5.6	gap junction protein, alpha 4, 37kDa (connexin 37) (GJA4), mRNA	NM_002060	Hs.296310	NM_002060
MYOZ2	10.9	250.1	myozenin 2 (MYOZ2), mRNA	NM_016599	Hs.381047	NM_016599
PDGFC	10.9	9.9	platelet derived growth factor C (PDGFC), mRNA	NM_016205	Hs.570855	AF091434
SMPX	10.9	87.4	small muscle protein, X-linked (SMPX), mRNA	NM_014332	Hs.86492	BF693607
PYGO1	10.9	4.8	mRNA; cDNA DKFZp564G0982 (from clone DKFZp564G0982).	AL049925	Hs.256587	AL049925
GJB2	10.9	9.8	gap junction protein, beta 2, 26kDa (connexin 26) (GJB2), mRNA	NM_004004	Hs.591234	NM_004004
SYNPO2	10.9	6.9	mRNA; cDNA DKFZp451G189 (from clone DKFZp451G189).	AL833294	Hs.655519	CR936673
THC2522470	10.9	2.1	BG504229 602552769F1 NIH_MGC_61 cDNA clone IMAGE:4665305 5', mRNA sequence	THC2522470	Unknown	
WNT1	10.9	4.8	wingless-type MMTV integration site family, member 1 (WNT1), mRNA	NM_005430	Hs.248164	NM_005430
KIF26B	10.9	17.1	cDNA FLJ10157 fis, clone HEMBA1003461.	AK001019	Hs.368096	AY923834
NFIA	10.9	13.6	nuclear factor I/A (NFIA), mRNA	NM_005595	Hs.191911	BX648791
ZFPM2	10.9	40.0	zinc finger protein, multitype 2 (ZFPM2), mRNA	NM_012082	Hs.431009	NM_012082
ONECUT2	10.9	6.6	one cut domain, family member 2 (ONECUT2), mRNA	NM_004852	Hs.194725	NM_004852
ENST00000355691	10.9	16.5	Copper-transporting ATPase 1 (EC 3.6.3.4) (Copper pump 1) (Menkes disease-associated protein).	ENST00000355691	Unknown	
APOB	10.9	19.7	apolipoprotein B (including Ag(x) antigen) (APOB), mRNA	NM_000384	Hs.120759	NM_000384
THC2679528	10.9	12.6	THC2679528	THC2679528	Unknown	
COMMD3	10.9	8.4	COMM domain containing 3 (COMMD3), mRNA	NM_012071	Hs.534398	BX647275
ENST00000303697	10.8	13.0	CDNA FLJ46154 fis, clone TESTI4001348.	ENST00000303697	Unknown	
PRDM16	10.8	23.7	PR domain containing 16 (PRDM16), transcript variant 1, mRNA	NM_022114	Hs.99500	NM_022114
UNC5C	10.8	14.2	unc-5 homolog C (C. elegans) (UNC5C), mRNA	NM_003728	Hs.584831	AF055634
LSAMP	10.8	6.5	limbic system-associated membrane protein (LSAMP), mRNA	NM_002338	Hs.657246	NM_002338
FGF18	10.8	6.1	fibroblast growth factor 18 (FGF18), mRNA	NM_003862	Hs.87191	AF075292
FLJ30064	10.8	3.3	cDNA FLJ30064 fis, clone ADRGL2000323.	AK054626	Unknown	
AF086261	10.8	25.2	full length insert cDNA clone ZD42A11.	AF086261	Hs.58690	AK128633
F2	10.8	15.9	coagulation factor II (thrombin) (F2), mRNA	NM_000506	Hs.655207	BC051332
THC2654231	10.8	4.1	ALU5_HUMAN (P39192) Alu subfamily SC sequence contamination warning entry, partial (8%)	THC2654231	Unknown	
GJB2	10.8	6.9	gap junction protein, beta 2, 26kDa (connexin 26) (GJB2), mRNA	NM_004004	Hs.591234	NM_004004
MYLPF	10.7	25.2	fast skeletal myosin light chain 2 (MYLPF), mRNA	NM_013292	Hs.50889	BF575830
THC2635591	10.7	2.9	THC2635591	THC2635591	Unknown	

MUM1L1	10.7	12.1	melanoma associated antigen (mutated) 1-like 1 (MUM1L1), mRNA	NM_152423	Hs.592221	AK056478
HHAT	10.7	8.6	hedgehog acyltransferase (HHAT), mRNA	NM_018194	Hs.58650	BC051191
TMEM98	10.7	4.3	transmembrane protein 98 (TMEM98), transcript variant 1, mRNA	NM_015544	Hs.695982	CR617078
NPHP1	10.7	3.9	nephronophthisis 1 (juvenile) (NPHP1), transcript variant 1, mRNA	NM_000272	Hs.280388	BX571745
LOC441212	10.7	9.5	PNAS-13 mRNA, complete cds.	AF274938	Hs.648086	CR618508
CNR1	10.6	9.7	cannabinoid receptor 1 (brain) (CNR1), transcript variant 2, mRNA	NM_033181	Hs.75110	NM_016083
CCDC126	10.6	2.5	coiled-coil domain containing 126 (CCDC126), mRNA	NM_138771	Hs.232296	AY358713
F2	10.6	17.2	coagulation factor II (thrombin) (F2), mRNA	NM_000506	Hs.655207	BC051332
FLJ30901	10.6	12.0	cDNA FLJ30901 fis, clone FEBRA2005778, weakly similar to INTEGUMENTARY MUCIN A.1 PRECURSOR.	AK055463	Unknown	
PSD3	10.6	2.5	pleckstrin and Sec7 domain containing 3 (PSD3), transcript variant 1, mRNA	NM_015310	Hs.434255	NM_015310
NAB1	10.6	2.4	NGFI-A binding protein 1 (EGR1 binding protein 1) (NAB1), mRNA	NM_005966	Hs.570078	NM_005966
C9orf18	10.6	6.8	chromosome 9 open reading frame 18 (C9orf18), mRNA	NM_198469	Hs.71428	BX110216
SYNE1	10.6	7.0	spectrin repeat containing, nuclear envelope 1 (SYNE1), transcript variant longer, mRNA	NM_033071	Hs.12967	NM_182961
GPR158	10.6	4.1	G protein-coupled receptor 158 (GPR158), mRNA	NM_020752	Hs.499108	NM_020752
ENST00000370857	10.6	4.0	Muscleblind-like X-linked protein (Muscleblind-like protein 3) (Cys3His CCG1-required protein) (Protein HCHCR).	ENST00000370857	Unknown	
THC2679484	10.6	3.6	THC2679484	THC2679484	Unknown	
SNCAIP	10.6	10.0	synuclein, alpha interacting protein (synphilin) (SNCAIP), mRNA	NM_005460	Hs.426463	BC040552
ZFP2	10.6	4.5	zinc finger protein 2 homolog (mouse) (ZFP2), mRNA	NM_030613	Hs.654533	BC142989
DACH2	10.6	7.8	dachshund homolog 2 (Drosophila) (DACH2), mRNA	NM_053281	Hs.86603	BC048423
BAALC	10.5	6.9	brain and acute leukemia, cytoplasmic (BAALC), transcript variant 1, mRNA	NM_024812	Hs.533446	NM_024812
CABLES2	10.5	3.4	Cdk5 and Abl enzyme substrate 2 (CABLES2), mRNA	NM_031215	Hs.301040	NM_031215
BCL2	10.5	9.4	B-cell CLL/lymphoma 2 (BCL2), nuclear gene encoding mitochondrial protein, transcript variant alpha, mRNA	NM_000633	Hs.150749	NM_000633
DYNLRB2	10.5	23.3	dynein, light chain, roadblock-type 2 (DYNLRB2), mRNA	NM_130897	Hs.98849	BC035232
COL21A1	10.5	5.0	collagen, type XXI, alpha 1 (COL21A1), mRNA	NM_030820	Hs.47629	NM_030820
YAF2	10.5	6.3	YY1 associated factor 2 (YAF2), transcript variant 2, mRNA	NM_001012424	Unknown	
THC2733296	10.5	58.4	THC2733296	THC2733296	Unknown	
PON2	10.5	2.6	paraoxonase 2 (PON2), transcript variant 1, mRNA	NM_000305	Hs.530077	AK054688
ZNF503	10.5	8.4	zinc finger protein 503 (ZNF503), mRNA	NM_032772	Hs.195710	NM_032772
LONRF2	10.5	7.1	LON peptidase N-terminal domain and ring finger 2 (LONRF2), mRNA	NM_198461	Hs.21380	NM_198461
DKFZP686A01247	10.4	5.3	hypothetical protein (DKFZP686A01247), mRNA	NM_014988	Hs.335163	CR749205
FAM116A	10.4	2.3	family with sequence similarity 116, member A (FAM116A), mRNA	NM_152678	Hs.91085	NM_152678
AF289562	10.4	4.2	clone pp6337 unknown mRNA.	AF289562	Hs.276808	NM_002409
COL11A2	10.4	5.0	collagen, type XI, alpha 2 (COL11A2), transcript variant 1, mRNA	NM_080680	Hs.390171	NM_080680
LOC283874	10.4	3.6	hypothetical protein LOC283874 (LOC283874), mRNA	NM_001012731	Unknown	
SOSTDC1	10.4	6.0	sclerostin domain containing 1 (SOSTDC1), mRNA	NM_015464	Hs.648106	AK093408
RDH10	10.4	7.2	retinol dehydrogenase 10 (all-trans) (RDH10), mRNA	NM_172037	Hs.244940	BC067131
KBTBD11	10.4	2.5	kelch repeat and BTB (POZ) domain containing 11 (KBTBD11), mRNA	NM_014867	Hs.5333	AB018254
KLHL9	10.4	3.2	kelch-like 9 (Drosophila) (KLHL9), mRNA	NM_018847	Hs.522029	NM_018847
THC2672869	10.4	18.9	Q822A8_CHLCV (Q822A8) Ribonucleoside-diphosphate reductase, beta subunit, partial (5%)	THC2672869	Unknown	
KBTBD9	10.3	6.1	Kelch repeat and BTB domain-containing protein 9.	ENST00000288548	Unknown	
TSHZ2	10.3	17.8	Teashirt homolog 2 (Zinc finger protein 218) (Ovarian cancer-related protein 10-2) (OVC10-2).	ENST00000371497	Unknown	
SLC1A2	10.3	4.6	solute carrier family 1 (glial high affinity glutamate transporter), member 2 (SLC1A2), mRNA	NM_004171	Hs.502338	NM_004171
RASSF3	10.3	3.8	cDNA FLJ26410 fis, clone HRT09622.	AK129920	Unknown	
A_24_P555510	10.3	4.5	A_24_P555510	A_24_P555510	Unknown	
PHACTR3	10.3	15.2	phosphatase and actin regulator 3 (PHACTR3), transcript variant 1, mRNA	NM_080672	Hs.473218	NM_080672
WDR22	10.3	1.9	WD repeat domain 22 (WDR22), mRNA	NM_003861	Hs.509780	BC150267
AK123627	10.2	4.0	cDNA FLJ41633 fis, clone FCBBF3003435.	AK123627	Hs.387867	AK123627
THC2648398	10.2	4.1	THC2648398	THC2648398	Unknown	
MGC33212	10.2	3.0	hypothetical protein MGC33212 (MGC33212), mRNA	NM_152773	Hs.135997	BM810125
ENST00000304372	10.2	3.7	KCTD19 protein.	ENST00000304372	Unknown	
LZTFL1	10.2	4.0	leucine zipper transcription factor-like 1 (LZTFL1), mRNA	NM_020347	Hs.30824	BC042483
EYA4	10.2	41.5	Eyes absent homolog 4 (EC 3.1.3.48).	ENST00000367895	Unknown	

ZNF395	10.2	3.5	zinc finger protein 395 (ZNF395), mRNA	NM_018660	Hs.699209	NM_018660
KIAA0644	10.2	7.1	KIAA0644 gene product (KIAA0644), mRNA	NM_014817	Hs.21572	NM_014817
PRRX2	10.2	4.4	paired related homeobox 2 (PRRX2), mRNA	NM_016307	Hs.660115	BM909706
PLN	10.2	44.4	phospholamban (PLN), mRNA	NM_002667	Hs.170839	AK129844
CXorf57	10.2	3.8	chromosome X open reading frame 57 (CXorf57), mRNA	NM_018015	Hs.274267	BC070110
LOC730391	10.2	6.8	PREDICTED: hypothetical protein LOC730391 (LOC730391), mRNA	XM_001125845	Unknown	
RFX2	10.2	4.1	regulatory factor X, 2 (influences HLA class II expression) (RFX2), transcript variant 1, mRNA	NM_000635	Hs.465709	NM_000635
PCGF5	10.1	7.3	polycomb group ring finger 5 (PCGF5), mRNA	NM_032373	Hs.500512	NM_032373
CNTNAP4	10.1	50.2	cDNA FLJ30224 fis, clone BRACE2001834.	AK054786	Unknown	
ADPRHL1	10.1	9.7	ADP-ribosylhydrolase like 1 (ADPRHL1), transcript variant 2, mRNA	NM_199162	Hs.98669	NM_199162
CNTN4	10.1	32.0	contactin 4 (CNTN4), transcript variant 1, mRNA	NM_175607	Hs.298705	NM_175607
KIAA1545	10.1	3.9	mRNA for KIAA1545 protein, partial cds.	AB046765	Hs.411138	XM_495939
PCAF	10.1	6.6	p300/CBP-associated factor (PCAF), mRNA	NM_003884	Hs.533055	NM_003884
G31710	10.1	8.9	sWSS2257 Eric D. Green STS cDNA, sequence tagged site.	G31710	Unknown	
THC2772589	10.1	2.5	Q5NNJ5_ZYMMO (Q5NNJ5) DNA polymerase III delta prime subunit , partial (5%)	THC2772589	Unknown	
KIAA1407	10.1	9.2	KIAA1407 (KIAA1407), mRNA	NM_020817	Hs.477159	AF509494
THC2613107	10.1	2.8	Q8NH31_HUMAN (Q8NH31) Seven transmembrane helix receptor, partial (5%)	THC2613107	Unknown	
THC2491396	10.1	2.4	CB133932 K-EST0185164 L9SNU354 cDNA clone L9SNU354-10-C01 5', mRNA sequence	THC2491396	Unknown	
SERINC1	10.1	2.7	serine incorporator 1 (SERINC1), mRNA	NM_020755	Hs.146668	AF087902
C4orf19	10.1	3.4	chromosome 4 open reading frame 19 (C4orf19), mRNA	NM_018302	Hs.107527	AK001879
AK092875	10.0	8.8	cDNA FLJ35556 fis, clone SPLEN2004844.	AK092875	Hs.31037	AK092875
FGF20	10.0	5.5	fibroblast growth factor 20 (FGF20), mRNA	NM_019851	Hs.199905	NM_019851
FLJ13236	10.0	4.6	hypothetical protein FLJ13236 (FLJ13236), mRNA	NM_024902	Hs.659300	AK055747
AL833456	10.0	6.3	mRNA; cDNA DKFZp686I18116 (from clone DKFZp686I18116).	AL833456	Hs.31474	CR749529
AK022936	10.0	2.6	cDNA FLJ12874 fis, clone NT2RP2003769.	AK022936	Hs.594424	AK022936
RBMS3	10.0	8.2	RNA binding motif, single stranded interacting protein (RBMS3), transcript variant 2, mRNA	NM_014483	Hs.696468	AL831860
BVES	10.0	2.3	blood vessel epicardial substance (BVES), transcript variant 5, mRNA	NM_147147	Hs.221660	BC040502
LGALS2	10.0	11.3	lectin, galactoside-binding, soluble, 2 (galectin 2) (LGALS2), mRNA	NM_006498	Hs.531776	AK130682
EPHB3	9.9	8.3	EPH receptor B3 (EPHB3), mRNA	NM_004443	Hs.2913	NM_004443
FLJ10081	9.9	2.0	hypothetical protein FLJ10081 (FLJ10081), mRNA	NM_017991	Hs.516341	NM_017991
PDGFRA	9.9	17.4	platelet-derived growth factor receptor, alpha polypeptide (PDGFRA), mRNA	NM_006206	Hs.74615	NM_006206
RKHD3	9.9	4.6	ring finger and KH domain containing 3 (RKHD3), mRNA	NM_032246	Hs.104744	AK131424
DCAMKL2	9.9	5.4	doublecortin and CaM kinase-like 2 (DCAMKL2), transcript variant 1, mRNA	NM_001040260	Hs.591683	AB209181
BF446608	9.9	2.8	7q89f08.x1 NCI_CGAP_Lu24 cDNA clone IMAGE:3705734 3', mRNA sequence	BF446608	Hs.701411	BF446608
BQ064481	9.9	3.7	AGENCOURT_6853651 NIH_MGC_99 cDNA clone IMAGE:5926217 5', mRNA sequence	BQ064481	Hs.223583	BQ064481
ZNRF1	9.9	2.2	zinc and ring finger 1 (ZNRF1), mRNA	NM_032268	Hs.427284	NM_032268
ZBTB1	9.9	6.1	zinc finger and BTB domain containing 1 (ZBTB1), mRNA	NM_014950	Hs.655536	AB023214
RFTN2	9.9	22.4	raftlin family member 2 (RFTN2), mRNA	NM_144629	Hs.591615	BC111069
ARHGAP6	9.9	13.8	Rho GTPase activating protein 6 (ARHGAP6), transcript variant 2, mRNA	NM_001174	Hs.435291	AB208792
IFT57	9.9	2.3	intraflagellar transport 57 homolog (Chlamydomonas) (IFT57), mRNA	NM_018010	Hs.412196	NM_018010
ST8SIA1	9.9	6.7	ST8 alpha-N-acetyl-neuraminide alpha-2,8-sialyltransferase 1 (ST8SIA1), mRNA	NM_003034	Hs.408614	NM_003034
DDC	9.9	10.8	dopa decarboxylase (aromatic L-amino acid decarboxylase) (DDC), mRNA	NM_000790	Hs.359698	NM_001082971
MBD2	9.9	18.3	methyl-CpG binding domain protein 2 (MBD2), transcript variant 1, mRNA	NM_003927	Hs.25674	NM_003927
CDR1	9.9	7.9	cDNA FLJ30359 fis, clone BRACE2007760, highly similar to 40S RIBOSOMAL PROTEIN S15A.	AK054921	Hs.571748	AK054921
BC039457	9.9	2.7	cDNA clone IMAGE:5312122.	BC039457	Hs.406492	AK001640
LRRIQ1	9.9	5.2	leucine-rich repeats and IQ motif containing 1 (LRRIQ1), mRNA	NM_032165	Hs.402200	NM_001079910
C4orf31	9.9	11.2	chromosome 4 open reading frame 31 (C4orf31), mRNA	NM_024574	Hs.90250	NM_024574
EPHA3	9.8	59.4	EPH receptor A3 (EPHA3), transcript variant 1, mRNA	NM_005233	Hs.123642	NM_005233
LEF1	9.8	14.9	lymphoid enhancer-binding factor 1 (LEF1), mRNA	NM_016269	Hs.555947	AK128255
PDE11A	9.8	24.8	phosphodiesterase 11A (PDE11A), transcript variant 4, mRNA	NM_016953	Hs.570273	NM_016953
RAB7B	9.8	6.9	RAB7B, member RAS oncogene family (RAB7B), mRNA	NM_177403	Hs.534612	NM_177403
FLJ10038	9.8	2.4	cDNA FLJ10038 fis, clone HEMBA1000971.	AK000900	Hs.511316	BQ061333

ENST00000299694	9.8	5.7	BEAN protein (Fragment).	ENST00000299694	Unknown	
THC2781239	9.8	17.4	THC2781239	THC2781239	Unknown	
APOA2	9.8	13.6	apolipoprotein A-II (APOA2), mRNA	NM_001643	Hs.237658	CR619946
TLX2	9.8	7.3	T-cell leukemia homeobox 2 (TLX2), mRNA	NM_016170	Hs.168586	BC006396
ASCL1	9.8	4.5	achaete-scute complex homolog 1 (Drosophila) (ASCL1), mRNA	NM_004316	Hs.700574	NM_004316
NDN	9.8	3.6	necdin homolog (mouse) (NDN), mRNA	NM_002487	Hs.50130	NM_002487
C6orf89	9.8	3.2	chromosome 6 open reading frame 89 (C6orf89), mRNA	NM_152734	Hs.433381	NM_152734
MYOZ2	9.8	7.3	myozenin 2 (MYOZ2), mRNA	NM_016599	Hs.381047	NM_016599
RNF150	9.8	6.5	ring finger protein 150 (RNF150), mRNA	NM_020724	Hs.659104	AB033040
C3orf15	9.7	6.7	chromosome 3 open reading frame 15 (C3orf15), mRNA	NM_033364	Hs.341906	NM_033364
PROS1	9.7	14.8	protein S (alpha) (PROS1), mRNA	NM_000313	Hs.64016	M14338
CR602022	9.7	3.4	full-length cDNA clone CS0DJ004YB09 of T cells (Jurkat cell line) Cot 10-normalized of (human).	CR602022	Hs.90286	BC041837
RECK	9.7	5.4	reversion-inducing-cysteine-rich protein with kazal motifs (RECK), mRNA	NM_021111	Hs.388918	BX648668
AK026099	9.7	9.2	cDNA: FLJ22446 fis, clone HRC09457.	AK026099	Hs.671998	AK026099
C14orf45	9.7	6.9	chromosome 14 open reading frame 45 (C14orf45), mRNA	NM_025057	Hs.644621	BC044808
SIX1	9.7	15.5	sine oculis homeobox homolog 1 (Drosophila) (SIX1), mRNA	NM_005982	Hs.633506	AK093780
FLJ90013	9.7	2.4	hypothetical protein FLJ90013 (FLJ90013), mRNA	NM_153365	Hs.479223	NM_153365
ODZ2	9.7	7.0	Teneurin-2 (Ten-2) (Tenascin-M2) (Ten-m2) (Protein Odd Oz/ten-m homolog 2).	ENST00000314238	Unknown	
IL11RA	9.7	7.2	interleukin 11 receptor, alpha (IL11RA), transcript variant 1, mRNA	NM_004512	Hs.591088	BC003110
TXNDC13	9.7	4.5	Thioredoxin domain-containing protein 13 precursor.	ENST00000246024	Unknown	
ASB2	9.7	9.3	ankyrin repeat and SOCS box-containing 2 (ASB2), mRNA	NM_016150	Hs.510327	NM_016150
C10orf32	9.6	5.4	chromosome 10 open reading frame 32 (C10orf32), mRNA	NM_144591	Hs.34492	NM_020682
USP6NL	9.6	4.4	USP6 N-terminal like, mRNA (cDNA clone IMAGE:4047207), partial cds.	BC010351	Hs.498661	XM_927409
LOC339745	9.6	2.3	hypothetical protein LOC339745 (LOC339745), mRNA	NM_001001664	Hs.333297	BC071613
TNFRSF19	9.6	12.2	tumor necrosis factor receptor superfamily, member 19 (TNFRSF19), transcript variant 2, mRNA	NM_148957	Hs.149168	NM_148957
LOC619208	9.6	2.7	hypothetical protein LOC619208 (LOC619208), mRNA	NM_001033564	Hs.591340	NM_001033564
ALDH5A1	9.6	3.6	aldehyde dehydrogenase 5 family, member A1 (succinate-semialdehyde dehydrogenase) (ALDH5A1), nuclear gene encoding mitochondrial protein, transcript variant 1, mRNA	NM_170740	Hs.371723	NM_170740
NAV2	9.6	10.8	HELAD1S mRNA for helicase, complete cds.	AB063115	Hs.502116	NM_182964
THC2651324	9.6	12.4	THC2651324	THC2651324	Unknown	
SSPN	9.6	4.6	sarcospan (Kras oncogene-associated gene) (SSPN), mRNA	NM_005086	Hs.183428	NM_005086
ANKRD46	9.6	2.4	ankyrin repeat domain 46 (ANKRD46), mRNA	NM_198401	Hs.530199	BC035087
COLQ	9.6	7.2	collagen-like tail subunit (single strand of homotrimer) of asymmetric acetylcholinesterase (COLQ), transcript variant II, mRNA	NM_080538	Hs.146735	NM_080538
F2	9.6	15.5	coagulation factor II (thrombin) (F2), mRNA	NM_000506	Hs.655207	BC051332
FLJ10159	9.6	3.5	hypothetical protein FLJ10159 (FLJ10159), mRNA	NM_018013	Hs.445244	NM_018013
PCDH20	9.6	9.7	protocadherin 20 (PCDH20), mRNA	NM_022843	Hs.391781	AL833830
LOC645722	9.6	15.2	PREDICTED: hypothetical LOC645722 (LOC645722), mRNA	XM_944447	Unknown	
NCOA6	9.6	3.8	nuclear receptor coactivator 6 (NCOA6), mRNA	NM_014071	Hs.368971	AF208227
C1RL	9.5	2.7	complement component 1, r subcomponent-like (C1RL), mRNA	NM_016546	Hs.631730	AF178985
GUCY1A3	9.5	10.1	guanylate cyclase 1, soluble, alpha 3 (GUCY1A3), mRNA	NM_000856	Hs.24258	BX647200
BC036599	9.5	27.5	cDNA clone IMAGE:5273964.	BC036599	Hs.24321	AK022090
PDGFRB	9.5	7.6	platelet-derived growth factor receptor, beta polypeptide (PDGFRB), mRNA	NM_002609	Hs.509067	BC032224
F2	9.5	17.0	coagulation factor II (thrombin) (F2), mRNA	NM_000506	Hs.655207	BC051332
F2	9.5	16.6	coagulation factor II (thrombin) (F2), mRNA	NM_000506	Hs.655207	BC051332
RABL2A	9.5	2.6	RAB, member of RAS oncogene family-like 2A (RABL2A), transcript variant 1, mRNA	NM_013412	Hs.446425	AK092971
NRIP1	9.5	5.6	nuclear receptor interacting protein 1 (NRIP1), mRNA	NM_003489	Hs.155017	NM_003489
ANTXR1	9.5	2.5	anthrax toxin receptor 1 (ANTXR1), transcript variant 1, mRNA	NM_032208	Hs.165859	AF279145
THC2657781	9.5	8.5	THC2657781	THC2657781	Unknown	
TXNDC13	9.5	3.1	thioredoxin domain containing 13 (TXNDC13), mRNA	NM_021156	Hs.169358	BC044777
RTDR1	9.5	3.2	rhabdoid tumor deletion region gene 1 (RTDR1), mRNA	NM_014433	Hs.526920	NM_014433
AK055501	9.5	4.7	cDNA FLJ30939 fis, clone FEBRA2007414.	AK055501	Hs.381219	BC081565
UCRC	9.5	3.2	ubiquinol-cytochrome c reductase complex (7.2 kD) (UCRC), transcript variant 2, mRNA	NM_001003684	Hs.284292	BF965131
BNIP3L	9.5	4.7	BCL2/adenovirus E1B 19kDa interacting protein 3-like (BNIP3L), mRNA	NM_004331	Hs.131226	AF370457

KIAA1856	9.5	5.8	cDNA FLJ14435 fis, clone HEMBA1007085.	AK027341	Hs.556754	NM_001080495
KCNJ8	9.5	11.1	potassium inwardly-rectifying channel, subfamily J, member 8 (KCNJ8), mRNA	NM_004982	Hs.102308	NM_004982
SEMA3D	9.5	4.7	sema domain, immunoglobulin domain (Ig), short basic domain, secreted, (semaphorin) 3D (SEMA3D), mRNA	NM_152754	Hs.201340	NM_152754
ENST00000265149	9.5	7.9	mRNA for KIAA1546 protein, partial cds.	ENST00000265149	Unknown	
THC2606573	9.5	3.8	AY151386 NAP1 {Homo sapiens} (exp=-1; wgp=0; cg=0), partial (35%)	THC2606573	Unknown	
ZNF25	9.4	21.1	zinc finger protein 25 (ZNF25), mRNA	NM_145011	Hs.499429	AL834125
AB002449	9.4	2.3	mRNA from chromosome 5q21-22, clone:843Ex.	AB002449	Hs.699267	AB002449
BX100171	9.4	9.3	BX100171 Soares_testis_NHT cDNA clone IMAGp998C011795, mRNA sequence	BX100171	Hs.569103	BX100171
ADAMTS3	9.4	6.8	ADAM metallopeptidase with thrombospondin type 1 motif, 3 (ADAMTS3), mRNA	NM_014243	Hs.590919	NM_014243
THC2631150	9.4	3.2	THC2631150	THC2631150	Unknown	
AGTR1	9.4	15.7	angiotensin II receptor, type 1 (AGTR1), transcript variant 4, mRNA	NM_031850	Hs.477887	NM_031850
FNDC3A	9.4	2.9	fibronectin type III domain containing 3A (FNDC3A), transcript variant 1, mRNA	NM_001079673	Hs.508010	NM_001079673
AK123107	9.4	4.7	cDNA FLJ41112 fis, clone BRACE1000239.	AK123107	Hs.558076	AK123107
FLJ31818	9.4	4.6	hypothetical protein FLJ31818 (FLJ31818), mRNA	NM_152556	Hs.489734	NM_152556
COL11A1	9.4	8.0	collagen, type XI, alpha 1 (COL11A1), transcript variant B, mRNA	NM_080629	Hs.523446	NM_080629
PLAT	9.4	10.2	plasminogen activator, tissue (PLAT), transcript variant 1, mRNA	NM_000930	Hs.491582	BX641021
PLG	9.4	10.4	plasminogen (PLG), mRNA	NM_000301	Hs.143436	CR749293
USP34	9.4	2.4	mRNA; cDNA DKFZp586J101 (from clone DKFZp586J101).	AL050376	Unknown	
AF275804	9.4	4.3	PNAS-108 mRNA, partial sequence.	AF275804	Hs.237396	BQ431041
DAZAP2	9.4	3.3	DAZ associated protein 2 (DAZAP2), mRNA	NM_014764	Hs.369761	AK125855
GJB2	9.4	7.3	gap junction protein, beta 2, 26kDa (connexin 26) (GJB2), mRNA	NM_004004	Hs.591234	NM_004004
PLAT	9.4	10.2	plasminogen activator, tissue (PLAT), transcript variant 1, mRNA	NM_000930	Hs.491582	BX641021
EYA1	9.4	17.9	eyes absent homolog 1 (Drosophila) (EYA1), transcript variant 3, mRNA	NM_000503	Hs.491997	NM_000503
F2	9.3	17.1	coagulation factor II (thrombin) (F2), mRNA	NM_000506	Hs.655207	BC051332
FLJ40330	9.3	3.9	mRNA; cDNA DKFZp686O04183 (from clone DKFZp686O04183).	BX648045	Hs.105323	BX537549
MYOM2	9.3	9.2	myomesin (M-protein) 2, 165kDa (MYOM2), mRNA	NM_003970	Hs.443683	NM_003970
PXMP2	9.3	2.8	peroxisomal membrane protein 2, 22kDa (PXMP2), mRNA	NM_018663	Hs.430299	BM454192
PCDH17	9.3	7.7	protocadherin 17 (PCDH17), mRNA	NM_001040429	Hs.106511	NM_001040429
A_32_P93584	9.3	16.7	A_32_P93584	A_32_P93584	Unknown	
TWF1	9.3	2.5	twinfilin, actin-binding protein, homolog 1 (Drosophila) (TWF1), mRNA	NM_002822	Hs.189075	AK127868
ASXL1	9.3	2.8	additional sex combs like 1 (Drosophila) (ASXL1), mRNA	NM_015338	Hs.374043	NM_015338
CDON	9.3	3.1	Cdon homolog (mouse) (CDON), mRNA	NM_016952	Hs.38034	NM_016952
PLAT	9.3	10.2	plasminogen activator, tissue (PLAT), transcript variant 1, mRNA	NM_000930	Hs.491582	BX641021
TPO	9.3	5.7	thyroid peroxidase (TPO), transcript variant 1, mRNA	NM_000547	Hs.467554	AB208960
HERPUD2	9.3	4.1	HERPUD family member 2, mRNA (cDNA clone IMAGE:4821170).	BC049371	Hs.599851	BC035153
MSRB3	9.3	13.5	methionine sulfoxide reductase B3 (MSRB3), transcript variant 2, mRNA	NM_001031679	Hs.339024	NM_001031679
MXRA5	9.3	5.3	matrix-remodelling associated 5 (MXRA5), mRNA	NM_015419	Hs.369422	NM_015419
PLAT	9.3	10.0	plasminogen activator, tissue (PLAT), transcript variant 1, mRNA	NM_000930	Hs.491582	BX641021
TNS3	9.3	3.5	tensin 3 (TNS3), mRNA	NM_022748	Hs.520814	NM_022748
USP30	9.3	3.6	mRNA; cDNA DKFZp547N1013 (from clone DKFZp547N1013).	AL834278	Hs.486434	AL834278
ZADH2	9.3	4.1	zinc binding alcohol dehydrogenase, domain containing 2 (ZADH2), mRNA	NM_175907	Hs.591065	NM_175907
THC2656116	9.3	4.0	THC2656116	THC2656116	Unknown	
ZNF688	9.3	2.5	zinc finger protein 688 (ZNF688), transcript variant 1, mRNA	NM_145271	Hs.301463	AK122680
PLAT	9.3	10.1	plasminogen activator, tissue (PLAT), transcript variant 1, mRNA	NM_000930	Hs.491582	BX641021
SEPP1	9.3	5.0	selenoprotein P, plasma, 1 (SEPP1), mRNA	NM_005410	Hs.700640	BC030009
C20orf108	9.3	4.2	chromosome 20 open reading frame 108 (C20orf108), mRNA	NM_080821	Hs.143736	NM_080821
WASF1	9.2	3.8	WAS protein family, member 1 (WASF1), transcript variant 1, mRNA	NM_003931	Hs.75850	NM_003931
PLAT	9.2	10.4	plasminogen activator, tissue (PLAT), transcript variant 1, mRNA	NM_000930	Hs.491582	BX641021
TTC28	9.2	2.9	cDNA FLJ35019 fis, clone OCBBF2014541.	AK092338	Hs.387856	XM_929318
ENST00000320378	9.2	6.4	Epithelial membrane protein 2 (EMP-2) (Protein XMP).	ENST00000320378	Unknown	
ACF	9.2	3.4	apobec-1 complementation factor (ACF), transcript variant 3, mRNA	NM_138933	Hs.499643	NM_138933
BC004287	9.2	12.7	Homo sapiens, clone IMAGE:3618365, mRNA.	BC004287	Hs.434957	BC004287
AK092715	9.2	9.3	cDNA FLJ35396 fis, clone SKNSH2003483.	AK092715	Hs.26409	BC041405
FLJ42875	9.2	17.5	mRNA; cDNA DKFZp666E184 (from clone DKFZp666E184).	AL833006	Hs.531041	AL832943

GAMT	9.2	3.2	guanidinoacetate N-methyltransferase (GAMT), transcript variant 1, mRNA	NM_000156	Hs.81131	BM928125
F2	9.2	15.0	coagulation factor II (thrombin) (F2), mRNA	NM_000506	Hs.655207	BC051332
THC2651751	9.2	4.4	THC2651751	THC2651751	Unknown	
AGT	9.2	23.9	angiotensinogen (serpin peptidase inhibitor, clade A, member 8) (AGT), mRNA	NM_000029	Hs.19383	NM_000029
PLAT	9.2	10.1	plasminogen activator, tissue (PLAT), transcript variant 1, mRNA	NM_000930	Hs.491582	BX641021
C20orf132	9.2	6.1	chromosome 20 open reading frame 132 (C20orf132), transcript variant 1, mRNA	NM_152503	Hs.349125	BC030006
HRC	9.2	3.8	histidine rich calcium binding protein (HRC), mRNA	NM_002152	Hs.436885	NM_002152
LOC440934	9.2	9.9	full-length cDNA clone CS0DC029YA18 of Neuroblastoma Cot 25-normalized of (human).	CR593560	Hs.238964	CR593560
THC2641682	9.2	22.7	Q2H7G5_CHAGB (Q2H7G5) Predicted protein, partial (5%)	THC2641682	Unknown	
ARL13B	9.2	2.8	ADP-ribosylation factor-like 13B (ARL13B), transcript variant 1, mRNA	NM_182896	Hs.533086	CR936763
PLAC1	9.2	4.3	placenta-specific 1 (PLAC1), mRNA	NM_021796	Hs.496811	BC022335
ELAVL4	9.1	2.9	ELAV (embryonic lethal, abnormal vision, Drosophila)-like 4 (Hu antigen D) (ELAVL4), mRNA	NM_021952	Hs.213050	BC036071
THC2632909	9.1	26.1	Q6E5T4_FUGRU (Q6E5T4) Claudin 2, partial (5%)	THC2632909	Unknown	
BC040991	9.1	2.6	cDNA clone IMAGE:4817695.	BC040991	Hs.379018	U18422
MEOX1	9.1	4.4	mesenchyme homeobox 1 (MEOX1), transcript variant 1, mRNA	NM_004527	Hs.438	NM_004527
RAB33A	9.1	4.8	RAB33A, member RAS oncogene family (RAB33A), mRNA	NM_004794	Hs.654356	AK094927
CCDC46	9.1	3.3	coiled-coil domain containing 46 (CCDC46), transcript variant 1, mRNA	NM_145036	Hs.408676	NM_145036
AK021531	9.1	33.9	cDNA FLJ11469 fis, clone HEMBA1001658.	AK021531	Hs.443625	NM_000090
NANOS3	9.1	4.4	PREDICTED: nanos homolog 3 (Drosophila) (NANOS3), misc RNA	XR_017750	Unknown	
LHFP	9.1	7.6	lipoma HMGIC fusion partner (LHFP), mRNA	NM_005780	Hs.507798	NM_005780
AK002023	9.1	3.2	cDNA FLJ11161 fis, clone PLACE1007021.	AK002023	Hs.368518	AK002023
THC2646608	9.1	5.3	ALU7_HUMAN (P39194) Alu subfamily SQ sequence contamination warning entry, partial (19%)	THC2646608	Unknown	
PRPH	9.1	4.3	peripherin (PRPH), mRNA	NM_006262	Hs.37044	AK125587
BQ354462	9.1	18.2	BQ354462 CM2-HT0970-131100-502-h12 HT0970 cDNA, mRNA sequence	BQ354462	Unknown	
AF143325	9.1	4.1	clone IMAGE:110436 mRNA sequence.	AF143325	Hs.655976	AF143325
BQ050540	9.1	2.3	AGENCOURT_6808794 NIH_MGC_71 cDNA clone IMAGE:5784515 5', mRNA sequence	BQ050540	Hs.26579	BQ050540
CCRK	9.1	5.2	cell cycle related kinase (CCRK), transcript variant 3, mRNA	NM_001039803	Hs.522274	AK226135
AMBP	9.0	34.7	alpha-1-microglobulin/bikunin precursor (AMBP), mRNA	NM_001633	Hs.436911	NM_001633
ZNF575	9.0	4.2	zinc finger protein 575 (ZNF575), mRNA	NM_174945	Hs.213534	AK057129
THC2664831	9.0	4.3	THC2664831	THC2664831	Unknown	
PLAT	9.0	10.0	plasminogen activator, tissue (PLAT), transcript variant 1, mRNA	NM_000930	Hs.491582	BX641021
KIAA1546	9.0	4.9	KIAA1546 (KIAA1546), mRNA	NM_017628	Hs.367639	BX640738
ENST00000377116	9.0	3.5	NEDD4 family-interacting protein 2 (NEDD4 WW domain-binding protein 5A) (Putative MAPK-activating protein PM04/PM05/PM06/PM07) (Putative NF-kappa-B-activating protein 413).	ENST00000377116	Unknown	
MAN2A2	9.0	2.6	mannosidase, alpha, class 2A, member 2 (MAN2A2), mRNA	NM_006122	Hs.116459	NM_006122
PKNOX2	9.0	4.6	PBX/knotted 1 homeobox 2 (PKNOX2), mRNA	NM_022062	Hs.696454	AK023792
THC2505770	9.0	2.3	THC2505770	THC2505770	Unknown	
FOXP1	9.0	7.6	forkhead box P1 (FOXP1), transcript variant 1, mRNA	NM_032682	Hs.431498	NM_032682
ATBF1	9.0	8.0	AT-binding transcription factor 1 (ATBF1), mRNA	NM_006885	Hs.652666	NM_006885
THC2663329	9.0	11.1	THC2663329	THC2663329	Unknown	
IFIT1	9.0	4.2	interferon-induced protein with tetratricopeptide repeats 1 (IFIT1), transcript variant 2, mRNA	NM_001548	Hs.20315	AK095515
SPON1	9.0	4.9	spondin 1, extracellular matrix protein (SPON1), mRNA	NM_006108	Hs.643864	NM_006108
LRP12	8.9	2.7	low density lipoprotein-related protein 12 (LRP12), mRNA	NM_013437	Hs.654804	CR749434
C20orf119	8.9	4.3	cDNA: FLJ23107 fis, clone LNG07738.	AK026760	Hs.641481	AK124047
GZF1	8.9	2.4	GDNF-inducible zinc finger protein 1 (GZF1), mRNA	NM_022482	Hs.699339	NM_022482
C6orf204	8.9	3.1	C6orf204 protein (Fragment).	ENST00000368491	Unknown	
SLC17A6	8.9	6.3	solute carrier family 17 (sodium-dependent inorganic phosphate cotransporter), member 6 (SLC17A6), mRNA	NM_020346	Hs.242821	AB032435
THC2760755	8.9	4.9	THC2760755	THC2760755	Unknown	
SGCG	8.9	13.5	sarcoglycan, gamma (35kDa dystrophin-associated glycoprotein) (SGCG), mRNA	NM_000231	Hs.37167	NM_000231
SENP7	8.9	6.1	SUMO1/sentrin specific peptidase 7 (SENP7), transcript variant 1, mRNA	NM_020654	Hs.529551	NM_020654
ARHGAP28	8.9	3.5	Rho GTPase activating protein 28 (ARHGAP28), transcript variant 1, mRNA	NM_001010000	Hs.183114	NM_001010000
GARNL3	8.9	5.6	GTPase activating Rap/RanGAP domain-like 3 (GARNL3), mRNA	NM_032293	Hs.29304	AK126704

NPNT	8.9	14.6	nephronectin (NPNT), mRNA	NM_001033047	Hs.518921	NM_001033047
SPOP	8.9	2.5	speckle-type POZ protein (SPOP), transcript variant 1, mRNA	NM_001007226	Hs.463382	AK125087
NETO2	8.9	2.6	neuropilin (NRP) and tolloid (TLL)-like 2 (NETO2), mRNA	NM_018092	Hs.444046	NM_018092
GLRB	8.9	9.3	glycine receptor, beta (GLRB), mRNA	NM_000824	Hs.32973	NM_000824
AK125038	8.9	4.6	cDNA FLJ43048 fis, clone BRTHA3004502.	AK125038	Hs.593760	AB007969
C5orf21	8.9	2.7	chromosome 5 open reading frame 21 (C5orf21), mRNA	NM_032042	Hs.655651	NM_032042
NR2F2	8.9	10.2	nuclear receptor subfamily 2, group F, member 2 (NR2F2), mRNA	NM_021005	Hs.347991	BC042897
DNER	8.9	5.7	delta/notch-like EGF repeat containing (DNER), mRNA	NM_139072	Hs.234074	BC035009
TBC1D19	8.9	2.8	TBC1 domain family, member 19 (TBC1D19), mRNA	NM_018317	Hs.479403	AK001944
THC2648227	8.9	2.6	THC2648227	THC2648227	Unknown	
AGT	8.9	24.7	angiotensinogen (serpin peptidase inhibitor, clade A, member 8) (AGT), mRNA	NM_000029	Hs.19383	NM_000029
MDFIC	8.9	5.7	MyoD family inhibitor domain containing (MDFIC), mRNA	NM_199072	Hs.427236	NM_199072
LOC440905	8.9	3.7	PREDICTED: hypothetical protein LOC440905, transcript variant 4 (LOC440905), mRNA	XM_943718	Unknown	
MOXD1	8.9	7.8	monooxygenase, DBH-like 1 (MOXD1), transcript variant 2, mRNA	NM_015529	Hs.6909	NM_015529
THC2729213	8.9	3.6	2103155A neuronal apoptosis inhibitory protein. {Homo sapiens} (exp=-1; wgp=0; cg=0), partial (11%)	THC2729213	Unknown	
TCEB1	8.9	1.8	Transcription elongation factor B polypeptide 1 (RNA polymerase II transcription factor SIII subunit C) (SIII p15) (Elongin-C) (EloC) (Elongin 15 kDa subunit).	ENST00000284811	Unknown	
LOC150759	8.8	4.0	cDNA FLJ33034 fis, clone THYMU2000236.	AK057596	Hs.700794	AK057596
WNT3A	8.8	10.4	wingless-type MMTV integration site family, member 3A (WNT3A), mRNA	NM_033131	Hs.336930	NM_033131
CR601260	8.8	5.7	full-length cDNA clone CS0DM001YA20 of Fetal liver of (human).	CR601260	Hs.291319	CR627122
TDO2	8.8	10.0	tryptophan 2,3-dioxygenase (TDO2), mRNA	NM_005651	Hs.183671	BX647341
AKAP11	8.8	2.4	A kinase (PRKA) anchor protein 11 (AKAP11), transcript variant 1, mRNA	NM_016248	Hs.105105	AF176555
AGT	8.8	25.4	angiotensinogen (serpin peptidase inhibitor, clade A, member 8) (AGT), mRNA	NM_000029	Hs.19383	NM_000029
TTN	8.8	22.6	titin (TTN), transcript variant N2-A, mRNA	NM_133378	Hs.654592	NM_133378
RFX3	8.8	4.5	Transcription factor RFX3.	ENST00000382004	Unknown	
GNG2	8.8	4.5	guanine nucleotide binding protein (G protein), gamma 2 (GNG2), mRNA	NM_053064	Hs.695989	NM_053064
ENO3	8.8	3.6	enolase 3 (beta, muscle) (ENO3), transcript variant 1, mRNA	NM_001976	Hs.224171	NM_001976
LRRC27	8.8	7.2	leucine rich repeat containing 27 (LRRC27), mRNA	NM_030626	Hs.119897	NM_030626
KIAA1456	8.8	10.9	KIAA1456 protein (KIAA1456), mRNA	NM_020844	Hs.202521	NM_020844
LCOR	8.8	2.7	ligand dependent nuclear receptor corepressor (LCOR), mRNA	NM_032440	Hs.500695	AL834245
BMI1	8.8	5.3	B lymphoma Mo-MLV insertion region (mouse) (BMI1), mRNA	NM_005180	Hs.496613	NM_005180
BM802662	8.8	19.3	AGENCOURT_6460073 NIH_MGC_67 cDNA clone IMAGE:5581075 5', mRNA sequence	BM802662	Hs.586812	BM802662
PLAGL1	8.8	26.2	pleiomorphic adenoma gene-like 1 (PLAGL1), transcript variant 2, mRNA	NM_006718	Hs.444975	CR749329
NOTCH4	8.8	3.6	Notch homolog 4 (Drosophila) (NOTCH4), mRNA	NM_004557	Hs.436100	NM_004557
CREB5	8.8	9.8	cAMP responsive element binding protein 5 (CREB5), transcript variant 1, mRNA	NM_182898	Hs.437075	NM_182898
AK024399	8.8	8.1	cDNA FLJ14337 fis, clone PLACE4000494.	AK024399	Hs.505141	AK024399
AF088033	8.8	2.2	full length insert cDNA clone ZC24E10.	AF088033	Hs.632066	CR936659
CCDC121	8.8	2.7	coiled-coil domain containing 121 (CCDC121), mRNA	NM_024584	Hs.21081	AK125354
AF086375	8.8	5.5	full length insert cDNA clone ZD68B12.	AF086375	Hs.264606	BG571805
FAM92A1	8.8	2.1	mRNA; cDNA DKFZp564I0278 (from clone DKFZp564I0278).	CR627475	Unknown	
SMPDL3A	8.8	5.6	sphingomyelin phosphodiesterase, acid-like 3A (SMPDL3A), mRNA	NM_006714	Hs.486357	AK096144
RFTN1	8.8	5.3	raftlin, lipid raft linker 1 (RFTN1), mRNA	NM_015150	Hs.98910	NM_015150
C1orf54	8.8	2.3	chromosome 1 open reading frame 54 (C1orf54), mRNA	NM_024579	Hs.91283	BQ718781
THC2668193	8.8	4.0	THC2668193	THC2668193	Unknown	
PLAT	8.7	9.8	plasminogen activator, tissue (PLAT), transcript variant 1, mRNA	NM_000930	Hs.491582	BX641021
RAB22A	8.7	2.3	RAB22A, member RAS oncogene family (RAB22A), mRNA	NM_020673	Hs.529044	NM_020673
PLAT	8.7	9.9	plasminogen activator, tissue (PLAT), transcript variant 1, mRNA	NM_000930	Hs.491582	BX641021
KLF3	8.7	4.2	Krueppel-like factor 3 (Basic krueppel-like factor) (CACCC-box-binding protein BKLF) (TEF-2).	ENST00000381956	Unknown	
ACTN2	8.7	29.3	actinin, alpha 2 (ACTN2), mRNA	NM_001103	Hs.498178	NM_001103
AK021543	8.7	55.2	cDNA FLJ11481 fis, clone HEMBA1001803.	AK021543	Hs.584880	AK021543
VPS35	8.7	1.9	vacuolar protein sorting 35 homolog (S. cerevisiae) (VPS35), mRNA	NM_018206	Hs.696029	AK025774
LOC284244	8.7	16.1	clones 24714 and 24715 mRNA sequence.	AF070541	Hs.4267	AK055244
AGT	8.7	26.6	angiotensinogen (serpin peptidase inhibitor, clade A, member 8) (AGT), mRNA	NM_000029	Hs.19383	NM_000029

AGT	8.7	27.3	angiotensinogen (serpin peptidase inhibitor, clade A, member 8) (AGT), mRNA	NM_000029	Hs.19383	NM_000029
GPR124	8.7	6.9	G protein-coupled receptor 124 (GPR124), mRNA	NM_032777	Hs.699314	NM_032777
SMOC2	8.7	19.7	SPARC related modular calcium binding 2 (SMOC2), mRNA	NM_022138	Hs.487200	BC028420
SLC12A2	8.7	2.2	solute carrier family 12 (sodium/potassium/chloride transporters), member 2 (SLC12A2), mRNA	NM_001046	Hs.162585	NM_001046
SCRN3	8.7	1.9	secernin 3 (SCRN3), mRNA	NM_024583	Hs.470679	NM_024583
AGT	8.7	22.9	angiotensinogen (serpin peptidase inhibitor, clade A, member 8) (AGT), mRNA	NM_000029	Hs.19383	NM_000029
GATA6	8.7	11.9	GATA binding protein 6 (GATA6), mRNA	NM_005257	Hs.514746	X95701
MITF	8.7	8.3	microphthalmia-associated transcription factor (MITF), transcript variant 1, mRNA	NM_198159	Hs.166017	NM_198159
ENST00000381298	8.7	7.5	Interleukin-6 receptor subunit beta precursor (IL-6R-beta) (Interleukin-6 signal transducer) (Membrane glycoprotein 130) (gp130) (Oncostatin-M receptor alpha subunit) (CD130 antigen) (CDw130).	ENST00000381298	Unknown	
THC2607337	8.7	6.5	THC2607337	THC2607337	Unknown	
CNTNAP4	8.7	10.9	contactin associated protein-like 4 (CNTNAP4), transcript variant 1, mRNA	NM_033401	Hs.461389	NM_033401
THC2582897	8.6	2.3	THC2582897	THC2582897	Unknown	
FLJ45187	8.6	4.1	hypothetical protein LOC387640 (FLJ45187), mRNA	NM_207371	Hs.350848	NM_207371
AK128756	8.6	3.7	cDNA FLJ44869 fis, clone BRAMY2015516.	AK128756	Hs.632969	AK128756
BMP5	8.6	19.5	bone morphogenetic protein 5 (BMP5), mRNA	NM_021073	Hs.296648	NM_021073
RBM18	8.6	2.6	Probable RNA-binding protein 18 (RNA-binding motif protein 18).	ENST00000373748	Unknown	
U56433	8.6	3.3	Human HeLa mRNA isolated as a false positive in a two-hybrid-screen.	U56433	Hs.657756	NM_005921
MAP4K5	8.6	2.4	mitogen-activated protein kinase kinase kinase kinase 5 (MAP4K5), transcript variant 2, mRNA	NM_198794	Hs.130491	NM_198794
TSPYL4	8.6	2.9	TSPY-like 4 (TSPYL4), mRNA	NM_021648	Hs.284141	NM_021648
MGC24039	8.6	6.1	full length insert cDNA clone ZD50F09.	AF086301	Hs.118166	NM_144973
IGSF4	8.6	7.9	immunoglobulin superfamily, member 4 (IGSF4), mRNA	NM_014333	Hs.370510	BX641042
GOLGA7	8.6	1.8	golgi autoantigen, golgin subfamily a, 7 (GOLGA7), transcript variant 2, mRNA	NM_001002296	Hs.654773	NM_001002296
AGT	8.6	26.8	angiotensinogen (serpin peptidase inhibitor, clade A, member 8) (AGT), mRNA	NM_000029	Hs.19383	NM_000029
AGT	8.6	25.9	angiotensinogen (serpin peptidase inhibitor, clade A, member 8) (AGT), mRNA	NM_000029	Hs.19383	NM_000029
PDIK1L	8.6	4.8	PDLIM1 interacting kinase 1 like (PDIK1L), mRNA	NM_152835	Hs.468801	AF411102
SCML1	8.6	16.6	sex comb on midleg-like 1 (Drosophila) (SCML1), transcript variant 1, mRNA	NM_001037540	Hs.109655	NM_001037540
ULK2	8.6	2.9	unc-51-like kinase 2 (C. elegans) (ULK2), mRNA	NM_014683	Hs.168762	AB014523
GLT8D2	8.5	3.4	glycosyltransferase 8 domain containing 2 (GLT8D2), mRNA	NM_031302	Hs.631650	BC022343
KCNJ3	8.5	5.6	potassium inwardly-rectifying channel, subfamily J, member 3 (KCNJ3), mRNA	NM_002239	Hs.591606	NM_002239
LOC400027	8.5	2.2	cDNA clone IMAGE:5288894.	BC047417	Hs.597122	BC047417
AK125361	8.5	5.5	cDNA FLJ43371 fis, clone NTONG2005969.	AK125361	Hs.276808	NM_002409
SMAD3	8.5	2.7	SMAD family member 3 (SMAD3), mRNA	NM_005902	Hs.36915	NM_005902
LOC641518	8.5	5.9	hypothetical protein LOC641518, mRNA (cDNA clone IMAGE:4809416), partial cds.	BC020624	Hs.535760	BC094788
OSR1	8.5	7.5	odd-skipped related 1 (Drosophila) (OSR1), mRNA	NM_145260	Hs.123933	BC025712
THC2664742	8.5	35.0	THC2664742	THC2664742	Unknown	
AK025975	8.5	13.1	cDNA: FLJ22322 fis, clone HRC05532.	AK025975	Hs.380705	AK125423
KIAA1450	8.5	3.3	cDNA FLJ25252 fis, clone STM03814.	AK057981	Hs.652441	NM_020840
DPY19L4	8.5	2.4	dpy-19-like 4 (C. elegans) (DPY19L4), mRNA	NM_181787	Hs.567828	BX538174
SMAD3	8.5	2.7	SMAD family member 3 (SMAD3), mRNA	NM_005902	Hs.36915	NM_005902
DCTN3	8.5	3.1	dynactin 3 (p22) (DCTN3), transcript variant 1, mRNA	NM_007234	Hs.511768	BM920638
LOC401317	8.5	2.6	cDNA clone IMAGE:30398108.	ENST00000381802	Unknown	
FOXF1	8.5	7.9	forkhead box F1 (FOXF1), mRNA	NM_001451	Hs.155591	NM_001451
TMEM106C	8.5	4.6	transmembrane protein 106C (TMEM106C), mRNA	NM_024056	Hs.596726	AK056442
CAMK2N1	8.5	4.8	calcium/calmodulin-dependent protein kinase II inhibitor 1 (CAMK2N1), mRNA	NM_018584	Hs.197922	NM_018584
BCL2	8.5	8.4	B-cell CLL/lymphoma 2 (BCL2), nuclear gene encoding mitochondrial protein, transcript variant alpha, mRNA	NM_000633	Hs.150749	NM_000633
TNNC1	8.5	8.0	troponin C type 1 (slow) (TNNC1), mRNA	NM_003280	Hs.118845	CF553054
LOC346887	8.5	2.2	similar to solute carrier family 16 (monocarboxylic acid transporters), member 14, mRNA (cDNA clone IMAGE:5726657).	BC040619	Hs.127286	BC040619
AGT	8.5	26.0	angiotensinogen (serpin peptidase inhibitor, clade A, member 8) (AGT), mRNA	NM_000029	Hs.19383	NM_000029
FLJ31485	8.5	3.9	cDNA FLJ31485 fis, clone NT2NE2001698.	AK056047	Hs.288262	AK123838
SVOP	8.5	5.3	SV2 related protein homolog (rat) (SVOP), mRNA	NM_018711	Hs.4221	BC033587
ZNF189	8.5	2.2	zinc finger protein 189 (ZNF189), transcript variant 2, mRNA	NM_197977	Hs.50123	NM_197977
TM2D3	8.5	1.9	TM2 domain containing 3 (TM2D3), transcript variant 1, mRNA	NM_078474	Hs.288912	BX641093

GLT25D2	8.5	4.1	glycosyltransferase 25 domain containing 2 (GLT25D2), mRNA	NM_015101	Hs.387995	NM_015101
TPD52L1	8.5	4.3	tumor protein D52-like 1 (TPD52L1), transcript variant 2, mRNA	NM_001003395	Hs.591347	U44429
AK057720	8.5	2.5	cDNA FLJ33158 fis, clone UTERU2000418.	AK057720	Hs.62314	AK057720
CCDC3	8.5	12.1	coiled-coil domain containing 3 (CCDC3), mRNA	NM_031455	Hs.498720	AK095792
KCNK10	8.5	3.7	potassium channel, subfamily K, member 10 (KCNK10), transcript variant 1, mRNA	NM_021161	Hs.592299	AF279890
FER	8.4	3.2	Proto-oncogene tyrosine-protein kinase FER (EC 2.7.10.2) (p94-FER) (c- FER).	ENST00000379734	Unknown	
MAGEF1	8.4	2.1	melanoma antigen family F, 1 (MAGEF1), mRNA	NM_022149	Hs.306123	NM_022149
LOC400027	8.4	2.7	PREDICTED: hypothetical gene supported by BC047417, transcript variant 2 (LOC400027), mRNA	XM_931434	Unknown	
THC2585656	8.4	8.7	Q6NVT1_XENTR (Q6NVT1) RNA binding motif protein 25, partial (7%)	THC2585656	Unknown	
C9orf150	8.4	6.6	chromosome 9 open reading frame 150 (C9orf150), mRNA	NM_203403	Hs.445356	NM_203403
SOX9	8.4	6.9	SRY (sex determining region Y)-box 9 (campomelic dysplasia, autosomal sex-reversal) (SOX9), mRNA	NM_000346	Hs.700579	NM_000346
ALS2CR4	8.4	2.9	amyotrophic lateral sclerosis 2 (juvenile) chromosome region, candidate 4 (ALS2CR4), transcript variant 1, mRNA	NM_001044385	Hs.12319	NM_001044385
C1orf102	8.4	3.5	chromosome 1 open reading frame 102 (C1orf102), transcript variant 1, mRNA	NM_145047	Hs.202207	NM_145047
C9orf3	8.4	3.1	chromosome 9 open reading frame 3 (C9orf3), mRNA	NM_032823	Hs.434253	AF043897
ENST00000341591	8.4	3.2	PHD finger protein 10 (XAP135).	ENST00000341591	Unknown	
PTGER4	8.4	10.0	prostaglandin E receptor 4 (subtype EP4) (PTGER4), mRNA	NM_000958	Hs.199248	NM_000958
ZIC1	8.4	20.8	Zic family member 1 (odd-paired homolog, Drosophila) (ZIC1), mRNA	NM_003412	Hs.647962	NM_003412
PNPLA4	8.4	2.5	patatin-like phospholipase domain containing 4 (PNPLA4), mRNA	NM_004650	Hs.264	NM_004650
HPCAL4	8.4	7.8	hippocalcin like 4 (HPCAL4), mRNA	NM_016257	Hs.696062	AL136591
KIAA1706	8.4	3.4	KIAA1706 protein (KIAA1706), mRNA	NM_030636	Hs.487994	NM_030636
NCALD	8.4	4.8	neurocalcin delta (NCALD), transcript variant 7, mRNA	NM_001040630	Hs.492427	NM_001040630
GRRP1	8.4	3.5	glycine/arginine rich protein 1 (GRRP1), mRNA	NM_024869	Hs.694119	NM_024869
ENST00000371189	8.4	4.2	Nuclear factor 1 A-type (Nuclear factor 1/A) (NF1-A) (NFI-A) (NF-I/A) (CCAAT-box-binding transcription factor) (CTF) (TGGCA-binding protein).	ENST00000371189	Unknown	
MYOM1	8.4	14.0	myomesin 1 (skelemin) 185kDa (MYOM1), mRNA	NM_003803	Hs.464469	NM_003803
KIRREL3	8.4	3.9	Kin of IRRE-like protein 3 precursor (Kin of irregular chiasm-like protein 3) (Nephrin-like 2).	ENST00000278934	Unknown	
RSAD2	8.3	6.1	radical S-adenosyl methionine domain containing 2 (RSAD2), mRNA	NM_080657	Hs.17518	NM_080657
C2orf33	8.3	2.2	chromosome 2 open reading frame 33 (C2orf33), mRNA	NM_020194	Hs.471528	NM_020194
THC2701088	8.3	3.0	THC2701088	THC2701088	Unknown	
CCNG2	8.3	4.5	cyclin G2 (CCNG2), mRNA	NM_004354	Hs.13291	BC032518
MMP11	8.3	3.5	matrix metallopeptidase 11 (stromelysin 3) (MMP11), mRNA	NM_005940	Hs.143751	NM_005940
TSPAN3	8.3	2.8	tetraspanin 3 (TSPAN3), transcript variant 1, mRNA	NM_005724	Hs.5062	AK027793
NELL1	8.3	36.9	NEL-like 1 (chicken) (NELL1), mRNA	NM_006157	Hs.657172	AK127805
BCL2	8.3	7.5	B-cell CLL/lymphoma 2 (BCL2), nuclear gene encoding mitochondrial protein, transcript variant alpha, mRNA	NM_000633	Hs.150749	NM_000633
CR623787	8.3	2.5	full-length cDNA clone CS0DI079YL01 of Placenta Cot 25-normalized of (human).	CR623787	Hs.687264	CR623787
PAFAH1B1	8.3	2.1	platelet-activating factor acetylhydrolase, isoform Ib, alpha subunit 45kDa (PAFAH1B1), mRNA	NM_000430	Hs.77318	NM_000430
PIGC	8.3	6.7	H.sapiens gene from PAC 106H8.	AL035301	Unknown	
PLCE1	8.3	6.7	phospholipase C, epsilon 1 (PLCE1), mRNA	NM_016341	Hs.655033	NM_016341
EPHX2	8.3	2.5	epoxide hydrolase 2, cytoplasmic (EPHX2), mRNA	NM_001979	Hs.212088	NM_001979
BX648950	8.3	2.3	mRNA; cDNA DKFZp686E1648 (from clone DKFZp686E1648).	BX648950	Hs.563560	BX640652
LRP2	8.3	4.3	low density lipoprotein-related protein 2 (LRP2), mRNA	NM_004525	Hs.700749	NM_004525
NFIB	8.3	3.2	nuclear factor I/B (NFIB), mRNA	NM_005596	Hs.699215	NM_005596
RAGE	8.3	4.2	renal tumor antigen (RAGE), mRNA	NM_014226	Hs.104119	AK131542
BAHCC1	8.3	5.3	BAH domain and coiled-coil containing 1, mRNA (cDNA clone IMAGE:5019335), partial cds.	BC033222	Hs.514580	NM_001080519
PDGFC	8.3	8.1	platelet derived growth factor C (PDGFC), mRNA	NM_016205	Hs.570855	AF091434
C3orf54	8.3	3.2	chromosome 3 open reading frame 54 (C3orf54), mRNA	NM_203370	Hs.86674	NM_203370
DPYSL4	8.3	3.7	dihydropyrimidinase-like 4 (DPYSL4), mRNA	NM_006426	Hs.100058	NM_006426
WDR19	8.3	2.4	WD repeat domain 19 (WDR19), mRNA	NM_025132	Hs.438482	NM_025132
MXD1	8.3	2.7	MAX dimerization protein 1 (MXD1), mRNA	NM_002357	Hs.468908	BC098396
ADAMTS5	8.3	5.4	ADAM metallopeptidase with thrombospondin type 1 motif, 5 (aggrecanase-2) (ADAMTS5), mRNA	NM_007038	Hs.58324	NM_007038
LOC285733	8.3	8.1	cDNA FLJ34581 fis, clone KIDNE2008480.	AK091900	Hs.388715	XM_379432
ENST00000376682	8.2	12.4	Probable G-protein coupled receptor 133 precursor (G-protein coupled receptor PGR25).	ENST00000376682	Unknown	

MSX1	8.2	6.5	msh homeobox 1 (MSX1), mRNA	NM_002448	Hs.424414	NM_002448
PTPRN2	8.2	6.1	protein tyrosine phosphatase, receptor type, N polypeptide 2 (PTPRN2), transcript variant 1, mRNA	NM_002847	Hs.490789	AB002385
ARID5B	8.2	13.5	AT rich interactive domain 5B (MRF1-like) (ARID5B), mRNA	NM_032199	Hs.535297	NM_032199
NTRK1	8.2	7.7	neurotrophic tyrosine kinase, receptor, type 1 (NTRK1), transcript variant 2, mRNA	NM_002529	Hs.406293	CR936794
C6orf52	8.2	3.9	chromosome 6 open reading frame 52, mRNA (cDNA clone IMAGE:4096427), partial cds.	BC016820	Hs.61389	AW001000
CLUAP1	8.2	2.6	clusterin associated protein 1 (CLUAP1), transcript variant 2, mRNA	NM_024793	Hs.155995	AB014543
TAGLN3	8.2	4.4	transgelin 3 (TAGLN3), transcript variant 1, mRNA	NM_013259	Hs.169330	NM_013259
A_32_P121978	8.2	10.4	A_32_P121978	A_32_P121978	Unknown	
RDH10	8.2	2.6	retinol dehydrogenase 10 (all-trans) (RDH10), mRNA	NM_172037	Hs.244940	BC067131
LRRC36	8.2	5.2	leucine rich repeat containing 36 (LRRC36), mRNA	NM_018296	Hs.125139	NM_018296
MAP9	8.2	3.2	microtubule-associated protein 9 (MAP9), mRNA	NM_001039580	Hs.61271	NM_001039580
FOXC1	8.2	19.3	forkhead box C1 (FOXC1), mRNA	NM_001453	Hs.348883	NM_001453
WDR5B	8.2	2.3	WD repeat domain 5B (WDR5B), mRNA	NM_019069	Hs.567513	NM_019069
GABARAPL2	8.2	2.4	GABA(A) receptor-associated protein-like 2 (GABARAPL2), mRNA	NM_007285	Hs.461379	BC040312
MUC20	8.2	6.3	mucin 20, cell surface associated (MUC20), mRNA	NM_152673	Hs.599259	NM_001098516
TMEM26	8.2	9.0	transmembrane protein 26 (TMEM26), mRNA	NM_178505	Hs.623955	CR749606
GTF2IRD2	8.2	6.8	GTF2I repeat domain containing 2 (GTF2IRD2), mRNA	NM_173537	Hs.647017	NM_173537
DB153264	8.2	2.2	DB153264 THYMU3 cDNA clone THYMU3030831 5', mRNA sequence	DB153264	Hs.374251	DB153264
FBXL3	8.2	2.4	F-box and leucine-rich repeat protein 3 (FBXL3), mRNA	NM_012158	Hs.508284	AL833187
ARHGAP24	8.2	6.2	cDNA FLJ27066 fis, clone SPL01327.	AK130576	Hs.667822	AK130576
CDH15	8.2	2.3	cadherin 15, M-cadherin (myotubule) (CDH15), mRNA	NM_004933	Hs.148090	NM_004933
AZI2	8.2	2.5	5-azacytidine induced 2 (AZI2), mRNA	NM_022461	Hs.700605	BX648471
7-Sep	8.1	2.1	septin 7 (SEPT7), transcript variant 2, mRNA	NM_001011553	Hs.191346	AB209677
FBXL17	8.1	6.9	F-box and leucine-rich repeat protein 17, mRNA (cDNA clone IMAGE:4215262), partial cds.	BC018548	Hs.657225	AL133602
RNF103	8.1	3.6	ring finger protein 103 (RNF103), mRNA	NM_005667	Hs.469199	NM_005667
AK123310	8.1	2.3	cDNA FLJ41316 fis, clone BRAMY2043314.	AK123310	Hs.654746	AK123310
SYT4	8.1	13.5	synaptotagmin IV (SYT4), mRNA	NM_020783	Hs.8059	AB037763
CFL2	8.1	4.0	cofilin 2 (muscle) (CFL2), transcript variant 1, mRNA	NM_021914	Hs.180141	NM_021914
ARL2BP	8.1	3.2	ADP-ribosylation factor-like 2 binding protein (ARL2BP), mRNA	NM_012106	Hs.632873	NM_012106
LPA	8.1	8.5	lipoprotein, Lp(a) (LPA), mRNA	NM_005577	Hs.520120	X06290
ANTXR2	8.1	4.6	anthrax toxin receptor 2 (ANTXR2), mRNA	NM_058172	Hs.162963	NM_058172
SH3BGRL	8.1	2.6	SH3 domain binding glutamic acid-rich protein like (SH3BGRL), mRNA	NM_003022	Hs.108029	BC103762
SH3PXD2A	8.1	3.4	SH3 and PX domains 2A (SH3PXD2A), mRNA	NM_014631	Hs.594708	NM_014631
TLE4	8.1	1.9	transducin-like enhancer of split 4 (E(sp1) homolog, Drosophila) (TLE4), mRNA	NM_007005	Hs.444213	CR749553
PCDH9	8.1	12.0	protocadherin 9 (PCDH9), transcript variant 2, mRNA	NM_020403	Hs.654709	NM_203487
ITIH4	8.1	1.9	inter-alpha (globulin) inhibitor H4 (plasma Kallikrein-sensitive glycoprotein) (ITIH4), mRNA	NM_002218	Hs.556077	AB209388
CAPN3	8.1	2.6	calpain 3, (p94) (CAPN3), transcript variant 1, mRNA	NM_000070	Hs.143261	AB117941
THC2666687	8.1	4.1	ALU1_HUMAN (P39188) Alu subfamily J sequence contamination warning entry, partial (4%)	THC2666687	Unknown	
CR597270	8.1	5.3	full-length cDNA clone CS0DI016YF21 of Placenta Cot 25-normalized of (human).	CR597270	Hs.173705	NM_001001701
GOLGA1	8.1	2.8	golgi autoantigen, golgin subfamily a, 1 (GOLGA1), mRNA	NM_002077	Hs.133469	U51587
C21orf51	8.1	4.3	chromosome 21 open reading frame 51, mRNA (cDNA clone IMAGE:5259529).	BC045820	Hs.656195	BC064410
EDN3	8.1	99.9	endothelin 3 (EDN3), transcript variant 2, mRNA	NM_207032	Hs.1408	BC053866
TBX5	8.1	222.6	T-box 5 (TBX5), transcript variant 1, mRNA	NM_000192	Hs.381715	NM_000192
LNPEP	8.1	5.0	Leucyl-cystinyl aminopeptidase (EC 3.4.11.3) (Cystinyl aminopeptidase) (Oxytocinase) (OTase) (Insulin-regulated membrane aminopeptidase) (Insulin-responsive aminopeptidase) (IRAP) (Placental leucine aminopeptidase) (P-LAP)....	ENST00000379870	Unknown	
BF217859	8.1	15.6	601885412F1 NIH_MGC_57 cDNA clone IMAGE:4103859 5', mRNA sequence	BF217859	Hs.522180	BF217859
BX095281	8.1	2.8	BX095281 Soares melanocyte 2NbHM cDNA clone IMAGp998E03596, mRNA sequence	BX095281	Hs.93780	BX095281
HOXB2	8.1	5.2	homeobox B2 (HOXB2), mRNA	NM_002145	Hs.514289	NM_002145
BX114764	8.1	3.9	BX114764 NCI_CGAP_GCB1 cDNA clone IMAGp998M182012, mRNA sequence	BX114764	Hs.634305	BX114764
REEP1	8.0	6.0	receptor accessory protein 1 (REEP1), mRNA	NM_022912	Hs.368884	AK023172
BRP44L	8.0	2.5	brain protein 44-like (BRP44L), mRNA	NM_016098	Hs.172755	BF698899

BTG3	8.0	3.2	Homo sapiens, clone IMAGE:5240818, mRNA.	BC028229	Unknown	
TMEM121	8.0	7.2	transmembrane protein 121 (TMEM121), mRNA	NM_025268	Hs.157527	NM_025268
IFT81	8.0	3.7	intraflagellar transport 81 homolog (Chlamydomonas) (IFT81), transcript variant 1, mRNA	NM_014055	Hs.528382	AF332010
C20orf132	8.0	4.1	chromosome 20 open reading frame 132 (C20orf132), transcript variant 1, mRNA	NM_152503	Hs.349125	BC030006
FOXD2	8.0	27.4	forkhead box D2 (FOXD2), mRNA	NM_004474	Hs.166188	NM_004474
DDR2	8.0	10.3	mRNA; cDNA DKFZp686C0390 (from clone DKFZp686C0390).	BX537651	Hs.591469	BX537651
THC2687042	8.0	3.5	Q8YX09_ANASP (Q8YX09) Alr1410 protein, partial (10%)	THC2687042	Unknown	
ZNF180	8.0	1.6	zinc finger protein 180 (ZNF180), mRNA	NM_013256	Hs.130683	BC051903
STARD3NL	8.0	1.9	STARD3 N-terminal like (STARD3NL), mRNA	NM_032016	Hs.309753	AK091579
BBS1	8.0	1.9	Bardet-Biedl syndrome 1 (BBS1), mRNA	NM_024649	Hs.502915	AK095638
AF086547	8.0	11.3	full length insert cDNA clone ZE12B03.	AF086547	Hs.418279	CK299542
NUDT6	8.0	4.3	nudix (nucleoside diphosphate linked moiety X)-type motif 6 (NUDT6), transcript variant 2, mRNA	NM_198041	Hs.558459	AB209758
IQCK	8.0	2.8	IQ motif containing K (IQCK), mRNA	NM_153208	Hs.460217	AF520570
ZBTB47	8.0	2.7	zinc finger and BTB domain containing 47 (ZBTB47), mRNA	NM_145166	Hs.409561	NM_145166
POPDC2	8.0	32.0	popeye domain containing 2 (POPDC2), mRNA	NM_022135	Hs.656031	AK124602
SULT1A2	8.0	3.5	sulfotransferase family, cytosolic, 1A, phenol-preferring, member 2 (SULT1A2), transcript variant 2, mRNA	NM_177528	Hs.546304	BC052280
RG9MTD2	8.0	2.3	RNA (guanine-9-) methyltransferase domain containing 2 (RG9MTD2), mRNA	NM_152292	Hs.380412	BC028373
SPATA9	8.0	9.8	spermatogenesis associated 9 (SPATA9), transcript variant 2, mRNA	NM_173360	Unknown	
LOC147650	8.0	2.3	cDNA FLJ14300 fis, clone PLACE1011891.	AK024362	Hs.467174	BC041134
THC2611641	8.0	2.8	Q6DIG3_XENTR (Q6DIG3) Hedgehog interacting protein, partial (3%)	THC2611641	Unknown	
THC2665933	8.0	2.2	Q5NNJ5_ZYMMO (Q5NNJ5) DNA polymerase III delta prime subunit , partial (5%)	THC2665933	Unknown	
TMEM107	8.0	3.2	transmembrane protein 107 (TMEM107), transcript variant 1, mRNA	NM_032354	Hs.513933	AK127891
CR601835	8.0	4.7	full-length cDNA clone CS0DF019YG19 of Fetal brain of (human).	CR601835	Hs.410889	XM_371074
CTDSP1	8.0	3.0	CTD (carboxy-terminal domain, RNA polymerase II, polypeptide A) small phosphatase 1 (CTDSP1), transcript variant 1, mRNA	NM_021198	Hs.444468	AF229162
SVIL	8.0	5.6	supervillin (SVIL), transcript variant 2, mRNA	NM_021738	Hs.499209	NM_021738
EFNB3	8.0	2.6	ephrin-B3 (EFNB3), mRNA	NM_001406	Hs.26988	NM_001406
SMAD3	8.0	2.7	SMAD family member 3 (SMAD3), mRNA	NM_005902	Hs.36915	NM_005902
THC2655485	7.9	5.3	AF512556 integrin, alpha 2 (CD49B, alpha 2 subunit of VLA-2 receptor) {Homo sapiens} (exp=-1; wgp=0; cg=0), partial (3%)	THC2655485	Unknown	
PRTG	7.9	5.5	protogenin homolog (Gallus gallus) (PRTG), mRNA	NM_173814	Hs.130957	NM_173814
SMAD3	7.9	2.7	SMAD family member 3 (SMAD3), mRNA	NM_005902	Hs.36915	NM_005902
NPAS3	7.9	2.9	neuronal PAS domain protein 3 (NPAS3), transcript variant 1, mRNA	NM_022123	Hs.659456	AF164438
UGCGL2	7.9	2.8	UDP-glucose ceramide glucosyltransferase-like 2 (UGCGL2), mRNA	NM_020121	Hs.193226	NM_020121
THC2667632	7.9	11.2	THC2667632	THC2667632	Unknown	
TPM1	7.9	2.8	tropomyosin 1 (alpha) (TPM1), transcript variant 5, mRNA	NM_000366	Hs.133892	BX648171
CDK6	7.9	6.7	cyclin-dependent kinase 6 (CDK6), mRNA	NM_001259	Hs.119882	NM_001259
CD47	7.9	4.6	CD47 molecule (CD47), transcript variant 1, mRNA	NM_001777	Hs.446414	NM_001777
RP11-301I17.1	7.9	32.7	proliferation-inducing protein 38 (PIG38), mRNA	NM_017993	Hs.128258	BC068521
CACNA1C	7.9	12.1	calcium channel, voltage-dependent, L type, alpha 1C subunit (CACNA1C), mRNA	NM_000719	Hs.118262	NM_000719
AGT	7.9	26.3	angiotensinogen (serpin peptidase inhibitor, clade A, member 8) (AGT), mRNA	NM_000029	Hs.19383	NM_000029
BU160948	7.9	19.8	AGENCOURT_7984218 NIH_MGC_72 cDNA clone IMAGE:6166584 5', mRNA sequence	BU160948	Hs.657271	NM_007078
SMAD3	7.9	2.7	SMAD family member 3 (SMAD3), mRNA	NM_005902	Hs.36915	NM_005902
HIST1H1C	7.9	3.2	histone cluster 1, H1c (HIST1H1C), mRNA	NM_005319	Hs.7644	BQ940876
C8orf53	7.9	5.8	cDNA FLJ30717 fis, clone FCBBF2001672.	AK055279	Hs.86970	AK055279
DPY19L2	7.9	3.8	dpy-19-like 2 (C. elegans) (DPY19L2), mRNA	NM_173812	Hs.533644	NM_173812
ALDH1A2	7.9	61.3	aldehyde dehydrogenase 1 family, member A2 (ALDH1A2), transcript variant 3, mRNA	NM_170697	Hs.699620	AK128709
AK091904	7.9	11.6	cDNA FLJ34585 fis, clone KIDNE2008758.	AK091904	Hs.202577	AK091904
RTN1	7.9	3.5	reticulon 1 (RTN1), transcript variant 1, mRNA	NM_021136	Hs.368626	NM_021136
MXI1	7.9	3.3	MAX interactor 1 (MXI1), transcript variant 2, mRNA	NM_130439	Hs.501023	NM_130439
SEMA6D	7.9	4.7	sema domain, transmembrane domain (TM), and cytoplasmic domain, (semaphorin) 6D (SEMA6D), transcript variant 4, mRNA	NM_153618	Hs.511265	AF389429
THC2544148	7.9	2.0	Q6TXI7_RAT (Q6TXI7) LRRGT00012, partial (5%)	THC2544148	Unknown	
THSD1	7.9	2.3	thrombospondin, type I, domain containing 1 (THSD1), transcript variant 1, mRNA	NM_018676	Hs.325667	AK096289

ZNF521	7.9	7.8	zinc finger protein 521 (ZNF521), mRNA	NM_015461	Hs.116935	AK027354
M69296	7.9	17.4	Human estrogen receptor-related protein (variant ER from breast cancer) mRNA, complete cds.	M69296	Unknown	
AK023401	7.9	5.3	cDNA FLJ13339 fis, clone OVARC1001928.	AK023401	Hs.199853	AK127591
MEST	7.9	2.3	mesoderm specific transcript homolog (mouse) (MEST), transcript variant 1, mRNA	NM_002402	Hs.270978	AK055108
SFRS2B	7.9	3.2	splicing factor, arginine/serine-rich 2B (SFRS2B), mRNA	NM_032102	Hs.648465	NM_032102
FOXP2	7.9	11.1	forkhead box P2 (FOXP2), transcript variant 1, mRNA	NM_014491	Hs.656280	CR749236
DLX1	7.9	7.8	distal-less homeobox 1 (DLX1), transcript variant 1, mRNA	NM_178120	Hs.407015	NM_178120
PRKD1	7.9	6.3	protein kinase D1 (PRKD1), mRNA	NM_002742	Hs.508999	X75756
SOST	7.9	8.9	sclerosteosis (SOST), mRNA	NM_025237	Hs.349204	AY358627
ADCYAP1	7.9	9.1	adenylate cyclase activating polypeptide 1 (pituitary) (ADCYAP1), mRNA	NM_001117	Hs.531719	AK095723
THC2556456	7.9	3.4	Q5YKG5_HUMAN (Q5YKG5) C114 SLIT-like testicular protein, partial (5%)	THC2556456	Unknown	
ZNF228	7.8	2.7	zinc finger protein 228 (ZNF228), mRNA	NM_013380	Hs.48589	AL833447
LOC283658	7.8	20.1	cDNA FLJ30808 fis, clone FEBRA2001383.	AK055370	Hs.87194	AL833463
C10orf84	7.8	2.1	Novel protein.	ENST00000369183	Unknown	
FRMD4A	7.8	4.3	FERM domain containing 4A (FRMD4A), mRNA	NM_018027	Hs.330463	BC151244
LOC283481	7.8	1.8	hypothetical protein LOC283481, mRNA (cDNA clone IMAGE:5296747).	BC033993	Hs.646604	BI560826
C16orf48	7.8	2.4	chromosome 16 open reading frame 48 (C16orf48), mRNA	NM_032140	Hs.307084	AL136786
EMILIN1	7.8	5.6	elastin microfibril interfacer 1 (EMILIN1), mRNA	NM_007046	Hs.63348	BC090957
AK124941	7.8	2.6	cDNA FLJ42951 fis, clone BRSTN2007765.	AK124941	Hs.656242	AK124941
ARL3	7.8	2.2	ADP-ribosylation factor-like 3 (ARL3), mRNA	NM_004311	Hs.182215	NM_004311
HECTD2	7.8	2.5	HECT domain containing 2 (HECTD2), transcript variant 1, mRNA	NM_182765	Hs.656960	BC040187
CCDC82	7.8	2.7	coiled-coil domain containing 82 (CCDC82), mRNA	NM_024725	Hs.525088	AK074306
AGPAT3	7.8	4.6	1-acylglycerol-3-phosphate O-acyltransferase 3 (AGPAT3), transcript variant 1, mRNA	NM_020132	Hs.248785	NM_020132
DHRS7C	7.8	5.2	PREDICTED: dehydrogenase	ENST00000330255	Unknown	
CLIPR-59	7.8	3.9	CLIP-170-related protein (CLIPR-59), mRNA	NM_015526	Hs.466539	AK094738
C5	7.8	6.2	complement component 5 (C5), mRNA	NM_001735	Hs.494997	AB209031
VASH1	7.8	3.5	vasohibin 1 (VASH1), mRNA	NM_014909	Hs.525479	AL832588
IRX3	7.8	16.1	iroquois homeobox protein 3 (IRX3), mRNA	NM_024336	Hs.499205	AY335943
SSBP2	7.8	4.6	Single-stranded DNA-binding protein 2 (Sequence-specific single- stranded-DNA-binding protein 2).	ENST00000380186	Unknown	
BICC1	7.8	8.5	CDNA: FLJ22476 fis, clone HRC10682.	ENST00000373886	Unknown	
ARMCX6	7.8	2.4	armadillo repeat containing, X-linked 6 (ARMCX6), transcript variant 1, mRNA	NM_019007	Hs.83530	AK097018
TNAP	7.8	4.0	NIK-associated protein mRNA, complete cds.	AF463496	Hs.687024	AF463496
AFTPH	7.8	1.7	aftiphilin (AFTPH), transcript variant 1, mRNA	NM_203437	Hs.655167	AL833962
TMEM71	7.8	8.2	transmembrane protein 71 (TMEM71), mRNA	NM_144649	Hs.293842	BC062592
A_32_P144381	7.8	3.8	A_32_P144381	A_32_P144381	Unknown	
A_32_P101420	7.8	4.3	A_32_P101420	A_32_P101420	Unknown	
NPY2R	7.7	7.8	neuropeptide Y receptor Y2 (NPY2R), mRNA	NM_000910	Hs.37125	NM_000910
TRAF3	7.7	2.5	TNF-receptor associated factor-3 (TRAF-3) mRNA, partial cds; and 3'UTR.	AF110908	Hs.510528	AF110908
GOLGA1	7.7	2.8	golgi autoantigen, golgin subfamily a, 1 (GOLGA1), mRNA	NM_002077	Hs.133469	U51587
TMEM128	7.7	1.8	transmembrane protein 128 (TMEM128), mRNA	NM_032927	Hs.12845	CR933611
FLJ32447	7.7	8.7	hypothetical protein LOC151278 (FLJ32447), mRNA	NM_153038	Hs.350729	AK057009
BQ897248	7.7	4.9	AGENCOURT_8122036 Lupski_dorsal_root_ganglion cDNA clone IMAGE:6179261 5', mRNA sequence	BQ897248	Hs.69297	BQ897248
TTC30B	7.7	2.9	tetratricopeptide repeat domain 30B (TTC30B), mRNA	NM_152517	Hs.447659	NM_152517
PALLD	7.7	3.7	palladin, cytoskeletal associated protein (PALLD), mRNA	NM_016081	Hs.151220	NM_016081
FRMD4B	7.7	4.9	mRNA for KIAA1013 protein, partial cds.	AB023230	Hs.371681	NM_015123
KIAA1370	7.7	2.8	KIAA1370 (KIAA1370), mRNA	NM_019600	Hs.152385	NM_019600
CXorf6	7.7	4.9	chromosome X open reading frame 6 (CXorf6), mRNA	NM_005491	Hs.20136	BX537560
THC2682152	7.7	3.0	Q9F8M7_CARHY (Q9F8M7) DTDP-glucose 4,6-dehydratase (Fragment), partial (11%)	THC2682152	Unknown	
PHF2	7.7	2.4	PHD finger protein 2 (PHF2), mRNA	NM_005392	Hs.211441	NM_005392
THC2660651	7.7	2.2	Q9KBG2_BACHD (Q9KBG2) Metal-tetracycline/H+ antiporter, partial (4%)	THC2660651	Unknown	
THC2664215	7.7	3.2	THC2664215	THC2664215	Unknown	
PLGLB1	7.7	4.5	plasminogen-like B1 (PLGLB1), mRNA	NM_001032392	Hs.652174	NM_001032392

QKI	7.7	3.3	quaking homolog, KH domain RNA binding (mouse) (QKI), transcript variant 4, mRNA	NM_206855	Hs.510324	NM_206855
FLJ21127	7.7	2.8	tectonic (FLJ21127), mRNA	NM_024549	Hs.211511	BC033811
XPA	7.7	3.6	xeroderma pigmentosum, complementation group A (XPA), mRNA	NM_000380	Hs.654364	AK021661
AK057652	7.7	7.4	cDNA FLJ33090 fis, clone TRACH2000559.	AK057652	Hs.118947	AK057652
VPS28	7.7	2.3	vacuolar protein sorting 28 homolog (S. cerevisiae) (VPS28), transcript variant 2, mRNA	NM_183057	Hs.418175	AK091979
DECR1	7.7	2.7	2,4-dienoyl CoA reductase 1, mitochondrial (DECR1), nuclear gene encoding mitochondrial protein, mRNA	NM_001359	Hs.492212	BM920635
AK128814	7.7	7.0	cDNA FLJ46049 fis, clone SYNOV2020463.	AK128814	Hs.432914	AK128814
6-Sep	7.7	4.4	septin 6 (SEPT6), transcript variant V, mRNA	NM_145802	Hs.496666	NM_145799
C7orf41	7.7	2.1	chromosome 7 open reading frame 41 (C7orf41), mRNA	NM_152793	Hs.200100	NM_152793
GJB1	7.6	4.3	gap junction protein, beta 1, 32kDa (connexin 32, Charcot-Marie-Tooth neuropathy, X-linked) (GJB1), mRNA	NM_000166	Hs.333303	BF690836
BDH2	7.6	2.4	3-hydroxybutyrate dehydrogenase, type 2 (BDH2), mRNA	NM_020139	Hs.124696	NM_020139
STARD9	7.6	2.2	StAR-related lipid transfer protein 9 (StARD9) (START domain- containing protein 9) (Fragment).	ENST00000290607	Unknown	
SMAD9	7.6	4.0	SMAD family member 9 (SMAD9), mRNA	NM_005905	Hs.123119	BC067766
UNC45B	7.6	11.5	unc-45 homolog B (C. elegans) (UNC45B), transcript variant 1, mRNA	NM_173167	Hs.379636	NM_173167
RABL2A	7.6	2.3	RAB, member of RAS oncogene family-like 2A (RABL2A), transcript variant 1, mRNA	NM_013412	Hs.446425	AK092971
ENST00000354771	7.6	4.0	mRNA; cDNA DKFZp434L187 (from clone DKFZp434L187); partial cds.	ENST00000354771	Unknown	
PDGFRA	7.6	6.6	AA599881 ag32e07.s1 Human bone marrow stromal cells cDNA clone IMAGE:1091268 3' similar to gb:M21574 ALPHA PLATELET-DERIVED GROWTH FACTOR RECEPTOR PRECURSOR (HUMAN);, mRNA sequence	AA599881	Hs.74615	NM_006206
TSPAN18	7.6	2.4	tetraspanin 18 (TSPAN18), transcript variant 2, mRNA	NM_130783	Hs.385634	AY358087
FBXL7	7.6	2.5	F-box and leucine-rich repeat protein 7 (FBXL7), mRNA	NM_012304	Hs.433057	AB020647
DOCK1	7.6	2.0	dedicator of cytokinesis 1 (DOCK1), mRNA	NM_001380	Hs.645702	NM_001380
THC2508270	7.6	4.2	THC2508270	THC2508270	Unknown	
CACNA1C	7.6	3.4	calcium channel, voltage-dependent, L type, alpha 1C subunit (CACNA1C), mRNA	NM_000719	Hs.118262	NM_000719
THC2740317	7.6	2.8	THC2740317	THC2740317	Unknown	
APPBP2	7.6	3.6	amyloid beta precursor protein (cytoplasmic tail) binding protein 2 (APPBP2), mRNA	NM_006380	Hs.84084	NM_006380
FLJ36032	7.6	4.1	cDNA FLJ36032 fis, clone TESTI2017069.	AK093351	Hs.297967	AK093351
FRG1	7.6	1.8	FSHD region gene 1 (FRG1), mRNA	NM_004477	Hs.203772	AK057099
BU685299	7.6	4.1	UI-CF-DU1-aav-k-21-0-UI.s2 UI-CF-DU1 cDNA clone UI-CF-DU1-aav-k-21-0-UI 3', mRNA sequence	BU685299	Hs.622344	BU685299
YPEL5	7.6	3.1	yippee-like 5 (Drosophila) (YPEL5), mRNA	NM_016061	Hs.515890	AK025730
ZSWIM6	7.6	2.2	Zinc finger SWIM domain-containing protein 6 (Fragment).	ENST00000252744	Unknown	
GPC5	7.6	23.7	glypican 5 (GPC5), mRNA	NM_004466	Hs.655675	NM_004466
ARRDC4	7.6	5.3	arrestin domain containing 4 (ARRDC4), mRNA	NM_183376	Hs.6093	NM_183376
PAIP1	7.5	1.9	poly(A) binding protein interacting protein 1 (PAIP1), transcript variant 1, mRNA	NM_006451	Hs.482038	NM_006451
C3orf45	7.5	2.5	chromosome 3 open reading frame 45 (C3orf45), mRNA	NM_153215	Hs.534543	BC028000
ZC3H8	7.5	1.9	zinc finger CCCH-type containing 8 (ZC3H8), mRNA	NM_032494	Hs.418416	BC032001
ZNF654	7.5	3.1	zinc finger protein 654 (ZNF654), mRNA	NM_018293	Hs.591650	NM_018293
THC2541331	7.5	1.7	HSU02032 ribosomal protein L23a {Homo sapiens} (exp=-1; wgp=0; cg=0), partial (71%)	THC2541331	Unknown	
C10orf32	7.5	5.5	chromosome 10 open reading frame 32 (C10orf32), mRNA	NM_144591	Hs.34492	NM_020682
OSR2	7.5	19.0	odd-skipped related 2 (Drosophila) (OSR2), mRNA	NM_053001	Hs.253247	AK074518
GSTM3	7.5	9.6	glutathione S-transferase M3 (brain) (GSTM3), mRNA	NM_000849	Hs.2006	NM_000849
BACE1	7.5	7.4	beta-site APP-cleaving enzyme 1 (BACE1), transcript variant a, mRNA	NM_012104	Hs.504003	AF201468
NBPF11	7.5	5.4	neuroblastoma breakpoint family, member 11 (NBPF11), mRNA	NM_183372	Hs.636561	BX648497
UNC45B	7.5	14.1	unc-45 homolog B (C. elegans) (UNC45B), transcript variant 1, mRNA	NM_173167	Hs.379636	NM_173167
JMJD1A	7.5	3.4	jumonji domain containing 1A (JMJD1A), mRNA	NM_018433	Hs.557425	BX640698
THC2631465	7.5	3.8	THC2631465	THC2631465	Unknown	
5-Mar	7.5	2.4	membrane-associated ring finger (C3HC4) 5 (MARCH5), mRNA	NM_017824	Hs.573490	NM_017824
CFI	7.5	173.0	complement factor I (CFI), mRNA	NM_000204	Hs.312485	AK122686
FZD3	7.5	2.7	Frizzled-3 precursor (Fz-3) (hFz3).	ENST00000380239	Unknown	
AK024680	7.5	7.1	cDNA: FLJ21027 fis, clone CAE07110.	AK024680	Hs.660596	AK024680
THC2740111	7.5	2.7	THC2740111	THC2740111	Unknown	
ENY2	7.5	3.1	cDNA FLJ38332 fis, clone FCBBF3025528.	AK095651	Hs.492555	AK095651

PAG1	7.5	9.2	phosphoprotein associated with glycosphingolipid microdomains 1 (PAG1), mRNA	NM_018440	Hs.266175	NM_018440
PLEKHG1	7.5	8.4	pleckstrin homology domain containing, family G (with RhoGef domain) member 1 (PLEKHG1), mRNA	NM_001029884	Hs.189781	NM_001029884
ARNT2	7.5	2.8	aryl-hydrocarbon receptor nuclear translocator 2 (ARNT2), mRNA	NM_014862	Hs.459070	NM_014862
BQ071984	7.5	2.7	AGENCOURT_6859850 NIH_MGC_47 cDNA clone IMAGE:5928467 5', mRNA sequence	BQ071984	Hs.638240	BQ948285
TRH	7.5	23.7	thyrotropin-releasing hormone (TRH), mRNA	NM_007117	Hs.182231	NM_007117
LPPR4	7.5	2.8	plasticity related gene 1 (LPPR4), mRNA	NM_014839	Hs.13245	AF541281
DPF3	7.5	36.0	cDNA FLJ42956 fis, clone BRSTN2009899.	AK124946	Unknown	
ENST00000324709	7.5	6.5	Plasminogen-related protein A (Fragment).	ENST00000324709	Unknown	
ATRNL1	7.5	3.7	attractin-like 1 (ATRNL1), mRNA	NM_207303	Hs.501127	NM_207303
CR610468	7.4	5.0	full-length cDNA clone CS0DF029YL18 of Fetal brain of (human).	CR610468	Hs.563205	NM_024582
THC2537219	7.4	4.8	Q6ATL9_ORYSA (Q6ATL9) Expressed protein, partial (13%)	THC2537219	Unknown	
EVL	7.4	2.2	Enah/Vasp-like (EVL), mRNA	NM_016337	Hs.125867	AL133642
CCDC104	7.4	2.7	coiled-coil domain containing 104 (CCDC104), mRNA	NM_080667	Hs.264208	AY358097
LMO2	7.4	10.7	LIM domain only 2 (rhombotin-like 1) (LMO2), mRNA	NM_005574	Hs.34560	NM_005574
SEMA3B	7.4	2.1	sema domain, immunoglobulin domain (Ig), short basic domain, secreted, (semaphorin) 3B (SEMA3B), transcript variant 1, mRNA	NM_004636	Hs.82222	AB209322
SPAG11	7.4	6.7	sperm associated antigen 11 (SPAG11), transcript variant A, mRNA	NM_016512	Hs.2717	BM563643
ANXA8	7.4	4.7	annexin A8 (ANXA8), mRNA	NM_001630	Hs.700586	NM_001630
ZNF452	7.4	2.5	zinc finger protein 452 (ZNF452), mRNA	NM_052923	Hs.176980	AY517631
USP47	7.4	2.7	ubiquitin specific peptidase 47 (USP47), mRNA	NM_017944	Hs.577256	NM_017944
KIAA1715	7.4	4.9	KIAA1715 (KIAA1715), mRNA	NM_030650	Hs.209561	CR936742
C20orf75	7.4	11.2	cDNA FLJ23994 fis, clone HRC11286.	AK172833	Hs.149133	BC027720
FANK1	7.4	3.9	fibronectin type III and ankyrin repeat domains 1 (FANK1), mRNA	NM_145235	Hs.352591	CR627249
AHI1	7.4	2.0	Abelson helper integration site 1 (AHI1), mRNA	NM_017651	Hs.386684	NM_017651
ENST00000320547	7.4	2.1	CDNA: FLJ22509 fis, clone HRC11803.	ENST00000320547	Unknown	
TXNDC4	7.4	2.2	thioredoxin domain containing 4 (endoplasmic reticulum) (TXNDC4), mRNA	NM_015051	Hs.591899	NM_015051
ELMOD2	7.4	1.6	ELMO domain-containing protein 2.	ENST00000323570	Unknown	
THC2773657	7.4	5.0	THC2773657	THC2773657	Unknown	
SRR	7.4	3.3	serine racemase (SRR), mRNA	NM_021947	Hs.461954	AK023169
ARMC3	7.4	7.4	armadillo repeat containing 3 (ARMC3), mRNA	NM_173081	Hs.659807	BC039312
C5orf13	7.4	3.2	chromosome 5 open reading frame 13 (C5orf13), mRNA	NM_004772	Hs.36053	CR607755
GFRA1	7.4	3.3	GDNF family receptor alpha 1 (GFRA1), transcript variant 2, mRNA	NM_145793	Hs.591913	NM_005264
MGC3771	7.4	6.5	cDNA clone IMAGE:2959625.	BC001809	Hs.669490	BC001824
C14orf43	7.4	3.1	chromosome 14 open reading frame 43 (C14orf43), transcript variant 1, mRNA	NM_194278	Hs.656506	NM_194278
MBNL1	7.4	2.8	muscleblind-like (Drosophila) (MBNL1), transcript variant 1, mRNA	NM_021038	Hs.478000	NM_021038
SMAD3	7.4	2.7	SMAD family member 3 (SMAD3), mRNA	NM_005902	Hs.36915	NM_005902
ZNF148	7.4	1.9	zinc finger protein 148 (ZNF148), mRNA	NM_021964	Hs.592591	NM_021964
PITPNC1	7.4	2.3	cDNA FLJ37405 fis, clone BRAMY2028269.	AK094724	Hs.696160	AK094724
GALNTL4	7.4	3.3	UDP-N-acetyl-alpha-D-galactosamine:polypeptide N-acetylgalactosaminyltransferase-like 4 (GALNTL4), mRNA	NM_198516	Hs.655152	AK124690
HNRPDL	7.4	1.7	heterogeneous nuclear ribonucleoprotein D-like (HNRPDL), transcript variant 2, mRNA	NM_031372	Hs.527105	NM_031372
LMO2	7.4	11.1	LIM domain only 2 (rhombotin-like 1) (LMO2), mRNA	NM_005574	Hs.34560	NM_005574
ENST00000332074	7.4	5.3	LMOD2 protein (Fragment).	ENST00000332074	Unknown	
TMEM45A	7.4	3.9	transmembrane protein 45A (TMEM45A), mRNA	NM_018004	Hs.658956	BX648832
THC2676139	7.4	3.3	THC2676139	THC2676139	Unknown	
LOC440248	7.3	2.3	hypothetical LOC440248 (LOC440248), mRNA	NM_199045	Unknown	
AASDHPPT	7.3	2.2	aminoadipate-semialdehyde dehydrogenase-phosphopantetheinyl transferase (AASDHPPT), mRNA	NM_015423	Hs.524009	BX537665
PHC2	7.3	4.3	polyhomeotic homolog 2 (Drosophila) (PHC2), transcript variant 1, mRNA	NM_198040	Hs.524271	NM_198040
THC2643233	7.3	3.0	Q3VNU0_9CHLB (Q3VNU0) DNA/RNA non-specific endonuclease precursor, partial (5%)	THC2643233	Unknown	
GATS	7.3	3.6	opposite strand transcription unit to STAG3, mRNA (cDNA clone MGC:102871 IMAGE:6179098), complete cds.	BC090867	Hs.556063	AL831967
LMO3	7.3	8.0	LIM domain only 3 (rhombotin-like 2) (LMO3), transcript variant 1, mRNA	NM_018640	Hs.504908	NM_018640
AK129652	7.3	2.7	cDNA FLJ26141 fis, clone TST03911.	AK129652	Hs.593620	AK129652
A_32_P80697	7.3	4.6	A_32_P80697	A_32_P80697	Unknown	

MTR	7.3	3.0	Methionine synthase (EC 2.1.1.13) (5-methyltetrahydrofolate-- homocysteine methyltransferase) (Methionine synthase, vitamin-B12 dependent) (MS).	ENST00000366577	Unknown	
RNASE4	7.3	3.2	ribonuclease, RNase A family, 4 (RNASE4), transcript variant 1, mRNA	NM_194430	Unknown	
NAP1L5	7.3	4.6	nucleosome assembly protein 1-like 5 (NAP1L5), mRNA	NM_153757	Hs.12554	BC022544
C1orf198	7.3	2.6	chromosome 1 open reading frame 198 (C1orf198), mRNA	NM_032800	Hs.568242	BC066649
TRPS1	7.3	5.5	trichorhinophalangeal syndrome I (TRPS1), mRNA	NM_014112	Hs.657018	AF183810
ISX	7.3	8.7	intestine-specific homeobox (ISX), mRNA	NM_001008494	Hs.567637	CR749372
C11orf1	7.3	2.2	chromosome 11 open reading frame 1 (C11orf1), mRNA	NM_022761	Hs.17546	BC036892
PJA2	7.3	2.3	praja 2, RING-H2 motif containing (PJA2), mRNA	NM_014819	Hs.483036	BC030826
LOC286334	7.3	2.0	mRNA full length insert cDNA clone EUROIMAGE 1517766.	AJ420454	Hs.349208	NM_133374
BF207040	7.3	5.2	601870125F1 NIH_MGC_19 cDNA clone IMAGE:4100239 5', mRNA sequence	BF207040	Hs.353024	BF207040
PNMA1	7.3	1.8	paraneoplastic antigen MA1 (PNMA1), mRNA	NM_006029	Hs.194709	NM_006029
PRICKLE2	7.3	4.2	prickle homolog 2 (Drosophila) (PRICKLE2), mRNA	NM_198859	Hs.699317	AL833539
FOXP4	7.3	4.2	forkhead box P4 (FOXP4), transcript variant 1, mRNA	NM_001012426	Hs.131436	NM_001012426
EFHC1	7.3	2.3	EF-hand domain (C-terminal) containing 1 (EFHC1), mRNA	NM_018100	Hs.403171	AY608689
LOC286161	7.3	4.3	cDNA FLJ34353 fis, clone FEBRA2011665.	AK091672	Unknown	
SMAD3	7.3	2.6	SMAD family member 3 (SMAD3), mRNA	NM_005902	Hs.36915	NM_005902
LMO2	7.3	10.8	LIM domain only 2 (rhombotin-like 1) (LMO2), mRNA	NM_005574	Hs.34560	NM_005574
LOC285831	7.3	3.9	cDNA FLJ37752 fis, clone BRHIP2023309.	AK095071	Hs.654655	AK095071
SPAG6	7.3	10.9	sperm associated antigen 6 (SPAG6), transcript variant 2, mRNA	NM_172242	Hs.655170	NM_012443
LOC400960	7.3	3.0	cDNA FLJ32260 fis, clone PROST1000334.	AK056822	Hs.657791	AK056822
ABCA1	7.3	4.4	ATP-binding cassette, sub-family A (ABC1), member 1 (ABCA1), mRNA	NM_005502	Hs.429294	AF285167
A_24_P896373	7.3	2.9	A_24_P896373	A_24_P896373	Unknown	
NUCB2	7.3	4.5	cDNA FLJ40079 fis, clone TESTI2001498, highly similar to DNA-BINDING PROTEIN NEFA PRECURSOR.	AK097398	Hs.654599	AK128739
UACA	7.3	2.7	uveal autoantigen with coiled-coil domains and ankyrin repeats (UACA), transcript variant 2, mRNA	NM_001008224	Hs.108049	NM_001008224
SEMA3C	7.3	16.4	sema domain, immunoglobulin domain (Ig), short basic domain, secreted, (semaphorin) 3C (SEMA3C), mRNA	NM_006379	Hs.269109	NM_006379
ARL13B	7.3	3.3	ADP-ribosylation factor-like 13B (ARL13B), transcript variant 1, mRNA	NM_182896	Hs.533086	CR936763
FLJ42709	7.3	4.5	hypothetical gene supported by AK124699, mRNA (cDNA clone IMAGE:5270292).	BC044619	Hs.457407	AK124699
COL8A2	7.3	6.4	collagen, type VIII, alpha 2 (COL8A2), mRNA	NM_005202	Hs.353001	NM_005202
FLJ31438	7.3	3.7	hypothetical protein FLJ31438 (FLJ31438), mRNA	NM_152385	Hs.468590	BC029502
COG6	7.3	1.8	component of oligomeric golgi complex 6 (COG6), mRNA	NM_020751	Hs.507805	AB032960
SLC39A6	7.3	1.7	solute carrier family 39 (zinc transporter), member 6 (SLC39A6), mRNA	NM_012319	Hs.79136	NM_012319
DKK2	7.3	14.0	dickkopf homolog 2 (Xenopus laevis) (DKK2), mRNA	NM_014421	Hs.211869	AF177395
THC2533385	7.3	2.4	THC2533385	THC2533385	Unknown	
ANGPT1	7.3	5.3	angiopoietin 1 (ANGPT1), mRNA	NM_001146	Hs.369675	BX648814
LOC254057	7.3	5.9	cDNA: FLJ21000 fis, clone CAE03359.	AK024653	Hs.586109	AK024653
PARD3	7.3	2.5	atypical PKC isotype-specific interacting protein long variant mRNA, complete cds.	AF196185	Hs.131489	AF196185
AA203154	7.2	4.2	AA203154 zx53h12.r1 Soares_fetal_liver_spleen_1NFLS_S1 cDNA clone IMAGE:446279 5' similar to contains Alu repetitive element;contains element LTR4 repetitive element ;, mRNA sequence	AA203154	Unknown	
THC2617409	7.2	4.4	Q6F0E0_MESFL (Q6F0E0) Recombination protein, partial (8%)	THC2617409	Unknown	
AK095600	7.2	2.3	cDNA FLJ38281 fis, clone FCBBF3005729, moderately similar to GIOT-4 mRNA for gonadotropin inducible transcription repressor-4.	AK095600	Unknown	
VPS24	7.2	4.1	vacuolar protein sorting 24 homolog (S. cerevisiae) (VPS24), transcript variant 1, mRNA	NM_016079	Hs.591582	NM_016079
AK056230	7.2	9.0	cDNA FLJ31668 fis, clone NT2RI2004916.	AK056230	Hs.533357	BC035889
PLG	7.2	14.7	plasminogen (PLG), mRNA	NM_000301	Hs.143436	CR749293
TTC12	7.2	2.9	tetratricopeptide repeat domain 12 (TTC12), mRNA	NM_017868	Hs.288772	AK125909
RAD23B	7.2	2.3	RAD23 homolog B (S. cerevisiae) (RAD23B), mRNA	NM_002874	Hs.521640	NM_002874
ENST00000369334	7.2	3.6	Notch homolog 2 N-terminal like protein	ENST00000369334	Unknown	
NIN	7.2	3.0	ninein (GSK3B interacting protein) (NIN), transcript variant 2, mRNA	NM_020921	Hs.310429	NM_020921
THC2646628	7.2	50.7	THC2646628	THC2646628	Unknown	
NFE2L2	7.2	2.1	nuclear factor (erythroid-derived 2)-like 2 (NFE2L2), mRNA	NM_006164	Hs.155396	NM_006164
SORCS3	7.2	15.7	sortilin-related VPS10 domain containing receptor 3 (SORCS3), mRNA	NM_014978	Hs.671950	AB028982
HSD17B11	7.2	3.0	hydroxysteroid (17-beta) dehydrogenase 11 (HSD17B11), mRNA	NM_016245	Hs.284414	NM_178135
ZNF83	7.2	1.9	zinc finger protein 83 (ZNF83), mRNA	NM_018300	Hs.467210	BX647540

FAM89B	7.2	3.7	family with sequence similarity 89, member B (FAM89B), mRNA	NM_152832	Hs.25723	BC023991
RAB22A	7.2	2.1	RAB22A, member RAS oncogene family (RAB22A), mRNA	NM_020673	Hs.529044	NM_020673
ZNF84	7.2	1.9	zinc finger protein 84 (ZNF84), mRNA	NM_003428	Hs.654730	BC036656
PLAG1	7.2	1.8	pleiomorphic adenoma gene 1 (PLAG1), mRNA	NM_002655	Hs.14968	NM_002655
THC2719717	7.2	3.7	THC2719717	THC2719717	Unknown	
ZFP36L1	7.2	2.6	zinc finger protein 36, C3H type-like 1 (ZFP36L1), mRNA	NM_004926	Hs.85155	AK024202
BC018675	7.2	5.7	Homo sapiens, clone IMAGE:4332461, mRNA.	BC018675	Hs.191841	AK023739
CP110	7.2	1.9	CP110 protein (CP110), mRNA	NM_014711	Hs.279912	NM_014711
TCBA1	7.2	4.0	T-cell lymphoma breakpoint associated target 1 (TCBA1), mRNA	NM_001040214	Hs.656604	NM_001040214
AK055981	7.2	2.1	cDNA FLJ31419 fis, clone NT2NE2000356.	AK055981	Hs.655249	AK055981
A2M	7.2	17.7	alpha-2-macroglobulin (A2M), mRNA	NM_000014	Hs.212838	CR749334
SMAD3	7.2	2.7	SMAD family member 3 (SMAD3), mRNA	NM_005902	Hs.36915	NM_005902
BC037827	7.2	9.0	cDNA clone IMAGE:4811567.	BC037827	Hs.648649	BC037827
COL3A1	7.2	20.3	collagen, type III, alpha 1 (Ehlers-Danlos syndrome type IV, autosomal dominant) (COL3A1), mRNA	NM_000090	Hs.443625	NM_000090
MSX2	7.2	3.6	msh homeobox 2 (MSX2), mRNA	NM_002449	Hs.89404	NM_002449
A_24_P919283	7.2	3.2	A_24_P919283	A_24_P919283	Unknown	
INSM1	7.2	4.6	insulinoma-associated 1 (INSM1), mRNA	NM_002196	Hs.89584	NM_002196
MTHFR	7.1	2.2	5,10-methylenetetrahydrofolate reductase (NADPH) (MTHFR), mRNA	NM_005957	Hs.214142	NM_005957
SMAD3	7.1	2.7	SMAD family member 3 (SMAD3), mRNA	NM_005902	Hs.36915	NM_005902
LMO2	7.1	11.4	LIM domain only 2 (rhombotin-like 1) (LMO2), mRNA	NM_005574	Hs.34560	NM_005574
GHR	7.1	4.4	growth hormone receptor (GHR), mRNA	NM_000163	Hs.125180	X06562
SMAD3	7.1	2.7	SMAD family member 3 (SMAD3), mRNA	NM_005902	Hs.36915	NM_005902
AB046850	7.1	1.9	mRNA for KIAA1630 protein, partial cds.	AB046850	Unknown	
LMO2	7.1	11.0	LIM domain only 2 (rhombotin-like 1) (LMO2), mRNA	NM_005574	Hs.34560	NM_005574
KIDINS220	7.1	3.0	kinase D-interacting substance of 220 kDa (KIDINS220), mRNA	NM_020738	Hs.9873	AB033076
USP47	7.1	2.3	ubiquitin specific peptidase 47 (USP47), mRNA	NM_017944	Hs.577256	NM_017944
LMO2	7.1	11.1	LIM domain only 2 (rhombotin-like 1) (LMO2), mRNA	NM_005574	Hs.34560	NM_005574
GNPDA2	7.1	3.2	glucosamine-6-phosphate deaminase 2 (GNPDA2), mRNA	NM_138335	Hs.21398	AB209904
PTPRO	7.1	4.6	protein tyrosine phosphatase, receptor type, O (PTPRO), transcript variant 1, mRNA	NM_030667	Hs.160871	NM_030667
ATP2A2	7.1	2.6	ATPase, Ca++ transporting, cardiac muscle, slow twitch 2 (ATP2A2), transcript variant 2, mRNA	NM_001681	Hs.506759	BX648282
CR616309	7.1	3.3	full-length cDNA clone CS0DF015YK23 of Fetal brain of (human).	CR616309	Hs.701350	CR616309
BC040982	7.1	2.6	cDNA clone IMAGE:4798675.	BC040982	Hs.656958	BC040982
MEIS2	7.1	28.7	Meis1, myeloid ecotropic viral integration site 1 homolog 2 (mouse) (MEIS2), transcript variant d, mRNA	NM_170676	Hs.510989	NM_170677
DLC1	7.1	5.0	deleted in liver cancer 1 (DLC1), transcript variant 1, mRNA	NM_182643	Hs.134296	NM_182643
RPS6KA5	7.1	2.7	ribosomal protein S6 kinase, 90kDa, polypeptide 5 (RPS6KA5), transcript variant 1, mRNA	NM_004755	Hs.510225	AB209667
ERBB2IP	7.1	2.5	erbb2 interacting protein (ERBB2IP), transcript variant 2, mRNA	NM_018695	Hs.591774	NM_018695
RFXDC1	7.1	18.5	regulatory factor X domain containing 1 (RFXDC1), mRNA	NM_173560	Hs.352276	NM_173560
FLJ31951	7.1	2.5	hypothetical protein FLJ31951 (FLJ31951), mRNA	NM_144726	Hs.349306	BC042684
NKD1	7.1	2.8	naked cuticle homolog 1 (Drosophila) (NKD1), mRNA	NM_033119	Hs.592059	BC051288
WNT11	7.1	7.5	wingless-type MMTV integration site family, member 11 (WNT11), mRNA	NM_004626	Hs.108219	Y12692
C6orf165	7.1	7.4	CMP-sialic acid transporter (CMP-Sia-Tr) (CMP-SA-Tr) (Solute carrier family 35 member A1).	ENST00000369562	Unknown	
SNX14	7.1	2.0	sorting nexin 14 (SNX14), transcript variant 1, mRNA	NM_153816	Hs.485871	AK095380
PCF11	7.1	1.8	PCF11, cleavage and polyadenylation factor subunit, homolog (S. cerevisiae) (PCF11), mRNA	NM_015885	Hs.128959	BC146778
LMO2	7.1	11.1	LIM domain only 2 (rhombotin-like 1) (LMO2), mRNA	NM_005574	Hs.34560	NM_005574
THC2582042	7.1	2.0	BC031749 potassium channel tetramerisation domain containing 15 {Mus musculus} (exp=-1; wgp=0; cg=0), partial (10%)	THC2582042	Unknown	
THC2501618	7.1	3.4	O51948_NEIGO (O51948) Outer membrane protein I precursor, partial (6%)	THC2501618	Unknown	
H3F3B	7.1	1.8	H3 histone, family 3B (H3.3B) (H3F3B), mRNA	NM_005324	Hs.180877	BX537379
DDHD2	7.1	2.7	DDHD domain containing 2	ENST00000319246	Unknown	
COL14A1	7.1	2.5	collagen, type XIV, alpha 1 (undulin) (COL14A1), mRNA	NM_021110	Hs.409662	NM_021110
DKK2	7.1	4.6	dickkopf homolog 2 (Xenopus laevis) (DKK2), mRNA	NM_014421	Hs.211869	AF177395
GSTM3	7.1	8.8	glutathione S-transferase M3 (brain) (GSTM3), mRNA	NM_000849	Hs.2006	NM_000849
FHOD3	7.1	2.7	formin homology 2 domain containing 3 (FHOD3), mRNA	NM_025135	Hs.436636	NM_025135

CNTN2	7.1	8.1	contactin 2 (axonal) (CNTN2), mRNA	NM_005076	Hs.519220	NM_005076
AF339771	7.1	2.9	clone IMAGE:1257951, mRNA sequence.	AF339771	Hs.344872	AF339771
RCBTB2	7.1	3.5	regulator of chromosome condensation (RCC1) and BTB (POZ) domain containing protein 2 (RCBTB2), mRNA	NM_001268	Hs.657385	NM_001268
A_32_P18630	7.1	3.0	A_32_P18630	A_32_P18630	Unknown	
PAX8	7.1	5.1	paired box gene 8 (PAX8), transcript variant PAX8A, mRNA	NM_003466	Hs.469728	NM_003466
BF760214	7.1	18.0	BF760214 CM1-CT0638-201200-683-e08 CT0638 cDNA, mRNA sequence	BF760214	Hs.443625	NM_000090
LSMD1	7.1	2.5	LSM domain containing 1 (LSMD1), mRNA	NM_032356	Hs.565094	NM_032356
KIAA1713	7.1	6.4	cDNA FLJ16608 fis, clone TESTI4011070.	AK131454	Hs.464876	NM_030632
LMO2	7.0	10.8	LIM domain only 2 (rhombotin-like 1) (LMO2), mRNA	NM_005574	Hs.34560	NM_005574
KIAA1370	7.0	2.6	KIAA1370 (KIAA1370), mRNA	NM_019600	Hs.152385	NM_019600
VTI1A	7.0	2.2	vesicle transport through interaction with t-SNAREs homolog 1A (yeast) (VTI1A), transcript variant 2, mRNA	NM_145206	Hs.194554	NM_145206
BC062753	7.0	3.0	cDNA clone IMAGE:3933366, partial cds.	BC062753	Hs.655589	BM455300
THC2523793	7.0	3.5	Q9DC07_MOUSE (Q9DC07) Adult male lung cDNA, RIKEN full-length enriched library, clone:1200007O21 product:LIM AND SH3 PROTEIN (LASP Homolog), full insert sequence (Nebulette), partial (11%)	THC2523793	Unknown	
ZNF516	7.0	2.9	Zinc finger protein 516.	ENST00000217537	Unknown	
BC030100	7.0	3.6	cDNA clone IMAGE:4796595.	BC030100	Hs.562145	BQ049185
AHSG	7.0	43.9	alpha-2-HS-glycoprotein (AHSG), mRNA	NM_001622	Hs.324746	BC048198
THC2604598	7.0	4.3	AF154107 UDP-GalNAc:polypeptide N-acetylgalactosaminyltransferase 5 {Homo sapiens} (exp=-1; wgp=0; cg=0), partial (25%)	THC2604598	Unknown	
THC2666431	7.0	4.4	THC2666431	THC2666431	Unknown	
SKIP	7.0	107.8	SPHK1 (sphingosine kinase type 1) interacting protein (SKIP), mRNA	NM_030623	Hs.436306	CR749494
DMRTC1	7.0	16.8	DMRT-like family C1 (DMRTC1), mRNA	NM_033053	Hs.658177	NM_033053
PLN	7.0	218.9	cDNA FLJ26334 fis, clone HRT02648.	AK129844	Hs.170839	AK129844
LDOC1L	7.0	2.9	leucine zipper, down-regulated in cancer 1-like (LDOC1L), mRNA	NM_032287	Hs.332795	NM_032287
A_32_P47874	7.0	5.0	A_32_P47874	A_32_P47874	Unknown	
MTSS1	7.0	6.4	metastasis suppressor 1 (MTSS1), mRNA	NM_014751	Hs.336994	NM_014751
PTN	7.0	4.4	pleiotrophin (heparin binding growth factor 8, neurite growth-promoting factor 1) (PTN), mRNA	NM_002825	Hs.371249	CR624136
ADCY7	7.0	3.0	adenylate cyclase 7 (ADCY7), mRNA	NM_001114	Hs.513578	D25538
NT5C3	7.0	2.2	5'-nucleotidase, cytosolic III (NT5C3), transcript variant 2, mRNA	NM_001002009	Hs.487933	NM_001002009
GJA3	7.0	11.6	gap junction protein, alpha 3, 46kDa (connexin 46) (GJA3), mRNA	NM_021954	Hs.130313	NM_021954
LPIN2	7.0	2.4	lipin 2 (LPIN2), mRNA	NM_014646	Hs.132342	NM_014646
C14orf132	7.0	2.4	chromosome 14 open reading frame 132 (C14orf132), mRNA	NM_020215	Unknown	
C12orf29	7.0	2.3	chromosome 12 open reading frame 29 (C12orf29), mRNA	NM_001009894	Hs.591009	CR936776
ZNF222	7.0	1.8	zinc finger protein 222 (ZNF222), mRNA	NM_013360	Hs.279840	AK095196
THC2514496	7.0	2.5	THC2514496	THC2514496	Unknown	
AK128396	7.0	2.4	cDNA FLJ46539 fis, clone THYMU3037836.	AK128396	Hs.572086	AK128396
UGT2B7	7.0	5.0	UDP glucuronosyltransferase 2 family, polypeptide B7 (UGT2B7), mRNA	NM_001074	Hs.654424	J05428
AK025344	7.0	2.2	cDNA: FLJ21691 fis, clone COL09555.	AK025344	Hs.476353	NM_018403
THC2674306	7.0	9.8	Q9F8M7_CARHY (Q9F8M7) DTDP-glucose 4,6-dehydratase (Fragment), partial (11%)	THC2674306	Unknown	
IKZF4	7.0	2.2	mRNA; cDNA DKFZp686K2231 (from clone DKFZp686K2231).	BX647761	Hs.553221	NM_022465
AK096020	7.0	1.9	cDNA FLJ38701 fis, clone KIDNE2002198.	AK096020	Hs.435845	NM_004452
TNFRSF19	7.0	4.9	tumor necrosis factor receptor superfamily, member 19 (TNFRSF19), transcript variant 1, mRNA	NM_018647	Hs.149168	NM_148957
PHF20L1	7.0	4.1	PHD finger protein 20-like 1 (PHF20L1), transcript variant 3, mRNA	NM_198513	Hs.304362	NM_016018
LOC120379	7.0	3.1	hypothetical protein BC019238 (LOC120379), mRNA	NM_138789	Hs.420662	NM_001082619
TTC25	7.0	3.5	tetratricopeptide repeat domain 25 (TTC25), mRNA	NM_031421	Hs.201134	NM_031421
FILIP1	7.0	31.6	mRNA; cDNA DKFZp451B134 (from clone DKFZp451B134); complete cds.	AL832009	Hs.696158	AL832009
GPR161	7.0	2.3	G protein-coupled receptor 161 (GPR161), transcript variant 2, mRNA	NM_153832	Hs.632453	BC028163
LMO2	7.0	11.1	LIM domain only 2 (rhombotin-like 1) (LMO2), mRNA	NM_005574	Hs.34560	NM_005574
CREM	7.0	3.2	cAMP responsive element modulator (CREM), transcript variant 19, mRNA	NM_183013	Hs.200250	AB209533
SSFA2	7.0	8.0	sperm specific antigen 2 (SSFA2), mRNA	NM_006751	Hs.591602	BX648182
AK090762	7.0	4.7	cDNA FLJ33443 fis, clone BRALZ1000103.	AK090762	Hs.531632	AK090762
KALRN	7.0	3.1	full-length cDNA clone CS0DF007YD09 of Fetal brain of (human).	CR605792	Hs.8004	NM_001024660
CD248	7.0	6.7	CD248 molecule, endosialin (CD248), mRNA	NM_020404	Hs.195727	BC051340
FYTTD1	6.9	2.4	forty-two-three domain containing 1 (FYTTD1), transcript variant 2, mRNA	NM_001011537	Hs.277533	NM_001011537

TBCEL	6.9	2.2	leucine rich repeat containing 35, mRNA (cDNA clone IMAGE:3913004).	BC020501	Hs.632108	BC020501
SORBS2	6.9	4.6	sorbin and SH3 domain containing 2 (SORBS2), transcript variant 2, mRNA	NM_021069	Hs.655143	NM_021069
AHSG	6.9	21.3	alpha-2-HS-glycoprotein (AHSG), mRNA	NM_001622	Hs.324746	BC048198
ZFHX1B	6.9	6.9	zinc finger homeobox 1b (ZFHX1B), mRNA	NM_014795	Hs.34871	NM_014795
FLJ39609	6.9	3.9	cDNA FLJ39609 fis, clone SKNSH2008043.	AK096928	Hs.200644	AK096928
MGC33846	6.9	8.6	cDNA FLJ37735 fis, clone BRHIP2020859.	AK095054	Unknown	
C6orf165	6.9	4.5	chromosome 6 open reading frame 165 (C6orf165), transcript variant 1, mRNA	NM_001031743	Hs.82921	NM_178823
RCBTB2	6.9	1.9	cDNA FLJ43180 fis, clone FCBBF3013846.	AK125170	Hs.25447	AK125170
USP47	6.9	2.6	ubiquitin specific peptidase 47 (USP47), mRNA	NM_017944	Hs.577256	NM_017944
IL18R1	6.9	6.7	interleukin 18 receptor 1 (IL18R1), mRNA	NM_003855	Hs.469521	NM_003855
THC2739159	6.9	3.2	ALU8_HUMAN (P39195) Alu subfamily SX sequence contamination warning entry, partial (8%)	THC2739159	Unknown	
SPATA7	6.9	2.4	spermatogenesis associated 7 (SPATA7), transcript variant 1, mRNA	NM_018418	Hs.525518	BC090875
KLHL14	6.9	3.6	kelch-like 14 (Drosophila) (KLHL14), mRNA	NM_020805	Hs.446164	AB037805
GJB2	6.9	10.5	gap junction protein, beta 2, 26kDa (connexin 26) (GJB2), mRNA	NM_004004	Hs.591234	NM_004004
AK123438	6.9	6.2	cDNA FLJ41444 fis, clone BRSTN2001801.	AK123438	Hs.659105	AK123438
THC2665663	6.9	16.5	THC2665663	THC2665663	Unknown	
BC038432	6.9	5.2	cDNA clone IMAGE:5162874, partial cds.	BC038432	Hs.655717	BC038432
CUEDC1	6.9	3.2	CUE domain containing 1 (CUEDC1), mRNA	NM_017949	Hs.46679	CR627470
DMD	6.9	3.3	dystrophin (muscular dystrophy, Duchenne and Becker types) (DMD), transcript variant Dp427p2, mRNA	NM_004010	Hs.495912	NM_004010
NBPF3	6.9	3.4	neuroblastoma breakpoint family, member 3 (NBPF3), mRNA	NM_032264	Hs.325422	AK095602
SLU7	6.9	1.7	SLU7 splicing factor homolog (S. cerevisiae) (SLU7), mRNA	NM_006425	Hs.435342	NM_006425
TERF2IP	6.9	2.9	telomeric repeat binding factor 2, interacting protein (TERF2IP), mRNA	NM_018975	Hs.301419	AF289599
ST6GALNAC3	6.9	4.4	ST6 (alpha-N-acetyl-neuraminyl-2,3-beta-galactosyl-1,3)-N-acetylgalactosaminide alpha-2,6-sialyltransferase 3 (ST6GALNAC3), mRNA	NM_152996	Hs.337040	BX648274
BC048201	6.9	3.1	Homo sapiens, clone IMAGE:3660074, mRNA.	BC048201	Hs.558872	BC070147
C12orf46	6.9	6.8	chromosome 12 open reading frame 46 (C12orf46), mRNA	NM_152321	Hs.162143	BC030218
MICAL2	6.9	4.3	microtubule associated monoxygenase, calponin and LIM domain containing 2 (MICAL2), mRNA	NM_014632	Hs.501928	BX538021
FBXO11	6.9	1.9	F-box protein 11 (FBXO11), transcript variant 1, mRNA	NM_025133	Hs.352677	BC043258
ITGB1BP2	6.9	6.0	integrin beta 1 binding protein (melusin) 2 (ITGB1BP2), mRNA	NM_012278	Hs.109999	BM808220
BC030112	6.9	3.0	cDNA clone IMAGE:4799578.	BC030112	Hs.201918	NM_005734
CPE	6.9	4.3	carboxypeptidase E (CPE), mRNA	NM_001873	Hs.699184	NM_001873
AK095564	6.9	4.2	cDNA FLJ38245 fis, clone FCBBF2007186.	AK095564	Hs.697104	AK095564
CASC1	6.9	6.3	cancer susceptibility candidate 1 (CASC1), mRNA	NM_018272	Hs.407771	NM_001082972
NFIB	6.9	4.8	nuclear factor I/B (NFIB), mRNA	NM_005596	Hs.699215	NM_005596
C6orf118	6.9	4.9	chromosome 6 open reading frame 118 (C6orf118), mRNA	NM_144980	Hs.144734	BC026278
THC2625884	6.9	3.7	THC2625884	THC2625884	Unknown	
HES4	6.9	3.4	hairy and enhancer of split 4 (Drosophila) (HES4), mRNA	NM_021170	Hs.154029	BM543941
ZNF438	6.9	2.5	zinc finger protein 438 (ZNF438), mRNA	NM_182755	Hs.660642	AK097092
TMEM169	6.9	3.4	transmembrane protein 169 (TMEM169), mRNA	NM_138390	Hs.334916	NM_138390
LMO2	6.9	10.9	LIM domain only 2 (rhombotin-like 1) (LMO2), mRNA	NM_005574	Hs.34560	NM_005574
HGD	6.9	5.9	homogentisate 1,2-dioxygenase (homogentisate oxidase) (HGD), mRNA	NM_000187	Hs.368254	NM_000187
LOC399947	6.9	3.2	LOH11CR1A gene, loss of heterozygosity, 11, chromosomal region 1 gene A product.	AB096240	Hs.172982	AB096240
KLHDC8B	6.9	3.2	kelch domain containing 8B (KLHDC8B), mRNA	NM_173546	Hs.13781	NM_173546
MORN2	6.9	2.8	MORN repeat containing 2 (MORN2), mRNA	NM_194270	Unknown	
CR611332	6.9	2.4	full-length cDNA clone CS0DF014YA22 of Fetal brain of (human).	CR611332	Hs.592631	CR611332
MYBPC3	6.9	12.9	myosin binding protein C, cardiac (MYBPC3), mRNA	NM_000256	Hs.524906	BC142685
AW268902	6.9	7.7	AW268902 xv48h10.x1 Soares_NFL_T_GBC_S1 cDNA clone IMAGE:2816419 3', mRNA sequence	AW268902	Hs.699467	AF055585
BBS1	6.9	2.2	Bardet-Biedl syndrome 1 (BBS1), mRNA	NM_024649	Hs.502915	AK095638
THC2668561	6.9	12.1	THC2668561	THC2668561	Unknown	
CUL3	6.9	2.9	cullin 3 (CUL3), mRNA	NM_003590	Hs.372286	NM_003590
IFIT5	6.9	2.4	interferon-induced protein with tetratricopeptide repeats 5 (IFIT5), mRNA	NM_012420	Hs.252839	NM_012420
THC2650514	6.9	2.5	THC2650514	THC2650514	Unknown	
THC2609493	6.9	23.2	Q9U4W7_PLAFA (Q9U4W7) DBL alpha protein (Fragment), partial (14%)	THC2609493	Unknown	
CDKL3	6.9	2.0	cyclin-dependent kinase-like 3 (CDKL3), mRNA	NM_016508	Hs.105818	BC041799

WDR31	6.8	3.6	WD repeat domain 31 (WDR31), transcript variant 1, mRNA	NM_001012361	Hs.133331	BC012352
SERPINA6	6.8	4.9	serpin peptidase inhibitor, clade A (alpha-1 antiproteinase, antitrypsin), member 6 (SERPINA6), mRNA	NM_001756	Hs.532635	BC036818
EPHA7	6.8	5.6	Ephrin type-A receptor 7 precursor (EC 2.7.10.1) (Tyrosine-protein kinase receptor EHK-3) (EPH homology kinase 3) (Receptor protein- tyrosine kinase HEK11).	ENST00000369303	Unknown	
ARL5B	6.8	1.8	ADP-ribosylation factor-like protein 5B (ADP-ribosylation factor-like protein 8).	ENST00000377275	Unknown	
GPRASP1	6.8	3.7	G protein-coupled receptor associated sorting protein 1 (GPRASP1), mRNA	NM_014710	Hs.522729	NM_014710
TPST1	6.8	2.8	tyrosylprotein sulfotransferase 1 (TPST1), mRNA	NM_003596	Hs.421194	NM_003596
CLIC5	6.8	6.9	chloride intracellular channel 5 (CLIC5), mRNA	NM_016929	Hs.485489	DQ679794
SLIT3	6.8	8.1	slit homolog 3 (Drosophila) (SLIT3), mRNA	NM_003062	Hs.604116	BC098388
AK130514	6.8	3.7	cDNA FLJ27004 fis, clone SLV04966.	AK130514	Hs.699296	AL833852
AF305816	6.8	3.4	PRO0633 mRNA, complete cds.	AF305816	Hs.575067	AK022313
NRXN1	6.8	2.9	neurexin 1 (NRXN1), transcript variant alpha, mRNA	NM_004801	Hs.637685	BC150247
THC2668359	6.8	4.2	THC2668359	THC2668359	Unknown	
BX647543	6.8	5.8	mRNA; cDNA DKFZp779F2345 (from clone DKFZp779F2345).	BX647543	Hs.444595	BX647543
AK026078	6.8	3.2	cDNA: FLJ22425 fis, clone HRC08686.	AK026078	Hs.591015	NM_005230
C14orf44	6.8	2.5	chromosome 14 open reading frame 44 (C14orf44), mRNA	NM_152445	Hs.660789	AK056259
LDOC1L	6.8	2.8	leucine zipper, down-regulated in cancer 1-like (LDOC1L), mRNA	NM_032287	Hs.332795	NM_032287
GRK5	6.8	9.6	G protein-coupled receptor kinase 5 (GRK5), mRNA	NM_005308	Hs.524625	AK097099
PARC	6.8	3.1	p53-associated parkin-like cytoplasmic protein (PARC), mRNA	NM_015089	Hs.485434	CR749511
KIAA0430	6.8	2.2	KIAA0430 (KIAA0430), mRNA	NM_014647	Hs.173524	NM_014647
S100B	6.8	5.2	S100 calcium binding protein B (S100B), mRNA	NM_006272	Hs.422181	BC041935
KIAA1799	6.8	6.1	KIAA1799 protein (KIAA1799), mRNA	NM_032437	Hs.652324	AB058702
WDR63	6.8	2.9	WD repeat domain 63 (WDR63), mRNA	NM_145172	Hs.97933	BX648851
TCF12	6.8	3.4	transcription factor 12 (HTF4, helix-loop-helix transcription factors 4) (TCF12), transcript variant 4, mRNA	NM_207038	Hs.511504	NM_207037
LOC388401	6.8	1.6	PREDICTED: similar to 60S ribosomal protein L7 (LOC388401), mRNA	XR_016879	Hs.646579	XR_016879
GRIA2	6.8	9.2	glutamate receptor, ionotropic, AMPA 2 (GRIA2), mRNA	NM_000826	Hs.32763	NM_000826
RP3-377H14.5	6.8	1.7	cDNA FLJ35429 fis, clone SMINT2002126.	AK092748	Hs.655949	AL832418
BC042026	6.8	60.4	cDNA clone IMAGE:5312689.	BC042026	Hs.201488	BC042026
NBPF3	6.8	5.4	neuroblastoma breakpoint family, member 3 (NBPF3), mRNA	NM_032264	Hs.325422	AK095602
LRRC4C	6.8	18.3	leucine rich repeat containing 4C (LRRC4C), mRNA	NM_020929	Hs.135736	AB046800
BF312639	6.8	3.3	BF312639 601898132F1 NIH_MGC_19 cDNA clone IMAGE:4127478 5', mRNA sequence	BF312639	Hs.655654	U79716
BQ716254	6.8	15.3	AGENCOURT_8291361 Lupski_sympathetic_trunk cDNA clone IMAGE:6194571 5', mRNA sequence	BQ716254	Hs.596087	BQ716254
AKT3	6.8	3.3	RAC-gamma serine/threonine-protein kinase (EC 2.7.11.1) (RAC-PK-gamma) (Protein kinase Akt-3) (Protein kinase B, gamma) (PKB gamma) (STK-2).	ENST00000366539	Unknown	
RSHL2	6.8	2.2	radial spokehead-like 2 (RSHL2), mRNA	NM_031924	Hs.154628	BC050604
FLJ23577	6.8	5.6	KPL2 protein (FLJ23577), transcript variant 2, mRNA	NM_144722	Hs.298863	AL832607
CR617560	6.8	2.7	full-length cDNA clone CS0DC013YG14 of Neuroblastoma Cot 25-normalized of (human).	CR617560	Hs.463010	BC005066
SLC4A4	6.8	5.0	solute carrier family 4, sodium bicarbonate cotransporter, member 4 (SLC4A4), mRNA	NM_003759	Hs.5462	NM_001098484
MTHFR	6.8	2.7	5,10-methylenetetrahydrofolate reductase (NADPH) (MTHFR), mRNA	NM_005957	Hs.214142	NM_005957
PDE4D	6.7	3.4	phosphodiesterase 4D, cAMP-specific (phosphodiesterase E3 dunce homolog, Drosophila) (PDE4D), mRNA	NM_006203	Hs.654358	NM_006203
LRIG3	6.7	2.5	leucine-rich repeats and immunoglobulin-like domains 3 (LRIG3), mRNA	NM_153377	Hs.253736	AY358288
CN364769	6.7	4.0	17000583182148 GRN_PRENEU cDNA 5', mRNA sequence	CN364769	Hs.596910	CN364769
GFRA1	6.7	2.1	GDNF family receptor alpha 1 (GFRA1), transcript variant 2, mRNA	NM_145793	Hs.591913	NM_005264
CNTN6	6.7	9.2	contactin 6 (CNTN6), mRNA	NM_014461	Hs.387300	AB003592
AUTS2	6.7	3.6	autism susceptibility candidate 2, mRNA (cDNA clone IMAGE:4131814), complete cds.	BC011643	Hs.700600	AF326917
FBN1	6.7	5.1	fibrillin 1 (FBN1), mRNA	NM_000138	Hs.591133	NM_000138
AF075112	6.7	3.0	full length insert cDNA YU76E12.	AF075112	Hs.559248	BQ006833
PPP1R14C	6.7	21.7	protein phosphatase 1, regulatory (inhibitor) subunit 14C (PPP1R14C), mRNA	NM_030949	Hs.486798	AF407165
SOX1	6.7	5.4	SRY (sex determining region Y)-box 1 (SOX1), mRNA	NM_005986	Hs.202526	NM_005986
CXCL12	6.7	3.5	chemokine (C-X-C motif) ligand 12 (stromal cell-derived factor 1) (CXCL12), transcript variant 1, mRNA	NM_199168	Hs.522891	AK090482
ENST00000373930	6.7	5.1	Multiple epidermal growth factor-like domains 9 precursor (EGF-like domain-containing protein 5) (Multiple EGF-like domain protein 5).	ENST00000373930	Unknown	
SAP18	6.7	2.3	Sin3A-associated protein, 18kDa (SAP18), mRNA	NM_005870	Hs.524899	AK126385

TKTL1	6.7	3.9	transketolase-like 1 (TKTL1), mRNA	NM_012253	Hs.102866	NM_012253
GABRA2	6.7	6.1	gamma-aminobutyric acid (GABA) A receptor, alpha 2 (GABRA2), mRNA	NM_000807	Hs.116250	AK125179
PLXDC1	6.7	5.3	plexin domain containing 1 (PLXDC1), mRNA	NM_020405	Hs.125036	NM_020405
LOC286272	6.7	2.6	cDNA FLJ10077 fis, clone HEMBA1001864.	AK000939	Hs.657301	AK000939
LOC497190	6.7	4.1	secretory protein LOC497190 (LOC497190), mRNA	NM_001011880	Hs.556045	AL833339
TRO	6.7	2.6	trophinin (TRO), transcript variant 3, mRNA	NM_016157	Hs.695979	NM_001039705
AXIN2	6.7	2.1	axin 2 (conductin, axil) (AXIN2), mRNA	NM_004655	Hs.156527	CR933657
CNTFR	6.7	5.7	ciliary neurotrophic factor receptor (CNTFR), transcript variant 1, mRNA	NM_147164	Hs.129966	AK127444
FCHO2	6.7	2.3	FCH domain only 2 (FCHO2), mRNA	NM_138782	Hs.165762	NM_138782
CGI-38	6.7	6.9	brain specific protein (CGI-38), mRNA	NM_016140	Hs.534458	CR622352
SSBP2	6.7	2.5	single-stranded DNA binding protein 2 (SSBP2), mRNA	NM_012446	Hs.699214	NM_012446
KIAA0738	6.7	2.2	KIAA0738 gene product (KIAA0738), mRNA	NM_014719	Hs.406492	AK001640
A_24_P341176	6.7	1.5	A_24_P341176	A_24_P341176	Unknown	
DPF3	6.7	3.9	D4, zinc and double PHD fingers, family 3 (DPF3), mRNA	NM_012074	Hs.162868	AK024141
SPARCL1	6.7	12.8	SPARC-like 1 (mast9, hevin) (SPARCL1), mRNA	NM_004684	Hs.62886	X86693
THC2770336	6.7	4.6	Q2Z8W5_9GAMM (Q2Z8W5) Thiolase , partial (5%)	THC2770336	Unknown	
FAM92A3	6.7	2.1	family with sequence similarity 92, member A3, mRNA (cDNA clone IMAGE:5265575), complete cds.	BC059396	Hs.652187	BC059396
CLN5	6.7	2.6	ceroid-lipofuscinosis, neuronal 5 (CLN5), mRNA	NM_006493	Hs.30213	AF068227
THC2651036	6.7	2.6	THC2651036	THC2651036	Unknown	
CR606969	6.7	2.7	full-length cDNA clone CS0DF003YF10 of Fetal brain of (human).	CR606969	Hs.553146	CR606969
ENST00000332696	6.7	1.6	similar to 60S ribosomal protein L23a (LOC644384), mRNA	ENST00000332696	Unknown	
KIAA0280	6.7	2.1	Human mRNA for KIAA0280 gene, partial cds.	D87470	Hs.475334	D87470
ONECUT1	6.7	3.5	one cut domain, family member 1 (ONECUT1), mRNA	NM_004498	Hs.658573	U96173
NTNG2	6.7	3.7	netrin G2 (NTNG2), mRNA	NM_032536	Hs.163642	AY358165
ALPK2	6.7	10.6	heart alpha-kinase (HAK), mRNA	NM_052947	Hs.656805	BX647796
DIP13B	6.7	2.7	DIP13 beta (DIP13B), mRNA	NM_018171	Hs.506603	BX649010
ENST00000340158	6.7	2.1	Exocyst complex component 3 (Exocyst complex component Sec6).	ENST00000340158	Unknown	
KIAA1328	6.7	2.3	KIAA1328 (KIAA1328), mRNA	NM_020776	Hs.4045	AB037749
NHLRC2	6.7	2.6	cDNA FLJ44798 fis, clone BRACE3040863.	AK126751	Hs.594372	BX647663
LOC375295	6.7	11.4	hypothetical gene supported by BC013438, mRNA (cDNA clone IMAGE:3899073), partial cds.	ENST00000295549	Unknown	
LOC285047	6.7	7.8	PREDICTED: hypothetical LOC285047 (LOC285047), mRNA	XM_211749	Hs.369330	XM_211749
PUNC	6.7	4.9	putative neuronal cell adhesion molecule (PUNC), mRNA	NM_004884	Hs.128292	NM_004884
FREM1	6.7	12.8	FRAS1 related extracellular matrix 1 (FREM1), mRNA	NM_144966	Hs.50850	BX648240
BEX2	6.7	1.7	brain expressed X-linked 2 (BEX2), mRNA	NM_032621	Hs.398989	BC050651
GPAM	6.7	1.6	glycerol-3-phosphate acyltransferase, mitochondrial (GPAM), mRNA	NM_020918	Hs.42586	AL833061
MMP23B	6.6	3.3	matrix metallopeptidase 23B (MMP23B), mRNA	NM_006983	Hs.192316	BM805143
SRD5A2L2	6.6	206.7	steroid 5 alpha-reductase 2-like 2 (SRD5A2L2), mRNA	NM_001010874	Hs.227752	AL833108
LOC375295	6.6	5.7	hypothetical gene supported by BC013438, mRNA (cDNA clone IMAGE:3899073), partial cds.	ENST00000295549	Unknown	
BM680083	6.6	1.8	BM680083 UI-E-EO1-aix-j-14-0-UI.s1 UI-E-EO1 cDNA clone UI-E-EO1-aix-j-14-0-UI 3', mRNA sequence	BM680083	Hs.432638	AB028641
Z99394	6.6	7.1	Z99394 HSZ99394 DKFZphamy1 cDNA clone DKFZphamy1_1c10 3', mRNA sequence	Z99394	Hs.615360	BX640646
BC038371	6.6	3.4	cDNA clone IMAGE:4829282.	BC038371	Hs.657602	BC038371
A_32_P224040	6.6	2.5	A_32_P224040	A_32_P224040	Unknown	
TTN	6.6	27.4	titin (TTN), transcript variant novex-3, mRNA	NM_133379	Hs.654592	NM_133378
C5orf25	6.6	1.8	chromosome 5 open reading frame 25 (C5orf25), mRNA	NM_198567	Hs.699402	BC037298
RHOU	6.6	9.8	ras homolog gene family, member U (RHOU), mRNA	NM_021205	Hs.647774	NM_021205
TMEM16A	6.6	4.0	transmembrane protein 16A (TMEM16A), mRNA	NM_018043	Hs.503074	AY728143
GPR155	6.6	3.4	G protein-coupled receptor 155 (GPR155), transcript variant 9, mRNA	NM_001033045	Hs.516604	NM_001033045
CCPG1	6.6	2.9	cell cycle progression 1 (CCPG1), transcript variant 2, mRNA	NM_020739	Hs.612814	NM_020739
ARHGEF3	6.6	3.7	Rho guanine nucleotide exchange factor (GEF) 3 (ARHGEF3), mRNA	NM_019555	Hs.476402	AB209661
THC2683470	6.6	5.8	THC2683470	THC2683470	Unknown	
OXR1	6.6	2.2	oxidation resistance 1 (OXR1), mRNA	NM_181354	Hs.148778	NM_181354
GUCY1B3	6.6	5.5	guanylate cyclase 1, soluble, beta 3 (GUCY1B3), mRNA	NM_000857	Hs.77890	BC047620
APOLD1	6.6	14.8	apolipoprotein L domain containing 1 (APOLD1), mRNA	NM_030817	Hs.23388	BC042478

TMTC4	6.6	1.9	transmembrane and tetratricopeptide repeat containing 4 (TMTC4), transcript variant 1, mRNA	NM_032813	Hs.696247	AK124599
VASN	6.6	4.4	vasorin (VASN), mRNA	NM_138440	Hs.372579	NM_138440
ENST00000382878	6.6	3.0	CH455 (Fragment).	ENST00000382878	Unknown	
HS2ST1	6.6	2.8	heparan sulfate 2-O-sulfotransferase 1 (HS2ST1), mRNA	NM_012262	Hs.48823	NM_012262
NRTN	6.6	2.4	neurturin (NRTN), mRNA	NM_004558	Hs.234775	AL161995
CDH7	6.6	5.0	cadherin 7, type 2 (CDH7), transcript variant b, mRNA	NM_004361	Hs.657522	BC036786
LOC149134	6.6	6.3	hypothetical protein LOC149134 (LOC149134), mRNA	NM_207326	Unknown	
C14orf111	6.6	2.5	chromosome 14 open reading frame 111 (C14orf111), mRNA	NM_015962	Hs.579828	BX647810
AL049990	6.6	46.2	mRNA; cDNA DKFZp564G112 (from clone DKFZp564G112).	AL049990	Hs.90250	NM_024574
NBPF15	6.6	5.8	neuroblastoma breakpoint family, member 15 (NBPF15), mRNA	NM_173638	Hs.655246	NM_173638
SLC2A5	6.6	4.1	solute carrier family 2 (facilitated glucose/fructose transporter), member 5 (SLC2A5), mRNA	NM_003039	Hs.530003	BC035878
RBM4B	6.6	2.4	RNA binding motif protein 4B (RBM4B), mRNA	NM_031492	Hs.656291	AK095158
ARL6IP5	6.6	2.7	ADP-ribosylation-like factor 6 interacting protein 5 (ARL6IP5), mRNA	NM_006407	Hs.518060	NM_006407
TBC1D12	6.6	2.9	partial mRNA for KIAA0608 transcript, 3' UTR.	AJ404330	Hs.500598	NM_015188
RPL36A	6.6	1.6	ribosomal protein L36a (RPL36A), mRNA	NM_021029	Hs.432485	CR601778
AK056630	6.6	3.7	cDNA FLJ32068 fis, clone OCBBF1000114.	AK056630	Hs.694880	AK056630
LOC441257	6.6	3.8	hypothetical LOC441257 (LOC441257), mRNA	NM_001023562	Unknown	
ENST00000380344	6.6	2.1	Beta-1,3-glucosyltransferase (EC 2.4.1.-) (Beta3Glc-T) (Beta-3-glycosyltransferase-like).	ENST00000380344	Unknown	
THC2741529	6.6	14.7	Q9F8M7_CARHY (Q9F8M7) DTDP-glucose 4,6-dehydratase (Fragment), partial (11%)	THC2741529	Unknown	
MTR	6.6	2.3	5-methyltetrahydrofolate-homocysteine methyltransferase (MTR), mRNA	NM_000254	Hs.498187	U73338
CCDC14	6.6	1.5	coiled-coil domain containing 14 (CCDC14), mRNA	NM_022757	Hs.656256	BX537652
CCDC80	6.6	11.5	coiled-coil domain containing 80 (CCDC80), transcript variant 1, mRNA	NM_199511	Hs.477128	NM_199511
SGIP1	6.6	8.9	SH3-domain GRB2-like (endophilin) interacting protein 1 (SGIP1), mRNA	NM_032291	Hs.132121	AB210039
CFL2	6.6	2.8	cofilin 2 (muscle) (CFL2), transcript variant 1, mRNA	NM_021914	Hs.180141	NM_021914
RHOBTB3	6.6	3.2	Rho-related BTB domain containing 3 (RHOBTB3), mRNA	NM_014899	Hs.445030	NM_014899
GSTA1	6.6	7.1	glutathione S-transferase A1 (GSTA1), mRNA	NM_145740	Hs.446309	AL096729
KLB	6.6	4.2	Beta klotho (BetaKlotho) (Klotho beta-like protein).	ENST00000381889	Unknown	
C11orf69	6.6	3.1	chromosome 11 open reading frame 69 (C11orf69), mRNA	NM_152314	Unknown	
EPC2	6.6	1.8	enhancer of polycomb homolog 2 (Drosophila) (EPC2), mRNA	NM_015630	Hs.23270	AK125789
A_32_P222241	6.6	15.0	A_32_P222241	A_32_P222241	Unknown	
STMN1	6.6	2.7	stathmin 1/oncoprotein 18 (STMN1), transcript variant 1, mRNA	NM_203401	Hs.700575	BX647885
MTHFR	6.6	2.7	5,10-methylenetetrahydrofolate reductase (NADPH) (MTHFR), mRNA	NM_005957	Hs.214142	NM_005957
A_23_P170713	6.6	2.2	A_23_P170713	A_23_P170713	Unknown	
GJB2	6.6	7.4	gap junction protein, beta 2, 26kDa (connexin 26) (GJB2), mRNA	NM_004004	Hs.591234	NM_004004
A_24_P298238	6.6	1.8	A_24_P298238	A_24_P298238	Unknown	
BNC1	6.5	92.7	basonuclin 1 (BNC1), mRNA	NM_001717	Hs.459153	L03427
SYNPO2	6.5	21.3	mRNA; cDNA DKFZp451G189 (from clone DKFZp451G189).	AL833294	Hs.655519	CR936673
RGS13	6.5	12.7	regulator of G-protein signalling 13 (RGS13), transcript variant 1, mRNA	NM_002927	Hs.497220	BC036950
BEX1	6.5	2.1	brain expressed, X-linked 1 (BEX1), mRNA	NM_018476	Hs.334370	BM804232
RSPO2	6.5	9.4	R-spondin 2 homolog (Xenopus laevis) (RSPO2), mRNA	NM_178565	Hs.444834	AK123023
MIA	6.5	4.8	melanoma inhibitory activity (MIA), mRNA	NM_006533	Hs.646364	BG765502
BC033539	6.5	5.4	cDNA clone IMAGE:4819052.	BC033539	Hs.524828	AL834266
RABL5	6.5	2.5	RAB, member RAS oncogene family-like 5 (RABL5), mRNA	NM_022777	Hs.389104	AL157469
AF086071	6.5	3.2	full length insert cDNA clone YZ38E04.	AF086071	Hs.197962	AK125077
IK	6.5	1.7	IK cytokine, down-regulator of HLA II, mRNA (cDNA clone MGC:88657 IMAGE:6461871), complete cds.	BC071964	Hs.421245	AK225924
COL1A2	6.5	5.0	collagen, type I, alpha 2 (COL1A2), mRNA	NM_000089	Hs.489142	J03464
PRDM6	6.5	3.4	PR domain zinc finger protein 6 (PR domain-containing protein 6) (Fragment).	ENST00000261364	Unknown	
AXIN2	6.5	2.1	conductin mRNA, complete cds.	AF078165	Hs.156527	CR933657
AK091357	6.5	5.1	cDNA FLJ34038 fis, clone FCBBF2005645.	AK091357	Hs.587465	AK091357
SERTAD4	6.5	6.1	SERTA domain containing 4 (SERTAD4), mRNA	NM_019605	Hs.699400	NM_019605
ATOH8	6.5	3.1	atonal homolog 8 (Drosophila) (ATOH8), mRNA	NM_032827	Hs.135569	CR749611
AK096415	6.5	1.9	cDNA FLJ39096 fis, clone NTONG1000247.	AK096415	Hs.13268	AK096415
FLJ30851	6.5	5.2	hypothetical LOC653140 (FLJ30851), mRNA	NM_001040710	Hs.467868	AK092423

THC2605327	6.5	74.0	AB013803 hNB-2s {Homo sapiens} (exp=-1; wgp=0; cg=0), partial (3%)	THC2605327	Unknown	
TMEM106B	6.5	2.0	transmembrane protein 106B (TMEM106B), mRNA	NM_018374	Hs.396358	NM_018374
AL050285	6.5	3.1	mRNA; cDNA DKFZp586N0819 (from clone DKFZp586N0819).	AL050285	Unknown	
SMYD2	6.5	5.0	SET and MYND domain containing 2 (SMYD2), mRNA	NM_020197	Hs.66170	BC049367
TBC1D8	6.5	2.5	cDNA FLJ40805 fis, clone TRACH2009060.	AK098124	Unknown	
NAPE-PLD	6.5	1.8	N-acyl-phosphatidylethanolamine-hydrolyzing phospholipase D	ENST00000341533	Unknown	
BAI3	6.5	11.5	brain-specific angiogenesis inhibitor 3 (BAI3), mRNA	NM_001704	Hs.13261	AB011122
HS2ST1	6.5	3.4	heparan sulfate 2-O-sulfotransferase 1 (HS2ST1), mRNA	NM_012262	Hs.48823	NM_012262
PLA2G4A	6.5	3.6	phospholipase A2, group IVA (cytosolic, calcium-dependent) (PLA2G4A), mRNA	NM_024420	Hs.497200	NM_024420
KBTBD10	6.5	44.7	kelch repeat and BTB (POZ) domain containing 10 (KBTBD10), mRNA	NM_006063	Hs.50550	NM_006063
THC2538822	6.5	1.7	THC2538822	THC2538822	Unknown	
COL1A2	6.5	5.0	collagen, type I, alpha 2 (COL1A2), mRNA	NM_000089	Hs.489142	J03464
NHLRC2	6.5	1.9	mRNA; cDNA DKFZp451L096 (from clone DKFZp451L096).	BX647641	Hs.369924	BX647641
LOC730663	6.5	1.7	PREDICTED: similar to 60S ribosomal protein L23a (LOC730663), mRNA	XM_001128309	Unknown	
NTF3	6.5	4.1	neurotrophin 3 (NTF3), mRNA	NM_002527	Hs.99171	NM_002527
HEL308	6.5	4.0	DNA helicase HEL308 (HEL308), mRNA	NM_133636	Hs.480101	NM_133636
AF086154	6.5	4.0	full length insert cDNA clone ZB55F04.	AF086154	Hs.142505	AF086154
CXCL12	6.5	3.6	chemokine (C-X-C motif) ligand 12 (stromal cell-derived factor 1) (CXCL12), transcript variant 1, mRNA	NM_199168	Hs.522891	AK090482
LRRC37A3	6.5	3.7	leucine rich repeat containing 37, member A3 (LRRC37A3), mRNA	NM_199340	Hs.551962	NM_199340
SLC40A1	6.5	10.0	solute carrier family 40 (iron-regulated transporter), member 1 (SLC40A1), mRNA	NM_014585	Hs.643005	BC037733
AK056624	6.5	3.2	cDNA FLJ32062 fis, clone OCBBF1000042.	AK056624	Hs.647082	AF459743
SYNE1	6.5	4.5	spectrin repeat containing, nuclear envelope 1 (SYNE1), transcript variant longest, mRNA	NM_182961	Hs.12967	NM_182961
BC039246	6.5	2.1	cDNA clone IMAGE:5278517.	BC039246	Hs.699210	BC039246
ENST00000380344	6.5	2.2	Beta-1,3-glucosyltransferase (EC 2.4.1.-) (Beta3Glc-T) (Beta-3-glycosyltransferase-like).	ENST00000380344	Unknown	
DKFZp434K191	6.5	4.5	hypothetical protein DKFZp434K191 (DKFZp434K191), mRNA	NM_001029950	Unknown	
FOXJ1	6.5	2.8	forkhead box J1 (FOXJ1), mRNA	NM_001454	Hs.651204	BC046460
RSHL3	6.5	4.1	radial spokehead-like 3 (RSHL3), mRNA	NM_001010892	Hs.160380	NM_001010892
PBK	6.5	3.0	PDZ binding kinase (PBK), mRNA	NM_018492	Hs.104741	NM_018492
TGFB3	6.5	18.4	transforming growth factor, beta 3 (TGFB3), mRNA	NM_003239	Hs.592317	AK122902
PIK3R1	6.5	2.4	phosphoinositide-3-kinase, regulatory subunit 1 (p85 alpha) (PIK3R1), transcript variant 1, mRNA	NM_181523	Hs.132225	NM_181523
KLF10	6.5	2.9	Kruppel-like factor 10 (KLF10), transcript variant 1, mRNA	NM_005655	Hs.435001	S81439
NEFM	6.5	9.4	neurofilament, medium polypeptide 150kDa (NEFM), mRNA	NM_005382	Hs.458657	BC096757
ITIH5	6.5	20.2	inter-alpha trypsin inhibitor heavy chain precursor 5 isoform 1	ENST00000256861	Unknown	
NOTCH2	6.5	3.4	Notch homolog 2 (Drosophila) (NOTCH2), mRNA	NM_024408	Hs.487360	NM_024408
KIAA0247	6.5	2.4	KIAA0247 (KIAA0247), mRNA	NM_014734	Hs.440025	BC064697
C1orf125	6.5	4.0	chromosome 1 open reading frame 125 (C1orf125), transcript variant 1, mRNA	NM_144696	Hs.658505	BX647935
GAB1	6.4	2.4	GRB2-associated binding protein 1 (GAB1), transcript variant 1, mRNA	NM_207123	Hs.80720	BC064848
THC2681437	6.4	4.2	THC2681437	THC2681437	Unknown	
FLJ20920	6.4	2.7	hypothetical protein FLJ20920 (FLJ20920), mRNA	NM_025149	Unknown	
LOC643641	6.4	5.4	mRNA for KIAA0543 protein, partial cds.	AB011115	Unknown	
AK001998	6.4	2.6	cDNA FLJ11136 fis, clone PLACE1006414.	AK001998	Unknown	
CXCL12	6.4	3.5	chemokine (C-X-C motif) ligand 12 (stromal cell-derived factor 1) (CXCL12), transcript variant 1, mRNA	NM_199168	Hs.522891	AK090482
THC2672325	6.4	2.1	THC2672325	THC2672325	Unknown	
C6orf142	6.4	16.0	chromosome 6 open reading frame 142 (C6orf142), mRNA	NM_138569	Hs.591803	BX647567
KCNMA1	6.4	2.9	potassium large conductance calcium-activated channel, subfamily M, alpha member 1 (KCNMA1), transcript variant 1, mRNA	NM_001014797	Hs.144795	NM_001014797
ABT1	6.4	2.3	activator of basal transcription 1 (ABT1), mRNA	NM_013375	Hs.254406	NM_013375
LOC401152	6.4	3.3	HCV F-transactivated protein 1 (LOC401152), mRNA	NM_001001701	Hs.173705	NM_001001701
SMTNL2	6.4	9.7	smoothelin-like 2 (SMTNL2), mRNA	NM_198501	Hs.441709	BC110859
SNTA1	6.4	2.5	syntrophin, alpha 1 (dystrophin-associated protein A1, 59kDa, acidic component) (SNTA1), mRNA	NM_003098	Hs.31121	NM_003098
EVC	6.4	2.4	tissue-type heart Ellis-van Creveld syndrome protein (EVC) mRNA, complete cds.	AF216184	Unknown	
FRMD6	6.4	2.5	FERM domain containing 6 (FRMD6), transcript variant 1, mRNA	NM_001042481	Hs.434914	BX648295

THC2671679	6.4	2.1	THC2671679	THC2671679	Unknown	
C14orf179	6.4	2.3	chromosome 14 open reading frame 179 (C14orf179), mRNA	NM_052873	Hs.532626	AK056735
COL1A2	6.4	5.1	collagen, type I, alpha 2 (COL1A2), mRNA	NM_000089	Hs.489142	J03464
DKFZP564O0823	6.4	4.3	DKFZP564O0823 protein (DKFZP564O0823), mRNA	NM_015393	Hs.105460	NM_015393
RANBP6	6.4	2.2	RAN binding protein 6 (RANBP6), mRNA	NM_012416	Hs.167496	BX537405
PAM	6.4	2.8	peptidylglycine alpha-amidating monooxygenase (PAM), transcript variant 1, mRNA	NM_000919	Hs.369430	NM_000919
ABLIM1	6.4	2.0	actin binding LIM protein 1 (ABLIM1), transcript variant 3, mRNA	NM_001003408	Hs.438236	NM_001003408
AK096984	6.4	2.8	cDNA FLJ39665 fis, clone SMINT2007294.	AK096984	Hs.373571	AK096984
ZNF403	6.4	2.2	zinc finger protein 403 (ZNF403), mRNA	NM_024835	Hs.514116	AF268387
OSBPL2	6.4	1.9	oxysterol binding protein-like 2 (OSBPL2), transcript variant 2, mRNA	NM_144498	Hs.473254	NM_144498
HSPA12A	6.4	3.3	heat shock 70kDa protein 12A (HSPA12A), mRNA	NM_025015	Hs.654682	BC041412
AK000872	6.4	16.7	cDNA FLJ10010 fis, clone HEMBA1000302.	AK000872	Hs.58690	AK128633
CPB2	6.4	34.4	carboxypeptidase B2 (plasma, carboxypeptidase U) (CPB2), transcript variant 1, mRNA	NM_001872	Hs.512937	NM_001872
MRPL43	6.4	5.8	mitochondrial ribosomal protein L43 (MRPL43), nuclear gene encoding mitochondrial protein, transcript variant 4, mRNA	NM_176794	Hs.421848	NM_176792
UNC5B	6.4	3.9	unc-5 homolog B (C. elegans) (UNC5B), mRNA	NM_170744	Hs.585457	AB096256
TTC30A	6.4	2.3	tetratricopeptide repeat domain 30A (TTC30A), mRNA	NM_152275	Hs.128384	NM_152275
A_32_P145159	6.4	2.3	A_32_P145159	A_32_P145159	Unknown	
ENST00000389155	6.4	9.1	similar to RIKEN cDNA 1700011J18 (LOC286187), mRNA	ENST00000389155	Unknown	
MTHFR	6.4	3.1	5,10-methylenetetrahydrofolate reductase (NADPH) (MTHFR), mRNA	NM_005957	Hs.214142	NM_005957
DNM3	6.4	4.1	dynamin 3 (DNM3), mRNA	NM_015569	Hs.654775	AL136712
ODZ4	6.4	3.7	mRNA for KIAA1302 protein, partial cds.	AB037723	Hs.213087	NM_001098816
WDR52	6.4	11.8	cDNA FLJ11142 fis, clone PLACE1006552.	AK002004	Hs.584936	CR936646
USP6NL	6.4	1.8	USP6 N-terminal-like protein (Related to the N-terminus of tre) (RN- tre).	ENST00000277575	Unknown	
SPATA17	6.4	6.2	spermatogenesis associated 17 (SPATA17), mRNA	NM_138796	Hs.171130	CR597267
CR607569	6.4	1.7	full-length cDNA clone CS0DF027YA11 of Fetal brain of (human).	CR607569	Hs.516075	AB209211
CDH19	6.4	11.4	cadherin 19, type 2 (CDH19), mRNA	NM_021153	Hs.42771	NM_021153
AK124426	6.4	2.4	cDNA FLJ42435 fis, clone BLADE2006849.	AK124426	Hs.356481	AB209869
ENPP3	6.4	4.2	ectonucleotide pyrophosphatase/phosphodiesterase 3 (ENPP3), mRNA	NM_005021	Hs.486489	NM_005021
THC2643088	6.4	2.0	Q8CD65_MOUSE (Q8CD65) 13 days embryo male testis cDNA, RIKEN full-length enriched library, clone:6030413H17 product:potassium voltage-gated channel, shaker-related subfamily, beta member 1, full insert sequence, partial (12%)	THC2643088	Unknown	
KITLG	6.4	3.3	KIT ligand (KITLG), transcript variant b, mRNA	NM_000899	Hs.1048	NM_000899
SORCS2	6.4	5.9	sortilin-related VPS10 domain containing receptor 2 (SORCS2), mRNA	NM_020777	Hs.479099	NM_020777
CCDC48	6.4	7.0	coiled-coil domain containing 48 (CCDC48), mRNA	NM_024768	Hs.134807	BX649124
RAB14	6.4	2.2	RAB14, member RAS oncogene family (RAB14), mRNA	NM_016322	Hs.371563	AL162081
TP53INP1	6.4	5.4	tumor protein p53 inducible nuclear protein 1 (TP53INP1), mRNA	NM_033285	Hs.700624	AK125880
AF289610	6.4	3.2	clone pp9372 unknown mRNA.	AF289610	Hs.675915	AF289610
CD164	6.4	2.8	CD164 molecule, sialomucin (CD164), mRNA	NM_006016	Hs.520313	BC040317
RCOR3	6.4	4.4	REST corepressor 3 (RCOR3), mRNA	NM_018254	Hs.696152	AK125810
COL1A2	6.4	5.0	collagen, type I, alpha 2 (COL1A2), mRNA	NM_000089	Hs.489142	J03464
COL1A2	6.4	5.1	collagen, type I, alpha 2 (COL1A2), mRNA	NM_000089	Hs.489142	J03464
CYFIP2	6.4	1.7	cytoplasmic FMR1 interacting protein 2 (CYFIP2), transcript variant 2, mRNA	NM_001037332	Hs.519702	NM_001037332
LOC648294	6.4	1.6	PREDICTED: hypothetical LOC648294 (LOC648294), mRNA	XR_018553	Hs.693351	XR_018553
ZNF664	6.4	2.2	zinc finger protein 664 (ZNF664), mRNA	NM_152437	Hs.524828	AL834266
BC037919	6.4	5.1	cDNA clone IMAGE:5278089.	BC037919	Hs.113418	BC037919
COL1A2	6.4	5.0	collagen, type I, alpha 2 (COL1A2), mRNA	NM_000089	Hs.489142	J03464
CA437634	6.4	2.4	UI-H-DH0-aup-d-15-0-UI.s1 NCI_CGAP_DH0 cDNA clone UI-H-DH0-aup-d-15-0-UI 3', mRNA sequence	CA437634	Hs.638027	CA437634
C12orf57	6.4	2.3	chromosome 12 open reading frame 57 (C12orf57), mRNA	NM_138425	Hs.591045	BM548362
THC2634957	6.4	5.4	Q3S279_9STRA (Q3S279) NADH dehydrogenase subunit 9, partial (7%)	THC2634957	Unknown	
GREM2	6.4	3.9	gremlin 2, cysteine knot superfamily, homolog (Xenopus laevis) (GREM2), mRNA	NM_022469	Hs.98206	NM_022469
A_32_P108592	6.4	2.3	A_32_P108592	A_32_P108592	Unknown	
PCSK2	6.4	6.4	proprotein convertase subtilisin/kexin type 2 (PCSK2), mRNA	NM_002594	Hs.315186	NM_002594
RAB36	6.4	2.8	RAB36, member RAS oncogene family (RAB36), mRNA	NM_004914	Hs.369557	AB023061

EPS8	6.4	4.2	epidermal growth factor receptor pathway substrate 8 (EPS8), mRNA	NM_004447	Hs.591160	NM_004447
ITIH5	6.3	6.1	inter-alpha (globulin) inhibitor H5 (ITIH5), transcript variant 1, mRNA	NM_030569	Hs.498586	CR627109
THC2662587	6.3	4.2	THC2662587	THC2662587	Unknown	
CTBS	6.3	2.5	chitobiase, di-N-acetyl- (CTBS), mRNA	NM_004388	Hs.513557	BC096752
KIAA1683	6.3	5.0	KIAA1683 (KIAA1683), mRNA	NM_025249	Hs.313471	AB051470
ELAC1	6.3	2.8	elaC homolog 1 (E. coli) (ELAC1), mRNA	NM_018696	Hs.657360	NM_018696
THC2639653	6.3	2.6	Q2BEK2_9BACI (Q2BEK2) RNA polymerase sigma-70 factor , partial (7%)	THC2639653	Unknown	
FLJ21839	6.3	2.2	hypothetical protein FLJ21839 (FLJ21839), transcript variant 1, mRNA	NM_021831	Hs.138207	NM_021831
AYTL1	6.3	5.4	mRNA; cDNA DKFZp686H22112 (from clone DKFZp686H22112).	BX641069	Hs.460857	BX641069
RB1	6.3	3.2	retinoblastoma 1 (including osteosarcoma) (RB1), mRNA	NM_000321	Hs.408528	L41870
COMMD8	6.3	2.6	COMM domain containing 8 (COMMD8), mRNA	NM_017845	Hs.23956	BC015145
AK128756	6.3	2.6	cDNA FLJ44869 fis, clone BRAMY2015516.	AK128756	Hs.632969	AK128756
DKFZp313A2432	6.3	3.9	mRNA; cDNA DKFZp313A2432 (from clone DKFZp313A2432).	AL833119	Hs.349096	XM_934482
CNO	6.3	2.0	cappuccino homolog (mouse) (CNO), mRNA	NM_018366	Hs.7570	CR626399
BM544548	6.3	7.0	AGENCOURT_6491969 NIH_MGC_124 cDNA clone IMAGE:5728337 5', mRNA sequence	BM544548	Hs.596112	BM544548
H2AFY	6.3	2.3	H2A histone family, member Y (H2AFY), transcript variant 3, mRNA	NM_138610	Hs.696013	AB209490
AK024516	6.3	2.5	cDNA: FLJ20863 fis, clone ADKA01804.	AK024516	Hs.657178	AK024516
EPC1	6.3	2.5	enhancer of polycomb homolog 1 (Drosophila), mRNA (cDNA clone MGC:33656 IMAGE:4828057), complete cds.	BC036529	Hs.167805	BC036529
TBC1D9	6.3	6.5	TBC1 domain family, member 9 (with GRAM domain) (TBC1D9), mRNA	NM_015130	Hs.480819	NM_015130
AK023040	6.3	3.6	cDNA FLJ12978 fis, clone NT2RP2006321.	AK023040	Hs.696175	BC065573
AK094603	6.3	9.6	cDNA FLJ37284 fis, clone BRAMY2013590.	AK094603	Unknown	
LEF1	6.3	7.5	lymphoid enhancer-binding factor 1 (LEF1), mRNA	NM_016269	Hs.555947	AK128255
ZC3H7A	6.3	1.6	zinc finger CCCH-type containing 7A (ZC3H7A), mRNA	NM_014153	Hs.371856	NM_014153
DPYD	6.3	12.1	dihydropyrimidine dehydrogenase (DPYD), mRNA	NM_000110	Hs.335034	NM_000110
AK026225	6.3	7.4	cDNA: FLJ22572 fis, clone HSI02313.	AK026225	Hs.382865	AB209907
HMCN1	6.3	4.6	hemicentin 1 (HMCN1), mRNA	NM_031935	Hs.58877	NM_031935
FAM82A	6.3	2.9	family with sequence similarity 82, member A (FAM82A), mRNA	NM_144713	Hs.591566	AK095462
LARP7	6.3	2.1	La ribonucleoprotein domain family, member 7 (LARP7), transcript variant 1, mRNA	NM_016648	Hs.696461	BC066945
C19orf18	6.3	4.1	chromosome 19 open reading frame 18 (C19orf18), mRNA	NM_152474	Hs.134209	BC033933
GALC	6.3	2.7	galactosylceramidase (GALC), transcript variant 1, mRNA	NM_000153	Hs.513439	NM_000153
AL133118	6.3	29.8	mRNA; cDNA DKFZp586N0121 (from clone DKFZp586N0121).	AL133118	Hs.596517	AL133118
ZNF235	6.3	2.2	zinc finger protein 235 (ZNF235), mRNA	NM_004234	Hs.298089	AK226126
GSTM1	6.3	4.4	glutathione S-transferase M1 (GSTM1), transcript variant 2, mRNA	NM_146421	Hs.301961	BQ880398
C10orf56	6.3	2.9	chromosome 10 open reading frame 56 (C10orf56), mRNA	NM_153367	Hs.523080	BC028617
SPAG6	6.3	9.5	sperm associated antigen 6 (SPAG6), transcript variant 1, mRNA	NM_012443	Hs.655170	NM_012443
RB1	6.3	3.3	retinoblastoma 1 (including osteosarcoma) (RB1), mRNA	NM_000321	Hs.408528	L41870
ENST00000355898	6.3	2.3	Zinc finger protein 507.	ENST00000355898	Unknown	
APOC3	6.3	5.4	apolipoprotein C-III (APOC3), mRNA	NM_000040	Hs.73849	BI521580
PLK2	6.3	4.0	polo-like kinase 2 (Drosophila) (PLK2), mRNA	NM_006622	Hs.398157	AF059617
AK129547	6.3	3.2	cDNA FLJ26036 fis, clone PRS00145.	AK129547	Hs.640055	AK129547
TMEM17	6.3	2.0	transmembrane protein 17 (TMEM17), mRNA	NM_198276	Hs.308028	AK091902
C13orf24	6.3	2.2	chromosome 13 open reading frame 24 (C13orf24), mRNA	NM_006346	Hs.441926	AF330046
FAM14A	6.3	2.5	family with sequence similarity 14, member A (FAM14A), mRNA	NM_032036	Hs.94695	BU596980
ZNF641	6.3	3.0	zinc finger protein 641 (ZNF641), mRNA	NM_152320	Hs.23492	AY842285
ZCCHC14	6.3	1.7	zinc finger, CCHC domain containing 14 (ZCCHC14), mRNA	NM_015144	Hs.156231	NM_015144
RB1	6.3	3.2	retinoblastoma 1 (including osteosarcoma) (RB1), mRNA	NM_000321	Hs.408528	L41870
ARL6IP5	6.3	2.4	ADP-ribosylation-like factor 6 interacting protein 5 (ARL6IP5), mRNA	NM_006407	Hs.518060	NM_006407
WDR37	6.3	1.8	WD repeat domain 37 (WDR37), mRNA	NM_014023	Hs.188495	NM_014023
SERPINF1	6.3	2.6	serpin peptidase inhibitor, clade F (alpha-2 antiplasmin, pigment epithelium derived factor), member 1 (SERPINF1), mRNA	NM_002615	Hs.694727	BM918904
THC2738152	6.3	5.8	ALU1_HUMAN (P39188) Alu subfamily J sequence contamination warning entry, partial (5%)	THC2738152	Unknown	
C20orf132	6.3	3.6	chromosome 20 open reading frame 132 (C20orf132), transcript variant 2, mRNA	NM_213631	Hs.349125	BC030006
COL1A2	6.3	5.0	collagen, type I, alpha 2 (COL1A2), mRNA	NM_000089	Hs.489142	J03464
IGFBP5	6.3	5.0	insulin-like growth factor binding protein 5 (IGFBP5), mRNA	NM_000599	Hs.635441	NM_000599

IFT172	6.3	2.1	intraflagellar transport 172 homolog (Chlamydomonas) (IFT172), mRNA	NM_015662	Hs.127401	NM_015662
SEC23A	6.3	2.4	Sec23 homolog A (S. cerevisiae) (SEC23A), mRNA	NM_006364	Hs.272927	BC036649
BX114329	6.3	10.1	BX114329 BX114329 Soares_NhHMPu_S1 cDNA clone IMAGp998G064741 ; IMAGE:1932317, mRNA sequence	BX114329	Unknown	
PLA2G4A	6.3	3.7	phospholipase A2, group IVA (cytosolic, calcium-dependent) (PLA2G4A), mRNA	NM_024420	Hs.497200	NM_024420
RB1	6.3	3.2	retinoblastoma 1 (including osteosarcoma) (RB1), mRNA	NM_000321	Hs.408528	L41870
KIAA1840	6.3	1.6	KIAA1840 (KIAA1840), mRNA	NM_025137	Hs.656271	NM_025137
ISOC1	6.3	2.1	isochorismatase domain containing 1 (ISOC1), mRNA	NM_016048	Hs.483296	BC008367
KCNJ2	6.3	6.4	potassium inwardly-rectifying channel, subfamily J, member 2 (KCNJ2), mRNA	NM_000891	Hs.1547	NM_000891
LOC651130	6.3	3.4	PREDICTED: similar to RIKEN cDNA 0610013E23 (LOC651130), mRNA	XR_019042	Hs.647423	XR_019042
AK124396	6.3	42.2	cDNA FLJ42405 fis, clone ASTRO3000474.	AK124396	Hs.49265	AK124396
TMEM50B	6.3	2.9	transmembrane protein 50B (TMEM50B), mRNA	NM_006134	Hs.433668	NM_006134
SHC4	6.2	9.8	SHC (Src homology 2 domain containing) family, member 4 (SHC4), mRNA	NM_203349	Hs.642615	NM_203349
C3orf57	6.2	3.7	chromosome 3 open reading frame 57 (C3orf57), mRNA	NM_001040100	Hs.369104	AK123348
CR590573	6.2	3.2	full-length cDNA clone CS0DI042YD07 of Placenta Cot 25-normalized of (human).	CR590573	Hs.484967	CR590573
ARL8B	6.2	1.8	ADP-ribosylation factor-like 8B (ARL8B), mRNA	NM_018184	Hs.250009	BC063125
MAPK10	6.2	2.4	mitogen-activated protein kinase 10 (MAPK10), transcript variant 3, mRNA	NM_138980	Hs.125503	AK124791
GLT8D3	6.2	2.3	glycosyltransferase 8 domain containing 3, mRNA (cDNA clone MGC:33359 IMAGE:5266607), complete cds.	BC030023	Hs.259347	NM_173601
LOC497661	6.2	2.1	putative NFkB activating protein (LOC497661), mRNA	NM_001035005	Hs.654638	BC093004
RSPO2	6.2	6.6	R-spondin 2 homolog (Xenopus laevis) (RSPO2), mRNA	NM_178565	Hs.444834	AK123023
TNNT2	6.2	26.1	troponin T type 2 (cardiac) (TNNT2), transcript variant 1, mRNA	NM_000364	Hs.533613	AL832707
COL1A2	6.2	5.0	collagen, type I, alpha 2 (COL1A2), mRNA	NM_000089	Hs.489142	J03464
PALM2	6.2	6.5	paralemmin 2 (PALM2), transcript variant 1, mRNA	NM_053016	Hs.591908	NM_053016
BBS2	6.2	2.1	Bardet-Biedl syndrome 2 (BBS2), mRNA	NM_031885	Hs.333738	AB208905
ITIH2	6.2	29.7	inter-alpha (globulin) inhibitor H2 (ITIH2), mRNA	NM_002216	Hs.75285	NM_002216
MFAP4	6.2	4.8	microfibrillar-associated protein 4 (MFAP4), mRNA	NM_002404	Hs.296049	BC035560
ALB	6.2	191.9	albumin (ALB), mRNA	NM_000477	Hs.418167	AF130077
FABP1	6.2	10.6	fatty acid binding protein 1, liver (FABP1), mRNA	NM_001443	Hs.380135	BX641099
LOC344595	6.2	2.3	hypothetical LOC344595, mRNA (cDNA clone IMAGE:5760770).	BC039550	Hs.677855	BC039550
PHF2	6.2	2.6	PHD finger protein 2 (PHF2), mRNA	NM_005392	Hs.211441	NM_005392
POLR3GL	6.2	3.4	polymerase (RNA) III (DNA directed) polypeptide G (32kD)-like (POLR3GL), mRNA	NM_032305	Hs.591456	BC050418
AF277175	6.2	2.5	PNAS-138 mRNA, complete cds.	AF277175	Hs.520710	D87454
ZNF302	6.2	2.6	zinc finger protein 302 (ZNF302), transcript variant 1, mRNA	NM_018443	Hs.436350	AK122855
CETN2	6.2	2.1	centrin, EF-hand protein, 2 (CETN2), mRNA	NM_004344	Hs.82794	BG567463
CASD1	6.2	1.7	CAS1 domain containing 1 (CASD1), mRNA	NM_022900	Hs.260041	BC063284
RB1	6.2	3.2	retinoblastoma 1 (including osteosarcoma) (RB1), mRNA	NM_000321	Hs.408528	L41870
TTC28	6.2	2.9	mRNA for KIAA1043 protein, partial cds.	AB028966	Hs.387856	XM_929318
SOCS5	6.2	2.0	suppressor of cytokine signaling 5 (SOCS5), transcript variant 2, mRNA	NM_144949	Hs.468426	NM_144949
THEM4	6.2	3.3	thioesterase superfamily member 4 (THEM4), mRNA	NM_053055	Hs.164070	AK096211
FLJ36166	6.2	4.5	cDNA clone IMAGE:5271477, containing frame-shift errors.	BC094802	Hs.148768	BC094802
DUSP12	6.2	2.3	dual specificity phosphatase 12 (DUSP12), mRNA	NM_007240	Hs.416216	BC006286
RPL6	6.2	1.7	ribosomal protein L6 (RPL6), transcript variant 1, mRNA	NM_001024662	Hs.546283	BQ055135
BU608568	6.2	2.0	UI-CF-FN0-aep-h-11-0-UI.s1 UI-CF-FN0 cDNA clone UI-CF-FN0-aep-h-11-0-UI 3', mRNA sequence	BU608568	Hs.621731	BU608568
TSPAN2	6.2	3.0	tetraspanin 2 (TSPAN2), mRNA	NM_005725	Hs.310458	NM_005725
F2RL2	6.2	11.5	coagulation factor II (thrombin) receptor-like 2 (F2RL2), mRNA	NM_004101	Hs.42502	NM_004101
COL1A2	6.2	5.0	collagen, type I, alpha 2 (COL1A2), mRNA	NM_000089	Hs.489142	J03464
BX109843	6.2	1.9	BX109843 Soares placenta Nb2HP cDNA clone IMAGp998B14208, mRNA sequence	BX109843	Hs.78050	BX109843
NRP1	6.2	13.2	neuropilin 1 (NRP1), transcript variant 1, mRNA	NM_003873	Hs.131704	CR749333
LOC150759	6.2	3.6	cDNA FLJ33034 fis, clone THYMU2000236.	AK057596	Hs.700794	AK057596
A_32_P171043	6.2	10.3	A_32_P171043	A_32_P171043	Unknown	
ZBED5	6.2	1.5	zinc finger, BED-type containing 5 (ZBED5), mRNA	NM_021211	Unknown	
CNOT6	6.2	2.0	CCR4-NOT transcription complex, subunit 6 (CNOT6), mRNA	NM_015455	Hs.654984	AB033020
PDZRN3	6.2	5.1	PDZ domain containing RING finger 3 (PDZRN3), mRNA	NM_015009	Hs.434900	AB029018

GSTA4	6.2	3.0	glutathione S-transferase A4 (GSTA4), mRNA	NM_001512	Hs.485557	BC071631
RGS12	6.2	2.8	regulator of G-protein signalling 12 (RGS12), transcript variant 1, mRNA	NM_198229	Hs.527061	NM_002926
RB1	6.2	3.2	retinoblastoma 1 (including osteosarcoma) (RB1), mRNA	NM_000321	Hs.408528	L41870
C20orf133	6.2	4.3	chromosome 20 open reading frame 133 (C20orf133), transcript variant 1, mRNA	NM_080676	Hs.661576	NM_080676
LOC388965	6.2	1.8	similar to hepatitis C virus core-binding protein 6; cervical cancer oncogene 3 (LOC388965), mRNA	NM_001013648	Unknown	
TRPC3	6.2	4.1	transient receptor potential cation channel, subfamily C, member 3 (TRPC3), mRNA	NM_003305	Hs.150981	AY865574
C7orf31	6.2	2.1	chromosome 7 open reading frame 31 (C7orf31), mRNA	NM_138811	Hs.122055	NM_138811
FRZB	6.2	7.2	frizzled-related protein (FRZB), mRNA	NM_001463	Hs.128453	NM_001463
KIAA1731	6.2	2.0	mRNA for KIAA1731 protein, partial cds.	AB051518	Hs.458418	AB051518
AW961597	6.2	3.5	EST373669 MAGE resequences, MAGG cDNA, mRNA sequence	AW961597	Hs.657591	BI497430
DACT1	6.2	4.1	dapper, antagonist of beta-catenin, homolog 1 (Xenopus laevis) (DACT1), transcript variant 1, mRNA	NM_016651	Hs.48950	NM_016651
LZTFL1	6.2	2.0	leucine zipper transcription factor-like 1 (LZTFL1), mRNA	NM_020347	Hs.30824	BC042483
MEF2C	6.2	28.4	MADS box transcription enhancer factor 2, polypeptide C (myocyte enhancer factor 2C) (MEF2C), mRNA	NM_002397	Hs.699175	AL833268
PLA2G4A	6.2	3.9	phospholipase A2, group IVA (cytosolic, calcium-dependent) (PLA2G4A), mRNA	NM_024420	Hs.497200	NM_024420
ENST00000340576	6.2	13.7	cDNA FLJ13189 fis, clone NT2RP3004253.	ENST00000340576	Unknown	
PLEKHA6	6.2	9.4	pleckstrin homology domain containing, family A member 6 (PLEKHA6), mRNA	NM_014935	Hs.253146	NM_014935
HNRPR	6.2	1.8	heterogeneous nuclear ribonucleoprotein R (HNRPR), mRNA	NM_005826	Unknown	
AK023159	6.2	2.7	cDNA FLJ13097 fis, clone NT2RP3002173.	AK023159	Hs.23648	AK055297
BC031342	6.2	22.7	Homo sapiens, clone IMAGE:5019307, mRNA.	BC031342	Hs.445239	AK022839
HBD	6.2	20.7	hemoglobin, delta (HBD), mRNA	NM_000519	Hs.699280	AY034468
KLHL24	6.2	3.1	kelch-like 24 (Drosophila) (KLHL24), mRNA	NM_017644	Hs.407709	NM_017644
BMPR2	6.1	7.4	bone morphogenetic protein receptor, type II (serine/threonine kinase) (BMPR2), mRNA	NM_001204	Hs.471119	NM_001204
RB1	6.1	3.2	retinoblastoma 1 (including osteosarcoma) (RB1), mRNA	NM_000321	Hs.408528	L41870
LOC283666	6.1	2.9	hypothetical protein LOC283666, mRNA (cDNA clone IMAGE:4415549), partial cds.	BC048264	Hs.560343	AL832164
A_24_P303080	6.1	2.1	A_24_P303080	A_24_P303080	Unknown	
FNDC5	6.1	8.9	fibronectin type III domain containing 5 (FNDC5), mRNA	NM_153756	Hs.524234	AK092102
LOC728473	6.1	6.7	cDNA FLJ41084 fis, clone ADRGL2010974.	AK123079	Hs.658343	AK123079
THC2527306	6.1	2.9	Q9HBV3_HUMAN (Q9HBV3) PP3111, partial (17%)	THC2527306	Unknown	
DGKB	6.1	5.6	diacylglycerol kinase, beta 90kDa (DGKB), transcript variant 1, mRNA	NM_004080	Hs.567255	NM_004080
RB1	6.1	3.2	retinoblastoma 1 (including osteosarcoma) (RB1), mRNA	NM_000321	Hs.408528	L41870
OR7D2	6.1	3.9	olfactory receptor, family 7, subfamily D, member 2 (OR7D2), mRNA	NM_175883	Hs.531755	NM_175883
THC2669419	6.1	2.4	ALU7_HUMAN (P39194) Alu subfamily SQ sequence contamination warning entry, partial (13%)	THC2669419	Unknown	
LOC144233	6.1	2.5	hypothetical protein LOC144233 (LOC144233), mRNA	NM_181708	Hs.142736	BC040957
C9orf127	6.1	2.2	chromosome 9 open reading frame 127 (C9orf127), transcript variant 3, mRNA	NM_016446	Hs.493808	NM_001042590
A_24_P650011	6.1	2.7	A_24_P650011	A_24_P650011	Unknown	
AHI1	6.1	3.6	Abelson helper integration site 1 (AHI1), mRNA	NM_017651	Hs.386684	NM_017651
RASD1	6.1	5.6	RAS, dexamethasone-induced 1 (RASD1), mRNA	NM_016084	Hs.25829	BC042688
PAM	6.1	2.9	peptidylglycine alpha-amidating monooxygenase (PAM), transcript variant 1, mRNA	NM_000919	Hs.369430	NM_000919
POU4F1	6.1	8.8	POU domain, class 4, transcription factor 1 (POU4F1), mRNA	NM_006237	Hs.654522	L20433
NEDD4	6.1	2.2	neural precursor cell expressed, developmentally down-regulated 4 (NEDD4), transcript variant 2, mRNA	NM_198400	Hs.1565	AL832063
SPAG17	6.1	5.3	sperm associated antigen 17 (SPAG17), mRNA	NM_206996	Hs.528821	AY555274
GRK4	6.1	4.4	G protein-coupled receptor kinase 4 (GRK4), transcript variant 3, mRNA	NM_001004057	Hs.32959	BC027597
DMXL2	6.1	3.8	Dmx-like 2 (DMXL2), mRNA	NM_015263	Hs.511386	NM_015263
ARHGEF17	6.1	2.6	Rho guanine nucleotide exchange factor (GEF) 17 (ARHGEF17), mRNA	NM_014786	Hs.533719	NM_014786
TNNT2	6.1	27.8	troponin T type 2 (cardiac) (TNNT2), transcript variant 1, mRNA	NM_000364	Hs.533613	AL832707
A_32_P42780	6.1	2.9	A_32_P42780	A_32_P42780	Unknown	
MFSD1	6.1	2.8	major facilitator superfamily domain containing 1 (MFSD1), mRNA	NM_022736	Hs.58663	BC030542
BCDO2	6.1	14.5	beta-carotene dioxygenase 2 (BCDO2), transcript variant 1, mRNA	NM_031938	Hs.647227	NM_031938
FLJ13231	6.1	2.3	hypothetical protein FLJ13231 (FLJ13231), mRNA	NM_023073	Hs.643420	NM_023073
RUNX1T1	6.1	3.4	runt-related transcription factor 1; translocated to, 1 (cyclin D-related) (RUNX1T1), transcript variant 1, mRNA	NM_004349	Hs.368431	NM_004349
LASS6	6.1	1.9	LAG1 homolog, ceramide synthase 6 (S. cerevisiae) (LASS6), mRNA	NM_203463	Hs.506829	NM_203463

AK026750	6.1	2.9	cDNA: FLJ23097 fis, clone LNG07418.	AK026750	Hs.152432	AK026750
IGSF3	6.1	2.6	immunoglobulin superfamily, member 3 (IGSF3), transcript variant 1, mRNA	NM_001542	Hs.171057	NM_001542
IMMP2L	6.1	2.1	IMP2 inner mitochondrial membrane peptidase-like (S. cerevisiae) (IMMP2L), mRNA	NM_032549	Hs.655722	AF359563
FNDC1	6.1	3.3	fibronectin type III domain containing 1 (FNDC1), mRNA	NM_032532	Hs.520525	NM_032532
EIF2C4	6.1	2.7	Eukaryotic translation initiation factor 2C 4 (eIF2C 4) (eIF-2C 4) (Argonaute-4).	ENST00000373210	Unknown	
ZAK	6.1	2.6	sterile alpha motif and leucine zipper containing kinase AZK (ZAK), transcript variant 2, mRNA	NM_133646	Hs.444451	AF480462
SLC6A2	6.1	2.4	solute carrier family 6 (neurotransmitter transporter, noradrenalin), member 2 (SLC6A2), mRNA	NM_001043	Hs.78036	NM_001043
LOC285831	6.1	4.2	cDNA FLJ37752 fis, clone BRHIP2023309.	AK095071	Hs.654655	AK095071
WDR73	6.1	2.0	WD repeat domain 73 (WDR73), mRNA	NM_032856	Hs.165736	AK090406
BC030106	6.1	2.4	cDNA clone IMAGE:4797878.	BC030106	Hs.547831	BI914892
AK022168	6.1	4.0	cDNA FLJ12106 fis, clone HEMBB1002702.	AK022168	Hs.688481	AK022168
CR600305	6.1	2.4	full-length cDNA clone CS0DL001YI02 of B cells (Ramos cell line) Cot 25-normalized of (human).	CR600305	Hs.516182	AB037808
AK130118	6.1	7.6	cDNA FLJ26608 fis, clone LVR00914.	AK130118	Hs.654777	AK130118
SARM1	6.1	4.4	sterile alpha and TIR motif containing 1 (SARM1), mRNA	NM_015077	Hs.532781	NM_015077
FSD2	6.1	5.9	fibronectin type III and SPRY domain containing 2	ENST00000334574	Unknown	
EFNB1	6.1	2.7	ephrin-B1 (EFNB1), mRNA	NM_004429	Hs.144700	NM_004429
THC2651722	6.1	2.7	THC2651722	THC2651722	Unknown	
C6orf199	6.1	2.9	chromosome 6 open reading frame 199 (C6orf199), mRNA	NM_145025	Hs.486169	AL832162
ENST00000374465	6.1	2.8	amyotrophic lateral sclerosis 2 (juvenile) chromosome region, candidate 19, mRNA (cDNA clone IMAGE:3628944).	ENST00000374465	Unknown	
CDADC1	6.1	2.2	cytidine and dCMP deaminase domain containing 1 (CDADC1), mRNA	NM_030911	Hs.388220	BC048092
EMCN	6.1	13.7	endomucin (EMCN), mRNA	NM_016242	Hs.152913	NM_016242
DIRAS3	6.1	26.4	DIRAS family, GTP-binding RAS-like 3 (DIRAS3), mRNA	NM_004675	Hs.194695	AK096393
PDCD4	6.1	3.4	programmed cell death 4 (neoplastic transformation inhibitor) (PDCD4), transcript variant 2, mRNA	NM_145341	Hs.232543	BX537500
TSEN34	6.1	2.3	tRNA splicing endonuclease 34 homolog (S. cerevisiae) (TSEN34), transcript variant 2, mRNA	NM_001077446	Hs.15580	BM919151
AA778291	6.1	3.0	AA778291 zl56d07.s1 Soares_pregnant_uterus_NbHPU cDNA clone IMAGE:505933 3', mRNA sequence	AA778291	Hs.166017	NM_198159
CDR1	6.1	5.9	cerebellar degeneration-related protein 1, 34kDa (CDR1), mRNA	NM_004065	Hs.571748	AK054921
PTGIS	6.1	3.9	prostaglandin I2 (prostacyclin) synthase (PTGIS), mRNA	NM_000961	Hs.302085	NM_000961
CPNE5	6.1	9.5	copine V (CPNE5), mRNA	NM_020939	Hs.657869	AB046819
LOC341511	6.1	1.6	PREDICTED: similar to 60S ribosomal protein L23a (LOC341511), mRNA	ENST00000237840	Unknown	
PPARGC1A	6.1	6.0	peroxisome proliferator-activated receptor gamma, coactivator 1 alpha (PPARGC1A), mRNA	NM_013261	Hs.527078	NM_013261
CD302	6.1	3.1	CD302 molecule (CD302), mRNA	NM_014880	Hs.130014	AY314007
ENST00000378179	6.1	2.1	Peripheral plasma membrane protein CASK (EC 2.7.11.1) (hCASK) (Calcium/calmodulin-dependent serine protein kinase) (Lin-2 homolog).	ENST00000378179	Unknown	
GRID1	6.1	4.6	glutamate receptor, ionotropic, delta 1 (GRID1), mRNA	NM_017551	Hs.530653	NM_017551
SNRPE	6.1	1.7	small nuclear ribonucleoprotein polypeptide E (SNRPE), mRNA	NM_003094	Hs.334612	BC072433
MKL2	6.1	2.3	MKL/myocardin-like 2 (MKL2), mRNA	NM_014048	Hs.592047	NM_014048
CXCL12	6.1	3.6	chemokine (C-X-C motif) ligand 12 (stromal cell-derived factor 1) (CXCL12), transcript variant 1, mRNA	NM_199168	Hs.522891	AK090482
KIAA0232	6.1	1.9	KIAA0232 gene product (KIAA0232), mRNA	NM_014743	Hs.79276	NM_014743
COL1A2	6.1	4.9	collagen, type I, alpha 2 (COL1A2), mRNA	NM_000089	Hs.489142	J03464
THC2683231	6.1	2.6	Q77CB1_9ALPH (Q77CB1) UL6 protein, partial (3%)	THC2683231	Unknown	
BC030084	6.1	2.9	cDNA clone IMAGE:4791887.	BC030084	Hs.167087	BC048014
AK057956	6.1	2.9	cDNA FLJ25227 fis, clone STM01429.	AK057956	Hs.516023	AK095574
PHF2	6.1	2.1	PHD-finger protein (GRC5) mRNA, complete cds.	AF043725	Unknown	
AASDHPPT	6.1	2.4	aminoadipate-semialdehyde dehydrogenase-phosphopantetheinyl transferase (AASDHPPT), mRNA	NM_015423	Hs.524009	BX537665
FZD10	6.1	15.4	frizzled homolog 10 (Drosophila) (FZD10), mRNA	NM_007197	Hs.31664	BC070037
CXCL14	6.1	5.0	chemokine (C-X-C motif) ligand 14 (CXCL14), mRNA	NM_004887	Hs.483444	NM_004887
MYL3	6.1	6.0	myosin, light chain 3, alkali; ventricular, skeletal, slow (MYL3), mRNA	NM_000258	Hs.517939	BC009790
GREM1	6.1	5.7	gremlin 1, cysteine knot superfamily, homolog (Xenopus laevis) (GREM1), mRNA	NM_013372	Hs.40098	NM_013372
ZBTB20	6.1	5.1	zinc finger and BTB domain containing 20 (ZBTB20), mRNA	NM_015642	Hs.655108	BC090936
C14orf167	6.1	2.2	chromosome 14 open reading frame 167, mRNA (cDNA clone IMAGE:4706815), partial cds.	BC013143	Hs.601265	BC062348
TSPAN6	6.0	2.7	tetraspanin 6 (TSPAN6), mRNA	NM_003270	Hs.43233	AB209876

SOAT1	6.0	4.3	Sterol O-acyltransferase 1 (EC 2.3.1.26) (Cholesterol acyltransferase 1) (Acyl coenzyme A:cholesterol acyltransferase 1) (ACAT-1).	ENST00000367620	Unknown	
BC033590	6.0	2.4	Homo sapiens, clone IMAGE:4344826, mRNA.	BC033590	Hs.597585	BC033590
CLK4	6.0	2.5	CDC-like kinase 4 (CLK4), mRNA	NM_020666	Hs.406557	BC151233
FRG1	6.0	1.7	FSHD region gene 1 (FRG1), mRNA	NM_004477	Hs.203772	AK057099
RPL30	6.0	1.8	ribosomal protein L30 (RPL30), mRNA	NM_000989	Hs.400295	AK128768
ADAMTS1	6.0	2.4	ADAM metallopeptidase with thrombospondin type 1 motif, 1 (ADAMTS1), mRNA	NM_006988	Hs.643357	NM_006988
AKAP9	6.0	2.2	A kinase (PRKA) anchor protein (yotiao) 9 (AKAP9), transcript variant 1, mRNA	NM_147171	Hs.651221	NM_147171
THRA	6.0	3.6	thyroid hormone receptor, alpha (erythroblastic leukemia viral (v-erb-a) oncogene homolog, avian) (THRA), transcript variant 2, mRNA	NM_003250	Hs.724	AB209346
C2orf3	6.0	1.8	chromosome 2 open reading frame 3 (C2orf3), mRNA	NM_003203	Hs.303808	NM_003203
ADAMTS6	6.0	18.7	ADAM metallopeptidase with thrombospondin type 1 motif, 6 (ADAMTS6), mRNA	NM_197941	Hs.482291	NM_197941
DYNC2H1	6.0	1.8	dynein, cytoplasmic 2, heavy chain 1, mRNA (cDNA clone IMAGE:5265846), complete cds.	BC037496	Hs.503721	NM_001080463
TLE2	6.0	3.1	transducin-like enhancer of split 2 (E(sp1) homolog, Drosophila) (TLE2), mRNA	NM_003260	Hs.332173	NM_003260
MGC23284	6.0	3.9	FP17581 mRNA, complete cds.	AY203928	Hs.499548	BX640980
THC2725128	6.0	5.0	CD688871 EST5393 human nasopharynx cDNA, mRNA sequence	THC2725128	Unknown	
NEDD4	6.0	2.6	neural precursor cell expressed, developmentally down-regulated 4 (NEDD4), transcript variant 2, mRNA	NM_198400	Hs.1565	AL832063
NID2	6.0	6.8	nidogen 2 (osteonidogen) (NID2), mRNA	NM_007361	Hs.369840	NM_007361
CR616033	6.0	2.6	full-length cDNA clone CS0DF034YF04 of Fetal brain of (human).	CR616033	Hs.699548	NM_005897
HAND2	6.0	6.7	heart and neural crest derivatives expressed 2 (HAND2), mRNA	NM_021973	Hs.388245	NM_021973
CG018	6.0	7.7	hypothetical gene CG018 (CG018), transcript variant 1, mRNA	NM_052818	Hs.161220	BX648509
MYL4	6.0	2.4	myosin, light chain 4, alkali; atrial, embryonic (MYL4), transcript variant 2, mRNA	NM_002476	Hs.463300	BM919855
LOC492311	6.0	3.1	similar to bovine IgA regulatory protein (LOC492311), mRNA	NM_001007189	Hs.696360	NM_001007189
NICN1	6.0	1.8	nicolin 1 (NICN1), mRNA	NM_032316	Hs.191460	NM_032316
GSTM1	6.0	4.9	glutathione S-transferase M1 (GSTM1), transcript variant 2, mRNA	NM_146421	Hs.301961	BQ880398
AK024110	6.0	2.0	cDNA FLJ14048 fis, clone HEMBA1006650, weakly similar to ARP2/3 COMPLEX 20 KD SUBUNIT.	AK024110	Unknown	
SVEP1	6.0	5.3	sushi, von Willebrand factor type A, EGF and pentraxin domain containing 1 (SVEP1), mRNA	NM_153366	Hs.522334	NM_153366
ABCC6	6.0	2.7	ATP-binding cassette, sub-family C (CFTR/MRP), member 6 (ABCC6), transcript variant 2, mRNA	NM_001079528	Hs.643018	NM_001171
RSN	6.0	1.9	restin (Reed-Steinberg cell-expressed intermediate filament-associated protein) (RSN), transcript variant 1, mRNA	NM_002956	Hs.524809	NM_002956
C6orf152	6.0	2.6	chromosome 6 open reading frame 152 (C6orf152), mRNA	NM_181714	Hs.21945	BC050327
PLA2G4A	6.0	3.5	phospholipase A2, group IVA (cytosolic, calcium-dependent) (PLA2G4A), mRNA	NM_024420	Hs.497200	NM_024420
ZNF641	6.0	5.6	zinc finger protein 641	ENST00000301042	Unknown	
ZNF395	6.0	2.7	zinc finger protein 395 (ZNF395), mRNA	NM_018660	Hs.699209	NM_018660
AL049387	6.0	2.1	mRNA; cDNA DKFZp586N1918 (from clone DKFZp586N1918).	AL049387	Hs.458447	BC037530
ENST00000290390	6.0	2.3	ENST00000290390	ENST00000290390	Unknown	
PKIA	6.0	2.8	protein kinase (cAMP-dependent, catalytic) inhibitor alpha (PKIA), transcript variant 6, mRNA	NM_006823	Hs.433700	NM_006823
LMOD1	6.0	2.0	leiomodin 1 (smooth muscle) (LMOD1), mRNA	NM_012134	Hs.519075	AK127212
C14orf179	6.0	2.0	chromosome 14 open reading frame 179 (C14orf179), mRNA	NM_052873	Hs.532626	AK056735
FGG	6.0	19.2	fibrinogen gamma chain (FGG), transcript variant gamma-A, mRNA	NM_000509	Hs.546255	CR620626
B3GNT5	6.0	3.0	UDP-GlcNAc:betaGal beta-1,3-N-acetylglucosaminyltransferase 5 (B3GNT5), mRNA	NM_032047	Hs.208267	NM_032047
RB1	6.0	3.1	retinoblastoma 1 (including osteosarcoma) (RB1), mRNA	NM_000321	Hs.408528	L41870
SLC24A3	6.0	3.9	solute carrier family 24 (sodium/potassium/calcium exchanger), member 3 (SLC24A3), mRNA	NM_020689	Hs.654790	NM_020689
BX114329	6.0	10.3	BX114329 BX114329 Soares_NhHMPu_S1 cDNA clone IMAGp998G064741 ; IMAGE:1932317, mRNA sequence	BX114329	Unknown	
MYH6	6.0	96.9	myosin, heavy chain 6, cardiac muscle, alpha (cardiomyopathy, hypertrophic 1) (MYH6), mRNA	NM_002471	Hs.278432	NM_000257
BRD8	6.0	1.6	bromodomain containing 8 (BRD8), transcript variant 1, mRNA	NM_006696	Hs.519337	NM_139199
FAM82A	6.0	3.0	unknown mRNA.	AF435956	Hs.591566	AK095462
F5	6.0	3.7	coagulation factor V (proaccelerin, labile factor) (F5), mRNA	NM_000130	Hs.30054	NM_000130
DW443340	6.0	1.9	HHAGE041070 Human liver regeneration after partial hepatectomy cDNA, mRNA sequence	DW443340	Hs.631395	AI928355
GPC3	6.0	5.5	glypican 3 (GPC3), mRNA	NM_004484	Hs.699193	NM_004484
RP5-875H10.1	6.0	7.9	SAM domain containing 1 (LOC389432), mRNA	NM_001030060	Hs.567973	NM_001030060
CROP	6.0	2.4	cisplatin resistance-associated overexpressed protein (CROP), transcript variant 1, mRNA	NM_016424	Hs.130293	NM_016424

CGGBP1	6.0	2.3	CGG triplet repeat binding protein 1 (CGGBP1), transcript variant 1, mRNA	NM_001008390	Hs.444818	NM_001008390
THC2771474	6.0	2.2	Q8NFF8_HUMAN (Q8NFF8) MLL5, partial (3%)	THC2771474	Unknown	
BC053880	6.0	3.1	cDNA clone IMAGE:5753455.	BC053880	Unknown	
TCF1	6.0	3.6	transcription factor 1, hepatic; LF-B1, hepatic nuclear factor (HNF1), albumin proximal factor (TCF1), mRNA	NM_000545	Hs.654455	NM_000545
PIGH	6.0	2.7	phosphatidylinositol glycan anchor biosynthesis, class H (PIGH), mRNA	NM_004569	Hs.553497	NM_004569
HPS1	6.0	2.6	Hermansky-Pudlak syndrome 1 (HPS1), transcript variant 1, mRNA	NM_000195	Hs.404568	NM_000195
RB1	6.0	3.2	retinoblastoma 1 (including osteosarcoma) (RB1), mRNA	NM_000321	Hs.408528	L41870
GPR30	5.9	7.6	G protein-coupled receptor 30 (GPR30), transcript variant 3, mRNA	NM_001039966	Hs.20961	NM_001039966
ENST00000355570	5.9	2.6	solute carrier family 35, member E2	ENST00000355570	Unknown	
BMF	5.9	2.5	Bcl2 modifying factor (BMF), transcript variant 1, mRNA	NM_001003940	Hs.591104	NM_001003940
ALB	5.9	7.5	clone FLB9714 PRO2619 mRNA, complete cds.	AF130077	Hs.418167	AF130077
SLC39A6	5.9	1.7	solute carrier family 39 (zinc transporter), member 6 (SLC39A6), mRNA	NM_012319	Hs.79136	NM_012319
NFIB	5.9	3.2	nuclear factor I/B (NFIB), mRNA	NM_005596	Hs.699215	NM_005596
BE535679	5.9	3.2	601060331F1 NIH_MGC_10 cDNA clone IMAGE:3446983 5', mRNA sequence	BE535679	Hs.460002	NM_018340
C1QTNF3	5.9	4.6	C1q and tumor necrosis factor related protein 3 (C1QTNF3), transcript variant 2, mRNA	NM_181435	Hs.171929	NM_181435
CACNA1G	5.9	4.4	calcium channel, voltage-dependent, alpha 1G subunit (CACNA1G), transcript variant 1, mRNA	NM_018896	Hs.591169	NM_198396
TBC1D10C	5.9	3.9	TBC1 domain family, member 10C (TBC1D10C), mRNA	NM_198517	Hs.534648	AK122585
CCDC53	5.9	2.4	coiled-coil domain containing 53 (CCDC53), mRNA	NM_016053	Hs.405692	BQ067713
THC2642212	5.9	6.5	THC2642212	THC2642212	Unknown	
SLK	5.9	2.2	STE20-like kinase (yeast) (SLK), mRNA	NM_014720	Hs.591922	D86959
THC2680414	5.9	2.7	THC2680414	THC2680414	Unknown	
BC040293	5.9	3.8	cDNA clone IMAGE:4820330.	BC040293	Hs.587484	BC040293
PRPSAP1	5.9	1.7	phosphoribosyl pyrophosphate synthetase-associated protein 1 (PRPSAP1), mRNA	NM_002766	Hs.77498	CR609147
NEFL	5.9	2.4	neurofilament, light polypeptide 68kDa (NEFL), mRNA	NM_006158	Hs.521461	NM_006158
THAP2	5.9	6.7	THAP domain containing, apoptosis associated protein 2 (THAP2), mRNA	NM_031435	Hs.245798	AL136607
RPL30	5.9	1.9	ribosomal protein L30 (RPL30), mRNA	NM_000989	Hs.400295	AK128768
FLRT2	5.9	17.3	fibronectin leucine rich transmembrane protein 2 (FLRT2), mRNA	NM_013231	Hs.533710	NM_013231
SLC35D1	5.9	2.5	UDP-glucuronic acid/UDP-N-acetylgalactosamine transporter (UDP- GlcA/UDP-GalNAc transporter) (Solute carrier family 35 member D1) (UDP-galactose transporter-related protein 7) (UGTrel7).	ENST00000235345	Unknown	
AK022035	5.9	4.4	cDNA FLJ11973 fis, clone HEMBB1001221.	AK022035	Hs.659665	AK022035
THC2507152	5.9	2.0	THC2507152	THC2507152	Unknown	
CLN3	5.9	2.0	ceroid-lipofuscinosis, neuronal 3, juvenile (Batten, Spielmeyer-Vogt disease), mRNA (cDNA clone MGC:102840 IMAGE:5764535), complete cds.	BC111068	Hs.628393	AK090709
PROS1	5.9	8.0	protein S (alpha) (PROS1), mRNA	NM_000313	Hs.64016	M14338
THC2631248	5.9	4.5	Q7WVU3_ACTPL (Q7WVU3) Collagen adhesin (Fragment), partial (5%)	THC2631248	Unknown	
BBS5	5.9	4.9	Bardet-Biedl syndrome 5 (BBS5), mRNA	NM_152384	Hs.233398	NM_152384
HNRPA3	5.9	1.7	heterogeneous nuclear ribonucleoprotein A3 (HNRPA3), mRNA	NM_194247	Hs.516539	NM_194247
AI470277	5.9	8.0	AI470277 tj18h01.x1 NCI_CGAP_Gas4 cDNA clone IMAGE:2141905 3', mRNA sequence	AI470277	Hs.513779	AL136861
PLA2G4A	5.9	3.8	phospholipase A2, group IVA (cytosolic, calcium-dependent) (PLA2G4A), mRNA	NM_024420	Hs.497200	NM_024420
CYYR1	5.9	1.9	cysteine/tyrosine-rich 1 (CYYR1), mRNA	NM_052954	Hs.37445	NM_052954
ATF2	5.9	1.8	cDNA FLJ46899 fis, clone UTERU3022588, highly similar to Cyclic-AMP-dependent transcription factor ATF-2.	AK128731	Hs.592510	BC107698
NEBL	5.9	5.3	nebulette (NEBL), transcript variant 1, mRNA	NM_006393	Hs.5025	Y16241
NBPF11	5.9	3.1	neuroblastoma breakpoint family, member 11 (NBPF11), mRNA	NM_183372	Hs.636561	BX648497
SLC25A36	5.9	1.7	mRNA; cDNA DKFZp564C053 (from clone DKFZp564C053).	AL049246	Hs.144130	AL049246
CXCL12	5.9	3.5	chemokine (C-X-C motif) ligand 12 (stromal cell-derived factor 1) (CXCL12), transcript variant 1, mRNA	NM_199168	Hs.522891	AK090482
PLA2G4A	5.9	4.2	phospholipase A2, group IVA (cytosolic, calcium-dependent) (PLA2G4A), mRNA	NM_024420	Hs.497200	NM_024420
BC019667	5.9	1.8	cDNA clone IMAGE:4453251, partial cds.	BC019667	Hs.12798	BC067285
RPL7	5.9	1.4	ribosomal protein L7 (RPL7), mRNA	NM_000971	Hs.571841	BM808571
KIAA0485	5.9	4.8	mRNA, chromosome 1 specific transcript KIAA0485.	AB007954	Hs.604754	AB007954
RPS15A	5.9	1.6	ribosomal protein S15a (RPS15A), transcript variant 2, mRNA	NM_001019	Hs.370504	BM907705
THC2731029	5.9	4.4	THC2731029	THC2731029	Unknown	
IQCH	5.9	5.0	IQ motif containing H (IQCH), transcript variant 2, mRNA	NM_022784	Hs.657894	AK022538

CRSP6	5.9	1.4	cofactor required for Sp1 transcriptional activation, subunit 6, 77kDa (CRSP6), mRNA	NM_004268	Hs.444931	NM_004268
ARG1	5.9	89.2	arginase, liver (ARG1), mRNA	NM_000045	Hs.440934	AK128314
ATP10D	5.9	5.6	ATPase, Class V, type 10D (ATP10D), mRNA	NM_020453	Hs.437241	AJ441078
AQP4	5.9	3.7	aquaporin 4 (AQP4), transcript variant a, mRNA	NM_001650	Hs.315369	NM_001650
RPIA	5.9	2.1	ribose 5-phosphate isomerase A (ribose 5-phosphate epimerase) (RPIA), mRNA	NM_144563	Hs.469264	NM_144563
SERTAD4	5.9	7.9	SERTA domain containing 4	ENST00000367012	Unknown	
CR610949	5.9	29.3	full-length cDNA clone CS0DD005YM02 of Neuroblastoma Cot 50-normalized of (human).	CR610949	Hs.388565	BX538341
RPS11	5.9	1.6	ribosomal protein S11 (RPS11), mRNA	NM_001015	Hs.433529	AK130324
SDK1	5.9	2.1	sidekick homolog 1 (chicken) (SDK1), transcript variant 1, mRNA	NM_152744	Hs.655699	NM_152744
STAM2	5.9	2.4	signal transducing adaptor molecule (SH3 domain and ITAM motif) 2 (STAM2), mRNA	NM_005843	Hs.17200	NM_005843
AF086536	5.9	2.4	full length insert cDNA clone ZE08A03.	AF086536	Hs.700067	W95952
RBMS3	5.9	2.2	RNA binding motif, single stranded interacting protein (RBMS3), transcript variant 2, mRNA	NM_014483	Hs.696468	AL831860
CCDC103	5.9	2.0	coiled-coil domain containing 103	ENST00000357776	Unknown	
HDAC4	5.9	1.6	histone deacetylase 4 (HDAC4), mRNA	NM_006037	Hs.20516	NM_006037
SCD5	5.9	18.7	stearoyl-CoA desaturase 5 (SCD5), transcript variant 1, mRNA	NM_001037582	Hs.379191	NM_001037582
TMEM38B	5.9	2.1	transmembrane protein 38B (TMEM38B), mRNA	NM_018112	Hs.411925	AK001355
EVI5	5.9	2.3	ecotropic viral integration site 5 (EVI5), mRNA	NM_005665	Hs.656836	NM_005665
TMEM123	5.9	1.8	transmembrane protein 123 (TMEM123), mRNA	NM_052932	Hs.503709	NM_052932
THC2643327	5.9	3.6	THC2643327	THC2643327	Unknown	
PLOD2	5.9	2.8	procollagen-lysine, 2-oxoglutarate 5-dioxygenase 2 (PLOD2), transcript variant 1, mRNA	NM_182943	Hs.477866	NM_182943
CR610181	5.9	2.1	full-length cDNA clone CS0DM006YA12 of Fetal liver of (human).	CR610181	Hs.663111	CR610181
FLJ35834	5.9	4.5	hypothetical protein FLJ35834 (FLJ35834), mRNA	NM_178827	Hs.159650	AK127736
BC089156	5.9	2.9	cDNA clone IMAGE:6576427.	BC089156	Hs.30567	BC020163
MGC70863	5.9	1.5	similar to RPL23AP7 protein (MGC70863), transcript variant 1, mRNA	NM_203477	Hs.406135	BC065556
CCT6B	5.9	2.8	chaperonin containing TCP1, subunit 6B (zeta 2) (CCT6B), mRNA	NM_006584	Hs.73072	CR933688
ARL1	5.8	1.9	ADP-ribosylation factor-like 1 (ARL1), mRNA	NM_001177	Hs.372616	BX537387
THC2582300	5.8	2.8	Q2IIH7_ANADE (Q2IIH7) PE-PGRS family protein, partial (3%)	THC2582300	Unknown	
HP1BP3	5.8	4.0	cDNA FLJ13067 fis, clone NT2RP3001712, highly similar to HP1-BP74 protein mRNA.	AK023129	Hs.142442	BC045660
FLJ37453	5.8	2.4	cDNA FLJ37453 fis, clone BRAWH2010754.	AK094772	Hs.119298	AK124018
MRAS	5.8	2.0	muscle RAS oncogene homolog (MRAS), mRNA	NM_012219	Hs.527021	NM_012219
HECTD2	5.8	1.8	HECT domain containing 2 (HECTD2), transcript variant 2, mRNA	NM_173497	Hs.656960	BC040187
A_24_P255252	5.8	2.8	A_24_P255252	A_24_P255252	Unknown	
KIFAP3	5.8	10.3	kinesin-associated protein 3 (KIFAP3), mRNA	NM_014970	Hs.433442	AK122897
TXLNB	5.8	5.0	taxilin beta (TXLNB), mRNA	NM_153235	Hs.535820	BX647477
LOC442260	5.8	1.6	PREDICTED: similar to 60S ribosomal protein L23a (LOC442260), mRNA	XR_019361	Hs.648040	XR_019361
AP3M2	5.8	2.2	adaptor-related protein complex 3, mu 2 subunit (AP3M2), mRNA	NM_006803	Hs.654529	BX648390
AK129956	5.8	2.9	cDNA FLJ26446 fis, clone KDN02743.	AK129956	Hs.170131	NM_205843
WDR78	5.8	11.7	WD repeat domain 78 (WDR78), transcript variant 2, mRNA	NM_207014	Hs.49421	AK127011
RPL9	5.8	1.8	ribosomal protein L9 (RPL9), transcript variant 2, mRNA	NM_001024921	Hs.412370	CR595992
FLJ37453	5.8	1.9	cDNA FLJ37453 fis, clone BRAWH2010754.	AK094772	Hs.119298	AK124018
AMHR2	5.8	7.7	anti-Mullerian hormone receptor, type II (AMHR2), mRNA	NM_020547	Hs.659889	CR627318
RPL23	5.8	1.4	ribosomal protein L23 (RPL23), mRNA	NM_000978	Hs.406300	CR604268
C1orf162	5.8	3.5	chromosome 1 open reading frame 162 (C1orf162), mRNA	NM_174896	Hs.288010	AK123160
PHF20L1	5.8	2.1	PHD finger protein 20-like 1 (PHF20L1), transcript variant 1, mRNA	NM_016018	Hs.304362	NM_016018
ZNF187	5.8	1.8	zinc finger protein 187 (ZNF187), transcript variant 1, mRNA	NM_007151	Hs.157883	NM_007151
COL5A2	5.8	5.7	collagen, type V, alpha 2 (COL5A2), mRNA	NM_000393	Hs.445827	NM_000393
RPL23	5.8	1.8	ribosomal protein L23 (RPL23), mRNA	NM_000978	Hs.406300	CR604268
LOC664727	5.8	3.0	cDNA FLJ36340 fis, clone THYMU2006468.	AK093659	Hs.472508	AK092606
TMEM56	5.8	3.9	transmembrane protein 56 (TMEM56), mRNA	NM_152487	Hs.483512	BX537731
CR603450	5.8	2.3	full-length cDNA clone CS0DI004YB08 of Placenta Cot 25-normalized of (human).	CR603450	Hs.458412	AB209243
DHFRL1	5.8	2.5	dihydrofolate reductase-like 1 (DHFRL1), mRNA	NM_176815	Hs.448003	BC045541
PCDHB14	5.8	3.6	protocadherin beta 14 (PCDHB14), mRNA	NM_018934	Hs.658497	BC050417

FABP7	5.8	5.5	fatty acid binding protein 7, brain (FABP7), mRNA	NM_001446	Hs.26770	AB208815
AW235110	5.8	2.3	xn18f11.x1 NCI_CGAP_Kid11 cDNA clone IMAGE:2694093 3' similar to gb:M65131 METHYLMALONYL-COA MUTASE PRECURSOR (HUMAN);, mRNA sequence	AW235110	Hs.653296	AW235110
KCNV1	5.8	12.2	potassium channel, subfamily V, member 1 (KCNV1), mRNA	NM_014379	Hs.13285	NM_014379
GDNF	5.8	3.3	Glial cell line-derived neurotrophic factor precursor (Astrocyte- derived trophic factor 1) (ATF-1).	ENST00000381827	Unknown	
ST3GAL5	5.8	4.0	ST3 beta-galactoside alpha-2,3-sialyltransferase 5 (ST3GAL5), transcript variant 1, mRNA	NM_003896	Hs.415117	AK127346
TP73L	5.8	9.5	tumor protein p73-like (TP73L), mRNA	NM_003722	Hs.137569	NM_003722
LOC647580	5.8	3.7	PREDICTED: hypothetical LOC647580 (LOC647580), mRNA	XR_018208	Hs.651063	XR_018208
MYH6	5.8	39.3	myosin, heavy chain 6, cardiac muscle, alpha (cardiomyopathy, hypertrophic 1) (MYH6), mRNA	NM_002471	Hs.278432	NM_000257
A_24_P561165	5.8	2.1	A_24_P561165	A_24_P561165	Unknown	
PDE4DIP	5.8	11.5	phosphodiesterase 4D interacting protein (myomegalin) (PDE4DIP), transcript variant 3, mRNA	NM_022359	Hs.654651	NM_014644
KLHL1	5.8	4.3	kelch-like 1 (Drosophila) (KLHL1), mRNA	NM_020866	Hs.508201	AB040923
A_24_P50489	5.8	1.8	A_24_P50489	A_24_P50489	Unknown	
AK123297	5.8	2.6	cDNA FLJ41303 fis, clone BRAMY2042131.	AK123297	Hs.648656	AK123297
DDEF2	5.8	2.9	development and differentiation enhancing factor 2 (DDEF2), mRNA	NM_003887	Hs.555902	NM_003887
HSBP1	5.8	2.1	heat shock factor binding protein 1 (HSBP1), mRNA	NM_001537	Hs.250899	BX648254
THC2708803	5.8	4.5	THC2708803	THC2708803	Unknown	
SLC44A3	5.8	4.5	solute carrier family 44, member 3 (SLC44A3), mRNA	NM_152369	Hs.483423	BC033858
SERPINA5	5.8	6.0	serpin peptidase inhibitor, clade A (alpha-1 antiproteinase, antitrypsin), member 5 (SERPINA5), mRNA	NM_000624	Hs.510334	NM_000624
NAIP	5.8	5.1	NLR family, apoptosis inhibitory protein (NAIP), transcript variant 1, mRNA	NM_004536	Hs.654500	NM_004536
BAMBI	5.8	5.0	BMP and activin membrane-bound inhibitor homolog (Xenopus laevis) (BAMBI), mRNA	NM_012342	Hs.533336	NM_012342
GKAP1	5.8	2.1	G kinase anchoring protein 1 (GKAP1), mRNA	NM_025211	Hs.522255	AK026487
AF010236	5.8	8.6	mRNA from chromosome 5q31-33 region.	AF010236	Hs.387207	AF010236
AI827481	5.8	8.6	AI827481 wf29h01.x1 Soares_NFL_T_GBC_S1 cDNA clone IMAGE:2357041 3', mRNA sequence	AI827481	Hs.154652	NM_147189
CCDC93	5.8	2.0	coiled-coil domain containing 93 (CCDC93), mRNA	NM_019044	Hs.107845	NM_019044
ZNF222	5.8	1.8	zinc finger protein 222 (ZNF222), mRNA	NM_013360	Hs.279840	AK095196
A_32_P49392	5.8	1.6	A_32_P49392	A_32_P49392	Unknown	
GSTA2	5.8	5.9	glutathione S-transferase A2 (GSTA2), mRNA	NM_000846	Hs.94107	BI762502
ANGPT1	5.8	2.7	angiopoietin 1 (ANGPT1), mRNA	NM_001146	Hs.369675	BX648814
LOC401725	5.8	1.7	PREDICTED: similar to 60S ribosomal protein L6 (TAX-responsive enhancer element-binding protein 107) (TAXREB107) (Neoplasm-related protein C140) (LOC401725), mRNA	XR_019536	Hs.646417	XR_019536
C3orf9	5.8	1.7	chromosome 3 open reading frame 9 (C3orf9), mRNA	NM_152305	Hs.231750	AK126736
RNF19	5.8	3.4	ring finger protein 19 (RNF19), transcript variant 1, mRNA	NM_183419	Hs.292882	NM_183419
THNSL1	5.8	2.1	Threonine synthase-like 1.	ENST00000376356	Unknown	
BC011779	5.8	2.2	cDNA clone IMAGE:3941306, partial cds.	BC011779	Hs.634705	BC065527
THC2717825	5.8	8.8	THC2717825	THC2717825	Unknown	
5-Sep	5.8	4.5	septin 5 (SEPT5), mRNA	NM_002688	Hs.283743	NM_002688
RRAD	5.8	5.1	Ras-related associated with diabetes (RRAD), mRNA	NM_004165	Hs.1027	BC057815
SEC24B	5.8	1.7	SEC24 related gene family, member B (S. cerevisiae) (SEC24B), transcript variant 1, mRNA	NM_006323	Hs.292472	AJ131245
PRDM2	5.8	2.5	PR domain containing 2, with ZNF domain (PRDM2), transcript variant 2, mRNA	NM_015866	Hs.371823	NM_012231
CYBRD1	5.8	2.6	cytochrome b reductase 1 (CYBRD1), mRNA	NM_024843	Hs.221941	AL136693
LMBRD1	5.8	1.9	LMBR1 domain containing 1 (LMBRD1), mRNA	NM_018368	Hs.271643	AB074281
A_24_P392436	5.8	1.6	A_24_P392436	A_24_P392436	Unknown	
ARMCX3	5.8	2.9	armadillo repeat containing, X-linked 3 (ARMCX3), transcript variant 1, mRNA	NM_016607	Hs.592225	CR933656
H2AFV	5.8	2.4	Histone H2AV (H2A.F/Z).	ENST00000222690	Unknown	
AK025669	5.8	2.5	cDNA: FLJ22016 fis, clone HEP07422.	AK025669	Unknown	
BC089451	5.8	23.6	cDNA clone IMAGE:30554612.	BC089451	Hs.44098	AB075498
C10orf107	5.8	16.8	chromosome 10 open reading frame 107 (C10orf107), mRNA	NM_173554	Hs.673160	NM_173554
FMN1	5.8	3.0	formin 1, mRNA (cDNA clone IMAGE:4213061).	BC029107	Hs.657649	CR749577
KCNA5	5.8	9.9	potassium voltage-gated channel, shaker-related subfamily, member 5 (KCNA5), mRNA	NM_002234	Hs.150208	M55513
ZRANB2	5.8	1.9	zinc finger, RAN-binding domain containing 2 (ZRANB2), transcript variant 2, mRNA	NM_005455	Hs.194718	CR749325

RBM24	5.8	4.8	RNA binding motif protein 24 (RBM24), mRNA	NM_153020	Hs.519904	AL832199
CD302	5.8	4.0	CD302 molecule (CD302), mRNA	NM_014880	Hs.130014	AY314007
KIAA1462	5.8	5.4	Novel protein.	ENST00000375377	Unknown	
THC2669961	5.8	6.3	THC2669961	THC2669961	Unknown	
HECA	5.8	3.6	headcase homolog (Drosophila) (HECA), mRNA	NM_016217	Hs.197644	BC070068
OLIG3	5.8	7.8	oligodendrocyte transcription factor 3 (OLIG3), mRNA	NM_175747	Hs.195398	BC051352
AMOT	5.8	14.5	angiomotin (AMOT), mRNA	NM_133265	Hs.528051	NM_133265
ZNF214	5.8	2.5	zinc finger protein 214 (ZNF214), mRNA	NM_013249	Hs.445849	NM_013249
SEC31B	5.8	2.2	SEC31 homolog B (S. cerevisiae) (SEC31B), mRNA	NM_015490	Hs.18889	BC071580
FLJ36748	5.7	3.3	hypothetical protein FLJ36748 (FLJ36748), mRNA	NM_152406	Hs.483793	NM_152406
ANGPTL1	5.7	10.3	angiopoietin-like 1 (ANGPTL1), mRNA	NM_004673	Hs.591474	NM_004673
THC2607703	5.7	2.3	THC2607703	THC2607703	Unknown	
DAB2	5.7	3.0	disabled homolog 2, mitogen-responsive phosphoprotein (Drosophila) (DAB2), mRNA	NM_001343	Hs.481980	NM_001343
NBPF3	5.7	3.3	neuroblastoma breakpoint family, member 3 (NBPF3), mRNA	NM_032264	Hs.325422	AK095602
THC2507925	5.7	2.8	ALU7_HUMAN (P39194) Alu subfamily SQ sequence contamination warning entry, partial (29%)	THC2507925	Unknown	
PCBP4	5.7	2.1	poly(rC) binding protein 4 (PCBP4), transcript variant 4, mRNA	NM_033010	Hs.20930	NM_020418
CFB	5.7	5.9	complement factor B (CFB), mRNA	NM_001710	Hs.69771	NM_001710
C21orf122	5.7	2.1	chromosome 21 open reading frame 122, mRNA (cDNA clone MGC:10960 IMAGE:3633193), complete cds.	BC004343	Hs.309203	BC004343
C21orf34	5.7	17.0	chromosome 21 open reading frame 34 (C21orf34), transcript variant 1, mRNA	NM_001005732	Hs.473394	AK095614
VCAM1	5.7	42.0	vascular cell adhesion molecule 1 (VCAM1), transcript variant 1, mRNA	NM_001078	Hs.109225	CR749464
EFCAB1	5.7	5.2	EF-hand calcium binding domain 1 (EFCAB1), mRNA	NM_024593	Hs.23245	NM_024593
PXK	5.7	4.1	PX ser/thr kinase v2 mRNA, complete cds, alternatively spliced.	AY437879	Hs.190544	BX537600
ENC1	5.7	5.9	ectodermal-neural cortex (with BTB-like domain) (ENC1), mRNA	NM_003633	Hs.104925	AF059611
TCF8	5.7	10.1	transcription factor 8 (represses interleukin 2 expression) (TCF8), mRNA	NM_030751	Hs.124503	BX647794
NFX1	5.7	2.0	nuclear transcription factor, X-box binding 1 (NFX1), transcript variant 1, mRNA	NM_002504	Hs.413074	NM_002504
BQ004014	5.7	13.9	BQ004014 UI-1-BC0-afv-f-01-0-UI.s1 NCI_CGAP_Pl1 cDNA clone UI-1-BC0-afv-f-01-0-UI 3', mRNA sequence	BQ004014	Hs.694789	NM_001920
RSL1D1	5.7	3.4	ribosomal L1 domain containing 1 (RSL1D1), mRNA	NM_015659	Hs.401842	NM_015659
EFNA1	5.7	13.8	ephrin-A1 (EFNA1), transcript variant 1, mRNA	NM_004428	Hs.516664	AK057845
DSCR1	5.7	4.4	Down syndrome critical region gene 1 (DSCR1), transcript variant 1, mRNA	NM_004414	Hs.282326	AY325903
PDE11A	5.7	13.7	phosphodiesterase 11A (PDE11A), transcript variant 4, mRNA	NM_016953	Hs.570273	NM_016953
FMO4	5.7	3.2	flavin containing monooxygenase 4 (FMO4), mRNA	NM_002022	Hs.386502	BC002780
PLA2G4A	5.7	3.5	phospholipase A2, group IVA (cytosolic, calcium-dependent) (PLA2G4A), mRNA	NM_024420	Hs.497200	NM_024420
ENST00000369326	5.7	3.4	Notch homolog 2 N-terminal like protein	ENST00000369326	Unknown	
PCGF3	5.7	2.0	polycomb group ring finger 3 (PCGF3), mRNA	NM_006315	Hs.144309	NM_006315
THC2615680	5.7	2.7	ALU8_HUMAN (P39195) Alu subfamily SX sequence contamination warning entry, partial (4%)	THC2615680	Unknown	
CR608563	5.7	6.8	full-length cDNA clone CS0DI029YI16 of Placenta Cot 25-normalized of (human).	CR608563	Hs.655797	AK124757
TMED5	5.7	2.6	transmembrane emp24 protein transport domain containing 5 (TMED5), mRNA	NM_016040	Hs.482873	CR936808
MAP9	5.7	4.3	microtubule-associated protein 9 (MAP9), mRNA	NM_001039580	Hs.61271	NM_001039580
LRCH2	5.7	2.6	leucine-rich repeats and calponin homology (CH) domain containing 2 (LRCH2), mRNA	NM_020871	Hs.65366	BX647193
ENST00000378953	5.7	2.4	CDNA FLJ39622 fis, clone SMINT2001199.	ENST00000378953	Unknown	
WNT5A	5.7	6.1	wingless-type MMTV integration site family, member 5A (WNT5A), mRNA	NM_003392	Hs.696364	NM_003392
ZNF343	5.7	2.5	zinc finger protein 343 (ZNF343), mRNA	NM_024325	Hs.516846	NM_024325
NTRK2	5.7	70.2	neurotrophic tyrosine kinase, receptor, type 2 (NTRK2), transcript variant b, mRNA	NM_001007097	Hs.494312	NM_001018065
CXCL12	5.7	3.5	chemokine (C-X-C motif) ligand 12 (stromal cell-derived factor 1) (CXCL12), transcript variant 1, mRNA	NM_199168	Hs.522891	AK090482
THC2611636	5.7	2.7	THC2611636	THC2611636	Unknown	
C18orf34	5.7	8.1	cDNA FLJ26445 fis, clone KDN02608.	AK129955	Hs.115461	AK126038
BC039414	5.7	35.3	cDNA clone IMAGE:5302158.	BC039414	Hs.655414	BC039414
AK055641	5.7	1.6	cDNA FLJ31079 fis, clone HSYRA2001595.	AK055641	Hs.349283	AK095108
AK131023	5.7	14.7	cDNA FLJ27513 fis, clone TST08509.	AK131023	Hs.662541	BC062771
NUCB2	5.7	2.7	nucleobindin 2 (NUCB2), mRNA	NM_005013	Hs.654599	AK128739
THC2718727	5.7	1.9	THC2718727	THC2718727	Unknown	

VPS13B	5.7	2.0	vacuolar protein sorting 13 homolog B (yeast) (VPS13B), transcript variant 5, mRNA	NM_017890	Hs.191540	NM_017890
SMYD1	5.7	9.3	SET and MYND domain containing 1 (SMYD1), mRNA	NM_198274	Hs.516176	NM_198274
A_24_P587938	5.7	1.4	A_24_P587938	A_24_P587938	Unknown	
KIAA1450	5.7	2.3	mRNA for KIAA1450 protein, partial cds.	AB040883	Hs.652441	NM_020840
NR2F1	5.7	18.7	nuclear receptor subfamily 2, group F, member 1 (NR2F1), mRNA	NM_005654	Hs.519445	NM_005654
CXCL12	5.7	3.5	chemokine (C-X-C motif) ligand 12 (stromal cell-derived factor 1) (CXCL12), transcript variant 1, mRNA	NM_199168	Hs.522891	AK090482
ZADH2	5.7	5.2	zinc binding alcohol dehydrogenase, domain containing 2 (ZADH2), mRNA	NM_175907	Hs.591065	NM_175907
THC2669092	5.7	2.2	THC2669092	THC2669092	Unknown	
GJB2	5.7	5.9	gap junction protein, beta 2, 26kDa (connexin 26) (GJB2), mRNA	NM_004004	Hs.591234	NM_004004
LOC652558	5.7	1.6	PREDICTED: similar to 60S ribosomal protein L7 (LOC652558), mRNA	XR_019386	Hs.647657	XR_019386
C9orf103	5.7	4.2	chromosome 9 open reading frame 103 (C9orf103), mRNA	NM_001001551	Hs.530261	BC036421
TBX3	5.7	7.0	T-box 3 (ulnar mammary syndrome) (TBX3), transcript variant 2, mRNA	NM_016569	Hs.129895	NM_016569
HEMK1	5.7	2.4	HemK methyltransferase family member 1 (HEMK1), mRNA	NM_016173	Hs.517987	NM_016173
LOC286025	5.7	2.2	cDNA FLJ38703 fis, clone KIDNE2002265.	AK096022	Hs.605380	NM_020910
PLXNA4A	5.7	4.2	mRNA for KIAA1550 protein, partial cds.	AB046770	Hs.511454	AB046770
KCNQ2	5.7	3.9	Potassium voltage-gated channel subfamily KQT member 2 (Voltage-gated potassium channel subunit Kv7.2) (Neuroblastoma-specific potassium channel subunit alpha KvLQT2) (KQT-like 2).	ENST00000370237	Unknown	
MLF1	5.7	2.0	myeloid leukemia factor 1 variant 3 (MLF1) mRNA, complete cds, alternatively spliced.	AY848702	Hs.85195	BX641078
ACYP2	5.7	2.2	acylphosphatase 2, muscle type (ACYP2), mRNA	NM_138448	Hs.516173	BX647332
KIAA0644	5.7	2.6	KIAA0644 gene product (KIAA0644), mRNA	NM_014817	Hs.21572	NM_014817
PLCL1	5.7	5.7	phospholipase C-like 1 (PLCL1), mRNA	NM_006226	Hs.153322	NM_006226
BC031320	5.7	1.9	cDNA clone IMAGE:5278682.	BC031320	Hs.220864	NM_001271
SFRS12	5.7	2.4	splicing factor, arginine/serine-rich 12 (SFRS12), transcript variant 2, mRNA	NM_139168	Hs.519347	NM_139168
CRYZL1	5.7	2.3	crystallin, zeta (quinone reductase)-like 1 (CRYZL1), mRNA	NM_145858	Hs.352671	BX648547
THC2727290	5.7	2.3	T32824 EST54797 Human Brain cDNA 3' end similar to None., mRNA sequence	THC2727290	Unknown	
SNAP25	5.7	5.0	synaptosomal-associated protein, 25kDa (SNAP25), transcript variant 1, mRNA	NM_003081	Hs.167317	AK090857
FLI1	5.7	9.0	Friend leukemia virus integration 1 (FLI1), mRNA	NM_002017	Hs.504281	BX647094
BAZ2B	5.7	2.1	bromodomain adjacent to zinc finger domain, 2B (BAZ2B), mRNA	NM_013450	Hs.470369	NM_013450
NBPF10	5.7	3.2	neuroblastoma breakpoint family, member 10 (NBPF10), mRNA	NM_001039703	Hs.515947	BX538005
GNPDA2	5.7	2.4	putative glucosamine-6-phosphate isomerase mRNA, complete cds.	AY173948	Hs.21398	AB209904
JRKL	5.7	2.4	jerky homolog-like (mouse) (JRKL), mRNA	NM_003772	Hs.105940	NM_003772
RP11-35N6.1	5.7	2.4	plasticity related gene 3 (PRG-3), transcript variant 1, mRNA	NM_207299	Hs.382683	NM_207299
KCNJ5	5.6	4.0	potassium inwardly-rectifying channel, subfamily J, member 5 (KCNJ5), mRNA	NM_000890	Hs.632109	NM_000890
THC2645586	5.6	2.5	Q9P3E1_NEUCR (Q9P3E1) Related to rna-binding protein fus/tls, partial (5%)	THC2645586	Unknown	
PTN	5.6	4.4	pleiotrophin (heparin binding growth factor 8, neurite growth-promoting factor 1) (PTN), mRNA	NM_002825	Hs.371249	CR624136
ID4	5.6	5.6	inhibitor of DNA binding 4, dominant negative helix-loop-helix protein (ID4), mRNA	NM_001546	Hs.519601	NM_001546
THC2611971	5.6	2.7	Q23IV5_TETTH (Q23IV5) Leucine Rich Repeat family protein, partial (3%)	THC2611971	Unknown	
TTLL7	5.6	2.8	Tubulin--tyrosine ligase-like protein 7 (Protein NYD-SP30).	ENST00000370703	Unknown	
ENST00000371189	5.6	6.9	Nuclear factor 1 A-type (Nuclear factor 1/A) (NF1-A) (NFI-A) (NF-I/A) (CCAAT-box-binding transcription factor) (CTF) (TGGCA-binding protein).	ENST00000371189	Unknown	
TIGD1	5.6	2.3	tigger transposable element derived 1 (TIGD1), mRNA	NM_145702	Hs.211823	BC035143
C5orf15	5.6	1.8	chromosome 5 open reading frame 15 (C5orf15), mRNA	NM_020199	Hs.355177	NM_020199
FLJ11171	5.6	2.1	hypothetical protein FLJ11171 (FLJ11171), mRNA	NM_018348	Hs.72782	NM_018348
AK124806	5.6	3.7	cDNA FLJ42816 fis, clone BRCAN2014881.	AK124806	Hs.34871	NM_014795
FXYD6	5.6	2.6	FXYD domain containing ion transport regulator 6 (FXYD6), mRNA	NM_022003	Hs.635508	AK092198
CXCL12	5.6	3.6	chemokine (C-X-C motif) ligand 12 (stromal cell-derived factor 1) (CXCL12), transcript variant 1, mRNA	NM_199168	Hs.522891	AK090482
SH3BP5	5.6	3.0	SH3-domain binding protein 5 (BTK-associated) (SH3BP5), transcript variant 1, mRNA	NM_004844	Hs.257761	NM_004844
CRISPLD1	5.6	1.8	cysteine-rich secretory protein LCCL domain containing 1 (CRISPLD1), mRNA	NM_031461	Hs.436542	NM_031461
LRRC48	5.6	10.4	leucine rich repeat containing 48 (LRRC48), mRNA	NM_031294	Hs.579264	AK096528
MBOAT2	5.6	1.9	membrane bound O-acyltransferase domain containing 2 (MBOAT2), mRNA	NM_138799	Hs.467634	NM_138799
ZFPM2	5.6	4.2	zinc finger protein, multitype 2 (ZFPM2), mRNA	NM_012082	Hs.431009	NM_012082
IGSF4	5.6	6.1	immunoglobulin superfamily, member 4 (IGSF4), mRNA	NM_014333	Hs.370510	BX641042
KALRN	5.6	5.6	kalirin, RhoGEF kinase (KALRN), transcript variant 1, mRNA	NM_001024660	Hs.8004	NM_001024660

RSPO3	5.6	5.2	R-spondin 3 homolog (Xenopus laevis) (RSPO3), mRNA	NM_032784	Hs.135254	AF251057
STRA6	5.6	2.8	stimulated by retinoic acid gene 6 homolog (mouse) (STRA6), mRNA	NM_022369	Hs.24553	AK091152
PLA2G4A	5.6	3.7	phospholipase A2, group IVA (cytosolic, calcium-dependent) (PLA2G4A), mRNA	NM_024420	Hs.497200	NM_024420
IMPAD1	5.6	3.7	inositol monophosphatase domain containing 1 (IMPAD1), mRNA	NM_017813	Hs.591872	BC067814
RNASEL	5.6	2.8	ribonuclease L (2',5'-oligoisoadenylate synthetase-dependent) (RNASEL), mRNA	NM_021133	Hs.518545	BC090934
ABCA8	5.6	7.7	ATP-binding cassette, sub-family A (ABC1), member 8 (ABCA8), mRNA	NM_007168	Hs.58351	NM_007168
ENST00000260045	5.6	1.9	52 kDa repressor of the inhibitor of the protein kinase (p58IPK- interacting protein) (58 kDa interferon-induced protein kinase- interacting protein) (P52rIPK) (Death-associated protein 4) (THAP domain-containing protein 0),...	ENST00000260045	Unknown	
LOC440248	5.6	2.0	hypothetical LOC440248 (LOC440248), mRNA	NM_199045	Unknown	
AF147412	5.6	1.6	full length insert cDNA clone YP59C02.	AF147412	Hs.684614	AF147412
EXOC1	5.6	1.6	exocyst complex component 1 (EXOC1), transcript variant 3, mRNA	NM_001024924	Hs.269665	NM_001024924
MGC52110	5.6	2.1	hypothetical protein MGC52110 (MGC52110), mRNA	NM_001008215	Hs.596537	BC047722
MLLT11	5.6	2.5	myeloid/lymphoid or mixed-lineage leukemia (trithorax homolog, Drosophila); translocated to, 11 (MLLT11), mRNA	NM_006818	Hs.75823	BC022448
DIXDC1	5.6	2.4	DIX domain containing 1 (DIXDC1), transcript variant 1, mRNA	NM_001037954	Hs.655626	NM_001037954
PTPMT1	5.6	1.7	protein tyrosine phosphatase, mitochondrial 1, mRNA (cDNA clone IMAGE:3348134), partial cds.	BC014048	Hs.700861	BC018974
TRAK1	5.6	1.5	trafficking protein, kinesin binding 1 (TRAK1), transcript variant 1, mRNA	NM_001042646	Hs.535711	NM_001042646
C20orf199	5.6	1.6	unknown protein 2 mRNA, complete cds.	AY513723	Hs.356766	CR936805
GABARAPL2	5.6	2.1	GABA(A) receptor-associated protein-like 2 (GABARAPL2), mRNA	NM_007285	Hs.461379	BC040312
TEX10	5.6	3.6	cDNA clone IMAGE:5725529.	BC110864	Hs.655024	BC110864
C6orf52	5.6	7.3	chromosome 6 open reading frame 52, mRNA (cDNA clone IMAGE:4096427), partial cds.	BC016820	Hs.61389	AW001000
NEK9	5.6	2.2	Serine/threonine-protein kinase Nek9 (EC 2.7.11.1) (NimA-related protein kinase 9) (Never in mitosis A-related kinase 9) (Nercc1 kinase) (NIMA-related kinase 8) (Nek8).	ENST00000238616	Unknown	
THC2633136	5.6	1.7	Q9F8M7_CARHY (Q9F8M7) DTDP-glucose 4,6-dehydratase (Fragment), partial (11%)	THC2633136	Unknown	
LAMA2	5.6	3.6	laminin, alpha 2 (merosin, congenital muscular dystrophy) (LAMA2), transcript variant 1, mRNA	NM_000426	Hs.200841	NM_000426
TNNI3K	5.6	3.1	TNNI3 interacting kinase (TNNI3K), mRNA	NM_015978	Hs.480085	BX640903
JPH2	5.6	2.5	junctophilin 2 (JPH2), transcript variant 1, mRNA	NM_020433	Hs.441737	NM_020433
LOC727835	5.6	1.7	PREDICTED: similar to 60S ribosomal protein L9 (LOC727835), mRNA	XR_015189	Hs.678843	XR_015767
BE798911	5.6	40.4	601585434F1 NIH_MGC_7 cDNA clone IMAGE:3939776 5', mRNA sequence	BE798911	Hs.599655	BE798911
TMEM98	5.6	3.9	transmembrane protein 98 (TMEM98), transcript variant 1, mRNA	NM_015544	Hs.695982	CR617078
HSZFP36	5.6	2.7	Zinc finger protein ZFP-36 (Fragment).	ENST00000341191	Unknown	
TBX5	5.6	11.8	T-box 5 (TBX5), transcript variant 2, mRNA	NM_080718	Hs.381715	NM_000192
A_32_P9532	5.6	4.3	A_32_P9532	A_32_P9532	Unknown	
CD34	5.6	18.5	CD34 molecule (CD34), transcript variant 2, mRNA	NM_001773	Hs.374990	BX640941
FLJ10324	5.6	2.3	hypothetical protein FLJ10324 (FLJ10324), mRNA	NM_018059	Hs.667336	AB058752
ENST00000333351	5.6	1.5	PREDICTED: hypothetical LOC391504 (LOC391504), mRNA	ENST00000333351	Unknown	
PDZD3	5.6	3.4	PDZ domain containing 3 (PDZD3), mRNA	NM_024791	Hs.374726	AB094096
SNAI2	5.6	9.3	snail homolog 2 (Drosophila) (SNAI2), mRNA	NM_003068	Hs.360174	NM_003068
PIK3R3	5.6	3.0	phosphoinositide-3-kinase, regulatory subunit 3 (p55, gamma) (PIK3R3), mRNA	NM_003629	Hs.655387	NM_003629
KRT222P	5.6	4.3	keratin 222 pseudogene (KRT222P), mRNA	NM_152349	Hs.6920	NM_152349
THC2544977	5.6	2.2	THC2544977	THC2544977	Unknown	
ZC3H11A	5.6	3.2	zinc finger CCCH-type containing 11A (ZC3H11A), mRNA	NM_014827	Hs.532399	CR627439
ACYP2	5.6	2.6	acylphosphatase 2, muscle type (ACYP2), mRNA	NM_138448	Hs.516173	BX647332
C6orf70	5.6	2.5	chromosome 6 open reading frame 70 (C6orf70), mRNA	NM_018341	Hs.47546	NM_018341
KAZALD1	5.6	6.2	Kazal-type serine protease inhibitor domain-containing protein 1 precursor.	ENST00000224809	Unknown	
BBS12	5.6	2.1	Bardet-Biedl syndrome 12 (BBS12), mRNA	NM_152618	Hs.400698	BC055426
AZI2	5.6	2.0	5-azacytidine induced 2 (AZI2), mRNA	NM_022461	Hs.700605	BX648471
ASTN1	5.6	3.1	astrotactin 1 (ASTN1), transcript variant 1, mRNA	NM_004319	Hs.495897	NM_004319
THC2524584	5.6	3.7	Q9N083_MACFA (Q9N083) Unnamed portein product, partial (29%)	THC2524584	Unknown	
CR611098	5.6	1.8	full-length cDNA clone CS0DI060YD22 of Placenta Cot 25-normalized of (human).	CR611098	Hs.544586	AK095117
LOC153222	5.6	3.5	adult retina protein (LOC153222), mRNA	NM_153607	Hs.484195	AY139008
LYPD1	5.6	5.1	LY6/PLAUR domain containing 1 (LYPD1), transcript variant 1, mRNA	NM_144586	Hs.694844	NM_144586
ZNF800	5.6	2.7	zinc finger protein 800 (ZNF800), mRNA	NM_176814	Hs.159006	NM_176814

GOPC	5.6	2.5	golgi associated PDZ and coiled-coil motif containing (GOPC), transcript variant 1, mRNA	NM_020399	Hs.191539	AB209385
BRWD1	5.6	2.4	Bromodomain and WD repeat domain-containing protein 1 (WD repeat protein 9).	ENST00000380831	Unknown	
CXCL12	5.6	3.6	chemokine (C-X-C motif) ligand 12 (stromal cell-derived factor 1) (CXCL12), transcript variant 1, mRNA	NM_199168	Hs.522891	AK090482
ZNF268	5.6	1.6	H.sapiens HZF3 mRNA for zinc finger protein.	X78926	Hs.654533	BC142989
GLS	5.6	4.7	glutaminase (GLS), mRNA	NM_014905	Hs.116448	CR749593
SDCCAG8	5.6	6.8	serologically defined colon cancer antigen 8 (SDCCAG8), mRNA	NM_006642	Hs.591530	BX537630
FBXO34	5.5	1.5	F-box protein 34 (FBXO34), mRNA	NM_017943	Hs.525348	NM_017943
AQP1	5.5	4.8	aquaporin 1 (Colton blood group) (AQP1), mRNA	NM_198098	Hs.76152	NM_198098
MGC4093	5.5	2.2	hypothetical protein MGC4093 (MGC4093), mRNA	NM_030578	Hs.567596	BQ900587
FLJ10781	5.5	2.1	hypothetical protein FLJ10781 (FLJ10781), mRNA	NM_018215	Hs.8395	BC032508
RBP4	5.5	3.8	retinol binding protein 4, plasma (RBP4), mRNA	NM_006744	Hs.50223	BQ064708
SDCBP	5.5	2.0	syndecan binding protein (syntenin) (SDCBP), transcript variant 1, mRNA	NM_005625	Hs.200804	AK128645
ZNF436	5.5	17.5	zinc finger protein 436 (ZNF436), transcript variant 1, mRNA	NM_001077195	Hs.293798	NM_001077195
MTCH2	5.5	2.2	mitochondrial carrier homolog 2 (C. elegans) (MTCH2), nuclear gene encoding mitochondrial protein, mRNA	NM_014342	Hs.269944	AY380792
LOC399959	5.5	80.1	cDNA FLJ11490 fis, clone HEMBA1001918.	AK021552	Hs.44098	AB075498
C11orf9	5.5	6.7	chromosome 11 open reading frame 9 (C11orf9), mRNA	NM_013279	Hs.473109	AB023171
THC2519126	5.5	2.1	THC2519126	THC2519126	Unknown	
CTDSP2	5.5	2.1	CTD (carboxy-terminal domain, RNA polymerase II, polypeptide A) small phosphatase 2 (CTDSP2), mRNA	NM_005730	Hs.524530	NM_005730
PRMT2	5.5	1.8	protein arginine methyltransferase 2 (PRMT2), transcript variant 1, mRNA	NM_206962	Hs.154163	AK123352
KIAA1009	5.5	3.2	KIAA1009 (KIAA1009), mRNA	NM_014895	Hs.485865	NM_014895
GPR137B	5.5	2.7	G protein-coupled receptor 137B (GPR137B), mRNA	NM_003272	Hs.498160	AF027826
ZNF704	5.5	3.1	zinc finger protein 704 (ZNF704), mRNA	NM_001033723	Hs.632067	AK131274
GSTM1	5.5	4.3	glutathione S-transferase M1 (GSTM1), transcript variant 2, mRNA	NM_146421	Hs.301961	BQ880398
LRRC31	5.5	3.0	leucine rich repeat containing 31 (LRRC31), mRNA	NM_024727	Hs.411295	NM_024727
ZIC4	5.5	5.4	Zic family member 4 (ZIC4), mRNA	NM_032153	Hs.415766	AK124600
RPL9	5.5	1.6	ribosomal protein L9 (RPL9), transcript variant 2, mRNA	NM_001024921	Hs.412370	CR595992
PRR16	5.5	9.7	proline rich 16 (PRR16), mRNA	NM_016644	Hs.157461	AF242769
S100P	5.5	4.3	S100 calcium binding protein P (S100P), mRNA	NM_005980	Hs.2962	BG571732
CCDC126	5.5	2.6	coiled-coil domain containing 126 (CCDC126), mRNA	NM_138771	Hs.232296	AY358713
ITGA9	5.5	2.2	integrin, alpha 9 (ITGA9), mRNA	NM_002207	Hs.113157	BX647350
MPDZ	5.5	1.9	multiple PDZ domain protein (MPDZ), mRNA	NM_003829	Hs.169378	AB210041
IQCE	5.5	3.4	mRNA for KIAA1023 protein, partial cds.	AB028946	Unknown	
MTTP	5.5	6.4	microsomal triglyceride transfer protein (MTTP), mRNA	NM_000253	Hs.195799	NM_000253
BE004814	5.5	9.8	BE004814 MR2-BN0114-020500-006-e07 BN0114 cDNA, mRNA sequence	BE004814	Unknown	
THC2656116	5.5	6.4	THC2656116	THC2656116	Unknown	
HOXA3	5.5	197.6	homeobox A3 (HOXA3), transcript variant 2, mRNA	NM_153631	Hs.659337	NM_153631
MBOAT2	5.5	2.2	membrane bound O-acyltransferase domain containing 2 (MBOAT2), mRNA	NM_138799	Hs.467634	NM_138799
C11orf74	5.5	1.9	chromosome 11 open reading frame 74 (C11orf74), mRNA	NM_138787	Hs.406726	AK095997
THC2632286	5.5	2.1	AA665072 nu76b01.s1 NCI_CGAP_Alv1 cDNA clone IMAGE:1216585, mRNA sequence	THC2632286	Unknown	
ROR2	5.5	3.7	receptor tyrosine kinase-like orphan receptor 2 (ROR2), mRNA	NM_004560	Hs.98255	AB209154
KCNE1	5.5	8.2	potassium voltage-gated channel, Isk-related family, member 1 (KCNE1), mRNA	NM_000219	Hs.121495	NM_000219
C10orf6	5.5	2.7	chromosome 10 open reading frame 6 (C10orf6), mRNA	NM_018121	Hs.447458	NM_018121
MBTPS1	5.5	1.7	membrane-bound transcription factor peptidase, site 1 (MBTPS1), transcript variant 1, mRNA	NM_003791	Hs.75890	NM_003791
CPM	5.5	5.8	carboxypeptidase M (CPM), transcript variant 1, mRNA	NM_001874	Hs.654387	NM_001874
CPAMD8	5.5	3.1	C3 and PZP-like, alpha-2-macroglobulin domain containing 8 (CPAMD8), mRNA	NM_015692	Hs.631644	NM_015692
RIN2	5.5	2.8	Ras and Rab interactor 2 (RIN2), mRNA	NM_018993	Hs.472270	NM_018993
THC2528386	5.5	11.0	Q9LQ44_ARATH (Q9LQ44) T30E16.29, partial (8%)	THC2528386	Unknown	
ENST00000378240	5.5	4.4	Meiosis expressed gene 1 homolog (Mouse).	ENST00000378240	Unknown	
FAM101A	5.5	3.1	family with sequence similarity 101, member A (FAM101A), mRNA	NM_181709	Hs.432901	NM_181709
SLC17A8	5.5	6.9	solute carrier family 17 (sodium-dependent inorganic phosphate cotransporter), member 8 (SLC17A8), mRNA	NM_139319	Hs.116871	AK128319
GSTA5	5.5	5.9	glutathione S-transferase A5 (GSTA5), mRNA	NM_153699	Hs.553652	NM_153699

GPSM1	5.5	3.5	G-protein signalling modulator 1 (AGS3-like, C. elegans), mRNA (cDNA clone IMAGE:5242824), complete cds.	BC048343	Hs.239370	AL117478
BI916497	5.5	3.6	603182667F1 NIH_MGC_121 cDNA clone IMAGE:5246609 5', mRNA sequence	BI916497	Hs.514795	NM_003787
ATRNL1	5.5	5.1	attractin-like 1 (ATRNL1), mRNA	NM_207303	Hs.501127	NM_207303
HS3ST3A1	5.5	2.8	heparan sulfate (glucosamine) 3-O-sulfotransferase 3A1 (HS3ST3A1), mRNA	NM_006042	Hs.462270	NM_006042
THC2611661	5.5	2.9	RR12_SPIMX (P42344) Chloroplast 30S ribosomal protein S12, partial (11%)	THC2611661	Unknown	
THC2505214	5.5	5.4	THC2505214	THC2505214	Unknown	
BMP7	5.5	2.3	Bone morphogenetic protein 7 precursor (BMP-7) (Osteogenic protein 1) (OP-1) (Eptotermin alfa).	ENST00000371291	Unknown	
SORBS2	5.5	3.5	sorbin and SH3 domain containing 2 (SORBS2), transcript variant 2, mRNA	NM_021069	Hs.655143	NM_021069
GBP4	5.5	11.6	guanylate binding protein 4 (GBP4), mRNA	NM_052941	Hs.409925	AL832576
F5	5.5	3.2	coagulation factor V (proaccelerin, labile factor) (F5), mRNA	NM_000130	Hs.30054	NM_000130
FSTL5	5.5	7.0	follistatin-like 5 (FSTL5), mRNA	NM_020116	Hs.591707	NM_020116
PEX3	5.5	1.7	peroxisomal biogenesis factor 3 (PEX3), mRNA	NM_003630	Hs.7277	BX648803
AF136408	5.5	5.5	unknown mRNA.	AF136408	Unknown	
GZMA	5.5	6.1	granzyme A (granzyme 1, cytotoxic T-lymphocyte-associated serine esterase 3) (GZMA), mRNA	NM_006144	Hs.90708	BM917804
CCNL1	5.5	1.7	cyclin L1 (CCNL1), mRNA	NM_020307	Hs.4859	BX641146
DDX26B	5.5	5.6	DEAD/H (Asp-Glu-Ala-Asp/His) box polypeptide 26B (DDX26B), mRNA	NM_182540	Hs.496829	BX648113
KIAA1443	5.5	1.9	KIAA1443 (KIAA1443), mRNA	NM_020834	Hs.632332	NM_020834
VAPA	5.5	1.7	VAMP (vesicle-associated membrane protein)-associated protein A, 33kDa (VAPA), transcript variant 1, mRNA	NM_003574	Hs.165195	NM_003574
ELF4	5.5	6.7	E74-like factor 4 (ets domain transcription factor) (ELF4), mRNA	NM_001421	Hs.271940	U32645
FAM107B	5.5	2.3	family with sequence similarity 107, member B (FAM107B), mRNA	NM_031453	Hs.446315	AK127413
GSTM1	5.5	3.9	glutathione S-transferase M1 (GSTM1), transcript variant 2, mRNA	NM_146421	Hs.301961	BQ880398
C6orf199	5.5	2.9	chromosome 6 open reading frame 199 (C6orf199), mRNA	NM_145025	Hs.486169	AL832162
ATP9A	5.5	2.4	ATPase, Class II, type 9A (ATP9A), mRNA	NM_006045	Hs.700629	NM_006045
TAOK3	5.5	2.0	TAO kinase 3 (TAOK3), mRNA	NM_016281	Hs.644420	NM_016281
CA414006	5.5	3.4	CA414006 UI-H-EZ0-ban-f-19-0-UI.s1 NCI_CGAP_Ch1 cDNA clone UI-H-EZ0-ban-f-19-0-UI 3', mRNA sequence	CA414006	Hs.699396	NM_001031804
STCH	5.5	2.1	stress 70 protein chaperone, microsome-associated, 60kDa (STCH), mRNA	NM_006948	Hs.352341	NM_006948
LOC643932	5.5	1.6	PREDICTED: similar to 40S ribosomal protein S3a (V-fos transformation effector protein) (LOC643932), mRNA	XR_017289	Hs.631966	BM804554
LOC56757	5.5	3.3	cDNA: FLJ23595 fis, clone LNG15262.	AK027248	Hs.592566	AL833381
ZNF292	5.5	1.8	Zinc finger protein 292.	ENST00000339907	Unknown	
HISPPD1	5.4	1.9	histidine acid phosphatase domain containing 1 (HISPPD1), mRNA	NM_015216	Hs.212046	AB007893
FAM118A	5.4	2.7	family with sequence similarity 118, member A (FAM118A), mRNA	NM_017911	Hs.265018	AK125027
LOC285535	5.4	3.9	cDNA FLJ33738 fis, clone BRAWH2018527.	AK091057	Hs.400256	AK091057
AQP1	5.4	4.5	aquaporin 1 (Colton blood group) (AQP1), mRNA	NM_198098	Hs.76152	NM_198098
MBNL3	5.4	6.9	cDNA FLJ38120 fis, clone D3OST3000195.	AK095439	Hs.641091	AK095439
SOX8	5.4	2.6	SRY (sex determining region Y)-box 8 (SOX8), mRNA	NM_014587	Hs.243678	NM_014587
GSTM1	5.4	4.5	glutathione S-transferase M1 (GSTM1), transcript variant 2, mRNA	NM_146421	Hs.301961	BQ880398
THC2586367	5.4	1.9	Q9D7P1_MOUSE (Q9D7P1) Adult male tongue cDNA, RIKEN full-length enriched library, clone:2300009C10 product:ribosomal protein S24, full insert sequence, partial (95%)	THC2586367	Unknown	
C13orf21	5.4	3.3	chromosome 13 open reading frame 21 (C13orf21), mRNA	NM_001010897	Hs.377972	AK123212
TM4SF4	5.4	77.4	transmembrane 4 L six family member 4 (TM4SF4), mRNA	NM_004617	Hs.133527	AK055577
CRYL1	5.4	6.0	crystallin, lambda 1 (CRYL1), mRNA	NM_015974	Hs.370703	BC071810
FGF7	5.4	7.2	fibroblast growth factor 7 (keratinocyte growth factor) (FGF7), mRNA	NM_002009	Hs.567268	NM_002009
VPS24	5.4	2.3	vacuolar protein sorting 24 homolog (S. cerevisiae) (VPS24), transcript variant 1, mRNA	NM_016079	Hs.591582	NM_016079
C22orf32	5.4	1.7	chromosome 22 open reading frame 32 (C22orf32), mRNA	NM_033318	Hs.306083	AK095636
EFNA5	5.4	2.1	Ephrin-A5 precursor (EPH-related receptor tyrosine kinase ligand 7) (LERK-7) (AL-1).	ENST00000379748	Unknown	
NAALAD2	5.4	4.1	N-acetylated alpha-linked acidic dipeptidase 2 (NAALAD2), mRNA	NM_005467	Hs.503560	AK075390
TSGA2	5.4	4.4	testis specific A2 homolog (mouse) (TSGA2), mRNA	NM_080860	Hs.661069	AK057315
ZDHHC17	5.4	1.8	zinc finger, DHHC-type containing 17 (ZDHHC17), mRNA	NM_015336	Hs.4014	AB024494
C1orf133	5.4	3.5	Novel protein.	ENST00000367013	Unknown	
IRS2	5.4	1.9	insulin receptor substrate 2 (IRS2), mRNA	NM_003749	Hs.442344	NM_003749
GDF6	5.4	31.3	growth differentiation factor 6 (GDF6), mRNA	NM_001001557	Hs.492277	AJ537424
HTATSF1	5.4	1.8	HIV-1 Tat specific factor 1 (HTATSF1), mRNA	NM_014500	Hs.204475	AB209303

MDS1	5.4	8.4	myelodysplasia syndrome 1 (MDS1), mRNA	NM_004991	Hs.659873	U43293
KIAA0895	5.4	12.4	KIAA0895 protein (KIAA0895), mRNA	NM_015314	Hs.6224	NM_015314
WDR47	5.4	4.0	WD repeat domain 47 (WDR47), mRNA	NM_014969	Hs.654760	BC034964
THC2657587	5.4	14.4	Q3MWI9_9DELT (Q3MWI9) Fumarate reductase flavoprotein subunit precursor, partial (11%)	THC2657587	Unknown	
PPAP2B	5.4	4.0	phosphatidic acid phosphatase type 2B (PPAP2B), transcript variant 1, mRNA	NM_003713	Hs.696099	NM_003713
FOXN4	5.4	4.0	forkhead box N4 (FOXN4), mRNA	NM_213596	Hs.528316	BC146825
FLJ42875	5.4	2.7	FLJ42875 protein, mRNA (cDNA clone MGC:35434 IMAGE:5190167), complete cds.	BC029785	Hs.531041	AL832943
ENST00000324808	5.4	1.8	coiled-coil domain containing 57	ENST00000324808	Unknown	
FKBP9	5.4	3.2	FK506 binding protein 9, 63 kDa (FKBP9), mRNA	NM_007270	Hs.103934	AB209352
TMPRSS5	5.4	2.8	transmembrane protease, serine 5 (spinesin) (TMPRSS5), mRNA	NM_030770	Hs.46720	AF495727
CR601823	5.4	5.4	full-length cDNA clone CS0DJ013YF20 of T cells (Jurkat cell line) Cot 10-normalized of (human).	CR601823	Hs.648369	AL512747
FBS1	5.4	2.9	fibrosin 1 (FBS1), mRNA	NM_022452	Unknown	
FEZ2	5.4	2.9	fasciculation and elongation protein zeta 2 (zygin II) (FEZ2), transcript variant 2, mRNA	NM_001042548	Hs.258563	NM_001042548
TTC30A	5.4	2.1	tetratricopeptide repeat domain 30A (TTC30A), mRNA	NM_152275	Hs.128384	NM_152275
MUT	5.4	1.5	methylmalonyl Coenzyme A mutase (MUT), nuclear gene encoding mitochondrial protein, mRNA	NM_000255	Hs.485527	NM_000255
ZBED3	5.4	2.6	zinc finger, BED-type containing 3 (ZBED3), mRNA	NM_032367	Hs.584988	BU570509
GABARAP	5.4	1.9	GABA(A) receptor-associated protein (GABARAP), mRNA	NM_007278	Hs.647421	BM803698
PGAP1	5.4	2.3	GPI deacylase (PGAP1), mRNA	NM_024989	Hs.229988	NM_024989
CCDC102B	5.4	5.4	coiled-coil domain containing 102B (CCDC102B), mRNA	NM_024781	Hs.280781	NM_001093729
POPDC3	5.4	1.9	popeye domain containing 3 (POPDC3), mRNA	NM_022361	Hs.458336	NM_022361
THC2525827	5.4	2.1	THC2525827	THC2525827	Unknown	
BX419129	5.4	1.9	BX419129 FETAL BRAIN cDNA clone CS0DF013YC22 5-PRIME, mRNA sequence	BX419129	Hs.595920	BX419129
KREMEN1	5.4	2.7	kringle containing transmembrane protein 1 (KREMEN1), transcript variant 3, mRNA	NM_001039570	Hs.229335	NM_001039570
WIT1	5.4	15.2	Wilms tumor upstream neighbor 1 (WIT1), mRNA	NM_015855	Hs.567499	M60614
GLIS3	5.4	7.7	GLIS family zinc finger 3 (GLIS3), transcript variant 1, mRNA	NM_001042413	Hs.162125	NM_001042413
AFP	5.4	40.2	alpha-fetoprotein (AFP), mRNA	NM_001134	Hs.518808	BC027881
ABHD14A	5.4	1.9	abhydrolase domain containing 14A (ABHD14A), mRNA	NM_015407	Hs.534400	BM466791
RBBP9	5.4	2.0	retinoblastoma binding protein 9 (RBBP9), mRNA	NM_006606	Hs.69330	BC015938
CR610205	5.4	4.9	full-length cDNA clone CS0DC020YH17 of Neuroblastoma Cot 25-normalized of (human).	CR610205	Hs.657100	BG035888
C1orf131	5.4	1.9	chromosome 1 open reading frame 131 (C1orf131), mRNA	NM_152379	Hs.556017	AK055124
HES1	5.4	3.5	hairy and enhancer of split 1, (Drosophila) (HES1), mRNA	NM_005524	Hs.250666	NM_005524
IRS1	5.4	2.0	insulin receptor substrate 1 (IRS1), mRNA	NM_005544	Hs.471508	NM_005544
C20orf96	5.4	2.4	chromosome 20 open reading frame 96 (C20orf96), mRNA	NM_153269	Hs.348112	AK126082
C14orf128	5.4	2.1	chromosome 14 open reading frame 128, mRNA (cDNA clone MGC:15504 IMAGE:2990071), complete cds.	BC007251	Hs.496755	BC007251
MTHFR	5.4	2.7	5,10-methylenetetrahydrofolate reductase (NADPH) (MTHFR), mRNA	NM_005957	Hs.214142	NM_005957
SAMD13	5.4	3.1	sterile alpha motif domain containing 13 (SAMD13), mRNA	NM_001010971	Hs.591445	NM_001010971
CCDC130	5.4	1.6	coiled-coil domain containing 130 (CCDC130), mRNA	NM_030818	Hs.24998	CR620805
FOXL1	5.4	2.2	forkhead box L1 (FOXL1), mRNA	NM_005250	Hs.533830	NM_005250
CRABP2	5.4	2.5	cellular retinoic acid binding protein 2 (CRABP2), mRNA	NM_001878	Hs.405662	BI194589
RDH5	5.4	1.8	retinol dehydrogenase 5 (11-cis/9-cis) (RDH5), mRNA	NM_002905	Hs.632719	BC028298
KNG1	5.4	6.1	kininogen 1 (KNG1), mRNA	NM_000893	Hs.77741	NM_000893
ST3GAL5	5.4	2.4	ST3 beta-galactoside alpha-2,3-sialyltransferase 5 (ST3GAL5), transcript variant 1, mRNA	NM_003896	Hs.415117	AK127346
C14orf135	5.4	1.9	chromosome 14 open reading frame 135 (C14orf135), mRNA	NM_022495	Hs.509499	NM_022495
PSCD1	5.4	2.1	pleckstrin homology, Sec7 and coiled-coil domains 1(cytohesin 1) (PSCD1), transcript variant 1, mRNA	NM_004762	Hs.191215	AK123894
XPR1	5.4	3.4	xenotropic and polytropic retrovirus receptor	ENST00000367590	Unknown	
PCMTD2	5.4	1.9	protein-L-isoaspartate (D-aspartate) O-methyltransferase domain containing 2 (PCMTD2), mRNA	NM_018257	Hs.473317	AK001745
MDM1	5.4	2.0	Mdm4, transformed 3T3 cell double minute 1, p53 binding protein (mouse) (MDM1), transcript variant 1, mRNA	NM_017440	Hs.655702	BC028355
CR613436	5.4	12.1	full-length cDNA clone CS0DF024YN04 of Fetal brain of (human).	CR613436	Hs.656886	BX538139
GPR161	5.4	6.9	cDNA FLJ33952 fis, clone CTONG2018614.	AK091271	Hs.271809	AK095302
SLC16A12	5.4	2.1	cDNA FLJ42911 fis, clone BRHIP3024118, weakly similar to Monocarboxylate transporter 4.	AK124901	Hs.530338	CR936657

LOC285398	5.4	1.7	cDNA FLJ25945 fis, clone JTH12731.	AK098811	Hs.681813	AK098811
ZNF711	5.4	2.1	zinc finger protein 711 (ZNF711), mRNA	NM_021998	Hs.326801	BX648117
PPP2R5C	5.4	1.7	protein phosphatase 2, regulatory subunit B (B56), gamma isoform (PPP2R5C), transcript variant 1, mRNA	NM_002719	Hs.368264	NM_002719
AK130514	5.4	2.7	cDNA FLJ27004 fis, clone SLV04966.	AK130514	Hs.699296	AL833852
LOC283904	5.4	3.7	full-length cDNA clone CS0DK001YK13 of HeLa cells Cot 25-normalized of (human).	CR605298	Hs.676511	CR605298
CD174733	5.4	1.8	CD174733 AGENCOURT_13961604 NIH_MGC_172 cDNA 5', mRNA sequence	CD174733	Hs.265174	CR596982
NBPF20	5.4	3.6	neuroblastoma breakpoint family, member 20 (NBPF20), mRNA	NM_001037675	Hs.515947	BX538005
ADARB1	5.3	2.3	adenosine deaminase, RNA-specific, B1 (RED1 homolog rat) (ADARB1), transcript variant 1, mRNA	NM_001112	Hs.474018	AB194370
C6orf70	5.3	2.2	chromosome 6 open reading frame 70 (C6orf70), mRNA	NM_018341	Hs.47546	NM_018341
BU686948	5.3	5.1	UI-CF-DU1-ado-e-06-0-UI.s1 UI-CF-DU1 cDNA clone UI-CF-DU1-ado-e-06-0-UI 3', mRNA sequence	BU686948	Hs.638070	BU686948
BM984396	5.3	4.8	BM984396 UI-CF-DU1-abf-e-18-0-UI.s1 UI-CF-DU1 cDNA clone UI-CF-DU1-abf-e-18-0-UI 3', mRNA sequence	BM984396	Hs.652928	BM984396
FLJ20701	5.3	3.9	hypothetical protein FLJ20701 (FLJ20701), mRNA	NM_017933	Hs.409352	NM_017933
CCDC45	5.3	1.5	coiled-coil domain containing 45 (CCDC45), mRNA	NM_138363	Hs.569713	BX641136
ENST00000376347	5.3	1.9	kinesin family member 27	ENST00000376347	Unknown	
SLC26A1	5.3	4.7	solute carrier family 26 (sulfate transporter), member 1 (SLC26A1), transcript variant 2, mRNA	NM_134425	Hs.658244	AY124771
LAMB1	5.3	2.2	laminin, beta 1 (LAMB1), mRNA	NM_002291	Hs.650585	NM_002291
FAM19A2	5.3	5.1	family with sequence similarity 19 (chemokine (C-C motif)-like), member A2 (FAM19A2), mRNA	NM_178539	Hs.269745	NM_178539
THC2724111	5.3	3.4	THC2724111	THC2724111	Unknown	
C1orf165	5.3	2.5	chromosome 1 open reading frame 165 (C1orf165), mRNA	NM_024603	Hs.475348	AK096573
THC2668270	5.3	2.0	Q8I727_TRYCR (Q8I727) TcC31.32, partial (14%)	THC2668270	Unknown	
ZNF187	5.3	1.9	zinc finger protein 187 (ZNF187), transcript variant 1, mRNA	NM_007151	Hs.157883	NM_007151
TTC33	5.3	2.3	tetratricopeptide repeat domain 33 (TTC33), mRNA	NM_012382	Hs.348915	NM_012382
GATA5	5.3	16.3	GATA binding protein 5 (GATA5), mRNA	NM_080473	Hs.352250	BC047790
FBN2	5.3	3.1	fibrillin 2 (congenital contractural arachnodactyly) (FBN2), mRNA	NM_001999	Hs.519294	NM_001999
A_24_P186764	5.3	1.8	A_24_P186764	A_24_P186764	Unknown	
KIAA0776	5.3	1.7	KIAA0776 (KIAA0776), mRNA	NM_015323	Hs.149367	BX538302
GPR162	5.3	2.8	G protein-coupled receptor 162 (GPR162), transcript variant A-2, mRNA	NM_019858	Hs.631654	NM_019858
LOC389240	5.3	1.3	PREDICTED: similar to nascent polypeptide-associated complex alpha polypeptide (LOC389240), mRNA	ENST00000240349	Unknown	
HISPPD1	5.3	1.8	histidine acid phosphatase domain containing 1 (HISPPD1), mRNA	NM_015216	Hs.212046	AB007893
PLAGL2	5.3	1.6	pleiomorphic adenoma gene-like 2 (PLAGL2), mRNA	NM_002657	Hs.154104	NM_002657
THC2673888	5.3	4.9	Q65549_9ALPH (Q65549) Glycoprotein C, partial (4%)	THC2673888	Unknown	
HEATR2	5.3	1.6	HEAT repeat containing 2, mRNA (cDNA clone IMAGE:4213551), **** WARNING: chimeric clone ****.	BC008192	Unknown	
TPT1	5.3	2.8	tumor protein, translationally-controlled 1 (TPT1), mRNA	NM_003295	Hs.374596	BG033621
MBTPS1	5.3	1.7	membrane-bound transcription factor peptidase, site 1 (MBTPS1), transcript variant 1, mRNA	NM_003791	Hs.75890	NM_003791
EN1	5.3	2.6	engrailed homolog 1 (EN1), mRNA	NM_001426	Hs.271977	NM_001426
AK023391	5.3	12.7	cDNA FLJ13329 fis, clone OVARC1001795.	AK023391	Hs.592775	AK125888
ChGn	5.3	9.5	chondroitin beta1,4 N-acetylgalactosaminyltransferase (ChGn), mRNA	NM_018371	Hs.655166	BX649103
CR618523	5.3	2.1	full-length cDNA clone CS0DI011YC23 of Placenta Cot 25-normalized of (human).	CR618523	Hs.585745	NM_001080530
A_24_P50567	5.3	2.6	A_24_P50567	A_24_P50567	Unknown	
RPL23A	5.3	1.6	ribosomal protein L23a (RPL23A), mRNA	NM_000984	Hs.419463	CR616046
CLGN	5.3	2.4	calmegin (CLGN), mRNA	NM_004362	Hs.86368	AK093096
UBE2B	5.3	2.3	ubiquitin-conjugating enzyme E2B (RAD6 homolog) (UBE2B), mRNA	NM_003337	Hs.695927	NM_003337
ZNF655	5.3	1.7	zinc finger protein 655 (ZNF655), transcript variant 6, mRNA	NM_001009956	Unknown	
KLHDC5	5.3	1.4	kelch domain containing 5 (KLHDC5), mRNA	NM_020782	Hs.505104	NM_020782
AMIGO1	5.3	2.9	Amphoterin-induced protein 1 precursor (AMIGO-1) (Alivin-2).	ENST00000369864	Unknown	
THC2613186	5.3	6.6	THC2613186	THC2613186	Unknown	
DISP1	5.3	5.2	dispatched homolog 1 (Drosophila) (DISP1), mRNA	NM_032890	Hs.528817	NM_032890
HUNK	5.3	2.2	hormonally upregulated Neu-associated kinase (HUNK), mRNA	NM_014586	Hs.109437	AJ271722
CD83	5.3	2.4	CD83 molecule (CD83), transcript variant 1, mRNA	NM_004233	Hs.654558	NM_004233
CENTD1	5.3	3.8	centaurin, delta 1 (CENTD1), transcript variant 1, mRNA	NM_015230	Hs.479451	NM_015230
KIAA1799	5.3	6.3	KIAA1799 protein (KIAA1799), mRNA	NM_032437	Hs.652324	AB058702

SEC63	5.3	1.8	SEC63 homolog (S. cerevisiae) (SEC63), mRNA	NM_007214	Hs.529957	NM_007214
BC013423	5.3	3.6	Homo sapiens, clone IMAGE:3892140, mRNA.	BC013423	Hs.659712	BC013423
CEP290	5.3	2.5	centrosomal protein 290kDa (CEP290), mRNA	NM_025114	Hs.150444	NM_025114
SLC10A7	5.3	2.3	cDNA PSEC0051 fis, clone NT2RP2000168.	AK075364	Hs.659209	CR933647
LOC439911	5.3	3.4	cDNA FLJ45542 fis, clone BRTHA2033320.	AK127450	Unknown	
JMJD1A	5.3	3.2	jumonji domain containing 1A (JMJD1A), mRNA	NM_018433	Hs.557425	BX640698
MAGED2	5.3	2.3	melanoma antigen family D, 2 (MAGED2), transcript variant 3, mRNA	NM_201222	Hs.522665	AK092463
IGFBP1	5.3	12.5	insulin-like growth factor binding protein 1 (IGFBP1), transcript variant 1, mRNA	NM_000596	Hs.642938	NM_000596
SDCCAG8	5.3	5.3	serologically defined colon cancer antigen 8 (SDCCAG8), mRNA	NM_006642	Hs.591530	BX537630
BBS10	5.3	1.6	Bardet-Biedl syndrome 10 (BBS10), mRNA	NM_024685	Hs.96322	NM_024685
A_24_P334208	5.3	8.6	A_24_P334208	A_24_P334208	Unknown	
GSTM1	5.3	4.3	glutathione S-transferase M1 (GSTM1), transcript variant 2, mRNA	NM_146421	Hs.301961	BQ880398
HISPPD1	5.3	1.9	histidine acid phosphatase domain containing 1 (HISPPD1), mRNA	NM_015216	Hs.212046	AB007893
MAF	5.3	4.0	short form transcription factor C-MAF (c-maf) mRNA, complete cds.	AF055376	Hs.699396	NM_001031804
RELN	5.3	10.4	reelin (RELN), transcript variant 1, mRNA	NM_005045	Hs.655654	U79716
AK093713	5.3	1.9	cDNA FLJ36394 fis, clone THYMU2009104.	AK093713	Unknown	
KLHDC1	5.3	6.5	kelch domain containing 1 (KLHDC1), mRNA	NM_172193	Hs.509258	AF111806
AK125077	5.3	4.9	cDNA FLJ43087 fis, clone BRTHA3019105.	AK125077	Hs.197962	AK125077
C3orf62	5.3	2.1	chromosome 3 open reading frame 62 (C3orf62), mRNA	NM_198562	Hs.403828	AK125642
KIAA1109	5.3	1.7	KIAA1109, mRNA (cDNA clone IMAGE:3924668), complete cds.	BC108274	Hs.408142	DQ335469
AX721252	5.3	1.5	Sequence 212 from Patent WO0220754.	AX721252	Unknown	
MAPKBP1	5.3	2.4	mitogen activated protein kinase binding protein 1 (MAPKBP1), mRNA	NM_014994	Hs.699259	NM_014994
ALG5	5.3	1.8	asparagine-linked glycosylation 5 homolog (S. cerevisiae, dolichyl-phosphate beta-glucosyltransferase) (ALG5), mRNA	NM_013338	Hs.507769	AK097252
AI652920	5.3	2.9	AI652920 wb40g09.x1 NCI_CGAP_GC6 cDNA clone IMAGE:2308192 3' similar to SW:NMA_HUMAN Q13145 PUTATIVE TRANSMEMBRANE PROTEIN NMA PRECURSOR. ;, mRNA sequence	AI652920	Hs.533336	NM_012342
DNAJB9	5.3	2.0	DnaJ (Hsp40) homolog, subfamily B, member 9 (DNAJB9), mRNA	NM_012328	Hs.6790	AK092204
PPARA	5.3	2.8	peroxisome proliferator-activated receptor alpha (PPARA), transcript variant 5, mRNA	NM_005036	Hs.103110	NM_005036
KIAA1377	5.3	3.9	KIAA1377 (KIAA1377), mRNA	NM_020802	Hs.156352	NM_020802
SSBP4	5.3	1.9	single stranded DNA binding protein 4 (SSBP4), transcript variant 1, mRNA	NM_032627	Hs.515259	NM_032627
PHF6	5.3	1.6	PHD finger protein 6 (PHF6), transcript variant 2, mRNA	NM_032458	Hs.356501	NM_032458
THC2770350	5.3	12.5	ATS18_HUMAN (Q8TE60) ADAMTS-18 precursor (A disintegrin and metalloproteinase with thrombospondin motifs 18) (ADAM-TS 18) (ADAM-TS18) , partial (82%)	THC2770350	Unknown	
WNT10B	5.3	2.3	wingless-type MMTV integration site family, member 10B (WNT10B), mRNA	NM_003394	Hs.91985	U81787
NEXN	5.3	5.0	nexilin (F actin binding protein) (NEXN), mRNA	NM_144573	Hs.632387	NM_144573
FRY	5.3	3.7	furry homolog (Drosophila) (FRY), mRNA	NM_023037	Hs.591225	NM_023037
TSHZ1	5.3	8.9	teashirt family zinc finger 1 (TSHZ1), mRNA	NM_005786	Hs.284217	AB210042
CRTAC1	5.3	3.6	cartilage acidic protein 1 (CRTAC1), mRNA	NM_018058	Hs.500736	NM_018058
A_24_P306968	5.3	1.7	A_24_P306968	A_24_P306968	Unknown	
CXCL12	5.3	3.6	chemokine (C-X-C motif) ligand 12 (stromal cell-derived factor 1) (CXCL12), transcript variant 1, mRNA	NM_199168	Hs.522891	AK090482
GOLGA8A	5.3	2.8	golgi autoantigen, golgin subfamily a, 8A (GOLGA8A), mRNA	NM_181077	Hs.182982	BX648160
SCYL3	5.3	2.1	SCY1-like 3 (S. cerevisiae) (SCYL3), transcript variant 1, mRNA	NM_020423	Hs.435560	BX647352
BBS10	5.3	1.6	Bardet-Biedl syndrome 10 (BBS10), mRNA	NM_024685	Hs.96322	NM_024685
ZNF226	5.3	2.1	zinc finger protein 226 (ZNF226), transcript variant 3, mRNA	NM_001032374	Hs.145956	BX648775
BC045174	5.3	1.8	cDNA clone IMAGE:5273245.	BC045174	Hs.24701	BC045174
SEC63D1	5.3	5.6	SEC63 domain containing 1 (SEC63D1), mRNA	NM_198550	Unknown	
CLUL1	5.3	16.8	clusterin-like 1 (retinal) (CLUL1), transcript variant 1, mRNA	NM_014410	Hs.632357	NM_014410
CPS1	5.3	2.0	carbamoyl-phosphate synthetase 1, mitochondrial (CPS1), mRNA	NM_001875	Hs.149252	NM_001875
SLC6A13	5.3	30.2	solute carrier family 6 (neurotransmitter transporter, GABA), member 13, mRNA (cDNA clone IMAGE:4594185), complete cds.	BC020867	Hs.504398	NM_016615
EGFL8	5.3	1.7	EGF-like-domain, multiple 8 (EGFL8), mRNA	NM_030652	Hs.332138	BC052591
SLC26A10	5.3	2.7	solute carrier family 26, member 10 (SLC26A10), mRNA	NM_133489	Hs.159481	NM_133489
CR620892	5.3	5.0	full-length cDNA clone CS0DL007YK20 of B cells (Ramos cell line) Cot 25-normalized of (human).	CR620892	Hs.584916	AK000271
APC	5.2	2.0	adenomatosis polyposis coli (APC), mRNA	NM_000038	Hs.158932	NM_000038
DAAM2	5.2	2.8	dishevelled associated activator of morphogenesis 2 (DAAM2), mRNA	NM_015345	Hs.652207	AL833083

KIF27	5.2	2.3	kinesin family member 27 (KIF27), mRNA	NM_017576	Hs.697514	AY237536
RPS6KB1	5.2	1.8	ribosomal protein S6 kinase, 70kDa, polypeptide 1 (RPS6KB1), mRNA	NM_003161	Hs.463642	NM_003161
PFDN5	5.2	1.9	prefoldin subunit 5 (PFDN5), transcript variant 1, mRNA	NM_002624	Hs.655327	BM562920
AW327568	5.2	4.5	AW327568 dq04b07.y1 NIH_MGC_2 cDNA clone IMAGE:2846557 3', mRNA sequence	AW327568	Hs.76884	AK225965
AVIL	5.2	4.0	mRNA; cDNA DKFZp779O1812 (from clone DKFZp779O1812).	BX647344	Hs.584854	BX647344
NXPH2	5.2	4.2	neurexophilin 2 (NXPH2), mRNA	NM_007226	Hs.435019	BC101462
FLJ31568	5.2	7.7	FLJ31568 protein (FLJ31568), mRNA	NM_152509	Hs.386693	AK092530
DNAJA1	5.2	2.2	DnaJ homolog subfamily A member 1 (Heat shock 40 kDa protein 4) (DnaJ protein homolog 2) (HSJ-2) (HSDJ).	ENST00000330899	Unknown	
EFNA1	5.2	5.0	ephrin-A1 (EFNA1), transcript variant 1, mRNA	NM_004428	Hs.516664	AK057845
C15orf29	5.2	3.0	chromosome 15 open reading frame 29 (C15orf29), mRNA	NM_024713	Hs.633566	AL136908
OXR1	5.2	2.4	oxidation resistance 1 (OXR1), mRNA	NM_181354	Hs.148778	NM_181354
TMED8	5.2	1.6	Protein TMED8.	ENST00000216468	Unknown	
LOC129530	5.2	4.5	hypothetical protein LOC129530 (LOC129530), mRNA	NM_174898	Hs.164589	AK126514
IRF2BP2	5.2	3.7	interferon regulatory factor 2 binding protein 2 (IRF2BP2), transcript variant 1, mRNA	NM_182972	Hs.350268	NM_182972
CSPG2	5.2	2.0	chondroitin sulfate proteoglycan 2 (versican) (CSPG2), mRNA	NM_004385	Hs.695930	NM_004385
RSPO1	5.2	9.3	R-spondin homolog (Xenopus laevis) (RSPO1), mRNA	NM_001038633	Hs.135015	NM_001038633
LRRC28	5.2	3.1	cDNA FLJ45242 fis, clone BRCOC2006639.	AK127177	Hs.578684	AL833556
C19orf36	5.2	2.1	chromosome 19 open reading frame 36 (C19orf36), transcript variant 1, mRNA	NM_001031735	Hs.424049	BC040964
CORT	5.2	1.9	cortistatin (CORT), mRNA	NM_001302	Hs.412311	NM_198544
C2orf32	5.2	7.0	chromosome 2 open reading frame 32 (C2orf32), mRNA	NM_015463	Hs.212885	BC035125
SCN2B	5.2	3.8	sodium channel, voltage-gated, type II, beta (SCN2B), mRNA	NM_004588	Hs.129783	NM_004588
HISPPD1	5.2	1.9	histidine acid phosphatase domain containing 1 (HISPPD1), mRNA	NM_015216	Hs.212046	AB007893
KITLG	5.2	3.8	KIT ligand (KITLG), transcript variant b, mRNA	NM_000899	Hs.1048	NM_000899
SSPO	5.2	4.0	cDNA FLJ36112 fis, clone TESTI2022023, weakly similar to Bos taurus Reissner's fiber glycoprotein I mRNA.	AK093431	Hs.632022	NM_198455
TSNAXIP1	5.2	4.0	translin-associated factor X interacting protein 1 (TSNAXIP1), mRNA	NM_018430	Hs.632212	BC111018
LOC131873	5.2	11.9	mRNA; cDNA DKFZp667J1615 (from clone DKFZp667J1615).	AL713792	Hs.591282	XM_067585
MTHFR	5.2	2.7	5,10-methylenetetrahydrofolate reductase (NADPH) (MTHFR), mRNA	NM_005957	Hs.214142	NM_005957
LOC730999	5.2	4.7	PREDICTED: hypothetical protein LOC730999 (LOC730999), mRNA	XM_001131389	Unknown	
SPAG17	5.2	4.9	sperm associated antigen 17 (SPAG17), mRNA	NM_206996	Hs.528821	AY555274
KLB	5.2	10.2	Beta klotho (BetaKlotho) (Klotho beta-like protein).	ENST00000381889	Unknown	
UCP3	5.2	3.5	uncoupling protein 3 (mitochondrial, proton carrier) (UCP3), nuclear gene encoding mitochondrial protein, transcript variant long, mRNA	NM_003356	Hs.101337	NM_003356
MAP6	5.2	2.5	microtubule-associated protein 6 (MAP6), transcript variant 1, mRNA	NM_033063	Hs.585540	BC150254
ZC3H11A	5.2	1.9	zinc finger CCCH-type containing 11A (ZC3H11A), mRNA	NM_014827	Hs.532399	CR627439
IFT80	5.2	1.7	intraflagellar transport 80 homolog (Chlamydomonas) (IFT80), mRNA	NM_020800	Hs.478095	NM_020800
LRP12	5.2	2.6	low density lipoprotein-related protein 12 (LRP12), mRNA	NM_013437	Hs.654804	CR749434
GSTM1	5.2	4.5	glutathione S-transferase M1 (GSTM1), transcript variant 2, mRNA	NM_146421	Hs.301961	BQ880398
C1orf78	5.2	3.0	chromosome 1 open reading frame 78 (C1orf78), mRNA	NM_018166	Hs.87016	BM911450
ZNF452	5.2	2.4	zinc finger protein 452 (ZNF452), mRNA	NM_052923	Hs.176980	AY517631
RP1-32F7.2	5.2	2.1	hypothetical protein FLJ37659 (RP1-32F7.2), mRNA	NM_173698	Hs.110069	NM_173698
EFHA1	5.2	1.7	EF-hand domain family, member A1 (EFHA1), mRNA	NM_152726	Hs.412103	BX641028
ZNF650	5.2	2.1	zinc finger protein 650 (ZNF650), mRNA	NM_172070	Hs.379548	BC044060
RAB2	5.2	2.1	mRNA; cDNA DKFZp434P231 (from clone DKFZp434P231).	AL137321	Hs.369017	AL137321
MBOAT2	5.2	2.2	O-acyltransferase (membrane bound) domain containing 2	ENST00000354442	Unknown	
PARP11	5.2	2.3	poly (ADP-ribose) polymerase family, member 11 (PARP11), mRNA	NM_020367	Hs.657268	CR749294
PLA2G4A	5.2	3.0	phospholipase A2, group IVA (cytosolic, calcium-dependent) (PLA2G4A), mRNA	NM_024420	Hs.497200	NM_024420
FLJ20152	5.2	2.4	hypothetical protein FLJ20152 (FLJ20152), transcript variant 1, mRNA	NM_001034850	Hs.481704	NM_001034850
C20orf133	5.2	3.5	chromosome 20 open reading frame 133 (C20orf133), transcript variant 1, mRNA	NM_080676	Hs.661576	NM_080676
AF086017	5.2	3.2	full length insert cDNA clone YW19E12.	AF086017	Hs.567828	BX538174
VASH1	5.2	3.0	vasohibin 1 (VASH1), mRNA	NM_014909	Hs.525479	AL832588
RPS15A	5.2	1.6	ribosomal protein S15a (RPS15A), transcript variant 2, mRNA	NM_001019	Hs.370504	BM907705
KIAA1958	5.2	2.4	KIAA1958 (KIAA1958), mRNA	ENST00000337530	Unknown	
THC2547195	5.2	3.1	O60448_HUMAN (O60448) Neuronal thread protein AD7c-NTP, partial (13%)	THC2547195	Unknown	

BX103476	5.2	5.1	BX103476 NCI_CGAP_Lu5 cDNA clone IMAGp998C053946, mRNA sequence	BX103476	Hs.553068	BX103476
CFC1	5.2	10.0	cripto, FRL-1, cryptic family 1 (CFC1), mRNA	NM_032545	Hs.567542	NM_032545
LOC55565	5.2	1.9	hypothetical protein LOC55565 (LOC55565), mRNA	NM_017530	Hs.643531	BC004563
SEC31A	5.2	1.9	SEC31 homolog A (S. cerevisiae) (SEC31A), transcript variant 5, mRNA	NM_001077207	Hs.370024	AK128047
TRIM55	5.2	16.4	tripartite motif-containing 55 (TRIM55), transcript variant 3, mRNA	NM_184086	Hs.85524	NM_033058
AV702101	5.2	2.1	AV702101 ADB cDNA clone ADBCGB06 5', mRNA sequence	AV702101	Hs.97579	AV702101
SHOX2	5.2	3.2	short stature homeobox 2 (SHOX2), transcript variant SHOX2a, mRNA	NM_006884	Hs.55967	NM_006884
CBFB	5.2	2.8	core-binding factor, beta subunit (CBFB), transcript variant 2, mRNA	NM_001755	Hs.460988	NM_001755
AF085351	5.2	1.9	ELISC-1 mRNA, partial cds.	AF085351	Hs.128434	CD358456
PIN4	5.2	1.9	protein (peptidylprolyl cis/trans isomerase) NIMA-interacting, 4 (parvulin) (PIN4), mRNA	NM_006223	Hs.655623	AK127605
NBPF14	5.2	3.6	neuroblastoma breakpoint family, member 14 (NBPF14), mRNA	NM_015383	Hs.607640	AK095459
C14orf10	5.2	1.5	chromosome 14 open reading frame 10 (C14orf10), mRNA	NM_017917	Hs.530712	CR602202
ASGR1	5.2	2.2	asialoglycoprotein receptor 1 (ASGR1), mRNA	NM_001671	Hs.12056	AK124286
SLC1A3	5.2	2.6	solute carrier family 1 (glial high affinity glutamate transporter), member 3 (SLC1A3), mRNA	NM_004172	Hs.481918	NM_004172
BC037919	5.2	2.0	cDNA clone IMAGE:5278089.	BC037919	Hs.113418	BC037919
SLBP	5.2	1.5	stem-loop (histone) binding protein (SLBP), mRNA	NM_006527	Hs.298345	AK094257
C6orf134	5.2	2.5	chromosome 6 open reading frame 134 (C6orf134), transcript variant 2, mRNA	NM_024909	Hs.654798	BC006105
SH3TC2	5.2	4.4	SH3 domain and tetratricopeptide repeats 2 (SH3TC2), mRNA	NM_024577	Hs.483784	NM_024577
LOC647502	5.2	5.9	PREDICTED: similar to angiogenic factor VG5Q (LOC647502), mRNA	XR_018142	Hs.693329	XR_018142
AF086092	5.2	8.1	full length insert cDNA clone YZ87G11.	AF086092	Hs.48372	AF086092
ENST00000381298	5.2	15.6	Interleukin-6 receptor subunit beta precursor (IL-6R-beta) (Interleukin-6 signal transducer) (Membrane glycoprotein 130) (gp130) (Oncostatin-M receptor alpha subunit) (CD130 antigen) (CDw130).	ENST00000381298	Unknown	
RFK	5.2	2.4	riboflavin kinase (RFK), mRNA	NM_018339	Hs.37558	NM_018339
A_24_P204454	5.2	1.9	A_24_P204454	A_24_P204454	Unknown	
LOC729449	5.2	1.6	PREDICTED: similar to 60S ribosomal protein L7 (LOC729449), mRNA	XR_015548	Hs.534938	XR_015548
HNRPA1	5.2	1.4	heterogeneous nuclear ribonucleoprotein A1 (HNRPA1), transcript variant 2, mRNA	NM_031157	Hs.699190	NM_031157
AGTR2	5.2	18.7	angiotensin II receptor, type 2 (AGTR2), mRNA	NM_000686	Hs.405348	NM_000686
VPS26A	5.2	1.6	vacuolar protein sorting 26 homolog A (yeast) (VPS26A), transcript variant 1, mRNA	NM_004896	Hs.499925	BX647725
THC2658816	5.2	6.9	THC2658816	THC2658816	Unknown	
CYB5R1	5.2	2.5	cytochrome b5 reductase 1 (CYB5R1), mRNA	NM_016243	Hs.334832	AK123705
KLHL8	5.2	1.9	kelch-like 8 (Drosophila) (KLHL8), mRNA	NM_020803	Hs.696012	BX640744
FKSG2	5.2	2.6	apoptosis inhibitor (FKSG2), mRNA	NM_021631	Hs.651853	AF300871
C9orf3	5.2	2.5	chromosome 9 open reading frame 3 (C9orf3), mRNA	NM_032823	Hs.434253	AF043897
RND3	5.1	2.3	Rho family GTPase 3 (RND3), mRNA	NM_005168	Hs.6838	X97758
RERG	5.1	2.8	RAS-like, estrogen-regulated, growth inhibitor (RERG), mRNA	NM_032918	Hs.199487	BC007997
A_24_P50057	5.1	2.7	A_24_P50057	A_24_P50057	Unknown	
FLJ11235	5.1	3.6	cDNA FLJ11235 fis, clone PLACE1008488.	AK002097	Hs.591264	AK002097
LOC641790	5.1	1.5	PREDICTED: similar to ribosomal protein L31 (LOC641790), mRNA	XR_018025	Hs.647529	XR_018025
LOC730259	5.1	2.7	PREDICTED: hypothetical protein LOC730259 (LOC730259), mRNA	XM_001133381	Hs.652173	XM_001133381
ZNF706	5.1	2.4	zinc finger protein 706 (ZNF706), transcript variant 1, mRNA	NM_001042510	Hs.374485	NM_001042510
RPL23AP7	5.1	1.5	ribosomal protein L23a pseudogene 7 (RPL23AP7) on chromosome 2	NR_000029	Unknown	
HISPPD1	5.1	1.9	histidine acid phosphatase domain containing 1 (HISPPD1), mRNA	NM_015216	Hs.212046	AB007893
HIST2H2AA4	5.1	6.4	histone cluster 2, H2aa4 (HIST2H2AA4), mRNA	NM_001040874	Hs.701937	NM_001040874
RP11-50D16.3	5.1	1.9	full-length cDNA clone CS0DC001YJ02 of Neuroblastoma Cot 25-normalized of (human).	CR601315	Hs.507783	NM_001012754
RNF152	5.1	3.1	ring finger protein 152 (RNF152), mRNA	NM_173557	Hs.667457	AK122758
SSU72	5.1	2.3	cDNA FLJ13048 fis, clone NT2RP3001399, weakly similar to SSU72 PROTEIN.	AK023110	Hs.657061	AK023110
TFAP2B	5.1	12.0	transcription factor AP-2 beta (activating enhancer binding protein 2 beta) (TFAP2B), mRNA	NM_003221	Hs.33102	NM_003221
A_32_P201616	5.1	5.6	A_32_P201616	A_32_P201616	Unknown	
KIAA1984	5.1	2.1	mRNA for KIAA1984 protein.	AB075864	Hs.370555	AB075864
THC2684051	5.1	12.1	THC2684051	THC2684051	Unknown	
MAP2	5.1	3.8	microtubule-associated protein 2 (MAP2), transcript variant 1, mRNA	NM_002374	Hs.368281	NM_002374
LY6H	5.1	6.3	lymphocyte antigen 6 complex, locus H (LY6H), mRNA	NM_002347	Hs.159590	BC053355
KIAA1841	5.1	2.3	KIAA1841, mRNA (cDNA clone MGC:39557 IMAGE:5314819), complete cds.	BC039298	Hs.468653	AL833595

DHX40	5.1	2.3	DEAH (Asp-Glu-Ala-His) box polypeptide 40 (DHX40), mRNA	NM_024612	Hs.29403	AF260270
SNAI1	5.1	2.7	snail homolog 1 (Drosophila) (SNAI1), mRNA	NM_005985	Hs.48029	NM_005985
ZFP1	5.1	1.9	zinc finger protein 1 homolog (mouse) (ZFP1), mRNA	NM_153688	Hs.388813	AL133038
FCRLB	5.1	2.4	Fc receptor-like B (FCRLB), mRNA	NM_001002901	Hs.517422	BC067080
GSTM1	5.1	4.3	glutathione S-transferase M1 (GSTM1), transcript variant 2, mRNA	NM_146421	Hs.301961	BQ880398
CAB39L	5.1	1.9	calcium binding protein 39-like (CAB39L), transcript variant 1, mRNA	NM_030925	Hs.87159	NM_030925
SHOX2	5.1	9.8	short stature homeobox 2 (SHOX2), transcript variant SHOX2a, mRNA	NM_006884	Hs.55967	NM_006884
SKIP	5.1	9.2	SPHK1 (sphingosine kinase type 1) interacting protein (SKIP), mRNA	NM_030623	Hs.436306	CR749494
ZC3H11A	5.1	3.2	zinc finger CCCH-type containing 11A (ZC3H11A), mRNA	NM_014827	Hs.532399	CR627439
AK021798	5.1	4.7	cDNA FLJ11736 fis, clone HEMBA1005468.	AK021798	Unknown	
SPTLC3	5.1	6.0	serine palmitoyltransferase, long chain base subunit 3 (SPTLC3), mRNA	NM_018327	Hs.425023	NM_018327
ZNF160	5.1	1.7	zinc finger protein 160 (ZNF160), transcript variant 2, mRNA	NM_198893	Hs.655967	NM_198893
RNF43	5.1	3.7	ring finger protein 43 (RNF43), mRNA	NM_017763	Hs.656319	CR749257
NOTCH2NL	5.1	2.6	cDNA FLJ90584 fis, clone PLACE1000740, highly similar to Neurogenic locus notch homolog protein 2 precursor.	AK075065	Hs.515947	BX538005
DCP1A	5.1	2.0	DCP1 decapping enzyme homolog A (S. cerevisiae) (DCP1A), mRNA	NM_018403	Hs.476353	NM_018403
A_24_P324224	5.1	1.4	A_24_P324224	A_24_P324224	Unknown	
BF592096	5.1	2.3	BF592096 7o04d07.x1 NCI_CGAP_Kid11 cDNA clone IMAGE:3573157 3', mRNA sequence	BF592096	Hs.515329	NM_000983
ADHFE1	5.1	2.5	alcohol dehydrogenase, iron containing, 1 (ADHFE1), transcript variant 2,	NM_144650	Hs.268869	BC028400
THC2509288	5.1	2.4	ALU4_HUMAN (P39191) Alu subfamily SB2 sequence contamination warning entry, partial (29%)	THC2509288	Unknown	
GSTA2	5.1	2.7	glutathione S-transferase A2 (GSTA2), mRNA	NM_000846	Hs.94107	BI762502
CD1B	5.1	2.6	CD1b molecule (CD1B), mRNA	NM_001764	Hs.1310	NM_001764
THC2610628	5.1	3.1	THC2610628	THC2610628	Unknown	
DYNC1LI2	5.1	1.6	dynein, cytoplasmic 1, light intermediate chain 2 (DYNC1LI2), mRNA	NM_006141	Hs.369068	NM_006141
RND3	5.1	2.3	Rho family GTPase 3 (RND3), mRNA	NM_005168	Hs.6838	X97758
ADPRHL1	5.1	3.4	ADP-ribosylhydrolase like 1 (ADPRHL1), transcript variant 1, mRNA	NM_138430	Hs.98669	NM_199162
RASSF4	5.1	2.1	Ras association (RalGDS/AF-6) domain family 4 (RASSF4), mRNA	NM_032023	Hs.522895	AB209446
TTC12	5.1	2.7	tetratricopeptide repeat domain 12 (TTC12), mRNA	NM_017868	Hs.288772	AK125909
GALNTL2	5.1	3.4	UDP-N-acetyl-alpha-D-galactosamine:polypeptide N-acetylgalactosaminyltransferase-like 2 (GALNTL2), mRNA	NM_054110	Hs.411308	AL832575
RIN2	5.1	2.4	Ras and Rab interactor 2 (RIN2), mRNA	NM_018993	Hs.472270	NM_018993
ACTC1	5.1	4.7	actin, alpha, cardiac muscle 1 (ACTC1), mRNA	NM_005159	Hs.696107	NM_005159
RND3	5.1	2.3	Rho family GTPase 3 (RND3), mRNA	NM_005168	Hs.6838	X97758
LOC728649	5.1	8.5	mRNA; cDNA DKFZp779F2127 (from clone DKFZp779F2127).	CR627148	Hs.351215	CR627148
SOCS6	5.1	1.8	suppressor of cytokine signaling 6 (SOCS6), mRNA	NM_004232	Hs.44439	NM_004232
NTF5	5.1	2.0	neurotrophin 5 (neurotrophin 4/5) (NTF5), mRNA	NM_006179	Hs.266902	NM_006179
EDG1	5.1	7.8	endothelial differentiation, sphingolipid G-protein-coupled receptor, 1 (EDG1), mRNA	NM_001400	Hs.154210	NM_001400
SDCCAG1	5.1	1.5	serologically defined colon cancer antigen 1 (SDCCAG1), mRNA	NM_004713	Hs.655964	NM_004713
A_24_P315654	5.1	1.8	A_24_P315654	A_24_P315654	Unknown	
APOBEC2	5.1	14.3	apolipoprotein B mRNA editing enzyme, catalytic polypeptide-like 2 (APOBEC2), mRNA	NM_006789	Hs.555915	BC047767
XRN1	5.1	2.1	5'-3' exoribonuclease 1 (XRN1), transcript variant 1, mRNA	NM_019001	Hs.435103	BX640905
ANKRD45	5.1	2.7	ankyrin repeat domain 45 (ANKRD45), mRNA	NM_198493	Hs.130054	AK127170
MAPT	5.1	6.5	microtubule-associated protein tau (MAPT), transcript variant 1, mRNA	NM_016835	Hs.101174	AK226139
PLEKHO1	5.1	2.3	pleckstrin homology domain containing, family O member 1 (PLEKHO1), mRNA	NM_016274	Hs.438824	AK125609
DOCK7	5.1	2.1	dedicator of cytokinesis 7 (DOCK7), mRNA	NM_033407	Hs.538059	NM_033407
TNS1	5.1	3.8	tensin 1 (TNS1), mRNA	NM_022648	Unknown	
JOSD1	5.1	2.1	Josephin domain containing 1 (JOSD1), mRNA	NM_014876	Hs.3094	CR596979
BC035518	5.1	8.9	Homo sapiens, clone IMAGE:4214654, mRNA.	BC035518	Hs.592414	BC016962
R3HCC1	5.1	1.9	mRNA; cDNA DKFZp564N123 (from clone DKFZp564N123).	ENST00000265806	Unknown	
PIK3C2B	5.1	2.0	phosphoinositide-3-kinase, class 2, beta polypeptide (PIK3C2B), mRNA	NM_002646	Hs.497487	Y11312
MUSTN1	5.1	1.6	musculoskeletal, embryonic nuclear protein 1 (MUSTN1), mRNA	NM_205853	Hs.699481	BQ773442
JMJD1A	5.1	3.5	jumonji domain containing 1A (JMJD1A), mRNA	NM_018433	Hs.557425	BX640698
STAT4	5.1	5.3	signal transducer and activator of transcription 4 (STAT4), mRNA	NM_003151	Hs.80642	NM_003151
IBRDC1	5.1	2.0	IBR domain containing 1 (IBRDC1), mRNA	NM_152553	Hs.368639	AK122811

LOC731731	5.1	1.8	PREDICTED: similar to Nucleosome assembly protein 1-like 1 (NAP-1-related protein) (hNRP) (LOC731731), mRNA	XR_015583	Hs.647218	XR_015583
VIT	5.1	9.3	vitrin (VIT), mRNA	NM_053276	Hs.137415	AY358338
THC2559650	5.1	7.3	ALU5_HUMAN (P39192) Alu subfamily SC sequence contamination warning entry, partial (11%)	THC2559650	Unknown	
CTNNA2	5.1	3.3	catenin (cadherin-associated protein), alpha 2 (CTNNA2), mRNA	NM_004389	Hs.167368	AK127226
PPP1R13B	5.1	1.9	protein phosphatase 1, regulatory (inhibitor) subunit 13B (PPP1R13B), mRNA	NM_015316	Hs.436113	NM_015316
AK123912	5.1	6.2	cDNA FLJ41918 fis, clone PERIC2002243.	AK123912	Hs.658808	BX648566
DNAJC9	5.1	1.7	DnaJ (Hsp40) homolog, subfamily C, member 9 (DNAJC9), mRNA	NM_015190	Hs.654694	AK094162
ACOX2	5.1	2.6	acyl-Coenzyme A oxidase 2, branched chain (ACOX2), mRNA	NM_003500	Hs.444959	BC033517
AK095719	5.1	4.6	cDNA FLJ38400 fis, clone FEBRA2008159.	AK095719	Hs.457407	AK124699
THC2643265	5.1	1.8	ALU1_HUMAN (P39188) Alu subfamily J sequence contamination warning entry, partial (5%)	THC2643265	Unknown	
CRB1	5.1	3.1	crumbs homolog 1 (Drosophila) (CRB1), mRNA	NM_201253	Hs.126135	BX640729
NPFF	5.1	1.8	neuropeptide FF-amide peptide precursor (NPFF), mRNA	NM_003717	Hs.104555	AK128838
ENST00000339388	5.1	3.4	neuroblastoma breakpoint family, member 11 (NBPF11), mRNA	ENST00000339388	Unknown	
CALCRL	5.1	14.6	calcitonin receptor-like (CALCRL), mRNA	NM_005795	Hs.470882	NM_005795
DB381305	5.1	5.4	DB381305 PLACE3 cDNA clone PLACE3000400 3', mRNA sequence	DB381305	Hs.629427	DB381305
ENST00000342584	5.1	1.8	PP7080.	ENST00000342584	Unknown	
FLJ37035	5.1	2.7	cDNA FLJ37035 fis, clone BRACE2011545.	AK094354	Hs.535286	AK094354
ROPN1	5.1	4.9	ropporin, rhophilin associated protein 1 (ROPN1), mRNA	NM_017578	Hs.567516	AL133624
ZNF71	5.1	1.8	zinc finger protein 71 (ZNF71), mRNA	NM_021216	Hs.301431	NM_021216
HISPPD1	5.1	1.9	histidine acid phosphatase domain containing 1 (HISPPD1), mRNA	NM_015216	Hs.212046	AB007893
IMPAD1	5.1	2.4	cDNA FLJ33669 fis, clone BRAMY2028740.	AK090988	Hs.595255	AK090988
NEO1	5.1	2.2	neogenin homolog 1 (chicken) (NEO1), mRNA	NM_002499	Hs.699228	AB209412
LGI2	5.1	2.3	leucine-rich repeat LGI family, member 2 (LGI2), mRNA	NM_018176	Hs.12488	AB067503
ALG14	5.1	2.4	asparagine-linked glycosylation 14 homolog (yeast) (ALG14), mRNA	NM_144988	Hs.408927	BC011706
TUSC3	5.1	2.0	tumor suppressor candidate 3 (TUSC3), transcript variant 2, mRNA	NM_178234	Hs.591845	BX641112
THC2682291	5.1	3.3	THC2682291	THC2682291	Unknown	
TMOD1	5.0	6.4	tropomodulin 1 (TMOD1), mRNA	NM_003275	Hs.494595	AK096156
TRAM1L1	5.0	3.6	translocation associated membrane protein 1-like 1 (TRAM1L1), mRNA	NM_152402	Hs.570737	BC030831
ITGAV	5.0	2.6	integrin, alpha V (vitronectin receptor, alpha polypeptide, antigen CD51) (ITGAV), mRNA	NM_002210	Hs.436873	NM_002210
MARCKS	5.0	2.1	myristoylated alanine-rich protein kinase C substrate (MARCKS), mRNA	NM_002356	Hs.519909	NM_002356
C14orf11	5.0	1.9	chromosome 14 open reading frame 11 (C14orf11), mRNA	NM_018453	Hs.433269	NM_018453
G43356	5.0	7.5	WIAF-2034-STS Human THudson EST STS cDNA, sequence tagged site.	G43356	Unknown	
ACYP2	5.0	1.9	H.sapiens mRNA for acylphosphatase, muscle type (MT) isoenzyme.	X84195	Unknown	
TLOC1	5.0	1.9	translocation protein 1 (TLOC1), mRNA	NM_003262	Hs.592561	NM_003262
SUFU	5.0	2.3	suppressor of fused homolog (Drosophila) (SUFU), mRNA	NM_016169	Hs.404089	NM_016169
C5orf4	5.0	3.6	chromosome 5 open reading frame 4 (C5orf4), transcript variant 2, mRNA	NM_032385	Hs.699510	AK023820
CB051804	5.0	2.7	NISC_gl03c01.x1 NCI_CGAP_Lei2 cDNA clone IMAGE:3289728 3', mRNA sequence	CB051804	Hs.624034	CB051804
C10orf30	5.0	1.5	chromosome 10 open reading frame 30, mRNA (cDNA clone MGC:35247 IMAGE:5172600), complete cds.	BC031618	Hs.498740	AL832195
NXT1	5.0	1.9	NTF2-like export factor 1 (NXT1), mRNA	NM_013248	Hs.516933	BM809200
SPCS2	5.0	1.6	signal peptidase complex subunit 2 homolog (S. cerevisiae) (SPCS2), mRNA	NM_014752	Hs.282700	AK091429
DCC	5.0	3.6	deleted in colorectal carcinoma (DCC), mRNA	NM_005215	Hs.579550	NM_005215
C21orf71	5.0	2.7	full length insert cDNA clone ZD81A03.	AF086441	Hs.384586	BU192089
CCDC93	5.0	1.7	coiled-coil domain containing 93 (CCDC93), mRNA	NM_019044	Hs.107845	NM_019044
ENST00000377492	5.0	3.0	FAM27E1 protein.	ENST00000377492	Unknown	
AK026687	5.0	2.8	cDNA: FLJ23034 fis, clone LNG02018.	AK026687	Hs.699196	CR936719
THC2596018	5.0	3.7	Q99834_HUMAN (Q99834) Nuclear antigen H731, partial (21%)	THC2596018	Unknown	
AK094525	5.0	1.9	cDNA FLJ37206 fis, clone BRALZ2007545.	AK094525	Hs.587467	BX647732
CBX8	5.0	2.2	chromobox homolog 8 (Pc class homolog, Drosophila) (CBX8), mRNA	NM_020649	Hs.387258	AF174482
INSIG2	5.0	3.2	insulin induced gene 2 (INSIG2), mRNA	NM_016133	Hs.7089	BX647805
PDZRN4	5.0	21.6	PDZ domain containing RING finger 4 (PDZRN4), mRNA	NM_013377	Hs.380044	AK131554
HADHB	5.0	1.9	hydroxyacyl-Coenzyme A dehydrogenase/3-ketoacyl-Coenzyme A thiolase/enoyl-Coenzyme A hydratase (trifunctional protein), beta subunit (HADHB), nuclear gene encoding mitochondrial protein, mRNA	NM_000183	Hs.534639	AK095278

PLA2G4A	5.0	3.5	phospholipase A2, group IVA (cytosolic, calcium-dependent) (PLA2G4A), mRNA	NM_024420	Hs.497200	NM_024420
EPB41L4A	5.0	2.6	erythrocyte membrane protein band 4.1 like 4A (EPB41L4A), mRNA	NM_022140	Hs.584954	NM_022140
THC2656519	5.0	6.7	THC2656519	THC2656519	Unknown	
DENND2A	5.0	1.8	DENN/MADD domain containing 2A (DENND2A), mRNA	NM_015689	Hs.6385	BC115004
A_32_P196615	5.0	3.7	A_32_P196615	A_32_P196615	Unknown	
15E1.2	5.0	2.6	hypothetical protein LOC283459, mRNA (cDNA clone IMAGE:4822098).	BC034962	Hs.369624	BC034962
ZNF557	5.0	1.5	zinc finger protein 557 (ZNF557), transcript variant 1, mRNA	NM_024341	Hs.591380	NM_024341
BM806490	5.0	2.7	AGENCOURT_6553853 NIH_MGC_71 cDNA clone IMAGE:5555887 5', mRNA sequence	BM806490	Hs.553851	BM806490
FAM76B	5.0	1.6	family with sequence similarity 76, member B (FAM76B), mRNA	NM_144664	Hs.288304	NM_144664
THC2685688	5.0	6.4	Q9BZV5_HUMAN (Q9BZV5) RANBP21, partial (3%)	THC2685688	Unknown	
LOC392505	5.0	1.6	PREDICTED: similar to 60S ribosomal protein L6 (TAX-responsive enhancer element-binding protein 107) (TAXREB107) (Neoplasm-related protein C140) (LOC392505), mRNA	XR_018334	Hs.648330	XR_018334
SNED1	5.0	8.0	mRNA for FLJ00133 protein.	AK074062	Hs.471834	BX640653
RP11-78J21.1	5.0	1.4	heterogeneous nuclear ribonucleoprotein A1-like (LOC144983), transcript variant 1, mRNA	NM_001011724	Hs.447506	AK126454
THC2633920	5.0	6.7	Q3RFB1_XYLFA (Q3RFB1) Aminotransferase, class I and II, partial (4%)	THC2633920	Unknown	
BC041417	5.0	10.9	cDNA clone IMAGE:5286005.	BC041417	Hs.635442	BC041417
ITGAV	5.0	2.6	integrin, alpha V (vitronectin receptor, alpha polypeptide, antigen CD51) (ITGAV), mRNA	NM_002210	Hs.436873	NM_002210
MED31	5.0	2.3	mediator of RNA polymerase II transcription, subunit 31 homolog (S. cerevisiae) (MED31), mRNA	NM_016060	Hs.567493	NM_016060
THC2688475	5.0	3.2	Q29PE7_DROPS (Q29PE7) GA16406-PA (Fragment), partial (7%)	THC2688475	Unknown	
LRP1B	5.0	8.3	low density lipoprotein-related protein 1B (deleted in tumors) (LRP1B), mRNA	NM_018557	Hs.656461	NM_018557
THC2730823	5.0	1.6	O19057_PONPY (O19057) Fertilin alpha protein, partial (19%)	THC2730823	Unknown	
ANKMY2	5.0	2.0	ankyrin repeat and MYND domain containing 2 (ANKMY2), mRNA	NM_020319	Hs.157378	AK001740
SLC6A12	5.0	4.1	solute carrier family 6 (neurotransmitter transporter, betaine/GABA), member 12 (SLC6A12), mRNA	NM_003044	Hs.437174	AK125026
CMTM3	5.0	2.0	CKLF-like MARVEL transmembrane domain containing 3 (CMTM3), transcript variant 1, mRNA	NM_144601	Hs.298198	AK056324
LOC646949	5.0	1.7	PREDICTED: similar to ribosomal protein L23 (LOC646949), mRNA	XR_017294	Hs.512542	CD556150
PPFIA4	5.0	3.9	protein tyrosine phosphatase, receptor type, f polypeptide (PTPRF), interacting protein (liprin), alpha 4 (PPFIA4), mRNA	NM_015053	Hs.153648	NM_015053
ZNF410	5.0	2.2	zinc finger protein 410 (ZNF410), mRNA	NM_021188	Hs.270869	BC034973
ARHGEF7	5.0	2.9	Rho guanine nucleotide exchange factor (GEF) 7 (ARHGEF7), transcript variant 2, mRNA	NM_145735	Hs.508738	BX648030
ZNF333	5.0	1.8	zinc finger protein 333 (ZNF333), mRNA	NM_032433	Hs.515215	AF372702
A_24_P33213	5.0	1.6	A_24_P33213	A_24_P33213	Unknown	
PSD2	5.0	4.0	pleckstrin and Sec7 domain containing 2 (PSD2), mRNA	NM_032289	Hs.21963	AL136559
SCML1	5.0	8.1	sex comb on midleg-like 1 (Drosophila) (SCML1), transcript variant 1, mRNA	NM_001037540	Hs.109655	NM_001037540
RP5-1022P6.2	5.0	2.4	Putative glycerophosphodiester phosphodiesterase 5 (EC 3.1.-.-).	ENST00000379019	Unknown	
ACPL2	5.0	1.7	acid phosphatase-like 2 (ACPL2), transcript variant 1, mRNA	NM_152282	Hs.657887	BC036701
IKZF2	5.0	3.6	IKAROS family zinc finger 2 (Helios) (IKZF2), transcript variant 2, mRNA	NM_001079526	Hs.604950	NM_001079526
ZNF673	5.0	1.8	zinc finger protein 673 (ZNF673), mRNA	NM_017776	Hs.632800	AK097159
ADAMTS1	5.0	2.1	ADAM metallopeptidase with thrombospondin type 1 motif, 1 (ADAMTS1), mRNA	NM_006988	Hs.643357	NM_006988
MAK	5.0	3.4	male germ cell-associated kinase (MAK), mRNA	NM_005906	Hs.446125	NM_005906
NBPF10	5.0	3.3	neuroblastoma breakpoint family, member 10 (NBPF10), mRNA	NM_001039703	Hs.515947	BX538005
ORM2	5.0	3.2	orosomucoid 2 (ORM2), mRNA	NM_000608	Hs.522356	AK055563
UBL3	5.0	1.7	ubiquitin-like 3 (UBL3), mRNA	NM_007106	Hs.145575	BC044582
A2BP1	5.0	3.2	ataxin 2-binding protein 1 (A2BP1), transcript variant 3, mRNA	NM_145893	Hs.459842	BC026312
THC2650352	5.0	2.8	G6PI_HALSA (Q9HNQ6) Probable glucose-6-phosphate isomerase (GPI) (Phosphoglucose isomerase) (PGI) (Phosphohexose isomerase) (PHI) , partial (5%)	THC2650352	Unknown	
IK	5.0	1.6	IK cytokine, down-regulator of HLA II (IK), mRNA	NM_006083	Hs.421245	AK225924
WTAP	5.0	1.5	Wilms tumor 1 associated protein (WTAP), transcript variant 3, mRNA	NM_152858	Hs.446091	CR627456
TNFAIP3	5.0	4.2	tumor necrosis factor, alpha-induced protein 3 (TNFAIP3), mRNA	NM_006290	Hs.591338	BC041790
NAV3	5.0	5.5	neuron navigator 3 (NAV3), mRNA	NM_014903	Hs.655301	NM_014903
KIAA1305	5.0	2.2	KIAA1305, mRNA (cDNA clone IMAGE:3865984), complete cds.	BC008219	Hs.288348	NM_025081
RPS27	5.0	1.7	ribosomal protein S27 (metallopanstimulin 1) (RPS27), mRNA	NM_001030	Hs.546291	BQ278503
AK093691	5.0	4.8	cDNA FLJ36372 fis, clone THYMU2008072.	AK093691	Hs.561160	AK093691
SLC20A2	5.0	1.7	solute carrier family 20 (phosphate transporter), member 2 (SLC20A2), mRNA	NM_006749	Hs.653173	NM_006749

THC2624002	5.0	5.6	Q9BXR7_HUMAN (Q9BXR7) Interleukin 10 (Fragment), partial (93%)	THC2624002	Unknown	
RPL13	5.0	1.5	ribosomal protein L13 (RPL13), transcript variant 2, mRNA	NM_033251	Hs.410817	AK095954
RGAG4	5.0	8.5	retrotransposon gag domain containing 4 (RGAG4), mRNA	NM_001024455	Hs.694084	NM_001024455
FAM120B	5.0	2.2	PPARgamma constitutive coactivator 1	ENST00000366751	Unknown	
TCAP	5.0	4.5	titin-cap (telethonin) (TCAP), mRNA	NM_003673	Hs.514146	AK096328
AA630774	5.0	2.6	ac14c08.s1 Stratagene HeLa cell s3 937216 cDNA clone IMAGE:856430 3', mRNA sequence	AA630774	Hs.694979	AA630774
PQLC1	5.0	2.1	cDNA: FLJ22378 fis, clone HRC07430.	AK026031	Hs.288284	AK126188
A_32_P149246	5.0	2.0	A_32_P149246	A_32_P149246	Unknown	
TSPAN8	5.0	3.5	tetraspanin 8 (TSPAN8), mRNA	NM_004616	Hs.170563	BC070168
AK098422	5.0	2.9	cDNA FLJ25556 fis, clone JTH02629.	AK098422	Hs.633903	AK098422
ITGAV	5.0	2.5	integrin, alpha V (vitronectin receptor, alpha polypeptide, antigen CD51) (ITGAV), mRNA	NM_002210	Hs.436873	NM_002210
RND3	5.0	2.3	Rho family GTPase 3 (RND3), mRNA	NM_005168	Hs.6838	X97758
THC2637644	5.0	7.7	THC2637644	THC2637644	Unknown	
CAPSL	5.0	3.2	calcyphosine-like (CAPSL), transcript variant 1, mRNA	NM_144647	Hs.55150	NM_144647
ARHGEF12	5.0	2.3	Rho guanine nucleotide exchange factor (GEF) 12 (ARHGEF12), mRNA	NM_015313	Hs.24598	AF180681
C14orf152	5.0	3.0	chromosome 14 open reading frame 152 (C14orf152), mRNA	NM_138344	Hs.525550	AK093387
DCUN1D4	5.0	1.7	DCN1, defective in cullin neddylation 1, domain containing 4 (S. cerevisiae) (DCUN1D4), transcript variant 1, mRNA	NM_001040402	Hs.605388	NM_001040402
THC2623121	5.0	2.4	THC2623121	THC2623121	Unknown	
KCTD12	5.0	4.1	potassium channel tetramerisation domain containing 12 (KCTD12), mRNA	NM_138444	Hs.693617	NM_138444
THC2657989	5.0	4.3	ALU2_HUMAN (P39189) Alu subfamily SB sequence contamination warning entry, partial (27%)	THC2657989	Unknown	
PNRC1	5.0	3.7	proline-rich nuclear receptor coactivator 1 (PNRC1), mRNA	NM_006813	Hs.75969	NM_006813
PBLD	5.0	1.8	phenazine biosynthesis-like protein domain containing (PBLD), transcript variant 1, mRNA	NM_022129	Hs.198158	NM_022129
HIST1H2AD	5.0	2.7	histone cluster 1, H2ad (HIST1H2AD), mRNA	NM_021065	Hs.679229	DA450711
NPHP3	5.0	1.6	nephronophthisis 3 (adolescent) (NPHP3), mRNA	NM_153240	Hs.511991	CR749498
C1orf149	5.0	2.2	chromosome 1 open reading frame 149 (C1orf149), mRNA	NM_022756	Hs.17118	BX640719
THC2518705	5.0	2.4	THC2518705	THC2518705	Unknown	
NDUFC2	5.0	2.0	NADH dehydrogenase (ubiquinone) 1, subcomplex unknown, 2, 14.5kDa (NDUFC2), mRNA	NM_004549	Hs.407860	NM_004549
GRIK2	5.0	8.0	Glutamate receptor, ionotropic kainate 2 precursor (Glutamate receptor 6) (GluR-6) (GluR6) (Excitatory amino acid receptor 4) (EAA4).	ENST00000333309	Unknown	
SSX2IP	5.0	1.9	synovial sarcoma, X breakpoint 2 interacting protein (SSX2IP), mRNA	NM_014021	Hs.22587	AB023140
ITGB8	5.0	2.2	integrin, beta 8 (ITGB8), mRNA	NM_002214	Hs.592171	NM_002214
THC2657355	5.0	18.7	THC2657355	THC2657355	Unknown	
A2BP1	5.0	2.9	ataxin 2-binding protein 1 (A2BP1), transcript variant 4, mRNA	NM_018723	Hs.459842	BC026312
NR4A3	5.0	2.9	nuclear receptor subfamily 4, group A, member 3 (NR4A3), transcript variant 2, mRNA	NM_173198	Hs.279522	NM_173198
ZNF584	5.0	1.9	zinc finger protein 584 (ZNF584), mRNA	NM_173548	Hs.439551	AK097218
BACH1	4.9	1.7	BTB and CNC homology 1, basic leucine zipper transcription factor 1 (BACH1), transcript variant 1, mRNA	NM_206866	Hs.154276	NM_206866
PIGH	4.9	2.5	phosphatidylinositol glycan anchor biosynthesis, class H (PIGH), mRNA	NM_004569	Hs.553497	NM_004569
RICTOR	4.9	1.9	rapamycin-insensitive companion of mTOR (RICTOR), mRNA	NM_152756	Hs.407926	NM_152756
ITGAV	4.9	2.6	integrin, alpha V (vitronectin receptor, alpha polypeptide, antigen CD51) (ITGAV), mRNA	NM_002210	Hs.436873	NM_002210
DPY19L2	4.9	7.7	dpy-19-like 2 (C. elegans) (DPY19L2), mRNA	NM_173812	Hs.533644	NM_173812
AK022299	4.9	3.0	cDNA FLJ12237 fis, clone MAMMA1001249.	AK022299	Hs.565253	AK094188
CECR7	4.9	1.9	cat eye syndrome chromosome region, candidate 7, mRNA (cDNA clone IMAGE:5289002).	BC043198	Hs.129751	NM_014339
ENST00000372493	4.9	3.2	ENST00000372493	ENST00000372493	Unknown	
KLHL5	4.9	1.8	kelch-like 5 (Drosophila) (KLHL5), transcript variant 1, mRNA	NM_015990	Hs.272251	NM_015990
CA13	4.9	2.2	carbonic anhydrase XIII (CA13), mRNA	NM_198584	Hs.127189	NM_198584
C18orf18	4.9	2.0	chromosome 18 open reading frame 18, mRNA (cDNA clone MGC:17515 IMAGE:3457488), complete cds.	BC010538	Hs.657197	BQ067788
SRP14	4.9	1.7	signal recognition particle 14kDa (homologous Alu RNA binding protein) (SRP14), mRNA	NM_003134	Hs.533732	BM805684
SOAT1	4.9	2.8	sterol O-acyltransferase (acyl-Coenzyme A: cholesterol acyltransferase) 1 (SOAT1), transcript variant 688113, mRNA	NM_003101	Hs.496383	L21934
ACCN2	4.9	2.7	amiloride-sensitive cation channel 2, neuronal (ACCN2), transcript variant 1, mRNA	NM_020039	Hs.274361	NM_020039
AF143331	4.9	4.0	clone IMAGE:112850 mRNA sequence.	AF143331	Hs.248162	NM_005422
RRAGA	4.9	1.7	Ras-related GTP binding A (RRAGA), mRNA	NM_006570	Hs.432330	NM_006570

AF318337	4.9	1.9	pp13759 mRNA, complete cds.	AF318337	Hs.375766	AK055081
N6AMT2	4.9	3.0	N-6 adenine-specific DNA methyltransferase 2 (putative) (N6AMT2), mRNA	NM_174928	Hs.26674	BG403594
MTHFR	4.9	2.8	5,10-methylenetetrahydrofolate reductase (NADPH) (MTHFR), mRNA	NM_005957	Hs.214142	NM_005957
RRAGB	4.9	5.1	Ras-related GTP binding B (RRAGB), transcript variant RAGBl, mRNA	NM_016656	Hs.50282	NM_016656
PAX3	4.9	19.1	paired box gene 3 (Waardenburg syndrome 1) (PAX3), transcript variant PAX3D, mRNA	NM_181458	Hs.42146	NM_181458
C9orf102	4.9	1.8	chromosome 9 open reading frame 102 (C9orf102), transcript variant 1, mRNA	NM_020207	Hs.632686	NM_020207
LOC440498	4.9	3.2	cDNA clone IMAGE:3887603.	BC017654	Hs.191582	BM043035
C10orf79	4.9	10.8	chromosome 10 open reading frame 79 (C10orf79), mRNA	NM_025145	Hs.288927	NM_025145
A_32_P230537	4.9	2.9	A_32_P230537	A_32_P230537	Unknown	
DDX50	4.9	1.5	DEAD (Asp-Glu-Ala-Asp) box polypeptide 50 (DDX50), mRNA	NM_024045	Hs.522984	BC000272
CB240827	4.9	2.6	UI-CF-FN0-afw-h-15-0-UI.s1 UI-CF-FN0 cDNA clone UI-CF-FN0-afw-h-15-0-UI 3', mRNA sequence	CB240827	Hs.621746	CB240827
PAG1	4.9	5.9	phosphoprotein associated with glycosphingolipid microdomains 1 (PAG1), mRNA	NM_018440	Hs.266175	NM_018440
LRP2BP	4.9	2.5	LRP2 binding protein (LRP2BP), mRNA	NM_018409	Hs.558513	AB037746
RWDD4A	4.9	1.6	RWD domain containing 4A (RWDD4A), mRNA	NM_152682	Hs.133337	NM_152682
A_24_P110601	4.9	6.2	A_24_P110601	A_24_P110601	Unknown	
RFPL1S	4.9	9.3	ret finger protein-like 1 antisense (RFPL1S) on chromosome 22	NR_002727	Unknown	
CARTPT	4.9	6.9	CART prepropeptide (CARTPT), mRNA	NM_004291	Hs.1707	NM_004291
MIA2	4.9	5.7	melanoma inhibitory activity 2 (MIA2), mRNA	NM_054024	Hs.287694	BX648228
RABL5	4.9	1.5	RAB, member RAS oncogene family-like 5 (RABL5), mRNA	NM_022777	Hs.389104	AL157469
WDR78	4.9	14.9	WD repeat domain 78 (WDR78), transcript variant 1, mRNA	NM_024763	Hs.49421	AK127011
LUC7L2	4.9	1.7	LUC7-like 2 (S. cerevisiae) (LUC7L2), mRNA	NM_016019	Hs.370475	BC041001
STX12	4.9	3.2	syntaxin 12 (STX12), mRNA	NM_177424	Hs.523855	NM_177424
LOC730259	4.9	3.2	PREDICTED: hypothetical protein LOC730259 (LOC730259), mRNA	XM_001133381	Hs.652173	XM_001133381
NDUFB3	4.9	1.8	NADH dehydrogenase (ubiquinone) 1 beta subcomplex, 3, 12kDa (NDUFB3), mRNA	NM_002491	Hs.109760	BM552702
LGALS3	4.9	4.5	lectin, galactoside-binding, soluble, 3 (galectin 3) (LGALS3), transcript variant 1, mRNA	NM_002306	Hs.531081	AB209391
NCAM1	4.9	3.1	neural cell adhesion molecule 1 (NCAM1), transcript variant 3, mRNA	NM_001076682	Hs.503878	NM_181351
BU561429	4.9	3.6	AGENCOURT_10278917 NIH_MGC_82 cDNA clone IMAGE:6592459 5', mRNA sequence	BU561429	Hs.374243	BU561429
ATP6V1G2	4.9	1.7	ATPase, H+ transporting, lysosomal 13kDa, V1 subunit G2 (ATP6V1G2), transcript variant 1, mRNA	NM_130463	Hs.249227	NM_130463
AI801879	4.9	5.5	tx28f05.x1 NCI_CGAP_Lu24 cDNA clone IMAGE:2270913 3', mRNA sequence	AI801879	Hs.144030	AI801879
ATP11B	4.9	2.2	ATPase, Class VI, type 11B (ATP11B), mRNA	NM_014616	Hs.478429	NM_014616
DMTF1	4.9	1.4	cyclin D binding myb-like transcription factor 1 (DMTF1), mRNA	NM_021145	Hs.654981	AK126664
LOC149351	4.9	4.5	hypothetical protein LOC149351, mRNA (cDNA clone IMAGE:5273076).	BC036441	Hs.546492	AK022898
ITGAV	4.9	2.6	integrin, alpha V (vitronectin receptor, alpha polypeptide, antigen CD51) (ITGAV), mRNA	NM_002210	Hs.436873	NM_002210
AMDHD1	4.9	1.8	amidohydrolase domain containing 1 (AMDHD1), mRNA	NM_152435	Hs.424907	AK054617
BCORL1	4.9	2.1	BCL6 co-repressor-like 1 (BCORL1), mRNA	NM_021946	Hs.496748	NM_021946
HYDIN	4.9	2.5	hydrocephalus inducing homolog (mouse) (HYDIN), transcript variant 1, mRNA	NM_032821	Hs.461229	NM_032821
HISPPD1	4.9	1.9	histidine acid phosphatase domain containing 1 (HISPPD1), mRNA	NM_015216	Hs.212046	AB007893
RND3	4.9	2.3	Rho family GTPase 3 (RND3), mRNA	NM_005168	Hs.6838	X97758
RND3	4.9	2.3	Rho family GTPase 3 (RND3), mRNA	NM_005168	Hs.6838	X97758
ZRANB1	4.9	1.9	Zinc finger Ran-binding domain-containing protein 1 (Protein TRABID).	ENST00000359653	Unknown	
ITSN1	4.9	6.9	Intersectin-1 (SH3 domain-containing protein 1A) (SH3P17).	ENST00000381318	Unknown	
FLJ23588	4.9	4.3	CAP-binding protein complex interacting protein 1 (FLJ23588), transcript variant 1, mRNA	NM_022785	Hs.658996	NM_022785
FLJ31306	4.9	2.5	full-length cDNA clone CS0DF021YK18 of Fetal brain of (human).	CR606347	Hs.531089	BC034618
BDNF	4.9	6.0	brain-derived neurotrophic factor (BDNF), transcript variant 1, mRNA	NM_170735	Hs.502182	EF689009
TRAPPC2	4.9	1.9	trafficking protein particle complex 2 (TRAPPC2), transcript variant 1, mRNA	NM_001011658	Hs.592238	NM_001011658
CR610211	4.9	1.9	full-length cDNA clone CS0DD002YI15 of Neuroblastoma Cot 50-normalized of (human).	CR610211	Hs.556090	CR610211
CART1	4.9	7.6	cartilage paired-class homeoprotein 1 (CART1), mRNA	NM_006982	Hs.41683	U31986
CTDSPL2	4.9	1.5	CTD (carboxy-terminal domain, RNA polymerase II, polypeptide A) small phosphatase like 2 (CTDSPL2), mRNA	NM_016396	Hs.646495	NM_016396
ITGAV	4.9	2.6	integrin, alpha V (vitronectin receptor, alpha polypeptide, antigen CD51) (ITGAV), mRNA	NM_002210	Hs.436873	NM_002210
PCOLCE	4.9	2.5	procollagen C-endopeptidase enhancer (PCOLCE), mRNA	NM_002593	Hs.202097	BC000574
ST3GAL4	4.9	3.3	cDNA FLJ11867 fis, clone HEMBA1006976, weakly similar to H.sapiens mRNA for Gal-beta(1-3/1-4)GlcNAc alpha-2.3-sialyltransferase.	AK021929	Hs.591947	AK128605

BC031359	4.9	2.4	Homo sapiens, clone IMAGE:4778855, mRNA, partial cds.	BC031359	Hs.552202	BC031359
AK123861	4.9	2.8	cDNA FLJ41867 fis, clone OCBBF2005546.	AK123861	Hs.658919	AK123861
RPS27	4.9	1.7	ribosomal protein S27 (metallopanstimulin 1) (RPS27), mRNA	NM_001030	Hs.546291	BQ278503
PARP16	4.9	2.0	poly (ADP-ribose) polymerase family, member 16 (PARP16), mRNA	NM_017851	Hs.30634	BC006389
WDR60	4.9	1.5	WD repeat domain 60 (WDR60), mRNA	NM_018051	Hs.389945	NM_018051
LUC7L2	4.9	1.7	LUC7-like 2 (S. cerevisiae) (LUC7L2), mRNA	NM_016019	Hs.370475	BC041001
HACE1	4.9	1.6	HECT domain and ankyrin repeat containing, E3 ubiquitin protein ligase 1 (HACE1), mRNA	NM_020771	Hs.434340	AB037741
VGLL4	4.9	2.1	vestigial like 4 (Drosophila) (VGLL4), mRNA	NM_014667	Hs.373959	AK126479
Gcom1	4.9	1.9	GRINL1A combined protein (Gcom1), transcript variant 1, mRNA	NM_001018090	Hs.437256	NM_001018090
A_32_P82293	4.9	3.6	A_32_P82293	A_32_P82293	Unknown	
SPIN	4.9	1.8	spindlin (SPIN), mRNA	NM_006717	Hs.146804	NM_006717
ENST00000340612	4.9	23.8	Novel protein (Fragment).	ENST00000340612	Unknown	
RND3	4.9	2.3	Rho family GTPase 3 (RND3), mRNA	NM_005168	Hs.6838	X97758
PDCD4	4.9	2.5	programmed cell death 4 (neoplastic transformation inhibitor) (PDCD4), transcript variant 2, mRNA	NM_145341	Hs.232543	BX537500
ZNF251	4.9	2.5	Zinc finger protein 251 (Fragment).	ENST00000292562	Unknown	
SLC30A7	4.9	2.4	solute carrier family 30 (zinc transporter), member 7 (SLC30A7), mRNA	NM_133496	Hs.533903	BC064692
FBXO15	4.9	5.4	F-box protein 15 (FBXO15), mRNA	NM_152676	Hs.664011	AK093252
THC2529684	4.9	4.2	1WAO_1 Chain 1, Pp5 Structure. {Homo sapiens} (exp=-1; wgp=0; cg=0), partial (19%)	THC2529684	Unknown	
TBX4	4.9	7.1	T-box 4 (TBX4), mRNA	NM_018488	Hs.143907	BC142620
AK090897	4.9	2.6	cDNA FLJ33578 fis, clone BRAMY2011639.	AK090897	Hs.420757	BC063474
THC2586657	4.9	2.5	THC2586657	THC2586657	Unknown	
DKFZp666G057	4.9	3.6	hypothetical protein DKFZp666G057 (DKFZp666G057), mRNA	NM_001008226	Hs.459117	AL833762
C20orf177	4.9	1.6	Uncharacterized protein C20orf177.	ENST00000358293	Unknown	
HIST1H2BK	4.9	5.4	histone cluster 1, H2bk (HIST1H2BK), mRNA	NM_080593	Hs.437275	BU500887
RP11-50D16.3	4.9	2.2	similar to RIKEN cDNA 8030451K01 (LOC387921), transcript variant 1, mRNA	NM_001012754	Hs.507783	NM_001012754
FAM89A	4.9	3.6	family with sequence similarity 89, member A (FAM89A), mRNA	NM_198552	Hs.38516	CR617492
C1orf213	4.9	2.9	chromosome 1 open reading frame 213 (C1orf213), transcript variant 1, mRNA	NM_138479	Hs.61884	BC054115
GNG8	4.9	2.8	guanine nucleotide binding protein (G protein), gamma 8 (GNG8), mRNA	NM_033258	Hs.283961	CB565924
A_32_P131870	4.9	5.1	A_32_P131870	A_32_P131870	Unknown	
AK057725	4.9	3.2	cDNA FLJ33163 fis, clone UTERU2000541.	AK057725	Hs.659751	AK128076
ITGAV	4.9	2.6	integrin, alpha V (vitronectin receptor, alpha polypeptide, antigen CD51) (ITGAV), mRNA	NM_002210	Hs.436873	NM_002210
EPM2A	4.9	1.7	epilepsy, progressive myoclonus type 2A, Lafora disease (laforin) (EPM2A), transcript variant 1, mRNA	NM_005670	Hs.486696	AF454492
CDC42	4.9	1.8	cell division cycle 42 (GTP binding protein, 25kDa) (CDC42), transcript variant 3, mRNA	NM_001039802	Hs.690198	NM_001039802
KYNU	4.9	2.2	kynureninase (L-kynurenine hydrolase) (KYNU), transcript variant 1, mRNA	NM_003937	Hs.470126	CR609484
PAIP1	4.9	1.5	poly(A) binding protein interacting protein 1 (PAIP1), transcript variant 1, mRNA	NM_006451	Hs.482038	NM_006451
SLC13A5	4.9	4.4	solute carrier family 13 (sodium-dependent citrate transporter), member 5 (SLC13A5), mRNA	NM_177550	Hs.399496	BX647354
C12orf4	4.9	1.9	chromosome 12 open reading frame 4 (C12orf4), mRNA	NM_020374	Hs.302977	NM_020374
ITGAV	4.9	2.6	integrin, alpha V (vitronectin receptor, alpha polypeptide, antigen CD51) (ITGAV), mRNA	NM_002210	Hs.436873	NM_002210
SCN9A	4.9	3.6	sodium channel, voltage-gated, type IX, alpha (SCN9A), mRNA	NM_002977	Hs.699185	NM_002977
HISPPD1	4.9	1.8	histidine acid phosphatase domain containing 1 (HISPPD1), mRNA	NM_015216	Hs.212046	AB007893
RAI1	4.9	3.1	retinoic acid induced 1 (RAI1), mRNA	NM_030665	Hs.655395	NM_030665
KLHL20	4.9	2.0	kelch-like 20 (Drosophila) (KLHL20), mRNA	NM_014458	Hs.495035	NM_014458
THC2661496	4.9	1.9	Q3C9X1_9CLOT (Q3C9X1) Cobalt transport protein, partial (5%)	THC2661496	Unknown	
ZNF527	4.9	2.4	cDNA FLJ34266 fis, clone FEBRA2002682, moderately similar to ZINC FINGER PROTEIN 91.	AK091585	Hs.590940	NM_032453
VPS13A	4.9	2.0	Vacuolar protein sorting-associated protein 13A (Chorein) (Chorea-acanthocytosis protein).	ENST00000376646	Unknown	
NNT	4.9	2.0	nicotinamide nucleotide transhydrogenase (NNT), mRNA	NM_182977	Hs.482043	NM_012343
RND3	4.8	2.3	Rho family GTPase 3 (RND3), mRNA	NM_005168	Hs.6838	X97758
GARNL1	4.8	1.6	GTPase activating Rap/RanGAP domain-like 1 (GARNL1), transcript variant 1, mRNA	NM_014990	Hs.113150	AY596970
DYNC1I2	4.8	1.6	dynein, cytoplasmic 1, intermediate chain 2 (DYNC1I2), mRNA	NM_001378	Hs.546250	AK055491
ITGAV	4.8	2.5	integrin, alpha V (vitronectin receptor, alpha polypeptide, antigen CD51) (ITGAV), mRNA	NM_002210	Hs.436873	NM_002210
RSPO3	4.8	9.1	R-spondin 3 homolog (Xenopus laevis) (RSPO3), mRNA	NM_032784	Hs.135254	AF251057

AK022030	4.8	1.7	cDNA FLJ11968 fis, clone HEMBB1001133.	AK022030	Hs.288178	CR936683
KIAA1211	4.8	2.1	mRNA; cDNA DKFZp434F117 (from clone DKFZp434F117).	AL133028	Hs.596667	NM_020722
GSTM1	4.8	4.3	glutathione S-transferase M1 (GSTM1), transcript variant 2, mRNA	NM_146421	Hs.301961	BQ880398
AK130741	4.8	3.1	cDNA FLJ27231 fis, clone SYN06240.	AK130741	Hs.347034	AK130741
PGM5	4.8	2.7	phosphoglucomutase 5 (PGM5), mRNA	NM_021965	Hs.307835	AL137698
MYL3	4.8	31.6	myosin, light chain 3, alkali; ventricular, skeletal, slow (MYL3), mRNA	NM_000258	Hs.517939	BC009790
PDE8B	4.8	2.1	phosphodiesterase 8B (PDE8B), transcript variant 1, mRNA	NM_003719	Hs.584830	NM_003719
TIMP3	4.8	5.6	TIMP metallopeptidase inhibitor 3 (Sorsby fundus dystrophy, pseudoinflammatory) (TIMP3), mRNA	NM_000362	Hs.644633	NM_000362
AL080082	4.8	3.1	mRNA; cDNA DKFZp564G1162 (from clone DKFZp564G1162).	AL080082	Hs.598166	AL080082
THC2543854	4.8	7.4	ALU4_HUMAN (P39191) Alu subfamily SB2 sequence contamination warning entry, partial (40%)	THC2543854	Unknown	
AK027158	4.8	5.9	cDNA: FLJ23505 fis, clone LNG03017.	AK027158	Unknown	
FAM118A	4.8	2.2	Protein FAM118A.	ENST00000216214	Unknown	
AU184995	4.8	2.9	AU184995 AU184995 T-cell library (Sugita Y) cDNA clone B00751-019, mRNA sequence	AU184995	Hs.533977	S73591
ST3GAL6	4.8	2.7	ST3 beta-galactoside alpha-2,3-sialyltransferase 6 (ST3GAL6), mRNA	NM_006100	Hs.148716	CR749468
BU567832	4.8	5.1	AGENCOURT_10399047 NIH_MGC_82 cDNA clone IMAGE:6614537 5', mRNA sequence	BU567832	Hs.634599	BU567832
CCNB3	4.8	3.3	cyclin B3 (CCNB3), transcript variant 3, mRNA	NM_033031	Hs.130310	AJ416458
TRIM9	4.8	7.7	tripartite motif-containing 9 (TRIM9), transcript variant 1, mRNA	NM_015163	Hs.654750	BC063872
FAM33A	4.8	1.5	family with sequence similarity 33, member A (FAM33A), mRNA	NM_182620	Hs.463607	NM_182620
BC041389	4.8	4.2	cDNA clone IMAGE:5276307.	BC041389	Unknown	
SLC26A10	4.8	2.8	cDNA FLJ16735 fis, clone BRACE2005457, weakly similar to PENDRIN.	AK122981	Unknown	
EPB41L5	4.8	2.7	erythrocyte membrane protein band 4.1 like 5 (EPB41L5), mRNA	NM_020909	Hs.654802	BC054508
ABAT	4.8	4.2	4-aminobutyrate aminotransferase (ABAT), nuclear gene encoding mitochondrial protein, transcript variant 2, mRNA	NM_000663	Hs.336768	NM_000663
AQP10	4.8	2.1	aquaporin 10 (AQP10), mRNA	NM_080429	Hs.259048	CR614330
AI570381	4.8	5.2	AI570381 to78g05.x1 NCI_CGAP_Gas4 cDNA clone IMAGE:2184440 3', mRNA sequence	AI570381	Hs.434966	AK125904
RND3	4.8	2.3	Rho family GTPase 3 (RND3), mRNA	NM_005168	Hs.6838	X97758
AL133627	4.8	1.7	mRNA; cDNA DKFZp434K0722 (from clone DKFZp434K0722).	AL133627	Hs.245869	AL133627
NFYC	4.8	2.4	nuclear transcription factor Y, gamma (NFYC), mRNA	NM_014223	Hs.233458	AK055329
NBPF1	4.8	3.6	neuroblastoma breakpoint family, member 1, mRNA (cDNA clone IMAGE:3896523), complete cds.	BC034418	Hs.515947	BX538005
ONECUT2	4.8	4.2	one cut domain, family member 2 (ONECUT2), mRNA	NM_004852	Hs.194725	NM_004852
SERTAD2	4.8	4.4	SERTA domain containing 2 (SERTAD2), mRNA	NM_014755	Hs.693696	NM_014755
FLJ25439	4.8	4.2	hypothetical protein FLJ25439, mRNA (cDNA clone IMAGE:5264902), partial cds.	BC032871	Hs.435742	BC032871
CLK1	4.8	2.4	CDC-like kinase 1 (CLK1), transcript variant 1, mRNA	NM_004071	Hs.433732	NM_004071
THC2653459	4.8	2.5	AC134346 hypothetcial protein {Oryza sativa (japonica cultivar-group)} (exp=0; wgp=1; cg=0), partial (6%)	THC2653459	Unknown	
GLT8D1	4.8	1.6	glycosyltransferase 8 domain containing 1 (GLT8D1), transcript variant 3, mRNA	NM_001010983	Hs.297304	NM_001010983
ZDHHC11	4.8	1.8	zinc finger, DHHC-type containing 11 (ZDHHC11), mRNA	NM_024786	Hs.659832	AK024233
A_24_P174063	4.8	2.6	A_24_P174063	A_24_P174063	Unknown	
PLAGL1	4.8	2.2	pleiomorphic adenoma gene-like 1 (PLAGL1), transcript variant 2, mRNA	NM_006718	Hs.444975	CR749329
BX104493	4.8	2.0	BX104493 Soares fetal liver spleen 1NFLS cDNA clone IMAGp998E24531, mRNA sequence	BX104493	Hs.15833	BX104493
THC2532209	4.8	11.3	THC2532209	THC2532209	Unknown	
A_32_P113462	4.8	18.9	A_32_P113462	A_32_P113462	Unknown	
CTA-246H3.1	4.8	1.9	similar to omega protein (LOC91353), mRNA	NM_001013618	Hs.567636	NM_001013618
EYA4	4.8	25.3	eyes absent homolog 4 (Drosophila) (EYA4), transcript variant 1, mRNA	NM_004100	Hs.661127	NM_004100
GREB1	4.8	2.7	GREB1 protein (GREB1), transcript variant a, mRNA	NM_014668	Hs.467733	NM_014668
AMOT	4.8	10.5	Angiomotin.	ENST00000304758	Unknown	
CTTNBP2	4.8	2.1	cortactin binding protein 2 (CTTNBP2), mRNA	NM_033427	Hs.592285	NM_033427
C11orf57	4.8	2.3	chromosome 11 open reading frame 57 (C11orf57), mRNA	NM_018195	Hs.195060	NM_018195
DNAJC1	4.8	1.7	DnaJ (Hsp40) homolog, subfamily C, member 1 (DNAJC1), mRNA	NM_022365	Hs.499000	BC110894
RPL15	4.8	1.7	ribosomal protein L15 (RPL15), mRNA	NM_002948	Hs.381219	BC081565
C12orf22	4.8	1.9	chromosome 12 open reading frame 22 (C12orf22), mRNA	NM_030809	Hs.524425	AJ298133
THC2703681	4.8	5.4	Q9BVD9_HUMAN (Q9BVD9) FTO protein, partial (16%)	THC2703681	Unknown	
PHTF2	4.8	1.7	putative homeodomain transcription factor 2 (PHTF2), mRNA	NM_020432	Hs.203965	AK225854

ZBTB1	4.8	1.9	zinc finger and BTB domain containing 1 (ZBTB1), mRNA	NM_014950	Hs.655536	AB023214
FBXO25	4.8	2.5	F-box protein 25 (FBXO25), transcript variant 1, mRNA	NM_183421	Hs.438454	NM_183421
FAM11A	4.8	1.7	family with sequence similarity 11, member A (FAM11A), mRNA	NM_032508	Hs.522172	AK128688
WDR19	4.8	1.8	WD repeat domain 19 (WDR19), mRNA	NM_025132	Hs.438482	NM_025132
CDH18	4.8	4.2	cadherin 18, type 2 (CDH18), mRNA	NM_004934	Hs.317632	BC031051
L3MBTL3	4.8	2.0	l(3)mbt-like 3 (Drosophila) (L3MBTL3), transcript variant 1, mRNA	NM_032438	Hs.658051	NM_032438
BX648831	4.8	3.2	mRNA; cDNA DKFZp686J06116 (from clone DKFZp686J06116).	BX648831	Hs.49889	BX648831
NAT8	4.8	5.7	N-acetyltransferase 8 (NAT8), mRNA	NM_003960	Hs.14637	BQ644205
HSF2	4.8	2.0	heat shock transcription factor 2 (HSF2), mRNA	NM_004506	Hs.158195	BC112323
GSTM1	4.8	4.5	glutathione S-transferase M1 (GSTM1), transcript variant 2, mRNA	NM_146421	Hs.301961	BQ880398
THC2661861	4.8	1.9	THC2661861	THC2661861	Unknown	
A_24_P408981	4.8	2.0	A_24_P408981	A_24_P408981	Unknown	
CHD6	4.8	1.4	chromodomain helicase DNA binding protein 6 (CHD6), mRNA	NM_032221	Hs.371979	NM_032221
ARMCX1	4.8	1.5	armadillo repeat containing, X-linked 1 (ARMCX1), mRNA	NM_016608	Hs.9728	AB039670
A_24_P33385	4.8	1.5	A_24_P33385	A_24_P33385	Unknown	
A_23_P357504	4.8	2.1	A_23_P357504	A_23_P357504	Unknown	
USP16	4.8	1.6	ubiquitin specific peptidase 16 (USP16), transcript variant 3, mRNA	NM_001032410	Hs.99819	NM_001032410
DCN	4.8	10.8	decorin (DCN), transcript variant A1, mRNA	NM_001920	Hs.694789	NM_001920
THC2634465	4.8	2.2	THC2634465	THC2634465	Unknown	
SNX4	4.8	1.8	sorting nexin 4 (SNX4), mRNA	NM_003794	Hs.507243	NM_003794
SEC31A	4.8	2.1	SEC31 homolog A (S. cerevisiae) (SEC31A), transcript variant 5, mRNA	NM_001077207	Hs.370024	AK128047
AJ295982	4.8	2.9	mRNA for hypothetical protein (ORF1), clone Telethon(Italy_B41)_Strait14635_FL303.	AJ295982	Hs.668296	AJ295982
ICK	4.8	2.0	intestinal cell (MAK-like) kinase (ICK), transcript variant 2, mRNA	NM_016513	Hs.417022	NM_016513
MGC42090	4.8	6.6	hypothetical protein MGC42090 (MGC42090), mRNA	NM_152774	Hs.487670	BX647256
LPIN1	4.8	2.3	lipin 1 (LPIN1), mRNA	NM_145693	Hs.467740	AK127039
RAMP1	4.8	5.0	receptor (G protein-coupled) activity modifying protein 1 (RAMP1), mRNA	NM_005855	Hs.471783	BG036385
ANKH	4.8	2.4	ankylosis, progressive homolog (mouse) (ANKH), mRNA	NM_054027	Hs.156727	NM_054027
FBXO32	4.8	13.0	F-box protein 32 (FBXO32), transcript variant 1, mRNA	NM_058229	Hs.403933	NM_058229
DYRK4	4.8	1.8	dual-specificity tyrosine-(Y)-phosphorylation regulated kinase 4 (DYRK4), mRNA	NM_003845	Hs.439530	NM_003845
EMID1	4.8	2.2	EMI domain containing 1 (EMID1), mRNA	NM_133455	Hs.289106	BC046358
CRB1	4.8	17.4	crumbs homolog 1 (Drosophila) (CRB1), mRNA	NM_201253	Hs.126135	BX640729
AK022110	4.8	2.1	cDNA FLJ12048 fis, clone HEMBB1001990.	AK022110	Hs.700615	BX537894
EDEM3	4.8	2.5	ER degradation enhancer, mannosidase alpha-like 3 (EDEM3), mRNA	NM_025191	Hs.523811	AF288393
AHCTF1	4.8	1.7	AT hook containing transcription factor 1 (AHCTF1), mRNA	NM_015446	Hs.300887	NM_015446
BC032027	4.8	9.7	cDNA clone IMAGE:4825733.	BC032027	Hs.683656	BC032027
CTSB	4.8	2.1	cathepsin B (CTSB), transcript variant 2, mRNA	NM_147780	Hs.520898	NM_147780
IGF1	4.8	3.8	insulin-like growth factor 1 (somatomedin C) (IGF1), mRNA	NM_000618	Hs.160562	NM_000618
RNF19	4.8	2.2	ring finger protein 19 (RNF19), transcript variant 1, mRNA	NM_183419	Hs.292882	NM_183419
RND3	4.8	2.2	Rho family GTPase 3 (RND3), mRNA	NM_005168	Hs.6838	X97758
RP1-93H18.5	4.8	2.5	hypothetical protein LOC441168 (LOC441168), mRNA	NM_001010919	Hs.381220	NM_001010919
ZMYND11	4.8	2.3	zinc finger, MYND domain containing 11 (ZMYND11), transcript variant 1, [illegible]	NM_006624	Hs.292265	NM_006624
CREM	4.8	2.8	cAMP responsive element modulator (CREM), transcript variant 19, mRNA	NM_183013	Hs.200250	AB209533
ADH1A	4.8	12.2	alcohol dehydrogenase 1A (class I), alpha polypeptide (ADH1A), mRNA	NM_000667	Hs.654433	NM_000667
HES1	4.8	2.6	hairy and enhancer of split 1, (Drosophila) (HES1), mRNA	NM_005524	Hs.250666	NM_005524
IFT80	4.8	2.0	intraflagellar transport 80 homolog (Chlamydomonas) (IFT80), mRNA	NM_020800	Hs.478095	NM_020800
ST3GAL6	4.8	2.6	ST3 beta-galactoside alpha-2,3-sialyltransferase 6 (ST3GAL6), mRNA	NM_006100	Hs.148716	CR749468
CCDC121	4.8	2.7	coiled-coil domain containing 121 (CCDC121), mRNA	NM_024584	Hs.21081	AK125354
F5	4.8	2.7	coagulation factor V (proaccelerin, labile factor) (F5), mRNA	NM_000130	Hs.30054	NM_000130
MDM4	4.8	1.7	Mdm4, transformed 3T3 cell double minute 4, p53 binding protein (mouse) (MDM4), mRNA	NM_002393	Hs.658187	BX640923
DLX2	4.8	7.5	distal-less homeobox 2 (DLX2), mRNA	NM_004405	Hs.419	AB208823
A_24_P118874	4.8	1.4	A_24_P118874	A_24_P118874	Unknown	
MTHFR	4.8	2.6	5,10-methylenetetrahydrofolate reductase (NADPH) (MTHFR), mRNA	NM_005957	Hs.214142	NM_005957
ZNF629	4.8	1.6	DNA-binding protein (Fragment).	ENST00000262525	Unknown	
C16orf30	4.8	13.4	chromosome 16 open reading frame 30 (C16orf30), mRNA	NM_024600	Hs.459652	AY676494

MEOX2	4.8	14.9	mesenchyme homeobox 2 (MEOX2), mRNA	NM_005924	Hs.170355	NM_005924
ADC	4.8	2.1	arginine decarboxylase (ADC), mRNA	NM_052998	Hs.101807	AL832640
DECR1	4.8	2.3	2,4-dienoyl CoA reductase 1, mitochondrial (DECR1), nuclear gene encoding mitochondrial protein, mRNA	NM_001359	Hs.492212	BM920635
THC2739211	4.8	2.8	THC2739211	THC2739211	Unknown	
LAMC1	4.8	2.8	laminin, gamma 1 (formerly LAMB2) (LAMC1), mRNA	NM_002293	Hs.609663	NM_002293
C9orf125	4.8	1.7	C9orf125 protein (Fragment).	ENST00000374851	Unknown	
ZC3H11A	4.7	3.3	zinc finger CCCH-type containing 11A (ZC3H11A), mRNA	NM_014827	Hs.532399	CR627439
LOC731681	4.7	1.6	PREDICTED: similar to 60S ribosomal protein L9 (LOC731681), mRNA	XR_015944	Hs.646365	XR_015944
HIST2H2BE	4.7	2.1	histone cluster 2, H2be (HIST2H2BE), mRNA	NM_003528	Hs.2178	BC069193
LCP2	4.7	1.8	lymphocyte cytosolic protein 2 (SH2 domain containing leukocyte protein of 76kDa) (LCP2), mRNA	NM_005565	Hs.304475	NM_005565
CCDC74B	4.7	2.0	coiled-coil domain containing 74B (CCDC74B), mRNA	NM_207310	Hs.29383	AL133619
SDCCAG8	4.7	3.9	serologically defined colon cancer antigen 8 (SDCCAG8), mRNA	NM_006642	Hs.591530	BX537630
THC2689181	4.7	4.0	Q9EQG5_MOUSE (Q9EQG5) Nedd4-binding brain specific protein BEAN (Fragment), partial (5%)	THC2689181	Unknown	
SATB1	4.7	1.7	special AT-rich sequence binding protein 1 (binds to nuclear matrix/scaffold-associating DNA's) (SATB1), mRNA	NM_002971	Hs.517717	AK127242
HSD17B11	4.7	2.3	hydroxysteroid (17-beta) dehydrogenase 11 (HSD17B11), mRNA	NM_016245	Hs.284414	NM_178135
LMBRD2	4.7	3.1	LMBR1 domain containing 2 (LMBRD2), mRNA	NM_001007527	Hs.294103	CR749399
TPT1	4.7	2.9	tumor protein, translationally-controlled 1 (TPT1), mRNA	NM_003295	Hs.374596	BG033621
IFI44L	4.7	4.8	interferon-induced protein 44-like (IFI44L), mRNA	NM_006820	Hs.389724	AK223087
MPPED1	4.7	5.1	metallophosphoesterase domain containing 1 (MPPED1), mRNA	NM_001044370	Hs.592198	NM_001044370
NTRK2	4.7	5.6	mRNA; cDNA DKFZp686D1394 (from clone DKFZp686D1394).	BX649001	Hs.494312	NM_001018065
THC2614136	4.7	3.2	THC2614136	THC2614136	Unknown	
THC2644389	4.7	18.3	THC2644389	THC2644389	Unknown	
THC2713545	4.7	2.3	Q6TDT1_HUMAN (Q6TDT1) Protein transactivated by hepatitis B virus E antigen, partial (34%)	THC2713545	Unknown	
TMEM65	4.7	3.3	transmembrane protein 65 (TMEM65), mRNA	NM_194291	Hs.187646	NM_194291
A_32_P166653	4.7	8.8	A_32_P166653	A_32_P166653	Unknown	
THC2673830	4.7	2.1	RING1_RAT Polycomb complex protein RING1 (RING finger protein 1). {Rattus norvegicus} (exp=-1; wgp=-1; cg=-1), partial (7%)	THC2673830	Unknown	
WIF1	4.7	2.0	WNT inhibitory factor 1 (WIF1), mRNA	NM_007191	Hs.284122	AY358344
A_32_P6107	4.7	2.0	A_32_P6107	A_32_P6107	Unknown	
RAB40B	4.7	3.3	RAB40B, member RAS oncogene family (RAB40B), mRNA	NM_006822	Hs.484068	AK095782
CR603951	4.7	3.5	full-length cDNA clone CS0DM011YC22 of Fetal liver of (human).	CR603951	Hs.632886	BC032409
IQCG	4.7	2.4	IQ motif containing G (IQCG), mRNA	NM_032263	Hs.591675	AK095094
BC038559	4.7	4.6	Homo sapiens, clone IMAGE:3851018, mRNA.	BC038559	Hs.529860	AK130729
WHDC1	4.7	1.9	cDNA FLJ44939 fis, clone BRAMY3018754, weakly similar to Mus musculus junction-mediating and regulatory protein (Jmy-pending).	AK126887	Hs.377360	NM_001080435
PURB	4.7	2.4	purine-rich element binding protein B (PURB), mRNA	NM_033224	Hs.349150	NM_033224
LOC440295	4.7	1.9	hypothetical protein LOC440295 (LOC440295), mRNA	NM_198181	Hs.534900	XM_001130543
PLCH2	4.7	3.2	phospholipase C, eta 2 (PLCH2), mRNA	NM_014638	Hs.170156	AK122589
THC2701763	4.7	3.1	Q9F8M7_CARHY (Q9F8M7) DTDP-glucose 4,6-dehydratase (Fragment), partial (11%)	THC2701763	Unknown	
CR610169	4.7	2.6	full-length cDNA clone CS0DF004YE18 of Fetal brain of (human).	CR610169	Hs.323349	CF457366
C22orf15	4.7	1.7	chromosome 22 open reading frame 15 (C22orf15), mRNA	NM_182520	Hs.116254	NM_182520
MXRA8	4.7	8.7	matrix-remodelling associated 8 (MXRA8), mRNA	NM_032348	Hs.558570	AK095966
BAI2	4.7	2.5	brain-specific angiogenesis inhibitor 2 (BAI2), mRNA	NM_001703	Hs.524138	NM_001703
THC2636529	4.7	4.4	THC2636529	THC2636529	Unknown	
SPEG	4.7	2.4	cDNA FLJ30825 fis, clone FEBRA2001706, highly similar to Human APEG-1 mRNA.	AK055387	Unknown	
CORO6	4.7	1.7	coronin 6 (CORO6), mRNA	NM_032854	Hs.143046	AK094683
C17orf85	4.7	2.1	chromosome 17 open reading frame 85 (C17orf85), mRNA	NM_018553	Hs.120963	AK125048
F5	4.7	3.5	coagulation factor V (proaccelerin, labile factor) (F5), mRNA	NM_000130	Hs.30054	NM_000130
MDM1	4.7	1.7	Mdm4, transformed 3T3 cell double minute 1, p53 binding protein (mouse) (MDM1), transcript variant 2, mRNA	NM_020128	Hs.655702	BC028355
DGKI	4.7	7.8	diacylglycerol kinase, iota (DGKI), mRNA	NM_004717	Hs.242947	AB209167
LOC730902	4.7	1.4	PREDICTED: similar to 60S ribosomal protein L7 (LOC730902), mRNA	XR_015600	Hs.653167	XR_015600
C3orf50	4.7	2.2	cDNA clone IMAGE:4156795.	BC011266	Hs.478158	BC011266
PPP1R3C	4.7	5.0	protein phosphatase 1, regulatory (inhibitor) subunit 3C (PPP1R3C), mRNA	NM_005398	Hs.303090	BX537399

BQ777622	4.7	14.7	BQ777622 il38g05.x1 HR85 islet cDNA clone IMAGE:6032433 3', mRNA sequence	BQ777622	Hs.518921	NM_001033047
ZC3H12C	4.7	2.4	LOH11CR1B gene, loss of heterozygosity, 11, chromosomal region 1 gene B product.	ENST00000278590	Unknown	
RHOU	4.7	2.7	ras homolog gene family, member U (RHOU), mRNA	NM_021205	Hs.647774	NM_021205
STK40	4.7	2.6	serine/threonine kinase 40 (STK40), mRNA	NM_032017	Hs.471768	AK024504
ROBO2	4.7	19.8	roundabout, axon guidance receptor, homolog 2 (Drosophila) (ROBO2), mRNA	NM_002942	Hs.13305	BX648828
KIAA1875	4.7	1.5	KIAA1875 (KIAA1875), mRNA	NM_032529	Unknown	
KIRREL	4.7	2.3	cDNA FLJ33235 fis, clone ASTRO2002202.	AK090554	Hs.609291	AK090554
THC2693401	4.7	4.9	THC2693401	THC2693401	Unknown	
LOC152573	4.7	7.6	Homo sapiens, clone IMAGE:4477067, mRNA, partial cds.	BC012029	Hs.370904	NM_001080505
LOC387763	4.7	4.3	hypothetical LOC387763, mRNA (cDNA clone IMAGE:6272440), partial cds.	ENST00000339446	Unknown	
NRXN1	4.7	3.1	mRNA for KIAA0578 protein, partial cds.	AB011150	Hs.637685	BC150247
ZBTB34	4.7	1.6	mRNA for KIAA1993 protein.	AB082524	Hs.177633	NM_001099270
MGC33657	4.7	8.4	similar to hypothetical protein (MGC33657), mRNA	NM_001029996	Hs.496753	AK126306
GALNT9	4.7	3.3	UDP-N-acetyl-alpha-D-galactosamine:polypeptide N-acetylgalactosaminyltransferase 9 (GalNAc-T9) (GALNT9), mRNA	NM_021808	Hs.301062	AF458594
LOC643974	4.7	1.6	PREDICTED: similar to 60S ribosomal protein L6 (TAX-responsive enhancer element-binding protein 107) (TAXREB107) (Neoplasm-related protein C140) (LOC643974), mRNA	XR_018643	Hs.648000	XR_018643
PRKAR1A	4.7	2.6	protein kinase, cAMP-dependent, regulatory, type I, alpha (tissue specific extinguisher 1) (PRKAR1A), transcript variant 3, mRNA	NM_212472	Hs.280342	CR749311
AK123757	4.7	20.8	cDNA FLJ41763 fis, clone IMR322005293.	AK123757	Hs.573143	AK123757
NDFIP1	4.7	1.9	Nedd4 family interacting protein 1 (NDFIP1), mRNA	NM_030571	Hs.9788	AK124884
HMGN2	4.7	1.5	high-mobility group nucleosomal binding domain 2 (HMGN2), mRNA	NM_005517	Hs.181163	BC110390
THC2574620	4.7	3.7	Q6DQW6_BORTU (Q6DQW6) Alanine racemase (Fragment), partial (11%)	THC2574620	Unknown	
C2orf39	4.7	2.9	chromosome 2 open reading frame 39 (C2orf39), mRNA	NM_145038	Hs.393714	AK128286
PNMA2	4.7	2.6	paraneoplastic antigen MA2 (PNMA2), mRNA	NM_007257	Hs.591838	BC036489
FLJ30092	4.7	1.4	mRNA for KIAA0614 protein, partial cds.	AB014514	Hs.695995	AB014514
FLJ40298	4.7	3.6	hypothetical protein FLJ40298 (FLJ40298), mRNA	NM_173486	Unknown	
FAP	4.7	48.7	fibroblast activation protein, alpha (FAP), mRNA	NM_004460	Hs.654370	U09278
FMN2	4.7	3.0	formin 2 (FMN2), mRNA	NM_020066	Hs.24889	NM_020066
ENST00000344441	4.7	2.8	MIA3 protein (Fragment).	ENST00000344441	Unknown	
THC2669096	4.7	14.3	THC2669096	THC2669096	Unknown	
MLLT6	4.7	2.4	mRNA; cDNA DKFZp686J1719 (from clone DKFZp686J1719).	AL832481	Hs.91531	NM_005937
DSC3	4.7	5.4	desmocollin 3 (DSC3), transcript variant Dsc3b, mRNA	NM_024423	Hs.41690	NM_024423
CCDC74B	4.7	2.1	coiled-coil domain containing 74B (CCDC74B), mRNA	NM_207310	Hs.29383	AL133619
THC2750786	4.7	3.5	CI096_HUMAN (Q8NE28) Protein kinase-like protein C9orf96, partial (29%)	THC2750786	Unknown	
LOC643669	4.7	1.7	PREDICTED: hypothetical LOC643669, transcript variant 1 (LOC643669), mRNA	XM_933621	Hs.355357	XM_933621
NME7	4.7	2.6	non-metastatic cells 7, protein expressed in (nucleoside-diphosphate kinase) (NME7), transcript variant 1, mRNA	NM_013330	Hs.699593	AB209049
ANKRD42	4.7	2.4	ankyrin repeat domain 42 (ANKRD42), mRNA	NM_182603	Hs.503438	BC045621
HISPPD1	4.7	1.8	histidine acid phosphatase domain containing 1 (HISPPD1), mRNA	NM_015216	Hs.212046	AB007893
HOXA4	4.7	53.5	homeobox A4 (HOXA4), mRNA	NM_002141	Hs.654466	NM_002141
CYB5D2	4.7	2.1	cytochrome b5 domain containing 2 (CYB5D2), mRNA	NM_144611	Hs.513871	BC051697
CMPK	4.7	3.5	cytidylate kinase (CMPK), mRNA	NM_016308	Hs.11463	AK025258
MB	4.7	12.3	myoglobin (MB), transcript variant 2, mRNA	NM_203377	Hs.517586	BF670653
RP11-130N24.1	4.7	3.0	KIAA2022 protein (KIAA2022), mRNA	NM_001008537	Hs.124128	AY563507
KCNK3	4.7	3.0	potassium channel, subfamily K, member 3 (KCNK3), mRNA	NM_002246	Hs.645288	NM_002246
A_32_P128781	4.7	1.5	A_32_P128781	A_32_P128781	Unknown	
MLF1	4.7	2.2	myeloid leukemia factor 1 (MLF1), mRNA	NM_022443	Hs.85195	BX641078
LOC390595	4.7	1.9	cDNA FLJ13740 fis, clone PLACE3000199.	AK023802	Unknown	
AK093982	4.7	6.1	cDNA FLJ36663 fis, clone UTERU2002826.	AK093982	Hs.125056	AK093982
FLJ10081	4.7	1.9	hypothetical protein FLJ10081 (FLJ10081), mRNA	NM_017991	Hs.516341	NM_017991
KIAA0350	4.7	1.9	KIAA0350 (KIAA0350), mRNA	NM_015226	Hs.35490	NM_015226
MATN2	4.7	2.5	matrilin 2 (MATN2), transcript variant 2, mRNA	NM_030583	Hs.189445	BX648291
RPL36A	4.7	1.6	ribosomal protein L36a (RPL36A), mRNA	NM_021029	Hs.432485	CR601778
ITGAV	4.7	2.6	integrin, alpha V (vitronectin receptor, alpha polypeptide, antigen CD51) (ITGAV), mRNA	NM_002210	Hs.436873	NM_002210

MKLN1	4.7	1.9	muskelin 1, intracellular mediator containing kelch motifs (MKLN1), mRNA	NM_013255	Hs.44693	NM_013255
RUFY2	4.7	1.7	RUN and FYVE domain containing 2 (RUFY2), transcript variant 1, mRNA	NM_017987	Hs.653144	NM_017987
E2F2	4.7	2.1	E2F transcription factor 2 (E2F2), mRNA	NM_004091	Hs.194333	NM_004091
AF119905	4.7	4.8	PRO2853 mRNA, complete cds.	AF119905	Unknown	
LOC647500	4.7	3.7	PREDICTED: similar to phosphodiesterase 4D interacting protein isoform 1 (LOC647500), mRNA	XM_001128547	Hs.656830	CR610073
ERCC5	4.7	1.7	excision repair cross-complementing rodent repair deficiency, complementation group 5 (xeroderma pigmentosum, complementation group G (Cockayne syndrome)) (ERCC5), mRNA	NM_000123	Hs.258429	NM_000123
THC2770932	4.7	4.9	THC2770932	THC2770932	Unknown	
NFATC1	4.7	2.3	nuclear factor of activated T-cells, cytoplasmic, calcineurin-dependent 1 (NFATC1), transcript variant 3, mRNA	NM_172387	Hs.534074	NM_006162
THC2572108	4.7	1.5	Q495B5_HUMAN (Q495B5) C15orf21 protein, partial (75%)	THC2572108	Unknown	
WSB1	4.7	2.0	WD repeat and SOCS box-containing 1 (WSB1), transcript variant 1, mRNA	NM_015626	Hs.446017	NM_015626
MTHFR	4.7	2.8	5,10-methylenetetrahydrofolate reductase (NADPH) (MTHFR), mRNA	NM_005957	Hs.214142	NM_005957
REL	4.7	1.5	v-rel reticuloendotheliosis viral oncogene homolog (avian) (REL), mRNA	NM_002908	Hs.631886	NM_002908
TPBG	4.7	2.3	trophoblast glycoprotein (TPBG), mRNA	NM_006670	Hs.82128	NM_006670
LOC645277	4.7	2.0	PREDICTED: hypothetical LOC645277 (LOC645277), mRNA	XM_928321	Hs.282811	BX379759
SCUBE1	4.7	2.4	signal peptide, CUB domain, EGF-like 1 (SCUBE1), mRNA	NM_173050	Hs.133995	NM_173050
GJB6	4.7	3.5	gap junction protein, beta 6 (connexin 30) (GJB6), mRNA	NM_006783	Hs.511757	AK075247
ALS2CR8	4.7	5.5	ALS2CR8 mRNA, complete cds, short form.	AB053310	Hs.444982	NM_024744
PIGY	4.7	1.8	phosphatidylinositol glycan anchor biosynthesis, class Y (PIGY), transcript variant 2, mRNA	NM_001042616	Hs.648007	NM_001042616
THC2649719	4.7	6.4	ALU2_HUMAN (P39189) Alu subfamily SB sequence contamination warning entry, partial (8%)	THC2649719	Unknown	
LOC653773	4.7	1.5	PREDICTED: similar to ribosomal protein L31 (LOC653773), mRNA	XR_017639	Hs.647888	XR_017639
C9orf6	4.7	1.9	chromosome 9 open reading frame 6 (C9orf6), mRNA	NM_017832	Hs.29276	BC051827
2-Sep	4.7	1.6	septin 2 (SEPT2), transcript variant 1, mRNA	NM_001008491	Hs.335057	NM_001008491
MON2	4.7	1.8	MON2 homolog (S. cerevisiae) (MON2), mRNA	NM_015026	Hs.389378	NM_015026
SCGN	4.7	2.3	secretagogin, EF-hand calcium binding protein (SCGN), mRNA	NM_006998	Hs.116428	NM_006998
WFDC1	4.7	7.3	WAP four-disulfide core domain 1 (WFDC1), mRNA	NM_021197	Hs.36688	AK128711
AKR1D1	4.7	3.7	aldo-keto reductase family 1, member D1 (delta 4-3-ketosteroid-5-beta-reductase) (AKR1D1), mRNA	NM_005989	Hs.201667	Z28339
PLXNA2	4.7	10.9	plexin A2 (PLXNA2), mRNA	NM_025179	Hs.497626	NM_025179
IER3IP1	4.6	1.9	immediate early response 3 interacting protein 1 (IER3IP1), mRNA	NM_016097	Hs.653122	AK027108
COPS4	4.6	1.6	COP9 constitutive photomorphogenic homolog subunit 4 (Arabidopsis) (COPS4), mRNA	NM_016129	Hs.190384	AK094238
A_23_P206741	4.6	3.9	A_23_P206741	A_23_P206741	Unknown	
BTBD1	4.6	1.8	BTB (POZ) domain containing 1 (BTBD1), transcript variant 1, mRNA	NM_025238	Hs.459149	NM_025238
AK123264	4.6	2.4	cDNA FLJ41270 fis, clone BRAMY2036387.	AK123264	Hs.445414	AK123264
STARD9	4.6	1.7	StAR-related lipid transfer protein 9 (StARD9) (START domain- containing protein 9) (Fragment).	ENST00000290607	Unknown	
KLF9	4.6	4.1	Kruppel-like factor 9 (KLF9), mRNA	NM_001206	Hs.150557	NM_001206
AK024516	4.6	2.7	cDNA: FLJ20863 fis, clone ADKA01804.	AK024516	Hs.657178	AK024516
AK130431	4.6	5.0	cDNA FLJ26921 fis, clone RCT04780.	AK130431	Hs.639694	AK130431
AW015426	4.6	5.6	UI-H-BI0-aat-h-12-0-UI.s1 NCI_CGAP_Sub1 cDNA clone IMAGE:2710535 3', mRNA sequence	AW015426	Hs.700076	AW015426
WT1	4.6	15.5	Wilms tumor 1 (WT1), transcript variant D, mRNA	NM_024426	Hs.591980	BC046461
C10orf84	4.6	1.7	chromosome 10 open reading frame 84 (C10orf84), mRNA	NM_022063	Hs.372309	AK023250
PDE1A	4.6	9.6	phosphodiesterase 1A, calmodulin-dependent (PDE1A), transcript variant 2, mRNA	NM_001003683	Hs.191046	NM_005019
HS3ST1	4.6	5.0	heparan sulfate (glucosamine) 3-O-sulfotransferase 1 (HS3ST1), mRNA	NM_005114	Hs.507348	AK096823
PEG10	4.6	2.4	paternally expressed 10 (PEG10), transcript variant 1, mRNA	NM_001040152	Hs.147492	NM_001040152
CSRP3	4.6	9.0	cysteine and glycine-rich protein 3 (cardiac LIM protein) (CSRP3), mRNA	NM_003476	Hs.83577	NM_003476
C1QTNF6	4.6	2.4	C1q and tumor necrosis factor related protein 6 (C1QTNF6), transcript variant 1, mRNA	NM_031910	Hs.22011	AK128125
U09197	4.6	37.4	Human 5.5 kb mRNA upregulated in retinoic acid treated HL-60 neutrophilic cells	U09197	Hs.180284	U09197
GBP4	4.6	3.0	guanylate binding protein 4 (GBP4), mRNA	NM_052941	Hs.409925	AL832576
HISPPD1	4.6	1.8	histidine acid phosphatase domain containing 1 (HISPPD1), mRNA	NM_015216	Hs.212046	AB007893
FLJ41603	4.6	5.3	FLJ41603 protein (FLJ41603), mRNA	NM_001001669	Hs.256206	NM_001001669
A_24_P101661	4.6	1.9	A_24_P101661	A_24_P101661	Unknown	
THC2551948	4.6	4.9	Q86TS6_HUMAN (Q86TS6) Full-length cDNA 5-PRIME end of clone CS0CAP004YO05 of Thymus of (human) (Fragment), partial (22%)	THC2551948	Unknown	

ATXN1	4.6	3.3	ataxin 1 (ATXN1), mRNA	NM_000332	Hs.434961	NM_000332
GPM6A	4.6	4.9	glycoprotein M6A (GPM6A), transcript variant 2, mRNA	NM_201591	Hs.75819	NM_201591
THC2637568	4.6	2.8	THC2637568	THC2637568	Unknown	
RAB2	4.6	2.1	Ras-related protein Rab-2A.	ENST00000262646	Unknown	
ZNF10	4.6	1.9	zinc finger protein 10 (ZNF10), mRNA	NM_015394	Hs.507355	BC024182
DPH3	4.6	1.8	CSL-type zinc finger-containing protein 2 (DelGEF-interacting protein 1) (DelGIP1).	ENST00000285082	Unknown	
TBL1XR1	4.6	2.0	transducin (beta)-like 1X-linked receptor 1, mRNA (cDNA clone IMAGE:5527421), complete cds.	BC048298	Hs.699293	BX648935
AKR1C1	4.6	2.9	aldo-keto reductase family 1, member C1 (dihydrodiol dehydrogenase 1; 20-alpha (3-alpha)-hydroxysteroid dehydrogenase) (AKR1C1), mRNA	NM_001353	Hs.460260	AK226067
THC2648584	4.6	2.1	THC2648584	THC2648584	Unknown	
GABRA2	4.6	6.0	gamma-aminobutyric acid (GABA) A receptor, alpha 2 (GABRA2), mRNA	NM_000807	Hs.116250	AK125179
MGC5457	4.6	3.2	hypothetical protein MGC5457, mRNA (cDNA clone MGC:5457 IMAGE:3450898), complete cds.	BC000988	Hs.661178	BC000988
FRZB	4.6	4.1	frizzled-related protein (FRZB), mRNA	NM_001463	Hs.128453	NM_001463
CR614803	4.6	4.2	full-length cDNA clone CS0DI053YO09 of Placenta Cot 25-normalized of (human).	CR614803	Hs.561040	CR614803
COL1A1	4.6	2.3	H.sapiens mRNA for prepro-alpha1(I) collagen.	Z74615	Hs.172928	Z74615
CR596712	4.6	3.8	full-length cDNA clone CS0DI028YF10 of Placenta Cot 25-normalized of (human).	CR596712	Hs.130652	XM_001131009
C20orf142	4.6	2.8	chromosome 20 open reading frame 142, mRNA (cDNA clone IMAGE:4933017), with apparent retained intron.	BC029662	Unknown	
FBLN5	4.6	3.8	fibulin 5 (FBLN5), mRNA	NM_006329	Hs.332708	BX537531
PBX4	4.6	2.4	pre-B-cell leukemia transcription factor 4 (PBX4), mRNA	NM_025245	Hs.466257	BC141859
LOC129881	4.6	4.7	hypothetical LOC129881, mRNA (cDNA clone MGC:151054 IMAGE:40125996), complete cds.	ENST00000284676	Unknown	
DUSP27	4.6	21.3	Novel protein.	ENST00000361200	Unknown	
BCDO2	4.6	20.8	beta-carotene dioxygenase 2 (BCDO2), transcript variant 1, mRNA	NM_031938	Hs.647227	NM_031938
DKFZP779L1068	4.6	2.1	cDNA clone IMAGE:5555490.	BC110326	Hs.440643	BC110326
CB049993	4.6	7.3	CB049993 NISC_gj14d12.y1 NCI_CGAP_Pr28 cDNA clone IMAGE:3271727 5', mRNA sequence	CB049993	Hs.303023	DB323009
CASC4	4.6	1.5	cancer susceptibility candidate 4 (CASC4), transcript variant 1, mRNA	NM_138423	Hs.512867	NM_138423
ZNF580	4.6	2.1	zinc finger protein 580 (ZNF580), transcript variant 1, mRNA	NM_016202	Hs.631551	AK123294
TM2D1	4.6	2.4	TM2 domain containing 1 (TM2D1), mRNA	NM_032027	Hs.656790	NM_032027
HOXB5	4.6	17.9	homeobox B5 (HOXB5), mRNA	NM_002147	Hs.654456	M92299
ENST00000378289	4.6	5.1	Artemis protein (EC 3.1.-.-) (DNA cross-link repair 1C protein) (SNM1- like protein) (A-SCID protein) (hSNM1C).	ENST00000378289	Unknown	
BXDC5	4.6	2.3	mRNA; cDNA DKFZp761G0415 (from clone DKFZp761G0415).	AL359584	Hs.481202	CR627468
KGFLP1	4.6	6.8	keratinocyte growth factor-like protein 1 (KGFLP1) mRNA, complete cds.	AY098593	Hs.439341	AY098593
DISC1	4.6	3.7	disrupted in schizophrenia 1 (DISC1), transcript variant L, mRNA	NM_018662	Hs.13318	NM_018662
ZBED5	4.6	1.4	zinc finger, BED-type containing 5 (ZBED5), mRNA	NM_021211	Unknown	
LOC253264	4.6	12.9	cDNA FLJ13383 fis, clone PLACE1001024.	AK023445	Hs.662111	AK023445
FLJ32065	4.6	1.8	hypothetical protein FLJ32065, mRNA (cDNA clone MGC:90301 IMAGE:5502453), complete cds.	BC073870	Unknown	
CCDC82	4.6	2.4	coiled-coil domain containing 82 (CCDC82), mRNA	NM_024725	Hs.525088	AK074306
IKIP	4.6	2.7	IKK interacting protein (IKIP), transcript variant 2, mRNA	NM_201612	Hs.252543	AJ539425
THC2642694	4.6	3.6	Q6TDT1_HUMAN (Q6TDT1) Protein transactivated by hepatitis B virus E antigen, partial (11%)	THC2642694	Unknown	
AK001846	4.6	3.4	cDNA FLJ10984 fis, clone PLACE1001810.	AK001846	Hs.373763	BX647784
CXCR4	4.6	2.6	chemokine (C-X-C motif) receptor 4 (CXCR4), transcript variant 1, mRNA	NM_001008540	Hs.593413	AF147204
THC2673265	4.6	2.7	Q6IHS4_DROME (Q6IHS4) HDC01261, partial (9%)	THC2673265	Unknown	
THC2551395	4.6	2.2	Q3SZ59_BOVIN (Q3SZ59) Ribosomal protein L36a, complete	THC2551395	Unknown	
SAMD11	4.6	5.7	sterile alpha motif domain containing 11 (SAMD11), mRNA	NM_152486	Hs.335293	NM_152486
RPS3A	4.6	1.6	ribosomal protein S3A (RPS3A), mRNA	NM_001006	Hs.356572	BI087817
ALPK1	4.6	2.6	alpha-kinase 1 (ALPK1), mRNA	NM_025144	Unknown	
CRYBA1	4.6	10.1	crystallin, beta A1 (CRYBA1), mRNA	NM_005208	Hs.46275	NM_005208
THC2646425	4.6	6.4	THC2646425	THC2646425	Unknown	
HMGCL	4.6	2.3	3-hydroxymethyl-3-methylglutaryl-Coenzyme A lyase (hydroxymethylglutaricaciduria) (HMGCL), mRNA	NM_000191	Hs.533444	AK122801
COBLL1	4.6	2.4	COBL-like 1 (COBLL1), mRNA	NM_014900	Hs.470457	BX649112
TBX1	4.6	22.2	T-box 1 (TBX1), transcript variant C, mRNA	NM_080647	Hs.173984	AF373867
OR7E104P	4.6	1.9	cDNA FLJ46084 fis, clone TESTI2006543.	AK127969	Hs.568153	AK127969
LXN	4.6	2.3	latexin (LXN), mRNA	NM_020169	Hs.478067	NM_020169

SUV420H1	4.6	2.0	suppressor of variegation 4-20 homolog 1 (Drosophila) (SUV420H1), transcript variant 1, mRNA	NM_017635	Hs.632120	NM_017635
GRAMD1C	4.6	2.2	GRAM domain containing 1C (GRAMD1C), mRNA	NM_017577	Hs.24583	AL133661
SCN3A	4.6	7.7	sodium channel, voltage-gated, type III, alpha (SCN3A), mRNA	NM_006922	Hs.435274	NM_006922
AL833114	4.6	8.0	mRNA; cDNA DKFZp313L2229 (from clone DKFZp313L2229).	AL833114	Hs.684006	AL833114
TMEM166	4.6	2.9	transmembrane protein 166 (TMEM166), mRNA	NM_032181	Hs.302346	BC063016
ID3	4.6	3.2	inhibitor of DNA binding 3, dominant negative helix-loop-helix protein (ID3), mRNA	NM_002167	Hs.76884	AK225965
ARMCX6	4.6	1.8	armadillo repeat containing, X-linked 6 (ARMCX6), transcript variant 1, mRNA	NM_019007	Hs.83530	AK097018
SMARCA1	4.6	3.3	SWI/SNF related, matrix associated, actin dependent regulator of chromatin, subfamily a, member 1 (SMARCA1), transcript variant 1, mRNA	NM_003069	Hs.152292	NM_003069
ATP9B	4.6	1.5	ATPase, Class II, type 9B (ATP9B), mRNA	NM_198531	Hs.465475	NM_198531
RGS2	4.6	3.0	regulator of G-protein signalling 2, 24kDa (RGS2), mRNA	NM_002923	Hs.78944	BC042755
AK054895	4.6	2.6	cDNA FLJ30333 fis, clone BRACE2007262.	AK054895	Hs.664520	AK054895
ZNF432	4.6	3.5	Zinc finger protein 432.	ENST00000221315	Unknown	
PGBD3	4.6	2.0	piggyBac transposable element derived 3 (PGBD3), mRNA	NM_170753	Hs.654449	NM_000124
THC2521656	4.6	1.5	Q3KI19_PSEPF (Q3KI19) Nitrilase/cyanide hydratase and apolipoprotein N-acyltransferase, partial (5%)	THC2521656	Unknown	
APRIN	4.6	2.2	androgen-induced proliferation inhibitor (APRIN), mRNA	NM_015032	Hs.699308	AL137201
THC2690338	4.6	4.3	Q9SL08_ARATH (Q9SL08) Expressed protein, partial (5%)	THC2690338	Unknown	
CXCR4	4.6	2.6	chemokine (C-X-C motif) receptor 4 (CXCR4), transcript variant 1, mRNA	NM_001008540	Hs.593413	AF147204
RPS6KA3	4.6	2.8	ribosomal protein S6 kinase, 90kDa, polypeptide 3 (RPS6KA3), mRNA	NM_004586	Hs.445387	NM_004586
HSD11B1L	4.6	2.2	hydroxysteroid (11-beta) dehydrogenase 1-like (HSD11B1L), transcript variant b, mRNA	NM_198706	Hs.631840	CR622145
EFHB	4.6	3.3	EF-hand domain family, member B (EFHB), mRNA	NM_144715	Hs.670883	BC028198
CPM	4.6	6.3	carboxypeptidase M (CPM), transcript variant 1, mRNA	NM_001874	Hs.654387	NM_001874
AF131762	4.6	2.4	clone 25218 mRNA sequence.	AF131762	Hs.17569	AL833176
JMJD2D	4.6	1.6	jumonji domain containing 2D (JMJD2D), mRNA	NM_018039	Hs.503598	NM_018039
AL109695	4.6	3.6	mRNA full length insert cDNA clone EUROIMAGE 39820.	AL109695	Hs.311187	AF205074
KIAA1217	4.6	2.0	KIAA1217 (KIAA1217), mRNA	NM_019590	Hs.445885	NM_019590
POLK	4.6	1.6	polymerase (DNA directed) kappa (POLK), mRNA	NM_016218	Hs.135756	BC041798
XIST	4.6	8.1	X (inactive)-specific transcript (XIST) on chromosome X	NR_001564	Unknown	
C9orf93	4.6	1.9	chromosome 9 open reading frame 93 (C9orf93), mRNA	NM_173550	Hs.17267	CR936775
SETBP1	4.6	5.6	SET binding protein 1 (SETBP1), mRNA	NM_015559	Hs.435458	BC146776
CRIP1	4.6	6.0	cysteine-rich protein 1 (intestinal) (CRIP1), mRNA	NM_001311	Hs.70327	AK092741
LOC643837	4.6	13.7	full-length cDNA clone CS0DF031YH08 of Fetal brain of (human).	CR594200	Hs.133183	BC086872
ZNF266	4.6	2.0	zinc finger protein 266 (ZNF266), mRNA	NM_006631	Hs.656185	AB095928
BIRC3	4.6	9.4	baculoviral IAP repeat-containing 3 (BIRC3), transcript variant 1, mRNA	NM_001165	Hs.127799	NM_001165
ANKRA2	4.6	1.6	ankyrin repeat, family A (RFXANK-like), 2 (ANKRA2), mRNA	NM_023039	Hs.239154	AF251051
PDE1A	4.6	4.5	phosphodiesterase 1A, calmodulin-dependent (PDE1A), transcript variant 2, mRNA	NM_001003683	Hs.191046	NM_005019
CDH6	4.6	5.0	Cadherin-6 precursor (Kidney-cadherin) (K-cadherin).	ENST00000382216	Unknown	
AK128751	4.6	1.6	cDNA FLJ44726 fis, clone BRACE3024444.	AK128751	Hs.592928	BC007841
A_24_P84408	4.6	1.7	A_24_P84408	A_24_P84408	Unknown	
FIGF	4.6	26.6	c-fos induced growth factor (vascular endothelial growth factor D) (FIGF), mRNA	NM_004469	Hs.11392	NM_004469
PDGFRA	4.6	3.5	platelet-derived growth factor receptor, alpha polypeptide, mRNA (cDNA clone IMAGE:4043984), complete cds.	BC015186	Hs.74615	NM_006206
ENST00000356216	4.6	10.5	CDNA: FLJ20972 fis, clone ADSU01569.	ENST00000356216	Unknown	
BMI1	4.6	2.5	B lymphoma Mo-MLV insertion region (mouse) (BMI1), mRNA	NM_005180	Hs.496613	NM_005180
NOV	4.6	4.2	nephroblastoma overexpressed gene (NOV), mRNA	NM_002514	Hs.235935	NM_002514
CR601196	4.6	11.4	full-length cDNA clone CS0DB002YH03 of Neuroblastoma Cot 10-normalized of (human).	CR601196	Hs.592518	AK055418
TRMT5	4.6	1.5	TRM5 tRNA methyltransferase 5 homolog (S. cerevisiae) (TRMT5), mRNA	NM_020810	Hs.380159	NM_020810
AF087985	4.6	3.0	full length insert cDNA clone YW29F03.	AF087985	Hs.125008	BQ227432
PYGO1	4.6	2.9	pygopus homolog 1 (Drosophila) (PYGO1), mRNA	NM_015617	Hs.256587	AL049925
ADNP	4.5	1.4	activity-dependent neuroprotector (ADNP), transcript variant 1, mRNA	NM_015339	Hs.570355	BC075794
MYF6	4.5	3.6	myogenic factor 6 (herculin) (MYF6), mRNA	NM_002469	Hs.35937	NM_002469
THC2729730	4.5	7.5	Q91873_XENLA (Q91873) Xenopus laevis tandemly arranged embryonic U1 snRNA genes U1a/U1b, partial (13%)	THC2729730	Unknown	
HNRPUL2	4.5	1.5	PREDICTED: heterogeneous nuclear ribonucleoprotein U-like 2 (HNRPUL2), mRNA	XM_495877	Unknown	

FMN2	4.5	2.3	formin 2 (FMN2), mRNA	NM_020066	Hs.24889	NM_020066
LOC153914	4.5	2.4	cDNA FLJ13531 fis, clone PLACE1006288, highly similar to VOLTAGE-DEPENDENT ANION-SELECTIVE CHANNEL PROTEIN 1.	AK023593	Hs.7277	BX648803
SERPINA5	4.5	7.8	serpin peptidase inhibitor, clade A (alpha-1 antiproteinase, antitrypsin), member 5 (SERPINA5), mRNA	NM_000624	Hs.510334	NM_000624
SLC41A3	4.5	1.8	solute carrier family 41, member 3 (SLC41A3), transcript variant 2, mRNA	NM_017836	Hs.573007	AK128054
ZNF446	4.5	2.1	zinc finger protein 446 (ZNF446), mRNA	NM_017908	Hs.697107	AK055359
FBXL4	4.5	1.8	F-box and leucine-rich repeat protein 4 (FBXL4), mRNA	NM_012160	Hs.536850	BC032641
LOC441743	4.5	1.5	similar to C367G8.3 (novel protein similar to RPL23A (60S ribosomal protein L23A)) (LOC441743), mRNA	NM_001045548	Unknown	
DPY19L2	4.5	3.9	dpy-19-like 2 (C. elegans) (DPY19L2), mRNA	NM_173812	Hs.533644	NM_173812
BC035417	4.5	2.2	Homo sapiens, clone IMAGE:5167600, mRNA.	BC035417	Hs.178144	BM563086
ADD3	4.5	1.6	adducin 3 (gamma) (ADD3), transcript variant 1, mRNA	NM_016824	Hs.501012	NM_016824
HIST2H2BE	4.5	4.2	histone cluster 2, H2be (HIST2H2BE), mRNA	NM_003528	Hs.2178	BC069193
FBXO33	4.5	1.5	F-box protein 33 (FBXO33), mRNA	NM_203301	Hs.324342	BC053537
THC2716965	4.5	5.2	THC2716965	THC2716965	Unknown	
UBXD3	4.5	3.7	UBX domain containing 3 (UBXD3), mRNA	NM_152376	Hs.432503	BX648631
CX3CL1	4.5	2.9	chemokine (C-X3-C motif) ligand 1 (CX3CL1), mRNA	NM_002996	Hs.531668	AB209037
THC2669541	4.5	5.3	COAT_BPHK7 (P49861) Major capsid protein precursor (Gp5) (Head protein), partial (5%)	THC2669541	Unknown	
TGFB2	4.5	17.2	transforming growth factor, beta 2 (TGFB2), mRNA	NM_003238	Hs.133379	AB209842
FGF18	4.5	2.9	fibroblast growth factor 18 (FGF18), mRNA	NM_003862	Hs.87191	AF075292
LOC284542	4.5	4.3	mRNA for FLJ00388 protein.	AK090467	Hs.61504	AK090467
ISLR	4.5	2.6	immunoglobulin superfamily containing leucine-rich repeat (ISLR), transcript variant 1, mRNA	NM_005545	Hs.699822	AK074668
SLC2A2	4.5	13.0	solute carrier family 2 (facilitated glucose transporter), member 2 (SLC2A2), mRNA	NM_000340	Hs.167584	NM_000340
LOC652890	4.5	1.5	PREDICTED: similar to 60S ribosomal protein L7 (LOC652890), mRNA	XR_019544	Hs.648276	XR_019544
FLJ21062	4.5	1.7	hypothetical protein FLJ21062 (FLJ21062), mRNA	NM_001039706	Hs.657403	AL833446
BC030115	4.5	2.4	cDNA clone IMAGE:4801326.	BC030115	Hs.632864	CD245238
FLJ34515	4.5	12.5	cDNA FLJ34515 fis, clone HLUNG2006614.	AK091834	Hs.448825	XM_001131899
ORMDL1	4.5	1.7	ORM1-like 1 (S. cerevisiae) (ORMDL1), mRNA	NM_016467	Hs.700632	AK126336
ZNF694	4.5	1.7	zinc finger protein 694 (ZNF694), mRNA	NM_001012981	Hs.513451	BX648785
BM461836	4.5	2.1	AGENCOURT_6418378 NIH_MGC_71 cDNA clone IMAGE:5533889 5', mRNA sequence	BM461836	Hs.547454	BM461836
SLC35A1	4.5	1.8	solute carrier family 35 (CMP-sialic acid transporter), member A1 (SLC35A1), mRNA	NM_006416	Hs.423163	BC017807
SMYD3	4.5	2.9	SET and MYND domain containing 3 (SMYD3), mRNA	NM_022743	Hs.567571	AK023594
PKP2	4.5	2.6	plakophilin 2 (PKP2), transcript variant 2b, mRNA	NM_004572	Hs.164384	NM_004572
BZRAP1	4.5	1.8	benzodiazapine receptor (peripheral) associated protein 1 (BZRAP1), mRNA	NM_004758	Hs.112499	BC150279
CXCR4	4.5	2.6	chemokine (C-X-C motif) receptor 4 (CXCR4), transcript variant 1, mRNA	NM_001008540	Hs.593413	AF147204
POGZ	4.5	2.1	pogo transposable element with ZNF domain (POGZ), transcript variant 1, mRNA	NM_015100	Hs.591471	BX537838
EIF3S6	4.5	1.5	eukaryotic translation initiation factor 3, subunit 6 48kDa (EIF3S6), mRNA	NM_001568	Hs.405590	AK124178
VGLL4	4.5	2.3	vestigial like 4 (Drosophila) (VGLL4), mRNA	NM_014667	Hs.373959	AK126479
FLJ22671	4.5	4.7	hypothetical protein FLJ22671 (FLJ22671), mRNA	NM_024861	Hs.193745	NM_001085437
LOC728449	4.5	2.4	Annexin A8 (Annexin VIII) (Vascular anticoagulant-beta) (VAC-beta).	ENST00000335083	Unknown	
C22orf32	4.5	2.6	chromosome 22 open reading frame 32 (C22orf32), mRNA	NM_033318	Hs.306083	AK095636
DENND4A	4.5	2.3	DENN/MADD domain containing 4A (DENND4A), mRNA	NM_005848	Hs.654567	AL833317
TNNT2	4.5	14.0	troponin T {exons 14-15} .	S71126	Unknown	
ZZZ3	4.5	2.1	zinc finger, ZZ-type containing 3 (ZZZ3), mRNA	NM_015534	Hs.480506	AK074119
PCDHB10	4.5	2.6	protocadherin beta 10 (PCDHB10), mRNA	NM_018930	Hs.657953	AY358720
FLJ20054	4.5	4.3	hypothetical protein FLJ20054 (FLJ20054), mRNA	NM_019049	Hs.657779	BC022561
KIAA1841	4.5	2.0	KIAA1841, mRNA (cDNA clone MGC:39557 IMAGE:5314819), complete cds.	BC039298	Hs.468653	AL833595
BC020829	4.5	2.0	mRNA similar to hypothetical protein FLJ21394 (cDNA clone IMAGE:4770900).	BC020829	Unknown	
RGS5	4.5	10.1	regulator of G-protein signalling 5 (RGS5), mRNA	NM_003617	Hs.24950	NM_003617
SUSD1	4.5	2.5	sushi domain containing 1 (SUSD1), mRNA	NM_022486	Hs.494827	NM_022486
GTF2IRD2	4.5	2.5	transcription factor GTF2IRD2 isoform 2 (GTF2IRD2) mRNA, complete cds, alternatively spliced.	AY336979	Hs.647017	NM_173537
RUFY3	4.5	2.4	RUN and FYVE domain containing 3 (RUFY3), transcript variant 2, mRNA	NM_014961	Hs.7972	BC051716
LYRM5	4.5	2.1	LYR motif containing 5 (LYRM5), mRNA	NM_001001660	Hs.209151	CR616971

NMNAT1	4.5	2.8	nicotinamide nucleotide adenylyltransferase 1 (NMNAT1), mRNA	NM_022787	Hs.633762	NM_022787
SKAP2	4.5	3.4	src kinase associated phosphoprotein 2 (SKAP2), mRNA	NM_003930	Hs.200770	NM_003930
RABGAP1	4.5	2.0	RAB GTPase activating protein 1 (RABGAP1), mRNA	NM_012197	Hs.271341	BC054492
APC	4.5	1.8	adenomatosis polyposis coli (APC), mRNA	NM_000038	Hs.158932	NM_000038
PLEKHA4	4.5	1.9	pleckstrin homology domain containing, family A (phosphoinositide binding specific) member 4 (PLEKHA4), mRNA	NM_020904	Hs.9469	AB208908
EGLN1	4.5	5.7	Egl nine homolog 1 (EC 1.14.11.-) (Hypoxia-inducible factor prolyl hydroxylase 2) (HIF-prolyl hydroxylase 2) (HIF-PH2) (HPH-2) (Prolyl hydroxylase domain-containing protein 2) (PHD2) (SM-20).	ENST00000357180	Unknown	
PLCB1	4.5	2.1	phospholipase C, beta 1 (phosphoinositide-specific) (PLCB1), transcript variant 2, mRNA	NM_182734	Hs.431173	AB011153
TMEM29	4.5	1.6	transmembrane protein 29 (TMEM29), mRNA	NM_014138	Hs.653131	BX649013
RPL31	4.5	1.5	ribosomal protein L31 (RPL31), mRNA	NM_000993	Hs.469473	CR595074
ENST00000389905	4.5	2.7	CDNA FLJ38262 fis, clone FCBBF3001594, moderately similar to Chlamydomonas reinhardtii dhc1 1-alpha dynein heavy chain. (Fragment).	ENST00000389905	Unknown	
DB534761	4.5	2.7	DB534761 RIKEN full-length enriched human cDNA library, hippocampus cDNA clone H023029H14 3', mRNA sequence	DB534761	Hs.134215	DB534761
AV661884	4.5	5.3	AV661884 AV661884 GLC cDNA clone GLCGYF05 3', mRNA sequence	AV661884	Hs.547909	AV661884
A_24_P366457	4.5	1.7	A_24_P366457	A_24_P366457	Unknown	
RBMS3	4.5	6.2	RNA binding motif, single stranded interacting protein (RBMS3), transcript variant 1, mRNA	NM_001003793	Hs.696468	AL831860
MTR	4.5	2.6	5-methyltetrahydrofolate-homocysteine methyltransferase (MTR), mRNA	NM_000254	Hs.498187	U73338
ARFGAP3	4.5	1.8	ADP-ribosylation factor GTPase activating protein 3 (ARFGAP3), mRNA	NM_014570	Hs.162877	AK127525
RNF182	4.5	2.6	ring finger protein 182 (RNF182), mRNA	NM_152737	Hs.111164	BC030666
A_32_P218079	4.5	3.5	A_32_P218079	A_32_P218079	Unknown	
MEIS3P1	4.5	2.8	Meis1 homolog 3 (mouse) pseudogene 1 (MEIS3P1) on chromosome 17	NR_002211	Unknown	
LOC572558	4.5	2.5	chromosome 2 mRNA sequence.	AY343891	Hs.9015	CF127520
FOXD1	4.5	6.0	forkhead box D1 (FOXD1), mRNA	NM_004472	Hs.519385	NM_004472
AF052141	4.5	3.9	clone 24626 mRNA sequence.	AF052141	Hs.13438	AF052141
A_24_P264549	4.5	1.6	A_24_P264549	A_24_P264549	Unknown	
THC2670898	4.5	4.3	THC2670898	THC2670898	Unknown	
PLAC9	4.5	2.9	placenta-specific 9	ENST00000372263	Unknown	
DIP	4.5	1.8	death-inducing-protein (DIP), mRNA	NM_015124	Hs.475150	AB018310
TRIM63	4.5	30.9	tripartite motif-containing 63 (TRIM63), mRNA	NM_032588	Hs.279709	AF353673
GIT2	4.5	2.2	G protein-coupled receptor kinase interactor 2 (GIT2), transcript variant 1, mRNA	NM_057169	Hs.434996	NM_057169
ZNF658B	4.5	2.1	zinc finger protein 658B (ZNF658B), mRNA	NM_001032297	Hs.534812	NM_001032297
DNAH5	4.5	2.4	Ciliary dynein heavy chain 5 (Axonemal beta dynein heavy chain 5) (HL1).	ENST00000382416	Unknown	
BG547557	4.5	3.5	BG547557 602575410F1 NIH_MGC_77 cDNA clone IMAGE:4703546 5', mRNA sequence	BG547557	Hs.7155	NM_207315
AI207522	4.5	5.1	AI207522 HA2878 Human fetal liver cDNA library cDNA, mRNA sequence	AI207522	Hs.134859	BG401887
LOC391595	4.5	1.7	PREDICTED: similar to 60S ribosomal protein L32 (LOC391595), mRNA	XR_019529	Hs.647922	XR_019529
7-Sep	4.5	1.7	septin 7 (SEPT7), transcript variant 2, mRNA	NM_001011553	Hs.191346	AB209677
TAGAP	4.5	4.2	T-cell activation GTPase activating protein (TAGAP), transcript variant 2, mRNA	NM_054114	Hs.529984	NM_054114
F5	4.5	2.7	coagulation factor V (proaccelerin, labile factor) (F5), mRNA	NM_000130	Hs.30054	NM_000130
C1orf77	4.5	2.2	chromosome 1 open reading frame 77, mRNA (cDNA clone MGC:131924 IMAGE:4450075), complete cds.	BC108721	Hs.611057	AK095230
AKR1C1	4.5	2.8	aldo-keto reductase family 1, member C1 (dihydrodiol dehydrogenase 1; 20-alpha (3-alpha)-hydroxysteroid dehydrogenase) (AKR1C1), mRNA	NM_001353	Hs.460260	AK226067
PLVAP	4.5	3.5	plasmalemma vesicle associated protein (PLVAP), mRNA	NM_031310	Hs.107125	BC056414
LRRK2	4.5	6.4	leucine-rich repeat kinase 2 (LRRK2), mRNA	NM_198578	Hs.187636	NM_198578
A_24_P93452	4.5	2.1	A_24_P93452	A_24_P93452	Unknown	
C7	4.5	14.1	complement component 7 (C7), mRNA	NM_000587	Hs.78065	NM_000587
EXT1	4.5	3.0	Exostosin-1 (EC 2.4.1.224) (EC 2.4.1.225) (Glucuronosyl-N- acetylglucosaminyl-proteoglycan/N-acetylglucosaminyl-proteoglycan 4- alpha-N-acetylglucosaminyltransferase) (Putative tumor suppressor protein EXT1) (Multiple exostoses protein 1)....	ENST00000378204	Unknown	
CCDC111	4.5	2.4	coiled-coil domain containing 111 (CCDC111), mRNA	NM_152683	Hs.481307	BX647575
PHF14	4.5	2.5	PHD finger protein 14 (PHF14), transcript variant 2, mRNA	NM_014660	Hs.655688	NM_014660
A_24_P924240	4.5	3.4	A_24_P924240	A_24_P924240	Unknown	
ENST00000328043	4.5	2.1	LRP11 protein (Fragment).	ENST00000328043	Unknown	
COL1A1	4.5	2.3	H.sapiens mRNA for prepro-alpha1(I) collagen.	Z74615	Hs.172928	Z74615
ENST00000338358	4.5	2.5	Nuclear factor erythroid 2-related factor 2 (NF-E2-related factor 2) (NFE2-related factor 2) (Nuclear factor, erythroid derived 2, like 2) (HEBP1).	ENST00000338358	Unknown	

KIAA0467	4.5	2.5	KIAA0467 (KIAA0467), mRNA	NM_015284	Hs.696004	BC151232
THC2652700	4.5	2.4	THC2652700	THC2652700	Unknown	
MAGED2	4.5	2.2	melanoma antigen family D, 2 (MAGED2), transcript variant 3, mRNA	NM_201222	Hs.522665	AK092463
MGC16169	4.5	2.0	hypothetical protein MGC16169 (MGC16169), mRNA	NM_033115	Hs.292986	BX647851
KIAA0737	4.5	1.9	KIAA0737 (KIAA0737), mRNA	NM_014828	Hs.555910	NM_014828
PTPRM	4.5	1.9	protein tyrosine phosphatase, receptor type, M (PTPRM), mRNA	NM_002845	Hs.49774	BC051651
THC2650264	4.5	4.0	Q8CHC2_MOUSE (Q8CHC2) MKIAA0940 protein (Fragment), partial (57%)	THC2650264	Unknown	
NP1252191	4.5	2.0	GB|AACC02000041.1|EAL24419.1 similar to 60S ribosomal protein L32	NP1252191	Unknown	
ENST00000273844	4.5	1.6	PREDICTED: similar to ribosomal protein S15a (LOC391656), mRNA	ENST00000273844	Unknown	
C15orf21	4.5	1.5	chromosome 15 open reading frame 21 (C15orf21), transcript variant 1, mRNA	NM_001005266	Hs.574240	NM_001005266
IFT81	4.5	2.7	intraflagellar transport 81 homolog (Chlamydomonas) (IFT81), transcript variant 2, mRNA	NM_031473	Hs.528382	AF332010
BRP44	4.5	3.1	Brain protein 44.	ENST00000367846	Unknown	
AK094991	4.5	2.8	cDNA FLJ37672 fis, clone BRHIP2012059.	AK094991	Hs.633316	AK094991
FANCC	4.5	1.5	Fanconi anemia, complementation group C (FANCC), mRNA	NM_000136	Hs.494529	NM_000136
CCBE1	4.5	3.5	collagen and calcium binding EGF domains 1 (CCBE1), mRNA	NM_133459	Hs.34333	AB075863
FLJ37464	4.5	4.3	hypothetical protein FLJ37464 (FLJ37464), mRNA	NM_173815	Hs.346947	BC041823
THC2667995	4.5	3.0	THC2667995	THC2667995	Unknown	
CFI	4.5	122.8	complement factor I (CFI), mRNA	NM_000204	Hs.312485	AK122686
HAL	4.5	3.8	histidine ammonia-lyase (HAL), mRNA	NM_002108	Hs.190783	NM_002108
PBX1	4.5	2.8	Pre-B-cell leukemia transcription factor 1 (Homeobox protein PBX1) (Homeobox protein PRL).	ENST00000328681	Unknown	
C1orf124	4.5	1.8	chromosome 1 open reading frame 124 (C1orf124), transcript variant 2, mRNA	NM_001010984	Hs.554892	NM_001010984
DHRS9	4.5	6.0	dehydrogenase/reductase (SDR family) member 9 (DHRS9), transcript variant 1, mRNA	NM_005771	Hs.179608	NM_005771
HKDC1	4.5	3.1	hexokinase domain containing 1 (HKDC1), mRNA	NM_025130	Unknown	
AK1	4.5	2.1	adenylate kinase 1 (AK1), mRNA	NM_000476	Hs.175473	NM_000476
ZZZ3	4.5	2.1	zinc finger, ZZ-type containing 3 (ZZZ3), mRNA	NM_015534	Hs.480506	AK074119
RPL34	4.5	1.4	ribosomal protein L34 (RPL34), transcript variant 2, mRNA	NM_033625	Hs.438227	BG112770
TRIP4	4.5	1.7	thyroid hormone receptor interactor 4 (TRIP4), mRNA	NM_016213	Hs.500340	NM_016213
AF119895	4.5	6.2	PRO2743 mRNA, complete cds.	AF119895	Unknown	
CDKN1B	4.5	1.9	cyclin-dependent kinase inhibitor 1B (p27, Kip1) (CDKN1B), mRNA	NM_004064	Hs.238990	NM_004064
THC2725553	4.5	8.7	THC2725553	THC2725553	Unknown	
THC2685686	4.5	3.3	ALU7_HUMAN (P39194) Alu subfamily SQ sequence contamination warning entry, partial (11%)	THC2685686	Unknown	
HLCS	4.4	1.5	holocarboxylase synthetase (biotin-(proprionyl-Coenzyme A-carboxylase (ATP-hydrolysing)) ligase) (HLCS), mRNA	NM_000411	Hs.371350	D87328
NKX3-1	4.4	3.2	NK3 transcription factor related, locus 1 (Drosophila) (NKX3-1), mRNA	NM_006167	Hs.55999	NM_006167
NEXN	4.4	3.7	nexilin (F actin binding protein) (NEXN), mRNA	NM_144573	Hs.632387	NM_144573
AMN1	4.4	2.2	antagonist of mitotic exit network 1 homolog (S. cerevisiae) (AMN1), mRNA	NM_207337	Hs.591146	BC045831
ST3GAL6	4.4	2.8	ST3 beta-galactoside alpha-2,3-sialyltransferase 6 (ST3GAL6), mRNA	NM_006100	Hs.148716	CR749468
LOC253482	4.4	1.5	PREDICTED: similar to 40S ribosomal protein S6 (LOC253482), mRNA	XR_016415	Hs.664168	BI194585
CR595522	4.4	2.1	full-length cDNA clone CS0DC001YI12 of Neuroblastoma Cot 25-normalized of (human).	CR595522	Hs.637017	NM_005909
LOC440295	4.4	1.8	hypothetical protein LOC440295 (LOC440295), mRNA	NM_198181	Hs.534900	XM_001130543
THC2718728	4.4	5.1	THC2718728	THC2718728	Unknown	
TMEM132C	4.4	25.4	cDNA FLJ44761 fis, clone BRACE3031728.	ENST00000315208	Unknown	
THC2705799	4.4	2.1	ALU1_HUMAN (P39188) Alu subfamily J sequence contamination warning entry, partial (4%)	THC2705799	Unknown	
AK097724	4.4	2.0	cDNA FLJ40405 fis, clone TESTI2037382.	AK097724	Hs.631930	AK097724
HIST1H2AE	4.4	1.8	histone cluster 1, H2ae (HIST1H2AE), mRNA	NM_021052	Hs.121017	BE741093
RBM5	4.4	2.1	RNA binding motif protein 5 (RBM5), mRNA	NM_005778	Hs.439480	AB208813
LYST	4.4	21.9	lysosomal trafficking regulator (LYST), transcript variant 1, mRNA	NM_000081	Hs.532411	NM_000081
THC2651447	4.4	16.5	THC2651447	THC2651447	Unknown	
LEPROTL1	4.4	2.2	leptin receptor overlapping transcript-like 1 (LEPROTL1), mRNA	NM_015344	Hs.146585	AF063605
RAB11A	4.4	2.5	RAB11A, member RAS oncogene family (RAB11A), mRNA	NM_004663	Hs.321541	BC013348
KCTD16	4.4	3.4	potassium channel tetramerisation domain containing 16 (KCTD16), mRNA	NM_020768	Hs.693927	AB037738
APOH	4.4	34.0	apolipoprotein H (beta-2-glycoprotein I) (APOH), mRNA	NM_000042	Hs.445358	NM_000042
BAIAP2	4.4	2.1	BAI1-associated protein 2 (BAIAP2), transcript variant 2, mRNA	NM_017451	Hs.128316	AK127291

RPS3A	4.4	1.6	ribosomal protein S3A (RPS3A), mRNA	NM_001006	Hs.356572	BI087817
SCUBE3	4.4	2.0	signal peptide, CUB domain, EGF-like 3 (SCUBE3), mRNA	NM_152753	Hs.12923	CR936873
CXCR4	4.4	2.6	chemokine (C-X-C motif) receptor 4 (CXCR4), transcript variant 1, mRNA	NM_001008540	Hs.593413	AF147204
BRUNOL6	4.4	3.0	bruno-like 6, RNA binding protein (Drosophila) (BRUNOL6), mRNA	NM_052840	Hs.348342	AK131098
GOLGA8A	4.4	3.8	golgi autoantigen, golgin subfamily a, 8A (GOLGA8A), mRNA	NM_181077	Hs.182982	BX648160
THC2704994	4.4	4.1	THC2704994	THC2704994	Unknown	
CXCR4	4.4	2.6	chemokine (C-X-C motif) receptor 4 (CXCR4), transcript variant 1, mRNA	NM_001008540	Hs.593413	AF147204
AF086124	4.4	1.9	full length insert cDNA clone ZA79C08.	AF086124	Hs.178144	BM563086
MAPK8	4.4	1.8	Mitogen-activated protein kinase 8 (EC 2.7.11.24) (Stress-activated protein kinase JNK1) (c-Jun N-terminal kinase 1) (JNK-46).	ENST00000374189	Unknown	
WASF3	4.4	1.6	WAS protein family, member 3 (WASF3), mRNA	NM_006646	Hs.635221	NM_006646
ACTR10	4.4	2.4	actin-related protein 10 homolog (S. cerevisiae) (ACTR10), mRNA	NM_018477	Hs.509451	NM_018477
ZZZ3	4.4	2.2	zinc finger, ZZ-type containing 3 (ZZZ3), mRNA	NM_015534	Hs.480506	AK074119
FLJ34503	4.4	4.7	hypothetical protein FLJ34503 (FLJ34503), mRNA	NM_001039528	Unknown	
COL23A1	4.4	1.9	collagen, type XXIII, alpha 1 (COL23A1), mRNA	NM_173465	Hs.660026	NM_173465
CR590862	4.4	1.9	full-length cDNA clone CS0DI036YC04 of Placenta Cot 25-normalized of (human).	CR590862	Hs.259347	NM_173601
A_32_P5205	4.4	3.9	A_32_P5205	A_32_P5205	Unknown	
BC041459	4.4	9.0	Homo sapiens, clone IMAGE:5207242, mRNA.	BC041459	Hs.102408	BC041459
COL1A1	4.4	2.2	H.sapiens mRNA for prepro-alpha1(I) collagen.	Z74615	Hs.172928	Z74615
IER5L	4.4	3.4	immediate early response 5-like (IER5L), mRNA	NM_203434	Hs.529857	NM_203434
LOC340888	4.4	5.6	PREDICTED: similar to aldo-keto reductase family 1, member B10 (LOC340888), mRNA	XR_018726	Hs.646726	XR_018726
ANAPC13	4.4	2.0	anaphase promoting complex subunit 13 (ANAPC13), mRNA	NM_015391	Hs.106909	AK001285
DCTN3	4.4	2.0	dynactin 3 (p22) (DCTN3), transcript variant 1, mRNA	NM_007234	Hs.511768	BM920638
CNKSR3	4.4	2.1	CNKSR family member 3 (CNKSR3), mRNA	NM_173515	Hs.16064	AY328891
ZNF583	4.4	2.5	zinc finger protein 583 (ZNF583), mRNA	NM_152478	Hs.146854	AK055592
A_32_P23872	4.4	5.1	A_32_P23872	A_32_P23872	Unknown	
ADCYAP1	4.4	4.7	Pituitary adenylate cyclase-activating polypeptide precursor (PACAP) [Contains: PACAP-related peptide (PRP-48); Pituitary adenylate cyclase-activating polypeptide 27 (PACAP-27) (PACAP27); Pituitary adenylate cyclase-activating polypeptide 38...	ENST00000269200	Unknown	
ZNF181	4.4	1.9	zinc finger protein 181 (ZNF181), mRNA	NM_001029997	Hs.659191	BC043228
MGC4093	4.4	1.7	hypothetical protein MGC4093 (MGC4093), mRNA	NM_030578	Hs.567596	BQ900587
BC023330	4.4	2.4	Homo sapiens, clone IMAGE:4944844, mRNA.	BC023330	Hs.180408	NM_152707
NAP5	4.4	3.8	cDNA FLJ42668 fis, clone BRAMY2020270.	AK124659	Hs.537329	NM_207363
CYP4V2	4.4	4.5	cytochrome P450, family 4, subfamily V, polypeptide 2 (CYP4V2), mRNA	NM_207352	Hs.237642	BX648730
C1orf9	4.4	2.2	chromosome 1 open reading frame 9 (C1orf9), transcript variant 2, mRNA	NM_016227	Hs.204559	NM_016227
PCGF5	4.4	1.9	polycomb group ring finger 5 (PCGF5), mRNA	NM_032373	Hs.500512	NM_032373
MSRB2	4.4	1.9	methionine sulfoxide reductase B2 (MSRB2), mRNA	NM_012228	Hs.461420	NM_012228
LRRC23	4.4	2.6	leucine rich repeat containing 23 (LRRC23), transcript variant 2, mRNA	NM_006992	Hs.155586	BC029858
CB987747	4.4	5.8	AGENCOURT_13631433 NIH_MGC_184 cDNA clone IMAGE:30327753 5', mRNA sequence	CB987747	Hs.527515	CB987747
RFX4	4.4	5.8	regulatory factor X, 4 (influences HLA class II expression) (RFX4), transcript variant 3, mRNA	NM_213594	Hs.388827	AB095366
PREPL	4.4	2.0	prolyl endopeptidase-like (PREPL), transcript variant C, mRNA	NM_006036	Hs.444349	BC151236
ST3GAL6	4.4	2.5	ST3 beta-galactoside alpha-2,3-sialyltransferase 6 (ST3GAL6), mRNA	NM_006100	Hs.148716	CR749468
CXCR4	4.4	2.6	chemokine (C-X-C motif) receptor 4 (CXCR4), transcript variant 1, mRNA	NM_001008540	Hs.593413	AF147204
THC2766709	4.4	3.3	ALU1_HUMAN (P39188) Alu subfamily J sequence contamination warning entry, partial (13%)	THC2766709	Unknown	
NFIL3	4.4	2.0	nuclear factor, interleukin 3 regulated (NFIL3), mRNA	NM_005384	Hs.79334	NM_005384
ENST00000334994	4.4	3.1	CDNA FLJ41026 fis, clone BRAMY2004771, weakly similar to CHONDROADHERIN.	ENST00000334994	Unknown	
ING4	4.4	2.0	inhibitor of growth family, member 4 (ING4), mRNA	NM_016162	Hs.524210	NM_016162
GUCA2B	4.4	6.5	guanylate cyclase activator 2B (uroguanylin) (GUCA2B), mRNA	NM_007102	Hs.32966	NM_007102
RCSD1	4.4	13.2	RCSD domain containing 1 (RCSD1), mRNA	NM_052862	Hs.493867	NM_052862
SLC22A5	4.4	2.4	solute carrier family 22 (organic cation transporter), member 5 (SLC22A5), mRNA	NM_003060	Hs.696078	AB209484
SCN9A	4.4	2.3	sodium channel, voltage-gated, type IX, alpha (SCN9A), mRNA	NM_002977	Hs.699185	NM_002977
RP11-298P3.3	4.4	2.0	Human BRCA2 region, mRNA sequence CG016.	U50529	Hs.507680	AL049783
ESD	4.4	1.8	esterase D/formylglutathione hydrolase (ESD), mRNA	NM_001984	Hs.432491	AK021825

RGPD1	4.4	2.0	RANBP2-like and GRIP domain containing 1 (RGPD1), mRNA	NM_001024457	Hs.656849	CR749330
LOC145853	4.4	1.8	AGENCOURT_10399027 NIH_MGC_82 cDNA clone IMAGE:6614823 5', mRNA sequence	BU568037	Hs.662358	BU568037
RPS15A	4.4	1.6	ribosomal protein S15a, mRNA (cDNA clone MGC:2466 IMAGE:2967511), complete cds.	BC001697	Hs.370504	BM907705
CORIN	4.4	6.9	corin, serine peptidase (CORIN), mRNA	NM_006587	Hs.518618	BX648285
WNT4	4.4	9.1	wingless-type MMTV integration site family, member 4 (WNT4), mRNA	NM_030761	Hs.591521	AY358947
COL1A1	4.4	2.3	H.sapiens mRNA for prepro-alpha1(I) collagen.	Z74615	Hs.172928	Z74615
ZBTB46	4.4	2.2	cDNA FLJ44766 fis, clone BRACE3032537.	AK126720	Hs.585028	AK131482
BC042172	4.4	3.8	zinc finger, MYM-type 6, mRNA (cDNA clone MGC:52473 IMAGE:5455214), complete cds.	BC042172	Unknown	
C17orf25	4.4	1.4	chromosome 17 open reading frame 25 (C17orf25), mRNA	NM_016080	Hs.279061	AK001488
THC2533996	4.4	1.7	HSU09954 ribosomal protein L9 {Homo sapiens} (exp=-1; wgp=0; cg=0), partial (42%)	THC2533996	Unknown	
MYT1	4.4	2.2	myelin transcription factor 1 (MYT1), mRNA	NM_004535	Hs.279562	AB028973
ZZZ3	4.4	2.3	zinc finger, ZZ-type containing 3 (ZZZ3), mRNA	NM_015534	Hs.480506	AK074119
C9orf123	4.4	3.9	chromosome 9 open reading frame 123 (C9orf123), mRNA	NM_033428	Hs.7517	NM_033428
FOXJ2	4.4	1.7	forkhead box J2 (FOXJ2), mRNA	NM_018416	Hs.120844	NM_018416
DYNLT1	4.4	1.6	dynein, light chain, Tctex-type 1 (DYNLT1), mRNA	NM_006519	Hs.445999	AK026669
CREBBP	4.4	1.5	CREB binding protein (Rubinstein-Taybi syndrome) (CREBBP), transcript variant 1, mRNA	NM_004380	Hs.459759	NM_004380
PTHR1	4.4	2.5	parathyroid hormone receptor 1 (PTHR1), mRNA	NM_000316	Hs.1019	AF495723
NEK7	4.4	5.5	Serine/threonine-protein kinase Nek7 (EC 2.7.11.1) (NimA-related protein kinase 7).	ENST00000367385	Unknown	
AK023526	4.4	2.3	cDNA FLJ13464 fis, clone PLACE1003478.	AK023526	Hs.670477	AK023526
VPS54	4.4	1.6	vacuolar protein sorting 54 homolog (S. cerevisiae) (VPS54), transcript variant 1, mRNA	NM_016516	Hs.48499	AK124436
CIR	4.4	1.9	CBF1 interacting corepressor (CIR), mRNA	NM_004882	Hs.632531	CR611416
FLJ20433	4.4	2.9	hypothetical protein FLJ20433 (FLJ20433), mRNA	NM_017820	Hs.495553	AK091390
ALK	4.4	5.4	anaplastic lymphoma kinase (Ki-1) (ALK), mRNA	NM_004304	Hs.654469	U62540
SLIT1	4.4	10.1	Slit homolog 1 protein precursor (Slit-1) (Multiple epidermal growth factor-like domains 4).	ENST00000266058	Unknown	
COL1A1	4.4	2.2	H.sapiens mRNA for prepro-alpha1(I) collagen.	Z74615	Hs.172928	Z74615
THRAP2	4.4	1.8	thyroid hormone receptor associated protein 2 (THRAP2), mRNA	NM_015335	Hs.654691	NM_015335
ODC1	4.4	2.0	ornithine decarboxylase 1 (ODC1), mRNA	NM_002539	Hs.467701	CR614398
SERPINA11	4.4	4.4	human full-length cDNA clone CS0DM004YD06 of Fetal liver of (human).	BX248259	Hs.145890	NM_001080451
SETD7	4.4	3.3	SET domain containing (lysine methyltransferase) 7 (SETD7), mRNA	NM_030648	Hs.480792	NM_030648
AFF2	4.4	5.8	AF4/FMR2 family, member 2 (AFF2), mRNA	NM_002025	Hs.496911	NM_002025
ZNF221	4.4	2.7	zinc finger protein 221 (ZNF221), mRNA	NM_013359	Hs.631598	NM_013359
PHYHIPL	4.4	6.0	phytanoyl-CoA 2-hydroxylase interacting protein-like (PHYHIPL), mRNA	NM_032439	Hs.499704	AB058699
KAZALD1	4.4	2.9	Kazal-type serine peptidase inhibitor domain 1 (KAZALD1), mRNA	NM_030929	Hs.632079	AK172864
AK000901	4.4	1.7	cDNA FLJ10039 fis, clone HEMBA1000975.	AK000901	Hs.697792	XM_933842
FBXL2	4.4	2.2	F-box and leucine-rich repeat protein 2 (FBXL2), mRNA	NM_012157	Hs.475872	AK096492
RHOBTB2	4.4	2.0	Rho-related BTB domain containing 2 (RHOBTB2), mRNA	NM_015178	Hs.372688	AB018260
THC2647388	4.4	2.2	Q4TD86_TETNG (Q4TD86) Chromosome undetermined SCAF6431, whole genome shotgun sequence. (Fragment), partial (9%)	THC2647388	Unknown	
BM468849	4.4	1.7	AGENCOURT_6447766 NIH_MGC_92 cDNA clone IMAGE:5587170 5', mRNA sequence	BM468849	Hs.270571	BM468849
CA314185	4.4	2.2	UI-CF-FN0-aez-k-14-0-UI.s1 UI-CF-FN0 cDNA clone UI-CF-FN0-aez-k-14-0-UI 3', mRNA sequence	CA314185	Hs.623068	CA314185
TTC30A	4.4	2.5	tetratricopeptide repeat domain 30A (TTC30A), mRNA	NM_152275	Hs.128384	NM_152275
AI620901	4.4	11.2	AI620901 tu05d02.x1 NCI_CGAP_Pr28 cDNA clone IMAGE:2250147 3', mRNA sequence	AI620901	Hs.696364	NM_003392
HGF	4.4	24.4	Hepatocyte growth factor precursor (Scatter factor) (SF) (Hepatopoeitin A) .	ENST00000222390	Unknown	
C6orf48	4.4	1.7	chromosome 6 open reading frame 48 (C6orf48), transcript variant 1, mRNA	NM_001040437	Hs.640836	BQ057480
DHDH	4.4	2.1	dihydrodiol dehydrogenase (dimeric) (DHDH), mRNA	NM_014475	Hs.631555	BM454993
CASP2	4.4	1.6	caspase 2, apoptosis-related cysteine peptidase (neural precursor cell expressed, developmentally down-regulated 2) (CASP2), transcript variant 1, mRNA	NM_032982	Hs.368982	AB209640
GUCY1A2	4.4	5.7	guanylate cyclase 1, soluble, alpha 2 (GUCY1A2), mRNA	NM_000855	Hs.654555	Z50053
BC037740	4.4	1.5	cDNA clone IMAGE:5263531.	BC037740	Hs.597434	BC037740
AK095904	4.4	2.7	cDNA FLJ38585 fis, clone HCHON2009191.	AK095904	Hs.660473	AK094157
KCNE2	4.4	3.1	potassium voltage-gated channel, Isk-related family, member 2 (KCNE2), mRNA	NM_172201	Hs.551521	AF302095
PDE4DIP	4.4	3.1	phosphodiesterase 4D interacting protein (myomegalin) (PDE4DIP), transcript variant 1, mRNA	NM_014644	Hs.654651	NM_014644

USP40	4.4	2.5	ubiquitin specific peptidase 40 (USP40), mRNA	NM_018218	Hs.96513	NM_018218
LYPD1	4.4	3.1	LY6/PLAUR domain containing 1 (LYPD1), transcript variant 1, mRNA	NM_144586	Hs.694844	NM_144586
ENTPD5	4.4	2.3	ectonucleoside triphosphate diphosphohydrolase 5, mRNA (cDNA clone IMAGE:3847878), with apparent retained intron.	BC020966	Unknown	
CXCR4	4.4	2.6	chemokine (C-X-C motif) receptor 4 (CXCR4), transcript variant 1, mRNA	NM_001008540	Hs.593413	AF147204
LOC728555	4.4	1.8	cDNA FLJ40901 fis, clone UTERU2003704.	AK098220	Hs.664334	AK098220
FILIP1	4.4	3.3	filamin A interacting protein 1 (FILIP1), mRNA	NM_015687	Hs.696158	AL832009
AK026459	4.4	3.8	cDNA: FLJ22806 fis, clone KAIA2845.	AK026459	Hs.605145	AK026459
DYRK3	4.4	2.5	dual-specificity tyrosine-(Y)-phosphorylation regulated kinase 3 (DYRK3), transcript variant 2, mRNA	NM_001004023	Hs.164267	AF186774
ZZZ3	4.4	2.3	zinc finger, ZZ-type containing 3 (ZZZ3), mRNA	NM_015534	Hs.480506	AK074119
ZZZ3	4.4	2.2	zinc finger, ZZ-type containing 3 (ZZZ3), mRNA	NM_015534	Hs.480506	AK074119
RARA	4.4	2.6	retinoic acid receptor, alpha (RARA), mRNA	NM_001033603	Unknown	
THC2731042	4.4	3.6	Q4SSA7_TETNG (Q4SSA7) Chromosome 11 SCAF14479, whole genome shotgun sequence. (Fragment), partial (5%)	THC2731042	Unknown	
BQ184357	4.4	2.8	UI-E-EJ1-ajs-n-23-0-UI.s1 UI-E-EJ1 cDNA clone UI-E-EJ1-ajs-n-23-0-UI 3', mRNA sequence	BQ184357	Hs.619929	BQ184357
USP40	4.4	2.5	ubiquitin specific peptidase 40 (USP40), mRNA	NM_018218	Hs.96513	NM_018218
TTC13	4.4	2.9	tetratricopeptide repeat domain 13 (TTC13), mRNA	NM_024525	Hs.424788	NM_024525
PURG	4.4	3.0	purine-rich element binding protein G (PURG), transcript variant B, mRNA	NM_001015508	Hs.373778	AF195513
WHSC1	4.4	1.9	Wolf-Hirschhorn syndrome candidate 1 (WHSC1), transcript variant 7, mRNA	NM_133334	Hs.113876	NM_133334
MYL5	4.3	2.3	myosin, light chain 5, regulatory (MYL5), mRNA	NM_002477	Hs.410970	AK123974
ZFAND2B	4.3	2.1	zinc finger, AN1-type domain 2B (ZFAND2B), mRNA	NM_138802	Hs.534540	AK091345
LOC730452	4.3	1.4	PREDICTED: similar to 60S ribosomal protein L7 (LOC730452), mRNA	XM_001125895	Unknown	
ATRN	4.3	1.7	attractin (ATRN), transcript variant 1, mRNA	NM_139321	Hs.276252	NM_139321
CDKN1B	4.3	1.9	cyclin-dependent kinase inhibitor 1B (p27, Kip1) (CDKN1B), mRNA	NM_004064	Hs.238990	NM_004064
LOC388965	4.3	1.7	similar to hepatitis C virus core-binding protein 6; cervical cancer oncogene 3 (LOC388965), mRNA	NM_001013648	Unknown	
ATP5E	4.3	2.3	ATP synthase, H+ transporting, mitochondrial F1 complex, epsilon subunit (ATP5E), nuclear gene encoding mitochondrial protein, mRNA	NM_006886	Hs.177530	CR595852
COPB1	4.3	1.6	coatomer protein complex, subunit beta 1 (COPB1), mRNA	NM_016451	Hs.339278	NM_016451
BMPR2	4.3	1.8	bone morphogenetic protein receptor, type II (serine/threonine kinase) (BMPR2), mRNA	NM_001204	Hs.471119	NM_001204
AK124173	4.3	5.6	cDNA FLJ42179 fis, clone THYMU2030796.	AK124173	Hs.598050	AK124173
CXCR4	4.3	2.6	chemokine (C-X-C motif) receptor 4 (CXCR4), transcript variant 1, mRNA	NM_001008540	Hs.593413	AF147204
HSPB3	4.3	66.2	heat shock 27kDa protein 3 (HSPB3), mRNA	NM_006308	Hs.41707	NM_006308
C11orf41	4.3	2.1	Human G2 protein mRNA, partial cds.	U10991	Hs.502266	NM_012194
LOC283859	4.3	5.8	cDNA FLJ20269 fis, clone HEP01293.	AK000276	Hs.592059	BC051288
NACAL	4.3	1.3	nascent-polypeptide-associated complex alpha polypeptide-like (NACAL), mRNA	NM_199290	Hs.591178	BU567791
LYRM1	4.3	2.2	LYR motif containing 1 (LYRM1), mRNA	NM_020424	Hs.185489	NM_020424
BC041926	4.3	2.2	cDNA clone IMAGE:5300349.	BC041926	Hs.397465	BX538009
ENST00000334827	4.3	2.6	Leucine-rich repeats and calponin homology domain-containing protein 3 precursor.	ENST00000334827	Unknown	
ST3GAL6	4.3	2.6	ST3 beta-galactoside alpha-2,3-sialyltransferase 6 (ST3GAL6), mRNA	NM_006100	Hs.148716	CR749468
CDK4	4.3	1.5	cyclin-dependent kinase 4 (CDK4), mRNA	NM_000075	Hs.95577	BM467999
RPS3A	4.3	1.5	ribosomal protein S3A (RPS3A), mRNA	NM_001006	Hs.356572	BI087817
ENST00000346061	4.3	1.7	Putative FRG1-like protein C20orf80.	ENST00000346061	Unknown	
SPHK1	4.3	3.2	sphingosine kinase 1 (SPHK1), transcript variant 1, mRNA	NM_021972	Hs.68061	AK095578
ACAD9	4.3	1.4	acyl-Coenzyme A dehydrogenase family, member 9 (ACAD9), mRNA	NM_014049	Hs.567482	AB209427
APC	4.3	2.1	adenomatosis polyposis coli (APC), mRNA	NM_000038	Hs.158932	NM_000038
CXCR4	4.3	2.6	chemokine (C-X-C motif) receptor 4 (CXCR4), transcript variant 1, mRNA	NM_001008540	Hs.593413	AF147204
LOC645954	4.3	3.0	PREDICTED: similar to supervillin isoform 2 (LOC645954), mRNA	XR_017086	Hs.408581	XR_017086
THC2481891	4.3	7.4	ALU7_HUMAN (P39194) Alu subfamily SQ sequence contamination warning entry, partial (12%)	THC2481891	Unknown	
THC2646590	4.3	2.4	Q2Y6J7_NITMU (Q2Y6J7) Binding-protein-dependent transport systems inner membrane component, partial (6%)	THC2646590	Unknown	
THC2662740	4.3	3.3	ALU1_HUMAN (P39188) Alu subfamily J sequence contamination warning entry, partial (6%)	THC2662740	Unknown	
A_24_P384411	4.3	1.5	A_24_P384411	A_24_P384411	Unknown	
AGPAT4	4.3	2.1	1-acylglycerol-3-phosphate O-acyltransferase 4 (lysophosphatidic acid acyltransferase, delta) (AGPAT4), mRNA	NM_020133	Hs.353175	NM_020133
SLC25A37	4.3	1.7	FP15737 mRNA, complete cds.	AF495725	Hs.596025	AF495725
MXRA8	4.3	3.9	matrix-remodelling associated 8 (MXRA8), mRNA	NM_032348	Hs.558570	AK095966

CNOT6	4.3	2.0	CCR4-NOT transcription complex, subunit 6 (CNOT6), mRNA	NM_015455	Hs.654984	AB033020
TPT1	4.3	2.5	tumor protein, translationally-controlled 1 (TPT1), mRNA	NM_003295	Hs.374596	BG033621
CD47	4.3	3.0	CD47 molecule (CD47), transcript variant 2, mRNA	NM_198793	Hs.446414	NM_001777
AK123083	4.3	1.9	cDNA FLJ41088 fis, clone ASTRO2002459.	AK123083	Hs.594085	AK123083
PTP4A1	4.3	1.8	protein tyrosine phosphatase type IVA, member 1 (PTP4A1), mRNA	NM_003463	Hs.227777	NM_003463
ENST00000333010	4.3	5.4	Jak and microtubule interacting protein 2	ENST00000333010	Unknown	
LOC643201	4.3	1.5	cDNA clone IMAGE:5268504.	BC052945	Hs.390285	BC052945
MBL2	4.3	20.8	mannose-binding lectin (protein C) 2, soluble (opsonic defect) (MBL2), mRNA	NM_000242	Hs.499674	NM_000242
ACP1	4.3	1.6	acid phosphatase 1, soluble (ACP1), transcript variant 3, mRNA	NM_004300	Hs.558296	NM_004300
PKP2	4.3	2.1	H.sapiens mRNA for plakophilin 2a and b.	X97675	Hs.164384	NM_004572
DCTN6	4.3	1.8	dynactin 6 (DCTN6), mRNA	NM_006571	Hs.158427	NM_006571
F5	4.3	2.9	coagulation factor V (proaccelerin, labile factor) (F5), mRNA	NM_000130	Hs.30054	NM_000130
MRPL53	4.3	1.6	mitochondrial ribosomal protein L53 (MRPL53), nuclear gene encoding mitochondrial protein, mRNA	NM_053050	Hs.534527	BM467462
ST3GAL6	4.3	2.6	ST3 beta-galactoside alpha-2,3-sialyltransferase 6 (ST3GAL6), mRNA	NM_006100	Hs.148716	CR749468
FLJ33790	4.3	2.1	hypothetical protein FLJ33790 (FLJ33790), mRNA	NM_001039548	Hs.292451	AL050370
KIAA1414	4.3	1.8	KIAA1414 protein (KIAA1414), mRNA	NM_019024	Hs.591564	NM_019024
RNF152	4.3	2.7	RING finger protein 152.	ENST00000312828	Unknown	
WDR26	4.3	2.7	WD repeat domain 26 (WDR26), mRNA	NM_025160	Hs.497873	AY221751
MBNL2	4.3	7.3	muscleblind-like 2 (Drosophila) (MBNL2), transcript variant 1, mRNA	NM_144778	Hs.657347	NM_144778
C5orf3	4.3	1.9	chromosome 5 open reading frame 3 (C5orf3), mRNA	NM_018691	Hs.166551	CR749447
ENST00000326678	4.3	3.5	hypothetical protein LOC643837, mRNA (cDNA clone IMAGE:4431274), partial cds.	ENST00000326678	Unknown	
SLFNL1	4.3	4.0	schlafen-like 1 (SLFNL1), mRNA	NM_144990	Hs.194609	BC050353
THBS3	4.3	1.8	thrombospondin 3 (THBS3), mRNA	NM_007112	Hs.169875	NM_007112
CDC20B	4.3	2.9	cell division cycle 20 homolog B (S. cerevisiae) (CDC20B), mRNA	NM_152623	Hs.669184	AK095246
ATP2B4	4.3	5.8	ATPase, Ca++ transporting, plasma membrane 4 (ATP2B4), transcript variant 1, mRNA	NM_001001396	Hs.343522	BX537444
ELAVL4	4.3	11.4	ELAV (embryonic lethal, abnormal vision, Drosophila)-like 4 (Hu antigen D), mRNA (cDNA clone MGC:33705 IMAGE:5286347), complete cds.	BC036071	Hs.213050	BC036071
LBR	4.3	1.8	lamin B receptor (LBR), transcript variant 1, mRNA	NM_002296	Hs.435166	NM_002296
PRKRA	4.3	1.5	protein kinase, interferon-inducible double stranded RNA dependent activator (PRKRA), mRNA	NM_003690	Hs.632540	AK092918
GNE	4.3	1.7	glucosamine (UDP-N-acetyl)-2-epimerase/N-acetylmannosamine kinase (GNE), mRNA	NM_005476	Hs.5920	NM_005476
DKFZp667M2411	4.3	1.5	mRNA; cDNA DKFZp667M2411 (from clone DKFZp667M2411).	AL713754	Hs.568209	AK124759
SGIP1	4.3	3.3	SH3-domain GRB2-like (endophilin) interacting protein 1 (SGIP1), mRNA	NM_032291	Hs.132121	AB210039
BX099457	4.3	7.9	BX099457 Soares placenta Nb2HP cDNA clone IMAGp998L09189, mRNA sequence	BX099457	Hs.268689	BX099457
A_32_P25809	4.3	2.2	A_32_P25809	A_32_P25809	Unknown	
ZNF425	4.3	1.8	zinc finger protein 425 (ZNF425), mRNA	NM_001001661	Hs.31743	AY621067
PCDH7	4.3	2.0	BH-protocadherin (brain-heart) (PCDH7), transcript variant c, mRNA	NM_032457	Hs.570785	NM_032457
TCEA3	4.3	14.9	transcription elongation factor A (SII), 3 (TCEA3), mRNA	NM_003196	Hs.446354	NM_003196
ZZZ3	4.3	2.3	zinc finger, ZZ-type containing 3 (ZZZ3), mRNA	NM_015534	Hs.480506	AK074119
FAM8A1	4.3	3.1	family with sequence similarity 8, member A1 (FAM8A1), mRNA	NM_016255	Hs.95260	NM_016255
KIAA0562	4.3	2.5	glycine-, glutamate-, thienylcyclohexylpiperidine-binding protein	ENST00000263739	Unknown	
SLC31A2	4.3	2.1	solute carrier family 31 (copper transporters), member 2 (SLC31A2), mRNA	NM_001860	Hs.24030	AK131071
COL1A1	4.3	2.2	H.sapiens mRNA for prepro-alpha1(I) collagen.	Z74615	Hs.172928	Z74615
CDKN1B	4.3	1.9	cyclin-dependent kinase inhibitor 1B (p27, Kip1) (CDKN1B), mRNA	NM_004064	Hs.238990	NM_004064
P2RY14	4.3	5.2	purinergic receptor P2Y, G-protein coupled, 14 (P2RY14), mRNA	NM_014879	Hs.2465	NM_001081455
RPL23AP13	4.3	2.2	ribosomal protein L23a pseudogene 13 (RPL23AP13) on chromosome 2	NR_002229	Unknown	
A_24_P367199	4.3	1.5	A_24_P367199	A_24_P367199	Unknown	
OIT3	4.3	8.1	oncoprotein induced transcript 3 (OIT3), mRNA	NM_152635	Hs.8366	AK096435
ODF2	4.3	4.6	outer dense fiber of sperm tails 2 (ODF2), transcript variant 1, mRNA	NM_002540	Hs.129055	NM_002540
MDK	4.3	2.6	midkine (neurite growth-promoting factor 2) (MDK), transcript variant 1, mRNA	NM_001012334	Hs.82045	CR590366
COPS4	4.3	1.6	COP9 constitutive photomorphogenic homolog subunit 4 (Arabidopsis) (COPS4), mRNA	NM_016129	Hs.190384	AK094238
TMEM59	4.3	2.3	transmembrane protein 59 (TMEM59), mRNA	NM_004872	Hs.523262	AK074636
EPHA7	4.3	2.8	EPH receptor A7 (EPHA7), mRNA	NM_004440	Hs.73962	NM_004440
C1orf21	4.3	2.6	chromosome 1 open reading frame 21 (C1orf21), mRNA	NM_030806	Hs.497159	NM_030806

COL1A1	4.3	2.2	H.sapiens mRNA for prepro-alpha1(I) collagen.	Z74615	Hs.172928	Z74615
FGD3	4.3	3.3	FYVE, RhoGEF and PH domain containing 3 (FGD3), mRNA	NM_033086	Hs.411081	NM_001083536
ITIH3	4.3	4.8	inter-alpha (globulin) inhibitor H3 (ITIH3), mRNA	NM_002217	Hs.76716	NM_002217
TXK	4.3	2.0	TXK tyrosine kinase (TXK), mRNA	NM_003328	Hs.479669	NM_003328
KCNH2	4.3	4.2	potassium voltage-gated channel, subfamily H (eag-related), member 2 (KCNH2), transcript variant 1, mRNA	NM_000238	Hs.647099	DQ525913
ROPN1B	4.3	8.1	ropporin, rhophilin associated protein 1B (ROPN1B), mRNA	NM_001012337	Hs.663128	BM552738
RPL34	4.3	1.6	ribosomal protein L34 (RPL34), transcript variant 2, mRNA	NM_033625	Hs.438227	BG112770
A_24_P144383	4.3	1.7	A_24_P144383	A_24_P144383	Unknown	
COL1A1	4.3	2.3	H.sapiens mRNA for prepro-alpha1(I) collagen.	Z74615	Hs.172928	Z74615
AK056744	4.3	1.9	cDNA FLJ32182 fis, clone PLACE6001823.	AK056744	Hs.511668	AJ608771
CYP3A5	4.3	3.6	cytochrome P450, family 3, subfamily A, polypeptide 5 (CYP3A5), mRNA	NM_000777	Hs.695915	BX537676
AK027294	4.3	11.3	cDNA FLJ14388 fis, clone HEMBA1002716.	AK027294	Hs.593316	AK027294
THC2764893	4.3	4.5	THC2764893	THC2764893	Unknown	
CD93	4.3	29.1	CD93 molecule (CD93), mRNA	NM_012072	Hs.97199	NM_012072
NEBL	4.3	4.5	nebulette (NEBL), transcript variant 1, mRNA	NM_006393	Hs.5025	Y16241
RNF141	4.3	1.7	ring finger protein 141 (RNF141), mRNA	NM_016422	Hs.44685	NM_016422
RFXANK	4.3	1.5	regulatory factor X-associated ankyrin-containing protein (RFXANK), transcript variant 1, mRNA	NM_003721	Hs.296776	BM455790
SIGLEC11	4.3	3.5	sialic acid binding Ig-like lectin 11 (SIGLEC11), mRNA	NM_052884	Hs.661852	AF337818
LOC283143	4.3	2.5	cDNA FLJ33283 fis, clone ASTRO2009177.	AK090602	Hs.130499	AK090602
BC019907	4.3	1.9	cDNA clone IMAGE:4914394, partial cds.	BC019907	Hs.638683	BC019907
TAF7	4.3	1.5	TAF7 RNA polymerase II, TATA box binding protein (TBP)-associated factor, 55kDa (TAF7), mRNA	NM_005642	Hs.438838	NM_005642
A_24_P49860	4.3	2.4	A_24_P49860	A_24_P49860	Unknown	
NTNG1	4.3	4.6	netrin G1 (NTNG1), mRNA	NM_014917	Hs.657434	AB023193
MTHFR	4.3	2.5	5,10-methylenetetrahydrofolate reductase (NADPH) (MTHFR), mRNA	NM_005957	Hs.214142	NM_005957
C21orf58	4.3	1.9	C21orf58 form B (C21orf58) mRNA, complete cds, alternatively spliced.	AY039244	Hs.236572	NM_058180
ZZZ3	4.3	2.3	zinc finger, ZZ-type containing 3 (ZZZ3), mRNA	NM_015534	Hs.480506	AK074119
ZNF781	4.3	4.7	zinc finger protein 781 (ZNF781), mRNA	NM_152605	Hs.631565	NM_152605
SGCD	4.3	2.6	sarcoglycan, delta (35kDa dystrophin-associated glycoprotein) (SGCD), transcript variant 1, mRNA	NM_000337	Hs.591727	NM_000337
AK091528	4.3	1.9	cDNA FLJ34209 fis, clone FCBBF3020599.	AK091528	Hs.592819	AK091528
CCDC57	4.3	1.7	coiled-coil domain containing 57 (CCDC57), mRNA	NM_198082	Hs.631724	AK074059
A_24_P298835	4.3	1.7	A_24_P298835	A_24_P298835	Unknown	
TCF21	4.3	8.4	transcription factor 21 (TCF21), transcript variant 2, mRNA	NM_003206	Hs.78061	NM_198392
GPA33	4.3	4.7	glycoprotein A33 (transmembrane) (GPA33), mRNA	NM_005814	Hs.651244	NM_005814
THC2657736	4.3	13.6	THC2657736	THC2657736	Unknown	
MTERFD3	4.3	2.6	MTERF domain containing 3 (MTERFD3), transcript variant 1, mRNA	NM_001033050	Hs.5009	BC050361
C4orf32	4.3	3.8	chromosome 4 open reading frame 32 (C4orf32), mRNA	NM_152400	Hs.23439	BC041964
BM983766	4.3	2.5	BM983766 UI-CF-DU1-aay-k-01-0-UI.s1 UI-CF-DU1 cDNA clone UI-CF-DU1-aay-k-01-0-UI 3', mRNA sequence	BM983766	Hs.694335	BX418370
RPL6	4.3	1.7	ribosomal protein L6 (RPL6), transcript variant 1, mRNA	NM_001024662	Hs.546283	BQ055135
AK096154	4.3	2.3	cDNA FLJ38835 fis, clone MESAN2002424.	AK096154	Hs.594968	AK096154
DKFZP686A01247	4.3	2.4	hypothetical protein (DKFZP686A01247), mRNA	NM_014988	Hs.335163	CR749205
PRDM2	4.3	2.3	PR domain containing 2, with ZNF domain (PRDM2), transcript variant 1, mRNA	NM_012231	Hs.371823	NM_012231
A_24_P418712	4.3	1.4	A_24_P418712	A_24_P418712	Unknown	
SYNJ1	4.3	2.7	synaptojanin 1 (SYNJ1), transcript variant 2, mRNA	NM_203446	Hs.473632	NM_203446
PRL	4.3	22.6	prolactin (PRL), mRNA	NM_000948	Hs.1905	CD512992
AK092378	4.3	2.7	cDNA FLJ35059 fis, clone OCBBF2018827.	AK092378	Hs.592572	BC035116
RNF6	4.3	1.4	ring finger protein (C3H2C3 type) 6 (RNF6), transcript variant 1, mRNA	NM_005977	Hs.136885	NM_005977
SHC2	4.3	2.5	SHC-transforming protein 2 (SH2 domain protein C2) (Src homology 2 domain-containing-transforming protein C2) (Protein Sck).	ENST00000264554	Unknown	
FLJ20323	4.3	2.0	cDNA FLJ39510 fis, clone PROST2018487.	AK096829	Unknown	
VAMP4	4.3	2.7	Vesicle-associated membrane protein 4 (VAMP-4).	ENST00000367740	Unknown	
USP47	4.3	3.3	ubiquitin specific peptidase 47 (USP47), mRNA	NM_017944	Hs.577256	NM_017944
THC2708710	4.3	10.1	THC2708710	THC2708710	Unknown	
GTF2B	4.3	2.9	general transcription factor IIB (GTF2B), mRNA	NM_001514	Hs.481852	BC021000
RPL21	4.3	1.8	ribosomal protein L21 (RPL21), mRNA	NM_000982	Hs.381123	CR602527

THC2659848	4.3	2.1	Q8TIK0_METAC (Q8TIK0) Predicted protein, partial (13%)	THC2659848	Unknown	
CRYGS	4.3	2.2	crystallin, gamma S (CRYGS), mRNA	NM_017541	Hs.376209	BX648612
SHE	4.2	8.6	Src homology 2 domain containing E (SHE), mRNA	NM_001010846	Hs.591481	CR936736
THC2669157	4.2	3.7	ALU1_HUMAN (P39188) Alu subfamily J sequence contamination warning entry, partial (10%)	THC2669157	Unknown	
SLC6A2	4.2	7.2	mRNA for norepinephrine transporter isoform 2, partial cds.	AB022847	Hs.78036	NM_001043
PTPRH	4.2	4.4	protein tyrosine phosphatase, receptor type, H (PTPRH), mRNA	NM_002842	Hs.179770	D15049
A_24_P213375	4.2	1.5	A_24_P213375	A_24_P213375	Unknown	
NAV3	4.2	4.9	neuron navigator 3 (NAV3), mRNA	NM_014903	Hs.655301	NM_014903
ANKRD43	4.2	4.3	ankyrin repeat domain 43 (ANKRD43), mRNA	NM_175873	Hs.13308	NM_175873
ENST00000375672	4.2	3.4	Uncharacterized protein C20orf112.	ENST00000375672	Unknown	
CR622342	4.2	1.9	full-length cDNA clone CS0DJ006YC05 of T cells (Jurkat cell line) Cot 10-normalized of (human).	CR622342	Hs.527105	NM_031372
THEM4	4.2	1.9	thioesterase superfamily member 4 (THEM4), mRNA	NM_053055	Hs.164070	AK096211
MAN1A2	4.2	2.5	Mannosyl-oligosaccharide 1,2-alpha-mannosidase IB (EC 3.2.1.113) (Processing alpha-1,2-mannosidase IB) (Alpha-1,2-mannosidase IB) (Mannosidase alpha class 1A member 2).	ENST00000369453	Unknown	
AK094929	4.2	2.0	cDNA FLJ37610 fis, clone BRCOC2011398.	AK094929	Hs.185701	AK094929
AK074776	4.2	2.4	cDNA FLJ90295 fis, clone NT2RP2000240.	AK074776	Hs.438950	AK074776
ABTB2	4.2	1.7	ankyrin repeat and BTB (POZ) domain containing 2 (ABTB2), mRNA	NM_145804	Hs.23361	AK095632
DYNC1I1	4.2	2.4	dynein, cytoplasmic 1, intermediate chain 1 (DYNC1I1), mRNA	NM_004411	Hs.440364	BC053542
UBE2E3	4.2	1.9	ubiquitin-conjugating enzyme E2E 3 (UBC4/5 homolog, yeast) (UBE2E3), transcript variant 1, mRNA	NM_006357	Hs.470804	BM912832
SMPD3	4.2	19.9	sphingomyelin phosphodiesterase 3, neutral membrane (neutral sphingomyelinase II) (SMPD3), mRNA	NM_018667	Hs.368421	NM_018667
CCDC19	4.2	2.6	coiled-coil domain containing 19 (CCDC19), mRNA	NM_012337	Hs.647705	BC089391
KIAA1713	4.2	5.0	mRNA for KIAA1713 protein, partial cds.	AB051500	Hs.464876	NM_030632
LOC441257	4.2	3.7	hypothetical LOC441257 (LOC441257), mRNA	NM_001023562	Unknown	
AB011119	4.2	3.9	mRNA for KIAA0547 protein, partial cds.	AB011119	Unknown	
ENST00000370892	4.2	2.7	Leucine-rich repeat-containing protein 1 (LAP and no PDZ protein) (LANO adapter protein).	ENST00000370892	Unknown	
NARF	4.2	1.5	nuclear prelamin A recognition factor (NARF), transcript variant 3, mRNA	NM_001038618	Hs.256526	CR620704
ZNF174	4.2	1.4	zinc finger protein 174 (ZNF174), transcript variant 1, mRNA	NM_003450	Hs.155204	NM_003450
LOC653464	4.2	2.2	PREDICTED: similar to SLIT-ROBO Rho GTPase-activating protein 2 (srGAP2) (Formin-binding protein 2) (LOC653464), mRNA	XM_209227	Hs.523529	XM_209227
LRRTM3	4.2	2.5	leucine rich repeat transmembrane neuronal 3 (LRRTM3), mRNA	NM_178011	Hs.652155	NM_178011
C5orf24	4.2	2.2	chromosome 5 open reading frame 24 (C5orf24), mRNA	NM_152409	Hs.406549	BC053677
C4orf18	4.2	14.8	chromosome 4 open reading frame 18 (C4orf18), transcript variant 2, mRNA	NM_016613	Hs.567498	AL832589
SUPT3H	4.2	2.8	Transcription initiation protein SPT3 homolog (SPT3-like protein).	ENST00000371461	Unknown	
C8orf48	4.2	4.3	chromosome 8 open reading frame 48, mRNA (cDNA clone MGC:39641 IMAGE:5266320), complete cds.	BC031245	Hs.104941	AK058131
AL833655	4.2	6.1	mRNA; cDNA DKFZp667O0320 (from clone DKFZp667O0320).	AL833655	Hs.700867	AL833655
SPECC1L	4.2	1.4	SPECC1-like (SPECC1L), mRNA	NM_015330	Hs.474384	NM_015330
AK098629	4.2	1.6	cDNA FLJ25763 fis, clone TST06294.	AK098629	Hs.657362	BF789913
KLF5	4.2	3.8	Kruppel-like factor 5 (intestinal) (KLF5), mRNA	NM_001730	Hs.508234	AF132818
ZNF546	4.2	1.6	zinc finger protein 546 (ZNF546), mRNA	NM_178544	Hs.696156	BC045649
DOK7	4.2	2.3	docking protein 7 (DOK7), mRNA	NM_173660	Hs.122110	NM_173660
REEP3	4.2	1.9	Receptor expression-enhancing protein 3.	ENST00000298249	Unknown	
A_32_P186348	4.2	2.1	A_32_P186348	A_32_P186348	Unknown	
DPY19L1	4.2	2.1	DPY-19-like protein 1 (DPY19L1) mRNA, complete cds.	DQ287932	Hs.408623	NM_015283
STMN2	4.2	7.5	stathmin-like 2 (STMN2), mRNA	NM_007029	Hs.521651	AK091336
PPP1R12B	4.2	3.3	protein phosphatase 1, regulatory (inhibitor) subunit 12B (PPP1R12B), transcript variant 3, mRNA	NM_032103	Hs.444403	NM_032105
ST3GAL3	4.2	2.0	ST3 beta-galactoside alpha-2,3-sialyltransferase 3 (ST3GAL3), transcript variant 1, mRNA	NM_174963	Hs.597915	NM_174963
ST3GAL6	4.2	2.6	ST3 beta-galactoside alpha-2,3-sialyltransferase 6 (ST3GAL6), mRNA	NM_006100	Hs.148716	CR749468
CDC42	4.2	3.0	cell division cycle 42 (GTP binding protein, 25kDa) (CDC42), transcript variant 2, mRNA	NM_044472	Hs.690198	NM_001039802
ZHX3	4.2	2.5	zinc fingers and homeoboxes 3 (ZHX3), mRNA	NM_015035	Hs.380133	NM_015035
AK124576	4.2	2.3	cDNA FLJ42585 fis, clone BRACE3009237.	AK124576	Hs.4749	AK124576
THC2718728	4.2	5.4	THC2718728	THC2718728	Unknown	
LOC285260	4.2	1.4	PREDICTED: similar to ribosomal protein L31 (LOC285260), mRNA	XR_019376	Hs.646857	XR_019376
DCTN6	4.2	1.8	dynactin 6 (DCTN6), mRNA	NM_006571	Hs.158427	NM_006571

ENST00000261569	4.2	2.1	Microtubule-associated serine/threonine-protein kinase 4 (EC 2.7.11.1).	ENST00000261569	Unknown	
LHX4	4.2	2.5	cDNA FLJ38931 fis, clone NT2NE2013189.	AK096250	Hs.496545	AK096250
THC2586873	4.2	2.9	THC2586873	THC2586873	Unknown	
LZIC	4.2	3.1	leucine zipper and CTNNBIP1 domain containing	ENST00000377223	Unknown	
KIAA0753	4.2	1.5	KIAA0753 (KIAA0753), mRNA	NM_014804	Hs.28070	NM_014804
RFPL3S	4.2	2.1	ret finger protein-like 3 antisense (RFPL3S) on chromosome 22	NR_001450	Unknown	
MBL2	4.2	5.1	mannose-binding lectin (protein C) 2, soluble (opsonic defect) (MBL2), mRNA	NM_000242	Hs.499674	NM_000242
CDKN1B	4.2	1.9	cyclin-dependent kinase inhibitor 1B (p27, Kip1) (CDKN1B), mRNA	NM_004064	Hs.238990	NM_004064
CEBPA	4.2	2.4	CCAAT/enhancer binding protein (C/EBP), alpha (CEBPA), mRNA	NM_004364	Hs.699463	NM_004364
ENST00000377492	4.2	3.5	FAM27E1 protein.	ENST00000377492	Unknown	
C10orf12	4.2	1.6	cDNA: FLJ21513 fis, clone COL05778.	AK025166	Hs.14555	AK025166
AATK	4.2	2.4	cDNA FLJ16758 fis, clone BRACE3038687, moderately similar to apoptosis-associated tyrosine kinase (AATK).	AK131529	Hs.514575	NM_001080395
ENST00000343903	4.2	5.1	hypothetical gene supported by BC008048, mRNA (cDNA clone IMAGE:3631157), partial cds.	ENST00000343903	Unknown	
LOC728537	4.2	1.7	cDNA clone IMAGE:5271446.	BC039374	Hs.469369	BC039374
BC007549	4.2	2.8	Homo sapiens, clone IMAGE:2960704, mRNA.	BC007549	Hs.664915	BC007549
CR603982	4.2	2.4	full-length cDNA clone CS0DF021YL03 of Fetal brain of (human).	CR603982	Hs.120633	BX649128
CXCR4	4.2	2.6	chemokine (C-X-C motif) receptor 4 (CXCR4), transcript variant 1, mRNA	NM_001008540	Hs.593413	AF147204
WNT2B	4.2	8.1	wingless-type MMTV integration site family, member 2B (WNT2B), transcript variant WNT-2B1, mRNA	NM_004185	Hs.258575	AK127449
C2orf12	4.2	1.8	chromosome 2 open reading frame 12, mRNA (cDNA clone MGC:70597 IMAGE:5205269), complete cds.	BC065192	Hs.470412	NM_016839
ZNF318	4.2	1.6	zinc finger protein 318 (ZNF318), mRNA	NM_014345	Hs.509718	AF090114
GMFG	4.2	3.0	glia maturation factor, gamma (GMFG), mRNA	NM_004877	Hs.5210	BU934772
POLK	4.2	1.8	polymerase (DNA directed) kappa (POLK), mRNA	NM_016218	Hs.135756	BC041798
1-Mar	4.2	3.0	cDNA FLJ20668 fis, clone KAIA585.	AK000675	Hs.696248	AK025005
FLJ12825	4.2	3.1	cDNA FLJ31855 fis, clone NT2RP7000926.	AK056417	Hs.350378	AK056417
C1orf27	4.2	2.1	chromosome 1 open reading frame 27 (C1orf27), mRNA	NM_017847	Hs.371210	AY854248
KIDINS220	4.2	2.1	kinase D-interacting substance of 220 kDa (KIDINS220), mRNA	NM_020738	Hs.9873	AB033076
RPL23AP13	4.2	3.5	ribosomal protein L23a pseudogene 13 (RPL23AP13) on chromosome 2	NR_002229	Unknown	
ST3GAL6	4.2	2.6	ST3 beta-galactoside alpha-2,3-sialyltransferase 6 (ST3GAL6), mRNA	NM_006100	Hs.148716	CR749468
THC2611204	4.2	5.1	THC2611204	THC2611204	Unknown	
CDKN1B	4.2	1.9	cyclin-dependent kinase inhibitor 1B (p27, Kip1) (CDKN1B), mRNA	NM_004064	Hs.238990	NM_004064
BMP4	4.2	4.4	bone morphogenetic protein 4 (BMP4), transcript variant 1, mRNA	NM_001202	Hs.68879	NM_001202
A_32_P184417	4.2	2.1	A_32_P184417	A_32_P184417	Unknown	
A_24_P160920	4.2	1.3	A_24_P160920	A_24_P160920	Unknown	
GPD1L	4.2	1.9	glycerol-3-phosphate dehydrogenase 1-like (GPD1L), mRNA	NM_015141	Hs.82432	D42047
A_32_P107859	4.2	1.6	A_32_P107859	A_32_P107859	Unknown	
FLJ22795	4.2	1.6	colon cancer-associated antigen AgSK1-2HT-ECS mRNA, complete cds.	AF316855	Hs.498322	AF316855
LNPEP	4.2	2.9	leucyl/cystinyl aminopeptidase (LNPEP), transcript variant 1, mRNA	NM_005575	Hs.656905	AB208883
AK057923	4.2	5.1	cDNA FLJ25194 fis, clone REC04095.	AK057923	Hs.660881	AL832788
C8ORFK32	4.2	2.9	C8orfK32 protein (C8ORFK32), mRNA	NM_015912	Hs.126024	NM_015912
MGC70863	4.2	1.4	similar to RPL23AP7 protein (MGC70863), transcript variant 1, mRNA	NM_203477	Hs.406135	BC065556
CDKN1B	4.2	1.9	cyclin-dependent kinase inhibitor 1B (p27, Kip1) (CDKN1B), mRNA	NM_004064	Hs.238990	NM_004064
LIN7B	4.2	1.8	lin-7 homolog B (C. elegans) (LIN7B), mRNA	NM_022165	Hs.221737	BG749971
CR619760	4.2	1.6	full-length cDNA clone CS0DD006YO12 of Neuroblastoma Cot 50-normalized of (human).	CR619760	Hs.380930	CR619760
AKAP13	4.2	1.8	A kinase (PRKA) anchor protein 13 (AKAP13), transcript variant 1, mRNA	NM_006738	Hs.459211	NM_006738
PRKRIR	4.2	1.6	protein-kinase, interferon-inducible double stranded RNA dependent inhibitor, repressor of (P58 repressor) (PRKRIR), mRNA	NM_004705	Hs.503315	BX641144
C13orf1	4.2	1.9	chromosome 13 open reading frame 1 (C13orf1), mRNA	NM_020456	Hs.44235	AF334405
ZNF627	4.2	2.0	zinc finger protein 627 (ZNF627), mRNA	NM_145295	Hs.526665	BC098416
LOC440181	4.2	2.6	PREDICTED: hypothetical LOC440181 (LOC440181), mRNA	XR_018458	Hs.662146	XR_018458
FLJ31818	4.2	4.7	hypothetical protein FLJ31818 (FLJ31818), mRNA	NM_152556	Hs.489734	NM_152556
MGC3032	4.2	1.9	cDNA FLJ38987 fis, clone NT2RI2005818.	AK096306	Hs.568945	XM_933539
NOSTRIN	4.2	3.3	nitric oxide synthase trafficker (NOSTRIN), transcript variant 1, mRNA	NM_052946	Hs.189780	AK093444
THC2653641	4.2	4.6	THC2653641	THC2653641	Unknown	
COMMD6	4.2	1.8	COMM domain containing 6 (COMMD6), transcript variant 1, mRNA	NM_203497	Hs.508266	NM_203497

FAM70A	4.2	17.4	family with sequence similarity 70, member A (FAM70A), mRNA	NM_017938	Hs.437563	AF450484
ATF6	4.2	1.9	activating transcription factor 6 (ATF6), mRNA	NM_007348	Hs.492740	BX538263
RBM17	4.2	1.6	RNA binding motif protein 17 (RBM17), mRNA	NM_032905	Hs.498548	BX648288
CR609843	4.2	3.5	full-length cDNA clone CS0DD006YM23 of Neuroblastoma Cot 50-normalized of (human).	CR609843	Hs.526415	BX538249
VEGFC	4.2	4.0	vascular endothelial growth factor C (VEGFC), mRNA	NM_005429	Hs.435215	NM_005429
DCTN3	4.2	2.0	dynactin 3 (p22) (DCTN3), transcript variant 1, mRNA	NM_007234	Hs.511768	BM920638
SIAE	4.2	3.0	sialic acid acetylesterase, mRNA (cDNA clone IMAGE:5751560), complete cds.	BC040966	Unknown	
ENST00000377492	4.2	3.4	FAM27E1 protein.	ENST00000377492	Unknown	
LTBP1	4.2	2.2	latent transforming growth factor beta binding protein 1 (LTBP1), transcript variant 1, mRNA	NM_206943	Hs.654497	NM_206943
PLD1	4.2	2.6	cDNA FLJ34578 fis, clone KIDNE2008404, highly similar to PHOSPHOLIPASE D1 (EC 3.1.4.4).	AK091897	Hs.382865	AB209907
KIAA1466	4.2	5.7	mRNA for KIAA1466 protein, partial cds.	AB040899	Unknown	
SNAPAP	4.2	2.3	SNAP-associated protein (SNAPAP), mRNA	NM_012437	Hs.32018	BQ947794
LOC253970	4.2	6.2	cDNA: FLJ23494 fis, clone LNG01885.	AK027147	Hs.509165	AK027147
5-Mar	4.2	1.6	membrane-associated ring finger (C3HC4) 5 (MARCH5), mRNA	NM_017824	Hs.573490	NM_017824
ZNF2	4.2	2.1	zinc finger protein 2 (ZNF2), transcript variant 1, mRNA	NM_021088	Hs.590916	NM_021088
EYA2	4.2	1.9	eyes absent homolog 2 (Drosophila) (EYA2), transcript variant 2, mRNA	NM_172113	Hs.472877	AL833552
NXPH1	4.2	4.3	neurexophilin 1 (NXPH1), mRNA	NM_152745	Hs.487564	NM_152745
HMGN2	4.2	1.4	high-mobility group nucleosomal binding domain 2 (HMGN2), mRNA	NM_005517	Hs.181163	BC110390
KIAA0232	4.2	2.1	KIAA0232 gene product (KIAA0232), mRNA	NM_014743	Hs.79276	NM_014743
SYTL3	4.2	4.2	synaptotagmin-like 3 (SYTL3), mRNA	NM_001009991	Hs.436977	AL833750
MAP2	4.2	3.8	microtubule-associated protein 2 (MAP2), transcript variant 1, mRNA	NM_002374	Hs.368281	NM_002374
H2AFY2	4.2	1.8	H2A histone family, member Y2 (H2AFY2), mRNA	NM_018649	Hs.499953	AL359572
OTUD1	4.2	2.0	mRNA, clone: TH020D07.	AB188491	Hs.499042	XM_166659
PEX11A	4.2	2.0	peroxisomal biogenesis factor 11A (PEX11A), mRNA	NM_003847	Hs.31034	AL360141
IKZF2	4.2	2.4	IKAROS family zinc finger 2 (Helios) (IKZF2), transcript variant 2, mRNA	NM_001079526	Hs.604950	NM_001079526
AK022430	4.2	5.4	cDNA FLJ12368 fis, clone MAMMA1002417.	AK022430	Hs.659110	AK022430
RBL2	4.2	1.6	retinoblastoma-like 2 (p130) (RBL2), mRNA	NM_005611	Hs.513609	BC034490
ARMC8	4.2	1.4	mRNA; cDNA DKFZp434A043 (from clone DKFZp434A043); partial cds.	AL096748	Hs.266826	AL096748
THC2739236	4.2	3.4	Q21ZB1_9BURK (Q21ZB1) Peptidase S11, D-alanyl-D-alanine carboxypeptidase 1 precursor, partial (5%)	THC2739236	Unknown	
FAM104B	4.2	1.6	family with sequence similarity 104, member B, mRNA (cDNA clone IMAGE:3939163), partial cds.	BC006406	Hs.415414	BM906096
SIPA1L2	4.2	4.4	signal-induced proliferation-associated 1 like 2 (SIPA1L2), mRNA	NM_020808	Hs.268774	AY168879
RPGR	4.2	1.5	retinitis pigmentosa GTPase regulator (RPGR), transcript variant B, mRNA	NM_001023582	Unknown	
FOXP2	4.2	5.5	forkhead box P2 (FOXP2), transcript variant 4, mRNA	NM_148900	Unknown	
EBI2	4.2	4.5	Epstein-Barr virus induced gene 2 (lymphocyte-specific G protein-coupled receptor) (EBI2), mRNA	NM_004951	Hs.784	NM_004951
THC2712710	4.2	3.1	AL590556 elastase 3B, pancreatic {Homo sapiens} (exp=0; wgp=1; cg=0), partial (10%)	THC2712710	Unknown	
LOC90246	4.2	2.2	cDNA FLJ13573 fis, clone PLACE1008584.	AK023635	Hs.635164	AK023635
KLHL9	4.2	2.9	cDNA FLJ12743 fis, clone NT2RP2000678.	AK022805	Unknown	
MKRN2	4.2	2.0	makorin, ring finger protein, 2 (MKRN2), mRNA	NM_014160	Hs.591666	BC015715
CCDC23	4.2	2.4	coiled-coil domain containing 23 (CCDC23), mRNA	NM_199342	Hs.113919	CD243680
TMEM5	4.2	2.0	transmembrane protein 5 (TMEM5), mRNA	NM_014254	Hs.699516	BX647170
BTBD7	4.2	1.5	BTB (POZ) domain containing 7 (BTBD7), transcript variant 1, mRNA	NM_001002860	Hs.525549	NM_001002860
TXNDC6	4.2	1.9	thioredoxin domain containing 6 (TXNDC6), mRNA	NM_178130	Hs.660992	NM_178130
C1orf64	4.2	2.6	chromosome 1 open reading frame 64 (C1orf64), mRNA	NM_178840	Hs.29190	AK127425
DBH	4.2	5.3	dopamine beta-hydroxylase (dopamine beta-monooxygenase) (DBH), mRNA	NM_000787	Hs.591890	NM_000787
PAFAH1B3	4.2	1.5	platelet-activating factor acetylhydrolase, isoform Ib, gamma subunit 29kDa (PAFAH1B3), mRNA	NM_002573	Hs.466831	BM904583
ALKBH2	4.2	1.8	alkB, alkylation repair homolog 2 (E. coli) (ALKBH2), mRNA	NM_001001655	Hs.374458	BG256986
THC2526765	4.2	1.6	CV569217 od02h01.y1 Human keratoconus cornea, unamplified, (od/oe) cDNA clone od02h01 5', mRNA sequence	THC2526765	Unknown	
AK056855	4.2	1.8	cDNA FLJ32293 fis, clone PROST2001739.	AK056855	Hs.367885	AK056855
PAH	4.2	3.6	phenylalanine hydroxylase (PAH), mRNA	NM_000277	Hs.643451	NM_000277
IL11RA	4.2	2.4	interleukin 11 receptor, alpha (IL11RA), transcript variant 2, mRNA	NM_147162	Hs.591088	BC003110
NDFIP2	4.2	1.8	Nedd4 family interacting protein 2 (NDFIP2), mRNA	NM_019080	Hs.525093	AB032991
ICA1L	4.2	2.5	islet cell autoantigen 1,69kDa-like (ICA1L), transcript variant 1, mRNA	NM_138468	Hs.516629	NM_138468

THC2710559	4.2	3.5	THC2710559	THC2710559	Unknown	
BCLAF1	4.2	1.5	BCL2-associated transcription factor 1 (BCLAF1), transcript variant 1, mRNA	NM_014739	Hs.486542	NM_014739
BC064969	4.2	1.6	cDNA clone IMAGE:6149643, partial cds.	BC064969	Hs.458747	AK127498
AF085995	4.2	3.2	full length insert cDNA clone YU26H08.	AF085995	Hs.28425	AK124747
CDKN1B	4.2	1.9	cyclin-dependent kinase inhibitor 1B (p27, Kip1) (CDKN1B), mRNA	NM_004064	Hs.238990	NM_004064
FLRT1	4.1	5.4	fibronectin leucine rich transmembrane protein 1 (FLRT1), mRNA	NM_013280	Hs.584876	AY358308
TXNIP	4.1	5.1	thioredoxin interacting protein (TXNIP), mRNA	NM_006472	Hs.533977	S73591
KIAA1345	4.1	1.5	mRNA for KIAA1345 protein, partial cds.	AB037766	Hs.590928	BX647334
MOBKL1A	4.1	1.4	MOB1, Mps One Binder kinase activator-like 1A (yeast) (MOBKL1A), mRNA	NM_173468	Hs.31422	NM_173468
MSL3L1	4.1	1.9	male-specific lethal 3-like 1 (Drosophila) (MSL3L1), transcript variant 1, mRNA	NM_078629	Hs.655288	NM_078628
AK022443	4.1	2.0	cDNA FLJ12381 fis, clone MAMMA1002566.	AK022443	Hs.656237	AK022443
NEFL	4.1	2.5	neurofilament, light polypeptide 68kDa (NEFL), mRNA	NM_006158	Hs.521461	NM_006158
MYH7	4.1	8.4	myosin, heavy chain 7, cardiac muscle, beta (MYH7), mRNA	NM_000257	Hs.278432	NM_000257
PYGM	4.1	2.7	Glycogen phosphorylase, muscle form (EC 2.4.1.1) (Myophosphorylase).	ENST00000377444	Unknown	
EPHB2	4.1	2.8	EPH receptor B2 (EPHB2), transcript variant 2, mRNA	NM_004442	Hs.523329	NM_004442
OPCML	4.1	3.3	opioid binding protein/cell adhesion molecule-like (OPCML), transcript variant 2, mRNA	NM_001012393	Hs.4817	NM_001012393
THC2537951	4.1	2.8	ALU1_HUMAN (P39188) Alu subfamily J sequence contamination warning entry, partial (27%)	THC2537951	Unknown	
AK057710	4.1	1.6	cDNA FLJ33148 fis, clone UTERU2000238.	AK057710	Hs.597376	AK057710
HBP1	4.1	2.3	HMG-box transcription factor 1 (HBP1), mRNA	NM_012257	Hs.162032	NM_012257
ENST00000360150	4.1	1.8	Endoplasmic reticulum-Golgi intermediate compartment protein 2.	ENST00000360150	Unknown	
IL7	4.1	2.7	interleukin 7 (IL7), mRNA	NM_000880	Hs.591873	BC047698
AMH	4.1	1.6	anti-Mullerian hormone (AMH), mRNA	NM_000479	Hs.112432	NM_000479
CRTC3	4.1	2.3	CREB regulated transcription coactivator 3 (CRTC3), transcript variant 1, mRNA	NM_022769	Hs.567572	AK090443
BC040678	4.1	3.5	cDNA clone IMAGE:4817707.	BC040678	Hs.491856	AK127279
C10orf22	4.1	1.6	chromosome 10 open reading frame 22 (C10orf22), mRNA	NM_032804	Hs.99821	NM_032804
AK094342	4.1	4.6	cDNA FLJ37023 fis, clone BRACE2010828.	AK094342	Hs.649971	AK094342
RNF139	4.1	1.5	ring finger protein 139 (RNF139), mRNA	NM_007218	Hs.632057	NM_007218
MON2	4.1	1.8	MON2 homolog (S. cerevisiae) (MON2), mRNA	NM_015026	Hs.389378	NM_015026
RGS3	4.1	3.2	regulator of G-protein signalling 3 (RGS3), transcript variant 4, mRNA	NM_134427	Hs.494875	NM_144488
THC2673108	4.1	6.1	HSCLOCK17 clock {Homo sapiens} (exp=-1; wgp=0; cg=0), partial (3%)	THC2673108	Unknown	
APC	4.1	1.7	adenomatosis polyposis coli (APC), mRNA	NM_000038	Hs.158932	NM_000038
CDKN1B	4.1	1.9	cyclin-dependent kinase inhibitor 1B (p27, Kip1) (CDKN1B), mRNA	NM_004064	Hs.238990	NM_004064
RBJ	4.1	1.5	Ras-associated protein Rap1 (RBJ), mRNA	NM_016544	Hs.434993	BC034049
TRIM13	4.1	2.9	tripartite motif-containing 13 (TRIM13), transcript variant 3, mRNA	NM_213590	Hs.436922	NM_213590
THSD7A	4.1	4.2	cDNA FLJ11022 fis, clone PLACE1003771.	AK001884	Hs.648482	AK001884
ADH6	4.1	2.8	mRNA; cDNA DKFZp686N1452 (from clone DKFZp686N1452).	BX647987	Hs.660296	BX647987
HOXD3	4.1	16.7	homeobox D3 (HOXD3), mRNA	NM_006898	Hs.93574	BC005124
GDF10	4.1	19.3	growth differentiation factor 10 (GDF10), mRNA	NM_004962	Hs.2171	NM_004962
DZIP1	4.1	1.4	DAZ interacting protein 1 (DZIP1), transcript variant 2, mRNA	NM_198968	Hs.656580	NM_198968
WWOX	4.1	1.6	WW domain containing oxidoreductase (WWOX), transcript variant 1, mRNA	NM_016373	Hs.461453	NM_016373
FLJ43080	4.1	4.3	cDNA FLJ43080 fis, clone BRTHA3017047.	AK125070	Hs.535781	AK125070
DSCR6	4.1	3.5	Down syndrome critical region gene 6 (DSCR6), mRNA	NM_018962	Hs.254560	NM_018962
LOC391181	4.1	1.7	PREDICTED: similar to 60S ribosomal protein L6 (TAX-responsive enhancer element-binding protein 107) (TAXREB107) (Neoplasm-related protein C140) (LOC391181), mRNA	XR_017056	Hs.693417	XR_018994
PDE1A	4.1	19.7	phosphodiesterase 1A, calmodulin-dependent (PDE1A), transcript variant 2, mRNA	NM_001003683	Hs.191046	NM_005019
GJB2	4.1	11.4	gap junction protein, beta 2, 26kDa (connexin 26) (GJB2), mRNA	NM_004004	Hs.591234	NM_004004
LOC647768	4.1	5.6	PREDICTED: similar to Tetratricopeptide repeat protein 3 (TPR repeat protein 3) (TPR repeat protein D) (LOC647768), mRNA	XR_018202	Hs.567832	XR_018202
OCA2	4.1	3.8	oculocutaneous albinism II (pink-eye dilution homolog, mouse) (OCA2), mRNA	NM_000275	Hs.654411	NM_000275
NOTCH2NL	4.1	3.1	Notch homolog 2 (Drosophila) N-terminal like (NOTCH2NL), mRNA	NM_203458	Hs.515947	BX538005
COL27A1	4.1	1.8	cDNA FLJ11895 fis, clone HEMBA1007301, weakly similar to COLLAGEN ALPHA 1(III) CHAIN.	AK021957	Hs.494892	NM_032888
THC2497780	4.1	2.1	THC2497780	THC2497780	Unknown	
C1orf119	4.1	2.1	chromosome 1 open reading frame 119 (C1orf119), mRNA	NM_020141	Hs.82933	BC066918
DLL3	4.1	3.2	delta-like 3 (Drosophila) (DLL3), transcript variant 1, mRNA	NM_016941	Hs.127792	NM_016941

DSP	4.1	1.6	desmoplakin (DSP), transcript variant 1, mRNA	NM_004415	Hs.519873	NM_004415
IGLL1	4.1	2.2	immunoglobulin lambda-like polypeptide 1 (IGLL1), transcript variant 1, mRNA	NM_020070	Hs.348935	NM_020070
CDKN1B	4.1	1.9	cyclin-dependent kinase inhibitor 1B (p27, Kip1) (CDKN1B), mRNA	NM_004064	Hs.238990	NM_004064
RANBP9	4.1	1.3	RAN binding protein 9 (RANBP9), mRNA	NM_005493	Hs.306242	BC052781
PLRG1	4.1	2.1	Pleiotropic regulator 1.	ENST00000302078	Unknown	
GCNT3	4.1	2.3	glucosaminyl (N-acetyl) transferase 3, mucin type	ENST00000267857	Unknown	
ZNF177	4.1	3.0	zinc finger protein 177 (ZNF177), mRNA	NM_003451	Hs.172979	BC034489
AK090803	4.1	2.2	cDNA FLJ33484 fis, clone BRAMY2003117.	AK090803	Hs.254414	NM_080743
EIF2C1	4.1	1.4	eukaryotic translation initiation factor 2C, 1 (EIF2C1), mRNA	NM_012199	Hs.22867	NM_012199
NBR1	4.1	1.8	neighbor of BRCA1 gene 1 (NBR1), transcript variant 2, mRNA	NM_031858	Hs.546264	NM_031858
FZD3	4.1	1.7	frizzled homolog 3 (Drosophila) (FZD3), mRNA	NM_017412	Hs.40735	NM_017412
BAG1	4.1	1.8	BCL2-associated athanogene (BAG1), mRNA	NM_004323	Hs.377484	NM_004323
COL1A1	4.1	2.2	H.sapiens mRNA for prepro-alpha1(I) collagen.	Z74615	Hs.172928	Z74615
ACTR8	4.1	1.6	ARP8 actin-related protein 8 homolog (yeast) (ACTR8), mRNA	NM_022899	Hs.412186	NM_022899
VEZT	4.1	1.5	vezatin, adherens junctions transmembrane protein (VEZT), mRNA	NM_017599	Hs.24135	NM_017599
RNF11	4.1	2.0	ring finger protein 11 (RNF11), mRNA	NM_014372	Hs.696036	NM_014372
C1orf125	4.1	3.4	chromosome 1 open reading frame 125 (C1orf125), transcript variant 1, mRNA	NM_144696	Hs.658505	BX647935
MLC1	4.1	5.3	megalencephalic leukoencephalopathy with subcortical cysts 1 (MLC1), transcript variant 1, mRNA	NM_015166	Hs.517729	NM_015166
TMEM99	4.1	1.8	transmembrane protein 99 (TMEM99), mRNA	NM_145274	Hs.353163	NM_145274
LOC647500	4.1	3.3	PREDICTED: similar to phosphodiesterase 4D interacting protein isoform 1 (LOC647500), mRNA	XM_001128547	Hs.656830	CR610073
AF075027	4.1	2.0	full length insert cDNA YI37C01.	AF075027	Hs.432924	CD673367
ARV1	4.1	2.0	ARV1 homolog (S. cerevisiae) (ARV1), mRNA	NM_022786	Hs.700665	AK092987
CDKN1B	4.1	1.9	cyclin-dependent kinase inhibitor 1B (p27, Kip1) (CDKN1B), mRNA	NM_004064	Hs.238990	NM_004064
CAMK1G	4.1	2.7	calcium/calmodulin-dependent protein kinase IG (CAMK1G), mRNA	NM_020439	Hs.199068	AK095713
ZNF251	4.1	2.6	cDNA FLJ14380 fis, clone HEMBA1001819, moderately similar to ZINC FINGER PROTEIN 184.	AK027286	Hs.534516	NM_138367
ENST00000321614	4.1	2.0	TIGA1 (Chromosome 5 open reading frame 26).	ENST00000321614	Unknown	
ARID4B	4.1	2.8	AT rich interactive domain 4B (RBP1-like) (ARID4B), transcript variant 1, mRNA	NM_016374	Hs.575782	NM_016374
SEC63	4.1	2.5	Translocation protein SEC63 homolog.	ENST00000369002	Unknown	
RPS25	4.1	1.5	ribosomal protein S25 (RPS25), mRNA	NM_001028	Hs.512676	BM554735
RPL5	4.1	2.2	ribosomal protein L5 (RPL5), mRNA	NM_000969	Hs.532359	BM903678
AK130896	4.1	3.5	cDNA FLJ27386 fis, clone UBA09375.	AK130896	Hs.666839	AK130896
ST8SIA2	4.1	2.5	ST8 alpha-N-acetyl-neuraminide alpha-2,8-sialyltransferase 2 (ST8SIA2), mRNA	NM_006011	Hs.302341	NM_006011
VEZF1	4.1	1.9	vascular endothelial zinc finger 1 (VEZF1), mRNA	NM_007146	Hs.463569	NM_007146
GPR56	4.1	6.6	G protein-coupled receptor 56 (GPR56), transcript variant 3, mRNA	NM_201525	Hs.513633	NM_201524
CPS1	4.1	2.2	carbamoyl-phosphate synthetase 1, mitochondrial (CPS1), mRNA	NM_001875	Hs.149252	NM_001875
HMGB1	4.1	1.3	high-mobility group box 1 (HMGB1), mRNA	NM_002128	Hs.434102	AK122825
AF119866	4.1	2.0	PRO2206 mRNA, complete cds.	AF119866	Unknown	
CR591289	4.1	2.3	full-length cDNA clone CS0DF034YI03 of Fetal brain of (human).	CR591289	Hs.7572	AK054902
MSRA	4.1	2.3	methionine sulfoxide reductase A (MSRA), mRNA	NM_012331	Hs.490981	AK123132
ST3GAL6	4.1	2.5	ST3 beta-galactoside alpha-2,3-sialyltransferase 6 (ST3GAL6), mRNA	NM_006100	Hs.148716	CR749468
CPS1	4.1	2.1	carbamoyl-phosphate synthetase 1, mitochondrial (CPS1), mRNA	NM_001875	Hs.149252	NM_001875
GJB2	4.1	8.2	gap junction protein, beta 2, 26kDa (connexin 26) (GJB2), mRNA	NM_004004	Hs.591234	NM_004004
BM819787	4.1	4.2	BM819787 K-EST0087976 S16N667673 cDNA clone S16N667673-2-E05 5', mRNA sequence	BM819787	Hs.648544	DA395839
PHPT1	4.1	1.7	phosphohistidine phosphatase 1 (PHPT1), mRNA	NM_014172	Hs.409834	BM908690
LOC400642	4.1	4.1	hypothetical gene supported by BC041875; BX648984, mRNA (cDNA clone IMAGE:5271951).	BC041875	Hs.643553	BC041875
MYST4	4.1	1.7	MYST histone acetyltransferase (monocytic leukemia) 4 (MYST4), mRNA	NM_012330	Hs.35758	NM_012330
THC2740750	4.1	2.0	THC2740750	THC2740750	Unknown	
DYRK3	4.1	2.5	dual-specificity tyrosine-(Y)-phosphorylation regulated kinase 3 (DYRK3), transcript variant 2, mRNA	NM_001004023	Hs.164267	AF186774
GOLGB1	4.1	1.6	golgi autoantigen, golgin subfamily b, macrogolgin (with transmembrane signal), 1 (GOLGB1), mRNA	NM_004487	Hs.213389	NM_004487
GJB2	4.1	3.6	gap junction protein, beta 2, 26kDa (connexin 26) (GJB2), mRNA	NM_004004	Hs.591234	NM_004004
HNRPK	4.1	1.5	heterogeneous nuclear ribonucleoprotein K (HNRPK), transcript variant 1, mRNA	NM_002140	Hs.695973	NM_002140
RPL31P10	4.1	1.5	PREDICTED: similar to ribosomal protein L31 (LOC390283), mRNA	XR_018695	Hs.646426	XR_018695

LEAP2	4.1	2.0	liver expressed antimicrobial peptide 2 (LEAP2), mRNA	NM_052971	Hs.337588	AJ409065
HNRPA1	4.1	1.4	heterogeneous nuclear ribonucleoprotein A1 (HNRPA1), transcript variant 2, mRNA	NM_031157	Hs.699190	NM_031157
AL049443	4.1	3.4	mRNA; cDNA DKFZp586N2020 (from clone DKFZp586N2020).	AL049443	Hs.660870	AL049443
AL117599	4.1	2.0	mRNA; cDNA DKFZp564I0463 (from clone DKFZp564I0463).	AL117599	Hs.481186	NM_173872
RPL34	4.1	1.6	ribosomal protein L34 (RPL34), transcript variant 2, mRNA	NM_033625	Hs.438227	BG112770
RAPGEF4	4.1	2.6	Rap guanine nucleotide exchange factor (GEF) 4 (RAPGEF4), mRNA	NM_007023	Hs.470646	AB209681
MEIS3P1	4.1	1.7	Meis1 homolog 3 (mouse) pseudogene 1 (MEIS3P1) on chromosome 17	NR_002211	Unknown	
TYMS	4.1	1.8	thymidylate synthetase (TYMS), mRNA	NM_001071	Hs.592338	BQ056428
HNRPA3	4.1	1.5	heterogeneous nuclear ribonucleoprotein A3 (HNRPA3), mRNA	NM_194247	Hs.516539	NM_194247
AK127132	4.1	1.7	cDNA FLJ45189 fis, clone BRAWH3049068.	AK127132	Hs.596399	AK127132
PIGZ	4.1	3.1	phosphatidylinositol glycan anchor biosynthesis, class Z (PIGZ), mRNA	NM_025163	Hs.518403	NM_025163
PCSK6	4.1	2.0	proprotein convertase subtilisin/kexin type 6 (PCSK6), transcript variant 1, mRNA	NM_002570	Hs.498494	NM_002570
LOC137886	4.1	1.7	hypothetical protein LOC137886 (LOC137886), mRNA	NM_001077619	Hs.155572	NM_001077619
DMRT3	4.1	3.3	doublesex and mab-3 related transcription factor 3 (DMRT3), mRNA	NM_021240	Hs.189174	NM_021240
PKN2	4.1	2.1	protein kinase N2 (PKN2), mRNA	NM_006256	Hs.440833	NM_006256
THC2701372	4.1	2.2	Q6NZ52_HUMAN (Q6NZ52) Ribosomal protein L27a, partial (24%)	THC2701372	Unknown	
HADH	4.1	1.4	hydroxyacyl-Coenzyme A dehydrogenase (HADH), nuclear gene encoding mitochondrial protein, mRNA	NM_005327	Hs.438289	NM_005327
MAGED1	4.1	2.0	NRAGE mRNA, complete cds.	AF217963	Hs.5258	NM_001005333
THC2685646	4.1	2.1	THC2685646	THC2685646	Unknown	
COG3	4.1	1.5	component of oligomeric golgi complex 3 (COG3), mRNA	NM_031431	Hs.507948	AF349676
FTCD	4.1	3.3	formiminotransferase cyclodeaminase (FTCD), transcript variant A, mRNA	NM_206965	Hs.415846	AF289022
THC2686940	4.1	3.8	THC2686940	THC2686940	Unknown	
A_24_P333112	4.1	1.5	A_24_P333112	A_24_P333112	Unknown	
RP11-130N24.1	4.1	3.4	KIAA2022 protein (KIAA2022), mRNA	NM_001008537	Hs.124128	AY563507
ABCB1	4.1	2.3	ATP-binding cassette, sub-family B (MDR/TAP), member 1 (ABCB1), mRNA	NM_000927	Hs.489033	NM_000927
HEXIM1	4.1	4.8	hexamethylene bis-acetamide inducible 1 (HEXIM1), mRNA	NM_006460	Hs.15299	NM_006460
MGC33846	4.1	2.3	hypothetical protein MGC33846 (MGC33846), mRNA	NM_175885	Hs.448218	NM_175885
C5orf35	4.1	1.4	chromosome 5 open reading frame 35 (C5orf35), mRNA	NM_153706	Hs.85950	AK093147
TPBG	4.1	4.5	Trophoblast glycoprotein precursor (5T4 oncofetal trophoblast glycoprotein) (5T4 oncotrophoblast glycoprotein) (5T4 oncofetal antigen) (M6P1).	ENST00000369750	Unknown	
MAPK8	4.1	1.7	Mitogen-activated protein kinase 8 (EC 2.7.11.24) (Stress-activated protein kinase JNK1) (c-Jun N-terminal kinase 1) (JNK-46).	ENST00000374189	Unknown	
LOC256021	4.1	3.9	cDNA FLJ30877 fis, clone FEBRA2004443.	AK055439	Hs.651357	BC009050
MAPK8	4.1	1.7	Mitogen-activated protein kinase 8 (EC 2.7.11.24) (Stress-activated protein kinase JNK1) (c-Jun N-terminal kinase 1) (JNK-46).	ENST00000374189	Unknown	
ITFG1	4.1	1.8	integrin alpha FG-GAP repeat containing 1 (ITFG1), mRNA	NM_030790	Hs.42217	BC024162
FSIP1	4.1	2.5	fibrous sheath interacting protein 1 (FSIP1), mRNA	NM_152597	Hs.129598	BC045191
SORCS1	4.1	2.1	sortilin-related VPS10 domain containing receptor 1 (SORCS1), transcript variant 1, mRNA	NM_052918	Hs.591915	NM_052918
RP9	4.1	1.7	retinitis pigmentosa 9 (autosomal dominant) (RP9), mRNA	NM_203288	Hs.326805	NM_203288
CAPN9	4.1	3.2	calpain 9 (CAPN9), transcript variant 1, mRNA	NM_006615	Hs.498021	AB038463
LOC400456	4.1	11.4	hypothetical gene supported by BC040875, mRNA (cDNA clone IMAGE:5721930).	BC040875	Hs.632160	BC040875
THC2636875	4.1	3.0	Q7KZF6_HUMAN (Q7KZF6) Thyroid transcription factor 1, partial (15%)	THC2636875	Unknown	
BRMS1L	4.1	2.1	breast cancer metastasis-suppressor 1-like (BRMS1L), mRNA	NM_032352	Hs.525299	NM_032352
F5	4.1	3.7	coagulation factor V (proaccelerin, labile factor) (F5), mRNA	NM_000130	Hs.30054	NM_000130
TLE6	4.1	1.8	transducin-like enhancer of split 6 (E(sp1) homolog, Drosophila) (TLE6), mRNA	NM_024760	Hs.334507	BC020206
PXMP3	4.1	2.6	peroxisomal membrane protein 3, 35kDa (Zellweger syndrome) (PXMP3), mRNA	NM_000318	Hs.437966	NM_000318
TRIM68	4.1	1.8	tripartite motif-containing 68 (TRIM68), mRNA	NM_018073	Hs.523438	AF360739
WBP4	4.1	1.7	WW domain binding protein 4 (formin binding protein 21) (WBP4), mRNA	NM_007187	Hs.411300	AF071185
HGSNAT	4.1	2.6	heparan-alpha-glucosaminide N-acetyltransferase, mRNA (cDNA clone IMAGE:3880903), complete cds.	BC012452	Hs.600384	NM_152419
DCTN4	4.1	1.7	dynactin 4 (p62) (DCTN4), mRNA	NM_016221	Hs.328865	AK125973
DOCK11	4.1	4.5	dedicator of cytokinesis 11 (DOCK11), mRNA	NM_144658	Hs.368203	NM_144658
PSD3	4.1	2.1	pleckstrin and Sec7 domain containing 3 (PSD3), transcript variant 1, mRNA	NM_015310	Hs.434255	NM_015310
A_24_P929974	4.1	1.6	A_24_P929974	A_24_P929974	Unknown	
APC	4.1	1.9	adenomatosis polyposis coli (APC), mRNA	NM_000038	Hs.158932	NM_000038

ARHGAP20	4.1	2.8	Rho GTPase activating protein 20 (ARHGAP20), mRNA	NM_020809	Hs.6136	NM_020809
SIRPA	4.1	1.9	signal-regulatory protein alpha (SIRPA), transcript variant 1, mRNA	NM_001040022	Hs.581021	NM_001040022
AFTPH	4.1	1.4	aftiphilin (AFTPH), transcript variant 2, mRNA	NM_017657	Hs.655167	AL833962
RAB4A	4.0	2.5	RAB4A, member RAS oncogene family (RAB4A), mRNA	NM_004578	Hs.296169	AY585832
CCDC101	4.0	1.6	coiled-coil domain containing 101 (CCDC101), mRNA	NM_138414	Hs.655476	BC011981
BICD2	4.0	1.9	bicaudal D homolog 2 (Drosophila) (BICD2), transcript variant 2, mRNA	NM_015250	Hs.436939	BC073970
PDK2	4.0	1.6	pyruvate dehydrogenase kinase, isozyme 2 (PDK2), mRNA	NM_002611	Hs.256667	AK055119
CYP4V2	4.0	3.5	cytochrome P450, family 4, subfamily V, polypeptide 2 (CYP4V2), mRNA	NM_207352	Hs.237642	BX648730
ADAMTS20	4.0	2.1	cDNA FLJ13166 fis, clone NT2RP3003701, weakly similar to F-SPONDIN PRECURSOR.	AK023228	Hs.287554	AF488804
ADAM28	4.0	2.1	ADAM metallopeptidase domain 28 (ADAM28), transcript variant 1, mRNA	NM_014265	Hs.174030	AK225921
CNTN4	4.0	9.8	contactin 4 (CNTN4), transcript variant 3, mRNA	NM_175613	Hs.298705	NM_175607
LRRN6C	4.0	1.5	leucine rich repeat neuronal 6C (LRRN6C), mRNA	NM_152570	Hs.699432	AK056372
TAS2R14	4.0	1.5	taste receptor, type 2, member 14 (TAS2R14), mRNA	NM_023922	Hs.679406	CD558711
HCRTR2	4.0	2.8	hypocretin (orexin) receptor 2 (HCRTR2), mRNA	NM_001526	Hs.151624	AF041245
TIGD4	4.0	1.8	tigger transposable element derived 4 (TIGD4), mRNA	NM_145720	Hs.301243	BC037869
AK024956	4.0	2.1	cDNA: FLJ21303 fis, clone COL02107.	AK024956	Hs.677304	AK024956
ZCWPW1	4.0	1.8	zinc finger, CW type with PWWP domain 1 (ZCWPW1), mRNA	NM_017984	Hs.105191	AL136735
ZZZ3	4.0	2.2	zinc finger, ZZ-type containing 3 (ZZZ3), mRNA	NM_015534	Hs.480506	AK074119
PSMF1	4.0	1.8	proteasome (prosome, macropain) inhibitor subunit 1 (PI31) (PSMF1), transcript variant 1, mRNA	NM_006814	Hs.471917	NM_006814
C7orf42	4.0	1.7	chromosome 7 open reading frame 42 (C7orf42), mRNA	NM_017994	Hs.488478	NM_017994
RAD9B	4.0	7.4	RAD9 homolog B (S. cerevisiae) (RAD9B), mRNA	NM_152442	Hs.97794	NM_152442
AK002005	4.0	3.0	cDNA FLJ11143 fis, clone PLACE1006598.	AK002005	Hs.574781	AK002005
THC2686813	4.0	1.9	THC2686813	THC2686813	Unknown	
MGST3	4.0	3.5	microsomal glutathione S-transferase 3 (MGST3), mRNA	NM_004528	Hs.191734	BX537737
ENST00000389400	4.0	1.6	similar to 40S ribosomal protein S3a (V-fos transformation effector protein) (LOC391706), mRNA	ENST00000389400	Unknown	
MLLT3	4.0	2.6	myeloid/lymphoid or mixed-lineage leukemia (trithorax homolog, Drosophila); translocated to, 3 (MLLT3), mRNA	NM_004529	Hs.591085	NM_004529
THC2538610	4.0	2.2	THC2538610	THC2538610	Unknown	
LOC220594	4.0	2.0	TL132 protein (LOC220594), mRNA	NM_145809	Unknown	
LBH	4.0	2.2	limb bud and heart development homolog (mouse) (LBH), mRNA	NM_030915	Hs.567598	BC109376
FHIT	4.0	1.7	fragile histidine triad gene (FHIT), mRNA	NM_002012	Hs.655995	AK127931
MGC33556	4.0	3.6	hypothetical LOC339541 (MGC33556), mRNA	NM_001004307	Unknown	
KIF9	4.0	1.6	kinesin family member 9 (KIF9), transcript variant 3, mRNA	NM_182903	Hs.373947	NM_182902
KIAA1729	4.0	2.5	KIAA1729 protein (KIAA1729), mRNA	NM_053042	Hs.455089	NM_053042
CPS1	4.0	2.0	carbamoyl-phosphate synthetase 1, mitochondrial (CPS1), mRNA	NM_001875	Hs.149252	NM_001875
AK129542	4.0	3.7	cDNA FLJ26031 fis, clone PNC08078.	AK129542	Hs.380132	AK129542
COL4A5	4.0	1.8	collagen, type IV, alpha 5 (Alport syndrome) (COL4A5), transcript variant 2, mRNA	NM_033380	Hs.369089	NM_033380
KIAA1377	4.0	1.8	KIAA1377 (KIAA1377), mRNA	NM_020802	Hs.156352	NM_020802
PRKCE	4.0	2.7	protein kinase C, epsilon (PRKCE), mRNA	NM_005400	Hs.580351	NM_005400
ZNF326	4.0	1.6	zinc finger protein 326 (ZNF326), transcript variant 1, mRNA	NM_182976	Hs.306221	BC090866
RPL39	4.0	1.6	ribosomal protein L39 (RPL39), mRNA	NM_001000	Hs.558387	CR625387
CSRP2	4.0	1.5	cysteine and glycine-rich protein 2 (CSRP2), mRNA	NM_001321	Hs.530904	AB209321
PMS2CL	4.0	1.7	PMS2-C terminal-like, mRNA (cDNA clone MGC:43831 IMAGE:5273238), complete cds.	BC041364	Hs.73105	BC041364
DFNA5	4.0	2.6	deafness, autosomal dominant 5 (DFNA5), mRNA	NM_004403	Hs.520708	AK094714
PPM1D	4.0	1.6	protein phosphatase 1D magnesium-dependent, delta isoform (PPM1D), mRNA	NM_003620	Hs.591184	NM_003620
FGFR1OP2	4.0	1.7	FGFR1 oncogene partner 2 (FGFR1OP2), mRNA	NM_015633	Hs.591162	AK094888
SDSL	4.0	2.4	serine dehydratase-like (SDSL), mRNA	NM_138432	Hs.337594	BC009849
KNDC1	4.0	2.3	kinase non-catalytic C-lobe domain (KIND) containing 1 (KNDC1), transcript variant 1, mRNA	NM_152643	Hs.530685	NM_152643
C3orf39	4.0	1.6	chromosome 3 open reading frame 39 (C3orf39), mRNA	NM_032806	Hs.12313	AK124737
LOC391560	4.0	1.6	PREDICTED: similar to 60S ribosomal protein L32 (LOC391560), mRNA	XR_016842	Hs.693395	XR_018524
SEC61A2	4.0	2.3	Sec61 alpha 2 subunit (S. cerevisiae), mRNA (cDNA clone MGC:32910 IMAGE:4823130), complete cds.	BC026179	Hs.112955	AK057532
ZNF781	4.0	6.6	zinc finger protein 781 (ZNF781), mRNA	NM_152605	Hs.631565	NM_152605
LOC731748	4.0	10.1	PREDICTED: similar to core1 UDP-galactose:N-acetylgalactosamine-alpha-R beta 1,3-galactosyltransferase (LOC731748), mRNA	XR_015594	Hs.587721	XR_015594

DCLRE1B	4.0	1.7	DNA cross-link repair 1B (PSO2 homolog, S. cerevisiae) (DCLRE1B), mRNA	NM_022836	Hs.591412	NM_022836
GARNL1	4.0	1.5	GTPase activating Rap/RanGAP domain-like 1 (GARNL1), transcript variant 1, mRNA	NM_014990	Hs.113150	AY596970
GPBAR1	4.0	1.6	G protein-coupled bile acid receptor 1 (GPBAR1), transcript variant 1, mRNA	NM_001077191	Hs.160954	NM_001077191
C10orf65	4.0	1.8	chromosome 10 open reading frame 65 (C10orf65), mRNA	NM_138413	Hs.180346	AK094791
ALPK1	4.0	4.9	alpha-kinase 1 (ALPK1), mRNA	NM_025144	Unknown	
AK124953	4.0	1.6	cDNA FLJ42963 fis, clone BRSTN2012380.	AK124953	Hs.404449	AK124953
THC2651723	4.0	1.4	HUMUBCP pro-ubiquitin {Homo sapiens} (exp=-1; wgp=0; cg=0), partial (39%)	THC2651723	Unknown	
AK124192	4.0	1.6	cDNA FLJ42198 fis, clone THYMU2034338.	AK124192	Hs.584833	AK124192
PRKACB	4.0	2.1	protein kinase, cAMP-dependent, catalytic, beta (PRKACB), transcript variant 2, mRNA	NM_002731	Hs.487325	BX537705
PPAPDC2	4.0	1.4	phosphatidic acid phosphatase type 2 domain containing 2 (PPAPDC2), mRNA	NM_203453	Hs.107510	BC038108
ALDOB	4.0	27.3	aldolase B, fructose-bisphosphate (ALDOB), mRNA	NM_000035	Hs.530274	NM_000035
BM968705	4.0	1.5	BM968705 UI-CF-DU1-aak-f-20-0-UI.s1 UI-CF-DU1 cDNA clone UI-CF-DU1-aak-f-20-0-UI 3', mRNA sequence	BM968705	Hs.200804	AK128645
PRKAR1A	4.0	2.6	protein kinase, cAMP-dependent, regulatory, type I, alpha (tissue specific extinguisher 1) (PRKAR1A), transcript variant 3, mRNA	NM_212472	Hs.280342	CR749311
MLN	4.0	2.0	motilin (MLN), transcript variant 1, mRNA	NM_002418	Hs.2813	NM_002418
YIPF4	4.0	1.8	Yip1 domain family, member 4 (YIPF4), mRNA	NM_032312	Hs.468099	AK098486
C10orf79	4.0	4.4	mRNA; cDNA DKFZp434P078 (from clone DKFZp434P078).	AL136901	Hs.288927	NM_025145
TANC2	4.0	2.2	mRNA for KIAA1636 protein, partial cds.	AB046856	Hs.410889	XM_371074
C11orf65	4.0	1.8	chromosome 11 open reading frame 65 (C11orf65), mRNA	NM_152587	Hs.653180	NM_152587
C20orf56	4.0	3.2	chromosome 20 open reading frame 56 (C20orf56) on chromosome 20	NR_001558	Unknown	
KATNAL1	4.0	2.2	katanin p60 subunit A-like 1 (KATNAL1), transcript variant 1, mRNA	NM_032116	Hs.243596	NM_032116
C4orf27	4.0	1.6	chromosome 4 open reading frame 27 (C4orf27), mRNA	NM_017867	Hs.406756	AK124036
THOC2	4.0	2.0	THO complex 2, mRNA (cDNA clone IMAGE:6043316), complete cds.	BC072400	Hs.592243	NM_001081550
RPL23A	4.0	1.5	ribosomal protein L23a (RPL23A), mRNA	NM_000984	Hs.419463	CR616046
PTGS2	4.0	6.5	prostaglandin-endoperoxide synthase 2 (prostaglandin G/H synthase and cyclooxygenase) (PTGS2), mRNA	NM_000963	Hs.196384	NM_000963
HSPB7	4.0	6.8	heat shock 27kDa protein family, member 7 (cardiovascular) (HSPB7), mRNA	NM_014424	Hs.502612	AL832181
THC2695576	4.0	3.3	BF735554 CM1-AN0080-071100-534-d05 AN0080 cDNA, mRNA sequence	THC2695576	Unknown	
DYNC2LI1	4.0	2.2	dynein, cytoplasmic 2, light intermediate chain 1 (DYNC2LI1), transcript variant 2, mRNA	NM_015522	Hs.371597	BC040558
CPS1	4.0	2.1	carbamoyl-phosphate synthetase 1, mitochondrial (CPS1), mRNA	NM_001875	Hs.149252	NM_001875
BE835321	4.0	2.0	BE835321 RC5-FN0022-300600-022-G12 FN0022 cDNA, mRNA sequence	BE835321	Hs.676515	BQ448273
PCDH18	4.0	1.8	protocadherin 18 (PCDH18), mRNA	NM_019035	Hs.591691	NM_019035
GC	4.0	19.9	group-specific component (vitamin D binding protein) (GC), mRNA	NM_000583	Hs.418497	AK223458
AK022059	4.0	2.0	cDNA FLJ11997 fis, clone HEMBB1001458.	AK022059	Hs.432755	BC060791
THC2698150	4.0	2.7	Q9N083_MACFA (Q9N083) Unnamed portein product, partial (23%)	THC2698150	Unknown	
PPP3CC	4.0	2.3	protein phosphatase 3 (formerly 2B), catalytic subunit, gamma isoform (calcineurin A gamma) (PPP3CC), mRNA	NM_005605	Hs.655661	NM_005605
LOC653464	4.0	2.1	PREDICTED: similar to SLIT-ROBO Rho GTPase-activating protein 2 (srGAP2) (Formin-binding protein 2) (LOC653464), mRNA	XM_209227	Hs.523529	XM_209227
USP1	4.0	1.9	ubiquitin specific peptidase 1 (USP1), transcript variant 1, mRNA	NM_003368	Hs.35086	NM_003368
RWDD3	4.0	2.1	RWD domain containing 3 (RWDD3), mRNA	NM_015485	Hs.483512	BX537731
ST8SIA4	4.0	4.3	ST8 alpha-N-acetyl-neuraminide alpha-2,8-sialyltransferase 4 (ST8SIA4), transcript variant 1, mRNA	NM_005668	Hs.308628	NM_005668
SAP30	4.0	1.6	Sin3A-associated protein, 30kDa (SAP30), mRNA	NM_003864	Hs.591715	BC016757
TXNDC13	4.0	1.7	thioredoxin domain containing 13 (TXNDC13), mRNA	NM_021156	Hs.169358	BC044777
CCDC132	4.0	2.1	coiled-coil domain containing 132 (CCDC132), transcript variant 1, mRNA	NM_017667	Hs.222282	NM_017667
CPS1	4.0	2.2	carbamoyl-phosphate synthetase 1, mitochondrial (CPS1), mRNA	NM_001875	Hs.149252	NM_001875
SDK2	4.0	3.3	sidekick homolog 2 (chicken), mRNA (cDNA clone IMAGE:6172764), partial cds.	BC066363	Hs.435719	BC066363
OLFM3	4.0	10.6	olfactomedin 3 (OLFM3), mRNA	NM_058170	Hs.484475	NM_058170
BU633484	4.0	14.4	BU633484 UI-H-FL1-bgu-j-06-0-UI.s1 NCI_CGAP_FL1 cDNA clone UI-H-FL1-bgu-j-06-0-UI 3', mRNA sequence	BU633484	Hs.591085	NM_004529
RPL21	4.0	1.5	ribosomal protein L21 (RPL21), mRNA	NM_000982	Hs.381123	CR602527
FAM78A	4.0	2.9	family with sequence similarity 78, member A (FAM78A), mRNA	NM_033387	Hs.143878	AK024434
LOC649839	4.0	1.7	PREDICTED: similar to large subunit ribosomal protein L36a (LOC649839), mRNA	ENST00000330370	Unknown	
NKTR	4.0	1.6	natural killer-tumor recognition sequence (NKTR), transcript variant 2, mRNA	NM_001012651	Unknown	
HIST1H2BM	4.0	3.0	histone cluster 1, H2bm (HIST1H2BM), mRNA	NM_003521	Hs.182432	BM752802
VPS35	4.0	1.6	vacuolar protein sorting 35 homolog (S. cerevisiae) (VPS35), mRNA	NM_018206	Hs.696029	AK025774

GPR137C	4.0	2.3	cDNA FLJ41959 fis, clone PUAEN2002489, moderately similar to putative seven pass transmembrane protein (TM7SF1) mRNA.	ENST00000321662	Unknown	
THC2645667	4.0	2.5	ALU6_HUMAN (P39193) Alu subfamily SP sequence contamination warning entry, partial (23%)	THC2645667	Unknown	
BNIP3	4.0	2.4	BCL2/adenovirus E1B 19kDa interacting protein 3 (BNIP3), nuclear gene encoding mitochondrial protein, mRNA	NM_004052	Hs.144873	BX647339
ZC3H11A	4.0	2.9	zinc finger CCCH-type containing 11A (ZC3H11A), mRNA	NM_014827	Hs.532399	CR627439
IDH2	4.0	1.9	isocitrate dehydrogenase 2 (NADP+), mitochondrial (IDH2), mRNA	NM_002168	Hs.596461	X69433
RAB23	4.0	1.5	RAB23, member RAS oncogene family (RAB23), transcript variant 1, mRNA	NM_016277	Hs.555016	NM_016277
BQ932957	4.0	2.9	AGENCOURT_8821387 Lupski_sciatic_nerve cDNA clone IMAGE:6203487 5', mRNA sequence	BQ932957	Hs.507978	BQ932957
A_32_P122951	4.0	2.6	A_32_P122951	A_32_P122951	Unknown	
ASNSD1	4.0	1.5	asparagine synthetase domain containing 1 (ASNSD1), mRNA	NM_019048	Hs.101364	AK000759
LOC157562	4.0	1.9	mRNA; cDNA DKFZp566J123 (from clone DKFZp566J123).	AL050061	Hs.27371	BX649145
BM678897	4.0	4.9	BM678897 UI-E-EJ0-ahv-n-06-0-UI.s1 UI-E-EJ0 cDNA clone UI-E-EJ0-ahv-n-06-0-UI 3', mRNA sequence	BM678897	Hs.194766	AB023144
COL1A1	4.0	2.2	H.sapiens mRNA for prepro-alpha1(I) collagen.	Z74615	Hs.172928	Z74615
CD99L2	4.0	5.2	CD99 molecule-like 2 (CD99L2), transcript variant 1, mRNA	NM_031462	Hs.522805	BC025729
THC2733814	4.0	4.2	THC2733814	THC2733814	Unknown	
ENST00000329784	4.0	1.5	PREDICTED: similar to large subunit ribosomal protein L36a (LOC284230), mRNA	ENST00000329784	Unknown	
AK124263	4.0	1.6	cDNA FLJ42269 fis, clone TKIDN2015285.	AK124263	Hs.649522	AK127313
C9orf5	4.0	1.6	chromosome 9 open reading frame 5 (C9orf5), mRNA	NM_032012	Hs.308074	AF153415
SMC5	4.0	1.8	SMC5 protein	ENST00000377144	Unknown	
WDR66	4.0	5.8	WD repeat domain 66 (WDR66), mRNA	NM_144668	Hs.131151	BC028421
ITM2B	4.0	1.7	integral membrane protein 2B (ITM2B), mRNA	NM_021999	Hs.699207	BX537657
C6orf32	4.0	3.3	mRNA for KIAA0386 gene, partial cds.	AB002384	Hs.559459	AB002384
KLHDC1	4.0	3.1	kelch domain containing 1 (KLHDC1), mRNA	NM_172193	Hs.509258	AF111806
AB040974	4.0	1.6	mRNA for KIAA1541 protein, partial cds.	AB040974	Unknown	
IPO9	4.0	2.1	importin 9 (IPO9), mRNA	NM_018085	Hs.596014	NM_018085
KLHDC2	4.0	1.7	kelch domain containing 2 (KLHDC2), mRNA	NM_014315	Hs.509264	AK056298
ST3GAL6	4.0	2.6	ST3 beta-galactoside alpha-2,3-sialyltransferase 6 (ST3GAL6), mRNA	NM_006100	Hs.148716	CR749468
BBS5	4.0	1.9	Bardet-Biedl syndrome 5 (BBS5), mRNA	NM_152384	Hs.233398	NM_152384
C14orf100	4.0	1.7	chromosome 14 open reading frame 100 (C14orf100), mRNA	NM_016475	Hs.446850	AK128628
ITCH	4.0	1.6	itchy homolog E3 ubiquitin protein ligase (mouse) (ITCH), mRNA	NM_031483	Hs.632272	NM_031483
UNQ1887	4.0	2.2	cDNA FLJ39830 fis, clone SPLEN2012846.	AK097149	Unknown	
BQ189494	4.0	5.1	BQ189494 UI-E-EJ1-aka-g-05-0-UI.r1 UI-E-EJ1 cDNA clone UI-E-EJ1-aka-g-05-0-UI 5', mRNA sequence	BQ189494	Hs.664796	BQ189494
PTGS2	4.0	5.5	prostaglandin-endoperoxide synthase 2 (prostaglandin G/H synthase and cyclooxygenase) (PTGS2), mRNA	NM_000963	Hs.196384	NM_000963
AK2	4.0	2.0	adenylate kinase 2 (AK2), transcript variant AK2A, mRNA	NM_001625	Hs.470907	NM_013411
FAHD1	4.0	1.7	fumarylacetoacetate hydrolase domain containing 1 (FAHD1), transcript variant 2, mRNA	NM_031208	Hs.513265	AL136720
KCNIP4	4.0	6.9	Kv channel interacting protein 4 (KCNIP4), transcript variant 5, mRNA	NM_001035003	Hs.655705	AK125392
CEP68	4.0	2.4	mRNA for KIAA0582 protein, partial cds.	AB011154	Hs.699217	NM_015147
C20orf102	4.0	3.0	chromosome 20 open reading frame 102 (C20orf102), mRNA	NM_080607	Hs.517029	NM_080607
IFI16	4.0	4.1	interferon, gamma-inducible protein 16 (IFI16), mRNA	NM_005531	Hs.380250	NM_005531
RP11-54H7.1	4.0	2.8	myosin heavy chain Myr 8 (MYR8), mRNA	NM_015011	Hs.656587	BC146791
SULT1C2	4.0	2.7	sulfotransferase family, cytosolic, 1C, member 2 (SULT1C2), mRNA	NM_006588	Hs.312644	BC058861
THC2514262	4.0	4.5	Q6DQW2_MANSE (Q6DQW2) CAPA, partial (7%)	THC2514262	Unknown	
PDE4DIP	4.0	3.4	phosphodiesterase 4D interacting protein (myomegalin) (PDE4DIP), transcript variant 5, mRNA	NM_001002811	Hs.654651	NM_014644
GIMAP8	4.0	6.1	GTPase, IMAP family member 8 (GIMAP8), mRNA	NM_175571	Hs.647121	NM_175571
CAST	4.0	2.1	calpastatin (CAST), transcript variant 1, mRNA	NM_001750	Hs.440961	NM_001750
ORMDL1	4.0	1.5	ORM1-like 1 (S. cerevisiae) (ORMDL1), mRNA	NM_016467	Hs.700632	AK126336
AK074467	4.0	2.6	cDNA FLJ23887 fis, clone LNG14332.	AK074467	Hs.435500	AK074467
KIAA0372	4.0	1.4	Tetratricopeptide repeat protein KIAA0372 (TPR repeat protein KIAA0372).	ENST00000380021	Unknown	
LOC731599	4.0	1.5	PREDICTED: hypothetical protein LOC731599 (LOC731599), mRNA	XR_015536	Hs.651110	XR_015536
C20orf12	4.0	2.4	chromosome 20 open reading frame 12 (C20orf12), mRNA	NM_018152	Unknown	
IFT74	4.0	1.8	intraflagellar transport 74 homolog (Chlamydomonas) (IFT74), mRNA	NM_025103	Hs.145402	NM_001099222
KIAA1571	4.0	1.7	mRNA for KIAA1571 protein, partial cds.	AB046791	Hs.110489	XM_371590

UPF3A	4.0	1.6	UPF3 regulator of nonsense transcripts homolog A (yeast) (UPF3A), transcript variant 1, mRNA	NM_023011	Hs.533855	AK092586
MAPK8	4.0	1.7	Mitogen-activated protein kinase 8 (EC 2.7.11.24) (Stress-activated protein kinase JNK1) (c-Jun N-terminal kinase 1) (JNK-46).	ENST00000374189	Unknown	
THC2656875	4.0	3.2	THC2656875	THC2656875	Unknown	
MAPK8	4.0	1.7	Mitogen-activated protein kinase 8 (EC 2.7.11.24) (Stress-activated protein kinase JNK1) (c-Jun N-terminal kinase 1) (JNK-46).	ENST00000374189	Unknown	
MTHFR	4.0	2.3	5,10-methylenetetrahydrofolate reductase (NADPH) (MTHFR), mRNA	NM_005957	Hs.214142	NM_005957
ANKRD12	4.0	1.6	ankyrin repeat domain 12 (ANKRD12), mRNA	NM_015208	Hs.464585	NM_015208
A_32_P225328	4.0	20.9	A_32_P225328	A_32_P225328	Unknown	
FAM39DP	4.0	1.4	family with sequence similarity 39, member D pseudogene (FAM39DP), mRNA	NM_199163	Unknown	
PTGS2	4.0	6.5	prostaglandin-endoperoxide synthase 2 (prostaglandin G/H synthase and cyclooxygenase) (PTGS2), mRNA	NM_000963	Hs.196384	NM_000963
ZNF22	4.0	1.8	zinc finger protein 22 (KOX 15) (ZNF22), mRNA	NM_006963	Hs.462693	NM_006963
SEC22B	3.9	2.0	SEC22 vesicle trafficking protein homolog B (S. cerevisiae) (SEC22B), mRNA	NM_004892	Hs.696469	NM_004892
ENST00000309447	3.9	22.2	mRNA for KIAA1239 protein, partial cds.	ENST00000309447	Unknown	
SLC12A2	3.9	2.4	solute carrier family 12 (sodium/potassium/chloride transporters), member 2 (SLC12A2), mRNA	NM_001046	Hs.162585	NM_001046
AK124778	3.9	2.6	cDNA FLJ42788 fis, clone BRAWH3007129.	AK124778	Hs.254117	AK124778
A_24_P850187	3.9	1.4	A_24_P850187	A_24_P850187	Unknown	
KIAA2018	3.9	1.4	KIAA2018 (KIAA2018), mRNA	NM_001009899	Hs.632570	NM_001009899
PDCL	3.9	1.6	phosducin-like (PDCL), mRNA	NM_005388	Hs.271749	NM_005388
C10orf10	3.9	5.5	chromosome 10 open reading frame 10 (C10orf10), mRNA	NM_007021	Hs.93675	NM_007021
ARL6IP6	3.9	1.6	ADP-ribosylation-like factor 6 interacting protein 6 (ARL6IP6), mRNA	NM_152522	Hs.516468	AK023109
TAC1	3.9	3.9	tachykinin, precursor 1 (substance K, substance P, neurokinin 1, neurokinin 2, neuromedin L, neurokinin alpha, neuropeptide K, neuropeptide gamma) (TAC1), transcript variant beta, mRNA	NM_003182	Hs.2563	NM_003182
IL17B	3.9	3.4	interleukin 17B (IL17B), mRNA	NM_014443	Hs.156979	NM_014443
PDGFD	3.9	5.5	platelet derived growth factor D (PDGFD), transcript variant 1, mRNA	NM_025208	Hs.352298	BC030645
BC041979	3.9	8.2	cDNA clone IMAGE:5303125.	BC041979	Hs.254117	AK124778
SLC8A3	3.9	26.3	solute carrier family 8 (sodium-calcium exchanger), member 3 (SLC8A3), transcript variant c, mRNA	NM_183002	Hs.337696	NM_183002
IQCH	3.9	3.9	IQ motif containing H (IQCH), transcript variant 2, mRNA	NM_022784	Hs.657894	AK022538
A_24_P902195	3.9	3.0	A_24_P902195	A_24_P902195	Unknown	
SASP	3.9	2.3	skin aspartic protease (SASP), mRNA	NM_152792	Hs.556025	AK055994
C3orf15	3.9	2.7	chromosome 3 open reading frame 15 (C3orf15), mRNA	NM_033364	Hs.341906	NM_033364
THC2615857	3.9	2.6	THC2615857	THC2615857	Unknown	
TMEM31	3.9	2.0	transmembrane protein 31 (TMEM31), mRNA	NM_182541	Hs.98843	BQ429340
MAPK8	3.9	1.7	Mitogen-activated protein kinase 8 (EC 2.7.11.24) (Stress-activated protein kinase JNK1) (c-Jun N-terminal kinase 1) (JNK-46).	ENST00000374189	Unknown	
RPS25	3.9	1.3	ribosomal protein S25 (RPS25), mRNA	NM_001028	Hs.512676	BM554735
AGGF1	3.9	1.6	angiogenic factor with G patch and FHA domains 1 (AGGF1), mRNA	NM_018046	Hs.634849	NM_018046
CCDC65	3.9	4.5	coiled-coil domain containing 65 (CCDC65), mRNA	NM_033124	Hs.512805	AF382188
AK091508	3.9	2.9	cDNA FLJ34189 fis, clone FCBBF3017535.	AK091508	Unknown	
FLJ10986	3.9	1.8	hypothetical protein FLJ10986 (FLJ10986), mRNA	NM_018291	Hs.444301	AK090568
PARP6	3.9	1.6	poly (ADP-ribose) polymerase family, member 6 (PARP6), mRNA	NM_020214	Hs.270244	AK091172
KIAA0372	3.9	1.5	Tetratricopeptide repeat protein KIAA0372 (TPR repeat protein KIAA0372).	ENST00000380021	Unknown	
WDR51B	3.9	1.5	WD repeat domain 51B (WDR51B), mRNA	NM_172240	Hs.25130	BX648293
DPY19L3	3.9	2.1	cDNA FLJ43858 fis, clone TESTI4007373.	AK125846	Hs.194392	AL833713
PUM1	3.9	2.0	pumilio homolog 1 (Drosophila) (PUM1), transcript variant 1, mRNA	NM_001020658	Hs.281707	NM_001020658
DLG1	3.9	1.4	mRNA; cDNA DKFZp761P0818 (from clone DKFZp761P0818).	AL831922	Hs.292549	NM_004087
NOTCH2NL	3.9	2.8	cDNA FLJ11946 fis, clone HEMBB1000709.	AK022008	Hs.655156	AK022008
A_32_P213948	3.9	2.4	A_32_P213948	A_32_P213948	Unknown	
TRAM1	3.9	1.5	translocation associated membrane protein 1 (TRAM1), mRNA	NM_014294	Hs.491988	BC032018
RP9	3.9	2.1	retinitis pigmentosa 9 (autosomal dominant) (RP9), mRNA	NM_203288	Hs.326805	NM_203288
TXNRD3	3.9	2.0	TXNRD3 protein (Fragment).	ENST00000360201	Unknown	
RNF32	3.9	4.6	ring finger protein 32 (RNF32), mRNA	NM_030936	Hs.490715	BC015416
C20orf26	3.9	3.2	chromosome 20 open reading frame 26 (C20orf26), mRNA	NM_015585	Hs.176013	NM_015585
THC2506656	3.9	2.1	Q7T2T6_BOTJR (Q7T2T6) Ribosomal protein, partial (70%)	THC2506656	Unknown	
GLI1	3.9	2.0	glioma-associated oncogene homolog 1 (zinc finger protein) (GLI1), mRNA	NM_005269	Hs.632702	NM_005269

MMP21	3.9	2.3	matrix metallopeptidase 21 (MMP21), mRNA	NM_147191	Hs.314141	NM_147191
TITF1	3.9	2.8	thyroid transcription factor 1 (TITF1), transcript variant 2, mRNA	NM_003317	Hs.700584	NM_003317
SCCPDH	3.9	2.5	saccharopine dehydrogenase (putative) (SCCPDH), mRNA	NM_016002	Hs.498397	NM_016002
RASSF5	3.9	12.9	Ras association (RalGDS/AF-6) domain family 5 (RASSF5), transcript variant 1, mRNA	NM_182663	Hs.497579	NM_182663
LOC644246	3.9	1.6	hypothetical protein LOC644246, mRNA (cDNA clone IMAGE:4730995), partial cds.	BC020847	Hs.644600	BG620930
TIPARP	3.9	3.2	TCDD-inducible poly(ADP-ribose) polymerase (TIPARP), mRNA	NM_015508	Hs.12813	CR749647
FAM125B	3.9	2.1	family with sequence similarity 125, member B (FAM125B), transcript variant 1, mRNA	NM_033446	Hs.162659	NM_033446
ANP32A	3.9	1.6	Acidic leucine-rich nuclear phosphoprotein 32 family member A (Potent heat-stable protein phosphatase 2A inhibitor I1PP2A) (Acidic nuclear phosphoprotein pp32) (Leucine-rich acidic nuclear protein) (Lanp) (Putative HLA-DR-associated protein I) (PHAPI)...	ENST00000267918	Unknown	
AFAP	3.9	2.2	actin filament associated protein (AFAP), transcript variant 1, mRNA	NM_021638	Hs.529369	NM_198595
SH3GL3	3.9	1.8	SH3-domain GRB2-like 3 (SH3GL3), mRNA	NM_003027	Hs.270055	NM_003027
GBE1	3.9	2.6	glucan (1,4-alpha-), branching enzyme 1 (glycogen branching enzyme, Andersen disease, glycogen storage disease type IV) (GBE1), mRNA	NM_000158	Hs.436062	AK125918
C14orf174	3.9	2.9	chromosome 14 open reading frame 174 (C14orf174), mRNA	NM_001010860	Hs.421961	NM_001010860
ENST00000360758	3.9	1.9	cancer susceptibility candidate 2, transcript variant 1 (CASC2), misc RNA	ENST00000360758	Unknown	
DNALI1	3.9	2.3	dynein, axonemal, light intermediate chain 1 (DNALI1), mRNA	NM_003462	Hs.406050	BC039074
DHX32	3.9	1.7	DEAH (Asp-Glu-Ala-His) box polypeptide 32 (DHX32), mRNA	NM_018180	Hs.501379	AF427340
DPY19L2	3.9	5.4	dpy-19-like 2 (C. elegans) (DPY19L2), mRNA	NM_173812	Hs.533644	NM_173812
IL6R	3.9	2.1	interleukin 6 receptor (IL6R), transcript variant 1, mRNA	NM_000565	Hs.695954	NM_000565
AW302758	3.9	3.4	AW302758 xr55g08.x1 NCI_CGAP_Ov26 cDNA clone IMAGE:2764094 3', mRNA sequence	AW302758	Hs.268488	BC031301
ABCB1	3.9	2.0	ATP-binding cassette, sub-family B (MDR/TAP), member 1 (ABCB1), mRNA	NM_000927	Hs.489033	NM_000927
CLEC4A	3.9	2.1	C-type lectin domain family 4, member A (CLEC4A), transcript variant 1, mRNA	NM_016184	Hs.504657	AF328684
ESRRG	3.9	15.3	estrogen-related receptor gamma (ESRRG), transcript variant 2, mRNA	NM_206594	Hs.444225	BC064700
PGBD1	3.9	1.9	piggyBac transposable element derived 1 (PGBD1), mRNA	NM_032507	Hs.144527	AK223446
BC014370	3.9	1.7	cDNA clone IMAGE:3961179, partial cds.	BC014370	Hs.525922	BC014370
C11orf67	3.9	2.0	chromosome 11 open reading frame 67 (C11orf67), mRNA	NM_024684	Hs.503357	BG121856
IL10RB	3.9	1.8	interleukin 10 receptor, beta (IL10RB), mRNA	NM_000628	Hs.654593	AK124057
HDAC6	3.9	1.5	histone deacetylase 6 (HDAC6), mRNA	NM_006044	Hs.6764	BC069243
C14orf32	3.9	1.7	chromosome 14 open reading frame 32 (C14orf32), mRNA	NM_144578	Hs.594338	NM_144578
SFRS2B	3.9	1.7	splicing factor, arginine/serine-rich 2B (SFRS2B), mRNA	NM_032102	Hs.648465	NM_032102
ENST00000326678	3.9	5.7	cDNA FLJ39251 fis, clone OCBBF2008701.	ENST00000326678	Unknown	
PFAAP5	3.9	1.8	phosphonoformate immuno-associated protein 5 (PFAAP5), mRNA	NM_014887	Hs.507680	AL049783
PCSK5	3.9	2.2	Proprotein convertase subtilisin/kexin type 5 precursor (EC 3.4.21.-) (Proprotein convertase PC5) (Subtilisin/kexin-like protease PC5) (PC6) (hPC6).	ENST00000376752	Unknown	
SYT11	3.9	4.2	synaptotagmin XI (SYT11), mRNA	NM_152280	Hs.32984	NM_152280
AK091028	3.9	2.4	cDNA FLJ33709 fis, clone BRAWH2007890.	AK091028	Hs.188825	CR933638
CAPN2	3.9	2.6	calpain 2, (m/II) large subunit (CAPN2), mRNA	NM_001748	Hs.350899	AK124751
ST7L	3.9	2.1	suppression of tumorigenicity 7 like (ST7L), transcript variant 4, mRNA	NM_138729	Hs.201921	NM_017744
MYO18B	3.9	3.5	myosin XVIIIB (MYO18B), mRNA	NM_032608	Hs.417959	NM_032608
A_32_P24431	3.9	2.8	A_32_P24431	A_32_P24431	Unknown	
A_24_P839258	3.9	4.1	A_24_P839258	A_24_P839258	Unknown	
KIAA1712	3.9	1.5	KIAA1712, mRNA (cDNA clone MGC:33587 IMAGE:4823994), complete cds.	BC038667	Unknown	
BQ018742	3.9	1.9	BQ018742 UI-H-DH1-awu-d-06-0-UI.s1 NCI_CGAP_DH1 cDNA clone IMAGE:5823701 3', mRNA sequence	BQ018742	Hs.696469	NM_004892
BC112296	3.9	3.8	cDNA clone IMAGE:8327764.	BC112296	Hs.529860	AK130729
RAI2	3.9	2.1	retinoic acid induced 2 (RAI2), mRNA	NM_021785	Hs.701485	NM_021785
TANC2	3.9	5.3	cDNA FLJ11824 fis, clone HEMBA1006492.	AK021886	Unknown	
AK096498	3.9	4.2	cDNA FLJ39179 fis, clone OCBBF2004147.	AK096498	Hs.437281	AK096498
COX7B	3.9	2.0	Cytochrome c oxidase polypeptide VIIb, mitochondrial precursor (EC 1.9.3.1).	ENST00000373335	Unknown	
THC2499508	3.9	1.6	THC2499508	THC2499508	Unknown	
AK091801	3.9	1.7	cDNA FLJ34482 fis, clone HLUNG2004067.	AK091801	Hs.594647	AK091801
DKFZP434I0714	3.9	1.9	hypothetical protein DKFZP434I0714, mRNA (cDNA clone IMAGE:4822572).	ENST00000304389	Unknown	
AK124299	3.9	1.6	cDNA FLJ42306 fis, clone TRACH2001646.	AK124299	Hs.130036	AK124299
MSX2P	3.9	3.0	msh homeobox 2 pseudogene (MSX2P) on chromosome 17	NR_002307	Unknown	
RPL37	3.9	1.4	ribosomal protein L37 (RPL37), mRNA	NM_000997	Hs.80545	AL137450

SVEP1	3.9	7.2	cDNA clone MGC:22782 IMAGE:4730700, complete cds.	BC030816	Hs.522334	NM_153366
CR625594	3.9	1.8	full-length cDNA clone CS0DF026YM07 of Fetal brain of (human).	CR625594	Hs.125867	AL133642
C3orf34	3.9	2.4	chromosome 3 open reading frame 34 (C3orf34), mRNA	NM_032898	Hs.334526	BC007827
ATRX	3.9	2.4	alpha thalassemia/mental retardation syndrome X-linked (RAD54 homolog, S. cerevisiae) (ATRX), transcript variant 3, mRNA	NM_138271	Unknown	
ZNRF3	3.9	1.7	mRNA for KIAA1133 protein, partial cds.	AB051436	Hs.655242	AB051436
TMEM109	3.9	1.8	transmembrane protein 109 (TMEM109), mRNA	NM_024092	Hs.13662	BC001309
BF373107	3.9	2.2	BF373107 CM2-FT0123-280700-305-C12 FT0123 cDNA, mRNA sequence	BF373107	Hs.633993	BX438032
USP42	3.9	1.5	ubiquitin specific peptidase 42 (USP42), mRNA	NM_032172	Hs.31856	NM_032172
AK057787	3.9	7.1	cDNA FLJ25058 fis, clone CBL04608.	AK057787	Hs.673595	AK057787
THC2770741	3.9	1.7	Q5AUD6_EMENI (Q5AUD6) Predicted protein, partial (6%)	THC2770741	Unknown	
AA593970	3.9	2.8	AA593970 nn01c05.s1 NCI_CGAP_Co9 cDNA clone IMAGE:1076456 3', mRNA sequence	AA593970	Hs.608251	CB854567
RAB3C	3.9	4.4	Ras-related protein Rab-3C.	ENST00000381158	Unknown	
KIAA0372	3.9	1.5	Tetratricopeptide repeat protein KIAA0372 (TPR repeat protein KIAA0372).	ENST00000380021	Unknown	
CKM	3.9	22.2	creatine kinase, muscle (CKM), mRNA	NM_001824	Hs.334347	NM_001824
VMO1	3.9	3.2	vitelline membrane outer layer 1 homolog (chicken) (VMO1), mRNA	NM_182566	Hs.122561	BI838614
BRP44	3.9	2.2	brain protein 44 (BRP44), mRNA	NM_015415	Hs.517768	CR607153
ANKRD25	3.9	1.9	ankyrin repeat domain 25 (ANKRD25), mRNA	NM_015493	Hs.284208	NM_015493
THC2486950	3.9	1.9	BX346171 BX346171 T CELLS (JURKAT CELL LINE) COT 10-NORMALIZED cDNA clone CS0DJ010YI10 3-PRIME, mRNA sequence	THC2486950	Unknown	
EML1	3.9	1.6	echinoderm microtubule associated protein like 1 (EML1), transcript variant 1, mRNA	NM_001008707	Hs.12451	NM_001008707
AK056855	3.9	1.8	cDNA FLJ32293 fis, clone PROST2001739.	AK056855	Hs.367885	AK056855
CR593784	3.9	2.8	full-length cDNA clone CS0DI036YE11 of Placenta Cot 25-normalized of (human).	CR593784	Hs.304253	CR593784
AK026517	3.9	6.2	cDNA: FLJ22864 fis, clone KAT02164.	AK026517	Hs.653859	NM_012153
SLC22A15	3.9	1.7	solute carrier family 22 (organic cation transporter), member 15 (SLC22A15), mRNA	NM_018420	Hs.125482	BC026358
IL1RAP	3.9	1.8	interleukin 1 receptor accessory protein (IL1RAP), transcript variant 1, mRNA	NM_002182	Hs.478673	NM_002182
EFNB2	3.9	2.6	ephrin-B2 (EFNB2), mRNA	NM_004093	Hs.149239	NM_004093
CCDC96	3.9	2.1	coiled-coil domain containing 96 (CCDC96), mRNA	NM_153376	Hs.656757	NM_153376
DYNC2LI1	3.9	2.0	dynein, cytoplasmic 2, light intermediate chain 1 (DYNC2LI1), transcript variant 2, mRNA	NM_015522	Hs.371597	BC040558
SBDS	3.9	1.9	Shwachman-Bodian-Diamond syndrome (SBDS), mRNA	NM_016038	Hs.110445	AY169963
DDAH2	3.9	2.0	dimethylarginine dimethylaminohydrolase 2 (DDAH2), mRNA	NM_013974	Hs.247362	BM460795
TCP11L2	3.9	2.1	t-complex 11 (mouse)-like 2 (TCP11L2), mRNA	NM_152772	Hs.696047	AF306858
COL6A3	3.9	3.8	collagen, type VI, alpha 3 (COL6A3), transcript variant 1, mRNA	NM_004369	Hs.233240	NM_004369
GNAI1	3.9	1.5	guanine nucleotide binding protein (G protein), alpha inhibiting activity polypeptide 1 (GNAI1), mRNA	NM_002069	Hs.134587	BC026326
ZSWIM5	3.9	2.3	mRNA for KIAA1511 protein, partial cds.	AB040944	Hs.656613	NM_020883
CTBS	3.9	1.7	chitobiase, di-N-acetyl-, mRNA (cDNA clone IMAGE:4823479), complete cds.	BC024007	Hs.513557	BC096752
C1orf53	3.9	6.1	chromosome 1 open reading frame 53 (C1orf53), mRNA	NM_001024594	Hs.61329	BI832220
A_24_P118382	3.9	1.4	A_24_P118382	A_24_P118382	Unknown	
TCEA3	3.9	8.8	transcription elongation factor A (SII), 3 (TCEA3), mRNA	NM_003196	Hs.446354	NM_003196
RBM8A	3.9	1.7	RNA binding motif protein 8A (RBM8A), mRNA	NM_005105	Hs.654719	AK075009
A_32_P209163	3.9	1.6	A_32_P209163	A_32_P209163	Unknown	
SPHAR	3.9	2.5	S-phase response (cyclin-related) (SPHAR), mRNA	NM_006542	Hs.296169	AY585832
ORC4L	3.9	1.6	origin recognition complex, subunit 4-like (yeast) (ORC4L), transcript variant 2, mRNA	NM_002552	Hs.558364	NM_002552
GOLGA3	3.9	1.8	golgi autoantigen, golgin subfamily a, 3 (GOLGA3), mRNA	NM_005895	Hs.507333	NM_005895
YME1L1	3.9	1.4	YME1-like 1 (S. cerevisiae) (YME1L1), nuclear gene encoding mitochondrial protein, transcript variant 1, mRNA	NM_139312	Hs.499145	NM_139312
FCHSD2	3.9	1.9	FCH and double SH3 domains 2 (FCHSD2), mRNA	NM_014824	Hs.577053	AB018312
C13orf27	3.9	1.7	chromosome 13 open reading frame 27 (C13orf27), mRNA	NM_138779	Hs.398111	BC048011
SMO	3.9	1.5	smoothened homolog (Drosophila) (SMO), mRNA	NM_005631	Hs.437846	NM_005631
AK023647	3.9	2.2	cDNA FLJ13585 fis, clone PLACE1009150.	AK023647	Hs.43047	AK023647
MAN2A2	3.9	1.7	mannosidase, alpha, class 2A, member 2 (MAN2A2), mRNA	NM_006122	Hs.116459	NM_006122
CCPG1	3.9	2.0	cell cycle progression 1 (CCPG1), transcript variant 2, mRNA	NM_020739	Hs.612814	NM_020739
LOC541467	3.9	3.5	hypothetical LOC541467, mRNA (cDNA clone IMAGE:5263822), partial cds.	BC036693	Hs.675581	BC045815
MMP3	3.9	3.5	matrix metallopeptidase 3 (stromelysin 1, progelatinase) (MMP3), mRNA	NM_002422	Hs.375129	AK223291

LOC554248	3.9	2.0	hypothetical LOC554248, mRNA (cDNA clone MGC:87732 IMAGE:5768316), complete cds.	BC078169	Hs.699798	BC078169
ADCK5	3.9	1.7	aarF domain containing kinase 5 (ADCK5), mRNA	NM_174922	Hs.283374	BC032402
SKP1A	3.8	1.7	S-phase kinase-associated protein 1A (p19A) (SKP1A), transcript variant 2, mRNA	NM_170679	Hs.171626	NM_006930
MTMR6	3.8	1.9	myotubularin related protein 6 (MTMR6), mRNA	NM_004685	Hs.696140	NM_004685
SYTL5	3.8	5.0	mRNA; cDNA DKFZp779C093 (from clone DKFZp779C093).	BX647688	Hs.662334	BX647688
CCPG1	3.8	1.5	cell cycle progression restoration 8 protein (CPR8) mRNA, complete cds.	AF011794	Hs.612814	NM_020739
PRAF2	3.8	1.6	PRA1 domain family, member 2 (PRAF2), mRNA	NM_007213	Hs.29595	BF982837
ZCCHC11	3.8	2.4	zinc finger, CCHC domain containing 11 (ZCCHC11), transcript variant 1, mRNA	NM_001009881	Hs.655407	NM_001009881
PTGS2	3.8	7.4	prostaglandin-endoperoxide synthase 2 (prostaglandin G/H synthase and cyclooxygenase) (PTGS2), mRNA	NM_000963	Hs.196384	NM_000963
MYL1	3.8	7.4	myosin, light chain 1, alkali; skeletal, fast (MYL1), transcript variant 1f, mRNA	NM_079420	Hs.187338	BF790783
PRR3	3.8	1.5	proline rich 3 (PRR3), transcript variant 1, mRNA	NM_025263	Hs.118354	NM_025263
XAGE3	3.8	2.1	X antigen family, member 3 (XAGE3), transcript variant 2, mRNA	NM_130776	Hs.43879	CF993800
CCDC93	3.8	1.7	coiled-coil domain containing 93 (CCDC93), mRNA	NM_019044	Hs.107845	NM_019044
LOC390413	3.8	1.5	PREDICTED: similar to 60S ribosomal protein L7 (LOC390413), mRNA	XR_018341	Hs.646625	XR_018341
C1orf145	3.8	4.9	chromosome 1 open reading frame 145, mRNA (cDNA clone IMAGE:5204063), partial cds.	BC027909	Hs.650039	BU959659
AK096225	3.8	3.2	cDNA FLJ38906 fis, clone NT2NE2004378.	AK096225	Hs.659458	AK096225
NMNAT1	3.8	3.7	nicotinamide nucleotide adenylyltransferase 1 (NMNAT1), mRNA	NM_022787	Hs.633762	NM_022787
A_24_P724106	3.8	1.5	A_24_P724106	A_24_P724106	Unknown	
RAB3IL1	3.8	2.0	RAB3A interacting protein (rabin3)-like 1 (RAB3IL1), mRNA	NM_013401	Hs.13759	NM_013401
THC2654987	3.8	2.1	THC2654987	THC2654987	Unknown	
A_24_P755505	3.8	1.5	A_24_P755505	A_24_P755505	Unknown	
AA303143	3.8	3.3	AA303143 EST13031 Uterus tumor I cDNA 5' end similar to hypothetical protein KIAA0222, mRNA sequence	AA303143	Hs.436973	NM_014643
AK001979	3.8	3.5	cDNA FLJ11117 fis, clone PLACE1005990.	AK001979	Hs.675473	AK001979
MRPL2	3.8	1.4	mitochondrial ribosomal protein L2 (MRPL2), nuclear gene encoding mitochondrial protein, mRNA	NM_015950	Hs.55041	NM_015950
ENST00000237763	3.8	1.4	Transcription factor BTF3 homolog 1 (Basic transcription factor 3-like 1).	ENST00000237763	Unknown	
SUSD4	3.8	4.9	sushi domain containing 4 (SUSD4), transcript variant 1, mRNA	NM_017982	Hs.497841	AK096265
SERPINC1	3.8	27.9	serpin peptidase inhibitor, clade C (antithrombin), member 1 (SERPINC1), mRNA	NM_000488	Hs.75599	AF130100
BC004962	3.8	1.7	Homo sapiens, clone IMAGE:3632683, mRNA.	BC004962	Hs.560092	AK055961
ZNF30	3.8	1.4	zinc finger protein 30 (ZNF30), mRNA	NM_194325	Hs.657402	BX640666
MAN1A2	3.8	2.3	mannosidase, alpha, class 1A, member 2 (MAN1A2), mRNA	NM_006699	Hs.435938	BC052954
DIO3OS	3.8	3.5	deiodinase, iodothyronine, type III opposite strand (DIO3OS) on chromosome 14	NR_002770	Unknown	
ASAH1	3.8	2.4	N-acylsphingosine amidohydrolase (acid ceramidase) 1 (ASAH1), transcript variant 1, mRNA	NM_177924	Hs.527412	NM_177924
MAPK8	3.8	1.7	Mitogen-activated protein kinase 8 (EC 2.7.11.24) (Stress-activated protein kinase JNK1) (c-Jun N-terminal kinase 1) (JNK-46).	ENST00000374189	Unknown	
MBL1P1	3.8	2.5	mannose-binding lectin (protein A) 1, pseudogene 1 (MBL1P1) on chromosome 10	NR_002724	Unknown	
ZNF702	3.8	3.6	cDNA FLJ12985 fis, clone NT2RP3000050, moderately similar to ZINC FINGER PROTEIN 91.	AK023047	Hs.270435	BC032590
DUSP9	3.8	2.4	dual specificity phosphatase 9 (DUSP9), mRNA	NM_001395	Hs.144879	BC060837
LOC643837	3.8	1.7	full-length cDNA clone CS0DN005YI02 of Adult brain of (human).	CR601056	Hs.133183	BC086872
RGPD5	3.8	1.5	RANBP2-like and GRIP domain containing 5 (RGPD5), transcript variant 1, mRNA	NM_005054	Hs.469630	NM_005054
CR616528	3.8	1.9	full-length cDNA clone CS0DC014YA20 of Neuroblastoma Cot 25-normalized of (human).	CR616528	Hs.654552	NM_006854
KLHL20	3.8	4.7	kelch-like 20 (Drosophila), mRNA (cDNA clone IMAGE:3951189), complete cds.	BC005253	Hs.495035	NM_014458
CAMSAP1L1	3.8	2.1	calmodulin regulated spectrin-associated protein 1-like 1 (CAMSAP1L1), mRNA	NM_203459	Hs.23585	NM_203459
KLHL24	3.8	2.0	kelch-like 24 (Drosophila) (KLHL24), mRNA	NM_017644	Hs.407709	NM_017644
TTLL11	3.8	2.6	tubulin tyrosine ligase-like family, member 11 (TTLL11), mRNA	NM_194252	Hs.656140	NM_194252
SLC7A7	3.8	2.8	solute carrier family 7 (cationic amino acid transporter, y+ system), member 7 (SLC7A7), mRNA	NM_003982	Hs.513147	AB209591
SENP1	3.8	1.7	SUMO1/sentrin specific peptidase 1 (SENP1), mRNA	NM_014554	Hs.371957	BX640784
MAP2K4	3.8	1.6	mitogen-activated protein kinase kinase 4 (MAP2K4), mRNA	NM_003010	Hs.514681	AK131544
CEP290	3.8	2.1	centrosomal protein 290kDa (CEP290), mRNA	NM_025114	Hs.150444	NM_025114
LOC440181	3.8	2.8	PREDICTED: hypothetical LOC440181 (LOC440181), mRNA	XR_018458	Hs.662146	XR_018458
NOVA1	3.8	2.2	neuro-oncological ventral antigen 1 (NOVA1), transcript variant 1, mRNA	NM_002515	Hs.31588	AK226114
ZZZ3	3.8	2.1	zinc finger, ZZ-type containing 3 (ZZZ3), mRNA	NM_015534	Hs.480506	AK074119

AARSD1	3.8	1.8	alanyl-tRNA synthetase domain containing 1 (AARSD1), mRNA	NM_025267	Hs.317403	CR619011
AK025758	3.8	6.6	cDNA: FLJ22105 fis, clone HEP17660.	AK025758	Hs.699441	AK226146
ATP5E	3.8	2.2	ATP synthase, H+ transporting, mitochondrial F1 complex, epsilon subunit (ATP5E), nuclear gene encoding mitochondrial protein, mRNA	NM_006886	Hs.177530	CR595852
SKP1A	3.8	1.5	S-phase kinase-associated protein 1A (p19A) (SKP1A), transcript variant 1, mRNA	NM_006930	Hs.171626	NM_006930
CDC42EP4	3.8	1.7	CDC42 effector protein (Rho GTPase binding) 4 (CDC42EP4), mRNA	NM_012121	Hs.176479	BC002774
MTHFD2L	3.8	4.8	methylenetetrahydrofolate dehydrogenase (NADP+ dependent) 2-like (MTHFD2L), mRNA	NM_001004346	Hs.479954	AK122799
THC2699924	3.8	1.9	THC2699924	THC2699924	Unknown	
KIAA1005	3.8	1.7	KIAA1005 protein (KIAA1005), mRNA	NM_015272	Hs.298382	NM_015272
FUSIP1	3.8	1.6	FUS interacting protein (serine/arginine-rich) 1 (FUSIP1), transcript variant 2, mRNA	NM_054016	Hs.3530	AK125834
A_32_P179646	3.8	1.8	A_32_P179646	A_32_P179646	Unknown	
AMY1C	3.8	12.0	amylase, alpha 1C; salivary (AMY1C), mRNA	NM_001008219	Hs.655232	NM_004038
THC2653521	3.8	2.1	Q4V9S2_HUMAN (Q4V9S2) CXorf20 protein, partial (5%)	THC2653521	Unknown	
PHKB	3.8	1.5	phosphorylase kinase, beta (PHKB), transcript variant 2, mRNA	NM_001031835	Hs.78060	CR936710
FLJ40852	3.8	3.8	hypothetical protein FLJ40852 (FLJ40852), mRNA	NM_173677	Unknown	
LOC149351	3.8	10.9	hypothetical protein LOC149351, mRNA (cDNA clone IMAGE:5273076).	BC036441	Hs.546492	AK022898
OAF	3.8	2.6	OAF homolog (Drosophila) (OAF), mRNA	NM_178507	Hs.445081	BC047726
VPS13C	3.8	1.9	vacuolar protein sorting 13 homolog C (S. cerevisiae) (VPS13C), transcript variant 2A, mRNA	NM_020821	Hs.511668	AJ608771
THC2724764	3.8	2.4	THC2724764	THC2724764	Unknown	
GATA4	3.8	6.6	GATA binding protein 4 (GATA4), mRNA	NM_002052	Hs.243987	NM_002052
HOXC5	3.8	2.2	homeobox C5 (HOXC5), transcript variant 1, mRNA	NM_018953	Hs.549040	NM_014620
PPP1R2	3.8	1.4	protein phosphatase 1, regulatory (inhibitor) subunit 2 (PPP1R2), mRNA	NM_006241	Hs.535731	NM_006241
A_24_P928830	3.8	2.9	A_24_P928830	A_24_P928830	Unknown	
GGTA1	3.8	2.1	glycoprotein, alpha-galactosyltransferase 1 (GGTA1) on chromosome 9	NR_003191	Unknown	
ENST00000324982	3.8	2.2	cDNA FLJ43379 fis, clone OCBBF2002626.	ENST00000324982	Unknown	
CNOT2	3.8	1.4	CCR4-NOT transcription complex, subunit 2 (CNOT2), mRNA	NM_014515	Hs.133350	BX641116
KBTBD4	3.8	1.4	kelch repeat and BTB (POZ) domain containing 4 (KBTBD4), transcript variant 2, mRNA	NM_016506	Hs.656205	AK091607
ZBTB5	3.8	1.7	zinc finger and BTB domain containing 5 (ZBTB5), mRNA	NM_014872	Hs.161276	NM_014872
ENST00000315707	3.8	5.8	CDNA FLJ34790 fis, clone NT2NE2005323 (C17orf44 protein) (Hypothetical protein C17orf44).	ENST00000315707	Unknown	
THC2683448	3.8	2.4	Q7WZG3_PASPI (Q7WZG3) Ferric uptake regulator, partial (8%)	THC2683448	Unknown	
HSD3B1	3.8	2.3	hydroxy-delta-5-steroid dehydrogenase, 3 beta- and steroid delta-isomerase 1 (HSD3B1), mRNA	NM_000862	Hs.364941	CD014103
LRRC1	3.8	1.9	leucine rich repeat containing 1 (LRRC1), mRNA	NM_018214	Hs.700747	NM_018214
SLITRK4	3.8	6.1	SLIT and NTRK-like family, member 4 (SLITRK4), mRNA	NM_173078	Hs.272284	BC040986
ZNF23	3.8	1.5	zinc finger protein 23 (KOX 16) (ZNF23), mRNA	NM_145911	Hs.656643	BX537507
SELENBP1	3.8	2.1	selenium binding protein 1 (SELENBP1), mRNA	NM_003944	Hs.632460	BC032997
AK057476	3.8	2.1	cDNA FLJ32914 fis, clone TESTI2006409.	AK057476	Hs.651384	BC053597
FLJ40113	3.8	1.7	golgi autoantigen, golgin subfamily a-like pseudogene (FLJ40113) on chromosome 15	NR_003246	Unknown	
HSPC268	3.8	1.3	hypothetical protein HSPC268 (HSPC268), mRNA	NM_197964	Hs.370475	BC041001
KIAA2002	3.8	1.5	mRNA for KIAA2002 protein.	AB082533	Hs.9587	NM_024776
ZNF266	3.8	2.4	zinc finger protein 266 (ZNF266), mRNA	NM_006631	Hs.656185	AB095928
PDLIM5	3.8	2.0	PDZ and LIM domain 5 (PDLIM5), transcript variant 1, mRNA	NM_006457	Hs.480311	NM_006457
CRB2	3.8	3.4	crumbs homolog 2 (Drosophila) (CRB2), mRNA	NM_173689	Hs.568340	NM_173689
ENST00000295907	3.8	2.1	full-length cDNA clone CS0DF031YF04 of Fetal brain of (human).	ENST00000295907	Unknown	
PIGC	3.8	2.3	phosphatidylinositol glycan anchor biosynthesis, class C (PIGC), transcript variant 2, mRNA	NM_002642	Hs.188456	NM_002642
ENST00000221282	3.8	1.9	Zinc finger protein 302 (ZNF135-like) (ZNF140-like).	ENST00000221282	Unknown	
MPDZ	3.8	1.8	mRNA for MPDZ variant protein, partial cds, clone: pf00482.	AB210041	Hs.169378	AB210041
ST7L	3.8	1.6	suppression of tumorigenicity 7 like (ST7L), transcript variant 1, mRNA	NM_017744	Hs.201921	NM_017744
ADRA1B	3.8	5.5	adrenergic, alpha-1B-, receptor (ADRA1B), mRNA	NM_000679	Hs.368632	NM_000679
F5	3.8	3.6	coagulation factor V (proaccelerin, labile factor) (F5), mRNA	NM_000130	Hs.30054	NM_000130
MAP3K8	3.8	1.8	mitogen-activated protein kinase kinase kinase 8 (MAP3K8), mRNA	NM_005204	Hs.432453	AB209539
AK094415	3.8	2.1	cDNA FLJ37096 fis, clone BRACE2018759.	AK094415	Hs.343846	BC014254
REV1	3.8	1.5	REV1 homolog (S. cerevisiae) (REV1), transcript variant 2, mRNA	NM_001037872	Hs.443077	NM_016316

YPEL2	3.8	2.5	yippee-like 2 (Drosophila) (YPEL2), mRNA	NM_001005404	Hs.463613	NM_001005404
MAPK8	3.8	1.7	Mitogen-activated protein kinase 8 (EC 2.7.11.24) (Stress-activated protein kinase JNK1) (c-Jun N-terminal kinase 1) (JNK-46).	ENST00000374189	Unknown	
RAVER2	3.8	2.2	ribonucleoprotein, PTB-binding 2 (RAVER2), mRNA	NM_018211	Hs.591443	NM_018211
C1orf63	3.8	3.3	cDNA FLJ14412 fis, clone HEMBA1004669, weakly similar to SON PROTEIN.	AK027318	Hs.259412	AK096351
FER1L4	3.8	2.3	fer-1-like 4 (C. elegans) (FER1L4) on chromosome 20	NR_001442	Unknown	
THC2726661	3.8	2.0	T52140 yb29h11.r1 Stratagene fetal spleen (#937205) cDNA clone IMAGE:72645 5', mRNA sequence	THC2726661	Unknown	
BG196990	3.8	1.4	RST16222 Athersys RAGE Library cDNA, mRNA sequence	BG196990	Hs.454011	ES309195
RUNX2	3.8	4.3	runt-related transcription factor 2 (RUNX2), transcript variant 3, mRNA	NM_004348	Hs.535845	NM_004348
HSPA5	3.8	2.2	heat shock 70kDa protein 5 (glucose-regulated protein, 78kDa) (HSPA5), mRNA	NM_005347	Hs.605502	NM_005347
MGC42105	3.8	3.9	hypothetical protein MGC42105 (MGC42105), mRNA	NM_153361	Hs.25845	AK122985
SMAD1	3.8	1.5	SMAD family member 1 (SMAD1), transcript variant 1, mRNA	NM_005900	Hs.656534	NM_005900
HIST1H2BI	3.8	2.9	histone cluster 1, H2bi (HIST1H2BI), mRNA	NM_003525	Hs.553506	DA509995
SEZ6L	3.8	4.0	mRNA for KIAA0927 protein, partial cds.	AB023144	Hs.194766	AB023144
C14orf105	3.8	4.1	chromosome 14 open reading frame 105 (C14orf105), mRNA	NM_018168	Hs.659706	AK001512
GALNT4	3.8	4.1	UDP-N-acetyl-alpha-D-galactosamine:polypeptide N-acetylgalactosaminyltransferase 4 (GalNAc-T4) (GALNT4), mRNA	NM_003774	Hs.701301	BC036390
ENST00000367391	3.8	11.3	LIM/homeobox protein Lhx9.	ENST00000367391	Unknown	
FLRT3	3.8	3.2	fibronectin leucine rich transmembrane protein 3 (FLRT3), transcript variant 2, mRNA	NM_198391	Hs.41296	AB040902
F5	3.8	3.0	coagulation factor V (proaccelerin, labile factor) (F5), mRNA	NM_000130	Hs.30054	NM_000130
AF035290	3.8	10.4	clone 23556 mRNA sequence.	AF035290	Hs.656663	AF035290
AHNAK	3.8	3.2	AHNAK nucleoprotein (desmoyokin) (AHNAK), transcript variant 1, mRNA	NM_001620	Hs.502756	NM_001620
RBM11	3.8	1.8	RNA binding motif protein 11 (RBM11), mRNA	NM_144770	Hs.283828	BC030196
UTS2D	3.8	5.6	cDNA FLJ33311 fis, clone BNGH42005235.	AK090630	Hs.518492	AK090630
DPY19L1	3.8	2.1	DPY-19-like protein 1 (DPY19L1) mRNA, complete cds.	DQ287932	Hs.408623	NM_015283
SETDB1	3.8	2.0	SET domain, bifurcated 1 (SETDB1), mRNA	NM_012432	Hs.700587	NM_012432
AF119889	3.8	1.6	PRO2667 mRNA, complete cds.	AF119889	Unknown	
DNAH7	3.8	2.6	dynein, axonemal, heavy chain 7 (DNAH7), mRNA	NM_018897	Hs.97403	AB023161
MLCK	3.8	3.2	MLCK protein (MLCK), mRNA	NM_182493	Hs.130465	NM_182493
LOC343495	3.8	1.5	PREDICTED: similar to 60S ribosomal protein L6 (TAX-responsive enhancer element-binding protein 107) (TAXREB107) (Neoplasm-related protein C140) (LOC343495), mRNA	XR_016540	Hs.682141	XR_018840
EFHC1	3.8	1.9	EFHC1 (EFHC1) mRNA, partial cds.	AY608689	Hs.403171	AY608689
LOC90835	3.8	1.6	hypothetical protein LOC90835 (LOC90835), mRNA	NM_001014979	Hs.65735	AL122109
MTPN	3.8	2.5	myotrophin (MTPN), mRNA	NM_145808	Hs.654894	AL834231
A_32_P144629	3.8	1.4	A_32_P144629	A_32_P144629	Unknown	
GUK1	3.8	1.8	guanylate kinase 1 (GUK1), mRNA	NM_000858	Hs.376933	AK124677
ATP7B	3.8	2.3	ATPase, Cu++ transporting, beta polypeptide (ATP7B), transcript variant 1, mRNA	NM_000053	Hs.492280	NM_000053
AL355688	3.8	2.4	EST from clone 208499, full insert.	AL355688	Hs.655029	AL355688
AK092681	3.8	7.7	cDNA FLJ35362 fis, clone SKMUS2000330.	AK092681	Hs.668429	AK092681
CR596214	3.8	1.5	full-length cDNA clone CS0DC006YB07 of Neuroblastoma Cot 25-normalized of (human).	CR596214	Hs.662029	BG036557
MASP1	3.8	5.0	mannan-binding lectin serine peptidase 1 (C4/C2 activating component of Ra-reactive factor) (MASP1), transcript variant 3, mRNA	NM_001031849	Hs.89983	BX641029
ENST00000367696	3.8	1.7	Roquin (RING finger and C3H zinc finger protein 1).	ENST00000367696	Unknown	
TM9SF3	3.8	1.8	Transmembrane 9 superfamily protein member 3 precursor (SM-11044- binding protein) (EP70-P-iso).	ENST00000371142	Unknown	
ZBTB41	3.8	3.6	zinc finger and BTB domain containing 41 (ZBTB41), mRNA	NM_194314	Hs.529439	NM_194314
KLF11	3.8	1.8	Kruppel-like factor 11 (KLF11), mRNA	NM_003597	Hs.12229	BC063286
SVEP1	3.8	5.3	cDNA FLJ14964 fis, clone PLACE4000581, moderately similar to FIBROPELLIN I PRECURSOR.	AK027870	Unknown	
THC2603717	3.8	1.8	Q8WNW4_PIG (Q8WNW4) Beta-catenin, partial (11%)	THC2603717	Unknown	
THC2567891	3.8	1.5	Q6NXR8_HUMAN (Q6NXR8) Ribosomal protein S3a, partial (91%)	THC2567891	Unknown	
CCDC25	3.8	1.6	coiled-coil domain containing 25 (CCDC25), mRNA	NM_018246	Hs.445512	NM_018246
LOC284542	3.8	3.8	mRNA for FLJ00388 protein.	AK090467	Hs.61504	AK090467
CDH7	3.8	1.8	cadherin 7, type 2 (CDH7), transcript variant b, mRNA	NM_004361	Hs.657522	BC036786
FLJ25371	3.8	9.5	hypothetical protein FLJ25371 (FLJ25371), mRNA	NM_152543	Hs.415576	BG182943
ICA1L	3.8	2.8	islet cell autoantigen 1,69kDa-like (ICA1L), transcript variant 1, mRNA	NM_138468	Hs.516629	NM_138468
AK131287	3.8	2.0	cDNA FLJ16240 fis, clone HCASM2003357.	AK131287	Hs.633373	AK131287

CBWD5	3.8	2.7	COBW domain containing 5 (CBWD5), mRNA	NM_001024916	Hs.645337	BC067803
CTNNBIP1	3.8	1.8	catenin, beta interacting protein 1 (CTNNBIP1), transcript variant 1, mRNA	NM_020248	Hs.463759	NM_020248
FAM36A	3.8	2.2	family with sequence similarity 36, member A (FAM36A), mRNA	NM_198076	Hs.411490	AK095297
THC2664068	3.8	4.1	Q2IMJ3_ANADE (Q2IMJ3) LigA, partial (5%)	THC2664068	Unknown	
IL13RA1	3.8	3.3	interleukin 13 receptor, alpha 1 (IL13RA1), mRNA	NM_001560	Hs.496646	Y10659
AK026718	3.8	2.7	cDNA: FLJ23065 fis, clone LNG04894.	AK026718	Hs.125352	AK026718
NPY	3.8	3.4	neuropeptide Y (NPY), mRNA	NM_000905	Hs.1832	BF680552
RPL23A	3.8	1.5	ribosomal protein L23a (RPL23A), mRNA	NM_000984	Hs.419463	CR616046
UBQLN2	3.8	1.6	ubiquilin 2 (UBQLN2), mRNA	NM_013444	Hs.179309	AF189009
THC2638232	3.8	1.7	Q7RQ28_PLAYO (Q7RQ28) Nuclear protein snf7, partial (7%)	THC2638232	Unknown	
NEK1	3.8	1.8	NIMA (never in mitosis gene a)-related kinase 1 (NEK1), mRNA	NM_012224	Hs.481181	CR933642
CA3	3.8	4.7	carbonic anhydrase III, muscle specific (CA3), mRNA	NM_005181	Hs.82129	NM_005181
ZFYVE16	3.8	1.8	zinc finger, FYVE domain containing 16 (ZFYVE16), mRNA	NM_014733	Hs.482660	AB002303
LOC644907	3.8	1.5	PREDICTED: similar to 60S ribosomal protein L32 (LOC644907), mRNA	XM_930195	Unknown	
SERPIND1	3.8	2.4	serpin peptidase inhibitor, clade D (heparin cofactor), member 1 (SERPIND1), mRNA	NM_000185	Hs.474270	NM_000185
LOC389644	3.8	1.9	PREDICTED: similar to ribosomal protein L5 (LOC389644), mRNA	XR_018975	Hs.647124	XR_018975
A_32_P213946	3.8	2.2	A_32_P213946	A_32_P213946	Unknown	
AW977527	3.7	4.1	EST389636 MAGE resequences, MAGO cDNA, mRNA sequence	AW977527	Hs.611098	AW977527
SLC25A40	3.7	1.6	mRNA; cDNA DKFZp564D0472 (from clone DKFZp564D0472)	AL110179	Hs.208414	AL110179
C4orf12	3.7	1.9	cDNA FLJ42672 fis, clone BRAMY2026533.	AK124663	Hs.46730	AK124663
RPL22	3.7	1.8	ribosomal protein L22 (RPL22), mRNA	NM_000983	Hs.515329	NM_000983
HNRPH2	3.7	1.4	heterogeneous nuclear ribonucleoprotein H2 (H') (HNRPH2), transcript variant 1, mRNA	NM_019597	Hs.632828	NM_019597
UBE2J1	3.7	2.1	ubiquitin-conjugating enzyme E2, J1 (UBC6 homolog, yeast) (UBE2J1), mRNA	NM_016021	Hs.163776	NM_016021
AFF4	3.7	1.9	AF4/FMR2 family, member 4 (AFF4), mRNA	NM_014423	Hs.519313	NM_014423
DPYSL5	3.7	2.9	Dihydropyrimidinase-related protein 5 (DRP-5) (ULIP6 protein) (Collapsin response mediator protein 5) (CRMP-5) (CRMP3-associated molecule) (CRAM).	ENST00000380438	Unknown	
BU956542	3.7	1.4	BU956542 AGENCOURT_10615527 NIH_MGC_107 cDNA clone IMAGE:6730153 5', mRNA sequence	BU956542	Hs.448879	BQ055284
C12orf4	3.7	1.5	chromosome 12 open reading frame 4 (C12orf4), mRNA	NM_020374	Hs.302977	NM_020374
AL137571	3.7	2.8	mRNA; cDNA DKFZp586O1621 (from clone DKFZp586O1621).	AL137571	Unknown	
BC006271	3.7	2.8	KIAA1267, mRNA (cDNA clone IMAGE:3948445), complete cds.	BC006271	Unknown	
ZNF596	3.7	11.1	zinc finger protein 596 (ZNF596), transcript variant 1, mRNA	NM_001042416	Hs.591388	NM_001042416
SPG20	3.7	2.1	spastic paraplegia 20, spartin (Troyer syndrome) (SPG20), mRNA	NM_015087	Hs.440414	NM_015087
FABP3	3.7	3.9	fatty acid binding protein 3, muscle and heart (mammary-derived growth inhibitor) (FABP3), mRNA	NM_004102	Hs.657242	BG336702
ENST00000320746	3.7	2.9	similar to a disintegrin and metalloprotease domain 4 (LOC646548), mRNA	ENST00000320746	Unknown	
BC037838	3.7	1.5	cDNA clone IMAGE:4813920.	BC037838	Hs.594876	AK025546
ZFP30	3.7	4.2	zinc finger protein 30 homolog (mouse) (ZFP30), mRNA	NM_014898	Hs.116622	AB023178
TEKT2	3.7	2.8	tektin 2 (testicular) (TEKT2), mRNA	NM_014466	Hs.127111	BC022005
HOXD1	3.7	5.8	homeobox D1 (HOXD1), mRNA	NM_024501	Hs.83465	AF241528
FZD4	3.7	2.6	frizzled homolog 4 (Drosophila) (FZD4), mRNA	NM_012193	Hs.591968	AB032417
AK024171	3.7	1.6	cDNA FLJ14109 fis, clone MAMMA1001322, moderately similar to B-CELL GROWTH FACTOR PRECURSOR.	AK024171	Hs.633042	AK024171
THC2652887	3.7	1.8	Q2PGH6_HAELO (Q2PGH6) Mucin (Fragment), partial (14%)	THC2652887	Unknown	
KCTD10	3.7	1.9	potassium channel tetramerisation domain containing 10 (KCTD10), mRNA	NM_031954	Hs.524731	AB209358
RHOJ	3.7	6.4	ras homolog gene family, member J (RHOJ), mRNA	NM_020663	Hs.656339	AK096948
C3orf63	3.7	2.2	chromosome 3 open reading frame 63 (C3orf63), mRNA	NM_015224	Hs.116877	AF180425
THC2662240	3.7	8.3	THC2662240	THC2662240	Unknown	
AF229804	3.7	1.7	endozepine-like protein type 2 mutant mRNA, complete cds.	AF229804	Hs.662289	AF229804
AY358815	3.7	5.0	clone DNA108923 SFVP2550 (UNQ2550) mRNA, complete cds.	AY358815	Hs.689625	AY358815
LOC641522	3.7	2.5	ADP-ribosylation factor-like protein, mRNA (cDNA clone IMAGE:3907731).	BC071681	Hs.559259	AK021675
HMP19	3.7	2.2	HMP19 protein (HMP19), mRNA	NM_015980	Hs.559412	AK098398
BC047708	3.7	1.7	Homo sapiens, clone IMAGE:5750141, mRNA.	BC047708	Hs.652669	DQ287933
THC2756581	3.7	1.9	Q4RAV2_TETNG (Q4RAV2) Chromosome undetermined SCAF22857, whole genome shotgun sequence. (Fragment), partial (19%)	THC2756581	Unknown	
CSS3	3.7	3.1	chondroitin sulfate synthase 3 (CSS3), mRNA	NM_175856	Hs.213137	AJ578034
ZNF702	3.7	3.8	zinc finger protein 702 (ZNF702), mRNA	NM_024924	Unknown	

ZDHHC21	3.7	2.8	zinc finger, DHHC-type containing 21 (ZDHHC21), mRNA	NM_178566	Hs.649522	AK127313
KLB	3.7	2.5	klotho beta (KLB), mRNA	NM_175737	Hs.90756	BC033021
CXXC4	3.7	4.7	CXXC finger 4 (CXXC4), mRNA	NM_025212	Hs.12248	AK127778
KIAA0372	3.7	1.5	Tetratricopeptide repeat protein KIAA0372 (TPR repeat protein KIAA0372).	ENST00000380021	Unknown	
PLA2G12B	3.7	2.3	phospholipase A2, group XIIB (PLA2G12B), mRNA	NM_032562	Hs.333175	AF339053
TBC1D8B	3.7	3.4	TBC1 domain family, member 8B (with GRAM domain) (TBC1D8B), transcript variant 1, mRNA	NM_017752	Hs.351798	NM_017752
COLEC12	3.7	3.6	collectin sub-family member 12 (COLEC12), transcript variant II, mRNA	NM_030781	Unknown	
ODF2	3.7	1.8	outer dense fiber of sperm tails 2 (ODF2), transcript variant 2, mRNA	NM_153437	Hs.129055	NM_002540
FMO4	3.7	2.9	flavin containing monooxygenase 4 (FMO4), mRNA	NM_002022	Hs.386502	BC002780
OLFM4	3.7	6.0	olfactomedin 4 (OLFM4), mRNA	NM_006418	Hs.559736	AY358567
MAP3K1	3.7	2.5	Mitogen-activated protein kinase kinase kinase 1 (EC 2.7.11.25) (MAPK/ERK kinase kinase 1) (MEK kinase 1) (MEKK 1) (Fragment).	ENST00000264777	Unknown	
MTMR3	3.7	1.4	myotubularin related protein 3 (MTMR3), transcript variant 3, mRNA	NM_021090	Hs.474536	NM_021090
DMD	3.7	2.2	dystrophin (muscular dystrophy, Duchenne and Becker types) (DMD), transcript variant Dp427p2, mRNA	NM_004010	Hs.495912	NM_004010
KIAA0372	3.7	1.5	Tetratricopeptide repeat protein KIAA0372 (TPR repeat protein KIAA0372).	ENST00000380021	Unknown	
CDKN1C	3.7	2.7	cyclin-dependent kinase inhibitor 1C (p57, Kip2) (CDKN1C), mRNA	NM_000076	Hs.106070	BC067842
THC2530075	3.7	4.5	THC2530075	THC2530075	Unknown	
RARRES1	3.7	2.7	retinoic acid receptor responder (tazarotene induced) 1 (RARRES1), transcript variant 2, mRNA	NM_002888	Hs.131269	NM_206963
LOC441208	3.7	2.5	hypothetical gene supported by AK094370 (LOC441208), mRNA	NM_001013723	Unknown	
TTC21B	3.7	2.3	mRNA for KIAA1992 protein.	AB082523	Unknown	
FXYD1	3.7	2.7	FXYD domain containing ion transport regulator 1 (phospholemman) (FXYD1), transcript variant a, mRNA	NM_005031	Hs.442498	AK124802
STMN2	3.7	4.2	SCG10=neuron-specific growth-associated protein/stathmin homolog .	S82024	Unknown	
LOC645158	3.7	3.0	hypothetical protein LOC645158, mRNA (cDNA clone IMAGE:4798132), partial cds.	BC018088	Hs.699827	XM_932692
SNX19	3.7	2.0	sorting nexin 19 (SNX19), mRNA	NM_014758	Hs.444024	NM_014758
AK098354	3.7	3.0	cDNA FLJ25488 fis, clone CBR00232.	AK098354	Hs.525626	NM_001100913
PPA2	3.7	1.5	pyrophosphatase (inorganic) 2 (PPA2), nuclear gene encoding mitochondrial protein, transcript variant 1, mRNA	NM_176869	Hs.654957	AL833123
MAPK8	3.7	1.7	Mitogen-activated protein kinase 8 (EC 2.7.11.24) (Stress-activated protein kinase JNK1) (c-Jun N-terminal kinase 1) (JNK-46).	ENST00000374189	Unknown	
TMEM116	3.7	1.9	transmembrane protein 116 (TMEM116), mRNA	NM_138341	Hs.506815	AL833501
A_24_P298616	3.7	1.7	A_24_P298616	A_24_P298616	Unknown	
TCEA3	3.7	16.5	transcription elongation factor A (SII), 3 (TCEA3), mRNA	NM_003196	Hs.446354	NM_003196
PTGS2	3.7	6.7	prostaglandin-endoperoxide synthase 2 (prostaglandin G/H synthase and cyclooxygenase) (PTGS2), mRNA	NM_000963	Hs.196384	NM_000963
THC2646626	3.7	3.5	THC2646626	THC2646626	Unknown	
FLJ25476	3.7	2.1	FLJ25476 protein (FLJ25476), mRNA	NM_152493	Hs.524248	NM_152493
FLJ44048	3.7	59.3	FLJ44048 protein (FLJ44048), mRNA	NM_207482	Hs.98025	BX648733
ADH4	3.7	19.6	alcohol dehydrogenase 4 (class II), pi polypeptide (ADH4), mRNA	NM_000670	Hs.1219	M15943
MGC39900	3.7	2.2	hypothetical protein MGC39900 (MGC39900), mRNA	NM_194324	Unknown	
ZNF415	3.7	1.9	zinc finger protein 415 (ZNF415), mRNA	NM_018355	Hs.147765	AK095504
C20orf112	3.7	2.5	cDNA FLJ40485 fis, clone TESTI2043857, moderately similar to nolp mRNA.	AK097804	Hs.516978	AL122043
GLCE	3.7	2.5	UDP-glucuronic acid epimerase (GLCE), mRNA	NM_015554	Hs.183006	NM_015554
RANBP17	3.7	1.6	RAN binding protein 17 (RANBP17), mRNA	NM_022897	Hs.410810	AJ288954
THC2570021	3.7	2.2	AF124725 acinusS {Mus musculus} (exp=-1; wgp=0; cg=0), partial (3%)	THC2570021	Unknown	
DYRK1A	3.7	1.5	dual-specificity tyrosine-(Y)-phosphorylation regulated kinase 1A (DYRK1A), transcript variant 2, mRNA	NM_130436	Hs.696102	NM_130436
AKAP6	3.7	1.7	A kinase (PRKA) anchor protein 6 (AKAP6), mRNA	NM_004274	Hs.509083	BC150288
BC030118	3.7	1.9	cDNA clone IMAGE:4812643.	BC030118	Hs.594897	BC030118
MDM4	3.7	2.7	mRNA; cDNA DKFZp686B01123 (from clone DKFZp686B01123).	BX640923	Hs.658187	BX640923
LOC134145	3.7	1.7	hypothetical protein LOC134145 (LOC134145), mRNA	NM_199133	Hs.481569	AK000674
C20orf77	3.7	2.9	chromosome 20 open reading frame 77 (C20orf77), mRNA	NM_021215	Hs.278839	NM_021215
SPG20	3.7	2.0	spastic paraplegia 20, spartin (Troyer syndrome) (SPG20), mRNA	NM_015087	Hs.440414	NM_015087
MAF	3.7	2.3	v-maf musculoaponeurotic fibrosarcoma oncogene homolog (avian) (MAF), transcript variant 2, mRNA	NM_001031804	Hs.699396	NM_001031804
CRY1	3.7	1.6	cryptochrome 1 (photolyase-like) (CRY1), mRNA	NM_004075	Hs.151573	AK125915
ARPP-21	3.7	10.3	cyclic AMP-regulated phosphoprotein, 21 kD (ARPP-21), transcript variant 1, mRNA	NM_016300	Hs.475902	NM_016300
CCDC34	3.7	4.5	coiled-coil domain containing 34 (CCDC34), transcript variant 2, mRNA	NM_080654	Hs.143733	BC008496

ERG	3.7	5.8	v-ets erythroblastosis virus E26 oncogene homolog (avian) (ERG), transcript variant 2, mRNA	NM_004449	Hs.473819	M21535
ATP2A2	3.7	1.9	ATPase, Ca++ transporting, cardiac muscle, slow twitch 2 (ATP2A2), transcript variant 2, mRNA	NM_001681	Hs.506759	BX648282
KCNMA1	3.7	2.5	potassium large conductance calcium-activated channel, subfamily M, alpha member 1 (KCNMA1), transcript variant 2, mRNA	NM_002247	Hs.144795	NM_001014797
FAM44A	3.7	2.5	family with sequence similarity 44, member A, mRNA (cDNA clone IMAGE:5503684), with apparent retained intron.	BC043603	Hs.444517	NM_148894
BAD	3.7	1.8	BCL2-antagonist of cell death (BAD), transcript variant 1, mRNA	NM_004322	Hs.370254	AK023420
LOXL4	3.7	2.6	lysyl oxidase-like 4 (LOXL4), mRNA	NM_032211	Hs.306814	BC013153
METTL9	3.7	1.8	methyltransferase like 9 (METTL9), transcript variant 1, mRNA	NM_016025	Hs.279583	NM_016025
BC013657	3.7	6.3	cDNA clone IMAGE:4152983, partial cds.	BC013657	Hs.621337	BC013657
USP18	3.7	3.1	ubiquitin specific peptidase 18 (USP18), mRNA	NM_017414	Hs.38260	NM_017414
FRMD4A	3.7	2.0	cDNA FLJ10210 fis, clone HEMBA1006344, weakly similar to RADIXIN.	AK001072	Hs.330463	BC151244
PRKD3	3.7	1.7	protein kinase D3 (PRKD3), mRNA	NM_005813	Hs.696257	NM_005813
TITF1	3.7	7.3	thyroid transcription factor 1 (TITF1), transcript variant 2, mRNA	NM_003317	Hs.700584	NM_003317
MRFAP1L1	3.7	2.0	Morf4 family associated protein 1-like 1 (MRFAP1L1), transcript variant 1, [illegible]	NM_152301	Hs.593159	AF258591
APC	3.7	1.7	adenomatosis polyposis coli (APC), mRNA	NM_000038	Hs.158932	NM_000038
CDH17	3.7	4.3	cadherin 17, LI cadherin (liver-intestine) (CDH17), mRNA	NM_004063	Hs.591853	NM_004063
EVI1	3.7	5.4	mRNA; cDNA DKFZp686J18113 (from clone DKFZp686J18113).	BX640908	Hs.656395	BX640908
FLJ31659	3.7	4.0	hypothetical protein FLJ31659 (FLJ31659), mRNA	NM_153027	Hs.178648	AK056221
TNFAIP1	3.7	2.5	tumor necrosis factor, alpha-induced protein 1 (endothelial) (TNFAIP1), mRNA	NM_021137	Hs.76090	BC003694
F5	3.7	3.2	coagulation factor V (proaccelerin, labile factor) (F5), mRNA	NM_000130	Hs.30054	NM_000130
ACTR10	3.7	2.1	actin-related protein 10 homolog (S. cerevisiae) (ACTR10), mRNA	NM_018477	Hs.509451	NM_018477
C3orf60	3.7	1.8	chromosome 3 open reading frame 60 (C3orf60), transcript variant 5, mRNA	NM_199417	Hs.31387	BC111004
ENST00000380195	3.7	2.4	PCLO_HUMAN Isoform 3 of Q9Y6V0 - (Human)	ENST00000380195	Unknown	
THC2734830	3.7	3.3	THC2734830	THC2734830	Unknown	
SGCE	3.7	1.8	sarcoglycan, epsilon (SGCE), mRNA	NM_003919	Hs.371199	NM_001099401
THC2563387	3.7	2.1	CD676691 ho04g02.y1 Human Trabecular meshwork cDNA: hohphq cDNA clone ho04g02 5', mRNA sequence	THC2563387	Unknown	
MYH7B	3.7	6.4	myosin, heavy chain 7B, cardiac muscle, beta (MYH7B), mRNA	NM_020884	Hs.414122	BC151242
INTS12	3.7	1.7	integrator complex subunit 12 (INTS12), mRNA	NM_020395	Hs.480454	NM_020395
ZNF334	3.7	1.4	zinc finger protein 334 (ZNF334), transcript variant 2, mRNA	NM_199441	Hs.584933	BC026321
BC051742	3.7	2.6	cDNA clone IMAGE:4827065.	BC051742	Hs.586388	BC034827
ARPP-21	3.7	9.1	cyclic AMP-regulated phosphoprotein, 21 kD (ARPP-21), transcript variant 4, mRNA	NM_001025069	Hs.475902	NM_016300
KRCC1	3.7	2.0	lysine-rich coiled-coil 1 (KRCC1), mRNA	NM_016618	Hs.469254	AK025986
MTHFSD	3.7	1.4	cDNA FLJ43025 fis, clone BRTHA2018707.	AK125015	Unknown	
THC2485398	3.7	2.2	ALU1_HUMAN (P39188) Alu subfamily J sequence contamination warning entry, partial (18%)	THC2485398	Unknown	
FGF10	3.7	6.4	cDNA FLJ13446 fis, clone PLACE1002968.	AK023508	Hs.248049	AK023508
AVIL	3.7	3.0	advillin (AVIL), mRNA	NM_006576	Hs.584854	BX647344
PPARA	3.7	2.9	peroxisome proliferator-activated receptor alpha (PPARA), transcript variant 5, mRNA	NM_005036	Hs.103110	NM_005036
BG031574	3.7	2.1	602299712F1 NIH_MGC_87 cDNA clone IMAGE:4394138 5', mRNA sequence	BG031574	Hs.701122	BG031574
MAP4K5	3.7	1.9	mitogen-activated protein kinase kinase kinase kinase 5 (MAP4K5), transcript variant 2, mRNA	NM_198794	Hs.130491	NM_198794
LOC286334	3.7	1.4	mRNA full length insert cDNA clone EUROIMAGE 1517766.	AJ420454	Hs.349208	NM_133374
ARID5B	3.7	3.2	AT rich interactive domain 5B (MRF1-like) (ARID5B), mRNA	NM_032199	Hs.535297	NM_032199
LIFR	3.7	2.2	leukemia inhibitory factor receptor alpha (LIFR), mRNA	NM_002310	Hs.133421	NM_002310
LAMA3	3.7	2.1	laminin, alpha 3 (LAMA3), transcript variant 1, mRNA	NM_198129	Hs.436367	NM_198129
ATP11B	3.7	1.7	ATPase, Class VI, type 11B (ATP11B), mRNA	NM_014616	Hs.478429	NM_014616
PEX11B	3.7	2.6	peroxisomal biogenesis factor 11B (PEX11B), mRNA	NM_003846	Hs.699232	AK094173
ACVR2A	3.7	1.7	activin A receptor, type IIA (ACVR2A), mRNA	NM_001616	Hs.470174	NM_001616
C1orf63	3.7	3.2	chromosome 1 open reading frame 63, mRNA (cDNA clone MGC:74698 IMAGE:6147639), complete cds.	BC065040	Hs.259412	AK096351
A_32_P139260	3.7	3.6	A_32_P139260	A_32_P139260	Unknown	
RBMS1	3.7	1.5	RNA binding motif, single stranded interacting protein 1 (RBMS1), transcript variant 1, mRNA	NM_016836	Hs.470412	NM_016839
THC2526838	3.7	3.0	Q32R99_9PICI (Q32R99) NADH dehydrogenase subunit 2, partial (5%)	THC2526838	Unknown	
DDEF1	3.7	1.9	development and differentiation enhancing factor 1 (DDEF1), mRNA	NM_018482	Hs.655552	NM_018482
ALX3	3.7	4.8	aristaless-like homeobox 3 (ALX3), mRNA	NM_006492	Hs.669953	AF008203

CTGLF4	3.7	1.6	centaurin, gamma-like family, member 4 (CTGLF4), mRNA	NM_001077685	Hs.528346	NM_001077685
PB1	3.7	1.9	polybromo 1 (PB1), transcript variant 2, mRNA	NM_018313	Hs.189920	NM_018313
6-Sep	3.7	4.5	septin 6 (SEPT6), transcript variant I, mRNA	NM_145799	Hs.496666	NM_145799
AK001829	3.7	2.0	cDNA FLJ10967 fis, clone PLACE1000798.	AK001829	Hs.191582	BM043035
SPOP	3.7	1.9	speckle-type POZ protein (SPOP), transcript variant 1, mRNA	NM_001007226	Hs.463382	AK125087
ACY1L2	3.7	1.8	aminoacylase 1-like 2 (ACY1L2), mRNA	NM_001010853	Hs.699277	BC035036
CALB2	3.7	4.4	calbindin 2, 29kDa (calretinin) (CALB2), transcript variant CALB2, mRNA	NM_001740	Hs.106857	NM_001740
RPS6KA5	3.7	2.2	ribosomal protein S6 kinase, 90kDa, polypeptide 5 (RPS6KA5), transcript variant 1, mRNA	NM_004755	Hs.510225	AB209667
ENST00000309260	3.7	1.3	Transcription factor BTF3 homolog 3 (Basic transcription factor 3-like 3).	ENST00000309260	Unknown	
CRIP2	3.7	3.8	cysteine-rich protein 2 (CRIP2), mRNA	NM_001312	Hs.534309	AK091845
LOC400566	3.7	1.6	hypothetical gene supported by AK128660 (LOC400566), mRNA	NM_001013672	Hs.499607	BC057385
LIFR	3.7	2.5	leukemia inhibitory factor receptor alpha (LIFR), mRNA	NM_002310	Hs.133421	NM_002310
ASB9	3.7	3.5	ankyrin repeat and SOCS box-containing 9 (ASB9), transcript variant 1, mRNA	NM_001031739	Hs.19404	AK000643
PRR4	3.7	1.3	proline rich 4 (lacrimal) (PRR4), mRNA	NM_007244	Hs.408153	BX648418
THC2663894	3.7	1.8	THC2663894	THC2663894	Unknown	
AK129879	3.7	2.6	cDNA FLJ26369 fis, clone HRT06001.	AK129879	Hs.656600	CR603940
BC035156	3.7	1.9	cDNA clone IMAGE:5265210.	BC035156	Hs.658127	BC035156
FAM27E1	3.7	2.9	family with sequence similarity 27, member E1 (FAM27E1), mRNA	NM_001024608	Unknown	
THC2679213	3.7	2.9	THC2679213	THC2679213	Unknown	
GOLGA8A	3.7	2.6	golgi autoantigen, golgin subfamily a, 8A (GOLGA8A), mRNA	NM_181077	Hs.182982	BX648160
THC2532458	3.7	2.1	NM_205486 non-histone chromosomal protein {Gallus gallus} (exp=-1; wgp=0; cg=0), partial (21%)	THC2532458	Unknown	
ZNF650	3.7	1.7	zinc finger protein 650 (ZNF650), mRNA	NM_172070	Hs.379548	BC044060
TCTEX1D1	3.7	2.6	Tctex1 domain containing 1 (TCTEX1D1), mRNA	NM_152665	Hs.479226	AK098192
DFNB59	3.7	1.8	deafness, autosomal recessive 59 (DFNB59), mRNA	NM_001042702	Hs.87734	BC020859
ZNF284	3.7	1.8	mRNA; cDNA DKFZp781F1775 (from clone DKFZp781F1775).	CR936662	Hs.445395	CR936662
MRPL53	3.7	1.6	mitochondrial ribosomal protein L53 (MRPL53), nuclear gene encoding mitochondrial protein, mRNA	NM_053050	Hs.534527	BM467462
THC2688038	3.7	15.2	THC2688038	THC2688038	Unknown	
C20orf39	3.7	1.7	chromosome 20 open reading frame 39 (C20orf39), mRNA	NM_024893	Hs.124638	AK024282
EFNA4	3.7	1.6	ephrin-A4 (EFNA4), transcript variant 3, mRNA	NM_182690	Hs.449913	NM_005227
RNF111	3.7	1.5	ring finger protein 111 (RNF111), mRNA	NM_017610	Hs.404423	NM_017610
ALDH1A2	3.7	2.2	aldehyde dehydrogenase 1 family, member A2 (ALDH1A2), transcript variant 3, mRNA	NM_170697	Hs.699620	AK128709
PGBD2	3.7	1.6	piggyBac transposable element derived 2 (PGBD2), transcript variant 1, mRNA	NM_170725	Hs.602037	BX647065
PPIL3	3.6	1.3	peptidylprolyl isomerase (cyclophilin)-like 3 (PPIL3), transcript variant PPIL3c, mRNA	NM_131916	Hs.121076	NM_131916
ZBTB8	3.6	1.5	zinc finger and BTB domain containing 8 (ZBTB8), mRNA	NM_001040441	Hs.546479	NM_001040441
TAS2R10	3.6	2.1	taste receptor, type 2, member 10 (TAS2R10), mRNA	NM_023921	Hs.533756	BC101764
LNPEP	3.6	3.0	cDNA FLJ39485 fis, clone PROST2015124.	AK096804	Hs.527199	AK096804
AK125176	3.6	8.0	cDNA FLJ43186 fis, clone FCBBF3022767.	AK125176	Hs.648505	AK125176
RHOJ	3.6	6.5	ras homolog gene family, member J (RHOJ), mRNA	NM_020663	Hs.656339	AK096948
SRGAP2	3.6	1.8	SLIT-ROBO Rho GTPase activating protein 2 (SRGAP2), transcript variant 1, mRNA	NM_015326	Hs.497575	NM_015326
DNAJB12	3.6	1.9	DnaJ (Hsp40) homolog, subfamily B, member 12 (DNAJB12), transcript variant 1, mRNA	NM_001002762	Hs.696014	NM_001002762
BC047708	3.6	2.1	Homo sapiens, clone IMAGE:5750141, mRNA.	BC047708	Hs.652669	DQ287933
RDH14	3.6	1.5	retinol dehydrogenase 14 (all-trans/9-cis/11-cis) (RDH14), mRNA	NM_020905	Hs.288880	AK023625
H6PD	3.6	2.3	hexose-6-phosphate dehydrogenase (glucose 1-dehydrogenase) (H6PD), mRNA	NM_004285	Hs.463511	NM_004285
BX648566	3.6	7.2	mRNA; cDNA DKFZp779F0655 (from clone DKFZp779F0655).	BX648566	Hs.658808	BX648566
PLD1	3.6	3.1	phospholipase D1, phosphatidylcholine-specific (PLD1), mRNA	NM_002662	Hs.382865	AB209907
ENST00000247761	3.6	1.3	ENST00000247761	ENST00000247761	Unknown	
VGLL1	3.6	2.1	vestigial like 1 (Drosophila) (VGLL1), mRNA	NM_016267	Hs.496843	NM_016267
ZNF189	3.6	1.6	zinc finger protein 189 (ZNF189), transcript variant 2, mRNA	NM_197977	Hs.50123	NM_197977
PREX1	3.6	2.4	phosphatidylinositol 3,4,5-trisphosphate-dependent RAC exchanger 1 (PREX1), mRNA	NM_020820	Hs.153310	NM_020820
KIAA1641	3.6	2.9	KIAA1641 (KIAA1641), mRNA	NM_020970	Unknown	
LOC731688	3.6	1.6	PREDICTED: similar to ribosomal protein L21 (LOC731688), mRNA	XR_015992	Hs.648368	XR_015992
TP53RK	3.6	1.8	TP53 regulating kinase (TP53RK), mRNA	NM_033550	Hs.440263	NM_033550

IGSF9	3.6	2.4	immunoglobulin superfamily, member 9 (IGSF9), mRNA	NM_020789	Hs.591472	AK056650
TMPRSS4	3.6	2.4	transmembrane protease, serine 4 (TMPRSS4), transcript variant 1, mRNA	NM_019894	Hs.161985	NM_019894
EID2B	3.6	1.8	EP300 interacting inhibitor of differentiation 2B (EID2B), mRNA	NM_152361	Hs.135181	AK096263
CES2	3.6	1.9	carboxylesterase 2 (intestine, liver) (CES2), transcript variant 1, mRNA	NM_003869	Hs.282975	NM_003869

Supplemental Table A3

Genes Downregulated in CM vs EB (2389)

SAM FDR 0.1%

Gene Name	Score(d)	Fold Downreg CM	Description	Acc	UGCluster	UGRepAcc
LIX1	-45.3	4.8	Lix1 homolog (mouse) (LIX1), mRNA [NM_153234]	NM_153234	Hs.656702	BC036467
LIX1	-24.4	7.2	Lix1 homolog (mouse) (LIX1), mRNA [NM_153234]	NM_153234	Hs.656702	BC036467
TWIST1	-19.8	5.2	twist homolog 1 (acrocephalosyndactyly 3; Saethre-Chotzen syndrome) (Drosophila) (TWIST1), mRNA [NM_000474]	NM_000474	Hs.66744	NM_000474
EVI1	-18.2	7.5	ecotropic viral integration site 1 (EVI1), mRNA [NM_005241]	NM_005241	Hs.656395	BX640908
CXCL12	-18.0	8.2	chemokine (C-X-C motif) ligand 12 (stromal cell-derived factor 1) (CXCL12), transcript variant 2, mRNA [NM_000609]	NM_000609	Hs.522891	AK090482
EVI1	-17.5	6.8	ecotropic viral integration site 1 (EVI1), mRNA [NM_005241]	NM_005241	Hs.656395	BX640908
CR620977	-16.7	4.4	full-length cDNA clone CS0CAP004YK15 of Thymus of (human). [CR620977]	CR620977	Hs.377961	CR620977
KLF12	-16.7	2.5	Kruppel-like factor 12 (KLF12), mRNA [NM_007249]	NM_007249	Hs.373857	NM_007249
THC2633747	-16.5	1.9	ALU2_HUMAN (P39189) Alu subfamily SB sequence contamination warning entry, partial (3%) [THC2691931]	THC2633747	Unknown	
CR623787	-16.2	4.6	full-length cDNA clone CS0DI079YL01 of Placenta Cot 25-normalized of (human). [CR623787]	CR623787	Hs.687264	CR623787
ITGA8	-15.9	6.7	integrin, alpha 8 (ITGA8), mRNA [NM_003638]	NM_003638	Hs.171311	NM_003638
FLJ21986	-15.4	15.8	hypothetical protein FLJ21986 (FLJ21986), mRNA [NM_024913]	NM_024913	Hs.189652	NM_024913
ARHGAP28	-15.0	5.1	Rho GTPase activating protein 28 (ARHGAP28), transcript variant 1, mRNA [NM_001010000]	NM_001010000	Hs.183114	NM_001010000
BC092503	-15.0	1.7	cDNA clone IMAGE:30334866. [BC092503]	BC092503	Hs.520804	AL832059
FGF9	-14.7	2.3	fibroblast growth factor 9 (glia-activating factor) (FGF9), mRNA [NM_002010]	NM_002010	Hs.111	D14838
IGF2AS	-14.5	5.2	insulin-like growth factor 2 antisense (IGF2AS), mRNA [NM_016412]	NM_016412	Hs.272259	NM_016412
TSHZ3	-14.4	3.6	teashirt family zinc finger 3 (TSHZ3), mRNA [NM_020856]	NM_020856	Hs.278436	NM_020856
LOC285733	-14.3	5.1	cDNA FLJ34581 fis, clone KIDNE2008480. [AK091900]	AK091900	Hs.388715	XM_379432
PRRX2	-14.2	8.5	paired related homeobox 2 (PRRX2), mRNA [NM_016307]	NM_016307	Hs.660115	BM909706
THC2523685	-14.0	2.5	BQ101125 ij24c06.y1 Melton Normalized Human Islet 4 N4-HIS 1 cDNA clone IMAGE:6135562 5', mRNA sequence [BQ101125]	THC2523685	Unknown	
EBF3	-14.0	3.3	early B-cell factor 3 (EBF3), mRNA [NM_001005463]	NM_001005463	Hs.699395	NM_001005463
CBFA2T3	-13.7	2.6	core-binding factor, runt domain, alpha subunit 2; translocated to, 3 (CBFA2T3), transcript variant 1, mRNA [NM_005187]	NM_005187	Hs.513811	NM_005187
ST7OT1	-13.6	3.9	ST7 overlapping transcript 1 (antisense non-coding RNA) (ST7OT1) on chromosome 7 [NR_002330]	NR_002330	Unknown	
GJA4	-13.3	11.6	gap junction protein, alpha 4, 37kDa (connexin 37) (GJA4), mRNA [NM_002060]	NM_002060	Hs.296310	NM_002060
APOB	-13.2	7.8	apolipoprotein B (including Ag(x) antigen) (APOB), mRNA [NM_000384]	NM_000384	Hs.120759	NM_000384
DNAJC12	-13.2	3.3	DnaJ (Hsp40) homolog, subfamily C, member 12 (DNAJC12), transcript variant 1, mRNA [NM_021800]	NM_021800	Hs.260720	NM_021800
HSF2	-13.2	1.8	heat shock transcription factor 2 (HSF2), mRNA [NM_004506]	NM_004506	Hs.158195	BC112323
FLJ21986	-13.1	13.7	hypothetical protein FLJ21986 (FLJ21986), mRNA [NM_024913]	NM_024913	Hs.189652	NM_024913
APOB	-12.9	8.2	apolipoprotein B (including Ag(x) antigen) (APOB), mRNA [NM_000384]	NM_000384	Hs.120759	NM_000384
HEYL	-12.9	3.8	hairy/enhancer-of-split related with YRPW motif-like (HEYL), mRNA [NM_014571]	NM_014571	Hs.472566	NM_014571
PLAG1	-12.9	2.4	pleiomorphic adenoma gene 1 (PLAG1), mRNA [NM_002655]	NM_002655	Hs.14968	NM_002655
SST	-12.8	4.1	somatostatin (SST), mRNA [NM_001048]	NM_001048	Hs.12409	BI918626
AK124941	-12.7	4.4	cDNA FLJ42951 fis, clone BRSTN2007765. [AK124941]	AK124941	Hs.656242	AK124941
CR590573	-12.4	3.7	full-length cDNA clone CS0DI042YD07 of Placenta Cot 25-normalized of (human). [CR590573]	CR590573	Hs.484967	CR590573
ZHX1	-12.4	2.2	zinc fingers and homeoboxes 1 (ZHX1), transcript variant 1, mRNA [NM_001017926]	NM_001017926	Hs.612084	NM_001017926
A_24_P92212	-12.4	1.7	A_24_P922120	A_24_P922120	Unknown	
HNRPDL	-12.3	3.0	heterogeneous nuclear ribonucleoprotein D-like (HNRPDL), transcript variant 2, mRNA [NM_031372]	NM_031372	Hs.527105	NM_031372
AK091028	-12.3	3.2	cDNA FLJ33709 fis, clone BRAWH2007890. [AK091028]	AK091028	Hs.188825	CR933638
ENST0000038	-12.3	4.1	ADAMTS-9 precursor (EC 3.4.24.-) (A disintegrin and metalloproteinase with thrombospondin motifs 9) (ADAM-TS 9) (ADAM-TS9). [Source:Uniprot/SWISSPROT;Acc:Q9P2N4] [ENST00000383706]	ENST0000038370	Unknown	
CXCR7	-12.2	2.6	chemokine (C-X-C motif) receptor 7 (CXCR7), transcript variant 1, mRNA [NM_001047841]	NM_001047841	Unknown	
HEY1	-12.1	6.3	hairy/enhancer-of-split related with YRPW motif 1 (HEY1), transcript variant 2, mRNA [NM_001040708]	NM_001040708	Hs.234434	NM_001040708
HS3ST3B1	-12.1	3.6	Heparan sulfate glucosamine 3-O-sulfotransferase 3B1 (EC 2.8.2.30) (Heparan sulfate D-glucosaminyl 3-O-sulfotransferase 3B1) (Heparan sulfate 3-O-sulfotransferase 3B1) (h3-OST-3B). [Source:Uniprot/SWISSPROT;Acc:O9Y662] [ENST00000360954]	ENST0000036095	Unknown	
ZNF395	-12.1	3.1	zinc finger protein 395 (ZNF395), mRNA [NM_018660]	NM_018660	Hs.699209	NM_018660
THC2582897	-12.1	3.9	THC2582897	THC2582897	Unknown	

IL17D	-12.0	2.7	interleukin 17D (IL17D), mRNA [NM_138284]	NM_138284	Hs.655142	AK055408
LOC728555	-12.0	2.6	cDNA FLJ40901 fis, clone UTERU2003704. [AK098220]	AK098220	Hs.664334	AK098220
APOB	-11.9	8.1	apolipoprotein B (including Ag(x) antigen) (APOB), mRNA [NM_000384]	NM_000384	Hs.120759	NM_000384
APOB	-11.9	7.9	apolipoprotein B (including Ag(x) antigen) (APOB), mRNA [NM_000384]	NM_000384	Hs.120759	NM_000384
APOB	-11.8	7.8	apolipoprotein B (including Ag(x) antigen) (APOB), mRNA [NM_000384]	NM_000384	Hs.120759	NM_000384
SENP7	-11.8	3.0	SUMO1/sentrin specific peptidase 7 (SENP7), transcript variant 1, mRNA [NM_020654]	NM_020654	Hs.529551	NM_020654
EDG2	-11.7	5.5	endothelial differentiation, lysophosphatidic acid G-protein-coupled receptor, 2 (EDG2), transcript variant 2, mRNA [NM_057159]	NM_057159	Hs.126667	BC036034
EBF3	-11.7	3.8	early B-cell factor 3 (EBF3), mRNA [NM_001005463]	NM_001005463	Hs.699395	NM_001005463
FLJ10038	-11.7	2.4	cDNA FLJ10038 fis, clone HEMBA1000971. [AK000900]	AK000900	Hs.511316	BQ061333
SMOC1	-11.7	3.4	SPARC related modular calcium binding 1 (SMOC1), transcript variant 1, mRNA [NM_001034852]	NM_001034852	Hs.497349	NM_001034852
ELMOD1	-11.6	4.4	ELMO/CED-12 domain containing 1 (ELMOD1), mRNA [NM_018712]	NM_018712	Hs.495779	NM_018712
FLJ13197	-11.6	2.9	hypothetical protein FLJ13197 (FLJ13197), mRNA [NM_024614]	NM_024614	Unknown	
ENST0000038	-11.6	2.3	similar to 40S ribosomal protein S3a (V-fos transformation effector protein) (LOC391706), mRNA [Source:RefSeq_dna;Acc:XR_018587] [ENST00000389400]	ENST0000038940	Unknown	
BDH2	-11.6	2.8	3-hydroxybutyrate dehydrogenase, type 2 (BDH2), mRNA [NM_020139]	NM_020139	Hs.124696	NM_020139
ELMOD2	-11.6	2.1	ELMO domain-containing protein 2. [Source:Uniprot/SWISSPROT;Acc:Q8IZ81] [ENST00000323570]	ENST0000032357	Unknown	
C5orf23	-11.5	5.4	chromosome 5 open reading frame 23 (C5orf23), mRNA [NM_024563]	NM_024563	Hs.13528	BC022250
LYRM7	-11.5	2.1	Lyrm7 homolog (mouse) (LYRM7), mRNA [NM_181705]	NM_181705	Hs.115467	NM_181705
ENST0000037	-11.4	10.2	Protein patched homolog 1 (PTC1) (PTC). [Source:Uniprot/SWISSPROT;Acc:Q13635] [ENST00000375284]	ENST0000037528	Unknown	
APOB	-11.4	7.6	apolipoprotein B (including Ag(x) antigen) (APOB), mRNA [NM_000384]	NM_000384	Hs.120759	NM_000384
APOB	-11.4	8.1	apolipoprotein B (including Ag(x) antigen) (APOB), mRNA [NM_000384]	NM_000384	Hs.120759	NM_000384
KIAA1212	-11.2	2.1	KIAA1212 (KIAA1212), mRNA [NM_018084]	NM_018084	Hs.292925	NM_018084
APOB	-11.2	8.2	apolipoprotein B (including Ag(x) antigen) (APOB), mRNA [NM_000384]	NM_000384	Hs.120759	NM_000384
ZNF268	-11.2	2.7	H.sapiens HZF3 mRNA for zinc finger protein. [X78926]	X78926	Hs.654533	BC142989
LOC285260	-11.1	1.9	PREDICTED: similar to ribosomal protein L31 (LOC285260), mRNA [XR_019376]	XR_019376	Hs.646857	XR_019376
LOC653773	-11.1	1.9	PREDICTED: similar to ribosomal protein L31 (LOC653773), mRNA [XR_017639]	XR_017639	Hs.647888	XR_017639
HOXB3	-11.1	4.0	homeobox B3 (HOXB3), mRNA [NM_002146]	NM_002146	Hs.654560	NM_002146
FAM44A	-11.0	2.0	family with sequence similarity 44, member A (FAM44A), mRNA [NM_148894]	NM_148894	Hs.444517	NM_148894
KIAA1212	-10.9	2.1	KIAA1212 (KIAA1212), mRNA [NM_018084]	NM_018084	Hs.292925	NM_018084
RPL7	-10.9	1.7	ribosomal protein L7 (RPL7), mRNA [NM_000971]	NM_000971	Hs.571841	BM808571
AK021785	-10.9	5.0	cDNA FLJ11723 fis, clone HEMBA1005314. [AK021785]	AK021785	Hs.586722	AK021785
MAP2K6	-10.8	3.0	mitogen-activated protein kinase kinase 6 (MAP2K6), mRNA [NM_002758]	NM_002758	Hs.463978	BX641121
ENST0000037	-10.8	5.4	Novel protein. [Source:Uniprot/SPTREMBL;Acc:Q5VSD8] [ENST00000377548]	ENST0000037754	Unknown	
HNRPH3	-10.7	2.6	heterogeneous nuclear ribonucleoprotein H3 (2H9) (HNRPH3), transcript variant 2H9, mRNA [NM_012207]	NM_012207	Hs.699337	BC039824
ISL1	-10.6	4.3	ISL1 transcription factor, LIM/homeodomain, (islet-1) (ISL1), mRNA [NM_002202]	NM_002202	Hs.505	NM_002202
THC2714296	-10.6	2.5	THC2714296	THC2714296	Unknown	
CTTNBP2	-10.6	6.8	cortactin binding protein 2 (CTTNBP2), mRNA [NM_033427]	NM_033427	Hs.592285	NM_033427
F2	-10.6	4.7	coagulation factor II (thrombin) (F2), mRNA [NM_000506]	NM_000506	Hs.655207	BC051332
GAS1	-10.5	2.6	growth arrest-specific 1 (GAS1), mRNA [NM_002048]	NM_002048	Hs.65029	NM_002048
EMP2	-10.5	2.4	epithelial membrane protein 2 (EMP2), mRNA [NM_001424]	NM_001424	Hs.655130	AK096403
LIPC	-10.4	6.8	lipase, hepatic (LIPC), mRNA [NM_000236]	NM_000236	Hs.654472	BC146659
KIAA1212	-10.4	2.1	KIAA1212 (KIAA1212), mRNA [NM_018084]	NM_018084	Hs.292925	NM_018084
ZNF395	-10.4	3.5	zinc finger protein 395 (ZNF395), mRNA [NM_018660]	NM_018660	Hs.699209	NM_018660
CR607569	-10.4	2.6	full-length cDNA clone CS0DF027YA11 of Fetal brain of (human). [CR607569]	CR607569	Hs.516075	AB209211
STARD9	-10.3	1.8	StAR-related lipid transfer protein 9 (StARD9) (START domain- containing protein 9) (Fragment). [Source:Uniprot/SWISSPROT;Acc:Q9P2P6] [ENST00000290607]	ENST0000029060	Unknown	
THC2713715	-10.3	2.9	BQ188033 UI-E-EJ1-aju-o-13-0-UI.r1 UI-E-EJ1 cDNA clone UI-E-EJ1-aju-o-13-0-UI 5', mRNA sequence [BQ188033]	THC2713715	Unknown	
NARG2	-10.3	1.8	NMDA receptor regulated 2 (NARG2), transcript variant 1, mRNA [NM_024611]	NM_024611	Hs.657694	NM_024611
EBF3	-10.3	5.0	early B-cell factor 3 (EBF3), mRNA [NM_001005463]	NM_001005463	Hs.699395	NM_001005463
BCL11A	-10.2	2.0	B-cell CLL/lymphoma 11A (zinc finger protein) (BCL11A), transcript variant 1, mRNA [NM_022893]	NM_022893	Hs.370549	NM_022893
ENST0000036	-10.2	3.0	cDNA FLJ38790 fis, clone LIVER2002842. [AK096109]	ENST0000036051	Unknown	
GAS2	-10.2	3.4	growth arrest-specific 2 (GAS2), transcript variant 1, mRNA [NM_005256]	NM_005256	Hs.632151	BC013326
VWCE	-10.2	5.0	von Willebrand factor C and EGF domains (VWCE), mRNA [NM_152718]	NM_152718	Hs.60640	NM_152718

CCDC45	-10.2	1.8	coiled-coil domain containing 45 (CCDC45), mRNA [NM_138363]	NM_138363	Hs.569713	BX641136
APOB	-10.1	7.7	apolipoprotein B (including Ag(x) antigen) (APOB), mRNA [NM_000384]	NM_000384	Hs.120759	NM_000384
APOB	-10.1	8.1	apolipoprotein B (including Ag(x) antigen) (APOB), mRNA [NM_000384]	NM_000384	Hs.120759	NM_000384
CR617018	-10.1	1.8	full-length cDNA clone CS0DG001YH13 of B cells (Ramos cell line) of (human). [CR617018]	CR617018	Hs.434075	CR607989
C1RL	-10.1	2.8	complement component 1, r subcomponent-like (C1RL), mRNA [NM_016546]	NM_016546	Hs.631730	AF178985
F2	-10.1	4.7	coagulation factor II (thrombin) (F2), mRNA [NM_000506]	NM_000506	Hs.655207	BC051332
C6orf111	-10.1	2.6	Splicing factor, arginine/serine-rich 130 (Serine-arginine-rich- splicing regulatory protein 130) (SRrp130) (SR-rich protein) (SR- related protein). [Source:Uniprot/SWISSPROT;Acc:Q8TF01] [ENST00000369239]	ENST0000036923	Unknown	
KIAA1212	-10.1	2.0	KIAA1212 (KIAA1212), mRNA [NM_018084]	NM_018084	Hs.292925	NM_018084
ZNRF1	-10.1	1.8	zinc and ring finger 1 (ZNRF1), mRNA [NM_032268]	NM_032268	Hs.427284	NM_032268
RCBTB2	-10.0	2.9	cDNA FLJ43180 fis, clone FCBBF3013846. [AK125170]	AK125170	Hs.25447	AK125170
RP11-50D16.3	-10.0	2.2	full-length cDNA clone CS0DC001YJ02 of Neuroblastoma Cot 25-normalized of (human). [CR601315]	CR601315	Hs.507783	NM_001012754
C6orf130	-10.0	2.3	chromosome 6 open reading frame 130 (C6orf130), mRNA [NM_145063]	NM_145063	Hs.227457	BQ065260
PAQR9	-10.0	3.2	progestin and adipoQ receptor family member IX (PAQR9), mRNA [NM_198504]	NM_198504	Hs.656111	NM_198504
SATB1	-10.0	2.6	special AT-rich sequence binding protein 1 (binds to nuclear matrix/scaffold-associating DNA's) (SATB1), mRNA [NM_002971]	NM_002971	Hs.517717	AK127242
AK022045	-10.0	2.5	cDNA FLJ11983 fis, clone HEMBB1001337. [AK022045]	AK022045	Hs.655386	AK024201
NAIP	-9.9	3.3	NLR family, apoptosis inhibitory protein (NAIP), transcript variant 1, mRNA [NM_004536]	NM_004536	Hs.654500	NM_004536
FAM116A	-9.9	1.9	family with sequence similarity 116, member A (FAM116A), mRNA [NM_152678]	NM_152678	Hs.91085	NM_152678
AK024516	-9.9	1.6	cDNA: FLJ20863 fis, clone ADKA01804. [AK024516]	AK024516	Hs.657178	AK024516
KIAA1212	-9.9	2.0	KIAA1212 (KIAA1212), mRNA [NM_018084]	NM_018084	Hs.292925	NM_018084
MEIS1	-9.9	2.6	Meis1, myeloid ecotropic viral integration site 1 homolog (mouse) (MEIS1), mRNA [NM_002398]	NM_002398	Hs.526754	CR749827
CNTNAP4	-9.9	21.5	cDNA FLJ30224 fis, clone BRACE2001834. [AK054786]	AK054786	Unknown	
LRRN3	-9.9	1.8	leucine rich repeat neuronal 3 (LRRN3), mRNA [NM_018334]	NM_018334	Hs.3781	NM_001099660
AX721252	-9.9	1.9	Sequence 212 from Patent WO0220754. [AX721252]	AX721252	Unknown	
THC2665933	-9.9	2.1	Q5NNJ5_ZYMMO (Q5NNJ5) DNA polymerase III delta prime subunit , partial (5%) [THC2665933]	THC2665933	Unknown	
THC2708549	-9.8	4.3	N51961 yz07b02.s1 Soares_multiple_sclerosis_2NbHMSP cDNA clone IMAGE:282315 3', mRNA sequence [N51961]	THC2708549	Unknown	
F2	-9.8	4.5	coagulation factor II (thrombin) (F2), mRNA [NM_000506]	NM_000506	Hs.655207	BC051332
DZIP3	-9.8	2.1	zinc finger DAZ interacting protein 3 (DZIP3), mRNA [NM_014648]	NM_014648	Hs.409210	BC063882
BACH2	-9.8	1.7	BTB and CNC homology 1, basic leucine zipper transcription factor 2 (BACH2), mRNA [NM_021813]	NM_021813	Hs.269764	AJ271878
ZRANB1	-9.8	2.0	Zinc finger Ran-binding domain-containing protein 1 (Protein TRABID). [Source:Uniprot/SWISSPROT;Acc:Q9UGI0] [ENST00000359653]	ENST0000035965	Unknown	
GJB2	-9.8	3.1	gap junction protein, beta 2, 26kDa (connexin 26) (GJB2), mRNA [NM_004004]	NM_004004	Hs.591234	NM_004004
RPL36A	-9.7	2.3	ribosomal protein L36a (RPL36A), mRNA [NM_021029]	NM_021029	Hs.432485	CR601778
CK820941	-9.7	4.0	ie09h05.y5 HR85 islet cDNA clone IMAGE:5086712 5', mRNA sequence [CK820941]	CK820941	Hs.596906	CK820941
HNRPA1	-9.7	3.6	heterogeneous nuclear ribonucleoprotein A1 (HNRPA1), transcript variant 2, mRNA [NM_031157]	NM_031157	Hs.699190	NM_031157
FLJ30901	-9.7	5.6	cDNA FLJ30901 fis, clone FEBRA2005778, weakly similar to INTEGUMENTARY MUCIN A.1 PRECURSOR. [AK055463]	AK055463	Unknown	
AF086261	-9.7	6.9	full length insert cDNA clone ZD42A11. [AF086261]	AF086261	Hs.58690	AK128633
KIAA1212	-9.6	2.0	KIAA1212 (KIAA1212), mRNA [NM_018084]	NM_018084	Hs.292925	NM_018084
C8orf57	-9.6	1.9	mRNA; cDNA DKFZp761D112 (from clone DKFZp761D112). [AL136588]	AL136588	Hs.492187	AL136588
THC2652700	-9.6	2.9	THC2652700	THC2652700	Unknown	
ASGR2	-9.6	4.2	asialoglycoprotein receptor 2 (ASGR2), transcript variant H2', mRNA [NM_080912]	NM_080912	Hs.654440	NM_080912
FOXF1	-9.6	9.3	forkhead box F1 (FOXF1), mRNA [NM_001451]	NM_001451	Hs.155591	NM_001451
SNCAIP	-9.6	5.1	synuclein, alpha interacting protein (synphilin) (SNCAIP), mRNA [NM_005460]	NM_005460	Hs.426463	BC040552
C20orf199	-9.6	2.4	unknown protein 2 mRNA, complete cds. [AY513723]	AY513723	Hs.356766	CR936805
THC2635591	-9.6	2.3	THC2635591	THC2635591	Unknown	
ASGR1	-9.6	3.2	asialoglycoprotein receptor 1 (ASGR1), mRNA [NM_001671]	NM_001671	Hs.12056	AK124286
KIAA1212	-9.5	2.1	KIAA1212 (KIAA1212), mRNA [NM_018084]	NM_018084	Hs.292925	NM_018084
C2orf3	-9.5	2.1	chromosome 2 open reading frame 3 (C2orf3), mRNA [NM_003203]	NM_003203	Hs.303808	NM_003203
NTF5	-9.5	1.8	neurotrophin 5 (neurotrophin 4/5) (NTF5), mRNA [NM_006179]	NM_006179	Hs.266902	NM_006179
APOM	-9.5	6.4	apolipoprotein M (APOM), mRNA [NM_019101]	NM_019101	Hs.534468	BG567934
FLJ13305	-9.5	2.2	hypothetical protein FLJ13305 (FLJ13305), mRNA [NM_032180]	NM_032180	Hs.440466	BX648834
HNRPA1	-9.4	3.4	heterogeneous nuclear ribonucleoprotein A1 (HNRPA1), transcript variant 2, mRNA [NM_031157]	NM_031157	Hs.699190	NM_031157

KIAA1155	-9.4	2.8	mRNA for KIAA1155 protein, partial cds. [AB032981]	ENST0000024422	Unknown	
BX337332	-9.4	6.2	BX337332 PLACENTA COT 25-NORMALIZED cDNA clone CS0DI040YE21 5-PRIME, mRNA sequence [BX337332]	BX337332	Hs.635297	BX337332
PSIP1	-9.4	2.5	PC4 and SFRS1 interacting protein 1 (PSIP1), transcript variant 2, mRNA [NM_033222]	NM_033222	Hs.658434	BX649155
LOC389240	-9.4	1.7	PREDICTED: similar to nascent polypeptide-associated complex alpha polypeptide (LOC389240), mRNA [XM_371715]	ENST0000024034	Unknown	
ZMIZ1	-9.4	2.7	zinc finger, MIZ-type containing 1 (ZMIZ1), mRNA [NM_020338]	NM_020338	Hs.193118	NM_020338
THC2772589	-9.3	2.2	Q5NNJ5_ZYMMO (Q5NNJ5) DNA polymerase III delta prime subunit , partial (5%) [THC2665933]	THC2772589	Unknown	
CYYR1	-9.3	3.5	cysteine/tyrosine-rich 1 (CYYR1), mRNA [NM_052954]	NM_052954	Hs.37445	NM_052954
CNTNAP4	-9.3	15.3	contactin associated protein-like 4 (CNTNAP4), transcript variant 1, mRNA [NM_033401]	NM_033401	Hs.461389	NM_033401
DMRT3	-9.3	7.4	doublesex and mab-3 related transcription factor 3 (DMRT3), mRNA [NM_021240]	NM_021240	Hs.189174	NM_021240
FOXC1	-9.3	3.2	forkhead box C1 (FOXC1), mRNA [NM_001453]	NM_001453	Hs.348883	NM_001453
PDGFRA	-9.2	8.7	platelet-derived growth factor receptor, alpha polypeptide (PDGFRA), mRNA [NM_006206]	NM_006206	Hs.74615	NM_006206
ZNF254	-9.2	3.9	zinc finger protein 254 (ZNF254), transcript variant 1, mRNA [NM_203282]	NM_203282	Hs.434406	BC043147
LOC641790	-9.2	1.7	PREDICTED: similar to ribosomal protein L31 (LOC641790), mRNA [XR_018025]	XR_018025	Hs.647529	XR_018025
KBTBD11	-9.2	2.5	kelch repeat and BTB (POZ) domain containing 11 (KBTBD11), mRNA [NM_014867]	NM_014867	Hs.5333	AB018254
F2	-9.2	4.7	coagulation factor II (thrombin) (F2), mRNA [NM_000506]	NM_000506	Hs.655207	BC051332
PCDH18	-9.2	3.7	protocadherin 18 (PCDH18), mRNA [NM_019035]	NM_019035	Hs.591691	NM_019035
C1orf54	-9.2	2.6	chromosome 1 open reading frame 54 (C1orf54), mRNA [NM_024579]	NM_024579	Hs.91283	BQ718781
F2	-9.2	4.4	coagulation factor II (thrombin) (F2), mRNA [NM_000506]	NM_000506	Hs.655207	BC051332
NPR3	-9.1	2.3	natriuretic peptide receptor C/guanylate cyclase C (atrionatriuretic peptide receptor C) (NPR3), mRNA [NM_000908]	NM_000908	Hs.237028	NM_000908
KIAA1212	-9.1	2.0	KIAA1212 (KIAA1212), mRNA [NM_018084]	NM_018084	Hs.292925	NM_018084
LOC286334	-9.1	2.4	mRNA full length insert cDNA clone EUROIMAGE 1517766. [AJ420454]	AJ420454	Hs.349208	NM_133374
BC037328	-9.1	5.4	cDNA clone IMAGE:5263455. [BC037328]	BC037328	Hs.49329	BX538299
CPS1	-9.1	6.9	carbamoyl-phosphate synthetase 1, mitochondrial (CPS1), mRNA [NM_001875]	NM_001875	Hs.149252	NM_001875
RPS15A	-9.1	2.1	ribosomal protein S15a, mRNA (cDNA clone MGC:2466 IMAGE:2967511), complete cds. [BC001697]	BC001697	Hs.370504	BM907705
RP11-78J21.1	-9.1	3.3	heterogeneous nuclear ribonucleoprotein A1-like (LOC144983), transcript variant 1, mRNA [NM_001011724]	NM_001011724	Hs.447506	AK126454
ZNF84	-9.1	2.5	zinc finger protein 84 (ZNF84), mRNA [NM_003428]	NM_003428	Hs.654730	BC036656
AK075484	-9.1	2.4	cDNA PSEC0178 fis, clone OVARC1000636, moderately similar to Sterile alpha motif domain containing protein 4. [AK075484]	AK075484	Unknown	
LOC391836	-9.0	1.9	PREDICTED: similar to ribosomal protein L10a (LOC391836), mRNA [XR_018785]	XR_018785	Hs.646677	XR_018785
AU146383	-9.0	6.7	AU146383 HEMBB1 cDNA clone HEMBB1000334 3', mRNA sequence [AU146383]	AU146383	Hs.653898	BX405503
PPIL3	-9.0	1.7	peptidylprolyl isomerase (cyclophilin)-like 3 (PPIL3), transcript variant PPIL3c, mRNA [NM_131916]	NM_131916	Hs.121076	NM_131916
ULK2	-9.0	2.0	unc-51-like kinase 2 (C. elegans) (ULK2), mRNA [NM_014683]	NM_014683	Hs.168762	AB014523
A_32_P67303	-9.0	2.7	A_32_P67303	A_32_P67303	Unknown	
A_24_P22137	-9.0	2.0	A_24_P221375	A_24_P221375	Unknown	
MPPED2	-9.0	3.4	metallophosphoesterase domain containing 2 (MPPED2), mRNA [NM_001584]	NM_001584	Hs.289795	AB209163
FANCL	-9.0	2.3	Fanconi anemia, complementation group L (FANCL), mRNA [NM_018062]	NM_018062	Hs.699478	BC037570
CPAMD8	-9.0	3.0	C3 and PZP-like, alpha-2-macroglobulin domain containing 8 (CPAMD8), mRNA [NM_015692]	NM_015692	Hs.631644	NM_015692
AK097080	-9.0	1.8	cDNA FLJ39761 fis, clone SPLEN1000083. [AK097080]	AK097080	Hs.534942	XR_017612
LOC341412	-9.0	1.9	PREDICTED: hypothetical LOC341412 (LOC341412), mRNA [XR_016541]	XR_016541	Hs.686862	CA455253
ALS2CR13	-9.0	3.2	amyotrophic lateral sclerosis 2 (juvenile) chromosome region, candidate 13 (ALS2CR13), mRNA [NM_173511]	NM_173511	Hs.471130	NM_173511
THC2668193	-9.0	3.9	THC2668193	THC2668193	Unknown	
DYNC2H1	-9.0	3.5	G protein interaction factor 1-like mRNA sequence. [AF288405]	AF288405	Hs.503721	NM_001080463
IRX5	-9.0	3.9	iroquois homeobox protein 5 (IRX5), mRNA [NM_005853]	NM_005853	Hs.435730	NM_005853
C3orf17	-8.9	1.7	chromosome 3 open reading frame 17 (C3orf17), transcript variant 1, mRNA [NM_015412]	NM_015412	Hs.591288	NM_015412
KIAA1212	-8.9	2.1	KIAA1212 (KIAA1212), mRNA [NM_018084]	NM_018084	Hs.292925	NM_018084
CREB3L4	-8.9	3.0	cAMP responsive element binding protein 3-like 4 (CREB3L4), mRNA [NM_130898]	NM_130898	Hs.372924	AY049977
LOC652558	-8.9	1.7	PREDICTED: similar to 60S ribosomal protein L7 (LOC652558), mRNA [XR_019386]	XR_019386	Hs.647657	XR_019386
AK000872	-8.8	3.2	cDNA FLJ10010 fis, clone HEMBA1000302. [AK000872]	AK000872	Hs.58690	AK128633
RP11-78J21.1	-8.8	3.6	heterogeneous nuclear ribonucleoprotein A1-like (LOC144983), transcript variant 1, mRNA [NM_001011724]	NM_001011724	Hs.447506	AK126454
A_24_P92955	-8.8	2.5	A_24_P929558	A_24_P929558	Unknown	
AASDH	-8.8	2.3	2-aminoadipic 6-semialdehyde dehydrogenase (AASDH), mRNA [NM_181806]	NM_181806	Hs.104347	BX640635

TMEM16A	-8.8	6.0	transmembrane protein 16A (TMEM16A), mRNA [NM_018043]	NM_018043	Hs.503074	AY728143
ARID2	-8.8	2.8	AT rich interactive domain 2 (ARID, RFX-like) (ARID2), mRNA [NM_152641]	NM_152641	Hs.696080	NM_152641
CCDC14	-8.8	2.0	coiled-coil domain containing 14 (CCDC14), mRNA [NM_022757]	NM_022757	Hs.656256	BX537652
NOTCH4	-8.8	3.3	Notch homolog 4 (Drosophila) (NOTCH4), mRNA [NM_004557]	NM_004557	Hs.436100	NM_004557
APOM	-8.8	6.4	apolipoprotein M (APOM), mRNA [NM_019101]	NM_019101	Hs.534468	BG567934
THC2675062	-8.8	2.4	THC2675062	THC2675062	Unknown	
ANKRD46	-8.8	2.1	ankyrin repeat domain 46 (ANKRD46), mRNA [NM_198401]	NM_198401	Hs.530199	BC035087
DET1	-8.8	2.3	de-etiolated homolog 1 (Arabidopsis) (DET1), mRNA [NM_017996]	NM_017996	Hs.567523	NM_017996
EYA1	-8.8	8.5	eyes absent homolog 1 (Drosophila) (EYA1), transcript variant 3, mRNA [NM_000503]	NM_000503	Hs.491997	NM_000503
AK026826	-8.7	2.9	cDNA: FLJ23173 fis, clone LNG10019. [AK026826]	AK026826	Hs.113157	BX647350
AF086536	-8.7	2.3	full length insert cDNA clone ZE08A03. [AF086536]	AF086536	Hs.700067	W95952
THC2719717	-8.7	5.1	THC2719717	THC2719717	Unknown	
SIX1	-8.7	5.9	sine oculis homeobox homolog 1 (Drosophila) (SIX1), mRNA [NM_005982]	NM_005982	Hs.633506	AK093780
RPS6KA5	-8.7	3.4	ribosomal protein S6 kinase, 90kDa, polypeptide 5 (RPS6KA5), transcript variant 1, mRNA [NM_004755]	NM_004755	Hs.510225	AB209667
SSBP2	-8.7	4.3	Single-stranded DNA-binding protein 2 (Sequence-specific single- stranded-DNA-binding protein 2). [Source:Uniprot/SWISSPROT;Acc:P81877] [ENST00000380186]	ENST0000038018	Unknown	
CPS1	-8.7	5.4	carbamoyl-phosphate synthetase 1, mitochondrial (CPS1), mRNA [NM_001875]	NM_001875	Hs.149252	NM_001875
IKZF4	-8.6	3.0	mRNA; cDNA DKFZp686K2231 (from clone DKFZp686K2231). [BX647761]	BX647761	Hs.553221	NM_022465
C6orf192	-8.6	3.2	chromosome 6 open reading frame 192 (C6orf192), mRNA [NM_052831]	NM_052831	Hs.347144	NM_052831
NPY2R	-8.6	11.8	neuropeptide Y receptor Y2 (NPY2R), mRNA [NM_000910]	NM_000910	Hs.37125	NM_000910
LYRM7	-8.6	1.8	Lyrm7 homolog (mouse) (LYRM7), mRNA [NM_181705]	NM_181705	Hs.115467	NM_181705
C6orf189	-8.6	7.0	chromosome 6 open reading frame 189, mRNA (cDNA clone IMAGE:6059932), partial cds. [BC038997]	BC038997	Hs.126712	BC038997
THC2544148	-8.6	2.0	Q6TXI7_RAT (Q6TXI7) LRRGT00012, partial (5%) [THC2506462]	THC2544148	Unknown	
LOC389831	-8.6	2.5	cDNA: FLJ23285 fis, clone HEP09071. [AK026938]	AK026938	Hs.389638	AL832779
IFT80	-8.6	2.0	intraflagellar transport 80 homolog (Chlamydomonas) (IFT80), mRNA [NM_020800]	NM_020800	Hs.478095	NM_020800
LOC388401	-8.6	1.7	PREDICTED: similar to 60S ribosomal protein L7 (LOC388401), mRNA [XR_016879]	XR_016879	Hs.646579	XR_016879
LOC730391	-8.5	3.6	PREDICTED: hypothetical protein LOC730391 (LOC730391), mRNA [XM_001125845]	XM_001125845	Unknown	
KIAA0528	-8.5	2.5	KIAA0528 (KIAA0528), mRNA [NM_014802]	NM_014802	Hs.271014	BX537485
ZNRF1	-8.5	1.8	zinc and ring finger 1 (ZNRF1), mRNA [NM_032268]	NM_032268	Hs.427284	NM_032268
DLEU7	-8.5	3.6	deleted in lymphocytic leukemia 7 (DLEU7) mRNA, complete cds. [AY357595]	AY357595	Hs.673860	BC035481
CBFA2T2	-8.5	1.9	core-binding factor, runt domain, alpha subunit 2; translocated to, 2 (CBFA2T2), transcript variant 2, mRNA [NM_005093]	NM_005093	Hs.153934	NM_005093
VCX2	-8.5	3.2	variable charge, X-linked 2 (VCX2), mRNA [NM_016378]	NM_016378	Hs.279737	AF167079
ENST0000030	-8.4	2.0	PREDICTED: similar to ribosomal protein L31 (LOC729005), mRNA [XM_001133428]	ENST0000030898	Unknown	
CXXC4	-8.4	2.4	CXXC finger 4 [Source:RefSeq_peptide;Acc:NP_079488] [ENST00000305749]	ENST0000030574	Unknown	
NEK3	-8.4	2.2	NIMA (never in mitosis gene a)-related kinase 3 (NEK3), transcript variant 1, mRNA [NM_002498]	NM_002498	Hs.409989	AK131359
RPL9	-8.4	2.1	ribosomal protein L9 (RPL9), transcript variant 2, mRNA [NM_001024921]	NM_001024921	Hs.412370	CR595992
A_24_P58793	-8.4	1.6	A_24_P587938	A_24_P587938	Unknown	
RP11-78J21.1	-8.4	3.3	heterogeneous nuclear ribonucleoprotein A1-like (LOC144983), transcript variant 1, mRNA [NM_001011724]	NM_001011724	Hs.447506	AK126454
THC2676284	-8.4	3.4	Q6DN14_HUMAN (Q6DN14) MCTP1L, partial (4%) [THC2676284]	THC2676284	Unknown	
CCDC98	-8.4	2.2	coiled-coil domain containing 98 (CCDC98), mRNA [NM_139076]	NM_139076	Hs.334772	NM_139076
USP34	-8.4	2.3	mRNA; cDNA DKFZp586J101 (from clone DKFZp586J101). [AL050376]	AL050376	Unknown	
ENST0000036	-8.4	1.5	dopey family member 1 (DOPEY1), mRNA [Source:RefSeq_dna;Acc:NM_015018] [ENST00000369739]	ENST0000036973	Unknown	
PDGFRA	-8.4	6.9	AA599881 ag32e07.s1 Human bone marrow stromal cells cDNA clone IMAGE:1091268 3' similar to gb:M21574 ALPHA PLATELET-DERIVED GROWTH FACTOR RECEPTOR PRECURSOR (HUMAN);, mRNA sequence [AA599881]	AA599881	Hs.74615	NM_006206
SNTG2	-8.4	3.8	syntrophin, gamma 2 (SNTG2), mRNA [NM_018968]	NM_018968	Hs.657453	AJ003029
A_24_P32422	-8.4	1.7	A_24_P324224	A_24_P324224	Unknown	
THC2631150	-8.4	3.0	THC2631150	THC2631150	Unknown	
F2	-8.3	4.8	coagulation factor II (thrombin) (F2), mRNA [NM_000506]	NM_000506	Hs.655207	BC051332
A_24_P11838	-8.3	2.2	A_24_P118382	A_24_P118382	Unknown	
AK095738	-8.3	2.6	cDNA FLJ38419 fis, clone FEBRA2009846. [AK095738]	AK095738	Hs.586950	AK095738
AB046850	-8.3	1.9	mRNA for KIAA1630 protein, partial cds. [AB046850]	AB046850	Unknown	
DCP1B	-8.3	2.0	DCP1 decapping enzyme homolog B (S. cerevisiae) (DCP1B), mRNA [NM_152640]	NM_152640	Hs.130934	NM_152640
ALKBH2	-8.3	3.1	alkB, alkylation repair homolog 2 (E. coli) (ALKBH2), mRNA [NM_001001655]	NM_001001655	Hs.374458	BG256986

KIAA1212	-8.3	2.1	KIAA1212 (KIAA1212), mRNA [NM_018084]	NM_018084	Hs.292925	NM_018084
ZNF334	-8.3	3.6	zinc finger protein 334 (ZNF334), transcript variant 2, mRNA [NM_199441]	NM_199441	Hs.584933	BC026321
THC2648398	-8.3	2.5	THC2648398	THC2648398	Unknown	
CCNB1IP1	-8.3	3.0	cyclin B1 interacting protein 1 (CCNB1IP1), transcript variant 3, mRNA [NM_182851]	NM_182851	Hs.107003	NM_182851
F2	-8.2	4.9	coagulation factor II (thrombin) (F2), mRNA [NM_000506]	NM_000506	Hs.655207	BC051332
MEIS2	-8.2	3.7	Meis1, myeloid ecotropic viral integration site 1 homolog 2 (mouse) (MEIS2), transcript variant a, mRNA [NM_170677]	NM_170677	Hs.510989	NM_170677
AK090766	-8.2	3.2	cDNA FLJ33447 fis, clone BRAMY1000098. [AK090766]	AK090766	Hs.655064	AK125793
CR601260	-8.2	4.8	full-length cDNA clone CS0DM001YA20 of Fetal liver of (human). [CR601260]	CR601260	Hs.291319	CR627122
AK123310	-8.2	2.2	cDNA FLJ41316 fis, clone BRAMY2043314. [AK123310]	AK123310	Hs.654746	AK123310
MRE11A	-8.2	1.6	MRE11 meiotic recombination 11 homolog A (S. cerevisiae) (MRE11A), transcript variant 2, mRNA [NM_005590]	NM_005590	Hs.192649	NM_005590
KIAA1706	-8.2	2.8	KIAA1706 protein (KIAA1706), mRNA [NM_030636]	NM_030636	Hs.487994	NM_030636
LOC731307	-8.2	2.8	PREDICTED: similar to Heterogeneous nuclear ribonucleoprotein A1 (Helix-destabilizing protein) (Single-strand RNA-binding protein) (hnRNP core protein A1) (HDP) (LOC731307), mRNA [XR_015746]	XR_015746	Hs.647283	XR_015746
KIAA0644	-8.2	4.4	KIAA0644 gene product (KIAA0644), mRNA [NM_014817]	NM_014817	Hs.21572	NM_014817
ZFP62	-8.2	2.1	cDNA FLJ34231 fis, clone FCBBF3025905, highly similar to Mus musculus (clone pMLZ-1) zinc finger protein (Zfp) mRNA. [AK091550]	AK091550	Hs.509227	AL832408
ZNF606	-8.2	2.6	zinc finger protein 606 (ZNF606), mRNA [NM_025027]	NM_025027	Hs.654967	AF455357
HACE1	-8.2	1.8	HECT domain and ankyrin repeat containing, E3 ubiquitin protein ligase 1 (HACE1), mRNA [NM_020771]	NM_020771	Hs.434340	AB037741
ZRANB3	-8.2	2.2	zinc finger, RAN-binding domain containing 3 (ZRANB3), mRNA [NM_032143]	NM_032143	Hs.658422	BX647838
DKFZP779L10	-8.2	1.8	cDNA clone IMAGE:5555490. [BC110326]	BC110326	Hs.440643	BC110326
MYST4	-8.1	3.1	MYST histone acetyltransferase (monocytic leukemia) 4 (MYST4), mRNA [NM_012330]	NM_012330	Hs.35758	NM_012330
LOC645277	-8.1	2.7	PREDICTED: hypothetical LOC645277 (LOC645277), mRNA [XM_928321]	XM_928321	Hs.282811	BX379759
FLJ45244	-8.1	2.7	FLJ45244 protein (FLJ45244), mRNA [NM_207443]	NM_207443	Unknown	
AF161353	-8.1	2.0	HSPC090 mRNA, partial cds. [AF161353]	AF161353	Hs.669325	AF150232
GRAMD1C	-8.1	3.1	GRAM domain containing 1C (GRAMD1C), mRNA [NM_017577]	NM_017577	Hs.24583	AL133661
AK022936	-8.1	1.9	cDNA FLJ12874 fis, clone NT2RP2003769. [AK022936]	AK022936	Hs.594424	AK022936
LOC402562	-8.1	3.9	PREDICTED: similar to Heterogeneous nuclear ribonucleoprotein A1 (Helix-destabilizing protein) (Single-strand binding protein) (hnRNP core protein A1) (HDP-1) (Topoisomerase-inhibitor suppressed) (LOC402562), mRNA [XM_208373]	XM_208373	Unknown	
F2	-8.1	4.6	coagulation factor II (thrombin) (F2), mRNA [NM_000506]	NM_000506	Hs.655207	BC051332
AF147412	-8.1	2.0	full length insert cDNA clone YP59C02. [AF147412]	AF147412	Hs.684614	AF147412
THC2541331	-8.1	2.0	HSU02032 ribosomal protein L23a {Homo sapiens} (exp=-1; wgp=0; cg=0), partial (71%) [THC2541331]	THC2541331	Unknown	
THC2660651	-8.1	2.8	Q9KBG2_BACHD (Q9KBG2) Metal-tetracycline/H+ antiporter, partial (4%) [THC2660651]	THC2660651	Unknown	
AL133577	-8.0	3.5	mRNA; cDNA DKFZp434G0972 (from clone DKFZp434G0972). [AL133577]	AL133577	Hs.656803	AL133577
AK026099	-8.0	2.4	cDNA: FLJ22446 fis, clone HRC09457. [AK026099]	AK026099	Hs.671998	AK026099
EIF3S6	-8.0	2.2	eukaryotic translation initiation factor 3, subunit 6 48kDa (EIF3S6), mRNA [NM_001568]	NM_001568	Hs.405590	AK124178
STK31	-8.0	2.2	serine/threonine kinase 31 (STK31), transcript variant 2, mRNA [NM_032944]	NM_032944	Hs.309767	BC036476
TMEM98	-8.0	2.3	transmembrane protein 98 (TMEM98), transcript variant 1, mRNA [NM_015544]	NM_015544	Hs.695982	CR617078
CCDC98	-8.0	2.2	coiled-coil domain containing 98 (CCDC98), mRNA [NM_139076]	NM_139076	Hs.334772	NM_139076
AK057720	-8.0	1.9	cDNA FLJ33158 fis, clone UTERU2000418. [AK057720]	AK057720	Hs.62314	AK057720
EDNRA	-8.0	4.3	endothelin receptor type A (EDNRA), mRNA [NM_001957]	NM_001957	Hs.183713	NM_001957
THC2656116	-8.0	3.2	THC2656116	THC2656116	Unknown	
RAMP2	-8.0	5.7	receptor (G protein-coupled) activity modifying protein 2 (RAMP2), mRNA [NM_005854]	NM_005854	Hs.514193	BX420269
GLI1	-8.0	9.2	glioma-associated oncogene homolog 1 (zinc finger protein) (GLI1), mRNA [NM_005269]	NM_005269	Hs.632702	NM_005269
ARHGAP6	-8.0	5.1	Rho GTPase activating protein 6 (ARHGAP6), transcript variant 2, mRNA [NM_001174]	NM_001174	Hs.435291	AB208792
FLJ40330	-8.0	2.4	mRNA; cDNA DKFZp686O04183 (from clone DKFZp686O04183). [BX648045]	BX648045	Hs.105323	BX537549
C20orf82	-8.0	7.1	cDNA FLJ23893 fis, clone LNG14589. [AK074473]	AK074473	Unknown	
A_24_P29823	-8.0	1.9	A_24_P298238	A_24_P298238	Unknown	
C9orf93	-7.9	3.1	chromosome 9 open reading frame 93 (C9orf93), mRNA [NM_173550]	NM_173550	Hs.17267	CR936775
LRRC36	-7.9	4.5	leucine rich repeat containing 36 (LRRC36), mRNA [NM_018296]	NM_018296	Hs.125139	NM_018296
HOXB4	-7.9	2.3	homeobox B4 (HOXB4), mRNA [NM_024015]	NM_024015	Hs.664706	NM_024015
ADNP	-7.9	2.1	activity-dependent neuroprotector (ADNP), transcript variant 1, mRNA [NM_015339]	NM_015339	Hs.570355	BC075794
TIGD4	-7.9	6.7	tigger transposable element derived 4 (TIGD4), mRNA [NM_145720]	NM_145720	Hs.301243	BC037869

SP3	-7.9	3.2	mRNA; cDNA DKFZp686N17231 (from clone DKFZp686N17231). [BX648857]	BX648857	Hs.531587	AB209334
AK023682	-7.9	3.2	cDNA FLJ13620 fis, clone PLACE1010947. [AK023682]	AK023682	Hs.593575	AK023682
AK026192	-7.9	3.4	cDNA: FLJ22539 fis, clone HRC13227. [AK026192]	AK026192	Hs.17519	AK026192
F2	-7.9	4.5	coagulation factor II (thrombin) (F2), mRNA [NM_000506]	NM_000506	Hs.655207	BC051332
A_24_P33311	-7.8	1.8	A_24_P333112	A_24_P333112	Unknown	
F2	-7.8	4.6	coagulation factor II (thrombin) (F2), mRNA [NM_000506]	NM_000506	Hs.655207	BC051332
CR610181	-7.8	3.4	full-length cDNA clone CS0DM006YA12 of Fetal liver of (human). [CR610181]	CR610181	Hs.663111	CR610181
LOC442260	-7.8	2.0	PREDICTED: similar to 60S ribosomal protein L23a (LOC442260), mRNA [XR_019361]	XR_019361	Hs.648040	XR_019361
ITPR1	-7.8	3.6	inositol 1,4,5-triphosphate receptor, type 1 (ITPR1), mRNA [NM_002222]	NM_002222	Hs.699169	NM_001099952
SNAI1	-7.8	11.4	snail homolog 1 (Drosophila) (SNAI1), mRNA [NM_005985]	NM_005985	Hs.48029	NM_005985
CNOT2	-7.8	1.8	CCR4-NOT transcription complex, subunit 2 (CNOT2), mRNA [NM_014515]	NM_014515	Hs.133350	BX641116
AK124698	-7.8	2.7	cDNA FLJ42708 fis, clone BRAMY3007311. [AK124698]	AK124698	Hs.411472	CR749843
CSPG2	-7.8	2.7	chondroitin sulfate proteoglycan 2 (versican) (CSPG2), mRNA [NM_004385]	NM_004385	Hs.695930	NM_004385
C20orf96	-7.8	3.0	chromosome 20 open reading frame 96 (C20orf96), mRNA [NM_153269]	NM_153269	Hs.348112	AK126082
RGS13	-7.8	30.5	regulator of G-protein signalling 13 (RGS13), transcript variant 1, mRNA [NM_002927]	NM_002927	Hs.497220	BC036950
FOXN4	-7.8	3.0	forkhead box N4 (FOXN4), mRNA [NM_213596]	NM_213596	Hs.528316	BC146825
KIAA1212	-7.8	2.3	KIAA1212 (KIAA1212), mRNA [NM_018084]	NM_018084	Hs.292925	NM_018084
ALG9	-7.8	1.9	asparagine-linked glycosylation 9 homolog (S. cerevisiae, alpha- 1,2-mannosyltransferase) (ALG9), transcript variant 3, mRNA [NM_001077691]	NM_001077691	Hs.503850	NM_001077691
PELI1	-7.8	2.7	pellino homolog 1 (Drosophila) (PELI1), mRNA [NM_020651]	NM_020651	Hs.7886	AF302505
PHF6	-7.7	3.0	PHD finger protein 6 (PHF6), transcript variant 2, mRNA [NM_032458]	NM_032458	Hs.356501	NM_032458
PCCA	-7.7	2.3	propionyl Coenzyme A carboxylase, alpha polypeptide (PCCA), mRNA [NM_000282]	NM_000282	Hs.80741	AL122056
ENST0000027	-7.7	2.0	PREDICTED: similar to ribosomal protein S15a (LOC391656), mRNA [XM_373027]	ENST0000027384	Unknown	
SPATA7	-7.7	2.3	spermatogenesis associated 7 (SPATA7), transcript variant 1, mRNA [NM_018418]	NM_018418	Hs.525518	BC090875
SPAG11	-7.7	8.0	sperm associated antigen 11 (SPAG11), transcript variant A, mRNA [NM_016512]	NM_016512	Hs.2717	BM563643
C6orf148	-7.7	3.4	chromosome 6 open reading frame 148 (C6orf148), mRNA [NM_030568]	NM_030568	Hs.433062	AK090984
SENP7	-7.7	3.2	SUMO1/sentrin specific peptidase 7 (SENP7), transcript variant 1, mRNA [NM_020654]	NM_020654	Hs.529551	NM_020654
ADH5	-7.7	2.0	alcohol dehydrogenase 5 (class III), chi polypeptide (ADH5), mRNA [NM_000671]	NM_000671	Hs.78989	AK226177
PTGIS	-7.7	8.8	prostaglandin I2 (prostacyclin) synthase (PTGIS), mRNA [NM_000961]	NM_000961	Hs.302085	NM_000961
AK092942	-7.7	1.8	cDNA FLJ35623 fis, clone SPLEN2010986. [AK092942]	AK092942	Hs.660700	XR_015431
C9orf102	-7.7	2.2	chromosome 9 open reading frame 102 (C9orf102), transcript variant 1, mRNA [NM_020207]	NM_020207	Hs.632686	NM_020207
THC2507152	-7.7	2.3	THC2507152	THC2507152	Unknown	
THC2679484	-7.7	2.2	THC2679484	THC2679484	Unknown	
LRRC49	-7.6	1.6	leucine rich repeat containing 49 (LRRC49), mRNA [NM_017691]	NM_017691	Hs.12692	NM_017691
PSD3	-7.6	1.9	pleckstrin and Sec7 domain containing 3 (PSD3), transcript variant 1, mRNA [NM_015310]	NM_015310	Hs.434255	NM_015310
C8orf72	-7.6	2.1	chromosome 8 open reading frame 72 (C8orf72), mRNA [NM_147189]	NM_147189	Hs.154652	NM_147189
JAG1	-7.6	1.6	jagged 1 (Alagille syndrome) (JAG1), mRNA [NM_000214]	NM_000214	Hs.224012	AF003837
CR619760	-7.6	2.2	full-length cDNA clone CS0DD006YO12 of Neuroblastoma Cot 50-normalized of (human). [CR619760]	CR619760	Hs.380930	CR619760
CCDC98	-7.6	2.1	coiled-coil domain containing 98 (CCDC98), mRNA [NM_139076]	NM_139076	Hs.334772	NM_139076
SLC25A36	-7.6	2.0	mRNA; cDNA DKFZp564C053 (from clone DKFZp564C053). [AL049246]	AL049246	Hs.144130	AL049246
POLD3	-7.6	2.1	polymerase (DNA-directed), delta 3, accessory subunit (POLD3), mRNA [NM_006591]	NM_006591	Hs.82502	NM_006591
CHD6	-7.6	1.9	chromodomain helicase DNA binding protein 6 (CHD6), mRNA [NM_032221]	NM_032221	Hs.371979	NM_032221
MTERFD2	-7.6	2.0	MTERF domain containing 2 (MTERFD2), mRNA [NM_182501]	NM_182501	Hs.159556	NM_182501
KCNAB3	-7.6	2.2	Voltage-gated potassium channel subunit beta-3 (K(+) channel subunit beta-3) (Kv-beta-3). [Source:Uniprot/SWISSPROT;Acc:O43448] [ENST00000380304]	ENST0000038030	Unknown	
RBAK	-7.6	2.2	RB-associated KRAB zinc finger (RBAK), mRNA [NM_021163]	NM_021163	Hs.592827	NM_021163
C3orf54	-7.6	3.1	chromosome 3 open reading frame 54 (C3orf54), mRNA [NM_203370]	NM_203370	Hs.86674	NM_203370
RP11-78J21.1	-7.6	3.0	heterogeneous nuclear ribonucleoprotein A1-like (LOC144983), transcript variant 1, mRNA [NM_001011724]	NM_001011724	Hs.447506	AK126454
ENST0000032	-7.6	3.3	Epithelial membrane protein 2 (EMP-2) (Protein XMP). [Source:Uniprot/SWISSPROT;Acc:P54851] [ENST00000320378]	ENST0000032037	Unknown	
A_24_P33213	-7.6	2.0	A_24_P33213	A_24_P33213	Unknown	
CCDC98	-7.6	2.2	coiled-coil domain containing 98 (CCDC98), mRNA [NM_139076]	NM_139076	Hs.334772	NM_139076
CSNK1G3	-7.6	1.8	casein kinase 1, gamma 3 (CSNK1G3), transcript variant 4, mRNA [NM_001044723]	NM_001044723	Hs.129206	NM_001044723
FLJ10159	-7.6	2.4	hypothetical protein FLJ10159 (FLJ10159), mRNA [NM_018013]	NM_018013	Hs.445244	NM_018013

RP3-377H14.5	-7.6	2.2	cDNA FLJ35429 fis, clone SMINT2002126. [AK092748]	AK092748	Hs.655949	AL832418
A_24_P26454	-7.6	2.0	A_24_P264549	A_24_P264549	Unknown	
C16orf75	-7.6	3.2	chromosome 16 open reading frame 75 (C16orf75), mRNA [NM_152308]	NM_152308	Hs.347524	BC039361
THC2651751	-7.6	2.9	THC2651751	THC2651751	Unknown	
THC2523877	-7.5	2.8	ALU6_HUMAN (P39193) Alu subfamily SP sequence contamination warning entry, partial (9%) [THC2523877]	THC2523877	Unknown	
ATF2	-7.5	2.1	cDNA FLJ46899 fis, clone UTERU3022588, highly similar to Cyclic-AMP-dependent transcription factor ATF-2. [AK128731]	AK128731	Hs.592510	BC107698
ZC3H6	-7.5	2.2	zinc finger CCCH-type containing 6 (ZC3H6), mRNA [NM_198581]	NM_198581	Hs.190477	NM_198581
FOXG1B	-7.5	17.7	forkhead box G1B (FOXG1B), mRNA [NM_005249]	NM_005249	Hs.695962	NM_005249
LOC646949	-7.5	1.7	PREDICTED: similar to ribosomal protein L23 (LOC646949), mRNA [XR_017294]	XR_017294	Hs.512542	CD556150
LGALS2	-7.5	4.6	lectin, galactoside-binding, soluble, 2 (galectin 2) (LGALS2), mRNA [NM_006498]	NM_006498	Hs.531776	AK130682
ZNF256	-7.5	2.0	zinc finger protein 256 (ZNF256), mRNA [NM_005773]	NM_005773	Hs.596242	NM_005773
CPS1	-7.5	6.6	carbamoyl-phosphate synthetase 1, mitochondrial (CPS1), mRNA [NM_001875]	NM_001875	Hs.149252	NM_001875
ZC3H6	-7.5	2.0	zinc finger CCCH-type containing 6 (ZC3H6), mRNA [NM_198581]	NM_198581	Hs.190477	NM_198581
CPS1	-7.5	7.4	carbamoyl-phosphate synthetase 1, mitochondrial (CPS1), mRNA [NM_001875]	NM_001875	Hs.149252	NM_001875
CSTF2T	-7.4	1.7	cleavage stimulation factor, 3' pre-RNA, subunit 2, 64kDa, tau variant (CSTF2T), mRNA [NM_015235]	NM_015235	Hs.591358	NM_015235
FOXP2	-7.4	7.1	forkhead box P2 (FOXP2), transcript variant 1, mRNA [NM_014491]	NM_014491	Hs.656280	CR749236
ZNF519	-7.4	3.9	zinc finger protein 519 (ZNF519), mRNA [NM_145287]	NM_145287	Hs.352635	AB209417
ASB13	-7.4	1.9	ankyrin repeat and SOCS box-containing 13 (ASB13), mRNA [NM_024701]	NM_024701	Hs.445899	BC012056
HCG12	-7.4	1.5	HLA complex group 12 (HCG12) on chromosome 6 [NR_002831]	NR_002831	Unknown	
CR616309	-7.4	2.8	full-length cDNA clone CS0DF015YK23 of Fetal brain of (human). [CR616309]	CR616309	Hs.701350	CR616309
BC030757	-7.4	2.2	cDNA clone IMAGE:4797534. [BC030757]	BC030757	Hs.683603	BC030757
CPS1	-7.4	8.1	carbamoyl-phosphate synthetase 1, mitochondrial (CPS1), mRNA [NM_001875]	NM_001875	Hs.149252	NM_001875
1-Dec	-7.4	2.3	deleted in esophageal cancer 1 (DEC1), mRNA [NM_017418]	NM_017418	Hs.148841	AK056153
RAD17	-7.4	1.6	RAD17 homolog (S. pombe) (RAD17), transcript variant 8, mRNA [NM_002873]	NM_002873	Hs.16184	AF076838
GPBP1	-7.4	1.9	GC-rich promoter binding protein 1 (GPBP1), mRNA [NM_022913]	NM_022913	Hs.444279	AL161991
NR2C1	-7.4	1.8	nuclear receptor subfamily 2, group C, member 1 (NR2C1), transcript variant 1, mRNA [NM_003297]	NM_003297	Hs.108301	BC026074
LOC137107	-7.4	1.9	PREDICTED: similar to ribosomal protein L10a (LOC137107), mRNA [XM_070233]	XM_070233	Unknown	
RBM25	-7.3	1.8	RNA binding motif protein 25 (RBM25), mRNA [NM_021239]	NM_021239	Hs.531106	BX647116
BC047111	-7.3	2.3	cDNA clone IMAGE:5314178. [BC047111]	BC047111	Hs.595378	BC047111
CCDC45	-7.3	2.0	coiled-coil domain containing 45 (CCDC45), mRNA [NM_138363]	NM_138363	Hs.569713	BX641136
MGC27345	-7.3	2.1	hypothetical protein MGC27345, mRNA (cDNA clone MGC:27345 IMAGE:4670552), complete cds. [BC024231]	BC024231	Hs.696551	AK001384
DHFRL1	-7.3	1.9	dihydrofolate reductase-like 1 (DHFRL1), mRNA [NM_176815]	NM_176815	Hs.448003	BC045541
CENPC1	-7.3	2.1	centromere protein C 1 (CENPC1), mRNA [NM_001812]	NM_001812	Hs.479867	BC030695
GPAM	-7.3	2.6	glycerol-3-phosphate acyltransferase, mitochondrial (GPAM), mRNA [NM_020918]	NM_020918	Hs.42586	AL833061
THC2626557	-7.3	2.5	THC2626557	THC2626557	Unknown	
THC2771474	-7.3	2.4	Q8NFF8_HUMAN (Q8NFF8) MLL5, partial (3%) [THC2771474]	THC2771474	Unknown	
LOC440905	-7.3	3.0	PREDICTED: hypothetical protein LOC440905, transcript variant 4 (LOC440905), mRNA [XM_943718]	XM_943718	Unknown	
HNRPA3	-7.3	2.3	heterogeneous nuclear ribonucleoprotein A3 (HNRPA3), mRNA [NM_194247]	NM_194247	Hs.516539	NM_194247
LOC400027	-7.3	1.9	cDNA clone IMAGE:5288894. [BC047417]	BC047417	Hs.597122	BC047417
FOXD2	-7.3	9.3	forkhead box D2 (FOXD2), mRNA [NM_004474]	NM_004474	Hs.166188	NM_004474
NACAL	-7.3	1.7	nascent-polypeptide-associated complex alpha polypeptide-like (NACAL), mRNA [NM_199290]	NM_199290	Hs.591178	BU567791
C9orf45	-7.3	2.0	cDNA FLJ41850 fis, clone NT2RI3003738, highly similar to GL012 mRNA. [AK123844]	AK123844	Hs.657064	CR749230
THC2545558	-7.3	2.8	Q9F8M7_CARHY (Q9F8M7) DTDP-glucose 4,6-dehydratase (Fragment), partial (11%) [THC2545558]	THC2545558	Unknown	
LOC400027	-7.3	2.1	PREDICTED: hypothetical gene supported by BC047417, transcript variant 2 (LOC400027), mRNA [XM_931434]	XM_931434	Unknown	
MEIS3P1	-7.3	1.9	Meis1 homolog 3 (mouse) pseudogene 1 (MEIS3P1) on chromosome 17 [NR_002211]	NR_002211	Unknown	
MUSTN1	-7.3	2.7	musculoskeletal, embryonic nuclear protein 1 (MUSTN1), mRNA [NM_205853]	NM_205853	Hs.699481	BQ773442
KLB	-7.3	3.6	Beta klotho (BetaKlotho) (Klotho beta-like protein). [Source:Uniprot/SWISSPROT;Acc:Q86Z14] [ENST00000381889]	ENST0000038188	Unknown	
SMARCE1	-7.3	1.9	SWI/SNF related, matrix associated, actin dependent regulator of chromatin, subfamily e, member 1 (SMARCE1), mRNA [NM_003079]	NM_003079	Hs.696086	BC069196
CR617560	-7.3	2.3	full-length cDNA clone CS0DC013YG14 of Neuroblastoma Cot 25-normalized of (human). [CR617560]	CR617560	Hs.463010	BC005066
AK057710	-7.3	2.3	cDNA FLJ33148 fis, clone UTERU2000238. [AK057710]	AK057710	Hs.597376	AK057710
CCDC98	-7.3	2.1	coiled-coil domain containing 98 (CCDC98), mRNA [NM_139076]	NM_139076	Hs.334772	NM_139076

SLCO1B3	-7.3	3.1	solute carrier organic anion transporter family, member 1B3 (SLCO1B3), mRNA [NM_019844]	NM_019844	Hs.504966	NM_019844
ANKS6	-7.3	2.1	ankyrin repeat and sterile alpha motif domain containing 6 (ANKS6), mRNA [NM_173551]	NM_173551	Hs.406890	NM_173551
CPS1	-7.3	6.5	carbamoyl-phosphate synthetase 1, mitochondrial (CPS1), mRNA [NM_001875]	NM_001875	Hs.149252	NM_001875
MSL2L1	-7.3	2.5	male-specific lethal 2-like 1 (Drosophila) (MSL2L1), mRNA [NM_018133]	NM_018133	Hs.18631	NM_018133
PELI2	-7.2	2.2	pellino homolog 2 (Drosophila) (PELI2), mRNA [NM_021255]	NM_021255	Hs.657926	NM_021255
LEF1	-7.2	3.0	lymphoid enhancer-binding factor 1 (LEF1), mRNA [NM_016269]	NM_016269	Hs.555947	AK128255
RPL37	-7.2	1.8	ribosomal protein L37 (RPL37), mRNA [NM_000997]	NM_000997	Hs.80545	AL137450
TRERF1	-7.2	4.4	Transcriptional-regulating factor 1 (Transcriptional-regulating protein 132) (Zinc finger transcription factor TReP-132) (Zinc finger protein rapa). [Source:Uniprot/SWISSPROT;Acc:Q96PN7] [ENST00000372922]	ENST0000037292	Unknown	
CDCA7	-7.2	4.2	cell division cycle associated 7 (CDCA7), transcript variant 1, mRNA [NM_031942]	NM_031942	Hs.470654	AL834186
HEY2	-7.2	6.1	hairy/enhancer-of-split related with YRPW motif 2 (HEY2), mRNA [NM_012259]	NM_012259	Hs.144287	NM_012259
GJB2	-7.2	3.3	gap junction protein, beta 2, 26kDa (connexin 26) (GJB2), mRNA [NM_004004]	NM_004004	Hs.591234	NM_004004
AK124299	-7.2	1.8	cDNA FLJ42306 fis, clone TRACH2001646. [AK124299]	AK124299	Hs.130036	AK124299
APOA2	-7.2	2.8	apolipoprotein A-II (APOA2), mRNA [NM_001643]	NM_001643	Hs.237658	CR619946
SPATA5	-7.2	2.4	mRNA; cDNA DKFZp686D13227 (from clone DKFZp686D13227) [BX641009]	BX641009	Hs.689178	BX641009
IRX3	-7.2	7.1	iroquois homeobox protein 3 (IRX3), mRNA [NM_024336]	NM_024336	Hs.499205	AY335943
AK056624	-7.2	3.9	cDNA FLJ32062 fis, clone OCBBF1000042. [AK056624]	AK056624	Hs.647082	AF459743
SP5	-7.2	6.7	Sp5 transcription factor (SP5), mRNA [NM_001003845]	NM_001003845	Hs.368802	AB096175
RPL6	-7.2	1.8	ribosomal protein L6 (RPL6), transcript variant 1, mRNA [NM_001024662]	NM_001024662	Hs.546283	BQ055135
Gcom1	-7.2	2.3	GRINL1A combined protein (Gcom1), transcript variant 1, mRNA [NM_001018090]	NM_001018090	Hs.437256	NM_001018090
THC2668359	-7.2	3.6	THC2668359	THC2668359	Unknown	
AF150379	-7.2	3.5	AF150379 Human mRNA from cd34+ stem cells cDNA clone CBMAJE07, mRNA sequence [AF150379]	AF150379	Hs.568999	AF150379
ALDH1A2	-7.2	9.8	aldehyde dehydrogenase 1 family, member A2 (ALDH1A2), transcript variant 3, mRNA [NM_170697]	NM_170697	Hs.699620	AK128709
CASD1	-7.2	3.0	CAS1 domain containing 1 (CASD1), mRNA [NM_022900]	NM_022900	Hs.260041	BC063284
HLA-DOA	-7.2	7.2	major histocompatibility complex, class II, DO alpha (HLA-DOA), mRNA [NM_002119]	NM_002119	Hs.631991	NM_002119
CDCA7	-7.2	4.5	cell division cycle associated 7 (CDCA7), transcript variant 1, mRNA [NM_031942]	NM_031942	Hs.470654	AL834186
RPS25	-7.2	1.6	ribosomal protein S25 (RPS25), mRNA [NM_001028]	NM_001028	Hs.512676	BM554735
VCX3A	-7.2	3.1	variable charge, X-linked 3A (VCX3A), mRNA [NM_016379]	NM_016379	Hs.278906	AF167078
A_24_P55551	-7.2	2.2	A_24_P555510	A_24_P555510	Unknown	
RFTN2	-7.1	3.3	raftlin family member 2 (RFTN2), mRNA [NM_144629]	NM_144629	Hs.591615	BC111069
C11orf1	-7.1	2.8	chromosome 11 open reading frame 1 (C11orf1), mRNA [NM_022761]	NM_022761	Hs.17546	BC036892
CCDC98	-7.1	2.1	coiled-coil domain containing 98 (CCDC98), mRNA [NM_139076]	NM_139076	Hs.334772	NM_139076
ZRANB3	-7.1	2.0	zinc finger, RAN-binding domain containing 3 (ZRANB3), mRNA [NM_032143]	NM_032143	Hs.658422	BX647838
RSF1	-7.1	1.7	remodeling and spacing factor 1 (RSF1), mRNA [NM_016578]	NM_016578	Hs.420229	AF227948
AK095167	-7.1	2.2	cDNA FLJ37848 fis, clone BRSSN2013544. [AK095167]	AK095167	Hs.587264	AK094546
CPS1	-7.1	6.8	carbamoyl-phosphate synthetase 1, mitochondrial (CPS1), mRNA [NM_001875]	NM_001875	Hs.149252	NM_001875
RPL21	-7.1	1.7	ribosomal protein L21, mRNA (cDNA clone IMAGE:6605832), complete cds. [BC104478]	BC104478	Hs.693999	AV759013
ZNF624	-7.1	2.4	zinc finger protein 624 (ZNF624), mRNA [NM_020787]	NM_020787	Hs.128078	AK131401
ENST0000030	-7.1	2.8	KCTD19 protein. [Source:Uniprot/SPTREMBL;Acc:Q17RG1] [ENST00000304372]	ENST0000030437	Unknown	
CTNNA2	-7.1	2.7	catenin (cadherin-associated protein), alpha 2 (CTNNA2), mRNA [NM_004389]	NM_004389	Hs.167368	AK127226
BC037838	-7.1	2.5	cDNA clone IMAGE:4813920. [BC037838]	BC037838	Hs.594876	AK025546
KIAA1713	-7.1	2.5	mRNA for KIAA1713 protein, partial cds. [AB051500]	AB051500	Hs.464876	NM_030632
C6orf111	-7.1	2.2	chromosome 6 open reading frame 111 (C6orf111), mRNA [NM_032870]	NM_032870	Hs.520287	NM_032870
FADS1	-7.1	2.5	fatty acid desaturase 1 (FADS1), mRNA [NM_013402]	NM_013402	Hs.503546	NM_013402
LCP2	-7.1	3.5	lymphocyte cytosolic protein 2 (SH2 domain containing leukocyte protein of 76kDa) (LCP2), mRNA [NM_005565]	NM_005565	Hs.304475	NM_005565
THC2700133	-7.1	3.8	THC2700133	THC2700133	Unknown	
AF275804	-7.1	2.5	PNAS-108 mRNA, partial sequence. [AF275804]	AF275804	Hs.237396	BQ431041
ENST0000035	-7.1	2.8	mRNA; cDNA DKFZp434L187 (from clone DKFZp434L187); partial cds. [AL117445]	ENST0000035477	Unknown	
C4orf31	-7.1	4.0	chromosome 4 open reading frame 31 (C4orf31), mRNA [NM_024574]	NM_024574	Hs.90250	NM_024574
A_24_P49860	-7.1	3.6	A_24_P49860	A_24_P49860	Unknown	
FAM3B	-7.1	1.7	family with sequence similarity 3, member B (FAM3B), transcript variant 1, mRNA [NM_058186]	NM_058186	Hs.473877	BC036314
A_23_P20095	-7.0	2.0	A_23_P200955	A_23_P200955	Unknown	

ZNF292	-7.0	2.3	Zinc finger protein 292. [Source:Uniprot/SWISSPROT;Acc:O60281] [ENST00000339907]	ENST0000033990	Unknown	
P2RY5	-7.0	6.7	purinergic receptor P2Y, G-protein coupled, 5 (P2RY5), mRNA [NM_005767]	NM_005767	Hs.123464	BC045651
C20orf19	-7.0	2.4	chromosome 20 open reading frame 19, mRNA (cDNA clone IMAGE:5261585), complete cds. [BC039296]	BC039296	Hs.187635	BC039296
HECTD2	-7.0	1.7	HECT domain containing 2 (HECTD2), transcript variant 2, mRNA [NM_173497]	NM_173497	Hs.656960	BC040187
LOC283904	-7.0	4.9	full-length cDNA clone CS0DK001YK13 of HeLa cells Cot 25-normalized of (human). [CR605298]	CR605298	Hs.676511	CR605298
BC048201	-7.0	8.0	Homo sapiens, clone IMAGE:3660074, mRNA. [BC048201]	BC048201	Hs.558872	BC070147
CR624880	-7.0	1.6	full-length cDNA clone CS0DB005YI23 of Neuroblastoma Cot 10-normalized of (human). [CR624880]	CR624880	Hs.505983	AK056140
HOXB3	-7.0	2.9	homeobox B3 (HOXB3), mRNA [NM_002146]	NM_002146	Hs.654560	NM_002146
ZNF518	-7.0	3.3	zinc finger protein 518 (ZNF518), mRNA [NM_014803]	NM_014803	Hs.657337	NM_014803
WDR54	-7.0	2.0	WD repeat domain 54 (WDR54), mRNA [NM_032118]	NM_032118	Hs.643480	BM542003
CCDC98	-7.0	2.1	coiled-coil domain containing 98 (CCDC98), mRNA [NM_139076]	NM_139076	Hs.334772	NM_139076
C14orf167	-7.0	2.5	chromosome 14 open reading frame 167, mRNA (cDNA clone IMAGE:4706815), partial cds. [BC013143]	BC013143	Hs.601265	BC062348
BC082970	-7.0	1.8	cDNA clone IMAGE:6598034. [BC082970]	BC082970	Hs.329266	NM_005338
CRSP6	-7.0	2.5	cDNA FLJ12094 fis, clone HEMBB1002607, highly similar to vitamin D3 receptor interacting protein (DRIP80) mRNA. [AK022156]	AK022156	Unknown	
IRX5	-7.0	2.3	iroquois homeobox protein 5 (IRX5), mRNA [NM_005853]	NM_005853	Hs.435730	NM_005853
MYCN	-7.0	7.3	v-myc myelocytomatosis viral related oncogene, neuroblastoma derived (avian) (MYCN), mRNA [NM_005378]	NM_005378	Hs.25960	NM_005378
LOC401725	-7.0	1.7	PREDICTED: similar to 60S ribosomal protein L6 (TAX-responsive enhancer element-binding protein 107) (TAXREB107) (Neoplasm-related protein C140) (LOC401725), mRNA [XR_019536]	XR_019536	Hs.646417	XR_019536
POT1	-7.0	1.9	POT1 protection of telomeres 1 homolog (S. pombe) (POT1), transcript variant 1, mRNA [NM_015450]	NM_015450	Hs.31968	NM_015450
LTA4H	-7.0	1.7	leukotriene A4 hydrolase (LTA4H), mRNA [NM_000895]	NM_000895	Hs.524648	AB209740
NRIP1	-7.0	3.3	nuclear receptor interacting protein 1 (NRIP1), mRNA [NM_003489]	NM_003489	Hs.155017	NM_003489
RPS15A	-7.0	1.9	ribosomal protein S15a (RPS15A), transcript variant 2, mRNA [NM_001019]	NM_001019	Hs.370504	BM907705
CBLB	-7.0	1.7	Cas-Br-M (murine) ecotropic retroviral transforming sequence b (CBLB), mRNA [NM_170662]	NM_170662	Hs.430589	BX537484
THC2586367	-7.0	2.2	Q9D7P1_MOUSE (Q9D7P1) Adult male tongue cDNA, RIKEN full-length enriched library, clone:2300009C10 product:ribosomal protein S24, full insert sequence, partial (95%) [THC2586367]	THC2586367	Unknown	
RP11-564C4.1	-7.0	3.4	mRNA; cDNA DKFZp779G2222 (from clone DKFZp779G2222). [CR749831]	CR749831	Hs.282795	CR749831
CDH11	-7.0	2.1	cadherin 11, type 2, OB-cadherin (osteoblast) (CDH11), mRNA [NM_001797]	NM_001797	Hs.116471	D21255
ENST0000035	-7.0	2.1	Protein AD-013. [Source:Uniprot/SWISSPROT;Acc:Q9UHM2] [ENST00000355629]	ENST0000035562	Unknown	
LOC284033	-7.0	2.5	cDNA FLJ37733 fis, clone BRHIP2020827. [AK095052]	AK095052	Hs.592124	AK095052
THC2609205	-7.0	1.9	THC2609205	THC2609205	Unknown	
PFAAP5	-6.9	2.0	phosphonoformate immuno-associated protein 5 (PFAAP5), mRNA [NM_014887]	NM_014887	Hs.507680	AL049783
A_24_P21337	-6.9	2.3	A_24_P213375	A_24_P213375	Unknown	
LOC391655	-6.9	1.7	PREDICTED: similar to 60S ribosomal protein L17 (L23) (LOC391655), mRNA [XR_018405]	XR_018405	Hs.646313	XR_018405
MAGI2	-6.9	2.2	membrane associated guanylate kinase, WW and PDZ domain containing 2 (MAGI2), mRNA [NM_012301]	NM_012301	Hs.654788	NM_012301
ZNF30	-6.9	1.7	zinc finger protein 30 (ZNF30), mRNA [NM_194325]	NM_194325	Hs.657402	BX640666
ENST0000033	-6.9	14.5	CDNA FLJ41026 fis, clone BRAMY2004771, weakly similar to CHONDROADHERIN. [Source:Uniprot/SPTREMBL;Acc:Q6ZWI5] [ENST00000334994]	ENST0000033499	Unknown	
C13orf7	-6.9	1.9	chromosome 13 open reading frame 7 (C13orf7), mRNA [NM_024546]	NM_024546	Hs.567576	CR936752
LOC441743	-6.9	2.0	similar to C367G8.3 (novel protein similar to RPL23A (60S ribosomal protein L23A)) (LOC441743), mRNA [NM_001045548]	NM_001045548	Unknown	
A_24_P50489	-6.9	1.9	A_24_P50489	A_24_P50489	Unknown	
ENST0000031	-6.9	2.8	Protein kinase C-binding protein 1 (Rack7) (Cutaneous T-cell lymphoma- associated antigen se14-3) (CTCL tumor antigen se14-3) (Zinc finger MYND domain-containing protein 8). [Source:Uniprot/SWISSPROT;Acc:Q9ULU4] [ENST00000311275]	ENST0000031127	Unknown	
DHDH	-6.9	2.6	dihydrodiol dehydrogenase (dimeric) (DHDH), mRNA [NM_014475]	NM_014475	Hs.631555	BM454993
ZNF518	-6.9	2.5	zinc finger protein 518 (ZNF518), mRNA [NM_014803]	NM_014803	Hs.657337	NM_014803
RPL24	-6.9	1.9	ribosomal protein L24 (RPL24), mRNA [NM_000986]	NM_000986	Hs.477028	CR608385
AK021543	-6.9	4.7	cDNA FLJ11481 fis, clone HEMBA1001803. [AK021543]	AK021543	Hs.584880	AK021543
BU956542	-6.9	1.7	BU956542 AGENCOURT_10615527 NIH_MGC_107 cDNA clone IMAGE:6730153 5', mRNA sequence [BU956542]	BU956542	Hs.448879	BQ055284
AL133090	-6.9	2.0	mRNA; cDNA DKFZp434E0528 (from clone DKFZp434E0528). [AL133090]	AL133090	Hs.592567	AL133090
SR140	-6.9	3.5	Human mRNA for KIAA0332 gene, partial cds. [AB002330]	AB002330	Hs.596572	NM_001080415
LMO2	-6.9	5.3	LIM domain only 2 (rhombotin-like 1) (LMO2), mRNA [NM_005574]	NM_005574	Hs.34560	NM_005574
ZNF187	-6.9	1.8	zinc finger protein 187 (ZNF187), transcript variant 1, mRNA [NM_007151]	NM_007151	Hs.157883	NM_007151

CCDC98	-6.9	2.1	coiled-coil domain containing 98 (CCDC98), mRNA [NM_139076]	NM_139076	Hs.334772	NM_139076
ZMYM2	-6.9	2.7	mRNA; cDNA DKFZp564B162 (from clone DKFZp564B162). [AL136621]	AL136621	Hs.644041	BX648905
OR7E104P	-6.9	3.1	cDNA FLJ46084 fis, clone TESTI2006543. [AK127969]	AK127969	Hs.568153	AK127969
ROBO2	-6.9	3.8	cDNA FLJ90299 fis, clone NT2RP2000514, highly similar to roundabout 2 (robo2) mRNA. [AK074780]	AK074780	Hs.13305	BX648828
PHIP	-6.9	2.2	pleckstrin homology domain interacting protein (PHIP), mRNA [NM_017934]	NM_017934	Hs.511817	NM_017934
LOC652890	-6.9	1.7	PREDICTED: similar to 60S ribosomal protein L7 (LOC652890), mRNA [XR_019544]	XR_019544	Hs.648276	XR_019544
BPTF	-6.9	1.9	bromodomain PHD finger transcription factor (BPTF), transcript variant 1, mRNA [NM_182641]	NM_182641	Hs.444200	NM_182641
CR619772	-6.9	3.0	full-length cDNA clone CS0DI044YN21 of Placenta Cot 25-normalized of (human). [CR619772]	CR619772	Hs.518326	AK125413
BX648950	-6.9	1.7	mRNA; cDNA DKFZp686E1648 (from clone DKFZp686E1648). [BX648950]	BX648950	Hs.563560	BX640652
KIAA1984	-6.9	2.9	mRNA for KIAA1984 protein. [AB075864]	AB075864	Hs.370555	AB075864
RPL31	-6.9	1.6	ribosomal protein L31 (RPL31), mRNA [NM_000993]	NM_000993	Hs.469473	CR595074
PCDH17	-6.9	2.3	protocadherin 17 (PCDH17), mRNA [NM_001040429]	NM_001040429	Hs.106511	NM_001040429
RPS15A	-6.9	1.9	ribosomal protein S15a (RPS15A), transcript variant 2, mRNA [NM_001019]	NM_001019	Hs.370504	BM907705
LOC286272	-6.8	2.9	cDNA FLJ10077 fis, clone HEMBA1001864. [AK000939]	AK000939	Hs.657301	AK000939
KIAA1430	-6.8	1.7	mRNA for KIAA1430 protein, partial cds. [AB037851]	AB037851	Hs.535734	NM_020827
THC2538822	-6.8	2.4	THC2538822	THC2538822	Unknown	
CR597846	-6.8	2.3	full-length cDNA clone CS0DC012YL18 of Neuroblastoma Cot 25-normalized of (human). [CR597846]	CR597846	Hs.628886	CR597846
LOC283666	-6.8	2.4	hypothetical protein LOC283666, mRNA (cDNA clone IMAGE:4415549), partial cds. [BC048264]	BC048264	Hs.560343	AL832164
THC2631465	-6.8	4.5	THC2631465	THC2631465	Unknown	
BU608568	-6.8	1.6	UI-CF-FN0-aep-h-11-0-UI.s1 UI-CF-FN0 cDNA clone UI-CF-FN0-aep-h-11-0-UI 3', mRNA sequence [BU608568]	BU608568	Hs.621731	BU608568
ACVR2B	-6.8	2.9	activin A receptor, type IIB (ACVR2B), mRNA [NM_001106]	NM_001106	Hs.174273	NM_001106
CSRP2BP	-6.8	2.3	CSRP2 binding protein (CSRP2BP), transcript variant 1, mRNA [NM_020536]	NM_020536	Hs.488051	NM_020536
DOCK1	-6.8	2.8	dedicator of cytokinesis 1 (DOCK1), mRNA [NM_001380]	NM_001380	Hs.645702	NM_001380
LMO2	-6.8	5.5	LIM domain only 2 (rhombotin-like 1) (LMO2), mRNA [NM_005574]	NM_005574	Hs.34560	NM_005574
CCDC98	-6.8	2.1	coiled-coil domain containing 98 (CCDC98), mRNA [NM_139076]	NM_139076	Hs.334772	NM_139076
ODZ4	-6.8	2.8	mRNA for KIAA1302 protein, partial cds. [AB037723]	AB037723	Hs.213087	NM_001098816
ZNF226	-6.8	2.0	zinc finger protein 226 (ZNF226), transcript variant 1, mRNA [NM_001032372]	NM_001032372	Hs.145956	BX648775
DLGAP1	-6.8	4.0	discs, large (Drosophila) homolog-associated protein 1 (DLGAP1), transcript variant alpha, mRNA [NM_004746]	NM_004746	Hs.654793	NM_004746
PURG	-6.8	2.6	purine-rich element binding protein G (PURG), transcript variant A, mRNA [NM_013357]	NM_013357	Hs.373778	AF195513
TMEM106B	-6.8	1.8	transmembrane protein 106B (TMEM106B), mRNA [NM_018374]	NM_018374	Hs.396358	NM_018374
DST	-6.8	1.8	cDNA: FLJ21489 fis, clone COL05450. [AK025142]	AK025142	Unknown	
THC2657781	-6.8	2.8	THC2657781	THC2657781	Unknown	
THC2679528	-6.8	2.6	THC2679528	THC2679528	Unknown	
SOSTDC1	-6.8	2.1	sclerostin domain containing 1 (SOSTDC1), mRNA [NM_015464]	NM_015464	Hs.648106	AK093408
COX6B2	-6.8	6.5	cytochrome c oxidase subunit VIb polypeptide 2 (testis) (COX6B2), mRNA [NM_144613]	NM_144613	Hs.550544	AK057427
CROP	-6.8	1.8	cisplatin resistance-associated overexpressed protein (CROP), transcript variant 1, mRNA [NM_016424]	NM_016424	Hs.130293	NM_016424
HGF	-6.8	6.5	hepatocyte growth factor (hepapoietin A; scatter factor) (HGF), transcript variant 2, mRNA [NM_001010931]	NM_001010931	Hs.396530	NM_000601
CEP70	-6.8	2.3	centrosomal protein 70kDa (CEP70), mRNA [NM_024491]	NM_024491	Hs.531962	NM_024491
C20orf177	-6.8	1.6	Uncharacterized protein C20orf177. [Source:Uniprot/SWISSPROT;Acc:Q9NTX9] [ENST00000358293]	ENST0000035829	Unknown	
HNRPA3	-6.8	2.8	heterogeneous nuclear ribonucleoprotein A3 (HNRPA3), mRNA [NM_194247]	NM_194247	Hs.516539	NM_194247
LMO2	-6.7	5.5	LIM domain only 2 (rhombotin-like 1) (LMO2), mRNA [NM_005574]	NM_005574	Hs.34560	NM_005574
THC2698177	-6.7	2.6	Q99PF7_CRIGR (Q99PF7) Ribosomal protein S28 (Fragment), partial (32%) [THC2698177]	THC2698177	Unknown	
DYNC2H1	-6.7	2.8	dynein, cytoplasmic 2, heavy chain 1, mRNA (cDNA clone IMAGE:5265846), complete cds. [BC037496]	BC037496	Hs.503721	NM_001080463
ALDH5A1	-6.7	1.9	aldehyde dehydrogenase 5 family, member A1 (succinate-semialdehyde dehydrogenase) (ALDH5A1), nuclear gene encoding mitochondrial protein, transcript variant 1, mRNA [NM_170740]	NM_170740	Hs.371723	NM_170740
TWIST2	-6.7	3.4	twist homolog 2 (Drosophila) (TWIST2), mRNA [NM_057179]	NM_057179	Hs.590904	BC017907
S79672	-6.7	2.2	EWS/WT1 fusion protein (EWS1/WT1) mRNA, partial cds. [S79672]	S79672	Unknown	
RP11-78J21.1	-6.7	2.4	heterogeneous nuclear ribonucleoprotein A1-like (LOC144983), transcript variant 1, mRNA [NM_001011724]	NM_001011724	Hs.447506	AK126454
VPS13B	-6.7	1.9	vacuolar protein sorting 13 homolog B (yeast) (VPS13B), transcript variant 5, mRNA [NM_017890]	NM_017890	Hs.191540	NM_017890
THC2641682	-6.7	3.4	Q2H7G5_CHAGB (Q2H7G5) Predicted protein, partial (5%) [THC2641682]	THC2641682	Unknown	

BQ897248	-6.7	3.1	AGENCOURT_8122036 Lupski_dorsal_root_ganglion cDNA clone IMAGE:6179261 5', mRNA sequence [BQ897248]	BQ897248	Hs.69297	BQ897248
LOC390413	-6.7	1.9	PREDICTED: similar to 60S ribosomal protein L7 (LOC390413), mRNA [XR_018341]	XR_018341	Hs.646625	XR_018341
LOC343495	-6.7	2.5	PREDICTED: similar to 60S ribosomal protein L6 (TAX-responsive enhancer element-binding protein 107) (TAXREB107) (Neoplasm-related protein C140) (LOC343495), mRNA [XR_016540]	XR_016540	Hs.682141	XR_018840
MPDZ	-6.7	1.7	multiple PDZ domain protein (MPDZ), mRNA [NM_003829]	NM_003829	Hs.169378	AB210041
RPL23A	-6.7	1.8	ribosomal protein L23a (RPL23A), mRNA [NM_000984]	NM_000984	Hs.419463	CR616046
ZC3H8	-6.7	1.7	zinc finger CCCH-type containing 8 (ZC3H8), mRNA [NM_032494]	NM_032494	Hs.418416	BC032001
ATXN7L1	-6.7	2.3	mRNA for KIAA1218 protein, partial cds. [AB033044]	AB033044	Hs.489603	AB033044
SYNE2	-6.7	2.9	spectrin repeat containing, nuclear envelope 2 (SYNE2), transcript variant 1, mRNA [NM_015180]	NM_015180	Hs.525392	NM_182914
TBC1D8	-6.7	4.7	cDNA FLJ40805 fis, clone TRACH2009060. [AK098124]	AK098124	Unknown	
PCMTD2	-6.7	2.4	protein-L-isoaspartate (D-aspartate) O-methyltransferase domain containing 2 (PCMTD2), mRNA [NM_018257]	NM_018257	Hs.473317	AK001745
NFATC1	-6.7	3.3	nuclear factor of activated T-cells, cytoplasmic, calcineurin-dependent 1 (NFATC1), transcript variant 3, mRNA [NM_172387]	NM_172387	Hs.534074	NM_006162
PNPLA4	-6.7	1.9	patatin-like phospholipase domain containing 4 (PNPLA4), mRNA [NM_004650]	NM_004650	Hs.264	NM_004650
CR615245	-6.7	1.8	full-length cDNA clone CL0BB022ZD10 of Neuroblastoma of (human). [CR615245]	CR615245	Hs.648444	AK092544
THC2730823	-6.7	2.2	O19057_PONPY (O19057) Fertilin alpha protein, partial (19%) [THC2730823]	THC2730823	Unknown	
A_32_P14438	-6.7	2.3	A_32_P144381	A_32_P144381	Unknown	
AUH	-6.7	1.9	AU RNA binding protein/enoyl-Coenzyme A hydratase (AUH), nuclear gene encoding mitochondrial protein, mRNA [NM_001698]	NM_001698	Hs.175905	AK124142
GNG2	-6.7	2.5	guanine nucleotide binding protein (G protein), gamma 2 (GNG2), mRNA [NM_053064]	NM_053064	Hs.695989	NM_053064
RECK	-6.7	2.6	reversion-inducing-cysteine-rich protein with kazal motifs (RECK), mRNA [NM_021111]	NM_021111	Hs.388918	BX648668
LOC401863	-6.7	1.9	PREDICTED: similar to ribosomal protein L10a (LOC401863), mRNA [XR_019597]	XR_019597	Hs.647361	XR_019597
CCDC121	-6.7	1.7	coiled-coil domain containing 121 (CCDC121), mRNA [NM_024584]	NM_024584	Hs.21081	AK125354
AK025975	-6.6	3.5	cDNA: FLJ22322 fis, clone HRC05532. [AK025975]	AK025975	Hs.380705	AK125423
SIN3A	-6.6	1.7	SIN3 homolog A, transcription regulator (yeast) (SIN3A), mRNA [NM_015477]	NM_015477	Hs.513039	NM_015477
C20orf12	-6.6	2.3	chromosome 20 open reading frame 12 (C20orf12), mRNA [NM_018152]	NM_018152	Unknown	
A_24_P38441	-6.6	2.1	A_24_P384411	A_24_P384411	Unknown	
CR610468	-6.6	2.8	full-length cDNA clone CS0DF029YL18 of Fetal brain of (human). [CR610468]	CR610468	Hs.563205	NM_024582
RPL34	-6.6	1.6	ribosomal protein L34 (RPL34), transcript variant 2, mRNA [NM_033625]	NM_033625	Hs.438227	BG112770
CR602569	-6.6	3.1	full-length cDNA clone CS0DC001YL20 of Neuroblastoma Cot 25-normalized of (human). [CR602569]	CR602569	Hs.416013	CR602569
LOC727835	-6.6	1.8	PREDICTED: similar to 60S ribosomal protein L9 (LOC727835), mRNA [XR_015189]	XR_015189	Hs.678843	XR_015767
VCX	-6.6	3.1	variable charge, X-linked (VCX), mRNA [NM_013452]	NM_013452	Hs.567503	AF167081
AGTR1	-6.6	3.6	angiotensin II receptor, type 1 (AGTR1), transcript variant 4, mRNA [NM_031850]	NM_031850	Hs.477887	NM_031850
THC2507829	-6.6	2.0	THC2507829	THC2507829	Unknown	
CDC7	-6.6	2.5	cell division cycle 7 homolog (S. cerevisiae) (CDC7), mRNA [NM_003503]	NM_003503	Hs.533573	AB209337
POLI	-6.6	2.2	polymerase (DNA directed) iota (POLI), mRNA [NM_007195]	NM_007195	Hs.438533	NM_007195
TSHZ2	-6.6	2.5	Teashirt homolog 2 (Zinc finger protein 218) (Ovarian cancer-related protein 10-2) (OVC10-2). [Source:Uniprot/SWISSPROT;Acc:Q9NRE2] [ENST00000371497]	ENST0000037149	Unknown	
A_24_P68572	-6.6	2.0	A_24_P685729	A_24_P685729	Unknown	
RPL36A	-6.6	2.0	ribosomal protein L36a (RPL36A), mRNA [NM_021029]	NM_021029	Hs.432485	CR601778
LOC643932	-6.6	1.8	PREDICTED: similar to 40S ribosomal protein S3a (V-fos transformation effector protein) (LOC643932), mRNA [XR_017289]	XR_017289	Hs.631966	BM804554
AK125648	-6.6	3.3	cDNA FLJ43660 fis, clone SYNOV4004823. [AK125648]	AK125648	Hs.155085	AK125648
THC2613107	-6.6	3.4	Q8NH31_HUMAN (Q8NH31) Seven transmembrane helix receptor, partial (5%) [THC2613107]	THC2613107	Unknown	
LMO2	-6.6	5.6	LIM domain only 2 (rhombotin-like 1) (LMO2), mRNA [NM_005574]	NM_005574	Hs.34560	NM_005574
IFT88	-6.6	1.8	intraflagellar transport 88 homolog (Chlamydomonas) (IFT88), transcript variant 1, mRNA [NM_175605]	NM_175605	Hs.187376	AK126668
HVCN1	-6.6	5.5	hydrogen voltage-gated channel 1 (HVCN1), transcript variant 1, mRNA [NM_001040107]	NM_001040107	Hs.334637	BC032672
FGF20	-6.6	4.2	fibroblast growth factor 20 (FGF20), mRNA [NM_019851]	NM_019851	Hs.199905	NM_019851
LOC399744	-6.6	1.6	hypothetical LOC399744 (LOC399744), mRNA [NM_001013665]	NM_001013665	Unknown	
LMO2	-6.5	5.5	LIM domain only 2 (rhombotin-like 1) (LMO2), mRNA [NM_005574]	NM_005574	Hs.34560	NM_005574
A_24_P39243	-6.5	2.1	A_24_P392436	A_24_P392436	Unknown	
SLTM	-6.5	1.6	SAFB-like, transcription modulator (SLTM), transcript variant 1, mRNA [NM_024755]	NM_024755	Hs.512932	NM_024755
EIF3S6IP	-6.5	2.1	eukaryotic translation initiation factor 3, subunit 6 interacting protein (EIF3S6IP), mRNA [NM_016091]	NM_016091	Hs.446852	AK056129
ADAMTS20	-6.5	6.8	cDNA FLJ13166 fis, clone NT2RP3003701, weakly similar to F-SPONDIN PRECURSOR. [AK023228]	AK023228	Hs.287554	AF488804

LMO2	-6.5	5.4	LIM domain only 2 (rhombotin-like 1) (LMO2), mRNA [NM_005574]	NM_005574	Hs.34560	NM_005574
RPS3A	-6.5	1.9	ribosomal protein S3A (RPS3A), mRNA [NM_001006]	NM_001006	Hs.356572	BI087817
GOLGA8E	-6.5	2.7	golgi autoantigen, golgin subfamily a, 8E (GOLGA8E), mRNA [NM_001012423]	NM_001012423	Hs.454647	BC063309
AF075112	-6.5	2.7	full length insert cDNA YU76E12. [AF075112]	AF075112	Hs.559248	BQ006833
LMO2	-6.5	5.4	LIM domain only 2 (rhombotin-like 1) (LMO2), mRNA [NM_005574]	NM_005574	Hs.34560	NM_005574
LMO2	-6.5	5.4	LIM domain only 2 (rhombotin-like 1) (LMO2), mRNA [NM_005574]	NM_005574	Hs.34560	NM_005574
LOC645683	-6.5	1.6	cDNA clone MGC:87657 IMAGE:5271409, complete cds. [BC067891]	BC067891	Hs.663461	BC067891
AK023040	-6.5	4.6	cDNA FLJ12978 fis, clone NT2RP2006321. [AK023040]	AK023040	Hs.696175	BC065573
PMS1	-6.5	2.3	PMS1 postmeiotic segregation increased 1 (S. cerevisiae) (PMS1), mRNA [NM_000534]	NM_000534	Hs.111749	CR749432
AF113008	-6.5	1.9	clone FLB0708 mRNA sequence. [AF113008]	AF113008	Unknown	
JMJD1B	-6.5	1.9	jumonji domain containing 1B (JMJD1B), mRNA [NM_016604]	NM_016604	Hs.483486	NM_016604
HOOK1	-6.5	2.1	hook homolog 1 (Drosophila) (HOOK1), mRNA [NM_015888]	NM_015888	Hs.378836	NM_015888
SFRS7	-6.5	2.3	splicing factor, arginine/serine-rich 7, 35kDa (SFRS7), mRNA [NM_001031684]	NM_001031684	Hs.309090	AK091425
GPC3	-6.5	5.5	glypican 3 (GPC3), mRNA [NM_004484]	NM_004484	Hs.699193	NM_004484
OVOS2	-6.5	6.8	ovostatin 2, mRNA (cDNA clone IMAGE:4827636). [BC039117]	BC039117	Hs.524331	CR749395
TMEM31	-6.5	4.3	transmembrane protein 31 (TMEM31), mRNA [NM_182541]	NM_182541	Hs.98843	BQ429340
BX419129	-6.5	2.0	BX419129 FETAL BRAIN cDNA clone CS0DF013YC22 5-PRIME, mRNA sequence [BX419129]	BX419129	Hs.595920	BX419129
ZBED5	-6.5	1.5	zinc finger, BED-type containing 5 (ZBED5), mRNA [NM_021211]	NM_021211	Unknown	
PKNOX1	-6.5	1.7	PBX/knotted 1 homeobox 1 (PKNOX1), mRNA [NM_004571]	NM_004571	Hs.431043	NM_004571
WDR19	-6.5	1.7	WD repeat domain 19 (WDR19), mRNA [NM_025132]	NM_025132	Hs.438482	NM_025132
CXorf57	-6.5	2.1	chromosome X open reading frame 57 (CXorf57), mRNA [NM_018015]	NM_018015	Hs.274267	BC070110
AK092715	-6.5	2.5	cDNA FLJ35396 fis, clone SKNSH2003483. [AK092715]	AK092715	Hs.26409	BC041405
CMTM5	-6.4	2.5	CKLF-like MARVEL transmembrane domain containing 5 (CMTM5), transcript variant 3, mRNA [NM_001037288]	NM_001037288	Hs.99272	BM926366
PDZD2	-6.4	3.9	PDZ domain containing 2 (PDZD2), mRNA [NM_178140]	NM_178140	Hs.481819	AB002298
NACA	-6.4	1.6	nascent-polypeptide-associated complex alpha polypeptide (NACA), mRNA [NM_005594]	NM_005594	Hs.505735	AK096699
RP5-1022P6.2	-6.4	1.7	hypothetical protein KIAA1434 (KIAA1434), mRNA [NM_019593]	NM_019593	Hs.636359	NM_019593
SMA5	-6.4	1.8	SMA5 (SMA5), mRNA [NM_021036]	NM_021036	Unknown	
LOC391181	-6.4	2.2	PREDICTED: similar to 60S ribosomal protein L6 (TAX-responsive enhancer element-binding protein 107) (TAXREB107) (Neoplasm-related protein C140) (LOC391181), mRNA [XR_017056]	XR_017056	Hs.693417	XR_018994
LOC731599	-6.4	2.2	PREDICTED: hypothetical protein LOC731599 (LOC731599), mRNA [XR_015536]	XR_015536	Hs.651110	XR_015536
ALS2CR4	-6.4	2.0	amyotrophic lateral sclerosis 2 (juvenile) chromosome region, candidate 4 (ALS2CR4), transcript variant 1, mRNA [NM_001044385]	NM_001044385	Hs.12319	NM_001044385
RPL37	-6.4	1.7	ribosomal protein L37 (RPL37), mRNA [NM_000997]	NM_000997	Hs.80545	AL137450
RPS25	-6.4	1.8	ribosomal protein S25 (RPS25), mRNA [NM_001028]	NM_001028	Hs.512676	BM554735
AK026750	-6.4	2.5	cDNA: FLJ23097 fis, clone LNG07418. [AK026750]	AK026750	Hs.152432	AK026750
KIAA1324L	-6.4	1.9	KIAA1324-like (KIAA1324L), mRNA [NM_152748]	NM_152748	Hs.208093	NM_152748
NKD2	-6.4	2.4	naked cuticle homolog 2 (Drosophila) (NKD2), mRNA [NM_033120]	NM_033120	Hs.240951	BC012176
FLJ13305	-6.4	2.2	mRNA; cDNA DKFZp686O21143 (from clone DKFZp686O21143). [BX648834]	BX648834	Hs.440466	BX648834
LMO2	-6.4	5.5	LIM domain only 2 (rhombotin-like 1) (LMO2), mRNA [NM_005574]	NM_005574	Hs.34560	NM_005574
KIAA2018	-6.4	2.2	KIAA2018 (KIAA2018), mRNA [NM_001009899]	NM_001009899	Hs.632570	NM_001009899
ZCCHC3	-6.4	2.0	zinc finger, CCHC domain containing 3 (ZCCHC3), mRNA [NM_033089]	NM_033089	Hs.28608	BC069238
BC019703	-6.4	3.2	mRNA similar to hypothetical protein FLJ21463 (cDNA clone MGC:24970 IMAGE:4941290), complete cds. [BC019703]	BC019703	Unknown	
ANXA9	-6.4	2.2	annexin A9 (ANXA9), mRNA [NM_003568]	NM_003568	Hs.653223	NM_003568
NR1H3	-6.4	2.3	nuclear receptor subfamily 1, group H, member 3 (NR1H3), mRNA [NM_005693]	NM_005693	Hs.438863	AK122661
TSPAN18	-6.4	1.5	tetraspanin 18 (TSPAN18), transcript variant 2, mRNA [NM_130783]	NM_130783	Hs.385634	AY358087
AK125361	-6.4	2.3	cDNA FLJ43371 fis, clone NTONG2005969. [AK125361]	AK125361	Hs.276808	NM_002409
SLC37A3	-6.4	1.7	solute carrier family 37 (glycerol-3-phosphate transporter), member 3 (SLC37A3), transcript variant 1, mRNA [NM_207113]	NM_207113	Hs.446021	NM_207113
A_32_P16989	-6.4	2.6	A_32_P16989	A_32_P16989	Unknown	
STAU2	-6.4	1.6	staufen, RNA binding protein, homolog 2 (Drosophila) (STAU2), mRNA [NM_014393]	NM_014393	Hs.561815	NM_014393
PRKCB1	-6.4	3.3	protein kinase C, beta 1 (PRKCB1), transcript variant 2, mRNA [NM_002738]	NM_002738	Hs.460355	AL833252
SMAD1	-6.3	1.7	SMAD family member 1 (SMAD1), transcript variant 1, mRNA [NM_005900]	NM_005900	Hs.656534	NM_005900
ENST0000033	-6.3	2.1	PREDICTED: similar to large subunit ribosomal protein L36a (LOC728202), mRNA [XM_001129191]	ENST0000033130	Unknown	
MERTK	-6.3	2.6	Human cellular proto-oncogene (c-mer) mRNA, complete cds. [U08023]	U08023	Hs.306178	NM_006343

LMO2	-6.3	5.3	LIM domain only 2 (rhombotin-like 1) (LMO2), mRNA [NM_005574]	NM_005574	Hs.34560	NM_005574
THC2609493	-6.3	8.2	Q9U4W7_PLAFA (Q9U4W7) DBL alpha protein (Fragment), partial (14%) [THC2609493]	THC2609493	Unknown	
RBM11	-6.3	3.4	RNA binding motif protein 11 (RBM11), mRNA [NM_144770]	NM_144770	Hs.283828	BC030196
C18orf18	-6.3	2.9	chromosome 18 open reading frame 18, mRNA (cDNA clone MGC:17515 IMAGE:3457488), complete cds. [BC010538]	BC010538	Hs.657197	BQ067788
THC2679021	-6.3	1.8	THC2679021	THC2679021	Unknown	
AF088007	-6.3	3.4	full length insert cDNA clone YY74A01. [AF088007]	AF088007	Hs.46689	AF088007
THC2638025	-6.3	2.0	THC2638025	THC2638025	Unknown	
DKFZP686A01	-6.3	1.8	hypothetical protein (DKFZP686A01247), mRNA [NM_014988]	NM_014988	Hs.335163	CR749205
RBM16	-6.3	1.9	RNA binding motif protein 16 (RBM16), mRNA [NM_014892]	NM_014892	Hs.591329	NM_014892
RPL31P10	-6.3	1.7	PREDICTED: similar to ribosomal protein L31 (LOC390283), mRNA [XR_018695]	XR_018695	Hs.646426	XR_018695
BM999256	-6.3	2.8	UI-H-DI0-avo-g-06-0-UI.s1 NCI_CGAP_DI0 cDNA clone IMAGE:5882141 3', mRNA sequence [BM999256]	BM999256	Hs.619312	BM999256
C20orf19	-6.3	2.6	chromosome 20 open reading frame 19 (C20orf19), mRNA [NM_018474]	NM_018474	Hs.187635	BC039296
LOC91431	-6.3	2.8	prematurely terminated mRNA decay factor-like (LOC91431), mRNA [NM_138698]	NM_138698	Unknown	
LOC91431	-6.3	2.7	prematurely terminated mRNA decay factor-like (LOC91431), mRNA [NM_138698]	NM_138698	Unknown	
HAS2	-6.3	19.1	hyaluronan synthase 2 (HAS2), mRNA [NM_005328]	NM_005328	Hs.571528	NM_005328
ENST0000033	-6.3	2.3	similar to 60S ribosomal protein L23a (LOC644384), mRNA [Source:RefSeq_dna;Acc:XR_017413] [ENST00000332696]	ENST0000033269	Unknown	
LOC286272	-6.3	2.3	cDNA FLJ10077 fis, clone HEMBA1001864. [AK000939]	AK000939	Hs.657301	AK000939
RPS3A	-6.3	1.7	ribosomal protein S3A (RPS3A), mRNA [NM_001006]	NM_001006	Hs.356572	BI087817
EIF3S6	-6.3	2.0	eukaryotic translation initiation factor 3, subunit 6 48kDa (EIF3S6), mRNA [NM_001568]	NM_001568	Hs.405590	AK124178
BC038355	-6.3	1.5	Homo sapiens, clone IMAGE:3858719, mRNA. [BC038355]	BC038355	Hs.654953	BC038355
TDRD10	-6.3	5.4	tudor domain containing 10 (TDRD10), mRNA [NM_182499]	NM_182499	Hs.387671	NM_001098475
MKS1	-6.3	2.0	cDNA FLJ20345 fis, clone HEP13723. [AK000352]	AK000352	Hs.408843	NM_017777
THC2521188	-6.3	2.3	CA942877 ir64h07.y1 HR85 islet cDNA clone IMAGE:6607525 5' similar to SW:RL9_HUMAN P32969 60S RIBOSOMAL PROTEIN L9. [1] ;, mRNA sequence [CA942877]	THC2521188	Unknown	
LOC283663	-6.3	2.9	cDNA FLJ33196 fis, clone ADRGL2006034. [AK090515]	AK090515	Hs.181297	AK097083
LOC654170	-6.3	1.8	PREDICTED: similar to 40S ribosomal protein S16 (LOC654170), mRNA [XR_019059]	XR_019059	Hs.650960	XR_019059
AF088004	-6.3	2.8	full length insert cDNA clone YY51E04. [AF088004]	AF088004	Hs.659586	BQ774355
GUSBP1	-6.3	2.7	glucuronidase, beta pseudogene 1 (GUSBP1), mRNA [NM_207331]	NM_207331	Unknown	
PARD6G	-6.3	2.0	par-6 partitioning defective 6 homolog gamma (C. elegans) (PARD6G), mRNA [NM_032510]	NM_032510	Hs.654920	NM_032510
A_24_P85018	-6.3	2.2	A_24_P850187	A_24_P850187	Unknown	
YPEL1	-6.3	2.4	yippee-like 1 (Drosophila) (YPEL1), mRNA [NM_013313]	NM_013313	Hs.517436	CR933630
AL133118	-6.3	21.2	mRNA; cDNA DKFZp586N0121 (from clone DKFZp586N0121). [AL133118]	AL133118	Hs.596517	AL133118
ELF2	-6.3	1.6	E74-like factor 2 (ets domain transcription factor) (ELF2), transcript variant 1, mRNA [NM_201999]	NM_201999	Hs.699401	U43188
LOC643981	-6.3	2.0	PREDICTED: similar to 40S ribosomal protein S3a (V-fos transformation effector protein) (LOC643981), mRNA [XR_018444]	XR_018444	Hs.647181	XR_018444
TSHZ2	-6.3	2.4	teashirt family zinc finger 2 (TSHZ2), mRNA [NM_173485]	NM_173485	Hs.473117	NM_173485
AK023737	-6.3	5.9	cDNA FLJ13675 fis, clone PLACE1011875, highly similar to mRNA for KIAA0580 protein. [AK023737]	AK023737	Unknown	
SH3YL1	-6.3	3.9	SH3 domain containing, Ysc84-like 1 (S. cerevisiae), mRNA (cDNA clone IMAGE:4825726), complete cds. [BC030778]	BC030778	Hs.515951	AK123829
RCBTB2	-6.3	3.0	regulator of chromosome condensation (RCC1) and BTB (POZ) domain containing protein 2 (RCBTB2), mRNA [NM_001268]	NM_001268	Hs.657385	NM_001268
A_32_P12228	-6.3	1.6	A_32_P122285	A_32_P122285	Unknown	
THC2579654	-6.2	2.3	Q6P9U5_RAT (Q6P9U5) Ribosomal protein L9, partial (74%) [THC2579654]	THC2579654	Unknown	
LRP2	-6.2	4.0	low density lipoprotein-related protein 2 (LRP2), mRNA [NM_004525]	NM_004525	Hs.700749	NM_004525
NR2F2	-6.2	2.1	nuclear receptor subfamily 2, group F, member 2 (NR2F2), mRNA [NM_021005]	NM_021005	Hs.347991	BC042897
A_24_P12825	-6.2	2.6	A_24_P128255	A_24_P128255	Unknown	
KBTBD7	-6.2	2.4	kelch repeat and BTB (POZ) domain containing 7 (KBTBD7), mRNA [NM_032138]	NM_032138	Hs.63841	BC022033
A_24_P28988	-6.2	1.9	A_24_P289884	A_24_P289884	Unknown	
7-Sep	-6.2	1.3	septin 7 (SEPT7), transcript variant 2, mRNA [NM_001011553]	NM_001011553	Hs.191346	AB209677
CBX1	-6.2	1.8	chromobox homolog 1 (HP1 beta homolog Drosophila) (CBX1), mRNA [NM_006807]	NM_006807	Hs.77254	NM_006807
FLJ10324	-6.2	3.1	hypothetical protein FLJ10324 (FLJ10324), mRNA [NM_018059]	NM_018059	Hs.667336	AB058752
RPL34	-6.2	1.7	ribosomal protein L34 (RPL34), transcript variant 2, mRNA [NM_033625]	NM_033625	Hs.438227	BG112770
SLU7	-6.2	1.3	SLU7 splicing factor homolog (S. cerevisiae) (SLU7), mRNA [NM_006425]	NM_006425	Hs.435342	NM_006425
PYGO1	-6.2	1.9	mRNA; cDNA DKFZp564G0982 (from clone DKFZp564G0982). [AL049925]	AL049925	Hs.256587	AL049925

AK097080	-6.2	1.8	cDNA FLJ39761 fis, clone SPLEN1000083. [AK097080]	AK097080	Hs.534942	XR_017612
THC2533996	-6.2	2.2	HSU09954 ribosomal protein L9 {Homo sapiens} (exp=-1; wgp=0; cg=0), partial (42%) [THC2533996]	THC2533996	Unknown	
PRTG	-6.2	3.8	protogenin homolog (Gallus gallus) (PRTG), mRNA [NM_173814]	NM_173814	Hs.130957	NM_173814
THC2654987	-6.2	2.5	THC2654987	THC2654987	Unknown	
SLC9A5	-6.2	2.1	solute carrier family 9 (sodium/hydrogen exchanger), member 5 (SLC9A5), mRNA [NM_004594]	NM_004594	Hs.439650	BC150207
MTTP	-6.2	4.1	microsomal triglyceride transfer protein (MTTP), mRNA [NM_000253]	NM_000253	Hs.195799	NM_000253
MDFIC	-6.2	2.1	MyoD family inhibitor domain containing (MDFIC), mRNA [NM_199072]	NM_199072	Hs.427236	NM_199072
THC2606490	-6.2	2.8	THC2606490	THC2606490	Unknown	
EMCN	-6.2	11.8	endomucin (EMCN), mRNA [NM_016242]	NM_016242	Hs.152913	NM_016242
NKTR	-6.2	2.0	natural killer-tumor recognition sequence (NKTR), transcript variant 2, mRNA [NM_001012651]	NM_001012651	Unknown	
NFYB	-6.2	2.5	nuclear transcription factor Y, beta (NFYB), mRNA [NM_006166]	NM_006166	Hs.84928	NM_006166
ENST0000033	-6.2	1.7	PREDICTED: similar to 60S ribosomal protein L17 (L23) (LOC650848), mRNA [XR_019013]	ENST0000033468	Unknown	
LOC341511	-6.2	2.3	PREDICTED: similar to 60S ribosomal protein L23a (LOC341511), mRNA [XM_292109]	ENST0000023784	Unknown	
SNN	-6.2	1.8	stannin (SNN), mRNA [NM_003498]	NM_003498	Hs.700592	NM_003498
CUL3	-6.2	2.7	cullin 3 (CUL3), mRNA [NM_003590]	NM_003590	Hs.372286	NM_003590
EDN3	-6.2	4.8	endothelin 3 (EDN3), transcript variant 2, mRNA [NM_207032]	NM_207032	Hs.1408	BC053866
PRKD1	-6.2	3.0	protein kinase D1 (PRKD1), mRNA [NM_002742]	NM_002742	Hs.508999	X75756
GKAP1	-6.2	1.8	G kinase anchoring protein 1 (GKAP1), mRNA [NM_025211]	NM_025211	Hs.522255	AK026487
AEBP2	-6.2	1.7	AE binding protein 2 (AEBP2), mRNA [NM_153207]	NM_153207	Hs.126497	AB209384
THC2632459	-6.2	1.7	Q6VEP1_HUMAN (Q6VEP1) F379 retina specific protein, partial (28%) [THC2632459]	THC2632459	Unknown	
BM468849	-6.2	2.6	AGENCOURT_6447766 NIH_MGC_92 cDNA clone IMAGE:5587170 5', mRNA sequence [BM468849]	BM468849	Hs.270571	BM468849
LOC648294	-6.2	1.9	PREDICTED: hypothetical LOC648294 (LOC648294), mRNA [XR_018553]	XR_018553	Hs.693351	XR_018553
LOC731048	-6.2	1.9	PREDICTED: similar to 60S ribosomal protein L17 (L23) (LOC731048), mRNA [XR_015710]	XR_015710	Hs.665966	AV696226
BF312639	-6.2	3.1	BF312639 601898132F1 NIH_MGC_19 cDNA clone IMAGE:4127478 5', mRNA sequence [BF312639]	BF312639	Hs.655654	U79716
MGC42174	-6.1	1.8	cDNA FLJ36974 fis, clone BRACE2006264. [AK094293]	AK094293	Hs.471637	AL834174
LOC120318	-6.1	1.8	PREDICTED: similar to 40S ribosomal protein S6 (LOC120318), mRNA [XR_018482]	XR_018482	Hs.647225	XR_018482
AF098968	-6.1	1.6	familial Mediterranean fever locus region, mRNA sequence. [AF098968]	AF098968	Hs.522143	AF098968
IRAK1BP1	-6.1	2.9	interleukin-1 receptor-associated kinase 1 binding protein 1 (IRAK1BP1), mRNA [NM_001010844]	NM_001010844	Hs.656212	AK098678
CPS1	-6.1	6.1	carbamoyl-phosphate synthetase 1, mitochondrial (CPS1), mRNA [NM_001875]	NM_001875	Hs.149252	NM_001875
ZRANB3	-6.1	2.4	zinc finger, RAN-binding domain containing 3, mRNA (cDNA clone IMAGE:5575956), complete cds. [BC064616]	BC064616	Hs.658422	BX647838
CDH19	-6.1	9.2	cadherin 19, type 2 (CDH19), mRNA [NM_021153]	NM_021153	Hs.42771	NM_021153
CCDC98	-6.1	2.1	coiled-coil domain containing 98 (CCDC98), mRNA [NM_139076]	NM_139076	Hs.334772	NM_139076
AK023526	-6.1	4.2	cDNA FLJ13464 fis, clone PLACE1003478. [AK023526]	AK023526	Hs.670477	AK023526
FBXO17	-6.1	3.0	F-box protein 17 (FBXO17), transcript variant 2, mRNA [NM_024907]	NM_024907	Hs.531770	AK226156
SLC29A3	-6.1	2.0	solute carrier family 29 (nucleoside transporters), member 3 (SLC29A3), mRNA [NM_018344]	NM_018344	Hs.438419	NM_018344
BC040982	-6.1	2.0	cDNA clone IMAGE:4798675. [BC040982]	BC040982	Hs.656958	BC040982
NT5DC1	-6.1	2.4	5'-nucleotidase domain containing 1 (NT5DC1), mRNA [NM_152729]	NM_152729	Hs.520341	NM_152729
LOC399744	-6.1	1.7	hypothetical LOC399744 (LOC399744), mRNA [NM_001013665]	NM_001013665	Unknown	
RPS7	-6.1	1.8	ribosomal protein S7 (RPS7), mRNA [NM_001011]	NM_001011	Hs.546287	AB209386
AF037219	-6.1	2.8	PIX1 mRNA sequence. [AF037219]	AF037219	Hs.654383	NM_130839
ZNF16	-6.1	2.0	zinc finger protein 16 (ZNF16), transcript variant 2, mRNA [NM_001029976]	NM_001029976	Hs.493225	DQ117529
C14orf132	-6.1	1.8	chromosome 14 open reading frame 132 (C14orf132), mRNA [NM_020215]	NM_020215	Unknown	
PARP11	-6.1	2.2	poly (ADP-ribose) polymerase family, member 11 (PARP11), mRNA [NM_020367]	NM_020367	Hs.657268	CR749294
ZNF160	-6.1	1.9	zinc finger protein 160 (ZNF160), transcript variant 2, mRNA [NM_198893]	NM_198893	Hs.655967	NM_198893
KIAA1840	-6.1	1.4	KIAA1840 (KIAA1840), mRNA [NM_025137]	NM_025137	Hs.656271	NM_025137
NSUN6	-6.1	2.2	NOL1/NOP2/Sun domain family, member 6 (NSUN6), mRNA [NM_182543]	NM_182543	Hs.396175	BC033534
THC2650423	-6.1	2.2	THC2650423	THC2650423	Unknown	
RPL23A	-6.1	1.7	ribosomal protein L23a (RPL23A), mRNA [NM_000984]	NM_000984	Hs.419463	CR616046
AK130118	-6.1	7.4	cDNA FLJ26608 fis, clone LVR00914. [AK130118]	AK130118	Hs.654777	AK130118
THC2655194	-6.1	2.0	ALU6_HUMAN (P39193) Alu subfamily SP sequence contamination warning entry, partial (5%) [THC2655194]	THC2655194	Unknown	
AK092942	-6.1	1.8	cDNA FLJ35623 fis, clone SPLEN2010986. [AK092942]	AK092942	Hs.660700	XR_015431

RSPO2	-6.1	2.1	R-spondin 2 homolog (Xenopus laevis) (RSPO2), mRNA [NM_178565]	NM_178565	Hs.444834	AK123023
RPL31P4	-6.1	1.6	PREDICTED: ribosomal protein L31 pseudogene 4 (RPL31P4), mRNA [XR_018222]	XR_018222	Hs.638676	BG675689
LOC375748	-6.1	1.6	RAD26L hypothetical protein (LOC375748), mRNA [NM_001010895]	NM_001010895	Hs.432364	BC075860
RPL30	-6.1	1.7	ribosomal protein L30 (RPL30), mRNA [NM_000989]	NM_000989	Hs.400295	AK128768
SEMA6A	-6.1	2.9	sema domain, transmembrane domain (TM), and cytoplasmic domain, (semaphorin) 6A (SEMA6A), mRNA [NM_020796]	NM_020796	Hs.156967	BC032619
AK123439	-6.1	2.3	cDNA FLJ41445 fis, clone BRSTN2002105. [AK123439]	AK123439	Hs.701639	AK123439
CCNL1	-6.1	2.2	cyclin L1 (CCNL1), mRNA [NM_020307]	NM_020307	Hs.4859	BX641146
LOC392505	-6.1	2.1	PREDICTED: similar to 60S ribosomal protein L6 (TAX-responsive enhancer element-binding protein 107) (TAXREB107) (Neoplasm-related protein C140) (LOC392505), mRNA [XR_018334]	XR_018334	Hs.648330	XR_018334
BF931515	-6.1	1.6	BF931515 IL2-NT0202-141200-308-H03 NT0202 cDNA, mRNA sequence [BF931515]	BF931515	Unknown	
DTNB	-6.1	1.6	dystrobrevin, beta (DTNB), transcript variant 4, mRNA [NM_183360]	NM_183360	Hs.307720	Y15718
LRP16	-6.1	2.2	LRP16 protein (LRP16), mRNA [NM_014067]	NM_014067	Hs.602898	AL833017
DLG3	-6.1	2.0	discs, large homolog 3 (neuroendocrine-dlg, Drosophila) (DLG3), transcript variant 1, mRNA [NM_021120]	NM_021120	Hs.522680	NM_021120
AASDHPPT	-6.1	1.6	aminoadipate-semialdehyde dehydrogenase-phosphopantetheinyl transferase (AASDHPPT), mRNA [NM_015423]	NM_015423	Hs.524009	BX537665
WASF1	-6.1	3.0	WAS protein family, member 1 (WASF1), transcript variant 1, mRNA [NM_003931]	NM_003931	Hs.75850	NM_003931
FLJ12716	-6.1	1.6	FLJ12716 protein (FLJ12716), transcript variant 1, mRNA [NM_021942]	NM_021942	Hs.443240	BX647127
CCDC122	-6.1	1.6	coiled-coil domain containing 122 (CCDC122), mRNA [NM_144974]	NM_144974	Hs.170849	NM_144974
ZNF180	-6.1	2.6	zinc finger protein 180 (ZNF180), mRNA [NM_013256]	NM_013256	Hs.130683	BC051903
PUNC	-6.0	8.7	putative neuronal cell adhesion molecule (PUNC), mRNA [NM_004884]	NM_004884	Hs.128292	NM_004884
LOC729449	-6.0	1.7	PREDICTED: similar to 60S ribosomal protein L7 (LOC729449), mRNA [XR_015548]	XR_015548	Hs.534938	XR_015548
LOC730663	-6.0	1.7	PREDICTED: similar to 60S ribosomal protein L23a (LOC730663), mRNA [XM_001128309]	XM_001128309	Unknown	
BNC1	-6.0	11.9	basonuclin 1 (BNC1), mRNA [NM_001717]	NM_001717	Hs.459153	L03427
ENST0000033	-6.0	3.0	Nuclear factor erythroid 2-related factor 2 (NF-E2-related factor 2) (NFE2-related factor 2) (Nuclear factor, erythroid derived 2, like 2) (HEBP1). [Source:Uniprot/SWISSPROT;Acc:Q16236] [ENST00000338358]	ENST0000033835	Unknown	
A_32_P19661	-6.0	5.0	A_32_P196615	A_32_P196615	Unknown	
SMC3	-6.0	2.1	structural maintenance of chromosomes 3 (SMC3), mRNA [NM_005445]	NM_005445	Hs.24485	NM_005445
BRD8	-6.0	1.6	bromodomain containing 8 (BRD8), transcript variant 1, mRNA [NM_006696]	NM_006696	Hs.519337	NM_139199
ENST0000037	-6.0	2.4	hypothetical protein LOC348751, mRNA (cDNA clone IMAGE:5311172). [BC039445]	ENST0000037471	Unknown	
LRRCC1	-6.0	2.3	leucine rich repeat and coiled-coil domain containing 1 (LRRCC1), transcript variant 1, mRNA [NM_033402]	NM_033402	Hs.193115	AB051551
NUDT6	-6.0	2.7	nudix (nucleoside diphosphate linked moiety X)-type motif 6 (NUDT6), transcript variant 2, mRNA [NM_198041]	NM_198041	Hs.558459	AB209758
AK094187	-6.0	2.1	cDNA FLJ36868 fis, clone ASTRO2016681. [AK094187]	AK094187	Unknown	
A_24_P30696	-6.0	1.9	A_24_P306968	A_24_P306968	Unknown	
BLMH	-6.0	1.6	bleomycin hydrolase (BLMH), mRNA [NM_000386]	NM_000386	Hs.371914	NM_000386
C11orf65	-6.0	2.3	chromosome 11 open reading frame 65 (C11orf65), mRNA [NM_152587]	NM_152587	Hs.653180	NM_152587
A_24_P63683	-6.0	2.5	A_24_P636834	A_24_P636834	Unknown	
CR596214	-6.0	2.6	full-length cDNA clone CS0DC006YB07 of Neuroblastoma Cot 25-normalized of (human). [CR596214]	CR596214	Hs.662029	BG036557
ZBTB26	-6.0	2.5	Zinc finger and BTB domain-containing protein 26 (Zinc finger protein 481) (Zinc finger protein Bioref). [Source:Uniprot/SWISSPROT;Acc:Q9HCK0] [ENST00000373656]	ENST0000037365	Unknown	
AL079294	-6.0	2.0	mRNA full length insert cDNA clone EUROIMAGE 362780. [AL079294]	AL079294	Hs.29952	AK096998
AK123297	-6.0	2.2	cDNA FLJ41303 fis, clone BRAMY2042131. [AK123297]	AK123297	Hs.648656	AK123297
AL831999	-6.0	2.0	mRNA; cDNA DKFZp451K063 (from clone DKFZp451K063). [AL831999]	AL831999	Hs.547396	AL831999
WWOX	-6.0	3.2	WW domain containing oxidoreductase (WWOX), transcript variant 1, mRNA [NM_016373]	NM_016373	Hs.461453	NM_016373
ATXN7	-6.0	1.6	ataxin 7 (ATXN7), mRNA [NM_000333]	NM_000333	Hs.476595	NM_000333
GJB1	-6.0	2.5	gap junction protein, beta 1, 32kDa (connexin 32, Charcot-Marie-Tooth neuropathy, X-linked) (GJB1), mRNA [NM_000166]	NM_000166	Hs.333303	BF690836
LYRM4	-6.0	2.6	LYR motif containing 4 (LYRM4), mRNA [NM_020408]	NM_020408	Hs.696067	NM_020408
THC2704037	-6.0	2.2	THC2704037	THC2704037	Unknown	
PDGFRA	-6.0	4.9	platelet-derived growth factor receptor, alpha polypeptide, mRNA (cDNA clone IMAGE:4043984), complete cds. [BC015186]	BC015186	Hs.74615	NM_006206
ANGPT1	-6.0	3.0	angiopoietin 1 (ANGPT1), mRNA [NM_001146]	NM_001146	Hs.369675	BX648814
CABLES2	-6.0	1.8	Cdk5 and Abl enzyme substrate 2 (CABLES2), mRNA [NM_031215]	NM_031215	Hs.301040	NM_031215
CEP110	-6.0	2.6	centrosomal protein 110kDa (CEP110), mRNA [NM_007018]	NM_007018	Hs.653263	NM_007018
FRMD4B	-6.0	2.5	mRNA for KIAA1013 protein, partial cds. [AB023230]	AB023230	Hs.371681	NM_015123

AK021866	-6.0	3.7	cDNA FLJ11804 fis, clone HEMBA1006272, moderately similar to RETROVIRUS-RELATED PROTEASE (EC 3.4.23.-). [AK021866]	AK021866	Unknown	
NPR3	-6.0	2.2	natriuretic peptide receptor C/guanylate cyclase C (atrionatriuretic peptide receptor C) (NPR3), mRNA [NM_000908]	NM_000908	Hs.237028	NM_000908
CPS1	-6.0	7.7	carbamoyl-phosphate synthetase 1, mitochondrial (CPS1), mRNA [NM_001875]	NM_001875	Hs.149252	NM_001875
RPL23A	-6.0	2.1	ribosomal protein L23a (RPL23A), mRNA [NM_000984]	NM_000984	Hs.419463	CR616046
BM690036	-6.0	4.3	UI-E-CK1-abr-b-07-0-UI.r1 UI-E-CK1 cDNA clone UI-E-CK1-abr-b-07-0-UI 5', mRNA sequence [BM690036]	BM690036	Hs.121667	BM690036
PARD6B	-6.0	2.4	par-6 partitioning defective 6 homolog beta (C. elegans) (PARD6B), mRNA [NM_032521]	NM_032521	Hs.589848	NM_032521
RPL7	-6.0	1.7	ribosomal protein L7 (RPL7), mRNA [NM_000971]	NM_000971	Hs.571841	BM808571
CYP26B1	-6.0	2.0	cytochrome P450, family 26, subfamily B, polypeptide 1 (CYP26B1), mRNA [NM_019885]	NM_019885	Hs.91546	NM_019885
ENST0000032	-6.0	3.5	TIGA1 (Chromosome 5 open reading frame 26). [Source:Uniprot/SPTREMBL;Acc:Q96Q82] [ENST00000321614]	ENST0000032161	Unknown	
RPL17	-6.0	1.7	ribosomal protein L17 (RPL17), transcript variant 1, mRNA [NM_000985]	NM_000985	Hs.374588	BF970890
CR593048	-5.9	1.6	full-length cDNA clone CS0DC002YJ17 of Neuroblastoma Cot 25-normalized of (human). [CR593048]	CR593048	Hs.574684	CR593048
APRIN	-5.9	2.2	androgen-induced proliferation inhibitor (APRIN), mRNA [NM_015032]	NM_015032	Hs.699308	AL137201
ACTR3B	-5.9	2.4	ARP3 actin-related protein 3 homolog B (yeast) (ACTR3B), transcript variant 1, mRNA [NM_020445]	NM_020445	Hs.647117	AB209174
ACVR2A	-5.9	1.8	activin A receptor, type IIA (ACVR2A), mRNA [NM_001616]	NM_001616	Hs.470174	NM_001616
RKHD3	-5.9	3.1	ring finger and KH domain containing 3 (RKHD3), mRNA [NM_032246]	NM_032246	Hs.104744	AK131424
TANC1	-5.9	1.4	tetratricopeptide repeat, ankyrin repeat and coiled-coil containing 1 (TANC1), mRNA [NM_033394]	NM_033394	Hs.158728	NM_033394
MAP4K5	-5.9	1.6	mitogen-activated protein kinase kinase kinase kinase 5 (MAP4K5), transcript variant 2, mRNA [NM_198794]	NM_198794	Hs.130491	NM_198794
REV3L	-5.9	1.7	REV3-like, catalytic subunit of DNA polymerase zeta (yeast) (REV3L), mRNA [NM_002912]	NM_002912	Hs.232021	AF078695
CR615016	-5.9	3.5	full-length cDNA clone CS0DB006YM19 of Neuroblastoma Cot 10-normalized of (human). [CR615016]	CR615016	Hs.19193	BC009385
A_32_P38228	-5.9	2.3	A_32_P38228	A_32_P38228	Unknown	
RPL23	-5.9	1.7	ribosomal protein L23 (RPL23), mRNA [NM_000978]	NM_000978	Hs.406300	CR604268
THC2679528	-5.9	2.4	THC2679528	THC2679528	Unknown	
TGIF2	-5.9	3.0	TGFB-induced factor 2 (TALE family homeobox) (TGIF2), mRNA [NM_021809]	NM_021809	Hs.632264	BC012816
CTDSPL2	-5.9	1.3	CTD (carboxy-terminal domain, RNA polymerase II, polypeptide A) small phosphatase like 2 (CTDSPL2), mRNA [NM_016396]	NM_016396	Hs.646495	NM_016396
HESX1	-5.9	3.5	homeobox, ES cell expressed 1 (HESX1), mRNA [NM_003865]	NM_003865	Hs.171980	NM_003865
RTTN	-5.9	2.0	rotatin (RTTN), mRNA [NM_173630]	NM_173630	Hs.654809	NM_173630
GREB1	-5.9	3.6	GREB1 protein (GREB1), transcript variant a, mRNA [NM_014668]	NM_014668	Hs.467733	NM_014668
BC014971	-5.9	2.3	Homo sapiens, Similar to tubulin, beta, 2, clone IMAGE:4873024, mRNA. [BC014971]	BC014971	Hs.513833	BC014971
ENST0000033	-5.9	1.8	PREDICTED: hypothetical LOC391504 (LOC391504), mRNA [XR_018627]	ENST0000033335	Unknown	
ZBED5	-5.9	1.8	zinc finger, BED-type containing 5 (ZBED5), mRNA [NM_021211]	NM_021211	Unknown	
LOC391140	-5.9	1.9	PREDICTED: similar to ribosomal protein L13 (LOC391140), mRNA [XR_018970]	XR_018970	Hs.647731	XR_018970
HSPBAP1	-5.9	1.8	HSPB (heat shock 27kDa) associated protein 1 (HSPBAP1), mRNA [NM_024610]	NM_024610	Hs.29169	AK096705
DCUN1D4	-5.9	1.8	DCN1, defective in cullin neddylation 1, domain containing 4 (S. cerevisiae) (DCUN1D4), transcript variant 1, mRNA [NM_001040402]	NM_001040402	Hs.605388	NM_001040402
C10orf84	-5.9	2.2	Novel protein. [Source:Uniprot/SPTREMBL;Acc:Q5T373] [ENST00000369183]	ENST0000036918	Unknown	
ZBTB24	-5.9	4.3	zinc finger and BTB domain containing 24 (ZBTB24), mRNA [NM_014797]	NM_014797	Hs.409876	NM_014797
FXR1	-5.9	1.7	fragile X mental retardation, autosomal homolog 1 (FXR1), transcript variant 3, mRNA [NM_001013439]	NM_001013439	Hs.478407	NM_001013439
PFTK1	-5.9	1.9	PFTAIRE protein kinase 1 (PFTK1), mRNA [NM_012395]	NM_012395	Hs.430742	AB020641
C3orf17	-5.9	1.9	chromosome 3 open reading frame 17 (C3orf17), transcript variant 1, mRNA [NM_015412]	NM_015412	Hs.591288	NM_015412
STAR	-5.9	3.6	steroidogenic acute regulator (STAR), nuclear gene encoding mitochondrial protein, transcript variant 1, mRNA [NM_000349]	NM_000349	Hs.521535	NM_000349
GCA	-5.9	2.8	grancalcin, EF-hand calcium binding protein (GCA), mRNA [NM_012198]	NM_012198	Hs.377894	BC005214
BRUNOL6	-5.9	2.7	bruno-like 6, RNA binding protein (Drosophila) (BRUNOL6), mRNA [NM_052840]	NM_052840	Hs.348342	AK131098
PCDH17	-5.9	2.9	protocadherin 17 (PCDH17), mRNA [NM_001040429]	NM_001040429	Hs.106511	NM_001040429
BQ028240	-5.9	2.7	BQ028240 UI-H-CO0-arn-b-02-0-UI.s1 NCI_CGAP_Sub9 cDNA clone IMAGE:3106874 3', mRNA sequence [BQ028240]	BQ028240	Unknown	
SALL2	-5.9	2.2	sal-like 2 (Drosophila) (SALL2), mRNA [NM_005407]	NM_005407	Hs.416358	NM_005407
A_24_P37593	-5.9	2.0	A_24_P375932	A_24_P375932	Unknown	
STARD8	-5.9	2.3	START domain containing 8 (STARD8), mRNA [NM_014725]	NM_014725	Hs.95140	CR749411
THC2505102	-5.9	2.3	Q9V0W2_PYRAB (Q9V0W2) Predicted permease, partial (6%) [THC2505102]	THC2505102	Unknown	
CCDC25	-5.9	2.3	coiled-coil domain containing 25 (CCDC25), mRNA [NM_018246]	NM_018246	Hs.445512	NM_018246
PPWD1	-5.9	2.1	peptidylprolyl isomerase domain and WD repeat containing 1 (PPWD1), mRNA [NM_015342]	NM_015342	Hs.121432	AK093675

AZI2	-5.9	1.5	5-azacytidine induced 2 (AZI2), mRNA [NM_022461]	NM_022461	Hs.700605	BX648471
A_24_P34117	-5.9	1.9	A_24_P341176	A_24_P341176	Unknown	
C13orf25	-5.9	4.2	C13orf25 v_2 mRNA, complete cds, miR-91-precursor-13 micro RNA, microRNA miR-91, microRNA miR-17, miR-18-precursor-13 micro RNA, microRNA miR-18, miR-19a-precursor-13 micro RNA, microRNA miR-19a, microRNA miR-20, miR-19b-precursor-13...	AB176708	Hs.24115	AB176708
ENST0000025	-5.9	1.9	F<lambda>8 protein (Fragment). [Source:Uniprot/SPTREMBL;Acc:Q99919] [ENST00000255896]	ENST0000025589	Unknown	
CD242823	-5.9	1.6	AGENCOURT_14126724 NIH_MGC_179 cDNA clone IMAGE:30385216 5', mRNA sequence [CD242823]	CD242823	Hs.597139	CD242823
THC2694873	-5.9	6.3	THC2694873	THC2694873	Unknown	
THC2732032	-5.9	2.5	AA524654 nh31h10.s1 NCI_CGAP_Pr3 cDNA clone IMAGE:954019, mRNA sequence [AA524654]	THC2732032	Unknown	
RPL34	-5.9	2.1	ribosomal protein L34 (RPL34), transcript variant 2, mRNA [NM_033625]	NM_033625	Hs.438227	BG112770
CA312250	-5.9	1.7	CA312250 UI-CF-FN0-afm-d-23-0-UI.s1 UI-CF-FN0 cDNA clone UI-CF-FN0-afm-d-23-0-UI 3', mRNA sequence [CA312250]	CA312250	Hs.307836	BQ723235
PLA2G12B	-5.9	2.9	phospholipase A2, group XIIB (PLA2G12B), mRNA [NM_032562]	NM_032562	Hs.333175	AF339053
RPL35A	-5.9	1.9	ribosomal protein L35a (RPL35A), mRNA [NM_000996]	NM_000996	Hs.529631	AK021571
BRWD1	-5.9	2.6	bromodomain and WD repeat domain containing 1 (BRWD1), transcript variant 1, mRNA [NM_018963]	NM_018963	Hs.654740	NM_033656
MDM4	-5.8	1.8	Mdm4, transformed 3T3 cell double minute 4, p53 binding protein (mouse) (MDM4), mRNA [NM_002393]	NM_002393	Hs.658187	BX640923
FLJ22536	-5.8	1.8	cDNA FLJ12803 fis, clone NT2RP2002172. [AK022865]	AK022865	Hs.399719	AK094718
PFKFB3	-5.8	4.7	6-phosphofructo-2-kinase/fructose-2,6-biphosphatase 3 (PFKFB3), mRNA [NM_004566]	NM_004566	Hs.195471	BC042656
SYT15	-5.8	1.8	cDNA FLJ29001 fis, clone CBL08678. [AK131036]	AK131036	Hs.696346	NM_031912
ADAMTS1	-5.8	2.2	ADAM metallopeptidase with thrombospondin type 1 motif, 1 (ADAMTS1), mRNA [NM_006988]	NM_006988	Hs.643357	NM_006988
AV753543	-5.8	2.4	AV753543 NPD cDNA clone NPDBEC03 5', mRNA sequence [AV753543]	AV753543	Hs.59093	AV753543
RP11-78J21.1	-5.8	2.5	heterogeneous nuclear ribonucleoprotein A1-like (LOC144983), transcript variant 1, mRNA [NM_001011724]	NM_001011724	Hs.447506	AK126454
AK098629	-5.8	1.9	cDNA FLJ25763 fis, clone TST06294. [AK098629]	AK098629	Hs.657362	BF789913
AA714537	-5.8	2.0	nw20g12.s1 NCI_CGAP_GCB0 cDNA clone IMAGE:1241062 3' similar to gb:M84711 40S RIBOSOMAL PROTEIN S3A (HUMAN);, mRNA sequence [AA714537]	AA714537	Hs.567702	BM911048
KPNA5	-5.8	2.6	Importin alpha-6 subunit (Karyopherin alpha-5 subunit). [Source:Uniprot/SWISSPROT;Acc:O15131] [ENST00000368564]	ENST0000036856	Unknown	
GRRP1	-5.8	1.7	glycine/arginine rich protein 1 (GRRP1), mRNA [NM_024869]	NM_024869	Hs.694119	NM_024869
ZNF614	-5.8	2.1	zinc finger protein 614 (ZNF614), mRNA [NM_025040]	NM_025040	Hs.292336	NM_025040
NIPBL	-5.8	1.9	Nipped-B homolog (Drosophila) (NIPBL), transcript variant A, mRNA [NM_133433]	NM_133433	Hs.481927	AJ627032
ZFP37	-5.8	2.3	zinc finger protein 37 homolog (mouse) (ZFP37), mRNA [NM_003408]	NM_003408	Hs.150406	AF022158
AK096580	-5.8	1.8	cDNA FLJ39261 fis, clone OCBBF2009391. [AK096580]	AK096580	Hs.13281	AK096580
A_24_P92997	-5.8	2.0	A_24_P929974	A_24_P929974	Unknown	
FLJ14803	-5.8	1.8	hypothetical protein FLJ14803 (FLJ14803), mRNA [NM_032842]	NM_032842	Hs.267245	NM_032842
TAS2R14	-5.8	1.8	taste receptor, type 2, member 14 (TAS2R14), mRNA [NM_023922]	NM_023922	Hs.679406	CD558711
CA14	-5.8	2.6	carbonic anhydrase XIV (CA14), mRNA [NM_012113]	NM_012113	Hs.528988	AK074765
LOC643974	-5.8	2.2	PREDICTED: similar to 60S ribosomal protein L6 (TAX-responsive enhancer element-binding protein 107) (TAXREB107) (Neoplasm-related protein C140) (LOC643974), mRNA [XR_018643]	XR_018643	Hs.648000	XR_018643
JMJD1C	-5.8	2.1	jumonji domain containing 1C (JMJD1C), transcript variant 2, mRNA [NM_004241]	NM_004241	Hs.413416	NM_032776
AK058000	-5.8	3.3	cDNA FLJ25271 fis, clone STM05584. [AK058000]	AK058000	Hs.594921	AK058000
LOC647722	-5.8	2.0	PREDICTED: similar to ribosomal protein L3 isoform a (LOC647722), mRNA [XR_018187]	XR_018187	Hs.647553	XR_018187
TSGA14	-5.8	2.4	testis specific, 14 (TSGA14), mRNA [NM_018718]	NM_018718	Hs.368315	AF429308
WDR20	-5.8	2.0	WD repeat domain 20, mRNA (cDNA clone IMAGE:5267974), complete cds. [BC030654]	BC030654	Hs.36859	AB209148
MYF6	-5.8	9.0	myogenic factor 6 (herculin) (MYF6), mRNA [NM_002469]	NM_002469	Hs.35937	NM_002469
C12orf35	-5.8	2.3	chromosome 12 open reading frame 35 (C12orf35), mRNA [NM_018169]	NM_018169	Hs.445129	NM_018169
BCL11A	-5.8	2.1	B-cell CLL/lymphoma 11A (zinc finger protein) (BCL11A), transcript variant 5, mRNA [NM_138553]	NM_138553	Unknown	
THC2577090	-5.8	2.2	RL37_HUMAN (P61927) 60S ribosomal protein L37 (G1.16), partial (60%) [THC2577090]	THC2577090	Unknown	
AMDHD1	-5.8	2.2	amidohydrolase domain containing 1 (AMDHD1), mRNA [NM_152435]	NM_152435	Hs.424907	AK054617
BG196990	-5.8	1.8	RST16222 Athersys RAGE Library cDNA, mRNA sequence [BG196990]	BG196990	Hs.454011	ES309195
EIF3S6IP	-5.8	2.0	eukaryotic translation initiation factor 3, subunit 6 interacting protein (EIF3S6IP), mRNA [NM_016091]	NM_016091	Hs.446852	AK056129
LOC401975	-5.8	2.1	PREDICTED: similar to ribosomal protein S3a (LOC401975), mRNA [XR_017247]	XR_017247	Hs.647716	XR_017247
LOC390282	-5.8	1.7	PREDICTED: similar to eukaryotic translation initiation factor 3, subunit 5 (epsilon) (LOC390282), mRNA [XM_372447]	ENST0000033373	Unknown	
NR0B1	-5.8	3.2	nuclear receptor subfamily 0, group B, member 1 (NR0B1), mRNA [NM_000475]	NM_000475	Hs.268490	S74720

ZNF690	-5.8	1.8	zinc finger protein 690 (ZNF690), mRNA [NM_152455]	NM_152455	Hs.418287	NM_152455
LOC730452	-5.8	1.6	PREDICTED: similar to 60S ribosomal protein L7 (LOC730452), mRNA [XM_001125895]	XM_001125895	Unknown	
METTL9	-5.7	2.0	methyltransferase like 9 (METTL9), transcript variant 1, mRNA [NM_016025]	NM_016025	Hs.279583	NM_016025
BAHCC1	-5.7	2.7	BAH domain and coiled-coil containing 1, mRNA (cDNA clone IMAGE:5019335), partial cds. [BC033222]	BC033222	Hs.514580	NM_001080519
AB002449	-5.7	1.4	mRNA from chromosome 5q21-22, clone:843Ex. [AB002449]	AB002449	Hs.699267	AB002449
SEMA6D	-5.7	2.9	sema domain, transmembrane domain (TM), and cytoplasmic domain, (semaphorin) 6D (SEMA6D), transcript variant 4, mRNA [NM_153618]	NM_153618	Hs.511265	AF389429
BC004287	-5.7	2.5	Homo sapiens, clone IMAGE:3618365, mRNA. [BC004287]	BC004287	Hs.434957	BC004287
ZNF75	-5.7	1.5	zinc finger protein 75 (D8C6) (ZNF75), mRNA [NM_007131]	NM_007131	Hs.533540	NM_007131
FLJ10213	-5.7	2.1	hypothetical protein FLJ10213 (FLJ10213), mRNA [NM_018029]	NM_018029	Hs.658858	BC051316
A_24_P18676	-5.7	2.0	A_24_P186764	A_24_P186764	Unknown	
CD241953	-5.7	1.6	AGENCOURT_14122420 NIH_MGC_187 cDNA clone IMAGE:30381796 5', mRNA sequence [CD241953]	CD241953	Hs.592784	CD241953
A_24_P72410	-5.7	2.0	A_24_P724106	A_24_P724106	Unknown	
ZFP2	-5.7	2.2	zinc finger protein 2 homolog (mouse) (ZFP2), mRNA [NM_030613]	NM_030613	Hs.654533	BC142989
CR605444	-5.7	1.8	full-length cDNA clone CS0DK008YC22 of HeLa cells Cot 25-normalized of (human). [CR605444]	CR605444	Hs.536850	BC032641
PARP16	-5.7	2.3	poly (ADP-ribose) polymerase family, member 16 (PARP16), mRNA [NM_017851]	NM_017851	Hs.30634	BC006389
H3F3B	-5.7	2.9	H3 histone, family 3B (H3.3B) (H3F3B), mRNA [NM_005324]	NM_005324	Hs.180877	BX537379
THC2673888	-5.7	7.6	Q65549_9ALPH (Q65549) Glycoprotein C, partial (4%) [THC2673888]	THC2673888	Unknown	
MRE11A	-5.7	1.6	MRE11 meiotic recombination 11 homolog A (S. cerevisiae) (MRE11A), transcript variant 2, mRNA [NM_005590]	NM_005590	Hs.192649	NM_005590
SYNE2	-5.7	2.3	spectrin repeat containing, nuclear envelope 2 (SYNE2), transcript variant 1, mRNA [NM_015180]	NM_015180	Hs.525392	NM_182914
MRPL50	-5.7	2.1	mitochondrial ribosomal protein L50 [Source:RefSeq_peptide;Acc:NP_061924] [ENST00000374865]	ENST0000037486	Unknown	
LOC286334	-5.7	1.8	mRNA full length insert cDNA clone EUROIMAGE 1517766. [AJ420454]	AJ420454	Hs.349208	NM_133374
LOC283658	-5.7	3.5	cDNA FLJ30808 fis, clone FEBRA2001383. [AK055370]	AK055370	Hs.87194	AL833463
CD1B	-5.7	3.4	CD1b molecule (CD1B), mRNA [NM_001764]	NM_001764	Hs.1310	NM_001764
USP47	-5.7	1.9	ubiquitin specific peptidase 47 (USP47), mRNA [NM_017944]	NM_017944	Hs.577256	NM_017944
GRRP1	-5.7	3.3	glycine/arginine rich protein 1 (GRRP1), mRNA [NM_024869]	NM_024869	Hs.694119	NM_024869
PGAP1	-5.7	2.9	GPI deacylase (PGAP1), mRNA [NM_024989]	NM_024989	Hs.229988	NM_024989
IGBP1	-5.7	1.4	immunoglobulin (CD79A) binding protein 1 (IGBP1), mRNA [NM_001551]	NM_001551	Hs.496267	AK054596
PTN	-5.7	2.4	pleiotrophin (heparin binding growth factor 8, neurite growth-promoting factor 1) (PTN), mRNA [NM_002825]	NM_002825	Hs.371249	CR624136
THC2645690	-5.7	2.2	BX089289 BX089289 Soares fetal liver spleen 1NFLS cDNA clone IMAGp998O17111 ; IMAGE:120808, mRNA sequence [BX089289]	THC2645690	Unknown	
ENST0000024	-5.7	1.9	Zinc finger CCCH domain-containing protein 13. [Source:Uniprot/SWISSPROT;Acc:Q5T200] [ENST00000242848]	ENST0000024284	Unknown	
LMO3	-5.7	3.7	LIM domain only 3 (rhombotin-like 2) (LMO3), transcript variant 1, mRNA [NM_018640]	NM_018640	Hs.504908	NM_018640
CD34	-5.7	27.8	CD34 molecule (CD34), transcript variant 2, mRNA [NM_001773]	NM_001773	Hs.374990	BX640941
MGC70863	-5.7	1.7	similar to RPL23AP7 protein (MGC70863), transcript variant 1, mRNA [NM_203477]	NM_203477	Hs.406135	BC065556
CDC25C	-5.7	2.0	cell division cycle 25 homolog C (S. cerevisiae) (CDC25C), transcript variant 1, mRNA [NM_001790]	NM_001790	Hs.656	NM_001790
BAIAP2L2	-5.7	2.2	BAI1-associated protein 2-like 2 (BAIAP2L2), mRNA [NM_025045]	NM_025045	Hs.474822	NM_025045
DKFZP586K15	-5.7	3.9	mRNA; cDNA DKFZp586K1520 (from clone DKFZp586K1520) [AL050153]	AL050153	Hs.516864	AL050153
PRIM1	-5.7	2.4	primase, polypeptide 1, 49kDa (PRIM1), mRNA [NM_000946]	NM_000946	Hs.534339	NM_000946
BDH2	-5.7	1.9	3-hydroxybutyrate dehydrogenase, type 2 (BDH2), mRNA [NM_020139]	NM_020139	Hs.124696	NM_020139
CSTF1	-5.7	1.7	cleavage stimulation factor, 3' pre-RNA, subunit 1, 50kDa (CSTF1), transcript variant 2, mRNA [NM_001324]	NM_001324	Hs.172865	NM_001324
HHIP	-5.7	6.2	cDNA FLJ90230 fis, clone NT2RM2000410. [AK074711]	AK074711	Hs.507991	AY009951
RNF150	-5.7	2.8	ring finger protein 150 (RNF150), mRNA [NM_020724]	NM_020724	Hs.659104	AB033040
VPS13A	-5.7	2.4	Vacuolar protein sorting-associated protein 13A (Chorein) (Chorea- acanthocytosis protein). [Source:Uniprot/SWISSPROT;Acc:Q96RL7] [ENST00000376646]	ENST0000037664	Unknown	
RP4-691N24.1	-5.7	1.9	KIAA0980 protein (KIAA0980), mRNA [NM_025176]	NM_025176	Hs.696157	NM_025176
FUT10	-5.7	2.4	fucosyltransferase 10 (alpha (1,3) fucosyltransferase) (FUT10), mRNA [NM_032664]	NM_032664	Hs.458713	BC063462
LOC389831	-5.7	2.2	mRNA; cDNA DKFZp667B1610 (from clone DKFZp667B1610). [AL713796]	AL713796	Hs.389638	AL832779
LOC731681	-5.7	1.6	PREDICTED: similar to 60S ribosomal protein L9 (LOC731681), mRNA [XR_015944]	XR_015944	Hs.646365	XR_015944
C5	-5.7	2.8	complement component 5 (C5), mRNA [NM_001735]	NM_001735	Hs.494997	AB209031
BX538248	-5.7	1.8	mRNA; cDNA DKFZp686O04200 (from clone DKFZp686O04200). [BX538248]	BX538248	Hs.159188	BX538248
THC2501636	-5.7	1.4	ALU1_HUMAN (P39188) Alu subfamily J sequence contamination warning entry, partial (14%) [THC2501636]	THC2501636	Unknown	

ENST0000032	-5.7	2.1	PREDICTED: similar to large subunit ribosomal protein L36a (LOC284230), mRNA [XM_208185]	ENST0000032978	Unknown	
C21orf66	-5.7	2.0	cDNA clone IMAGE:5497083, containing frame-shift errors. [BC062992]	BC062992	Hs.700618	BC062992
ENST0000035	-5.7	2.0	Sequence 237 from Patent WO0220754. [AX721277]	ENST0000035944	Unknown	
ETV1	-5.7	5.1	ets variant gene 1 (ETV1), mRNA [NM_004956]	NM_004956	Hs.22634	NM_004956
DMTF1	-5.7	1.6	cyclin D binding myb-like transcription factor 1 (DMTF1), mRNA [NM_021145]	NM_021145	Hs.654981	AK126664
BU153693	-5.7	2.2	AGENCOURT_7782569 NIH_MGC_67 cDNA clone IMAGE:6137082 5', mRNA sequence [BU153693]	BU153693	Hs.587037	BU153693
EMID1	-5.7	1.8	EMI domain containing 1 (EMID1), mRNA [NM_133455]	NM_133455	Hs.289106	BC046358
TAGAP	-5.7	3.7	T-cell activation GTPase activating protein (TAGAP), transcript variant 2, mRNA [NM_054114]	NM_054114	Hs.529984	NM_054114
CCNG2	-5.7	2.2	cyclin G2 (CCNG2), mRNA [NM_004354]	NM_004354	Hs.13291	BC032518
BM928667	-5.7	5.7	AGENCOURT_6726860 NIH_MGC_100 cDNA clone IMAGE:5798808 5', mRNA sequence [BM928667]	BM928667	Hs.113170	BM928667
GNL3L	-5.7	2.5	guanine nucleotide binding protein-like 3 (nucleolar)-like (GNL3L), mRNA [NM_019067]	NM_019067	Hs.654677	NM_019067
BRWD1	-5.6	2.5	Bromodomain and WD repeat domain-containing protein 1 (WD repeat protein 9). [Source:Uniprot/SWISSPROT;Acc:Q9NSI6] [ENST00000380831]	ENST0000038083	Unknown	
KLF3	-5.6	2.5	Krueppel-like factor 3 (Basic krueppel-like factor) (CACCC-box-binding protein BKLF) (TEF-2). [Source:Uniprot/SWISSPROT;Acc:P57682] [ENST00000381956]	ENST0000038195	Unknown	
RAD54B	-5.6	2.8	fibrinogen silencer binding protein mRNA, complete cds. [AF007866]	AF007866	Hs.30561	NM_012415
GNG13	-5.6	5.2	guanine nucleotide binding protein (G protein), gamma 13 (GNG13), mRNA [NM_016541]	NM_016541	Hs.247888	AB030207
RPS25	-5.6	1.8	ribosomal protein S25 (RPS25), mRNA [NM_001028]	NM_001028	Hs.512676	BM554735
AK091357	-5.6	3.4	cDNA FLJ34038 fis, clone FCBBF2005645. [AK091357]	AK091357	Hs.587465	AK091357
RNF175	-5.6	4.2	ring finger protein 175 (RNF175), mRNA [NM_173662]	NM_173662	Hs.388364	AK091509
HMGA2	-5.6	3.5	high mobility group AT-hook 2 (HMGA2), transcript variant 1, mRNA [NM_003483]	NM_003483	Hs.505924	AB209853
MOXD1	-5.6	2.5	monooxygenase, DBH-like 1 (MOXD1), transcript variant 2, mRNA [NM_015529]	NM_015529	Hs.6909	NM_015529
NAB1	-5.6	1.6	NGFI-A binding protein 1 (EGR1 binding protein 1) (NAB1), mRNA [NM_005966]	NM_005966	Hs.570078	NM_005966
MGC51025	-5.6	1.9	hypothetical protein MGC51025 (MGC51025), mRNA [NM_178571]	NM_178571	Hs.567880	BC028414
DCP1A	-5.6	1.5	DCP1 decapping enzyme homolog A (S. cerevisiae) (DCP1A), mRNA [NM_018403]	NM_018403	Hs.476353	NM_018403
ADAMTS3	-5.6	3.5	ADAM metallopeptidase with thrombospondin type 1 motif, 3 (ADAMTS3), mRNA [NM_014243]	NM_014243	Hs.590919	NM_014243
FZD7	-5.6	3.1	frizzled homolog 7 (Drosophila) (FZD7), mRNA [NM_003507]	NM_003507	Hs.173859	AB017365
ZNF616	-5.6	2.8	zinc finger protein 616, mRNA (cDNA clone MGC:45556 IMAGE:4186857), complete cds. [BC032805]	BC032805	Hs.645225	BM544555
ADH5	-5.6	1.7	alcohol dehydrogenase 5 (class III), chi polypeptide (ADH5), mRNA [NM_000671]	NM_000671	Hs.78989	AK226177
A_24_P36719	-5.6	1.8	A_24_P367199	A_24_P367199	Unknown	
EFCAB4A	-5.6	1.9	EF-hand calcium binding domain 4A (EFCAB4A), mRNA [NM_173584]	NM_173584	Hs.660936	NM_173584
OR5T2	-5.6	2.0	cDNA FLJ25625 fis, clone STM02974. [AK098491]	AK098491	Hs.462086	BC062623
ZNF555	-5.6	1.6	zinc finger protein 555 (ZNF555), mRNA [NM_152791]	NM_152791	Hs.47712	AL832140
PRIM1	-5.6	2.4	primase, polypeptide 1, 49kDa (PRIM1), mRNA [NM_000946]	NM_000946	Hs.534339	NM_000946
CN333321	-5.6	4.7	17000532577741 GRN_ES cDNA 5', mRNA sequence [CN333321]	CN333321	Hs.603772	CN333321
AK127132	-5.6	1.8	cDNA FLJ45189 fis, clone BRAWH3049068. [AK127132]	AK127132	Hs.596399	AK127132
A_32_P49392	-5.6	1.8	A_32_P49392	A_32_P49392	Unknown	
BTN2A2	-5.6	1.9	butyrophilin, subfamily 2, member A2 (BTN2A2), transcript variant 2, mRNA [NM_181531]	NM_181531	Hs.373938	U90550
LOC399818	-5.6	1.6	similar to CG9643-PA (LOC399818), mRNA [NM_212554]	NM_212554	Hs.468488	AK022354
PTBP2	-5.6	1.9	polypyrimidine tract binding protein 2 (PTBP2), mRNA [NM_021190]	NM_021190	Hs.591430	AB209266
CTDSP1	-5.6	1.7	CTD (carboxy-terminal domain, RNA polymerase II, polypeptide A) small phosphatase 1 (CTDSP1), transcript variant 1, mRNA [NM_021198]	NM_021198	Hs.444468	AF229162
CDH3	-5.6	4.2	cadherin 3, type 1, P-cadherin (placental) (CDH3), mRNA [NM_001793]	NM_001793	Hs.461074	BC040486
NTF3	-5.6	2.4	neurotrophin 3 (NTF3), mRNA [NM_002527]	NM_002527	Hs.99171	NM_002527
MRPL45	-5.6	1.9	mitochondrial ribosomal protein L45 (MRPL45), nuclear gene encoding mitochondrial protein, mRNA [NM_032351]	NM_032351	Hs.462913	NM_032351
THC2643265	-5.6	2.9	ALU1_HUMAN (P39188) Alu subfamily J sequence contamination warning entry, partial (5%) [THC2643265]	THC2643265	Unknown	
HAPLN4	-5.6	2.2	hyaluronan and proteoglycan link protein 4 (HAPLN4), mRNA [NM_023002]	NM_023002	Hs.367829	BC142698
THC2575678	-5.6	2.8	Q753E4_ASHGO (Q753E4) AFR372Wp, partial (3%) [THC2575678]	THC2575678	Unknown	
KIAA1407	-5.6	1.9	KIAA1407 (KIAA1407), mRNA [NM_020817]	NM_020817	Hs.477159	AF509494
FAM76B	-5.6	1.9	family with sequence similarity 76, member B (FAM76B), mRNA [NM_144664]	NM_144664	Hs.288304	NM_144664
A_32_P14515	-5.6	1.9	A_32_P145159	A_32_P145159	Unknown	
MLLT10	-5.6	2.0	myeloid/lymphoid or mixed-lineage leukemia (trithorax homolog, Drosophila); translocated to, 10 (MLLT10), transcript variant 1, mRNA [NM_004641]	NM_004641	Hs.30385	AB209755
AK094525	-5.6	1.9	cDNA FLJ37206 fis, clone BRALZ2007545. [AK094525]	AK094525	Hs.587467	BX647732

PAH	-5.6	6.1	phenylalanine hydroxylase (PAH), mRNA [NM_000277]	NM_000277	Hs.643451	NM_000277
THC2549494	-5.6	1.9	ALU6_HUMAN (P39193) Alu subfamily SP sequence contamination warning entry, partial (11%) [THC2549494]	THC2549494	Unknown	
FLI1	-5.6	6.1	Friend leukemia virus integration 1 (FLI1), mRNA [NM_002017]	NM_002017	Hs.504281	BX647094
BC069749	-5.6	3.1	cDNA clone IMAGE:7262596, with apparent retained intron. [BC069749]	BC069749	Hs.16229	BX649096
KCNQ2	-5.6	3.6	potassium voltage-gated channel, KQT-like subfamily, member 2 (KCNQ2), transcript variant 5, mRNA [NM_172109]	NM_172109	Hs.161851	NM_172107
DPY19L1P1	-5.6	1.8	cDNA: FLJ23115 fis, clone LNG07933. [AK026768]	AK026768	Hs.633705	AK026768
ENST0000030	-5.6	7.1	ENST00000303979	ENST0000030397	Unknown	
AL080082	-5.5	3.5	mRNA; cDNA DKFZp564G1162 (from clone DKFZp564G1162). [AL080082]	AL080082	Hs.598166	AL080082
KIAA0644	-5.5	2.9	KIAA0644 gene product (KIAA0644), mRNA [NM_014817]	NM_014817	Hs.21572	NM_014817
RPL13	-5.5	1.8	60S ribosomal protein L13 (Breast basic conserved protein 1). [Source:Uniprot/SWISSPROT;Acc:P26373] [ENST00000311528]	ENST0000031152	Unknown	
PRDM5	-5.5	2.3	PR domain containing 5 (PRDM5), mRNA [NM_018699]	NM_018699	Hs.660435	NM_018699
AK022793	-5.5	1.8	cDNA FLJ12731 fis, clone NT2RP2000108. [AK022793]	AK022793	Hs.162105	BC043213
DKFZp762P21	-5.5	1.8	cDNA FLJ12914 fis, clone NT2RP2004523. [AK022976]	AK022976	Hs.490512	NM_001004302
A_32_P13081	-5.5	2.4	A_32_P13081	A_32_P13081	Unknown	
TNNC2	-5.5	3.2	troponin C type 2 (fast) (TNNC2), mRNA [NM_003279]	NM_003279	Hs.182421	BF574800
SOX11	-5.5	2.5	SRY (sex determining region Y)-box 11 (SOX11), mRNA [NM_003108]	NM_003108	Hs.432638	AB028641
FAM13A1	-5.5	2.2	family with sequence similarity 13, member A1 (FAM13A1), transcript variant 1, mRNA [NM_014883]	NM_014883	Hs.97270	NM_014883
THC2685727	-5.5	1.7	THC2685727	THC2685727	Unknown	
AK124953	-5.5	1.5	cDNA FLJ42963 fis, clone BRSTN2012380. [AK124953]	AK124953	Hs.404449	AK124953
LOC646980	-5.5	1.7	PREDICTED: similar to 60S ribosomal protein L7a (LOC646980), mRNA [XR_018451]	XR_018451	Hs.693429	XR_018451
ACF	-5.5	2.4	apobec-1 complementation factor (ACF), transcript variant 3, mRNA [NM_138933]	NM_138933	Hs.499643	NM_138933
AK024956	-5.5	3.4	cDNA: FLJ21303 fis, clone COL02107. [AK024956]	AK024956	Hs.677304	AK024956
DYRK1A	-5.5	1.7	dual-specificity tyrosine-(Y)-phosphorylation regulated kinase 1A (DYRK1A), transcript variant 2, mRNA [NM_130436]	NM_130436	Hs.696102	NM_130436
LOC152573	-5.5	110.1	Homo sapiens, clone IMAGE:4477067, mRNA, partial cds. [BC012029]	BC012029	Hs.370904	NM_001080505
A_32_P18441	-5.5	3.3	A_32_P184417	A_32_P184417	Unknown	
KIAA1731	-5.5	1.8	mRNA for KIAA1731 protein, partial cds. [AB051518]	AB051518	Hs.458418	AB051518
CR590573	-5.5	2.6	full-length cDNA clone CS0DI042YD07 of Placenta Cot 25-normalized of (human). [CR590573]	CR590573	Hs.484967	CR590573
ANK3	-5.5	1.7	ankyrin 3, node of Ranvier (ankyrin G) (ANK3), transcript variant 1, mRNA [NM_020987]	NM_020987	Hs.499725	NM_020987
THC2508355	-5.5	2.5	Q25GJ8_MACFA (Q25GJ8) Brain cDNA, clone: QflA-22015, complete [THC2508355]	THC2508355	Unknown	
ENST0000037	-5.5	1.5	kinesin family member 27 [Source:RefSeq_peptide;Acc:NP_060046] [ENST00000376347]	ENST0000037634	Unknown	
ENST0000026	-5.5	2.5	52 kDa repressor of the inhibitor of the protein kinase (p58IPK- interacting protein) (58 kDa interferon-induced protein kinase- interacting protein) (P52rIPK) (Death-associated protein 4) (THAP domain-containing protein 0)....	ENST0000026004	Unknown	
ZNF235	-5.5	1.9	zinc finger protein 235 (ZNF235), mRNA [NM_004234]	NM_004234	Hs.298089	AK226126
THC2672257	-5.5	4.6	THC2672257	THC2672257	Unknown	
A_24_P84408	-5.5	1.6	A_24_P84408	A_24_P84408	Unknown	
AK024870	-5.5	1.9	cDNA: FLJ21217 fis, clone COL00536. [AK024870]	AK024870	Hs.173135	NM_006482
AK055501	-5.5	2.2	cDNA FLJ30939 fis, clone FEBRA2007414. [AK055501]	AK055501	Hs.381219	BC081565
SGEF	-5.5	2.0	infant liver cDNA, clone:HMFN1864, full insert sequence. [AB073386]	AB073386	Hs.570682	AK127282
BC031320	-5.5	2.1	cDNA clone IMAGE:5278682. [BC031320]	BC031320	Hs.220864	NM_001271
PRIM1	-5.5	2.4	primase, polypeptide 1, 49kDa (PRIM1), mRNA [NM_000946]	NM_000946	Hs.534339	NM_000946
A_24_P33525	-5.5	2.2	A_24_P33525	A_24_P33525	Unknown	
CCT6B	-5.5	2.7	chaperonin containing TCP1, subunit 6B (zeta 2) (CCT6B), mRNA [NM_006584]	NM_006584	Hs.73072	CR933688
DTL	-5.5	1.9	denticleless homolog (Drosophila) (DTL), mRNA [NM_016448]	NM_016448	Hs.656473	NM_016448
SUZ12	-5.5	2.1	suppressor of zeste 12 homolog (Drosophila) (SUZ12), mRNA [NM_015355]	NM_015355	Hs.462732	D63881
LOC643220	-5.5	1.9	PREDICTED: similar to ribosomal protein L3 isoform a (LOC643220), mRNA [XR_016530]	XR_016530	Hs.646960	XR_016530
LCOR	-5.5	1.4	ligand dependent nuclear receptor corepressor (LCOR), mRNA [NM_032440]	NM_032440	Hs.500695	AL834245
A_32_P17104	-5.5	4.7	A_32_P171043	A_32_P171043	Unknown	
C14orf32	-5.5	1.7	chromosome 14 open reading frame 32 (C14orf32), mRNA [NM_144578]	NM_144578	Hs.594338	NM_144578
ENST0000032	-5.5	3.0	Zinc finger CCHC domain-containing protein 7. [Source:Uniprot/SWISSPROT;Acc:Q8N3Z6] [ENST00000322831]	ENST0000032283	Unknown	
APOC1	-5.5	3.7	apolipoprotein C-I (APOC1), mRNA [NM_001645]	NM_001645	Hs.110675	AJ249921
PIP5K3	-5.5	1.8	phosphatidylinositol-3-phosphate/phosphatidylinositol 5-kinase, type III (PIP5K3), transcript variant 2, mRNA [NM_015040]	NM_015040	Hs.173939	NM_015040

ANKRD12	-5.5	1.5	ankyrin repeat domain 12 (ANKRD12), mRNA [NM_015208]	NM_015208	Hs.464585	NM_015208
RMND5A	-5.5	1.6	mRNA; cDNA DKFZp586M141 (from clone DKFZp586M141). [AL050139]	AL050139	Hs.75277	AL832022
ZNF222	-5.5	1.6	zinc finger protein 222 (ZNF222), mRNA [NM_013360]	NM_013360	Hs.279840	AK095196
C9orf41	-5.5	1.7	mRNA; cDNA DKFZp686J16172 (from clone DKFZp686J16172). [BX538061]	ENST0000037683	Unknown	
CCDC41	-5.5	1.8	coiled-coil domain containing 41 (CCDC41), transcript variant 1, mRNA [NM_016122]	NM_016122	Hs.279209	NM_016122
ABCC2	-5.5	3.2	ATP-binding cassette, sub-family C (CFTR/MRP), member 2 (ABCC2), mRNA [NM_000392]	NM_000392	Hs.368243	U49248
THC2505214	-5.5	3.8	THC2505214	THC2505214	Unknown	
A_24_P16092	-5.5	1.9	A_24_P160920	A_24_P160920	Unknown	
CPS1	-5.5	7.0	carbamoyl-phosphate synthetase 1, mitochondrial (CPS1), mRNA [NM_001875]	NM_001875	Hs.149252	NM_001875
WFIKKN1	-5.5	5.3	WAP, follistatin/kazal, immunoglobulin, kunitz and netrin domain containing 1 (WFIKKN1), mRNA [NM_053284]	NM_053284	Hs.345818	AK075356
C12orf35	-5.5	4.0	chromosome 12 open reading frame 35 (C12orf35), mRNA [NM_018169]	NM_018169	Hs.445129	NM_018169
MGC3032	-5.5	2.5	cDNA FLJ38987 fis, clone NT2RI2005818. [AK096306]	AK096306	Hs.568945	XM_933539
THC2666219	-5.5	4.3	Q7Z6P5_HUMAN (Q7Z6P5) MCM3 minichromosome maintenance deficient 3 (S. cerevisiae) (Fragment), partial (6%) [THC2666219]	THC2666219	Unknown	
C3orf9	-5.5	1.8	chromosome 3 open reading frame 9 (C3orf9), mRNA [NM_152305]	NM_152305	Hs.231750	AK126736
TMEM128	-5.5	1.7	transmembrane protein 128 (TMEM128), mRNA [NM_032927]	NM_032927	Hs.12845	CR933611
DDX50	-5.5	1.6	DEAD (Asp-Glu-Ala-Asp) box polypeptide 50 (DDX50), mRNA [NM_024045]	NM_024045	Hs.522984	BC000272
THC2597502	-5.5	1.9	BC002110 Rpl24 protein {Mus musculus} (exp=-1; wgp=0; cg=0), partial (82%) [THC2597502]	THC2597502	Unknown	
FLJ10232	-5.5	4.9	cDNA FLJ10232 fis, clone HEMBB1000244. [AK001094]	AK001094	Hs.551150	AK001094
BCLAF1	-5.5	5.3	BCL2-associated transcription factor 1 (BCLAF1), transcript variant 1, mRNA [NM_014739]	NM_014739	Hs.486542	NM_014739
FLJ10154	-5.5	1.8	hypothetical protein FLJ10154 (FLJ10154), mRNA [NM_018011]	NM_018011	Hs.508644	CR936748
AK026718	-5.5	3.8	cDNA: FLJ23065 fis, clone LNG04894. [AK026718]	AK026718	Hs.125352	AK026718
FLJ11236	-5.4	1.7	cDNA FLJ11236 fis, clone PLACE1008524. [AK002098]	AK002098	Hs.374076	BX537595
PARD3	-5.4	1.6	atypical PKC isotype-specific interacting protein long variant mRNA, complete cds. [AF196185]	AF196185	Hs.131489	AF196185
LOC646161	-5.4	1.6	PREDICTED: similar to 60S ribosomal protein L26 (LOC646161), mRNA [XR_018048]	XR_018048	Hs.648093	XR_018048
A_24_P84355	-5.4	2.3	A_24_P843552	A_24_P843552	Unknown	
C9orf102	-5.4	2.3	chromosome 9 open reading frame 102 (C9orf102), transcript variant 1, mRNA [NM_020207]	NM_020207	Hs.632686	NM_020207
THC2629174	-5.4	2.2	THC2629174	THC2629174	Unknown	
TTC32	-5.4	2.4	tetratricopeptide repeat domain 32 (TTC32), mRNA [NM_001008237]	NM_001008237	Hs.591547	BC050088
THNSL1	-5.4	1.8	Threonine synthase-like 1. [Source:Uniprot/SWISSPROT;Acc:Q8IYQ7] [ENST00000376356]	ENST0000037635	Unknown	
THC2655811	-5.4	3.1	THC2655811	THC2655811	Unknown	
CR613654	-5.4	2.0	full-length cDNA clone CS0DN005YK16 of Adult brain of (human). [CR613654]	CR613654	Hs.672807	CR613654
THC2583971	-5.4	2.0	Q5PR09_MOUSE (Q5PR09) Ribosomal protein L32, partial (87%) [THC2583971]	THC2583971	Unknown	
TCF12	-5.4	2.1	transcription factor 12 (HTF4, helix-loop-helix transcription factors 4) (TCF12), transcript variant 4, mRNA [NM_207038]	NM_207038	Hs.511504	NM_207037
SP5	-5.4	5.5	Sp5 transcription factor (SP5), mRNA [NM_001003845]	NM_001003845	Hs.368802	AB096175
TTC28	-5.4	2.2	mRNA for KIAA1043 protein, partial cds. [AB028966]	AB028966	Hs.387856	XM_929318
CCDC102B	-5.4	6.0	coiled-coil domain containing 102B (CCDC102B), mRNA [NM_024781]	NM_024781	Hs.280781	NM_001093729
A_24_P35820	-5.4	1.7	A_24_P358205	A_24_P358205	Unknown	
LXN	-5.4	2.6	latexin (LXN), mRNA [NM_020169]	NM_020169	Hs.478067	NM_020169
PRIM1	-5.4	2.4	primase, polypeptide 1, 49kDa (PRIM1), mRNA [NM_000946]	NM_000946	Hs.534339	NM_000946
KIAA1274	-5.4	4.9	KIAA1274 (KIAA1274), mRNA [NM_014431]	NM_014431	Hs.202351	AB033100
ARHGAP19	-5.4	1.8	Rho GTPase activating protein 19 (ARHGAP19), mRNA [NM_032900]	NM_032900	Hs.80305	AK090447
A_24_P35839	-5.4	2.4	A_24_P358390	A_24_P358390	Unknown	
VAPA	-5.4	1.7	VAMP (vesicle-associated membrane protein)-associated protein A, 33kDa (VAPA), transcript variant 1, mRNA [NM_003574]	NM_003574	Hs.165195	NM_003574
A_24_P33229	-5.4	2.2	A_24_P332292	A_24_P332292	Unknown	
CCDC77	-5.4	2.1	coiled-coil domain containing 77 (CCDC77), mRNA [NM_032358]	NM_032358	Hs.631656	AK027638
LOC342994	-5.4	1.7	PREDICTED: similar to ribosomal protein L34 (LOC342994), mRNA [XM_938484]	ENST0000031362	Unknown	
WDR7	-5.4	1.5	WD repeat domain 7 (WDR7), transcript variant 1, mRNA [NM_015285]	NM_015285	Hs.465213	NM_015285
PCF11	-5.4	2.1	PCF11, cleavage and polyadenylation factor subunit, homolog (S. cerevisiae) (PCF11), mRNA [NM_015885]	NM_015885	Hs.128959	BC146778
BCLAF1	-5.4	2.0	BCL2-associated transcription factor 1 (BCLAF1), transcript variant 1, mRNA [NM_014739]	NM_014739	Hs.486542	NM_014739
RPL34	-5.4	2.1	ribosomal protein L34 (RPL34), transcript variant 2, mRNA [NM_033625]	NM_033625	Hs.438227	BG112770

EIF3S3	-5.4	1.7	eukaryotic translation initiation factor 3, subunit 3 gamma, 40kDa (EIF3S3), mRNA [NM_003756]	NM_003756	Hs.492599	AK093128
UBE2L6	-5.4	1.8	ubiquitin-conjugating enzyme E2L 6 (UBE2L6), transcript variant 2, mRNA [NM_198183]	NM_198183	Hs.425777	AK093462
ZNF614	-5.4	2.4	zinc finger protein 614 (ZNF614), mRNA [NM_025040]	NM_025040	Hs.292336	NM_025040
CDKN1B	-5.4	1.5	cyclin-dependent kinase inhibitor 1B (p27, Kip1) (CDKN1B), mRNA [NM_004064]	NM_004064	Hs.238990	NM_004064
FOXC1	-5.4	3.5	forkhead box C1 (FOXC1), mRNA [NM_001453]	NM_001453	Hs.348883	NM_001453
CHD9	-5.4	1.9	chromodomain helicase DNA binding protein 9 (CHD9), mRNA [NM_025134]	NM_025134	Hs.59159	DQ333316
AL517609	-5.4	3.0	AL517609 NEUROBLASTOMA cDNA clone CS0DA003YM13 3-PRIME, mRNA sequence [AL517609]	AL517609	Hs.583741	AL517609
AF086375	-5.4	1.8	full length insert cDNA clone ZD68B12. [AF086375]	AF086375	Hs.264606	BG571805
ZFPM2	-5.4	2.7	zinc finger protein, multitype 2 (ZFPM2), mRNA [NM_012082]	NM_012082	Hs.431009	NM_012082
THC2669975	-5.4	2.6	THC2669975	THC2669975	Unknown	
AL049990	-5.4	5.0	mRNA; cDNA DKFZp564G112 (from clone DKFZp564G112). [AL049990]	AL049990	Hs.90250	NM_024574
THC2624002	-5.4	7.1	Q9BXR7_HUMAN (Q9BXR7) Interleukin 10 (Fragment), partial (93%) [THC2730601]	THC2624002	Unknown	
C9orf125	-5.4	1.6	chromosome 9 open reading frame 125 (C9orf125), mRNA [NM_032342]	NM_032342	Hs.655738	BC033550
ZNF543	-5.4	2.6	zinc finger protein 543 (ZNF543), mRNA [NM_213598]	NM_213598	Hs.202544	AK131547
EPHA7	-5.4	2.8	Ephrin type-A receptor 7 precursor (EC 2.7.10.1) (Tyrosine-protein kinase receptor EHK-3) (EPH homology kinase 3) (Receptor protein- tyrosine kinase HEK11). [Source:Uniprot/SWISSPROT;Acc:Q15375] [ENST00000369303]	ENST0000036930	Unknown	
CHSY1	-5.4	1.5	carbohydrate (chondroitin) synthase 1 (CHSY1), mRNA [NM_014918]	NM_014918	Hs.110488	BC046247
LOC644907	-5.4	1.9	PREDICTED: similar to 60S ribosomal protein L32 (LOC644907), mRNA [XM_930195]	XM_930195	Unknown	
LOC439949	-5.4	2.5	clone CDABP0095 mRNA sequence. [AY007155]	AY007155	Hs.590987	XM_001128367
C14orf143	-5.4	2.4	chromosome 14 open reading frame 143 (C14orf143), mRNA [NM_145231]	NM_145231	Hs.123232	AL832321
AW235110	-5.3	1.9	xn18f11.x1 NCI_CGAP_Kid11 cDNA clone IMAGE:2694093 3' similar to gb:M65131 METHYLMALONYL-COA MUTASE PRECURSOR (HUMAN);, mRNA sequence [AW235110]	AW235110	Hs.653296	AW235110
CG018	-5.3	4.4	hypothetical gene CG018 (CG018), transcript variant 1, mRNA [NM_052818]	NM_052818	Hs.161220	BX648509
RPL21	-5.3	1.7	ribosomal protein L21 (RPL21), mRNA [NM_000982]	NM_000982	Hs.381123	CR602527
A_24_P36645	-5.3	2.1	A_24_P366457	A_24_P366457	Unknown	
PHF3	-5.3	2.0	PHD finger protein 3 (PHF3), mRNA [NM_015153]	NM_015153	Hs.348921	BX648268
THC2506656	-5.3	3.0	Q7T2T6_BOTJR (Q7T2T6) Ribosomal protein, partial (70%) [THC2506656]	THC2506656	Unknown	
PRIM1	-5.3	2.4	primase, polypeptide 1, 49kDa (PRIM1), mRNA [NM_000946]	NM_000946	Hs.534339	NM_000946
A_24_P36736	-5.3	2.1	A_24_P367369	A_24_P367369	Unknown	
TBC1D10C	-5.3	2.9	TBC1 domain family, member 10C (TBC1D10C), mRNA [NM_198517]	NM_198517	Hs.534648	AK122585
SNHG1	-5.3	2.0	small nucleolar RNA host gene (non-protein coding) 1 (SNHG1) on chromosome 11 [NR_003098]	NR_003098	Unknown	
ROPN1	-5.3	5.8	ropporin, rhophilin associated protein 1 (ROPN1), mRNA [NM_017578]	NM_017578	Hs.567516	AL133624
APRIN	-5.3	3.0	androgen-induced proliferation inhibitor (APRIN), mRNA [NM_015032]	NM_015032	Hs.699308	AL137201
POT1	-5.3	1.8	POT1 protection of telomeres 1 homolog (S. pombe) (POT1), transcript variant 1, mRNA [NM_015450]	NM_015450	Hs.31968	NM_015450
RPS7	-5.3	1.7	ribosomal protein S7 (RPS7), mRNA [NM_001011]	NM_001011	Hs.546287	AB209386
AK021664	-5.3	2.7	cDNA FLJ11602 fis, clone HEMBA1003908. [AK021664]	AK021664	Hs.653123	BX537526
RTF1	-5.3	1.7	Rtf1, Paf1/RNA polymerase II complex component, homolog (S. cerevisiae) (RTF1), mRNA [NM_015138]	NM_015138	Hs.511096	NM_015138
RPS11	-5.3	1.6	ribosomal protein S11 (RPS11), mRNA [NM_001015]	NM_001015	Hs.433529	AK130324
LOC388532	-5.3	1.8	PREDICTED: similar to ribosomal protein L21 (LOC388532), mRNA [XM_001127035]	ENST0000031082	Unknown	
A_24_P11887	-5.3	1.8	A_24_P118874	A_24_P118874	Unknown	
DTL	-5.3	1.9	denticleless homolog (Drosophila) (DTL), mRNA [NM_016448]	NM_016448	Hs.656473	NM_016448
PRIM1	-5.3	2.4	primase, polypeptide 1, 49kDa (PRIM1), mRNA [NM_000946]	NM_000946	Hs.534339	NM_000946
C5orf25	-5.3	2.2	chromosome 5 open reading frame 25 (C5orf25), mRNA [NM_198567]	NM_198567	Hs.699402	BC037298
PLCH1	-5.3	3.9	phospholipase C, eta 1 (PLCH1), mRNA [NM_014996]	NM_014996	Hs.567423	AY691170
A_24_P41871	-5.3	1.6	A_24_P418712	A_24_P418712	Unknown	
SLC24A1	-5.3	2.6	solute carrier family 24 (sodium/potassium/calcium exchanger), member 1 (SLC24A1), mRNA [NM_004727]	NM_004727	Hs.173092	NM_004727
LOC120376	-5.3	4.5	hypothetical protein LOC120376, mRNA (cDNA clone IMAGE:40030426), partial cds. [BC110079]	BC110079	Hs.31409	AL831866
AK002023	-5.3	1.7	cDNA FLJ11161 fis, clone PLACE1007021. [AK002023]	AK002023	Hs.368518	AK002023
MSH2	-5.3	1.6	mutS homolog 2, colon cancer, nonpolyposis type 1 (E. coli) (MSH2), mRNA [NM_000251]	NM_000251	Hs.597656	AK223284
THC2617409	-5.3	2.8	Q6F0E0_MESFL (Q6F0E0) Recombination protein, partial (8%) [THC2617409]	THC2617409	Unknown	
AK096401	-5.3	1.9	cDNA FLJ39082 fis, clone NT2RP7018586. [AK096401]	AK096401	Hs.696073	XR_018616

A_24_P87838	-5.3	2.1	A_24_P878388	A_24_P878388	Unknown	
BM802662	-5.3	2.3	AGENCOURT_6460073 NIH_MGC_67 cDNA clone IMAGE:5581075 5', mRNA sequence [BM802662]	BM802662	Hs.586812	BM802662
MDS1	-5.3	6.5	myelodysplasia syndrome 1 (MDS1), mRNA [NM_004991]	NM_004991	Hs.659873	U43293
CD174733	-5.3	1.8	CD174733 AGENCOURT_13961604 NIH_MGC_172 cDNA 5', mRNA sequence [CD174733]	CD174733	Hs.265174	CR596982
THC2582042	-5.3	2.7	BC031749 potassium channel tetramerisation domain containing 15 {Mus musculus} (exp=-1; wgp=0; cg=0), partial (10%) [THC2582042]	THC2582042	Unknown	
GJB2	-5.3	2.5	gap junction protein, beta 2, 26kDa (connexin 26) (GJB2), mRNA [NM_004004]	NM_004004	Hs.591234	NM_004004
CEP290	-5.3	2.3	centrosomal protein 290kDa (CEP290), mRNA [NM_025114]	NM_025114	Hs.150444	NM_025114
RBM12B	-5.3	2.0	RNA binding motif protein 12B (RBM12B), mRNA [NM_203390]	NM_203390	Hs.192788	NM_203390
LOC730887	-5.3	2.2	PREDICTED: similar to 40S ribosomal protein S7 (S8) (LOC730887), mRNA [XR_015607]	XR_015607	Hs.646897	XR_015607
KIAA1712	-5.3	2.4	KIAA1712, mRNA (cDNA clone MGC:33587 IMAGE:4823994), complete cds. [BC038667]	BC038667	Unknown	
HS3ST3A1	-5.3	2.2	heparan sulfate (glucosamine) 3-O-sulfotransferase 3A1 (HS3ST3A1), mRNA [NM_006042]	NM_006042	Hs.462270	NM_006042
GON4L	-5.3	1.8	gon-4-like (C. elegans) (GON4L), transcript variant 2, mRNA [NM_032292]	NM_032292	Hs.656361	AY335491
A_24_P20445	-5.3	1.9	A_24_P204454	A_24_P204454	Unknown	
STOX1	-5.3	2.2	storkhead box 1 (STOX1), mRNA [NM_152709]	NM_152709	Hs.37636	AY842014
DEADC1	-5.3	2.3	deaminase domain containing 1 (DEADC1), mRNA [NM_182503]	NM_182503	Hs.643943	NM_182503
LOC645676	-5.3	1.9	cDNA FLJ44595 fis, clone BLADE2004849. [AK126559]	AK126559	Hs.701419	AK126559
ZNF721	-5.3	1.8	zinc finger protein 721 (ZNF721), mRNA [NM_133474]	NM_133474	Hs.428360	NM_133474
A_24_P32836	-5.3	1.6	A_24_P32836	A_24_P32836	Unknown	
PDE11A	-5.3	2.7	phosphodiesterase 11A (PDE11A), transcript variant 4, mRNA [NM_016953]	NM_016953	Hs.570273	NM_016953
PRKRIR	-5.3	2.3	protein-kinase, interferon-inducible double stranded RNA dependent inhibitor, repressor of (P58 repressor) (PRKRIR), mRNA [NM_004705]	NM_004705	Hs.503315	BX641144
SP3	-5.3	2.0	Sp3 transcription factor (SP3), transcript variant 1, mRNA [NM_003111]	NM_003111	Hs.531587	AB209334
ZNF167	-5.3	2.2	zinc finger protein 167 (ZNF167), transcript variant 1, mRNA [NM_018651]	NM_018651	Hs.529512	AY280798
SPIN	-5.3	1.9	spindlin (SPIN), mRNA [NM_006717]	NM_006717	Hs.146804	NM_006717
THC2530793	-5.3	1.7	CB850536 UI-CF-EN1-acw-k-22-0-UI.s1 UI-CF-EN1 cDNA clone UI-CF-EN1-acw-k-22-0-UI 3', mRNA sequence [CB850536]	THC2530793	Unknown	
SORBS1	-5.3	2.0	sorbin and SH3 domain containing 1 (SORBS1), transcript variant 3, mRNA [NM_001034954]	NM_001034954	Hs.696027	AM260536
SLC44A1	-5.3	3.2	Choline transporter-like protein 1 (Solute carrier family 44 member 1) (CD92 antigen) (CDw92). [Source:Uniprot/SWISSPROT;Acc:Q8WWI5] [ENST00000361080]	ENST0000036108	Unknown	
ZNF221	-5.3	4.1	zinc finger protein 221 (ZNF221), mRNA [NM_013359]	NM_013359	Hs.631598	NM_013359
KIAA1549	-5.3	1.9	mRNA; cDNA DKFZp434O0710 (from clone DKFZp434O0710). [AL136736]	AL136736	Hs.605380	NM_020910
DARS	-5.3	1.9	aspartyl-tRNA synthetase (DARS), mRNA [NM_001349]	NM_001349	Hs.503787	NM_001349
VPRBP	-5.3	2.8	Vpr (HIV-1) binding protein (VPRBP), mRNA [NM_014703]	NM_014703	Hs.118738	AB018343
5-Mar	-5.3	1.8	membrane-associated ring finger (C3HC4) 5 (MARCH5), mRNA [NM_017824]	NM_017824	Hs.573490	NM_017824
KIAA1109	-5.3	1.7	KIAA1109, mRNA (cDNA clone IMAGE:3924668), complete cds. [BC108274]	BC108274	Hs.408142	DQ335469
POLD3	-5.2	1.5	polymerase (DNA-directed), delta 3, accessory subunit (POLD3), mRNA [NM_006591]	NM_006591	Hs.82502	NM_006591
CRYM	-5.2	15.5	crystallin, mu (CRYM), transcript variant 1, mRNA [NM_001888]	NM_001888	Hs.924	BX648477
ZNF551	-5.2	1.8	zinc finger protein 551 (ZNF551), mRNA [NM_138347]	NM_138347	Hs.656485	AK126625
BLMH	-5.2	1.7	bleomycin hydrolase (BLMH), mRNA [NM_000386]	NM_000386	Hs.371914	NM_000386
GATA6	-5.2	2.3	GATA binding protein 6 (GATA6), mRNA [NM_005257]	NM_005257	Hs.514746	X95701
GLTSCR2	-5.2	2.1	glioma tumor suppressor candidate region gene 2 (GLTSCR2), mRNA [NM_015710]	NM_015710	Hs.421907	AK024486
IMPACT	-5.2	2.0	Impact homolog (mouse) (IMPACT), mRNA [NM_018439]	NM_018439	Hs.515317	AB026264
PRIM1	-5.2	2.4	primase, polypeptide 1, 49kDa (PRIM1), mRNA [NM_000946]	NM_000946	Hs.534339	NM_000946
ENST0000030	-5.2	2.0	ribosomal protein S6 pseudogene 1 (RPS6P1), mRNA [Source:RefSeq_dna;Acc:XR_016837] [ENST00000308118]	ENST0000030811	Unknown	
BC107568	-5.2	2.8	cDNA clone IMAGE:3683736. [BC107568]	BC107568	Hs.527211	BX647680
PRIM1	-5.2	2.3	primase, polypeptide 1, 49kDa (PRIM1), mRNA [NM_000946]	NM_000946	Hs.534339	NM_000946
USP47	-5.2	1.8	ubiquitin specific peptidase 47 (USP47), mRNA [NM_017944]	NM_017944	Hs.577256	NM_017944
ALMS1	-5.2	2.2	Alstrom syndrome 1 (ALMS1), mRNA [NM_015120]	NM_015120	Hs.184720	AJ417593
THC2632286	-5.2	2.7	AA665072 nu76b01.s1 NCI_CGAP_Alv1 cDNA clone IMAGE:1216585, mRNA sequence [AA665072]	THC2632286	Unknown	
THC2497780	-5.2	2.6	THC2497780	THC2497780	Unknown	
SDCCAG1	-5.2	1.2	serologically defined colon cancer antigen 1 (SDCCAG1), mRNA [NM_004713]	NM_004713	Hs.655964	NM_004713
MEIS2	-5.2	3.5	Meis1, myeloid ecotropic viral integration site 1 homolog 2 (mouse) (MEIS2), transcript variant d, mRNA [NM_170676]	NM_170676	Hs.510989	NM_170677
CD200	-5.2	2.2	CD200 molecule (CD200), transcript variant 2, mRNA [NM_001004196]	NM_001004196	Hs.79015	NM_001004196

DTL	-5.2	1.9	denticleless homolog (Drosophila) (DTL), mRNA [NM_016448]	NM_016448	Hs.656473	NM_016448
HGF	-5.2	5.6	hepatocyte growth factor (hepapoietin A; scatter factor) (HGF), transcript variant 2, mRNA [NM_001010931]	NM_001010931	Hs.396530	NM_000601
DTL	-5.2	1.8	denticleless homolog (Drosophila) (DTL), mRNA [NM_016448]	NM_016448	Hs.656473	NM_016448
LIPT1	-5.2	2.0	lipoyltransferase 1 (LIPT1), transcript variant 3, mRNA [NM_145197]	NM_145197	Hs.516235	NM_145197
PRIM1	-5.2	2.4	primase, polypeptide 1, 49kDa (PRIM1), mRNA [NM_000946]	NM_000946	Hs.534339	NM_000946
CDKN1B	-5.2	1.5	cyclin-dependent kinase inhibitor 1B (p27, Kip1) (CDKN1B), mRNA [NM_004064]	NM_004064	Hs.238990	NM_004064
A_24_P14438	-5.2	2.2	A_24_P144383	A_24_P144383	Unknown	
PLG	-5.2	2.7	plasminogen (PLG), mRNA [NM_000301]	NM_000301	Hs.143436	CR749293
REV1	-5.2	1.7	REV1 homolog (S. cerevisiae) (REV1), transcript variant 1, mRNA [NM_016316]	NM_016316	Hs.443077	NM_016316
AF289562	-5.2	1.5	clone pp6337 unknown mRNA. [AF289562]	AF289562	Hs.276808	NM_002409
PLEKHH2	-5.2	2.3	pleckstrin homology domain containing, family H (with MyTH4 domain) member 2 (PLEKHH2), mRNA [NM_172069]	NM_172069	Hs.164162	AL832207
DTL	-5.2	1.8	denticleless homolog (Drosophila) (DTL), mRNA [NM_016448]	NM_016448	Hs.656473	NM_016448
RFC3	-5.2	2.2	replication factor C (activator 1) 3, 38kDa (RFC3), transcript variant 2, mRNA [NM_181558]	NM_181558	Hs.115474	AK128459
ZNF92	-5.2	2.4	zinc finger protein 92 (ZNF92), transcript variant 1, mRNA [NM_007139]	NM_007139	Hs.9521	NM_152626
POLR1B	-5.2	2.0	polymerase (RNA) I polypeptide B, 128kDa (POLR1B), mRNA [NM_019014]	NM_019014	Hs.86337	BX647683
ITPR2	-5.2	2.6	inositol 1,4,5-triphosphate receptor, type 2 (ITPR2), mRNA [NM_002223]	NM_002223	Hs.512235	NM_002223
DB304731	-5.2	3.1	DB304731 BRAWH3 cDNA clone BRAWH3006941 3', mRNA sequence [DB304731]	DB304731	Hs.593335	DB304731
SMO	-5.2	1.6	smoothened homolog (Drosophila) (SMO), mRNA [NM_005631]	NM_005631	Hs.437846	NM_005631
LOC392358	-5.2	1.7	PREDICTED: similar to 40S ribosomal protein S6 (LOC392358), mRNA [XR_018386]	XR_018386	Hs.647173	XR_018386
AK024171	-5.2	2.0	cDNA FLJ14109 fis, clone MAMMA1001322, moderately similar to B-CELL GROWTH FACTOR PRECURSOR. [AK024171]	AK024171	Hs.633042	AK024171
RBPSUH	-5.2	2.0	mRNA for H-2K binding factor-2, complete cds. [D14041]	D14041	Unknown	
THC2669157	-5.2	4.0	ALU1_HUMAN (P39188) Alu subfamily J sequence contamination warning entry, partial (10%) [THC2669157]	THC2669157	Unknown	
PRIM1	-5.2	2.4	primase, polypeptide 1, 49kDa (PRIM1), mRNA [NM_000946]	NM_000946	Hs.534339	NM_000946
A_24_P12744	-5.2	1.8	A_24_P127442	A_24_P127442	Unknown	
ADAMTS19	-5.2	3.3	ADAM metallopeptidase with thrombospondin type 1 motif, 19 (ADAMTS19), mRNA [NM_133638]	NM_133638	Hs.23751	NM_133638
HISPPD1	-5.2	1.9	histidine acid phosphatase domain containing 1 (HISPPD1), mRNA [NM_015216]	NM_015216	Hs.212046	AB007893
PTN	-5.2	2.8	pleiotrophin (heparin binding growth factor 8, neurite growth-promoting factor 1) (PTN), mRNA [NM_002825]	NM_002825	Hs.371249	CR624136
AK054895	-5.2	2.8	cDNA FLJ30333 fis, clone BRACE2007262. [AK054895]	AK054895	Hs.664520	AK054895
RPS29	-5.2	1.8	ribosomal protein S29 (RPS29), transcript variant 1, mRNA [NM_001032]	NM_001032	Hs.156367	BX161511
TNNT1	-5.2	2.0	troponin T type 1 (skeletal, slow), mRNA (cDNA clone MGC:104241 IMAGE:4247379), complete cds. [BC107798]	BC107798	Hs.631558	BI087655
DTL	-5.2	1.8	denticleless homolog (Drosophila) (DTL), mRNA [NM_016448]	NM_016448	Hs.656473	NM_016448
AGTR2	-5.2	19.0	angiotensin II receptor, type 2 (AGTR2), mRNA [NM_000686]	NM_000686	Hs.405348	NM_000686
MKL2	-5.2	2.0	MKL/myocardin-like 2 (MKL2), mRNA [NM_014048]	NM_014048	Hs.592047	NM_014048
WIT1	-5.2	9.7	Wilms tumor upstream neighbor 1 (WIT1), mRNA [NM_015855]	NM_015855	Hs.567499	M60614
C20orf132	-5.2	1.7	chromosome 20 open reading frame 132 (C20orf132), transcript variant 1, mRNA [NM_152503]	NM_152503	Hs.349125	BC030006
GBP4	-5.2	2.9	guanylate binding protein 4 (GBP4), mRNA [NM_052941]	NM_052941	Hs.409925	AL832576
C6orf148	-5.2	2.4	chromosome 6 open reading frame 148 (C6orf148), mRNA [NM_030568]	NM_030568	Hs.433062	AK090984
KDELC1	-5.2	1.6	KDEL (Lys-Asp-Glu-Leu) containing 1 (KDELC1), mRNA [NM_024089]	NM_024089	Hs.408629	BC051860
JMJD2D	-5.2	2.8	jumonji domain containing 2D (JMJD2D), mRNA [NM_018039]	NM_018039	Hs.503598	NM_018039
HIG2	-5.2	1.5	hypoxia-inducible protein 2 (HIG2), mRNA [NM_013332]	NM_013332	Hs.433213	NM_018396
SLC13A5	-5.2	5.9	solute carrier family 13 (sodium-dependent citrate transporter), member 5 (SLC13A5), mRNA [NM_177550]	NM_177550	Hs.399496	BX647354
CX164944	-5.2	2.7	HESC2_22_H03.g1_A035 NIH_MGC_258 cDNA clone IMAGE:7468640 5', mRNA sequence [CX164944]	CX164944	Hs.598840	CX164944
RPS3A	-5.2	1.7	ribosomal protein S3A (RPS3A), mRNA [NM_001006]	NM_001006	Hs.356572	BI087817
THC2656116	-5.2	4.5	THC2656116	THC2656116	Unknown	
SCLT1	-5.2	1.9	sodium channel and clathrin linker 1 (SCLT1), mRNA [NM_144643]	NM_144643	Hs.654690	AK122852
ZNF236	-5.1	2.1	zinc finger protein 236 (ZNF236), mRNA [NM_007345]	NM_007345	Hs.189826	NM_007345
THC2484646	-5.1	2.2	THC2484646	THC2484646	Unknown	
AK023559	-5.1	2.6	cDNA FLJ13497 fis, clone PLACE1004518. [AK023559]	AK023559	Hs.133444	AK023559
LOC441876	-5.1	1.6	PREDICTED: similar to 40S ribosomal protein S16, transcript variant 1 (LOC441876), mRNA [XM_497657]	XM_497657	Unknown	
THC2675641	-5.1	2.5	Q3ECA2_ARATH (Q3ECA2) Protein At1g79810, partial (6%) [THC2675641]	THC2675641	Unknown	
AF119866	-5.1	2.4	PRO2206 mRNA, complete cds. [AF119866]	AF119866	Unknown	

DPPA2	-5.1	3.9	developmental pluripotency associated 2 (DPPA2), mRNA [NM_138815]	NM_138815	Hs.351113	AY283672
ANGPT2	-5.1	5.1	angiopoietin 2 (ANGPT2), mRNA [NM_001147]	NM_001147	Hs.583870	AK225698
C1orf123	-5.1	1.6	chromosome 1 open reading frame 123 (C1orf123), mRNA [NM_017887]	NM_017887	Hs.525391	AK092734
XRCC1	-5.1	1.7	X-ray repair complementing defective repair in Chinese hamster cells 1 (XRCC1), mRNA [NM_006297]	NM_006297	Hs.98493	CR591751
KIAA1009	-5.1	2.4	KIAA1009 (KIAA1009), mRNA [NM_014895]	NM_014895	Hs.485865	NM_014895
THC2682280	-5.1	2.4	THC2682280	THC2682280	Unknown	
THC2651723	-5.1	2.2	HUMUBCP pro-ubiquitin {Homo sapiens} (exp=-1; wgp=0; cg=0), partial (39%) [THC2651723]	THC2651723	Unknown	
DTL	-5.1	1.8	denticleless homolog (Drosophila) (DTL), mRNA [NM_016448]	NM_016448	Hs.656473	NM_016448
PTMA	-5.1	2.3	prothymosin, alpha (gene sequence 28) (PTMA), mRNA [NM_002823]	NM_002823	Hs.459927	BM470466
LOC642958	-5.1	3.0	PREDICTED: similar to Heterogeneous nuclear ribonucleoprotein A1 (Helix-destabilizing protein) (Single-strand RNA-binding protein) (hnRNP core protein A1) (HDP) (LOC642958), mRNA [XR_018441]	XR_018441	Hs.646321	XR_018441
CENPJ	-5.1	2.1	centromere protein J (CENPJ), mRNA [NM_018451]	NM_018451	Hs.513379	NM_018451
BC036599	-5.1	2.1	cDNA clone IMAGE:5273964. [BC036599]	BC036599	Hs.24321	AK022090
ENST0000037	-5.1	3.5	Protein CXorf42. [Source:Uniprot/SWISSPROT;Acc:Q8N9T2] [ENST00000371386]	ENST0000037138	Unknown	
ACCN2	-5.1	2.2	amiloride-sensitive cation channel 2, neuronal (ACCN2), transcript variant 1, mRNA [NM_020039]	NM_020039	Hs.274361	NM_020039
RNMT	-5.1	1.9	RNA (guanine-7-) methyltransferase (RNMT), mRNA [NM_003799]	NM_003799	Hs.592347	AF067791
DDC	-5.1	2.1	dopa decarboxylase (aromatic L-amino acid decarboxylase) (DDC), mRNA [NM_000790]	NM_000790	Hs.359698	NM_001082971
SLC25A26	-5.1	1.9	solute carrier family 25, member 26 (SLC25A26), mRNA [NM_173471]	NM_173471	Hs.379386	NM_173471
UST	-5.1	2.3	uronyl-2-sulfotransferase (UST), mRNA [NM_005715]	NM_005715	Hs.657370	AB020316
FAM44B	-5.1	1.9	family with sequence similarity 44, member B (FAM44B), mRNA [NM_138369]	NM_138369	Hs.425091	BM908302
TBX2	-5.1	2.6	T-box 2 (TBX2), mRNA [NM_005994]	NM_005994	Hs.699297	AB209378
AK021751	-5.1	1.9	cDNA FLJ11689 fis, clone HEMBA1004977. [AK021751]	AK021751	Hs.645624	AK021751
CLNS1A	-5.1	1.7	chloride channel, nucleotide-sensitive, 1A (CLNS1A), mRNA [NM_001293]	NM_001293	Hs.430733	NM_001293
ZNF451	-5.1	1.9	zinc finger protein 451 (ZNF451), transcript variant 1, mRNA [NM_001031623]	NM_001031623	Hs.485628	NM_001031623
C14orf106	-5.1	2.7	chromosome 14 open reading frame 106 (C14orf106), mRNA [NM_018353]	NM_018353	Hs.437941	BC065544
CR624390	-5.1	1.6	full-length cDNA clone CS0DC018YB19 of Neuroblastoma Cot 25-normalized of (human). [CR624390]	CR624390	Hs.400432	BC024195
A_24_P13577	-5.1	2.4	A_24_P135771	A_24_P135771	Unknown	
ENST0000033	-5.1	2.8	cDNA FLJ46467 fis, clone THYMU3022668. [AK128325]	ENST0000033545	Unknown	
CDKN1B	-5.1	1.5	cyclin-dependent kinase inhibitor 1B (p27, Kip1) (CDKN1B), mRNA [NM_004064]	NM_004064	Hs.238990	NM_004064
UIMC1	-5.1	1.4	ubiquitin interaction motif containing 1 (UIMC1), mRNA [NM_016290]	NM_016290	Hs.232721	BX537376
RPS7	-5.1	2.1	ribosomal protein S7 (RPS7), mRNA [NM_001011]	NM_001011	Hs.546287	AB209386
THC2656699	-5.1	2.1	THC2656699	THC2656699	Unknown	
HNRPH1	-5.1	2.1	heterogeneous nuclear ribonucleoprotein H1 (H) (HNRPH1), mRNA [NM_005520]	NM_005520	Hs.604001	BX647205
A_24_P33244	-5.1	1.7	A_24_P332441	A_24_P332441	Unknown	
CDKL3	-5.1	1.8	cyclin-dependent kinase-like 3 (CDKL3), mRNA [NM_016508]	NM_016508	Hs.105818	BC041799
TCF15	-5.1	2.1	transcription factor 15 (basic helix-loop-helix) (TCF15), mRNA [NM_004609]	NM_004609	Hs.437	NM_004609
LOC645412	-5.1	2.0	PREDICTED: similar to 40S ribosomal protein S16 (LOC645412), mRNA [XR_016930]	XR_016930	Hs.646362	XR_016930
A_24_P31565	-5.1	1.7	A_24_P315654	A_24_P315654	Unknown	
MOBKL1A	-5.1	1.8	MOB1, Mps One Binder kinase activator-like 1A (yeast) (MOBKL1A), mRNA [NM_173468]	NM_173468	Hs.31422	NM_173468
BCORL1	-5.1	1.5	BCL6 co-repressor-like 1 (BCORL1), mRNA [NM_021946]	NM_021946	Hs.496748	NM_021946
SHPRH	-5.1	2.2	SNF2 histone linker PHD RING helicase (SHPRH), transcript variant 1, mRNA [NM_001042683]	NM_001042683	Hs.124537	NM_001042683
MCM3APAS	-5.1	2.4	MCM3 minichromosome maintenance deficient 3 (S. cerevisiae) associated protein antisense (MCM3APAS) on chromosome 21 [NR_002776]	NR_002776	Unknown	
G31710	-5.1	1.9	sWSS2257 Eric D. Green STS cDNA, sequence tagged site. [G31710]	G31710	Unknown	
FNBP4	-5.1	1.5	formin binding protein 4 (FNBP4), mRNA [NM_015308]	NM_015308	Hs.6834	AB023231
CHD7	-5.1	3.4	chromodomain helicase DNA binding protein 7 (CHD7), mRNA [NM_017780]	NM_017780	Hs.20395	NM_017780
ANKHD1	-5.1	1.9	ankyrin repeat and KH domain containing 1 (ANKHD1), transcript variant 1, mRNA [NM_017747]	NM_017747	Hs.653135	NM_020690
A_24_P29883	-5.1	1.9	A_24_P298835	A_24_P298835	Unknown	
FNDC3A	-5.1	2.0	fibronectin type III domain containing 3A (FNDC3A), transcript variant 1, mRNA [NM_001079673]	NM_001079673	Hs.508010	NM_001079673
PEX1	-5.1	1.7	peroxisome biogenesis factor 1 (PEX1), mRNA [NM_000466]	NM_000466	Hs.164682	NM_000466
SOAT2	-5.1	3.7	sterol O-acyltransferase 2 (SOAT2), mRNA [NM_003578]	NM_003578	Hs.656544	NM_003578
A_24_P7181	-5.1	1.9	A_24_P7181	A_24_P7181	Unknown	

RPL15	-5.1	1.9	ribosomal protein L15 (RPL15), mRNA [NM_002948]	NM_002948	Hs.381219	BC081565
RP1-93H18.5	-5.1	2.6	hypothetical protein LOC441168 (LOC441168), mRNA [NM_001010919]	NM_001010919	Hs.381220	NM_001010919
PRND	-5.1	2.7	prion protein 2 (dublet) (PRND), mRNA [NM_012409]	NM_012409	Hs.406696	NM_012409
A_32_P23053	-5.1	2.5	A_32_P230537	A_32_P230537	Unknown	
CFC1	-5.1	7.8	cripto, FRL-1, cryptic family 1 (CFC1), mRNA [NM_032545]	NM_032545	Hs.567542	NM_032545
KIAA1212	-5.1	2.0	KIAA1212 (KIAA1212), mRNA [NM_018084]	NM_018084	Hs.292925	NM_018084
RPL21	-5.1	1.5	ribosomal protein L21 (RPL21), mRNA [NM_000982]	NM_000982	Hs.381123	CR602527
HGF	-5.1	4.1	hepatocyte growth factor (hepapoietin A; scatter factor) (HGF), transcript variant 2, mRNA [NM_001010931]	NM_001010931	Hs.396530	NM_000601
KIAA1166	-5.0	1.4	KIAA1166 (KIAA1166), mRNA [NM_018684]	NM_018684	Hs.28249	AB032992
BC038512	-5.0	1.8	cDNA clone IMAGE:5262734. [BC038512]	BC038512	Hs.296031	BC038512
CEP68	-5.0	3.0	mRNA for KIAA0582 protein, partial cds. [AB011154]	AB011154	Hs.699217	NM_015147
BNIP3L	-5.0	2.1	BCL2/adenovirus E1B 19kDa interacting protein 3-like (BNIP3L), mRNA [NM_004331]	NM_004331	Hs.131226	AF370457
THC2657355	-5.0	17.9	THC2657355	THC2657355	Unknown	
RBM16	-5.0	1.6	RNA binding motif protein 16 (RBM16), mRNA [NM_014892]	NM_014892	Hs.591329	NM_014892
EPO	-5.0	5.9	erythropoietin (EPO), mRNA [NM_000799]	NM_000799	Hs.2303	X02157
AGER	-5.0	2.1	advanced glycosylation end product-specific receptor (AGER), transcript variant 1, mRNA [NM_001136]	NM_001136	Hs.700564	AB061669
MTERFD2	-5.0	1.4	MTERF domain containing 2 (MTERFD2), mRNA [NM_182501]	NM_182501	Hs.159556	NM_182501
MYCNOS	-5.0	2.3	N-cym=DNA-binding transcriptional activator homolog {oncogene} [human, Kelly neuroblastoma cell line, mRNA, 778 nt]. [S49953]	S49953	Hs.651453	BC002892
MGC40405	-5.0	1.8	hypothetical protein MGC40405 (MGC40405), transcript variant 2, mRNA [NM_001040057]	NM_001040057	Hs.489105	NM_001040057
RC74	-5.0	1.6	integrator complex subunit 9 (RC74), mRNA [NM_018250]	NM_018250	Hs.162397	BC016687
BX095281	-5.0	2.2	BX095281 Soares melanocyte 2NbHM cDNA clone IMAGp998E03596, mRNA sequence [BX095281]	BX095281	Hs.93780	BX095281
EML4	-5.0	2.6	echinoderm microtubule associated protein like 4 (EML4), mRNA [NM_019063]	NM_019063	Hs.593614	NM_019063
LOC126147	-5.0	2.4	hypothetical protein BC018697 (LOC126147), mRNA [NM_145807]	NM_145807	Hs.326217	BC021210
RBP1	-5.0	1.6	retinol binding protein 1, cellular (RBP1), mRNA [NM_002899]	NM_002899	Hs.529571	BM926478
KIAA1505	-5.0	1.4	KIAA1505 protein (KIAA1505), mRNA [NM_020879]	NM_020879	Hs.113940	BX649000
LOC402069	-5.0	2.1	PREDICTED: similar to 40S ribosomal protein S16 (LOC402069), mRNA [XR_019634]	XR_019634	Hs.651054	XR_019634
LOC153914	-5.0	2.2	cDNA FLJ13531 fis, clone PLACE1006288, highly similar to VOLTAGE-DEPENDENT ANION-SELECTIVE CHANNEL PROTEIN 1. [AK023593]	AK023593	Hs.7277	BX648803
CR590862	-5.0	1.6	full-length cDNA clone CS0DI036YC04 of Placenta Cot 25-normalized of (human). [CR590862]	CR590862	Hs.259347	NM_173601
WDR86	-5.0	4.5	cDNA FLJ38667 fis, clone HLUNG2006843. [AK095986]	AK095986	Hs.659231	AK095986
MTBP	-5.0	3.3	Mdm2, transformed 3T3 cell double minute 2, p53 binding protein (mouse) binding protein, 104kDa (MTBP), mRNA [NM_022045]	NM_022045	Hs.657656	AL832671
ENST0000034	-5.0	3.8	hypothetical protein mRNA, complete cds. [AY070435]	ENST0000034511	Unknown	
HISPPD1	-5.0	1.9	histidine acid phosphatase domain containing 1 (HISPPD1), mRNA [NM_015216]	NM_015216	Hs.212046	AB007893
THEM4	-5.0	2.2	thioesterase superfamily member 4 (THEM4), mRNA [NM_053055]	NM_053055	Hs.164070	AK096211
NSBP1	-5.0	2.2	nucleosomal binding protein 1 (NSBP1), mRNA [NM_030763]	NM_030763	Hs.282204	AF250329
THC2567891	-5.0	1.7	Q6NXR8_HUMAN (Q6NXR8) Ribosomal protein S3a, partial (91%) [THC2567891]	THC2567891	Unknown	
BC065737	-5.0	1.9	cDNA clone IMAGE:30404477, partial cds. [BC065737]	BC065737	Hs.626174	XR_015810
C10orf82	-5.0	4.2	chromosome 10 open reading frame 82 (C10orf82), mRNA [NM_144661]	NM_144661	Hs.121347	BX537582
COQ7	-5.0	1.6	coenzyme Q7 homolog, ubiquinone (yeast) (COQ7), mRNA [NM_016138]	NM_016138	Hs.157113	BC043272
WAPAL	-5.0	2.0	wings apart-like homolog (Drosophila) (WAPAL), mRNA [NM_015045]	NM_015045	Hs.203099	AB065003
JMJD1A	-5.0	1.9	jumonji domain containing 1A (JMJD1A), mRNA [NM_018433]	NM_018433	Hs.557425	BX640698
SSPO	-5.0	3.6	cDNA FLJ36112 fis, clone TESTI2022023, weakly similar to Bos taurus Reissner's fiber glycoprotein I mRNA. [AK093431]	AK093431	Hs.632022	NM_198455
KCNH8	-5.0	3.4	potassium voltage-gated channel, subfamily H (eag-related), member 8 (KCNH8), mRNA [NM_144633]	NM_144633	Hs.475656	AY053503
ZFP36L1	-5.0	1.4	zinc finger protein 36, C3H type-like 1 (ZFP36L1), mRNA [NM_004926]	NM_004926	Hs.85155	AK024202
COL23A1	-5.0	2.6	collagen, type XXIII, alpha 1 (COL23A1), mRNA [NM_173465]	NM_173465	Hs.660026	NM_173465
AK124576	-5.0	2.1	cDNA FLJ42585 fis, clone BRACE3009237. [AK124576]	AK124576	Hs.4749	AK124576
CENPK	-5.0	2.0	centromere protein K (CENPK), mRNA [NM_022145]	NM_022145	Hs.529778	NM_022145
AI630435	-5.0	1.6	AI630435 ad10b05.y1 Hembase; Erythroid Progenitor Cells (LCB:ad library) cDNA clone ad10b05 random, mRNA sequence [AI630435]	AI630435	Hs.698003	BQ057523
A_24_P23842	-5.0	2.1	A_24_P238427	A_24_P238427	Unknown	
KIAA1729	-5.0	2.0	KIAA1729 protein (KIAA1729), mRNA [NM_053042]	NM_053042	Hs.455089	NM_053042
USP47	-5.0	1.4	ubiquitin specific peptidase 47 (USP47), mRNA [NM_017944]	NM_017944	Hs.577256	NM_017944

MSH2	-5.0	1.6	mutS homolog 2, colon cancer, nonpolyposis type 1 (E. coli) (MSH2), mRNA [NM_000251]	NM_000251	Hs.597656	AK223284
KDELC1	-5.0	1.7	KDEL (Lys-Asp-Glu-Leu) containing 1 (KDELC1), mRNA [NM_024089]	NM_024089	Hs.408629	BC051860
FABP1	-5.0	2.5	fatty acid binding protein 1, liver (FABP1), mRNA [NM_001443]	NM_001443	Hs.380135	BX641099
TTC14	-5.0	1.3	tetratricopeptide repeat domain 14 (TTC14), transcript variant 2, mRNA [NM_001042601]	NM_001042601	Hs.43213	NM_001042601
U69195	-5.0	1.5	U69195 Soares infant brain 1NIB cDNA clone 32996, mRNA sequence [U69195]	U69195	Hs.697380	U69195
ZNF689	-5.0	1.8	zinc finger protein 689 (ZNF689), mRNA [NM_138447]	NM_138447	Hs.454685	AB065282
ZNF223	-5.0	1.7	zinc finger protein 223 (ZNF223), mRNA [NM_013361]	NM_013361	Hs.532870	BC022466
ZNF557	-5.0	1.4	zinc finger protein 557 (ZNF557), transcript variant 1, mRNA [NM_024341]	NM_024341	Hs.591380	NM_024341
CNTN4	-5.0	1.9	contactin 4 (CNTN4), transcript variant 1, mRNA [NM_175607]	NM_175607	Hs.298705	NM_175607
LOC649839	-5.0	2.3	PREDICTED: similar to large subunit ribosomal protein L36a (LOC649839), mRNA [XM_001129410]	ENST0000033037	Unknown	
FBL	-5.0	2.3	fibrillarin (FBL), mRNA [NM_001436]	NM_001436	Hs.299002	BG324446
C19orf46	-5.0	2.2	chromosome 19 open reading frame 46 (C19orf46), mRNA [NM_001039876]	NM_001039876	Hs.436743	NM_001039876
YPEL1	-5.0	1.8	yippee-like 1 (Drosophila) (YPEL1), mRNA [NM_013313]	NM_013313	Hs.517436	CR933630
DTL	-5.0	1.9	denticleless homolog (Drosophila) (DTL), mRNA [NM_016448]	NM_016448	Hs.656473	NM_016448
THC2676596	-5.0	2.3	ALU8_HUMAN (P39195) Alu subfamily SX sequence contamination warning entry, partial (24%) [THC2722939]	THC2676596	Unknown	
HSD17B8	-5.0	1.9	hydroxysteroid (17-beta) dehydrogenase 8 (HSD17B8), mRNA [NM_014234]	NM_014234	Hs.415058	BQ651523
PMPCB	-5.0	1.4	peptidase (mitochondrial processing) beta (PMPCB), nuclear gene encoding mitochondrial protein, mRNA [NM_004279]	NM_004279	Hs.184211	NM_004279
KIF13A	-5.0	1.9	kinesin family member 13A (KIF13A), mRNA [NM_022113]	NM_022113	Hs.189915	AY014403
A_24_P56116	-5.0	1.9	A_24_P561165	A_24_P561165	Unknown	
PAIP1	-5.0	2.6	poly(A) binding protein interacting protein 1 (PAIP1), transcript variant 1, mRNA [NM_006451]	NM_006451	Hs.482038	NM_006451
HISPPD1	-5.0	1.9	histidine acid phosphatase domain containing 1 (HISPPD1), mRNA [NM_015216]	NM_015216	Hs.212046	AB007893
THC2587432	-5.0	2.8	THC2587432	THC2587432	Unknown	
TANC1	-5.0	1.4	tetratricopeptide repeat, ankyrin repeat and coiled-coil containing 1 (TANC1), mRNA [NM_033394]	NM_033394	Hs.158728	NM_033394
THEG	-5.0	5.5	Theg homolog (mouse) (THEG), transcript variant 1, mRNA [NM_016585]	NM_016585	Hs.250002	NM_016585
LOC391490	-5.0	2.0	PREDICTED: similar to 60S ribosomal protein L23a (LOC391490), mRNA [XR_018768]	XR_018768	Hs.647862	XR_018768
RPL24	-5.0	1.6	ribosomal protein L24 (RPL24), mRNA [NM_000986]	NM_000986	Hs.477028	CR608385
ARG1	-5.0	7.2	arginase, liver (ARG1), mRNA [NM_000045]	NM_000045	Hs.440934	AK128314
TIGD1	-5.0	2.0	tigger transposable element derived 1 (TIGD1), mRNA [NM_145702]	NM_145702	Hs.211823	BC035143
SVEP1	-5.0	3.5	sushi, von Willebrand factor type A, EGF and pentraxin domain containing 1 (SVEP1), mRNA [NM_153366]	NM_153366	Hs.522334	NM_153366
KIAA1345	-5.0	1.5	mRNA for KIAA1345 protein, partial cds. [AB037766]	AB037766	Hs.590928	BX647334
ZNF184	-5.0	2.3	zinc finger protein 184 (ZNF184), mRNA [NM_007149]	NM_007149	Hs.158174	AK123011
LOC283481	-5.0	3.3	hypothetical protein LOC283481, mRNA (cDNA clone IMAGE:5296747). [BC033993]	BC033993	Hs.646604	BI560826
MGC16169	-5.0	1.7	hypothetical protein MGC16169 (MGC16169), mRNA [NM_033115]	NM_033115	Hs.292986	BX647851
LOC130728	-5.0	2.1	PREDICTED: similar to 60S ribosomal protein L7 (LOC130728), mRNA [XR_019248]	XR_019248	Hs.549947	XR_019248
BTBD7	-5.0	1.5	BTB (POZ) domain containing 7 (BTBD7), transcript variant 1, mRNA [NM_001002860]	NM_001002860	Hs.525549	NM_001002860
AK091569	-5.0	1.7	cDNA FLJ34250 fis, clone FCBBF4000529. [AK091569]	AK091569	Hs.594418	AK091569
NSBP1	-5.0	2.2	nucleosomal binding protein 1 (NSBP1), mRNA [NM_030763]	NM_030763	Hs.282204	AF250329
MSH2	-5.0	1.6	mutS homolog 2, colon cancer, nonpolyposis type 1 (E. coli) (MSH2), mRNA [NM_000251]	NM_000251	Hs.597656	AK223284
ZNF318	-5.0	1.6	zinc finger protein 318 (ZNF318), mRNA [NM_014345]	NM_014345	Hs.509718	AF090114
KIAA1377	-5.0	3.3	KIAA1377 (KIAA1377), mRNA [NM_020802]	NM_020802	Hs.156352	NM_020802
THC2668270	-5.0	2.1	Q8I727_TRYCR (Q8I727) TcC31.32, partial (14%) [THC2668270]	THC2668270	Unknown	
CALCRL	-5.0	10.0	calcitonin receptor-like (CALCRL), mRNA [NM_005795]	NM_005795	Hs.470882	NM_005795
BACH1	-5.0	1.7	BTB and CNC homology 1, basic leucine zipper transcription factor 1 (BACH1), transcript variant 1, mRNA [NM_206866]	NM_206866	Hs.154276	NM_206866
PFKFB4	-5.0	2.2	6-phosphofructo-2-kinase/fructose-2,6-biphosphatase 4 (PFKFB4), mRNA [NM_004567]	NM_004567	Hs.476217	NM_004567
MGA	-5.0	1.5	CDNA FLJ13298 fis, clone OVARC1001306, weakly similar to N-MYC PROTO-ONCOGENE PROTEIN. (Fragment). [Source:Uniprot/SPTREMBL;Acc:Q9H8R3] [ENST00000310492]	ENST0000031049	Unknown	
TRIB2	-5.0	2.6	tribbles homolog 2 (Drosophila) (TRIB2), mRNA [NM_021643]	NM_021643	Hs.696139	NM_021643
RPS27A	-4.9	1.9	ribosomal protein S27a (RPS27A), mRNA [NM_002954]	NM_002954	Hs.546292	BU537386
C3orf41	-4.9	6.1	mRNA; cDNA DKFZp434B172 (from clone DKFZp434B172). [AL117530]	AL117530	Hs.697193	AL117530
FAM101A	-4.9	1.7	family with sequence similarity 101, member A (FAM101A), mRNA [NM_181709]	NM_181709	Hs.432901	NM_181709

CTDSP2	-4.9	1.8	CTD (carboxy-terminal domain, RNA polymerase II, polypeptide A) small phosphatase 2 (CTDSP2), mRNA [NM_005730]	NM_005730	Hs.524530	NM_005730
A_24_P51177	-4.9	2.1	A_24_P511776	A_24_P511776	Unknown	
DKFZp434K19	-4.9	3.2	hypothetical protein DKFZp434K191 (DKFZp434K191), mRNA [NM_001029950]	NM_001029950	Unknown	
DFNB59	-4.9	2.4	deafness, autosomal recessive 59 (DFNB59), mRNA [NM_001042702]	NM_001042702	Hs.87734	BC020859
ZNF567	-4.9	1.9	zinc finger protein 567 (ZNF567), mRNA [NM_152603]	NM_152603	Hs.697242	AK126691
LOC731731	-4.9	1.8	PREDICTED: similar to Nucleosome assembly protein 1-like 1 (NAP-1-related protein) (hNRP) (LOC731731), mRNA [XR_015583]	XR_015583	Hs.647218	XR_015583
CDKN1B	-4.9	1.5	cyclin-dependent kinase inhibitor 1B (p27, Kip1) (CDKN1B), mRNA [NM_004064]	NM_004064	Hs.238990	NM_004064
RBM12	-4.9	2.7	RNA binding motif protein 12 (RBM12), transcript variant 1, mRNA [NM_006047]	NM_006047	Hs.246413	NM_006047
C11orf57	-4.9	2.1	chromosome 11 open reading frame 57 (C11orf57), mRNA [NM_018195]	NM_018195	Hs.195060	NM_018195
ANP32A	-4.9	2.7	Acidic leucine-rich nuclear phosphoprotein 32 family member A (Potent heat-stable protein phosphatase 2A inhibitor I1PP2A) (Acidic nuclear phosphoprotein pp32) (Leucine-rich acidic nuclear protein) (Lanp) (Putative HLA-DR-associated protein I) (PHAPI)...	ENST0000026791	Unknown	
HERPUD2	-4.9	1.8	HERPUD family member 2, mRNA (cDNA clone IMAGE:4821170). [BC049371]	BC049371	Hs.599851	BC035153
TMEM101	-4.9	1.9	transmembrane protein 101 (TMEM101), mRNA [NM_032376]	NM_032376	Hs.514211	BM542308
FAM122C	-4.9	4.0	family with sequence similarity 122C (FAM122C), mRNA [NM_138819]	NM_138819	Hs.269127	AK090611
PRR4	-4.9	1.5	proline rich 4 (lacrimal) (PRR4), mRNA [NM_007244]	NM_007244	Hs.408153	BX648418
SPECC1L	-4.9	1.5	SPECC1-like (SPECC1L), mRNA [NM_015330]	NM_015330	Hs.474384	NM_015330
LOC120364	-4.9	2.7	PREDICTED: similar to Heterogeneous nuclear ribonucleoprotein A1 (Helix-destabilizing protein) (Single-strand binding protein) (hnRNP core protein A1) (HDP-1) (Topoisomerase-inhibitor suppressed) (LOC120364), mRNA [XM_062025]	ENST0000035486	Unknown	
AIF1	-4.9	7.5	allograft inflammatory factor 1 (AIF1), transcript variant 2, mRNA [NM_004847]	NM_004847	Unknown	
TESK2	-4.9	2.1	testis-specific kinase 2 (TESK2), mRNA [NM_007170]	NM_007170	Hs.591499	NM_007170
KLB	-4.9	2.8	klotho beta (KLB), mRNA [NM_175737]	NM_175737	Hs.90756	BC033021
CDKN1B	-4.9	1.5	cyclin-dependent kinase inhibitor 1B (p27, Kip1) (CDKN1B), mRNA [NM_004064]	NM_004064	Hs.238990	NM_004064
NARG1L	-4.9	2.3	NMDA receptor regulated 1-like (NARG1L), transcript variant 1, mRNA [NM_024561]	NM_024561	Hs.512914	CR627327
BC066984	-4.9	1.8	cDNA clone IMAGE:4826623. [BC066984]	BC066984	Hs.30174	AK024763
THC2693842	-4.9	2.2	THC2693842	THC2693842	Unknown	
C11orf74	-4.9	1.8	chromosome 11 open reading frame 74 (C11orf74), mRNA [NM_138787]	NM_138787	Hs.406726	AK095997
STOX1	-4.9	2.1	storkhead box 1 (STOX1), mRNA [NM_152709]	NM_152709	Hs.37636	AY842014
GNG8	-4.9	3.0	guanine nucleotide binding protein (G protein), gamma 8 (GNG8), mRNA [NM_033258]	NM_033258	Hs.283961	CB565924
AV739664	-4.9	2.5	AV739664 CB cDNA clone CBNAWB09 5', mRNA sequence [AV739664]	AV739664	Hs.686885	AV739664
ID2	-4.9	2.7	inhibitor of DNA binding 2, dominant negative helix-loop-helix protein (ID2), mRNA [NM_002166]	NM_002166	Hs.180919	CR623038
ENST0000033	-4.9	1.5	PREDICTED: hypothetical protein LOC730438 (LOC730438), mRNA [XR_015115]	ENST0000033261	Unknown	
FLJ10324	-4.9	2.6	mRNA for KIAA1849 protein, partial cds. [AB058752]	AB058752	Hs.667336	AB058752
PEX7	-4.9	1.5	peroxisomal biogenesis factor 7 (PEX7), mRNA [NM_000288]	NM_000288	Hs.280932	BC031606
BATF2	-4.9	2.8	basic leucine zipper transcription factor, ATF-like 2 (BATF2), mRNA [NM_138456]	NM_138456	Hs.124840	NM_138456
MSH2	-4.9	1.6	mutS homolog 2, colon cancer, nonpolyposis type 1 (E. coli) (MSH2), mRNA [NM_000251]	NM_000251	Hs.597656	AK223284
SYT15	-4.9	1.9	synaptotagmin XV (SYT15), transcript variant b, mRNA [NM_181519]	NM_181519	Hs.696346	NM_031912
AL080134	-4.9	2.8	mRNA; cDNA DKFZp434G043 (from clone DKFZp434G043). [AL080134]	AL080134	Hs.168762	AB014523
SENP1	-4.9	2.0	SUMO1/sentrin specific peptidase 1 (SENP1), mRNA [NM_014554]	NM_014554	Hs.371957	BX640784
REL	-4.9	1.6	v-rel reticuloendotheliosis viral oncogene homolog (avian) (REL), mRNA [NM_002908]	NM_002908	Hs.631886	NM_002908
APOC1	-4.9	3.5	apolipoprotein C-I (APOC1), mRNA [NM_001645]	NM_001645	Hs.110675	AJ249921
ZCCHC14	-4.9	1.5	zinc finger, CCHC domain containing 14 (ZCCHC14), mRNA [NM_015144]	NM_015144	Hs.156231	NM_015144
THC2739352	-4.9	2.2	Q3GZE6_9ACTO (Q3GZE6) AAA ATPase, central region, partial (3%) [THC2739352]	THC2739352	Unknown	
EXOSC7	-4.9	2.2	exosome component 7 (EXOSC7), mRNA [NM_015004]	NM_015004	Hs.115792	AK090511
A_24_P73025	-4.9	1.6	A_24_P730256	A_24_P730256	Unknown	
KERA	-4.9	2.0	keratocan (KERA), mRNA [NM_007035]	NM_007035	Hs.125750	NM_007035
BC024649	-4.9	1.7	cDNA clone IMAGE:3864427, **** WARNING: chimeric clone ****. [BC024649]	BC024649	Unknown	
C11orf69	-4.9	2.4	chromosome 11 open reading frame 69 (C11orf69), mRNA [NM_152314]	NM_152314	Unknown	
ZNF284	-4.9	2.3	mRNA; cDNA DKFZp781F1775 (from clone DKFZp781F1775). [CR936662]	CR936662	Hs.445395	CR936662
ST6GALNAC3	-4.9	2.2	ST6 (alpha-N-acetyl-neuraminyl-2,3-beta-galactosyl-1,3)-N-acetylgalactosaminide alpha-2,6-sialyltransferase 3 (ST6GALNAC3), mRNA [NM_152996]	NM_152996	Hs.337040	BX648274
CPM	-4.9	3.1	carboxypeptidase M (CPM), transcript variant 1, mRNA [NM_001874]	NM_001874	Hs.654387	NM_001874
DMRTC1	-4.9	2.9	DMRT-like family C1 (DMRTC1), mRNA [NM_033053]	NM_033053	Hs.658177	NM_033053

LRCH2	-4.9	1.9	leucine-rich repeats and calponin homology (CH) domain containing 2 (LRCH2), mRNA [NM_020871]	NM_020871	Hs.65366	BX647193
FLJ21839	-4.9	3.0	hypothetical protein FLJ21839 (FLJ21839), transcript variant 1, mRNA [NM_021831]	NM_021831	Hs.138207	NM_021831
BC002470	-4.9	2.0	mRNA similar to protein kinase, cAMP dependent regulatory, type I beta (cDNA clone IMAGE:3349336). [BC002470]	BC002470	Unknown	
SUHW3	-4.9	2.5	suppressor of hairy wing homolog 3 (Drosophila) (SUHW3), mRNA [NM_017666]	NM_017666	Hs.308418	BC051728
CR593388	-4.9	1.9	full-length cDNA clone CS0DI067YA03 of Placenta Cot 25-normalized of (human). [CR593388]	CR593388	Hs.700704	CR593388
RPS20	-4.9	1.6	ribosomal protein S20 (RPS20), mRNA [NM_001023]	NM_001023	Hs.8102	BQ053882
A_32_P12878	-4.9	1.5	A_32_P128781	A_32_P128781	Unknown	
ENST0000037	-4.9	2.0	Muscleblind-like X-linked protein (Muscleblind-like protein 3) (Cys3His CCG1-required protein) (Protein HCHCR). [Source:Uniprot/SWISSPROT;Acc:Q9NUK0] [ENST00000370857]	ENST0000037085	Unknown	
NSBP1	-4.9	2.2	nucleosomal binding protein 1 (NSBP1), mRNA [NM_030763]	NM_030763	Hs.282204	AF250329
CENPJ	-4.9	2.2	centromere protein J (CENPJ), mRNA [NM_018451]	NM_018451	Hs.513379	NM_018451
CROP	-4.9	1.7	cisplatin resistance-associated overexpressed protein (CROP), transcript variant 2, mRNA [NM_006107]	NM_006107	Hs.130293	NM_016424
ENST0000037	-4.9	2.1	hypothetical protein LOC348751, mRNA (cDNA clone IMAGE:5311172). [BC039445]	ENST0000037471	Unknown	
C14orf8	-4.9	2.7	chromosome 14 open reading frame 8 (C14orf8), mRNA [NM_173846]	NM_173846	Hs.406966	AK093070
ZNF432	-4.9	3.8	Zinc finger protein 432. [Source:Uniprot/SWISSPROT;Acc:O94892] [ENST00000221315]	ENST0000022131	Unknown	
KDELC1	-4.9	1.6	KDEL (Lys-Asp-Glu-Leu) containing 1 (KDELC1), mRNA [NM_024089]	NM_024089	Hs.408629	BC051860
HBD	-4.9	4.1	hemoglobin, delta (HBD), mRNA [NM_000519]	NM_000519	Hs.699280	AY034468
A_24_P13539	-4.8	2.6	A_24_P135391	A_24_P135391	Unknown	
CDKN1B	-4.8	1.5	cyclin-dependent kinase inhibitor 1B (p27, Kip1) (CDKN1B), mRNA [NM_004064]	NM_004064	Hs.238990	NM_004064
AK123446	-4.8	1.4	cDNA FLJ41452 fis, clone BRSTN2010363. [AK123446]	AK123446	Hs.520589	XM_499022
USP30	-4.8	1.7	mRNA; cDNA DKFZp547N1013 (from clone DKFZp547N1013). [AL834278]	AL834278	Hs.486434	AL834278
ANXA8	-4.8	1.6	annexin A8 (ANXA8), mRNA [NM_001630]	NM_001630	Hs.700586	NM_001630
MSH2	-4.8	1.6	mutS homolog 2, colon cancer, nonpolyposis type 1 (E. coli) (MSH2), mRNA [NM_000251]	NM_000251	Hs.597656	AK223284
C20orf12	-4.8	2.1	chromosome 20 open reading frame 12 (C20orf12), mRNA [NM_018152]	NM_018152	Unknown	
MSH6	-4.8	2.2	mutS homolog 6 (E. coli) (MSH6), mRNA [NM_000179]	NM_000179	Hs.445052	BC071594
FZD4	-4.8	3.2	frizzled homolog 4 (Drosophila) (FZD4), mRNA [NM_012193]	NM_012193	Hs.591968	AB032417
MLLT3	-4.8	1.7	myeloid/lymphoid or mixed-lineage leukemia (trithorax homolog, Drosophila); translocated to, 3 (MLLT3), mRNA [NM_004529]	NM_004529	Hs.591085	NM_004529
A_24_P12762	-4.8	1.5	A_24_P127621	A_24_P127621	Unknown	
DW443340	-4.8	1.6	HHAGE041070 Human liver regeneration after partial hepatectomy cDNA, mRNA sequence [DW443340]	DW443340	Hs.631395	AI928355
KIAA1086	-4.8	2.7	mRNA for KIAA1086 protein, partial cds. [AB029009]	ENST0000026296	Unknown	
C20orf133	-4.8	2.6	chromosome 20 open reading frame 133 (C20orf133), transcript variant 1, mRNA [NM_080676]	NM_080676	Hs.661576	NM_080676
ORC4L	-4.8	1.8	origin recognition complex, subunit 4-like (yeast) (ORC4L), transcript variant 2, mRNA [NM_002552]	NM_002552	Hs.558364	NM_002552
MPPED1	-4.8	5.7	metallophosphoesterase domain containing 1 (MPPED1), mRNA [NM_001044370]	NM_001044370	Hs.592198	NM_001044370
LOC402176	-4.8	1.9	similar to 60S ribosomal protein L21 (LOC402176), mRNA [NM_001011538]	NM_001011538	Hs.452943	NM_001011538
ANUBL1	-4.8	1.6	AN1, ubiquitin-like, homolog (Xenopus laevis) (ANUBL1), mRNA [NM_174890]	NM_174890	Hs.89029	BC045587
TTC12	-4.8	1.7	tetratricopeptide repeat domain 12 (TTC12), mRNA [NM_017868]	NM_017868	Hs.288772	AK125909
PAFAH1B1	-4.8	1.3	platelet-activating factor acetylhydrolase, isoform Ib, alpha subunit 45kDa (PAFAH1B1), mRNA [NM_000430]	NM_000430	Hs.77318	NM_000430
HSD17B11	-4.8	2.1	hydroxysteroid (17-beta) dehydrogenase 11 (HSD17B11), mRNA [NM_016245]	NM_016245	Hs.284414	NM_178135
PLCXD1	-4.8	1.9	phosphatidylinositol-specific phospholipase C, X domain containing 1 (PLCXD1), mRNA [NM_018390]	NM_018390	Hs.522568	AK091006
FAM44A	-4.8	1.5	family with sequence similarity 44, member A (FAM44A), mRNA [NM_148894]	NM_148894	Hs.444517	NM_148894
EIF2C4	-4.8	1.9	Eukaryotic translation initiation factor 2C 4 (eIF2C 4) (eIF-2C 4) (Argonaute-4). [Source:Uniprot/SWISSPROT;Acc:Q9HCK5] [ENST00000373210]	ENST0000037321	Unknown	
FRMD4A	-4.8	2.0	cDNA FLJ10210 fis, clone HEMBA1006344, weakly similar to RADIXIN. [AK001072]	AK001072	Hs.330463	BC151244
RPL9	-4.8	1.6	ribosomal protein L9 (RPL9), transcript variant 2, mRNA [NM_001024921]	NM_001024921	Hs.412370	CR595992
THC2572360	-4.8	1.9	THC2572360	THC2572360	Unknown	
FLJ22374	-4.8	1.8	hypothetical protein FLJ22374 (FLJ22374), mRNA [NM_032222]	NM_032222	Hs.660192	AK128558
HNRPH1	-4.8	2.2	heterogeneous nuclear ribonucleoprotein H1 (H) (HNRPH1), mRNA [NM_005520]	NM_005520	Hs.604001	BX647205
THC2623121	-4.8	2.1	THC2623121	THC2623121	Unknown	
ZDHHC17	-4.8	1.9	zinc finger, DHHC-type containing 17 (ZDHHC17), mRNA [NM_015336]	NM_015336	Hs.4014	AB024494
COL2A1	-4.8	2.4	collagen, type II, alpha 1 (primary osteoarthritis, spondyloepiphyseal dysplasia, congenital) (COL2A1), transcript variant 1, mRNA [NM_001844]	NM_001844	Hs.408182	NM_001844
ABCC2	-4.8	2.1	ATP-binding cassette, sub-family C (CFTR/MRP), member 2 (ABCC2), mRNA [NM_000392]	NM_000392	Hs.368243	U49248

HMGB2	-4.8	2.4	high-mobility group box 2 (HMGB2), mRNA [NM_002129]	NM_002129	Hs.434953	CR600021
MSH2	-4.8	1.6	mutS homolog 2, colon cancer, nonpolyposis type 1 (E. coli) (MSH2), mRNA [NM_000251]	NM_000251	Hs.597656	AK223284
CEP290	-4.8	2.2	centrosomal protein 290kDa (CEP290), mRNA [NM_025114]	NM_025114	Hs.150444	NM_025114
NEK3	-4.8	1.7	NIMA (never in mitosis gene a)-related kinase 3 (NEK3), transcript variant 1, mRNA [NM_002498]	NM_002498	Hs.409989	AK131359
LOC731201	-4.8	2.3	PREDICTED: similar to zinc finger protein 91 (LOC731201), mRNA [XR_015409]	XR_015409	Hs.655305	XR_015409
A_24_P67656	-4.8	4.4	A_24_P676566	A_24_P676566	Unknown	
ATF7IP2	-4.8	2.1	activating transcription factor 7 interacting protein 2 (ATF7IP2), mRNA [NM_024997]	NM_024997	Hs.513343	BC033891
KIAA1009	-4.8	2.7	KIAA1009 (KIAA1009), mRNA [NM_014895]	NM_014895	Hs.485865	NM_014895
WASF3	-4.8	2.3	WAS protein family, member 3 (WASF3), mRNA [NM_006646]	NM_006646	Hs.635221	NM_006646
MND1	-4.8	2.2	meiotic nuclear divisions 1 homolog (S. cerevisiae) (MND1), mRNA [NM_032117]	NM_032117	Hs.294088	BG496847
A_24_P40952	-4.8	2.9	A_24_P409521	A_24_P409521	Unknown	
FLJ10292	-4.8	3.8	mago-nashi homolog 2 (FLJ10292), mRNA [NM_018048]	NM_018048	Hs.104650	NM_018048
THC2643977	-4.8	3.3	Q7TPA6_RAT (Q7TPA6) Ab1-042, partial (25%) [THC2643977]	THC2643977	Unknown	
LPA	-4.8	2.7	lipoprotein, Lp(a) (LPA), mRNA [NM_005577]	NM_005577	Hs.520120	X06290
RKHD3	-4.8	2.9	ring finger and KH domain containing 3 (RKHD3), mRNA [NM_032246]	NM_032246	Hs.104744	AK131424
BRD7	-4.8	1.7	bromodomain containing 7 (BRD7), mRNA [NM_013263]	NM_013263	Hs.437894	BC094706
Gcom1	-4.8	1.7	GRINL1A combined protein (Gcom1), transcript variant 1, mRNA [NM_001018090]	NM_001018090	Hs.437256	NM_001018090
ZNRD1	-4.8	2.4	zinc ribbon domain containing 1 (ZNRD1), transcript variant a, mRNA [NM_170783]	NM_170783	Hs.57813	BM557164
UNC5C	-4.8	3.5	unc-5 homolog C (C. elegans) (UNC5C), mRNA [NM_003728]	NM_003728	Hs.584831	AF055634
THC2491396	-4.8	2.5	CB133932 K-EST0185164 L9SNU354 cDNA clone L9SNU354-10-C01 5', mRNA sequence [CB133932]	THC2491396	Unknown	
ATM	-4.8	1.7	ataxia telangiectasia mutated (includes complementation groups A, C and D) (ATM), transcript variant 1, mRNA [NM_000051]	NM_000051	Hs.367437	NM_000051
HISPPD1	-4.8	1.9	histidine acid phosphatase domain containing 1 (HISPPD1), mRNA [NM_015216]	NM_015216	Hs.212046	AB007893
HADH	-4.8	1.6	hydroxyacyl-Coenzyme A dehydrogenase (HADH), nuclear gene encoding mitochondrial protein, mRNA [NM_005327]	NM_005327	Hs.438289	NM_005327
NPY1R	-4.8	2.3	neuropeptide Y receptor Y1 (NPY1R), mRNA [NM_000909]	NM_000909	Hs.519057	AB209237
RNF139	-4.8	1.5	ring finger protein 139 (RNF139), mRNA [NM_007218]	NM_007218	Hs.632057	NM_007218
RASSF3	-4.8	1.9	cDNA FLJ26410 fis, clone HRT09622. [AK129920]	AK129920	Unknown	
GSTA2	-4.8	2.8	glutathione S-transferase A2 (GSTA2), mRNA [NM_000846]	NM_000846	Hs.94107	BI762502
TMEM177	-4.8	2.3	transmembrane protein 177 (TMEM177), mRNA [NM_030577]	NM_030577	Hs.439991	AK057313
RNF130	-4.8	2.0	ring finger protein 130 (RNF130), mRNA [NM_018434]	NM_018434	Hs.484363	AL831873
LOC283152	-4.8	2.1	hypothetical protein LOC283152 (LOC283152), mRNA [NM_001033658]	NM_001033658	Hs.114777	BC043548
ADAM22	-4.8	1.9	ADAM metallopeptidase domain 22 (ADAM22), transcript variant 1, mRNA [NM_021723]	NM_021723	Hs.592282	NM_021723
RFX3	-4.8	2.6	Transcription factor RFX3. [Source:Uniprot/SWISSPROT;Acc:P48380] [ENST00000382004]	ENST0000038200	Unknown	
LOC339804	-4.8	2.0	hypothetical gene supported by AK075484; BC014578, mRNA (cDNA clone IMAGE:3950925). [BC014578]	BC014578	Hs.140617	CF272590
THC2617699	-4.8	1.9	AL353671 match: proteins: BAC05300 {Homo sapiens} (exp=0; wgp=1; cg=0), partial (12%) [THC2617699]	THC2617699	Unknown	
GJB2	-4.8	1.8	gap junction protein, beta 2, 26kDa (connexin 26) (GJB2), mRNA [NM_004004]	NM_004004	Hs.591234	NM_004004
C20orf75	-4.8	2.3	cDNA FLJ23994 fis, clone HRC11286. [AK172833]	AK172833	Hs.149133	BC027720
CR591264	-4.8	2.6	full-length cDNA clone CS0DC023YK19 of Neuroblastoma Cot 25-normalized of (human). [CR591264]	CR591264	Hs.556108	AK091492
ENST0000035	-4.8	2.2	FP588. [Source:Uniprot/SPTREMBL;Acc:Q71RF5] [ENST00000354349]	ENST0000035434	Unknown	
LOC400769	-4.8	2.9	PREDICTED: similar to Heterogeneous nuclear ribonucleoprotein A1 (Helix-destabilizing protein) (Single-strand RNA-binding protein) (hnRNP core protein A1) (HDP) (LOC400769), mRNA [XR_019349]	XR_019349	Hs.647696	XR_019349
GSTA5	-4.8	3.1	glutathione S-transferase A5 (GSTA5), mRNA [NM_153699]	NM_153699	Hs.553652	NM_153699
EPB41L5	-4.8	2.6	erythrocyte membrane protein band 4.1 like 5 (EPB41L5), mRNA [NM_020909]	NM_020909	Hs.654802	BC054508
CR619482	-4.8	1.6	full-length cDNA clone CS0DK012YM11 of HeLa cells Cot 25-normalized of (human). [CR619482]	CR619482	Hs.534012	XM_931016
FUSIP1	-4.8	2.4	FUS interacting protein (serine/arginine-rich) 1 (FUSIP1), transcript variant 2, mRNA [NM_054016]	NM_054016	Hs.3530	AK125834
LOC391595	-4.8	1.7	PREDICTED: similar to 60S ribosomal protein L32 (LOC391595), mRNA [XR_019529]	XR_019529	Hs.647922	XR_019529
THC2611971	-4.8	2.1	Q23IV5_TETTH (Q23IV5) Leucine Rich Repeat family protein, partial (3%) [THC2777279]	THC2611971	Unknown	
AS3MT	-4.8	1.9	arsenic (+3 oxidation state) methyltransferase (AS3MT), mRNA [NM_020682]	NM_020682	Hs.34492	NM_020682
C20orf94	-4.8	1.6	chromosome 20 open reading frame 94 (C20orf94), mRNA [NM_001009608]	NM_001009608	Hs.668782	NM_001009608
OSGEPL1	-4.8	2.0	O-sialoglycoprotein endopeptidase-like 1 (OSGEPL1), mRNA [NM_022353]	NM_022353	Hs.60772	NM_022353
KDELC1	-4.8	1.5	KDEL (Lys-Asp-Glu-Leu) containing 1 (KDELC1), mRNA [NM_024089]	NM_024089	Hs.408629	BC051860

MEOX2	-4.8	15.1	mesenchyme homeobox 2 (MEOX2), mRNA [NM_005924]	NM_005924	Hs.170355	NM_005924
RBBP6	-4.8	1.9	retinoblastoma binding protein 6 (RBBP6), transcript variant 1, mRNA [NM_006910]	NM_006910	Hs.188553	NM_006910
ZNF222	-4.8	1.5	zinc finger protein 222 (ZNF222), mRNA [NM_013360]	NM_013360	Hs.279840	AK095196
KIAA0430	-4.8	1.5	KIAA0430 (KIAA0430), mRNA [NM_014647]	NM_014647	Hs.173524	NM_014647
CDH7	-4.8	3.4	cadherin 7, type 2 (CDH7), transcript variant b, mRNA [NM_004361]	NM_004361	Hs.657522	BC036786
CHD2	-4.8	1.6	chromodomain helicase DNA binding protein 2 (CHD2), transcript variant 1, mRNA [NM_001271]	NM_001271	Hs.220864	NM_001271
C9orf39	-4.8	2.0	chromosome 9 open reading frame 39 (C9orf39), mRNA [NM_017738]	NM_017738	Hs.435381	BX647069
FLJ37440	-4.8	1.9	hypothetical protein FLJ37440 (FLJ37440), mRNA [NM_153214]	NM_153214	Hs.437696	CR933697
FAM92A1	-4.8	1.9	mRNA; cDNA DKFZp564I0278 (from clone DKFZp564I0278). [CR627475]	CR627475	Unknown	
THRAP2	-4.7	1.6	thyroid hormone receptor associated protein 2 (THRAP2), mRNA [NM_015335]	NM_015335	Hs.654691	NM_015335
NMU	-4.7	2.2	neuromedin U (NMU), mRNA [NM_006681]	NM_006681	Hs.418367	BF034907
AF143325	-4.7	2.1	clone IMAGE:110436 mRNA sequence. [AF143325]	AF143325	Hs.655976	AF143325
ENST0000032	-4.7	2.2	Plasminogen-related protein A (Fragment). [Source:Uniprot/SPTREMBL;Acc:Q9UE70] [ENST00000324709]	ENST0000032470	Unknown	
RPL30	-4.7	1.8	ribosomal protein L30 (RPL30), mRNA [NM_000989]	NM_000989	Hs.400295	AK128768
THC2533385	-4.7	1.7	THC2533385	THC2533385	Unknown	
SON	-4.7	1.5	SON DNA binding protein (SON), transcript variant f, mRNA [NM_138927]	NM_138927	Hs.517262	NM_138927
BC021073	-4.7	1.8	cDNA clone IMAGE:3010311, **** WARNING: chimeric clone ****. [BC021073]	BC021073	Unknown	
THC2563238	-4.7	2.0	THC2563238	THC2563238	Unknown	
LYRM4	-4.7	2.0	LYR motif containing 4 (LYRM4), mRNA [NM_020408]	NM_020408	Hs.696067	NM_020408
ALS2CR8	-4.7	5.7	ALS2CR8 mRNA, complete cds, short form. [AB053310]	AB053310	Hs.444982	NM_024744
KLB	-4.7	5.5	Beta klotho (BetaKlotho) (Klotho beta-like protein). [Source:Uniprot/SWISSPROT;Acc:Q86Z14] [ENST00000381889]	ENST0000038188	Unknown	
VAV3	-4.7	4.4	vav 3 oncogene (VAV3), transcript variant 1, mRNA [NM_006113]	NM_006113	Hs.267659	NM_006113
BC035417	-4.7	1.7	Homo sapiens, clone IMAGE:5167600, mRNA. [BC035417]	BC035417	Hs.178144	BM563086
A_24_P21259	-4.7	2.6	A_24_P212596	A_24_P212596	Unknown	
WDR60	-4.7	1.4	WD repeat domain 60 (WDR60), mRNA [NM_018051]	NM_018051	Hs.389945	NM_018051
YTHDC1	-4.7	2.8	YTH domain containing 1 (YTHDC1), transcript variant 1, mRNA [NM_001031732]	NM_001031732	Hs.175955	NM_001031732
AY952884	-4.7	1.6	antigen MMSA-4 mRNA sequence. [AY952884]	AY952884	Hs.374596	BG033621
NHS	-4.7	3.7	Nance-Horan syndrome (congenital cataracts and dental anomalies) (NHS), mRNA [NM_198270]	NM_198270	Hs.201623	AY456993
ZNF673	-4.7	1.8	zinc finger protein 673 (ZNF673), mRNA [NM_017776]	NM_017776	Hs.632800	AK097159
ZNF187	-4.7	1.6	zinc finger protein 187 (ZNF187), transcript variant 1, mRNA [NM_007151]	NM_007151	Hs.157883	NM_007151
MSH2	-4.7	1.6	mutS homolog 2, colon cancer, nonpolyposis type 1 (E. coli) (MSH2), mRNA [NM_000251]	NM_000251	Hs.597656	AK223284
THC2650120	-4.7	2.6	ALU6_HUMAN (P39193) Alu subfamily SP sequence contamination warning entry, partial (14%) [THC2650120]	THC2650120	Unknown	
PRODH2	-4.7	4.8	proline dehydrogenase (oxidase) 2 (PRODH2), mRNA [NM_021232]	NM_021232	Hs.515366	AK130040
DQ786246	-4.7	2.0	clone HLS_IMAGE_204740 mRNA sequence. [DQ786246]	DQ786246	Hs.374577	DQ786246
SEMA6A	-4.7	3.3	sema domain, transmembrane domain (TM), and cytoplasmic domain, (semaphorin) 6A (SEMA6A), mRNA [NM_020796]	NM_020796	Hs.156967	BC032619
AKAP11	-4.7	1.5	A kinase (PRKA) anchor protein 11 (AKAP11), transcript variant 1, mRNA [NM_016248]	NM_016248	Hs.105105	AF176555
POU4F1	-4.7	3.2	POU domain, class 4, transcription factor 1 (POU4F1), mRNA [NM_006237]	NM_006237	Hs.654522	L20433
EP400	-4.7	1.7	E1A binding protein p400 (EP400), mRNA [NM_015409]	NM_015409	Hs.699245	NM_015409
KYNU	-4.7	3.5	kynureninase (L-kynurenine hydrolase) (KYNU), transcript variant 1, mRNA [NM_003937]	NM_003937	Hs.470126	CR609484
LOC649314	-4.7	1.9	cDNA FLJ35212 fis, clone PROST1000136. [AK092531]	AK092531	Hs.648444	AK092544
LOC392497	-4.7	1.8	PREDICTED: similar to 40S ribosomal protein S6 (LOC392497), mRNA [XR_018138]	XR_018138	Hs.648325	XR_018138
THC2618142	-4.7	3.5	THC2618142	THC2618142	Unknown	
THC2718727	-4.7	1.8	THC2718727	THC2718727	Unknown	
CD52	-4.7	2.3	CD52 molecule (CD52), mRNA [NM_001803]	NM_001803	Hs.276770	BQ276596
KIAA1411	-4.7	1.7	KIAA1411 (KIAA1411), mRNA [NM_020819]	NM_020819	Hs.211700	AB037832
SEPHS1	-4.7	2.7	Selenide, water dikinase 1 (EC 2.7.9.3) (Selenophosphate synthetase 1) (Selenium donor protein 1). [Source:Uniprot/SWISSPROT;Acc:P49903] [ENST00000327347]	ENST0000032734	Unknown	
BC041959	-4.7	2.6	cDNA clone IMAGE:5302136. [BC041959]	BC041959	Hs.655573	BC041959
FBXL10	-4.7	1.8	F-box and leucine-rich repeat protein 10 (FBXL10), transcript variant 1, mRNA [NM_032590]	NM_032590	Hs.524800	NM_032590
BC052613	-4.7	1.5	cDNA clone MGC:59872 IMAGE:6301163, complete cds. [BC052613]	BC052613	Hs.674392	BC052613
U01925	-4.7	2.3	Human BTK region clone 2f10-rpi mRNA. [U01925]	U01925	Unknown	
PUM2	-4.7	1.8	pumilio homolog 2 (Drosophila) (PUM2), mRNA [NM_015317]	NM_015317	Hs.467824	AF315591

FLJ30428	-4.7	4.7	cDNA FLJ30428 fis, clone BRACE2008941. [AK054990]	AK054990	Hs.131740	BC131487
METTL9	-4.7	1.7	methyltransferase like 9 (METTL9), transcript variant 1, mRNA [NM_016025]	NM_016025	Hs.279583	NM_016025
MCM3APAS	-4.7	2.2	MCM3 minichromosome maintenance deficient 3 (S. cerevisiae) associated protein antisense (MCM3APAS) on chromosome 21 [NR_002776]	NR_002776	Unknown	
MOGAT1	-4.7	3.1	monoacylglycerol O-acyltransferase 1 (MOGAT1), mRNA [NM_058165]	NM_058165	Hs.344090	NM_058165
G36631	-4.7	2.6	SHGC-53577 Human STS cDNA, sequence tagged site. [G36631]	G36631	Unknown	
AK098605	-4.7	1.9	cDNA FLJ25739 fis, clone TST05834. [AK098605]	AK098605	Hs.677723	XR_015825
KIAA1344	-4.7	2.0	KIAA1344 (KIAA1344), mRNA [NM_020784]	NM_020784	Hs.532609	BC142650
A_32_P19418	-4.7	3.0	A_32_P194182	A_32_P194182	Unknown	
A_24_P39219	-4.7	2.0	A_24_P392195	A_24_P392195	Unknown	
TTC28	-4.7	1.5	cDNA FLJ35019 fis, clone OCBBF2014541. [AK092338]	AK092338	Hs.387856	XM_929318
ZNF613	-4.7	2.2	cDNA FLJ13590 fis, clone PLACE1009398, moderately similar to ZINC FINGER PROTEIN 135. [AK023652]	AK023652	Hs.183390	BC057776
SERPINA6	-4.7	2.9	serpin peptidase inhibitor, clade A (alpha-1 antiproteinase, antitrypsin), member 6 (SERPINA6), mRNA [NM_001756]	NM_001756	Hs.532635	BC036818
AK096984	-4.7	2.1	cDNA FLJ39665 fis, clone SMINT2007294. [AK096984]	AK096984	Hs.373571	AK096984
A_24_P28957	-4.7	2.0	A_24_P289573	A_24_P289573	Unknown	
LOC391701	-4.7	2.3	PREDICTED: similar to ribosomal protein S23 (LOC391701), mRNA [XR_018509]	XR_018509	Hs.648018	XR_018509
BC038747	-4.7	1.9	cDNA clone IMAGE:5268658. [BC038747]	BC038747	Hs.572495	BC038747
MGC24103	-4.7	2.2	hypothetical protein MGC24103, mRNA (cDNA clone MGC:24103 IMAGE:4613905), complete cds. [BC020879]	BC020879	Hs.664877	BX648870
A_24_P10166	-4.7	2.3	A_24_P101661	A_24_P101661	Unknown	
CR593845	-4.7	1.4	full-length cDNA clone CS0DF024YI14 of Fetal brain of (human). [CR593845]	CR593845	Hs.670258	CR620748
CCT8	-4.7	3.6	chaperonin containing TCP1, subunit 8 (theta), mRNA (cDNA clone IMAGE:3930332), complete cds. [BC005220]	BC005220	Hs.125113	BC012584
TAS2R48	-4.7	3.2	taste receptor, type 2, member 48 (TAS2R48), mRNA [NM_176888]	NM_176888	Hs.688196	BC111998
KHDRBS1	-4.7	2.0	KH domain containing, RNA binding, signal transduction associated 1 (KHDRBS1), mRNA [NM_006559]	NM_006559	Hs.699176	BC010132
BX098326	-4.7	2.1	BX098326 NCI_CGAP_CLL1 cDNA clone IMAGp998A025198, mRNA sequence [BX098326]	BX098326	Hs.306727	BX098326
10-Sep	-4.7	1.7	septin 10 (SEPT10), transcript variant 1, mRNA [NM_144710]	NM_144710	Hs.469615	AB208875
RAVER2	-4.7	2.3	ribonucleoprotein, PTB-binding 2 (RAVER2), mRNA [NM_018211]	NM_018211	Hs.591443	NM_018211
HK2	-4.7	2.0	hexokinase 2 (HK2), mRNA [NM_000189]	NM_000189	Hs.591588	NM_000189
PDCL	-4.7	2.7	phosducin-like (PDCL), mRNA [NM_005388]	NM_005388	Hs.271749	NM_005388
KDELC1	-4.7	1.6	KDEL (Lys-Asp-Glu-Leu) containing 1 (KDELC1), mRNA [NM_024089]	NM_024089	Hs.408629	BC051860
TMTC4	-4.7	2.5	transmembrane and tetratricopeptide repeat containing 4 (TMTC4), transcript variant 1, mRNA [NM_032813]	NM_032813	Hs.696247	AK124599
FLJ39660	-4.7	1.9	hypothetical protein FLJ39660, mRNA (cDNA clone IMAGE:4556987), complete cds. [BC062449]	BC062449	Hs.132519	NM_001080539
THC2554453	-4.7	1.8	HUMRRL3A ribosomal protein L3 {Homo sapiens} (exp=-1; wgp=0; cg=0), partial (28%) [THC2554453]	THC2554453	Unknown	
CD83	-4.7	2.0	CD83 molecule (CD83), transcript variant 1, mRNA [NM_004233]	NM_004233	Hs.654558	NM_004233
ANAPC4	-4.7	1.7	anaphase promoting complex subunit 4 (ANAPC4), mRNA [NM_013367]	NM_013367	Hs.152173	AL353932
MYST4	-4.7	2.3	MYST histone acetyltransferase (monocytic leukemia) 4 (MYST4), mRNA [NM_012330]	NM_012330	Hs.35758	NM_012330
RPS23	-4.7	1.8	ribosomal protein S23 (RPS23), mRNA [NM_001025]	NM_001025	Hs.527193	AK125939
DCLRE1B	-4.7	1.7	DNA cross-link repair 1B (PSO2 homolog, S. cerevisiae) (DCLRE1B), mRNA [NM_022836]	NM_022836	Hs.591412	NM_022836
BNIP2	-4.7	1.3	BCL2/adenovirus E1B 19kDa interacting protein 2 (BNIP2), mRNA [NM_004330]	NM_004330	Hs.646490	BC002461
MSH2	-4.7	1.6	mutS homolog 2, colon cancer, nonpolyposis type 1 (E. coli) (MSH2), mRNA [NM_000251]	NM_000251	Hs.597656	AK223284
C1orf21	-4.7	2.7	chromosome 1 open reading frame 21 (C1orf21), mRNA [NM_030806]	NM_030806	Hs.497159	NM_030806
THC2666431	-4.7	2.3	THC2666431	THC2666431	Unknown	
RAD21	-4.7	1.7	RAD21 homolog (S. pombe) (RAD21), mRNA [NM_006265]	NM_006265	Hs.81848	NM_006265
C20orf56	-4.7	7.4	chromosome 20 open reading frame 56 (C20orf56) on chromosome 20 [NR_001558]	NR_001558	Unknown	
NSBP1	-4.7	2.2	nucleosomal binding protein 1 (NSBP1), mRNA [NM_030763]	NM_030763	Hs.282204	AF250329
AHSG	-4.7	3.2	alpha-2-HS-glycoprotein (AHSG), mRNA [NM_001622]	NM_001622	Hs.324746	BC048198
HISPPD1	-4.7	1.9	histidine acid phosphatase domain containing 1 (HISPPD1), mRNA [NM_015216]	NM_015216	Hs.212046	AB007893
ORM2	-4.7	3.0	orosomucoid 2 (ORM2), mRNA [NM_000608]	NM_000608	Hs.522356	AK055563
LOC650177	-4.7	2.7	PREDICTED: similar to sphingomyelinase, intestinal alkaline (LOC650177), mRNA [XR_018734]	XR_018734	Hs.613123	XR_018734
NEK3	-4.7	2.2	NIMA (never in mitosis gene a)-related kinase 3 (NEK3), transcript variant 1, mRNA [NM_002498]	NM_002498	Hs.409989	AK131359
A_24_P17833	-4.7	1.7	A_24_P178333	A_24_P178333	Unknown	
HS2ST1	-4.7	2.1	heparan sulfate 2-O-sulfotransferase 1 (HS2ST1), mRNA [NM_012262]	NM_012262	Hs.48823	NM_012262

TMEM181	-4.7	1.5	mRNA for KIAA1423 protein, partial cds. [AB037844]	AB037844	Hs.99145	NM_020823
PUM2	-4.7	1.7	pumilio homolog 2 (Drosophila) (PUM2), mRNA [NM_015317]	NM_015317	Hs.467824	AF315591
NSBP1	-4.7	2.1	nucleosomal binding protein 1 (NSBP1), mRNA [NM_030763]	NM_030763	Hs.282204	AF250329
MCM3	-4.7	1.7	MCM3 minichromosome maintenance deficient 3 (S. cerevisiae) (MCM3), mRNA [NM_002388]	NM_002388	Hs.179565	NM_002388
A_24_P29861	-4.7	1.9	A_24_P298616	A_24_P298616	Unknown	
CDKN1B	-4.7	1.5	cyclin-dependent kinase inhibitor 1B (p27, Kip1) (CDKN1B), mRNA [NM_004064]	NM_004064	Hs.238990	NM_004064
DACH1	-4.7	3.4	dachshund homolog 1 (Drosophila) (DACH1), transcript variant 1, mRNA [NM_080759]	NM_080759	Hs.129452	NM_080759
AHSG	-4.7	3.3	alpha-2-HS-glycoprotein (AHSG), mRNA [NM_001622]	NM_001622	Hs.324746	BC048198
LOC284288	-4.6	2.7	PREDICTED: similar to ribosomal protein L24-like (LOC284288), mRNA [XM_210365]	ENST0000026947	Unknown	
HSD17B11	-4.6	2.1	hydroxysteroid (17-beta) dehydrogenase 11 (HSD17B11), mRNA [NM_016245]	NM_016245	Hs.284414	NM_178135
RPL21	-4.6	1.7	ribosomal protein L21 (RPL21), mRNA [NM_000982]	NM_000982	Hs.381123	CR602527
GBP4	-4.6	3.7	guanylate binding protein 4 (GBP4), mRNA [NM_052941]	NM_052941	Hs.409925	AL832576
FIGF	-4.6	43.5	c-fos induced growth factor (vascular endothelial growth factor D) (FIGF), mRNA [NM_004469]	NM_004469	Hs.11392	NM_004469
KIAA1712	-4.6	2.3	KIAA1712 (KIAA1712), mRNA [NM_001040157]	NM_001040157	Hs.555989	NM_001040157
C10orf82	-4.6	3.3	cDNA FLJ40268 fis, clone TESTI2026537. [AK097587]	ENST0000038888	Unknown	
THC2605327	-4.6	3.8	AB013803 hNB-2s {Homo sapiens} (exp=-1; wgp=0; cg=0), partial (3%) [THC2605327]	THC2605327	Unknown	
TBX4	-4.6	5.2	T-box 4 (TBX4), mRNA [NM_018488]	NM_018488	Hs.143907	BC142620
RPL31	-4.6	1.8	ribosomal protein L31, mRNA (cDNA clone MGC:88191 IMAGE:4714258), complete cds. [BC070210]	BC070210	Hs.469473	CR595074
FLJ32745	-4.6	3.5	cDNA FLJ13088 fis, clone NT2RP3002102. [AK023150]	AK023150	Hs.362702	AK023150
VCY	-4.6	2.0	variable charge, Y-linked (VCY), mRNA [NM_004679]	NM_004679	Hs.632284	AF000979
LOC732182	-4.6	2.7	PREDICTED: similar to Heterogeneous nuclear ribonucleoprotein A1 (Helix-destabilizing protein) (Single-strand RNA-binding protein) (hnRNP core protein A1) (HDP) (LOC732182), mRNA [XR_015752]	XR_015752	Hs.646819	XR_015752
LOC344423	-4.6	1.7	cDNA FLJ42751 fis, clone BRAWH3000491, moderately similar to 40S ribosomal protein S12. [AK124741]	AK124741	Hs.585046	AK124741
KIAA1856	-4.6	1.8	cDNA FLJ14435 fis, clone HEMBA1007085. [AK027341]	AK027341	Hs.556754	NM_001080495
A_32_P31827	-4.6	2.4	A_32_P31827	A_32_P31827	Unknown	
DTL	-4.6	1.8	denticleless homolog (Drosophila) (DTL), mRNA [NM_016448]	NM_016448	Hs.656473	NM_016448
PTPN13	-4.6	3.6	protein tyrosine phosphatase, non-receptor type 13 (APO-1/CD95 (Fas)-associated phosphatase) (PTPN13), transcript variant 4, mRNA [NM_080685]	NM_080685	Hs.436142	U12128
THC2544212	-4.6	1.7	Q96I38_HUMAN (Q96I38) EEF1D protein (Eukaryotic translation elongation factor 1 delta) (Guanine nucleotide exchange protein), partial (37%) [THC2544212]	THC2544212	Unknown	
TIFA	-4.6	1.8	TRAF-interacting protein with a forkhead-associated domain (TIFA), mRNA [NM_052864]	NM_052864	Hs.310640	NM_052864
THC2614136	-4.6	3.2	THC2614136	THC2614136	Unknown	
ZNF452	-4.6	2.4	zinc finger protein 452 (ZNF452), mRNA [NM_052923]	NM_052923	Hs.176980	AY517631
NSBP1	-4.6	2.1	nucleosomal binding protein 1 (NSBP1), mRNA [NM_030763]	NM_030763	Hs.282204	AF250329
UHRF2	-4.6	1.8	ubiquitin-like, containing PHD and RING finger domains, 2 (UHRF2), mRNA [NM_152896]	NM_152896	Hs.493401	BC028397
AI801879	-4.6	4.3	tx28f05.x1 NCI_CGAP_Lu24 cDNA clone IMAGE:2270913 3', mRNA sequence [AI801879]	AI801879	Hs.144030	AI801879
GATS	-4.6	2.0	opposite strand transcription unit to STAG3, mRNA (cDNA clone MGC:102871 IMAGE:6179098), complete cds. [BC090867]	BC090867	Hs.556063	AL831967
ZNF571	-4.6	1.9	zinc finger protein 571 (ZNF571), mRNA [NM_016536]	NM_016536	Hs.590944	BC114479
AK096225	-4.6	4.4	cDNA FLJ38906 fis, clone NT2NE2004378. [AK096225]	AK096225	Hs.659458	AK096225
THC2677432	-4.6	5.4	THC2677432	THC2677432	Unknown	
THC2610628	-4.6	2.7	THC2610628	THC2610628	Unknown	
PSIP1	-4.6	1.9	PC4 and SFRS1 interacting protein 1 (PSIP1), transcript variant 2, mRNA [NM_033222]	NM_033222	Hs.658434	BX649155
RPS3A	-4.6	1.7	ribosomal protein S3A (RPS3A), mRNA [NM_001006]	NM_001006	Hs.356572	BI087817
RGPD2	-4.6	1.4	RANBP2-like and GRIP domain containing 2 (RGPD2), mRNA [NM_001078170]	NM_001078170	Hs.656849	CR749330
A_24_P33420	-4.6	2.5	A_24_P334208	A_24_P334208	Unknown	
THC2743390	-4.6	1.8	THC2743390	THC2743390	Unknown	
MAGEF1	-4.6	1.4	melanoma antigen family F, 1 (MAGEF1), mRNA [NM_022149]	NM_022149	Hs.306123	NM_022149
RP11-78J21.1	-4.6	2.5	heterogeneous nuclear ribonucleoprotein A1-like (LOC144983), transcript variant 1, mRNA [NM_001011724]	NM_001011724	Hs.447506	AK126454
ZNF643	-4.6	2.8	zinc finger protein 643 (ZNF643), mRNA [NM_023070]	NM_023070	Hs.133034	AK091612
C8orf40	-4.6	1.7	chromosome 8 open reading frame 40 (C8orf40), mRNA [NM_138436]	NM_138436	Hs.655320	CR619782
BX109843	-4.6	2.0	BX109843 Soares placenta Nb2HP cDNA clone IMAGp998B14208, mRNA sequence [BX109843]	BX109843	Hs.78050	BX109843
XRN1	-4.6	1.7	5'-3' exoribonuclease 1 (XRN1), transcript variant 1, mRNA [NM_019001]	NM_019001	Hs.435103	BX640905

AL137571	-4.6	3.9	mRNA; cDNA DKFZp586O1621 (from clone DKFZp586O1621). [AL137571]	AL137571	Unknown	
ZNF678	-4.6	1.9	zinc finger protein 678 (ZNF678), mRNA [NM_178549]	NM_178549	Hs.656372	AK026475
COQ5	-4.6	1.6	coenzyme Q5 homolog, methyltransferase (S. cerevisiae) (COQ5), mRNA [NM_032314]	NM_032314	Hs.700658	BX647562
TIMELESS	-4.6	1.8	timeless homolog (Drosophila) (TIMELESS), mRNA [NM_003920]	NM_003920	Hs.118631	BC050557
AK123972	-4.6	1.9	cDNA FLJ41978 fis, clone SKNSH2000482. [AK123972]	AK123972	Hs.435458	BC146776
RERG	-4.6	2.4	RAS-like, estrogen-regulated, growth inhibitor (RERG), mRNA [NM_032918]	NM_032918	Hs.199487	BC007997
ENST0000026	-4.6	2.1	mRNA for KIAA1546 protein, partial cds. [AB046766]	ENST0000026514	Unknown	
C1QTNF7	-4.6	7.5	C1q and tumor necrosis factor related protein 7 (C1QTNF7), mRNA [NM_031911]	NM_031911	Hs.153714	BX647781
GYG2	-4.6	2.1	glycogenin 2 (GYG2), transcript variant 2, mRNA [NM_003918]	NM_003918	Hs.567381	NM_003918
AKAP7	-4.6	2.3	A kinase (PRKA) anchor protein 7 (AKAP7), transcript variant gamma, mRNA [NM_016377]	NM_016377	Hs.486483	AF152929
SMAD2	-4.6	2.1	SMAD family member 2 (SMAD2), transcript variant 2, mRNA [NM_001003652]	NM_001003652	Hs.12253	NM_001003652
THC2672768	-4.6	2.1	THC2672768	THC2672768	Unknown	
NSBP1	-4.6	2.2	nucleosomal binding protein 1 (NSBP1), mRNA [NM_030763]	NM_030763	Hs.282204	AF250329
THC2551774	-4.6	3.6	HUMSEF21A SEF2-1A protein {Homo sapiens} (exp=-1; wgp=0; cg=0), partial (5%) [THC2551774]	THC2551774	Unknown	
THC2734388	-4.6	1.7	MMFXR1H9 fragile-X-related protein 1 isoform f {Mus musculus} (exp=-1; wgp=0; cg=0), partial (4%) [THC2734388]	THC2734388	Unknown	
LOC285047	-4.6	3.6	PREDICTED: hypothetical LOC285047 (LOC285047), mRNA [XM_211749]	XM_211749	Hs.369330	XM_211749
DNMT3A	-4.6	1.9	DNA (cytosine-5-)-methyltransferase 3 alpha (DNMT3A), transcript variant 1, mRNA [NM_175629]	NM_175629	Hs.515840	AB208833
AF119905	-4.6	4.0	PRO2853 mRNA, complete cds. [AF119905]	AF119905	Unknown	
KLHL15	-4.6	1.8	Kelch-like protein 15. [Source:Uniprot/SWISSPROT;Acc:Q96M94] [ENST00000328046]	ENST0000032804	Unknown	
ETAA1	-4.6	1.8	Ewing's tumor-associated antigen 1 (ETAA1), mRNA [NM_019002]	NM_019002	Hs.353022	AJ242682
HISPPD1	-4.6	1.9	histidine acid phosphatase domain containing 1 (HISPPD1), mRNA [NM_015216]	NM_015216	Hs.212046	AB007893
CCNL1	-4.6	1.5	cyclin L1 (CCNL1), mRNA [NM_020307]	NM_020307	Hs.4859	BX641146
A_24_P13615	-4.6	2.0	A_24_P136155	A_24_P136155	Unknown	
LOC150383	-4.6	1.5	similar to RIKEN cDNA 2210021J22, mRNA (cDNA clone MGC:87534 IMAGE:30338205), complete cds. [BC067871]	BC067871	Hs.592209	BC036462
SDK1	-4.6	1.8	sidekick homolog 1 (chicken) (SDK1), transcript variant 1, mRNA [NM_152744]	NM_152744	Hs.655699	NM_152744
MSH2	-4.6	1.6	mutS homolog 2, colon cancer, nonpolyposis type 1 (E. coli) (MSH2), mRNA [NM_000251]	NM_000251	Hs.597656	AK223284
CHST9	-4.6	4.0	carbohydrate (N-acetylgalactosamine 4-0) sulfotransferase 9 (CHST9), mRNA [NM_031422]	NM_031422	Hs.657938	NM_031422
ZNF438	-4.6	1.7	zinc finger protein 438 (ZNF438), mRNA [NM_182755]	NM_182755	Hs.660642	AK097092
CCDC15	-4.6	1.6	coiled-coil domain containing 15 (CCDC15), mRNA [NM_025004]	NM_025004	Hs.287555	NM_025004
CCT6AP1	-4.6	2.5	cDNA FLJ34861 fis, clone NT2NE2012847. [AK092180]	AK092180	Unknown	
HPN	-4.6	2.1	hepsin (transmembrane protease, serine 1) (HPN), transcript variant 1, mRNA [NM_182983]	NM_182983	Hs.182385	AK125670
GPC5	-4.6	3.1	glypican 5 (GPC5), mRNA [NM_004466]	NM_004466	Hs.655675	NM_004466
A_24_P75550	-4.6	1.8	A_24_P755505	A_24_P755505	Unknown	
MRPL16	-4.6	1.9	mitochondrial ribosomal protein L16 (MRPL16), nuclear gene encoding mitochondrial protein, mRNA [NM_017840]	NM_017840	Hs.530734	BQ058130
ZNF694	-4.6	1.4	zinc finger protein 694 (ZNF694), mRNA [NM_001012981]	NM_001012981	Hs.513451	BX648785
GPBAR1	-4.6	1.7	G protein-coupled bile acid receptor 1 (GPBAR1), transcript variant 1, mRNA [NM_001077191]	NM_001077191	Hs.160954	NM_001077191
ANGPTL6	-4.6	3.1	angiopoietin-like 6 (ANGPTL6), mRNA [NM_031917]	NM_031917	Hs.699819	AF230330
CDCA7L	-4.6	2.2	cell division cycle associated 7-like (CDCA7L), mRNA [NM_018719]	NM_018719	Hs.520245	AK095965
LOC128977	-4.6	1.3	hypothetical protein LOC128977 (LOC128977), mRNA [NM_173793]	NM_173793	Hs.659565	AK095271
A_32_P93584	-4.6	2.3	A_32_P93584	A_32_P93584	Unknown	
KCNV1	-4.6	4.3	potassium channel, subfamily V, member 1 (KCNV1), mRNA [NM_014379]	NM_014379	Hs.13285	NM_014379
RABL3	-4.6	1.4	RAB, member of RAS oncogene family-like 3 (RABL3), mRNA [NM_173825]	NM_173825	Hs.444360	NM_173825
A_32_P16347	-4.6	2.3	A_32_P163472	A_32_P163472	Unknown	
FLJ14712	-4.6	2.5	cDNA FLJ14712 fis, clone NT2RP3000825, weakly similar to NEUROGENIC LOCUS NOTCH 3 PROTEIN. [AK027618]	AK027618	Hs.669526	AK027618
TMEM107	-4.6	1.7	transmembrane protein 107 (TMEM107), transcript variant 1, mRNA [NM_032354]	NM_032354	Hs.513933	AK127891
AK055641	-4.6	2.1	cDNA FLJ31079 fis, clone HSYRA2001595. [AK055641]	AK055641	Hs.349283	AK095108
MSH2	-4.6	1.6	mutS homolog 2, colon cancer, nonpolyposis type 1 (E. coli) (MSH2), mRNA [NM_000251]	NM_000251	Hs.597656	AK223284
ONECUT1	-4.6	2.1	one cut domain, family member 1 (ONECUT1), mRNA [NM_004498]	NM_004498	Hs.658573	U96173
GPR125	-4.6	1.9	G protein-coupled receptor 125 (GPR125), mRNA [NM_145290]	NM_145290	Hs.99195	NM_145290
THC2587773	-4.5	2.1	HUMUBCP pro-ubiquitin {Homo sapiens} (exp=-1; wgp=0; cg=0), partial (34%) [THC2587773]	THC2587773	Unknown	

BCKDHB	-4.5	2.2	branched chain keto acid dehydrogenase E1, beta polypeptide (maple syrup urine disease) (BCKDHB), nuclear gene encoding mitochondrial protein, transcript variant 1, mRNA [NM_183050]	NM_183050	Hs.654441	BC034481
E2F3	-4.5	1.7	E2F transcription factor 3 (E2F3), mRNA [NM_001949]	NM_001949	Hs.269408	NM_001949
A_32_P68205	-4.5	2.2	A_32_P68205	A_32_P68205	Unknown	
LANCL1	-4.5	1.7	LanC lantibiotic synthetase component C-like 1 (bacterial) (LANCL1), mRNA [NM_006055]	NM_006055	Hs.13351	NM_006055
THC2575761	-4.5	1.9	HUMRPL7Y ribosomal protein L7 {Homo sapiens} (exp=-1; wgp=0; cg=0), partial (40%) [THC2575761]	THC2575761	Unknown	
RPS16	-4.5	1.6	ribosomal protein S16 (RPS16), mRNA [NM_001020]	NM_001020	Hs.397609	AK055869
AK026497	-4.5	1.6	cDNA: FLJ22844 fis, clone KAIA5181. [AK026497]	AK026497	Hs.388927	BX647456
NHLRC2	-4.5	2.6	cDNA FLJ44798 fis, clone BRACE3040863. [AK126751]	AK126751	Hs.594372	BX647663
GSTA1	-4.5	2.4	glutathione S-transferase A1 (GSTA1), mRNA [NM_145740]	NM_145740	Hs.446309	AL096729
THC2550474	-4.5	3.1	THC2550474	THC2550474	Unknown	
AHI1	-4.5	2.2	Abelson helper integration site 1 (AHI1), mRNA [NM_017651]	NM_017651	Hs.386684	NM_017651
AK022443	-4.5	2.1	cDNA FLJ12381 fis, clone MAMMA1002566. [AK022443]	AK022443	Hs.656237	AK022443
KDELC1	-4.5	1.5	KDEL (Lys-Asp-Glu-Leu) containing 1 (KDELC1), mRNA [NM_024089]	NM_024089	Hs.408629	BC051860
KDELC1	-4.5	1.5	KDEL (Lys-Asp-Glu-Leu) containing 1 (KDELC1), mRNA [NM_024089]	NM_024089	Hs.408629	BC051860
A_24_P23842	-4.5	1.6	A_24_P238426	A_24_P238426	Unknown	
TSC1	-4.5	1.5	tuberous sclerosis 1 (TSC1), transcript variant 1, mRNA [NM_000368]	NM_000368	Hs.370854	NM_000368
LOC375295	-4.5	2.7	hypothetical gene supported by BC013438, mRNA (cDNA clone IMAGE:3899073), partial cds. [BC013438]	ENST0000029554	Unknown	
THG1L	-4.5	1.8	interphase cytoplasmic foci protein 45 [Source:RefSeq_peptide;Acc:NP_060342] [ENST00000231198]	ENST0000023119	Unknown	
TCF7L2	-4.5	2.1	Transcription factor 7-like 2 (HMG box transcription factor 4) (T- cell-specific transcription factor 4) (TCF-4) (hTCF-4). [Source:Uniprot/SWISSPROT;Acc:Q9NQB0] [ENST00000369397]	ENST0000036939	Unknown	
CR626634	-4.5	2.2	full-length cDNA clone CS0DI056YF22 of Placenta Cot 25-normalized of (human). [CR626634]	CR626634	Hs.293236	XM_085831
MKLN1	-4.5	1.9	muskelin 1, intracellular mediator containing kelch motifs (MKLN1), mRNA [NM_013255]	NM_013255	Hs.44693	NM_013255
HAL	-4.5	4.3	histidine ammonia-lyase (HAL), mRNA [NM_002108]	NM_002108	Hs.190783	NM_002108
TNRC15	-4.5	1.5	trinucleotide repeat containing 15 (TNRC15), mRNA [NM_015575]	NM_015575	Hs.565319	BC146775
CD34	-4.5	3.0	CD34 molecule (CD34), transcript variant 2, mRNA [NM_001773]	NM_001773	Hs.374990	BX640941
ENY2	-4.5	2.9	cDNA FLJ38332 fis, clone FCBBF3025528. [AK095651]	AK095651	Hs.492555	AK095651
ZNF3	-4.5	1.6	zinc finger protein 3 (ZNF3), transcript variant 2, mRNA [NM_032924]	NM_032924	Hs.435302	BX647182
NAT8	-4.5	5.3	N-acetyltransferase 8 (NAT8), mRNA [NM_003960]	NM_003960	Hs.14637	BQ644205
NAALAD2	-4.5	2.7	N-acetylated alpha-linked acidic dipeptidase 2 (NAALAD2), mRNA [NM_005467]	NM_005467	Hs.503560	AK075390
PCTP	-4.5	2.0	phosphatidylcholine transfer protein (PCTP), mRNA [NM_021213]	NM_021213	Unknown	
C7orf31	-4.5	2.2	chromosome 7 open reading frame 31 (C7orf31), mRNA [NM_138811]	NM_138811	Hs.122055	NM_138811
LOC641784	-4.5	1.6	xr55h07.x1 NCI_CGAP_Ov26 cDNA clone IMAGE:2764093 3' similar to gb:X69181 60S RIBOSOMAL PROTEIN L31 (HUMAN);, mRNA sequence [AW302767]	AW302767	Hs.647521	AW302767
MBNL3	-4.5	3.6	muscleblind-like 3 (Drosophila) (MBNL3), transcript variant R, mRNA [NM_133486]	NM_133486	Hs.105134	NM_018388
COL27A1	-4.5	1.6	cDNA FLJ11895 fis, clone HEMBA1007301, weakly similar to COLLAGEN ALPHA 1(III) CHAIN. [AK021957]	AK021957	Hs.494892	NM_032888
THC2718406	-4.5	1.4	THC2718406	THC2718406	Unknown	
THC2515611	-4.5	2.1	Q502Z6_HUMAN (Q502Z6) Ribosomal protein L30, complete [THC2515611]	THC2515611	Unknown	
NUDT21	-4.5	2.2	nudix (nucleoside diphosphate linked moiety X)-type motif 21 (NUDT21), mRNA [NM_007006]	NM_007006	Hs.528834	BX537360
HNRPA1	-4.5	2.9	heterogeneous nuclear ribonucleoprotein A1 (HNRPA1), transcript variant 1, mRNA [NM_002136]	NM_002136	Hs.699190	NM_031157
FLJ34515	-4.5	11.6	cDNA FLJ34515 fis, clone HLUNG2006614. [AK091834]	AK091834	Hs.448825	XM_001131899
THC2618570	-4.5	2.7	THC2618570	THC2618570	Unknown	
BX116997	-4.5	2.9	BX116997 Soares_placenta_8to9weeks_2NbHP8to9W cDNA clone IMAGp998G16558, mRNA sequence [BX116997]	BX116997	Hs.26297	BX116997
LOC643313	-4.5	2.3	cDNA clone IMAGE:4129277, partial cds. [BC006361]	BC006361	Hs.676949	BC006361
BC017507	-4.5	2.0	Homo sapiens, clone IMAGE:4850148, mRNA. [BC017507]	BC017507	Hs.661377	BC017507
LOC391560	-4.5	1.6	PREDICTED: similar to 60S ribosomal protein L32 (LOC391560), mRNA [XR_016842]	XR_016842	Hs.693395	XR_018524
CDKN1B	-4.5	1.5	cyclin-dependent kinase inhibitor 1B (p27, Kip1) (CDKN1B), mRNA [NM_004064]	NM_004064	Hs.238990	NM_004064
BCL2L11	-4.5	1.6	BCL2-like 11 (apoptosis facilitator) (BCL2L11), transcript variant 1, mRNA [NM_138621]	NM_138621	Hs.469658	NM_138621
AK096154	-4.5	2.5	cDNA FLJ38835 fis, clone MESAN2002424. [AK096154]	AK096154	Hs.594968	AK096154
SUPT16H	-4.5	1.7	suppressor of Ty 16 homolog (S. cerevisiae) (SUPT16H), mRNA [NM_007192]	NM_007192	Hs.213724	NM_007192
AK021693	-4.5	1.5	cDNA FLJ11631 fis, clone HEMBA1004267. [AK021693]	AK021693	Hs.699348	AK021693

TBL1XR1	-4.5	1.9	transducin (beta)-like 1X-linked receptor 1, mRNA (cDNA clone IMAGE:5527421), complete cds. [BC048298]	BC048298	Hs.699293	BX648935
ANKRD11	-4.5	1.9	ankyrin repeat domain 11 (ANKRD11), mRNA [NM_013275]	NM_013275	Hs.335003	AY373756
NFYA	-4.5	2.3	Nuclear transcription factor Y subunit alpha (Nuclear transcription factor Y subunit A) (NF-YA) (CAAT-box DNA-binding protein subunit A). [Source:Uniprot/SWISSPROT;Acc:P23511] [ENST00000341376]	ENST0000034137	Unknown	
X62691	-4.5	2.1	H.sapiens mRNA for ribosomal protein (homologuous to yeast S24). [X62691]	X62691	Unknown	
THSD1	-4.5	1.6	thrombospondin, type I, domain containing 1 (THSD1), transcript variant 1, mRNA [NM_018676]	NM_018676	Hs.325667	AK096289
ENST0000024	-4.5	2.1	ENST00000247761	ENST0000024776	Unknown	
WT1	-4.5	10.5	Wilms tumor 1 (WT1), transcript variant D, mRNA [NM_024426]	NM_024426	Hs.591980	BC046461
RP11-78J21.1	-4.5	2.9	heterogeneous nuclear ribonucleoprotein A1-like (LOC144983), transcript variant 1, mRNA [NM_001011724]	NM_001011724	Hs.447506	AK126454
PLG	-4.5	2.9	plasminogen (PLG), mRNA [NM_000301]	NM_000301	Hs.143436	CR749293
HISPPD1	-4.5	1.9	histidine acid phosphatase domain containing 1 (HISPPD1), mRNA [NM_015216]	NM_015216	Hs.212046	AB007893
PLCL2	-4.5	2.5	phospholipase C-like 2 (PLCL2), mRNA [NM_015184]	NM_015184	Hs.202010	AB029015
HISPPD1	-4.5	1.9	histidine acid phosphatase domain containing 1 (HISPPD1), mRNA [NM_015216]	NM_015216	Hs.212046	AB007893
RBP4	-4.5	2.7	retinol binding protein 4, plasma (RBP4), mRNA [NM_006744]	NM_006744	Hs.50223	BQ064708
FLJ10081	-4.5	1.5	hypothetical protein FLJ10081 (FLJ10081), mRNA [NM_017991]	NM_017991	Hs.516341	NM_017991
FRG1	-4.5	2.0	FSHD region gene 1 (FRG1), mRNA [NM_004477]	NM_004477	Hs.203772	AK057099
HGF	-4.5	45.2	Hepatocyte growth factor precursor (Scatter factor) (SF) (Hepatopoeitin A) [Contains: Hepatocyte growth factor alpha chain; Hepatocyte growth factor beta chain]. [Source:Uniprot/SWISSPROT;Acc:P14210] [ENST00000222390]	ENST0000022239	Unknown	
BC040991	-4.5	1.6	cDNA clone IMAGE:4817695. [BC040991]	BC040991	Hs.379018	U18422
RBM17	-4.5	2.0	RNA binding motif protein 17 (RBM17), mRNA [NM_032905]	NM_032905	Hs.498548	BX648288
RPL17	-4.5	1.5	ribosomal protein L17 (RPL17), transcript variant 1, mRNA [NM_000985]	NM_000985	Hs.374588	BF970890
C16orf30	-4.5	6.6	chromosome 16 open reading frame 30 (C16orf30), mRNA [NM_024600]	NM_024600	Hs.459652	AY676494
MGC70863	-4.5	1.5	similar to RPL23AP7 protein (MGC70863), transcript variant 1, mRNA [NM_203477]	NM_203477	Hs.406135	BC065556
TMEM16A	-4.5	4.0	transmembrane protein 16A (TMEM16A), mRNA [NM_018043]	NM_018043	Hs.503074	AY728143
PHF2	-4.5	1.4	PHD finger protein 2 (PHF2), mRNA [NM_005392]	NM_005392	Hs.211441	NM_005392
LOC285074	-4.5	1.8	hypothetical protein LOC285074 (LOC285074), mRNA [NM_001012626]	NM_001012626	Unknown	
AW378392	-4.5	1.9	RC3-HT0220-031299-012-e09 HT0220 cDNA, mRNA sequence [AW378392]	AW378392	Hs.649746	AW378392
CCDC52	-4.5	2.0	coiled-coil domain containing 52 (CCDC52), mRNA [NM_144718]	NM_144718	Hs.477144	NM_144718
THUMPD1	-4.5	2.0	THUMP domain containing 1 (THUMPD1), mRNA [NM_017736]	NM_017736	Hs.460232	NM_017736
TCF12	-4.5	3.5	transcription factor 12 (HTF4, helix-loop-helix transcription factors 4) (TCF12), transcript variant 4, mRNA [NM_207038]	NM_207038	Hs.511504	NM_207037
CART1	-4.5	4.9	cartilage paired-class homeoprotein 1 (CART1), mRNA [NM_006982]	NM_006982	Hs.41683	U31986
RPL17	-4.5	1.5	ribosomal protein L17 (RPL17), transcript variant 1, mRNA [NM_000985]	NM_000985	Hs.374588	BF970890
CDC20B	-4.5	2.4	cell division cycle 20 homolog B (S. cerevisiae) (CDC20B), mRNA [NM_152623]	NM_152623	Hs.669184	AK095246
CD93	-4.5	38.5	CD93 molecule (CD93), mRNA [NM_012072]	NM_012072	Hs.97199	NM_012072
LOC643373	-4.5	2.1	PREDICTED: similar to Beta-glucuronidase precursor (Beta-G1), transcript variant 2 (LOC643373), mRNA [XM_932770]	ENST0000032772	Unknown	
BM723547	-4.5	2.2	UI-E-EJ0-aiq-m-17-0-UI.r1 UI-E-EJ0 cDNA clone UI-E-EJ0-aiq-m-17-0-UI 5', mRNA sequence [BM723547]	BM723547	Hs.133261	BM723547
RPS24	-4.5	1.6	ribosomal protein S24 (RPS24), transcript variant 2, mRNA [NM_001026]	NM_001026	Hs.356794	BX647260
ENST0000031	-4.5	2.3	Small EDRK-rich factor 1a. [Source:Uniprot/SWISSPROT;Acc:O75920] [ENST00000317633]	ENST0000031763	Unknown	
PAX8	-4.5	2.0	paired box gene 8 (PAX8), transcript variant PAX8A, mRNA [NM_003466]	NM_003466	Hs.469728	NM_003466
CDKN1B	-4.5	1.5	cyclin-dependent kinase inhibitor 1B (p27, Kip1) (CDKN1B), mRNA [NM_004064]	NM_004064	Hs.238990	NM_004064
RPS29	-4.5	1.8	ribosomal protein S29 (RPS29), transcript variant 1, mRNA [NM_001032]	NM_001032	Hs.156367	BX161511
AF217970	-4.5	2.4	clone PP1195 unknown mRNA. [AF217970]	AF217970	Hs.674508	AF217970
A_24_P92424	-4.5	2.9	A_24_P924240	A_24_P924240	Unknown	
FAM44A	-4.5	1.8	family with sequence similarity 44, member A (FAM44A), mRNA [NM_148894]	NM_148894	Hs.444517	NM_148894
RPL32	-4.5	1.6	ribosomal protein L32 (RPL32), transcript variant 3, mRNA [NM_001007074]	NM_001007074	Hs.265174	CR596982
AK000872	-4.5	3.3	cDNA FLJ10010 fis, clone HEMBA1000302. [AK000872]	AK000872	Hs.58690	AK128633
HISPPD1	-4.5	1.9	histidine acid phosphatase domain containing 1 (HISPPD1), mRNA [NM_015216]	NM_015216	Hs.212046	AB007893
AFP	-4.5	5.0	alpha-fetoprotein (AFP), mRNA [NM_001134]	NM_001134	Hs.518808	BC027881
PURG	-4.5	3.3	purine-rich element binding protein G (PURG), transcript variant B, mRNA [NM_001015508]	NM_001015508	Hs.373778	AF195513
LOC145853	-4.5	1.5	AGENCOURT_10399027 NIH_MGC_82 cDNA clone IMAGE:6614823 5', mRNA sequence [BU568037]	BU568037	Hs.662358	BU568037
ANP32B	-4.5	1.7	acidic (leucine-rich) nuclear phosphoprotein 32 family, member B (ANP32B), mRNA [NM_006401]	NM_006401	Hs.494604	BF683719
AK124396	-4.4	6.3	cDNA FLJ42405 fis, clone ASTRO3000474. [AK124396]	AK124396	Hs.49265	AK124396

MRPS31	-4.4	2.1	mitochondrial ribosomal protein S31 (MRPS31), nuclear gene encoding mitochondrial protein, mRNA [NM_005830]	NM_005830	Hs.154655	BC022045
HMGN1	-4.4	1.8	cDNA FLJ31471 fis, clone NT2NE2001435. [AK056033]	AK056033	Hs.356285	AB209245
TAGAP	-4.4	4.0	T-cell activation GTPase activating protein (TAGAP), transcript variant 2, mRNA [NM_054114]	NM_054114	Hs.529984	NM_054114
EFNA5	-4.4	1.6	Ephrin-A5 precursor (EPH-related receptor tyrosine kinase ligand 7) (LERK-7) (AL-1). [Source:Uniprot/SWISSPROT;Acc:P52803] [ENST00000379748]	ENST0000037974	Unknown	
RSPO2	-4.4	2.4	R-spondin 2 homolog (Xenopus laevis) (RSPO2), mRNA [NM_178565]	NM_178565	Hs.444834	AK123023
KIAA0240	-4.4	2.3	KIAA0240 (KIAA0240), mRNA [NM_015349]	NM_015349	Hs.658033	NM_015349
AW582601	-4.4	4.1	AW582601 RC4-ST0279-080100-012-c03 ST0279 cDNA, mRNA sequence [AW582601]	AW582601	Hs.686970	AW582601
ZNF416	-4.4	1.3	zinc finger protein 416 (ZNF416), mRNA [NM_017879]	NM_017879	Hs.247711	NM_017879
NP1252191	-4.4	2.2	GB|AACC02000041.1|EAL24419.1 similar to 60S ribosomal protein L32 [Homo sapiens] [NP1252191]	NP1252191	Unknown	
AF075028	-4.4	2.9	full length insert cDNA YI38B04. [AF075028]	AF075028	Hs.602484	DR760892
CSTF3	-4.4	1.7	cleavage stimulation factor, 3' pre-RNA, subunit 3, 77kDa (CSTF3), transcript variant 1, mRNA [NM_001326]	NM_001326	Hs.44402	NM_001326
DLG5	-4.4	1.6	discs, large homolog 5 (Drosophila) (DLG5), mRNA [NM_004747]	NM_004747	Hs.652690	BC146794
ALMS1	-4.4	2.8	Alstrom syndrome 1 (ALMS1), mRNA [NM_015120]	NM_015120	Hs.184720	AJ417593
LOC646119	-4.4	1.7	PREDICTED: similar to ribosomal protein S8 (LOC646119), mRNA [XM_929084]	XM_929084	Unknown	
BE531123	-4.4	1.7	BE531123 601278493F1 NIH_MGC_39 cDNA clone IMAGE:3610487 5', mRNA sequence [BE531123]	BE531123	Hs.513039	NM_015477
BTBD3	-4.4	1.9	BTB (POZ) domain containing 3 (BTBD3), transcript variant 1, mRNA [NM_014962]	NM_014962	Hs.244590	NM_014962
SEC31A	-4.4	1.9	cDNA FLJ46167 fis, clone TESTI4003179. [AK128047]	AK128047	Hs.370024	AK128047
FLJ45187	-4.4	3.1	hypothetical protein LOC387640 (FLJ45187), mRNA [NM_207371]	NM_207371	Hs.350848	NM_207371
AF086017	-4.4	2.3	full length insert cDNA clone YW19E12. [AF086017]	AF086017	Hs.567828	BX538174
AK128048	-4.4	1.6	cDNA FLJ46168 fis, clone TESTI4003279. [AK128048]	AK128048	Hs.635587	AK128048
KNTC2	-4.4	1.5	kinetochore associated 2 (KNTC2), mRNA [NM_006101]	NM_006101	Hs.414407	NM_006101
FLJ31306	-4.4	2.1	full-length cDNA clone CS0DF021YK18 of Fetal brain of (human). [CR606347]	CR606347	Hs.531089	BC034618
TEK	-4.4	4.2	TEK tyrosine kinase, endothelial (venous malformations, multiple cutaneous and mucosal) (TEK), mRNA [NM_000459]	NM_000459	Hs.89640	NM_000459
SH3GL3	-4.4	2.0	SH3-domain GRB2-like 3 (SH3GL3), mRNA [NM_003027]	NM_003027	Hs.270055	NM_003027
THC2685686	-4.4	2.6	ALU7_HUMAN (P39194) Alu subfamily SQ sequence contamination warning entry, partial (11%) [THC2685686]	THC2685686	Unknown	
LOC442237	-4.4	2.0	PREDICTED: similar to 40S ribosomal protein S7 (S8) (LOC442237), mRNA [XR_018963]	XR_018963	Hs.648064	XR_018963
THC2656802	-4.4	2.4	THC2656802	THC2656802	Unknown	
RPL21	-4.4	1.7	ribosomal protein L21 (RPL21), mRNA [NM_000982]	NM_000982	Hs.381123	CR602527
ANKRD12	-4.4	1.7	ankyrin repeat domain 12 (ANKRD12), mRNA [NM_015208]	NM_015208	Hs.464585	NM_015208
NO145	-4.4	5.4	145 kDa nucleolar protein (NO145), mRNA [NM_001040274]	NM_001040274	Hs.95600	NM_001040274
LARP7	-4.4	1.8	La ribonucleoprotein domain family, member 7 (LARP7), transcript variant 1, mRNA [NM_016648]	NM_016648	Hs.696461	BC066945
BX113895	-4.4	2.8	BX113895 Soares_pregnant_uterus_NbHPU cDNA clone IMAGp998P161113, mRNA sequence [BX113895]	BX113895	Hs.61481	BX113895
A_32_P14193	-4.4	2.2	A_32_P141938	A_32_P141938	Unknown	
RTDR1	-4.4	1.6	rhabdoid tumor deletion region gene 1 (RTDR1), mRNA [NM_014433]	NM_014433	Hs.526920	NM_014433
HMGB1	-4.4	2.3	high-mobility group box 1 (HMGB1), mRNA [NM_002128]	NM_002128	Hs.434102	AK122825
ZC3H13	-4.4	2.1	zinc finger CCCH-type containing 13 (ZC3H13), mRNA [NM_015070]	NM_015070	Hs.136102	AY283618
IFT74	-4.4	1.8	intraflagellar transport 74 homolog (Chlamydomonas) (IFT74), mRNA [NM_025103]	NM_025103	Hs.145402	NM_001099222
FLJ36492	-4.4	2.3	hypothetical protein FLJ36492 (FLJ36492), mRNA [NM_182568]	NM_182568	Hs.419859	EF553518
ROCK1	-4.4	1.8	Rho-associated, coiled-coil containing protein kinase 1 (ROCK1), mRNA [NM_005406]	NM_005406	Hs.696120	NM_005406
LOC130865	-4.4	1.6	PREDICTED: similar to 60S ribosomal protein L26 (Silica-induced gene 20 protein) (SIG-20) (LOC130865), mRNA [XR_019454]	XR_019454	Hs.646811	XR_019454
WIF1	-4.4	3.8	WNT inhibitory factor 1 (WIF1), mRNA [NM_007191]	NM_007191	Hs.284122	AY358344
MTMR4	-4.4	1.7	myotubularin related protein 4 (MTMR4), mRNA [NM_004687]	NM_004687	Hs.514373	NM_004687
CCDC4	-4.4	2.7	coiled-coil domain containing 4 (CCDC4), mRNA [NM_207406]	NM_207406	Hs.120591	NM_207406
C5orf21	-4.4	1.5	chromosome 5 open reading frame 21 (C5orf21), mRNA [NM_032042]	NM_032042	Hs.655651	NM_032042
A_32_P95502	-4.4	2.0	A_32_P95502	A_32_P95502	Unknown	
A_24_P20447	-4.4	2.1	A_24_P204474	A_24_P204474	Unknown	
LOC643669	-4.4	2.3	PREDICTED: hypothetical LOC643669, transcript variant 1 (LOC643669), mRNA [XM_933621]	XM_933621	Hs.355357	XM_933621
IRX1	-4.4	1.9	iroquois homeobox protein 1 (IRX1), mRNA [NM_024337]	NM_024337	Hs.424156	AY335938
EVL	-4.4	1.5	Enah/Vasp-like (EVL), mRNA [NM_016337]	NM_016337	Hs.125867	AL133642
DDX28	-4.4	1.6	DEAD (Asp-Glu-Ala-Asp) box polypeptide 28 (DDX28), nuclear gene encoding mitochondrial protein, mRNA [NM_018380]	NM_018380	Hs.458313	NM_018380

SYT15	-4.4	2.0	cDNA FLJ29001 fis, clone CBL08678. [AK131036]	AK131036	Hs.696346	NM_031912
THC2652466	-4.4	4.0	Q9BHM3_PARTE (Q9BHM3) Cyclophilin-RNA interacting protein, partial (4%) [THC2652466]	THC2652466	Unknown	
RDH14	-4.4	1.4	retinol dehydrogenase 14 (all-trans/9-cis/11-cis) (RDH14), mRNA [NM_020905]	NM_020905	Hs.288880	AK023625
ZNF658B	-4.4	2.0	zinc finger protein 658B (ZNF658B), mRNA [NM_001032297]	NM_001032297	Hs.534812	NM_001032297
AK054852	-4.4	3.0	cDNA FLJ30290 fis, clone BRACE2002884. [AK054852]	AK054852	Hs.486508	AL833602
SLC26A10	-4.4	2.6	cDNA FLJ16735 fis, clone BRACE2005457, weakly similar to PENDRIN. [AK122981]	AK122981	Unknown	
AK124192	-4.4	1.7	cDNA FLJ42198 fis, clone THYMU2034338. [AK124192]	AK124192	Hs.584833	AK124192
LOC400960	-4.4	1.8	cDNA FLJ32260 fis, clone PROST1000334. [AK056822]	AK056822	Hs.657791	AK056822
NUDT11	-4.4	2.9	nudix (nucleoside diphosphate linked moiety X)-type motif 11 (NUDT11), mRNA [NM_018159]	NM_018159	Hs.200016	NM_018159
KIAA1217	-4.4	2.1	KIAA1217 (KIAA1217), mRNA [NM_019590]	NM_019590	Hs.445885	NM_019590
RP11-50D16.3	-4.4	1.5	similar to RIKEN cDNA 8030451K01 (LOC387921), transcript variant 2, mRNA [NM_001017370]	NM_001017370	Hs.507783	NM_001012754
FAM124B	-4.4	15.6	family with sequence similarity 124B (FAM124B), mRNA [NM_024785]	NM_024785	Hs.147585	NM_024785
RP11-78J21.1	-4.4	2.4	heterogeneous nuclear ribonucleoprotein A1-like (LOC144983), transcript variant 1, mRNA [NM_001011724]	NM_001011724	Hs.447506	AK126454
A_24_P41007(	-4.4	1.7	A_24_P410070	A_24_P410070	Unknown	
C18orf34	-4.4	3.8	cDNA FLJ26445 fis, clone KDN02608. [AK129955]	AK129955	Hs.115461	AK126038
WAC	-4.4	1.6	WW domain-containing adapter protein with coiled-coil. [Source:Uniprot/SWISSPROT;Acc:Q9BTA9] [ENST00000375664]	ENST0000037566	Unknown	
SOX7	-4.4	4.1	SRY (sex determining region Y)-box 7 (SOX7), mRNA [NM_031439]	NM_031439	Hs.490991	NM_031439
A_23_P4462	-4.4	2.0	A_23_P4462	A_23_P4462	Unknown	
MAPK8	-4.4	2.1	Mitogen-activated protein kinase 8 (EC 2.7.11.24) (Stress-activated protein kinase JNK1) (c-Jun N-terminal kinase 1) (JNK-46). [Source:Uniprot/SWISSPROT;Acc:P45983] [ENST00000374189]	ENST0000037418	Unknown	
CR603215	-4.4	2.2	full-length cDNA clone CS0DI023YC17 of Placenta Cot 25-normalized of (human). [CR603215]	CR603215	Hs.463678	CR599799
FSTL5	-4.4	2.9	follistatin-like 5 (FSTL5), mRNA [NM_020116]	NM_020116	Hs.591707	NM_020116
OPRK1	-4.4	2.6	opioid receptor, kappa 1 (OPRK1), mRNA [NM_000912]	NM_000912	Hs.106795	NM_000912
NUDT16	-4.4	1.4	nudix (nucleoside diphosphate linked moiety X)-type motif 16 (NUDT16), mRNA [NM_152395]	NM_152395	Hs.591313	AK055827
AK057088	-4.4	1.3	cDNA FLJ32526 fis, clone SMINT2000073. [AK057088]	AK057088	Hs.592692	AF034176
KNTC1	-4.4	2.6	kinetochore associated 1 (KNTC1), mRNA [NM_014708]	NM_014708	Hs.300559	BC150278
BC047110	-4.4	2.8	cDNA clone IMAGE:5312754. [BC047110]	BC047110	Hs.633204	AL117598
RPL34	-4.4	1.7	ribosomal protein L34 (RPL34), transcript variant 2, mRNA [NM_033625]	NM_033625	Hs.438227	BG112770
GUSBP1	-4.4	2.3	glucuronidase, beta pseudogene 1 (GUSBP1), mRNA [NM_207331]	NM_207331	Unknown	
BC041459	-4.4	8.4	Homo sapiens, clone IMAGE:5207242, mRNA. [BC041459]	BC041459	Hs.102408	BC041459
TIMM9	-4.4	1.8	translocase of inner mitochondrial membrane 9 homolog (yeast) (TIMM9), mRNA [NM_012460]	NM_012460	Hs.440525	BG719789
SPG20	-4.4	1.6	spastic paraplegia 20, spartin (Troyer syndrome) (SPG20), mRNA [NM_015087]	NM_015087	Hs.440414	NM_015087
THC2746310	-4.4	2.6	ALU8_HUMAN (P39195) Alu subfamily SX sequence contamination warning entry, partial (7%) [THC2746310]	THC2746310	Unknown	
THC2691289	-4.4	5.8	THC2691289	THC2691289	Unknown	
LOC440311	-4.4	2.0	PREDICTED: similar to Glioma tumor suppressor candidate region gene 2 protein (p60) (LOC440311), mRNA [XR_016779]	XR_016779	Hs.547436	XR_016779
ANTXR1	-4.4	1.6	anthrax toxin receptor 1 (ANTXR1), transcript variant 1, mRNA [NM_032208]	NM_032208	Hs.165859	AF279145
A_24_P20448	-4.4	2.4	A_24_P204484	A_24_P204484	Unknown	
C12orf47	-4.4	1.7	full-length cDNA clone CS0DJ012YG18 of T cells (Jurkat cell line) Cot 10-normalized of (human). [CR620831]	CR620831	Hs.333120	AK096935
CEP57	-4.4	1.9	centrosomal protein 57kDa (CEP57), mRNA [NM_014679]	NM_014679	Hs.101014	NM_014679
CDH7	-4.4	2.0	cadherin 7, type 2 (CDH7), transcript variant b, mRNA [NM_004361]	NM_004361	Hs.657522	BC036786
NET1	-4.4	2.6	neuroepithelial cell transforming gene 1 (NET1), transcript variant 1, mRNA [NM_001047160]	NM_001047160	Hs.25155	NM_001047160
A_24_P49311	-4.4	2.8	A_24_P493116	A_24_P493116	Unknown	
A_32_P18630	-4.4	2.3	A_32_P18630	A_32_P18630	Unknown	
SOX8	-4.4	1.8	SRY (sex determining region Y)-box 8 (SOX8), mRNA [NM_014587]	NM_014587	Hs.243678	NM_014587
SLC39A5	-4.4	1.7	solute carrier family 39 (metal ion transporter), member 5 (SLC39A5), mRNA [NM_173596]	NM_173596	Hs.591018	AK172768
BBS1	-4.4	1.5	Bardet-Biedl syndrome 1 (BBS1), mRNA [NM_024649]	NM_024649	Hs.502915	AK095638
ZNF22	-4.4	2.4	zinc finger protein 22 (KOX 15) (ZNF22), mRNA [NM_006963]	NM_006963	Hs.462693	NM_006963
THC2654231	-4.4	1.4	ALU5_HUMAN (P39192) Alu subfamily SC sequence contamination warning entry, partial (8%) [THC2654231]	THC2654231	Unknown	
ENST0000034	-4.4	2.1	PHD finger protein 10 (XAP135). [Source:Uniprot/SWISSPROT;Acc:Q8WUB8] [ENST00000341591]	ENST0000034159	Unknown	
THC2706230	-4.4	2.2	THC2706230	THC2706230	Unknown	

ZNF302	-4.4	1.8	zinc finger protein 302 (ZNF302), transcript variant 1, mRNA [NM_018443]	NM_018443	Hs.436350	AK122855
PRTFDC1	-4.3	1.7	phosphoribosyl transferase domain containing 1 (PRTFDC1), mRNA [NM_020200]	NM_020200	Hs.405619	NM_020200
NPAT	-4.3	1.9	nuclear protein, ataxia-telangiectasia locus (NPAT), mRNA [NM_002519]	NM_002519	Unknown	
RPS3A	-4.3	1.7	ribosomal protein S3A (RPS3A), mRNA [NM_001006]	NM_001006	Hs.356572	BI087817
ENST0000036	-4.3	1.8	ENST00000361800	ENST0000036180	Unknown	
KIAA1641	-4.3	3.8	KIAA1641 (KIAA1641), mRNA [NM_020970]	NM_020970	Unknown	
IQGAP2	-4.3	3.5	IQ motif containing GTPase activating protein 2 (IQGAP2), mRNA [NM_006633]	NM_006633	Hs.291030	NM_006633
HISPPD1	-4.3	1.9	histidine acid phosphatase domain containing 1 (HISPPD1), mRNA [NM_015216]	NM_015216	Hs.212046	AB007893
DKKL1	-4.3	2.3	dickkopf-like 1 (soggy) (DKKL1), mRNA [NM_014419]	NM_014419	Hs.515855	AY358922
RPS13	-4.3	1.7	ribosomal protein S13 (RPS13), mRNA [NM_001017]	NM_001017	Hs.446588	BE621497
PKNOX1	-4.3	2.0	PBX/knotted 1 homeobox 1 (PKNOX1), mRNA [NM_004571]	NM_004571	Hs.431043	NM_004571
CDKN1B	-4.3	1.5	cyclin-dependent kinase inhibitor 1B (p27, Kip1) (CDKN1B), mRNA [NM_004064]	NM_004064	Hs.238990	NM_004064
PNPLA4	-4.3	1.5	patatin-like phospholipase domain containing 4 (PNPLA4), mRNA [NM_004650]	NM_004650	Hs.264	NM_004650
BC006438	-4.3	1.5	cDNA clone MGC:13162 IMAGE:3010103, complete cds. [BC006438]	BC006438	Hs.649107	BM545915
ZNF195	-4.3	1.6	zinc finger protein 195 (ZNF195), mRNA [NM_007152]	NM_007152	Hs.386294	AL833722
C3orf33	-4.3	1.8	chromosome 3 open reading frame 33 (C3orf33), mRNA [NM_173657]	NM_173657	Hs.350846	NM_173657
YLPM1	-4.3	1.5	YLP motif containing 1, mRNA (cDNA clone IMAGE:3835908), complete cds. [BC023570]	BC023570	Hs.531111	NM_019589
LOC285398	-4.3	1.5	cDNA FLJ25945 fis, clone JTH12731. [AK098811]	AK098811	Hs.681813	AK098811
KDELC1	-4.3	1.5	KDEL (Lys-Asp-Glu-Leu) containing 1 (KDELC1), mRNA [NM_024089]	NM_024089	Hs.408629	BC051860
LOC643790	-4.3	1.9	similar to Nonhistone chromosomal protein HMG-14 (High-mobility group nucleosome-binding domain-containing protein 1) (LOC644534), mRNA [Source:RefSeq_dna;Acc:XR_016359] [ENST00000378969]	ENST0000037896	Unknown	
HMGN1	-4.3	1.9	high-mobility group nucleosome binding domain 1 (HMGN1), mRNA [NM_004965]	NM_004965	Hs.356285	AB209245
FLJ37644	-4.3	1.8	cDNA FLJ37644 fis, clone BRHIP2000239. [AK094963]	AK094963	Hs.657374	AK094963
THC2663329	-4.3	2.2	THC2663329	THC2663329	Unknown	
A_32_P22404	-4.3	1.7	A_32_P224040	A_32_P224040	Unknown	
PLVAP	-4.3	4.5	plasmalemma vesicle associated protein (PLVAP), mRNA [NM_031310]	NM_031310	Hs.107125	BC056414
ZNF175	-4.3	1.7	zinc finger protein 175 (ZNF175), mRNA [NM_007147]	NM_007147	Hs.119014	BC052997
FLJ22795	-4.3	1.5	colon cancer-associated antigen AgSK1-2HT-ECS mRNA, complete cds. [AF316855]	AF316855	Hs.498322	AF316855
CYYR1	-4.3	2.6	cysteine/tyrosine-rich 1 (CYYR1), mRNA [NM_052954]	NM_052954	Hs.37445	NM_052954
BX393727	-4.3	1.8	BX393727 NEUROBLASTOMA COT 25-NORMALIZED cDNA clone CS0DC001YP02 5-PRIME, mRNA sequence [BX393727]	BX393727	Hs.440088	BX393727
KIAA0841	-4.3	2.5	mRNA for KIAA0841 protein, partial cds. [AB020648]	AB020648	Hs.7426	NM_015302
OIT3	-4.3	8.5	oncoprotein induced transcript 3 (OIT3), mRNA [NM_152635]	NM_152635	Hs.8366	AK096435
THC2484716	-4.3	4.9	AF547984 ORF_4 {Adoxophyes orana granulovirus} (exp=-1; wgp=0; cg=1), partial (8%) [THC2484716]	THC2484716	Unknown	
FOXP2	-4.3	6.5	forkhead box P2 (FOXP2), transcript variant 4, mRNA [NM_148900]	NM_148900	Unknown	
KCTD6	-4.3	1.8	potassium channel tetramerisation domain containing 6 (KCTD6), mRNA [NM_153331]	NM_153331	Hs.13982	AK027572
EIF4EBP2	-4.3	1.4	eukaryotic translation initiation factor 4E binding protein 2 (EIF4EBP2), mRNA [NM_004096]	NM_004096	Hs.695953	BC050633
GTF3C3	-4.3	1.9	General transcription factor 3C polypeptide 3 (Transcription factor IIIC-subunit gamma) (TF3C-gamma) (TFIIIC 102 kDa subunit) (TFIIIC102). [Source:Uniprot/SWISSPROT;Acc:Q9Y5Q9] [ENST00000263956]	ENST0000026395	Unknown	
SMOC2	-4.3	2.4	SPARC related modular calcium binding 2 (SMOC2), mRNA [NM_022138]	NM_022138	Hs.487200	BC028420
BC007784	-4.3	2.3	cDNA clone IMAGE:4304686, partial cds. [BC007784]	BC007784	Hs.397001	BC007784
DQ786249	-4.3	3.1	clone HLS_IMAGE_234376 mRNA sequence. [DQ786249]	DQ786249	Hs.546584	XR_015700
SLC38A1	-4.3	1.8	solute carrier family 38, member 1 (SLC38A1), transcript variant 1, mRNA [NM_030674]	NM_030674	Hs.699239	NM_030674
THC2644803	-4.3	4.1	THC2644803	THC2644803	Unknown	
BC062739	-4.3	1.8	cDNA clone IMAGE:4270911, partial cds. [BC062739]	BC062739	Hs.468729	BC062739
NSBP1	-4.3	2.2	nucleosomal binding protein 1 (NSBP1), mRNA [NM_030763]	NM_030763	Hs.282204	AF250329
KNTC1	-4.3	2.4	kinetochore associated 1 (KNTC1), mRNA [NM_014708]	NM_014708	Hs.300559	BC150278
PRPF4B	-4.3	1.8	PRP4 pre-mRNA processing factor 4 homolog B (yeast) (PRPF4B), mRNA [NM_003913]	NM_003913	Hs.159014	NM_003913
DTL	-4.3	1.8	denticleless homolog (Drosophila) (DTL), mRNA [NM_016448]	NM_016448	Hs.656473	NM_016448
RPS6KA5	-4.3	2.7	ribosomal protein S6 kinase, 90kDa, polypeptide 5 (RPS6KA5), transcript variant 2, mRNA [NM_182398]	NM_182398	Hs.510225	AB209667
THC2642212	-4.3	3.2	THC2642212	THC2642212	Unknown	
KDELC1	-4.3	1.5	KDEL (Lys-Asp-Glu-Leu) containing 1 (KDELC1), mRNA [NM_024089]	NM_024089	Hs.408629	BC051860
LARP7	-4.3	1.6	La ribonucleoprotein domain family, member 7 (LARP7), transcript variant 1, mRNA [NM_016648]	NM_016648	Hs.696461	BC066945

CA4	-4.3	4.0	carbonic anhydrase IV (CA4), mRNA [NM_000717]	NM_000717	Hs.89485	BC057792
SLC38A2	-4.3	1.5	solute carrier family 38, member 2 (SLC38A2), mRNA [NM_018976]	NM_018976	Hs.221847	NM_018976
ZNF559	-4.3	3.3	zinc finger protein 559 (ZNF559), mRNA [NM_032497]	NM_032497	Hs.655107	AK092348
RPL21	-4.3	1.9	ribosomal protein L21 (RPL21), mRNA [NM_000982]	NM_000982	Hs.381123	CR602527
A_24_P41530	-4.3	2.6	A_24_P41530	A_24_P41530	Unknown	
THC2649313	-4.3	1.9	THC2649313	THC2649313	Unknown	
RP11-151A6.2	-4.3	1.8	hypothetical protein BC004360, mRNA (cDNA clone IMAGE:2822295), complete cds. [BC001077]	BC001077	Hs.350868	AK095850
HGF	-4.3	5.2	hepatocyte growth factor (hepapoietin A; scatter factor) (HGF), transcript variant 2, mRNA [NM_001010931]	NM_001010931	Hs.396530	NM_000601
GIMAP8	-4.3	8.8	GTPase, IMAP family member 8 (GIMAP8), mRNA [NM_175571]	NM_175571	Hs.647121	NM_175571
TMEM169	-4.3	1.9	transmembrane protein 169 (TMEM169), mRNA [NM_138390]	NM_138390	Hs.334916	NM_138390
THC2690347	-4.3	3.1	O61886_CAEEL (O61886) Seven tm receptor protein 224, partial (5%) [THC2690347]	THC2690347	Unknown	
HNRPUL2	-4.3	1.4	PREDICTED: heterogeneous nuclear ribonucleoprotein U-like 2 (HNRPUL2), mRNA [XM_495877]	XM_495877	Unknown	
THC2726661	-4.3	1.7	T52140 yb29h11.r1 Stratagene fetal spleen (#937205) cDNA clone IMAGE:72645 5', mRNA sequence [T52140]	THC2726661	Unknown	
NEO1	-4.3	1.7	neogenin homolog 1 (chicken) (NEO1), mRNA [NM_002499]	NM_002499	Hs.699228	AB209412
C6orf111	-4.3	1.9	chromosome 6 open reading frame 111 (C6orf111), mRNA [NM_032870]	NM_032870	Hs.520287	NM_032870
PRH1	-4.3	1.4	proline-rich protein HaeIII subfamily 1, mRNA (cDNA clone MGC:74956 IMAGE:5553460), complete cds. [BC064553]	BC064553	Unknown	
KLF11	-4.3	2.1	Kruppel-like factor 11 (KLF11), mRNA [NM_003597]	NM_003597	Hs.12229	BC063286
CUL1	-4.3	1.5	cullin 1 (CUL1), mRNA [NM_003592]	NM_003592	Hs.146806	NM_003592
LOC339745	-4.3	1.5	hypothetical protein LOC339745 (LOC339745), mRNA [NM_001001664]	NM_001001664	Hs.333297	BC071613
ATP2B1	-4.3	1.8	ATPase, Ca++ transporting, plasma membrane 1 (ATP2B1), transcript variant 2, mRNA [NM_001682]	NM_001682	Hs.506276	NM_001001323
SLC26A10	-4.3	2.2	solute carrier family 26, member 10 (SLC26A10), mRNA [NM_133489]	NM_133489	Hs.159481	NM_133489
RPS6KA5	-4.3	2.3	ribosomal protein S6 kinase, 90kDa, polypeptide 5 (RPS6KA5), transcript variant 1, mRNA [NM_004755]	NM_004755	Hs.510225	AB209667
CD72	-4.3	2.5	CD72 molecule (CD72), mRNA [NM_001782]	NM_001782	Hs.116481	AB209587
THC2533546	-4.3	1.6	XM_750378 rRNA assembly protein Mis3 {Aspergillus fumigatus Af293} (exp=-1; wgp=0; cg=0), partial (6%) [THC2533546]	THC2533546	Unknown	
NBN	-4.3	2.2	nibrin (NBN), transcript variant 2, mRNA [NM_001024688]	NM_001024688	Hs.492208	BX640816
ZNF508	-4.3	1.5	zinc finger protein 508 (ZNF508), mRNA [NM_014913]	NM_014913	Hs.131915	BC071589
THC2683470	-4.3	3.2	THC2683470	THC2683470	Unknown	
A_24_P33385	-4.3	2.2	A_24_P33385	A_24_P33385	Unknown	
ENST0000036	-4.3	2.2	cancer susceptibility candidate 2, transcript variant 1 (CASC2), misc RNA [Source:RefSeq_dna;Acc:XR_017968] [ENST00000360758]	ENST0000036075	Unknown	
CENPQ	-4.3	2.3	centromere protein Q (CENPQ), mRNA [NM_018132]	NM_018132	Hs.88663	NM_018132
C9orf93	-4.3	2.7	chromosome 9 open reading frame 93 (C9orf93), mRNA [NM_173550]	NM_173550	Hs.17267	CR936775
HNRPDL	-4.3	2.8	heterogeneous nuclear ribonucleoprotein D-like (HNRPDL), transcript variant 2, mRNA [NM_031372]	NM_031372	Hs.527105	NM_031372
ZDHHC11	-4.3	1.6	zinc finger, DHHC-type containing 11 (ZDHHC11), mRNA [NM_024786]	NM_024786	Hs.659832	AK024233
FLJ12716	-4.3	1.6	FLJ12716 protein (FLJ12716), transcript variant 1, mRNA [NM_021942]	NM_021942	Hs.443240	BX647127
TTC23	-4.3	2.1	tetratricopeptide repeat domain 23 (TTC23), transcript variant 1, mRNA [NM_001040655]	NM_001040655	Hs.513195	AF411456
GLI3	-4.3	2.0	GLI-Kruppel family member GLI3 (Greig cephalopolysyndactyly syndrome) (GLI3), mRNA [NM_000168]	NM_000168	Hs.21509	NM_000168
THC2682884	-4.3	2.1	THC2682884	THC2682884	Unknown	
PYGM	-4.3	2.8	Glycogen phosphorylase, muscle form (EC 2.4.1.1) (Myophosphorylase). [Source:Uniprot/SWISSPROT;Acc:P11217] [ENST00000377444]	ENST0000037744	Unknown	
D55639	-4.3	3.5	Human monocyte PABL (pseudoautosomal boundary-like sequence) mRNA, clone Mo1. [D55639]	D55639	Hs.470126	CR609484
BC067244	-4.3	1.3	cDNA clone IMAGE:4807381, partial cds. [BC067244]	BC067244	Hs.153412	BC030623
BM819787	-4.3	3.8	BM819787 K-EST0087976 S16N667673 cDNA clone S16N667673-2-E05 5', mRNA sequence [BM819787]	BM819787	Hs.648544	DA395839
SLC44A1	-4.3	2.6	solute carrier family 44, member 1 (SLC44A1), mRNA [NM_080546]	NM_080546	Hs.573495	NM_080546
AW977527	-4.3	9.9	EST389636 MAGE resequences, MAGO cDNA, mRNA sequence [AW977527]	AW977527	Hs.611098	AW977527
SPG20	-4.3	1.4	spastic paraplegia 20, spartin (Troyer syndrome) (SPG20), mRNA [NM_015087]	NM_015087	Hs.440414	NM_015087
HSP90AB1	-4.3	1.6	heat shock protein 90kDa alpha (cytosolic), class B member 1 (HSP90AB1), mRNA [NM_007355]	NM_007355	Hs.509736	AY359878
THC2685646	-4.3	2.5	THC2685646	THC2685646	Unknown	
NSBP1	-4.3	2.2	nucleosomal binding protein 1 (NSBP1), mRNA [NM_030763]	NM_030763	Hs.282204	AF250329
LEPROTL1	-4.3	2.1	leptin receptor overlapping transcript-like 1 (LEPROTL1), mRNA [NM_015344]	NM_015344	Hs.146585	AF063605
KNTC1	-4.3	2.5	kinetochore associated 1 (KNTC1), mRNA [NM_014708]	NM_014708	Hs.300559	BC150278

LOC392522	-4.3	1.6	PREDICTED: similar to ribosomal protein L18a (LOC392522), mRNA [XR_018292]	XR_018292	Hs.648345	XR_018292
CR596712	-4.3	3.0	full-length cDNA clone CS0DI028YF10 of Placenta Cot 25-normalized of (human). [CR596712]	CR596712	Hs.130652	XM_001131009
KIF27	-4.3	1.8	kinesin family member 27 (KIF27), mRNA [NM_017576]	NM_017576	Hs.697514	AY237536
A_24_P30308	-4.3	1.7	A_24_P303080	A_24_P303080	Unknown	
PLLP	-4.3	2.1	transmembrane 4 superfamily member 11 (plasmolipin) (TM4SF11), mRNA [NM_015993]	NM_015993	Hs.632215	NM_015993
MED4	-4.3	1.6	mediator of RNA polymerase II transcription, subunit 4 homolog (S. cerevisiae) (MED4), mRNA [NM_014166]	NM_014166	Hs.181112	AK001934
LOC390183	-4.3	2.1	PREDICTED: similar to 40S ribosomal protein S4, X isoform (LOC390183), mRNA [XR_018242]	XR_018242	Hs.647251	XR_018242
ARID2	-4.3	2.0	cDNA FLJ30619 fis, clone CTONG2001533. [AK055181]	AK055181	Hs.696080	NM_152641
BC064969	-4.3	3.1	cDNA clone IMAGE:6149643, partial cds. [BC064969]	BC064969	Hs.458747	AK127498
ICK	-4.3	1.6	intestinal cell (MAK-like) kinase (ICK), transcript variant 2, mRNA [NM_016513]	NM_016513	Hs.417022	NM_016513
RPS3A	-4.3	1.6	ribosomal protein S3A (RPS3A), mRNA [NM_001006]	NM_001006	Hs.356572	BI087817
PPP2R5C	-4.3	1.4	protein phosphatase 2, regulatory subunit B (B56), gamma isoform (PPP2R5C), transcript variant 1, mRNA [NM_002719]	NM_002719	Hs.368264	NM_002719
ADAMTS1	-4.3	2.3	ADAM metallopeptidase with thrombospondin type 1 motif, 1 (ADAMTS1), mRNA [NM_006988]	NM_006988	Hs.643357	NM_006988
LOC341333	-4.3	3.2	PREDICTED: similar to Heterogeneous nuclear ribonucleoprotein A1 (Helix-destabilizing protein) (Single-strand RNA-binding protein) (hnRNP core protein A1) (LOC341333), mRNA [XR_018931]	XR_018931	Hs.439832	XR_018931
CCDC28A	-4.3	1.7	coiled-coil domain containing 28A (CCDC28A), mRNA [NM_015439]	NM_015439	Hs.412019	AY167571
A_24_P91137	-4.3	2.8	A_24_P911378	A_24_P911378	Unknown	
C20orf133	-4.3	1.8	OTTHUMP00000030317 (Chromosome 20 open reading frame 133). [Source:Uniprot/SPTREMBL;Acc:Q5W0V6] [ENST00000378066]	ENST0000037806	Unknown	
A_24_P86252	-4.3	1.6	A_24_P862524	A_24_P862524	Unknown	
DNAJC19	-4.3	2.0	DnaJ (Hsp40) homolog, subfamily C, member 19 (DNAJC19), mRNA [NM_145261]	NM_145261	Hs.230601	NM_145261
C2orf13	-4.3	3.0	chromosome 2 open reading frame 13 (C2orf13), mRNA [NM_173545]	NM_173545	Hs.258941	NM_173545
AK023645	-4.3	1.9	cDNA FLJ13583 fis, clone PLACE1009050. [AK023645]	AK023645	Hs.658324	AK023645
GSTA3	-4.3	2.5	glutathione S-transferase A3 (GSTA3), mRNA [NM_000847]	NM_000847	Hs.102484	NM_000847
TMEM46	-4.2	3.4	transmembrane protein 46 (TMEM46), mRNA [NM_001007538]	NM_001007538	Hs.433791	NM_001007538
RARB	-4.2	1.9	retinoic acid receptor, beta (RARB), transcript variant 1, mRNA [NM_000965]	NM_000965	Hs.654490	BC030234
LOC285831	-4.2	2.7	cDNA FLJ37752 fis, clone BRHIP2023309. [AK095071]	AK095071	Hs.654655	AK095071
MCM6	-4.2	2.3	minichromosome maintenance deficient 6 homolog (S. cerevisiae) (MCM6), mRNA [NM_005915]	NM_005915	Hs.444118	NM_005915
KNTC1	-4.2	2.5	kinetochore associated 1 (KNTC1), mRNA [NM_014708]	NM_014708	Hs.300559	BC150278
SCD	-4.2	2.9	stearoyl-CoA desaturase (delta-9-desaturase) (SCD), mRNA [NM_005063]	NM_005063	Hs.558396	NM_005063
THC2657587	-4.2	4.2	Q3MWI9_9DELT (Q3MWI9) Fumarate reductase flavoprotein subunit precursor, partial (11%) [THC2657587]	THC2657587	Unknown	
N34499	-4.2	2.9	N34499 yy55g04.s1 Soares_multiple_sclerosis_2NbHMSP cDNA clone IMAGE:277494 3' similar to contains Alu repetitive element;contains element MER9 repetitive element ;, mRNA sequence [N34499]	N34499	Hs.655670	AF007132
DHX35	-4.2	1.9	DEAH (Asp-Glu-Ala-His) box polypeptide 35 (DHX35), mRNA [NM_021931]	NM_021931	Hs.444520	BC033453
THC2653001	-4.2	2.1	BX098637 BX098637 Soares fetal liver spleen 1NFLS cDNA clone IMAGp998F16386 ; IMAGE:200847, mRNA sequence [BX098637]	THC2653001	Unknown	
CEP70	-4.2	1.8	centrosomal protein 70kDa (CEP70), mRNA [NM_024491]	NM_024491	Hs.531962	NM_024491
A_24_P13524	-4.2	1.4	A_24_P135242	A_24_P135242	Unknown	
LOC650848	-4.2	1.9	PREDICTED: similar to 60S ribosomal protein L17 (L23) (LOC650848), mRNA [XR_019013]	XR_019013	Hs.647122	XR_019013
KNTC1	-4.2	2.4	kinetochore associated 1 (KNTC1), mRNA [NM_014708]	NM_014708	Hs.300559	BC150278
LOC92345	-4.2	4.7	hypothetical protein BC008207 (LOC92345), mRNA [NM_138386]	NM_138386	Hs.655698	AK126037
HHEX	-4.2	5.0	homeobox, hematopoietically expressed (HHEX), mRNA [NM_002729]	NM_002729	Hs.118651	BC015110
BG719281	-4.2	1.8	BG719281 602690354F1 NIH_MGC_97 cDNA clone IMAGE:4822789 5', mRNA sequence [BG719281]	BG719281	Hs.431257	BE894056
THC2509970	-4.2	2.1	T97996 ye56c04.s1 Soares fetal liver spleen 1NFLS cDNA clone IMAGE:121734 3' similar to SP:RL2B_RAT P29316 60S RIBOSOMAL PROTEIN ;, mRNA sequence [T97996]	THC2509970	Unknown	
LOC387787	-4.2	2.0	PREDICTED: similar to CG9804-PA, transcript variant 1 (LOC387787), mRNA [XM_370636]	XM_370636	Hs.591971	XM_370636
ZDHHC20	-4.2	2.0	cDNA FLJ33660 fis, clone BRAMY2026395. [AK090979]	AK090979	Hs.564611	AK090979
PTPN4	-4.2	1.6	protein tyrosine phosphatase, non-receptor type 4 (megakaryocyte) (PTPN4), mRNA [NM_002830]	NM_002830	Hs.469809	BX648614
AK001846	-4.2	3.3	cDNA FLJ10984 fis, clone PLACE1001810. [AK001846]	AK001846	Hs.373763	BX647784
HDAC4	-4.2	2.3	histone deacetylase 4 (HDAC4), mRNA [NM_006037]	NM_006037	Hs.20516	NM_006037
RBMX	-4.2	1.8	RNA binding motif protein, X-linked (RBMX), mRNA [NM_002139]	NM_002139	Hs.380118	BX647131
THC2713242	-4.2	2.9	THC2713242	THC2713242	Unknown	

TMEM97	-4.2	2.6	transmembrane protein 97 (TMEM97), mRNA [NM_014573]	NM_014573	Hs.199695	NM_014573
A_23_P10091	-4.2	1.7	A_23_P10091	A_23_P10091	Unknown	
LOC375295	-4.2	2.3	hypothetical gene supported by BC013438, mRNA (cDNA clone IMAGE:3899073), partial cds. [BC013438]	ENST0000029554	Unknown	
HNRPU	-4.2	2.1	heterogeneous nuclear ribonucleoprotein U (scaffold attachment factor A) (HNRPU), transcript variant 1, mRNA [NM_031844]	NM_031844	Hs.166463	NM_031844
LOC391813	-4.2	1.7	PREDICTED: similar to zinc finger, DHHC domain containing 3 (LOC391813), mRNA [XR_018686]	XR_018686	Hs.651025	XR_018686
FAM122C	-4.2	2.9	cDNA FLJ33292 fis, clone BNGH42000130, weakly similar to beta glucuronidase isoform. [AK090611]	AK090611	Hs.269127	AK090611
WDR36	-4.2	2.0	WD repeat domain 36 (WDR36), mRNA [NM_139281]	NM_139281	Hs.533237	NM_139281
AF119889	-4.2	1.6	PRO2667 mRNA, complete cds. [AF119889]	AF119889	Unknown	
AK022893	-4.2	3.0	cDNA FLJ12831 fis, clone NT2RP2003099. [AK022893]	AK022893	Hs.288478	BC037533
AA451906	-4.2	1.6	AA451906 zx16h04.s1 Soares_total_fetus_Nb2HF8_9w cDNA clone IMAGE:786679 3', mRNA sequence [AA451906]	AA451906	Hs.193163	AL713697
ZNF181	-4.2	1.8	zinc finger protein 181 (ZNF181), mRNA [NM_001029997]	NM_001029997	Hs.659191	BC043228
LOC645158	-4.2	3.8	hypothetical protein LOC645158, mRNA (cDNA clone IMAGE:4798132), partial cds. [BC018088]	BC018088	Hs.699827	XM_932692
SMCR7L	-4.2	1.4	Smith-Magenis syndrome chromosome region, candidate 7-like (SMCR7L), mRNA [NM_019008]	NM_019008	Hs.148677	NM_019008
DEK	-4.2	2.0	DEK oncogene (DNA binding) (DEK), mRNA [NM_003472]	NM_003472	Hs.695957	BX641063
WDR51B	-4.2	2.1	WD repeat domain 51B (WDR51B), mRNA [NM_172240]	NM_172240	Hs.25130	BX648293
C10orf89	-4.2	1.8	chromosome 10 open reading frame 89 (C10orf89), mRNA [NM_153336]	NM_153336	Hs.281004	AL833506
LOC389435	-4.2	1.6	AGENCOURT_14302370 NIH_MGC_173 cDNA 5', mRNA sequence [CD385156]	CD385156	Hs.699334	CD385156
FLJ37953	-4.2	2.2	hypothetical protein FLJ37953 (FLJ37953), mRNA [NM_001039693]	NM_001039693	Hs.204619	NM_001039693
AA865794	-4.2	2.6	AA865794 og96h04.s1 NCI_CGAP_Kid3 cDNA clone IMAGE:1456183 3', mRNA sequence [AA865794]	AA865794	Hs.194143	NM_007295
A_24_P40968	-4.2	1.6	A_24_P409681	A_24_P409681	Unknown	
SPECC1L	-4.2	1.4	SPECC1-like (SPECC1L), mRNA [NM_015330]	NM_015330	Hs.474384	NM_015330
TTC23	-4.2	2.1	tetratricopeptide repeat domain 23 (TTC23), transcript variant 1, mRNA [NM_001040655]	NM_001040655	Hs.513195	AF411456
UBP1	-4.2	1.4	upstream binding protein 1 (LBP-1a) (UBP1), mRNA [NM_014517]	NM_014517	Hs.696578	CR749798
COL6A3	-4.2	3.6	collagen, type VI, alpha 3 (COL6A3), transcript variant 1, mRNA [NM_004369]	NM_004369	Hs.233240	NM_004369
A_24_P89637	-4.2	1.6	A_24_P896373	A_24_P896373	Unknown	
ROPN1B	-4.2	7.1	ropporin, rhophilin associated protein 1B (ROPN1B), mRNA [NM_001012337]	NM_001012337	Hs.663128	BM552738
WSB1	-4.2	1.6	WD repeat and SOCS box-containing 1 (WSB1), transcript variant 1, mRNA [NM_015626]	NM_015626	Hs.446017	NM_015626
KNTC1	-4.2	2.5	kinetochore associated 1 (KNTC1), mRNA [NM_014708]	NM_014708	Hs.300559	BC150278
CENPQ	-4.2	2.2	centromere protein Q (CENPQ), mRNA [NM_018132]	NM_018132	Hs.88663	NM_018132
SERGEF	-4.2	1.5	secretion regulating guanine nucleotide exchange factor (SERGEF), mRNA [NM_012139]	NM_012139	Hs.32470	BC000707
THC2633920	-4.2	4.0	Q3RFB1_XYLFA (Q3RFB1) Aminotransferase, class I and II, partial (4%) [THC2633920]	THC2633920	Unknown	
CAND1	-4.2	1.8	cullin-associated and neddylation-dissociated 1 (CAND1), mRNA [NM_018448]	NM_018448	Hs.546407	AB020636
PHF14	-4.2	2.4	PHD finger protein 14 (PHF14), transcript variant 2, mRNA [NM_014660]	NM_014660	Hs.655688	NM_014660
CPNE7	-4.2	2.1	copine VII (CPNE7), transcript variant 2, mRNA [NM_014427]	NM_014427	Hs.461775	NM_014427
THC2718728	-4.2	4.2	THC2718728	THC2718728	Unknown	
DAPK1	-4.2	1.9	death-associated protein kinase 1 (DAPK1), mRNA [NM_004938]	NM_004938	Hs.380277	NM_004938
SETDB2	-4.2	1.4	SET domain, bifurcated 2 (SETDB2), mRNA [NM_031915]	NM_031915	Hs.631789	AB209349
DMRT3	-4.2	4.0	doublesex and mab-3 related transcription factor 3 (DMRT3), mRNA [NM_021240]	NM_021240	Hs.189174	NM_021240
HMGB1	-4.2	2.3	high-mobility group box 1 (HMGB1), mRNA [NM_002128]	NM_002128	Hs.434102	AK122825
L3MBTL3	-4.2	1.7	l(3)mbt-like 3 (Drosophila) (L3MBTL3), transcript variant 1, mRNA [NM_032438]	NM_032438	Hs.658051	NM_032438
HLCS	-4.2	1.7	holocarboxylase synthetase (biotin-(proprionyl-Coenzyme A-carboxylase (ATP-hydrolysing)) ligase) (HLCS), mRNA [NM_000411]	NM_000411	Hs.371350	D87328
LOC286025	-4.2	1.9	cDNA FLJ38703 fis, clone KIDNE2002265. [AK096022]	AK096022	Hs.605380	NM_020910
SLC9A3R1	-4.2	2.1	solute carrier family 9 (sodium/hydrogen exchanger), member 3 regulator 1 (SLC9A3R1), mRNA [NM_004252]	NM_004252	Hs.699203	BX648303
BC039097	-4.2	1.9	cDNA clone IMAGE:4823416. [BC039097]	BC039097	Unknown	
A_24_P14416	-4.2	2.2	A_24_P144163	A_24_P144163	Unknown	
CR624679	-4.2	3.1	full-length cDNA clone CS0DF003YC20 of Fetal brain of (human). [CR624679]	CR624679	Hs.399952	CR624679
A_32_P18403	-4.2	1.6	A_32_P184039	A_32_P184039	Unknown	
PNN	-4.2	1.7	pinin, desmosome associated protein (PNN), mRNA [NM_002687]	NM_002687	Hs.409965	NM_002687
MTR	-4.2	1.7	Methionine synthase (EC 2.1.1.13) (5-methyltetrahydrofolate-- homocysteine methyltransferase) (Methionine synthase, vitamin-B12 dependent) (MS). [Source:Uniprot/SWISSPROT;Acc:Q99707] [ENST00000366577]	ENST0000036657	Unknown	

AF230200	-4.2	1.7	OVN6-2 mRNA, partial cds. [AF230200]	AF230200	Hs.514802	BM559175
MGC13379	-4.2	1.8	HSPC244 (MGC13379), mRNA [NM_016499]	NM_016499	Hs.26745	NM_016499
AK027667	-4.2	2.1	cDNA FLJ14761 fis, clone NT2RP3003302. [AK027667]	AK027667	Unknown	
RFC4	-4.2	1.8	replication factor C (activator 1) 4, 37kDa (RFC4), transcript variant 1, mRNA [NM_002916]	NM_002916	Hs.518475	NM_002916
EPC1	-4.2	1.6	enhancer of polycomb homolog 1 (Drosophila), mRNA (cDNA clone MGC:33656 IMAGE:4828057), complete cds. [BC036529]	BC036529	Hs.167805	BC036529
CCBE1	-4.2	3.8	collagen and calcium binding EGF domains 1 (CCBE1), mRNA [NM_133459]	NM_133459	Hs.34333	AB075863
KIF15	-4.2	3.3	kinesin family member 15 (KIF15), mRNA [NM_020242]	NM_020242	Hs.658939	NM_020242
LOC149134	-4.2	2.2	hypothetical protein LOC149134 (LOC149134), mRNA [NM_207326]	NM_207326	Unknown	
BCKDHB	-4.2	1.7	branched chain keto acid dehydrogenase E1, beta polypeptide (maple syrup urine disease) (BCKDHB), nuclear gene encoding mitochondrial protein, transcript variant 2, mRNA [NM_000056]	NM_000056	Hs.654441	BC034481
CRKL	-4.2	2.2	v-crk sarcoma virus CT10 oncogene homolog (avian)-like (CRKL), mRNA [NM_005207]	NM_005207	Hs.592205	NM_005207
C4orf27	-4.2	1.8	chromosome 4 open reading frame 27 (C4orf27), mRNA [NM_017867]	NM_017867	Hs.406756	AK124036
TRIM13	-4.2	1.8	tripartite motif-containing 13 (TRIM13), transcript variant 3, mRNA [NM_213590]	NM_213590	Hs.436922	NM_213590
EIF4ENIF1	-4.2	1.4	eukaryotic translation initiation factor 4E nuclear import factor 1 (EIF4ENIF1), mRNA [NM_019843]	NM_019843	Hs.517559	BC033028
C13orf18	-4.2	3.0	chromosome 13 open reading frame 18 (C13orf18), mRNA [NM_025113]	NM_025113	Unknown	
SAMD13	-4.2	2.4	sterile alpha motif domain containing 13 (SAMD13), mRNA [NM_001010971]	NM_001010971	Hs.591445	NM_001010971
BC037740	-4.2	1.6	cDNA clone IMAGE:5263531. [BC037740]	BC037740	Hs.597434	BC037740
ZNF45	-4.2	1.7	zinc finger protein 45 (ZNF45), mRNA [NM_003425]	NM_003425	Hs.381285	AK127739
RPL23A	-4.2	1.7	ribosomal protein L23a (RPL23A), mRNA [NM_000984]	NM_000984	Hs.419463	CR616046
RPS13	-4.2	1.8	ribosomal protein S13 (RPS13), mRNA [NM_001017]	NM_001017	Hs.446588	BE621497
SHANK2	-4.2	2.1	SH3 and multiple ankyrin repeat domains 2 (SHANK2), transcript variant 1, mRNA [NM_012309]	NM_012309	Hs.268726	NM_012309
LAG3	-4.2	2.8	lymphocyte-activation gene 3 (LAG3), mRNA [NM_002286]	NM_002286	Hs.409523	BC037962
RPL23	-4.2	1.5	ribosomal protein L23 (RPL23), mRNA [NM_000978]	NM_000978	Hs.406300	CR604268
AK026896	-4.2	1.6	cDNA: FLJ23243 fis, clone COL01757. [AK026896]	AK026896	Hs.661702	AK026896
KDELC1	-4.2	1.6	KDEL (Lys-Asp-Glu-Leu) containing 1 (KDELC1), mRNA [NM_024089]	NM_024089	Hs.408629	BC051860
AF086448	-4.2	1.6	full length insert cDNA clone ZD82B02. [AF086448]	AF086448	Hs.633012	BQ064859
BC014370	-4.2	1.9	cDNA clone IMAGE:3961179, partial cds. [BC014370]	BC014370	Hs.525922	BC014370
MCM2	-4.2	2.2	MCM2 minichromosome maintenance deficient 2, mitotin (S. cerevisiae) (MCM2), mRNA [NM_004526]	NM_004526	Hs.477481	NM_004526
BU622073	-4.2	1.9	BU622073 UI-H-FH1-bfu-a-21-0-UI.s1 NCI_CGAP_FH1 cDNA clone UI-H-FH1-bfu-a-21-0-UI 3', mRNA sequence [BU622073]	BU622073	Hs.503438	BC045621
KRT222P	-4.2	2.7	keratin 222 pseudogene (KRT222P), mRNA [NM_152349]	NM_152349	Hs.6920	NM_152349
KIAA0146	-4.2	1.5	full-length cDNA clone CS0DK002YC19 of HeLa cells Cot 25-normalized of (human). [CR614430]	ENST0000029742	Unknown	
PRR3	-4.2	1.7	proline rich 3 (PRR3), transcript variant 1, mRNA [NM_025263]	NM_025263	Hs.118354	NM_025263
ZNF195	-4.2	1.5	zinc finger protein 195 (ZNF195), mRNA [NM_007152]	NM_007152	Hs.386294	AL833722
FAM13A1	-4.2	3.4	mRNA; cDNA DKFZp686O13152 (from clone DKFZp686O13152). [BX647410]	BX647410	Hs.97270	NM_014883
HDHD2	-4.2	1.5	haloacid dehalogenase-like hydrolase domain containing 2 (HDHD2), mRNA [NM_032124]	NM_032124	Hs.465041	BX537623
PSPC1	-4.2	1.3	paraspeckle component 1 (PSPC1), transcript variant alpha, mRNA [NM_001042414]	NM_001042414	Hs.213198	AK090873
THC2676139	-4.2	1.7	THC2676139	THC2676139	Unknown	
ZNF521	-4.2	2.0	zinc finger protein 521 (ZNF521), mRNA [NM_015461]	NM_015461	Hs.116935	AK027354
THC2616009	-4.2	2.5	THC2616009	THC2616009	Unknown	
ZNF420	-4.2	1.8	zinc finger protein 420 (ZNF420), mRNA [NM_144689]	NM_144689	Hs.444992	BC026210
RBMX	-4.2	2.4	RNA binding motif protein, X-linked (RBMX), mRNA [NM_002139]	NM_002139	Hs.380118	BX647131
AK002005	-4.2	3.3	cDNA FLJ11143 fis, clone PLACE1006598. [AK002005]	AK002005	Hs.574781	AK002005
RFXANK	-4.2	1.6	regulatory factor X-associated ankyrin-containing protein (RFXANK), transcript variant 1, mRNA [NM_003721]	NM_003721	Hs.296776	BM455790
GALNTL2	-4.2	2.8	UDP-N-acetyl-alpha-D-galactosamine:polypeptide N-acetylgalactosaminyltransferase-like 2 (GALNTL2), mRNA [NM_054110]	NM_054110	Hs.411308	AL832575
CCNJL	-4.2	2.1	cyclin J-like (CCNJL), mRNA [NM_024565]	NM_024565	Hs.14070	NM_024565
KNTC1	-4.2	2.4	kinetochore associated 1 (KNTC1), mRNA [NM_014708]	NM_014708	Hs.300559	BC150278
A_24_P33232	-4.2	2.4	A_24_P332326	A_24_P332326	Unknown	
C9orf80	-4.2	8.1	chromosome 9 open reading frame 80, mRNA (cDNA clone IMAGE:4342185), partial cds. [BC013097]	BC013097	Hs.658575	AK000637
EPC2	-4.2	1.3	enhancer of polycomb homolog 2 (Drosophila) (EPC2), mRNA [NM_015630]	NM_015630	Hs.23270	AK125789
TKTL1	-4.2	1.9	transketolase-like 1 (TKTL1), mRNA [NM_012253]	NM_012253	Hs.102866	NM_012253

ENST0000033	-4.1	4.7	Nuclear factor erythroid 2-related factor 2 (NF-E2-related factor 2) (NFE2-related factor 2) (Nuclear factor, erythroid derived 2, like 2) (HEBP1). [Source:Uniprot/SWISSPROT;Acc:Q16236] [ENST00000338358]	ENST0000033835	Unknown	
PDE5A	-4.1	3.1	phosphodiesterase 5A, cGMP-specific (PDE5A), transcript variant 1, mRNA [NM_001083]	NM_001083	Hs.647971	NM_001083
ABCD4	-4.1	1.5	ATP-binding cassette, sub-family D (ALD), member 4 (ABCD4), transcript variant 1, mRNA [NM_005050]	NM_005050	Hs.94395	AK125109
KIAA1267	-4.1	2.1	KIAA1267 (KIAA1267), mRNA [NM_015443]	NM_015443	Hs.648744	BX538006
THC2678806	-4.1	3.8	HESX1_HUMAN (Q9UBX0) Homeobox expressed in ES cells 1 (Homeobox protein ANF) (hAnf), partial (70%) [THC2678806]	THC2678806	Unknown	
PEX3	-4.1	1.4	peroxisomal biogenesis factor 3 (PEX3), mRNA [NM_003630]	NM_003630	Hs.7277	BX648803
AK025344	-4.1	1.7	cDNA: FLJ21691 fis, clone COL09555. [AK025344]	AK025344	Hs.476353	NM_018403
C3orf26	-4.1	2.7	chromosome 3 open reading frame 26 (C3orf26), mRNA [NM_032359]	NM_032359	Hs.655111	BQ065087
ASCC3L1	-4.1	1.3	activating signal cointegrator 1 complex subunit 3-like 1 (ASCC3L1), mRNA [NM_014014]	NM_014014	Hs.246112	NM_014014
BI963219	-4.1	1.9	BI963219 ie57h11.x1 Melton Normalized Human Islet 4 N4-HIS 1 cDNA clone IMAGE:5671172 3', mRNA sequence [BI963219]	BI963219	Hs.118820	AK123020
CXorf42	-4.1	2.5	mRNA; cDNA DKFZp686B1195 (from clone DKFZp686B1195). [BX537594]	BX537594	Unknown	
CEBPA	-4.1	2.0	CCAAT/enhancer binding protein (C/EBP), alpha (CEBPA), mRNA [NM_004364]	NM_004364	Hs.699463	NM_004364
ATR	-4.1	1.5	ataxia telangiectasia and Rad3 related (ATR), mRNA [NM_001184]	NM_001184	Hs.271791	NM_001184
C12orf47	-4.1	1.9	full-length cDNA clone CS0DJ012YG18 of T cells (Jurkat cell line) Cot 10-normalized of (human). [CR620831]	CR620831	Hs.333120	AK096935
RPL21	-4.1	1.4	ribosomal protein L21 (RPL21), mRNA [NM_000982]	NM_000982	Hs.381123	CR602527
AK021980	-4.1	2.1	cDNA FLJ11918 fis, clone HEMBB1000272. [AK021980]	AK021980	Hs.644653	NM_001083962
FLJ11021	-4.1	1.7	similar to splicing factor, arginine/serine-rich 4 (FLJ11021), transcript variant 2, mRNA [NM_198261]	NM_198261	Hs.432996	NM_198261
FXN	-4.1	1.9	frataxin (FXN), nuclear gene encoding mitochondrial protein, transcript variant 2, mRNA [NM_181425]	NM_181425	Hs.29978	NM_181425
TMEM135	-4.1	1.9	transmembrane protein 135 (TMEM135), mRNA [NM_022918]	NM_022918	Hs.188591	BX648678
PDZRN3	-4.1	2.2	PDZ domain containing RING finger 3 (PDZRN3), mRNA [NM_015009]	NM_015009	Hs.434900	AB029018
RPL39	-4.1	1.8	ribosomal protein L39 (RPL39), mRNA [NM_001000]	NM_001000	Hs.558387	CR625387
SYT10	-4.1	2.3	synaptotagmin X (SYT10), mRNA [NM_198992]	NM_198992	Hs.118703	AY198413
HMCN1	-4.1	2.4	hemicentin 1 (HMCN1), mRNA [NM_031935]	NM_031935	Hs.58877	NM_031935
PIGC	-4.1	1.7	H.sapiens gene from PAC 106H8. [AL035301]	AL035301	Unknown	
ECHDC1	-4.1	1.6	enoyl Coenzyme A hydratase domain containing 1 (ECHDC1), mRNA [NM_018479]	NM_018479	Hs.486410	AL834469
CHRNB3	-4.1	1.8	cholinergic receptor, nicotinic, beta 3 (CHRNB3), mRNA [NM_000749]	NM_000749	Hs.654576	NM_000749
IGSF3	-4.1	1.6	immunoglobulin superfamily, member 3 (IGSF3), transcript variant 1, mRNA [NM_001542]	NM_001542	Hs.171057	NM_001542
LRRC31	-4.1	2.2	leucine rich repeat containing 31 (LRRC31), mRNA [NM_024727]	NM_024727	Hs.411295	NM_024727
AK056119	-4.1	1.6	cDNA FLJ31557 fis, clone NT2RI2001307. [AK056119]	AK056119	Hs.665456	AK056119
ZBTB34	-4.1	1.5	mRNA for KIAA1993 protein. [AB082524]	AB082524	Hs.177633	NM_001099270
EIF5	-4.1	2.3	eukaryotic translation initiation factor 5 (EIF5), transcript variant 1, mRNA [NM_001969]	NM_001969	Hs.433702	NM_001969
KIAA0240	-4.1	1.7	KIAA0240 (KIAA0240), mRNA [NM_015349]	NM_015349	Hs.658033	NM_015349
AK057956	-4.1	2.3	cDNA FLJ25227 fis, clone STM01429. [AK057956]	AK057956	Hs.516023	AK095574
DEPDC4	-4.1	1.9	cDNA FLJ33505 fis, clone BRAMY2004542. [AK090824]	AK090824	Hs.653118	AK090824
6-Sep	-4.1	1.8	septin 6 (SEPT6), transcript variant V, mRNA [NM_145802]	NM_145802	Hs.496666	NM_145799
THC2572908	-4.1	1.9	RATRPL5 ribosomal protein L5 {Rattus norvegicus} (exp=-1; wgp=0; cg=0), partial (17%) [THC2572908]	THC2572908	Unknown	
GUSBP1	-4.1	2.5	glucuronidase, beta pseudogene 1 (GUSBP1), mRNA [NM_207331]	NM_207331	Unknown	
LIN28B	-4.1	5.4	lin-28 homolog B (C. elegans) (LIN28B), mRNA [NM_001004317]	NM_001004317	Hs.23616	NM_001004317
STATIP1	-4.1	1.6	signal transducer and activator of transcription 3 interacting protein 1 (STATIP1), mRNA [NM_018255]	NM_018255	Hs.8739	AF403223
BCL2	-4.1	1.9	B-cell CLL/lymphoma 2 (BCL2), nuclear gene encoding mitochondrial protein, transcript variant alpha, mRNA [NM_000633]	NM_000633	Hs.150749	NM_000633
ZNF516	-4.1	2.1	Zinc finger protein 516. [Source:Uniprot/SWISSPROT;Acc:Q92618] [ENST00000217537]	ENST0000021753	Unknown	
NAP1L3	-4.1	2.0	nucleosome assembly protein 1-like 3 (NAP1L3), mRNA [NM_004538]	NM_004538	Hs.21365	BC094729
RPL31	-4.1	1.4	ribosomal protein L31 (RPL31), mRNA [NM_000993]	NM_000993	Hs.469473	CR595074
THC2509970	-4.1	2.0	T97996 ye56c04.s1 Soares fetal liver spleen 1NFLS cDNA clone IMAGE:121734 3' similar to SP:RL2B_RAT P29316 60S RIBOSOMAL PROTEIN ;, mRNA sequence [T97996]	THC2509970	Unknown	
AQR	-4.1	1.7	cDNA FLJ10311 fis, clone NT2RM2000359, highly similar to mRNA for KIAA0560 protein. [AK001173]	AK001173	Hs.510958	EF553519
SCTR	-4.1	2.6	secretin receptor (SCTR), mRNA [NM_002980]	NM_002980	Hs.700578	AK055551
BZRAP1	-4.1	1.5	benzodiazapine receptor (peripheral) associated protein 1 (BZRAP1), mRNA [NM_004758]	NM_004758	Hs.112499	BC150279
LOC388621	-4.1	1.6	PREDICTED: similar to ribosomal protein L21 (LOC388621), mRNA [XM_371243]	XM_371243	Unknown	

PALB2	-4.1	2.0	partner and localizer of BRCA2 (PALB2), mRNA [NM_024675]	NM_024675	Hs.444664	BX647125
GPRASP2	-4.1	1.6	G protein-coupled receptor associated sorting protein 2 (GPRASP2), transcript variant 1, mRNA [NM_001004051]	NM_001004051	Hs.656888	BC051707
A_32_P22056	-4.1	1.9	A_32_P220567	A_32_P220567	Unknown	
MGC27345	-4.1	1.6	hypothetical protein MGC27345, mRNA (cDNA clone MGC:27345 IMAGE:4670552), complete cds. [BC024231]	BC024231	Hs.696551	AK001384
C10orf65	-4.1	2.1	chromosome 10 open reading frame 65 (C10orf65), mRNA [NM_138413]	NM_138413	Hs.180346	AK094791
THC2618720	-4.1	1.9	THC2618720	THC2618720	Unknown	
AL137354	-4.1	2.1	mRNA; cDNA DKFZp434A0326 (from clone DKFZp434A0326). [AL137354]	AL137354	Hs.569174	BC042855
A_24_P78984	-4.1	1.9	A_24_P789842	A_24_P789842	Unknown	
FREM1	-4.1	2.3	FRAS1 related extracellular matrix 1 (FREM1), mRNA [NM_144966]	NM_144966	Hs.50850	BX648240
ALS2CR13	-4.1	1.9	amyotrophic lateral sclerosis 2 (juvenile) chromosome region, candidate 13 (ALS2CR13), mRNA [NM_173511]	NM_173511	Hs.471130	NM_173511
GTF2I	-4.1	1.6	general transcription factor II, i (GTF2I), transcript variant 1, mRNA [NM_032999]	NM_032999	Hs.647041	NM_032999
SLC24A1	-4.1	2.7	solute carrier family 24 (sodium/potassium/calcium exchanger), member 1 (SLC24A1), mRNA [NM_004727]	NM_004727	Hs.173092	NM_004727
KLHL15	-4.1	1.8	Kelch-like protein 15. [Source:Uniprot/SWISSPROT;Acc:Q96M94] [ENST00000328046]	ENST0000032804	Unknown	
A_24_P39290	-4.1	1.9	A_24_P392900	A_24_P392900	Unknown	
PDZRN4	-4.1	4.9	PDZ domain containing RING finger 4 (PDZRN4), mRNA [NM_013377]	NM_013377	Hs.380044	AK131554
RBMX	-4.1	2.3	RNA binding motif protein, X-linked (RBMX), mRNA [NM_002139]	NM_002139	Hs.380118	BX647131
OSTalpha	-4.1	2.3	organic solute transporter alpha (OSTalpha), mRNA [NM_152672]	NM_152672	Hs.651164	AK172837
LCOR	-4.1	1.3	ligand dependent nuclear receptor corepressor (LCOR), mRNA [NM_032440]	NM_032440	Hs.500695	AL834245
ZNF228	-4.1	1.8	zinc finger protein 228 (ZNF228), mRNA [NM_013380]	NM_013380	Hs.48589	AL833447
LOC391124	-4.1	1.8	PREDICTED: similar to ribosomal protein L27a (LOC391124), mRNA [XM_497719]	XM_497719	Unknown	
AK026215	-4.1	1.4	cDNA: FLJ22562 fis, clone HSI01814. [AK026215]	AK026215	Hs.699291	AB209215
CD8B	-4.1	2.7	CD8b molecule (CD8B), transcript variant 1, mRNA [NM_172099]	NM_172099	Hs.405667	BX647150
NUDT10	-4.1	2.3	nudix (nucleoside diphosphate linked moiety X)-type motif 10 (NUDT10), mRNA [NM_153183]	NM_153183	Hs.375178	AF469196
USP25	-4.1	2.4	ubiquitin specific peptidase 25 (USP25), mRNA [NM_013396]	NM_013396	Hs.473370	AL833500
AIG1	-4.1	1.4	androgen-induced 1 (AIG1), mRNA [NM_016108]	NM_016108	Hs.567501	BX640703
SPAG16	-4.1	1.6	sperm associated antigen 16 (SPAG16), transcript variant 2, mRNA [NM_001025436]	NM_001025436	Hs.699406	CR749477
ATF7IP	-4.1	1.5	activating transcription factor 7 interacting protein (ATF7IP), mRNA [NM_018179]	NM_018179	Hs.591151	NM_018179
KNTC1	-4.1	2.4	kinetochore associated 1 (KNTC1), mRNA [NM_014708]	NM_014708	Hs.300559	BC150278
THC2708826	-4.1	1.7	THC2708826	THC2708826	Unknown	
FGG	-4.1	2.9	fibrinogen gamma chain (FGG), transcript variant gamma-A, mRNA [NM_000509]	NM_000509	Hs.546255	CR620626
CLUAP1	-4.1	1.4	clusterin associated protein 1 (CLUAP1), transcript variant 2, mRNA [NM_024793]	NM_024793	Hs.155995	AB014543
TM6SF1	-4.1	2.9	cDNA FLJ30876 fis, clone FEBRA2004412. [AK055438]	AK055438	Hs.660989	AK055438
THUMPD2	-4.1	2.0	THUMP domain containing 2 (THUMPD2), mRNA [NM_025264]	NM_025264	Hs.468254	AF380578
THC2646626	-4.1	4.8	THC2646626	THC2646626	Unknown	
RSF1	-4.1	1.6	remodeling and spacing factor 1 (RSF1), mRNA [NM_016578]	NM_016578	Hs.420229	AF227948
BC030084	-4.1	1.8	cDNA clone IMAGE:4791887. [BC030084]	BC030084	Hs.167087	BC048014
LOC730902	-4.1	1.4	PREDICTED: similar to 60S ribosomal protein L7 (LOC730902), mRNA [XR_015600]	XR_015600	Hs.653167	XR_015600
ATP8B3	-4.1	2.3	ATPase, Class I, type 8B, member 3 (ATP8B3), mRNA [NM_138813]	NM_138813	Hs.306212	AK125969
LCORL	-4.1	2.1	cDNA FLJ30696 fis, clone FCBBF2000808. [AK055258]	AK055258	Hs.661849	AK055258
PRL	-4.1	12.3	prolactin (PRL), mRNA [NM_000948]	NM_000948	Hs.1905	CD512992
C11orf41	-4.1	2.9	Human G2 protein mRNA, partial cds. [U10991]	U10991	Hs.502266	NM_012194
CENPQ	-4.1	2.3	centromere protein Q (CENPQ), mRNA [NM_018132]	NM_018132	Hs.88663	NM_018132
ZNF718	-4.1	2.2	zinc finger protein 718 (ZNF718), mRNA [NM_001039127]	NM_001039127	Hs.428579	AL833758
POSTN	-4.1	2.1	periostin, osteoblast specific factor (POSTN), mRNA [NM_006475]	NM_006475	Hs.136348	D13666
AK129724	-4.1	2.8	cDNA FLJ26213 fis, clone ADG07906. [AK129724]	AK129724	Unknown	
AI541080	-4.1	2.2	pec1.2-1.H02 ecnorm cDNA 3', mRNA sequence [AI541080]	AI541080	Hs.605520	AI541080
THC2559650	-4.1	3.5	ALU5_HUMAN (P39192) Alu subfamily SC sequence contamination warning entry, partial (11%) [THC2559650]	THC2559650	Unknown	
THC2686813	-4.1	1.6	THC2686813	THC2686813	Unknown	
THC2682152	-4.1	1.6	Q9F8M7_CARHY (Q9F8M7) DTDP-glucose 4,6-dehydratase (Fragment), partial (11%) [THC2682152]	THC2682152	Unknown	
TFAM	-4.1	2.2	Transcription factor A, mitochondrial precursor (mtTFA) (Mitochondrial transcription factor 1) (MtTF1) (Transcription factor 6-like 2). [Source:Uniprot/SWISSPROT;Acc:Q00059] [ENST00000373899]	ENST0000037389	Unknown	
HGF	-4.1	6.2	hepatocyte growth factor (hepapoietin A; scatter factor) (HGF), transcript variant 2, mRNA [NM_001010931]	NM_001010931	Hs.396530	NM_000601

SRBD1	-4.1	2.0	S1 RNA binding domain 1 (SRBD1), mRNA [NM_018079]	NM_018079	Hs.14229	NM_018079
ENST0000037	-4.1	1.6	RAS-responsive element-binding protein 1 (RREB-1) (Raf-responsive zinc finger protein LZ321). [Source:Uniprot/SWISSPROT;Acc:Q92766] [ENST00000379938]	ENST0000037993	Unknown	
HS2ST1	-4.1	1.7	heparan sulfate 2-O-sulfotransferase 1 (HS2ST1), mRNA [NM_012262]	NM_012262	Hs.48823	NM_012262
TMEM100	-4.1	2.3	transmembrane protein 100 (TMEM100), mRNA [NM_018286]	NM_018286	Hs.173233	NM_001099640
TMEM26	-4.1	2.6	transmembrane protein 26 (TMEM26), mRNA [NM_178505]	NM_178505	Hs.623955	CR749606
ATAD2B	-4.1	2.0	CDNA FLJ37502 fis, clone BRAWH2016446, moderately similar to TAT- BINDING HOMOLOG 7. (Fragment). [Source:Uniprot/SPTREMBL;Acc:Q8N9E7] [ENST00000238789]	ENST0000023878	Unknown	
NAIP	-4.1	3.8	NLR family, apoptosis inhibitory protein (NAIP), transcript variant 1, mRNA [NM_004536]	NM_004536	Hs.654500	NM_004536
SETD6	-4.1	1.6	SET domain containing 6 (SETD6), mRNA [NM_024860]	NM_024860	Hs.592060	AK024801
SUV420H1	-4.1	1.9	suppressor of variegation 4-20 homolog 1 (Drosophila) (SUV420H1), transcript variant 2, mRNA [NM_016028]	NM_016028	Hs.632120	NM_017635
LDB2	-4.1	2.2	LIM domain binding 2 (LDB2), mRNA [NM_001290]	NM_001290	Hs.23748	AL832772
LOC391847	-4.1	1.5	PREDICTED: similar to ribosomal protein L19 (LOC391847), mRNA [XR_019168]	XR_019168	Hs.646918	XR_019168
ZFAND1	-4.1	1.6	zinc finger, AN1-type domain 1 (ZFAND1), mRNA [NM_024699]	NM_024699	Hs.655453	AL833030
THC2644389	-4.1	5.4	THC2644389	THC2644389	Unknown	
IL1F9	-4.1	1.8	interleukin 1 family, member 9 (IL1F9), mRNA [NM_019618]	NM_019618	Hs.211238	AY359111
C11orf54	-4.1	1.6	chromosome 11 open reading frame 54 (C11orf54), mRNA [NM_014039]	NM_014039	Hs.8360	BC046477
AK025613	-4.1	2.1	cDNA: FLJ21960 fis, clone HEP05517. [AK025613]	AK025613	Hs.696159	NM_018387
ZNF195	-4.1	1.5	zinc finger protein 195 (ZNF195), mRNA [NM_007152]	NM_007152	Hs.386294	AL833722
RP11-301I17.1	-4.1	2.0	proliferation-inducing protein 38 (PIG38), mRNA [NM_017993]	NM_017993	Hs.128258	BC068521
LOC136143	-4.1	1.8	PREDICTED: similar to ribosomal protein L18 (LOC136143), mRNA [XM_069734]	XM_069734	Unknown	
ADAMTS9	-4.1	2.8	ADAM metallopeptidase with thrombospondin type 1 motif, 9 (ADAMTS9), mRNA [NM_182920]	NM_182920	Hs.656071	NM_182920
A_24_P36706	-4.1	1.7	A_24_P367063	A_24_P367063	Unknown	
LRRCC1	-4.1	2.8	leucine rich repeat and coiled-coil domain containing 1 (LRRCC1), transcript variant 1, mRNA [NM_033402]	NM_033402	Hs.193115	AB051551
BC034623	-4.1	2.4	cDNA clone IMAGE:4837603. [BC034623]	BC034623	Hs.697821	AK125759
NSBP1	-4.1	2.2	nucleosomal binding protein 1 (NSBP1), mRNA [NM_030763]	NM_030763	Hs.282204	AF250329
MMP21	-4.1	1.7	matrix metallopeptidase 21 (MMP21), mRNA [NM_147191]	NM_147191	Hs.314141	NM_147191
T12588	-4.1	2.2	CHR90108 Chromosome 9 exon II cDNA clone P94_53 5' and 3', mRNA sequence [T12588]	T12588	Hs.584190	BY795122
NACAP1	-4.1	2.0	nascent-polypeptide-associated complex alpha polypeptide pseudogene 1 (NACAP1) on chromosome 8 [NR_002182]	NR_002182	Unknown	
A_32_P15928	-4.1	2.6	A_32_P159289	A_32_P159289	Unknown	
CA429641	-4.1	1.7	UI-H-FH1-bfo-d-01-0-UI.s1 NCI_CGAP_FH1 cDNA clone UI-H-FH1-bfo-d-01-0-UI 3', mRNA sequence [CA429641]	CA429641	Hs.621904	CA429641
TAF3	-4.1	2.1	Transcription initiation factor TFIID subunit 3 (TBP-associated factor 3) (Transcription initiation factor TFIID 140 kDa subunit) (140 kDa TATA box-binding protein-associated factor) (TAF140) (TAFII140). [Source:Uniprot/SWISSPROT;Acc:Q5VWG9]...	ENST0000034429	Unknown	
TTC30B	-4.1	1.7	tetratricopeptide repeat domain 30B (TTC30B), mRNA [NM_152517]	NM_152517	Hs.447659	NM_152517
CNOT1	-4.1	1.6	CCR4-NOT transcription complex, subunit 1 (CNOT1), transcript variant 1, mRNA [NM_016284]	NM_016284	Hs.460923	NM_016284
ZC3H7A	-4.1	1.3	zinc finger CCCH-type containing 7A (ZC3H7A), mRNA [NM_014153]	NM_014153	Hs.371856	NM_014153
A_24_P91240	-4.1	1.7	A_24_P912404	A_24_P912404	Unknown	
SLC6A13	-4.1	4.3	solute carrier family 6 (neurotransmitter transporter, GABA), member 13, mRNA (cDNA clone IMAGE:4594185), complete cds. [BC020867]	BC020867	Hs.504398	NM_016615
A_24_P25063	-4.1	1.9	A_24_P25063	A_24_P25063	Unknown	
PAQR8	-4.1	2.3	progestin and adipoQ receptor family member VIII (PAQR8), mRNA [NM_133367]	NM_133367	Hs.239388	AY424286
BCL2	-4.1	1.9	B-cell CLL/lymphoma 2 (BCL2), nuclear gene encoding mitochondrial protein, transcript variant alpha, mRNA [NM_000633]	NM_000633	Hs.150749	NM_000633
ZNF215	-4.0	2.4	zinc finger protein 215 (ZNF215), mRNA [NM_013250]	NM_013250	Hs.523457	NM_013250
AK127569	-4.0	1.5	cDNA FLJ45662 fis, clone CTONG2027150. [AK127569]	AK127569	Hs.700700	XR_015706
AI819757	-4.0	1.6	AI819757 wj11h10.x1 NCI_CGAP_Kid12 cDNA clone IMAGE:2402563 3' similar to gb:A18658 INSULIN RECEPTOR PRECURSOR (HUMAN);, mRNA sequence [AI819757]	AI819757	Hs.693974	BQ716464
CXXC6	-4.0	3.2	CXXC-type zinc finger protein 6 (Leukemia-associated protein with a CXXC domain). [Source:Uniprot/SWISSPROT;Acc:Q8NFU7] [ENST00000373644]	ENST0000037364	Unknown	
C15orf15	-4.0	1.9	chromosome 15 open reading frame 15 (C15orf15), mRNA [NM_016304]	NM_016304	Hs.274772	BF244626
TMEM170	-4.0	2.1	mRNA; cDNA DKFZp686O1555 (from clone DKFZp686O1555). [BX648484]	BX648484	Hs.487510	BX648484
PIK3C2B	-4.0	1.7	phosphoinositide-3-kinase, class 2, beta polypeptide (PIK3C2B), mRNA [NM_002646]	NM_002646	Hs.497487	Y11312
CENPQ	-4.0	2.3	centromere protein Q (CENPQ), mRNA [NM_018132]	NM_018132	Hs.88663	NM_018132
CREBBP	-4.0	2.0	CREB binding protein (Rubinstein-Taybi syndrome) (CREBBP), transcript variant 1, mRNA [NM_004380]	NM_004380	Hs.459759	NM_004380

C6orf162	-4.0	1.4	chromosome 6 open reading frame 162 (C6orf162), transcript variant 1, mRNA [NM_001042493]	NM_001042493	Hs.70769	NM_001042493
TRERF1	-4.0	2.2	transcriptional regulating factor 1 (TRERF1), mRNA [NM_033502]	NM_033502	Hs.485392	AM404182
KRT38	-4.0	1.4	keratin 38 (KRT38), mRNA [NM_006771]	NM_006771	Hs.248188	NM_006771
LOC200420	-4.0	2.4	LOC200420 (LOC200420), mRNA [NM_145300]	NM_145300	Unknown	
CR618523	-4.0	1.7	full-length cDNA clone CS0DI011YC23 of Placenta Cot 25-normalized of (human). [CR618523]	CR618523	Hs.585745	NM_001080530
PHOX2B	-4.0	1.6	paired-like homeobox 2b (PHOX2B), mRNA [NM_003924]	NM_003924	Hs.87202	NM_003924
KLHL14	-4.0	3.6	kelch-like 14 (Drosophila) (KLHL14), mRNA [NM_020805]	NM_020805	Hs.446164	AB037805
A_24_P99005	-4.0	2.6	A_24_P99005	A_24_P99005	Unknown	
MANEA	-4.0	2.1	mannosidase, endo-alpha (MANEA), mRNA [NM_024641]	NM_024641	Hs.533323	BC150199
THC2521656	-4.0	1.9	Q3KI19_PSEPF (Q3KI19) Nitrilase/cyanide hydratase and apolipoprotein N-acyltransferase, partial (5%) [THC2521656]	THC2521656	Unknown	
A_24_P27273	-4.0	1.9	A_24_P272735	A_24_P272735	Unknown	
KNTC1	-4.0	2.4	kinetochore associated 1 (KNTC1), mRNA [NM_014708]	NM_014708	Hs.300559	BC150278
THC2495785	-4.0	2.0	CN284574 17000531534200 GRN_EB cDNA 5', mRNA sequence [CN284574]	THC2495785	Unknown	
RPL6	-4.0	1.7	ribosomal protein L6 (RPL6), transcript variant 1, mRNA [NM_001024662]	NM_001024662	Hs.546283	BQ055135
THC2782843	-4.0	1.7	RL18A_HUMAN (Q02543) 60S ribosomal protein L18a, partial (89%) [THC2782843]	THC2782843	Unknown	
ZNF660	-4.0	2.1	zinc finger protein 660 (ZNF660), mRNA [NM_173658]	NM_173658	Hs.669324	BX538171
AK092378	-4.0	3.3	cDNA FLJ35059 fis, clone OCBBF2018827. [AK092378]	AK092378	Hs.592572	BC035116
ITGA9	-4.0	1.7	integrin, alpha 9 (ITGA9), mRNA [NM_002207]	NM_002207	Hs.113157	BX647350
FOXP1	-4.0	1.8	forkhead box P1 (FOXP1), transcript variant 1, mRNA [NM_032682]	NM_032682	Hs.431498	NM_032682
AA714537	-4.0	2.0	nw20g12.s1 NCI_CGAP_GCB0 cDNA clone IMAGE:1241062 3' similar to gb:M84711 40S RIBOSOMAL PROTEIN S3A (HUMAN);, mRNA sequence [AA714537]	AA714537	Hs.567702	BM911048
MEGF10	-4.0	2.8	multiple EGF-like-domains 10 (MEGF10), mRNA [NM_032446]	NM_032446	Hs.438709	CR749437
TGFBR3	-4.0	2.6	transforming growth factor, beta receptor III (betaglycan, 300kDa) (TGFBR3), mRNA [NM_003243]	NM_003243	Hs.482390	L07594
TULP4	-4.0	1.4	tubby like protein 4 (TULP4), transcript variant 1, mRNA [NM_020245]	NM_020245	Hs.486993	AF219946
LOC646791	-4.0	1.6	full-length cDNA clone CS0DI034YC12 of Placenta Cot 25-normalized of (human). [CR616535]	CR616535	Hs.631704	CR616535
SNED1	-4.0	3.4	mRNA for FLJ00133 protein. [AK074062]	AK074062	Hs.471834	BX640653
UPF3A	-4.0	1.5	UPF3 regulator of nonsense transcripts homolog A (yeast) (UPF3A), transcript variant 1, mRNA [NM_023011]	NM_023011	Hs.533855	AK092586
C1orf165	-4.0	1.8	chromosome 1 open reading frame 165 (C1orf165), mRNA [NM_024603]	NM_024603	Hs.475348	AK096573
MBIP	-4.0	2.1	MAP3K12 binding inhibitory protein 1 (MBIP), mRNA [NM_016586]	NM_016586	Hs.368647	AF208857
A_32_P14524	-4.0	1.9	A_32_P145241	A_32_P145241	Unknown	
CDK5RAP2	-4.0	1.7	CDK5 regulatory subunit associated protein 2 (CDK5RAP2), transcript variant 1, mRNA [NM_018249]	NM_018249	Hs.269560	BX640896
THC2540532	-4.0	1.7	THC2540532	THC2540532	Unknown	
THC2650514	-4.0	1.7	THC2650514	THC2650514	Unknown	
ARHGAP22	-4.0	5.3	Rho GTPase activating protein 22 (ARHGAP22), mRNA [NM_021226]	NM_021226	Hs.655672	BC047096
DMXL2	-4.0	2.0	Dmx-like 2 (DMXL2), mRNA [NM_015263]	NM_015263	Hs.511386	NM_015263
PATZ1	-4.0	2.1	POZ (BTB) and AT hook containing zinc finger 1 (PATZ1), transcript variant 1, mRNA [NM_014323]	NM_014323	Hs.517557	NM_014323
ZNF195	-4.0	1.5	zinc finger protein 195 (ZNF195), mRNA [NM_007152]	NM_007152	Hs.386294	AL833722
ENST0000036	-4.0	1.7	Roquin (RING finger and C3H zinc finger protein 1). [Source:Uniprot/SWISSPROT;Acc:Q5TC82] [ENST00000367696]	ENST0000036769	Unknown	
AK091508	-4.0	3.0	cDNA FLJ34189 fis, clone FCBBF3017535. [AK091508]	AK091508	Unknown	
ENST0000030	-4.0	2.8	PREDICTED: similar to ribosomal protein S15a (LOC728774), mRNA [XM_001129390]	ENST0000030401	Unknown	
LOC440104	-4.0	1.6	cDNA FLJ32617 fis, clone STOMA2000257. [AK057179]	AK057179	Hs.616500	XM_927712
THC2708803	-4.0	3.2	THC2708803	THC2708803	Unknown	
KIAA1922	-4.0	2.6	KIAA1922 protein, mRNA (cDNA clone IMAGE:2960715), complete cds. [BC033082]	BC033082	Hs.597881	AB067509
ZMYM2	-4.0	1.8	zinc finger, MYM-type 2 (ZMYM2), mRNA [NM_003453]	NM_003453	Hs.644041	BX648905
KNTC1	-4.0	2.4	kinetochore associated 1 (KNTC1), mRNA [NM_014708]	NM_014708	Hs.300559	BC150278
A_24_P54701	-4.0	1.4	A_24_P547010	A_24_P547010	Unknown	
YEATS2	-4.0	1.6	YEATS domain containing 2 (YEATS2), mRNA [NM_018023]	NM_018023	Hs.632575	BC150273
TMEM133	-4.0	3.1	transmembrane protein 133 (TMEM133), mRNA [NM_032021]	NM_032021	Hs.44004	AF247167
A_24_P83925	-4.0	4.4	A_24_P839258	A_24_P839258	Unknown	
IFT80	-4.0	1.7	intraflagellar transport 80 homolog (Chlamydomonas) (IFT80), mRNA [NM_020800]	NM_020800	Hs.478095	NM_020800
MGC24039	-4.0	1.6	hypothetical protein MGC24039, mRNA (cDNA clone IMAGE:4286826), complete cds. [BC020855]	BC020855	Hs.118166	NM_144973
PDIK1L	-4.0	2.1	PDLIM1 interacting kinase 1 like (PDIK1L), mRNA [NM_152835]	NM_152835	Hs.468801	AF411102

A_24_P93452	-4.0	2.1	A_24_P93452	A_24_P93452	Unknown	
LOC286161	-4.0	2.0	cDNA FLJ34353 fis, clone FEBRA2011665. [AK091672]	AK091672	Unknown	
FLRT2	-4.0	3.0	fibronectin leucine rich transmembrane protein 2 (FLRT2), mRNA [NM_013231]	NM_013231	Hs.533710	NM_013231
A_23_P12501	-4.0	2.4	A_23_P125016	A_23_P125016	Unknown	
TMEM117	-4.0	1.3	transmembrane protein 117 (TMEM117), mRNA [NM_032256]	NM_032256	Hs.444668	BC060798
ID2	-4.0	1.7	inhibitor of DNA binding 2, dominant negative helix-loop-helix protein (ID2), mRNA [NM_002166]	NM_002166	Hs.180919	CR623038
JOSD3	-4.0	1.6	Josephin domain containing 3 (JOSD3), mRNA [NM_024116]	NM_024116	Hs.355750	AK057183
ZNF195	-4.0	1.5	zinc finger protein 195 (ZNF195), mRNA [NM_007152]	NM_007152	Hs.386294	AL833722
NT5C3	-4.0	1.6	5'-nucleotidase, cytosolic III (NT5C3), transcript variant 2, mRNA [NM_001002009]	NM_001002009	Hs.487933	NM_001002009
PXK	-4.0	2.3	PX ser/thr kinase v2 mRNA, complete cds, alternatively spliced. [AY437879]	AY437879	Hs.190544	BX537600
ENST0000034	-4.0	1.7	full-length cDNA clone CS0DF038YM01 of Fetal brain of (human). [CR619663]	ENST0000034030	Unknown	
RANBP9	-4.0	1.3	RAN binding protein 9 (RANBP9), mRNA [NM_005493]	NM_005493	Hs.306242	BC052781
A_24_P24786	-4.0	2.3	A_24_P24786	A_24_P24786	Unknown	
LRBA	-4.0	1.8	LPS-responsive vesicle trafficking, beach and anchor containing (LRBA), mRNA [NM_006726]	NM_006726	Hs.480938	NM_006726
BI520341	-4.0	2.1	BI520341 603071814F1 NIH_MGC_119 cDNA clone IMAGE:5163794 5', mRNA sequence [BI520341]	BI520341	Unknown	
CHCHD6	-4.0	1.7	coiled-coil-helix-coiled-coil-helix domain containing 6 (CHCHD6), mRNA [NM_032343]	NM_032343	Hs.518119	BU146817
SERPINA1	-4.0	1.9	serpin peptidase inhibitor, clade A (alpha-1 antiproteinase, antitrypsin), member 1 (SERPINA1), transcript variant 2, mRNA [NM_001002236]	NM_001002236	Hs.525557	CR594071

Supplemental Table A4

Genes Upregulated in CM vs EB (1012)

SAM FDR 0.1%

Gene Name	Score(d)	Fold Upreg. CM	Description	Acc	UGCluster	UGRepAcc
PMCH	37.5	140.7	pro-melanin-concentrating hormone (PMCH), mRNA [NM_002674]	NM_002674	Hs.694729	BG216766
BC015449	36.2	47.3	Homo sapiens, clone IMAGE:4427279, mRNA. [BC015449]	BC015449	Hs.615028	BC015449
LMO7	27.2	3.5	LIM domain 7 (LMO7), mRNA [NM_005358]	NM_005358	Hs.207631	AF330045
ODZ2	21.1	3.8	Teneurin-2 (Ten-2) (Tenascin-M2) (Ten-m2) (Protein Odd Oz/ten-m homolog 2). [Source:Uniprot/SWISSPROT;Acc:Q9NT68] [ENST00000314238]	ENST00000314238	Unknown	
GDA	19.5	13.7	guanine deaminase (GDA), mRNA [NM_004293]	NM_004293	Hs.494163	NM_004293
DKK2	17.8	5.8	dickkopf homolog 2 (Xenopus laevis) (DKK2), mRNA [NM_014421]	NM_014421	Hs.211869	AF177395
CLIC5	17.2	4.6	chloride intracellular channel 5 (CLIC5), mRNA [NM_016929]	NM_016929	Hs.485489	DQ679794
LMO7	17.1	3.7	LIM domain 7 (LMO7), mRNA [NM_005358]	NM_005358	Hs.207631	AF330045
TMEM45B	16.7	8.3	transmembrane protein 45B (TMEM45B), mRNA [NM_138788]	NM_138788	Hs.504301	AK098106
PMCHL1	16.5	74.6	pro-melanin-concentrating hormone-like 1 protein (PMCHL1) mRNA, complete cds, alternatively spliced. [AY008410]	AY008410	Unknown	
ARL4C	15.2	3.9	ADP-ribosylation factor-like 4C (ARL4C), mRNA [NM_005737]	NM_005737	Hs.655990	NM_005737
ZIC4	14.9	12.3	zinc family member 4 protein HZIC4 (ZIC4) mRNA, complete cds. [AF332509]	AF332509	Unknown	
CALD1	13.8	2.9	caldesmon 1 (CALD1), transcript variant 1, mRNA [NM_033138]	NM_033138	Hs.490203	NM_033138
C18orf1	13.3	3.8	chromosome 18 open reading frame 1 (C18orf1), transcript variant a2, mRNA [NM_181482]	NM_181482	Hs.149363	NM_001003674
IDS	13.0	3.2	iduronate 2-sulfatase (Hunter syndrome) (IDS), transcript variant 2, mRNA [NM_006123]	NM_006123	Hs.460960	NM_000202
FLJ36032	13.0	2.4	cDNA FLJ36032 fis, clone TESTI2017069. [AK093351]	AK093351	Hs.297967	AK093351
SLC41A2	12.6	3.7	solute carrier family 41, member 2 (SLC41A2), mRNA [NM_032148]	NM_032148	Hs.577463	NM_032148
DSCR1	12.6	5.4	Down syndrome critical region gene 1 (DSCR1), transcript variant 1, mRNA [NM_004414]	NM_004414	Hs.282326	AY325903
TPM1	12.5	2.6	tropomyosin 1 (alpha) (TPM1), transcript variant 3, mRNA [NM_001018004]	NM_001018004	Hs.133892	BX648171
HIVEP3	12.5	2.5	human immunodeficiency virus type I enhancer binding protein 3 [Source:RefSeq_peptide;Acc:NP_078779] [ENST00000372583]	ENST00000372583	Unknown	
DUSP10	12.5	2.5	dual specificity phosphatase 10 (DUSP10), transcript variant 1, mRNA [NM_007207]	NM_007207	Hs.497822	BC031405
C8orf47	12.0	5.1	chromosome 8 open reading frame 47 (C8orf47), mRNA [NM_173549]	NM_173549	Hs.171455	NM_173549
FLJ46266	12.0	9.1	FLJ46266 protein (FLJ46266), mRNA [NM_207430]	NM_207430	Hs.411600	BM473494
LZTS1	11.9	6.5	leucine zipper, putative tumor suppressor 1 (LZTS1), mRNA [NM_021020]	NM_021020	Hs.521432	AF123659
ADRA1B	11.7	4.6	adrenergic, alpha-1B-, receptor (ADRA1B), mRNA [NM_000679]	NM_000679	Hs.368632	NM_000679
SYNGR2	11.7	3.2	synaptogyrin 2, mRNA (cDNA clone MGC:102914 IMAGE:4746277), complete cds. [BC105992]	BC105992	Hs.464210	BC000407
MYL6	11.6	3.7	myosin, light chain 6, alkali, smooth muscle and non-muscle (MYL6), transcript variant 2, mRNA [NM_079423]	NM_079423	Hs.632717	BM546656
CA3	11.5	3.9	carbonic anhydrase III, muscle specific (CA3), mRNA [NM_005181]	NM_005181	Hs.82129	NM_005181
MSX2P	11.5	1.9	msh homeobox 2 pseudogene (MSX2P) on chromosome 17 [NR_002307]	NR_002307	Unknown	
TRPM3	11.5	3.2	transient receptor potential cation channel, subfamily M, member 3 (TRPM3), transcript variant 9, mRNA [NM_001007471]	NM_001007471	Hs.47288	NM_001007471
A_32_P31206	11.4	5.0	A_32_P31206	A_32_P31206	Unknown	
TMEPAI	11.4	2.5	transmembrane, prostate androgen induced RNA (TMEPAI), transcript variant 1, mRNA [NM_020182]	NM_020182	Hs.517155	NM_020182
A_23_P16581	11.4	2.3	A_23_P165819	A_23_P165819	Unknown	
NEFL	11.2	2.3	neurofilament, light polypeptide 68kDa (NEFL), mRNA [NM_006158]	NM_006158	Hs.521461	NM_006158
MLLT11	11.1	2.2	myeloid/lymphoid or mixed-lineage leukemia (trithorax homolog, Drosophila); translocated to, 11 (MLLT11), mRNA [NM_006818]	NM_006818	Hs.75823	BC022448
C6orf85	11.0	2.7	chromosome 6 open reading frame 85, mRNA (cDNA clone IMAGE:3846727), complete cds. [BC022217]	BC022217	Hs.700628	NM_021945
CACNA2D3	10.7	2.9	voltage-gated calcium channel alpha(2)delta-3 subunit mRNA, complete cds. [AF516696]	AF516696	Hs.656687	NM_018398
CLIC6	10.6	11.2	chloride intracellular channel 6 (CLIC6), mRNA [NM_053277]	NM_053277	Hs.473695	AF448439
SOST	10.6	8.7	sclerosteosis (SOST), mRNA [NM_025237]	NM_025237	Hs.349204	AY358627
MFAP5	10.5	6.8	microfibrillar associated protein 5 (MFAP5), mRNA [NM_003480]	NM_003480	Hs.512842	NM_003480
AK022407	10.5	2.1	cDNA FLJ12345 fis, clone MAMMA1002294. [AK022407]	AK022407	Hs.636866	AK022407
EBI3	10.5	7.4	Epstein-Barr virus induced gene 3 (EBI3), mRNA [NM_005755]	NM_005755	Hs.501452	BM919170
TMEM2	10.4	2.6	transmembrane protein 2 (TMEM2), mRNA [NM_013390]	NM_013390	Hs.494146	AF137030
NOX4	10.4	2.8	NADPH oxidase 4 (NOX4), mRNA [NM_016931]	NM_016931	Hs.371036	AK057189
TAGLN	10.2	7.5	transgelin (TAGLN), transcript variant 1, mRNA [NM_001001522]	NM_001001522	Hs.632099	AB209555

RUNX1	10.2	4.1	runt-related transcription factor 1 (acute myeloid leukemia 1; aml1 oncogene) (RUNX1), transcript variant 2, mRNA [NM_001001890]	NM_001001890	Hs.149261	NM_001001890
PLAC2	10.1	8.2	placenta-specific 2 [Source:RefSeq_peptide;Acc:NP_699206] [ENST00000312785]	ENST00000312785	Unknown	
C10orf118	10.1	1.8	chromosome 10 open reading frame 118 (C10orf118), mRNA [NM_018017]	NM_018017	Hs.159066	NM_018017
ELN	10.1	2.9	cDNA PSEC0254 fis, clone NT2RP3003474, moderately similar to ELASTIN PRECURSOR. [AK075554]	AK075554	Hs.647061	NM_000501
S100A2	10.1	6.8	S100 calcium binding protein A2 (S100A2), mRNA [NM_005978]	NM_005978	Hs.516484	BU589956
SNTB1	10.0	4.1	syntrophin, beta 1 (dystrophin-associated protein A1, 59kDa, basic component 1) (SNTB1), mRNA [NM_021021]	NM_021021	Hs.655236	AK026095
THC2674391	10.0	3.1	ALU8_HUMAN (P39195) Alu subfamily SX sequence contamination warning entry, partial (8%) [THC2674391]	THC2674391	Unknown	
CCK	10.0	6.6	cholecystokinin (CCK), mRNA [NM_000729]	NM_000729	Hs.458426	BC111026
A_23_P14045	9.9	2.7	A_23_P140454	A_23_P140454	Unknown	
TES	9.9	2.5	testis derived transcript (3 LIM domains) (TES), transcript variant 2, mRNA [NM_152829]	NM_152829	Hs.592286	NM_015641
STK38L	9.8	4.2	serine/threonine kinase 38 like (STK38L), mRNA [NM_015000]	NM_015000	Hs.184523	AB023182
KIAA0574	9.7	2.4	mRNA for KIAA0574 protein, partial cds. [AB011146]	ENST00000261275	Unknown	
IL1A	9.7	12.5	interleukin 1, alpha (IL1A), mRNA [NM_000575]	NM_000575	Hs.1722	NM_000575
TPM1	9.6	3.1	tropomyosin 1 (alpha) (TPM1), transcript variant 5, mRNA [NM_000366]	NM_000366	Hs.133892	BX648171
IL1A	9.6	16.1	interleukin 1, alpha (IL1A), mRNA [NM_000575]	NM_000575	Hs.1722	NM_000575
HBEGF	9.5	4.0	heparin-binding EGF-like growth factor (HBEGF), mRNA [NM_001945]	NM_001945	Hs.799	BC033097
TMEM87B	9.5	2.8	transmembrane protein 87B (TMEM87B), mRNA [NM_032824]	NM_032824	Hs.656298	AK027587
TUBB2A	9.4	3.6	tubulin, beta 2A (TUBB2A), mRNA [NM_001069]	NM_001069	Hs.654543	BM470828
MYL6	9.3	3.3	myosin, light chain 6, alkali, smooth muscle and non-muscle (MYL6), transcript variant 2, mRNA [NM_079423]	NM_079423	Hs.632717	BM546656
AK057935	9.3	4.5	cDNA FLJ25206 fis, clone REC05955. [AK057935]	AK057935	Unknown	
TAGLN	9.2	4.6	transgelin (TAGLN), transcript variant 1, mRNA [NM_001001522]	NM_001001522	Hs.632099	AB209555
DPYSL3	9.2	5.1	dihydropyrimidinase-like 3 (DPYSL3), mRNA [NM_001387]	NM_001387	Hs.519659	BC077077
IER3	9.2	6.2	immediate early response 3 (IER3), mRNA [NM_003897]	NM_003897	Hs.591785	BM906591
BAIAP3	9.0	3.1	BAI1-associated protein 3 (BAIAP3), mRNA [NM_003933]	NM_003933	Hs.458427	AL834321
CTGF	8.9	6.0	connective tissue growth factor (CTGF), mRNA [NM_001901]	NM_001901	Hs.591346	NM_001901
TPM1	8.9	3.6	tropomyosin 1 (alpha) (TPM1), transcript variant 3, mRNA [NM_001018004]	NM_001018004	Hs.133892	BX648171
RP5-1054A22	8.9	2.8	KIAA1755 protein (KIAA1755), mRNA [NM_001029864]	NM_001029864	Hs.472690	NM_001029864
KITLG	8.9	4.1	KIT ligand (KITLG), transcript variant b, mRNA [NM_000899]	NM_000899	Hs.1048	NM_000899
ROPN1L	8.9	3.7	ropporin 1-like (ROPN1L), mRNA [NM_031916]	NM_031916	Hs.381089	BM563234
FILIP1L	8.9	4.2	filamin A interacting protein 1-like (FILIP1L), transcript variant 1, mRNA [NM_182909]	NM_182909	Hs.104672	NM_001042459
ENST0000029	8.8	2.5	F-box/LRR-repeat protein 18 (F-box and leucine-rich repeat protein 18). [Source:Uniprot/SWISSPROT;Acc:Q96ME1] [ENST00000297035]	ENST00000297035	Unknown	
OBFC2A	8.7	5.7	oligonucleotide/oligosaccharide-binding fold containing 2A (OBFC2A), mRNA [NM_001031716]	NM_001031716	Hs.591610	AL832659
LOC541471	8.7	5.6	cDNA FLJ10934 fis, clone OVARC1000640. [AK001796]	AK001796	Hs.652426	AK001796
CC2D1B	8.6	2.4	coiled-coil and C2 domain containing 1B (CC2D1B), mRNA [NM_032449]	NM_032449	Hs.591451	NM_032449
ENST0000036	8.6	4.4	Neurofascin precursor. [Source:Uniprot/SWISSPROT;Acc:O94856] [ENST00000367169]	ENST00000367169	Unknown	
LOC653364	8.6	3.9	PREDICTED: similar to Heat-shock protein beta-1 (HspB1) (Heat shock 27 kDa protein) (HSP 27) (Stress-responsive protein 27) (SRP27) (Estrogen-regulated 24 kDa protein) (28 kDa heat shock protein) (LOC653364), mRNA [XR_017030]	XR_017030	Hs.593381	BU539481
SLC26A9	8.6	5.3	solute carrier family 26, member 9 (SLC26A9), transcript variant 1, mRNA [NM_052934]	NM_052934	Hs.164073	AF331525
SHC3	8.6	3.1	SHC (Src homology 2 domain containing) transforming protein 3 (SHC3), mRNA [NM_016848]	NM_016848	Hs.656806	BX641139
EHD1	8.6	2.4	EH-domain containing 1 (EHD1), mRNA [NM_006795]	NM_006795	Hs.523774	AK124613
UGP2	8.6	2.0	UDP-glucose pyrophosphorylase 2 (UGP2), transcript variant 1, mRNA [NM_006759]	NM_006759	Hs.516217	BX537559
ZMYND10	8.5	2.6	zinc finger, MYND-type containing 10 (ZMYND10), mRNA [NM_015896]	NM_015896	Hs.526735	AK096525
ATP6V1D	8.5	2.1	ATPase, H+ transporting, lysosomal 34kDa, V1 subunit D (ATP6V1D), mRNA [NM_015994]	NM_015994	Hs.272630	AK024101
DKK2	8.5	4.9	dickkopf homolog 2 (Xenopus laevis) (DKK2), mRNA [NM_014421]	NM_014421	Hs.211869	AF177395
UPP1	8.4	6.9	uridine phosphorylase 1 (UPP1), transcript variant 2, mRNA [NM_181597]	NM_181597	Hs.488240	BC007348
MGC4677	8.4	3.7	hypothetical protein MGC4677 (MGC4677), mRNA [NM_052871]	NM_052871	Unknown	
PALLD	8.4	2.3	palladin, cytoskeletal associated protein (PALLD), mRNA [NM_016081]	NM_016081	Hs.151220	NM_016081
LOC497190	8.3	4.5	secretory protein LOC497190 (LOC497190), mRNA [NM_001011880]	NM_001011880	Hs.556045	AL833339
ALS2CL	8.3	4.0	ALS2 C-terminal like (ALS2CL), transcript variant 1, mRNA [NM_147129]	NM_147129	Hs.517937	CR627258
THRB	8.3	4.7	thyroid hormone receptor, beta (erythroblastic leukemia viral (v-erb-a) oncogene homolog 2, avian) (THRB), mRNA [NM_000461]	NM_000461	Hs.187861	NM_000461

NEGR1	8.3	6.9	neuronal growth regulator 1 (NEGR1), mRNA [NM_173808]	NM_173808	Hs.146542	NM_173808
CDH2	8.3	2.5	cadherin 2, type 1, N-cadherin (neuronal) (CDH2), mRNA [NM_001792]	NM_001792	Hs.464829	S42303
DDR1	8.3	2.7	discoidin domain receptor family, member 1 (DDR1), transcript variant 1, mRNA [NM_013993]	NM_013993	Hs.631988	NM_013993
TMEM2	8.3	3.5	transmembrane protein 2 (TMEM2), mRNA [NM_013390]	NM_013390	Hs.494146	AF137030
CNN3	8.3	2.0	calponin 3, acidic (CNN3), mRNA [NM_001839]	NM_001839	Hs.700591	NM_001839
DNAJC15	8.2	3.0	DnaJ (Hsp40) homolog, subfamily C, member 15 (DNAJC15), mRNA [NM_013238]	NM_013238	Hs.438830	NM_013238
OBFC2A	8.2	3.6	oligonucleotide/oligosaccharide-binding fold containing 2A (OBFC2A), mRNA [NM_001031716]	NM_001031716	Hs.591610	AL832659
AK055621	8.2	13.2	cDNA FLJ31059 fis, clone HSYRA2000832. [AK055621]	AK055621	Hs.583755	AK055621
SH3TC2	8.2	2.3	SH3 domain and tetratricopeptide repeats 2 (SH3TC2), mRNA [NM_024577]	NM_024577	Hs.483784	NM_024577
CHST7	8.2	3.8	carbohydrate (N-acetylglucosamine 6-O) sulfotransferase 7 (CHST7), mRNA [NM_019886]	NM_019886	Hs.129955	NM_019886
MRCL3	8.2	3.6	myosin regulatory light chain MRCL3 (MRCL3), mRNA [NM_006471]	NM_006471	Hs.190086	BF694761
TMEM30A	8.2	2.1	transmembrane protein 30A (TMEM30A), mRNA [NM_018247]	NM_018247	Hs.108530	NM_018247
C9orf24	8.1	5.8	chromosome 9 open reading frame 24 (C9orf24), transcript variant 1, mRNA [NM_032596]	NM_032596	Hs.50334	BM805429
PIK3CD	8.1	4.5	phosphoinositide-3-kinase, catalytic, delta polypeptide (PIK3CD), mRNA [NM_005026]	NM_005026	Hs.518451	NM_005026
YBX1	8.1	2.0	Y box binding protein 1 (YBX1), mRNA [NM_004559]	NM_004559	Hs.473583	BM907843
FBXO2	8.1	2.9	F-box protein 2 (FBXO2), mRNA [NM_012168]	NM_012168	Hs.132753	NM_012168
IL1A	8.0	15.4	interleukin 1, alpha (IL1A), mRNA [NM_000575]	NM_000575	Hs.1722	NM_000575
RAI2	8.0	2.4	retinoic acid induced 2 (RAI2), mRNA [NM_021785]	NM_021785	Hs.701485	NM_021785
YBX1	8.0	2.1	Y box binding protein 1 (YBX1), mRNA [NM_004559]	NM_004559	Hs.473583	BM907843
PLAC2	8.0	7.2	placenta-specific 2 (PLAC2), mRNA [NM_153375]	NM_153375	Unknown	
RAB6B	8.0	3.3	RAB6B, member RAS oncogene family (RAB6B), mRNA [NM_016577]	NM_016577	Hs.12152	NM_016577
NOX4	8.0	2.8	NADPH oxidase 4 (NOX4), mRNA [NM_016931]	NM_016931	Hs.371036	AK057189
CCDC81	8.0	3.7	coiled-coil domain containing 81 (CCDC81), mRNA [NM_021827]	NM_021827	Hs.144913	BC126412
REXO2	8.0	2.2	REX2, RNA exonuclease 2 homolog (S. cerevisiae) (REXO2), mRNA [NM_015523]	NM_015523	Hs.7527	BQ226060
CALM2	8.0	2.0	calmodulin 2 (phosphorylase kinase, delta) (CALM2), mRNA [NM_001743]	NM_001743	Hs.643483	BG110202
AGRIN	7.9	2.5	agrin (AGRIN), mRNA [NM_198576]	NM_198576	Hs.273330	AB191264
S100A6	7.9	7.2	S100 calcium binding protein A6 (S100A6), mRNA [NM_014624]	NM_014624	Hs.275243	BM904612
ODF2	7.9	2.0	outer dense fiber of sperm tails 2 (ODF2), transcript variant 2, mRNA [NM_153437]	NM_153437	Hs.129055	NM_002540
DOCK8	7.9	3.3	dedicator of cytokinesis 8 (DOCK8), mRNA [NM_203447]	NM_203447	Hs.132599	AL832270
ARF4	7.9	1.9	ADP-ribosylation factor 4 (ARF4), mRNA [NM_001660]	NM_001660	Hs.695943	BC016325
HABP4	7.9	3.0	hyaluronan binding protein 4 (HABP4), mRNA [NM_014282]	NM_014282	Hs.494567	AK091242
SPG3A	7.9	2.3	spastic paraplegia 3A (autosomal dominant) (SPG3A), transcript variant 2, mRNA [NM_181598]	NM_181598	Hs.584905	AF444143
ENDOD1	7.8	2.9	Endonuclease domain-containing 1 protein precursor (EC 3.1.30.-). [Source:Uniprot/SWISSPROT;Acc:O94919] [ENST00000278505]	ENST00000278505	Unknown	
SORT1	7.8	3.4	sortilin 1 (SORT1), mRNA [NM_002959]	NM_002959	Hs.485195	NM_002959
ENST0000030	7.7	3.6	LOC541471 protein (Hypothetical protein MGC4677). [Source:Uniprot/SPTREMBL;Acc:Q96C48] [ENST00000308604]	ENST00000308604	Unknown	
ATP6V1C1	7.7	2.4	ATPase, H+ transporting, lysosomal 42kDa, V1 subunit C1 (ATP6V1C1), transcript variant 1, mRNA [NM_001695]	NM_001695	Hs.86905	NM_001695
CR740121	7.7	14.5	CR740121 library (Ebert L) cDNA clone IMAGp971G1750 ; IMAGE:767753 5', mRNA sequence [CR740121]	CR740121	Hs.85989	CR740121
LOC649545	7.7	2.7	PREDICTED: similar to Keratin, type II cytoskeletal 8 (Cytokeratin-8) (CK-8) (Keratin-8) (K8) (LOC649545), mRNA [XR_018641]	XR_018641	Hs.693340	XR_018641
TTLL7	7.7	2.7	tubulin tyrosine ligase-like family, member 7 (TTLL7), mRNA [NM_024686]	NM_024686	Hs.445826	NM_024686
UQCRQ	7.7	2.0	ubiquinol-cytochrome c reductase, complex III subunit VII, 9.5kDa (UQCRQ), nuclear gene encoding mitochondrial protein, mRNA [NM_014402]	NM_014402	Hs.146602	NM_014402
KCNMB1	7.7	3.5	potassium large conductance calcium-activated channel, subfamily M, beta member 1 (KCNMB1), mRNA [NM_004137]	NM_004137	Hs.484099	BC025707
THC2618235	7.7	4.6	Q4IUS1_AZOVI (Q4IUS1) Aspartate racemase , partial (4%) [THC2618235]	THC2618235	Unknown	
LACTB	7.7	2.4	lactamase, beta (LACTB), nuclear gene encoding mitochondrial protein, transcript variant 2, mRNA [NM_171846]	NM_171846	Hs.410388	BC067288
EGF	7.7	6.6	epidermal growth factor (beta-urogastrone) (EGF), mRNA [NM_001963]	NM_001963	Hs.419815	NM_001963
CX3CL1	7.6	5.7	chemokine (C-X3-C motif) ligand 1 (CX3CL1), mRNA [NM_002996]	NM_002996	Hs.531668	AB209037
FLJ20160	7.6	3.2	FLJ20160 protein (FLJ20160), mRNA [NM_017694]	NM_017694	Hs.699926	NM_017694
OPTN	7.6	7.1	optineurin (OPTN), transcript variant 1, mRNA [NM_001008211]	NM_001008211	Hs.699222	NM_001008211
RAB18	7.6	2.0	RAB18, member RAS oncogene family (RAB18), mRNA [NM_021252]	NM_021252	Hs.406799	AB209698
AXUD1	7.6	3.9	AXIN1 up-regulated 1 (AXUD1), mRNA [NM_033027]	NM_033027	Hs.370950	NM_033027
CCND1	7.6	3.3	cyclin D1 (CCND1), mRNA [NM_053056]	NM_053056	Hs.523852	NM_053056

SYNGR2	7.6	4.1	synaptogyrin 2 (SYNGR2), mRNA [NM_004710]	NM_004710	Hs.464210	BC000407
TAPBP	7.6	2.8	TAP binding protein (tapasin) (TAPBP), transcript variant 1, mRNA [NM_003190]	NM_003190	Hs.370937	NM_003190
BX352604	7.6	4.1	BX352604 B CELLS (RAMOS CELL LINE) COT 25-NORMALIZED cDNA clone CS0DL006YE02 3-PRIME, mRNA sequence [BX352604]	BX352604	Hs.568876	BX352604
FOSL2	7.6	2.8	Fos-related antigen 2. [Source:Uniprot/SWISSPROT;Acc:P15408] [ENST00000379616]	ENST00000379616	Unknown	
SIRT7	7.6	3.6	sirtuin (silent mating type information regulation 2 homolog) 7 (S. cerevisiae) (SIRT7), mRNA [NM_016538]	NM_016538	Hs.514636	XR_017531
TEX261	7.6	1.7	testis expressed sequence 261 (TEX261), mRNA [NM_144582]	NM_144582	Hs.516087	NM_144582
LOC402429	7.5	2.3	PREDICTED: similar to Keratin, type II cytoskeletal 8 (Cytokeratin-8) (CK-8) (Keratin-8) (K8) (LOC402429), mRNA [XR_018415]	XR_018415	Hs.648362	XR_018415
CCND1	7.5	3.3	cyclin D1 (CCND1), mRNA [NM_053056]	NM_053056	Hs.523852	NM_053056
YBX1	7.5	1.9	Y box binding protein 1 (YBX1), mRNA [NM_004559]	NM_004559	Hs.473583	BM907843
THC2718172	7.5	18.2	THC2718172	THC2718172	Unknown	
CCND1	7.5	3.2	cyclin D1 (CCND1), mRNA [NM_053056]	NM_053056	Hs.523852	NM_053056
PRSS23	7.5	5.0	protease, serine, 23 (PRSS23), mRNA [NM_007173]	NM_007173	Hs.25338	AL832007
PTPN3	7.5	2.2	protein tyrosine phosphatase, non-receptor type 3 (PTPN3), mRNA [NM_002829]	NM_002829	Hs.436429	BX648253
CCND1	7.5	3.1	cyclin D1 (CCND1), mRNA [NM_053056]	NM_053056	Hs.523852	NM_053056
ELN	7.5	13.2	elastin (supravalvular aortic stenosis, Williams-Beuren syndrome), mRNA (cDNA clone MGC:70763 IMAGE:6153564), complete cds. [BC065566]	BC065566	Hs.647061	NM_000501
SERTAD1	7.5	2.7	SERTA domain containing 1 (SERTAD1), mRNA [NM_013376]	NM_013376	Hs.269898	AK074652
TSPAN13	7.5	2.5	tetraspanin 13 (TSPAN13), mRNA [NM_014399]	NM_014399	Hs.364544	AK128509
KIFC3	7.5	3.8	kinesin family member C3 (KIFC3), mRNA [NM_005550]	NM_005550	Hs.23131	NM_005550
HSPB1	7.4	3.6	heat shock 27kDa protein 1 (HSPB1), mRNA [NM_001540]	NM_001540	Hs.520973	BM454532
LOC342419	7.4	2.2	PREDICTED: similar to Keratin, type II cytoskeletal 8 (Cytokeratin-8) (CK-8) (Keratin-8) (K8) (Cytokeratin endo A) (LOC342419), mRNA [XR_017139]	XR_017139	Hs.647373	XR_017139
RP11-217H1.1	7.4	1.7	implantation-associated protein (DKFZp564K142), mRNA [NM_032121]	NM_032121	Hs.323562	BC060842
SHB	7.4	2.1	Src homology 2 domain containing adaptor protein B (SHB), mRNA [NM_003028]	NM_003028	Hs.521482	NM_003028
CCND1	7.4	3.2	cyclin D1 (CCND1), mRNA [NM_053056]	NM_053056	Hs.523852	NM_053056
SPATA13	7.4	1.4	spermatogenesis associated 13 (SPATA13), mRNA [NM_153023]	NM_153023	Hs.657121	BX648244
FOSL2	7.4	3.2	FOS-like antigen 2 (FOSL2), mRNA [NM_005253]	NM_005253	Hs.220971	BX647822
HGS	7.4	2.3	cDNA FLJ39878 fis, clone SPLEN2016045, moderately similar to mRNA for Hrs. [AK097197]	AK097197	Hs.514590	AK097197
GNG12	7.3	2.3	guanine nucleotide binding protein (G protein), gamma 12 (GNG12), mRNA [NM_018841]	NM_018841	Hs.700661	NM_018841
LOC90835	7.3	2.1	hypothetical protein LOC90835 (LOC90835), mRNA [NM_001014979]	NM_001014979	Hs.65735	AL122109
FKBP2	7.3	2.0	FK506 binding protein 2, 13kDa (FKBP2), transcript variant 1, mRNA [NM_004470]	NM_004470	Hs.227729	CB993963
BMP7	7.3	1.6	Bone morphogenetic protein 7 precursor (BMP-7) (Osteogenic protein 1) (OP-1) (Eptotermin alfa). [Source:Uniprot/SWISSPROT;Acc:P18075] [ENST00000371291]	ENST00000371291	Unknown	
DLSTP	7.3	2.4	E2k=alpha-ketoglutarate dehydrogenase complex dihydrolipoyl succinyltransferase [human, fetal brain, mRNA, 2987 nt]. [S72422]	S72422	Unknown	
CCND1	7.3	3.2	cyclin D1 (CCND1), mRNA [NM_053056]	NM_053056	Hs.523852	NM_053056
AGTRAP	7.3	2.2	angiotensin II receptor-associated protein (AGTRAP), transcript variant 4, mRNA [NM_001040196]	NM_001040196	Hs.464438	BQ422937
HSPB1	7.3	3.3	heat shock 27kDa protein 1 (HSPB1), mRNA [NM_001540]	NM_001540	Hs.520973	BM454532
PCGF5	7.3	2.1	polycomb group ring finger 5 (PCGF5), mRNA [NM_032373]	NM_032373	Hs.500512	NM_032373
DIRAS2	7.3	4.6	DIRAS family, GTP-binding RAS-like 2 (DIRAS2), mRNA [NM_017594]	NM_017594	Hs.165636	NM_017594
RAB3IP	7.3	1.7	RAB3A interacting protein (rabin3) (RAB3IP), transcript variant alpha 2, mRNA [NM_175623]	NM_175623	Hs.258209	NM_175623
THC2501739	7.3	38.4	Q47MR6_THEFY (Q47MR6) Uroporphyrin-III C-methyltransferase, C-terminal:Siroheme synthase, N-terminal, partial (5%) [THC2501739]	THC2501739	Unknown	
COL12A1	7.2	5.4	collagen, type XII, alpha 1 (COL12A1), transcript variant long, mRNA [NM_004370]	NM_004370	Hs.101302	NM_004370
CCND1	7.2	3.3	cyclin D1 (CCND1), mRNA [NM_053056]	NM_053056	Hs.523852	NM_053056
ENST0000038	7.2	3.1	PREDICTED: similar to myosin regulatory light chain-like (LOC649855), mRNA [XR_019636]	ENST00000383061	Unknown	
WSB2	7.2	2.4	WD repeat and SOCS box-containing 2 (WSB2), mRNA [NM_018639]	NM_018639	Hs.506985	NM_018639
RRP12	7.2	3.2	ribosomal RNA processing 12 homolog (S. cerevisiae) (RRP12), mRNA [NM_015179]	NM_015179	Hs.434251	AK022496
IER5	7.2	3.6	immediate early response 5 (IER5), mRNA [NM_016545]	NM_016545	Hs.15725	NM_016545
GADD45B	7.2	3.5	growth arrest and DNA-damage-inducible, beta (GADD45B), mRNA [NM_015675]	NM_015675	Hs.110571	AF087853
PCSK1N	7.2	3.2	proprotein convertase subtilisin/kexin type 1 inhibitor (PCSK1N), mRNA [NM_013271]	NM_013271	Hs.522640	BM805628
CCNL2	7.2	3.0	cyclin L2 (CCNL2), transcript variant 2, mRNA [NM_001039577]	NM_001039577	Hs.515704	NM_001039577
TTC9	7.2	2.8	Tetratricopeptide repeat protein 9 (TPR repeat protein 9) (Fragment). [Source:Uniprot/SWISSPROT;Acc:Q92623] [ENST00000256367]	ENST00000256367	Unknown	
PDE8B	7.2	1.6	phosphodiesterase 8B (PDE8B), transcript variant 1, mRNA [NM_003719]	NM_003719	Hs.584830	NM_003719
MRLC2	7.2	2.5	myosin regulatory light chain MRLC2 (MRLC2), mRNA [NM_033546]	NM_033546	Hs.464472	BQ067233

TUBB6	7.2	3.2	tubulin, beta 6 (TUBB6), mRNA [NM_032525]	NM_032525	Hs.193491	AK092677
CX3CL1	7.2	3.4	chemokine (C-X3-C motif) ligand 1 (CX3CL1), mRNA [NM_002996]	NM_002996	Hs.531668	AB209037
PTRF	7.2	8.0	polymerase I and transcript release factor (PTRF), mRNA [NM_012232]	NM_012232	Hs.437191	BC066123
FER1L3	7.1	4.9	fer-1-like 3, myoferlin (C. elegans) (FER1L3), transcript variant 2, mRNA [NM_133337]	NM_133337	Hs.655278	AF182316
ZDHHC2	7.1	2.7	zinc finger, DHHC-type containing 2 (ZDHHC2), mRNA [NM_016353]	NM_016353	Hs.443852	AL833080
NES	7.1	3.1	nestin (NES), mRNA [NM_006617]	NM_006617	Hs.527971	NM_006617
TNFAIP1	7.1	2.2	tumor necrosis factor, alpha-induced protein 1 (endothelial) (TNFAIP1), mRNA [NM_021137]	NM_021137	Hs.76090	BC003694
CCND1	7.1	3.3	cyclin D1 (CCND1), mRNA [NM_053056]	NM_053056	Hs.523852	NM_053056
LIF	7.1	5.6	leukemia inhibitory factor (cholinergic differentiation factor) (LIF), mRNA [NM_002309]	NM_002309	Hs.2250	NM_002309
SERINC2	7.1	2.2	serine incorporator 2 (SERINC2), mRNA [NM_178865]	NM_178865	Hs.270655	AK093784
LOC392288	7.1	2.6	PREDICTED: similar to microtubule-associated proteins 1A [XM_373277]	XM_373277	Unknown	
UGCG	7.0	1.9	Ceramide glucosyltransferase (EC 2.4.1.80) (Glucosylceramide synthase) (GCS) (UDP-glucose:N-acylsphingosine D-glucosyltransferase) (UDP- glucose ceramide glucosyltransferase) (GLCT-1). [Source:Uniprot/SWISSPROT;Acc:Q16739] [ENST00000374279]	ENST00000374279	Unknown	
LOC441768	7.0	2.7	PREDICTED: similar to RAB31, member RAS oncogene family (LOC441768), mRNA [XR_019199]	XR_019199	Hs.650966	XR_019199
RHOH	7.0	2.7	ras homolog gene family, member H (RHOH), mRNA [NM_004310]	NM_004310	Hs.694951	NM_004310
IDS	7.0	3.6	iduronate 2-sulfatase (Hunter syndrome) (IDS), transcript variant 2, mRNA [NM_006123]	NM_006123	Hs.460960	NM_000202
A_24_P24663	7.0	2.1	A_24_P246636	A_24_P246636	Unknown	
PRMT2	7.0	2.4	protein arginine methyltransferase 2 (PRMT2), transcript variant 1, mRNA [NM_206962]	NM_206962	Hs.154163	AK123352
TMEM49	7.0	2.7	transmembrane protein 49 (TMEM49), mRNA [NM_030938]	NM_030938	Hs.444569	BC053563
KIAA1244	6.9	2.7	KIAA1244 (KIAA1244), mRNA [NM_020340]	NM_020340	Hs.656215	NM_020340
MAP1LC3B	6.9	2.7	microtubule-associated protein 1 light chain 3 beta (MAP1LC3B), mRNA [NM_022818]	NM_022818	Hs.356061	BC041874
C20orf102	6.9	3.2	chromosome 20 open reading frame 102 (C20orf102), mRNA [NM_080607]	NM_080607	Hs.517029	NM_080607
ARPC5	6.9	2.0	actin related protein 2/3 complex, subunit 5, 16kDa (ARPC5), mRNA [NM_005717]	NM_005717	Hs.518609	BC034231
LOC644936	6.9	2.4	cDNA clone MGC:102982 IMAGE:30378151, complete cds. [BC092424]	BC092424	Hs.627173	BC092424
TMEM142B	6.9	3.3	transmembrane protein 142B (TMEM142B), mRNA [NM_032831]	NM_032831	Hs.363308	AK056497
C6orf165	6.9	1.9	CMP-sialic acid transporter (CMP-Sia-Tr) (CMP-SA-Tr) (Solute carrier family 35 member A1). [Source:Uniprot/SWISSPROT;Acc:P78382] [ENST00000369562]	ENST00000369562	Unknown	
C1orf151	6.8	1.8	chromosome 1 open reading frame 151 (C1orf151), mRNA [NM_001032363]	NM_001032363	Hs.466662	NM_001032363
GSG1	6.8	5.6	germ cell associated 1 (GSG1), transcript variant 1, mRNA [NM_031289]	NM_031289	Hs.240053	AK075322
TMEM50B	6.8	2.2	transmembrane protein 50B (TMEM50B), mRNA [NM_006134]	NM_006134	Hs.433668	NM_006134
THC2569153	6.8	7.2	Q804E3_GASAC (Q804E3) Neuronal 22 protein (Fragment), partial (18%) [THC2569153]	THC2569153	Unknown	
FAM38A	6.8	3.3	family with sequence similarity 38, member A (FAM38A), mRNA [NM_014745]	NM_014745	Hs.513807	BC150271
SSR4	6.8	1.7	signal sequence receptor, delta (translocon-associated protein delta) (SSR4), mRNA [NM_006280]	NM_006280	Hs.409223	AK057117
SP140	6.8	3.4	SP140 nuclear body protein (SP140), transcript variant 1, mRNA [NM_007237]	NM_007237	Hs.632549	U36500
MYH9	6.8	3.0	myosin, heavy chain 9, non-muscle (MYH9), mRNA [NM_002473]	NM_002473	Hs.474751	NM_002473
CCND1	6.8	3.2	cyclin D1 (CCND1), mRNA [NM_053056]	NM_053056	Hs.523852	NM_053056
UHMK1	6.8	1.9	Serine/threonine-protein kinase Kist (EC 2.7.11.1) (Kinase interacting with stathmin) (U2AF homology motif kinase 1). [Source:Uniprot/SWISSPROT;Acc:Q8TAS1] [ENST00000282169]	ENST00000282169	Unknown	
TMEM30A	6.8	2.3	transmembrane protein 30A (TMEM30A), mRNA [NM_018247]	NM_018247	Hs.108530	NM_018247
THC2550463	6.8	3.4	Q2KAS9_RHIEC (Q2KAS9) Probable two-component sensor histidine kinase protein, partial (4%) [THC2550463]	THC2550463	Unknown	
ENST0000033	6.8	4.0	Thymosin-like 4. [Source:Uniprot/SPTREMBL;Acc:Q5T4B6] [ENST00000334647]	ENST00000334647	Unknown	
TPM2	6.8	2.0	tropomyosin 2 (beta) (TPM2), transcript variant 2, mRNA [NM_213674]	NM_213674	Hs.300772	CR590682
BC040870	6.8	33.3	Homo sapiens, clone IMAGE:5583725, mRNA. [BC040870]	BC040870	Hs.103070	BC040870
CDH6	6.7	6.7	Cadherin-6 precursor (Kidney-cadherin) (K-cadherin). [Source:Uniprot/SWISSPROT;Acc:P55285] [ENST00000382216]	ENST00000382216	Unknown	
THC2657193	6.7	29.9	RASH_MSVHA (P01115) Transforming protein p29 precursor [Contains: Transforming protein p21], partial (6%) [THC2657193]	THC2657193	Unknown	
IDS	6.7	2.8	iduronate 2-sulfatase (Hunter syndrome) (IDS), transcript variant 1, mRNA [NM_000202]	NM_000202	Hs.460960	NM_000202
TUBA1	6.7	9.4	tubulin, alpha 1 (TUBA1), mRNA [NM_006000]	NM_006000	Hs.75318	AK054731
PTGFRN	6.7	1.9	prostaglandin F2 receptor negative regulator (PTGFRN), mRNA [NM_020440]	NM_020440	Hs.418093	NM_020440
TMSL3	6.7	3.5	thymosin-like 3 (TMSL3), mRNA [NM_183049]	NM_183049	Hs.555895	BU507525
TMEM165	6.7	2.0	transmembrane protein 165 (TMEM165), mRNA [NM_018475]	NM_018475	Hs.479766	BC107582
FLII	6.7	2.5	flightless I homolog (Drosophila) (FLII), mRNA [NM_002018]	NM_002018	Hs.513984	NM_002018

CYCS	6.7	1.7	cytochrome c, somatic (CYCS), nuclear gene encoding mitochondrial protein, mRNA [NM_018947]	NM_018947	Hs.437060	NM_018947
HSP90AA1	6.7	1.4	heat shock protein 90kDa alpha (cytosolic), class A member 1 (HSP90AA1), transcript variant 2, mRNA [NM_005348]	NM_005348	Hs.525600	NM_001017963
INHBB	6.7	5.6	inhibin, beta B (activin AB beta polypeptide) (INHBB), mRNA [NM_002193]	NM_002193	Hs.1735	NM_002193
JTB	6.7	2.2	jumping translocation breakpoint (JTB), mRNA [NM_006694]	NM_006694	Hs.6396	BQ062593
ADAM9	6.7	2.2	ADAM metallopeptidase domain 9 (meltrin gamma) (ADAM9), transcript variant 2, mRNA [NM_001005845]	NM_001005845	Hs.591852	NM_003816
BQ130701	6.6	4.1	BQ130701 ij86c06.y1 Human insulinoma cDNA clone IMAGE:5778418 5' similar to SW:CTGF_HUMAN P29279 CONNECTIVE TISSUE GROWTH FACTOR PRECURSOR. [1] ;, mRNA sequence [BQ130701]	BQ130701	Hs.591346	NM_001901
CCND1	6.6	3.2	cyclin D1 (CCND1), mRNA [NM_053056]	NM_053056	Hs.523852	NM_053056
SHCBP1	6.6	3.0	SHC SH2-domain binding protein 1 (SHCBP1), mRNA [NM_024745]	NM_024745	Hs.123253	BC030699
HSP90AA1	6.6	1.4	heat shock protein 90kDa alpha (cytosolic), class A member 1 (HSP90AA1), transcript variant 2, mRNA [NM_005348]	NM_005348	Hs.525600	NM_001017963
SLC7A6	6.6	2.9	solute carrier family 7 (cationic amino acid transporter, y+ system), member 6 (SLC7A6), transcript variant 2, mRNA [NM_001076785]	NM_001076785	Hs.700614	NM_001076785
CYB561	6.6	2.6	cytochrome b-561 (CYB561), transcript variant 2, mRNA [NM_001017916]	NM_001017916	Hs.355264	AK095244
LOC347333	6.6	2.2	PREDICTED: similar to Keratin, type II cytoskeletal 8 (Cytokeratin-8) (CK-8) (Keratin-8) (K8) (LOC347333), mRNA [XR_018299]	XR_018299	Hs.650946	XR_018299
GLIPR1	6.6	6.7	GLI pathogenesis-related 1 (glioma) (GLIPR1), mRNA [NM_006851]	NM_006851	Hs.205558	NM_006851
AY358690	6.6	3.2	clone DNA62876 LPPA601 (UNQ601) mRNA, complete cds. [AY358690]	AY358690	Hs.686554	AY358690
ENST0000037	6.6	2.4	Actin-like protein (Fragment). [Source:Uniprot/SPTREMBL;Acc:Q562T7] [ENST00000375923]	ENST00000375923	Unknown	
ACTB	6.6	2.8	actin, beta (ACTB), mRNA [NM_001101]	NM_001101	Hs.520640	AK125561
KRT8	6.6	2.3	keratin 8 (KRT8), mRNA [NM_002273]	NM_002273	Hs.533782	CR607281
TPM2	6.6	2.7	tropomyosin 2 (beta) (TPM2), transcript variant 2, mRNA [NM_213674]	NM_213674	Hs.300772	CR590682
PRDX4	6.6	1.8	peroxiredoxin 4 (PRDX4), mRNA [NM_006406]	NM_006406	Hs.83383	CD579519
REXO2	6.6	2.3	REX2, RNA exonuclease 2 homolog (S. cerevisiae) (REXO2), mRNA [NM_015523]	NM_015523	Hs.7527	BQ226060
JAZF1	6.5	2.4	JAZF zinc finger 1 (JAZF1), mRNA [NM_175061]	NM_175061	Hs.368944	NM_175061
LMX1A	6.5	1.9	LIM homeobox transcription factor 1, alpha (LMX1A), transcript variant 1, mRNA [NM_177398]	NM_177398	Hs.667312	AK127724
MUS81	6.5	1.8	MUS81 endonuclease homolog (S. cerevisiae) (MUS81), mRNA [NM_025128]	NM_025128	Unknown	
MAFB	6.5	3.0	v-maf musculoaponeurotic fibrosarcoma oncogene homolog B (avian) (MAFB), mRNA [NM_005461]	NM_005461	Hs.651210	NM_005461
GRAMD3	6.5	4.4	GRAM domain containing 3 (GRAMD3), mRNA [NM_023927]	NM_023927	Hs.363558	AK024966
ZMYM6	6.5	2.3	zinc finger, MYM-type 6, mRNA (cDNA clone IMAGE:4041437), complete cds. [BC007070]	BC007070	Hs.533986	AL833263
TLN1	6.5	2.8	talin 1 (TLN1), mRNA [NM_006289]	NM_006289	Hs.471014	AB028950
RP11-217H1.1	6.5	1.9	implantation-associated protein (DKFZp564K142), mRNA [NM_032121]	NM_032121	Hs.323562	BC060842
WHDC1	6.5	1.6	cDNA FLJ44939 fis, clone BRAMY3018754, weakly similar to Mus musculus junction-mediating and regulatory protein (Jmy-pending). [AK126887]	AK126887	Hs.377360	NM_001080435
MGC14376	6.5	2.5	hypothetical protein MGC14376 (MGC14376), transcript variant 1, mRNA [NM_032895]	NM_032895	Hs.597755	BM906128
GPR26	6.5	3.9	G protein-coupled receptor 26 (GPR26), mRNA [NM_153442]	NM_153442	Hs.12751	AJ505757
LOC654342	6.5	3.2	PREDICTED: lymphocyte-specific protein 1 (LSP1), mRNA [XM_946374]	XM_946374	Hs.656760	CR601542
ESRRA	6.5	2.3	estrogen-related receptor alpha (ESRRA), mRNA [NM_004451]	NM_004451	Hs.110849	BC092470
TMEM9B	6.5	2.4	TMEM9 domain family, member B (TMEM9B), mRNA [NM_020644]	NM_020644	Hs.501853	BC040124
CPNE8	6.5	2.4	copine VIII (CPNE8), mRNA [NM_153634]	NM_153634	Hs.40910	BX538055
FKSG30	6.5	2.6	actin-like protein (FKSG30), mRNA [NM_001017421]	NM_001017421	Unknown	
WDR1	6.5	2.8	WD repeat domain 1 (WDR1), transcript variant 1, mRNA [NM_017491]	NM_017491	Hs.128548	BX648190
HSP90AA1	6.5	1.4	heat shock protein 90kDa alpha (cytosolic), class A member 1 (HSP90AA1), transcript variant 2, mRNA [NM_005348]	NM_005348	Hs.525600	NM_001017963
LONRF2	6.5	1.9	LON peptidase N-terminal domain and ring finger 2 (LONRF2), mRNA [NM_198461]	NM_198461	Hs.21380	NM_198461
ABCA3	6.5	4.1	ATP-binding cassette, sub-family A (ABC1), member 3 (ABCA3), mRNA [NM_001089]	NM_001089	Hs.26630	NM_001089
TRPM3	6.4	8.8	transient receptor potential cation channel, subfamily M, member 3 (TRPM3), transcript variant 7, mRNA [NM_206948]	NM_206948	Hs.47288	NM_001007471
RHOH	6.4	3.0	ras homolog gene family, member H (RHOH), mRNA [NM_004310]	NM_004310	Hs.694951	NM_004310
ENST0000034	6.4	2.3	similar to microtubule-associated proteins 1A/1B light chain 3 (LOC387869), mRNA [Source:RefSeq_dna;Acc:XR_016993] [ENST00000344556]	ENST00000344556	Unknown	
BBOX1	6.4	9.8	butyrobetaine (gamma), 2-oxoglutarate dioxygenase (gamma-butyrobetaine hydroxylase) 1 (BBOX1), mRNA [NM_003986]	NM_003986	Hs.591996	NM_003986
LOC390472	6.4	2.3	PREDICTED: similar to Keratin, type II cytoskeletal 8 (Cytokeratin-8) (CK-8) (Keratin-8) (K8) (LOC390472), mRNA [XR_019203]	XR_019203	Hs.647551	XR_019203
AADACL1	6.4	4.4	arylacetamide deacetylase-like 1 (AADACL1), mRNA [NM_020792]	NM_020792	Hs.444099	BC028734
HSPB1	6.4	3.4	heat shock 27kDa protein 1 (HSPB1), mRNA [NM_001540]	NM_001540	Hs.520973	BM454532
PTP4A2	6.4	1.7	protein tyrosine phosphatase type IVA, member 2 (PTP4A2), transcript variant 2, mRNA [NM_080392]	NM_080392	Hs.470477	AF208850

AI084055	6.4	2.1	oy72a11.x1 NCI_CGAP_CLL1 cDNA clone IMAGE:1671356 3', mRNA sequence [AI084055]	AI084055	Hs.603294	AI084055
AK001903	6.3	4.7	cDNA FLJ11041 fis, clone PLACE1004405. [AK001903]	AK001903	Hs.28792	AK001903
SDC4	6.3	2.5	syndecan 4 (amphiglycan, ryudocan) (SDC4), mRNA [NM_002999]	NM_002999	Hs.632267	NM_002999
ADRB2	6.3	3.5	adrenergic, beta-2-, receptor, surface (ADRB2), mRNA [NM_000024]	NM_000024	Hs.591251	M15169
C18orf8	6.3	1.8	chromosome 18 open reading frame 8 (C18orf8), mRNA [NM_013326]	NM_013326	Hs.529006	AK024370
LOC388743	6.3	3.3	PREDICTED: similar to calpain 8, transcript variant 4 (LOC388743), mRNA [XM_938885]	XM_938885	Unknown	
QPCT	6.3	3.1	glutaminyl-peptide cyclotransferase (glutaminyl cyclase) (QPCT), mRNA [NM_012413]	NM_012413	Hs.79033	NM_012413
FAM92B	6.3	2.2	family with sequence similarity 92, member B (FAM92B), mRNA [NM_198491]	NM_198491	Hs.125713	AK126284
SLC1A3	6.3	2.1	solute carrier family 1 (glial high affinity glutamate transporter), member 3 (SLC1A3), mRNA [NM_004172]	NM_004172	Hs.481918	NM_004172
FAM50A	6.3	2.1	family with sequence similarity 50, member A (FAM50A), mRNA [NM_004699]	NM_004699	Hs.54277	CR612868
CR603184	6.3	1.8	full-length cDNA clone CS0DM012YE16 of Fetal liver of (human). [CR603184]	CR603184	Hs.469944	NM_001100623
HSP90AA1	6.3	1.4	heat shock protein 90kDa alpha (cytosolic), class A member 1 (HSP90AA1), transcript variant 2, mRNA [NM_005348]	NM_005348	Hs.525600	NM_001017963
DLEC1	6.3	2.3	deleted in lung and esophageal cancer 1 (DLEC1), transcript variant DLEC1-N1, mRNA [NM_007335]	NM_007335	Hs.699962	NM_007335
ELOVL1	6.2	3.4	elongation of very long chain fatty acids (FEN1/Elo2, SUR4/Elo3, yeast)-like 1 (ELOVL1), mRNA [NM_022821]	NM_022821	Hs.25597	CR600869
FTS	6.2	2.5	fused toes homolog (mouse) (FTS), transcript variant 1, mRNA [NM_001012398]	NM_001012398	Hs.380897	AK098222
ITGAV	6.2	2.6	integrin, alpha V (vitronectin receptor, alpha polypeptide, antigen CD51) (ITGAV), mRNA [NM_002210]	NM_002210	Hs.436873	NM_002210
AK027319	6.2	1.7	cDNA FLJ14413 fis, clone HEMBA1004670. [AK027319]	AK027319	Hs.334832	AK123705
HSP90AA1	6.2	1.4	heat shock protein 90kDa alpha (cytosolic), class A member 1 (HSP90AA1), transcript variant 2, mRNA [NM_005348]	NM_005348	Hs.525600	NM_001017963
PLEKHB2	6.2	2.6	pleckstrin homology domain containing, family B (evectins) member 2 (PLEKHB2), transcript variant 1, mRNA [NM_001031706]	NM_001031706	Unknown	
SORT1	6.2	2.7	sortilin 1 (SORT1), mRNA [NM_002959]	NM_002959	Hs.485195	NM_002959
ENST0000038	6.2	3.1	PREDICTED: similar to myosin regulatory light chain-like (LOC642045), mRNA [XM_936114]	ENST00000383061	Unknown	
PTPN3	6.2	1.9	Tyrosine-protein phosphatase non-receptor type 3 (EC 3.1.3.48) (Protein-tyrosine phosphatase H1) (PTP-H1). [Source:Uniprot/SWISSPROT;Acc:P26045] [ENST00000374541]	ENST00000374541	Unknown	
IL1A	6.2	12.5	interleukin 1, alpha (IL1A), mRNA [NM_000575]	NM_000575	Hs.1722	NM_000575
IL1A	6.2	16.5	interleukin 1, alpha (IL1A), mRNA [NM_000575]	NM_000575	Hs.1722	NM_000575
PGPEP1	6.2	2.6	pyroglutamyl-peptidase I (PGPEP1), mRNA [NM_017712]	NM_017712	Hs.131776	NM_017712
BIK	6.2	4.1	BCL2-interacting killer (apoptosis-inducing) (BIK), mRNA [NM_001197]	NM_001197	Hs.475055	BE561321
CDC42EP3	6.2	2.5	CDC42 effector protein (Rho GTPase binding) 3 (CDC42EP3), mRNA [NM_006449]	NM_006449	Hs.369574	NM_006449
NQO1	6.2	3.5	NAD(P)H dehydrogenase, quinone 1 (NQO1), transcript variant 1, mRNA [NM_000903]	NM_000903	Hs.406515	NM_000903
ENST0000037	6.2	3.3	ENST00000372493	ENST00000372493	Unknown	
ACTB	6.2	3.4	actin, beta (ACTB), mRNA [NM_001101]	NM_001101	Hs.520640	AK125561
ITGAV	6.1	2.6	integrin, alpha V (vitronectin receptor, alpha polypeptide, antigen CD51) (ITGAV), mRNA [NM_002210]	NM_002210	Hs.436873	NM_002210
DSCR1L2	6.1	2.9	Down syndrome critical region gene 1-like 2 (DSCR1L2), mRNA [NM_013441]	NM_013441	Hs.656799	BC035854
SAA1	6.1	3.6	serum amyloid A1 (SAA1), transcript variant 1, mRNA [NM_000331]	NM_000331	Hs.632144	BG564669
UPP1	6.1	4.3	uridine phosphorylase 1, mRNA (cDNA clone MGC:54255 IMAGE:5549432), complete cds. [BC047030]	BC047030	Hs.488240	BC007348
NEURL	6.1	2.7	neuralized homolog (Drosophila) (NEURL), mRNA [NM_004210]	NM_004210	Hs.654845	BX648827
W31297	6.1	1.8	W31297 zb64a03.r1 Soares_fetal_lung_NbHL19W cDNA clone IMAGE:308332 5' similar to gb:M36341 ADP-RIBOSYLATION FACTOR 4 (HUMAN);, mRNA sequence [W31297]	W31297	Hs.695943	BC016325
RAP1A	6.1	2.8	RAP1A, member of RAS oncogene family (RAP1A), transcript variant 1, mRNA [NM_001010935]	NM_001010935	Hs.190334	NM_001010935
CXorf6	6.1	2.1	chromosome X open reading frame 6 (CXorf6), mRNA [NM_005491]	NM_005491	Hs.20136	BX537560
HILS1	6.1	4.0	histone linker H1 domain, spermatid-specific 1 (HILS1), mRNA [NM_194072]	NM_194072	Unknown	
THC2739760	6.1	2.1	CD521096 AGENCOURT_14355292 NIH_MGC_191 cDNA clone IMAGE:30410815 5', mRNA sequence [CD521096]	THC2739760	Unknown	
HSP90AA1	6.1	1.4	heat shock protein 90kDa alpha (cytosolic), class A member 1 (HSP90AA1), transcript variant 2, mRNA [NM_005348]	NM_005348	Hs.525600	NM_001017963
MICALL1	6.1	3.7	MICAL-like 1 (MICALL1), mRNA [NM_033386]	NM_033386	Hs.517610	AB051455
HS3ST2	6.1	3.8	heparan sulfate (glucosamine) 3-O-sulfotransferase 2 (HS3ST2), mRNA [NM_006043]	NM_006043	Hs.115830	AY359095
BLZF1	6.1	1.6	Golgin 45 (Basic leucine zipper nuclear factor 1) (JEM-1) (p45 basic leucine-zipper nuclear factor). [Source:Uniprot/SWISSPROT;Acc:Q9H2G9] [ENST00000367808]	ENST00000367808	Unknown	
C12orf44	6.1	1.9	chromosome 12 open reading frame 44 (C12orf44), mRNA [NM_021934]	NM_021934	Hs.9911	BM911475
HBEGF	6.1	3.5	heparin-binding EGF-like growth factor (HBEGF), mRNA [NM_001945]	NM_001945	Hs.799	BC033097
SBF1	6.1	2.9	SET binding factor 1 (SBF1), transcript variant 1, mRNA [NM_002972]	NM_002972	Hs.589924	BC087612

A_24_P7750	6.1	2.6	A_24_P7750	A_24_P7750	Unknown	
NMNAT2	6.1	2.3	nicotinamide nucleotide adenylyltransferase 2 (NMNAT2), transcript variant 1, mRNA [NM_015039]	NM_015039	Hs.497123	AF288395
ITGAV	6.1	2.6	integrin, alpha V (vitronectin receptor, alpha polypeptide, antigen CD51) (ITGAV), mRNA [NM_002210]	NM_002210	Hs.436873	NM_002210
MMP19	6.1	5.6	matrix metallopeptidase 19 (MMP19), transcript variant 1, mRNA [NM_002429]	NM_002429	Hs.591033	U38320
SPINT2	6.0	1.9	serine peptidase inhibitor, Kunitz type, 2 (SPINT2), mRNA [NM_021102]	NM_021102	Hs.31439	AK127479
ITGAV	6.0	2.6	integrin, alpha V (vitronectin receptor, alpha polypeptide, antigen CD51) (ITGAV), mRNA [NM_002210]	NM_002210	Hs.436873	NM_002210
SLC17A8	6.0	2.2	solute carrier family 17 (sodium-dependent inorganic phosphate cotransporter), member 8 (SLC17A8), mRNA [NM_139319]	NM_139319	Hs.116871	AK128319
ITGAV	6.0	2.6	integrin, alpha V (vitronectin receptor, alpha polypeptide, antigen CD51) (ITGAV), mRNA [NM_002210]	NM_002210	Hs.436873	NM_002210
DDR1	6.0	3.7	discoidin domain receptor family, member 1 (DDR1), transcript variant 1, mRNA [NM_013993]	NM_013993	Hs.631988	NM_013993
EPHA2	6.0	5.7	EPH receptor A2 (EPHA2), mRNA [NM_004431]	NM_004431	Hs.171596	NM_004431
NKIRAS1	6.0	1.9	NFKB inhibitor interacting Ras-like 1 (NKIRAS1), mRNA [NM_020345]	NM_020345	Hs.173202	NM_020345
LOC401233	6.0	4.2	similar to HIV TAT specific factor 1; cofactor required for Tat activation of HIV-1 transcription (LOC401233), mRNA [NM_001013680]	NM_001013680	Unknown	
HSPB8	6.0	5.9	heat shock 22kDa protein 8 (HSPB8), mRNA [NM_014365]	NM_014365	Hs.400095	NM_014365
SYTL5	6.0	3.0	mRNA; cDNA DKFZp779C093 (from clone DKFZp779C093). [BX647688]	BX647688	Hs.662334	BX647688
DEGS1	6.0	2.8	degenerative spermatocyte homolog 1, lipid desaturase (Drosophila) (DEGS1), transcript variant 2, mRNA [NM_144780]	NM_144780	Hs.299878	NM_003676
SNF8	6.0	1.5	SNF8, ESCRT-II complex subunit, homolog (S. cerevisiae) (SNF8), mRNA [NM_007241]	NM_007241	Hs.127249	BF569053
GLIPR1	6.0	6.2	GLI pathogenesis-related 1 (glioma) (GLIPR1), mRNA [NM_006851]	NM_006851	Hs.205558	NM_006851
CYP1B1	6.0	4.1	cytochrome P450, family 1, subfamily B, polypeptide 1 (CYP1B1), mRNA [NM_000104]	NM_000104	Hs.154654	NM_000104
KIF1A	6.0	5.5	kinesin family member 1A (KIF1A), mRNA [NM_004321]	NM_004321	Hs.516802	NM_004321
TESK1	6.0	1.8	testis-specific kinase 1 (TESK1), mRNA [NM_006285]	NM_006285	Hs.699324	BC038448
SNX16	6.0	1.5	sorting nexin 16 (SNX16), transcript variant 1, mRNA [NM_022133]	NM_022133	Hs.492121	NM_022133
A_24_P16139	6.0	2.4	A_24_P161393	A_24_P161393	Unknown	
ATP6V0B	6.0	2.6	ATPase, H+ transporting, lysosomal 21kDa, V0 subunit b (ATP6V0B), transcript variant 1, mRNA [NM_004047]	NM_004047	Hs.632406	CR596289
THC2676635	6.0	3.0	THC2676635	THC2676635	Unknown	
MAFG	6.0	2.2	v-maf musculoaponeurotic fibrosarcoma oncogene homolog G (avian) (MAFG), transcript variant 1, mRNA [NM_002359]	NM_002359	Hs.252229	NM_002359
CALD1	6.0	5.2	caldesmon 1 (CALD1), transcript variant 1, mRNA [NM_033138]	NM_033138	Hs.490203	NM_033138
ITGAV	6.0	2.6	integrin, alpha V (vitronectin receptor, alpha polypeptide, antigen CD51) (ITGAV), mRNA [NM_002210]	NM_002210	Hs.436873	NM_002210
RNH1	6.0	2.7	ribonuclease/angiogenin inhibitor 1 (RNH1), transcript variant 1, mRNA [NM_002939]	NM_002939	Hs.530687	AB209196
HSP90AA1	6.0	1.4	heat shock protein 90kDa alpha (cytosolic), class A member 1 (HSP90AA1), transcript variant 2, mRNA [NM_005348]	NM_005348	Hs.525600	NM_001017963
TMEPAI	6.0	2.7	transmembrane, prostate androgen induced RNA (TMEPAI), transcript variant 1, mRNA [NM_020182]	NM_020182	Hs.517155	NM_020182
PPM2C	6.0	1.9	protein phosphatase 2C, magnesium-dependent, catalytic subunit (PPM2C), nuclear gene encoding mitochondrial protein, mRNA [NM_018444]	NM_018444	Hs.22265	NM_018444
RHOBTB2	5.9	1.9	Rho-related BTB domain containing 2 (RHOBTB2), mRNA [NM_015178]	NM_015178	Hs.372688	AB018260
THC2682885	5.9	4.0	Q6BEA3_RAT (Q6BEA3) WDNM1 homolog, partial (33%) [THC2682885]	THC2682885	Unknown	
TMSL3	5.9	3.4	thymosin-like 3 (TMSL3), mRNA [NM_183049]	NM_183049	Hs.555895	BU507525
ADRB2	5.9	3.2	adrenergic, beta-2-, receptor, surface (ADRB2), mRNA [NM_000024]	NM_000024	Hs.591251	M15169
METRNL	5.9	2.3	meteorin, glial cell differentiation regulator-like (METRNL), mRNA [NM_001004431]	NM_001004431	Hs.591142	AK093748
RAB27B	5.9	5.0	RAB27B, member RAS oncogene family (RAB27B), mRNA [NM_004163]	NM_004163	Hs.25318	AF131784
DTNA	5.9	2.1	dystrobrevin, alpha (DTNA), transcript variant 7, mRNA [NM_001392]	NM_001392	Hs.695993	NM_001390
BCAR1	5.9	2.5	breast cancer anti-estrogen resistance 1 (BCAR1), mRNA [NM_014567]	NM_014567	Hs.479747	AK124526
REEP5	5.9	2.5	receptor accessory protein 5 (REEP5), mRNA [NM_005669]	NM_005669	Hs.429608	NM_005669
PTP4A2	5.9	1.6	protein tyrosine phosphatase type IVA, member 2 (PTP4A2), transcript variant 1, mRNA [NM_080391]	NM_080391	Hs.470477	AF208850
HTATIP2	5.9	7.3	alternatively spliced product of metastasis-suppressor gene CC3 (TC3) mRNA, complete cds. [AF092095]	AF092095	Hs.90753	NM_001098522
RHOH	5.9	2.8	ras homolog gene family, member H (RHOH), mRNA [NM_004310]	NM_004310	Hs.694951	NM_004310
CREB5	5.9	2.1	cAMP responsive element binding protein 5 (CREB5), transcript variant 1, mRNA [NM_182898]	NM_182898	Hs.437075	NM_182898
ITGAV	5.9	2.6	integrin, alpha V (vitronectin receptor, alpha polypeptide, antigen CD51) (ITGAV), mRNA [NM_002210]	NM_002210	Hs.436873	NM_002210
ACTN1	5.9	5.3	actinin, alpha 1 (ACTN1), mRNA [NM_001102]	NM_001102	Hs.509765	BX641076
LASP1	5.9	2.0	LIM and SH3 protein 1 (LASP1), mRNA [NM_006148]	NM_006148	Hs.548018	NM_006148
ITGAV	5.9	2.6	integrin, alpha V (vitronectin receptor, alpha polypeptide, antigen CD51) (ITGAV), mRNA [NM_002210]	NM_002210	Hs.436873	NM_002210

OLFML2A	5.9	3.5	olfactomedin-like 2A (OLFML2A), mRNA [NM_182487]	NM_182487	Hs.357004	NM_182487
RHOH	5.9	2.7	ras homolog gene family, member H (RHOH), mRNA [NM_004310]	NM_004310	Hs.694951	NM_004310
DCTN3	5.9	1.6	dynactin 3 (p22) (DCTN3), transcript variant 1, mRNA [NM_007234]	NM_007234	Hs.511768	BM920638
WDR47	5.9	1.8	WD repeat domain 47 (WDR47), mRNA [NM_014969]	NM_014969	Hs.654760	BC034964
TUBA1	5.9	7.1	tubulin, alpha 1 (TUBA1), mRNA [NM_006000]	NM_006000	Hs.75318	AK054731
AL359062	5.9	25.1	mRNA full length insert cDNA clone EUROIMAGE 1913076. [AL359062]	AL359062	Hs.134830	AL359062
ATP6V0E1	5.9	2.0	ATPase, H+ transporting, lysosomal 9kDa, V0 subunit e1 (ATP6V0E1), mRNA [NM_003945]	NM_003945	Hs.484188	AV763803
GPR172A	5.9	2.4	G protein-coupled receptor 172A (GPR172A), mRNA [NM_024531]	NM_024531	Hs.6459	CR625605
LOC390610	5.9	2.1	PREDICTED: similar to Keratin, type II cytoskeletal 8 (Cytokeratin-8) (CK-8) (Keratin-8) (K8) (LOC390610), mRNA [XR_017341]	XR_017341	Hs.699134	XR_017341
CR613736	5.9	2.1	full-length cDNA clone CS0DI072YA21 of Placenta Cot 25-normalized of (human). [CR613736]	CR613736	Hs.596312	XM_001128905
FAM84B	5.9	1.7	family with sequence similarity 84, member B (FAM84B), mRNA [NM_174911]	NM_174911	Hs.124951	NM_174911
ACTN1	5.8	4.5	actinin, alpha 1 (ACTN1), mRNA [NM_001102]	NM_001102	Hs.509765	BX641076
LOC441783	5.8	2.3	PREDICTED: similar to T04C12.5 (LOC441783), mRNA [XR_018872]	XR_018872	Hs.647428	XR_018872
A_23_P57836	5.8	3.2	A_23_P57836	A_23_P57836	Unknown	
SPTAN1	5.8	2.2	spectrin, alpha, non-erythrocytic 1 (alpha-fodrin) (SPTAN1), mRNA [NM_003127]	NM_003127	Hs.372331	NM_003127
TLR3	5.8	3.4	toll-like receptor 3 (TLR3), mRNA [NM_003265]	NM_003265	Hs.657724	BC068487
ITGAV	5.8	2.6	integrin, alpha V (vitronectin receptor, alpha polypeptide, antigen CD51) (ITGAV), mRNA [NM_002210]	NM_002210	Hs.436873	NM_002210
RAB21	5.8	2.7	RAB21, member RAS oncogene family (RAB21), mRNA [NM_014999]	NM_014999	Hs.524590	BC009109
TNC	5.8	2.6	tenascin C (hexabrachion) (TNC), mRNA [NM_002160]	NM_002160	Hs.143250	NM_002160
GPC4	5.8	2.2	glypican 4 (GPC4), mRNA [NM_001448]	NM_001448	Hs.58367	AF030186
ITGAV	5.8	2.9	integrin, alpha V (vitronectin receptor, alpha polypeptide, antigen CD51) (ITGAV), mRNA [NM_002210]	NM_002210	Hs.436873	NM_002210
SHB	5.8	2.0	Src homology 2 domain containing adaptor protein B (SHB), mRNA [NM_003028]	NM_003028	Hs.521482	NM_003028
A_24_P14431	5.8	2.7	A_24_P144314	A_24_P144314	Unknown	
ARF1	5.8	1.6	cDNA FLJ13741 fis, clone PLACE3000208. [AK023803]	AK023803	Hs.286221	AK023803
CTSH	5.8	1.7	cathepsin H (CTSH), transcript variant 2, mRNA [NM_148979]	NM_148979	Hs.148641	CR749582
THC2717907	5.8	1.7	Q4IZP3_AZOVI (Q4IZP3) Outer membrane porin, partial (3%) [THC2717907]	THC2717907	Unknown	
MYO1C	5.8	3.5	myosin IC (MYO1C), mRNA [NM_033375]	NM_033375	Hs.286226	NM_001080779
FSTL3	5.7	7.2	follistatin-like 3 (secreted glycoprotein) (FSTL3), mRNA [NM_005860]	NM_005860	Hs.529038	NM_005860
ENST0000032	5.7	3.4	Numb-interacting protein [Source:RefSeq_peptide;Acc:NP_653166] [ENST00000322839]	ENST00000322839	Unknown	
ITGAV	5.7	2.6	integrin, alpha V (vitronectin receptor, alpha polypeptide, antigen CD51) (ITGAV), mRNA [NM_002210]	NM_002210	Hs.436873	NM_002210
PRKAR2A	5.7	2.3	protein kinase, cAMP-dependent, regulatory, type II, alpha (PRKAR2A), mRNA [NM_004157]	NM_004157	Hs.631923	BC002763
TMCO3	5.7	1.6	transmembrane and coiled-coil domains 3 (TMCO3), mRNA [NM_017905]	NM_017905	Hs.317593	BC068515
CDKN2D	5.7	3.2	cyclin-dependent kinase inhibitor 2D (p19, inhibits CDK4) (CDKN2D), transcript variant 1, mRNA [NM_001800]	NM_001800	Hs.435051	NM_001800
A_24_P49800	5.7	2.5	A_24_P49800	A_24_P49800	Unknown	
NEFL	5.7	2.1	neurofilament, light polypeptide 68kDa (NEFL), mRNA [NM_006158]	NM_006158	Hs.521461	NM_006158
ELL3	5.7	2.5	elongation factor RNA polymerase II-like 3 (ELL3), mRNA [NM_025165]	NM_025165	Hs.699876	AK126384
TSGA2	5.7	2.6	testis specific A2 homolog (mouse) (TSGA2), mRNA [NM_080860]	NM_080860	Hs.661069	AK057315
BMP7	5.7	3.5	bone morphogenetic protein 7 (osteogenic protein 1) (BMP7), mRNA [NM_001719]	NM_001719	Hs.473163	AK094784
MGC26963	5.7	5.9	hypothetical protein MGC26963 (MGC26963), mRNA [NM_152621]	NM_152621	Hs.595423	BX649142
TPM1	5.7	4.2	tropomyosin 1 (alpha) (TPM1), transcript variant 3, mRNA [NM_001018004]	NM_001018004	Hs.133892	BX648171
TES	5.7	2.7	testis derived transcript (3 LIM domains) (TES), transcript variant 2, mRNA [NM_152829]	NM_152829	Hs.592286	NM_015641
RAB36	5.7	1.7	RAB36, member RAS oncogene family (RAB36), mRNA [NM_004914]	NM_004914	Hs.369557	AB023061
HSP90AA1	5.7	1.4	heat shock protein 90kDa alpha (cytosolic), class A member 1 (HSP90AA1), transcript variant 2, mRNA [NM_005348]	NM_005348	Hs.525600	NM_001017963
YIPF6	5.7	1.7	Yip1 domain family, member 6 (YIPF6), mRNA [NM_173834]	NM_173834	Hs.700646	AK095555
AMPH	5.7	1.6	amphiphysin (Stiff-Man syndrome with breast cancer 128kDa autoantigen) (AMPH), transcript variant 1, mRNA [NM_001635]	NM_001635	Hs.592182	NM_001635
RNF14	5.7	1.9	ring finger protein 14 (RNF14), transcript variant 1, mRNA [NM_004290]	NM_004290	Hs.483616	NM_004290
CLTB	5.7	2.5	clathrin, light chain (Lcb) (CLTB), transcript variant 2, mRNA [NM_007097]	NM_007097	Hs.484241	BM908253
PLB1	5.7	3.0	phospholipase B1 (PLB1), mRNA [NM_153021]	NM_153021	Hs.444933	NM_153021
CSDC2	5.7	2.1	cold shock domain containing C2, RNA binding (CSDC2), mRNA [NM_014460]	NM_014460	Hs.310893	NM_014460
PCGF5	5.7	1.5	polycomb group ring finger 5 (PCGF5), mRNA [NM_032373]	NM_032373	Hs.500512	NM_032373
MGC10334	5.7	2.8	hypothetical protein MGC10334 (MGC10334), mRNA [NM_001029885]	NM_001029885	Hs.515689	NM_001029885

EME2	5.7	2.2	mRNA for FLJ00151 protein. [AK074080]	AK074080	Unknown	
EIF2C3	5.7	1.5	eukaryotic translation initiation factor 2C, 3 (EIF2C3), transcript variant 1, mRNA [NM_024852]	NM_024852	Hs.657659	NM_024852
TMEM139	5.6	2.5	transmembrane protein 139 (TMEM139), mRNA [NM_153345]	NM_153345	Hs.17558	BC035517
MAFK	5.6	3.8	v-maf musculoaponeurotic fibrosarcoma oncogene homolog K (avian) (MAFK), mRNA [NM_002360]	NM_002360	Hs.520612	NM_002360
THC2509288	5.6	1.7	ALU4_HUMAN (P39191) Alu subfamily SB2 sequence contamination warning entry, partial (29%) [THC2509288]	THC2509288	Unknown	
PTPN21	5.6	2.0	protein tyrosine phosphatase, non-receptor type 21 (PTPN21), mRNA [NM_007039]	NM_007039	Hs.437040	NM_007039
DEGS1	5.6	2.2	degenerative spermatocyte homolog 1, lipid desaturase (Drosophila) (DEGS1), transcript variant 1, mRNA [NM_003676]	NM_003676	Hs.299878	NM_003676
HCCS	5.6	2.0	holocytochrome c synthase (cytochrome c heme-lyase) (HCCS), mRNA [NM_005333]	NM_005333	Hs.211571	NM_005333
LOC286260	5.6	1.9	hypothetical protein LOC286260, mRNA (cDNA clone IMAGE:5115498), with apparent retained intron. [BC032375]	BC032375	Hs.593896	AK023312
LOC387856	5.6	5.5	similar to expressed sequence AI836003 (LOC387856), mRNA [NM_001013635]	NM_001013635	Hs.251699	BC049387
ADRB2	5.6	3.3	adrenergic, beta-2-, receptor, surface (ADRB2), mRNA [NM_000024]	NM_000024	Hs.591251	M15169
MRLC2	5.6	3.0	myosin regulatory light chain MRLC2 (MRLC2), mRNA [NM_033546]	NM_033546	Hs.464472	BQ067233
RABIF	5.6	2.5	RAB interacting factor (RABIF), mRNA [NM_002871]	NM_002871	Hs.90875	NM_002871
LOC649003	5.6	2.5	PREDICTED: similar to cytoplasmic beta-actin (LOC649003), mRNA [XR_018969]	XR_018969	Hs.647780	XR_018969
PWWP2	5.6	2.8	PWWP domain containing 2 (PWWP2), mRNA [NM_138499]	NM_138499	Hs.527751	AK096940
C16orf72	5.6	1.4	chromosome 16 open reading frame 72 (C16orf72), mRNA [NM_014117]	NM_014117	Hs.221497	NM_014117
KIAA1432	5.6	2.0	mRNA for KIAA1432 protein, partial cds. [AB037853]	AB037853	Hs.211520	AB037853
ZNF702	5.6	3.3	cDNA FLJ12985 fis, clone NT2RP3000050, moderately similar to ZINC FINGER PROTEIN 91. [AK023047]	AK023047	Hs.270435	BC032590
PEA15	5.6	2.8	phosphoprotein enriched in astrocytes 15 (PEA15), mRNA [NM_003768]	NM_003768	Hs.517216	NM_003768
ANXA2	5.6	4.5	annexin A2 (ANXA2), transcript variant 2, mRNA [NM_001002857]	NM_001002857	Hs.511605	BF242966
CDR1	5.6	2.5	cerebellar degeneration-related protein 1, 34kDa (CDR1), mRNA [NM_004065]	NM_004065	Hs.571748	AK054921
RP5-875H10.1	5.6	2.0	SAM domain containing 1 (LOC389432), mRNA [NM_001030060]	NM_001030060	Hs.567973	NM_001030060
LOC649344	5.6	2.1	PREDICTED: similar to Keratin, type II cytoskeletal 8 (Cytokeratin-8) (CK-8) (Keratin-8) (K8) (LOC649344), mRNA [XR_018597]	XR_018597	Hs.647727	XR_018597
AF131834	5.6	1.6	clone 24841 mRNA sequence. [AF131834]	AF131834	Hs.4892	AF131834
WDR42A	5.6	1.6	WD repeat domain 42A, mRNA (cDNA clone IMAGE:5743945), complete cds. [BC111063]	BC111063	Hs.632447	NM_015726
SYT17	5.6	2.4	synaptotagmin XVII (SYT17), mRNA [NM_016524]	NM_016524	Hs.258326	AK226136
TUBB4	5.6	4.2	tubulin, beta 4 (TUBB4), mRNA [NM_006087]	NM_006087	Hs.110837	NM_006087
CREB3	5.6	2.0	cAMP responsive element binding protein 3 (CREB3), mRNA [NM_006368]	NM_006368	Hs.522110	NM_006368
BC004969	5.6	2.4	cDNA clone IMAGE:2906290, complete cds. [BC004969]	BC004969	Unknown	
IL1A	5.6	19.2	interleukin 1, alpha (IL1A), mRNA [NM_000575]	NM_000575	Hs.1722	NM_000575
ELL3	5.6	2.8	elongation factor RNA polymerase II-like 3 (ELL3), mRNA [NM_025165]	NM_025165	Hs.699876	AK126384
A_24_P11059	5.6	1.9	A_24_P110591	A_24_P110591	Unknown	
PLAU	5.6	8.7	plasminogen activator, urokinase (PLAU), mRNA [NM_002658]	NM_002658	Hs.77274	NM_002658
RER1	5.6	2.0	RER1 retention in endoplasmic reticulum 1 homolog (S. cerevisiae) (RER1), mRNA [NM_007033]	NM_007033	Hs.525527	NM_007033
LOC643386	5.6	1.9	PREDICTED: similar to actin, alpha 2, smooth muscle, aorta (LOC643386), mRNA [XM_926717]	XM_926717	Unknown	
COL7A1	5.6	5.6	collagen, type VII, alpha 1 (epidermolysis bullosa, dystrophic, dominant and recessive) (COL7A1), mRNA [NM_000094]	NM_000094	Hs.476218	L02870
HIVEP2	5.6	1.8	human immunodeficiency virus type I enhancer binding protein 2 (HIVEP2), mRNA [NM_006734]	NM_006734	Hs.510172	NM_006734
GABRA5	5.6	5.2	gamma-aminobutyric acid (GABA) A receptor, alpha 5 (GABRA5), mRNA [NM_000810]	NM_000810	Hs.612087	NM_000810
THC2664573	5.6	4.9	THC2664573	THC2664573	Unknown	
SAMD9	5.5	3.7	sterile alpha motif domain containing 9 (SAMD9), mRNA [NM_017654]	NM_017654	Hs.65641	NM_017654
CAMK1D	5.5	1.7	calcium/calmodulin-dependent protein kinase ID (CAMK1D), transcript variant 2, mRNA [NM_153498]	NM_153498	Hs.659517	NM_153498
FLJ46266	5.5	8.6	CDNA FLJ46266 fis, clone TESTI4027139. [Source:Uniprot/SPTREMBL;Acc:Q6ZRL3] [ENST00000332814]	ENST00000332814	Unknown	
ANXA2P1	5.5	4.9	annexin A2 pseudogene 1 (ANXA2P1) on chromosome 4 [NR_001562]	NR_001562	Unknown	
WDR1	5.5	2.1	WD repeat domain 1 (WDR1), transcript variant 1, mRNA [NM_017491]	NM_017491	Hs.128548	BX648190
RANGAP1	5.5	2.6	Ran GTPase activating protein 1 (RANGAP1), mRNA [NM_002883]	NM_002883	Hs.183800	BC041396
RSU1	5.5	2.1	Ras suppressor protein 1 (RSU1), transcript variant 1, mRNA [NM_012425]	NM_012425	Hs.524161	NM_012425
MGC70863	5.5	2.4	similar to RPL23AP7 protein (MGC70863), transcript variant 2, mRNA [NM_203302]	NM_203302	Hs.406135	BC065556
AK093521	5.5	1.6	cDNA FLJ36202 fis, clone TESTI2028296. [AK093521]	AK093521	Hs.552095	AK093521
FGF1	5.5	5.5	fibroblast growth factor 1 (acidic) (FGF1), transcript variant 1, mRNA [NM_000800]	NM_000800	Hs.483635	NM_000800

PLAU	5.5	8.9	plasminogen activator, urokinase (PLAU), mRNA [NM_002658]	NM_002658	Hs.77274	NM_002658
NCF2	5.5	13.9	neutrophil cytosolic factor 2 (65kDa, chronic granulomatous disease, autosomal 2) (NCF2), mRNA [NM_000433]	NM_000433	Hs.587558	AB209647
SLC7A6	5.5	2.7	solute carrier family 7 (cationic amino acid transporter, y+ system), member 6 (SLC7A6), transcript variant 2, mRNA [NM_001076785]	NM_001076785	Hs.700614	NM_001076785
ADRB2	5.5	3.4	adrenergic, beta-2-, receptor, surface (ADRB2), mRNA [NM_000024]	NM_000024	Hs.591251	M15169
ZNF536	5.5	3.2	zinc finger protein 536 (ZNF536), mRNA [NM_014717]	NM_014717	Hs.378901	BC150171
GFPT1	5.5	2.5	glutamine-fructose-6-phosphate transaminase 1 (GFPT1), mRNA [NM_002056]	NM_002056	Hs.645286	BC045641
FAM18B2	5.5	2.0	family with sequence similarity 18, member B2 (FAM18B2), mRNA [NM_145301]	NM_145301	Hs.659357	BC011952
VDR	5.5	6.0	vitamin D (1,25- dihydroxyvitamin D3) receptor (VDR), transcript variant 2, mRNA [NM_001017535]	NM_001017535	Hs.524368	NM_001017535
RHOH	5.5	2.6	ras homolog gene family, member H (RHOH), mRNA [NM_004310]	NM_004310	Hs.694951	NM_004310
CDR2L	5.5	2.5	cerebellar degeneration-related protein 2-like (CDR2L), mRNA [NM_014603]	NM_014603	Hs.78358	NM_014603
RIN3	5.5	2.5	Ras and Rab interactor 3 (RIN3), mRNA [NM_024832]	NM_024832	Hs.326822	AK090451
FAM73B	5.5	2.4	family with sequence similarity 73, member B (FAM73B), mRNA [NM_032809]	NM_032809	Hs.632693	AK074127
ADRB2	5.5	3.3	adrenergic, beta-2-, receptor, surface (ADRB2), mRNA [NM_000024]	NM_000024	Hs.591251	M15169
PLAU	5.5	8.6	plasminogen activator, urokinase (PLAU), mRNA [NM_002658]	NM_002658	Hs.77274	NM_002658
PLAU	5.5	8.9	plasminogen activator, urokinase (PLAU), mRNA [NM_002658]	NM_002658	Hs.77274	NM_002658
IRAK1	5.5	3.1	interleukin-1 receptor-associated kinase 1 (IRAK1), transcript variant 1, mRNA [NM_001569]	NM_001569	Hs.522819	L76191
A_24_P93111	5.5	2.0	A_24_P93111	A_24_P93111	Unknown	
ARPC4	5.5	2.1	actin related protein 2/3 complex, subunit 4, 20kDa (ARPC4), transcript variant 1, mRNA [NM_005718]	NM_005718	Hs.323342	AK125875
TRIM16L	5.5	2.2	tripartite motif-containing 16-like (TRIM16L), mRNA [NM_001037330]	NM_001037330	Hs.164324	AK095390
TAP1	5.4	5.2	transporter 1, ATP-binding cassette, sub-family B (MDR/TAP) (TAP1), mRNA [NM_000593]	NM_000593	Hs.352018	BX648013
RPRC1	5.4	1.9	arginine/proline rich coiled-coil 1 (RPRC1), mRNA [NM_018067]	NM_018067	Hs.700595	AB033013
ADRB2	5.4	3.6	adrenergic, beta-2-, receptor, surface (ADRB2), mRNA [NM_000024]	NM_000024	Hs.591251	M15169
LGALS8	5.4	2.4	lectin, galactoside-binding, soluble, 8 (galectin 8) (LGALS8), transcript variant 1, mRNA [NM_006499]	NM_006499	Hs.4082	AB209914
ME3	5.4	2.8	malic enzyme 3, NADP(+)-dependent, mitochondrial (ME3), nuclear gene encoding mitochondrial protein, transcript variant 2, mRNA [NM_001014811]	NM_001014811	Hs.199743	NM_001014811
SDC2	5.4	2.7	syndecan 2 (heparan sulfate proteoglycan 1, cell surface-associated, fibroglycan) (SDC2), mRNA [NM_002998]	NM_002998	Hs.1501	BC030133
CK300181	5.4	1.9	UI-E-EJ1-ajx-g-19-0-UI.s1 UI-E-EJ1 cDNA clone UI-E-EJ1-ajx-g-19-0-UI 3', mRNA sequence [CK300181]	CK300181	Hs.626125	CK300181
ARPC2	5.4	1.8	actin related protein 2/3 complex, subunit 2, 34kDa (ARPC2), transcript variant 1, mRNA [NM_152862]	NM_152862	Hs.529303	BF240734
LOC651275	5.4	3.2	PREDICTED: similar to cytoplasmic beta-actin (LOC651275), mRNA [XR_019505]	XR_019505	Hs.648263	XR_019505
HSP90AA1	5.4	1.4	heat shock protein 90kDa alpha (cytosolic), class A member 1 (HSP90AA1), transcript variant 2, mRNA [NM_005348]	NM_005348	Hs.525600	NM_001017963
PPME1	5.4	2.2	protein phosphatase methylesterase 1 (PPME1), mRNA [NM_016147]	NM_016147	Hs.503251	AK095005
RHOH	5.4	2.4	ras homolog gene family, member H (RHOH), mRNA [NM_004310]	NM_004310	Hs.694951	NM_004310
SRP14	5.4	1.4	signal recognition particle 14kDa (homologous Alu RNA binding protein) (SRP14), mRNA [NM_003134]	NM_003134	Hs.533732	BM805684
NDUFB2	5.4	1.6	NADH dehydrogenase (ubiquinone) 1 beta subcomplex, 2, 8kDa (NDUFB2), nuclear gene encoding mitochondrial protein, mRNA [NM_004546]	NM_004546	Hs.655788	CR610711
MAMDC2	5.4	7.4	MAM domain containing 2 (MAMDC2), mRNA [NM_153267]	NM_153267	Hs.547172	BX538309
AGRIN	5.4	2.1	agrin (AGRIN), mRNA [NM_198576]	NM_198576	Hs.273330	AB191264
GNB2	5.4	2.4	guanine nucleotide binding protein (G protein), beta polypeptide 2 (GNB2), mRNA [NM_005273]	NM_005273	Hs.185172	BQ059784
A_24_P84410	5.4	2.3	A_24_P844100	A_24_P844100	Unknown	
PLAU	5.4	8.7	plasminogen activator, urokinase (PLAU), mRNA [NM_002658]	NM_002658	Hs.77274	NM_002658
RASSF1	5.4	2.4	Ras association (RalGDS/AF-6) domain family 1 (RASSF1), transcript variant C, mRNA [NM_170713]	NM_170713	Hs.476270	NM_170714
KCTD11	5.4	2.2	potassium channel tetramerisation domain containing 11 (KCTD11), mRNA [NM_001002914]	NM_001002914	Hs.592112	NM_001002914
LRRC2	5.4	8.6	leucine rich repeat containing 2 (LRRC2), mRNA [NM_024750]	NM_024750	Hs.657345	NM_024512
BIRC7	5.4	2.1	baculoviral IAP repeat-containing 7 (livin) (BIRC7), transcript variant 2, mRNA [NM_022161]	NM_022161	Hs.256126	AY358836
FTS	5.4	2.8	fused toes homolog (mouse) (FTS), transcript variant 1, mRNA [NM_001012398]	NM_001012398	Hs.380897	AK098222
VDR	5.4	5.8	vitamin D (1,25- dihydroxyvitamin D3) receptor (VDR), transcript variant 2, mRNA [NM_001017535]	NM_001017535	Hs.524368	NM_001017535
ZNF28	5.4	2.0	zinc finger protein 28 (ZNF28), mRNA [NM_006969]	NM_006969	Hs.554778	AK131368
AIFM2	5.4	3.3	apoptosis-inducing factor, mitochondrion-associated, 2 (AIFM2), mRNA [NM_032797]	NM_032797	Hs.655377	NM_032797
GRID1	5.4	2.8	glutamate receptor, ionotropic, delta 1 (GRID1), mRNA [NM_017551]	NM_017551	Hs.530653	NM_017551
ATP10A	5.4	3.0	ATPase, Class V, type 10A (ATP10A), mRNA [NM_024490]	NM_024490	Hs.659258	AB051358

PLAU	5.4	8.6	plasminogen activator, urokinase (PLAU), mRNA [NM_002658]	NM_002658	Hs.77274	NM_002658
CDH6	5.4	3.0	cadherin 6, type 2, K-cadherin (fetal kidney) (CDH6), mRNA [NM_004932]	NM_004932	Hs.171054	NM_004932
CITED2	5.4	1.7	Cbp/p300-interacting transactivator, with Glu/Asp-rich carboxy-terminal domain, 2 (CITED2), mRNA [NM_006079]	NM_006079	Hs.82071	NM_006079
THC2680668	5.4	4.0	BU678941 UI-CF-DU1-aat-a-09-0-UI.s1 UI-CF-DU1 cDNA clone UI-CF-DU1-aat-a-09-0-UI 3', mRNA sequence [BU678941]	THC2680668	Unknown	
PEA15	5.4	4.7	phosphoprotein enriched in astrocytes 15 (PEA15), mRNA [NM_003768]	NM_003768	Hs.517216	NM_003768
SFXN5	5.3	1.7	sideroflexin 5 (SFXN5), mRNA [NM_144579]	NM_144579	Hs.368171	BX640669
FREQ	5.3	1.9	frequenin homolog (Drosophila) (FREQ), mRNA [NM_014286]	NM_014286	Hs.694740	NM_014286
LOC399947	5.3	2.3	LOH11CR1A gene, loss of heterozygosity, 11, chromosomal region 1 gene A product. [AB096240]	AB096240	Hs.172982	AB096240
CCDC96	5.3	1.9	coiled-coil domain containing 96 (CCDC96), mRNA [NM_153376]	NM_153376	Hs.656757	NM_153376
TMEM142B	5.3	2.2	transmembrane protein 142B (TMEM142B), mRNA [NM_032831]	NM_032831	Hs.363308	AK056497
AGRIN	5.3	2.1	agrin (AGRIN), mRNA [NM_198576]	NM_198576	Hs.273330	AB191264
LRRC48	5.3	2.1	leucine rich repeat containing 48 (LRRC48), mRNA [NM_031294]	NM_031294	Hs.579264	AK096528
ENST0000025	5.3	2.5	MHC class I polypeptide-related sequence B (MICB), mRNA [Source:RefSeq_dna;Acc:NM_005931] [ENST00000252229]	ENST00000252229	Unknown	
NDFIP2	5.3	2.2	Nedd4 family interacting protein 2 (NDFIP2), mRNA [NM_019080]	NM_019080	Hs.525093	AB032991
RHOH	5.3	2.6	ras homolog gene family, member H (RHOH), mRNA [NM_004310]	NM_004310	Hs.694951	NM_004310
PLAU	5.3	8.8	plasminogen activator, urokinase (PLAU), mRNA [NM_002658]	NM_002658	Hs.77274	NM_002658
VIM	5.3	2.6	vimentin (VIM), mRNA [NM_003380]	NM_003380	Hs.642813	AK093924
FZD6	5.3	1.7	frizzled homolog 6 (Drosophila) (FZD6), mRNA [NM_003506]	NM_003506	Hs.591863	BX538222
MAF	5.3	2.2	short form transcription factor C-MAF (c-maf) mRNA, complete cds. [AF055376]	AF055376	Hs.699396	NM_001031804
ENST0000036	5.3	4.7	KIAA1244 (KIAA1244), mRNA [Source:RefSeq_dna;Acc:NM_020340] [ENST00000367711]	ENST00000367711	Unknown	
C11orf63	5.3	2.8	chromosome 11 open reading frame 63 (C11orf63), transcript variant 1, mRNA [NM_024806]	NM_024806	Hs.164705	NM_024806
RNF14	5.3	1.9	ring finger protein 14 (RNF14), transcript variant 1, mRNA [NM_004290]	NM_004290	Hs.483616	NM_004290
PKM2	5.3	2.1	pyruvate kinase, muscle (PKM2), transcript variant 2, mRNA [NM_182470]	NM_182470	Hs.534770	NM_182470
C18orf1	5.3	2.7	chromosome 18 open reading frame 1 (C18orf1), transcript variant a2, mRNA [NM_181482]	NM_181482	Hs.149363	NM_001003674
ESTRRA	5.3	2.7	estrogen-related receptor alpha pseudogene (ESTRRA) on chromosome 13 [NR_000033]	NR_000033	Unknown	
PPP2R2B	5.3	5.2	protein phosphatase 2 (formerly 2A), regulatory subunit B (PR 52), beta isoform (PPP2R2B), transcript variant 1, mRNA [NM_004576]	NM_004576	Hs.655213	M64930
WWC3	5.3	2.3	WWC family member 3 (WWC3), mRNA [NM_015691]	NM_015691	Hs.527524	NM_015691
GPR157	5.3	2.2	Probable G-protein coupled receptor 157. [Source:Uniprot/SWISSPROT;Acc:Q5UAW9] [ENST00000377411]	ENST00000377411	Unknown	
PAK6	5.3	1.6	p21(CDKN1A)-activated kinase 6 (PAK6), mRNA [NM_020168]	NM_020168	Hs.513645	AK131522
SDHB	5.3	2.2	succinate dehydrogenase complex, subunit B, iron sulfur (Ip) (SDHB), mRNA [NM_003000]	NM_003000	Hs.465924	BQ073692
GLIS3	5.3	3.4	GLIS family zinc finger 3 (GLIS3), transcript variant 1, mRNA [NM_001042413]	NM_001042413	Hs.162125	NM_001042413
ANXA5	5.3	3.0	annexin A5 (ANXA5), mRNA [NM_001154]	NM_001154	Hs.480653	NM_001154
PLAU	5.3	8.8	plasminogen activator, urokinase (PLAU), mRNA [NM_002658]	NM_002658	Hs.77274	NM_002658
RHOH	5.3	2.5	ras homolog gene family, member H (RHOH), mRNA [NM_004310]	NM_004310	Hs.694951	NM_004310
LAMP3	5.3	2.1	lysosomal-associated membrane protein 3 (LAMP3), mRNA [NM_014398]	NM_014398	Hs.518448	BC032940
GABRB3	5.3	4.5	gamma-aminobutyric acid (GABA) A receptor, beta 3 (GABRB3), transcript variant 1, mRNA [NM_000814]	NM_000814	Hs.302352	NM_000814
LOC651099	5.3	2.3	PREDICTED: similar to cytoplasmic beta-actin (LOC651099), mRNA [XR_019039]	XR_019039	Hs.648177	XR_019039
THC2596442	5.3	3.7	1W7B_A Chain A, Annexin A2: Does It Induce Membrane Aggregation By A New Multimeric State Of The Protein. {Homo sapiens} (exp=-1; wgp=0; cg=0), partial (24%) [THC2596442]	THC2596442	Unknown	
ST6GALNAC2	5.3	2.6	ST6 (alpha-N-acetyl-neuraminyl-2,3-beta-galactosyl-1,3)-N-acetylgalactosaminide alpha-2,6-sialyltransferase 2 (ST6GALNAC2), mRNA [NM_006456]	NM_006456	Hs.592105	BC038114
LRRFIP2	5.3	2.1	leucine rich repeat (in FLII) interacting protein 2 (LRRFIP2), transcript variant 1, mRNA [NM_006309]	NM_006309	Hs.475319	AK124432
A_24_P24332	5.3	2.0	A_24_P24332	A_24_P24332	Unknown	
KNS2	5.3	1.6	kinesin 2 (KNS2), transcript variant 2, mRNA [NM_182923]	NM_182923	Hs.20107	AK092888
NOV	5.3	4.5	nephroblastoma overexpressed gene (NOV), mRNA [NM_002514]	NM_002514	Hs.235935	NM_002514
BHLHB2	5.3	2.3	basic helix-loop-helix domain containing, class B, 2 (BHLHB2), mRNA [NM_003670]	NM_003670	Hs.171825	NM_003670
A_24_P89476	5.3	2.6	A_24_P894763	A_24_P894763	Unknown	
PLAU	5.3	8.6	plasminogen activator, urokinase (PLAU), mRNA [NM_002658]	NM_002658	Hs.77274	NM_002658
DDAH1	5.3	2.4	dimethylarginine dimethylaminohydrolase 1 (DDAH1), mRNA [NM_012137]	NM_012137	Hs.379858	NM_012137
LRP10	5.3	2.9	low density lipoprotein receptor-related protein 10 (LRP10), mRNA [NM_014045]	NM_014045	Hs.525232	NM_014045
LOC389976	5.3	2.3	PREDICTED: similar to Actin, cytoplasmic 2 (Gamma-actin) (LOC389976), mRNA [XR_018711]	XR_018711	Hs.646731	XR_018711

C12orf5	5.3	4.0	chromosome 12 open reading frame 5 (C12orf5), mRNA [NM_020375]	NM_020375	Hs.504545	NM_020375
C2orf39	5.2	2.5	chromosome 2 open reading frame 39 (C2orf39), mRNA [NM_145038]	NM_145038	Hs.393714	AK128286
A_24_P30661	5.2	2.6	A_24_P306614	A_24_P306614	Unknown	
TSPAN2	5.2	4.3	tetraspanin 2 (TSPAN2), mRNA [NM_005725]	NM_005725	Hs.310458	NM_005725
C3orf25	5.2	1.7	chromosome 3 open reading frame 25 (C3orf25), mRNA [NM_207307]	NM_207307	Hs.652347	AL133011
RIT1	5.2	2.1	Ras-like without CAAX 1 (RIT1), mRNA [NM_006912]	NM_006912	Hs.491234	NM_006912
ENST0000032	5.2	2.3	cDNA clone MGC:40177 IMAGE:5167345, complete cds. [BC028123]	ENST00000324928	Unknown	
THC2586959	5.2	1.8	1PK0_D Chain D, Crystal Structure Of The Ef3-Cam Complexed With Pmeapp. {Homo sapiens} (exp=-1; wgp=0; cg=0), partial (70%) [THC2586959]	THC2586959	Unknown	
ZMIZ2	5.2	1.6	zinc finger, MIZ-type containing 2 (ZMIZ2), transcript variant 1, mRNA [NM_031449]	NM_031449	Hs.77978	NM_031449
ADRB2	5.2	3.4	adrenergic, beta-2-, receptor, surface (ADRB2), mRNA [NM_000024]	NM_000024	Hs.591251	M15169
COX17	5.2	1.6	COX17 cytochrome c oxidase assembly homolog (S. cerevisiae) (COX17), nuclear gene encoding mitochondrial protein, mRNA [NM_005694]	NM_005694	Hs.534383	BU506590
KIF1C	5.2	1.8	Human kinesin-like motor protein KIF1C mRNA, complete cds. [U91329]	U91329	Hs.435120	AB014606
REEP3	5.2	2.0	receptor accessory protein 3 (REEP3), mRNA [NM_001001330]	NM_001001330	Hs.499833	CR936686
TRPM3	5.2	7.9	transient receptor potential cation channel, subfamily M, member 3 (TRPM3), transcript variant 7, mRNA [NM_206948]	NM_206948	Hs.47288	NM_001007471
ARID5A	5.2	2.3	AT rich interactive domain 5A (MRF1-like) (ARID5A), transcript variant 2, mRNA [NM_006673]	NM_006673	Unknown	
ADRB2	5.2	3.2	adrenergic, beta-2-, receptor, surface (ADRB2), mRNA [NM_000024]	NM_000024	Hs.591251	M15169
CPEB2	5.2	4.0	cytoplasmic polyadenylation element binding protein 2 (CPEB2), transcript variant B, mRNA [NM_182485]	NM_182485	Hs.656937	NM_182485
DOCK2	5.2	3.3	dedicator of cytokinesis 2 (DOCK2), mRNA [NM_004946]	NM_004946	Hs.586174	NM_004946
ISG20L2	5.2	1.3	interferon stimulated exonuclease gene 20kDa-like 2 (ISG20L2), mRNA [NM_030980]	NM_030980	Hs.301904	AL096734
ZBTB7B	5.2	2.3	zinc finger and BTB domain containing 7B (ZBTB7B), mRNA [NM_015872]	NM_015872	Hs.700874	CR749303
DENND3	5.2	2.1	DENN/MADD domain containing 3 (DENND3), mRNA [NM_014957]	NM_014957	Hs.656299	NM_014957
NF2	5.2	1.7	neurofibromin 2 (bilateral acoustic neuroma) (NF2), transcript variant 8, mRNA [NM_181832]	NM_181832	Hs.187898	NM_181832
MYH9	5.2	5.2	myosin, heavy chain 9, non-muscle (MYH9), mRNA [NM_002473]	NM_002473	Hs.474751	NM_002473
AF131798	5.2	3.0	clone 25119 mRNA sequence. [AF131798]	AF131798	Hs.448887	AK091326
CR598940	5.2	3.6	full-length cDNA clone CS0DI032YF23 of Placenta Cot 25-normalized of (human). [CR598940]	CR598940	Hs.561513	CR598940
TNFRSF11B	5.2	7.2	tumor necrosis factor receptor superfamily, member 11b (osteoprotegerin) (TNFRSF11B), mRNA [NM_002546]	NM_002546	Hs.81791	NM_002546
SLC13A4	5.2	8.7	solute carrier family 13 (sodium/sulfate symporters), member 4 (SLC13A4), mRNA [NM_012450]	NM_012450	Hs.490241	AB208809
SNTB1	5.2	1.8	Tax interaction protein 43 mRNA, partial cds. [AF028828]	AF028828	Hs.655236	AK026095
TNFRSF11B	5.2	6.7	tumor necrosis factor receptor superfamily, member 11b (osteoprotegerin) (TNFRSF11B), mRNA [NM_002546]	NM_002546	Hs.81791	NM_002546
GTSE1	5.2	2.1	G-2 and S-phase expressed 1 (GTSE1), mRNA [NM_016426]	NM_016426	Hs.386189	NM_016426
IL1A	5.2	11.6	interleukin 1, alpha (IL1A), mRNA [NM_000575]	NM_000575	Hs.1722	NM_000575
SLC27A4	5.2	2.5	solute carrier family 27 (fatty acid transporter), member 4 (SLC27A4), mRNA [NM_005094]	NM_005094	Hs.656699	NM_005094
C1orf87	5.2	3.3	chromosome 1 open reading frame 87 (C1orf87), mRNA [NM_152377]	NM_152377	Hs.47385	AK126061
TRIM8	5.2	1.6	tripartite motif-containing 8 (TRIM8), mRNA [NM_030912]	NM_030912	Hs.336810	NM_030912
CDK2AP2	5.2	2.3	CDK2-associated protein 2 (CDK2AP2), mRNA [NM_005851]	NM_005851	Hs.523835	BM926335
C6orf114	5.2	2.9	chromosome 6 open reading frame 114 (C6orf114), mRNA [NM_033069]	NM_033069	Hs.484686	AK000337
PLSCR3	5.2	1.8	phospholipid scramblase 3 (PLSCR3), mRNA [NM_020360]	NM_020360	Hs.655161	AF289602
LOC283537	5.2	4.4	hypothetical protein LOC283537 (LOC283537), mRNA [NM_181785]	NM_181785	Hs.117167	BX649127
MAP6	5.2	2.5	microtubule-associated protein 6 (MAP6), transcript variant 1, mRNA [NM_033063]	NM_033063	Hs.585540	BC150254
UBE2J2	5.2	2.1	ubiquitin-conjugating enzyme E2, J2 (UBC6 homolog, yeast) (UBE2J2), transcript variant 3, mRNA [NM_194458]	NM_194458	Hs.191987	NM_194458
TMEM49	5.2	3.7	transmembrane protein 49, mRNA (cDNA clone IMAGE:4295896), with apparent retained intron. [BC024020]	BC024020	Hs.659162	BQ050170
LOC162073	5.2	1.5	cDNA FLJ39008 fis, clone NT2RI2025255. [AK096327]	AK096327	Hs.530899	NM_001034841
PCQAP	5.2	2.1	PC2 (positive cofactor 2, multiprotein complex) glutamine/Q-rich-associated protein (PCQAP), transcript variant 1, mRNA [NM_001003891]	NM_001003891	Hs.517421	NM_001003891
SAMD4A	5.1	2.5	mRNA for KIAA1053 protein, partial cds. [AB028976]	AB028976	Hs.98259	AB028976
ELL2	5.1	1.9	elongation factor, RNA polymerase II, 2 (ELL2), mRNA [NM_012081]	NM_012081	Hs.592742	NM_012081
NOL6	5.1	2.5	nucleolar protein family 6 (RNA-associated) (NOL6), transcript variant alpha, mRNA [NM_022917]	NM_022917	Hs.493709	NM_022917
RHOH	5.1	2.6	ras homolog gene family, member H (RHOH), mRNA [NM_004310]	NM_004310	Hs.694951	NM_004310
INHBA	5.1	6.3	inhibin, beta A (activin A, activin AB alpha polypeptide) (INHBA), mRNA [NM_002192]	NM_002192	Hs.583348	BX648811
BTBD9	5.1	2.3	mRNA for KIAA1880 protein, partial cds. [AB067467]	AB067467	Unknown	

FASTK	5.1	1.8	Fas-activated serine/threonine kinase (FASTK), transcript variant 1, mRNA [NM_006712]	NM_006712	Hs.647094	CR611266
ENST0000031	5.1	2.8	Actin-like protein (Fragment). [Source:Uniprot/SPTREMBL;Acc:Q562T9] [ENST00000312847]	ENST00000312847	Unknown	
CXXC5	5.1	2.2	CXXC finger 5 (CXXC5), mRNA [NM_016463]	NM_016463	Hs.189119	BC006428
CDH6	5.1	11.9	Cadherin-6 precursor (Kidney-cadherin) (K-cadherin). [Source:Uniprot/SWISSPROT;Acc:P55285] [ENST00000382216]	ENST00000382216	Unknown	
STK38L	5.1	2.0	serine/threonine kinase 38 like (STK38L), mRNA [NM_015000]	NM_015000	Hs.184523	AB023182
TLL2	5.1	6.9	tolloid-like 2 (TLL2), mRNA [NM_012465]	NM_012465	Hs.154296	NM_012465
TMEM165	5.1	2.3	transmembrane protein 165 (TMEM165), mRNA [NM_018475]	NM_018475	Hs.479766	BC107582
C1orf124	5.1	1.7	chromosome 1 open reading frame 124 (C1orf124), transcript variant 1, mRNA [NM_032018]	NM_032018	Hs.554892	NM_001010984
EIF3S8	5.1	1.7	eukaryotic translation initiation factor 3, subunit 8, 110kDa (EIF3S8), transcript variant 2, mRNA [NM_001037808]	NM_001037808	Hs.567374	NM_001037808
SYT11	5.1	2.2	synaptotagmin XI (SYT11), mRNA [NM_152280]	NM_152280	Hs.32984	NM_152280
LOC132205	5.1	2.2	cDNA FLJ33859 fis, clone CTONG2006223, moderately similar to KERATIN, TYPE II CYTOSKELETAL 8. [AK091178]	AK091178	Hs.590045	AK091178
ACTR3	5.1	2.4	ARP3 actin-related protein 3 homolog (yeast) (ACTR3), mRNA [NM_005721]	NM_005721	Hs.433512	BC044590
1-Dec	5.1	22.3	Homo sapiens, clone IMAGE:5526534, mRNA, partial cds. [BC030567]	BC030567	Hs.148841	AK056153
A_24_P53097	5.1	3.3	A_24_P530977	A_24_P530977	Unknown	
SNF1LK	5.1	2.9	SNF1-like kinase (SNF1LK), mRNA [NM_173354]	NM_173354	Hs.282113	BC038504
TNFRSF11B	5.1	6.8	tumor necrosis factor receptor superfamily, member 11b (osteoprotegerin) (TNFRSF11B), mRNA [NM_002546]	NM_002546	Hs.81791	NM_002546
Z25424	5.1	1.9	H.sapiens protein-serine/threonine kinase gene, complete CDS. [Z25424]	Z25424	Hs.515032	AB209074
KIAA0284	5.1	4.3	KIAA0284 (KIAA0284), mRNA [NM_015005]	NM_015005	Hs.533721	BC112928
VDR	5.1	5.5	vitamin D (1,25- dihydroxyvitamin D3) receptor (VDR), transcript variant 2, mRNA [NM_001017535]	NM_001017535	Hs.524368	NM_001017535
SLC6A4	5.1	2.0	solute carrier family 6 (neurotransmitter transporter, serotonin), member 4 (SLC6A4), mRNA [NM_001045]	NM_001045	Hs.591192	NM_001045
A_23_P15660	5.1	3.1	A_23_P156609	A_23_P156609	Unknown	
SETD8	5.1	2.1	SET domain containing (lysine methyltransferase) 8 (SETD8), mRNA [NM_020382]	NM_020382	Hs.700682	BC050346
AK026351	5.1	1.5	cDNA: FLJ22698 fis, clone HSI12044. [AK026351]	AK026351	Hs.631923	BC002763
PLAU	5.1	8.7	plasminogen activator, urokinase (PLAU), mRNA [NM_002658]	NM_002658	Hs.77274	NM_002658
CHRM3	5.1	2.0	cholinergic receptor, muscarinic 3 (CHRM3), mRNA [NM_000740]	NM_000740	Hs.7138	NM_000740
SLC22A3	5.1	2.4	solute carrier family 22 (extraneuronal monoamine transporter), member 3 (SLC22A3), mRNA [NM_021977]	NM_021977	Hs.567337	BX640965
STK17A	5.1	2.1	serine/threonine kinase 17a (apoptosis-inducing) (STK17A), mRNA [NM_004760]	NM_004760	Hs.699353	NM_004760
ADRB2	5.1	3.5	adrenergic, beta-2-, receptor, surface (ADRB2), mRNA [NM_000024]	NM_000024	Hs.591251	M15169
PRKAR2A	5.1	2.8	protein kinase, cAMP-dependent, regulatory, type II, alpha, mRNA (cDNA clone MGC:3606 IMAGE:3629579), complete cds. [BC002763]	BC002763	Hs.631923	BC002763
TRADD	5.1	3.5	TNFRSF1A-associated via death domain (TRADD), mRNA [NM_003789]	NM_003789	Hs.460996	AK090673
SPARCL1	5.1	1.7	SPARC-like 1 (mast9, hevin) (SPARCL1), mRNA [NM_004684]	NM_004684	Hs.62886	X86693
WFIKKN2	5.0	2.1	WAP, follistatin/kazal, immunoglobulin, kunitz and netrin domain containing 2 (WFIKKN2), mRNA [NM_175575]	NM_175575	Hs.211475	NM_175575
ZMIZ2	5.0	1.5	zinc finger, MIZ-type containing 2 (ZMIZ2), transcript variant 1, mRNA [NM_031449]	NM_031449	Hs.77978	NM_031449
ADRB2	5.0	3.2	adrenergic, beta-2-, receptor, surface (ADRB2), mRNA [NM_000024]	NM_000024	Hs.591251	M15169
PTRF	5.0	5.6	polymerase I and transcript release factor (PTRF), mRNA [NM_012232]	NM_012232	Hs.437191	BC066123
FAM62C	5.0	3.4	chromosome 3 cDNA. [AJ697972]	AJ697972	Hs.477711	AJ697972
PTP4A2	5.0	2.1	protein tyrosine phosphatase type IVA, member 2 (PTP4A2), transcript variant 2, mRNA [NM_080392]	NM_080392	Hs.470477	AF208850
CBLN1	5.0	3.8	cerebellin 1 precursor (CBLN1), mRNA [NM_004352]	NM_004352	Hs.458423	NM_004352
LOC340508	5.0	2.2	hypothetical protein LOC340508 (LOC340508) on chromosome 9 [NR_002942]	NR_002942	Unknown	
THC2548576	5.0	1.3	THC2548576	THC2548576	Unknown	
TMCC2	5.0	2.0	transmembrane and coiled-coil domain family 2 (TMCC2), mRNA [NM_014858]	NM_014858	Hs.6360	AY358192
SLC35A3	5.0	1.9	solute carrier family 35 (UDP-N-acetylglucosamine (UDP-GlcNAc) transporter), member A3 (SLC35A3), mRNA [NM_012243]	NM_012243	Hs.448979	CR749816
RNF40	5.0	1.9	ring finger protein 40 (RNF40), mRNA [NM_014771]	NM_014771	Hs.65238	CR627431
THC2563227	5.0	2.4	CA312433 UI-CF-FN0-afk-i-18-0-UI.s1 UI-CF-FN0 cDNA clone UI-CF-FN0-afk-i-18-0-UI 3', mRNA sequence [CA312433]	THC2563227	Unknown	
THC2674406	5.0	7.1	PPU38804 {Porphyra purpurea} (exp=-1; wgp=0; cg=1), partial (5%) [THC2674406]	THC2674406	Unknown	
ZYX	5.0	3.0	zyxin (ZYX), transcript variant 1, mRNA [NM_003461]	NM_003461	Hs.490415	BC073825
AHR	5.0	2.9	aryl hydrocarbon receptor (AHR), mRNA [NM_001621]	NM_001621	Hs.171189	NM_001621
C15orf24	5.0	1.9	chromosome 15 open reading frame 24 (C15orf24), mRNA [NM_020154]	NM_020154	Hs.160565	BQ054039
CAP1	5.0	3.0	CAP, adenylate cyclase-associated protein 1 (yeast) (CAP1), mRNA [NM_006367]	NM_006367	Hs.370581	NM_006367
TNFRSF11B	5.0	7.0	tumor necrosis factor receptor superfamily, member 11b (osteoprotegerin) (TNFRSF11B), mRNA [NM_002546]	NM_002546	Hs.81791	NM_002546

CORO2B	5.0	2.5	coronin, actin binding protein, 2B (CORO2B), mRNA [NM_006091]	NM_006091	Hs.551213	NM_006091
ANKRD1	5.0	7.3	ankyrin repeat domain 1 (cardiac muscle) (ANKRD1), mRNA [NM_014391]	NM_014391	Hs.448589	NM_014391
MAPK1	5.0	1.8	mitogen-activated protein kinase 1 (MAPK1), transcript variant 2, mRNA [NM_138957]	NM_138957	Hs.431850	AL157438
CD276	5.0	2.9	CD276 molecule (CD276), transcript variant 1, mRNA [NM_001024736]	NM_001024736	Hs.77873	NM_001024736
CTA-126B4.3	5.0	2.0	Gastric cancer antigen Zg14. [Source:Uniprot/SWISSPROT;Acc:Q9Y3A4] [ENST00000323013]	ENST00000323013	Unknown	
COL4A4	5.0	16.6	collagen, type IV, alpha 4 (COL4A4), mRNA [NM_000092]	NM_000092	Hs.591645	NM_000092
RIPK3	5.0	5.3	receptor-interacting serine-threonine kinase 3 (RIPK3), mRNA [NM_006871]	NM_006871	Hs.268551	NM_006871
THC2677011	5.0	1.9	THC2677011	THC2677011	Unknown	
B3GAT3	5.0	2.4	beta-1,3-glucuronyltransferase 3 (glucuronosyltransferase I) (B3GAT3), mRNA [NM_012200]	NM_012200	Hs.502759	AK075166
CAMK2D	5.0	2.3	calcium/calmodulin-dependent protein kinase (CaM kinase) II delta (CAMK2D), transcript variant 3, mRNA [NM_001221]	NM_001221	Hs.144114	BX647363
APLP1	5.0	2.7	amyloid beta (A4) precursor-like protein 1 (APLP1), transcript variant 2, mRNA [NM_005166]	NM_005166	Hs.74565	NM_001024807
C14orf72	5.0	2.5	PREDICTED: chromosome 14 open reading frame 72 (C14orf72), mRNA [XM_096733]	XM_096733	Hs.150840	XM_096733
ENST0000038	5.0	2.5	ENST00000383490	ENST00000383490	Unknown	
LAMP2	5.0	2.4	lysosomal-associated membrane protein 2 (LAMP2), transcript variant LAMP2A, mRNA [NM_002294]	NM_002294	Hs.496684	BX648255
MSRB3	5.0	3.3	methionine sulfoxide reductase B3 (MSRB3), transcript variant 2, mRNA [NM_001031679]	NM_001031679	Hs.339024	NM_001031679
TMED4	5.0	2.1	cDNA FLJ90076 fis, clone HEMBA1004444, highly similar to Putative NFkB activating protein HNLF. [AK074557]	AK074557	Hs.632641	BC035467
COPB1	5.0	1.5	coatomer protein complex, subunit beta 1 (COPB1), mRNA [NM_016451]	NM_016451	Hs.339278	NM_016451
C19orf55	5.0	1.7	chromosome 19 open reading frame 55 (C19orf55), mRNA [NM_001039887]	NM_001039887	Hs.527982	BC110893
MGST2	5.0	1.6	microsomal glutathione S-transferase 2 (MGST2), mRNA [NM_002413]	NM_002413	Hs.81874	BX538128
NFKBIB	5.0	2.2	nuclear factor of kappa light polypeptide gene enhancer in B-cells inhibitor, beta (NFKBIB), transcript variant 2, mRNA [NM_001001716]	NM_001001716	Hs.9731	NM_001001716
CA2	5.0	2.5	carbonic anhydrase II (CA2), mRNA [NM_000067]	NM_000067	Hs.155097	AK123309
AK094995	5.0	9.3	cDNA FLJ37676 fis, clone BRHIP2012627. [AK094995]	AK094995	Hs.646953	AK094995
TNFRSF11B	5.0	6.5	tumor necrosis factor receptor superfamily, member 11b (osteoprotegerin) (TNFRSF11B), mRNA [NM_002546]	NM_002546	Hs.81791	NM_002546
KIAA0258	4.9	2.1	KIAA0258, mRNA (cDNA clone MGC:755 IMAGE:3533857), complete cds. [BC001725]	BC001725	Hs.493804	D87447
ITGB1	4.9	2.3	integrin, beta 1 (fibronectin receptor, beta polypeptide, antigen CD29 includes MDF2, MSK12) (ITGB1), transcript variant 1A, mRNA [NM_002211]	NM_002211	Hs.695946	NM_002211
HSP90AA1	4.9	1.4	heat shock protein 90kDa alpha (cytosolic), class A member 1 (HSP90AA1), transcript variant 2, mRNA [NM_005348]	NM_005348	Hs.525600	NM_001017963
C19orf28	4.9	2.1	chromosome 19 open reading frame 28 (C19orf28), transcript variant 1, mRNA [NM_021731]	NM_021731	Hs.656901	NM_021731
GCH1	4.9	2.8	GTP cyclohydrolase 1 (dopa-responsive dystonia) (GCH1), transcript variant 1, mRNA [NM_000161]	NM_000161	Hs.86724	NM_000161
RAB31	4.9	2.5	RAB31, member RAS oncogene family (RAB31), mRNA [NM_006868]	NM_006868	Hs.99528	NM_006868
NDUFS1	4.9	1.4	NADH dehydrogenase (ubiquinone) Fe-S protein 1, 75kDa (NADH-coenzyme Q reductase) (NDUFS1), nuclear gene encoding mitochondrial protein, mRNA [NM_005006]	NM_005006	Hs.471207	BC030833
B3GALNT2	4.9	1.8	beta-1,3-N-acetylgalactosaminyltransferase 2 (B3GALNT2), mRNA [NM_152490]	NM_152490	Hs.585040	AB209046
LOC389073	4.9	3.0	cDNA clone MGC:46742 IMAGE:5730487, complete cds. [BC035737]	ENST00000341287	Unknown	
PAFAH2	4.9	2.4	platelet-activating factor acetylhydrolase 2, 40kDa (PAFAH2), mRNA [NM_000437]	NM_000437	Hs.477083	NM_000437
BCAN	4.9	3.9	brevican, mRNA (cDNA clone IMAGE:3618761), partial cds. [BC005081]	BC005081	Unknown	
SLC44A5	4.9	1.9	Choline transporter-like protein 5 (Solute carrier family 44 member 5). [Source:Uniprot/SWISSPROT;Acc:Q8NCS7] [ENST00000370855]	ENST00000370855	Unknown	
TNFRSF11B	4.9	6.9	tumor necrosis factor receptor superfamily, member 11b (osteoprotegerin) (TNFRSF11B), mRNA [NM_002546]	NM_002546	Hs.81791	NM_002546
PDLIM7	4.9	2.0	PDZ and LIM domain 7 (enigma) (PDLIM7), transcript variant 1, mRNA [NM_005451]	NM_005451	Hs.533040	AK096826
ENST0000027	4.9	1.5	full-length cDNA clone CS0DI044YK15 of Placenta Cot 25-normalized of (human). [CR601728]	ENST00000273582	Unknown	
CDC42	4.9	1.8	cell division cycle 42 (GTP binding protein, 25kDa) (CDC42), transcript variant 2, mRNA [NM_044472]	NM_044472	Hs.690198	NM_001039802
VCL	4.9	2.8	vinculin (VCL), transcript variant 1, mRNA [NM_014000]	NM_014000	Hs.699180	NM_014000
GAP43	4.9	4.2	growth associated protein 43 (GAP43), mRNA [NM_002045]	NM_002045	Hs.134974	AK091466
LGI3	4.9	1.9	leucine-rich repeat LGI family, member 3 (LGI3), mRNA [NM_139278]	NM_139278	Hs.33470	NM_139278
UCHL1	4.9	2.8	ubiquitin carboxyl-terminal esterase L1 (ubiquitin thiolesterase) (UCHL1), mRNA [NM_004181]	NM_004181	Hs.518731	AB209038
ATP13A1	4.9	2.0	ATPase type 13A1 (ATP13A1), mRNA [NM_020410]	NM_020410	Hs.501794	AK074159
RNF19	4.9	1.8	ring finger protein 19 (RNF19), transcript variant 1, mRNA [NM_183419]	NM_183419	Hs.292882	NM_183419
IL1A	4.9	11.5	interleukin 1, alpha (IL1A), mRNA [NM_000575]	NM_000575	Hs.1722	NM_000575
A_23_P11345	4.9	1.9	A_23_P113453	A_23_P113453	Unknown	

A_24_P27976	4.9	2.4	A_24_P279760	A_24_P279760	Unknown	
TNFRSF11B	4.9	6.7	tumor necrosis factor receptor superfamily, member 11b (osteoprotegerin) (TNFRSF11B), mRNA [NM_002546]	NM_002546	Hs.81791	NM_002546
DNAPTP6	4.9	2.9	DNA polymerase-transactivated protein 6 (DNAPTP6), mRNA [NM_015535]	NM_015535	Hs.120323	NM_001100422
NOC2L	4.9	2.3	nucleolar complex associated 2 homolog (S. cerevisiae), mRNA (cDNA clone IMAGE:4274902), partial cds. [BC009786]	BC009786	Hs.405987	BC003555
CEBPD	4.9	2.4	CCAAT/enhancer binding protein (C/EBP), delta (CEBPD), mRNA [NM_005195]	NM_005195	Hs.440829	BM924801
C6orf154	4.9	3.6	chromosome 6 open reading frame 154 (C6orf154), mRNA [NM_001012974]	NM_001012974	Hs.445552	NM_001012974
RAB15	4.9	2.1	RAB15, member RAS onocogene family (RAB15), mRNA [NM_198686]	NM_198686	Hs.512492	NM_198686
SH3BGRL3	4.9	1.9	SH3 domain binding glutamic acid-rich protein like 3 (SH3BGRL3), mRNA [NM_031286]	NM_031286	Hs.109051	AF466367
CYB561	4.9	1.9	cytochrome b-561 (CYB561), transcript variant 2, mRNA [NM_001017916]	NM_001017916	Hs.355264	AK095244
DIRAS2	4.9	2.6	DIRAS family, GTP-binding RAS-like 2 (DIRAS2), mRNA [NM_017594]	NM_017594	Hs.165636	NM_017594
ATP13A2	4.8	2.3	ATPase type 13A2 (ATP13A2), mRNA [NM_022089]	NM_022089	Hs.128866	AY461712
MFI2	4.8	4.7	antigen p97 (melanoma associated) identified by monoclonal antibodies 133.2 and 96.5 (MFI2), transcript variant 2, mRNA [NM_033316]	NM_033316	Hs.184727	NM_005929
TNFRSF11B	4.8	6.7	tumor necrosis factor receptor superfamily, member 11b (osteoprotegerin) (TNFRSF11B), mRNA [NM_002546]	NM_002546	Hs.81791	NM_002546
TTLL7	4.8	1.6	Tubulin--tyrosine ligase-like protein 7 (Protein NYD-SP30). [Source:Uniprot/SWISSPROT;Acc:Q6ZT98] [ENST00000370703]	ENST00000370703	Unknown	
BAG3	4.8	2.2	BCL2-associated athanogene 3 (BAG3), mRNA [NM_004281]	NM_004281	Hs.523309	NM_004281
ACTG1	4.8	1.9	actin, gamma 1 (ACTG1), mRNA [NM_001614]	NM_001614	Hs.514581	CR606241
FLJ40172	4.8	2.7	hypothetical protein FLJ40172 (FLJ40172), mRNA [NM_173649]	NM_173649	Hs.531575	NM_173649
ZNF702	4.8	3.2	zinc finger protein 702 (ZNF702), mRNA [NM_024924]	NM_024924	Unknown	
LOC441714	4.8	1.9	PREDICTED: similar to ATP-binding cassette sub-family B member 10, mitochondrial precursor (ATP-binding cassette transporter 10) (ABC transporter 10 protein) (Mitochondrial ATP-binding cassette 2) (M-ABC2) (LOC441714), mRNA [XR_019458]	XR_019458	Hs.650933	XR_019458
PTPRC	4.8	2.3	protein tyrosine phosphatase, receptor type, C (PTPRC), transcript variant 1, mRNA [NM_002838]	NM_002838	Hs.654514	NM_002838
KIAA0999	4.8	1.6	KIAA0999 protein (KIAA0999), mRNA [NM_025164]	NM_025164	Hs.167451	AL832068
PARVA	4.8	1.9	parvin, alpha (PARVA), mRNA [NM_018222]	NM_018222	Hs.607144	AL832682
ZNF79	4.8	1.5	zinc finger protein 79 (ZNF79), mRNA [NM_007135]	NM_007135	Hs.522399	AK054606
LOC727891	4.8	1.4	PREDICTED: similar to Heat shock protein HSP 90-alpha (HSP 86) (NY-REN-38 antigen) (LOC727891), mRNA [XR_015156]	XR_015156	Hs.631939	BQ423056
VCL	4.8	2.1	vinculin (VCL), transcript variant 1, mRNA [NM_014000]	NM_014000	Hs.699180	NM_014000
VDR	4.8	5.9	vitamin D (1,25- dihydroxyvitamin D3) receptor (VDR), transcript variant 2, mRNA [NM_001017535]	NM_001017535	Hs.524368	NM_001017535
MAPK1	4.8	1.8	mitogen-activated protein kinase 1 (MAPK1), transcript variant 2, mRNA [NM_138957]	NM_138957	Hs.431850	AL157438
PLXDC2	4.8	2.0	plexin domain containing 2 (PLXDC2), mRNA [NM_032812]	NM_032812	Hs.658134	AY358486
WSB2	4.8	2.9	WD repeat and SOCS box-containing 2 (WSB2), mRNA [NM_018639]	NM_018639	Hs.506985	NM_018639
BTBD12	4.8	1.8	BTB (POZ) domain containing 12 (BTBD12), mRNA [NM_032444]	NM_032444	Hs.143681	NM_032444
ALDH4A1	4.8	2.1	aldehyde dehydrogenase 4 family, member A1 (ALDH4A1), nuclear gene encoding mitochondrial protein, transcript variant P5CDhS, mRNA [NM_170726]	NM_170726	Hs.77448	NM_003748
THC2701140	4.8	4.9	THC2701140	THC2701140	Unknown	
MAPK1	4.8	1.8	mitogen-activated protein kinase 1 (MAPK1), transcript variant 2, mRNA [NM_138957]	NM_138957	Hs.431850	AL157438
RHOH	4.8	2.7	ras homolog gene family, member H (RHOH), mRNA [NM_004310]	NM_004310	Hs.694951	NM_004310
DDR1	4.8	6.3	discoidin domain receptor family, member 1 (DDR1), transcript variant 1, mRNA [NM_013993]	NM_013993	Hs.631988	NM_013993
CDV3	4.8	1.8	CDV3 homolog (mouse) (CDV3), mRNA [NM_017548]	NM_017548	Hs.699298	AK096865
SLC25A23	4.8	1.8	solute carrier family 25 (mitochondrial carrier; phosphate carrier), member 23 (SLC25A23), mRNA [NM_024103]	NM_024103	Hs.356231	NM_024103
CR622110	4.8	2.7	full-length cDNA clone CS0DC025YP03 of Neuroblastoma Cot 25-normalized of (human). [CR622110]	CR622110	Hs.370423	CR622110
AK098185	4.8	2.0	cDNA FLJ40866 fis, clone TRACH2018718. [AK098185]	AK098185	Hs.645492	AK091407
CHRNB1	4.8	1.6	cholinergic receptor, nicotinic, beta 1 (muscle) (CHRNB1), mRNA [NM_000747]	NM_000747	Hs.330386	BC023553
VDR	4.8	6.2	vitamin D (1,25- dihydroxyvitamin D3) receptor (VDR), transcript variant 2, mRNA [NM_001017535]	NM_001017535	Hs.524368	NM_001017535
HIP1R	4.8	3.0	huntingtin interacting protein 1 related (HIP1R), mRNA [NM_003959]	NM_003959	Hs.654842	NM_003959
RAB3B	4.8	2.0	Ras-related protein Rab-3B. [Source:Uniprot/SWISSPROT;Acc:P20337] [ENST00000371655]	ENST00000371655	Unknown	
SYBL1	4.8	1.7	synaptobrevin-like 1 (SYBL1), mRNA [NM_005638]	NM_005638	Hs.24167	BC056141
TEX261	4.8	1.6	testis expressed sequence 261 (TEX261), mRNA [NM_144582]	NM_144582	Hs.516087	NM_144582
BP290435	4.8	1.9	BP290435 BP290435 Sugano cDNA library, lung fibroblast cDNA clone LNF05072, mRNA sequence [BP290435]	BP290435	Hs.369920	NM_015646
FNDC3B	4.7	2.3	fibronectin type III domain containing 3B (FNDC3B), mRNA [NM_022763]	NM_022763	Hs.159430	NM_022763

TMSB4X	4.7	3.3	Thymosin beta-4 (T beta 4) (Fx) [Contains: Hematopoietic system regulatory peptide (Seraspenide)]. [Source:Uniprot/SWISSPROT;Acc:P62328] [ENST00000380635]	ENST00000380635	Unknown	
RBM18	4.7	1.6	RNA binding motif protein 18 (RBM18), mRNA [NM_033117]	NM_033117	Hs.415842	AK057676
THC2741040	4.7	2.0	CB243285 UI-CF-FN0-agc-l-12-0-UI.s1 UI-CF-FN0 cDNA clone UI-CF-FN0-agc-l-12-0-UI 3', mRNA sequence [CB243285]	THC2741040	Unknown	
BX538057	4.7	2.9	mRNA; cDNA DKFZp686J1595 (from clone DKFZp686J1595) [BX538057]	BX538057	Hs.408455	BX538057
EPPK1	4.7	2.8	epiplakin 1 (EPPK1), mRNA [NM_031308]	NM_031308	Hs.200412	AB051895
ABHD12	4.7	2.0	abhydrolase domain containing 12 (ABHD12), transcript variant 2, mRNA [NM_015600]	NM_015600	Hs.441550	NM_001042472
A_32_P10258	4.7	2.4	A_32_P102581	A_32_P102581	Unknown	
RRAGD	4.7	3.0	Ras-related GTP binding D (RRAGD), mRNA [NM_021244]	NM_021244	Hs.485938	AL137502
COL4A3	4.7	11.8	collagen, type IV, alpha 3 (Goodpasture antigen) (COL4A3), transcript variant 1, mRNA [NM_000091]	NM_000091	Hs.570065	NM_000091
TMOD3	4.7	2.1	tropomodulin 3 (ubiquitous) (TMOD3), mRNA [NM_014547]	NM_014547	Hs.4998	AL137543
C12orf5	4.7	3.2	chromosome 12 open reading frame 5 (C12orf5), mRNA [NM_020375]	NM_020375	Hs.504545	NM_020375
TUBB8	4.7	2.6	tubulin, beta 8 (TUBB8), mRNA [NM_177987]	NM_177987	Hs.532659	CR606225
VDR	4.7	5.2	vitamin D (1,25- dihydroxyvitamin D3) receptor (VDR), transcript variant 2, mRNA [NM_001017535]	NM_001017535	Hs.524368	NM_001017535
K03200	4.7	2.6	Human melanoma-associated antigen p97 (melanotransferrin) mRNA, 3' flank. [K03200]	K03200	Unknown	
HYDIN	4.7	2.1	hydrocephalus inducing homolog (mouse) (HYDIN), transcript variant 1, mRNA [NM_032821]	NM_032821	Hs.461229	NM_032821
MXRA7	4.7	1.8	matrix-remodelling associated 7 (MXRA7), transcript variant 1, mRNA [NM_001008528]	NM_001008528	Hs.250723	NM_001008528
TNFRSF11B	4.7	7.0	tumor necrosis factor receptor superfamily, member 11b (osteoprotegerin) (TNFRSF11B), mRNA [NM_002546]	NM_002546	Hs.81791	NM_002546
BQ018742	4.7	2.0	BQ018742 UI-H-DH1-awu-d-06-0-UI.s1 NCI_CGAP_DH1 cDNA clone IMAGE:5823701 3', mRNA sequence [BQ018742]	BQ018742	Hs.696469	NM_004892
SGCB	4.7	1.9	sarcoglycan, beta (43kDa dystrophin-associated glycoprotein) (SGCB), mRNA [NM_000232]	NM_000232	Hs.438953	NM_000232
TNMD	4.7	8.9	tenomodulin (TNMD), mRNA [NM_022144]	NM_022144	Hs.132957	BC034030
MAP3K14	4.7	1.8	mitogen-activated protein kinase kinase kinase 14 (MAP3K14), mRNA [NM_003954]	NM_003954	Hs.404183	Y10256
NFKBIB	4.7	2.6	nuclear factor of kappa light polypeptide gene enhancer in B-cells inhibitor, beta (NFKBIB), transcript variant 2, mRNA [NM_001001716]	NM_001001716	Hs.9731	NM_001001716
PAK3	4.7	2.9	p21 (CDKN1A)-activated kinase 3 (PAK3), mRNA [NM_002578]	NM_002578	Hs.656789	NM_002578
BC032585	4.7	1.9	cDNA clone IMAGE:5500261, partial cds. [BC032585]	BC032585	Hs.655589	BM455300
PITX1	4.7	2.7	paired-like homeodomain transcription factor 1 (PITX1), mRNA [NM_002653]	NM_002653	Hs.84136	NM_002653
PKM2	4.7	2.6	pyruvate kinase, muscle (PKM2), transcript variant 2, mRNA [NM_182470]	NM_182470	Hs.534770	NM_182470
PVR	4.7	3.7	poliovirus receptor (PVR), mRNA [NM_006505]	NM_006505	Hs.171844	NM_006505
JAK2	4.7	1.8	Janus kinase 2 (a protein tyrosine kinase) (JAK2), mRNA [NM_004972]	NM_004972	Hs.656213	AF058925
CHST3	4.7	2.8	carbohydrate (chondroitin 6) sulfotransferase 3 (CHST3), mRNA [NM_004273]	NM_004273	Hs.158304	AB017915
ITGB1	4.7	2.0	integrin, beta 1 (fibronectin receptor, beta polypeptide, antigen CD29 includes MDF2, MSK12) (ITGB1), transcript variant 1E, mRNA [NM_133376]	NM_133376	Hs.695946	NM_002211
MARK2	4.7	1.9	MAP/microtubule affinity-regulating kinase 2 (MARK2), transcript variant 1, mRNA [NM_017490]	NM_017490	Hs.567261	AB188493
A_32_P23243	4.7	2.2	A_32_P232437	A_32_P232437	Unknown	
PRSS23	4.7	3.6	protease, serine, 23 (PRSS23), mRNA [NM_007173]	NM_007173	Hs.25338	AL832007
NFKBIE	4.7	2.6	nuclear factor of kappa light polypeptide gene enhancer in B-cells inhibitor, epsilon (NFKBIE), mRNA [NM_004556]	NM_004556	Hs.458276	NM_004556
CPEB2	4.7	6.8	cytoplasmic polyadenylation element binding protein 2 (CPEB2), transcript variant B, mRNA [NM_182485]	NM_182485	Hs.656937	NM_182485
MUS81	4.7	2.0	MUS81 endonuclease homolog (S. cerevisiae) (MUS81), mRNA [NM_025128]	NM_025128	Unknown	
AK058041	4.7	1.6	cDNA FLJ25312 fis, clone SYN01070. [AK058041]	AK058041	Hs.667544	AK058041
GADD45A	4.7	3.3	growth arrest and DNA-damage-inducible, alpha (GADD45A), mRNA [NM_001924]	NM_001924	Hs.80409	CR612719
TOR1AIP1	4.7	1.8	torsin A interacting protein 1 (TOR1AIP1), mRNA [NM_015602]	NM_015602	Hs.496459	NM_015602
THC2557762	4.7	2.3	ALU2_HUMAN (P39189) Alu subfamily SB sequence contamination warning entry, partial (8%) [THC2557762]	THC2557762	Unknown	
AK092662	4.7	3.8	cDNA FLJ35343 fis, clone PROST2015932. [AK092662]	AK092662	Hs.116233	AK092662
CXXC5	4.7	1.6	CXXC finger 5 (CXXC5), mRNA [NM_016463]	NM_016463	Hs.189119	BC006428
ITPKC	4.7	1.8	inositol 1,4,5-trisphosphate 3-kinase C (ITPKC), mRNA [NM_025194]	NM_025194	Hs.515415	BC060788
AF086187	4.7	29.2	full length insert cDNA clone ZC30H06. [AF086187]	AF086187	Hs.103070	BC040870
A_24_P21231	4.7	2.2	A_24_P212314	A_24_P212314	Unknown	
TNFRSF11B	4.7	6.9	tumor necrosis factor receptor superfamily, member 11b (osteoprotegerin) (TNFRSF11B), mRNA [NM_002546]	NM_002546	Hs.81791	NM_002546
NME2	4.7	1.6	non-metastatic cells 2, protein (NM23B) expressed in (NME2), transcript variant 1, mRNA [NM_002512]	NM_002512	Hs.463456	BG114681
IL28RA	4.7	3.2	interleukin 28 receptor, alpha (interferon, lambda receptor) (IL28RA), transcript variant 3, mRNA [NM_173065]	NM_173065	Hs.221375	NM_170743

ACRC	4.7	1.4	acidic repeat containing (ACRC), mRNA [NM_052957]	NM_052957	Hs.135167	AK127607
RP11-217H1.1	4.7	1.9	implantation-associated protein (DKFZp564K142), mRNA [NM_032121]	NM_032121	Hs.323562	BC060842
ANKRD45	4.7	2.4	ankyrin repeat domain 45 (ANKRD45), mRNA [NM_198493]	NM_198493	Hs.130054	AK127170
SELT	4.7	1.6	selenoprotein T (SELT), mRNA [NM_016275]	NM_016275	Hs.369052	NM_016275
C14orf153	4.7	1.6	chromosome 14 open reading frame 153 (C14orf153), mRNA [NM_032374]	NM_032374	Hs.656616	NM_032374
ST6GALNAC5	4.7	2.5	ST6 (alpha-N-acetyl-neuraminyl-2,3-beta-galactosyl-1,3)-N-acetylgalactosaminide alpha-2,6-sialyltransferase 5 (ST6GALNAC5), mRNA [NM_030965]	NM_030965	Hs.303609	AK056241
TNFRSF11B	4.7	7.0	tumor necrosis factor receptor superfamily, member 11b (osteoprotegerin) (TNFRSF11B), mRNA [NM_002546]	NM_002546	Hs.81791	NM_002546
VDR	4.7	5.7	vitamin D (1,25- dihydroxyvitamin D3) receptor (VDR), transcript variant 2, mRNA [NM_001017535]	NM_001017535	Hs.524368	NM_001017535
HIP1R	4.7	2.1	huntingtin interacting protein 1 related (HIP1R), mRNA [NM_003959]	NM_003959	Hs.654842	NM_003959
CNN1	4.7	4.9	calponin 1, basic, smooth muscle (CNN1), mRNA [NM_001299]	NM_001299	Hs.465929	BC036307
AK094929	4.7	2.0	cDNA FLJ37610 fis, clone BRCOC2011398. [AK094929]	AK094929	Hs.185701	AK094929
SLC27A6	4.7	2.4	solute carrier family 27 (fatty acid transporter), member 6 (SLC27A6), transcript variant 2, mRNA [NM_001017372]	NM_001017372	Hs.49765	BC041945
C1orf144	4.7	1.4	chromosome 1 open reading frame 144 (C1orf144), mRNA [NM_015609]	NM_015609	Hs.252967	AJ295987
BQ233868	4.7	1.9	AGENCOURT_7594186 NIH_MGC_70 cDNA clone IMAGE:6021635 5', mRNA sequence [BQ233868]	BQ233868	Hs.545818	BQ233868
CAV2	4.7	4.0	caveolin 2 (CAV2), transcript variant 1, mRNA [NM_001233]	NM_001233	Hs.212332	NM_001233
VDR	4.7	5.7	vitamin D (1,25- dihydroxyvitamin D3) receptor (VDR), transcript variant 2, mRNA [NM_001017535]	NM_001017535	Hs.524368	NM_001017535
THC2671344	4.6	2.7	Q3MHD6_HUMAN (Q3MHD6) COX17 homolog, cytochrome c oxidase assembly protein, partial (98%) [THC2671344]	THC2671344	Unknown	
PPP1R13L	4.6	2.1	protein phosphatase 1, regulatory (inhibitor) subunit 13 like (PPP1R13L), mRNA [NM_006663]	NM_006663	Hs.466937	AY869712
UBL3	4.6	1.3	ubiquitin-like 3 (UBL3), mRNA [NM_007106]	NM_007106	Hs.145575	BC044582
LRRFIP2	4.6	1.6	leucine rich repeat (in FLII) interacting protein 2 (LRRFIP2), transcript variant 2, mRNA [NM_017724]	NM_017724	Hs.475319	AK124432
CTDP1	4.6	2.5	CTD (carboxy-terminal domain, RNA polymerase II, polypeptide A) phosphatase, subunit 1 (CTDP1), transcript variant FCP1a, mRNA [NM_004715]	NM_004715	Hs.465490	NM_004715
TMEFF2	4.6	3.7	transmembrane protein with EGF-like and two follistatin-like domains 2 (TMEFF2), mRNA [NM_016192]	NM_016192	Hs.144513	DQ133599
SURF4	4.6	2.1	surfeit 4 (SURF4), mRNA [NM_033161]	NM_033161	Hs.696048	NM_033161
ATP6V0C	4.6	2.4	ATPase, H+ transporting, lysosomal 16kDa, V0 subunit c (ATP6V0C), mRNA [NM_001694]	NM_001694	Hs.389107	BM467334
KRT18	4.6	2.4	keratin 18 (KRT18), transcript variant 1, mRNA [NM_000224]	NM_000224	Hs.406013	CR616919
QPCT	4.6	3.1	glutaminyl-peptide cyclotransferase (glutaminyl cyclase) (QPCT), mRNA [NM_012413]	NM_012413	Hs.79033	NM_012413
NIPA1	4.6	2.4	non imprinted in Prader-Willi/Angelman syndrome 1 (NIPA1), mRNA [NM_144599]	NM_144599	Hs.511797	NM_144599
TSPAN2	4.6	3.0	tetraspanin 2 (TSPAN2), mRNA [NM_005725]	NM_005725	Hs.310458	NM_005725
ZBTB7B	4.6	2.3	Zinc finger and BTB domain-containing protein 7B (Zinc finger protein 67 homolog) (Zfp-67) (Zinc finger protein Th-POK) (T-helper-inducing POZ/Krueppel-like factor) (Krueppel-related zinc finger protein cKrox) (hcKrox) (Zinc finger and BTB...	ENST00000368426	Unknown	
FAM129B	4.6	2.6	MEG3 (MEG3) mRNA, complete cds. [AF151783]	AF151783	Hs.522401	NM_022833
MGAT2	4.6	1.5	mannosyl (alpha-1,6-)-glycoprotein beta-1,2-N-acetylglucosaminyltransferase (MGAT2), transcript variant 1, mRNA [NM_002408]	NM_002408	Hs.93338	NM_002408
ARPC4	4.6	2.1	actin related protein 2/3 complex, subunit 4, 20kDa (ARPC4), transcript variant 1, mRNA [NM_005718]	NM_005718	Hs.323342	AK125875
TRIM26	4.6	1.6	tripartite motif-containing 26 (TRIM26), mRNA [NM_003449]	NM_003449	Hs.485041	NM_003449
PPM1H	4.6	2.0	mRNA for KIAA1157 protein, partial cds. [AB032983]	AB032983	Hs.435479	NM_020700
VDR	4.6	5.9	vitamin D (1,25- dihydroxyvitamin D3) receptor (VDR), transcript variant 2, mRNA [NM_001017535]	NM_001017535	Hs.524368	NM_001017535
A_32_P15767	4.6	2.6	A_32_P157671	A_32_P157671	Unknown	
AK000085	4.6	2.3	cDNA FLJ20078 fis, clone COL02974. [AK000085]	AK000085	Hs.610959	AK000085
SDSL	4.6	2.1	serine dehydratase-like (SDSL), mRNA [NM_138432]	NM_138432	Hs.337594	BC009849
MAPKAPK2	4.6	2.1	mitogen-activated protein kinase-activated protein kinase 2 (MAPKAPK2), transcript variant 1, mRNA [NM_004759]	NM_004759	Hs.643566	NM_004759
EPSTI1	4.6	3.2	epithelial stromal interaction 1 (breast) (EPSTI1), transcript variant 2, mRNA [NM_033255]	NM_033255	Hs.546467	AL831953
MICA	4.6	1.8	MHC class I polypeptide-related sequence A (MICA), mRNA [NM_000247]	NM_000247	Hs.549053	AK094237
MIR16	4.6	1.6	membrane interacting protein of RGS16 (MIR16), mRNA [NM_016641]	NM_016641	Hs.512607	NM_016641
SYT14	4.6	4.0	synaptotagmin XIV (SYT14), mRNA [NM_153262]	NM_153262	Hs.658866	AJ617624
LOC391803	4.6	2.2	PREDICTED: similar to Keratin, type I cytoskeletal 18 (Cytokeratin-18) (CK-18) (Keratin-18) (K18) (LOC391803), mRNA [XR_018462]	XR_018462	Hs.646957	XR_018462
CA313037	4.6	2.0	CA313037 UI-CF-FN0-aex-g-14-0-UI.s1 UI-CF-FN0 cDNA clone UI-CF-FN0-aex-g-14-0-UI 3', mRNA sequence [CA313037]	CA313037	Hs.437422	BX537978
IFRD1	4.6	1.9	interferon-related developmental regulator 1 (IFRD1), transcript variant 2, mRNA [NM_001007245]	NM_001007245	Hs.7879	NM_001007245
PGM2L1	4.6	1.8	phosphoglucomutase 2-like 1 (PGM2L1), mRNA [NM_173582]	NM_173582	Hs.26612	NM_173582

CCDC19	4.6	3.6	coiled-coil domain containing 19 (CCDC19), mRNA [NM_012337]	NM_012337	Hs.647705	BC089391
SHC1	4.6	2.4	SHC (Src homology 2 domain containing) transforming protein 1 (SHC1), transcript variant 1, mRNA [NM_183001]	NM_183001	Hs.433795	AB208849
ENST0000025	4.6	1.9	Uncharacterized protein KIAA0819. [Source:Uniprot/SWISSPROT;Acc:O94909] [ENST00000252134]	ENST00000252134	Unknown	
KDELR2	4.6	1.7	KDEL (Lys-Asp-Glu-Leu) endoplasmic reticulum protein retention receptor 2 (KDELR2), mRNA [NM_006854]	NM_006854	Hs.654552	NM_006854
WDR63	4.6	2.7	WD repeat domain 63 (WDR63), mRNA [NM_145172]	NM_145172	Hs.97933	BX648851
FGF1	4.6	6.8	fibroblast growth factor 1 (acidic) (FGF1), transcript variant 1, mRNA [NM_000800]	NM_000800	Hs.483635	NM_000800
BQ019626	4.6	2.3	BQ019626 UI-H-ED0-axd-f-18-0-UI.s1 NCI_CGAP_ED0 cDNA clone IMAGE:5827217 3', mRNA sequence [BQ019626]	BQ019626	Hs.504687	BM473095
A_24_P42071	4.6	1.7	A_24_P42071	A_24_P42071	Unknown	
LOC286382	4.6	1.6	cDNA FLJ37037 fis, clone BRACE2011611. [AK094356]	AK094356	Hs.668691	AK094356
CKMT1A	4.6	1.9	creatine kinase, mitochondrial 1A (CKMT1A), nuclear gene encoding mitochondrial protein, mRNA [NM_001015001]	NM_001015001	Hs.425633	AK094322
BSCL2	4.6	2.4	Bernardinelli-Seip congenital lipodystrophy 2 (seipin) (BSCL2), mRNA [NM_032667]	NM_032667	Hs.533709	DQ470474
ITGB1	4.6	2.0	integrin, beta 1 (fibronectin receptor, beta polypeptide, antigen CD29 includes MDF2, MSK12) (ITGB1), transcript variant 1E, mRNA [NM_133376]	NM_133376	Hs.695946	NM_002211
FJX1	4.6	2.2	four jointed box 1 (Drosophila) (FJX1), mRNA [NM_014344]	NM_014344	Hs.39384	AJ245599
CAMTA1	4.5	1.9	calmodulin binding transcription activator 1 (CAMTA1), mRNA [NM_015215]	NM_015215	Hs.397705	NM_015215
TAGLN2	4.5	3.9	transgelin 2 (TAGLN2), mRNA [NM_003564]	NM_003564	Hs.517168	CR620277
IRF3	4.5	1.8	interferon regulatory factor 3 (IRF3), mRNA [NM_001571]	NM_001571	Hs.75254	AK057577
CR595522	4.5	1.7	full-length cDNA clone CS0DC001YI12 of Neuroblastoma Cot 25-normalized of (human). [CR595522]	CR595522	Hs.637017	NM_005909
CHST12	4.5	1.6	carbohydrate (chondroitin 4) sulfotransferase 12 (CHST12), mRNA [NM_018641]	NM_018641	Hs.213088	BC015954
ICAM1	4.5	6.4	intercellular adhesion molecule 1 (CD54), human rhinovirus receptor (ICAM1), mRNA [NM_000201]	NM_000201	Hs.700573	BC015969
LANCL3	4.5	13.9	LanC lantibiotic synthetase component C-like 3 (bacterial) (LANCL3), mRNA [NM_198511]	NM_198511	Hs.521932	AK124915
LOC442421	4.5	2.3	similar to prostaglandin E receptor 4, subtype EP4; PGE receptor, EP4 subtype; prostaglandin E2 receptor, mRNA (cDNA clone IMAGE:4830816), partial cds. [BC042539]	BC042539	Hs.585349	BC068605
CDKN2B	4.5	17.8	cyclin-dependent kinase inhibitor 2B (p15, inhibits CDK4) (CDKN2B), transcript variant 2, mRNA [NM_078487]	NM_078487	Hs.72901	NM_078487
KLHDC8B	4.5	2.0	kelch domain containing 8B (KLHDC8B), mRNA [NM_173546]	NM_173546	Hs.13781	NM_173546
VDR	4.5	5.5	vitamin D (1,25- dihydroxyvitamin D3) receptor (VDR), transcript variant 2, mRNA [NM_001017535]	NM_001017535	Hs.524368	NM_001017535
ENDOD1	4.5	3.0	Endonuclease domain-containing 1 protein precursor (EC 3.1.30.-). [Source:Uniprot/SWISSPROT;Acc:O94919] [ENST00000278505]	ENST00000278505	Unknown	
LOC399956	4.5	2.9	PREDICTED: similar to Keratin, type II cytoskeletal 8 (Cytokeratin-8) (CK-8) (Keratin-8) (K8) (LOC399956), mRNA [XR_018724]	XR_018724	Hs.595921	AK095401
LOC442249	4.5	2.4	PREDICTED: hypothetical LOC442249 (LOC442249), mRNA [XR_019231]	XR_019231	Hs.651269	XR_019231
DVL1	4.5	2.0	dishevelled, dsh homolog 1 (Drosophila) (DVL1), transcript variant 2, mRNA [NM_181870]	NM_181870	Unknown	
ZP4	4.5	2.7	mRNA; cDNA DKFZp434B2115 (from clone DKFZp434B2115). [AL122093]	AL122093	Hs.661736	AL122093
KIAA1949	4.5	2.2	KIAA1949, mRNA (cDNA clone MGC:71094 IMAGE:5575643), complete cds. [BC066644]	BC066644	Hs.696054	AK124880
DUSP18	4.5	2.7	dual specificity phosphatase 18 (DUSP18), mRNA [NM_152511]	NM_152511	Hs.517544	BC028724
KIAA0562	4.5	2.1	KIAA0562 (KIAA0562), mRNA [NM_014704]	NM_014704	Hs.509017	NM_014704
ATP13A2	4.5	2.3	ATPase type 13A2 (ATP13A2), mRNA [NM_022089]	NM_022089	Hs.128866	AY461712
CCND1	4.5	4.8	cyclin D1 (CCND1), mRNA [NM_053056]	NM_053056	Hs.523852	NM_053056
NUDT18	4.5	2.2	nudix (nucleoside diphosphate linked moiety X)-type motif 18 (NUDT18), mRNA [NM_024815]	NM_024815	Hs.527101	AK124446
BC028022	4.5	1.6	Homo sapiens, clone IMAGE:5218412, mRNA. [BC028022]	BC028022	Hs.659259	AK124475
ACTR3	4.5	2.1	ARP3 actin-related protein 3 homolog (yeast) (ACTR3), mRNA [NM_005721]	NM_005721	Hs.433512	BC044590
ENC1	4.5	2.1	ectodermal-neural cortex (with BTB-like domain) (ENC1), mRNA [NM_003633]	NM_003633	Hs.104925	AF059611
TRIM56	4.5	1.9	tripartite motif-containing 56 (TRIM56), mRNA [NM_030961]	NM_030961	Hs.521092	BC005847
LOC401076	4.5	2.8	PREDICTED: similar to Adenylyl cyclase-associated protein 1 (CAP 1) (LOC401076), mRNA [XR_018154]	XR_018154	Hs.646830	XR_018154
THC2613527	4.5	1.6	THC2613527	THC2613527	Unknown	
KIAA0319L	4.5	2.3	KIAA0319-like (KIAA0319L), transcript variant 1, mRNA [NM_024874]	NM_024874	Hs.456507	NM_182686
YWHAH	4.5	1.6	tyrosine 3-monooxygenase/tryptophan 5-monooxygenase activation protein, eta polypeptide (YWHAH), mRNA [NM_003405]	NM_003405	Hs.226755	CR622695
NADK	4.5	2.1	NAD kinase (NADK), mRNA [NM_023018]	NM_023018	Hs.654792	AF250320
OGDH	4.5	1.9	oxoglutarate (alpha-ketoglutarate) dehydrogenase (lipoamide) (OGDH), nuclear gene encoding mitochondrial protein, transcript variant 1, mRNA [NM_002541]	NM_002541	Hs.488181	NM_002541
TMEM25	4.5	2.0	transmembrane protein 25 (TMEM25), mRNA [NM_032780]	NM_032780	Hs.564188	AK124814
TSTA3	4.5	2.2	tissue specific transplantation antigen P35B (TSTA3), mRNA [NM_003313]	NM_003313	Hs.404119	AK096752

THBS1	4.5	4.1	thrombospondin 1 (THBS1), mRNA [NM_003246]	NM_003246	Hs.164226	NM_003246
BC053363	4.5	2.4	cDNA clone IMAGE:6141118, partial cds. [BC053363]	BC053363	Hs.649764	NM_152219
COPB2	4.5	1.6	coatomer protein complex, subunit beta 2 (beta prime) (COPB2), mRNA [NM_004766]	NM_004766	Hs.75724	AK128561
KITLG	4.5	2.7	KIT ligand (KITLG), transcript variant b, mRNA [NM_000899]	NM_000899	Hs.1048	NM_000899
PPP1R15B	4.5	1.9	protein phosphatase 1, regulatory (inhibitor) subunit 15B (PPP1R15B), mRNA [NM_032833]	NM_032833	Hs.304376	BC065280
CYB561D2	4.5	1.8	cytochrome b-561 domain containing 2 (CYB561D2), mRNA [NM_007022]	NM_007022	Hs.149443	BX641103
CD55	4.5	3.1	CD55 molecule, decay accelerating factor for complement (Cromer blood group) (CD55), mRNA [NM_000574]	NM_000574	Hs.527653	NM_000574
PNMA1	4.5	1.4	paraneoplastic antigen MA1 (PNMA1), mRNA [NM_006029]	NM_006029	Hs.194709	NM_006029
ETV7	4.5	18.4	ets variant gene 7 (TEL2 oncogene) (ETV7), mRNA [NM_016135]	NM_016135	Hs.272398	AK122796
ZNF697	4.5	1.9	zinc finger protein 697, mRNA (cDNA clone IMAGE:3050333), complete cds. [BC033126]	BC033126	Hs.381105	NM_001080470
MXRA7	4.5	1.9	matrix-remodelling associated 7 (MXRA7), transcript variant 1, mRNA [NM_001008528]	NM_001008528	Hs.250723	NM_001008528
AW444553	4.5	2.7	AW444553 UI-H-BI3-ajv-c-10-0-UI.s1 NCI_CGAP_Sub5 cDNA clone IMAGE:2732947 3', mRNA sequence [AW444553]	AW444553	Hs.124951	NM_174911
GNPDA2	4.5	1.8	putative glucosamine-6-phosphate isomerase mRNA, complete cds. [AY173948]	AY173948	Hs.21398	AB209904
TIMP2	4.5	2.8	TIMP metallopeptidase inhibitor 2 (TIMP2), mRNA [NM_003255]	NM_003255	Hs.633514	NM_003255
TUBB3	4.5	2.6	tubulin, beta 3 (TUBB3), mRNA [NM_006086]	NM_006086	Hs.511743	AK122757
TXNDC11	4.5	1.6	thioredoxin domain containing 11 (TXNDC11), mRNA [NM_015914]	NM_015914	Hs.313847	BC002856
ENST0000037	4.5	3.8	MHC class I HLA-J antigen (Fragment). [Source:Uniprot/SPTREMBL;Acc:Q30175] [ENST00000376793]	ENST00000376793	Unknown	
ANXA2	4.5	4.8	annexin A2 (ANXA2), transcript variant 2, mRNA [NM_001002857]	NM_001002857	Hs.511605	BF242966
BM702886	4.5	1.8	UI-E-CK1-afi-c-21-0-UI.r1 UI-E-CK1 cDNA clone UI-E-CK1-afi-c-21-0-UI 5', mRNA sequence [BM702886]	BM702886	Hs.637933	BM702886
GALNT10	4.5	2.6	UDP-N-acetyl-alpha-D-galactosamine:polypeptide N-acetylgalactosaminyltransferase 10 (GalNAc-T10) (GALNT10), transcript variant 1, mRNA [NM_198321]	NM_198321	Hs.655011	NM_198321
CAV1	4.5	5.4	caveolin 1, caveolae protein, 22kDa (CAV1), mRNA [NM_001753]	NM_001753	Hs.74034	NM_001753
TREX1	4.5	2.1	three prime repair exonuclease 1 (TREX1), transcript variant 1, mRNA [NM_016381]	NM_016381	Hs.694840	BC030597
RNH1	4.5	3.2	ribonuclease/angiogenin inhibitor 1 (RNH1), transcript variant 1, mRNA [NM_002939]	NM_002939	Hs.530687	AB209196
HSPA4L	4.5	2.2	heat shock 70kDa protein 4-like (HSPA4L), mRNA [NM_014278]	NM_014278	Hs.135554	NM_014278
HD	4.5	2.4	huntingtin (Huntington disease) (HD), mRNA [NM_002111]	NM_002111	Hs.518450	NM_002111
KIAA0363	4.5	2.7	Human mRNA for KIAA0363 gene, partial cds. [AB002361]	ENST00000258775	Unknown	
TOR1AIP1	4.5	1.9	torsin A interacting protein 1 (TOR1AIP1), mRNA [NM_015602]	NM_015602	Hs.496459	NM_015602
DKFZp761E19	4.5	3.8	DKFZp761E198 protein (DKFZp761E198), mRNA [NM_138368]	NM_138368	Hs.591957	NM_138368
RAB21	4.4	1.8	RAB21, member RAS oncogene family (RAB21), mRNA [NM_014999]	NM_014999	Hs.524590	BC009109
POMT2	4.4	1.6	protein-O-mannosyltransferase 2 (POMT2), mRNA [NM_013382]	NM_013382	Hs.132989	AB209114
A_23_P34710	4.4	1.6	A_23_P347100	A_23_P347100	Unknown	
A_24_P28160	4.4	2.3	A_24_P281605	A_24_P281605	Unknown	
PRMT2	4.4	1.7	protein arginine methyltransferase 2 (PRMT2), transcript variant 1, mRNA [NM_206962]	NM_206962	Hs.154163	AK123352
COL8A1	4.4	18.2	collagen, type VIII, alpha 1 (COL8A1), transcript variant 1, mRNA [NM_001850]	NM_001850	Hs.654548	AF170702
A_24_P23487	4.4	2.7	A_24_P234871	A_24_P234871	Unknown	
AR	4.4	3.6	androgen receptor (dihydrotestosterone receptor; testicular feminization; spinal and bulbar muscular atrophy; Kennedy disease) (AR), transcript variant 1, mRNA [NM_000044]	NM_000044	Hs.496240	NM_000044
FLJ20309	4.4	2.6	hypothetical protein FLJ20309 (FLJ20309), mRNA [NM_017759]	NM_017759	Hs.445036	NM_017759
ARRDC1	4.4	2.4	arrestin domain containing 1 (ARRDC1), mRNA [NM_152285]	NM_152285	Hs.12999	CR627251
STXBP6	4.4	4.2	syntaxin binding protein 6 (amisyn) (STXBP6), mRNA [NM_014178]	NM_014178	Hs.508958	AL834346
ITGB1	4.4	2.1	integrin, beta 1 (fibronectin receptor, beta polypeptide, antigen CD29 includes MDF2, MSK12) (ITGB1), transcript variant 1E, mRNA [NM_133376]	NM_133376	Hs.695946	NM_002211
SLC35E1	4.4	2.3	solute carrier family 35, member E1 (SLC35E1), mRNA [NM_024881]	NM_024881	Hs.585896	BX640756
AK055915	4.4	2.0	cDNA FLJ31353 fis, clone MESAN2000264. [AK055915]	AK055915	Hs.696089	AK055915
CCDC87	4.4	2.2	coiled-coil domain containing 87 (CCDC87), mRNA [NM_018219]	NM_018219	Hs.121072	BC034469
FLJ20152	4.4	2.6	hypothetical protein FLJ20152 (FLJ20152), transcript variant 1, mRNA [NM_001034850]	NM_001034850	Hs.481704	NM_001034850
SLC39A12	4.4	7.3	solute carrier family 39 (zinc transporter), member 12 (SLC39A12), mRNA [NM_152725]	NM_152725	Hs.350895	BC035118
ANXA11	4.4	1.6	annexin A11 (ANXA11), transcript variant c, mRNA [NM_145869]	NM_145869	Hs.530291	NM_145869
DVL1	4.4	2.4	dishevelled, dsh homolog 1 (Drosophila) (DVL1), transcript variant 2, mRNA [NM_181870]	NM_181870	Unknown	
YWHAG	4.4	1.3	tyrosine 3-monooxygenase/tryptophan 5-monooxygenase activation protein, gamma polypeptide (YWHAG), mRNA [NM_012479]	NM_012479	Hs.520974	NM_012479
MAPK1	4.4	1.8	mitogen-activated protein kinase 1 (MAPK1), transcript variant 2, mRNA [NM_138957]	NM_138957	Hs.431850	AL157438

ENST0000038	4.4	3.1	HLA class I histocompatibility antigen, Cw-5 alpha chain precursor (MHC class I antigen Cw*5). [Source:Uniprot/SWISSPROT;Acc:Q9TNN7] [ENST00000383518]	ENST00000383518	Unknown	
CAV1	4.4	5.5	caveolin 1, caveolae protein, 22kDa (CAV1), mRNA [NM_001753]	NM_001753	Hs.74034	NM_001753
C1orf108	4.4	1.7	chromosome 1 open reading frame 108 (C1orf108), mRNA [NM_024595]	NM_024595	Hs.293563	AK223276
ENST0000036	4.4	1.5	Uroporphyrinogen-III synthase (EC 4.2.1.75) (UROS) (Uroporphyrinogen- III cosynthetase) (Hydroxymethylbilane hydrolyase [cyclizing]) (UROIIIS). [Source:Uniprot/SWISSPROT;Acc:P10746] [ENST00000368778]	ENST00000368778	Unknown	
IFIH1	4.4	2.8	interferon induced with helicase C domain 1 (IFIH1), mRNA [NM_022168]	NM_022168	Hs.163173	NM_022168
OSGIN2	4.4	1.6	oxidative stress induced growth inhibitor family member 2 (OSGIN2), mRNA [NM_004337]	NM_004337	Hs.436445	AF061326
IL1A	4.4	15.4	interleukin 1, alpha (IL1A), mRNA [NM_000575]	NM_000575	Hs.1722	NM_000575
ITGB1	4.4	2.0	integrin, beta 1 (fibronectin receptor, beta polypeptide, antigen CD29 includes MDF2, MSK12) (ITGB1), transcript variant 1E, mRNA [NM_133376]	NM_133376	Hs.695946	NM_002211
C1orf96	4.4	2.5	chromosome 1 open reading frame 96 (C1orf96), mRNA [NM_145257]	NM_145257	Hs.585011	BC071609
PDLIM1	4.4	6.1	PDZ and LIM domain 1 (elfin) (PDLIM1), mRNA [NM_020992]	NM_020992	Hs.368525	AK091847
JAK2	4.4	1.9	Janus kinase 2 (a protein tyrosine kinase) (JAK2), mRNA [NM_004972]	NM_004972	Hs.656213	AF058925
AR	4.4	3.4	androgen receptor (dihydrotestosterone receptor; testicular feminization; spinal and bulbar muscular atrophy; Kennedy disease) (AR), transcript variant 1, mRNA [NM_000044]	NM_000044	Hs.496240	NM_000044
FSTL1	4.4	2.7	follistatin-like 1 (FSTL1), mRNA [NM_007085]	NM_007085	Hs.699187	BX647421
IBRDC3	4.4	2.1	IBR domain containing 3 (IBRDC3), mRNA [NM_153341]	NM_153341	Hs.591504	AK131439
ANK1	4.4	4.0	ankyrin 1, erythrocytic (ANK1), transcript variant 3, mRNA [NM_000037]	NM_000037	Hs.654438	NM_000037
POLR3H	4.4	1.7	polymerase (RNA) III (DNA directed) polypeptide H (22.9kD) (POLR3H), transcript variant 2, mRNA [NM_001018052]	NM_001018052	Hs.700703	NM_001018050
LOC283970	4.4	2.1	hypothetical protein LOC283970, mRNA (cDNA clone MGC:71024 IMAGE:4403073), complete cds. [BC063633]	BC063633	Hs.513695	BX647358
AI801539	4.4	2.2	to90g03.x1 NCI_CGAP_Gas4 cDNA clone IMAGE:2185588 3' similar to gb:Z23090 HEAT SHOCK 27 KD PROTEIN (HUMAN);, mRNA sequence [AI801539]	AI801539	Hs.606787	AI801539
YIF1A	4.4	1.9	Yip1 interacting factor homolog A (S. cerevisiae) (YIF1A), mRNA [NM_020470]	NM_020470	Hs.446445	BM462434
SLC30A5	4.4	1.4	solute carrier family 30 (zinc transporter), member 5 (SLC30A5), transcript variant 1, mRNA [NM_022902]	NM_022902	Hs.631975	BX537394
ARMC3	4.4	7.2	armadillo repeat containing 3 (ARMC3), mRNA [NM_173081]	NM_173081	Hs.659807	BC039312
BRI3BP	4.4	3.7	BRI3-binding protein (I3-binding protein) (Cervical cancer 1 proto- oncogene-binding protein KG19) (HCCR-1). [Source:Uniprot/SWISSPROT;Acc:Q8WY22] [ENST00000332107]	ENST00000332107	Unknown	
BC062324	4.4	3.1	cDNA clone MGC:71446 IMAGE:5420082, complete cds. [BC062324]	BC062324	Hs.181244	AB209117
SH3D19	4.4	1.4	SH3 domain protein D19 (SH3D19), mRNA [NM_001009555]	NM_001009555	Hs.567725	BX647422
WDR1	4.4	1.7	WD repeat domain 1 (WDR1), transcript variant 1, mRNA [NM_017491]	NM_017491	Hs.128548	BX648190
BG216229	4.4	3.3	BG216229 RST35803 Athersys RAGE Library cDNA, mRNA sequence [BG216229]	BG216229	Hs.127535	BX647549
SMAP1	4.4	1.5	stromal membrane-associated protein 1 (SMAP1), transcript variant 1, mRNA [NM_001044305]	NM_001044305	Hs.485717	NM_001044305
RHOC	4.4	2.9	ras homolog gene family, member C (RHOC), transcript variant 1, mRNA [NM_175744]	NM_175744	Hs.502659	AK094474
ITGB1	4.4	2.0	integrin, beta 1 (fibronectin receptor, beta polypeptide, antigen CD29 includes MDF2, MSK12) (ITGB1), transcript variant 1E, mRNA [NM_133376]	NM_133376	Hs.695946	NM_002211
POLM	4.4	1.5	polymerase (DNA directed), mu (POLM), mRNA [NM_013284]	NM_013284	Hs.596982	BC026306
DNM3	4.4	4.1	dynamin 3 (DNM3), mRNA [NM_015569]	NM_015569	Hs.654775	AL136712
CAV1	4.4	5.6	caveolin 1, caveolae protein, 22kDa (CAV1), mRNA [NM_001753]	NM_001753	Hs.74034	NM_001753
CENTB5	4.4	3.5	centaurin, beta 5 (CENTB5), mRNA [NM_030649]	NM_030649	Hs.535257	AB051503
LOC649843	4.4	2.5	PREDICTED: similar to cytoplasmic beta-actin (LOC649843), mRNA [XR_018979]	XR_018979	Hs.647290	XR_018979
TMEM63A	4.4	2.2	transmembrane protein 63A (TMEM63A), mRNA [NM_014698]	NM_014698	Hs.119387	AB007958
PITPNM1	4.4	5.3	phosphatidylinositol transfer protein, membrane-associated 1 (PITPNM1), mRNA [NM_004910]	NM_004910	Hs.372295	BC022230
ITGB1	4.4	2.0	integrin, beta 1 (fibronectin receptor, beta polypeptide, antigen CD29 includes MDF2, MSK12) (ITGB1), transcript variant 1E, mRNA [NM_133376]	NM_133376	Hs.695946	NM_002211
CD58	4.4	1.9	CD58 molecule (CD58), mRNA [NM_001779]	NM_001779	Hs.34341	AK125710
MAPK1	4.4	1.8	mitogen-activated protein kinase 1 (MAPK1), transcript variant 2, mRNA [NM_138957]	NM_138957	Hs.431850	AL157438
FOXE1	4.4	1.9	forkhead box E1 (thyroid transcription factor 2) (FOXE1), mRNA [NM_004473]	NM_004473	Hs.159234	U89995
CBL	4.4	3.3	Cas-Br-M (murine) ecotropic retroviral transforming sequence (CBL), mRNA [NM_005188]	NM_005188	Hs.504096	NM_005188
RASAL2	4.4	2.7	RAS protein activator like 2 (RASAL2), transcript variant 2, mRNA [NM_170692]	NM_170692	Hs.656823	NM_170692
PFN2	4.4	2.6	profilin 2 (PFN2), transcript variant 1, mRNA [NM_053024]	NM_053024	Hs.91747	BC043646
NMI	4.4	2.8	N-myc (and STAT) interactor (NMI), mRNA [NM_004688]	NM_004688	Hs.54483	AK124323
GFRA1	4.4	1.5	GDNF family receptor alpha 1 (GFRA1), transcript variant 2, mRNA [NM_145793]	NM_145793	Hs.591913	NM_005264
March4	4.4	6.2	membrane-associated ring finger (C3HC4) 4 (MARCH4), mRNA [NM_020814]	NM_020814	Hs.170388	AB037820

Supplemental Table A5

Genes Downregulated in FH vs CM (1451)

SAM FDR 0.1%

Gene Name	Score(d)	Fold Downreg. FH	Description	Acc	UGCluster	UGRepAcc
PMCH	-30.6	84.8	pro-melanin-concentrating hormone (PMCH), mRNA	NM_002674	Hs.694729	BG216766
DNHD3	-27.2	114.5	dynein heavy chain domain 3 (DNHD3), mRNA	NM_020877	Hs.367649	AB040936
BC015449	-25.2	19.5	clone IMAGE:4427279, mRNA.	BC015449	Hs.615028	BC015449
FLJ46266	-21.1	525.0	FLJ46266 protein (FLJ46266), mRNA	NM_207430	Hs.411600	BM473494
DKK2	-19.8	97.8	dickkopf homolog 2 (Xenopus laevis) (DKK2), mRNA	NM_014421	Hs.211869	AF177395
TM4SF4	-19.7	180.5	transmembrane 4 L six family member 4 (TM4SF4), mRNA	NM_004617	Hs.133527	AK055577
C6orf165	-17.1	10.4	CMP-sialic acid transporter (CMP-Sia-Tr) (CMP-SA-Tr) (Solute carrier family 35 member A1).	ENST00000369562	Unknown	
A_23_P121234	-16.6	76.6	A_23_P121234	A_23_P121234	Unknown	
SLC44A5	-16.1	6.7	Choline transporter-like protein 5 (Solute carrier family 44 member 5).	ENST00000370855	Unknown	
CA3	-16.0	55.7	carbonic anhydrase III, muscle specific (CA3), mRNA	NM_005181	Hs.82129	NM_005181
PMCHL1	-14.8	61.8	pro-melanin-concentrating hormone-like 1 protein (PMCHL1) mRNA, complete cds, alternatively spliced.	AY008410	Unknown	
ZIC4	-14.7	30.9	zinc family member 4 protein HZIC4 (ZIC4) mRNA, complete cds.	AF332509	Unknown	
NEFL	-14.6	13.3	neurofilament, light polypeptide 68kDa (NEFL), mRNA	NM_006158	Hs.521461	NM_006158
HS6ST2	-14.5	18.9	heparan sulfate 6-O-sulfotransferase 2 (HS6ST2), transcript variant L, mRNA	NM_001077188	Hs.385956	NM_001077188
GDA	-14.5	21.4	guanine deaminase (GDA), mRNA	NM_004293	Hs.494163	NM_004293
ROPN1L	-13.4	5.2	ropporin 1-like (ROPN1L), mRNA	NM_031916	Hs.381089	BM563234
TEKT2	-13.4	9.4	tektin 2 (testicular) (TEKT2), mRNA	NM_014466	Hs.127111	BC022005
A_32_P429046	-13.1	9.3	A_32_P429046	A_32_P429046	Unknown	
LIX1	-12.7	30.4	Lix1 homolog (mouse) (LIX1), mRNA	NM_153234	Hs.656702	BC036467
LRRN1	-12.5	60.1	leucine rich repeat neuronal 1 (LRRN1), mRNA	NM_020873	Hs.163244	NM_020873
ZFHX4	-12.4	15.2	zinc finger homeodomain 4 (ZFHX4), mRNA	NM_024721	Hs.458973	NM_024721
SLC17A8	-12.3	31.5	solute carrier family 17 (sodium-dependent inorganic phosphate cotransporter), member 8 (SLC17A8), mRNA	NM_139319	Hs.116871	AK128319
AK055302	-12.2	19.4	cDNA FLJ30740 fis, clone FEBRA2000319.	AK055302	Hs.609017	AK055302
CCDC74B	-11.9	6.8	coiled-coil domain containing 74B (CCDC74B), mRNA	NM_207310	Hs.29383	AL133619
ARL4C	-11.7	5.3	ADP-ribosylation factor-like 4C (ARL4C), mRNA	NM_005737	Hs.655990	NM_005737
CNTNAP2	-11.7	42.9	contactin associated protein-like 2 (CNTNAP2), mRNA	NM_014141	Hs.655684	NM_014141
THC2674391	-11.6	6.1	ALU8_HUMAN (P39195) Alu subfamily SX sequence contamination warning entry, partial (8%)	THC2674391	Unknown	
LPGAT1	-11.6	2.9	lysophosphatidylglycerol acyltransferase 1 (LPGAT1), mRNA	NM_014873	Hs.654626	NM_014873
ENST000003406	-11.3	13.7	Novel protein (Fragment).	ENST00000340612	Unknown	
SOST	-11.3	406.5	sclerosteosis (SOST), mRNA	NM_025237	Hs.349204	AY358627
OTX1	-11.2	83.0	orthodenticle homolog 1 (Drosophila) (OTX1), mRNA	NM_014562	Hs.445340	NM_014562
C6orf85	-11.1	7.0	chromosome 6 open reading frame 85, mRNA (cDNA clone IMAGE:3846727), complete cds.	BC022217	Hs.700628	NM_021945
CLIC6	-10.8	213.9	chloride intracellular channel 6 (CLIC6), mRNA	NM_053277	Hs.473695	AF448439
SNTB1	-10.8	5.3	Tax interaction protein 43 mRNA, partial cds.	AF028828	Hs.655236	AK026095
KCNC1	-10.8	21.1	potassium voltage-gated channel, Shaw-related subfamily, member 1 (KCNC1), mRNA	NM_004976	Hs.303870	BC107129
E2F2	-10.7	6.2	E2F transcription factor 2 (E2F2), mRNA	NM_004091	Hs.194333	NM_004091
MSX2P	-10.7	14.2	msh homeobox 2 pseudogene (MSX2P) on chromosome 17	NR_002307	Unknown	
WDR78	-10.6	7.6	WD repeat domain 78 (WDR78), transcript variant 1, mRNA	NM_024763	Hs.49421	AK127011
TCBA1	-10.5	24.2	T-cell lymphoma breakpoint associated target 1 (TCBA1), mRNA	NM_001040214	Hs.656604	NM_001040214
RAB36	-10.5	5.0	RAB36, member RAS oncogene family (RAB36), mRNA	NM_004914	Hs.369557	AB023061
CRISPLD1	-10.4	8.8	cysteine-rich secretory protein LCCL domain containing 1 (CRISPLD1), mRNA	NM_031461	Hs.436542	NM_031461
CCDC67	-10.4	8.4	coiled-coil domain containing 67 (CCDC67), mRNA	NM_181645	Hs.436625	NM_181645
AK092715	-10.4	12.2	cDNA FLJ35396 fis, clone SKNSH2003483.	AK092715	Hs.26409	BC041405
THC2703271	-10.3	4.7	THC2703271	THC2703271	Unknown	
PLAC2	-10.2	38.4	placenta-specific 2	ENST00000312785	Unknown	
STMN1	-10.2	5.2	stathmin 1/oncoprotein 18 (STMN1), transcript variant 1, mRNA	NM_203401	Hs.700575	BX647885
FAM92B	-10.2	4.4	family with sequence similarity 92, member B (FAM92B), mRNA	NM_198491	Hs.125713	AK126284
CDH18	-10.1	13.2	cadherin 18, type 2 (CDH18), mRNA	NM_004934	Hs.317632	BC031051

AF131834	-10.1	8.5	clone 24841 mRNA sequence.	AF131834	Hs.4892	AF131834
LOC286382	-10.1	15.1	cDNA FLJ37037 fis, clone BRACE2011611.	AK094356	Hs.668691	AK094356
SDK2	-10.1	9.4	sidekick homolog 2 (chicken), mRNA (cDNA clone IMAGE:6172764), partial cds.	BC066363	Hs.435719	BC066363
LEMD1	-10.1	33.2	LEM domain containing 1 (LEMD1), mRNA	NM_001001552	Hs.655520	BC036636
FAM3B	-10.0	23.9	family with sequence similarity 3, member B (FAM3B), transcript variant 1, mRNA	NM_058186	Hs.473877	BC036314
LONRF2	-10.0	14.7	LON peptidase N-terminal domain and ring finger 2 (LONRF2), mRNA	NM_198461	Hs.21380	NM_198461
EMX2	-10.0	16.5	empty spiracles homolog 2 (Drosophila) (EMX2), mRNA	NM_004098	Hs.202095	NM_004098
WFIKKN2	-9.9	8.0	WAP, follistatin/kazal, immunoglobulin, kunitz and netrin domain containing 2 (WFIKKN2), mRNA	NM_175575	Hs.211475	NM_175575
NEFL	-9.9	23.3	neurofilament, light polypeptide 68kDa (NEFL), mRNA	NM_006158	Hs.521461	NM_006158
ADRA1B	-9.9	9.2	adrenergic, alpha-1B-, receptor (ADRA1B), mRNA	NM_000679	Hs.368632	NM_000679
TTLL7	-9.8	3.9	Tubulin--tyrosine ligase-like protein 7 (Protein NYD-SP30).	ENST00000370703	Unknown	
DKK2	-9.8	54.1	dickkopf homolog 2 (Xenopus laevis) (DKK2), mRNA	NM_014421	Hs.211869	AF177395
TRPM3	-9.8	6.9	transient receptor potential cation channel, subfamily M, member 3 (TRPM3), transcript variant 9, mRNA	NM_001007471	Hs.47288	NM_001007471
LZTS1	-9.8	26.3	leucine zipper, putative tumor suppressor 1 (LZTS1), mRNA	NM_021020	Hs.521432	AF123659
CACNA2D3	-9.7	8.3	voltage-gated calcium channel alpha(2)delta-3 subunit mRNA, complete cds.	AF516696	Hs.656687	NM_018398
ODZ2	-9.7	7.7	Teneurin-2 (Ten-2) (Tenascin-M2) (Ten-m2) (Protein Odd Oz/ten-m homolog 2).	ENST00000314238	Unknown	
EIF2A	-9.7	4.1	eukaryotic translation initiation factor 2A, 65kDa (EIF2A), mRNA	NM_032025	Hs.655782	NM_032025
BEX2	-9.7	18.0	brain expressed X-linked 2 (BEX2), mRNA	NM_032621	Hs.398989	BC050651
THC2649719	-9.6	5.6	ALU2_HUMAN (P39189) Alu subfamily SB sequence contamination warning entry, partial (8%)	THC2649719	Unknown	
HOOK1	-9.6	10.6	hook homolog 1 (Drosophila) (HOOK1), mRNA	NM_015888	Hs.378836	NM_015888
A_23_P140454	-9.6	4.3	A_23_P140454	A_23_P140454	Unknown	
SYT4	-9.6	141.4	synaptotagmin IV (SYT4), mRNA	NM_020783	Hs.8059	AB037763
A_24_P555510	-9.6	5.4	A_24_P555510	A_24_P555510	Unknown	
LOC497190	-9.5	51.0	secretory protein LOC497190 (LOC497190), mRNA	NM_001011880	Hs.556045	AL833339
KIAA1799	-9.4	3.4	KIAA1799 protein (KIAA1799), mRNA	NM_032437	Hs.652324	AB058702
SLC8A3	-9.4	13.8	solute carrier family 8 (sodium-calcium exchanger), member 3 (SLC8A3), transcript variant c, mRNA	NM_183002	Hs.337696	NM_183002
FREM1	-9.4	18.1	FRAS1 related extracellular matrix 1 (FREM1), mRNA	NM_144966	Hs.50850	BX648240
EMX2OS	-9.3	10.5	empty spiracles homolog 2 (Drosophila) opposite strand (EMX2OS) on chromosome 10	NR_002791	Unknown	
OTUD3	-9.2	4.0	mRNA for KIAA0459 protein, partial cds.	AB007928	Hs.374987	NM_015207
FLRT3	-9.2	7.7	fibronectin leucine rich transmembrane protein 3 (FLRT3), transcript variant 2, mRNA	NM_198391	Hs.41296	AB040902
UGT2B7	-9.1	21.1	UDP glucuronosyltransferase 2 family, polypeptide B7 (UGT2B7), mRNA	NM_001074	Hs.654424	J05428
BEX1	-9.1	20.2	brain expressed, X-linked 1 (BEX1), mRNA	NM_018476	Hs.334370	BM804232
CCDC74B	-9.0	5.3	coiled-coil domain containing 74B (CCDC74B), mRNA	NM_207310	Hs.29383	AL133619
ZMYND10	-9.0	4.0	zinc finger, MYND-type containing 10 (ZMYND10), mRNA	NM_015896	Hs.526735	AK096525
TSGA2	-9.0	41.1	testis specific A2 homolog (mouse) (TSGA2), mRNA	NM_080860	Hs.661069	AK057315
ADCYAP1	-9.0	8.7	Pituitary adenylate cyclase-activating polypeptide precursor (PACAP) [Contains: PACAP-related peptide (PRP-48); Pituitary adenylate cyclase-activating polypeptide 27 (PACAP-27) (PACAP27); Pituitary adenylate cyclase-activating polypeptide 38...	ENST00000269200	Unknown	
TPR	-9.0	4.0	translocated promoter region (to activated MET oncogene) (TPR), mRNA	NM_003292	Hs.279640	NM_003292
AKAP9	-8.9	4.7	A kinase (PRKA) anchor protein (yotiao) 9 (AKAP9), transcript variant 1, mRNA	NM_147171	Hs.651221	NM_147171
KIAA1799	-8.9	3.1	KIAA1799 protein (KIAA1799), mRNA	NM_032437	Hs.652324	AB058702
TPR	-8.9	3.3	translocated promoter region (to activated MET oncogene) (TPR), mRNA	NM_003292	Hs.279640	NM_003292
C14orf132	-8.9	8.2	chromosome 14 open reading frame 132 (C14orf132), mRNA	NM_020215	Unknown	
DIRAS2	-8.8	15.5	DIRAS family, GTP-binding RAS-like 2 (DIRAS2), mRNA	NM_017594	Hs.165636	NM_017594
AK055101	-8.8	9.6	cDNA FLJ30539 fis, clone BRAWH2001255.	AK055101	Hs.486010	CR749795
SS18L1	-8.8	2.8	synovial sarcoma translocation gene on chromosome 18-like 1 (SS18L1), mRNA	NM_198935	Hs.154429	NM_198935
THC2654231	-8.8	2.5	ALU5_HUMAN (P39192) Alu subfamily SC sequence contamination warning entry, partial (8%)	THC2654231	Unknown	
CCDC65	-8.7	5.7	coiled-coil domain containing 65 (CCDC65), mRNA	NM_033124	Hs.512805	AF382188
UBXD3	-8.7	9.1	UBX domain containing 3 (UBXD3), mRNA	NM_152376	Hs.432503	BX648631
CAMTA1	-8.7	6.6	calmodulin binding transcription activator 1 (CAMTA1), mRNA	NM_015215	Hs.397705	NM_015215
NELL1	-8.7	89.9	NEL-like 1 (chicken) (NELL1), mRNA	NM_006157	Hs.657172	AK127805
APC2	-8.7	35.2	adenomatosis polyposis coli 2 (APC2), mRNA	NM_005883	Hs.446376	NM_005883
LOC149351	-8.7	8.3	hypothetical protein LOC149351, mRNA (cDNA clone IMAGE:5273076).	BC036441	Hs.546492	AK022898

MGC33846	-8.6	10.5	hypothetical protein MGC33846 (MGC33846), mRNA	NM_175885	Hs.448218	NM_175885
ADCYAP1	-8.6	41.2	adenylate cyclase activating polypeptide 1 (pituitary) (ADCYAP1), mRNA	NM_001117	Hs.531719	AK095723
DLEC1	-8.6	12.7	deleted in lung and esophageal cancer 1 (DLEC1), transcript variant DLEC1-N1, mRNA	NM_007335	Hs.699962	NM_007335
RIC3	-8.6	6.3	RIC3 isoform d (RIC3) mRNA, complete cds.	AY326436	Hs.655660	AL832601
CBFA2T3	-8.6	5.4	core-binding factor, runt domain, alpha subunit 2; translocated to, 3 (CBFA2T3), transcript variant 1, mRNA	NM_005187	Hs.513811	NM_005187
RP6-213H19.1	-8.5	13.4	serine/threonine protein kinase MST4 (MST4), transcript variant 1, mRNA	NM_016542	Hs.444247	NM_016542
RGS9	-8.5	15.0	regulator of G-protein signalling 9 (RGS9), mRNA	NM_003835	Hs.664380	NM_003835
STX6	-8.5	3.9	cDNA FLJ32095 fis, clone OCBBF2000998.	AK056657	Unknown	
LOC647768	-8.4	3.6	PREDICTED: similar to Tetratricopeptide repeat protein 3 (TPR repeat protein 3) (TPR repeat protein D) (LOC647768), mRNA	XR_018202	Hs.567832	XR_018202
TMEM108	-8.4	3.8	cDNA FLJ37731 fis, clone BRHIP2020743.	AK095050	Hs.191616	AB051477
ENST000003444	-8.3	3.2	MIA3 protein (Fragment).	ENST00000344441	Unknown	
TNRC4	-8.3	5.2	trinucleotide repeat containing 4 (TNRC4), mRNA	NM_007185	Hs.26047	NM_007185
AIM2	-8.2	37.2	absent in melanoma 2 (AIM2), mRNA	NM_004833	Hs.281898	BC010940
FLJ21272	-8.2	3.5	cDNA: FLJ21272 fis, clone COL01753.	AK024925	Hs.612891	AK024925
IGSF4	-8.2	13.5	immunoglobulin superfamily, member 4 (IGSF4), mRNA	NM_014333	Hs.370510	BX641042
AK021744	-8.2	5.0	cDNA FLJ11682 fis, clone HEMBA1004880.	AK021744	Hs.191828	AK021744
THC2564025	-8.2	2.6	BC065015 transmembrane protein 4 {Homo sapiens} (exp=-1; wgp=0; cg=0), partial (8%)	THC2564025	Unknown	
RAI2	-8.1	4.3	retinoic acid induced 2 (RAI2), mRNA	NM_021785	Hs.701485	NM_021785
FANK1	-8.1	4.9	fibronectin type III and ankyrin repeat domains 1 (FANK1), mRNA	NM_145235	Hs.352591	CR627249
BG695979	-8.1	4.6	602658119F1 NCI_CGAP_Skn3 cDNA clone IMAGE:4800772 5', mRNA sequence	BG695979	Hs.594262	BG695979
THC2706230	-8.1	11.5	THC2706230	THC2706230	Unknown	
STAG2	-8.0	4.9	stromal antigen 2 (STAG2), transcript variant 1, mRNA	NM_001042749	Hs.496710	NM_001042749
A_32_P122285	-8.0	2.6	A_32_P122285	A_32_P122285	Unknown	
THC2657736	-7.9	14.6	THC2657736	THC2657736	Unknown	
C6orf111	-7.9	3.8	Splicing factor, arginine/serine-rich 130 (Serine-arginine-rich- splicing regulatory protein 130) (SRrp130) (SR-rich protein) (SR- related protein).	ENST00000369239	Unknown	
HP1BP3	-7.9	2.9	cDNA FLJ13067 fis, clone NT2RP3001712, highly similar to HP1-BP74 protein mRNA.	AK023129	Hs.142442	BC045660
FLJ44048	-7.8	17.9	FLJ44048 protein (FLJ44048), mRNA	NM_207482	Hs.98025	BX648733
SSX2IP	-7.8	6.3	synovial sarcoma, X breakpoint 2 interacting protein (SSX2IP), mRNA	NM_014021	Hs.22587	AB023140
RGPD2	-7.8	9.4	RANBP2-like and GRIP domain containing 2 (RGPD2), mRNA	NM_001078170	Hs.656849	CR749330
BX111592	-7.7	5.1	BX111592 Soares_testis_NHT cDNA clone IMAGp998D162621, mRNA sequence	BX111592	Hs.112603	BX111592
GPA33	-7.7	21.7	glycoprotein A33 (transmembrane) (GPA33), mRNA	NM_005814	Hs.651244	NM_005814
ATP6V1G1	-7.7	2.3	ATPase, H+ transporting, lysosomal 13kDa, V1 subunit G1 (ATP6V1G1), mRNA	NM_004888	Hs.388654	NM_004888
KIAA0574	-7.7	7.0	mRNA for KIAA0574 protein, partial cds.	ENST00000261275	Unknown	
LOC63920	-7.7	2.7	transposon-derived Buster3 transposase-like (LOC63920), mRNA	NM_022090	Hs.529464	NM_022090
BX090520	-7.7	7.2	BX090520 Soares fetal liver spleen 1NFLS cDNA clone IMAGp998J11128 ; IMAGE:126826, mRNA sequence	BX090520	Hs.574305	BX090520
KIAA1505	-7.7	2.9	KIAA1505 protein (KIAA1505), mRNA	NM_020879	Hs.113940	BX649000
GPC4	-7.7	25.9	glypican 4 (GPC4), mRNA	NM_001448	Hs.58367	AF030186
KLHDC8A	-7.6	11.7	kelch domain containing 8A (KLHDC8A), mRNA	NM_018203	Hs.10414	AK096590
OLFM4	-7.6	19.2	olfactomedin 4 (OLFM4), mRNA	NM_006418	Hs.559736	AY358567
THC2652053	-7.6	4.2	THC2652053	THC2652053	Unknown	
PLAC2	-7.6	10.4	placenta-specific 2 (PLAC2), mRNA	NM_153375	Unknown	
THC2724764	-7.6	8.5	THC2724764	THC2724764	Unknown	
MAFB	-7.5	7.2	v-maf musculoaponeurotic fibrosarcoma oncogene homolog B (avian) (MAFB), mRNA	NM_005461	Hs.651210	NM_005461
RAB3C	-7.5	12.7	Ras-related protein Rab-3C.	ENST00000381158	Unknown	
GPR26	-7.5	6.8	G protein-coupled receptor 26 (GPR26), mRNA	NM_153442	Hs.12751	AJ505757
SAMD11	-7.5	13.5	sterile alpha motif domain containing 11 (SAMD11), mRNA	NM_152486	Hs.335293	NM_152486
CXXC4	-7.5	23.0	CXXC finger 4	ENST00000305749	Unknown	
CX3CL1	-7.5	13.0	chemokine (C-X3-C motif) ligand 1 (CX3CL1), mRNA	NM_002996	Hs.531668	AB209037
C21orf62	-7.5	6.7	chromosome 21 open reading frame 62 (C21orf62), mRNA	NM_019596	Hs.517235	NM_019596
DHFR	-7.5	3.9	dihydrofolate reductase (DHFR), mRNA	NM_000791	Hs.648635	NM_000791
LCOR	-7.4	2.9	ligand dependent nuclear receptor corepressor (LCOR), mRNA	NM_032440	Hs.500695	AL834245

C2orf39	-7.4	16.7	chromosome 2 open reading frame 39 (C2orf39), mRNA	NM_145038	Hs.393714	AK128286
ENST000003228	-7.4	9.7	Numb-interacting protein	ENST00000322839	Unknown	
THC2663494	-7.4	8.8	NOC3_YEAST (Q07896) Nucleolar complex-associated protein 3, partial (3%)	THC2663494	Unknown	
THC2553512	-7.4	3.0	THC2553512	THC2553512	Unknown	
CECR4	-7.4	3.9	Cat eye syndrome critical region candidate gene number 4 (CECR4) mRNA, partial cds.	AF307448	Hs.660066	CA413957
RP5-875H10.1	-7.4	3.6	SAM domain containing 1 (LOC389432), mRNA	NM_001030060	Hs.567973	NM_001030060
PHOX2B	-7.4	12.5	paired-like homeobox 2b (PHOX2B), mRNA	NM_003924	Hs.87202	NM_003924
PUM1	-7.4	2.1	pumilio homolog 1 (Drosophila) (PUM1), transcript variant 1, mRNA	NM_001020658	Hs.281707	NM_001020658
RSPO1	-7.4	49.2	R-spondin homolog (Xenopus laevis) (RSPO1), mRNA	NM_001038633	Hs.135015	NM_001038633
A_32_P232437	-7.4	26.5	A_32_P232437	A_32_P232437	Unknown	
CDH6	-7.4	111.9	Cadherin-6 precursor (Kidney-cadherin) (K-cadherin).	ENST00000382216	Unknown	
TMEM2	-7.3	3.7	transmembrane protein 2 (TMEM2), mRNA	NM_013390	Hs.494146	AF137030
MBOAT1	-7.3	4.2	cDNA FLJ16207 fis, clone CTONG2019822.	AK131269	Hs.377830	NM_001080480
AK094929	-7.3	15.8	cDNA FLJ37610 fis, clone BRCOC2011398.	AK094929	Hs.185701	AK094929
PCDH19	-7.3	11.2	protocadherin 19 (PCDH19), mRNA	NM_020766	Hs.4993	CR749278
TPM1	-7.3	4.3	tropomyosin 1 (alpha) (TPM1), transcript variant 5, mRNA	NM_000366	Hs.133892	BX648171
C6orf111	-7.2	3.2	chromosome 6 open reading frame 111 (C6orf111), mRNA	NM_032870	Hs.520287	NM_032870
BAIAP3	-7.2	5.4	BAI1-associated protein 3 (BAIAP3), mRNA	NM_003933	Hs.458427	AL834321
PCDHA11	-7.2	6.8	protocadherin alpha 11 (PCDHA11), transcript variant 1, mRNA	NM_018902	Hs.199343	NM_014005
OCA2	-7.2	64.2	oculocutaneous albinism II (pink-eye dilution homolog, mouse) (OCA2), mRNA	NM_000275	Hs.654411	NM_000275
KIAA0319	-7.2	5.2	KIAA0319 (KIAA0319), mRNA	NM_014809	Hs.26441	NM_014809
AK091348	-7.1	5.8	cDNA FLJ34029 fis, clone FCBBF2003636.	AK091348	Hs.657012	AK091348
THC2690338	-7.1	17.0	Q9SL08_ARATH (Q9SL08) Expressed protein, partial (5%)	THC2690338	Unknown	
FAM70A	-7.1	48.9	family with sequence similarity 70, member A (FAM70A), mRNA	NM_017938	Hs.437563	AF450484
SMPD3	-7.1	26.3	sphingomyelin phosphodiesterase 3, neutral membrane (neutral sphingomyelinase II) (SMPD3), mRNA	NM_018667	Hs.368421	NM_018667
LOC389073	-7.1	31.3	cDNA clone MGC:46742 IMAGE:5730487, complete cds.	ENST00000341287	Unknown	
THC2549494	-7.1	7.7	ALU6_HUMAN (P39193) Alu subfamily SP sequence contamination warning entry, partial (11%)	THC2549494	Unknown	
SPANXA1	-7.1	14.4	sperm protein associated with the nucleus, X-linked, family member A1 (SPANXA1), mRNA	NM_013453	Hs.334464	BF212177
THC2753396	-7.1	6.9	THC2753396	THC2753396	Unknown	
GCC2	-7.1	4.2	GRIP and coiled-coil domain containing 2 (GCC2), transcript variant 1, mRNA	NM_181453	Hs.436505	NM_181453
ZNF536	-7.1	8.8	zinc finger protein 536 (ZNF536), mRNA	NM_014717	Hs.378901	BC150171
ZRANB2	-7.1	3.7	zinc finger, RAN-binding domain containing 2 (ZRANB2), transcript variant 2, mRNA	NM_005455	Hs.194718	CR749325
ENST000003781	-7.1	3.8	Peripheral plasma membrane protein CASK (EC 2.7.11.1) (hCASK) (Calcium/calmodulin-dependent serine protein kinase) (Lin-2 homolog).	ENST00000378179	Unknown	
AK094175	-7.1	4.6	cDNA FLJ36856 fis, clone ASTRO2014863.	AK094175	Hs.133892	BX648171
ZNF644	-7.1	3.0	zinc finger protein 644 (ZNF644), transcript variant 1, mRNA	NM_201269	Hs.173001	BC150177
IGF1	-7.1	4.0	insulin-like growth factor 1 (somatomedin C) (IGF1), mRNA	NM_000618	Hs.160562	NM_000618
ZBTB33	-7.0	4.1	zinc finger and BTB domain containing 33 (ZBTB33), mRNA	NM_006777	Hs.143604	BC042753
NPY	-7.0	19.3	neuropeptide Y (NPY), mRNA	NM_000905	Hs.1832	BF680552
NMNAT2	-7.0	6.9	nicotinamide nucleotide adenylyltransferase 2 (NMNAT2), transcript variant 1, mRNA	NM_015039	Hs.497123	AF288395
PCSK1N	-7.0	5.8	proprotein convertase subtilisin/kexin type 1 inhibitor (PCSK1N), mRNA	NM_013271	Hs.522640	BM805628
ST6GALNAC5	-7.0	15.2	ST6 (alpha-N-acetyl-neuraminyl-2,3-beta-galactosyl-1,3)-N-acetylgalactosaminide alpha-2,6-sialyltransferase 5 (ST6GALNAC5), mRNA	NM_030965	Hs.303609	AK056241
THC2633747	-6.9	4.8	ALU2_HUMAN (P39189) Alu subfamily SB sequence contamination warning entry, partial (3%)	THC2633747	Unknown	
NTF5	-6.9	15.1	neurotrophin 5 (neurotrophin 4/5) (NTF5), mRNA	NM_006179	Hs.266902	NM_006179
APC2	-6.9	15.0	adenomatosis polyposis coli 2 (APC2), mRNA	NM_005883	Hs.446376	NM_005883
THC2766709	-6.9	2.8	ALU1_HUMAN (P39188) Alu subfamily J sequence contamination warning entry, partial (13%)	THC2766709	Unknown	
AK096580	-6.9	5.2	cDNA FLJ39261 fis, clone OCBBF2009391.	AK096580	Hs.13281	AK096580
IGF1	-6.9	4.3	insulin-like growth factor 1 (somatomedin C) (IGF1), mRNA	NM_000618	Hs.160562	NM_000618
A_24_P476718	-6.8	7.2	A_24_P476718	A_24_P476718	Unknown	
THC2718172	-6.8	14.0	THC2718172	THC2718172	Unknown	
TTLL7	-6.8	2.7	tubulin tyrosine ligase-like family, member 7 (TTLL7), mRNA	NM_024686	Hs.445826	NM_024686
TMEM132B	-6.8	6.9	transmembrane protein 132B (TMEM132B), mRNA	NM_052907	Hs.524838	NM_052907

C8ORFK32	-6.8	8.5	C8orfK32 protein (C8ORFK32), mRNA	NM_015912	Hs.126024	NM_015912
C14orf132	-6.8	14.7	chromosome 14 open reading frame 132 (C14orf132), mRNA	NM_020215	Unknown	
CDR1	-6.8	7.4	cerebellar degeneration-related protein 1, 34kDa (CDR1), mRNA	NM_004065	Hs.571748	AK054921
KIF5C	-6.8	15.2	kinesin family member 5C (KIF5C), mRNA	NM_004522	Hs.660699	NM_004522
EFCAB1	-6.8	56.7	EF-hand calcium binding domain 1 (EFCAB1), mRNA	NM_024593	Hs.23245	NM_024593
PNMA3	-6.7	4.9	paraneoplastic antigen MA3 (PNMA3), mRNA	NM_013364	Hs.449627	NM_013364
CASC1	-6.7	25.7	cancer susceptibility candidate 1 (CASC1), mRNA	NM_018272	Hs.407771	NM_001082972
GPRIN2	-6.7	11.7	G protein-regulated inducer of neurite outgrowth 2 (GRIN2).	ENST00000374314	Unknown	
RP11-50D16.3	-6.7	3.7	full-length cDNA clone CS0DC001YJ02 of Neuroblastoma Cot 25-normalized of (human).	CR601315	Hs.507783	NM_001012754
THC2673265	-6.7	3.8	Q6IHS4_DROME (Q6IHS4) HDC01261, partial (9%)	THC2673265	Unknown	
ENST000003681	-6.7	3.2	ETS translocation variant 3 (ETS-domain transcriptional repressor PE1) (PE-1) (Mitogenic Ets transcriptional suppressor).	ENST00000368192	Unknown	
TRIM33	-6.7	3.1	tripartite motif-containing 33 (TRIM33), transcript variant b, mRNA	NM_033020	Hs.26837	NM_015906
LRRN5	-6.7	29.1	leucine rich repeat neuronal 5 (LRRN5), transcript variant 2, mRNA	NM_201630	Hs.26312	AF030435
STXBP3	-6.7	2.6	syntaxin binding protein 3 (STXBP3), mRNA	NM_007269	Hs.530436	NM_007269
THC2652307	-6.7	2.7	ALU8_HUMAN (P39195) Alu subfamily SX sequence contamination warning entry, partial (10%)	THC2652307	Unknown	
AK022030	-6.7	3.2	cDNA FLJ11968 fis, clone HEMBB1001133.	AK022030	Hs.288178	CR936683
MAF	-6.7	11.3	short form transcription factor C-MAF (c-maf) mRNA, complete cds.	AF055376	Hs.699396	NM_001031804
AL049990	-6.7	23.9	mRNA; cDNA DKFZp564G112 (from clone DKFZp564G112).	AL049990	Hs.90250	NM_024574
EIF2C4	-6.6	2.5	Eukaryotic translation initiation factor 2C 4 (eIF2C 4) (eIF-2C 4) (Argonaute-4).	ENST00000373210	Unknown	
SLC10A4	-6.6	12.0	solute carrier family 10 (sodium/bile acid cotransporter family), member 4 (SLC10A4), mRNA	NM_152679	Hs.132591	BC019066
USP33	-6.6	2.3	ubiquitin specific peptidase 33 (USP33), transcript variant 1, mRNA	NM_015017	Hs.480597	NM_015017
THC2665111	-6.6	7.7	THC2665111	THC2665111	Unknown	
ENST000003036	-6.6	25.8	CDNA FLJ46154 fis, clone TESTI4001348.	ENST00000303697	Unknown	
LOC440585	-6.6	13.1	LOC440585	ENST00000335282	Unknown	
IGF1	-6.6	3.9	insulin-like growth factor 1 (somatomedin C) (IGF1), mRNA	NM_000618	Hs.160562	NM_000618
MSX2	-6.6	32.9	msh homeobox 2 (MSX2), mRNA	NM_002449	Hs.89404	NM_002449
MXRA8	-6.6	8.9	matrix-remodelling associated 8 (MXRA8), mRNA	NM_032348	Hs.558570	AK095966
MAP9	-6.5	5.7	microtubule-associated protein 9 (MAP9), mRNA	NM_001039580	Hs.61271	NM_001039580
USP47	-6.5	2.7	ubiquitin specific peptidase 47 (USP47), mRNA	NM_017944	Hs.577256	NM_017944
C21orf88	-6.5	25.0	cDNA clone MGC:99480 IMAGE:4874993, complete cds.	BC080530	Hs.375120	BC080530
LOC63920	-6.5	2.9	transposon-derived Buster3 transposase-like (LOC63920), mRNA	NM_022090	Hs.529464	NM_022090
THC2683448	-6.5	3.4	Q7WZG3_PASPI (Q7WZG3) Ferric uptake regulator, partial (8%)	THC2683448	Unknown	
BM802662	-6.5	2.8	AGENCOURT_6460073 NIH_MGC_67 cDNA clone IMAGE:5581075 5', mRNA sequence	BM802662	Hs.586812	BM802662
KIAA1909	-6.5	12.0	KIAA1909 protein (KIAA1909), mRNA	NM_052909	Hs.535800	NM_052909
DMRTC1	-6.5	31.0	DMRT-like family C1 (DMRTC1), mRNA	NM_033053	Hs.658177	NM_033053
THC2527306	-6.5	13.0	Q9HBV3_HUMAN (Q9HBV3) PP3111, partial (17%)	THC2527306	Unknown	
LY6H	-6.4	13.3	lymphocyte antigen 6 complex, locus H (LY6H), mRNA	NM_002347	Hs.159590	BC053355
ENST000003247	-6.4	2.5	Plasminogen-related protein A (Fragment).	ENST00000324709	Unknown	
A_32_P176790	-6.4	9.2	A_32_P176790	A_32_P176790	Unknown	
IGF1	-6.4	4.7	insulin-like growth factor 1 (somatomedin C) (IGF1), mRNA	NM_000618	Hs.160562	NM_000618
HCN3	-6.4	4.0	hyperpolarization activated cyclic nucleotide-gated potassium channel 3 (HCN3), mRNA	NM_020897	Hs.696550	NM_020897
STOML3	-6.4	6.3	stomatin (EPB72)-like 3 (STOML3), mRNA	NM_145286	Hs.327794	BC025760
RGPD1	-6.4	11.9	RANBP2-like and GRIP domain containing 1 (RGPD1), mRNA	NM_001024457	Hs.656849	CR749330
AF289562	-6.4	7.4	clone pp6337 unknown mRNA.	AF289562	Hs.276808	NM_002409
NAP1L2	-6.4	7.9	nucleosome assembly protein 1-like 2 (NAP1L2), mRNA	NM_021963	Hs.66180	NM_021963
GOLGA8A	-6.4	4.2	golgi autoantigen, golgin subfamily a, 8A (GOLGA8A), mRNA	NM_181077	Hs.182982	BX648160
AL080082	-6.4	8.2	mRNA; cDNA DKFZp564G1162 (from clone DKFZp564G1162).	AL080082	Hs.598166	AL080082
HECTD2	-6.4	3.1	HECT domain containing 2 (HECTD2), transcript variant 2, mRNA	NM_173497	Hs.656960	BC040187
RHOH	-6.4	6.0	ras homolog gene family, member H (RHOH), mRNA	NM_004310	Hs.694951	NM_004310
AP3M2	-6.4	2.0	adaptor-related protein complex 3, mu 2 subunit (AP3M2), mRNA	NM_006803	Hs.654529	BX648390
STAG2	-6.4	3.7	stromal antigen 2 (STAG2), transcript variant 1, mRNA	NM_001042749	Hs.496710	NM_001042749
C7orf29	-6.3	5.6	chromosome 7 open reading frame 29 (C7orf29), mRNA	NM_138434	Hs.655915	AL832660
IDS	-6.3	3.1	iduronate 2-sulfatase (Hunter syndrome) (IDS), transcript variant 1, mRNA	NM_000202	Hs.460960	NM_000202

CAMTA1	-6.3	5.2	calmodulin binding transcription activator 1 (CAMTA1), mRNA	NM_015215	Hs.397705	NM_015215
PLEKHA6	-6.3	8.4	pleckstrin homology domain containing, family A member 6 (PLEKHA6), mRNA	NM_014935	Hs.253146	NM_014935
FGFR2	-6.3	29.3	fibroblast growth factor receptor 2 (bacteria-expressed kinase, keratinocyte growth factor receptor, craniofacial dysostosis 1, Crouzon syndrome, Pfeiffer syndrome, Jackson-Weiss syndrome) (FGFR2), transcript variant 2, mRNA	NM_022970	Hs.533683	NM_022970
THC2741040	-6.3	15.3	CB243285 UI-CF-FN0-agc-l-12-0-UI.s1 UI-CF-FN0 cDNA clone UI-CF-FN0-agc-l-12-0-UI 3', mRNA sequence	THC2741040	Unknown	
FAM81B	-6.3	71.1	family with sequence similarity 81, member B (FAM81B), mRNA	NM_152548	Hs.276287	BC034772
C8orf16	-6.3	4.4	mRNA for hypothetical protein (C8ORF16).	AJ312026	Hs.700167	AJ312026
A_32_P139260	-6.3	4.7	A_32_P139260	A_32_P139260	Unknown	
KIF6	-6.3	4.7	kinesin family member 6 (KIF6), mRNA	NM_145027	Hs.588202	AL832634
GLCCI1	-6.3	4.4	glucocorticoid induced transcript 1 (GLCCI1), mRNA	NM_138426	Hs.131673	NM_138426
SMA5	-6.2	3.3	SMA5 (SMA5), mRNA	NM_021036	Unknown	
IPO9	-6.2	2.4	importin 9 (IPO9), mRNA	NM_018085	Hs.596014	NM_018085
ANKRD45	-6.2	6.2	ankyrin repeat domain 45 (ANKRD45), mRNA	NM_198493	Hs.130054	AK127170
FLJ46266	-6.2	65.4	CDNA FLJ46266 fis, clone TESTI4027139.	ENST00000332814	Unknown	
ARID4B	-6.2	2.8	AT rich interactive domain 4B (RBP1-like) (ARID4B), transcript variant 1, mRNA	NM_016374	Hs.575782	NM_016374
EYA4	-6.2	11.6	Eyes absent homolog 4 (EC 3.1.3.48).	ENST00000367895	Unknown	
FLJ10357	-6.2	7.6	hypothetical protein FLJ10357 (FLJ10357), mRNA	NM_018071	Hs.35125	NM_018071
BSN	-6.2	5.5	bassoon (presynaptic cytomatrix protein) (BSN), mRNA	NM_003458	Hs.696391	AF052224
RAB3GAP2	-6.2	2.5	Rab3 GTPase-activating protein non-catalytic subunit (Rab3 GTPase- activating protein 150 kDa subunit) (Rab3-GAP p150) (Rab3-GAP regulatory subunit) (RAB3-GAP150) (RGAP-iso).	ENST00000358951	Unknown	
BBOX1	-6.2	12.1	butyrobetaine (gamma), 2-oxoglutarate dioxygenase (gamma-butyrobetaine hydroxylase) 1 (BBOX1), mRNA	NM_003986	Hs.591996	NM_003986
PBX4	-6.2	18.5	pre-B-cell leukemia transcription factor 4 (PBX4), mRNA	NM_025245	Hs.466257	BC141859
KCNQ2	-6.2	13.1	Potassium voltage-gated channel subfamily KQT member 2 (Voltage-gated potassium channel subunit Kv7.2) (Neuroblastoma-specific potassium channel subunit alpha KvLQT2) (KQT-like 2).	ENST00000370237	Unknown	
SUHW3	-6.1	5.6	suppressor of hairy wing homolog 3 (Drosophila) (SUHW3), mRNA	NM_017666	Hs.308418	BC051728
FBXO2	-6.1	6.3	F-box protein 2 (FBXO2), mRNA	NM_012168	Hs.132753	NM_012168
PLGLB1	-6.1	2.4	plasminogen-like B1 (PLGLB1), mRNA	NM_001032392	Hs.652174	NM_001032392
A_23_P156609	-6.1	5.2	A_23_P156609	A_23_P156609	Unknown	
CNTN4	-6.1	5.3	contactin 4 (CNTN4), transcript variant 1, mRNA	NM_175607	Hs.298705	NM_175607
RHOU	-6.1	7.0	ras homolog gene family, member U (RHOU), mRNA	NM_021205	Hs.647774	NM_021205
H2AFY	-6.1	2.4	H2A histone family, member Y (H2AFY), transcript variant 3, mRNA	NM_138610	Hs.696013	AB209490
DPP10	-6.1	10.9	dipeptidyl-peptidase 10 (DPP10), transcript variant 1, mRNA	NM_020868	Hs.591555	BC030832
CXXC5	-6.1	5.9	CXXC finger 5 (CXXC5), mRNA	NM_016463	Hs.189119	BC006428
LOC728555	-6.1	4.1	cDNA FLJ40901 fis, clone UTERU2003704.	AK098220	Hs.664334	AK098220
SLC6A2	-6.1	15.1	mRNA for norepinephrine transporter isoform 2, partial cds.	AB022847	Hs.78036	NM_001043
FBXO15	-6.1	5.4	F-box protein 15 (FBXO15), mRNA	NM_152676	Hs.664011	AK093252
USP34	-6.1	5.0	mRNA; cDNA DKFZp586J101 (from clone DKFZp586J101).	AL050376	Unknown	
GDAP1L1	-6.1	9.1	ganglioside-induced differentiation-associated protein 1-like 1 (GDAP1L1), mRNA	NM_024034	Hs.517059	NM_024034
C20orf177	-6.1	2.9	Uncharacterized protein C20orf177.	ENST00000358293	Unknown	
HIVEP3	-6.1	3.1	human immunodeficiency virus type I enhancer binding protein 3	ENST00000372583	Unknown	
THC2533546	-6.1	6.7	XM_750378 rRNA assembly protein Mis3 {Aspergillus fumigatus Af293} (exp=-1; wgp=0; cg=0), partial (6%)	THC2533546	Unknown	
CNTN3	-6.1	21.6	contactin 3 (plasmacytoma associated) (CNTN3), mRNA	NM_020872	Hs.12723	NM_020872
FLJ22795	-6.1	4.0	colon cancer-associated antigen AgSK1-2HT-ECS mRNA, complete cds.	AF316855	Hs.498322	AF316855
LGI1	-6.1	151.8	leucine-rich, glioma inactivated 1 (LGI1), mRNA	NM_005097	Hs.533670	AB209408
C1orf9	-6.1	7.3	chromosome 1 open reading frame 9 (C1orf9), transcript variant 2, mRNA	NM_016227	Hs.204559	NM_016227
ATRNL1	-6.0	22.3	attractin-like 1 (ATRNL1), mRNA	NM_207303	Hs.501127	NM_207303
NEFM	-6.0	86.2	neurofilament, medium polypeptide 150kDa (NEFM), mRNA	NM_005382	Hs.458657	BC096757
ENST000003782	-6.0	2.2	Artemis protein (EC 3.1.-.-) (DNA cross-link repair 1C protein) (SNM1- like protein) (A-SCID protein) (hSNM1C).	ENST00000378289	Unknown	
BX114329	-6.0	3.5	BX114329 BX114329 Soares_NhHMPu_S1 cDNA clone IMAGp998G064741 ; IMAGE:1932317, mRNA sequence	BX114329	Unknown	
DTL	-6.0	3.9	denticleless homolog (Drosophila) (DTL), mRNA	NM_016448	Hs.656473	NM_016448
CBLN1	-6.0	19.8	cerebellin 1 precursor (CBLN1), mRNA	NM_004352	Hs.458423	NM_004352
SLC2A12	-6.0	3.4	solute carrier family 2 (facilitated glucose transporter), member 12 (SLC2A12), mRNA	NM_145176	Hs.486508	AL833602
GTF2IRD2	-6.0	2.1	transcription factor GTF2IRD2 isoform 2 (GTF2IRD2) mRNA, complete cds, alternatively spliced.	AY336979	Hs.647017	NM_173537

THC2652836	-6.0	41.1	THC2652836	THC2652836	Unknown	
AK095564	-6.0	3.4	cDNA FLJ38245 fis, clone FCBBF2007186.	AK095564	Hs.697104	AK095564
ENST000003728	-6.0	4.4	Chromsome 20 open reading frame 119 (Fragment).	ENST00000372821	Unknown	
LZTFL1	-6.0	3.5	leucine zipper transcription factor-like 1 (LZTFL1), mRNA	NM_020347	Hs.30824	BC042483
DOCK11	-6.0	5.5	dedicator of cytokinesis 11 (DOCK11), mRNA	NM_144658	Hs.368203	NM_144658
C6orf165	-5.9	4.3	chromosome 6 open reading frame 165 (C6orf165), transcript variant 1, mRNA	NM_001031743	Hs.82921	NM_178823
SPAG6	-5.9	24.0	sperm associated antigen 6 (SPAG6), transcript variant 1, mRNA	NM_012443	Hs.655170	NM_012443
AK124803	-5.9	2.8	cDNA FLJ42813 fis, clone BRCAN2012355.	AK124803	Hs.498015	AK124803
EPHB1	-5.9	5.0	EPH receptor B1 (EPHB1), mRNA	NM_004441	Hs.116092	NM_004441
NTRK2	-5.9	13.1	neurotrophic tyrosine kinase, receptor, type 2 (NTRK2), transcript variant b, mRNA	NM_001007097	Hs.494312	NM_001018065
LOC90835	-5.9	2.4	hypothetical protein LOC90835 (LOC90835), mRNA	NM_001014979	Hs.65735	AL122109
RHOU	-5.9	17.1	ras homolog gene family, member U (RHOU), mRNA	NM_021205	Hs.647774	NM_021205
KATNAL2	-5.9	9.8	katanin p60 subunit A-like 2 (KATNAL2), mRNA	NM_031303	Hs.404137	NM_031303
BI916497	-5.9	15.5	603182667F1 NIH_MGC_121 cDNA clone IMAGE:5246609 5', mRNA sequence	BI916497	Hs.514795	NM_003787
HBD	-5.9	9.0	hemoglobin, delta (HBD), mRNA	NM_000519	Hs.699280	AY034468
PAK3	-5.9	10.2	p21 (CDKN1A)-activated kinase 3 (PAK3), mRNA	NM_002578	Hs.656789	NM_002578
DTL	-5.9	3.9	denticleless homolog (Drosophila) (DTL), mRNA	NM_016448	Hs.656473	NM_016448
GSTM3	-5.9	3.5	glutathione S-transferase M3 (brain) (GSTM3), mRNA	NM_000849	Hs.2006	NM_000849
NEFH	-5.9	4.8	neurofilament, heavy polypeptide 200kDa (NEFH), mRNA	NM_021076	Hs.198760	AB020652
SPATA13	-5.9	1.9	spermatogenesis associated 13 (SPATA13), mRNA	NM_153023	Hs.657121	BX648244
KIF5A	-5.9	28.8	mRNA for KIF5A variant protein, partial cds, clone: ph00435.	AB210045	Hs.151219	AB210045
PDE8B	-5.9	5.0	phosphodiesterase 8B (PDE8B), transcript variant 1, mRNA	NM_003719	Hs.584830	NM_003719
KBTBD9	-5.9	2.5	Kelch repeat and BTB domain-containing protein 9.	ENST00000288548	Unknown	
HPCAL4	-5.9	7.0	hippocalcin like 4 (HPCAL4), mRNA	NM_016257	Hs.696062	AL136591
ZBTB41	-5.9	4.1	zinc finger and BTB domain containing 41 (ZBTB41), mRNA	NM_194314	Hs.529439	NM_194314
SYNE2	-5.9	3.4	spectrin repeat containing, nuclear envelope 2 (SYNE2), transcript variant 1, mRNA	NM_015180	Hs.525392	NM_182914
GRM8	-5.9	77.7	glutamate receptor, metabotropic 8 (GRM8), mRNA	NM_000845	Hs.449625	NM_000845
TRPM3	-5.8	7.8	transient receptor potential cation channel, subfamily M, member 3 (TRPM3), transcript variant 7, mRNA	NM_206948	Hs.47288	NM_001007471
AV724325	-5.8	5.4	AV724325 HTB cDNA clone HTBAYE05 5', mRNA sequence	AV724325	Hs.701196	AV724325
DTL	-5.8	4.0	denticleless homolog (Drosophila) (DTL), mRNA	NM_016448	Hs.656473	NM_016448
ZDHHC11	-5.8	6.6	zinc finger, DHHC-type containing 11 (ZDHHC11), mRNA	NM_024786	Hs.659832	AK024233
THC2611277	-5.8	5.7	NM_069973 F08G5.6 {Caenorhabditis elegans} (exp=-1; wgp=0; cg=0), partial (5%)	THC2611277	Unknown	
ARHGEF7	-5.8	7.2	Rho guanine nucleotide exchange factor (GEF) 7 (ARHGEF7), transcript variant 2, mRNA	NM_145735	Hs.508738	BX648030
DDX17	-5.8	2.5	DEAD (Asp-Glu-Ala-Asp) box polypeptide 17 (DDX17), transcript variant 1, mRNA	NM_006386	Hs.528305	NM_001098504
DTL	-5.8	4.0	denticleless homolog (Drosophila) (DTL), mRNA	NM_016448	Hs.656473	NM_016448
TSPAN2	-5.8	10.2	tetraspanin 2 (TSPAN2), mRNA	NM_005725	Hs.310458	NM_005725
FLJ40298	-5.8	3.7	hypothetical protein FLJ40298 (FLJ40298), mRNA	NM_173486	Unknown	
HECTD2	-5.8	4.0	HECT domain containing 2 (HECTD2), transcript variant 1, mRNA	NM_182765	Hs.656960	BC040187
DSC1	-5.8	7.2	desmocollin 1 (DSC1), transcript variant Dsc1b, mRNA	NM_004948	Hs.567260	X72925
ENAH	-5.8	4.9	enabled homolog (Drosophila) (ENAH), transcript variant 1, mRNA	NM_001008493	Hs.497893	NM_001008493
TTC3	-5.8	2.4	tetratricopeptide repeat domain 3 (TTC3), transcript variant 1, mRNA	NM_003316	Hs.368214	D84294
LSAMP	-5.8	7.1	limbic system-associated membrane protein (LSAMP), mRNA	NM_002338	Hs.657246	NM_002338
MS4A8B	-5.8	3.9	membrane-spanning 4-domains, subfamily A, member 8B (MS4A8B), mRNA	NM_031457	Hs.150878	AF237905
SUSD4	-5.8	8.2	sushi domain containing 4 (SUSD4), transcript variant 1, mRNA	NM_017982	Hs.497841	AK096265
FOXJ3	-5.8	2.5	forkhead box J3 (FOXJ3), mRNA	NM_014947	Hs.26023	AB028964
TXNDC6	-5.7	4.7	thioredoxin-like 2 mRNA, complete cds.	AF196568	Hs.660992	NM_178130
ENST000003562	-5.7	6.3	CDNA: FLJ20972 fis, clone ADSU01569.	ENST00000356216	Unknown	
KIRREL3	-5.7	5.1	Kin of IRRE-like protein 3 precursor (Kin of irregular chiasm-like protein 3) (Nephrin-like 2).	ENST00000278934	Unknown	
SPANXD	-5.7	7.9	SPANX family, member D (SPANXD), mRNA	NM_032417	Hs.375036	BM554791
LOC149134	-5.7	4.0	hypothetical protein LOC149134 (LOC149134), mRNA	NM_207326	Unknown	
ATP11C	-5.7	3.2	ATPase, Class VI, type 11C (ATP11C), transcript variant 1, mRNA	NM_173694	Hs.88252	NM_173694
CR620293	-5.7	72.9	full-length cDNA clone CS0DF028YD24 of Fetal brain of (human).	CR620293	Hs.696021	AB263414

C19orf57	-5.7	4.4	chromosome 19 open reading frame 57 (C19orf57), mRNA	NM_024323	Hs.143288	NM_024323
THC2726661	-5.7	6.7	T52140 yb29h11.r1 Stratagene fetal spleen (#937205) cDNA clone IMAGE:72645 5', mRNA sequence	THC2726661	Unknown	
DDR1	-5.7	5.4	discoidin domain receptor family, member 1 (DDR1), transcript variant 1, mRNA	NM_013993	Hs.631988	NM_013993
LOC200169	-5.7	10.6	cDNA FLJ23803 fis, clone HEP22811.	AK074383	Hs.159711	NM_001098722
BC035377	-5.7	2.5	cDNA clone IMAGE:4826240.	BC035377	Hs.633960	CD244199
RCOR3	-5.7	3.2	REST corepressor 3 (RCOR3), mRNA	NM_018254	Hs.696152	AK125810
ACYP1	-5.7	2.9	acylphosphatase 1, erythrocyte (common) type (ACYP1), transcript variant 2, mRNA	NM_203488	Hs.18573	BE777513
ENAH	-5.7	2.7	enabled homolog (Drosophila) (ENAH), transcript variant 2, mRNA	NM_018212	Hs.497893	NM_001008493
CLN3	-5.7	2.7	ceroid-lipofuscinosis, neuronal 3, juvenile (Batten, Spielmeyer-Vogt disease), mRNA (cDNA clone MGC:102840 IMAGE:5764535), complete cds.	BC111068	Hs.628393	AK090709
MAK10	-5.6	1.8	MAK10 homolog, amino-acid N-acetyltransferase subunit, (S. cerevisiae) (MAK10), mRNA	NM_024635	Hs.436098	NM_024635
CCDC14	-5.6	3.8	coiled-coil domain containing 14 (CCDC14), mRNA	NM_022757	Hs.656256	BX537652
AK097080	-5.6	6.7	cDNA FLJ39761 fis, clone SPLEN1000083.	AK097080	Hs.534942	XR_017612
ZNF10	-5.6	3.7	zinc finger protein 10 (ZNF10), mRNA	NM_015394	Hs.507355	BC024182
UPF3B	-5.6	3.4	UPF3 regulator of nonsense transcripts homolog B (yeast) (UPF3B), transcript variant 1, mRNA	NM_080632	Hs.103832	NM_080632
AK128756	-5.6	22.4	cDNA FLJ44869 fis, clone BRAMY2015516.	AK128756	Hs.632969	AK128756
C20orf119	-5.6	4.3	chromosome 20 open reading frame 119, mRNA (cDNA clone IMAGE:4745538), with apparent retained intron.	BC039151	Hs.641481	AK124047
TTC18	-5.6	17.3	tetratricopeptide repeat domain 18 (TTC18), mRNA	NM_145170	Hs.591367	AL833537
THC2643957	-5.6	13.5	THC2643957	THC2643957	Unknown	
JMJD1C	-5.6	2.1	jumonji domain containing 1C (JMJD1C), transcript variant 2, mRNA	NM_004241	Hs.413416	NM_032776
NOL4	-5.6	17.8	nucleolar protein 4 (NOL4), mRNA	NM_003787	Hs.514795	NM_003787
THC2705799	-5.6	37.7	ALU1_HUMAN (P39188) Alu subfamily J sequence contamination warning entry, partial (4%)	THC2705799	Unknown	
CR622844	-5.6	1.8	full-length cDNA clone CS0DF026YC16 of Fetal brain of (human).	CR622844	Hs.449098	BM906393
MAP9	-5.6	5.4	microtubule-associated protein 9 (MAP9), mRNA	NM_001039580	Hs.61271	NM_001039580
PDZRN4	-5.6	4.5	PDZ domain containing RING finger 4 (PDZRN4), mRNA	NM_013377	Hs.380044	AK131554
C22orf35	-5.6	46.7	cDNA FLJ25887 fis, clone CBR02996.	AK098753	Hs.696021	AB263414
MSX2	-5.6	18.9	msh homeobox 2 (MSX2), mRNA	NM_002449	Hs.89404	NM_002449
DTL	-5.6	3.9	denticleless homolog (Drosophila) (DTL), mRNA	NM_016448	Hs.656473	NM_016448
PGBD5	-5.5	8.3	piggyBac transposable element derived 5 (PGBD5), mRNA	NM_024554	Hs.520463	NM_024554
BACH2	-5.5	4.0	BTB and CNC homology 1, basic leucine zipper transcription factor 2 (BACH2), mRNA	NM_021813	Hs.269764	AJ271878
IGF1	-5.5	5.0	insulin-like growth factor 1 (somatomedin C) (IGF1), mRNA	NM_000618	Hs.160562	NM_000618
PPP2R2B	-5.5	19.8	protein phosphatase 2 (formerly 2A), regulatory subunit B (PR 52), beta isoform (PPP2R2B), transcript variant 1, mRNA	NM_004576	Hs.655213	M64930
DHFR	-5.5	3.4	dihydrofolate reductase (DHFR), mRNA	NM_000791	Hs.648635	NM_000791
FLJ25006	-5.5	2.7	hypothetical protein FLJ25006 (FLJ25006), mRNA	NM_144610	Hs.657973	BC045622
TSHZ3	-5.5	2.6	teashirt family zinc finger 3 (TSHZ3), mRNA	NM_020856	Hs.278436	NM_020856
C5orf23	-5.5	6.2	chromosome 5 open reading frame 23 (C5orf23), mRNA	NM_024563	Hs.13528	BC022250
BM678897	-5.5	19.3	BM678897 UI-E-EJ0-ahv-n-06-0-UI.s1 UI-E-EJ0 cDNA clone UI-E-EJ0-ahv-n-06-0-UI 3', mRNA sequence	BM678897	Hs.194766	AB023144
C1orf165	-5.5	5.2	chromosome 1 open reading frame 165 (C1orf165), mRNA	NM_024603	Hs.475348	AK096573
FLJ42875	-5.5	5.0	mRNA; cDNA DKFZp666E184 (from clone DKFZp666E184).	AL833006	Hs.531041	AL832943
AW856073	-5.5	8.6	AW856073 RC1-CT0286-060200-015-c06 CT0286 cDNA, mRNA sequence	AW856073	Unknown	
LCOR	-5.5	2.5	ligand dependent nuclear receptor corepressor (LCOR), mRNA	NM_032440	Hs.500695	AL834245
NELL2	-5.5	33.8	NEL-like 2 (chicken) (NELL2), mRNA	NM_006159	Hs.505326	AK095637
DTL	-5.5	3.8	denticleless homolog (Drosophila) (DTL), mRNA	NM_016448	Hs.656473	NM_016448
OR7D2	-5.5	16.5	olfactory receptor, family 7, subfamily D, member 2 (OR7D2), mRNA	NM_175883	Hs.531755	NM_175883
JMJD1A	-5.5	1.9	jumonji domain containing 1A (JMJD1A), mRNA	NM_018433	Hs.557425	BX640698
GDF10	-5.5	17.8	growth differentiation factor 10 (GDF10), mRNA	NM_004962	Hs.2171	NM_004962
RNF157	-5.5	7.6	ring finger protein 157 (RNF157), mRNA	NM_052916	Hs.500643	NM_052916
FLJ13197	-5.5	5.7	hypothetical protein FLJ13197 (FLJ13197), mRNA	NM_024614	Unknown	
AGPAT4	-5.5	2.8	1-acylglycerol-3-phosphate O-acyltransferase 4 (lysophosphatidic acid acyltransferase, delta) (AGPAT4), mRNA	NM_020133	Hs.353175	NM_020133
ANKRD6	-5.5	4.5	mRNA for KIAA0957 protein, partial cds.	AB023174	Hs.656539	NM_014942
KIAA1026	-5.5	3.7	kazrin (KIAA1026), transcript variant A, mRNA	NM_015209	Hs.699212	NM_001018000
CR598940	-5.5	9.8	full-length cDNA clone CS0DI032YF23 of Placenta Cot 25-normalized of (human).	CR598940	Hs.561513	CR598940

AK022035	-5.5	44.7	cDNA FLJ11973 fis, clone HEMBB1001221.	AK022035	Hs.659665	AK022035
USP47	-5.5	2.4	ubiquitin specific peptidase 47 (USP47), mRNA	NM_017944	Hs.577256	NM_017944
CDH6	-5.5	6.7	cadherin 6, type 2, K-cadherin (fetal kidney) (CDH6), mRNA	NM_004932	Hs.171054	NM_004932
ENST00000367932	-5.5	4.6	cDNA FLJ43132 fis, clone CTONG3005813.	ENST00000367932	Unknown	
GDF10	-5.5	20.5	growth differentiation factor 10 (GDF10), mRNA	NM_004962	Hs.2171	NM_004962
DTL	-5.5	3.7	denticleless homolog (Drosophila) (DTL), mRNA	NM_016448	Hs.656473	NM_016448
AK022430	-5.5	12.1	cDNA FLJ12368 fis, clone MAMMA1002417.	AK022430	Hs.659110	AK022430
GSTM3	-5.5	3.9	glutathione S-transferase M3 (brain) (GSTM3), mRNA	NM_000849	Hs.2006	NM_000849
LHX4	-5.5	3.1	cDNA FLJ38931 fis, clone NT2NE2013189.	AK096250	Hs.496545	AK096250
DBC1	-5.4	15.1	deleted in bladder cancer 1 (DBC1), mRNA	NM_014618	Hs.532316	NM_014618
SKI	-5.4	1.6	Ski oncogene (C-ski).	ENST00000378536	Unknown	
TMEM106B	-5.4	2.8	transmembrane protein 106B (TMEM106B), mRNA	NM_018374	Hs.396358	NM_018374
CTSH	-5.4	4.4	cathepsin H (CTSH), transcript variant 2, mRNA	NM_148979	Hs.148641	CR749582
TRPM3	-5.4	20.6	transient receptor potential cation channel, subfamily M, member 3 (TRPM3), transcript variant 7, mRNA	NM_206948	Hs.47288	NM_001007471
ENST00000369577	-5.4	3.1	Zinc finger protein 292.	ENST00000369577	Unknown	
ZNF91	-5.4	3.8	zinc finger protein 91 (ZNF91), mRNA	NM_003430	Hs.654471	NM_003430
CB987747	-5.4	2.7	AGENCOURT_13631433 NIH_MGC_184 cDNA clone IMAGE:30327753 5', mRNA sequence	CB987747	Hs.527515	CB987747
SMARCA1	-5.4	9.4	SWI/SNF related, matrix associated, actin dependent regulator of chromatin, subfamily a, member 1 (SMARCA1), transcript variant 1, mRNA	NM_003069	Hs.152292	NM_003069
SFRS11	-5.4	2.8	splicing factor, arginine/serine-rich 11 (SFRS11), mRNA	NM_004768	Hs.479693	BX640645
C21orf58	-5.4	3.2	C21orf58 form B (C21orf58) mRNA, complete cds, alternatively spliced.	AY039244	Hs.236572	NM_058180
LOC149351	-5.4	15.8	hypothetical protein LOC149351, mRNA (cDNA clone IMAGE:5273076).	BC036441	Hs.546492	AK022898
TMCC2	-5.4	2.9	transmembrane and coiled-coil domain family 2 (TMCC2), mRNA	NM_014858	Hs.6360	AY358192
BC029907	-5.4	8.8	cDNA clone IMAGE:5175565.	BC029907	Hs.405427	BC029907
DNM3	-5.4	24.0	dynamin 3 (DNM3), mRNA	NM_015569	Hs.654775	AL136712
DTL	-5.4	3.9	denticleless homolog (Drosophila) (DTL), mRNA	NM_016448	Hs.656473	NM_016448
IKZF2	-5.4	3.9	IKAROS family zinc finger 2 (Helios) (IKZF2), transcript variant 2, mRNA	NM_001079526	Hs.604950	NM_001079526
AK090827	-5.4	5.0	cDNA FLJ33508 fis, clone BRAMY2005094.	AK090827	Hs.595153	CR621577
THC2657193	-5.4	9.9	RASH_MSVHA (P01115) Transforming protein p29 precursor , partial (6%)	THC2657193	Unknown	
ENST00000375855	-5.4	9.2	Thioredoxin domain-containing protein C9orf121.	ENST00000375855	Unknown	
BU633484	-5.4	5.3	BU633484 UI-H-FL1-bgu-j-06-0-UI.s1 NCI_CGAP_FL1 cDNA clone UI-H-FL1-bgu-j-06-0-UI 3', mRNA sequence	BU633484	Hs.591085	NM_004529
DTL	-5.3	3.7	denticleless homolog (Drosophila) (DTL), mRNA	NM_016448	Hs.656473	NM_016448
KIAA1411	-5.3	2.7	KIAA1411 (KIAA1411), mRNA	NM_020819	Hs.211700	AB037832
THC2636529	-5.3	2.2	THC2636529	THC2636529	Unknown	
ENST00000367391	-5.3	6.0	LIM/homeobox protein Lhx9.	ENST00000367391	Unknown	
SNX10	-5.3	18.9	sorting nexin 10 (SNX10), mRNA	NM_013322	Hs.571296	BX648363
A_32_P154956	-5.3	4.7	A_32_P154956	A_32_P154956	Unknown	
BC004287	-5.3	5.0	clone IMAGE:3618365, mRNA.	BC004287	Hs.434957	BC004287
RABL2A	-5.3	2.0	RAB, member of RAS oncogene family-like 2A (RABL2A), transcript variant 1, mRNA	NM_013412	Hs.446425	AK092971
CF143262	-5.3	2.6	CF143262 UI-HF-BR0p-aqt-a-11-0-UI.r1 NIH_MGC_52 cDNA clone IMAGE:3101469 5', mRNA sequence	CF143262	Hs.252387	AF231024
THC2679804	-5.3	5.1	ALU1_HUMAN (P39188) Alu subfamily J sequence contamination warning entry, partial (4%)	THC2679804	Unknown	
GRIK3	-5.3	9.6	Glutamate receptor, ionotropic kainate 3 precursor (Glutamate receptor 7) (GluR-7) (GluR7) (Excitatory amino acid receptor 5) (EAA5).	ENST00000373093	Unknown	
CA2	-5.3	11.9	carbonic anhydrase II (CA2), mRNA	NM_000067	Hs.155097	AK123309
AMPH	-5.3	3.1	amphiphysin (Stiff-Man syndrome with breast cancer 128kDa autoantigen) (AMPH), transcript variant 1, mRNA	NM_001635	Hs.592182	NM_001635
THC2752542	-5.3	5.4	THC2752542	THC2752542	Unknown	
POTE15	-5.3	8.0	protein expressed in prostate, ovary, testis, and placenta 15 (POTE15), mRNA	NM_207355	Hs.558766	NM_207355
SORCS2	-5.3	4.1	sortilin-related VPS10 domain containing receptor 2 (SORCS2), mRNA	NM_020777	Hs.479099	NM_020777
ENST00000380195	-5.3	3.9	PCLO_HUMAN Isoform 3 of Q9Y6V0 - (Human)	ENST00000380195	Unknown	
NTRK2	-5.3	7.9	mRNA; cDNA DKFZp686D1394 (from clone DKFZp686D1394).	BX649001	Hs.494312	NM_001018065
BRCA2	-5.3	2.5	breast cancer 2, early onset (BRCA2), mRNA	NM_000059	Hs.34012	NM_000059
TRIM33	-5.3	3.0	tripartite motif-containing 33 (TRIM33), transcript variant b, mRNA	NM_033020	Hs.26837	NM_015906
RGPD5	-5.3	2.6	RANBP2-like and GRIP domain containing 5 (RGPD5), transcript variant 1, mRNA	NM_005054	Hs.469630	NM_005054
ISL1	-5.3	27.0	ISL1 transcription factor, LIM/homeodomain, (islet-1) (ISL1), mRNA	NM_002202	Hs.505	NM_002202

BZRAP1	-5.3	7.4	benzodiazapine receptor (peripheral) associated protein 1 (BZRAP1), mRNA	NM_004758	Hs.112499	BC150279
RHOH	-5.3	5.6	ras homolog gene family, member H (RHOH), mRNA	NM_004310	Hs.694951	NM_004310
SORCS1	-5.3	7.2	sortilin-related VPS10 domain containing receptor 1 (SORCS1), transcript variant 1, mRNA	NM_052918	Hs.591915	NM_052918
CNR1	-5.3	27.6	cannabinoid receptor 1 (brain) (CNR1), transcript variant 2, mRNA	NM_033181	Hs.75110	NM_016083
SLC25A36	-5.3	2.5	mRNA; cDNA DKFZp564C053 (from clone DKFZp564C053).	AL049246	Hs.144130	AL049246
SPDYA	-5.3	2.5	speedy homolog A (Drosophila) (SPDYA), transcript variant 1, mRNA	NM_001008779	Hs.511956	NM_182756
ZNF345	-5.3	2.3	zinc finger protein 345 (ZNF345), mRNA	NM_003419	Hs.362324	BC032863
LIN9	-5.3	2.9	lin-9 homolog (C. elegans) (LIN9), mRNA	NM_173083	Hs.120817	BC045625
C16orf55	-5.3	2.7	chromosome 16 open reading frame 55 (C16orf55), mRNA	NM_153025	Hs.655171	BC045670
C1orf63	-5.3	4.1	chromosome 1 open reading frame 63, mRNA (cDNA clone MGC:74698 IMAGE:6147639), complete cds.	BC065040	Hs.259412	AK096351
RHOH	-5.3	6.1	ras homolog gene family, member H (RHOH), mRNA	NM_004310	Hs.694951	NM_004310
IQCE	-5.3	2.8	mRNA for KIAA1023 protein, partial cds.	AB028946	Unknown	
RAB4A	-5.2	2.1	RAB4A, member RAS oncogene family (RAB4A), mRNA	NM_004578	Hs.296169	AY585832
CDH6	-5.2	70.1	Cadherin-6 precursor (Kidney-cadherin) (K-cadherin).	ENST00000382216	Unknown	
AF318318	-5.2	6.2	pp10394 mRNA, complete cds.	AF318318	Hs.684472	AF318318
WDR47	-5.2	1.6	WD repeat domain 47 (WDR47), mRNA	NM_014969	Hs.654760	BC034964
STRBP	-5.2	2.5	spermatid perinuclear RNA binding protein (STRBP), mRNA	NM_018387	Hs.696159	NM_018387
THC2618883	-5.2	13.5	THC2618883	THC2618883	Unknown	
MDGA1	-5.2	5.5	MAM domain containing glycosylphosphatidylinositol anchor 1 (MDGA1), mRNA	NM_153487	Hs.437993	AF478693
AK022045	-5.2	9.7	cDNA FLJ11983 fis, clone HEMBB1001337.	AK022045	Hs.655386	AK024201
UBE4B	-5.2	2.0	ubiquitination factor E4B (UFD2 homolog, yeast) (UBE4B), mRNA	NM_006048	Hs.632370	NM_006048
SORCS3	-5.2	14.3	sortilin-related VPS10 domain containing receptor 3 (SORCS3), mRNA	NM_014978	Hs.671950	AB028982
LOC389023	-5.2	13.9	hypothetical gene supported by BC032913; BC048425, mRNA (cDNA clone IMAGE:5265535).	BC032913	Hs.97540	BU569564
A_32_P6452	-5.2	3.5	A_32_P6452	A_32_P6452	Unknown	
SPAG6	-5.2	48.8	sperm associated antigen 6 (SPAG6), transcript variant 2, mRNA	NM_172242	Hs.655170	NM_012443
ADAM28	-5.2	10.5	ADAM metallopeptidase domain 28 (ADAM28), transcript variant 1, mRNA	NM_014265	Hs.174030	AK225921
A_24_P640212	-5.2	3.7	A_24_P640212	A_24_P640212	Unknown	
IL17RB	-5.2	6.1	interleukin 17 receptor B (IL17RB), mRNA	NM_018725	Hs.654970	NM_018725
KIAA0907	-5.2	2.3	KIAA0907 (KIAA0907), mRNA	NM_014949	Hs.24656	AB020714
AL133090	-5.2	10.4	mRNA; cDNA DKFZp434E0528 (from clone DKFZp434E0528).	AL133090	Hs.592567	AL133090
AF146694	-5.2	2.6	clone IMAGE:121412 mRNA sequence.	AF146694	Hs.372739	AF146694
AK027319	-5.2	2.1	cDNA FLJ14413 fis, clone HEMBA1004670.	AK027319	Hs.334832	AK123705
ANKRD21	-5.2	14.5	ankyrin repeat domain 21 (ANKRD21), mRNA	NM_174981	Hs.442712	AY172978
DFNB31	-5.2	3.7	cDNA FLJ31628 fis, clone NT2RI2003344, weakly similar to PRESYNAPTIC PROTEIN SAP97.	AK056190	Hs.93836	BC014524
TTC25	-5.2	4.6	tetratricopeptide repeat domain 25 (TTC25), mRNA	NM_031421	Hs.201134	NM_031421
ICK	-5.2	1.9	intestinal cell (MAK-like) kinase (ICK), transcript variant 2, mRNA	NM_016513	Hs.417022	NM_016513
ACVR1C	-5.2	6.4	activin A receptor, type IC (ACVR1C), mRNA	NM_145259	Hs.352338	BC022530
NXPH1	-5.2	9.7	neurexophilin 1 (NXPH1), mRNA	NM_152745	Hs.487564	NM_152745
DTL	-5.2	3.7	denticleless homolog (Drosophila) (DTL), mRNA	NM_016448	Hs.656473	NM_016448
THC2532458	-5.2	5.8	NM_205486 non-histone chromosomal protein {Gallus gallus} (exp=-1; wgp=0; cg=0), partial (21%)	THC2532458	Unknown	
LOC387856	-5.2	7.7	similar to expressed sequence AI836003 (LOC387856), mRNA	NM_001013635	Hs.251699	BC049387
THC2724111	-5.2	3.7	THC2724111	THC2724111	Unknown	
AK026811	-5.2	2.5	cDNA: FLJ23158 fis, clone LNG09623.	AK026811	Unknown	
UPF3A	-5.2	2.4	UPF3 regulator of nonsense transcripts homolog A (yeast) (UPF3A), transcript variant 1, mRNA	NM_023011	Hs.533855	AK092586
CLN3	-5.1	3.0	ceroid-lipofuscinosis, neuronal 3, juvenile (Batten, Spielmeyer-Vogt disease), mRNA (cDNA clone MGC:102840 IMAGE:5764535), complete cds.	BC111068	Hs.628393	AK090709
TPBG	-5.1	5.7	trophoblast glycoprotein (TPBG), mRNA	NM_006670	Hs.82128	NM_006670
CLK1	-5.1	2.0	CDC-like kinase 1 (CLK1), transcript variant 1, mRNA	NM_004071	Hs.433732	NM_004071
AK128756	-5.1	13.5	cDNA FLJ44869 fis, clone BRAMY2015516.	AK128756	Hs.632969	AK128756
ATP6V1A	-5.1	2.0	ATPase, H+ transporting, lysosomal 70kDa, V1 subunit A (ATP6V1A), mRNA	NM_001690	Hs.477155	NM_001690
SPANXB2	-5.1	14.7	SPANX family, member B2 (SPANXB2), mRNA	NM_145664	Hs.434105	BG721088
DCX	-5.1	72.5	doublecortex; lissencephaly, X-linked (doublecortin) (DCX), transcript variant 1, mRNA	NM_000555	Hs.34780	NM_000555
OLFM1	-5.1	10.3	olfactomedin 1 (OLFM1), transcript variant 1, mRNA	NM_014279	Hs.522484	NM_014279

BC040991	-5.1	3.4	cDNA clone IMAGE:4817695.	BC040991	Hs.379018	U18422
CABLES2	-5.1	2.4	Cdk5 and Abl enzyme substrate 2 (CABLES2), mRNA	NM_031215	Hs.301040	NM_031215
TDH	-5.1	8.0	cDNA FLJ25033 fis, clone CBL02720.	AK057762	Hs.583896	AK057762
AHDC1	-5.1	1.9	AT hook, DNA binding motif, containing 1 (AHDC1), mRNA	NM_001029882	Hs.469280	NM_001029882
CNTFR	-5.1	6.0	ciliary neurotrophic factor receptor (CNTFR), transcript variant 1, mRNA	NM_147164	Hs.129966	AK127444
ABAT	-5.1	4.6	4-aminobutyrate aminotransferase (ABAT), nuclear gene encoding mitochondrial protein, transcript variant 2, mRNA	NM_000663	Hs.336768	NM_000663
SLC13A4	-5.1	14.0	solute carrier family 13 (sodium/sulfate symporters), member 4 (SLC13A4), mRNA	NM_012450	Hs.490241	AB208809
AX721195	-5.1	6.0	Sequence 155 from Patent WO0220754.	AX721195	Unknown	
TRPV1	-5.1	3.2	transient receptor potential cation channel, subfamily V, member 1 (TRPV1), transcript variant 3, mRNA	NM_080706	Hs.655380	NM_080706
ENST00000370599	-5.1	8.9	fibroblast growth factor homologous factor 2 isoform 1Z+1Y (FHF-2) mRNA, partial cds.	ENST00000370599	Unknown	
ADRA2A	-5.1	5.1	adrenergic, alpha-2A-, receptor (ADRA2A), mRNA	NM_000681	Hs.249159	NM_000681
SAA4	-5.1	8.2	serum amyloid A4, constitutive (SAA4), mRNA	NM_006512	Hs.654493	BG564326
C17orf72	-5.1	2.7	chromosome 17 open reading frame 72, mRNA (cDNA clone IMAGE:5299340).	BC045676	Hs.694852	AK021786
INADL	-5.1	6.1	InaD-like (Drosophila) (INADL), mRNA	NM_176877	Hs.478125	NM_176877
SYNE2	-5.1	3.7	spectrin repeat containing, nuclear envelope 2 (SYNE2), transcript variant 1, mRNA	NM_015180	Hs.525392	NM_182914
BX114329	-5.1	3.2	BX114329 BX114329 Soares_NhHMPu_S1 cDNA clone IMAGp998G064741 ; IMAGE:1932317, mRNA sequence	BX114329	Unknown	
LRRN6C	-5.1	11.1	leucine rich repeat neuronal 6C (LRRN6C), mRNA	NM_152570	Hs.699432	AK056372
DVL1	-5.1	2.0	dishevelled, dsh homolog 1 (Drosophila) (DVL1), transcript variant 2, mRNA	NM_181870	Unknown	
ENST00000367169	-5.1	6.8	Neurofascin precursor.	ENST00000367169	Unknown	
CP110	-5.1	2.8	CP110 protein (CP110), mRNA	NM_014711	Hs.279912	NM_014711
AK022337	-5.1	2.9	cDNA FLJ12275 fis, clone MAMMA1001686.	AK022337	Hs.661289	AK022337
IGFBP2	-5.1	6.3	insulin-like growth factor binding protein 2, 36kDa (IGFBP2), mRNA	NM_000597	Hs.438102	AB209509
FBXL16	-5.1	6.7	F-box and leucine-rich repeat protein 16 (FBXL16), mRNA	NM_153350	Hs.513244	BC036680
SCYL1BP1	-5.1	2.9	SCY1-like 1 binding protein 1 (SCYL1BP1), mRNA	NM_152281	Hs.183702	BC034482
C1orf192	-5.1	6.1	chromosome 1 open reading frame 192 (C1orf192), mRNA	NM_001013625	Hs.534593	BM800357
DPP6	-5.1	13.1	dipeptidyl-peptidase 6 (DPP6), transcript variant 3, mRNA	NM_001039350	Hs.490684	BC150304
CLK4	-5.1	2.7	CDC-like kinase 4 (CLK4), mRNA	NM_020666	Hs.406557	BC151233
PPM1H	-5.1	17.5	mRNA for KIAA1157 protein, partial cds.	AB032983	Hs.435479	NM_020700
C6orf59	-5.1	2.7	cDNA: FLJ23112 fis, clone LNG07874.	AK026765	Hs.664873	AK026765
AF052141	-5.0	20.9	clone 24626 mRNA sequence.	AF052141	Hs.13438	AF052141
AF119913	-5.0	4.4	PRO3077 mRNA, complete cds.	AF119913	Unknown	
UBL3	-5.0	2.9	ubiquitin-like 3 (UBL3), mRNA	NM_007106	Hs.145575	BC044582
C10orf79	-5.0	25.3	chromosome 10 open reading frame 79 (C10orf79), mRNA	NM_025145	Hs.288927	NM_025145
AK055647	-5.0	7.7	cDNA FLJ31085 fis, clone IMR321000037.	AK055647	Hs.695349	AK055647
UHMK1	-5.0	1.7	Serine/threonine-protein kinase Kist (EC 2.7.11.1) (Kinase interacting with stathmin) (U2AF homology motif kinase 1).	ENST00000282169	Unknown	
LOC399744	-5.0	5.5	hypothetical LOC399744 (LOC399744), mRNA	NM_001013665	Unknown	
IQCA	-5.0	3.4	IQ motif containing with AAA domain (IQCA), mRNA	NM_024726	Hs.591594	BC028699
PPM1H	-5.0	3.3	mRNA for KIAA1157 protein, partial cds.	AB032983	Hs.435479	NM_020700
HLTF	-5.0	3.3	helicase-like transcription factor (HLTF), transcript variant 1, mRNA	NM_003071	Hs.3068	NM_003071
C3orf34	-5.0	2.2	chromosome 3 open reading frame 34 (C3orf34), mRNA	NM_032898	Hs.334526	BC007827
NBEA	-5.0	3.8	neurobeachin (NBEA), mRNA	NM_015678	Hs.491172	NM_015678
HS2ST1	-5.0	2.5	heparan sulfate 2-O-sulfotransferase 1 (HS2ST1), mRNA	NM_012262	Hs.48823	NM_012262
C15orf21	-5.0	2.8	chromosome 15 open reading frame 21 (C15orf21), transcript variant 1, mRNA	NM_001005266	Hs.574240	NM_001005266
C20orf58	-5.0	9.4	chromosome 20 open reading frame 58 (C20orf58), mRNA	NM_152864	Hs.46627	NM_152864
APOA2	-5.0	22.3	apolipoprotein A-II (APOA2), mRNA	NM_001643	Hs.237658	CR619946
H3F3A	-5.0	4.6	H3 histone, family 3A (H3F3A), mRNA	NM_002107	Hs.546259	CR621187
TMEM169	-5.0	9.9	transmembrane protein 169 (TMEM169), mRNA	NM_138390	Hs.334916	NM_138390
RHOH	-5.0	5.6	ras homolog gene family, member H (RHOH), mRNA	NM_004310	Hs.694951	NM_004310
IGF1	-5.0	3.4	insulin-like growth factor 1 (somatomedin C) (IGF1), mRNA	NM_000618	Hs.160562	NM_000618
AK092942	-5.0	3.8	cDNA FLJ35623 fis, clone SPLEN2010986.	AK092942	Hs.660700	XR_015431
BG216262	-5.0	7.0	RST35951 Athersys RAGE Library cDNA, mRNA sequence	BG216262	Hs.637431	BG216262
NTNG1	-5.0	5.1	netrin G1 (NTNG1), mRNA	NM_014917	Hs.657434	AB023193

AF037219	-5.0	3.3	PIX1 mRNA sequence.	AF037219	Hs.654383	NM_130839
CLUL1	-5.0	45.0	clusterin-like 1 (retinal) (CLUL1), transcript variant 1, mRNA	NM_014410	Hs.632357	NM_014410
AK091528	-5.0	1.8	cDNA FLJ34209 fis, clone FCBBF3020599.	AK091528	Hs.592819	AK091528
MAP3K9	-5.0	3.4	cDNA FLJ41436 fis, clone BRHIP2007741.	AK123430	Hs.445496	AK123430
INHBB	-5.0	3.8	inhibin, beta B (activin AB beta polypeptide) (INHBB), mRNA	NM_002193	Hs.1735	NM_002193
CACNA2D3	-5.0	10.5	calcium channel, voltage-dependent, alpha 2/delta 3 subunit (CACNA2D3), mRNA	NM_018398	Hs.656687	NM_018398
LOC389857	-5.0	65.7	clone IMAGE:24166 hypothetical protein mRNA, partial cds.	AY730278	Hs.631771	AY730278
B3GAT1	-5.0	34.4	beta-1,3-glucuronyltransferase 1 (glucuronosyltransferase P) (B3GAT1), transcript variant 2, mRNA	NM_054025	Hs.381050	BC010466
RBBP4	-5.0	3.3	retinoblastoma binding protein 4 (RBBP4), mRNA	NM_005610	Hs.647652	BC053904
C1orf87	-5.0	4.3	chromosome 1 open reading frame 87 (C1orf87), mRNA	NM_152377	Hs.47385	AK126061
C20orf26	-5.0	8.4	chromosome 20 open reading frame 26 (C20orf26), mRNA	NM_015585	Hs.176013	NM_015585
AK001089	-5.0	2.2	cDNA FLJ10227 fis, clone HEMBB1000103.	AK001089	Hs.472723	AK001089
UNC5D	-5.0	49.1	unc-5 homolog D (C. elegans) (UNC5D), mRNA	NM_080872	Hs.238889	NM_080872
LOC130940	-5.0	6.2	hypothetical protein BC015395 (LOC130940), mRNA	NM_138803	Hs.668597	NM_138803
CTDSPL2	-5.0	1.9	CTD (carboxy-terminal domain, RNA polymerase II, polypeptide A) small phosphatase like 2 (CTDSPL2), mRNA	NM_016396	Hs.646495	NM_016396
KCNK1	-5.0	5.6	potassium channel, subfamily K, member 1 (KCNK1), mRNA	NM_002245	Hs.208544	AK090902
GCG	-5.0	6.5	glucagon (GCG), mRNA	NM_002054	Hs.516494	BM802953
NKX6-2	-4.9	3.5	NK6 transcription factor related, locus 2 (Drosophila) (NKX6-2), mRNA	NM_177400	Hs.134013	NM_177400
RB1	-4.9	1.9	retinoblastoma 1 (including osteosarcoma) (RB1), mRNA	NM_000321	Hs.408528	L41870
POGZ	-4.9	2.7	pogo transposable element with ZNF domain (POGZ), transcript variant 1, mRNA	NM_015100	Hs.591471	BX537838
ANKRD10	-4.9	2.8	ankyrin repeat domain 10 (ANKRD10), mRNA	NM_017664	Hs.525163	BX641022
RSPO2	-4.9	28.4	R-spondin 2 homolog (Xenopus laevis) (RSPO2), mRNA	NM_178565	Hs.444834	AK123023
CR616939	-4.9	7.6	full-length cDNA clone CS0DF026YH11 of Fetal brain of (human).	CR616939	Hs.223770	XM_065166
BC031314	-4.9	13.7	cDNA clone IMAGE:5276765.	BC031314	Hs.26766	BM925867
HSP90AA1	-4.9	2.0	heat shock protein 90kDa alpha (cytosolic), class A member 1 (HSP90AA1), transcript variant 2, mRNA	NM_005348	Hs.525600	NM_001017963
MARK1	-4.9	3.6	MAP/microtubule affinity-regulating kinase 1 (MARK1), mRNA	NM_018650	Hs.497806	NM_018650
H3F3A	-4.9	2.7	H3 histone, family 3A (H3F3A), mRNA	NM_002107	Hs.546259	CR621187
RP11-50D16.3	-4.9	3.1	similar to RIKEN cDNA 8030451K01 (LOC387921), transcript variant 1, mRNA	NM_001012754	Hs.507783	NM_001012754
THC2731029	-4.9	6.6	THC2731029	THC2731029	Unknown	
GCC2	-4.9	2.8	GRIP and coiled-coil domain containing 2 (GCC2), transcript variant 1, mRNA	NM_181453	Hs.436505	NM_181453
AK094799	-4.9	17.6	cDNA FLJ37480 fis, clone BRAWH2013866, highly similar to stonin 2 mRNA.	AK094799	Hs.14248	BX648249
ARMC3	-4.9	72.7	armadillo repeat containing 3 (ARMC3), mRNA	NM_173081	Hs.659807	BC039312
GPM6A	-4.9	11.2	glycoprotein M6A (GPM6A), transcript variant 2, mRNA	NM_201591	Hs.75819	NM_201591
SNX10	-4.9	15.2	sorting nexin 10 (SNX10), mRNA	NM_013322	Hs.571296	BX648363
WDR26	-4.9	3.1	WD repeat domain 26 (WDR26), mRNA	NM_025160	Hs.497873	AY221751
A_32_P116088	-4.9	5.1	A_32_P116088	A_32_P116088	Unknown	
HTR2C	-4.9	56.0	5-hydroxytryptamine (serotonin) receptor 2C (HTR2C), mRNA	NM_000868	Hs.149037	NM_000868
CCRK	-4.9	4.4	cell cycle related kinase (CCRK), transcript variant 3, mRNA	NM_001039803	Hs.522274	AK226135
CR936771	-4.9	2.7	mRNA; cDNA DKFZp686A0668 (from clone DKFZp686A0668).	CR936771	Hs.632428	CR749219
LOC388272	-4.9	2.1	similar to RIKEN cDNA 4921524J17 (LOC388272), mRNA	NM_001001436	Hs.700758	BX647945
SOX8	-4.9	6.8	SRY (sex determining region Y)-box 8 (SOX8), mRNA	NM_014587	Hs.243678	NM_014587
PHACTR4	-4.9	2.5	phosphatase and actin regulator 4 (PHACTR4), transcript variant 1, mRNA	NM_001048183	Hs.225641	NM_001048183
TPM1	-4.9	3.6	tropomyosin 1 (alpha) (TPM1), transcript variant 3, mRNA	NM_001018004	Hs.133892	BX648171
THC2666687	-4.9	20.9	ALU1_HUMAN (P39188) Alu subfamily J sequence contamination warning entry, partial (4%)	THC2666687	Unknown	
DSCR1L2	-4.9	2.6	Down syndrome critical region gene 1-like 2 (DSCR1L2), mRNA	NM_013441	Hs.656799	BC035854
LOC728537	-4.9	3.1	cDNA clone IMAGE:5271446.	BC039374	Hs.469369	BC039374
FOXE1	-4.9	3.0	forkhead box E1 (thyroid transcription factor 2) (FOXE1), mRNA	NM_004473	Hs.159234	U89995
TPM1	-4.9	2.9	tropomyosin 1 (alpha) (TPM1), transcript variant 3, mRNA	NM_001018004	Hs.133892	BX648171
CB240827	-4.9	2.3	UI-CF-FN0-afw-h-15-0-UI.s1 UI-CF-FN0 cDNA clone UI-CF-FN0-afw-h-15-0-UI 3', mRNA sequence	CB240827	Hs.621746	CB240827
PITX1	-4.9	8.2	paired-like homeodomain transcription factor 1 (PITX1), mRNA	NM_002653	Hs.84136	NM_002653
DYNLRB2	-4.9	12.2	dynein, light chain, roadblock-type 2 (DYNLRB2), mRNA	NM_130897	Hs.98849	BC035232
AL359605	-4.9	6.4	mRNA; cDNA DKFZp547G036 (from clone DKFZp547G036).	AL359605	Hs.283851	AK124873
KAL1	-4.9	6.6	Kallmann syndrome 1 sequence (KAL1), mRNA	NM_000216	Hs.521869	NM_000216

ZC3H11A	-4.9	2.3	zinc finger CCCH-type containing 11A (ZC3H11A), mRNA	NM_014827	Hs.532399	CR627439
NR2E1	-4.9	5.7	nuclear receptor subfamily 2, group E, member 1 (NR2E1), mRNA	NM_003269	Hs.157688	NM_003269
A_32_P67303	-4.9	14.9	A_32_P67303	A_32_P67303	Unknown	
PPP1R9A	-4.9	2.3	protein phosphatase 1, regulatory (inhibitor) subunit 9A (PPP1R9A), mRNA	NM_017650	Hs.21816	NM_017650
INADL	-4.9	2.6	InaD-like (Drosophila) (INADL), mRNA	NM_176877	Hs.478125	NM_176877
ZFP14	-4.9	3.8	zinc finger protein 14 homolog (mouse) (ZFP14), mRNA	NM_020917	Hs.35524	NM_020917
FZD2	-4.9	5.1	frizzled homolog 2 (Drosophila) (FZD2), mRNA	NM_001466	Hs.142912	BC052266
ZC3H11A	-4.8	2.5	zinc finger CCCH-type containing 11A (ZC3H11A), mRNA	NM_014827	Hs.532399	CR627439
A_23_P165819	-4.8	2.2	A_23_P165819	A_23_P165819	Unknown	
GPR64	-4.8	8.8	G protein-coupled receptor 64 (GPR64), transcript variant 1, mRNA	NM_001079858	Hs.146978	BC063315
A_23_P135063	-4.8	33.7	A_23_P135063	A_23_P135063	Unknown	
ANKRD46	-4.8	2.6	ankyrin repeat domain 46 (ANKRD46), mRNA	NM_198401	Hs.530199	BC035087
ENST00000369[illegible]	-4.8	9.1	Novel protein.	ENST00000369945	Unknown	
WNT2	-4.8	29.8	wingless-type MMTV integration site family member 2 (WNT2), mRNA	NM_003391	Hs.567356	BC078170
ELAVL4	-4.8	4.9	ELAV (embryonic lethal, abnormal vision, Drosophila)-like 4 (Hu antigen D), mRNA (cDNA clone MGC:33705 IMAGE:5286347), complete cds.	BC036071	Hs.213050	BC036071
A_32_P31206	-4.8	3.2	A_32_P31206	A_32_P31206	Unknown	
EFHC2	-4.8	6.1	EF-hand domain (C-terminal) containing 2 (EFHC2), mRNA	NM_025184	Hs.521953	NM_025184
UBE2T	-4.8	2.6	ubiquitin-conjugating enzyme E2T (putative) (UBE2T), mRNA	NM_014176	Hs.5199	BF690859
MAP9	-4.8	4.1	microtubule-associated protein 9 (MAP9), mRNA	NM_001039580	Hs.61271	NM_001039580
ZFYVE16	-4.8	2.6	Zinc finger FYVE domain-containing protein 16 (Endofin) (Endosome- associated FYVE domain protein).	ENST00000380248	Unknown	
SYT17	-4.8	4.1	synaptotagmin XVII (SYT17), mRNA	NM_016524	Hs.258326	AK226136
THC2705431	-4.8	7.5	THC2705431	THC2705431	Unknown	
THC2586873	-4.8	2.6	THC2586873	THC2586873	Unknown	
MTR	-4.8	2.0	5-methyltetrahydrofolate-homocysteine methyltransferase (MTR), mRNA	NM_000254	Hs.498187	U73338
AF131798	-4.8	5.4	clone 25119 mRNA sequence.	AF131798	Hs.448887	AK091326
PTAR1	-4.8	3.3	mRNA; cDNA DKFZp313P0917 (from clone DKFZp313P0917).	AL832683	Hs.700761	NM_001099666
MEGF11	-4.8	13.0	multiple EGF-like-domains 11 (MEGF11), mRNA	NM_032445	Hs.438250	AB058677
RHOH	-4.8	5.0	ras homolog gene family, member H (RHOH), mRNA	NM_004310	Hs.694951	NM_004310
RB1	-4.8	1.8	retinoblastoma 1 (including osteosarcoma) (RB1), mRNA	NM_000321	Hs.408528	L41870
TM4SF5	-4.8	9.4	transmembrane 4 L six family member 5 (TM4SF5), mRNA	NM_003963	Hs.184194	W84524
ZNF28	-4.8	3.1	zinc finger protein 28 (ZNF28), mRNA	NM_006969	Hs.554778	AK131368
SYT13	-4.8	88.6	synaptotagmin XIII (SYT13), mRNA	NM_020826	Hs.436643	AB037848
A_23_P20328	-4.8	2.3	A_23_P20328	A_23_P20328	Unknown	
NTRK3	-4.8	4.8	neurotrophic tyrosine kinase, receptor, type 3 (NTRK3), transcript variant 1, mRNA	NM_001012338	Hs.410969	NM_001007156
DNER	-4.8	5.7	delta/notch-like EGF repeat containing (DNER), mRNA	NM_139072	Hs.234074	BC035009
BC031957	-4.8	2.3	cDNA clone IMAGE:4838390.	BC031957	Hs.638344	BC031957
HCG12	-4.8	2.3	HLA complex group 12 (HCG12) on chromosome 6	NR_002831	Unknown	
AI207522	-4.8	7.4	AI207522 HA2878 Human fetal liver cDNA library cDNA, mRNA sequence	AI207522	Hs.134859	BG401887
RP11-393H10.2	-4.8	4.3	hypothetical protein FLJ14503 (FLJ14503), mRNA	NM_152780	Hs.127951	BC089400
AK097080	-4.8	8.3	cDNA FLJ39761 fis, clone SPLEN1000083.	AK097080	Hs.534942	XR_017612
U92981	-4.8	3.3	clone DT1P1B6 mRNA, CAG repeat region.	U92981	Hs.596282	U92981
TRO	-4.8	6.2	trophinin (TRO), transcript variant 3, mRNA	NM_016157	Hs.695979	NM_001039705
SDCCAG1	-4.8	2.2	serologically defined colon cancer antigen 1 (SDCCAG1), mRNA	NM_004713	Hs.655964	NM_004713
GOLGA8A	-4.7	4.8	golgi autoantigen, golgin subfamily a, 8A (GOLGA8A), mRNA	NM_181077	Hs.182982	BX648160
PCDH11Y	-4.7	22.6	protocadherin 11 Y-linked (PCDH11Y), transcript variant c, mRNA	NM_032973	Hs.661308	NM_032973
RHOH	-4.7	5.4	ras homolog gene family, member H (RHOH), mRNA	NM_004310	Hs.694951	NM_004310
ENST00000373[illegible]	-4.7	4.3	Palmitoyltransferase ZDHHC15 (EC 2.3.1.-) (Zinc finger DHHC domain-containing protein 15) (DHHC-15).	ENST00000373361	Unknown	
KIAA0319	-4.7	6.1	KIAA0319 (KIAA0319), mRNA	NM_014809	Hs.26441	NM_014809
PAX6	-4.7	5.0	paired box gene 6 (aniridia, keratitis) (PAX6), transcript variant 1, mRNA	NM_000280	Hs.591993	XM_001130771
RHOH	-4.7	5.1	ras homolog gene family, member H (RHOH), mRNA	NM_004310	Hs.694951	NM_004310
TSPAN2	-4.7	6.6	tetraspanin 2 (TSPAN2), mRNA	NM_005725	Hs.310458	NM_005725
FAM89A	-4.7	4.1	family with sequence similarity 89, member A (FAM89A), mRNA	NM_198552	Hs.38516	CR617492
IGF1	-4.7	4.0	insulin-like growth factor 1 (somatomedin C) (IGF1), mRNA	NM_000618	Hs.160562	NM_000618

CRABP2	-4.7	5.8	cellular retinoic acid binding protein 2 (CRABP2), mRNA	NM_001878	Hs.405662	BI194589
PHACTR3	-4.7	12.3	phosphatase and actin regulator 3 (PHACTR3), transcript variant 1, mRNA	NM_080672	Hs.473218	NM_080672
GTF2I	-4.7	2.4	general transcription factor II, i (GTF2I), transcript variant 1, mRNA	NM_032999	Hs.647041	NM_032999
SALL3	-4.7	6.8	sal-like 3 (Drosophila) (SALL3), mRNA	NM_171999	Hs.699525	NM_171999
RP13-347D8.3	-4.7	16.9	mRNA for KIAA1210 protein, partial cds.	AB033036	Hs.97594	NM_020721
BC040982	-4.7	2.1	cDNA clone IMAGE:4798675.	BC040982	Hs.656958	BC040982
DDX26B	-4.7	13.1	DEAD/H (Asp-Glu-Ala-Asp/His) box polypeptide 26B (DDX26B), mRNA	NM_182540	Hs.496829	BX648113
THC2661011	-4.7	22.1	Q3H5V5_9ACTO (Q3H5V5) Thiolase, partial (3%)	THC2661011	Unknown	
TSPAN13	-4.7	3.1	tetraspanin 13 (TSPAN13), mRNA	NM_014399	Hs.364544	AK128509
ISG20L2	-4.7	1.5	interferon stimulated exonuclease gene 20kDa-like 2 (ISG20L2), mRNA	NM_030980	Hs.301904	AL096734
ATRNL1	-4.7	5.9	attractin-like 1 (ATRNL1), mRNA	NM_207303	Hs.501127	NM_207303
FAM44A	-4.7	2.2	family with sequence similarity 44, member A (FAM44A), mRNA	NM_148894	Hs.444517	NM_148894
AMPH	-4.7	3.8	amphiphysin (Stiff-Man syndrome with breast cancer 128kDa autoantigen) (AMPH), transcript variant 1, mRNA	NM_001635	Hs.592182	NM_001635
CXXC4	-4.7	20.3	CXXC finger 4 (CXXC4), mRNA	NM_025212	Hs.12248	AK127778
ZC3H11A	-4.7	2.8	zinc finger CCCH-type containing 11A (ZC3H11A), mRNA	NM_014827	Hs.532399	CR627439
AK123861	-4.7	2.6	cDNA FLJ41867 fis, clone OCBBF2005546.	AK123861	Hs.658919	AK123861
THC2753798	-4.7	2.8	BF238843 601904455F1 NIH_MGC_54 cDNA clone IMAGE:4132429 5', mRNA sequence	THC2753798	Unknown	
RTP3	-4.7	2.4	receptor (chemosensory) transporter protein 3 (RTP3), mRNA	NM_031440	Hs.196584	AJ312776
RIBC1	-4.7	2.7	RIB43A domain with coiled-coils 1 (RIBC1), transcript variant 2, mRNA	NM_144968	Hs.351743	AK057345
THC2662125	-4.7	3.1	THC2662125	THC2662125	Unknown	
TRSPAP1	-4.7	1.8	tRNA selenocysteine associated protein 1 (TRSPAP1), transcript variant 1, [illegible]	NM_017846	Hs.533626	NM_017846
AK125176	-4.7	9.0	cDNA FLJ43186 fis, clone FCBBF3022767.	AK125176	Hs.648505	AK125176
RB1	-4.7	1.9	retinoblastoma 1 (including osteosarcoma) (RB1), mRNA	NM_000321	Hs.408528	L41870
CR590573	-4.7	6.0	full-length cDNA clone CS0DI042YD07 of Placenta Cot 25-normalized of (human).	CR590573	Hs.484967	CR590573
KIAA1913	-4.7	3.6	KIAA1913 (KIAA1913), mRNA	NM_052913	Hs.591341	NM_052913
JMJD1A	-4.7	2.1	jumonji domain containing 1A (JMJD1A), mRNA	NM_018433	Hs.557425	BX640698
LEF1	-4.7	6.5	lymphoid enhancer-binding factor 1 (LEF1), mRNA	NM_016269	Hs.555947	AK128255
FLJ21272	-4.7	8.1	cDNA: FLJ21272 fis, clone COL01753.	AK024925	Hs.612891	AK024925
FOXN4	-4.7	12.0	forkhead box N4 (FOXN4), mRNA	NM_213596	Hs.528316	BC146825
PTPRD	-4.7	2.9	protein tyrosine phosphatase, receptor type, D (PTPRD), transcript variant 1, mRNA	NM_002839	Hs.446083	NM_002839
CR624880	-4.7	2.1	full-length cDNA clone CS0DB005YI23 of Neuroblastoma Cot 10-normalized of (human).	CR624880	Hs.505983	AK056140
OPRK1	-4.7	3.9	opioid receptor, kappa 1 (OPRK1), mRNA	NM_000912	Hs.106795	NM_000912
AL049443	-4.7	3.4	mRNA; cDNA DKFZp586N2020 (from clone DKFZp586N2020).	AL049443	Hs.660870	AL049443
THC2672319	-4.7	5.5	THC2672319	THC2672319	Unknown	
KCTD8	-4.7	20.9	potassium channel tetramerisation domain containing 8 (KCTD8), mRNA	NM_198353	Hs.479644	AK123347
A_24_P307395	-4.7	4.4	A_24_P307395	A_24_P307395	Unknown	
MLLT3	-4.6	4.8	myeloid/lymphoid or mixed-lineage leukemia (trithorax homolog, Drosophila); translocated to, 3 (MLLT3), mRNA	NM_004529	Hs.591085	NM_004529
CR620185	-4.6	15.8	full-length cDNA clone CS0DF014YC15 of Fetal brain of (human).	CR620185	Hs.593599	AK124702
AK124698	-4.6	42.5	cDNA FLJ42708 fis, clone BRAMY3007311.	AK124698	Hs.411472	CR749843
FBXO16	-4.6	5.4	F-box protein 16 (FBXO16), mRNA	NM_172366	Hs.659190	NM_172366
NCF2	-4.6	8.6	neutrophil cytosolic factor 2 (65kDa, chronic granulomatous disease, autosomal 2) (NCF2), mRNA	NM_000433	Hs.587558	AB209647
DMTF1	-4.6	2.5	cyclin D binding myb-like transcription factor 1 (DMTF1), mRNA	NM_021145	Hs.654981	AK126664
CCNL2	-4.6	2.6	cyclin L2 (CCNL2), transcript variant 2, mRNA	NM_001039577	Hs.515704	NM_001039577
ENAH	-4.6	3.1	enabled homolog (Drosophila) (ENAH), transcript variant 1, mRNA	NM_001008493	Hs.497893	NM_001008493
ZDHHC11	-4.6	7.0	zinc finger, DHHC-type containing 11 (ZDHHC11), mRNA	NM_024786	Hs.659832	AK024233
SEMA3C	-4.6	6.9	sema domain, immunoglobulin domain (Ig), short basic domain, secreted, (semaphorin) 3C (SEMA3C), mRNA	NM_006379	Hs.269109	NM_006379
S100PBP	-4.6	3.9	S100P binding protein (S100PBP), transcript variant 1, mRNA	NM_022753	Hs.440880	BX647916
POTE2	-4.6	17.4	protein expressed in prostate, ovary, testis, and placenta 2 (POTE2), transcript variant POTE-2D, mRNA	NM_001005359	Unknown	
CXorf57	-4.6	5.6	chromosome X open reading frame 57 (CXorf57), mRNA	NM_018015	Hs.274267	BC070110
CALD1	-4.6	2.9	caldesmon 1 (CALD1), transcript variant 1, mRNA	NM_033138	Hs.490203	NM_033138
MTM1	-4.6	2.2	myotubularin 1 (MTM1), mRNA	NM_000252	Hs.655056	NM_000252
CLASP2	-4.6	1.9	cytoplasmic linker associated protein 2 (CLASP2), mRNA	NM_015097	Hs.696092	NM_015097

C1orf149	-4.6	2.2	chromosome 1 open reading frame 149 (C1orf149), mRNA	NM_022756	Hs.17118	BX640719
BZRAP1	-4.6	7.7	benzodiazapine receptor (peripheral) associated protein 1 (BZRAP1), mRNA	NM_004758	Hs.112499	BC150279
FLJ22795	-4.6	3.5	colon cancer-associated antigen AgSK1-2HT-ECS mRNA, complete cds.	AF316855	Hs.498322	AF316855
THC2527406	-4.6	2.2	THC2527406	THC2527406	Unknown	
STK31	-4.6	8.6	serine/threonine kinase 31 (STK31), transcript variant 2, mRNA	NM_032944	Hs.309767	BC036476
UGT2B10	-4.6	5.2	UDP glucuronosyltransferase 2 family, polypeptide B10 (UGT2B10), mRNA	NM_001075	Hs.201634	BC113649
RBM34	-4.6	2.0	RNA binding motif protein 34 (RBM34), mRNA	NM_015014	Hs.535224	NM_015014
TPD52L1	-4.6	5.4	tumor protein D52-like 1 (TPD52L1), transcript variant 2, mRNA	NM_001003395	Hs.591347	U44429
INPP5F	-4.6	3.7	inositol polyphosphate-5-phosphatase F (INPP5F), transcript variant 1, mRNA	NM_014937	Hs.369755	NM_014937
ZFYVE16	-4.6	1.9	zinc finger, FYVE domain containing 16 (ZFYVE16), mRNA	NM_014733	Hs.482660	AB002303
A_23_P115902	-4.6	3.0	A_23_P115902	A_23_P115902	Unknown	
IFT80	-4.6	2.6	intraflagellar transport 80 homolog (Chlamydomonas) (IFT80), mRNA	NM_020800	Hs.478095	NM_020800
PRICKLE2	-4.6	3.3	prickle homolog 2 (Drosophila) (PRICKLE2), mRNA	NM_198859	Hs.699317	AL833539
LPHN1	-4.6	4.5	latrophilin 1 (LPHN1), transcript variant 1, mRNA	NM_001008701	Hs.654658	NM_001008701
AK7	-4.6	3.4	adenylate kinase 7 (AK7), mRNA	NM_152327	Hs.667462	NM_152327
OBSCN	-4.6	5.7	cDNA FLJ14124 fis, clone MAMMA1002498.	AK024186	Unknown	
HMGN2	-4.6	2.8	high-mobility group nucleosomal binding domain 2 (HMGN2), mRNA	NM_005517	Hs.181163	BC110390
PLB1	-4.6	3.3	phospholipase B1 (PLB1), mRNA	NM_153021	Hs.444933	NM_153021
COL4A3	-4.6	12.2	collagen, type IV, alpha 3 (Goodpasture antigen) (COL4A3), transcript variant 1, mRNA	NM_000091	Hs.570065	NM_000091
A1BG	-4.6	5.2	alpha-1-B glycoprotein (A1BG), mRNA	NM_130786	Hs.529161	NM_130786
SEPP1	-4.6	12.8	selenoprotein P, plasma, 1 (SEPP1), mRNA	NM_005410	Hs.700640	BC030009
CDKN1C	-4.6	9.8	cyclin-dependent kinase inhibitor 1C (p57, Kip2) (CDKN1C), mRNA	NM_000076	Hs.106070	BC067842
FLJ33790	-4.6	3.1	hypothetical protein FLJ33790 (FLJ33790), mRNA	NM_001039548	Hs.292451	AL050370
PTPRC	-4.6	2.5	protein tyrosine phosphatase, receptor type, C (PTPRC), transcript variant 1, mRNA	NM_002838	Hs.654514	NM_002838
EDN3	-4.6	51.0	endothelin 3 (EDN3), transcript variant 2, mRNA	NM_207032	Hs.1408	BC053866
THC2657613	-4.6	9.7	Q5NT18_ENTHI (Q5NT18) Small GTPase EhRabM3 (Fragment), partial (7%)	THC2657613	Unknown	
LOC399744	-4.6	3.4	hypothetical LOC399744 (LOC399744), mRNA	NM_001013665	Unknown	
GNAZ	-4.6	17.6	guanine nucleotide binding protein (G protein), alpha z polypeptide (GNAZ), mRNA	NM_002073	Hs.584760	BC037333
RAB3GAP2	-4.6	2.4	RAB3 GTPase activating protein subunit 2 (non-catalytic) (RAB3GAP2), mRNA	NM_012414	Hs.654849	BC036513
ZNF114	-4.6	7.5	zinc finger protein 114 (ZNF114), mRNA	NM_153608	Hs.511883	AK075062
LYST	-4.6	2.3	lysosomal trafficking regulator (LYST), transcript variant 1, mRNA	NM_000081	Hs.532411	NM_000081
LOC440248	-4.5	2.8	hypothetical LOC440248 (LOC440248), mRNA	NM_199045	Unknown	
AASDHPPT	-4.5	2.0	aminoadipate-semialdehyde dehydrogenase-phosphopantetheinyl transferase (AASDHPPT), mRNA	NM_015423	Hs.524009	BX537665
AK123972	-4.5	6.5	cDNA FLJ41978 fis, clone SKNSH2000482.	AK123972	Hs.435458	BC146776
RHOH	-4.5	4.3	ras homolog gene family, member H (RHOH), mRNA	NM_004310	Hs.694951	NM_004310
MAPK4	-4.5	5.6	H.sapiens 63 kDa protein kinase related to rat ERK3.	X59727	Hs.433728	BC050299
THC2668359	-4.5	8.7	THC2668359	THC2668359	Unknown	
GUSBP1	-4.5	3.3	glucuronidase, beta pseudogene 1 (GUSBP1), mRNA	NM_207331	Unknown	
SSTR2	-4.5	9.8	somatostatin receptor 2 (SSTR2), mRNA	NM_001050	Hs.514451	NM_001050
BRWD3	-4.5	2.3	bromodomain and WD repeat domain containing 3 (BRWD3), mRNA	NM_153252	Hs.170667	AY497052
AK094786	-4.5	11.5	cDNA FLJ37467 fis, clone BRAWH2011920.	AK094786	Hs.616436	AK094786
GPR137C	-4.5	4.2	cDNA FLJ41959 fis, clone PUAEN2002489, moderately similar to putative seven pass transmembrane protein (TM7SF1) mRNA.	ENST00000321662	Unknown	
RNF2	-4.5	2.8	ring finger protein 2 (RNF2), mRNA	NM_007212	Hs.591490	NM_007212
ALDOB	-4.5	72.7	aldolase B, fructose-bisphosphate (ALDOB), mRNA	NM_000035	Hs.530274	NM_000035
CXXC5	-4.5	4.3	CXXC finger 5 (CXXC5), mRNA	NM_016463	Hs.189119	BC006428
COL9A1	-4.5	9.0	collagen, type IX, alpha 1 (COL9A1), transcript variant 1, mRNA	NM_001851	Hs.590892	NM_001851
AB002449	-4.5	2.1	mRNA from chromosome 5q21-22, clone:843Ex.	AB002449	Hs.699267	AB002449
LRRC51	-4.5	1.7	leucine rich repeat containing 51 (LRRC51), mRNA	NM_145309	Hs.317243	AF289588
APLP1	-4.5	3.1	amyloid beta (A4) precursor-like protein 1 (APLP1), transcript variant 2, mRNA	NM_005166	Hs.74565	NM_001024807
SCML2	-4.5	2.6	sex comb on midleg-like 2 (Drosophila) (SCML2), mRNA	NM_006089	Hs.495774	BC040497
ONECUT1	-4.5	4.8	one cut domain, family member 1 (ONECUT1), mRNA	NM_004498	Hs.658573	U96173
IGF1	-4.5	4.1	insulin-like growth factor 1 (somatomedin C) (IGF1), mRNA	NM_000618	Hs.160562	NM_000618
SSTR2	-4.5	5.6	somatostatin receptor 2 (SSTR2), mRNA	NM_001050	Hs.514451	NM_001050

SCD5	-4.5	3.1	stearoyl-CoA desaturase 5 (SCD5), transcript variant 1, mRNA	NM_001037582	Hs.379191	NM_001037582
PTPRU	-4.5	5.1	protein tyrosine phosphatase, receptor type, U (PTPRU), transcript variant 3, mRNA	NM_005704	Hs.19718	NM_005704
FAM123A	-4.5	8.4	family with sequence similarity 123A (FAM123A), transcript variant 1, mRNA	NM_152704	Hs.528335	AL832586
HKDC1	-4.5	8.5	hexokinase domain containing 1 (HKDC1), mRNA	NM_025130	Unknown	
RFX3	-4.5	3.1	Transcription factor RFX3.	ENST00000382004	Unknown	
PPIG	-4.5	1.9	peptidylprolyl isomerase G (cyclophilin G) (PPIG), mRNA	NM_004792	Hs.470544	U40763
USP37	-4.5	2.0	ubiquitin specific peptidase 37 (USP37), mRNA	NM_020935	Hs.166068	BX538024
RSHL3	-4.5	2.8	radial spokehead-like 3 (RSHL3), mRNA	NM_001010892	Hs.160380	NM_001010892
CDKN2B	-4.5	34.9	cyclin-dependent kinase inhibitor 2B (p15, inhibits CDK4) (CDKN2B), transcript variant 2, mRNA	NM_078487	Hs.72901	NM_078487
RB1	-4.5	1.8	retinoblastoma 1 (including osteosarcoma) (RB1), mRNA	NM_000321	Hs.408528	L41870
FBXO41	-4.5	3.4	cDNA FLJ30559 fis, clone BRAWH2004095.	AK055121	Hs.23158	NM_001080410
ANKRD12	-4.5	2.3	ankyrin repeat domain 12 (ANKRD12), mRNA	NM_015208	Hs.464585	NM_015208
KIAA1571	-4.5	3.7	mRNA for KIAA1571 protein, partial cds.	AB046791	Hs.110489	XM_371590
HSP90AA1	-4.5	1.9	heat shock protein 90kDa alpha (cytosolic), class A member 1 (HSP90AA1), transcript variant 2, mRNA	NM_005348	Hs.525600	NM_001017963
A_32_P31827	-4.5	2.6	A_32_P31827	A_32_P31827	Unknown	
CR620599	-4.5	4.1	full-length cDNA clone CS0DF021YI14 of Fetal brain of (human).	CR620599	Hs.369581	NM_024615
SLC30A7	-4.5	2.1	solute carrier family 30 (zinc transporter), member 7 (SLC30A7), mRNA	NM_133496	Hs.533903	BC064692
BC033590	-4.5	3.4	clone IMAGE:4344826, mRNA.	BC033590	Hs.597585	BC033590
CHKA	-4.5	2.2	choline kinase alpha (CHKA), transcript variant 1, mRNA	NM_001277	Hs.569019	AK054792
C14orf178	-4.5	5.5	chromosome 14 open reading frame 178 (C14orf178), mRNA	NM_174943	Hs.375834	NM_174943
PDGFD	-4.5	3.9	platelet derived growth factor D (PDGFD), transcript variant 1, mRNA	NM_025208	Hs.352298	BC030645
BCLAF1	-4.5	2.0	BCL2-associated transcription factor 1 (BCLAF1), transcript variant 1, mRNA	NM_014739	Hs.486542	NM_014739
INPP5F	-4.5	4.2	inositol polyphosphate-5-phosphatase F (INPP5F), transcript variant 1, mRNA	NM_014937	Hs.369755	NM_014937
SAA4	-4.5	6.3	serum amyloid A4, constitutive (SAA4), mRNA	NM_006512	Hs.654493	BG564326
LIX1	-4.5	185.7	Lix1 homolog (mouse) (LIX1), mRNA	NM_153234	Hs.656702	BC036467
C22orf31	-4.5	3.1	chromosome 22 open reading frame 31 (C22orf31), mRNA	NM_015370	Hs.50891	AL035364
HSF2	-4.5	2.1	heat shock transcription factor 2 (HSF2), mRNA	NM_004506	Hs.158195	BC112323
DUSP10	-4.5	4.2	dual specificity phosphatase 10 (DUSP10), transcript variant 1, mRNA	NM_007207	Hs.497822	BC031405
NBPF11	-4.4	2.2	neuroblastoma breakpoint family, member 11 (NBPF11), mRNA	NM_183372	Hs.636561	BX648497
THC2663361	-4.4	3.8	THC2663361	THC2663361	Unknown	
THC2652887	-4.4	8.8	Q2PGH6_HAELO (Q2PGH6) Mucin (Fragment), partial (14%)	THC2652887	Unknown	
BC030106	-4.4	2.7	cDNA clone IMAGE:4797878.	BC030106	Hs.547831	BI914892
HMGN2	-4.4	2.5	high-mobility group nucleosomal binding domain 2 (HMGN2), mRNA	NM_005517	Hs.181163	BC110390
AMH	-4.4	4.0	anti-Mullerian hormone (AMH), mRNA	NM_000479	Hs.112432	NM_000479
WDR67	-4.4	2.3	WD repeat domain 67 (WDR67), mRNA	NM_145647	Hs.492716	AK056434
ORC6L	-4.4	3.4	origin recognition complex, subunit 6 like (yeast) (ORC6L), mRNA	NM_014321	Hs.49760	NM_014321
1-Dec	-4.4	15.6	clone IMAGE:5526534, mRNA, partial cds.	BC030567	Hs.148841	AK056153
AK094525	-4.4	7.5	cDNA FLJ37206 fis, clone BRALZ2007545.	AK094525	Hs.587467	BX647732
RSHL3	-4.4	8.1	radial spokehead-like 3 (RSHL3), mRNA	NM_001010892	Hs.160380	NM_001010892
THC2496620	-4.4	4.3	THC2496620	THC2496620	Unknown	
LOC92196	-4.4	10.2	similar to death-associated protein (LOC92196), mRNA	NM_001017920	Hs.59761	EL950760
HSP90AA1	-4.4	1.9	heat shock protein 90kDa alpha (cytosolic), class A member 1 (HSP90AA1), transcript variant 2, mRNA	NM_005348	Hs.525600	NM_001017963
AK055428	-4.4	3.6	cDNA FLJ30866 fis, clone FEBRA2004110, highly similar to PHOSPHOLIPASE ADRAB-B PRECURSOR (EC 3.1.-.-).	AK055428	Unknown	
CGI-38	-4.4	3.1	brain specific protein (CGI-38), mRNA	NM_016140	Hs.534458	CR622352
SLC16A14	-4.4	7.8	solute carrier family 16, member 14 (monocarboxylic acid transporter 14) (SLC16A14), mRNA	NM_152527	Hs.504317	BC065524
CYP3A5	-4.4	7.4	cytochrome P450, family 3, subfamily A, polypeptide 5 (CYP3A5), mRNA	NM_000777	Hs.695915	BX537676
TSNAXIP1	-4.4	2.1	translin-associated factor X interacting protein 1 (TSNAXIP1), mRNA	NM_018430	Hs.632212	BC111018
A_32_P13202	-4.4	3.3	A_32_P13202	A_32_P13202	Unknown	
FLJ36032	-4.4	3.1	cDNA FLJ36032 fis, clone TESTI2017069.	AK093351	Hs.297967	AK093351
DICER1	-4.4	2.9	Dicer1, Dcr-1 homolog (Drosophila) (DICER1), transcript variant 1, mRNA	NM_177438	Hs.87889	NM_177438
RFPL3S	-4.4	3.0	ret finger protein-like 3 antisense (RFPL3S) on chromosome 22	NR_001450	Unknown	
MANEAL	-4.4	5.1	mannosidase, endo-alpha-like (MANEAL), transcript variant 1, mRNA	NM_001031740	Hs.534562	AK055996
THC2527406	-4.4	2.2	THC2527406	THC2527406	Unknown	

C3orf39	-4.4	2.3	chromosome 3 open reading frame 39 (C3orf39), mRNA	NM_032806	Hs.12313	AK124737
RASEF	-4.4	4.4	RAS and EF-hand domain containing (RASEF), mRNA	NM_152573	Hs.657750	NM_152573
THC2536674	-4.4	3.6	AA353695 EST62114 Jurkat T-cells V cDNA 5' end, mRNA sequence	THC2536674	Unknown	
AK026418	-4.4	8.0	cDNA: FLJ22765 fis, clone KAIA1180.	AK026418	Hs.663064	AK026418
LOC285398	-4.4	2.1	cDNA FLJ25945 fis, clone JTH12731.	AK098811	Hs.681813	AK098811
NBPF15	-4.4	2.5	neuroblastoma breakpoint family, member 15 (NBPF15), mRNA	NM_173638	Hs.655246	NM_173638
HIST1H3G	-4.4	3.3	histone cluster 1, H3g, mRNA (cDNA clone MGC:20474 IMAGE:4563941), complete cds.	BC012185	Unknown	
TRMT5	-4.4	2.4	TRM5 tRNA methyltransferase 5 homolog (S. cerevisiae) (TRMT5), mRNA	NM_020810	Hs.380159	NM_020810
GUSBP1	-4.4	2.7	glucuronidase, beta pseudogene 1 (GUSBP1), mRNA	NM_207331	Unknown	
SCAMP5	-4.4	3.6	secretory carrier membrane protein 5 (SCAMP5), mRNA	NM_138967	Hs.374180	AL390185
ZSWIM5	-4.4	3.5	mRNA for KIAA1511 protein, partial cds.	AB040944	Hs.656613	NM_020883
THC2671048	-4.4	2.8	Q3DWD9_CHLAU (Q3DWD9) YLP motif, partial (6%)	THC2671048	Unknown	
DQ786194	-4.4	4.2	clone HLS_IMAGE_119768 mRNA sequence.	DQ786194	Hs.645602	DQ786194
PLB1	-4.4	3.4	phospholipase B1 (PLB1), mRNA	NM_153021	Hs.444933	NM_153021
PTN	-4.4	4.7	pleiotrophin (heparin binding growth factor 8, neurite growth-promoting factor 1) (PTN), mRNA	NM_002825	Hs.371249	CR624136
KIAA1383	-4.4	1.9	KIAA1383 protein, mRNA (cDNA clone MGC:33261 IMAGE:5266476), complete cds.	BC036663	Hs.160373	NM_019090
TM2D1	-4.4	1.7	TM2 domain containing 1 (TM2D1), mRNA	NM_032027	Hs.656790	NM_032027
PBX1	-4.4	4.3	Pre-B-cell leukemia transcription factor 1 (Homeobox protein PBX1) (Homeobox protein PRL).	ENST00000328681	Unknown	
C20orf119	-4.4	3.4	cDNA: FLJ23107 fis, clone LNG07738.	AK026760	Hs.641481	AK124047
ZMYM2	-4.4	3.4	mRNA; cDNA DKFZp564B162 (from clone DKFZp564B162).	AL136621	Hs.644041	BX648905
HSP90AA1	-4.4	1.9	heat shock protein 90kDa alpha (cytosolic), class A member 1 (HSP90AA1), transcript variant 2, mRNA	NM_005348	Hs.525600	NM_001017963
PUM1	-4.4	2.3	pumilio homolog 1 (Drosophila) (PUM1), transcript variant 1, mRNA	NM_001020658	Hs.281707	NM_001020658
AK056384	-4.4	3.5	cDNA FLJ31822 fis, clone NT2RP6000043.	AK056384	Hs.657123	BC038361
RB1	-4.4	1.8	retinoblastoma 1 (including osteosarcoma) (RB1), mRNA	NM_000321	Hs.408528	L41870
CD104030	-4.4	3.7	AGENCOURT_14006900 NIH_MGC_186 cDNA clone IMAGE:30372058 5', mRNA sequence	CD104030	Hs.271498	CD104030
AKAP14	-4.4	35.6	A kinase (PRKA) anchor protein 14 (AKAP14), transcript variant 1, mRNA	NM_178813	Hs.592245	BC066357
NSUN7	-4.4	16.5	NOL1/NOP2/Sun domain family, member 7 (NSUN7), mRNA	NM_024677	Hs.590923	BC042401
SMO	-4.4	5.0	smoothened homolog (Drosophila) (SMO), mRNA	NM_005631	Hs.437846	NM_005631
RHOH	-4.4	4.0	ras homolog gene family, member H (RHOH), mRNA	NM_004310	Hs.694951	NM_004310
ATCAY	-4.4	6.4	ataxia, cerebellar, Cayman type (caytaxin) (ATCAY), mRNA	NM_033064	Hs.418055	NM_033064
C10orf118	-4.4	2.5	chromosome 10 open reading frame 118 (C10orf118), mRNA	NM_018017	Hs.159066	NM_018017
RB1	-4.4	1.8	retinoblastoma 1 (including osteosarcoma) (RB1), mRNA	NM_000321	Hs.408528	L41870
NAALAD2	-4.4	9.0	N-acetylated alpha-linked acidic dipeptidase 2 (NAALAD2), mRNA	NM_005467	Hs.503560	AK075390
CR594735	-4.3	6.7	full-length cDNA clone CS0DE011YB11 of Placenta of (human).	CR594735	Hs.153408	AK001432
KIF6	-4.3	3.6	kinesin family member 6 (KIF6), mRNA	NM_145027	Hs.588202	AL832634
BIRC4	-4.3	2.2	baculoviral IAP repeat-containing 4 (BIRC4), mRNA	NM_001167	Hs.356076	NM_001167
WNT2B	-4.3	26.8	wingless-type MMTV integration site family, member 2B (WNT2B), transcript variant WNT-2B1, mRNA	NM_004185	Hs.258575	AK127449
LOC285498	-4.3	3.7	hypothetical protein LOC285498 (LOC285498), mRNA	NM_194439	Hs.248290	AK124578
RP11-130N24.1	-4.3	74.6	KIAA2022 protein (KIAA2022), mRNA	NM_001008537	Hs.124128	AY563507
ENST000003555	-4.3	2.7	solute carrier family 35, member E2	ENST00000355570	Unknown	
NR0B2	-4.3	2.9	nuclear receptor subfamily 0, group B, member 2 (NR0B2), mRNA	NM_021969	Hs.427055	BC030207
PRKCI	-4.3	1.9	protein kinase C, iota (PRKCI), mRNA	NM_002740	Hs.478199	NM_002740
AK057710	-4.3	3.4	cDNA FLJ33148 fis, clone UTERU2000238.	AK057710	Hs.597376	AK057710
A_32_P139311	-4.3	3.5	A_32_P139311	A_32_P139311	Unknown	
AK125361	-4.3	9.4	cDNA FLJ43371 fis, clone NTONG2005969.	AK125361	Hs.276808	NM_002409
EFHC1	-4.3	4.2	EFHC1 (EFHC1) mRNA, partial cds.	AY608689	Hs.403171	AY608689
GGPS1	-4.3	1.9	geranylgeranyl diphosphate synthase 1 (GGPS1), transcript variant 1, mRNA	NM_004837	Hs.647791	NM_004837
IVNS1ABP	-4.3	2.7	influenza virus NS1A binding protein (IVNS1ABP), transcript variant 2, mRNA	NM_016389	Unknown	
BLZF1	-4.3	1.9	Golgin 45 (Basic leucine zipper nuclear factor 1) (JEM-1) (p45 basic leucine-zipper nuclear factor).	ENST00000367808	Unknown	
THC2608652	-4.3	3.5	ALU1_HUMAN (P39188) Alu subfamily J sequence contamination warning entry, partial (12%)	THC2608652	Unknown	
RB1	-4.3	1.8	retinoblastoma 1 (including osteosarcoma) (RB1), mRNA	NM_000321	Hs.408528	L41870
DISP1	-4.3	3.7	dispatched homolog 1 (Drosophila) (DISP1), mRNA	NM_032890	Hs.528817	NM_032890

CAPSL	-4.3	12.7	calcyphosine-like (CAPSL), transcript variant 1, mRNA	NM_144647	Hs.55150	NM_144647
THC2648398	-4.3	2.9	THC2648398	THC2648398	Unknown	
FLJ46385	-4.3	2.4	FLJ46385 protein (FLJ46385), mRNA	NM_001001675	Unknown	
HSP90AA1	-4.3	1.9	heat shock protein 90kDa alpha (cytosolic), class A member 1 (HSP90AA1), transcript variant 2, mRNA	NM_005348	Hs.525600	NM_001017963
HYDIN	-4.3	4.0	hydrocephalus inducing homolog (mouse) (HYDIN), transcript variant 1, mRNA	NM_032821	Hs.461229	NM_032821
GLT25D2	-4.3	4.4	glycosyltransferase 25 domain containing 2 (GLT25D2), mRNA	NM_015101	Hs.387995	NM_015101
IRF2BP2	-4.3	2.4	interferon regulatory factor 2 binding protein 2 (IRF2BP2), transcript variant 1, mRNA	NM_182972	Hs.350268	NM_182972
NBPF20	-4.3	1.8	neuroblastoma breakpoint family, member 20 (NBPF20), mRNA	NM_001037675	Hs.515947	BX538005
SLCO1B1	-4.3	10.1	solute carrier organic anion transporter family, member 1B1 (SLCO1B1), mRNA	NM_006446	Hs.449738	AF205071
RB1	-4.3	1.9	retinoblastoma 1 (including osteosarcoma) (RB1), mRNA	NM_000321	Hs.408528	L41870
RLF	-4.3	2.6	rearranged L-myc fusion (RLF), mRNA	NM_012421	Hs.205627	U22377
THC2634862	-4.3	12.5	NM_063888 TAF (TBP-associated transcription factor) family member (taf-13) {Caenorhabditis elegans} (exp=-1; wgp=0; cg=0), partial (15%)	THC2634862	Unknown	
FGL1	-4.3	30.6	fibrinogen-like 1 (FGL1), transcript variant 4, mRNA	NM_201553	Hs.491143	CR598703
APPBP2	-4.3	2.4	amyloid beta precursor protein (cytoplasmic tail) binding protein 2 (APPBP2), mRNA	NM_006380	Hs.84084	NM_006380
AI090937	-4.3	3.7	AI090937 qa52c01.s1 Soares_NhHMPu_S1 cDNA clone IMAGE:1690368 3', mRNA sequence	AI090937	Hs.677678	BG329526
1-Mar	-4.3	4.0	cDNA FLJ20668 fis, clone KAIA585.	AK000675	Hs.696248	AK025005
THC2741789	-4.3	15.1	THC2741789	THC2741789	Unknown	
CDK6	-4.3	3.3	cyclin-dependent kinase 6 (CDK6), mRNA	NM_001259	Hs.119882	NM_001259
AL109696	-4.3	15.7	mRNA full length insert cDNA clone EUROIMAGE 21920.	AL109696	Hs.185701	AK094929
TRAF5	-4.3	2.5	TNF receptor-associated factor 5 (TRAF5), transcript variant 1, mRNA	NM_004619	Hs.523930	AB000509
TIGD1	-4.3	2.4	tigger transposable element derived 1 (TIGD1), mRNA	NM_145702	Hs.211823	BC035143
DIRAS2	-4.3	3.1	DIRAS family, GTP-binding RAS-like 2 (DIRAS2), mRNA	NM_017594	Hs.165636	NM_017594
DOCK8	-4.3	2.7	dedicator of cytokinesis 8 (DOCK8), mRNA	NM_203447	Hs.132599	AL832270
PAFAH1B1	-4.3	1.7	platelet-activating factor acetylhydrolase, isoform Ib, alpha subunit 45kDa (PAFAH1B1), mRNA	NM_000430	Hs.77318	NM_000430
RPE65	-4.3	21.1	retinal pigment epithelium-specific protein 65kDa (RPE65), mRNA	NM_000329	Hs.2133	U18991
C16orf72	-4.3	1.6	chromosome 16 open reading frame 72 (C16orf72), mRNA	NM_014117	Hs.221497	NM_014117
ONECUT2	-4.3	9.3	one cut domain, family member 2 (ONECUT2), mRNA	NM_004852	Hs.194725	NM_004852
C21orf62	-4.3	7.4	chromosome 21 open reading frame 62 (C21orf62), mRNA	NM_019596	Hs.517235	NM_019596
FLJ45445	-4.3	3.4	FLJ45445 protein (FLJ45445), mRNA	NM_001004321	Unknown	
ARL3	-4.3	2.4	ADP-ribosylation factor-like 3 (ARL3), mRNA	NM_004311	Hs.182215	NM_004311
MND1	-4.3	4.1	meiotic nuclear divisions 1 homolog (S. cerevisiae) (MND1), mRNA	NM_032117	Hs.294088	BG496847
ZNF254	-4.3	3.0	zinc finger protein 254 (ZNF254), transcript variant 1, mRNA	NM_203282	Hs.434406	BC043147
GLULD1	-4.3	14.7	glutamate-ammonia ligase (glutamine synthetase) domain containing 1 (GLULD1), mRNA	NM_016571	Hs.149585	AF242388
RB1	-4.3	1.8	retinoblastoma 1 (including osteosarcoma) (RB1), mRNA	NM_000321	Hs.408528	L41870
RBP1	-4.3	4.5	retinol binding protein 1, cellular (RBP1), mRNA	NM_002899	Hs.529571	BM926478
TMEM106C	-4.3	2.8	transmembrane protein 106C (TMEM106C), mRNA	NM_024056	Hs.596726	AK056442
ZBTB1	-4.3	3.2	zinc finger and BTB domain containing 1 (ZBTB1), mRNA	NM_014950	Hs.655536	AB023214
MYBL1	-4.3	2.2	H.sapiens a-myb mRNA.	X66087	Hs.654538	NM_001080416
C1orf125	-4.3	3.9	chromosome 1 open reading frame 125 (C1orf125), transcript variant 1, mRNA	NM_144696	Hs.658505	BX647935
HSDL1	-4.3	2.0	hydroxysteroid dehydrogenase like 1 (HSDL1), mRNA	NM_031463	Hs.555992	NM_031463
BC016291	-4.3	7.7	cDNA clone IMAGE:3910094, partial cds.	BC016291	Hs.638073	BC016291
THC2657612	-4.3	5.7	THC2657612	THC2657612	Unknown	
DUSP10	-4.3	5.1	dual specificity phosphatase 10 (DUSP10), transcript variant 1, mRNA	NM_007207	Hs.497822	BC031405
WDR78	-4.3	1.9	WD repeat domain 78 (WDR78), transcript variant 2, mRNA	NM_207014	Hs.49421	AK127011
SLC16A10	-4.3	6.3	solute carrier family 16, member 10 (aromatic amino acid transporter) (SLC16A10), mRNA	NM_018593	Hs.591327	AK172789
NOTCH2	-4.3	2.3	Notch homolog 2 (Drosophila) (NOTCH2), mRNA	NM_024408	Hs.487360	NM_024408
FLJ10781	-4.3	3.8	hypothetical protein FLJ10781 (FLJ10781), mRNA	NM_018215	Hs.8395	BC032508
A_32_P113462	-4.3	15.6	A_32_P113462	A_32_P113462	Unknown	
BQ926066	-4.3	2.7	AGENCOURT_8878877 Lupski_sciatic_nerve cDNA clone IMAGE:6202171 5', mRNA sequence	BQ926066	Hs.559765	BQ926066
PLS3	-4.3	4.5	plastin 3 (T isoform) (PLS3), mRNA	NM_005032	Hs.496622	BC056898
FLJ36032	-4.2	6.6	cDNA FLJ36032 fis, clone TESTI2017069.	AK093351	Hs.297967	AK093351
CR603982	-4.2	6.4	full-length cDNA clone CS0DF021YL03 of Fetal brain of (human).	CR603982	Hs.120633	BX649128

GALNT13	-4.2	5.0	UDP-N-acetyl-alpha-D-galactosamine:polypeptide N-acetylgalactosaminyltransferase 13 (GalNAc-T13) (GALNT13), mRNA	NM_052917	Hs.470277	NM_052917
HSP90AA1	-4.2	1.9	heat shock protein 90kDa alpha (cytosolic), class A member 1 (HSP90AA1), transcript variant 2, mRNA	NM_005348	Hs.525600	NM_001017963
GLI3	-4.2	2.7	GLI-Kruppel family member GLI3 (Greig cephalopolysyndactyly syndrome) (GLI3), mRNA	NM_000168	Hs.21509	NM_000168
SPHAR	-4.2	2.5	S-phase response (cyclin-related) (SPHAR), mRNA	NM_006542	Hs.296169	AY585832
PIWIL4	-4.2	2.7	piwi-like 4 (Drosophila) (PIWIL4), mRNA	NM_152431	Hs.660188	CR749642
AK091573	-4.2	10.3	cDNA FLJ34254 fis, clone FCBBF5000325.	AK091573	Hs.26409	BC041405
CR608907	-4.2	2.7	full-length cDNA clone CS0DM002YA18 of Fetal liver of (human).	CR608907	Hs.436379	CR608907
PRKCI	-4.2	2.0	protein kinase C, iota (PRKCI), mRNA	NM_002740	Hs.478199	NM_002740
C1QTNF3	-4.2	4.2	C1q and tumor necrosis factor related protein 3 (C1QTNF3), transcript variant 2, mRNA	NM_181435	Hs.171929	NM_181435
A_23_P357504	-4.2	4.1	A_23_P357504	A_23_P357504	Unknown	
CALD1	-4.2	3.4	cDNA FLJ12160 fis, clone MAMMA1000567.	AK022222	Unknown	
RHOH	-4.2	4.2	ras homolog gene family, member H (RHOH), mRNA	NM_004310	Hs.694951	NM_004310
UBXD3	-4.2	4.6	UBX domain-containing protein 3.	ENST00000375099	Unknown	
AHI1	-4.2	2.0	Abelson helper integration site 1 (AHI1), mRNA	NM_017651	Hs.386684	NM_017651
KIAA1715	-4.2	2.5	Protein lunapark.	ENST00000272748	Unknown	
SPINK5	-4.2	6.6	serine peptidase inhibitor, Kazal type 5 (SPINK5), mRNA	NM_006846	Hs.331555	DQ149928
PCDHA5	-4.2	3.1	protocadherin alpha 5 (PCDHA5), transcript variant 2, mRNA	NM_031501	Hs.199343	NM_014005
FOXF2	-4.2	69.6	forkhead box F2 (FOXF2), mRNA	NM_001452	Hs.484423	NM_001452
PCDHB2	-4.2	7.4	protocadherin beta 2 (PCDHB2), mRNA	NM_018936	Hs.533023	BC098575
SCGN	-4.2	2.9	secretagogin, EF-hand calcium binding protein (SCGN), mRNA	NM_006998	Hs.116428	NM_006998
LOC399744	-4.2	5.4	hypothetical LOC399744 (LOC399744), mRNA	NM_001013665	Unknown	
THC2654381	-4.2	2.6	THC2654381	THC2654381	Unknown	
PTPRC	-4.2	2.7	protein tyrosine phosphatase, receptor type, C (PTPRC), transcript variant 1, mRNA	NM_002838	Hs.654514	NM_002838
LOC340286	-4.2	11.8	cDNA FLJ42138 fis, clone TESTI2036684.	AK124132	Hs.144075	AK124132
DUOX1	-4.2	2.9	mRNA; cDNA DKFZp434L0610 (from clone DKFZp434L0610); partial cds.	AL137592	Hs.272813	NM_017434
BF207040	-4.2	3.5	601870125F1 NIH_MGC_19 cDNA clone IMAGE:4100239 5', mRNA sequence	BF207040	Hs.353024	BF207040
LEF1	-4.2	45.2	lymphoid enhancer-binding factor 1 (LEF1), mRNA	NM_016269	Hs.555947	AK128255
THC2733949	-4.2	9.4	Q96EM6_HUMAN (Q96EM6) LOC440700 protein, partial (42%)	THC2733949	Unknown	
THC2654993	-4.2	3.0	THC2654993	THC2654993	Unknown	
JTB	-4.2	1.8	jumping translocation breakpoint (JTB), mRNA	NM_006694	Hs.6396	BQ062593
KIAA1009	-4.2	4.1	KIAA1009 (KIAA1009), mRNA	NM_014895	Hs.485865	NM_014895
CEP290	-4.2	2.3	centrosomal protein 290kDa (CEP290), mRNA	NM_025114	Hs.150444	NM_025114
TRPV1	-4.2	3.2	transient receptor potential cation channel, subfamily V, member 1 (TRPV1), transcript variant 3, mRNA	NM_080706	Hs.655380	NM_080706
ENST000003763	-4.2	1.8	kinesin family member 27	ENST00000376347	Unknown	
RPA2	-4.2	2.4	replication protein A2, 32kDa (RPA2), mRNA	NM_002946	Hs.79411	NM_002946
BRUNOL4	-4.2	19.1	PREDICTED: bruno-like 4, RNA binding protein (Drosophila) (BRUNOL4), mRNA	XM_001128998	Unknown	
BC036626	-4.2	1.8	cDNA clone IMAGE:5288439.	BC036626	Hs.655991	BC013799
AB051467	-4.2	3.2	mRNA for KIAA1680 protein, partial cds.	AB051467	Unknown	
UPF3B	-4.2	2.9	UPF3 regulator of nonsense transcripts homolog B (yeast) (UPF3B), transcript variant 1, mRNA	NM_080632	Hs.103832	NM_080632
THC2498136	-4.2	2.2	Q96316_ARATH (Q96316) Blue-copper binging protein III (Uclacyanin 3), partial (5%)	THC2498136	Unknown	
PROM1	-4.2	42.7	prominin 1 (PROM1), mRNA	NM_006017	Hs.614734	AF117225
RFPL1S	-4.2	49.5	ret finger protein-like 1 antisense (RFPL1S) on chromosome 22	NR_002727	Unknown	
RP13-102H20.1	-4.2	5.6	hypothetical protein FLJ30058 (FLJ30058), mRNA	NM_144967	Hs.22905	AK128203
THC2733339	-4.2	8.3	Q414Q2_KINRA (Q414Q2) Peptidase M15B and M15C, D,D-carboxypeptidase VanY/endolysins, partial (7%)	THC2733339	Unknown	
A_32_P173922	-4.2	2.3	A_32_P173922	A_32_P173922	Unknown	
NGEF	-4.2	9.0	neuronal guanine nucleotide exchange factor (NGEF), mRNA	NM_019850	Hs.97316	NM_019850
HACE1	-4.2	2.3	HECT domain and ankyrin repeat containing, E3 ubiquitin protein ligase 1 (HACE1), mRNA	NM_020771	Hs.434340	AB037741
MARK1	-4.2	3.3	MAP/microtubule affinity-regulating kinase 1 (MARK1), mRNA	NM_018650	Hs.497806	NM_018650
PTN	-4.2	4.3	pleiotrophin (heparin binding growth factor 8, neurite growth-promoting factor 1) (PTN), mRNA	NM_002825	Hs.371249	CR624136
TPBG	-4.2	8.4	Trophoblast glycoprotein precursor (5T4 oncofetal trophoblast glycoprotein) (5T4 oncotrophoblast glycoprotein) (5T4 oncofetal antigen) (M6P1).	ENST00000369750	Unknown	
RAVER2	-4.2	2.1	ribonucleoprotein, PTB-binding 2 (RAVER2), mRNA	NM_018211	Hs.591443	NM_018211

SLC22A3	-4.2	4.8	solute carrier family 22 (extraneuronal monoamine transporter), member 3 (SLC22A3), mRNA	NM_021977	Hs.567337	BX640965
KIAA1737	-4.2	2.0	KIAA1737 (KIAA1737), mRNA	NM_033426	Hs.22452	NM_033426
SEZ6L	-4.2	72.4	mRNA for KIAA0927 protein, partial cds.	AB023144	Hs.194766	AB023144
THC2660320	-4.2	2.0	ALU8_HUMAN (P39195) Alu subfamily SX sequence contamination warning entry, partial (10%)	THC2660320	Unknown	
PDE7A	-4.2	1.8	phosphodiesterase 7A (PDE7A), transcript variant 1, mRNA	NM_002603	Hs.584788	AF332652
PIGC	-4.2	2.7	H.sapiens gene from PAC 106H8.	AL035301	Unknown	
UBE2E3	-4.2	1.9	ubiquitin-conjugating enzyme E2E 3 (UBC4/5 homolog, yeast) (UBE2E3), transcript variant 1, mRNA	NM_006357	Hs.470804	BM912832
RP5-1054A22.3	-4.2	2.8	KIAA1755 protein (KIAA1755), mRNA	NM_001029864	Hs.472690	NM_001029864
RB1CC1	-4.2	2.2	RB1-inducible coiled-coil 1 (RB1CC1), mRNA	NM_014781	Hs.196102	NM_014781
THC2614991	-4.2	21.5	THC2614991	THC2614991	Unknown	
LAYN	-4.2	14.9	layilin (LAYN), mRNA	NM_178834	Hs.503831	AK096925
THC2637644	-4.2	15.6	THC2637644	THC2637644	Unknown	
ENST000003321	-4.2	2.4	PREDICTED: similar to HIStone family member (his-72) (LOC391769), mRNA	ENST00000332101	Unknown	
NBPF3	-4.2	3.1	neuroblastoma breakpoint family, member 3 (NBPF3), mRNA	NM_032264	Hs.325422	AK095602
GRIA3	-4.1	42.4	Glutamate receptor 3 precursor (GluR-3) (GluR-C) (GluR-K3) (Glutamate receptor ionotropic, AMPA 3) (AMPA-selective glutamate receptor 3).	ENST00000371256	Unknown	
HS3ST2	-4.1	3.8	heparan sulfate (glucosamine) 3-O-sulfotransferase 2 (HS3ST2), mRNA	NM_006043	Hs.115830	AY359095
ZCCHC12	-4.1	14.7	zinc finger, CCHC domain containing 12 (ZCCHC12), mRNA	NM_173798	Hs.21417	BC036572
CNN3	-4.1	2.5	calponin 3, acidic (CNN3), mRNA	NM_001839	Hs.700591	NM_001839
A_24_P334208	-4.1	43.3	A_24_P334208	A_24_P334208	Unknown	
THC2544148	-4.1	2.4	Q6TXI7_RAT (Q6TXI7) LRRGT00012, partial (5%)	THC2544148	Unknown	
KIAA1622	-4.1	16.1	KIAA1622 (KIAA1622), transcript variant 1, mRNA	NM_058237	Hs.259599	NM_058237
NEO1	-4.1	5.3	neogenin homolog 1 (chicken) (NEO1), mRNA	NM_002499	Hs.699228	AB209412
H3F3A	-4.1	2.6	H3 histone, family 3A (H3F3A), mRNA	NM_002107	Hs.546259	CR621187
PDE11A	-4.1	25.4	phosphodiesterase 11A (PDE11A), transcript variant 4, mRNA	NM_016953	Hs.570273	NM_016953
A_24_P650011	-4.1	2.7	A_24_P650011	A_24_P650011	Unknown	
HSP90AA1	-4.1	1.9	heat shock protein 90kDa alpha (cytosolic), class A member 1 (HSP90AA1), transcript variant 2, mRNA	NM_005348	Hs.525600	NM_001017963
KIAA1840	-4.1	1.9	KIAA1840 (KIAA1840), mRNA	NM_025137	Hs.656271	NM_025137
C16orf73	-4.1	8.0	chromosome 16 open reading frame 73 (C16orf73), mRNA	NM_152764	Hs.662737	BC029829
SOSTDC1	-4.1	6.4	sclerostin domain containing 1 (SOSTDC1), mRNA	NM_015464	Hs.648106	AK093408
RRM2	-4.1	2.4	ribonucleotide reductase M2 polypeptide (RRM2), mRNA	NM_001034	Hs.226390	AK123010
KCTD16	-4.1	13.4	potassium channel tetramerisation domain containing 16 (KCTD16), mRNA	NM_020768	Hs.693927	AB037738
ENAH	-4.1	4.5	enabled homolog (Drosophila) (ENAH), transcript variant 1, mRNA	NM_001008493	Hs.497893	NM_001008493
BI771054	-4.1	2.5	603059613F1 NIH_MGC_122 cDNA clone IMAGE:5209035 5', mRNA sequence	BI771054	Hs.341729	BI771054
PCM1	-4.1	2.1	pericentriolar material 1 (PCM1), mRNA	NM_006197	Hs.491148	NM_006197
ZMYM2	-4.1	2.4	zinc finger, MYM-type 2 (ZMYM2), mRNA	NM_003453	Hs.644041	BX648905
DRD1	-4.1	3.9	dopamine receptor D1 (DRD1), mRNA	NM_000794	Hs.2624	NM_000794
KIAA1344	-4.1	2.6	KIAA1344 (KIAA1344), mRNA	NM_020784	Hs.532609	BC142650
DNAH7	-4.1	7.1	dynein, axonemal, heavy chain 7 (DNAH7), mRNA	NM_018897	Hs.97403	AB023161
AK094623	-4.1	3.3	cDNA FLJ37304 fis, clone BRAMY2016070.	AK094623	Hs.675200	AK094623
TMCO1	-4.1	1.8	transmembrane and coiled-coil domains 1 (TMCO1), mRNA	NM_019026	Hs.93832	CR614153
SPINT2	-4.1	4.5	serine peptidase inhibitor, Kunitz type, 2 (SPINT2), mRNA	NM_021102	Hs.31439	AK127479
CR605947	-4.1	2.3	full-length cDNA clone CS0DD004YC02 of Neuroblastoma Cot 50-normalized of (human).	CR605947	Hs.663861	BX395274
HSP90AA1	-4.1	1.9	heat shock protein 90kDa alpha (cytosolic), class A member 1 (HSP90AA1), transcript variant 2, mRNA	NM_005348	Hs.525600	NM_001017963
ZMAT1	-4.1	3.8	zinc finger, matrin type 1 (ZMAT1), transcript variant 3, mRNA	NM_032441	Hs.496512	NM_032441
KIAA1913	-4.1	3.2	KIAA1913, mRNA (cDNA clone MGC:50847 IMAGE:5760073), complete cds.	BC044246	Hs.591341	NM_052913
KIF27	-4.1	1.9	kinesin family member 27 (KIF27), mRNA	NM_017576	Hs.697514	AY237536
THC2520858	-4.1	2.4	ALU6_HUMAN (P39193) Alu subfamily SP sequence contamination warning entry, partial (12%)	THC2520858	Unknown	
BE786351	-4.1	3.0	BE786351 601474522F1 NIH_MGC_68 cDNA clone IMAGE:3877396 5', mRNA sequence	BE786351	Hs.351306	NM_004696
C10orf118	-4.1	2.6	chromosome 10 open reading frame 118 (C10orf118), mRNA	NM_018017	Hs.159066	NM_018017
A_24_P919283	-4.1	3.7	A_24_P919283	A_24_P919283	Unknown	
LOC221362	-4.1	3.3	cDNA FLJ38040 fis, clone CTONG2013985.	AK095359	Hs.7921	AK091117
AK021614	-4.1	3.5	cDNA FLJ11552 fis, clone HEMBA1003021.	AK021614	Hs.635391	AK021614

HSP90AA1	-4.1	1.9	heat shock protein 90kDa alpha (cytosolic), class A member 1 (HSP90AA1), transcript variant 2, mRNA	NM_005348	Hs.525600	NM_001017963
PRG2	-4.1	18.3	plasticity-related gene 2 (PRG2), mRNA	NM_024888	Hs.546439	AF357888
KCNK10	-4.1	24.5	potassium channel, subfamily K, member 10 (KCNK10), transcript variant 1, mRNA	NM_021161	Hs.592299	AF279890
SPTLC3	-4.1	6.6	serine palmitoyltransferase, long chain base subunit 3 (SPTLC3), mRNA	NM_018327	Hs.425023	NM_018327
JARID1B	-4.1	3.0	jumonji, AT rich interactive domain 1B (JARID1B), mRNA	NM_006618	Hs.443650	AJ243706
UBL3	-4.1	1.7	ubiquitin-like 3 (UBL3), mRNA	NM_007106	Hs.145575	BC044582
AK024516	-4.1	2.4	cDNA: FLJ20863 fis, clone ADKA01804.	AK024516	Hs.657178	AK024516
AK022390	-4.1	8.1	cDNA FLJ12328 fis, clone MAMMA1002145.	AK022390	Hs.656537	AK022390
TNC	-4.1	4.2	tenascin C (hexabrachion) (TNC), mRNA	NM_002160	Hs.143250	NM_002160
BC035106	-4.1	2.5	cDNA clone IMAGE:5262677.	BC035106	Hs.594844	BC035106
LRRC51	-4.1	1.8	leucine rich repeat containing 51 (LRRC51), mRNA	NM_145309	Hs.317243	AF289588
SETDB1	-4.1	2.1	SET domain, bifurcated 1 (SETDB1), mRNA	NM_012432	Hs.700587	NM_012432
KIAA1683	-4.1	8.3	KIAA1683 (KIAA1683), mRNA	NM_025249	Hs.313471	AB051470
C1orf168	-4.1	95.7	chromosome 1 open reading frame 168 (C1orf168), mRNA	NM_001004303	Hs.437655	BX648439
FBXO9	-4.1	2.3	F-box protein 9 (FBXO9), transcript variant 2, mRNA	NM_033480	Hs.216653	NM_033480
AF116677	-4.1	5.8	PRO1966 mRNA, complete cds.	AF116677	Unknown	
BAALC	-4.1	8.4	brain and acute leukemia, cytoplasmic (BAALC), transcript variant 1, mRNA	NM_024812	Hs.533446	NM_024812
LOC401588	-4.1	1.6	full-length cDNA clone CS0DI087YI13 of Placenta Cot 25-normalized of (human).	CR619110	Hs.129955	NM_019886
PCP4	-4.1	10.5	Purkinje cell protein 4 (PCP4), mRNA	NM_006198	Hs.80296	BI114436
AA541413	-4.1	7.9	AA541413 ni77g08.s1 NCI_CGAP_Pr12 cDNA clone IMAGE:982910, mRNA sequence	AA541413	Hs.663518	AA541413
RPL5	-4.1	1.8	ribosomal protein L5 (RPL5), mRNA	NM_000969	Hs.532359	BM903678
TYR	-4.1	13.7	tyrosinase (oculocutaneous albinism IA) (TYR), mRNA	NM_000372	Hs.503555	M27160
A_32_P25809	-4.1	1.6	A_32_P25809	A_32_P25809	Unknown	
PCNXL2	-4.1	3.6	pecanex-like 2 (Drosophila) (PCNXL2), transcript variant 1, mRNA	NM_014801	Hs.370605	NM_014801
CROP	-4.1	2.6	cisplatin resistance-associated overexpressed protein (CROP), transcript variant 1, mRNA	NM_016424	Hs.130293	NM_016424
AF052152	-4.1	5.6	clone 24628 mRNA sequence.	AF052152	Hs.593611	AF052152
RB1	-4.1	1.8	retinoblastoma 1 (including osteosarcoma) (RB1), mRNA	NM_000321	Hs.408528	L41870
N4BP2	-4.1	3.0	Nedd4 binding protein 2 (N4BP2), mRNA	NM_018177	Hs.396494	AY267013
MXRA8	-4.0	4.0	matrix-remodelling associated 8 (MXRA8), mRNA	NM_032348	Hs.558570	AK095966
PTBP2	-4.0	3.2	polypyrimidine tract binding protein 2 (PTBP2), mRNA	NM_021190	Hs.591430	AB209266
ROPN1L	-4.0	7.4	ropporin 1-like (ROPN1L), mRNA	NM_031916	Hs.381089	BM563234
PCDHA1	-4.0	3.3	protocadherin alpha 1 (PCDHA1), transcript variant 2, mRNA	NM_031410	Hs.199343	NM_014005
PROC	-4.0	6.9	protein C (inactivator of coagulation factors Va and VIIIa) (PROC), mRNA	NM_000312	Hs.224698	BC034377
NMU	-4.0	14.2	neuromedin U (NMU), mRNA	NM_006681	Hs.418367	BF034907
UACA	-4.0	2.7	uveal autoantigen with coiled-coil domains and ankyrin repeats (UACA), transcript variant 2, mRNA	NM_001008224	Hs.108049	NM_001008224
AY358106	-4.0	3.1	clone DNA119534 LCII3035 (UNQ3035) mRNA, complete cds.	AY358106	Hs.626045	AY358106
PLA2G1B	-4.0	3.1	phospholipase A2, group IB (pancreas) (PLA2G1B), mRNA	NM_000928	Hs.992	BE969664
KIAA0408	-4.0	10.2	KIAA0408 (KIAA0408), mRNA	NM_014702	Hs.319247	NM_014702
ATXN7L2	-4.0	2.0	ataxin 7-like 2 (ATXN7L2), mRNA	NM_153340	Hs.118248	BC036849
LOC728449	-4.0	3.5	Annexin A8 (Annexin VIII) (Vascular anticoagulant-beta) (VAC-beta).	ENST00000335083	Unknown	
SUZ12	-4.0	2.2	suppressor of zeste 12 homolog (Drosophila) (SUZ12), mRNA	NM_015355	Hs.462732	D63881
THC2614991	-4.0	11.8	THC2614991	THC2614991	Unknown	
C2orf15	-4.0	12.9	chromosome 2 open reading frame 15 (C2orf15), mRNA	NM_144706	Hs.352211	BC021264
ZBTB41	-4.0	3.3	zinc finger and BTB domain containing 41 (ZBTB41), mRNA	NM_194314	Hs.529439	NM_194314
BC089156	-4.0	2.1	cDNA clone IMAGE:6576427.	BC089156	Hs.30567	BC020163
HABP2	-4.0	2.9	hyaluronan binding protein 2 (HABP2), mRNA	NM_004132	Hs.422542	NM_004132
C1orf125	-4.0	5.8	chromosome 1 open reading frame 125 (C1orf125), transcript variant 1, mRNA	NM_144696	Hs.658505	BX647935
ENST000003693	-4.0	2.3	Notch homolog 2 N-terminal like protein	ENST00000369334	Unknown	
TLE4	-4.0	3.4	transducin-like enhancer of split 4 (E(sp1) homolog, Drosophila) (TLE4), mRNA	NM_007005	Hs.444213	CR749553
HIPK1	-4.0	2.2	homeodomain interacting protein kinase 1 (HIPK1), transcript variant 1, mRNA	NM_198268	Hs.532363	NM_198268
HS3ST3B1	-4.0	6.0	Heparan sulfate glucosamine 3-O-sulfotransferase 3B1 (EC 2.8.2.30) (Heparan sulfate D-glucosaminyl 3-O-sulfotransferase 3B1) (Heparan sulfate 3-O-sulfotransferase 3B1) (h3-OST-3B).	ENST00000360954	Unknown	
THC2634493	-4.0	5.9	Q76JW3_9NEOP (Q76JW3) NADH dehydrogenase subunit 5 (Fragment), partial (7%)	THC2634493	Unknown	

THC2641484	-4.0	7.6	THC2641484	THC2641484	Unknown	
FHAD1	-4.0	3.9	cDNA FLJ36564 fis, clone TRACH2009851.	AK093883	Hs.659997	XM_934889
HSP90AA1	-4.0	1.9	heat shock protein 90kDa alpha (cytosolic), class A member 1 (HSP90AA1), transcript variant 2, mRNA	NM_005348	Hs.525600	NM_001017963
GSTM1	-4.0	2.0	glutathione S-transferase M1 (GSTM1), transcript variant 2, mRNA	NM_146421	Hs.301961	BQ880398
ARHGAP5	-4.0	2.5	Rho GTPase activating protein 5 (ARHGAP5), transcript variant 1, mRNA	NM_001030055	Hs.592313	NM_001030055
C8orf13	-4.0	11.4	chromosome 8 open reading frame 13 (C8orf13), mRNA	NM_053279	Hs.124299	NM_053279
ZCCHC14	-4.0	2.2	zinc finger, CCHC domain containing 14 (ZCCHC14), mRNA	NM_015144	Hs.156231	NM_015144
AK090897	-4.0	2.5	cDNA FLJ33578 fis, clone BRAMY2011639.	AK090897	Hs.420757	BC063474
IGF1	-4.0	3.7	insulin-like growth factor 1 (somatomedin C) (IGF1), mRNA	NM_000618	Hs.160562	NM_000618
SLC1A3	-4.0	3.8	solute carrier family 1 (glial high affinity glutamate transporter), member 3 (SLC1A3), mRNA	NM_004172	Hs.481918	NM_004172
FABP1	-4.0	53.6	fatty acid binding protein 1, liver (FABP1), mRNA	NM_001443	Hs.380135	BX641099
CTNNA2	-4.0	6.1	catenin (cadherin-associated protein), alpha 2 (CTNNA2), mRNA	NM_004389	Hs.167368	AK127226
ZNF529	-4.0	2.2	zinc finger protein 529 (ZNF529), mRNA	NM_020951	Hs.700695	AK025110
LOC644246	-4.0	2.0	hypothetical protein LOC644246, mRNA (cDNA clone IMAGE:4730995), partial cds.	BC020847	Hs.644600	BG620930
PLAG1	-4.0	2.4	pleiomorphic adenoma gene 1 (PLAG1), mRNA	NM_002655	Hs.14968	NM_002655
PITPNC1	-4.0	3.9	cDNA FLJ37405 fis, clone BRAMY2028269.	AK094724	Hs.696160	AK094724
FLJ37453	-4.0	1.8	cDNA FLJ37453 fis, clone BRAWH2010754.	AK094772	Hs.119298	AK124018
ESCO1	-4.0	2.6	establishment of cohesion 1 homolog 1 (S. cerevisiae) (ESCO1), mRNA	NM_052911	Hs.464733	AL832041
PHIP	-4.0	1.9	pleckstrin homology domain interacting protein (PHIP), mRNA	NM_017934	Hs.511817	NM_017934
BQ189494	-4.0	6.1	BQ189494 UI-E-EJ1-aka-g-05-0-UI.r1 UI-E-EJ1 cDNA clone UI-E-EJ1-aka-g-05-0-UI 5', mRNA sequence	BQ189494	Hs.664796	BQ189494
THC2771474	-4.0	2.0	Q8NFF8_HUMAN (Q8NFF8) MLL5, partial (3%)	THC2771474	Unknown	
BMF	-4.0	4.1	Bcl2 modifying factor (BMF), transcript variant 1, mRNA	NM_001003940	Hs.591104	NM_001003940
KIAA1407	-4.0	2.8	KIAA1407 (KIAA1407), mRNA	NM_020817	Hs.477159	AF509494
A_32_P40673	-4.0	6.7	A_32_P40673	A_32_P40673	Unknown	
LOC727891	-4.0	1.7	PREDICTED: similar to Heat shock protein HSP 90-alpha (HSP 86) (NY-REN-38 antigen) (LOC727891), mRNA	XR_015156	Hs.631939	BQ423056
LYSMD3	-4.0	1.9	LysM, putative peptidoglycan-binding, domain containing 3 (LYSMD3), mRNA	NM_198273	Hs.136235	BX537972
LOC654433	-4.0	2.0	cDNA clone IMAGE:5266296.	BC036699	Hs.656660	AK126431
CCDC6	-4.0	1.9	coiled-coil domain containing 6 (CCDC6), mRNA	NM_005436	Hs.591360	NM_005436
RBAK	-4.0	2.4	RB-associated KRAB zinc finger (RBAK), mRNA	NM_021163	Hs.592827	NM_021163
RUFY3	-4.0	6.4	RUN and FYVE domain containing 3 (RUFY3), transcript variant 2, mRNA	NM_014961	Hs.7972	BC051716
LOC439911	-4.0	2.7	cDNA FLJ45542 fis, clone BRTHA2033320.	AK127450	Unknown	
PREPL	-4.0	2.4	prolyl endopeptidase-like (PREPL), transcript variant C, mRNA	NM_006036	Hs.444349	BC151236
AK074776	-4.0	2.0	cDNA FLJ90295 fis, clone NT2RP2000240.	AK074776	Hs.438950	AK074776
AF035290	-4.0	23.8	clone 23556 mRNA sequence.	AF035290	Hs.656663	AF035290
CACNA1B	-4.0	9.8	Voltage-dependent N-type calcium channel subunit alpha-1B (Voltage- gated calcium channel subunit alpha Cav2.2) (Calcium channel, L type, alpha-1 polypeptide isoform 5) (Brain calcium channel III) (BIII), ...	ENST00000371372	Unknown	
ATAD3B	-4.0	3.5	mRNA for KIAA1273 protein, partial cds.	AB033099	Hs.23413	AB033099
PLCB1	-4.0	1.8	phospholipase C, beta 1 (phosphoinositide-specific) (PLCB1), transcript variant 2, mRNA	NM_182734	Hs.431173	AB011153
RANBP2	-3.9	2.0	RAN binding protein 2 (RANBP2), mRNA	NM_006267	Hs.590897	NM_006267
LOC151438	-3.9	4.1	cDNA FLJ31315 fis, clone LIVER1000303.	AK055877	Hs.516245	AK055877
C12orf22	-3.9	1.7	chromosome 12 open reading frame 22 (C12orf22), mRNA	NM_030809	Hs.524425	AJ298133
A_32_P306001	-3.9	3.0	A_32_P306001	A_32_P306001	Unknown	
THC2585464	-3.9	2.2	Q86UT8_HUMAN (Q86UT8) DLNB14 protein, partial (7%)	THC2585464	Unknown	
LMO1	-3.9	2.9	LIM domain only 1 (rhombotin 1) (LMO1), mRNA	NM_002315	Hs.654426	M26682
LRP1B	-3.9	39.2	low density lipoprotein-related protein 1B (deleted in tumors) (LRP1B), mRNA	NM_018557	Hs.656461	NM_018557
KITLG	-3.9	3.4	KIT ligand (KITLG), transcript variant b, mRNA	NM_000899	Hs.1048	NM_000899
AF075063	-3.9	2.3	full length insert cDNA YP99D02.	AF075063	Hs.406337	BM983749
LEAP2	-3.9	2.6	liver expressed antimicrobial peptide 2 (LEAP2), mRNA	NM_052971	Hs.337588	AJ409065
THC2700191	-3.9	6.4	O41021_PBCV1 (O41021) A312aR protein, partial (26%)	THC2700191	Unknown	
ZNF84	-3.9	2.7	zinc finger protein 84 (ZNF84), mRNA	NM_003428	Hs.654730	BC036656
ENST00000333	-3.9	16.2	Jak and microtubule interacting protein 2	ENST00000333010	Unknown	
TLX2	-3.9	5.3	T-cell leukemia homeobox 2 (TLX2), mRNA	NM_016170	Hs.168586	BC006396
DDC	-3.9	31.6	dopa decarboxylase (aromatic L-amino acid decarboxylase) (DDC), mRNA	NM_000790	Hs.359698	NM_001082971

TMCC1	-3.9	2.1	transmembrane and coiled-coil domain family 1 (TMCC1), transcript variant 1, mRNA	NM_001017395	Hs.477547	NM_001017395
FLJ44060	-3.9	3.1	FLJ44060 protein (FLJ44060), mRNA	NM_207366	Hs.453629	NM_207366
BC039457	-3.9	1.9	cDNA clone IMAGE:5312122.	BC039457	Hs.406492	AK001640
AASDHPPT	-3.9	2.0	aminoadipate-semialdehyde dehydrogenase-phosphopantetheinyl transferase (AASDHPPT), mRNA	NM_015423	Hs.524009	BX537665
KIAA1598	-3.9	3.3	KIAA1598 (KIAA1598), mRNA	NM_018330	Hs.501140	AK091578
CR593845	-3.9	2.0	full-length cDNA clone CS0DF024YI14 of Fetal brain of (human).	CR593845	Hs.670258	CR620748
THC2557016	-3.9	2.3	Q3F710_9BURK (Q3F710) Inner-membrane translocator, partial (6%)	THC2557016	Unknown	
ZNF711	-3.9	5.6	zinc finger protein 711 (ZNF711), mRNA	NM_021998	Hs.326801	BX648117
IGSF4	-3.9	3.9	immunoglobulin superfamily, member 4 (IGSF4), mRNA	NM_014333	Hs.370510	BX641042
SERPINA1	-3.9	12.1	serpin peptidase inhibitor, clade A (alpha-1 antiproteinase, antitrypsin), member 1 (SERPINA1), transcript variant 2, mRNA	NM_001002236	Hs.525557	CR594071
THC2572108	-3.9	2.5	Q495B5_HUMAN (Q495B5) C15orf21 protein, partial (75%)	THC2572108	Unknown	
MAEL	-3.9	3.8	maelstrom homolog (Drosophila) (MAEL), mRNA	NM_032858	Hs.651245	BC034310
ADCY8	-3.9	6.1	adenylate cyclase 8 (brain) (ADCY8), mRNA	NM_001115	Hs.591859	NM_001115
BCAS2	-3.9	2.4	breast carcinoma amplified sequence 2 (BCAS2), mRNA	NM_005872	Hs.22960	BC022880
ZNF702	-3.9	2.5	cDNA FLJ12985 fis, clone NT2RP3000050, moderately similar to ZINC FINGER PROTEIN 91.	AK023047	Hs.270435	BC032590
FAM110C	-3.9	6.2	full-length cDNA clone CS0DJ001YJ05 of T cells (Jurkat cell line) Cot 10-normalized of (human).	CR601458	Hs.8379	CR601458
BC070363	-3.9	2.3	cDNA clone IMAGE:3960708, partial cds.	BC070363	Hs.465405	BI517643
SLC19A2	-3.9	3.6	solute carrier family 19 (thiamine transporter), member 2 (SLC19A2), mRNA	NM_006996	Hs.30246	AJ237724
ONECUT2	-3.9	17.9	one cut domain, family member 2 (ONECUT2), mRNA	NM_004852	Hs.194725	NM_004852
AK056630	-3.9	1.7	cDNA FLJ32068 fis, clone OCBBF1000114.	AK056630	Hs.694880	AK056630
KIF1B	-3.9	3.1	kinesin family member 1B (KIF1B), transcript variant 1, mRNA	NM_015074	Hs.97858	NM_015074
THC2654929	-3.9	3.2	THC2654929	THC2654929	Unknown	
HIST1H2AG	-3.9	4.1	histone cluster 1, H2ag (HIST1H2AG), mRNA	NM_021064	Hs.51011	BC067782
TMEM32	-3.9	2.3	transmembrane protein 32 (TMEM32), mRNA	NM_173470	Hs.110702	NM_173470
CR625594	-3.9	3.4	full-length cDNA clone CS0DF026YM07 of Fetal brain of (human).	CR625594	Hs.125867	AL133642
BRD8	-3.9	1.8	bromodomain containing 8 (BRD8), transcript variant 1, mRNA	NM_006696	Hs.519337	NM_139199
BF446608	-3.9	1.9	7q89f08.x1 NCI_CGAP_Lu24 cDNA clone IMAGE:3705734 3', mRNA sequence	BF446608	Hs.701411	BF446608
ASGR2	-3.9	9.0	asialoglycoprotein receptor 2 (ASGR2), transcript variant H2', mRNA	NM_080912	Hs.654440	NM_080912
SETBP1	-3.9	4.9	SET binding protein 1 (SETBP1), mRNA	NM_015559	Hs.435458	BC146776
B3GALT6	-3.9	1.7	UDP-Gal:betaGal beta 1,3-galactosyltransferase polypeptide 6 (B3GALT6), mRNA	NM_080605	Hs.284284	NM_080605
NBPF10	-3.9	2.5	neuroblastoma breakpoint family, member 10 (NBPF10), mRNA	NM_001039703	Hs.515947	BX538005
DNALI1	-3.9	5.9	dynein, axonemal, light intermediate chain 1 (DNALI1), mRNA	NM_003462	Hs.406050	BC039074
EMID1	-3.9	4.1	EMI domain containing 1 (EMID1), mRNA	NM_133455	Hs.289106	BC046358
KIAA1731	-3.9	2.5	mRNA for KIAA1731 protein, partial cds.	AB051518	Hs.458418	AB051518
LPAL2	-3.9	3.5	cDNA FLJ43922 fis, clone TESTI4012406, weakly similar to Apolipoprotein(A) (EC 3.4.21.-).	AK125910	Unknown	
THC2507925	-3.9	2.1	ALU7_HUMAN (P39194) Alu subfamily SQ sequence contamination warning entry, partial (29%)	THC2507925	Unknown	
ENST0000037395	-3.9	2.2	Protein FAM76A.	ENST00000373954	Unknown	
FOLR1	-3.9	15.9	folate receptor 1 (adult) (FOLR1), transcript variant 1, mRNA	NM_016725	Hs.73769	NM_016730
AK098579	-3.9	2.7	cDNA FLJ25713 fis, clone TST05089.	AK098579	Hs.649561	AK098579
C3orf50	-3.9	5.8	cDNA clone IMAGE:4156795.	BC011266	Hs.478158	BC011266
INADL	-3.9	4.3	InaD-like (Drosophila) (INADL), mRNA	NM_176877	Hs.478125	NM_176877
NPAS3	-3.9	18.5	neuronal PAS domain protein 3 (NPAS3), transcript variant 1, mRNA	NM_022123	Hs.659456	AF164438
GABRA5	-3.9	5.2	gamma-aminobutyric acid (GABA) A receptor, alpha 5 (GABRA5), mRNA	NM_000810	Hs.612087	NM_000810
ZFP37	-3.9	5.4	zinc finger protein 37 homolog (mouse) (ZFP37), mRNA	NM_003408	Hs.150406	AF022158
POLQ	-3.9	3.5	polymerase (DNA directed), theta (POLQ), mRNA	NM_199420	Hs.241517	AY032677
USH1C	-3.9	2.1	Usher syndrome 1C (autosomal recessive, severe) (USH1C), transcript variant 1, mRNA	NM_005709	Hs.502072	NM_153676
BC030084	-3.9	2.0	cDNA clone IMAGE:4791887.	BC030084	Hs.167087	BC048014
KIAA1529	-3.9	4.0	KIAA1529 (KIAA1529), mRNA	NM_020893	Hs.435629	CR627453
DKFZp667E0512	-3.9	2.3	mRNA; cDNA DKFZp667E0512 (from clone DKFZp667E0512).	AL713660	Hs.618463	AL713660
AK127572	-3.9	3.0	cDNA FLJ45665 fis, clone CTONG2027959.	AK127572	Hs.596577	AK127572
RP11-564C4.1	-3.9	10.2	mRNA; cDNA DKFZp779G2222 (from clone DKFZp779G2222).	CR749831	Hs.282795	CR749831
GSTM1	-3.9	2.0	glutathione S-transferase M1 (GSTM1), transcript variant 2, mRNA	NM_146421	Hs.301961	BQ880398

NBPF11	-3.9	2.5	neuroblastoma breakpoint family, member 11 (NBPF11), mRNA	NM_183372	Hs.636561	BX648497
PDE11A	-3.9	27.2	phosphodiesterase 11A (PDE11A), transcript variant 4, mRNA	NM_016953	Hs.570273	NM_016953
ENST00000355(	-3.9	22.3	protein expressed in prostate, ovary, testis, and placenta 14	ENST00000355077	Unknown	
KARCA1	-3.9	3.7	kelch/ankyrin repeat containing cyclin A1 interacting protein (KARCA1), transcript variant 2, mRNA	NM_001007255	Hs.507290	BC032482
AK093729	-3.9	2.5	cDNA FLJ36410 fis, clone THYMU2010637.	AK093729	Hs.465593	AK096776
SLC9A6	-3.9	3.9	solute carrier family 9 (sodium/hydrogen exchanger), member 6 (SLC9A6), transcript variant 1, mRNA	NM_001042537	Hs.62185	NM_001042537
TRIM2	-3.9	3.3	tripartite motif-containing 2 (TRIM2), mRNA	NM_015271	Hs.435711	AF220018
THC2605327	-3.9	42.5	AB013803 hNB-2s {Homo sapiens} (exp=-1; wgp=0; cg=0), partial (3%)	THC2605327	Unknown	
GOLGB1	-3.9	2.3	golgi autoantigen, golgin subfamily b, macrogolgin (with transmembrane signal), 1 (GOLGB1), mRNA	NM_004487	Hs.213389	NM_004487
BC032042	-3.9	3.4	cDNA clone IMAGE:4819488.	BC032042	Hs.666451	BQ940799
PLXDC2	-3.9	4.4	plexin domain containing 2 (PLXDC2), mRNA	NM_032812	Hs.658134	AY358486
IQCA	-3.9	3.6	IQ motif containing with AAA domain (IQCA), mRNA	NM_024726	Hs.591594	BC028699
ENST00000320(	-3.9	2.2	CDNA: FLJ22509 fis, clone HRC11803.	ENST00000320547	Unknown	
NSBP1	-3.9	4.0	nucleosomal binding protein 1 (NSBP1), mRNA	NM_030763	Hs.282204	AF250329
C18orf1	-3.9	3.2	chromosome 18 open reading frame 1 (C18orf1), transcript variant a2, mRNA	NM_181482	Hs.149363	NM_001003674
LOC442319	-3.9	2.4	PREDICTED: similar to zinc finger protein 92 (HTF12) isoform 2 (LOC442319), mRNA	XR_016680	Hs.640774	XR_016680
CR620977	-3.9	6.6	full-length cDNA clone CS0CAP004YK15 of Thymus of (human).	CR620977	Hs.377961	CR620977
FANCL	-3.9	2.7	Fanconi anemia, complementation group L (FANCL), mRNA	NM_018062	Hs.699478	BC037570
LOC127602	-3.9	4.2	PREDICTED: hypothetical protein LOC127602 (LOC127602), mRNA	XM_059166	Hs.655805	XM_059166
LOC285141	-3.9	10.9	full-length cDNA clone CS0DI052YH08 of Placenta Cot 25-normalized of (human).	ENST00000375280	Unknown	
THC2682291	-3.9	31.0	THC2682291	THC2682291	Unknown	
SERPINC1	-3.9	16.8	serpin peptidase inhibitor, clade C (antithrombin), member 1 (SERPINC1), mRNA	NM_000488	Hs.75599	AF130100
CP	-3.9	17.1	ceruloplasmin (ferroxidase) (CP), mRNA	NM_000096	Hs.558314	BC146801
CITED4	-3.9	2.7	Cbp/p300-interacting transactivator, with Glu/Asp-rich carboxy-terminal domain, 4 (CITED4), mRNA	NM_133467	Hs.355820	BM909126
THC2665223	-3.9	6.1	THC2665223	THC2665223	Unknown	
RTDR1	-3.9	2.7	rhabdoid tumor deletion region gene 1 (RTDR1), mRNA	NM_014433	Hs.526920	NM_014433
FAM57B	-3.9	4.1	family with sequence similarity 57, member B (FAM57B), mRNA	NM_031478	Hs.558560	NM_031478
THC2722365	-3.9	2.7	THC2722365	THC2722365	Unknown	
C14orf72	-3.9	2.8	PREDICTED: chromosome 14 open reading frame 72 (C14orf72), mRNA	XM_096733	Hs.150840	XM_096733
SLITRK1	-3.9	8.4	SLIT and NTRK-like family, member 1 (SLITRK1), mRNA	NM_052910	Hs.415478	NM_052910
APOC2	-3.9	4.3	apolipoprotein C-II (APOC2), mRNA	NM_000483	Hs.75615	BG568052
PTPRT	-3.8	8.7	protein tyrosine phosphatase, receptor type, T (PTPRT), transcript variant 1, mRNA	NM_133170	Hs.526879	NM_133170
HS3ST4	-3.8	157.8	heparan sulfate (glucosamine) 3-O-sulfotransferase 4 (HS3ST4), mRNA	NM_006040	Hs.655275	NM_006040
CAPN6	-3.8	27.5	calpain 6 (CAPN6), mRNA	NM_014289	Hs.496593	AJ000388
USP47	-3.8	2.2	ubiquitin specific peptidase 47 (USP47), mRNA	NM_017944	Hs.577256	NM_017944
AK093729	-3.8	2.4	cDNA FLJ36410 fis, clone THYMU2010637.	AK093729	Hs.465593	AK096776
MAST1	-3.8	5.6	microtubule associated serine/threonine kinase 1 (MAST1), mRNA	NM_014975	Hs.227489	AB023190
MLLT11	-3.8	2.1	myeloid/lymphoid or mixed-lineage leukemia (trithorax homolog, Drosophila); translocated to, 11 (MLLT11), mRNA	NM_006818	Hs.75823	BC022448
F2	-3.8	9.7	coagulation factor II (thrombin) (F2), mRNA	NM_000506	Hs.655207	BC051332
CR620892	-3.8	8.8	full-length cDNA clone CS0DL007YK20 of B cells (Ramos cell line) Cot 25-normalized of (human).	CR620892	Hs.584916	AK000271
APOH	-3.8	81.2	apolipoprotein H (beta-2-glycoprotein I) (APOH), mRNA	NM_000042	Hs.445358	NM_000042
RAP2C	-3.8	2.9	RAP2C, member of RAS oncogene family (RAP2C), mRNA	NM_021183	Hs.119889	AY298955
CSPG3	-3.8	3.3	chondroitin sulfate proteoglycan 3 (neurocan) (CSPG3), mRNA	NM_004386	Hs.169047	NM_004386
RIC3	-3.8	8.3	resistance to inhibitors of cholinesterase 3 homolog (C. elegans) (RIC3), mRNA	NM_024557	Hs.655660	AL832601
UNG2	-3.8	3.1	uracil-DNA glycosylase 2 (UNG2), mRNA	NM_021147	Hs.3041	BC004877
PKN2	-3.8	1.9	protein kinase N2 (PKN2), mRNA	NM_006256	Hs.440833	NM_006256
ZNF100	-3.8	2.6	zinc finger protein 100 (ZNF100), mRNA	NM_173531	Hs.365142	BC035579
CX3CL1	-3.8	2.2	chemokine (C-X3-C motif) ligand 1 (CX3CL1), mRNA	NM_002996	Hs.531668	AB209037
NSUN5C	-3.8	3.1	NOL1/NOP2/Sun domain family, member 5C (NSUN5C), transcript variant 1, mRNA	NM_032158	Hs.510927	AL117583
BC040293	-3.8	8.3	cDNA clone IMAGE:4820330.	BC040293	Hs.587484	BC040293
CAMSAP1L1	-3.8	2.5	calmodulin regulated spectrin-associated protein 1-like 1 (CAMSAP1L1), mRNA	NM_203459	Hs.23585	NM_203459

STXBP5L	-3.8	7.8	mRNA for KIAA1006 protein, partial cds.	AB023223	Hs.477315	NM_014980
FAM91A2	-3.8	3.5	cDNA clone IMAGE:4795401.	BC021732	Hs.645966	BC021732
RGPD5	-3.8	2.5	RANBP2-like and GRIP domain containing 5 (RGPD5), transcript variant 1, mRNA	NM_005054	Hs.469630	NM_005054
LOC440248	-3.8	2.6	hypothetical LOC440248 (LOC440248), mRNA	NM_199045	Unknown	
THC2727290	-3.8	2.1	T32824 EST54797 Human Brain cDNA 3' end similar to None., mRNA sequence	THC2727290	Unknown	
FAM43B	-3.8	3.8	family with sequence similarity 43, member B (FAM43B), mRNA	NM_207334	Hs.429179	AK126900
BC032332	-3.8	3.1	cDNA clone MGC:40288 IMAGE:5169056, complete cds.	BC032332	Hs.473317	AK001745
PITX2	-3.8	67.0	paired-like homeodomain transcription factor 2 (PITX2), transcript variant 2, mRNA	NM_153426	Hs.643588	AK127829
INADL	-3.8	3.6	InaD-like (Drosophila) (INADL), mRNA	NM_176877	Hs.478125	NM_176877
THC2646608	-3.8	1.7	ALU7_HUMAN (P39194) Alu subfamily SQ sequence contamination warning entry, partial (19%)	THC2646608	Unknown	
C2orf14	-3.8	5.1	mRNA; cDNA DKFZp434F1719 (from clone DKFZp434F1719).	AL136789	Hs.633271	XR_015755
FLJ35834	-3.8	6.3	hypothetical protein FLJ35834 (FLJ35834), mRNA	NM_178827	Hs.159650	AK127736
EFHC2	-3.8	5.0	EF-hand domain (C-terminal) containing 2 (EFHC2), mRNA	NM_025184	Hs.521953	NM_025184
A_32_P166653	-3.8	45.3	A_32_P166653	A_32_P166653	Unknown	
NUP62CL	-3.8	4.7	nucleoporin 62kDa C-terminal like (NUP62CL), mRNA	NM_017681	Hs.163629	BC016327
AK129879	-3.8	1.8	cDNA FLJ26369 fis, clone HRT06001.	AK129879	Hs.656600	CR603940
AGRIN	-3.8	2.2	agrin (AGRIN), mRNA	NM_198576	Hs.273330	AB191264
AK096104	-3.8	4.7	cDNA FLJ38785 fis, clone LIVER2001329.	AK096104	Hs.406990	AK096104
ENST00000317	-3.8	6.3	l(3)mbt-like 4	ENST00000317931	Unknown	
MSH2	-3.8	3.1	mutS homolog 2, colon cancer, nonpolyposis type 1 (E. coli) (MSH2), mRNA	NM_000251	Hs.597656	AK223284
THC2651501	-3.8	4.9	THC2651501	THC2651501	Unknown	
PNPLA3	-3.8	3.6	patatin-like phospholipase domain containing 3 (PNPLA3), mRNA	NM_025225	Hs.654800	AL138578
BPTF	-3.8	2.2	bromodomain PHD finger transcription factor (BPTF), transcript variant 1, mRNA	NM_182641	Hs.444200	NM_182641
CRMP1	-3.8	6.8	collapsin response mediator protein 1 (CRMP1), transcript variant 1, mRNA	NM_001014809	Hs.135270	NM_001014809
ZIC3	-3.8	309.3	Zic family member 3 heterotaxy 1 (odd-paired homolog, Drosophila) (ZIC3), mRNA	NM_003413	Hs.111227	NM_003413
ZNF684	-3.8	2.1	zinc finger protein 684 (ZNF684), mRNA	NM_152373	Hs.524767	AK094072
AK056119	-3.8	2.8	cDNA FLJ31557 fis, clone NT2RI2001307.	AK056119	Hs.665456	AK056119
AK130118	-3.8	5.4	cDNA FLJ26608 fis, clone LVR00914.	AK130118	Hs.654777	AK130118
DCAKD	-3.8	2.1	cDNA FLJ12161 fis, clone MAMMA1000576.	AK022223	Hs.656393	AK022223
CD641036	-3.8	1.7	CD641036 AGENCOURT_14532748 NIH_MGC_191 cDNA clone IMAGE:30415087 5', mRNA sequence	CD641036	Hs.538093	BC021004
NOTCH2NL	-3.8	1.9	cDNA FLJ90584 fis, clone PLACE1000740, highly similar to Neurogenic locus notch homolog protein 2 precursor.	AK075065	Hs.515947	BX538005
A_32_P71016	-3.8	5.5	A_32_P71016	A_32_P71016	Unknown	
KIAA1576	-3.8	6.0	KIAA1576 protein (KIAA1576), mRNA	NM_020927	Hs.461405	BC033537
TRIM36	-3.8	3.2	tripartite motif-containing 36 (TRIM36), transcript variant 1, mRNA	NM_018700	Hs.519514	NM_018700
SCN3A	-3.8	4.3	sodium channel, voltage-gated, type III, alpha (SCN3A), mRNA	NM_006922	Hs.435274	NM_006922
THC2540396	-3.8	2.0	ALU8_HUMAN (P39195) Alu subfamily SX sequence contamination warning entry, partial (23%)	THC2540396	Unknown	
NSBP1	-3.8	4.2	nucleosomal binding protein 1 (NSBP1), mRNA	NM_030763	Hs.282204	AF250329
STON1	-3.8	2.6	stonin 1 (STON1), mRNA	NM_006873	Hs.44385	NM_006873
EPC2	-3.8	2.3	enhancer of polycomb homolog 2 (Drosophila) (EPC2), mRNA	NM_015630	Hs.23270	AK125789
LOC388494	-3.8	4.4	full-length cDNA clone CS0DF014YD20 of Fetal brain of (human).	CR593500	Hs.65750	NM_015174
LOC440353	-3.8	2.2	nuclear pore complex interacting protein pseudogene (LOC440353) on chromosome 16	NR_002603	Unknown	
HMGN2	-3.8	2.7	high-mobility group nucleosomal binding domain 2 (HMGN2), mRNA	NM_005517	Hs.181163	BC110390
LOC349114	-3.8	2.6	hypothetical protein LOC349114, mRNA (cDNA clone IMAGE:6379934), partial cds.	BC053841	Hs.414183	BC053841
AK092468	-3.8	3.7	cDNA FLJ35149 fis, clone PLACE6010485.	AK092468	Hs.696747	AK092468
ZNF655	-3.8	3.2	zinc finger protein 655 (ZNF655), transcript variant 6, mRNA	NM_001009956	Unknown	
AK057923	-3.8	2.6	cDNA FLJ25194 fis, clone REC04095.	AK057923	Hs.660881	AL832788
ATRX	-3.8	2.6	alpha thalassemia/mental retardation syndrome X-linked (RAD54 homolog, S. cerevisiae) (ATRX), transcript variant 3, mRNA	NM_138271	Unknown	
DB153264	-3.8	2.2	DB153264 THYMU3 cDNA clone THYMU3030831 5', mRNA sequence	DB153264	Hs.374251	DB153264
MYH2	-3.8	6.3	myosin, heavy chain 2, skeletal muscle, adult (MYH2), mRNA	NM_017534	Hs.440895	NM_017534
NBPF3	-3.8	2.3	neuroblastoma breakpoint family, member 3 (NBPF3), mRNA	NM_032264	Hs.325422	AK095602
PTPLAD1	-3.8	2.0	protein tyrosine phosphatase-like A domain containing 1 (PTPLAD1), mRNA	NM_016395	Hs.512973	AK074898

ATP6V1C2	-3.8	3.2	ATPase, H+ transporting, lysosomal 42kDa, V1 subunit C2 (ATP6V1C2), transcript variant 1, mRNA	NM_001039362	Hs.580464	AK125449
KIAA1212	-3.8	1.9	KIAA1212 (KIAA1212), mRNA	NM_018084	Hs.292925	NM_018084
BCL11A	-3.8	4.1	B-cell CLL/lymphoma 11A (zinc finger protein) (BCL11A), transcript variant 1, mRNA	NM_022893	Hs.370549	NM_022893
CR617018	-3.8	2.3	full-length cDNA clone CS0DG001YH13 of B cells (Ramos cell line) of (human).	CR617018	Hs.434075	CR607989
THC2698311	-3.8	3.7	HUMKIP2 p57KIP2 {Homo sapiens} (exp=-1; wgp=0; cg=0), partial (13%)	THC2698311	Unknown	
GLIS3	-3.8	3.2	GLIS family zinc finger 3 (GLIS3), transcript variant 1, mRNA	NM_001042413	Hs.162125	NM_001042413
ARHGEF4	-3.8	13.4	Rho guanine nucleotide exchange factor (GEF) 4 (ARHGEF4), transcript variant 2, mRNA	NM_032995	Hs.469935	AK127124
MOSPD1	-3.8	2.7	motile sperm domain containing 1 (MOSPD1), mRNA	NM_019556	Hs.590789	BC005700
BC037827	-3.8	85.6	cDNA clone IMAGE:4811567.	BC037827	Hs.648649	BC037827
RCHY1	-3.8	1.6	ring finger and CHY zinc finger domain containing 1 (RCHY1), transcript variant 1, mRNA	NM_015436	Hs.48297	NM_015436
AFF2	-3.8	21.9	AF4/FMR2 family, member 2 (AFF2), mRNA	NM_002025	Hs.496911	NM_002025
ENST000003834	-3.8	2.5	ENST00000383490	ENST00000383490	Unknown	
ANGPTL3	-3.8	7.7	angiopoietin-like 3 (ANGPTL3), mRNA	NM_014495	Hs.209153	NM_014495
SH3BGRL	-3.8	1.9	SH3 domain binding glutamic acid-rich protein like (SH3BGRL), mRNA	NM_003022	Hs.108029	BC103762
HS6ST3	-3.8	4.3	heparan sulfate 6-O-sulfotransferase 3 (HS6ST3), mRNA	NM_153456	Hs.171001	NM_153456
C1orf121	-3.8	2.2	chromosome 1 open reading frame 121 (C1orf121), mRNA	NM_016076	Hs.498317	NM_016076
GALNTL1	-3.8	2.5	UDP-N-acetyl-alpha-D-galactosamine:polypeptide N-acetylgalactosaminyltransferase-like 1 (GALNTL1), mRNA	NM_020692	Hs.21035	BC036812
KIAA1432	-3.8	1.7	mRNA for KIAA1432 protein, partial cds.	AB037853	Hs.211520	AB037853
AK026209	-3.8	2.8	cDNA: FLJ22556 fis, clone HSI01326.	AK026209	Hs.513695	BX647358
ECEL1	-3.7	7.0	endothelin converting enzyme-like 1 (ECEL1), mRNA	NM_004826	Hs.26880	AB030579
USP48	-3.7	2.1	ubiquitin specific peptidase 48 (USP48), transcript variant 1, mRNA	NM_032236	Hs.467524	NM_032236
MSH2	-3.7	3.1	mutS homolog 2, colon cancer, nonpolyposis type 1 (E. coli) (MSH2), mRNA	NM_000251	Hs.597656	AK223284
UGT2B15	-3.7	6.8	UDP glucuronosyltransferase 2 family, polypeptide B15 (UGT2B15), mRNA	NM_001076	Hs.700565	XM_001127829
STOX1	-3.7	9.3	storkhead box 1 (STOX1), mRNA	NM_152709	Hs.37636	AY842014
LOC391040	-3.7	1.5	PREDICTED: similar to basic transcription factor 3-like 4 (LOC391040), mRNA	XR_019253	Hs.651019	XR_019253
SNIP	-3.7	14.4	SNAP25-interacting protein (SNIP), mRNA	NM_025248	Hs.448872	NM_025248
MCOLN3	-3.7	15.7	mucolipin 3 (MCOLN3), mRNA	NM_018298	Hs.535239	BC060765
CDKL2	-3.7	3.9	cyclin-dependent kinase-like 2 (CDC2-related kinase) (CDKL2), mRNA	NM_003948	Hs.591698	NM_003948
GCA	-3.7	2.3	grancalcin, EF-hand calcium binding protein (GCA), mRNA	NM_012198	Hs.377894	BC005214
ZNF680	-3.7	2.4	zinc finger protein 680 (ZNF680), mRNA	NM_178558	Hs.520886	BC030700
KIAA1706	-3.7	3.7	KIAA1706 protein (KIAA1706), mRNA	NM_030636	Hs.487994	NM_030636
DYNC2H1	-3.7	4.6	G protein interaction factor 1-like mRNA sequence.	AF288405	Hs.503721	NM_001080463
THC2518705	-3.7	4.0	THC2518705	THC2518705	Unknown	
ANGPT1	-3.7	5.4	angiopoietin 1 (ANGPT1), mRNA	NM_001146	Hs.369675	BX648814
GRAMD3	-3.7	3.6	GRAM domain containing 3 (GRAMD3), mRNA	NM_023927	Hs.363558	AK024966
RIMS2	-3.7	30.9	regulating synaptic membrane exocytosis 2 (RIMS2), mRNA	NM_014677	Hs.655271	NM_001100117
MAPK10	-3.7	6.3	mitogen-activated protein kinase 10 (MAPK10), transcript variant 3, mRNA	NM_138980	Hs.125503	AK124791
PLEKHA1	-3.7	1.9	pleckstrin homology domain containing, family A (phosphoinositide binding specific) member 1 (PLEKHA1), transcript variant 2, mRNA	NM_001001974	Hs.643512	BX647627
H3F3A	-3.7	2.5	H3 histone, family 3A (H3F3A), mRNA	NM_002107	Hs.546259	CR621187
CUTA	-3.7	2.4	cutA divalent cation tolerance homolog (E. coli) (CUTA), transcript variant 2, mRNA	NM_015921	Hs.520070	BM913222
SMYD3	-3.7	3.1	SET and MYND domain containing 3 (SMYD3), mRNA	NM_022743	Hs.567571	AK023594
FLJ31568	-3.7	7.1	FLJ31568 protein (FLJ31568), mRNA	NM_152509	Hs.386693	AK092530
C9orf18	-3.7	5.2	chromosome 9 open reading frame 18 (C9orf18), mRNA	NM_198469	Hs.71428	BX110216
GABRB3	-3.7	13.6	gamma-aminobutyric acid (GABA) A receptor, beta 3 (GABRB3), transcript variant 1, mRNA	NM_000814	Hs.302352	NM_000814
FLJ20054	-3.7	2.2	mRNA; cDNA DKFZp547O0715 (from clone DKFZp547O0715).	AL831839	Hs.657779	BC022561
NME5	-3.7	3.7	non-metastatic cells 5, protein expressed in (nucleoside-diphosphate kinase) (NME5), mRNA	NM_003551	Hs.519602	NM_003551
HMX1	-3.7	3.4	homeobox (H6 family) 1 (HMX1), mRNA	NM_018942	Hs.104134	NM_018942
THC2656479	-3.7	6.0	KTNA1_MOUSE (Q9WV86) Katanin p60 ATPase-containing subunit A1 (Katanin p60 subunit A1) (p60 katanin) (Lipotransin) , partial (29%)	THC2656479	Unknown	
THC2669157	-3.7	9.4	ALU1_HUMAN (P39188) Alu subfamily J sequence contamination warning entry, partial (10%)	THC2669157	Unknown	
C4orf26	-3.7	13.7	chromosome 4 open reading frame 26 (C4orf26), mRNA	NM_178497	Hs.24510	BC061701
PTPN3	-3.7	3.7	Tyrosine-protein phosphatase non-receptor type 3 (EC 3.1.3.48) (Protein-tyrosine phosphatase H1) (PTP-H1).	ENST00000374541	Unknown	

KIAA1212	-3.7	1.8	KIAA1212 (KIAA1212), mRNA	NM_018084	Hs.292925	NM_018084
STMN2	-3.7	35.9	stathmin-like 2 (STMN2), mRNA	NM_007029	Hs.521651	AK091336
KCNS3	-3.7	4.7	potassium voltage-gated channel, delayed-rectifier, subfamily S, member 3 (KCNS3), mRNA	NM_002252	Hs.414489	AK225833
AF339813	-3.7	10.2	clone IMAGE:297403, mRNA sequence.	AF339813	Hs.656512	AF339813
TSP50	-3.7	5.6	testes-specific protease 50 (TSP50), mRNA	NM_013270	Hs.120365	AK092598
THC2563387	-3.7	1.9	CD676691 ho04g02.y1 Human Trabecular meshwork cDNA: hohphq cDNA clone ho04g02 5', mRNA sequence	THC2563387	Unknown	
FAM20B	-3.7	1.7	family with sequence similarity 20, member B (FAM20B), mRNA	NM_014864	Hs.5737	AB007944
ZC3H11A	-3.7	2.4	zinc finger CCCH-type containing 11A (ZC3H11A), mRNA	NM_014827	Hs.532399	CR627439
HMGN2	-3.7	2.5	high-mobility group nucleosomal binding domain 2 (HMGN2), mRNA	NM_005517	Hs.181163	BC110390
NOS1AP	-3.7	2.8	nitric oxide synthase 1 (neuronal) adaptor protein (NOS1AP), mRNA	NM_014697	Hs.655000	AY841899
FLJ23049	-3.7	7.6	hypothetical protein FLJ23049 (FLJ23049), mRNA	NM_024687	Hs.478143	NM_024687
AK130071	-3.7	2.6	cDNA FLJ26561 fis, clone LNF03981.	AK130071	Hs.178499	BC040658
RFWD2	-3.7	1.7	ring finger and WD repeat domain 2 (RFWD2), transcript variant 1, mRNA	NM_022457	Hs.523744	NM_022457
LOC399947	-3.7	3.1	LOH11CR1A gene, loss of heterozygosity, 11, chromosomal region 1 gene A product.	AB096240	Hs.172982	AB096240
RGPD2	-3.7	2.2	RANBP2-like and GRIP domain containing 2 (RGPD2), mRNA	NM_001078170	Hs.656849	CR749330
SPAG17	-3.7	12.5	sperm associated antigen 17 (SPAG17), mRNA	NM_206996	Hs.528821	AY555274
SRP9	-3.7	2.3	signal recognition particle 9kDa (SRP9), mRNA	NM_003133	Hs.620844	BC064351
BC073935	-3.7	5.2	cDNA clone IMAGE:5219247, partial cds.	BC073935	Hs.434403	AK074886
RTN1	-3.7	11.9	reticulon 1 (RTN1), transcript variant 1, mRNA	NM_021136	Hs.368626	NM_021136
ZNF789	-3.7	2.9	cDNA FLJ16554 fis, clone SPLEN2016135.	AK131429	Hs.440384	BC031249
ASTN1	-3.7	4.0	astrotactin 1 (ASTN1), transcript variant 1, mRNA	NM_004319	Hs.495897	NM_004319
H3F3A	-3.7	3.3	H3 histone, family 3A (H3F3A), mRNA	NM_002107	Hs.546259	CR621187
THC2664215	-3.7	6.7	THC2664215	THC2664215	Unknown	
KIAA1713	-3.7	4.6	mRNA for KIAA1713 protein, partial cds.	AB051500	Hs.464876	NM_030632
AK127494	-3.7	2.1	cDNA FLJ45587 fis, clone BRTHA3014105.	AK127494	Hs.588006	AK127494
MAF	-3.7	9.3	v-maf musculoaponeurotic fibrosarcoma oncogene homolog (avian) (MAF), transcript variant 2, mRNA	NM_001031804	Hs.699396	NM_001031804
FLJ30901	-3.7	9.1	cDNA FLJ30901 fis, clone FEBRA2005778, weakly similar to INTEGUMENTARY MUCIN A.1 PRECURSOR.	AK055463	Unknown	
PRDM16	-3.7	3.9	PR domain containing 16 (PRDM16), transcript variant 1, mRNA	NM_022114	Hs.99500	NM_022114
THC2660448	-3.7	2.8	THC2660448	THC2660448	Unknown	
NSBP1	-3.7	3.9	nucleosomal binding protein 1 (NSBP1), mRNA	NM_030763	Hs.282204	AF250329
GALNTL4	-3.7	3.8	UDP-N-acetyl-alpha-D-galactosamine:polypeptide N-acetylgalactosaminyltransferase-like 4 (GALNTL4), mRNA	NM_198516	Hs.655152	AK124690
TMCO1	-3.7	1.8	transmembrane and coiled-coil domains 1 (TMCO1), mRNA	NM_019026	Hs.93832	CR614153
CTNNA2	-3.7	16.7	catenin (cadherin-associated protein), alpha 2 (CTNNA2), mRNA	NM_004389	Hs.167368	AK127226
TPM1	-3.7	3.2	tropomyosin 1 (alpha) (TPM1), transcript variant 3, mRNA	NM_001018004	Hs.133892	BX648171
CHGB	-3.7	22.4	chromogranin B (secretogranin 1) (CHGB), mRNA	NM_001819	Hs.516874	BC000375
THC2644853	-3.7	2.1	Q9VWG7_DROME (Q9VWG7) CG14218-PA, partial (7%)	THC2644853	Unknown	
AMBP	-3.7	54.6	alpha-1-microglobulin/bikunin precursor (AMBP), mRNA	NM_001633	Hs.436911	NM_001633
AK097877	-3.7	2.5	cDNA FLJ40558 fis, clone THYMU2002756.	AK097877	Unknown	
THC2671532	-3.7	2.2	Q4TC70_TETNG (Q4TC70) Chromosome undetermined SCAF7048, whole genome shotgun sequence, partial (7%)	THC2671532	Unknown	
AF087980	-3.7	5.3	full length insert cDNA clone YW27H10.	AF087980	Hs.643584	NM_178822
CECR6	-3.7	5.2	cat eye syndrome chromosome region, candidate 6 (CECR6), mRNA	NM_031890	Hs.209577	AF307451
UNQ1887	-3.7	1.8	cDNA FLJ39830 fis, clone SPLEN2012846.	AK097149	Unknown	
C7orf13	-3.7	2.1	processed retroposon C7orf13 (C7orf13) mRNA, partial cds.	AF439977	Hs.647014	AF439977
BAMBI	-3.7	5.3	BMP and activin membrane-bound inhibitor homolog (Xenopus laevis) (BAMBI), mRNA	NM_012342	Hs.533336	NM_012342
TUFT1	-3.7	4.9	tuftelin 1 (TUFT1), mRNA	NM_020127	Hs.489922	NM_020127
ARID1A	-3.7	2.2	AT rich interactive domain 1A (SWI-like) (ARID1A), transcript variant 1, mRNA	NM_006015	Hs.468972	NM_006015
THC2612020	-3.7	2.7	THC2612020	THC2612020	Unknown	
PCDH11Y	-3.7	5.9	protocadherin 11 Y-linked (PCDH11Y), transcript variant c, mRNA	NM_032973	Hs.661308	NM_032973
ZC3HAV1L	-3.7	1.8	cDNA FLJ40837 fis, clone TRACH2013495.	AK098156	Hs.512833	AK098156
MLF1IP	-3.7	2.6	MLF1 interacting protein (MLF1IP), mRNA	NM_024629	Hs.575032	AF516710
ZNF148	-3.7	1.6	zinc finger protein 148 (ZNF148), mRNA	NM_021964	Hs.592591	NM_021964
RABGAP1L	-3.7	5.7	mRNA for KIAA0471 protein, partial cds.	AB007940	Hs.585378	AB007940

THC2675062	-3.7	8.8	THC2675062	THC2675062	Unknown	
PHIP	-3.7	3.5	pleckstrin homology domain interacting protein (PHIP), mRNA	NM_017934	Hs.511817	NM_017934
TYR	-3.7	62.4	tyrosinase (oculocutaneous albinism IA) (TYR), mRNA	NM_000372	Hs.503555	M27160
THC2662740	-3.7	1.9	ALU1_HUMAN (P39188) Alu subfamily J sequence contamination warning entry, partial (6%)	THC2662740	Unknown	
NSBP1	-3.7	4.0	nucleosomal binding protein 1 (NSBP1), mRNA	NM_030763	Hs.282204	AF250329
TMEM16D	-3.7	19.3	transmembrane protein 16D (TMEM16D), mRNA	NM_178826	Hs.58785	NM_178826
LOC643313	-3.7	5.3	cDNA clone IMAGE:4129277, partial cds.	BC006361	Hs.676949	BC006361
LRRC37A3	-3.7	2.3	leucine rich repeat containing 37, member A3 (LRRC37A3), mRNA	NM_199340	Hs.551962	NM_199340
GTPBP9	-3.7	3.0	GTP-binding protein 9 (putative) (GTPBP9), transcript variant 1, mRNA	NM_013341	Hs.157351	NM_013341
ZNRF3	-3.7	2.8	mRNA for KIAA1133 protein, partial cds.	AB051436	Hs.655242	AB051436
AK123066	-3.7	26.9	cDNA FLJ41071 fis, clone 3NB692003538.	AK123066	Hs.674409	CR593779
GSTM1	-3.7	2.0	glutathione S-transferase M1 (GSTM1), transcript variant 2, mRNA	NM_146421	Hs.301961	BQ880398
C1QTNF3	-3.7	8.9	C1q and tumor necrosis factor related protein 3 (C1QTNF3), transcript variant 2, mRNA	NM_181435	Hs.171929	NM_181435
ENC1	-3.7	2.5	ectodermal-neural cortex (with BTB-like domain) (ENC1), mRNA	NM_003633	Hs.104925	AF059611
AK023774	-3.7	2.0	cDNA FLJ13712 fis, clone PLACE2000394.	AK023774	Hs.671113	AK023774
AF086375	-3.7	2.6	full length insert cDNA clone ZD68B12.	AF086375	Hs.264606	BG571805
GMEB1	-3.7	2.0	Glucocorticoid modulatory element-binding protein 1 (GMEB-1) (Parvovirus initiation factor p96) (PIF p96) (DNA-binding protein p96PIF).	ENST00000373816	Unknown	
THC2669975	-3.7	3.9	THC2669975	THC2669975	Unknown	
ID2	-3.7	5.5	inhibitor of DNA binding 2, dominant negative helix-loop-helix protein (ID2), mRNA	NM_002166	Hs.180919	CR623038
RDM1	-3.7	9.4	RAD52 motif 1 (RDM1), transcript variant 2, mRNA	NM_001034836	Hs.194411	NM_145654
PAQR6	-3.7	10.6	progestin and adipoQ receptor family member VI (PAQR6), transcript variant 1, mRNA	NM_024897	Hs.235873	NM_024897
GSTM1	-3.7	1.9	glutathione S-transferase M1 (GSTM1), transcript variant 2, mRNA	NM_146421	Hs.301961	BQ880398
THC2508355	-3.7	3.1	Q25GJ8_MACFA (Q25GJ8) Brain cDNA, clone: QflA-22015, complete	THC2508355	Unknown	
BAI1	-3.7	3.1	brain-specific angiogenesis inhibitor 1 (BAI1), mRNA	NM_001702	Hs.194654	NM_001702
CR936791	-3.7	3.7	mRNA; cDNA DKFZp781C2356 (from clone DKFZp781C2356).	CR936791	Hs.696414	CR936791
FGFR2	-3.7	19.9	fibroblast growth factor receptor 2 (bacteria-expressed kinase, keratinocyte growth factor receptor, craniofacial dysostosis 1, Crouzon syndrome, Pfeiffer syndrome, Jackson-Weiss syndrome) (FGFR2), transcript variant 2, mRNA	NM_022970	Hs.533683	NM_022970
C20orf133	-3.7	2.7	chromosome 20 open reading frame 133 (C20orf133), transcript variant 1, mRNA	NM_080676	Hs.661576	NM_080676
BG542103	-3.7	3.8	602571323F1 NIH_MGC_77 cDNA clone IMAGE:4695748 5', mRNA sequence	BG542103	Hs.560932	BG542103
TGFB2	-3.6	3.0	transforming growth factor, beta 2 (TGFB2), mRNA	NM_003238	Hs.133379	AB209842
SNCAIP	-3.6	2.9	synuclein, alpha interacting protein (synphilin) (SNCAIP), mRNA	NM_005460	Hs.426463	BC040552
EVI5	-3.6	1.7	ecotropic viral integration site 5 (EVI5), mRNA	NM_005665	Hs.656836	NM_005665
A_32_P95973	-3.6	4.5	A_32_P95973	A_32_P95973	Unknown	
ABCC5	-3.6	2.9	ATP-binding cassette, sub-family C (CFTR/MRP), member 5 (ABCC5), transcript variant 1, mRNA	NM_005688	Hs.368563	AF146074
SPATA6	-3.6	3.3	spermatogenesis associated 6	ENST00000371847	Unknown	
THC2611641	-3.6	5.6	Q6DIG3_XENTR (Q6DIG3) Hedgehog interacting protein, partial (3%)	THC2611641	Unknown	
SAA4	-3.6	4.6	serum amyloid A4, constitutive (SAA4), mRNA	NM_006512	Hs.654493	BG564326
ZFHX2	-3.6	5.6	zinc finger homeobox 2 (ZFHX2), mRNA	NM_033400	Unknown	
METTL3	-3.6	2.1	full-length cDNA clone CS0DF004YO04 of Fetal brain of (human).	CR594281	Hs.168799	AK097873
ACVR2B	-3.6	3.5	activin A receptor, type IIB (ACVR2B), mRNA	NM_001106	Hs.174273	NM_001106
ONECUT2	-3.6	45.2	one cut domain, family member 2 (ONECUT2), mRNA	NM_004852	Hs.194725	NM_004852
STK33	-3.6	3.5	serine/threonine kinase 33 (STK33), mRNA	NM_030906	Hs.501833	AK122808
SNAP25	-3.6	22.7	synaptosomal-associated protein, 25kDa (SNAP25), transcript variant 1, mRNA	NM_003081	Hs.167317	AK090857
PLCB1	-3.6	1.5	phospholipase C, beta 1 (phosphoinositide-specific) (PLCB1), transcript variant 1, mRNA	NM_015192	Hs.431173	AB011153
C14orf10	-3.6	1.6	chromosome 14 open reading frame 10 (C14orf10), mRNA	NM_017917	Hs.530712	CR602202
C20orf103	-3.6	10.4	chromosome 20 open reading frame 103 (C20orf103), mRNA	NM_012261	Hs.22920	NM_012261
HIST1H2AE	-3.6	3.3	histone cluster 1, H2ae (HIST1H2AE), mRNA	NM_021052	Hs.121017	BE741093
MAN1A2	-3.6	2.0	mannosidase, alpha, class 1A, member 2 (MAN1A2), mRNA	NM_006699	Hs.435938	BC052954
AKAP11	-3.6	2.2	A kinase (PRKA) anchor protein 11 (AKAP11), transcript variant 1, mRNA	NM_016248	Hs.105105	AF176555
MAGEH1	-3.6	2.2	melanoma antigen family H, 1 (MAGEH1), mRNA	NM_014061	Hs.279819	AF143235
A_32_P47474	-3.6	3.3	A_32_P47474	A_32_P47474	Unknown	
FAM39DP	-3.6	2.0	family with sequence similarity 39, member D pseudogene (FAM39DP), mRNA	NM_199163	Unknown	

LHX2	-3.6	4.3	LIM homeobox 2 (LHX2), mRNA	NM_004789	Hs.696425	AK027597
IGF1	-3.6	4.3	insulin-like growth factor 1 (somatomedin C) (IGF1), mRNA	NM_000618	Hs.160562	NM_000618
A_32_P103614	-3.6	7.6	A_32_P103614	A_32_P103614	Unknown	
OLFM1	-3.6	5.8	olfactomedin 1 (OLFM1), transcript variant 2, mRNA	NM_006334	Hs.522484	NM_014279
ZBED5	-3.6	2.2	zinc finger, BED-type containing 5 (ZBED5), mRNA	NM_021211	Unknown	

Supplemental Table A6

Genes Upregulated in FH vs CM (634)

SAM FDR 0.1%

Gene Name	Score(d)	Fold Upreg. FH	Description	Acc	UGCluster	UGRepAcc
TUSC1	20.0	34.6	tumor suppressor candidate 1 (TUSC1), mRNA	NM_001004125	Hs.26268	NM_001004125
TUSC1	19.8	42.3	tumor suppressor candidate 1 (TUSC1), mRNA	NM_001004125	Hs.26268	NM_001004125
ITGB3	17.2	13.3	integrin, beta 3 (platelet glycoprotein IIIa, antigen CD61) (ITGB3), mRNA	NM_000212	Hs.218040	NM_000212
AK092744	16.4	12.7	cDNA FLJ35425 fis, clone SMINT2001615, highly similar to FERRITIN LIGHT CHAIN.	AK092744	Unknown	
ITGB3	14.9	14.5	integrin, beta 3 (platelet glycoprotein IIIa, antigen CD61) (ITGB3), mRNA	NM_000212	Hs.218040	NM_000212
IRAK3	14.0	13.8	interleukin-1 receptor-associated kinase 3 (IRAK3), mRNA	NM_007199	Hs.369265	BC057800
AK023663	13.4	19.6	cDNA FLJ13601 fis, clone PLACE1010069.	AK023663	Hs.594861	AL049435
CDH13	13.2	8.6	cadherin 13, H-cadherin (heart) (CDH13), mRNA	NM_001257	Hs.654386	NM_001257
CSRP1	13.1	18.8	cysteine and glycine-rich protein 1 (CSRP1), mRNA	NM_004078	Hs.108080	CR627407
CAT	12.8	20.8	catalase (CAT), mRNA	NM_001752	Hs.502302	NM_001752
WDR69	12.7	19.0	WD repeat domain 69 (WDR69), mRNA	NM_178821	Hs.424594	AK127882
TSPYL5	11.7	56.7	TSPY-like 5 (TSPYL5), mRNA	NM_033512	Hs.173094	NM_033512
BST1	11.5	6.8	bone marrow stromal cell antigen 1 (BST1), mRNA	NM_004334	Hs.169998	NM_004334
ITGB3	10.9	13.5	integrin, beta 3 (platelet glycoprotein IIIa, antigen CD61) (ITGB3), mRNA	NM_000212	Hs.218040	NM_000212
AF390550	10.9	33.5	cervical cancer suppressor gene 5 mRNA, complete cds.	AF390550	Hs.592078	AY279347
CHCHD2	10.8	23.5	coiled-coil-helix-coiled-coil-helix domain containing 2 (CHCHD2), mRNA	NM_016139	Hs.389996	BM553650
CHCHD2	10.8	24.0	coiled-coil-helix-coiled-coil-helix domain containing 2 (CHCHD2), mRNA	NM_016139	Hs.389996	BM553650
ITGB3	10.4	10.8	integrin, beta 3 (platelet glycoprotein IIIa, antigen CD61) (ITGB3), mRNA	NM_000212	Hs.218040	NM_000212
MDM2	10.4	4.0	Mdm2, transformed 3T3 cell double minute 2, p53 binding protein (mouse) (MDM2), transcript variant MDM2b, mRNA	NM_006879	Hs.567303	M92424
AI925475	10.4	42.6	wo30h04.x1 NCI_CGAP_Gas4 cDNA clone IMAGE:2456887 3' similar to WP:C16C10.11 CE01491 HYPOTHETICAL 15.4 KD PROTEIN C16C10.11 IN CHROMOSOME III ;, mRNA sequence	AI925475	Hs.575202	AI925475
CAV3	10.3	6.9	caveolin 3 (CAV3), transcript variant 2, mRNA	NM_001234	Hs.98303	NM_033337
CSRP1	10.2	26.7	cysteine and glycine-rich protein 1 (CSRP1), mRNA	NM_004078	Hs.108080	CR627407
FLJ36644	9.9	5.9	cDNA FLJ36644 fis, clone UMVEN1000143.	AK093963	Hs.225661	XM_001126095
LOC730556	9.8	12.8	PREDICTED: similar to Coiled-coil-helix-coiled-coil-helix domain-containing protein 2 (HCV NS2 trans-regulated protein) (NS2TP) (LOC730556), mRNA	XR_015322	Hs.646956	XR_015322
ITGB3	9.8	8.8	integrin, beta 3 (platelet glycoprotein IIIa, antigen CD61) (ITGB3), mRNA	NM_000212	Hs.218040	NM_000212
TYW3	9.8	42.0	tRNA-yW synthesizing protein 3 homolog (S. cerevisiae) (TYW3), mRNA	NM_138467	Hs.348411	BX647591
ITGB3	9.8	11.0	integrin, beta 3 (platelet glycoprotein IIIa, antigen CD61) (ITGB3), mRNA	NM_000212	Hs.218040	NM_000212
SDPR	9.8	42.2	serum deprivation response (phosphatidylserine binding protein) (SDPR), mRNA	NM_004657	Hs.26530	NM_004657
CHCHD2	9.7	10.5	coiled-coil-helix-coiled-coil-helix domain containing 2 (CHCHD2), mRNA	NM_016139	Hs.389996	BM553650
HSPA4L	9.6	3.2	heat shock 70kDa protein 4-like (HSPA4L), mRNA	NM_014278	Hs.135554	NM_014278
BC015962	9.6	3.4	clone IMAGE:4081125, mRNA, partial cds.	BC015962	Hs.517946	AB062478
C17orf51	9.3	84.6	C17orf51 protein (Fragment).	ENST0000037[illegible]	Unknown	
ITGB3	9.3	12.6	integrin, beta 3 (platelet glycoprotein IIIa, antigen CD61) (ITGB3), mRNA	NM_000212	Hs.218040	NM_000212
DRAM	9.2	4.8	damage-regulated autophagy modulator (DRAM), mRNA	NM_018370	Hs.525634	NM_018370
ENST00000324[illegible]	9.2	5.9	Protein phosphatase Slingshot homolog 2 (EC 3.1.3.48) (EC 3.1.3.16) (SSH-2L) (hSSH-2L).	ENST0000003246[illegible]	Unknown	
STOM	9.1	5.0	stomatin (STOM), transcript variant 2, mRNA	NM_198194	Hs.253903	NM_004099
ChGn	9.1	3.6	chondroitin beta1,4 N-acetylgalactosaminyltransferase (ChGn), mRNA	NM_018371	Hs.655166	BX649103
ITGB3	8.7	13.4	integrin, beta 3 (platelet glycoprotein IIIa, antigen CD61) (ITGB3), mRNA	NM_000212	Hs.218040	NM_000212
C21orf119	8.6	30.8	chromosome 21 open reading frame 119 (C21orf119), mRNA	NM_032910	Unknown	
FADS3	8.6	5.3	fatty acid desaturase 3 (FADS3), mRNA	NM_021727	Hs.21765	AB209356
DRAM	8.5	4.7	damage-regulated autophagy modulator (DRAM), mRNA	NM_018370	Hs.525634	NM_018370
BIRC4BP	8.4	11.8	XIAP associated factor-1 (BIRC4BP), transcript variant 1, mRNA	NM_017523	Hs.441975	BX649188
CHCHD2	8.4	10.5	coiled-coil-helix-coiled-coil-helix domain containing 2 (CHCHD2), mRNA	NM_016139	Hs.389996	BM553650
COMT	8.4	14.8	catechol-O-methyltransferase (COMT), transcript variant MB-COMT, mRNA	NM_000754	Hs.700588	CR616943
EHD1	8.3	3.9	EH-domain containing protein testilin mRNA, complete cds.	AF099011	Unknown	
NLF2	8.3	17.7	PREDICTED: nuclear localized factor 2 (NLF2), mRNA	XM_940314	Unknown	
LOC205251	8.2	20.2	cDNA clone IMAGE:5763979.	BC064430	Hs.128499	BC064430
DYNLT3	8.2	18.0	dynein, light chain, Tctex-type 3 (DYNLT3), mRNA	NM_006520	Hs.446392	BC000968

CSDA	8.0	3.6	cold shock domain protein A (CSDA), mRNA	NM_003651	Hs.221889	AB209896
PXMP4	8.0	34.9	peroxisomal membrane protein 4, 24kDa (PXMP4), transcript variant 1, mRNA	NM_007238	Hs.654857	NM_007238
KIAA1652	8.0	12.3	mRNA for KIAA1652 protein, partial cds.	AB051439	Hs.443430	AB051439
MGP	8.0	5.1	matrix Gla protein (MGP), mRNA	NM_000900	Hs.365706	CR623037
CYB5R3	8.0	2.9	cytochrome b5 reductase 3 (CYB5R3), transcript variant S, mRNA	NM_007326	Hs.700572	NM_007326
ICA1	7.9	3.1	islet cell autoantigen 1, 69kDa (ICA1), transcript variant 2, mRNA	NM_004968	Hs.487561	NM_004968
HIST1H1A	7.9	26.1	histone cluster 1, H1a (HIST1H1A), mRNA	NM_005325	Hs.150206	BC112140
PCDH12	7.9	21.3	protocadherin 12 (PCDH12), mRNA	NM_016580	Hs.439474	NM_016580
ITGB3	7.8	13.7	integrin, beta 3 (platelet glycoprotein IIIa, antigen CD61) (ITGB3), mRNA	NM_000212	Hs.218040	NM_000212
PLA2G4C	7.8	8.2	phospholipase A2, group IVC (cytosolic, calcium-independent) (PLA2G4C), mRNA	NM_003706	Hs.631562	BC063416
PLP2	7.8	4.6	proteolipid protein 2 (colonic epithelium-enriched) (PLP2), mRNA	NM_002668	Hs.77422	CR607707
TYW3	7.7	14.8	tRNA-yW synthesizing protein 3 homolog (S. cerevisiae) (TYW3), mRNA	NM_138467	Hs.348411	BX647591
RP11-262H14.[illegible]	7.6	5.1	hypothetical protein MGC21881 (MGC21881), mRNA	NM_203448	Unknown	
THC2640472	7.6	6.8	Q2M1U4_HUMAN (Q2M1U4) Catalase, partial (31%)	THC2640472	Unknown	
MDM2	7.5	5.1	Mdm2, transformed 3T3 cell double minute 2, p53 binding protein (mouse) (MDM2), transcript variant MDM2, mRNA	NM_002392	Hs.567303	M92424
ZNF132	7.5	2.8	zinc finger protein 132 (ZNF132), mRNA	NM_003433	Hs.156169	NM_003433
FHL2	7.5	4.7	four and a half LIM domains 2 (FHL2), transcript variant 5, mRNA	NM_001039492	Hs.443687	CR936799
CAV2	7.5	5.2	caveolin 2 (CAV2), transcript variant 1, mRNA	NM_001233	Hs.212332	NM_001233
C13orf33	7.5	14.0	chromosome 13 open reading frame 33 (C13orf33), mRNA	NM_032849	Hs.646647	NM_032849
CSDA	7.5	3.2	cold shock domain protein A (CSDA), mRNA	NM_003651	Hs.221889	AB209896
AK123143	7.5	16.9	cDNA FLJ41148 fis, clone BRACE2037310.	AK123143	Hs.514016	XM_378661
POLH	7.4	3.0	cDNA: FLJ21978 fis, clone HEP06046, highly similar to AF158185 xeroderma pigmentosum variant RAD30 (RAD30) mRNA.	AK025631	Hs.655467	AB024313
ATP5E	7.4	2.6	ATP synthase, H+ transporting, mitochondrial F1 complex, epsilon subunit (ATP5E), nuclear gene encoding mitochondrial protein, mRNA	NM_006886	Hs.177530	CR595852
ANTXR2	7.4	3.4	anthrax toxin receptor 2 (ANTXR2), mRNA	NM_058172	Hs.162963	NM_058172
PCYT1A	7.4	2.7	phosphate cytidylyltransferase 1, choline, alpha (PCYT1A), mRNA	NM_005017	Hs.435767	BC042043
C21orf6	7.3	3.2	chromosome 21 open reading frame 6 (C21orf6), mRNA	NM_016940	Hs.34136	CR592518
SSR3	7.3	2.4	signal sequence receptor, gamma (translocon-associated protein gamma) (SSR3), mRNA	NM_007107	Hs.518346	NM_007107
GAS8	7.3	2.6	growth arrest-specific 8 (GAS8), mRNA	NM_001481	Hs.431792	CR749499
TADA2L	7.2	2.5	transcriptional adaptor 2 (ADA2 homolog, yeast)-like (TADA2L), transcript variant 1, mRNA	NM_001488	Hs.500066	AK225918
FGF5	7.2	5.5	fibroblast growth factor 5 (FGF5), transcript variant 1, mRNA	NM_004464	Hs.37055	NM_004464
THC2650457	7.2	12.0	ALU6_HUMAN (P39193) Alu subfamily SP sequence contamination warning entry, partial (12%)	THC2650457	Unknown	
CASP7	7.1	3.2	caspase 7, apoptosis-related cysteine peptidase (CASP7), transcript variant delta, mRNA	NM_033338	Hs.9216	NM_033338
CD70	7.1	13.4	CD70 molecule (CD70), mRNA	NM_001252	Hs.501497	BM464627
ENST00000371[illegible]	7.0	12.7	Ectonucleoside triphosphate diphosphohydrolase 1 (EC 3.6.1.5) (NTPDase 1) (Ecto-ATP diphosphohydrolase) (ATPDase) (Lymphoid cell activation antigen) (Ecto-apyrase) (CD39 antigen).	ENST00000371[illegible]	Unknown	
FAM26B	7.0	20.8	family with sequence similarity 26, member B (FAM26B), mRNA	NM_015916	Hs.241545	AK125276
ASAHL	7.0	4.3	N-acylsphingosine amidohydrolase (acid ceramidase)-like (ASAHL), transcript variant 1, mRNA	NM_014435	Hs.699178	NM_014435
FAM126A	7.0	4.7	family with sequence similarity 126, member A (FAM126A), mRNA	NM_032581	Hs.85603	AL833296
BX648207	6.9	10.9	mRNA; cDNA DKFZp686E16168 (from clone DKFZp686E16168).	BX648207	Hs.23554	BX648207
TRIM25	6.9	2.5	tripartite motif-containing 25 (TRIM25), mRNA	NM_005082	Hs.528952	NM_005082
CBR1	6.8	70.0	carbonyl reductase 1 (CBR1), mRNA	NM_001757	Hs.88778	BM810059
LY96	6.8	10.1	lymphocyte antigen 96 (LY96), mRNA	NM_015364	Hs.660766	BM918324
DTX3L	6.8	4.0	deltex 3-like (Drosophila) (DTX3L), mRNA	NM_138287	Hs.518201	BX648267
A_24_P152315	6.8	2.4	A_24_P152315	A_24_P152315	Unknown	
YKT6	6.8	2.8	YKT6 v-SNARE homolog (S. cerevisiae) (YKT6), mRNA	NM_006555	Hs.520794	NM_006555
C21orf119	6.8	14.9	chromosome 21 open reading frame 119 (C21orf119), mRNA	NM_032910	Unknown	
BIRC4BP	6.8	20.8	XIAP associated factor-1 (BIRC4BP), transcript variant 1, mRNA	NM_017523	Hs.441975	BX649188
PLSCR4	6.7	9.0	phospholipid scramblase 4 (PLSCR4), mRNA	NM_020353	Hs.477869	BC028354
C6orf150	6.7	9.3	chromosome 6 open reading frame 150 (C6orf150), mRNA	NM_138441	Hs.658405	BC113606
MRPL30	6.6	4.2	mitochondrial ribosomal protein L30 (MRPL30), nuclear gene encoding mitochondrial protein, transcript variant 1, mRNA	NM_145212	Hs.590896	NM_145212
NCOA7	6.6	3.4	nuclear receptor coactivator 7 (NCOA7), mRNA	NM_181782	Hs.171426	AL834442
CR608961	6.6	4.3	full-length cDNA clone CS0DI034YJ14 of Placenta Cot 25-normalized of (human).	CR608961	Hs.24178	AK225043

ITGB3	6.5	25.8	integrin beta 3 mRNA, partial cds, alternatively spliced.	S70348	Hs.218040	NM_000212
A_24_P903378	6.5	3.0	A_24_P903378	A_24_P903378	Unknown	
LOC392221	6.5	18.0	PREDICTED: similar to Coiled-coil-helix-coiled-coil-helix domain-containing protein 2 (HCV NS2 trans-regulated protein) (NS2TP) (LOC392221), mRNA	ENST00000331	Unknown	
DTX3L	6.5	2.3	deltex 3-like (Drosophila) (DTX3L), mRNA	NM_138287	Hs.518201	BX648267
FNDC3B	6.5	3.6	fibronectin type III domain containing 3B, mRNA (cDNA clone IMAGE:3882800), complete cds.	BC012204	Hs.159430	NM_022763
FLJ36748	6.5	3.9	hypothetical protein FLJ36748 (FLJ36748), mRNA	NM_152406	Hs.483793	NM_152406
IL12A	6.5	7.9	interleukin 12A (natural killer cell stimulatory factor 1, cytotoxic lymphocyte maturation factor 1, p35) (IL12A), mRNA	NM_000882	Hs.673	BC035571
THC2545456	6.5	5.5	THC2545456	THC2545456	Unknown	
LOC153277	6.5	5.1	cDNA FLJ20096 fis, clone COL04378.	AK000103	Hs.595953	AK000103
ENST00000370	6.4	21.1	Quinone oxidoreductase (EC 1.6.5.5) (NADPH:quinone reductase) (Zeta- crystallin).	ENST00000370	Unknown	
CD34	6.4	6.8	CD34 molecule (CD34), transcript variant 2, mRNA	NM_001773	Hs.374990	BX640941
CD274	6.4	11.2	CD274 molecule (CD274), mRNA	NM_014143	Hs.521989	NM_014143
PUS7L	6.4	15.9	pseudouridylate synthase 7 homolog (S. cerevisiae)-like (PUS7L), mRNA	NM_031292	Hs.445814	NM_001098615
TITF1	6.4	3.5	thyroid transcription factor 1 (TITF1), transcript variant 2, mRNA	NM_003317	Hs.700584	NM_003317
C14orf149	6.4	2.7	chromosome 14 open reading frame 149 (C14orf149), mRNA	NM_144581	Hs.29706	AK058165
HMGCS1	6.3	4.3	3-hydroxy-3-methylglutaryl-Coenzyme A synthase 1 (soluble) (HMGCS1), mRNA	NM_002130	Hs.397729	BX537620
KLF7	6.3	3.4	Kruppel-like factor 7 (ubiquitous) (KLF7), mRNA	NM_003709	Hs.471221	BX537890
NUDT16	6.2	2.4	nudix (nucleoside diphosphate linked moiety X)-type motif 16 (NUDT16), mRNA	NM_152395	Hs.591313	AK055827
MTHFD1L	6.2	3.6	methylenetetrahydrofolate dehydrogenase (NADP+ dependent) 1-like (MTHFD1L), mRNA	NM_015440	Hs.591343	AK127089
LOC285074	6.2	2.7	hypothetical protein LOC285074 (LOC285074), mRNA	NM_001012626	Unknown	
ENDOGL1	6.2	4.0	endonuclease G-like 1 (ENDOGL1), mRNA	NM_005107	Hs.517897	AK023235
ITGB3	6.2	10.8	integrin, beta 3 (platelet glycoprotein IIIa, antigen CD61) (ITGB3), mRNA	NM_000212	Hs.218040	NM_000212
BBC3	6.2	3.3	BCL2 binding component 3 (BBC3), mRNA	NM_014417	Hs.467020	AF332558
HEG1	6.1	4.0	mRNA for KIAA1237 protein, partial cds.	AB033063	Hs.477420	NM_020733
THC2559002	6.1	3.8	THC2559002	THC2559002	Unknown	
A_24_P289984	6.1	13.1	A_24_P289984	A_24_P289984	Unknown	
ZNF619	6.1	3.2	zinc finger protein 619 (ZNF619), mRNA	NM_173656	Hs.407159	NM_173656
ZBTB38	6.1	3.8	cDNA FLJ36882 fis, clone BLADE2000340, weakly similar to ZINC FINGER PROTEIN 177.	AK094201	Hs.518301	NM_001080412
TLN1	6.0	2.1	talin 1 (TLN1), mRNA	NM_006289	Hs.471014	AB028950
SCN1B	6.0	3.9	sodium channel, voltage-gated, type I, beta (SCN1B), transcript variant b, mRNA	NM_199037	Hs.436646	BC112922
TAF7L	6.0	6.8	TAF7-like RNA polymerase II, TATA box binding protein (TBP)-associated factor, 50kDa (TAF7L), mRNA	NM_024885	Hs.223806	BC043391
AF086329	6.0	3.3	full length insert cDNA clone ZD54C08.	AF086329	Unknown	
RP11-262H14.4	6.0	5.1	hypothetical protein MGC21881 (MGC21881), mRNA	NM_203448	Unknown	
USP36	6.0	2.3	ubiquitin specific peptidase 36 (USP36), mRNA	NM_025090	Hs.464243	AB040886
OAF	6.0	2.5	OAF homolog (Drosophila) (OAF), mRNA	NM_178507	Hs.445081	BC047726
C6orf128	5.9	5.9	chromosome 6 open reading frame 128 (C6orf128), mRNA	NM_145316	Hs.520101	NM_145316
ZNF248	5.9	20.0	zinc finger protein 248 (ZNF248), mRNA	NM_021045	Hs.572001	NM_021045
GOSR2	5.9	2.5	golgi SNAP receptor complex member 2 (GOSR2), transcript variant B, mRNA	NM_054022	Hs.463278	NM_004287
CAMK1	5.9	3.4	calcium/calmodulin-dependent protein kinase I (CAMK1), mRNA	NM_003656	Hs.434875	AB209395
MICAL2	5.9	2.4	microtubule associated monoxygenase, calponin and LIM domain containing 2 (MICAL2), mRNA	NM_014632	Hs.501928	BX538021
ASTE1	5.9	2.1	asteroid homolog 1 (Drosophila) (ASTE1), mRNA	NM_014065	Hs.100878	NM_014065
CD302	5.9	2.7	CD302 molecule (CD302), mRNA	NM_014880	Hs.130014	AY314007
ST6GALNAC4	5.9	3.1	ST6 (alpha-N-acetyl-neuraminyl-2,3-beta-galactosyl-1,3)-N-acetylgalactosaminide alpha-2,6-sialyltransferase 4 (ST6GALNAC4), transcript variant 1, mRNA	NM_175039	Hs.3972	NM_175039
STARD13	5.9	5.5	START domain containing 13 (STARD13), transcript variant alpha, mRNA	NM_178006	Hs.507704	BX647695
ATG7	5.9	2.7	ATG7 autophagy related 7 homolog (S. cerevisiae) (ATG7), mRNA	NM_006395	Hs.38032	AL122075
MMP10	5.9	6.9	matrix metallopeptidase 10 (stromelysin 2) (MMP10), mRNA	NM_002425	Hs.2258	AK222601
FKBP11	5.9	3.4	FK506 binding protein 11, 19 kDa (FKBP11), mRNA	NM_016594	Hs.655103	AB209018
LTBR	5.8	19.8	lymphotoxin beta receptor (TNFR superfamily, member 3) (LTBR), mRNA	NM_002342	Hs.1116	AK128019
CMBL	5.8	4.2	carboxymethylenebutenolidase homolog (Pseudomonas) (CMBL), mRNA	NM_138809	Hs.192586	NM_138809
LRRC17	5.8	3.2	leucine rich repeat containing 17 (LRRC17), transcript variant 2, mRNA	NM_005824	Hs.567412	AK225701
TMEM106A	5.8	3.7	transmembrane protein 106A (TMEM106A), mRNA	NM_145041	Hs.567647	U25750
D15Wsu75e	5.8	3.4	DNA segment, Chr 15, Wayne State University 75, expressed (D15Wsu75e), mRNA	NM_015704	Hs.570455	BE869593

MR1	5.8	5.2	major histocompatibility complex, class I-related (MR1), mRNA	NM_001531	Hs.101840	AF031469
LENG1	5.8	2.4	leukocyte receptor cluster (LRC) member 1 (LENG1), mRNA	NM_024316	Hs.590974	BG249859
BG207267	5.8	4.5	RST26737 Athersys RAGE Library cDNA, mRNA sequence	BG207267	Hs.436359	BG207267
FCHSD2	5.8	3.4	FCH and double SH3 domains 2 (FCHSD2), mRNA	NM_014824	Hs.577053	AB018312
ENST0000033[illegible]	5.8	2.5	ENST00000335078	ENST0000033[illegible]	Unknown	
MDM2	5.8	5.6	Mdm2, transformed 3T3 cell double minute 2, p53 binding protein (mouse) (MDM2), transcript variant MDM2, mRNA	NM_002392	Hs.567303	M92424
BGN	5.8	3.5	biglycan (BGN), mRNA	NM_001711	Hs.821	BC004244
NTN4	5.8	8.3	netrin 4 (NTN4), mRNA	NM_021229	Hs.201034	NM_021229
AA563626	5.8	5.0	AA563626 ng47f01.s1 NCI_CGAP_Co3 cDNA clone IMAGE:937945 3', mRNA sequence	AA563626	Hs.192586	NM_138809
MGC87631	5.7	4.5	similar to hypothetical protein FLJ36492 (MGC87631), mRNA	NM_001004306	Hs.674830	BC068597
EMP1	5.7	10.3	epithelial membrane protein 1 (EMP1), mRNA	NM_001423	Hs.696241	NM_001423
STX11	5.7	6.4	syntaxin 11 (STX11), mRNA	NM_003764	Hs.118958	NM_003764
MDM2	5.7	5.3	Mdm2, transformed 3T3 cell double minute 2, p53 binding protein (mouse) (MDM2), transcript variant MDM2, mRNA	NM_002392	Hs.567303	M92424
PNPLA2	5.7	2.9	patatin-like phospholipase domain containing 2 (PNPLA2), mRNA	NM_020376	Hs.654697	AY203925
BC047636	5.7	4.2	cDNA clone IMAGE:4822429.	BC047636	Hs.64341	BC047636
DYNLT3	5.7	13.6	Dynein light chain Tctex-type 3 (T-complex-associated testis-expressed 1-like) (Protein 91/23).	ENST00000378[illegible]	Unknown	
THC2534222	5.7	7.7	Q8WUP8_HUMAN (Q8WUP8) MGC21881 protein (Fragment), partial (36%)	THC2534222	Unknown	
EVI2A	5.7	6.5	ecotropic viral integration site 2A (EVI2A), transcript variant 1, mRNA	NM_001003927	Hs.591198	NM_001003927
AK131396	5.7	10.7	cDNA FLJ16488 fis, clone BRTHA3008826.	AK131396	Unknown	
THC2659974	5.7	11.5	NM_059685 C15A11.1 {Caenorhabditis elegans} (exp=-1; wgp=0; cg=0), partial (7%)	THC2659974	Unknown	
THC2672076	5.7	5.9	LETM1_HUMAN (O95202) Leucine zipper-EF-hand-containing transmembrane protein 1, mitochondrial precursor, partial (20%)	THC2672076	Unknown	
STAT1	5.7	2.7	signal transducer and activator of transcription 1, 91kDa (STAT1), transcript variant beta, mRNA	NM_139266	Hs.699271	NM_007315
THSD4	5.7	3.3	thrombospondin, type I, domain containing 4 (THSD4), mRNA	NM_024817	Hs.387057	NM_024817
A_32_P201620	5.6	3.0	A_32_P201620	A_32_P201620	Unknown	
KIAA0664	5.6	2.3	KIAA0664 (KIAA0664), mRNA	NM_015229	Hs.22616	NM_015229
RAD23A	5.6	2.2	RAD23 homolog A (S. cerevisiae) (RAD23A), mRNA	NM_005053	Hs.643267	NM_005053
STAT1	5.6	2.7	signal transducer and activator of transcription 1, 91kDa (STAT1), transcript variant beta, mRNA	NM_139266	Hs.699271	NM_007315
ANTXR2	5.6	4.6	anthrax toxin receptor 2 (ANTXR2), mRNA	NM_058172	Hs.162963	NM_058172
MAPRE2	5.6	2.9	microtubule-associated protein, RP/EB family, member 2 (MAPRE2), mRNA	NM_014268	Hs.532824	BC007318
TRIB3	5.6	4.6	tribbles homolog 3 (Drosophila) (TRIB3), mRNA	NM_021158	Hs.516826	AY247738
STK17A	5.6	2.1	serine/threonine kinase 17a (apoptosis-inducing) (STK17A), mRNA	NM_004760	Hs.699353	NM_004760
THSD1	5.6	3.6	thrombospondin, type I, domain containing 1 (THSD1), transcript variant 1, mRNA	NM_018676	Hs.325667	AK096289
AW851396	5.6	2.9	AW851396 IL3-CT0220-170200-066-D05 CT0220 cDNA, mRNA sequence	AW851396	Hs.699196	CR936719
EPAS1	5.6	7.7	endothelial PAS domain protein 1 (EPAS1), mRNA	NM_001430	Hs.468410	NM_001430
BU632168	5.6	6.3	BU632168 UI-H-FE1-bea-d-23-0-UI.s1 NCI_CGAP_FE1 cDNA clone UI-H-FE1-bea-d-23-0-UI 3', mRNA sequence	BU632168	Hs.253903	NM_004099
THC2628527	5.6	2.1	IRO548013 HMG20A {Homo sapiens} (exp=-1; wgp=0; cg=0), partial (35%)	THC2628527	Unknown	
A_24_P332953	5.6	2.9	A_24_P332953	A_24_P332953	Unknown	
RP11-262H14.[illegible]	5.5	6.3	hypothetical protein MGC21881 (MGC21881), mRNA	NM_203448	Unknown	
OMP	5.5	2.3	olfactory marker protein (OMP), mRNA	NM_006189	Hs.673853	EL736194
BC030813	5.5	2.6	cDNA clone MGC:22645 IMAGE:4700961, complete cds.	BC030813	Hs.449621	AK128524
TNFRSF10A	5.5	4.3	tumor necrosis factor receptor superfamily, member 10a (TNFRSF10A), mRNA	NM_003844	Hs.591834	NM_003844
OR5A1	5.5	2.1	olfactory receptor, family 5, subfamily A, member 1 (OR5A1), mRNA	NM_001004728	Hs.553649	NM_001004728
IL6ST	5.5	3.4	interleukin 6 signal transducer (gp130, oncostatin M receptor) (IL6ST), transcript variant 1, mRNA	NM_002184	Hs.532082	BC071555
STAT1	5.5	2.6	signal transducer and activator of transcription 1, 91kDa (STAT1), transcript variant beta, mRNA	NM_139266	Hs.699271	NM_007315
DEPDC2	5.5	2.8	cDNA FLJ12987 fis, clone NT2RP3000068, weakly similar to SON OF SEVENLESS PROTEIN HOMOLOG 1.	AK023049	Hs.591867	AJ437636
ZNF528	5.5	3.4	zinc finger protein 528 (ZNF528), mRNA	NM_032423	Hs.662043	AK056552
NAT11	5.5	2.5	N-acetyltransferase 11 (NAT11), mRNA	NM_024771	Hs.523753	BX647309
PCDHGB2	5.5	9.5	protocadherin gamma subfamily B, 2 (PCDHGB2), transcript variant 2, mRNA	NM_032096	Hs.368160	AB002325
EIF4E3	5.5	3.6	eukaryotic translation initiation factor 4E family member 3 (EIF4E3), mRNA	NM_173359	Hs.655608	AL161983
AHNAK	5.5	7.6	AHNAK nucleoprotein (desmoyokin) (AHNAK), transcript variant 2, mRNA	NM_024060	Hs.502756	NM_001620
A_32_P210193	5.5	9.1	A_32_P210193	A_32_P210193	Unknown	

DYNLT3	5.5	47.5	dynein, light chain, Tctex-type 3 (DYNLT3), mRNA	NM_006520	Hs.446392	BC000968
RGS20	5.5	5.2	regulator of G-protein signalling 20 (RGS20), transcript variant 1, mRNA	NM_170587	Hs.368733	NM_170587
PAPSS2	5.5	3.5	3'-phosphoadenosine 5'-phosphosulfate synthase 2 (PAPSS2), transcript variant 2, mRNA	NM_001015880	Hs.524491	NM_001015880
HFE	5.5	4.9	hemochromatosis (HFE), transcript variant 1, mRNA	NM_000410	Hs.233325	NM_000410
TADA2L	5.4	2.2	transcriptional adaptor 2 (ADA2 homolog, yeast)-like (TADA2L), transcript variant 2, mRNA	NM_133439	Hs.500066	AK225918
SUHW4	5.4	14.0	suppressor of hairy wing homolog 4 (Drosophila) (SUHW4), transcript variant 1, mRNA	NM_017661	Hs.511477	BC040532
LOC641844	5.4	2.1	PREDICTED: similar to Succinate dehydrogenase cytochrome b small subunit, mitochondrial precursor (CybS) (Succinate-ubiquinone reductase membrane anchor subunit) (QPs3) (CII-4) (Succinate dehydrogenase complex subunit D) (Su...	XR_018036	Hs.647513	XR_018036
TEX261	5.4	2.5	testis expressed sequence 261 (TEX261), mRNA	NM_144582	Hs.516087	NM_144582
KLK9	5.4	2.0	kallikrein-related peptidase 9 (KLK9), mRNA	NM_012315	Hs.448942	DQ267419
CCDC44	5.4	2.7	coiled-coil domain containing 44 (CCDC44), mRNA	NM_016360	Hs.174134	AK094052
TK2	5.4	4.7	thymidine kinase 2, mitochondrial (TK2), mRNA	NM_004614	Hs.512619	NM_004614
CPT1A	5.4	3.8	carnitine palmitoyltransferase 1A (liver) (CPT1A), nuclear gene encoding mitochondrial protein, transcript variant 2, mRNA	NM_001031847	Hs.503043	AK172798
TMBIM1	5.4	3.1	transmembrane BAX inhibitor motif containing 1 (TMBIM1), mRNA	NM_022152	Hs.591605	AK090618
PRKACA	5.4	2.6	protein kinase, cAMP-dependent, catalytic, alpha (PRKACA), transcript variant 1, mRNA	NM_002730	Hs.695950	AK131561
ALDH1B1	5.4	3.2	aldehyde dehydrogenase 1 family, member B1 (ALDH1B1), nuclear gene encoding mitochondrial protein, mRNA	NM_000692	Hs.436219	NM_000692
C6orf57	5.4	2.3	chromosome 6 open reading frame 57 (C6orf57), mRNA	NM_145267	Hs.418495	BU598152
PAPSS2	5.4	3.3	3'-phosphoadenosine 5'-phosphosulfate synthase 2 (PAPSS2), transcript variant 2, mRNA	NM_001015880	Hs.524491	NM_001015880
STAT1	5.4	2.7	signal transducer and activator of transcription 1, 91kDa (STAT1), transcript variant beta, mRNA	NM_139266	Hs.699271	NM_007315
IMPDH1	5.4	2.7	Human IMP dehydrogenase type 1 mRNA complete cds.	J05272	Hs.654401	NM_000883
PCBD2	5.4	2.3	pterin-4 alpha-carbinolamine dehydratase/dimerization cofactor of hepatocyte nuclear factor 1 alpha (TCF1) 2 (PCBD2), mRNA	NM_032151	Hs.631804	NM_032151
WDR55	5.4	2.6	WD repeat domain 55 (WDR55), mRNA	NM_017706	Hs.286261	NM_017706
MTRF1L	5.4	2.5	mitochondrial translational release factor 1-like (MTRF1L), mRNA	NM_019041	Hs.225836	BC011873
WNK4	5.4	3.1	WNK lysine deficient protein kinase 4 (WNK4), mRNA	NM_032387	Hs.105448	NM_032387
FEM1C	5.4	2.6	fem-1 homolog c (C. elegans) (FEM1C), mRNA	NM_020177	Hs.47367	NM_020177
U04815	5.3	4.8	Human protein kinase PITSLRE alpha 1 mRNA, complete cds.	U04815	Unknown	
HGSNAT	5.3	2.5	cDNA FLJ32731 fis, clone TESTI2001134.	AK057293	Hs.600384	NM_152419
SECTM1	5.3	6.8	secreted and transmembrane 1 (SECTM1), mRNA	NM_003004	Hs.558009	NM_003004
C20orf80	5.3	17.2	mRNA; cDNA DKFZp686M08106 (from clone DKFZp686M08106).	CR933606	Hs.653099	CR933606
SGCB	5.3	1.9	sarcoglycan, beta (43kDa dystrophin-associated glycoprotein) (SGCB), mRNA	NM_000232	Hs.438953	NM_000232
FRMD4B	5.3	2.8	mRNA for KIAA1013 protein, partial cds.	AB023230	Hs.371681	NM_015123
PROSC	5.3	1.9	proline synthetase co-transcribed homolog (bacterial) (PROSC), mRNA	NM_007198	Hs.304792	NM_007198
CD68	5.3	12.6	CD68 molecule (CD68), transcript variant 1, mRNA	NM_001251	Hs.647419	NM_001251
TLN1	5.3	2.1	talin 1 (TLN1), mRNA	NM_006289	Hs.471014	AB028950
THC2518594	5.3	2.7	Q6PJD4_HUMAN (Q6PJD4) LOC389906 protein (Fragment), partial (10%)	THC2518594	Unknown	
BIRC4BP	5.3	18.8	XIAP associated factor-1 (BIRC4BP), transcript variant 1, mRNA	NM_017523	Hs.441975	BX649188
TEAD4	5.3	2.8	TEA domain family member 4 (TEAD4), transcript variant 1, mRNA	NM_003213	Hs.94865	BM423370
ACO1	5.3	3.2	aconitase 1, soluble (ACO1), mRNA	NM_002197	Hs.699191	AB209480
FAM44A	5.3	2.6	family with sequence similarity 44, member A, mRNA (cDNA clone IMAGE:5503684), with apparent retained intron.	BC043603	Hs.444517	NM_148894
THSD4	5.3	5.4	thrombospondin, type I, domain containing 4 (THSD4), mRNA	NM_024817	Hs.387057	NM_024817
EHBP1L1	5.3	3.9	Signal-induced proliferation-associated protein 1 (Sipa-1) (GTPase- activating protein Spa-1) (p130 SPA-1).	ENST00000309[illegible]	Unknown	
LIX1L	5.3	2.1	Lix1 homolog (mouse)-like (LIX1L), mRNA	NM_153713	Hs.632435	AK128733
TXNRD2	5.2	15.0	thioredoxin reductase 2 (TXNRD2), nuclear gene encoding mitochondrial protein, mRNA	NM_006440	Hs.443430	AB051439
SLC43A3	5.2	4.5	solute carrier family 43, member 3 (SLC43A3), mRNA	NM_199329	Hs.99962	NM_014096
THC2722117	5.2	2.8	THC2722117	THC2722117	Unknown	
STAT1	5.2	2.7	signal transducer and activator of transcription 1, 91kDa (STAT1), transcript variant beta, mRNA	NM_139266	Hs.699271	NM_007315
TAP1	5.2	3.7	transporter 1, ATP-binding cassette, sub-family B (MDR/TAP) (TAP1), mRNA	NM_000593	Hs.352018	BX648013
CA441889	5.2	2.4	CA441889 UI-H-ED0-axl-n-03-0-UI.s1 NCI_CGAP_ED0 cDNA clone UI-H-ED0-axl-n-03-0-UI 3', mRNA sequence	CA441889	Hs.699278	NM_003831
CETP	5.2	7.0	cholesteryl ester transfer protein, plasma (CETP), mRNA	NM_000078	Hs.89538	NM_000078
SPIRE1	5.2	2.3	spire homolog 1 (Drosophila) (SPIRE1), mRNA	NM_020148	Hs.515283	AB032961
CTSC	5.2	5.4	cathepsin C (CTSC), transcript variant 2, mRNA	NM_148170	Hs.128065	BX537913

STAT1	5.2	2.7	signal transducer and activator of transcription 1, 91kDa (STAT1), transcript variant beta, mRNA	NM_139266	Hs.699271	NM_007315
NEK7	5.2	2.3	NIMA (never in mitosis gene a)-related kinase 7 (NEK7), mRNA	NM_133494	Hs.24119	BC043003
DHRS4	5.2	2.7	dehydrogenase/reductase (SDR family) member 4 (DHRS4), mRNA	NM_021004	Hs.528385	BM919053
TCP11L1	5.2	3.5	t-complex 11 (mouse)-like 1 (TCP11L1), mRNA	NM_018393	Hs.655341	BC030582
EID3	5.2	3.4	EP300 interacting inhibitor of differentiation 3 (EID3), mRNA	NM_001008394	Hs.659857	AK098698
FEM1C	5.2	2.6	fem-1 homolog c (C. elegans) (FEM1C), mRNA	NM_020177	Hs.47367	NM_020177
KLRC1	5.2	3.1	killer cell lectin-like receptor subfamily C, member 1 (KLRC1), transcript variant 2, mRNA	NM_007328	Hs.512576	BC012550
THC2616992	5.2	4.1	Q4SG10_TETNG (Q4SG10) Chromosome 7 SCAF14601, whole genome shotgun sequence, partial (61%)	THC2616992	Unknown	
AK124192	5.2	2.7	cDNA FLJ42198 fis, clone THYMU2034338.	AK124192	Hs.584833	AK124192
LOC344887	5.2	2.7	mRNA; cDNA DKFZp686B14224 (from clone DKFZp686B14224).	BX640843	Hs.128803	BX640843
CD34	5.1	38.8	CD34 molecule (CD34), transcript variant 2, mRNA	NM_001773	Hs.374990	BX640941
NR1H2	5.1	2.6	nuclear receptor subfamily 1, group H, member 2 (NR1H2), mRNA	NM_007121	Hs.432976	AK091535
KIAA2002	5.1	3.1	mRNA for KIAA2002 protein.	AB082533	Hs.9587	NM_024776
SLC7A11	5.1	7.1	solute carrier family 7, (cationic amino acid transporter, y+ system) member 11 (SLC7A11), mRNA	NM_014331	Hs.390594	NM_014331
LOC442272	5.1	2.8	PREDICTED: similar to YKT6 v-SNARE protein (LOC442272), mRNA	XR_019351	Hs.648080	XR_019351
STAT1	5.1	2.7	signal transducer and activator of transcription 1, 91kDa (STAT1), transcript variant beta, mRNA	NM_139266	Hs.699271	NM_007315
STAT1	5.1	2.8	signal transducer and activator of transcription 1, 91kDa (STAT1), transcript variant beta, mRNA	NM_139266	Hs.699271	NM_007315
DERL1	5.1	3.4	Der1-like domain family, member 1 (DERL1), mRNA	NM_024295	Hs.241576	AK023846
MDM2	5.1	5.3	Mdm2, transformed 3T3 cell double minute 2, p53 binding protein (mouse) (MDM2), transcript variant MDM2, mRNA	NM_002392	Hs.567303	M92424
A_24_P358305	5.1	3.1	A_24_P358305	A_24_P358305	Unknown	
AKAP2	5.1	3.5	A kinase (PRKA) anchor protein 2 (AKAP2), mRNA	NM_001004065	Hs.591908	NM_053016
MPDU1	5.1	3.2	mannose-P-dolichol utilization defect 1 (MPDU1), mRNA	NM_004870	Hs.632249	BM542186
HRAS	5.1	2.2	v-Ha-ras Harvey rat sarcoma viral oncogene homolog (HRAS), transcript variant 1, mRNA	NM_005343	Hs.37003	BG714207
STAM2	5.1	2.1	signal transducing adaptor molecule (SH3 domain and ITAM motif) 2 (STAM2), mRNA	NM_005843	Hs.17200	NM_005843
SGCE	5.1	2.5	sarcoglycan, epsilon (SGCE), mRNA	NM_003919	Hs.371199	NM_001099401
TPM4	5.1	2.4	tropomyosin 4 (TPM4), mRNA	NM_003290	Hs.631618	AK056565
DBNL	5.1	2.4	drebrin-like (DBNL), transcript variant 2, mRNA	NM_001014436	Hs.436500	AF218020
HRAS	5.1	2.3	v-Ha-ras Harvey rat sarcoma viral oncogene homolog (HRAS), transcript variant 1, mRNA	NM_005343	Hs.37003	BG714207
RMND1	5.1	2.0	required for meiotic nuclear division 1 homolog (S. cerevisiae) (RMND1), mRNA	NM_017909	Hs.486835	BC106065
BX379032	5.1	6.1	BX379032 PLACENTA COT 25-NORMALIZED cDNA clone CS0DI029YC06 3-PRIME, mRNA sequence	BX379032	Hs.656121	BX379032
C16orf45	5.0	1.8	chromosome 16 open reading frame 45 (C16orf45), mRNA	NM_033201	Hs.460095	AK092923
LDLR	5.0	5.2	low density lipoprotein receptor (familial hypercholesterolemia) (LDLR), mRNA	NM_000527	Hs.213289	BX648281
UNC84A	5.0	2.2	unc-84 homolog A (C. elegans) (UNC84A), mRNA	NM_025154	Hs.438072	AB018353
USP6	5.0	4.0	ubiquitin specific peptidase 6 (Tre-2 oncogene) (USP6), mRNA	NM_004505	Hs.448851	BX647719
GRB2	5.0	2.7	growth factor receptor-bound protein 2 (GRB2), transcript variant 1, mRNA	NM_002086	Hs.444356	NM_002086
TRIM7	5.0	2.8	tripartite motif-containing 7 (TRIM7), transcript variant 6, mRNA	NM_033342	Hs.487412	NM_203297
PPP1R15A	5.0	2.4	protein phosphatase 1, regulatory (inhibitor) subunit 15A (PPP1R15A), mRNA	NM_014330	Hs.631593	AK225632
THC2578949	5.0	2.4	AY918495 mitochondrial cytochrome c somatic {Macaca sylvanus} (exp=-1; wgp=0; cg=0), complete	THC2578949	Unknown	
MDM2	5.0	4.1	Mdm2, transformed 3T3 cell double minute 2, p53 binding protein (mouse) (MDM2), transcript variant MDM2, mRNA	NM_002392	Hs.567303	M92424
BQ130701	5.0	2.9	BQ130701 ij86c06.y1 Human insulinoma cDNA clone IMAGE:5778418 5' similar to SW:CTGF_HUMAN P29279 CONNECTIVE TISSUE GROWTH FACTOR PRECURSOR. ;, mRNA sequence	BQ130701	Hs.591346	NM_001901
HRAS	5.0	2.3	v-Ha-ras Harvey rat sarcoma viral oncogene homolog (HRAS), transcript variant 1, mRNA	NM_005343	Hs.37003	BG714207
HRAS	5.0	2.3	v-Ha-ras Harvey rat sarcoma viral oncogene homolog (HRAS), transcript variant 1, mRNA	NM_005343	Hs.37003	BG714207
ChGn	5.0	5.3	chondroitin beta1,4 N-acetylgalactosaminyltransferase (ChGn), mRNA	NM_018371	Hs.655166	BX649103
NFIX	5.0	2.8	nuclear factor I/X (CCAAT-binding transcription factor) (NFIX), mRNA	NM_002501	Hs.257970	NM_002501
PITPNA	5.0	2.2	phosphatidylinositol transfer protein, alpha (PITPNA), mRNA	NM_006224	Hs.429819	BC045108
ARPC4	5.0	2.0	actin related protein 2/3 complex, subunit 4, 20kDa (ARPC4), transcript variant 1, mRNA	NM_005718	Hs.323342	AK125875
CXorf38	5.0	2.5	chromosome X open reading frame 38 (CXorf38), mRNA	NM_144970	Hs.495961	AK095710
NDST1	5.0	2.7	N-deacetylase/N-sulfotransferase (heparan glucosaminyl) 1, mRNA (cDNA clone MGC:9410 IMAGE:3882074), complete cds.	BC012888	Hs.222055	NM_001543
ABHD2	4.9	2.3	abhydrolase domain containing 2 (ABHD2), transcript variant 1, mRNA	NM_007011	Hs.122337	NM_007011

UBE3C	4.9	5.7	ubiquitin protein ligase E3C (UBE3C), mRNA	NM_014671	Hs.118351	AK127280
STAT1	4.9	2.7	signal transducer and activator of transcription 1, 91kDa (STAT1), transcript variant beta, mRNA	NM_139266	Hs.699271	NM_007315
A_24_P578641	4.9	4.7	A_24_P578641	A_24_P578641	Unknown	
SAT1	4.9	2.2	spermidine/spermine N1-acetyltransferase 1 (SAT1), mRNA	NM_002970	Hs.28491	BF680536
TYW3	4.9	35.8	tRNA-yW synthesizing protein 3 homolog (S. cerevisiae) (TYW3), mRNA	NM_138467	Hs.348411	BX647591
A_24_P692030	4.9	4.5	A_24_P692030	A_24_P692030	Unknown	
FAM24B	4.9	23.4	family with sequence similarity 24, member B (FAM24B), mRNA	NM_152644	Hs.114648	AL832795
MFAP3	4.9	3.0	microfibrillar-associated protein 3 (MFAP3), mRNA	NM_005927	Hs.432818	NM_005927
SWS1	4.9	2.1	SWIM-domain containing Srs2 interacting protein 1 (SWS1), transcript variant 2, mRNA	NM_001042698	Hs.593985	NM_001042697
ALKBH3	4.9	5.2	alkB, alkylation repair homolog 3 (E. coli) (ALKBH3), mRNA	NM_139178	Hs.368920	AK125602
KLF9	4.9	3.8	Kruppel-like factor 9 (KLF9), mRNA	NM_001206	Hs.150557	NM_001206
PBEF1	4.9	3.9	pre-B-cell colony enhancing factor 1 (PBEF1), mRNA	NM_005746	Hs.489615	NM_005746
C9orf21	4.9	2.3	chromosome 9 open reading frame 21 (C9orf21), mRNA	NM_153698	Hs.44640	NM_153698
GAB3	4.9	4.6	GRB2-associated binding protein 3 (GAB3), mRNA	NM_080612	Hs.496982	NM_001081573
CRYZ	4.9	12.6	crystallin, zeta (quinone reductase) (CRYZ), mRNA	NM_001889	Hs.83114	BX649061
PPFIBP1	4.9	2.0	PTPRF interacting protein, binding protein 1 (liprin beta 1) (PPFIBP1), transcript variant 1, mRNA	NM_003622	Hs.172445	NM_003622
FAM114A1	4.9	2.8	family with sequence similarity 114, member A1 (FAM114A1), mRNA	NM_138389	Hs.476517	AK094179
BC043218	4.9	2.7	cDNA clone IMAGE:5295323.	BC043218	Hs.220558	BC043218
HRAS	4.9	2.3	v-Ha-ras Harvey rat sarcoma viral oncogene homolog (HRAS), transcript variant 1, mRNA	NM_005343	Hs.37003	BG714207
STARD13	4.9	7.3	START domain containing 13 (STARD13), transcript variant alpha, mRNA	NM_178006	Hs.507704	BX647695
TNFRSF1A	4.9	1.9	tumor necrosis factor receptor superfamily, member 1A (TNFRSF1A), mRNA	NM_001065	Hs.279594	NM_001065
HRAS	4.9	2.3	v-Ha-ras Harvey rat sarcoma viral oncogene homolog (HRAS), transcript variant 1, mRNA	NM_005343	Hs.37003	BG714207
HTR2B	4.9	6.3	5-hydroxytryptamine (serotonin) receptor 2B (HTR2B), mRNA	NM_000867	Hs.421649	BC063123
DHRS7B	4.9	2.8	dehydrogenase/reductase (SDR family) member 7B (DHRS7B), mRNA	NM_015510	Hs.386989	AK124055
RGMB	4.8	3.4	RGM domain family, member B (RGMB), transcript variant 1, mRNA	NM_001012761	Hs.526902	NM_001012761
A_23_P30411	4.8	2.6	A_23_P30411	A_23_P30411	Unknown	
THC2736113	4.8	15.5	E41LA_HUMAN (Q9HCS5) Band 4.1-like protein 4A (NBL4 protein), partial (31%)	THC2736113	Unknown	
GBP4	4.8	4.4	guanylate binding protein 4 (GBP4), mRNA	NM_052941	Hs.409925	AL832576
LOC96610	4.8	2.5	hypothetical protein similar to KIAA0187 gene product (LOC96610), mRNA	NM_080926	Unknown	
ENST00000298	4.8	2.9	UPF0317 protein C14orf159, mitochondrial precursor.	ENST00000298	Unknown	
GSTT2	4.8	2.8	glutathione S-transferase theta 2 (GSTT2), mRNA	NM_000854	Hs.654462	CD013992
STOM	4.8	4.6	stomatin (STOM), transcript variant 2, mRNA	NM_198194	Hs.253903	NM_004099
EDEM1	4.8	2.9	ER degradation enhancer, mannosidase alpha-like 1 (EDEM1), mRNA	NM_014674	Hs.224616	NM_014674
HDDC3	4.8	3.3	HD domain containing 3 (HDDC3), mRNA	NM_198527	Hs.349979	AK057584
PLEKHB2	4.8	2.1	pleckstrin homology domain containing, family B (evectins) member 2 (PLEKHB2), transcript variant 2, mRNA	NM_017958	Hs.469944	NM_001100623
CYP26B1	4.8	2.3	cytochrome P450, family 26, subfamily B, polypeptide 1 (CYP26B1), mRNA	NM_019885	Hs.91546	NM_019885
DGKA	4.8	3.5	diacylglycerol kinase, alpha 80kDa (DGKA), transcript variant 1, mRNA	NM_201444	Hs.524488	AK226134
TNFRSF1A	4.8	1.9	tumor necrosis factor receptor superfamily, member 1A (TNFRSF1A), mRNA	NM_001065	Hs.279594	NM_001065
AHNAK	4.8	3.5	AHNAK nucleoprotein (desmoyokin) (AHNAK), transcript variant 1, mRNA	NM_001620	Hs.502756	NM_001620
A_24_P120381	4.8	1.7	A_24_P120381	A_24_P120381	Unknown	
FLJ22222	4.8	2.1	hypothetical protein FLJ22222 (FLJ22222), transcript variant 1, mRNA	NM_024648	Hs.567578	BM923625
MDM2	4.8	4.3	Mdm2, transformed 3T3 cell double minute 2, p53 binding protein (mouse) (MDM2), transcript variant MDM2, mRNA	NM_002392	Hs.567303	M92424
ZADH2	4.8	1.8	zinc binding alcohol dehydrogenase, domain containing 2 (ZADH2), mRNA	NM_175907	Hs.591065	NM_175907
HRAS	4.7	2.2	v-Ha-ras Harvey rat sarcoma viral oncogene homolog (HRAS), transcript variant 1, mRNA	NM_005343	Hs.37003	BG714207
HRAS	4.7	2.3	v-Ha-ras Harvey rat sarcoma viral oncogene homolog (HRAS), transcript variant 1, mRNA	NM_005343	Hs.37003	BG714207
THC2691142	4.7	2.8	THC2691142	THC2691142	Unknown	
STAT1	4.7	2.7	signal transducer and activator of transcription 1, 91kDa (STAT1), transcript variant beta, mRNA	NM_139266	Hs.699271	NM_007315
ITGB3	4.7	10.6	integrin, beta 3 (platelet glycoprotein IIIa, antigen CD61) (ITGB3), mRNA	NM_000212	Hs.218040	NM_000212
SAMD8	4.7	2.4	sterile alpha motif domain containing 8 (SAMD8), mRNA	NM_144660	Hs.663616	BC080593
CNTNAP1	4.7	2.6	contactin associated protein 1 (CNTNAP1), mRNA	NM_003632	Hs.408730	NM_003632
THC2538882	4.7	5.3	Q5VT28_HUMAN (Q5VT28) Family with sequence similarity 27, member B (Family with sequence similarity 27, member A) (Family with sequence similarity 27, member C), partial (85%)	THC2538882	Unknown	

AK075484	4.7	2.4	cDNA PSEC0178 fis, clone OVARC1000636, moderately similar to Sterile alpha motif domain containing protein 4.	AK075484	Unknown	
ICA1	4.7	2.2	islet cell autoantigen 1, 69kDa (ICA1), transcript variant 2, mRNA	NM_004968	Hs.487561	NM_004968
GSTT2	4.7	3.2	glutathione S-transferase theta 2 (GSTT2), mRNA	NM_000854	Hs.654462	CD013992
AK127309	4.7	19.5	cDNA FLJ45377 fis, clone BRHIP3019956.	AK127309	Unknown	
BQ010172	4.7	4.4	BQ010172 UI-H-ED0-axz-j-15-0-UI.s1 NCI_CGAP_ED0 cDNA clone IMAGE:5835758 3', mRNA sequence	BQ010172	Hs.513617	NM_004530
MMP2	4.7	5.1	matrix metallopeptidase 2 (gelatinase A, 72kDa gelatinase, 72kDa type IV collagenase) (MMP2), mRNA	NM_004530	Hs.513617	NM_004530
SDHD	4.7	1.8	succinate dehydrogenase complex, subunit D, integral membrane protein (SDHD), nuclear gene encoding mitochondrial protein, mRNA	NM_003002	Hs.356270	BF240727
CLDN14	4.7	4.4	claudin 14 (CLDN14), transcript variant 1, mRNA	NM_144492	Hs.660278	NM_144492
P2RX7	4.7	4.0	purinergic receptor P2X, ligand-gated ion channel, 7 (P2RX7), mRNA	NM_002562	Hs.507102	NM_002562
A_24_P934541	4.7	2.7	A_24_P934541	A_24_P934541	Unknown	
ADAM23	4.7	2.8	ADAM metallopeptidase domain 23 (ADAM23), mRNA	NM_003812	Hs.591643	AK091800
PTRF	4.7	5.2	polymerase I and transcript release factor (PTRF), mRNA	NM_012232	Hs.437191	BC066123
THC2713242	4.7	5.3	THC2713242	THC2713242	Unknown	
ARFRP1	4.7	2.9	ADP-ribosylation factor related protein 1 (ARFRP1), mRNA	NM_003224	Hs.389277	AL833084
BNIP1	4.7	2.4	BCL2/adenovirus E1B 19kDa interacting protein 1 (BNIP1), transcript variant BNIP1-a, mRNA	NM_013978	Hs.145726	NM_013979
TMEM62	4.7	2.2	transmembrane protein 62 (TMEM62), mRNA	NM_024956	Hs.511175	NM_024956
GGTLA1	4.7	4.9	gamma-glutamyltransferase-like activity 1 (GGTLA1), mRNA	NM_004121	Hs.437156	NM_001099781
SQRDL	4.7	3.1	sulfide quinone reductase-like (yeast) (SQRDL), mRNA	NM_021199	Hs.511251	AK130140
WARS	4.7	2.7	tryptophanyl-tRNA synthetase (WARS), transcript variant 1, mRNA	NM_004184	Hs.497599	NM_004184
ACADSB	4.7	3.2	acyl-Coenzyme A dehydrogenase, short/branched chain (ACADSB), nuclear gene encoding mitochondrial protein, mRNA	NM_001609	Hs.81934	NM_001609
TRIM4	4.7	86.1	tripartite motif-containing 4 (TRIM4), transcript variant alpha, mRNA	NM_033017	Hs.50749	AF220023
GMPR	4.6	2.9	guanosine monophosphate reductase (GMPR), mRNA	NM_006877	Hs.484741	BC008281
A_24_P66908	4.6	2.5	A_24_P66908	A_24_P66908	Unknown	
C6orf128	4.6	4.4	chromosome 6 open reading frame 128 (C6orf128), mRNA	NM_145316	Hs.520101	NM_145316
TBC1D20	4.6	2.3	TBC1 domain family, member 20 (TBC1D20), mRNA	NM_144628	Hs.590876	BX647370
TTBK2	4.6	2.4	tau tubulin kinase 2 (TTBK2), mRNA	NM_173500	Hs.659846	NM_173500
ZNF248	4.6	9.5	zinc finger protein 248 (ZNF248), mRNA	NM_021045	Hs.572001	NM_021045
AK095151	4.6	2.2	cDNA FLJ37832 fis, clone BRSSN2009630.	AK095151	Hs.591856	NM_015902
PLEKHB2	4.6	2.6	pleckstrin homology domain containing, family B (evectins) member 2 (PLEKHB2), transcript variant 1, mRNA	NM_001031706	Unknown	
MPDU1	4.6	3.8	mannose-P-dolichol utilization defect 1 (MPDU1), mRNA	NM_004870	Hs.632249	BM542186
C20orf142	4.6	4.4	chromosome 20 open reading frame 142, mRNA (cDNA clone IMAGE:4933017), with apparent retained intron.	BC029662	Unknown	
TBC1D20	4.6	2.5	TBC1 domain family, member 20, mRNA (cDNA clone MGC:23197 IMAGE:4861869), complete cds.	BC014983	Unknown	
CCDC125	4.6	14.6	coiled-coil domain containing 125 (CCDC125), mRNA	NM_176816	Hs.654850	AL832658
TRIM35	4.6	1.9	tripartite motif-containing 35 (TRIM35), transcript variant 2, mRNA	NM_171982	Hs.104223	NM_171982
TMCO7	4.6	2.7	mRNA for KIAA1746 protein, partial cds.	AB051533	Hs.13526	NM_024562
BBC3	4.6	3.1	BCL2 binding component 3 (BBC3), mRNA	NM_014417	Hs.467020	AF332558
FBXW2	4.6	2.0	F-box and WD-40 domain protein 2 (FBXW2), mRNA	NM_012164	Hs.494985	NM_012164
MMP2	4.6	4.7	matrix metallopeptidase 2 (gelatinase A, 72kDa gelatinase, 72kDa type IV collagenase) (MMP2), mRNA	NM_004530	Hs.513617	NM_004530
C1orf144	4.6	2.1	chromosome 1 open reading frame 144 (C1orf144), mRNA	NM_015609	Hs.252967	AJ295987
ACSS1	4.6	2.9	acyl-CoA synthetase short-chain family member 1 (ACSS1), nuclear gene encoding mitochondrial protein, mRNA	NM_032501	Hs.529353	AK125058
LOC285074	4.6	2.4	hypothetical protein LOC285074 (LOC285074), mRNA	NM_001012626	Unknown	
DERL1	4.6	2.7	Der1-like domain family, member 1 (DERL1), mRNA	NM_024295	Hs.241576	AK023846
MRPL49	4.6	3.0	mitochondrial ribosomal protein L49 (MRPL49), nuclear gene encoding mitochondrial protein, mRNA	NM_004927	Hs.75859	AB209160
THC2717907	4.6	1.8	Q4IZP3_AZOVI (Q4IZP3) Outer membrane porin, partial (3%)	THC2717907	Unknown	
MMP2	4.6	4.8	matrix metallopeptidase 2 (gelatinase A, 72kDa gelatinase, 72kDa type IV collagenase) (MMP2), mRNA	NM_004530	Hs.513617	NM_004530
HIST1H2AA	4.6	3.5	histone cluster 1, H2aa (HIST1H2AA), mRNA	NM_170745	Hs.406739	BU567795
USP4	4.6	2.6	ubiquitin specific peptidase 4 (proto-oncogene) (USP4), transcript variant 1, mRNA	NM_003363	Hs.631919	NM_003363
LOC441893	4.6	2.4	PREDICTED: similar to Glyceraldehyde-3-phosphate dehydrogenase (GAPDH) (LOC441893), mRNA	XR_018352	Hs.693430	XR_018352
POLH	4.6	4.2	polymerase (DNA directed), eta (POLH), mRNA	NM_006502	Hs.655467	AB024313
TRIOBP	4.6	2.5	TRIO and F-actin binding protein (TRIOBP), transcript variant 2, mRNA	NM_138632	Hs.533030	NM_001039141

TNFRSF1A	4.5	1.9	tumor necrosis factor receptor superfamily, member 1A (TNFRSF1A), mRNA	NM_001065	Hs.279594	NM_001065
TP53I11	4.5	3.7	tumor protein p53 inducible protein 11 (TP53I11), mRNA	NM_001076787	Hs.554791	BC045666
GOPC	4.5	2.4	golgi associated PDZ and coiled-coil motif containing (GOPC), transcript variant 1, mRNA	NM_020399	Hs.191539	AB209385
CXorf38	4.5	2.4	chromosome X open reading frame 38 (CXorf38), mRNA	NM_144970	Hs.495961	AK095710
BX415272	4.5	5.8	BX415272 THYMUS cDNA clone CS0CAP005YK02 3-PRIME, mRNA sequence	BX415272	Hs.681876	BQ278534
BC044628	4.5	4.4	mRNA similar to collagen, type XVII, alpha 1 (cDNA clone IMAGE:5431129).	BC044628	Unknown	
C6orf35	4.5	3.3	chromosome 6 open reading frame 35 (C6orf35), mRNA	NM_018452	Hs.157212	NM_018452
KLHL3	4.5	6.1	kelch-like 3 (Drosophila) (KLHL3), mRNA	NM_017415	Hs.655084	AF208070
ENTPD1	4.5	16.1	ectonucleoside triphosphate diphosphohydrolase 1 (ENTPD1), mRNA	NM_001776	Hs.576612	NM_001776
ADAMTSL1	4.5	6.5	ADAMTS-like 1 (ADAMTSL1), transcript variant 3, mRNA	NM_139264	Unknown	
C6orf194	4.5	11.4	chromosome 6 open reading frame 194 (C6orf194), mRNA	NM_001007531	Hs.239181	NM_001007531
MMP2	4.5	4.9	matrix metallopeptidase 2 (gelatinase A, 72kDa gelatinase, 72kDa type IV collagenase) (MMP2), mRNA	NM_004530	Hs.513617	NM_004530
ETS2	4.5	2.7	v-ets erythroblastosis virus E26 oncogene homolog 2 (avian) (ETS2), mRNA	NM_005239	Hs.695937	NM_005239
KCTD9	4.5	2.1	potassium channel tetramerisation domain containing 9 (KCTD9), mRNA	NM_017634	Hs.72071	AL117436
RABGGTA	4.5	2.7	Rab geranylgeranyltransferase, alpha subunit (RABGGTA), transcript variant 2, mRNA	NM_004581	Hs.377992	CR625243
IAH1	4.5	52.7	isoamyl acetate-hydrolyzing esterase 1 homolog (S. cerevisiae) (IAH1), mRNA	NM_001039613	Hs.700710	AK092982
HYPE	4.5	2.3	Huntingtin interacting protein E (HYPE), mRNA	NM_007076	Hs.661891	BC064629
ZFAND3	4.5	2.3	zinc finger, AN1-type domain 3 (ZFAND3), mRNA	NM_021943	Hs.36959	AK023284
STK16	4.5	2.5	mRNA for PKL12 protein.	AJ005791	Hs.153003	AK096954
CS	4.5	2.9	citrate synthase (CS), nuclear gene encoding mitochondrial protein, transcript variant 2, mRNA	NM_198324	Hs.430606	NM_198324
EEFSEC	4.5	2.7	eukaryotic elongation factor, selenocysteine-tRNA-specific (EEFSEC), mRNA	NM_021937	Hs.477498	NM_021937
TNFRSF1A	4.5	1.9	tumor necrosis factor receptor superfamily, member 1A (TNFRSF1A), mRNA	NM_001065	Hs.279594	NM_001065
MMP2	4.4	4.7	matrix metallopeptidase 2 (gelatinase A, 72kDa gelatinase, 72kDa type IV collagenase) (MMP2), mRNA	NM_004530	Hs.513617	NM_004530
PLEKHF1	4.4	2.3	pleckstrin homology domain containing, family F (with FYVE domain) member 1 (PLEKHF1), mRNA	NM_024310	Hs.466383	AY037145
FAHD2A	4.4	2.7	fumarylacetoacetate hydrolase domain containing 2A (FAHD2A), mRNA	NM_016044	Hs.546387	CR623858
HRAS	4.4	2.2	v-Ha-ras Harvey rat sarcoma viral oncogene homolog (HRAS), transcript variant 1, mRNA	NM_005343	Hs.37003	BG714207
ABI3BP	4.4	9.3	cDNA: FLJ21551 fis, clone COL06266.	AK025204	Hs.477015	BX648726
BTG2	4.4	2.4	BTG family, member 2 (BTG2), mRNA	NM_006763	Hs.519162	NM_006763
PPFIBP1	4.4	3.4	PTPRF interacting protein, binding protein 1 (liprin beta 1) (PPFIBP1), transcript variant 1, mRNA	NM_003622	Hs.172445	NM_003622
NFIB	4.4	3.1	nuclear factor I/B (NFIB), mRNA	NM_005596	Hs.699215	NM_005596
TUBB6	4.4	2.2	tubulin, beta 6 (TUBB6), mRNA	NM_032525	Hs.193491	AK092677
THC2665061	4.4	2.3	Q5TVY2_ANOGA (Q5TVY2) ENSANGP00000027655 (Fragment), partial (7%)	THC2665061	Unknown	
LRP10	4.4	2.7	low density lipoprotein receptor-related protein 10 (LRP10), mRNA	NM_014045	Hs.525232	NM_014045
C5orf13	4.4	2.2	chromosome 5 open reading frame 13 (C5orf13), mRNA	NM_004772	Hs.36053	CR607755
BCL2L1	4.4	2.2	BCL2-like 1 (BCL2L1), nuclear gene encoding mitochondrial protein, transcript variant 1, mRNA	NM_138578	Hs.516966	CR936637
CHKB	4.4	2.0	choline kinase beta (CHKB), transcript variant 1, mRNA	NM_005198	Hs.654827	BC037162
BAG3	4.4	2.1	BCL2-associated athanogene 3 (BAG3), mRNA	NM_004281	Hs.523309	NM_004281
ASTL	4.4	1.9	astacin-like metallo-endopeptidase (M12 family) (ASTL), mRNA	NM_001002036	Hs.447993	AJ537600
CINP	4.4	3.8	cyclin-dependent kinase 2-interacting protein (CINP), mRNA	NM_032630	Hs.129634	BX248279
TNFRSF1A	4.4	1.9	tumor necrosis factor receptor superfamily, member 1A (TNFRSF1A), mRNA	NM_001065	Hs.279594	NM_001065
MMP2	4.4	4.8	matrix metallopeptidase 2 (gelatinase A, 72kDa gelatinase, 72kDa type IV collagenase) (MMP2), mRNA	NM_004530	Hs.513617	NM_004530
ADORA2A	4.4	2.6	adenosine A2a receptor (ADORA2A), mRNA	NM_000675	Unknown	
MMP2	4.4	4.8	matrix metallopeptidase 2 (gelatinase A, 72kDa gelatinase, 72kDa type IV collagenase) (MMP2), mRNA	NM_004530	Hs.513617	NM_004530
NPR1	4.4	5.2	natriuretic peptide receptor A/guanylate cyclase A (atrionatriuretic peptide receptor A) (NPR1), mRNA	NM_000906	Hs.490330	NM_000906
DCTD	4.4	2.2	dCMP deaminase (DCTD), transcript variant 1, mRNA	NM_001012732	Hs.183850	NM_001012732
THC2697511	4.4	10.5	THC2697511	THC2697511	Unknown	
RP11-262H14.4	4.4	2.7	cDNA FLJ45890 fis, clone OCBBF3022576.	AK127789	Hs.658041	AK127789
PHLDB2	4.4	2.1	pleckstrin homology-like domain, family B, member 2 (PHLDB2), mRNA	NM_145753	Hs.477114	AL832205
DHRS7B	4.4	2.2	dehydrogenase/reductase (SDR family) member 7B (DHRS7B), mRNA	NM_015510	Hs.386989	AK124055
A_24_P910381	4.4	7.8	A_24_P910381	A_24_P910381	Unknown	
GK	4.4	2.3	glycerol kinase (GK), transcript variant 1, mRNA	NM_203391	Hs.1466	BC042421

CD302	4.4	5.1	CD302 molecule (CD302), mRNA	NM_014880	Hs.130014	AY314007
MMP2	4.4	5.1	matrix metallopeptidase 2 (gelatinase A, 72kDa gelatinase, 72kDa type IV collagenase) (MMP2), mRNA	NM_004530	Hs.513617	NM_004530
LOC134466	4.4	19.2	cDNA FLJ39217 fis, clone OCBBF2006639, moderately similar to ZINC FINGER PROTEIN 84.	AK096536	Hs.661876	AK096536
NPAS2	4.4	7.3	neuronal PAS domain protein 2 (NPAS2), mRNA	NM_002518	Hs.156832	NM_002518
SLC7A2	4.4	4.9	solute carrier family 7 (cationic amino acid transporter, y+ system), member 2 (SLC7A2), transcript variant 1, mRNA	NM_003046	Hs.448520	NM_003046
A_24_P375405	4.4	2.0	A_24_P375405	A_24_P375405	Unknown	
PLD1	4.4	3.2	cDNA FLJ34578 fis, clone KIDNE2008404, highly similar to PHOSPHOLIPASE D1 (EC 3.1.4.4).	AK091897	Hs.382865	AB209907
LOC134505	4.4	2.4	PREDICTED: similar to eukaryotic translation initiation factor 3, subunit 12 (LOC134505), mRNA	XR_018118	Hs.646675	XR_018118
ZBTB38	4.4	2.5	cDNA clone IMAGE:6168734.	BC072415	Hs.518301	NM_001080412
A_24_P392622	4.3	2.5	A_24_P392622	A_24_P392622	Unknown	
ADORA2A	4.3	4.3	adenosine A2a receptor (ADORA2A), mRNA	NM_000675	Unknown	
IDH3A	4.3	2.6	isocitrate dehydrogenase 3 (NAD+) alpha (IDH3A), nuclear gene encoding mitochondrial protein, mRNA	NM_005530	Hs.591110	AK123316
MKKS	4.3	2.6	McKusick-Kaufman syndrome (MKKS), transcript variant 2, mRNA	NM_170784	Hs.472119	NM_018848
RASSF3	4.3	2.5	Ras association (RalGDS/AF-6) domain family 3 (RASSF3), mRNA	NM_178169	Hs.655521	AY217662
ACOT9	4.3	3.2	acyl-CoA thioesterase 9 (ACOT9), transcript variant 1, mRNA	NM_001037171	Hs.298885	AK024337
HLA-DQA1	4.3	3.3	Human (clones 5, 9) MHC class II HLA-DQA1*03011 mRNA, partial cds.	L34088	Hs.387679	AB209628
AES	4.3	2.1	amino-terminal enhancer of split (AES), transcript variant 3, mRNA	NM_198970	Hs.515053	AK095154
HRAS	4.3	2.2	v-Ha-ras Harvey rat sarcoma viral oncogene homolog (HRAS), transcript variant 1, mRNA	NM_005343	Hs.37003	BG714207
RABL3	4.3	2.8	RAB, member of RAS oncogene family-like 3 (RABL3), mRNA	NM_173825	Hs.444360	NM_173825
CD274	4.3	8.1	Programmed cell death 1 ligand 1 precursor (Programmed death ligand 1) (PD-L1) (PDCD1 ligand 1) (B7 homolog 1) (B7-H1) (CD274 antigen).	ENST00000381	Unknown	
FIBP	4.3	2.1	fibroblast growth factor (acidic) intracellular binding protein (FIBP), transcript variant 2, mRNA	NM_004214	Hs.7768	BQ050043
TRIM72	4.3	2.2	tripartite motif-containing 72 (TRIM72), mRNA	NM_001008274	Hs.661859	AK131485
LOC133609	4.3	1.7	PREDICTED: similar to 60S acidic ribosomal protein P1 (LOC133609), mRNA	ENST00000297	Unknown	
GALK2	4.3	2.0	galactokinase 2 (GALK2), transcript variant 2, mRNA	NM_001001556	Hs.122006	NM_001001556
OBFC1	4.3	2.3	oligonucleotide/oligosaccharide-binding fold containing 1 (OBFC1), mRNA	NM_024928	Hs.134491	BM551311
SDHD	4.3	1.9	succinate dehydrogenase complex, subunit D, integral membrane protein (SDHD), nuclear gene encoding mitochondrial protein, mRNA	NM_003002	Hs.356270	BF240727
PRMT2	4.3	1.9	protein arginine methyltransferase 2 (PRMT2), transcript variant 1, mRNA	NM_206962	Hs.154163	AK123352
TRIM47	4.3	5.1	tripartite motif-containing 47 (TRIM47), mRNA	NM_033452	Hs.293660	AY026763
ACOX1	4.3	2.4	acyl-Coenzyme A oxidase 1, palmitoyl (ACOX1), transcript variant 1, mRNA	NM_004035	Hs.464137	NM_004035
DNAJC18	4.3	2.0	DnaJ (Hsp40) homolog, subfamily C, member 18 (DNAJC18), mRNA	NM_152686	Hs.483537	BC030162
URM1	4.3	3.3	ubiquitin related modifier 1 homolog (S. cerevisiae) (URM1), mRNA	NM_030914	Hs.495229	BC011620
TNFRSF1A	4.3	1.9	tumor necrosis factor receptor superfamily, member 1A (TNFRSF1A), mRNA	NM_001065	Hs.279594	NM_001065
TXNL6	4.3	2.5	thioredoxin-like 6 (TXNL6), mRNA	NM_138454	Hs.661836	BM460222
ADRBK1	4.3	3.2	adrenergic, beta, receptor kinase 1 (ADRBK1), mRNA	NM_001619	Hs.83636	NM_001619
BC012026	4.3	3.8	cDNA clone IMAGE:4476475, complete cds.	BC012026	Hs.700295	BC012026
RPS6KA3	4.3	2.2	ribosomal protein S6 kinase, 90kDa, polypeptide 3 (RPS6KA3), mRNA	NM_004586	Hs.445387	NM_004586
A_24_P632160	4.3	1.9	A_24_P632160	A_24_P632160	Unknown	
IRAK4	4.3	16.3	interleukin-1 receptor-associated kinase 4 (IRAK4), mRNA	NM_016123	Hs.138499	AK000528
MSRB3	4.3	3.0	methionine sulfoxide reductase B3 (MSRB3), transcript variant 2, mRNA	NM_001031679	Hs.339024	NM_001031679
CSK	4.3	2.3	c-src tyrosine kinase (CSK), mRNA	NM_004383	Hs.77793	BC106073
C15orf38	4.3	3.3	chromosome 15 open reading frame 38 (C15orf38), mRNA	NM_182616	Hs.6734	AL832121
IL3RA	4.3	6.7	interleukin 3 receptor, alpha (low affinity) (IL3RA), mRNA	NM_002183	Hs.632790	BC035407
ETS1	4.3	3.5	v-ets erythroblastosis virus E26 oncogene homolog 1 (avian) (ETS1), mRNA	NM_005238	Hs.369438	NM_005238
THC2558878	4.2	18.0	THC2558878	THC2558878	Unknown	
LOC391367	4.2	2.9	PREDICTED: similar to Glyceraldehyde-3-phosphate dehydrogenase (GAPDH) (LOC391367), mRNA	XR_019184	Hs.646801	XR_019184
CFLAR	4.2	4.0	CASP8 and FADD-like apoptosis regulator (CFLAR), mRNA	NM_003879	Hs.390736	AB209600
CCDC134	4.2	2.0	coiled-coil domain containing 134 (CCDC134), mRNA	NM_024821	Hs.474991	BC017693
LOC731937	4.2	2.0	PREDICTED: similar to suppressor of initiator codon mutations, related sequence 1 (LOC731937), mRNA	XM_001131332	Unknown	
BC000772	4.2	2.6	cDNA clone IMAGE:3506689, partial cds.	BC000772	Hs.679410	BC000772
PRKCDBP	4.2	8.1	protein kinase C, delta binding protein (PRKCDBP), mRNA	NM_145040	Hs.434044	BQ073453
TMLHE	4.2	2.3	trimethyllysine hydroxylase, epsilon (TMLHE), mRNA	NM_018196	Hs.133321	AK001589

CHMP2A	4.2	1.7	chromatin modifying protein 2A (CHMP2A), transcript variant 1, mRNA	NM_014453	Hs.12107	BM542499
KIAA1754	4.2	2.6	KIAA1754 (KIAA1754), mRNA	NM_033397	Hs.523252	NM_033397
RXRA	4.2	2.8	retinoid X receptor, alpha (RXRA), mRNA	NM_002957	Hs.590886	AK131081
NF2	4.2	4.1	neurofibromin 2 (bilateral acoustic neuroma) (NF2), transcript variant 13, mRNA	NM_181831	Hs.187898	NM_181832
ENST00000305	4.2	4.9	Novel protein.	ENST00000305	Unknown	
MR1	4.2	4.2	major histocompatibility complex, class I-related (MR1), mRNA	NM_001531	Hs.101840	AF031469
AI051172	4.2	2.3	AI051172 oy94a06.x1 Soares_fetal_liver_spleen_1NFLS_S1 cDNA clone IMAGE:1673458 3' similar to SW:SSRG_RAT Q08013 TRANSLOCON-ASSOCIATED PROTEIN, GAMMA SUBUNIT ;, mRNA sequence	AI051172	Hs.518346	NM_007107
YPEL2	4.2	3.2	yippee-like 2 (Drosophila) (YPEL2), mRNA	NM_001005404	Hs.463613	NM_001005404
COMMD1	4.2	2.4	copper metabolism (Murr1) domain containing 1 (COMMD1), mRNA	NM_152516	Hs.468702	BQ054214
AA627222	4.2	9.5	AA627222 nq63b01.s1 NCI_CGAP_Ov6 cDNA clone IMAGE:1154665 similar to gb:J04456 GALECTIN-1 (HUMAN);, mRNA sequence	AA627222	Hs.445351	BF570935
THC2648791	4.2	2.7	THC2648791	THC2648791	Unknown	
PSMB8	4.2	4.5	proteasome (prosome, macropain) subunit, beta type, 8 (large multifunctional peptidase 7) (PSMB8), transcript variant 1, mRNA	NM_004159	Hs.180062	NM_004159
GIPR	4.2	1.9	gastric inhibitory polypeptide receptor (GIPR), mRNA	NM_000164	Hs.658534	U39231
A_24_P237820	4.2	2.7	A_24_P237820	A_24_P237820	Unknown	
TNS1	4.2	4.2	tensin 1 (TNS1), mRNA	NM_022648	Unknown	
FXN	4.2	2.7	frataxin (FXN), nuclear gene encoding mitochondrial protein, transcript variant 2, mRNA	NM_181425	Hs.29978	NM_181425
ASAHL	4.2	3.5	N-acylsphingosine amidohydrolase (acid ceramidase)-like (ASAHL), transcript variant 2, mRNA	NM_001042402	Hs.699178	NM_014435
NSDHL	4.2	3.1	NAD(P) dependent steroid dehydrogenase-like (NSDHL), mRNA	NM_015922	Hs.57698	AK026549
LOC648269	4.2	2.4	PREDICTED: hypothetical LOC648269 (LOC648269), mRNA	XR_018384	Hs.524283	XR_018384
THC2507846	4.2	1.9	THC2507846	THC2507846	Unknown	
THC2668267	4.2	2.5	Q3MAK1_ANAVT (Q3MAK1) Phosphoglucomutase/phosphomannomutase , partial (4%)	THC2668267	Unknown	
RAB6A	4.2	2.2	RAB6A, member RAS oncogene family (RAB6A), transcript variant 1, mRNA	NM_002869	Hs.503222	NM_002869
E4F1	4.2	1.8	E4F transcription factor 1, mRNA (cDNA clone IMAGE:3353716), partial cds.	BC001713	Hs.615283	BC001713
MMP2	4.2	4.7	matrix metallopeptidase 2 (gelatinase A, 72kDa gelatinase, 72kDa type IV collagenase) (MMP2), mRNA	NM_004530	Hs.513617	NM_004530
METT10D	4.2	1.9	methyltransferase 10 domain containing (METT10D), mRNA	NM_024086	Hs.632237	NM_024086
TMEM159	4.2	2.5	transmembrane protein 159 (TMEM159), mRNA	NM_020422	Hs.258212	CR596615
ACP2	4.2	2.4	acid phosphatase 2, lysosomal (ACP2), mRNA	NM_001610	Hs.532492	AK097626
KCNJ2	4.2	3.0	potassium inwardly-rectifying channel, subfamily J, member 2 (KCNJ2), mRNA	NM_000891	Hs.1547	NM_000891
TRIM25	4.2	2.5	tripartite motif-containing 25 (TRIM25), mRNA	NM_005082	Hs.528952	NM_005082
LOC649933	4.2	3.2	PREDICTED: similar to ATP-dependent RNA helicase DDX18 (DEAD box protein 18) (Myc-regulated DEAD box protein) (MrDb) (LOC649933), mRNA	XR_019180	Hs.646694	XR_019180
UBR1	4.2	2.3	ubiquitin protein ligase E3 component n-recognin 1 (UBR1), mRNA	NM_174916	Hs.591121	NM_174916
TRIM4	4.1	11.7	tripartite motif-containing 4 (TRIM4), transcript variant alpha, mRNA	NM_033017	Hs.50749	AF220023
LOC729839	4.1	4.6	PREDICTED: similar to DTW domain containing 2 (LOC729839), mRNA	XR_016021	Hs.661610	XR_016053
COX15	4.1	2.0	COX15 homolog, cytochrome c oxidase assembly protein (yeast) (COX15), nuclear gene encoding mitochondrial protein, transcript variant 1, mRNA	NM_078470	Hs.591916	BX537557
LOC344887	4.1	4.1	mRNA; cDNA DKFZp686B14224 (from clone DKFZp686B14224).	BX640843	Hs.128803	BX640843
NFIX	4.1	2.2	nuclear factor I/X (CCAAT-binding transcription factor) (NFIX), mRNA	NM_002501	Hs.257970	NM_002501
TNFRSF14	4.1	14.8	tumor necrosis factor receptor superfamily, member 14 (herpesvirus entry mediator) (TNFRSF14), mRNA	NM_003820	Hs.512898	AB208808
MIF4GD	4.1	2.4	MIF4G domain containing (MIF4GD), mRNA	NM_020679	Hs.325631	CR597523
A_32_P38806	4.1	2.6	A_32_P38806	A_32_P38806	Unknown	
PTDSR	4.1	2.3	phosphatidylserine receptor (PTDSR), mRNA	NM_015167	Hs.514505	NM_001081461
LOC96610	4.1	2.2	hypothetical protein similar to KIAA0187 gene product (LOC96610), mRNA	NM_080926	Unknown	
NLRC5	4.1	3.1	NLR family, CARD domain containing 5 (NLRC5), mRNA	NM_032206	Hs.528836	NM_032206
NCOA3	4.1	2.1	nuclear receptor coactivator 3 (NCOA3), transcript variant 1, mRNA	NM_181659	Hs.700663	NM_181659
C20orf44	4.1	2.9	chromosome 20 open reading frame 44 (C20orf44), transcript variant 1, mRNA	NM_018244	Hs.18128	AB209400
BX091259	4.1	2.8	BX091259 BX091259 Soares fetal liver spleen 1NFLS cDNA clone IMAGp998M13529 ; IMAGE:245556, mRNA sequence	BX091259	Unknown	
C6orf148	4.1	2.4	chromosome 6 open reading frame 148 (C6orf148), mRNA	NM_030568	Hs.433062	AK090984
MMP2	4.1	4.6	matrix metallopeptidase 2 (gelatinase A, 72kDa gelatinase, 72kDa type IV collagenase) (MMP2), mRNA	NM_004530	Hs.513617	NM_004530
CFH	4.1	3.6	complement factor H (CFH), transcript variant 1, mRNA	NM_000186	Hs.363396	AK226113
LY6K	4.1	8.0	lymphocyte antigen 6 complex, locus K (LY6K), mRNA	NM_017527	Hs.699597	AK092545
ARPC4	4.1	2.0	actin related protein 2/3 complex, subunit 4, 20kDa (ARPC4), transcript variant 1, mRNA	NM_005718	Hs.323342	AK125875

PAFAH1B2	4.1	2.4	platelet-activating factor acetylhydrolase, isoform Ib, beta subunit 30kDa (PAFAH1B2), mRNA	NM_002572	Hs.696131	BC021287
GRPEL1	4.1	2.9	GrpE-like 1, mitochondrial (E. coli) (GRPEL1), mRNA	NM_025196	Hs.443723	AK098475
YPEL2	4.1	2.9	yippee-like 2 (Drosophila) (YPEL2), mRNA	NM_001005404	Hs.463613	NM_001005404
TCL6	4.1	3.2	T-cell leukemia/lymphoma 6 (TCL6), transcript variant TCL6a1, mRNA	NM_012468	Hs.510368	AB035333
THPO	4.1	2.1	thrombopoietin (myeloproliferative leukemia virus oncogene ligand, megakaryocyte growth and development factor) (THPO), mRNA	NM_000460	Hs.1166	NM_000460
TPM3	4.1	3.5	tropomyosin 3 (TPM3), transcript variant 4, mRNA	NM_001043351	Hs.699198	NM_152263
GNRHR	4.1	2.1	gonadotropin-releasing hormone receptor (GNRHR), transcript variant 1, mRNA	NM_000406	Hs.407587	NM_000406
OAF	4.1	2.1	OAF homolog (Drosophila) (OAF), mRNA	NM_178507	Hs.445081	BC047726
A_32_P151470	4.1	3.5	A_32_P151470	A_32_P151470	Unknown	
TNFRSF1A	4.1	1.9	tumor necrosis factor receptor superfamily, member 1A (TNFRSF1A), mRNA	NM_001065	Hs.279594	NM_001065
PTP4A1	4.1	2.2	protein tyrosine phosphatase type IVA, member 1 (PTP4A1), mRNA	NM_003463	Hs.227777	NM_003463
A_24_P272653	4.1	3.0	A_24_P272653	A_24_P272653	Unknown	
LYSMD2	4.1	2.6	LysM, putative peptidoglycan-binding, domain containing 2 (LYSMD2), mRNA	NM_153374	Hs.631693	BC033515
LOC116236	4.1	2.1	PPWG6510.	ENST000003072	Unknown	
CTA-126B4.3	4.0	2.9	CGI-96 protein (CGI-96), mRNA	NM_015703	Hs.534041	BC073834
OSGIN1	4.0	4.6	oxidative stress induced growth inhibitor 1 (OSGIN1), transcript variant 1, mRNA	NM_013370	Hs.128055	NM_013370
SIN3B	4.0	3.4	SIN3 homolog B, transcription regulator (yeast), mRNA (cDNA clone IMAGE:3923074), partial cds.	BC025026	Hs.13999	AY706204
A_24_P272523	4.0	2.0	A_24_P272523	A_24_P272523	Unknown	
TM4SF19	4.0	4.7	transmembrane 4 L six family member 19 (TM4SF19), mRNA	NM_138461	Hs.135997	BM810125
TNFRSF1A	4.0	2.0	tumor necrosis factor receptor superfamily, member 1A (TNFRSF1A), mRNA	NM_001065	Hs.279594	NM_001065
AF334588	4.0	2.5	P25 mRNA, complete cds.	AF334588	Unknown	
MDM2	4.0	9.3	Mdm2, transformed 3T3 cell double minute 2, p53 binding protein (mouse) (MDM2), transcript variant MDM2, mRNA	NM_002392	Hs.567303	M92424
FAM98C	4.0	2.3	family with sequence similarity 98, member C (FAM98C), mRNA	NM_174905	Hs.355162	BC036482
TAPBP	4.0	2.0	TAP binding protein (tapasin) (TAPBP), transcript variant 3, mRNA	NM_172209	Hs.370937	NM_003190
MRPL46	4.0	2.6	mitochondrial ribosomal protein L46 (MRPL46), nuclear gene encoding mitochondrial protein, mRNA	NM_022163	Hs.534261	BI913482
M-RIP	4.0	2.0	myosin phosphatase-Rho interacting protein (M-RIP), transcript variant 1, mRNA	NM_015134	Hs.462341	AB020671
CD55	4.0	2.5	CD55 molecule, decay accelerating factor for complement (Cromer blood group) (CD55), mRNA	NM_000574	Hs.527653	NM_000574
EIF5A2	4.0	3.0	eukaryotic translation initiation factor 5A2 (EIF5A2), mRNA	NM_020390	Hs.164144	AY205261
SLC9A7	4.0	2.5	solute carrier family 9 (sodium/hydrogen exchanger), member 7 (SLC9A7), mRNA	NM_032591	Hs.496057	BC094742
NFIB	4.0	3.1	nuclear factor I/B (NFIB), mRNA	NM_005596	Hs.699215	NM_005596
SKIP	4.0	2.5	skeletal muscle and kidney enriched inositol phosphatase (SKIP), transcript variant 2, mRNA	NM_130766	Hs.632238	CR623148
IRF2	4.0	2.6	interferon regulatory factor 2 (IRF2), mRNA	NM_002199	Hs.654566	BX648934
NFKB1	4.0	2.0	nuclear factor of kappa light polypeptide gene enhancer in B-cells 1 (p105) (NFKB1), mRNA	NM_003998	Hs.654408	NM_003998
THC2739386	4.0	2.5	ALU8_HUMAN (P39195) Alu subfamily SX sequence contamination warning entry, partial (16%)	THC2739386	Unknown	
PGGT1B	4.0	2.5	protein geranylgeranyltransferase type I, beta subunit (PGGT1B), mRNA	NM_005023	Hs.254006	NM_005023
DHRS7B	4.0	2.1	dehydrogenase/reductase (SDR family) member 7B (DHRS7B), mRNA	NM_015510	Hs.386989	AK124055
NFKBIB	4.0	1.9	nuclear factor of kappa light polypeptide gene enhancer in B-cells inhibitor, beta (NFKBIB), transcript variant 2, mRNA	NM_001001716	Hs.9731	NM_001001716
A_24_P84608	4.0	2.2	A_24_P84608	A_24_P84608	Unknown	
GMPPB	4.0	2.5	GDP-mannose pyrophosphorylase B (GMPPB), transcript variant 2, mRNA	NM_021971	Hs.567488	AK097846
MICA	4.0	2.1	MHC class I polypeptide-related sequence A (MICA), mRNA	NM_000247	Hs.549053	AK094237
MGC50559	4.0	3.3	hypothetical protein MGC50559 (MGC50559), mRNA	NM_173802	Hs.585084	AL832339
MGC15523	4.0	2.5	hypothetical protein MGC15523 (MGC15523), transcript variant 2, mRNA	NM_138570	Hs.352240	NM_001037984
FCRLB	4.0	3.3	Fc receptor-like B (FCRLB), mRNA	NM_001002901	Hs.517422	BC067080
TNFRSF1A	4.0	1.8	tumor necrosis factor receptor superfamily, member 1A (TNFRSF1A), mRNA	NM_001065	Hs.279594	NM_001065
MGC33302	4.0	2.0	hypothetical protein MGC33302 (MGC33302), mRNA	NM_152778	Hs.480701	BC029503
FRMD5	4.0	4.3	FERM domain containing 5 (FRMD5), transcript variant 2, mRNA	NM_032892	Hs.578544	BC007796
GNA14	4.0	5.2	Guanine nucleotide-binding protein alpha-14 subunit (G-protein alpha subunit 14).	ENST000003411	Unknown	
PRO0478	4.0	3.6	clone HQ0478 PRO0478 mRNA, complete cds.	AF090930	Hs.612035	AF090930
FAM98A	4.0	1.9	family with sequence similarity 98, member A (FAM98A), mRNA	NM_015475	Hs.468140	AK096187
MTO1	4.0	2.1	mitochondrial translation optimization 1 homolog (S. cerevisiae) (MTO1), transcript variant 2, mRNA	NM_012123	Hs.347614	AY078986
LNPEP	4.0	2.3	leucyl/cystinyl aminopeptidase (LNPEP), transcript variant 1, mRNA	NM_005575	Hs.656905	AB208883

CFLAR	4.0	5.3	FLAME-1 mRNA, complete cds.	AF009616	Hs.390736	AB209600
RPS14	4.0	3.6	40S ribosomal protein S14.	ENST00000312(	Unknown	
ARL10	4.0	2.3	ADP-ribosylation factor-like 10 (ARL10), mRNA	NM_173664	Hs.424552	NM_173664
A_24_P912985	4.0	8.5	A_24_P912985	A_24_P912985	Unknown	
PMS2L3	4.0	1.5	postmeiotic segregation increased 2-like 3 (PMS2L3), transcript variant 1, mRNA	NM_005395	Hs.659871	CR621744
IL3RA	4.0	11.7	interleukin 3 receptor, alpha (low affinity) (IL3RA), mRNA	NM_002183	Hs.632790	BC035407
BC032091	4.0	2.8	cDNA clone IMAGE:3874046.	BC032091	Unknown	
SERPINB8	4.0	3.3	serpin peptidase inhibitor, clade B (ovalbumin), member 8 (SERPINB8), transcript variant 2, mRNA	NM_198833	Hs.368077	NM_198833
ANGPT2	4.0	14.6	angiopoietin 2 (ANGPT2), mRNA	NM_001147	Hs.583870	AK225698
PRKCDBP	4.0	8.0	protein kinase C, delta binding protein (PRKCDBP), mRNA	NM_145040	Hs.434044	BQ073453
NFIC	4.0	3.9	nuclear factor I/C (CCAAT-binding transcription factor) (NFIC), transcript variant 1, mRNA	NM_005597	Hs.170131	NM_205843
THC2631475	4.0	2.2	BC011812 DNAJB12 protein {Homo sapiens} (exp=-1; wgp=0; cg=0), partial (42%)	THC2631475	Unknown	
LOC442421	4.0	1.9	similar to prostaglandin E receptor 4, subtype EP4; PGE receptor, EP4 subtype; prostaglandin E2 receptor, mRNA (cDNA clone IMAGE:4830816), partial cds.	BC042539	Hs.585349	BC068605
SDHD	4.0	1.7	succinate dehydrogenase complex, subunit D, integral membrane protein (SDHD), nuclear gene encoding mitochondrial protein, mRNA	NM_003002	Hs.356270	BF240727
LOC401859	4.0	2.2	clone IMAGE:5167766, mRNA.	BC043527	Hs.39557	BC043527
MAPRE1	4.0	1.7	microtubule-associated protein, RP/EB family, member 1 (MAPRE1), mRNA	NM_012325	Hs.472437	BC106068
CDGAP	4.0	3.1	Cdc42 GTPase-activating protein (CDGAP), mRNA	NM_020754	Hs.657263	AB033030
LOC541471	4.0	2.0	cDNA FLJ10934 fis, clone OVARC1000640.	AK001796	Hs.652426	AK001796
DIRAS3	4.0	3.9	DIRAS family, GTP-binding RAS-like 3 (DIRAS3), mRNA	NM_004675	Hs.194695	AK096393
DBNL	3.9	2.3	drebrin-like (DBNL), transcript variant 1, mRNA	NM_014063	Hs.436500	AF218020
ENST0000029	3.9	26.6	MGC9913 protein.	ENST00000299	Unknown	
March3	3.9	3.5	membrane-associated ring finger (C3HC4) 3 (MARCH3), mRNA	NM_178450	Hs.132441	BC047569
DB363693	3.9	2.3	DB363693 NT2RP2 cDNA clone NT2RP2000215 3', mRNA sequence	DB363693	Hs.701607	DB363693
S79672	3.9	2.6	EWS/WT1 fusion protein (EWS1/WT1) mRNA, partial cds.	S79672	Unknown	
GGTLA1	3.9	9.1	gamma-glutamyltransferase-like activity 1 (GGTLA1), mRNA	NM_004121	Hs.437156	NM_001099781
MRPL15	3.9	1.8	mitochondrial ribosomal protein L15 (MRPL15), nuclear gene encoding mitochondrial protein, mRNA	NM_014175	Hs.18349	BQ278804
LOC729768	3.9	2.0	PREDICTED: similar to signal sequence receptor gamma subunit (LOC729768), mRNA	XR_016019	Hs.693431	XR_016076
ISG20	3.9	2.8	interferon stimulated exonuclease gene 20kDa (ISG20), mRNA	NM_002201	Hs.459265	AB209639
AF119881	3.9	2.6	PRO2435 mRNA, complete cds.	AF119881	Unknown	
