# Supplementary material for: Transcriptional and Functional Profiling of Human Embryonic Stem Cell-Derived Cardiomyocytes
Source: PLoS One. 2008 Oct 22;3(10):e3474. doi: 10.1371/journal.pone.0003474 (PMC2565131; doi:10.1371/journal.pone.0003474)
Supplement: Table S2 — Gene Ontology analysis of differentially-regulated genes. (0.30 MB PDF) [file pone.0003474.s011.pdf]

Supplemental Table A7

GO Biological Process Categories Overrepresented in ES Cells vs beating EB

GO CATEGORY	TOTAL GENES	OVEREXPRESSED GENES	LOG10(p)	FALSE DISCOVERY RATE
GO:0022403_cell_cycle_phase	516	74	-6.085431	0
GO:0000279_M_phase	405	61	-5.814436	0
GO:0000278_mitotic_cell_cycle	446	65	-5.652681	0
GO:0000087_M_phase_of_mitotic_cell_cycle	288	47	-5.564568	0
GO:0007067_mitosis	282	46	-5.453795	0.004
GO:0009113_purine_base_biosynthetic_process	4	4	-4.367838	0.013333
GO:0006267_pre-replicative_complex_formation	7	5	-4.1989	0.017143
GO:0051301_cell_division	337	48	-4.076262	0.01625
GO:0006268_DNA_unwinding_during_replication	21	8	-3.849652	0.018182
GO:0032392_DNA_geometric_change	21	8	-3.849652	0.018182
GO:0032508_DNA_duplex_unwinding	21	8	-3.849652	0.018182
GO:0031503_protein_complex_localization	5	4	-3.697868	0.034167
GO:0022613_ribonucleoprotein_complex_biogenesis_and_assembly	304	43	-3.646781	0.033077
GO:0006541_glutamine_metabolic_process	18	7	-3.491836	0.042857
GO:0016072_rRNA_metabolic_process	115	21	-3.478833	0.044667
GO:0006364_rRNA_processing	109	20	-3.369302	0.0475
GO:0007049_cell_cycle	1106	120	-3.338727	0.045882
GO:0042254_ribosome_biogenesis_and_assembly	186	29	-3.327582	0.044444
GO:0001676_long-chain_fatty_acid_metabolic_process	19	7	-3.323557	0.045789
GO:0015994_chlorophyll_metabolic_process	3	3	-3.275317	0.0615
GO:0006188_IMP_biosynthetic_process	6	4	-3.249662	0.061304
GO:0006189_'de_novo'_IMP_biosynthetic_process	6	4	-3.249662	0.061304
GO:0046040_IMP_metabolic_process	6	4	-3.249662	0.061304
GO:0007431_salivary_gland_development	44	11	-3.226309	0.059167
GO:0046112_nucleobase_biosynthetic_process	10	5	-3.209821	0.0608
GO:0045132_meiotic_chromosome_segregation	26	8	-3.123112	0.065769
GO:0006399_tRNA_metabolic_process	139	23	-3.117628	0.063704
GO:0022402_cell_cycle_process	1006	109	-3.053818	0.063571
GO:0009156_ribonucleoside_monophosphate_biosynthetic_process	22	7	-2.887666	0.088621
GO:0006270_DNA_replication_initiation	48	11	-2.886943	0.086333
GO:0006520_amino_acid_metabolic_process	362	46	-2.829489	0.087097
GO:0002504_antigen_processing_and_presentation_of_peptide_or_polysaccharide_antigen_via_MHC_class_II	23	7	-2.761175	0.098485
GO:0007076_mitotic_chromosome_condensation	23	7	-2.761175	0.098485
GO:0030261_chromosome_condensation	36	9	-2.733419	0.101176
GO:0035272_exocrine_system_development	50	11	-2.733417	0.098286
GO:0000819_sister_chromatid_segregation	66	13	-2.665934	0.123333
GO:0009127_purine_nucleoside_monophosphate_biosynthetic_process	18	6	-2.655128	0.122105
GO:0009168_purine_ribonucleoside_monophosphate_biosynthetic_process	18	6	-2.655128	0.122105
GO:0009161_ribonucleoside_monophosphate_metabolic_process	24	7	-2.642483	0.121026
GO:0046483_heterocycle_metabolic_process	143	22	-2.590199	0.13575
GO:0009112_nucleobase_metabolic_process	31	8	-2.577599	0.134146
GO:0009124_nucleoside_monophosphate_biosynthetic_process	25	7	-2.530839	0.137381
GO:0007059_chromosome_segregation	137	21	-2.478592	0.148837
GO:0043412_biopolymer_modification	1913	186	-2.472822	0.146818
GO:0019752_carboxylic_acid_metabolic_process	669	74	-2.457564	0.144444
GO:0043331_response_to_dsRNA	14	5	-2.428961	0.149348
GO:0006082_organic_acid_metabolic_process	671	74	-2.425034	0.14766
GO:0009126_purine_nucleoside_monophosphate_metabolic_process	20	6	-2.396303	0.162245

GO:0009167_purine_ribonucleoside_monophosphate_metabolic_process	20	6	-2.396303	0.162245
GO:0000070_mitotic_sister_chromatid_segregation	63	12	-2.376521	0.1636
GO:0006519_amino_acid_and_derivative_metabolic_process	464	54	-2.370305	0.161765
GO:0046653_tetrahydrofolate_metabolic_process	5	3	-2.329506	0.1925
GO:0009123_nucleoside_monophosphate_metabolic_process	27	7	-2.32619	0.190566
GO:0046148_pigment_biosynthetic_process	64	12	-2.318762	0.189259
GO:0009064_glutamine_family_amino_acid_metabolic_process	58	11	-2.206483	0.231818
GO:0015995_chlorophyll_biosynthetic_process	2	2	-2.183171	0.326964
GO:0006919_caspase_activation	43	9	-2.178109	0.323684
GO:0019538_protein_metabolic_process	3708	335	-2.084741	0.348966
GO:0009396_folic_acid_and_derivative_biosynthetic_process	11	4	-2.050507	0.397167
GO:0050910_detection_of_mechanical_stimulus_during_sensory_perception_of_sound	11	4	-2.050507	0.397167
GO:0000079_regulation_of_cyclin-dependent_protein_kinase_activity	53	10	-2.035173	0.396885
GO:0009308_amine_metabolic_process	551	60	-1.955999	0.424355
GO:0006144_purine_base_metabolic_process	18	5	-1.915628	0.435
GO:0006760_folic_acid_and_derivative_metabolic_process	18	5	-1.915628	0.435
GO:0006464_protein_modification_process	1821	172	-1.837467	0.524923
GO:0048488_synaptic_vesicle_endocytosis	26	6	-1.803785	0.55303
GO:0006260_DNA_replication	246	30	-1.803154	0.545672
GO:0006461_protein_complex_assembly	371	42	-1.774518	0.543529
GO:0042440_pigment_metabolic_process	75	12	-1.772982	0.536667
GO:0007435_salivary_gland_morphogenesis	13	4	-1.771386	0.551831
GO:0022612_gland_morphogenesis	13	4	-1.771386	0.551831
GO:0043280_positive_regulation_of_caspase_activity	50	9	-1.750742	0.558889
GO:0006471_protein_amino_acid_ADP-ribosylation	42	8	-1.746009	0.556301
GO:0008652_amino_acid_biosynthetic_process	76	12	-1.730436	0.555135
GO:0000189_nuclear_translocation_of_MAPK	3	2	-1.730123	0.649875
GO:0000290_deadenylation-dependent_decapping	3	2	-1.730123	0.649875
GO:0002053_positive_regulation_of_mesenchymal_cell_proliferation	3	2	-1.730123	0.649875
GO:0010149_senescence	3	2	-1.730123	0.649875
GO:0015886_heme_transport	3	2	-1.730123	0.649875
GO:0030913_paranodal_junction_assembly	3	2	-1.730123	0.649875
GO:0009267_cellular_response_to_starvation	27	6	-1.724637	0.648148
GO:0006379_mRNA_cleavage	20	5	-1.716664	0.649268
GO:0000050_urea_cycle	8	3	-1.661693	0.677176
GO:0001711_endodermal_cell_fate_commitment	8	3	-1.661693	0.677176
GO:0007474_imaginal_disc-derived_wing_vein_specification	8	3	-1.661693	0.677176
GO:0008033_tRNA_processing	61	10	-1.623471	0.72686
GO:0051329_interphase_of_mitotic_cell_cycle	154	20	-1.621941	0.72
GO:0044267_cellular_protein_metabolic_process	3493	311	-1.617152	0.714205
GO:0043281_regulation_of_caspase_activity	70	11	-1.612159	0.707753
GO:0006396_RNA_processing	510	54	-1.600838	0.702667
GO:0051325_interphase	155	20	-1.594966	0.698571
GO:0019748_secondary_metabolic_process	107	15	-1.594528	0.691304
GO:0009058_biosynthetic_process	1631	153	-1.587556	0.686667
GO:0007088_regulation_of_mitosis	89	13	-1.574813	0.691489
GO:0008360_regulation_of_cell_shape	80	12	-1.570227	0.674021
GO:0022603_regulation_of_anatomical_structure_morphogenesis	80	12	-1.570227	0.674021
GO:0022604_regulation_of_cell_morphogenesis	80	12	-1.570227	0.674021
GO:0050974_detection_of_mechanical_stimulus_during_sensory_perception	15	4	-1.546853	0.700816
GO:0006400_tRNA_modification	22	5	-1.544886	0.704747

Supplemental Table A8

GO Biological Process Categories Overrepresented in EB Cells vs ES (Upregulated in EB)

GO CATEGORY	TOTAL GENES	OVEREXPRESSED GENES	LOG10(p)	FALSE DISCOVERY RATE
GO:0007275_multicellular_organismal_development	2996	631	-25.339964	0
GO:0048731_system_development	2443	526	-22.52365	0
GO:0048856_anatomical_structure_development	2893	588	-19.208929	0
GO:0048513_organ_development	1901	408	-16.541504	0
GO:0032501_multicellular_organismal_process	4096	764	-15.518126	0
GO:0032502_developmental_process	3960	742	-15.422722	0
GO:0007399_nervous_system_development	1083	255	-14.787236	0
GO:0009653_anatomical_structure_morphogenesis	1708	357	-12.554367	0
GO:0009887_organ_morphogenesis	758	186	-12.47101	0
GO:0007517_muscle_development	277	82	-9.735311	0
GO:0007417_central_nervous_system_development	389	103	-8.971851	0
GO:0009790_embryonic_development	614	145	-8.562273	0
GO:0007389_pattern_specification_process	359	94	-7.976245	0
GO:0042692_muscle_cell_differentiation	102	38	-7.706968	0
GO:0001822_kidney_development	71	30	-7.667002	0
GO:0048598_embryonic_morphogenesis	251	71	-7.607313	0
GO:0001655_urogenital_system_development	79	32	-7.59966	0
GO:0007507_heart_development	216	63	-7.360846	0
GO:0050789_regulation_of_biological_process	5045	854	-7.071034	0
GO:0050794_regulation_of_cellular_process	4523	771	-6.681511	0
GO:0006355_regulation_of_transcription__DNA-dependent	2246	413	-6.61718	0
GO:0035295_tube_development	193	56	-6.534028	0
GO:0007519_striated_muscle_development	179	53	-6.525453	0
GO:0048762_mesenchymal_cell_differentiation	59	25	-6.523893	0
GO:0007420_brain_development	238	65	-6.408502	0
GO:0014031_mesenchymal_cell_development	56	24	-6.39855	0
GO:0001501_skeletal_development	293	75	-6.075275	0
GO:0045449_regulation_of_transcription	2411	435	-5.998019	0
GO:0065007_biological_regulation	5486	910	-5.885296	0
GO:0032774_RNA_biosynthetic_process	2325	420	-5.839478	0
GO:0006351_transcription__DNA-dependent	2320	419	-5.813657	0
GO:0008015_circulation	219	59	-5.667977	0.000312
GO:0019219_regulation_of_nucleobase__nucleoside__nucleotide_and_nucleic_acid_metabolic_process	2504	445	-5.385888	0.000303
GO:0030323_respiratory_tube_development	67	25	-5.305375	0.000588
GO:0022008_neurogenesis	540	117	-5.024014	0.000571
GO:0014032_neural_crest_cell_development	38	17	-5.008291	0.000556
GO:0030030_cell_projection_organization_and_biogenesis	398	91	-4.972883	0.000769
GO:0032990_cell_part_morphogenesis	398	91	-4.972883	0.000769
GO:0048858_cell_projection_morphogenesis	398	91	-4.972883	0.000769
GO:0030182_neuron_differentiation	442	99	-4.955654	0.00075
GO:0030902_hindbrain_development	54	21	-4.875482	0.000976
GO:0048699_generation_of_neurons	505	110	-4.864238	0.000952
GO:0016055_Wnt_receptor_signaling_pathway	139	40	-4.761383	0.001163
GO:0006350_transcription	2513	441	-4.745754	0.001136
GO:0014033_neural_crest_cell_differentiation	40	17	-4.647932	0.001778
GO:0030324_lung_development	64	23	-4.621236	0.001739
GO:0030326_embryonic_limb_morphogenesis	65	23	-4.49663	0.002083
GO:0035113_embryonic_appendage_morphogenesis	65	23	-4.49663	0.002083

GO:0009799_determination_of_symmetry	57	21	-4.457484	0.0022
GO:0009855_determination_of_bilateral_symmetry	57	21	-4.457484	0.0022
GO:0007368_determination_of_left_right_symmetry	49	19	-4.446645	0.002157
GO:0001656_metanephros_development	42	17	-4.318486	0.002308
GO:0048637_skeletal_muscle_development	121	35	-4.296651	0.002453
GO:0031323_regulation_of_cellular_metabolic_process	2755	475	-4.295077	0.002407
GO:0019222_regulation_of_metabolic_process	2894	496	-4.216139	0.002545
GO:0045445_myoblast_differentiation	68	23	-4.143627	0.002679
GO:0001657_ureteric_bud_development	32	14	-4.091454	0.002982
GO:0048741_skeletal_muscle_fiber_development	101	30	-4.000559	0.00322
GO:0048747_muscle_fiber_development	101	30	-4.000559	0.00322
GO:0016477_cell_migration	451	96	-3.925526	0.0035
GO:0006928_cell_motility	618	125	-3.886151	0.00371
GO:0051674_localization_of_cell	618	125	-3.886151	0.00371
GO:0035108_limb_morphogenesis	76	24	-3.765911	0.004603
GO:0048666_neuron_development	344	76	-3.747004	0.004531
GO:0000902_cell_morphogenesis	820	158	-3.65655	0.005455
GO:0032989_cellular_structure_morphogenesis	820	158	-3.65655	0.005455
GO:0001755_neural_crest_cell_migration	31	13	-3.612658	0.00597
GO:0000904_cellular_morphogenesis_during_differentiation	320	71	-3.598191	0.005882
GO:0051146_striated_muscle_cell_differentiation	43	16	-3.590336	0.006087
GO:0031175_neurite_development	310	69	-3.555944	0.006857
GO:0051145_smooth_muscle_cell_differentiation	11	7	-3.523912	0.007465
GO:0060048_cardiac_muscle_contraction	14	8	-3.520054	0.007361
GO:0003007_heart_morphogenesis	36	14	-3.438756	0.008356
GO:0016202_regulation_of_striated_muscle_development	21	10	-3.429104	0.008649
GO:0048519_negative_regulation_of_biological_process	1615	286	-3.393822	0.0108
GO:0003002_regionalization	254	58	-3.378484	0.010658
GO:0043583_ear_development	76	23	-3.334322	0.011818
GO:0001974_blood_vessel_remodeling	18	9	-3.329613	0.01175
GO:0042384_cilium_biogenesis	18	9	-3.329613	0.01175
GO:0048706_embryonic_skeletal_development	18	9	-3.329613	0.01175
GO:0035107_appendage_morphogenesis	110	30	-3.287428	0.013415
GO:0048736_appendage_development	110	30	-3.287428	0.013415
GO:0048523_negative_regulation_of_cellular_process	1522	270	-3.271638	0.013614
GO:0048704_embryonic_skeletal_morphogenesis	15	8	-3.249535	0.014286
GO:0045446_endothelial_cell_differentiation	22	10	-3.227522	0.014824
GO:0060047_heart_contraction	82	24	-3.217882	0.016163
GO:0048667_neuron_morphogenesis_during_differentiation	275	61	-3.174272	0.016477
GO:0048812_neurite_morphogenesis	275	61	-3.174272	0.016477
GO:0007167_enzyme_linked_receptor_protein_signaling_pathway	396	82	-3.056798	0.019213
GO:0007223_Wnt_receptor_signaling_pathway__calcium_modulating_pathway	23	10	-3.041291	0.019111
GO:0007411_axon_guidance	134	34	-3.024812	0.019341
GO:0045786_negative_regulation_of_progression_through_cell_cycle	219	50	-2.994671	0.021304
GO:0007409_axonogenesis	252	56	-2.983066	0.021183
GO:0048627_myoblast_development	48	16	-2.978652	0.021064
GO:0048839_inner_ear_development	71	21	-2.951788	0.021263
GO:0048103_somatic_stem_cell_division	7	5	-2.935596	0.023333
GO:0048701_embryonic_cranial_skeleton_morphogenesis	10	6	-2.885336	0.027347
GO:0048738_cardiac_muscle_development	10	6	-2.885336	0.027347
GO:0048705_skeletal_morphogenesis	24	10	-2.868654	0.028182

Supplemental Table A9

GO Biological Process Categories Underrepresented in CM Cells vs EB (Downregulated in CM)

GO CATEGORY	TOTAL GENES	UNDEREXPRESSED GENES	LOG10(p)	FALSE DISCOVERY RATE
GO:0006355_regulation_of_transcription__DNA-dependent	2246	275	-21.248489	0
GO:0032774_RNA_biosynthetic_process	2325	280	-20.601366	0
GO:0006351_transcription__DNA-dependent	2320	279	-20.418866	0
GO:0016070_RNA_metabolic_process	2861	323	-19.671188	0
GO:0019219_regulation_of_nucleobase__nucleoside__nucleotide_and_nucleic_acid_metabolic_process	2504	292	-19.471564	0
GO:0045449_regulation_of_transcription	2411	282	-18.859206	0
GO:0006350_transcription	2513	289	-18.317278	0
GO:0031323_regulation_of_cellular_metabolic_process	2755	306	-17.290765	0
GO:0019222_regulation_of_metabolic_process	2894	312	-15.74486	0
GO:0006139_nucleobase__nucleoside__nucleotide_and_nucleic_acid_metabolic_process	3801	374	-13.062261	0
GO:0043283_biopolymer_metabolic_process	4991	448	-9.615785	0
GO:0043170_macromolecule_metabolic_process	6555	561	-9.541731	0
GO:0050794_regulation_of_cellular_process	4523	409	-8.86746	0
GO:0050789_regulation_of_biological_process	5045	433	-6.380177	0
GO:0044238_primary_metabolic_process	7473	606	-6.243147	0
GO:0007420_brain_development	238	39	-6.056432	0
GO:0065007_biological_regulation	5486	458	-5.326315	0.001176
GO:0006366_transcription_from_RNA_polymerase_II_promoter	865	96	-5.090228	0.001111
GO:0044237_cellular_metabolic_process	7508	599	-4.895143	0.001053
GO:0007417_central_nervous_system_development	389	51	-4.749914	0.0015
GO:0006357_regulation_of_transcription_from_RNA_polymerase_II_promoter	670	76	-4.446037	0.002381
GO:0016071_mRNA_metabolic_process	339	45	-4.392472	0.002273
GO:0006325_establishment_and_or_maintenance_of_chromatin_architecture	383	49	-4.309555	0.002174
GO:0048762_mesenchymal_cell_differentiation	59	14	-4.273715	0.0025
GO:0006323_DNA_packaging	389	49	-4.137525	0.0032
GO:0008152_metabolic_process	8100	634	-4.102362	0.003077
GO:0014031_mesenchymal_cell_development	56	13	-3.904878	0.007407
GO:0007131_meiotic_recombination	39	10	-3.501692	0.017143
GO:0009834_cellulose_and_pectin-containing_secondary_cell_wall_biogenesis	3	3	-3.437496	0.026667
GO:0048025_negative_regulation_of_nuclear_mRNA_splicing__via_spliceosome	3	3	-3.437496	0.026667
GO:0006397_mRNA_processing	289	37	-3.404761	0.029032
GO:0001822_kidney_development	71	14	-3.365137	0.028438
GO:0016568_chromatin_modification	250	33	-3.335945	0.028182
GO:0051276_chromosome_organization_and_biogenesis	567	62	-3.297204	0.028235
GO:0007001_chromosome_organization_and_biogenesis_(sensu_Eukaryota)	547	60	-3.242992	0.028571
GO:0007399_nervous_system_development	1083	105	-3.157588	0.034444
GO:0045934_negative_regulation_of_nucleobase__nucleoside__nucleotide_and_nucleic_acid_metabolic_process	463	52	-3.115748	0.04
GO:0001655_urogenital_system_development	79	14	-2.883454	0.053947
GO:0009832_cellulose_and_pectin-containing_cell_wall_biogenesis	4	3	-2.859317	0.060465
GO:0030243_cellulose_metabolic_process	4	3	-2.859317	0.060465
GO:0030244_cellulose_biosynthetic_process	4	3	-2.859317	0.060465
GO:0050686_negative_regulation_of_mRNA_processing	4	3	-2.859317	0.060465
GO:0051253_negative_regulation_of_RNA_metabolic_process	4	3	-2.859317	0.060465
GO:0009887_organ_morphogenesis	758	76	-2.804106	0.072727
GO:0031324_negative_regulation_of_cellular_metabolic_process	538	57	-2.7546	0.075111
GO:0006261_DNA-dependent_DNA_replication	140	20	-2.64349	0.093261
GO:0045893_positive_regulation_of_transcription__DNA-dependent	341	39	-2.616292	0.094681
GO:0001656_metanephros_development	42	9	-2.609064	0.099792

GO:0016481_negative_regulation_of_transcription	421	46	-2.58347	0.099796
GO:0042475_odontogenesis_(sensu_Vertebrata)	35	8	-2.558956	0.1002
GO:0009954_proximal_distal_pattern_formation	21	6	-2.550078	0.09902
GO:0007507_heart_development	216	27	-2.49357	0.105577
GO:0009664_cellulose_and_pectin-containing_cell_wall_organization_and_biogenesis	5	3	-2.485166	0.120556
GO:0043570_maintenance_of_DNA_repeat_elements	5	3	-2.485166	0.120556
GO:0042476_odontogenesis	44	9	-2.463484	0.120364
GO:0048732_gland_development	116	17	-2.444952	0.119464
GO:0007127_meiosis_I	70	12	-2.424974	0.121228
GO:0045941_positive_regulation_of_transcription	418	45	-2.417964	0.119483
GO:0001568_blood_vessel_development	273	32	-2.406814	0.127288
GO:0030902_hindbrain_development	54	10	-2.349694	0.1355
GO:0001944_vasculature_development	276	32	-2.335014	0.135082
GO:0006259_DNA_metabolic_process	876	83	-2.329073	0.135161
GO:0001570_vasculogenesis	38	8	-2.324021	0.134219
GO:0014032_neural_crest_cell_development	38	8	-2.324021	0.134219
GO:0006288_base-excision_repair__DNA_ligation	2	2	-2.291236	0.197727
GO:0050434_positive_regulation_of_viral_transcription	2	2	-2.291236	0.197727
GO:0006412_translation	565	57	-2.290833	0.194776
GO:0000710_meiotic_mismatch_repair	6	3	-2.207833	0.237101
GO:0001958_endochondral_ossification	6	3	-2.207833	0.237101
GO:0001657_ureteric_bud_development	32	7	-2.18729	0.238286
GO:0014033_neural_crest_cell_differentiation	40	8	-2.182367	0.236197
GO:0035270_endocrine_system_development	57	10	-2.177443	0.234028
GO:0048514_blood_vessel_morphogenesis	240	28	-2.147657	0.235616
GO:0007368_determination_of_left_right_symmetry	49	9	-2.140395	0.235135
GO:0030323_respiratory_tube_development	67	11	-2.121743	0.2404
GO:0045935_positive_regulation_of_nucleobase__nucleoside__nucleotide_and_nucleic_acid_metabolic_process	435	45	-2.105984	0.240526
GO:0030437_sporulation_(sensu_Fungi)	12	4	-2.091679	0.245513
GO:0048622_reproductive_sporulation	12	4	-2.091679	0.245513
GO:0030900_forebrain_development	87	13	-2.062741	0.247595
GO:0001837_epithelial_to_mesenchymal_transition	19	5	-2.033108	0.256875
GO:0045165_cell_fate_commitment	170	21	-2.004662	0.258395
GO:0006267_pre-replicative_complex_formation	7	3	-1.988399	0.280488
GO:0000084_S_phase_of_mitotic_cell_cycle	27	6	-1.972953	0.277024
GO:0051320_S_phase	27	6	-1.972953	0.277024
GO:0009892_negative_regulation_of_metabolic_process	602	58	-1.91803	0.304
GO:0048646_anatomical_structure_formation	307	33	-1.908658	0.303256
GO:0006281_DNA_repair	273	30	-1.906531	0.30069
GO:0006268_DNA_unwinding_during_replication	21	5	-1.841687	0.316264
GO:0021536_diencephalon_development	21	5	-1.841687	0.316264
GO:0032392_DNA_geometric_change	21	5	-1.841687	0.316264
GO:0032508_DNA_duplex_unwinding	21	5	-1.841687	0.316264
GO:0007423_sensory_organ_development	265	29	-1.83774	0.313913
GO:0030878_thyroid_gland_development	14	4	-1.835687	0.323333
GO:0009073_aromatic_amino_acid_family_biosynthetic_process	3	2	-1.835298	0.379505
GO:0019083_viral_transcription	3	2	-1.835298	0.379505
GO:0021514_ventral_spinal_cord_interneuron_differentiation	3	2	-1.835298	0.379505
GO:0021521_ventral_spinal_cord_interneuron_specification	3	2	-1.835298	0.379505
GO:0032927_positive_regulation_of_activin_receptor_signaling_pathway	3	2	-1.835298	0.379505
GO:0046782_regulation_of_viral_transcription	3	2	-1.835298	0.379505

Supplemental Table A10

GO Biological Process Categories Overrepresented in CM Cells vs beating EB

GO CATEGORY	TOTAL GENES	OVEREXPRESSED GENES	LOG10(p)	FALSE DISCOVERY RATE
GO:0007010_cytoskeleton_organization_and_biogenesis	738	65	-9.552149	0
GO:0051179_localization	3673	200	-8.089998	0
GO:0030036_actin_cytoskeleton_organization_and_biogenesis	320	33	-6.580854	0
GO:0030029_actin_filament-based_process	339	34	-6.476718	0
GO:0051258_protein_polymerization	71	14	-6.365715	0
GO:0016043_cellular_component_organization_and_biogenesis	3674	187	-5.271385	0
GO:0051234_establishment_of_localization	3169	164	-4.942209	0.002857
GO:0006810_transport	3054	159	-4.922859	0.0025
GO:0008154_actin_polymerization_and_or_depolymerization	84	13	-4.73704	0.003333
GO:0045595_regulation_of_cell_differentiation	256	25	-4.713642	0.003
GO:0006928_cell_motility	618	45	-4.519444	0.0025
GO:0051674_localization_of_cell	618	45	-4.519444	0.0025
GO:0006890_retrograde_vesicle-mediated_transport__Golgi_to_ER	18	6	-4.398221	0.006154
GO:0007118_budding_cell_apical_bud_growth	3	3	-4.243441	0.012667
GO:0007119_budding_cell_isotropic_bud_growth	3	3	-4.243441	0.012667
GO:0046907_intracellular_transport	968	61	-4.039572	0.0175
GO:0048523_negative_regulation_of_cellular_process	1522	87	-4.020894	0.016471
GO:0051649_establishment_of_cellular_localization	1243	74	-4.007594	0.016111
GO:0030041_actin_filament_polymerization	39	8	-4.003074	0.015263
GO:0006809_nitric_oxide_biosynthetic_process	30	7	-3.940656	0.014762
GO:0046209_nitric_oxide_metabolic_process	30	7	-3.940656	0.014762
GO:0051641_cellular_localization	1274	75	-3.90216	0.014545
GO:0030154_cell_differentiation	2344	123	-3.89711	0.015217
GO:0048869_cellular_developmental_process	2346	123	-3.880955	0.014583
GO:0045597_positive_regulation_of_cell_differentiation	77	11	-3.773917	0.014
GO:0048519_negative_regulation_of_biological_process	1615	90	-3.757639	0.014231
GO:0032502_developmental_process	3960	190	-3.730582	0.014815
GO:0051246_regulation_of_protein_metabolic_process	386	30	-3.66554	0.014286
GO:0048856_anatomical_structure_development	2893	145	-3.626979	0.019655
GO:0000902_cell_morphogenesis	820	52	-3.574625	0.02129
GO:0032989_cellular_structure_morphogenesis	820	52	-3.574625	0.02129
GO:0050793_regulation_of_developmental_process	466	34	-3.558502	0.020625
GO:0007243_protein_kinase_cascade	486	35	-3.540318	0.02
GO:0016192_vesicle-mediated_transport	664	44	-3.500178	0.020294
GO:0051094_positive_regulation_of_developmental_process	125	14	-3.486925	0.019714
GO:0048518_positive_regulation_of_biological_process	1424	80	-3.481018	0.019167
GO:0048468_cell_development	1714	92	-3.274481	0.024595
GO:0007117_budding_cell_bud_growth	5	3	-3.268772	0.027436
GO:0048590_non-developmental_growth	5	3	-3.268772	0.027436
GO:0048522_positive_regulation_of_cellular_process	1280	72	-3.183614	0.0295
GO:0045045_secretory_pathway	432	31	-3.17184	0.029024
GO:0006996_organelle_organization_and_biogenesis	1682	90	-3.165368	0.028333
GO:0048731_system_development	2443	123	-3.150255	0.028605
GO:0009653_anatomical_structure_morphogenesis	1708	91	-3.136975	0.029091
GO:0008064_regulation_of_actin_polymerization_and_or_depolymerization	64	9	-3.134686	0.028667
GO:0006936_muscle_contraction	200	18	-3.12891	0.028043
GO:0009889_regulation_of_biosynthetic_process	252	21	-3.112648	0.028085
GO:0048637_skeletal_muscle_development	121	13	-3.104938	0.0275

GO:0045428_regulation_of_nitric_oxide_biosynthetic_process	20	5	-3.095548	0.027551
GO:0048646_anatomical_structure_formation	307	24	-3.079111	0.0274
GO:0007173_epidermal_growth_factor_receptor_signaling_pathway	41	7	-3.056956	0.029216
GO:0048514_blood_vessel_morphogenesis	240	20	-2.991015	0.031346
GO:0030705_cytoskeleton-dependent_intracellular_transport	140	14	-2.989971	0.030755
GO:0030832_regulation_of_actin_filament_length	67	9	-2.987988	0.030185
GO:0046852_positive_regulation_of_bone_remodeling	13	4	-2.926223	0.039636
GO:0022413_reproductive_process_in_single-celled_organism	32	6	-2.909173	0.038246
GO:0048610_reproductive_cellular_process	32	6	-2.909173	0.038246
GO:0001525_angiogenesis	192	17	-2.907818	0.037586
GO:0001767_establishment_of_lymphocyte_polarity	2	2	-2.828139	0.0595
GO:0001768_establishment_of_T_cell_polarity	2	2	-2.828139	0.0595
GO:0007268_synaptic_transmission	455	31	-2.807763	0.05918
GO:0030833_regulation_of_actin_filament_polymerization	23	5	-2.80055	0.059839
GO:0032940_secretion_by_cell	495	33	-2.798369	0.058889
GO:0045429_positive_regulation_of_nitric_oxide_biosynthetic_process	14	4	-2.793446	0.060156
GO:0046903_secretion	577	37	-2.772117	0.060308
GO:0007018_microtubule-based_movement	116	12	-2.76544	0.060152
GO:0006939_smooth_muscle_contraction	46	7	-2.752333	0.060746
GO:0045786_negative_regulation_of_progression_through_cell_cycle	219	18	-2.680974	0.067794
GO:0001568_blood_vessel_development	273	21	-2.677845	0.066957
GO:0051239_regulation_of_multicellular_organismal_process	465	31	-2.661082	0.068143
GO:0007242_intracellular_signaling_cascade	1637	85	-2.62416	0.070986
GO:0001944_vasculature_development	276	21	-2.620652	0.07
GO:0015031_protein_transport	820	48	-2.604694	0.069863
GO:0009887_organ_morphogenesis	758	45	-2.58772	0.07
GO:0007163_establishment_and_or_maintenance_of_cell_polarity	91	10	-2.583706	0.069067
GO:0009893_positive_regulation_of_metabolic_process	572	36	-2.575238	0.068289
GO:0032505_reproduction_of_a_single-celled_organism	37	6	-2.570741	0.067662
GO:0051592_response_to_calcium_ion	16	4	-2.560455	0.068718
GO:0051128_regulation_of_cellular_component_organization_and_biogenesis	124	12	-2.521056	0.07481
GO:0007275_multicellular_organismal_development	2996	142	-2.514688	0.074625
GO:0048771_tissue_remodeling	176	15	-2.47103	0.079136
GO:0030042_actin_filament_depolymerization	39	6	-2.451768	0.081829
GO:0007017_microtubule-based_process	287	21	-2.42054	0.08241
GO:0006417_regulation_of_translation	196	16	-2.414134	0.081548
GO:0007519_striated_muscle_development	179	15	-2.401896	0.081882
GO:0030204_chondroitin_sulfate_metabolic_process	9	3	-2.394855	0.083407
GO:0030206_chondroitin_sulfate_biosynthetic_process	9	3	-2.394855	0.083407
GO:0043330_response_to_exogenous_dsRNA	9	3	-2.394855	0.083407
GO:0045778_positive_regulation_of_ossification	9	3	-2.394855	0.083407
GO:0050650_chondroitin_sulfate_proteoglycan_biosynthetic_process	9	3	-2.394855	0.083407
GO:0050654_chondroitin_sulfate_proteoglycan_metabolic_process	9	3	-2.394855	0.083407
GO:0006937_regulation_of_muscle_contraction	53	7	-2.393505	0.083043
GO:0048513_organ_development	1901	95	-2.388343	0.082473
GO:0045184_establishment_of_protein_localization	886	50	-2.374255	0.082553
GO:0000011_vacuole_inheritance	3	2	-2.36229	0.103673
GO:0014012_axon_regeneration_in_the_peripheral_nervous_system	3	2	-2.36229	0.103673
GO:0032278_positive_regulation_of_gonadotropin_secretion	3	2	-2.36229	0.103673
GO:0046881_positive_regulation_of_follicle-stimulating_hormone_secretion	3	2	-2.36229	0.103673
GO:0045072_regulation_of_interferon-gamma_biosynthetic_process	18	4	-2.361376	0.104343

Supplemental Table A11

GO Biological Process Categories Underrepresented in FH Cells vs CM (Downregulated in FH)

GO CATEGORY	TOTAL GENES	UNDEREXPRESSED GENES	LOG10(p)	FALSE DISCOVERY RATE
GO:0007399_nervous_system_development	1083	100	-9.61274	0
GO:0007275_multicellular_organismal_development	2996	201	-6.619466	0
GO:0048731_system_development	2443	164	-5.293761	0
GO:0003002_regionalization	254	30	-5.166454	0
GO:0001505_regulation_of_neurotransmitter_levels	168	23	-5.139041	0
GO:0007268_synaptic_transmission	455	44	-4.933653	0
GO:0007389_pattern_specification_process	359	37	-4.839285	0.001429
GO:0048856_anatomical_structure_development	2893	186	-4.829193	0.00125
GO:0022008_neurogenesis	540	49	-4.683632	0.001111
GO:0030182_neuron_differentiation	442	42	-4.553324	0.002
GO:0019226_transmission_of_nerve_impulse	518	47	-4.513225	0.003636
GO:0003001_generation_of_a_signal_involved_in_cell-cell_signaling	176	22	-4.318705	0.003333
GO:0007269_neurotransmitter_secretion	119	17	-4.189015	0.003846
GO:0009799_determination_of_symmetry	57	11	-4.083181	0.004667
GO:0009855_determination_of_bilateral_symmetry	57	11	-4.083181	0.004667
GO:0032501_multicellular_organismal_process	4096	244	-3.997914	0.0075
GO:0007420_brain_development	238	26	-3.981552	0.007647
GO:0007368_determination_of_left_right_symmetry	49	10	-3.975628	0.007222
GO:0045110_intermediate_filament_bundle_assembly	3	3	-3.934374	0.011579
GO:0048699_generation_of_neurons	505	44	-3.853328	0.0115
GO:0032502_developmental_process	3960	235	-3.712365	0.015238
GO:0045055_regulated_secretory_pathway	131	17	-3.666601	0.016364
GO:0000904_cellular_morphogenesis_during_differentiation	320	31	-3.657074	0.016087
GO:0007267_cell-cell_signaling	876	66	-3.57067	0.017917
GO:0009952_anterior_posterior_pattern_formation	151	18	-3.38451	0.0244
GO:0001510_RNA_methylation	4	3	-3.348463	0.029231
GO:0007611_learning_and_or_memory	103	14	-3.325352	0.02963
GO:0009953_dorsal_ventral_pattern_formation	81	12	-3.269683	0.031071
GO:0010160_formation_of_organ_boundary	15	5	-3.261255	0.031724
GO:0009653_anatomical_structure_morphogenesis	1708	112	-3.235143	0.030667
GO:0001755_neural_crest_cell_migration	31	7	-3.216597	0.034194
GO:0009887_organ_morphogenesis	758	57	-3.13007	0.036562
GO:0048859_formation_of_anatomical_boundary	16	5	-3.116237	0.036667
GO:0006928_cell_motility	618	48	-3.008726	0.039429
GO:0051674_localization_of_cell	618	48	-3.008726	0.039429
GO:0048667_neuron_morphogenesis_during_differentiation	275	26	-3.003286	0.037838
GO:0048812_neurite_morphogenesis	275	26	-3.003286	0.037838
GO:0030900_forebrain_development	87	12	-2.986139	0.037368
GO:0042476_odontogenesis	44	8	-2.935532	0.050513
GO:0016055_Wnt_receptor_signaling_pathway	139	16	-2.907689	0.04975
GO:0048645_organ_formation	18	5	-2.858991	0.051951
GO:0007610_behavior	483	39	-2.844988	0.054286
GO:0007417_central_nervous_system_development	389	33	-2.834593	0.053488
GO:0048666_neuron_development	344	30	-2.810403	0.053182
GO:0045109_intermediate_filament_organization	6	3	-2.68167	0.076444
GO:0014032_neural_crest_cell_development	38	7	-2.664332	0.075435
GO:0007225_patched_ligand_processing	2	2	-2.622274	0.120213
GO:0048663_neuron_fate_commitment	29	6	-2.617288	0.118958

GO:0031175_neurite_development	310	27	-2.576958	0.119796
GO:0048468_cell_development	1714	108	-2.543549	0.1278
GO:0014033_neural_crest_cell_differentiation	40	7	-2.531565	0.130784
GO:0007409_axonogenesis	252	23	-2.525783	0.128462
GO:0007158_neuron_adhesion	7	3	-2.454658	0.144182
GO:0007310_oocyte_dorsal_ventral_axis_determination	7	3	-2.454658	0.144182
GO:0042416_dopamine_biosynthetic_process	7	3	-2.454658	0.144182
GO:0042417_dopamine_metabolic_process	22	5	-2.441774	0.144286
GO:0008347_glial_cell_migration	15	4	-2.299809	0.174035
GO:0048148_behavioral_response_to_cocaine	8	3	-2.266523	0.188644
GO:0060052_neurofilament_cytoskeleton_organization_and_biogenesis	8	3	-2.266523	0.188644
GO:0007423_sensory_organ_development	265	23	-2.25781	0.186
GO:0014031_mesenchymal_cell_development	56	8	-2.254669	0.184098
GO:0042475_odontogenesis_(sensu_Vertebrata)	35	6	-2.191481	0.197258
GO:0000902_cell_morphogenesis	820	56	-2.172632	0.195156
GO:0032989_cellular_structure_morphogenesis	820	56	-2.172632	0.195156
GO:0009790_embryonic_development	614	44	-2.159548	0.194462
GO:0006585_dopamine_biosynthetic_process_from_tyrosine	3	2	-2.1595	0.235
GO:0007113_endomitotic_cell_cycle	3	2	-2.1595	0.235
GO:0007506_gonadal_mesoderm_development	3	2	-2.1595	0.235
GO:0031133_regulation_of_axon_diameter	3	2	-2.1595	0.235
GO:0040010_positive_regulation_of_growth_rate	3	2	-2.1595	0.235
GO:0042133_neurotransmitter_metabolic_process	47	7	-2.131418	0.235211
GO:0007218_neuropeptide_signaling_pathway	124	13	-2.120799	0.23625
GO:0048762_mesenchymal_cell_differentiation	59	8	-2.116697	0.234384
GO:0042490_mechanoreceptor_differentiation	26	5	-2.114233	0.233514
GO:0019935_cyclic-nucleotide-mediated_signaling	167	16	-2.11079	0.230933
GO:0021537_telencephalon_development	9	3	-2.106375	0.243421
GO:0050789_regulation_of_biological_process	5045	276	-2.069965	0.252857
GO:0030178_negative_regulation_of_Wnt_receptor_signaling_pathway	27	5	-2.042739	0.25641
GO:0008088_axon_cargo_transport	18	4	-1.999805	0.26525
GO:0045665_negative_regulation_of_neuron_differentiation	18	4	-1.999805	0.26525
GO:0035272_exocrine_system_development	50	7	-1.985082	0.265926
GO:0048589_developmental_growth	115	12	-1.981444	0.263415
GO:0002763_positive_regulation_of_myeloid_leukocyte_differentiation	10	3	-1.967373	0.2775
GO:0008078_mesodermal_cell_migration	10	3	-1.967373	0.2775
GO:0048732_gland_development	116	12	-1.953054	0.277647
GO:0046530_photoreceptor_cell_differentiation	63	8	-1.948244	0.275233
GO:0035108_limb_morphogenesis	76	9	-1.937804	0.274368
GO:0000003_reproduction	844	56	-1.930201	0.271932
GO:0009880_embryonic_pattern_specification	90	10	-1.910798	0.278202
GO:0030030_cell_projection_organization_and_biogenesis	398	30	-1.908495	0.273696
GO:0032990_cell_part_morphogenesis	398	30	-1.908495	0.273696
GO:0048858_cell_projection_morphogenesis	398	30	-1.908495	0.273696
GO:0051179_localization	3673	205	-1.884052	0.275699
GO:0019222_regulation_of_metabolic_process	2894	165	-1.874479	0.275851
GO:0002119_larval_development_(sensu_Nematoda)	4	2	-1.872756	0.302353
GO:0008052_sensory_organ_boundary_specification	4	2	-1.872756	0.302353
GO:0010199_organ_boundary_specification_between_lateral_organs_and_the_meristem	4	2	-1.872756	0.302353
GO:0021952_central_nervous_system_projection_neuron_axonogenesis	4	2	-1.872756	0.302353
GO:0042167_heme_catabolic_process	4	2	-1.872756	0.302353

Supplemental Table A12

GO Biological Process Categories Overrepresented in FH Cells vs CM (Upregulated in FH)

GO CATEGORY	TOTAL GENES	OVEREXPRESSED GENES	LOG10(p)	FALSE DISCOVERY RATE
GO:0006084_acetyl-CoA_metabolic_process	44	7	-4.19997	0.08
GO:0045333_cellular_respiration	71	8	-3.631481	0.135
GO:0006099_tricarboxylic_acid_cycle	26	5	-3.536795	0.105
GO:0046356_acetyl-CoA_catabolic_process	26	5	-3.536795	0.105
GO:0007005_mitochondrion_organization_and_biogenesis	78	8	-3.349288	0.126
GO:0009109_coenzyme_catabolic_process	32	5	-3.100466	0.265
GO:0032496_response_to_lipopolysaccharide	19	4	-3.073099	0.238571
GO:0019882_antigen_processing_and_presentation	67	7	-3.042111	0.21625
GO:0007044_cell-substrate_junction_assembly	9	3	-3.024897	0.214444
GO:0051186_cofactor_metabolic_process	317	17	-2.910539	0.214
GO:0051187_cofactor_catabolic_process	37	5	-2.806283	0.25
GO:0007429_secondary_branching__open_tracheal_system	3	2	-2.797485	0.303333
GO:0016044_membrane_organization_and_biogenesis	417	20	-2.737702	0.315385
GO:0002237_response_to_molecule_of_bacterial_origin	24	4	-2.674877	0.307857
GO:0051716_cellular_response_to_stimulus	40	5	-2.652094	0.294
GO:0046785_microtubule_polymerization	12	3	-2.629289	0.289375
GO:0002474_antigen_processing_and_presentation_of_peptide_antigen_via_MHC_class_I	26	4	-2.542466	0.298235
GO:0045935_positive_regulation_of_nucleobase__nucleoside__nucleotide_and_nucleic_acid_metabolic_process	435	20	-2.525729	0.283333
GO:0001836_release_of_cytochrome_c_from_mitochondria	13	3	-2.52284	0.283158
GO:0009437_carnitine_metabolic_process	4	2	-2.503188	0.317143
GO:0043409_negative_regulation_of_MAPKKK_cascade	4	2	-2.503188	0.317143
GO:0048522_positive_regulation_of_cellular_process	1280	45	-2.490382	0.304091
GO:0009267_cellular_response_to_starvation	27	4	-2.480751	0.295652
GO:0000160_two-component_signal_transduction_system_(phosphorelay)	45	5	-2.424339	0.2992
GO:0030447_filamentous_growth	45	5	-2.424339	0.2992
GO:0006629_lipid_metabolic_process	805	31	-2.399197	0.293846
GO:0050896_response_to_stimulus	2926	88	-2.372192	0.287778
GO:0045941_positive_regulation_of_transcription	418	19	-2.371615	0.2775
GO:0008543_fibroblast_growth_factor_receptor_signaling_pathway	30	4	-2.310922	0.285333
GO:0031669_cellular_response_to_nutrient_levels	30	4	-2.310922	0.285333
GO:0006636_fatty_acid_desaturation	5	2	-2.28806	0.324688
GO:0008293_torso_signaling_pathway	5	2	-2.28806	0.324688
GO:0048518_positive_regulation_of_biological_process	1424	48	-2.274651	0.317576
GO:0031668_cellular_response_to_extracellular_stimulus	31	4	-2.258819	0.317941
GO:0006955_immune_response	655	26	-2.25434	0.309143
GO:0006732_coenzyme_metabolic_process	249	13	-2.251604	0.311111
GO:0045893_positive_regulation_of_transcription__DNA-dependent	341	16	-2.203587	0.316757
GO:0031325_positive_regulation_of_cellular_metabolic_process	532	22	-2.179674	0.314474
GO:0007428_primary_branching__open_tracheal_system	6	2	-2.118676	0.343333
GO:0007562_eclosion	6	2	-2.118676	0.343333
GO:0018344_protein_geranylgeranylation	6	2	-2.118676	0.343333
GO:0018348_protein_amino_acid_geranylgeranylation	6	2	-2.118676	0.343333
GO:0030433_ER-associated_protein_catabolic_process	18	3	-2.104855	0.349535
GO:0008152_metabolic_process	8100	209	-2.085488	0.344318
GO:0016049_cell_growth	266	13	-2.020964	0.371556
GO:0006888_ER_to_Golgi_vesicle-mediated_transport	102	7	-2.013374	0.367609
GO:0002376_immune_system_process	967	34	-1.988228	0.365745
GO:0009060_aerobic_respiration	57	5	-1.987298	0.359167

GO:0048002_antigen_processing_and_presentation_of_peptide_antigen	37	4	-1.984037	0.354082
GO:0044255_cellular_lipid_metabolic_process	689	26	-1.98212	0.3472
GO:0000916_cytokinesis__contractile_ring_contraction	7	2	-1.979242	0.347321
GO:0002483_antigen_processing_and_presentation_of_endogenous_peptide_antigen	7	2	-1.979242	0.347321
GO:0007029_endoplasmic_reticulum_organization_and_biogenesis	7	2	-1.979242	0.347321
GO:0019885_antigen_processing_and_presentation_of_endogenous_peptide_antigen_via_MHC_class_I	7	2	-1.979242	0.347321
GO:0030147_natriuresis	7	2	-1.979242	0.347321
GO:0031113_regulation_of_microtubule_polymerization	7	2	-1.979242	0.347321
GO:0006515_misfolded_or_incompletely_synthesized_protein_catabolic_process	20	3	-1.974513	0.347895
GO:0019221_cytokine_and_chemokine_mediated_signaling_pathway	60	5	-1.896255	0.379828
GO:0046916_transition_metal_ion_homeostasis	83	6	-1.894121	0.374576
GO:0032787_monocarboxylic_acid_metabolic_process	307	14	-1.890564	0.369
GO:0046902_regulation_of_mitochondrial_membrane_permeability	8	2	-1.860984	0.400645
GO:0048384_retinoic_acid_receptor_signaling_pathway	8	2	-1.860984	0.400645
GO:0008361_regulation_of_cell_size	279	13	-1.860804	0.394762
GO:0009893_positive_regulation_of_metabolic_process	572	22	-1.836627	0.401875
GO:0015980_energy_derivation_by_oxidation_of_organic_compounds	138	8	-1.813248	0.404923
GO:0006118_electron_transport	442	18	-1.811386	0.399242
GO:0030522_intracellular_receptor-mediated_signaling_pathway	63	5	-1.810992	0.394478
GO:0008637_apoptotic_mitochondrial_changes	23	3	-1.80544	0.396324
GO:0006879_iron_ion_homeostasis	64	5	-1.783755	0.394714
GO:0006944_membrane_fusion	64	5	-1.783755	0.394714
GO:0009612_response_to_mechanical_stimulus	43	4	-1.759515	0.396479
GO:0019883_antigen_processing_and_presentation_of_endogenous_antigen	9	2	-1.758507	0.422329
GO:0045466_R7_cell_differentiation	9	2	-1.758507	0.422329
GO:0006091_generation_of_precursor_metabolites_and_energy	652	24	-1.748267	0.423649
GO:0048193_Golgi_vesicle_transport	172	9	-1.71448	0.430933
GO:0006790_sulfur_metabolic_process	118	7	-1.692771	0.443816
GO:0040007_growth	491	19	-1.675804	0.442857
GO:0006995_cellular_response_to_nitrogen_starvation	10	2	-1.668252	0.46575
GO:0015074_DNA_integration	10	2	-1.668252	0.46575
GO:0043562_cellular_response_to_nitrogen_levels	10	2	-1.668252	0.46575
GO:0006695_cholesterol_biosynthetic_process	26	3	-1.660843	0.467901
GO:0002396_MHC_protein_complex_assembly	1	1	-1.633238	0.618667
GO:0002397_MHC_class_I_protein_complex_assembly	1	1	-1.633238	0.618667
GO:0006324_S-phase_regulated_histone_modification	1	1	-1.633238	0.618667
GO:0009257_10-formyltetrahydrofolate_biosynthetic_process	1	1	-1.633238	0.618667
GO:0009627_systemic_acquired_resistance	1	1	-1.633238	0.618667
GO:0009862_systemic_acquired_resistance__salicylic_acid_mediated_signaling_pathway	1	1	-1.633238	0.618667
GO:0009863_salicylic_acid_mediated_signaling_pathway	1	1	-1.633238	0.618667
GO:0009997_negative_regulation_of_cardioblast_cell_fate_specification	1	1	-1.633238	0.618667
GO:0010106_cellular_response_to_iron_ion_starvation	1	1	-1.633238	0.618667
GO:0015740_C4-dicarboxylate_transport	1	1	-1.633238	0.618667
GO:0019060_intracellular_transport_of_viral_proteins_in_host_cell	1	1	-1.633238	0.618667
GO:0019541_propionate_metabolic_process	1	1	-1.633238	0.618667
GO:0030581_intracellular_protein_transport_in_host	1	1	-1.633238	0.618667
GO:0031144_proteasome_localization	1	1	-1.633238	0.618667
GO:0032258_CVT_pathway	1	1	-1.633238	0.618667
GO:0032447_protein_urmylation	1	1	-1.633238	0.618667
GO:0035072_ecdysone-mediated_induction_of_salivary_gland_cell_autophagic_cell_death	1	1	-1.633238	0.618667
GO:0042504_tyrosine_phosphorylation_of_Stat4_protein	1	1	-1.633238	0.618667
