# Supplementary material for: Transcriptional and Functional Profiling of Human Embryonic Stem Cell-Derived Cardiomyocytes
Source: PLoS One. 2008 Oct 22;3(10):e3474. doi: 10.1371/journal.pone.0003474 (PMC2565131; doi:10.1371/journal.pone.0003474)
Supplement: Table S3 — K-means clustering analysis of microarray data. (3.37 MB PDF) [file pone.0003474.s012.pdf]

Supplemental Table B1

K-means Cluster 1 - - 1775 genes

Gene Name	Description	Acc	UGCluster	UGRepAcc
AA292106	AA292106 zr58h03.s1 Soares_NhHMPu_S1 cDNA clone IMAGE:667637 3', mRNA sequence	AA292106	Hs.471779	BX648107
AA554768	AA554768 ni37h02.s1 NCI_CGAP_Lu1 cDNA clone IMAGE:979059 3' similar to gb:D29805 N-ACETYLLACTOSAMINE SYNTHASE (HUMAN);, mRNA sequence	AA554768	Hs.651277	NM_001497
AA910154	AA910154 od92h01.s1 NCI_CGAP_Br5 cDNA clone IMAGE:1377745, mRNA sequence	AA910154	Hs.332197	AF200348
AAK1	AP2 associated kinase 1 (AAK1), mRNA	NM_014911	Hs.468878	NM_014911
ABCB1	ATP-binding cassette, sub-family B (MDR/TAP), member 1 (ABCB1), mRNA	NM_000927	Hs.489033	NM_000927
ABCB1	ATP-binding cassette, sub-family B (MDR/TAP), member 1 (ABCB1), mRNA	NM_000927	Hs.489033	NM_000927
ABCD3	ATP-binding cassette, sub-family D (ALD), member 3 (ABCD3), mRNA	NM_002858	Hs.700576	BX648715
ABHD2	abhydrolase domain containing 2 (ABHD2), transcript variant 1, mRNA	NM_007011	Hs.122337	NM_007011
ABI1	abl-interactor 1 (ABI1), transcript variant 1, mRNA	NM_005470	Hs.508148	NM_005470
ABI3BP	ABI gene family, member 3 (NESH) binding protein (ABI3BP), mRNA	NM_015429	Hs.477015	BX648726
ACOT4	acyl-CoA thioesterase 4 (ACOT4), mRNA	NM_152331	Hs.49433	AK055797
ACP5	acid phosphatase 5, tartrate resistant (ACP5), mRNA	NM_001611	Hs.1211	BM804974
ACSL5	acyl-CoA synthetase long-chain family member 5 (ACSL5), transcript variant 3, mRNA	NM_203380	Hs.11638	NM_203380
ACTR1A	ARP1 actin-related protein 1 homolog A, centractin alpha (yeast) (ACTR1A), mRNA	NM_005736	Hs.153961	AK096085
ACTR1A	ARP1 actin-related protein 1 homolog A, centractin alpha (yeast) (ACTR1A), mRNA	NM_005736	Hs.153961	AK096085
ACTR2	ARP2 actin-related protein 2 homolog (yeast) (ACTR2), transcript variant 1, mRNA	NM_001005386	Hs.699451	NM_001005386
ADAM10	ADAM metallopeptidase domain 10 (ADAM10), mRNA	NM_001110	Hs.578508	NM_001110
ADAM7	ADAM metallopeptidase domain 7 (ADAM7), mRNA	NM_003817	Hs.668805	NM_003817
ADAM9	ADAM metallopeptidase domain 9 (meltrin gamma) (ADAM9), transcript variant 1, mRNA	NM_003816	Hs.591852	NM_003816
ADAMTSL1	ADAMTS-like 1 (ADAMTSL1), transcript variant 3, mRNA	NM_139264	Unknown	
ADIPOQ	adiponectin, C1Q and collagen domain containing (ADIPOQ), mRNA	NM_004797	Hs.80485	NM_004797
AES	amino-terminal enhancer of split (AES), transcript variant 3, mRNA	NM_198970	Hs.515053	AK095154
AF075027	full length insert cDNA YI37C01.	AF075027	Hs.432924	CD673367
AF086044	full length insert cDNA clone YX74D05.	AF086044	Hs.439682	AK123448
AF086179	full length insert cDNA clone ZB96E04.	AF086179	Hs.463278	NM_004287
AF113674	clone FLB1727 PRO0398 mRNA, complete cds.	AF113674	Unknown	
AF277188	PNAS-130 mRNA, complete cds.	AF277188	Hs.534619	BC053679
AF289601	clone pp8142 unknown mRNA.	AF289601	Hs.638709	AF289601
AF305825	PRO2869 mRNA, complete cds.	AF305825	Unknown	
AF390550	cervical cancer suppressor gene 5 mRNA, complete cds.	AF390550	Hs.592078	AY279347
AFAP	actin filament associated protein (AFAP), transcript variant 1, mRNA	NM_021638	Hs.529369	NM_198595
AFF4	AF4/FMR2 family, member 4 (AFF4), mRNA	NM_014423	Hs.519313	NM_014423
AGA	aspartylglucosaminidase (AGA), mRNA	NM_000027	Hs.207776	NM_000027
AGPAT2	1-acylglycerol-3-phosphate O-acyltransferase 2 (lysophosphatidic acid acyltransferase, beta) (AGPAT2), transcript variant 1, mRNA	NM_006412	Hs.320151	NM_006412
AGXT2L2	alanine-glyoxylate aminotransferase 2-like 2 (AGXT2L2), mRNA	NM_153373	Hs.248746	AK123488
AHNAK	AHNAK nucleoprotein (desmoyokin) (AHNAK), transcript variant 1, mRNA	NM_001620	Hs.502756	NM_001620
AHNAK	AHNAK nucleoprotein (desmoyokin) (AHNAK), transcript variant 2, mRNA	NM_024060	Hs.502756	NM_001620
AHNAK	AHNAK nucleoprotein (desmoyokin) (AHNAK), transcript variant 1, mRNA	NM_001620	Hs.502756	NM_001620
AHRR	aryl-hydrocarbon receptor repressor (AHRR), mRNA	NM_020731	Hs.50823	AB033060
AI219192	AI219192 qg17f03.x1 Soares_placenta_8to9weeks_2NbHP8to9W cDNA clone IMAGE:1759805 3', mRNA sequence	AI219192	Hs.400095	NM_014365
AI344752	AI344752 qp05c06.x1 NCI_CGAP_Kid5 cDNA clone IMAGE:1917130 3', mRNA sequence	AI344752	Hs.74564	CR590829
AI559980	AI559980 tq77f07.x1 NCI_CGAP_Ut1 cDNA clone IMAGE:2214853 3', mRNA sequence	AI559980	Hs.292579	BC004192
AI669333	ty31a04.x1 NCI_CGAP_Ut2 cDNA clone IMAGE:2280654 3', mRNA sequence	AI669333	Hs.643975	AI669333
AI709405	AI709405 as37d03.x1 Barstead aorta HPLRB6 cDNA clone IMAGE:2319365 3' similar to contains Alu repetitive element;, mRNA sequence	AI709405	Hs.697020	BG535650
AIP	aryl hydrocarbon receptor interacting protein (AIP), mRNA	NM_003977	Hs.412433	BG913006
AIP	aryl hydrocarbon receptor interacting protein (AIP), mRNA	NM_003977	Hs.412433	BG913006
AK021933	cDNA FLJ11871 fis, clone HEMBA1007052.	AK021933	Unknown	
AK022252	cDNA FLJ12190 fis, clone MAMMA1000842.	AK022252	Unknown	

AK023572	cDNA FLJ13510 fis, clone PLACE1005146.	AK023572	Hs.658586	AK023572
AK023663	cDNA FLJ13601 fis, clone PLACE1010069.	AK023663	Hs.594861	AL049435
AK025762	cDNA: FLJ22109 fis, clone HEP18091.	AK025762	Unknown	
AK025797	cDNA: FLJ22144 fis, clone HEP21732.	AK025797	Hs.662004	BX647167
AK026195	cDNA: FLJ22542 fis, clone HSI00196.	AK026195	Hs.667309	AK026195
AK026477	cDNA: FLJ22824 fis, clone KAIA3991.	AK026477	Hs.150824	AK026477
AK092668	cDNA FLJ35349 fis, clone PUAEN1000039.	AK092668	Hs.483238	NM_004815
AK094950	cDNA FLJ37631 fis, clone BRCOC2015944.	AK094950	Hs.375762	AK056686
AK094991	cDNA FLJ37672 fis, clone BRHIP2012059.	AK094991	Hs.633316	AK094991
AK095719	cDNA FLJ38400 fis, clone FEBRA2008159.	AK095719	Hs.457407	AK124699
AK095727	cDNA FLJ38408 fis, clone FEBRA2009029.	AK095727	Hs.634471	AK095727
AK096256	cDNA FLJ38937 fis, clone NT2NE2015511.	AK096256	Hs.667790	AK096256
AK124426	cDNA FLJ42435 fis, clone BLADE2006849.	AK124426	Hs.356481	AB209869
AK127309	cDNA FLJ45377 fis, clone BRHIP3019956.	AK127309	Unknown	
AK129838	cDNA FLJ26328 fis, clone HRT01493.	AK129838	Hs.435767	BC042043
AK129956	cDNA FLJ26446 fis, clone KDN02743.	AK129956	Hs.170131	NM_205843
AK130514	cDNA FLJ27004 fis, clone SLV04966.	AK130514	Hs.699296	AL833852
AKAP2	A kinase (PRKA) anchor protein 2 (AKAP2), mRNA	NM_001004065	Hs.591908	NM_053016
AKR1C1	aldo-keto reductase family 1, member C1 (dihydrodiol dehydrogenase 1; 20-alpha (3-alpha)-hydroxysteroid dehydrogenase), mRNA (cDNA clone MGC:42600 IMAGE:4825338), complete cds.	BC040210	Hs.460260	AK226067
AL049270	mRNA; cDNA DKFZp564G223 (from clone DKFZp564G223).	AL049270	Hs.597836	AL049270
ALDH2	aldehyde dehydrogenase 2 family (mitochondrial) (ALDH2), nuclear gene encoding mitochondrial protein, mRNA	NM_000690	Hs.632733	NM_000690
ALDH2	aldehyde dehydrogenase 2 family (mitochondrial) (ALDH2), nuclear gene encoding mitochondrial protein, mRNA	NM_000690	Hs.632733	NM_000690
ALF	TFIIA-alpha/beta-like factor (ALF), transcript variant 2, mRNA	NM_172196	Hs.44385	NM_006873
ALPK1	alpha-kinase 1 (ALPK1), mRNA	NM_025144	Unknown	
ALPK1	alpha-kinase 1 (ALPK1), mRNA	NM_025144	Unknown	
AMOTL1	angiomotin like 1 (AMOTL1), mRNA	NM_130847	Hs.503594	NM_130847
AMOTL1	angiomotin like 1 (AMOTL1), mRNA	NM_130847	Hs.503594	NM_130847
ANGPTL2	angiopoietin-like 2 (ANGPTL2), mRNA	NM_012098	Hs.653262	NM_012098
ANGPTL2	angiopoietin-like 2 (ANGPTL2), mRNA	NM_012098	Hs.653262	NM_012098
ANKRD1	ankyrin repeat domain 1 (cardiac muscle) (ANKRD1), mRNA	NM_014391	Hs.448589	NM_014391
ANKRD54	ankyrin repeat domain 54 (ANKRD54), mRNA	NM_138797	Hs.135259	AK127583
ANKRD54	ankyrin repeat domain 54 (ANKRD54), mRNA	NM_138797	Hs.135259	AK127583
ANTXR2	anthrax toxin receptor 2 (ANTXR2), mRNA	NM_058172	Hs.162963	NM_058172
ANTXR2	anthrax toxin receptor 2 (ANTXR2), mRNA	NM_058172	Hs.162963	NM_058172
ANXA11	annexin A11 (ANXA11), transcript variant c, mRNA	NM_145869	Hs.530291	NM_145869
ANXA2	annexin A2 (ANXA2), transcript variant 2, mRNA	NM_001002857	Hs.511605	BF242966
ANXA2	annexin A2 (ANXA2), transcript variant 2, mRNA	NM_001002857	Hs.511605	BF242966
ANXA2	annexin A2 (ANXA2), transcript variant 2, mRNA	NM_001002857	Hs.511605	BF242966
ANXA2P1	annexin A2 pseudogene 1 (ANXA2P1) on chromosome 4	NR_001562	Unknown	
ANXA2P3	annexin A2 pseudogene 3 (ANXA2P3) on chromosome 10	NR_001446	Unknown	
ANXA4	annexin A4 (ANXA4), mRNA	NM_001153	Hs.422986	BX641114
AP1G1	adaptor-related protein complex 1, gamma 1 subunit (AP1G1), transcript variant 1, mRNA	NM_001030007	Hs.461253	NM_001030007
AP1M1	adaptor-related protein complex 1, mu 1 subunit (AP1M1), mRNA	NM_032493	Hs.71040	AB209808
AP2A2	adaptor-related protein complex 2, alpha 2 subunit (AP2A2), mRNA	NM_012305	Hs.19121	AB209422
AP4M1	adaptor-related protein complex 4, mu 1 subunit (AP4M1), mRNA	NM_004722	Hs.632317	NM_004722
APCS	amyloid P component, serum (APCS), mRNA	NM_001639	Hs.507080	BG533315
APOF	apolipoprotein F (APOF), mRNA	NM_001638	Hs.534302	BC026257
APOL6	apolipoprotein L, 6 (APOL6), mRNA	NM_030641	Hs.257352	NM_030641
ARHGAP10	Rho GTPase activating protein 10 (ARHGAP10), mRNA	NM_024605	Hs.368631	NM_024605
ARHGAP18	Rho GTPase activating protein 18 (ARHGAP18), mRNA	NM_033515	Hs.486458	NM_033515

ARHGAP23	PREDICTED: Rho GTPase activating protein 23, transcript variant 1 (ARHGAP23), mRNA	XM_290799	Hs.374446	XM_290799
ARHGAP24	Rho GTPase activating protein 24 (ARHGAP24), transcript variant 1, mRNA	NM_001025616	Hs.444229	NM_001025616
ARHGAP24	Rho GTPase activating protein 24 (ARHGAP24), transcript variant 1, mRNA	NM_001025616	Hs.444229	NM_001025616
ARHGAP6	Rho GTPase activating protein 6 (ARHGAP6), transcript variant 5, mRNA	NM_013422	Hs.435291	AB208792
ARHGDIA	Rho GDP dissociation inhibitor (GDI) alpha (ARHGDIA), mRNA	NM_004309	Hs.159161	BE792439
ARHGEF12	Rho guanine nucleotide exchange factor (GEF) 12 (ARHGEF12), mRNA	NM_015313	Hs.24598	AF180681
ARID5A	AT rich interactive domain 5A (MRF1-like) (ARID5A), transcript variant 2, mRNA	NM_006673	Unknown	
ARL1	ADP-ribosylation factor-like 1 (ARL1), mRNA	NM_001177	Hs.372616	BX537387
ARL10	ADP-ribosylation factor-like 10 (ARL10), mRNA	NM_173664	Hs.424552	NM_173664
ARL6IP4	ADP-ribosylation-like factor 6 interacting protein 4 (ARL6IP4), transcript variant 1, mRNA	NM_018694	Hs.103561	BM907906
ARL6IP4	ADP-ribosylation-like factor 6 interacting protein 4 (ARL6IP4), transcript variant 4, mRNA	NM_001002252	Hs.103561	BM907906
ARL8B	ADP-ribosylation factor-like 8B (ARL8B), mRNA	NM_018184	Hs.250009	BC063125
ARMC5	armadillo repeat containing 5 (ARMC5), mRNA	NM_024742	Hs.121915	AY217348
ARPC1A	actin related protein 2/3 complex, subunit 1A, 41kDa (ARPC1A), mRNA	NM_006409	Hs.124126	NM_006409
ARPC2	actin related protein 2/3 complex, subunit 2, 34kDa (ARPC2), transcript variant 1, mRNA	NM_152862	Hs.529303	BF240734
ARPC3	actin related protein 2/3 complex, subunit 3, 21kDa (ARPC3), mRNA	NM_005719	Hs.524741	BU943983
ARRDC3	arrestin domain containing 3 (ARRDC3), mRNA	NM_020801	Hs.24684	BC053619
ARSB	arylsulfatase B (ARSB), transcript variant 2, mRNA	NM_198709	Hs.149103	NM_000046
ARSJ	arylsulfatase family, member J (ARSJ), mRNA	NM_024590	Hs.22895	NM_024590
ASAHL	N-acylsphingosine amidohydrolase (acid ceramidase)-like (ASAHL), transcript variant 1, mRNA	NM_014435	Hs.699178	NM_014435
ASB8	ankyrin repeat and SOCS box-containing 8 (ASB8), mRNA	NM_024095	Hs.432699	NM_024095
ATF5	activating transcription factor 5 (ATF5), mRNA	NM_012068	Hs.9754	AB073613
ATF6	activating transcription factor 6 (ATF6), mRNA	NM_007348	Hs.492740	BX538263
ATF7	activating transcription factor 7, mRNA (cDNA clone IMAGE:5285571), complete cds.	BC042363	Hs.12286	NM_006856
ATG4A	ATG4 autophagy related 4 homolog A (S. cerevisiae) (ATG4A), transcript variant 3, mRNA	NM_178271	Unknown	
ATP11B	ATPase, Class VI, type 11B (ATP11B), mRNA	NM_014616	Hs.478429	NM_014616
ATP11B	ATPase, Class VI, type 11B (ATP11B), mRNA	NM_014616	Hs.478429	NM_014616
ATP5E	ATP synthase, H+ transporting, mitochondrial F1 complex, epsilon subunit (ATP5E), nuclear gene encoding mitochondrial protein, mRNA	NM_006886	Hs.177530	CR595852
ATP5E	ATP synthase, H+ transporting, mitochondrial F1 complex, epsilon subunit (ATP5E), nuclear gene encoding mitochondrial protein, mRNA	NM_006886	Hs.177530	CR595852
ATP6AP1	ATPase, H+ transporting, lysosomal accessory protein 1 (ATP6AP1), mRNA	NM_001183	Hs.6551	NM_001183
ATP8B1	ATPase, Class I, type 8B, member 1 (ATP8B1), mRNA	NM_005603	Hs.216623	NM_005603
ATPBD1B	ATP binding domain 1 family, member B (ATPBD1B), mRNA	NM_018066	Hs.14333	BM928391
AU144988	AU144988 AU144988 HEMBA1 cDNA clone HEMBA1003560 3', mRNA sequence	AU144988	Hs.695989	NM_053064
AV721971	AV721971 HTB cDNA clone HTBAUC04 5', mRNA sequence	AV721971	Hs.637018	AV721971
AW198179	AW198179 xo31f07.x1 NCI_CGAP_Pan1 cDNA clone IMAGE:2705605 3', mRNA sequence	AW198179	Hs.509765	BX641076
AW445156	UI-H-BI3-akc-c-06-0-UI.s1 NCI_CGAP_Sub5 cDNA clone IMAGE:2733706 3', mRNA sequence	AW445156	Hs.537297	BG426795
AW673984	AW673984 ba59d03.x1 NIH_MGC_10 cDNA clone IMAGE:2900837 3', mRNA sequence	AW673984	Hs.369920	NM_015646
AW851396	AW851396 IL3-CT0220-170200-066-D05 CT0220 cDNA, mRNA sequence	AW851396	Hs.699196	CR936719
AXL	AXL receptor tyrosine kinase (AXL), transcript variant 1, mRNA	NM_021913	Hs.590970	NM_021913
AY047582	neural precursor cell expressed developmentally down-regulated protein 8 (NEDD8) mRNA, complete cds.	AY047582	Hs.571861	AY047582
AYTL1	acyltransferase like 1 (AYTL1), mRNA	NM_017839	Hs.460857	BX641069
AZI1	5-azacytidine induced 1 (AZI1), transcript variant 1, mRNA	NM_014984	Hs.514578	BC012130
B3GALNT1	beta-1,3-N-acetylgalactosaminyltransferase 1 (globoside blood group) (B3GALNT1), transcript variant 5, mRNA	NM_001038628	Hs.418062	NM_001038628
B3GNT8	UDP-GlcNAc:betaGal beta-1,3-N-acetylglucosaminyltransferase 8 (B3GNT8), mRNA	NM_198540	Hs.441681	AY277592
B4GALT2	UDP-Gal:betaGlcNAc beta 1,4- galactosyltransferase, polypeptide 2 (B4GALT2), transcript variant 2, mRNA	NM_003780	Hs.632403	AK095873
BACH1	BTB and CNC homology 1, basic leucine zipper transcription factor 1 (BACH1), transcript variant 3, mRNA	NM_001011545	Hs.154276	NM_206866
BAG3	BCL2-associated athanogene 3 (BAG3), mRNA	NM_004281	Hs.523309	NM_004281
BBS5	Bardet-Biedl syndrome 5 (BBS5), mRNA	NM_152384	Hs.233398	NM_152384
BC000604	mRNA similar to cisplatin resistance-associated overexpressed protein (cDNA clone IMAGE:3346964).	BC000604	Unknown	
BC004503	cDNA clone IMAGE:3836890, **** WARNING: chimeric clone ****.	BC004503	Unknown	

BC011779	cDNA clone IMAGE:3941306, partial cds.	BC011779	Hs.634705	BC065527
BC012360	Homo sapiens, clone IMAGE:4641261, mRNA.	BC012360	Hs.509200	BC015671
BC015334	Homo sapiens, clone IMAGE:4391654, mRNA, partial cds.	BC015334	Hs.368563	AF146074
BC015783	Homo sapiens, clone IMAGE:4857346, mRNA.	BC015783	Unknown	
BC015835	cDNA clone IMAGE:4293096, partial cds.	BC015835	Hs.594143	BM542054
BC015836	Homo sapiens, clone IMAGE:4293240, mRNA.	BC015836	Hs.518341	BF790499
BC015903	cDNA clone IMAGE:3919515, partial cds.	BC015903	Hs.650085	BC052811
BC017209	Homo sapiens, clone IMAGE:3956555, mRNA, partial cds.	BC017209	Hs.676055	BC017209
BC017563	cDNA clone IMAGE:3940218, partial cds.	BC017563	Hs.684452	BC017563
BC020829	mRNA similar to hypothetical protein FLJ21394 (cDNA clone IMAGE:4770900).	BC020829	Unknown	
BC022362	cDNA clone MGC:23888 IMAGE:4704496, complete cds.	BC022362	Hs.449621	AK128524
BC023989	cDNA clone IMAGE:3931276, partial cds.	BC023989	Hs.621309	BC023989
BC031319	cDNA clone IMAGE:5278237.	BC031319	Hs.637627	BI458382
BC031867	cDNA clone IMAGE:4825132.	BC031867	Hs.593719	BC031867
BC040303	cDNA clone IMAGE:4830172.	BC040303	Hs.696202	XM_001126149
BC043218	cDNA clone IMAGE:5295323.	BC043218	Hs.220558	BC043218
BC047636	cDNA clone IMAGE:4822429.	BC047636	Hs.64341	BC047636
BC053632	cDNA clone IMAGE:6500775, partial cds.	BC053632	Hs.660080	BX537644
BC063022	cDNA clone IMAGE:5246259, partial cds.	BC063022	Hs.25338	AL832007
BCAP29	B-cell receptor-associated protein BAP29 mRNA, complete cds.	AF126020	Hs.303787	BX537729
BCL2A1	BCL2-related protein A1 (BCL2A1), mRNA	NM_004049	Hs.227817	BF690887
BCL2L1	BCL2-like 1 (BCL2L1), nuclear gene encoding mitochondrial protein, transcript variant 2, mRNA	NM_001191	Hs.516966	CR936637
BCL7B	B-cell CLL/lymphoma 7B (BCL7B), transcript variant 2, mRNA	NM_138707	Unknown	
BCL7C	B-cell CLL/lymphoma 7C (BCL7C), mRNA	NM_004765	Hs.658547	BC058863
BE672039	7a50b09.x1 NCI_CGAP_GC6 cDNA clone IMAGE:3222137 3', mRNA sequence	BE672039	Hs.697570	BE672039
BE710245	BE710245 IL3-HT0619-120700-212-G05 HT0619 cDNA, mRNA sequence	BE710245	Hs.203717	NM_212482
BE739632	BE739632 601556329T1 NIH_MGC_58 cDNA clone IMAGE:3826255 3', mRNA sequence	BE739632	Hs.458358	NM_003309
BE795374	601592904F1 NIH_MGC_7 cDNA clone IMAGE:3946800 5', mRNA sequence	BE795374	Hs.436899	BE795374
BE932552	BE932552 QV3-HT0637-310800-319-h10 HT0637 cDNA, mRNA sequence	BE932552	Hs.362728	NM_004261
BECN1	beclin 1 (coiled-coil, myosin-like BCL2 interacting protein) (BECN1), mRNA	NM_003766	Hs.12272	BX647788
BET1L	blocked early in transport 1 homolog (S. cerevisiae)-like (BET1L), mRNA	NM_016526	Hs.414418	NM_016526
BF213738	BF213738 601847628F1 NIH_MGC_55 cDNA clone IMAGE:4078519 5', mRNA sequence	BF213738	Hs.85201	NM_005127
BF326811	BF326811 QV0-BN0042-140900-388-e04 BN0042 cDNA, mRNA sequence	BF326811	Hs.534293	AK093049
BF803156	BF803156 CM0-CI0139-011100-675-h03 CI0139 cDNA, mRNA sequence	BF803156	Hs.114033	BF028477
BG011034	BG011034 QV1-GN0319-071200-559-b09 GN0319 cDNA, mRNA sequence	BG011034	Hs.184736	BC015794
BG461173	RST43919 Athersys RAGE Library cDNA, mRNA sequence	BG461173	Hs.598357	BG461173
BG612665	602641001F1 NIH_MGC_61 cDNA clone IMAGE:4771873 5', mRNA sequence	BG612665	Hs.564888	BG612665
BHLHB2	basic helix-loop-helix domain containing, class B, 2 (BHLHB2), mRNA	NM_003670	Hs.171825	NM_003670
BI869933	603393173F1 NIH_MGC_90 cDNA clone IMAGE:5403162 5', mRNA sequence	BI869933	Hs.645842	BF217373
BM677531	UI-E-EO1-aid-d-03-0-UI.s1 UI-E-EO1 cDNA clone UI-E-EO1-aid-d-03-0-UI 3', mRNA sequence	BM677531	Hs.617723	BM677531
BMP8B	bone morphogenetic protein 8b (osteogenic protein 2) (BMP8B), mRNA	NM_001720	Hs.664022	AY303955
BPIL1	bactericidal/permeability-increasing protein-like 1 (BPIL1), mRNA	NM_025227	Hs.257045	AK172819
BQ002790	UI-H-EI1-ayv-o-09-0-UI.s1 NCI_CGAP_EI1 cDNA clone IMAGE:5844320 3', mRNA sequence	BQ002790	Hs.646145	BF217116
BRE	brain and reproductive organ-expressed (TNFRSF1A modulator) (BRE), transcript variant 1, mRNA	NM_004899	Hs.258314	NM_199193
BRF1	BRF1 homolog, subunit of RNA polymerase III transcription initiation factor IIIB (S. cerevisiae) (BRF1), transcript variant 2, mRNA	NM_145696	Unknown	
BTBD10	BTB (POZ) domain containing 10 (BTBD10), mRNA	NM_032320	Hs.332382	AY221959
BTBD14A	BTB (POZ) domain containing 14A (BTBD14A), mRNA	NM_144653	Hs.112895	NM_144653
BTBD9	mRNA for KIAA1880 protein, partial cds.	AB067467	Unknown	
BTG2	BTG family, member 2 (BTG2), mRNA	NM_006763	Hs.519162	NM_006763
BTN2A1	butyrophilin, subfamily 2, member A1 (BTN2A1), transcript variant 2, mRNA	NM_078476	Hs.159028	BC016661
BTN3A2	butyrophilin, subfamily 3, member A2 (BTN3A2), mRNA	NM_007047	Hs.376046	NM_007047

BTN3A3	butyrophilin, subfamily 3, member A3 (BTN3A3), transcript variant 1, mRNA	NM_006994	Hs.167741	AB209581
BU507302	AGENCOURT_10309688 NIH_MGC_71 cDNA clone IMAGE:6501220 5', mRNA sequence	BU507302	Hs.598309	BU507302
BU624603	BU624603 UI-H-FG1-bgk-p-15-0-UI.s1 NCI_CGAP_FG1 cDNA clone UI-H-FG1-bgk-p-15-0-UI 3', mRNA sequence	BU624603	Hs.203717	NM_212482
BU629094	BU629094 UI-H-FL0-bdh-d-02-0-UI.s1 NCI_CGAP_FL0 cDNA clone UI-H-FL0-bdh-d-02-0-UI 3', mRNA sequence	BU629094	Unknown	
BU742669	BU742669 UI-E-EO1-aiv-g-14-0-UI.s1 UI-E-EO1 cDNA clone UI-E-EO1-aiv-g-14-0-UI 3', mRNA sequence	BU742669	Hs.496684	BX648255
BU753102	BU753102 UI-1-BB1-aii-b-10-0-UI.s1 NCI_CGAP_Pl5 cDNA clone UI-1-BB1-aii-b-10-0-UI 3', mRNA sequence	BU753102	Hs.699196	CR936719
BX107768	BX107768 Soares_testis_NHT cDNA clone IMAGp998E154173, mRNA sequence	BX107768	Hs.116864	BX107768
BX332393	BX332393 NEUROBLASTOMA COT 25-NORMALIZED cDNA clone CS0DC011YJ08 3-PRIME, mRNA sequence	BX332393	Hs.646203	BX332393
BX397553	BX397553 BX397553 PLACENTA COT 25-NORMALIZED cDNA clone CS0DI039YG01 3-PRIME, mRNA sequence	BX397553	Hs.634586	BX647928
BX415272	BX415272 THYMUS cDNA clone CS0CAP005YK02 3-PRIME, mRNA sequence	BX415272	Hs.681876	BQ278534
BX641669	BX641669 DKFZp686J19137_r1 686 (synonym: hlcc3) cDNA clone DKFZp686J19137 5', mRNA sequence	BX641669	Unknown	
BX648207	mRNA; cDNA DKFZp686E16168 (from clone DKFZp686E16168).	BX648207	Hs.23554	BX648207
BY798802	BY798802 eye cDNA clone HE3861.seq 5', mRNA sequence	BY798802	Hs.627877	BY798802
C10orf10	chromosome 10 open reading frame 10 (C10orf10), mRNA	NM_007021	Hs.93675	NM_007021
C10orf10	chromosome 10 open reading frame 10 (C10orf10), mRNA	NM_007021	Hs.93675	NM_007021
C10orf11	chromosome 10 open reading frame 11 (C10orf11), mRNA	NM_032024	Hs.118161	AK125328
C10orf97	chromosome 10 open reading frame 97 (C10orf97), mRNA	NM_024948	Hs.590985	BC067799
C11orf60	chromosome 11 open reading frame 60 (C11orf60), mRNA	NM_020153	Hs.533738	NM_020153
C11orf67	chromosome 11 open reading frame 67 (C11orf67), mRNA	NM_024684	Hs.503357	BG121856
C12orf25	chromosome 12 open reading frame 25 (C12orf25), mRNA	NM_032130	Hs.524406	BC035621
C12orf4	chromosome 12 open reading frame 4 (C12orf4), mRNA	NM_020374	Hs.302977	NM_020374
C12orf41	chromosome 12 open reading frame 41 (C12orf41), mRNA	NM_017822	Hs.505412	NM_017822
C12orf50	chromosome 12 open reading frame 50 (C12orf50), mRNA	NM_152589	Hs.112930	AL833338
C13orf1	chromosome 13 open reading frame 1 (C13orf1), mRNA	NM_020456	Hs.44235	AF334405
C13orf16	chromosome 13 open reading frame 16 (C13orf16), mRNA	NM_152324	Hs.210677	BI830521
C14orf142	chromosome 14 open reading frame 142 (C14orf142), mRNA	NM_032490	Hs.20142	NM_032490
C14orf153	chromosome 14 open reading frame 153 (C14orf153), mRNA	NM_032374	Hs.656616	NM_032374
C14orf159	chromosome 14 open reading frame 159 (C14orf159), mRNA	NM_024952	Unknown	
C14orf173	chromosome 14 open reading frame 173 (C14orf173), transcript variant 2, mRNA	NM_022489	Hs.24956	NM_022489
C14orf28	chromosome 14 open reading frame 28 (C14orf28), mRNA	NM_001017923	Hs.82098	NM_001017923
C14orf65	chromosome 14 open reading frame 65, mRNA (cDNA clone MGC:117455 IMAGE:5139193), complete cds.	BC110042	Hs.653294	AK123840
C15orf37	chromosome 15 open reading frame 37 (C15orf37), mRNA	NM_175898	Hs.512015	NM_175898
C15orf38	chromosome 15 open reading frame 38 (C15orf38), mRNA	NM_182616	Hs.6734	AL832121
C15orf38	chromosome 15 open reading frame 38 (C15orf38), mRNA	NM_182616	Hs.6734	AL832121
C15orf40	chromosome 15 open reading frame 40 (C15orf40), mRNA	NM_144597	Hs.352541	CR614786
C15orf49	chromosome 15 open reading frame 49, mRNA (cDNA clone MGC:95352 IMAGE:7216891), complete cds.	BC069077	Hs.640307	EL736379
C16orf30	chromosome 16 open reading frame 30 (C16orf30), mRNA	NM_024600	Hs.459652	AY676494
C16orf45	chromosome 16 open reading frame 45 (C16orf45), mRNA	NM_033201	Hs.460095	AK092923
C16orf58	chromosome 16 open reading frame 58 (C16orf58), mRNA	NM_022744	Hs.9003	AK023930
C17orf79	chromosome 17 open reading frame 79 (C17orf79), mRNA	NM_018405	Hs.462729	BQ946887
C19orf12	chromosome 19 open reading frame 12 (C19orf12), transcript variant 2, mRNA	NM_031448	Hs.529094	NM_031448
C19orf40	chromosome 19 open reading frame 40 (C19orf40), mRNA	NM_152266	Hs.579899	AK128668
C19orf56	chromosome 19 open reading frame 56 (C19orf56), mRNA	NM_016145	Hs.108969	BU506766
C19orf56	chromosome 19 open reading frame 56 (C19orf56), mRNA	NM_016145	Hs.108969	BU506766
C19orf56	chromosome 19 open reading frame 56 (C19orf56), mRNA	NM_016145	Hs.108969	BU506766
C1orf186	chromosome 1 open reading frame 186 (C1orf186), mRNA	NM_001007544	Hs.662248	NM_001007544
C1orf212	chromosome 1 open reading frame 212 (C1orf212), mRNA	NM_138428	Hs.27160	BC034598
C1orf58	chromosome 1 open reading frame 58 (C1orf58), mRNA	NM_144695	Hs.552608	AK094916
C1orf84	chromosome 1 open reading frame 84 (C1orf84), transcript variant 3, mRNA	NM_182518	Unknown	

C20orf117	cDNA FLJ32494 fis, clone SKNSH2000151.	AK057056	Unknown	
C20orf194	chromosome 20 open reading frame 194, mRNA (cDNA clone IMAGE:6189246), complete cds.	BC106086	Hs.516853	NM_001009984
C20orf29	chromosome 20 open reading frame 29 (C20orf29), mRNA	NM_018347	Hs.104806	AK002030
C20orf43	chromosome 20 open reading frame 43 (C20orf43), mRNA	NM_016407	Hs.517134	BM916890
C20orf45	chromosome 20 open reading frame 45 (C20orf45), mRNA	NM_016045	Hs.656865	BC013969
C21orf34	chromosome 21 open reading frame 34 (C21orf34), transcript variant 2, mRNA	NM_001005733	Hs.473394	AK095614
C21orf6	chromosome 21 open reading frame 6 (C21orf6), mRNA	NM_016940	Hs.34136	CR592518
C21orf69	chromosome 21 open reading frame 69 (C21orf69), mRNA	NM_058189	Unknown	
C2orf32	chromosome 2 open reading frame 32 (C2orf32), mRNA	NM_015463	Hs.212885	BC035125
C2orf34	chromosome 2 open reading frame 34, mRNA (cDNA clone IMAGE:4673016), complete cds.	BC029359	Hs.468349	AK027104
C2orf42	chromosome 2 open reading frame 42 (C2orf42), mRNA	NM_017880	Hs.413123	AK000565
C3orf23	chromosome 3 open reading frame 23 (C3orf23), transcript variant 3, mRNA	NM_001029840	Hs.55131	NM_173826
C4orf32	chromosome 4 open reading frame 32 (C4orf32), mRNA	NM_152400	Hs.23439	BC041964
C5orf13	chromosome 5 open reading frame 13 (C5orf13), mRNA	NM_004772	Hs.36053	CR607755
C5orf14	chromosome 5 open reading frame 14 (C5orf14), mRNA	NM_024715	Hs.696335	NM_024715
C6orf1	chromosome 6 open reading frame 1 (C6orf1), transcript variant 1, mRNA	NM_178508	Hs.381300	CR592917
C6orf114	chromosome 6 open reading frame 114 (C6orf114), mRNA	NM_033069	Hs.484686	AK000337
C6orf150	chromosome 6 open reading frame 150 (C6orf150), mRNA	NM_138441	Hs.658405	BC113606
C6orf188	chromosome 6 open reading frame 188 (C6orf188), mRNA	NM_153711	Hs.660142	NM_153711
C6orf188	chromosome 6 open reading frame 188 (C6orf188), mRNA	NM_153711	Hs.660142	NM_153711
C6orf194	chromosome 6 open reading frame 194 (C6orf194), mRNA	NM_001007531	Hs.239181	NM_001007531
C6orf64	chromosome 6 open reading frame 64 (C6orf64), mRNA	NM_018322	Hs.58382	CR627244
C8orf37	chromosome 8 open reading frame 37 (C8orf37), mRNA	NM_177965	Hs.548157	BC150301
C8orf4	chromosome 8 open reading frame 4 (C8orf4), mRNA	NM_020130	Hs.591849	NM_020130
C8orf55	chromosome 8 open reading frame 55 (C8orf55), mRNA	NM_016647	Hs.368402	BC001311
C8orf58	chromosome 8 open reading frame 58 (C8orf58), mRNA	NM_001013842	Hs.553539	NM_001013842
C9orf156	chromosome 9 open reading frame 156 (C9orf156), mRNA	NM_016481	Hs.9196	AY189280
C9orf156	chromosome 9 open reading frame 156 (C9orf156), mRNA	NM_016481	Hs.9196	AY189280
C9orf21	chromosome 9 open reading frame 21 (C9orf21), mRNA	NM_153698	Hs.44640	NM_153698
C9orf3	chromosome 9 open reading frame 3 (C9orf3), mRNA	NM_032823	Hs.434253	AF043897
C9orf30	chromosome 9 open reading frame 30 (C9orf30), mRNA	NM_080655	Hs.530272	AK092292
C9orf30	chromosome 9 open reading frame 30 (C9orf30), mRNA	NM_080655	Hs.530272	AK092292
C9orf47	chromosome 9 open reading frame 47 (C9orf47), mRNA	NM_001001938	Hs.585118	AL832194
C9orf89	chromosome 9 open reading frame 89 (C9orf89), mRNA	NM_032310	Hs.434213	AK091611
C9orf94	cDNA FLJ35283 fis, clone PROST2007528.	AK092602	Unknown	
CA13	carbonic anhydrase XIII (CA13), mRNA	NM_198584	Hs.127189	NM_198584
CA441889	CA441889 UI-H-ED0-axl-n-03-0-UI.s1 NCI_CGAP_ED0 cDNA clone UI-H-ED0-axl-n-03-0-UI 3', mRNA sequence	CA441889	Hs.699278	NM_003831
CALCOCO2	calcium binding and coiled-coil domain 2 (CALCOCO2), mRNA	NM_005831	Hs.514920	NM_005831
CAPNS2	calpain, small subunit 2 (CAPNS2), mRNA	NM_032330	Hs.660027	BM914030
CAPZA2	capping protein (actin filament) muscle Z-line, alpha 2 (CAPZA2), mRNA	NM_006136	Hs.695918	NM_006136
CASP4	caspase 4, apoptosis-related cysteine peptidase (CASP4), transcript variant gamma, mRNA	NM_033306	Hs.138378	AL050391
CASP4	caspase 4, apoptosis-related cysteine peptidase (CASP4), transcript variant gamma, mRNA	NM_033306	Hs.138378	AL050391
CASP5	caspase 5, apoptosis-related cysteine peptidase (CASP5), mRNA	NM_004347	Hs.213327	NM_004347
CASP7	caspase 7, apoptosis-related cysteine peptidase (CASP7), transcript variant delta, mRNA	NM_033338	Hs.9216	NM_033338
CAST	calpastatin (CAST), transcript variant 1, mRNA	NM_001750	Hs.440961	NM_001750
CAT	catalase (CAT), mRNA	NM_001752	Hs.502302	NM_001752
CAV2	caveolin 2 (CAV2), transcript variant 1, mRNA	NM_001233	Hs.212332	NM_001233
CAV2	caveolin 2 (CAV2), transcript variant 1, mRNA	NM_001233	Hs.212332	NM_001233
CBARA1	calcium binding atopy-related autoantigen 1 (CBARA1), mRNA	NM_006077	Hs.524367	NM_006077
CBR1	carbonyl reductase 1 (CBR1), mRNA	NM_001757	Hs.88778	BM810059
CBR3	mRNA for carbonyl reductase, trapped exon B1084-9, partial.	AJ000096	Hs.154510	AB041012

CCDC116	coiled-coil domain containing 116 (CCDC116), mRNA	NM_152612	Hs.131615	NM_152612
CCDC123	coiled-coil domain containing 123 (CCDC123), mRNA	NM_032816	Hs.599703	AK001375
CCDC125	coiled-coil domain containing 125 (CCDC125), mRNA	NM_176816	Hs.654850	AL832658
CCDC132	coiled-coil domain containing 132 (CCDC132), transcript variant 2, mRNA	NM_024553	Hs.222282	NM_017667
CCDC22	coiled-coil domain containing 22 (CCDC22), mRNA	NM_014008	Hs.26333	NM_014008
CCDC32	coiled-coil domain containing 32 (CCDC32), mRNA	NM_052849	Hs.654661	AK097986
CCDC32	coiled-coil domain containing 32 (CCDC32), mRNA	NM_052849	Hs.654661	AK097986
CCDC47	coiled-coil domain containing 47 (CCDC47), mRNA	NM_020198	Hs.202011	NM_020198
CCDC50	coiled-coil domain containing 50 (CCDC50), transcript variant 2, mRNA	NM_178335	Hs.478682	AJ557013
CCDC50	coiled-coil domain containing 50 (CCDC50), transcript variant 2, mRNA	NM_178335	Hs.478682	AJ557013
CCDC50	coiled-coil domain containing 50 (CCDC50), transcript variant 2, mRNA	NM_178335	Hs.478682	AJ557013
CCDC85A	mRNA for KIAA1912 protein, partial cds.	ENST00000233161	Unknown	
CCDC90B	coiled-coil domain containing 90B (CCDC90B), mRNA	NM_021825	Hs.368866	AK055972
CCL2	chemokine (C-C motif) ligand 2 (CCL2), mRNA	NM_002982	Hs.303649	BU570769
CCL2	chemokine (C-C motif) ligand 2 (CCL2), mRNA	NM_002982	Hs.303649	BU570769
CCL2	chemokine (C-C motif) ligand 2 (CCL2), mRNA	NM_002982	Hs.303649	BU570769
CCL2	chemokine (C-C motif) ligand 2 (CCL2), mRNA	NM_002982	Hs.303649	BU570769
CCL2	chemokine (C-C motif) ligand 2 (CCL2), mRNA	NM_002982	Hs.303649	BU570769
CCL2	chemokine (C-C motif) ligand 2 (CCL2), mRNA	NM_002982	Hs.303649	BU570769
CCL2	chemokine (C-C motif) ligand 2 (CCL2), mRNA	NM_002982	Hs.303649	BU570769
CCL2	chemokine (C-C motif) ligand 2 (CCL2), mRNA	NM_002982	Hs.303649	BU570769
CCL2	chemokine (C-C motif) ligand 2 (CCL2), mRNA	NM_002982	Hs.303649	BU570769
CCL2	chemokine (C-C motif) ligand 2 (CCL2), mRNA	NM_002982	Hs.303649	BU570769
CCND3	cyclin D3 (CCND3), mRNA	NM_001760	Hs.534307	AL833425
CCR8	chemokine (C-C motif) receptor 8 (CCR8), mRNA	NM_005201	Hs.113222	NM_005201
CD2BP2	CD2 (cytoplasmic tail) binding protein 2 (CD2BP2), mRNA	NM_006110	Hs.700708	AB033004
CD302	CD302 molecule (CD302), mRNA	NM_014880	Hs.130014	AY314007
CD59	CD59 molecule, complement regulatory protein (CD59), transcript variant 1, mRNA	NM_203330	Hs.278573	NM_203330
CD59	CD59 molecule, complement regulatory protein (CD59), transcript variant 1, mRNA	NM_203330	Hs.278573	NM_203330
CD59	CD59 molecule, complement regulatory protein (CD59), transcript variant 1, mRNA	NM_203330	Hs.278573	NM_203330
CD82	CD82 molecule (CD82), transcript variant 1, mRNA	NM_002231	Hs.527778	NM_002231
CDC42	cell division cycle 42 (GTP binding protein, 25kDa) (CDC42), transcript variant 2, mRNA	NM_044472	Hs.690198	NM_001039802
CDC42	cell division cycle 42 (GTP binding protein, 25kDa) (CDC42), transcript variant 2, mRNA	NM_044472	Hs.690198	NM_001039802
CDC42	cell division cycle 42 (GTP binding protein, 25kDa) (CDC42), transcript variant 2, mRNA	NM_044472	Hs.690198	NM_001039802
CDC42	cell division cycle 42 (GTP binding protein, 25kDa) (CDC42), transcript variant 3, mRNA	NM_001039802	Hs.690198	NM_001039802
CDC42	cell division cycle 42 (GTP binding protein, 25kDa) (CDC42), transcript variant 2, mRNA	NM_044472	Hs.690198	NM_001039802
CDC42	cell division cycle 42 (GTP binding protein, 25kDa) (CDC42), transcript variant 2, mRNA	NM_044472	Hs.690198	NM_001039802
CDC42	cell division cycle 42 (GTP binding protein, 25kDa) (CDC42), transcript variant 2, mRNA	NM_044472	Hs.690198	NM_001039802
CDC42	cell division cycle 42 (GTP binding protein, 25kDa) (CDC42), transcript variant 2, mRNA	NM_044472	Hs.690198	NM_001039802
CDC42	cell division cycle 42 (GTP binding protein, 25kDa) (CDC42), transcript variant 2, mRNA	NM_044472	Hs.690198	NM_001039802
CDC42	cell division cycle 42 (GTP binding protein, 25kDa) (CDC42), transcript variant 2, mRNA	NM_044472	Hs.690198	NM_001039802
CDC42	cell division cycle 42 (GTP binding protein, 25kDa) (CDC42), transcript variant 2, mRNA	NM_044472	Hs.690198	NM_001039802
CDGAP	Cdc42 GTPase-activating protein (CDGAP), mRNA	NM_020754	Hs.657263	AB033030
CDH13	cadherin 13, H-cadherin (heart) (CDH13), mRNA	NM_001257	Hs.654386	NM_001257
CF527929	CF527929 UI-1-BC0-aea-g-12-0-UI.s1 NCI_CGAP_Pl1 cDNA clone UI-1-BC0-aea-g-12-0-UI 3', mRNA sequence	CF527929	Hs.445999	AK026669
CFLAR	CASP8 and FADD-like apoptosis regulator (CFLAR), mRNA	NM_003879	Hs.390736	AB209600
CHCHD2	coiled-coil-helix-coiled-coil-helix domain containing 2 (CHCHD2), mRNA	NM_016139	Hs.389996	BM553650
CHCHD2	coiled-coil-helix-coiled-coil-helix domain containing 2 (CHCHD2), mRNA	NM_016139	Hs.389996	BM553650
CHCHD2	coiled-coil-helix-coiled-coil-helix domain containing 2 (CHCHD2), mRNA	NM_016139	Hs.389996	BM553650
CHCHD2	coiled-coil-helix-coiled-coil-helix domain containing 2 (CHCHD2), mRNA	NM_016139	Hs.389996	BM553650
ChGn	chondroitin beta1,4 N-acetylgalactosaminyltransferase (ChGn), mRNA	NM_018371	Hs.655166	BX649103
CHIC2	cysteine-rich hydrophobic domain 2 (CHIC2), mRNA	NM_012110	Hs.335393	AK130041

CHKB	choline kinase beta (CHKB), transcript variant 1, mRNA	NM_005198	Hs.654827	BC037162
CHM	choroideremia (Rab escort protein 1) (CHM), transcript variant 1, mRNA	NM_000390	Hs.496449	NM_000390
CHMP2A	chromatin modifying protein 2A (CHMP2A), transcript variant 1, mRNA	NM_014453	Hs.12107	BM542499
CHMP4A	chromatin modifying protein 4A (CHMP4A), mRNA	NM_014169	Hs.279761	AK094345
CHMP4A	chromatin modifying protein 4A (CHMP4A), mRNA	NM_014169	Hs.279761	AK094345
CHMP4B	chromatin modifying protein 4B (CHMP4B), mRNA	NM_176812	Hs.472471	BU150315
CHMP5	chromatin modifying protein 5 (CHMP5), mRNA	NM_016410	Hs.635313	BF790759
CHMP6	clone PP552 unknown mRNA.	AF218021	Hs.675838	AF218021
CHPT1	choline phosphotransferase 1 (CHPT1), mRNA	NM_020244	Hs.293077	AK226127
CHST1	carbohydrate (keratan sulfate Gal-6) sulfotransferase 1 (CHST1), mRNA	NM_003654	Hs.104576	BC022567
CIDEC	cell death-inducing DFFA-like effector c (CIDEC), mRNA	NM_022094	Hs.567562	NM_022094
CKAP4	cytoskeleton-associated protein 4 (CKAP4), mRNA	NM_006825	Hs.74368	NM_006825
CLCA1	chloride channel, calcium activated, family member 1 (CLCA1), mRNA	NM_001285	Hs.194659	AF039400
CLCA3	chloride channel, calcium activated, family member 3 (CLCA3), mRNA	NM_004921	Hs.673847	NM_004921
CLCF1	cardiotrophin-like cytokine factor 1 (CLCF1), mRNA	NM_013246	Hs.502977	NM_013246
CLCN4	chloride channel 4 (CLCN4), mRNA	NM_001830	Hs.495674	NM_001830
CLIC3	chloride intracellular channel 3 (CLIC3), mRNA	NM_004669	Hs.64746	BQ960229
CLK3	CDC-like kinase 3 (CLK3), transcript variant phclk3, mRNA	NM_003992	Hs.584748	CR933693
CMAH	cytidine monophosphate-N-acetylneuraminic acid hydroxylase (CMP-N-acetylneuraminate monooxygenase) (CMAH) on chromosome 6	NR_002174	Unknown	
CMTM3	CKLF-like MARVEL transmembrane domain containing 3 (CMTM3), transcript variant 1, mRNA	NM_144601	Hs.298198	AK056324
COBLL1	COBL-like 1, mRNA (cDNA clone IMAGE:3951455), complete cds.	BC006264	Hs.470457	BX649112
COBLL1	COBL-like 1 (COBLL1), mRNA	NM_014900	Hs.470457	BX649112
COG5	component of oligomeric golgi complex 5 (COG5), transcript variant 1, mRNA	NM_006348	Hs.239631	NM_006348
COMMD1	copper metabolism (Murr1) domain containing 1 (COMMD1), mRNA	NM_152516	Hs.468702	BQ054214
COMMD9	COMM domain containing 9 (COMMD9), mRNA	NM_014186	Hs.279836	AK055668
COPZ1	coatomer protein complex, subunit zeta 1 (COPZ1), mRNA	NM_016057	Hs.505652	AK025956
COQ4	coenzyme Q4 homolog (S. cerevisiae) (COQ4), mRNA	NM_016035	Hs.98541	AK128853
CORO1C	coronin, actin binding protein, 1C (CORO1C), mRNA	NM_014325	Hs.681389	NM_014325
COTL1	coactosin-like 1 (Dictyostelium) (COTL1), mRNA	NM_021149	Hs.289092	AK093712
COX4I1	cytochrome c oxidase subunit IV isoform 1 (COX4I1), mRNA	NM_001861	Hs.433419	BC047869
CR597075	full-length cDNA clone CS0DF037YI18 of Fetal brain of (human).	CR597075	Hs.532824	BC007318
CR597807	full-length cDNA clone CS0DC020YN03 of Neuroblastoma Cot 25-normalized of (human).	CR597807	Hs.306083	AK095636
CR601322	full-length cDNA clone CS0DI015YJ05 of Placenta Cot 25-normalized of (human).	CR601322	Hs.662205	BM552788
CR601567	full-length cDNA clone CS0DN003YI06 of Adult brain of (human).	CR601567	Hs.651229	NM_138350
CR602075	full-length cDNA clone CS0DD009YF23 of Neuroblastoma Cot 50-normalized of (human).	CR602075	Hs.570180	BX355423
CR602702	full-length cDNA clone CS0DI063YN06 of Placenta Cot 25-normalized of (human).	CR602702	Hs.521817	CR598768
CR603195	full-length cDNA clone CS0DL012YE01 of B cells (Ramos cell line) Cot 25-normalized of (human).	CR603195	Hs.655744	AK056312
CR604283	full-length cDNA clone CS0DF012YB15 of Fetal brain of (human).	CR604283	Hs.660633	CR604283
CR606637	full-length cDNA clone CS0DI007YA21 of Placenta Cot 25-normalized of (human).	CR606637	Hs.700799	BC027873
CR622189	full-length cDNA clone CS0DI081YP18 of Placenta Cot 25-normalized of (human).	CR622189	Hs.149363	NM_001003674
CR623684	full-length cDNA clone CS0DK004YM05 of HeLa cells Cot 25-normalized of (human).	CR623684	Hs.355809	XM_087225
CR625571	full-length cDNA clone CS0DD008YI13 of Neuroblastoma Cot 50-normalized of (human).	CR625571	Hs.647264	BU839374
CRCT1	cysteine-rich C-terminal 1 (CRCT1), mRNA	NM_019060	Hs.110196	AJ243662
CREB3	cAMP responsive element binding protein 3 (CREB3), mRNA	NM_006368	Hs.522110	NM_006368
CREB5	cAMP responsive element binding protein 5 (CREB5), transcript variant 1, mRNA	NM_182898	Hs.437075	NM_182898
CREM	cAMP responsive element modulator (CREM), transcript variant 2, mRNA	NM_001881	Hs.200250	AB209533
CRIM1	cysteine rich transmembrane BMP regulator 1 (chordin-like) (CRIM1), mRNA	NM_016441	Hs.699247	AF167706
CRIM1	cysteine rich transmembrane BMP regulator 1 (chordin-like) (CRIM1), mRNA	NM_016441	Hs.699247	AF167706
CROCC	ciliary rootlet coiled-coil, rootletin (CROCC), mRNA	NM_014675	Hs.309403	NM_014675
CRYZ	crystallin, zeta (quinone reductase) (CRYZ), mRNA	NM_001889	Hs.83114	BX649061
CSNK1A1	casein kinase 1, alpha 1 (CSNK1A1), transcript variant 1, mRNA	NM_001025105	Hs.699188	NM_001025105

CSNK1G1	casein kinase 1, gamma 1 (CSNK1G1), mRNA	NM_022048	Hs.646508	NM_022048
CSRP1	cysteine and glycine-rich protein 1 (CSRP1), mRNA	NM_004078	Hs.108080	CR627407
CSTB	cystatin B (stefin B) (CSTB), mRNA	NM_000100	Hs.695	CR591371
CTSB	cathepsin B (CTSB), transcript variant 2, mRNA	NM_147780	Hs.520898	NM_147780
CTSO	cathepsin O (CTSO), mRNA	NM_001334	Hs.75262	NM_001334
CTSS	cathepsin S (CTSS), mRNA	NM_004079	Hs.181301	NM_004079
CTSZ	cathepsin Z (CTSZ), mRNA	NM_001336	Hs.252549	NM_001336
CUEDC2	CUE domain containing 2 (CUEDC2), mRNA	NM_024040	Hs.500874	AK094695
CV323908	CV323908 CM4-CN0096-131200-614-d05 CN0096 cDNA, mRNA sequence	CV323908	Hs.592490	U59309
CXCL1	chemokine (C-X-C motif) ligand 1 (melanoma growth stimulating activity, alpha) (CXCL1), mRNA	NM_001511	Hs.789	BF032655
CYB5D2	cytochrome b5 domain containing 2 (CYB5D2), mRNA	NM_144611	Hs.513871	BC051697
CYB5D2	cytochrome b5 domain containing 2 (CYB5D2), mRNA	NM_144611	Hs.513871	BC051697
CYBRD1	cytochrome b reductase 1 (CYBRD1), mRNA	NM_024843	Hs.221941	AL136693
CYLD	cylindromatosis (turban tumor syndrome) (CYLD), transcript variant 1, mRNA	NM_015247	Hs.578973	NM_015247
CYLD	cylindromatosis (turban tumor syndrome) (CYLD), transcript variant 1, mRNA	NM_015247	Hs.578973	NM_015247
CYP11B1	cytochrome P450, family 11, subfamily B, polypeptide 1 (CYP11B1), nuclear gene encoding mitochondrial protein, transcript variant 1, mRNA	NM_000497	Hs.184927	NM_000497
CYP26B1	cytochrome P450, family 26, subfamily B, polypeptide 1 (CYP26B1), mRNA	NM_019885	Hs.91546	NM_019885
CYR61	cysteine-rich, angiogenic inducer, 61 (CYR61), mRNA	NM_001554	Hs.8867	Y11307
CYR61	cysteine-rich, angiogenic inducer, 61 (CYR61), mRNA	NM_001554	Hs.8867	Y11307
CYR61	cysteine-rich, angiogenic inducer, 61 (CYR61), mRNA	NM_001554	Hs.8867	Y11307
DA292134	DA292134 BRHIP2 cDNA clone BRHIP2003939 5', mRNA sequence	DA292134	Hs.627735	DA292134
DAP	death-associated protein (DAP), mRNA	NM_004394	Hs.75189	NM_004394
DB301332	DB301332 BRAMY1 cDNA clone BRAMY1000317 3', mRNA sequence	DB301332	Hs.631183	DB301332
DB380247	DB380247 PLACE1 cDNA clone PLACE1011259 3', mRNA sequence	DB380247	Hs.693756	DB380247
DB518505	DB518505 RIKEN full-length enriched human cDNA library, testis cDNA clone H013059P17 3', mRNA sequence	DB518505	Hs.701927	DB518505
DB728175	DB728175 RIKEN full-length enriched human cDNA library, hypothalamus cDNA clone H033001B22 3', mRNA sequence	DB728175	Hs.645207	DB728175
DCTN2	Dynactin subunit 2 (Dynactin complex 50 kDa subunit) (50 kDa dynein- associated polypeptide) (p50 dynamitin) (DCTN-50).	ENST00000354743	Unknown	
DCTN3	dynactin 3 (p22) (DCTN3), transcript variant 1, mRNA	NM_007234	Hs.511768	BM920638
DDEF1	development and differentiation enhancing factor 1 (DDEF1), mRNA	NM_018482	Hs.655552	NM_018482
DDHD2	DDHD domain containing 2 (DDHD2), mRNA	NM_015214	Hs.434966	AK125904
DDIT4	DNA-damage-inducible transcript 4 (DDIT4), mRNA	NM_019058	Hs.523012	AF335324
DDR2	discoidin domain receptor family, member 2 (DDR2), transcript variant 1, mRNA	NM_001014796	Hs.593833	BC052998
DENND4A	DENN/MADD domain containing 4A (DENND4A), mRNA	NM_005848	Hs.654567	AL833317
DENND4A	DENN/MADD domain containing 4A (DENND4A), mRNA	NM_005848	Hs.654567	AL833317
DERL2	Der1-like domain family, member 2 (DERL2), mRNA	NM_016041	Hs.286131	BM478493
DFNA5	deafness, autosomal dominant 5 (DFNA5), mRNA	NM_004403	Hs.520708	AK094714
DGKH	cDNA clone IMAGE:4823178, containing frame-shift errors.	BC044822	Hs.659437	BC044822
DGKH	diacylglycerol kinase, eta (DGKH), transcript variant 1, mRNA	NM_152910	Hs.659437	BC044822
DHRS3	dehydrogenase/reductase (SDR family) member 3 (DHRS3), mRNA	NM_004753	Hs.289347	NM_004753
DHRS7	dehydrogenase/reductase (SDR family) member 7 (DHRS7), mRNA	NM_016029	Hs.59719	BU541074
DHRS7B	dehydrogenase/reductase (SDR family) member 7B (DHRS7B), mRNA	NM_015510	Hs.386989	AK124055
DHRS7B	dehydrogenase/reductase (SDR family) member 7B (DHRS7B), mRNA	NM_015510	Hs.386989	AK124055
DHRS7B	dehydrogenase/reductase (SDR family) member 7B (DHRS7B), mRNA	NM_015510	Hs.386989	AK124055
DIP2B	DIP2 disco-interacting protein 2 homolog B (Drosophila) (DIP2B), mRNA	NM_173602	Hs.505516	NM_173602
DIXDC1	DIX domain containing 1 (DIXDC1), transcript variant 1, mRNA	NM_001037954	Hs.655626	NM_001037954
DKFZp313P036	mRNA; cDNA DKFZp313P036 (from clone DKFZp313P036).	BX537874	Hs.451336	BX537874
DKFZP564O052	hypothetical protein DKFZp564O0523 (DKFZP564O0523), mRNA	NM_032120	Hs.21590	CR936726
DKFZp779O175	hypothetical protein DKFZp779O175 (DKFZp779O175), mRNA	NM_001037232	Hs.124047	BX538288
DKK1	dickkopf homolog 1 (Xenopus laevis) (DKK1), mRNA	NM_012242	Hs.40499	CR594190
DKK3	dickkopf homolog 3 (Xenopus laevis) (DKK3), transcript variant 1, mRNA	NM_015881	Hs.292156	NM_015881

DKK3	dickkopf homolog 3 (Xenopus laevis) (DKK3), transcript variant 1, mRNA	NM_015881	Hs.292156	NM_015881
DLC1	deleted in liver cancer 1 (DLC1), transcript variant 1, mRNA	NM_182643	Hs.134296	NM_182643
DLGAP4	discs, large (Drosophila) homolog-associated protein 4 (DLGAP4), transcript variant 1, mRNA	NM_014902	Hs.249600	NM_014902
DNAJA4	DnaJ (Hsp40) homolog, subfamily A, member 4 (DNAJA4), mRNA	NM_018602	Hs.513053	NM_018602
DNAJA4	PRO1472 mRNA, complete cds.	AF116663	Unknown	
DNAJA4	DnaJ (Hsp40) homolog, subfamily A, member 4 (DNAJA4), mRNA	NM_018602	Hs.513053	NM_018602
DNAJB12	DnaJ (Hsp40) homolog, subfamily B, member 12 (DNAJB12), transcript variant 1, mRNA	NM_001002762	Hs.696014	NM_001002762
DNAJB12	DnaJ (Hsp40) homolog, subfamily B, member 12 (DNAJB12), transcript variant 1, mRNA	NM_001002762	Hs.696014	NM_001002762
DNAJC5	DnaJ homolog subfamily C member 5 (Cysteine string protein) (CSP).	ENST00000369914	Unknown	
DNM1L	dynamin 1-like (DNM1L), transcript variant 1, mRNA	NM_012062	Hs.556296	AB209070
DOK5	docking protein 5 (DOK5), mRNA	NM_018431	Hs.656582	CR601525
DOPEY2	dopey family member 2 (DOPEY2), mRNA	NM_005128	Hs.204575	NM_005128
DPP8	dipeptidyl-peptidase 8 (DPP8), transcript variant 1, mRNA	NM_130434	Hs.591106	BC040203
DPYD	dihydropyrimidine dehydrogenase (DPYD), mRNA	NM_000110	Hs.335034	NM_000110
DRAM	damage-regulated autophagy modulator (DRAM), mRNA	NM_018370	Hs.525634	NM_018370
DRAM	damage-regulated autophagy modulator (DRAM), mRNA	NM_018370	Hs.525634	NM_018370
DRAP1	DR1-associated protein 1 (negative cofactor 2 alpha) (DRAP1), mRNA	NM_006442	Hs.356742	BF969132
DRAP1	DR1-associated protein 1 (negative cofactor 2 alpha) (DRAP1), mRNA	NM_006442	Hs.356742	BF969132
DSCR8	Down syndrome critical region gene 8 (DSCR8), transcript variant 3, mRNA	NM_203429	Unknown	
DUSP11	dual specificity phosphatase 11 (RNA/RNP complex 1-interacting) (DUSP11), mRNA	NM_003584	Hs.14611	CR627368
DUSP14	dual specificity phosphatase 14 (DUSP14), mRNA	NM_007026	Hs.91448	AK027210
DUSP18	dual specificity phosphatase 18 (DUSP18), mRNA	NM_152511	Hs.517544	BC028724
DUSP19	dual specificity phosphatase 19 (DUSP19), mRNA	NM_080876	Hs.132237	NM_080876
DYNLRB1	dynein, light chain, roadblock-type 1 (DYNLRB1), mRNA	NM_014183	Hs.593920	AK097281
EDG1	endothelial differentiation, sphingolipid G-protein-coupled receptor, 1 (EDG1), mRNA	NM_001400	Hs.154210	NM_001400
EDN1	endothelin 1 (EDN1), mRNA	NM_001955	Hs.511899	NM_001955
EDN1	endothelin 1 (EDN1), mRNA	NM_001955	Hs.511899	NM_001955
EDN1	endothelin 1 (EDN1), mRNA	NM_001955	Hs.511899	NM_001955
EDN1	endothelin 1 (EDN1), mRNA	NM_001955	Hs.511899	NM_001955
EDN1	endothelin 1 (EDN1), mRNA	NM_001955	Hs.511899	NM_001955
EDN1	endothelin 1 (EDN1), mRNA	NM_001955	Hs.511899	NM_001955
EDN1	endothelin 1 (EDN1), mRNA	NM_001955	Hs.511899	NM_001955
EDN1	endothelin 1 (EDN1), mRNA	NM_001955	Hs.511899	NM_001955
EDN1	endothelin 1 (EDN1), mRNA	NM_001955	Hs.511899	NM_001955
EDN1	endothelin 1 (EDN1), mRNA	NM_001955	Hs.511899	NM_001955
EEA1	early endosome antigen 1, 162kD (EEA1), mRNA	NM_003566	Hs.567367	NM_003566
EFEMP1	EGF-containing fibulin-like extracellular matrix protein 1 (EFEMP1), transcript variant 1, mRNA	NM_004105	Hs.76224	NM_004105
EGFR	epidermal growth factor receptor (erythroblastic leukemia viral (v-erb-b) oncogene homolog, avian) (EGFR), transcript variant 1, mRNA	NM_005228	Hs.488293	NM_005228
EGFR	epidermal growth factor receptor (erythroblastic leukemia viral (v-erb-b) oncogene homolog, avian) (EGFR), transcript variant 1, mRNA	NM_005228	Hs.488293	NM_005228
EGLN1	egl nine homolog 1 (C. elegans) (EGLN1), mRNA	NM_022051	Hs.444450	AF229245
EGLN1	egl nine homolog 1 (C. elegans) (EGLN1), mRNA	NM_022051	Hs.444450	AF229245
EHD1	EH-domain containing protein testilin mRNA, complete cds.	AF099011	Unknown	
EHD1	EH-domain containing 1 (EHD1), mRNA	NM_006795	Hs.523774	AK124613
EHD2	EH-domain containing 2 (EHD2), mRNA	NM_014601	Hs.631554	BC062554
EHD2	EH-domain containing 2 (EHD2), mRNA	NM_014601	Hs.631554	BC062554
EHHADH	enoyl-Coenzyme A, hydratase/3-hydroxyacyl Coenzyme A dehydrogenase (EHHADH), mRNA	NM_001966	Hs.429879	NM_001966
EID3	EP300 interacting inhibitor of differentiation 3 (EID3), mRNA	NM_001008394	Hs.659857	AK098698
EMP1	epithelial membrane protein 1 (EMP1), mRNA	NM_001423	Hs.696241	NM_001423
ENDOD1	Endonuclease domain-containing 1 protein precursor (EC 3.1.30.-).	ENST00000278505	Unknown	
ENDOD1	Endonuclease domain-containing 1 protein precursor (EC 3.1.30.-).	ENST00000278505	Unknown	
ENDOGL1	endonuclease G-like 1 (ENDOGL1), mRNA	NM_005107	Hs.517897	AK023235

ENO1B	H.sapiens mRNA for enolase.	X66610	Unknown	
ENST00000261	Microtubule-associated serine/threonine-protein kinase 4 (EC 2.7.11.1).	ENST00000261569	Unknown	
ENST00000262	PREDICTED: similar to Transcription factor Ovo-like 2 (hOvo2) (Zinc finger protein 339) (LOC728361), mRNA	ENST00000262637	Unknown	
ENST00000269	HSPC254 mRNA, partial cds.	ENST00000269290	Unknown	
ENST00000273	full-length cDNA clone CS0DI044YK15 of Placenta Cot 25-normalized of (human).	ENST00000273582	Unknown	
ENST00000273	CDNA FLJ33534 fis, clone BRAMY2007411.	ENST00000273641	Unknown	
ENST00000299	MGC9913 protein.	ENST00000299997	Unknown	
ENST00000304	Olfactory receptor 7A2.	ENST00000304105	Unknown	
ENST00000317	cDNA FLJ40995 fis, clone UTERU2015830.	ENST00000317427	Unknown	
ENST00000321	CDNA FLJ90757 fis, clone SKNMC1000014 (FLJ90757 protein).	ENST00000321795	Unknown	
ENST00000322	CDNA FLJ33768 fis, clone BRHIP2000021 (Hypothetical protein TMEM84).	ENST00000322370	Unknown	
ENST00000324	hypothetical protein LOC375196, mRNA (cDNA clone IMAGE:4792618), partial cds.	ENST00000324414	Unknown	
ENST00000324	Protein phosphatase Slingshot homolog 2 (EC 3.1.3.48) (EC 3.1.3.16) (SSH-2L) (hSSH-2L).	ENST00000324677	Unknown	
ENST00000327	GB|AK090480.1|BAC03461.1 FLJ00402 protein	ENST00000327469	Unknown	
ENST00000328	chromosome 13 open reading frame 29, mRNA (cDNA clone MGC:120939 IMAGE:7939749), complete cds.	ENST00000328711	Unknown	
ENST00000331	HUMIGKPB Ig kappa chain {Homo sapiens} (exp=-1; wgp=0; cg=0), complete	ENST00000331696	Unknown	
ENST00000332	HGSNAT protein.	ENST00000332689	Unknown	
ENST00000333	PREDICTED: similar to 60S ribosomal protein L23a (LOC391282), mRNA	ENST00000333546	Unknown	
ENST00000338	FA86A_HUMAN (Q96G04) Protein FAM86A, partial (47%)	ENST00000338711	Unknown	
ENST00000354	Leucine-rich repeats and calponin homology domain-containing protein 3 precursor.	ENST00000354261	Unknown	
ENST00000354	UI-E-CQ1-afy-d-12-0-UI.r1 UI-E-CQ1 cDNA clone UI-E-CQ1-afy-d-12-0-UI 5', mRNA sequence	ENST00000354343	Unknown	
ENST00000355	ZNF252 protein (Fragment).	ENST00000355436	Unknown	
ENST00000356	T-cell receptor beta chain C region.	ENST00000356506	Unknown	
ENST00000358	similar to 60S ribosomal protein L22 (Heparin-binding protein HBp15) (LOC652429), mRNA	ENST00000358916	Unknown	
ENST00000360	lines homolog 1	ENST00000360903	Unknown	
ENST00000368	Trichohyalin.	ENST00000368804	Unknown	
ENST00000377	FAM27E1 protein.	ENST00000377492	Unknown	
ENST00000378	CDNA FLJ39622 fis, clone SMINT2001199.	ENST00000378953	Unknown	
EPAS1	endothelial PAS domain protein 1 (EPAS1), mRNA	NM_001430	Hs.468410	NM_001430
EPB41L3	erythrocyte membrane protein band 4.1-like 3 (EPB41L3), mRNA	NM_012307	Hs.213394	AL832598
EPHA5	mRNA; cDNA DKFZp686C0686 (from clone DKFZp686C0686); complete cds.	BX537946	Hs.654492	BX537946
EPHB2	EPH receptor B2 (EPHB2), transcript variant 2, mRNA	NM_004442	Hs.523329	NM_004442
ERCC1	excision repair cross-complementing rodent repair deficiency, complementation group 1 (includes overlapping antisense sequence) (ERCC1), transcript variant 2, mRNA	NM_001983	Hs.435981	AK092039
ERGIC2	ERGIC and golgi 2 (ERGIC2), mRNA	NM_016570	Hs.339453	AL834128
ERGIC2	ERGIC and golgi 2 (ERGIC2), mRNA	NM_016570	Hs.339453	AL834128
ERO1L	ERO1-like (S. cerevisiae) (ERO1L), mRNA	NM_014584	Hs.592304	AF081886
ESR1	estrogen receptor 1 (ESR1), mRNA	NM_000125	Hs.208124	NM_000125
ESR1	estrogen receptor 1 (ESR1), mRNA	NM_000125	Hs.208124	NM_000125
ESR1	estrogen receptor 1 (ESR1), mRNA	NM_000125	Hs.208124	NM_000125
ESR1	estrogen receptor 1 (ESR1), mRNA	NM_000125	Hs.208124	NM_000125
ETHE1	ethylmalonic encephalopathy 1 (ETHE1), mRNA	NM_014297	Hs.7486	CB993249
ETV5	ets variant gene 5 (ets-related molecule) (ETV5), mRNA	NM_004454	Hs.43697	NM_004454
ETV6	ets variant gene 6 (TEL oncogene) (ETV6), mRNA	NM_001987	Hs.504765	NM_001987
F25965	protein F25965 (F25965), mRNA	NM_019104	Hs.529100	CR612042
FAHD1	fumarylacetoacetate hydrolase domain containing 1 (FAHD1), transcript variant 2, mRNA	NM_031208	Hs.513265	AL136720
FAM101A	family with sequence similarity 101, member A (FAM101A), mRNA	NM_181709	Hs.432901	NM_181709
FAM114A1	family with sequence similarity 114, member A1 (FAM114A1), mRNA	ENST00000381942	Unknown	
FAM114A1	family with sequence similarity 114, member A1 (FAM114A1), mRNA	NM_138389	Hs.476517	AK094179
FAM11B	family with sequence similarity 11, member B (FAM11B) on chromosome 2	NR_000034	Unknown	
FAM125B	family with sequence similarity 125, member B (FAM125B), transcript variant 2, mRNA	NM_001011703	Hs.162659	NM_033446
FAM127B	family with sequence similarity 127, member B (FAM127B), mRNA	NM_001078172	Hs.460924	NM_001078172

FAM18B	family with sequence similarity 18, member B (FAM18B), mRNA	NM_016078	Hs.87295	AK128119
FAM20B	family with sequence similarity 20, member B (FAM20B), mRNA	NM_014864	Hs.5737	AB007944
FAM21C	family with sequence similarity 21, member C (FAM21C), mRNA	NM_015262	Hs.365286	AB011164
FAM27E1	family with sequence similarity 27, member E1 (FAM27E1), mRNA	NM_001024608	Unknown	
FAM32A	family with sequence similarity 32, member A (FAM32A), mRNA	NM_014077	Hs.631614	BM923512
FAM3C	family with sequence similarity 3, member C (FAM3C), transcript variant 1, mRNA	NM_014888	Hs.434053	BC024200
FAM41C	family with sequence similarity 41, member C, mRNA (cDNA clone IMAGE:5201580), partial cds.	BC047940	Hs.449006	BC047940
FAM44A	family with sequence similarity 44, member A, mRNA (cDNA clone IMAGE:5503684), with apparent retained intron.	BC043603	Hs.444517	NM_148894
FAM50B	family with sequence similarity 50, member B (FAM50B), mRNA	NM_012135	Hs.140944	BC001261
FAM62B	family with sequence similarity 62 (C2 domain containing) member B (FAM62B), mRNA	NM_020728	Hs.490795	AY368150
FAM65A	family with sequence similarity 65, member A (FAM65A), mRNA	NM_024519	Hs.152717	AK127792
FAM86A	family with sequence similarity 86, member A (FAM86A), transcript variant 1, mRNA	NM_201400	Hs.406461	BC010084
FAM91A1	family with sequence similarity 91, member A1 (FAM91A1), mRNA	NM_144963	Hs.459174	AL832999
FAM98C	family with sequence similarity 98, member C (FAM98C), mRNA	NM_174905	Hs.355162	BC036482
FAS	Fas (TNF receptor superfamily, member 6) (FAS), transcript variant 1, mRNA	NM_000043	Hs.244139	AB209361
FAS	Fas (TNF receptor superfamily, member 6) (FAS), transcript variant 1, mRNA	NM_000043	Hs.244139	AB209361
FAS	Fas (TNF receptor superfamily, member 6) (FAS), transcript variant 1, mRNA	NM_000043	Hs.244139	AB209361
FAS	Fas (TNF receptor superfamily, member 6) (FAS), transcript variant 1, mRNA	NM_000043	Hs.244139	AB209361
FAS	Fas (TNF receptor superfamily, member 6) (FAS), transcript variant 1, mRNA	NM_000043	Hs.244139	AB209361
FAS	Fas (TNF receptor superfamily, member 6) (FAS), transcript variant 1, mRNA	NM_000043	Hs.244139	AB209361
FAS	Fas (TNF receptor superfamily, member 6) (FAS), transcript variant 1, mRNA	NM_000043	Hs.244139	AB209361
FAS	Fas (TNF receptor superfamily, member 6) (FAS), transcript variant 1, mRNA	NM_000043	Hs.244139	AB209361
FAS	Fas (TNF receptor superfamily, member 6) (FAS), transcript variant 1, mRNA	NM_000043	Hs.244139	AB209361
FAS	Fas (TNF receptor superfamily, member 6) (FAS), transcript variant 1, mRNA	NM_000043	Hs.244139	AB209361
FBXO31	F-box protein 31 (FBXO31), mRNA	NM_024735	Hs.567582	AF318348
FBXO34	CGI-301 protein mRNA, complete cds.	AF531436	Hs.654725	AL834124
FBXW9	F-box and WD-40 domain protein 9 (FBXW9), mRNA	NM_032301	Hs.515154	AK093207
FCGR2B	Low affinity immunoglobulin gamma Fc region receptor II-b precursor (Fc-gamma RII-b) (FcRII-b) (IgG Fc receptor II-b) (Fc-gamma-RIIb) (CD32 antigen) (CDw32).	ENST00000367960	Unknown	
FCGRT	Fc fragment of IgG, receptor, transporter, alpha (FCGRT), mRNA	NM_004107	Hs.111903	AK074734
FCHSD2	FCH and double SH3 domains 2 (FCHSD2), mRNA	NM_014824	Hs.577053	AB018312
FCRLB	Fc receptor-like B (FCRLB), mRNA	NM_001002901	Hs.517422	BC067080
FDPS	farnesyl diphosphate synthase (farnesyl pyrophosphate synthetase, dimethylallyltranstransferase, geranyltranstransferase) (FDPS), mRNA	NM_002004	Hs.335918	AK021828
FDX1	ferredoxin 1 (FDX1), nuclear gene encoding mitochondrial protein, mRNA	NM_004109	Hs.744	NM_004109
FEM1C	fem-1 homolog c (C. elegans) (FEM1C), mRNA	NM_020177	Hs.47367	NM_020177
FEM1C	fem-1 homolog c (C. elegans) (FEM1C), mRNA	NM_020177	Hs.47367	NM_020177
FER1L3	fer-1-like 3, myoferlin (C. elegans) (FER1L3), transcript variant 2, mRNA	NM_133337	Hs.655278	AF182316
FER1L3	fer-1-like 3, myoferlin (C. elegans) (FER1L3), transcript variant 1, mRNA	NM_013451	Hs.655278	AF182316
FER1L3	fer-1-like 3, myoferlin (C. elegans) (FER1L3), transcript variant 1, mRNA	NM_013451	Hs.655278	AF182316
FEV	FEV (ETS oncogene family) (FEV), mRNA	NM_017521	Hs.234759	NM_017521
FGF5	fibroblast growth factor 5 (FGF5), transcript variant 1, mRNA	NM_004464	Hs.37055	NM_004464
FGF5	fibroblast growth factor 5 (FGF5), transcript variant 1, mRNA	NM_004464	Hs.37055	NM_004464
FGFR1OP2	FGFR1 oncogene partner 2 (FGFR1OP2), mRNA	NM_015633	Hs.591162	AK094888
FHL2	four and a half LIM domains 2 (FHL2), transcript variant 5, mRNA	NM_001039492	Hs.443687	CR936799
FIS1	fission 1 (mitochondrial outer membrane) homolog (S. cerevisiae) (FIS1), mRNA	NM_016068	Hs.423968	CR624091
FIS1	fission 1 (mitochondrial outer membrane) homolog (S. cerevisiae) (FIS1), mRNA	NM_016068	Hs.423968	CR624091
FKBP9	FK506 binding protein 9, 63 kDa (FKBP9), mRNA	NM_007270	Hs.103934	AB209352
FKBP9L	FK506 binding protein 9-like (FKBP9L), mRNA	NM_182827	Hs.446691	NM_182827
FLJ10815	amino acid transporter (FLJ10815), mRNA	NM_018231	Hs.10499	BC063399
FLJ20186	hypothetical protein FLJ20186 (FLJ20186), transcript variant 2, mRNA	NM_017702	Hs.62771	NM_207514
FLJ20186	hypothetical protein FLJ20186 (FLJ20186), transcript variant 2, mRNA	NM_017702	Hs.62771	NM_207514
FLJ22028	hypothetical protein FLJ22028 (FLJ22028), mRNA	NM_024854	Hs.700677	AK125461

FLJ22028	hypothetical protein FLJ22028 (FLJ22028), mRNA	NM_024854	Hs.700677	AK125461
FLJ22028	hypothetical protein FLJ22028 (FLJ22028), mRNA	NM_024854	Hs.700677	AK125461
FLJ22028	hypothetical protein FLJ22028 (FLJ22028), mRNA	NM_024854	Hs.700677	AK125461
FLJ22222	hypothetical protein FLJ22222 (FLJ22222), transcript variant 1, mRNA	NM_024648	Hs.567578	BM923625
FLJ22596	cDNA: FLJ22596 fis, clone HSI03808.	AK026249	Hs.677369	AK026249
FLJ27365	FLJ27365 protein (FLJ27365), mRNA	NM_207477	Unknown	
FLJ31033	cDNA FLJ13681 fis, clone PLACE2000014, weakly similar to HYPOTHETICAL HELICASE C28H8.3 IN CHROMOSOME III.	AK023743	Hs.535011	NM_001012967
FLJ32312	hypothetical protein FLJ32312 (FLJ32312), mRNA	NM_144709	Hs.368348	AL832208
FLJ36144	CDNA FLJ36144 fis, clone TESTI2025022, weakly similar to TRICHOHYALIN.	ENST00000312015	Unknown	
FLJ36492	cDNA FLJ36492 fis, clone THYMU2018455.	AK093811	Hs.419859	EF553518
FLJ36644	cDNA FLJ36644 fis, clone UMVEN1000143.	AK093963	Hs.225661	XM_001126095
FLJ36748	hypothetical protein FLJ36748 (FLJ36748), mRNA	NM_152406	Hs.483793	NM_152406
FLJ39582	full-length cDNA clone CS0DI082YF06 of Placenta Cot 25-normalized of (human).	CR600536	Hs.517430	CR600536
FLJ40243	hypothetical protein FLJ40243 (FLJ40243), mRNA	NM_173489	Hs.97714	NM_173489
FLJ45055	hypothetical protein LOC644128, mRNA (cDNA clone IMAGE:6158500).	BC064938	Hs.652159	BC064938
FLJ45248	CDNA FLJ45248 fis, clone BRHIP2006819.	ENST00000360312	Unknown	
FMO3	flavin containing monooxygenase 3 (FMO3), transcript variant 2, mRNA	NM_001002294	Hs.445350	AK223166
FN1	fibronectin 1 (FN1), transcript variant 1, mRNA	NM_212482	Hs.203717	NM_212482
FN1	fibronectin 1 (FN1), transcript variant 7, mRNA	NM_054034	Hs.203717	NM_212482
FNDC3B	fibronectin type III domain containing 3B (FNDC3B), mRNA	NM_022763	Hs.159430	NM_022763
FNDC3B	fibronectin type III domain containing 3B, mRNA (cDNA clone IMAGE:3882800), complete cds.	BC012204	Hs.159430	NM_022763
FOSL2	FOS-like antigen 2 (FOSL2), mRNA	NM_005253	Hs.220971	BX647822
FREQ	frequenin homolog (Drosophila) (FREQ), mRNA	NM_014286	Hs.694740	NM_014286
FRMD3	FERM domain containing 3 (FRMD3), mRNA	NM_174938	Hs.127535	BX647549
FRMD4A	cDNA FLJ25099 fis, clone CBR01272.	AK057828	Hs.330463	BC151244
FRMD4A	FERM domain containing 4A (FRMD4A), mRNA	NM_018027	Hs.330463	BC151244
FRMD6	FERM domain containing 6 (FRMD6), transcript variant 1, mRNA	NM_001042481	Hs.434914	BX648295
FRMD6	FERM domain containing 6 (FRMD6), transcript variant 1, mRNA	NM_001042481	Hs.434914	BX648295
FRYL	FRY-like (FRYL), mRNA	NM_015030	Hs.646327	NM_015030
FUNDC2	FUN14 domain containing 2 (FUNDC2), mRNA	NM_023934	Hs.356050	AK091092
FXR1	H.sapiens mRNA for FXR1 protein.	X90874	Hs.478407	NM_001013439
FYN	FYN oncogene related to SRC, FGR, YES (FYN), transcript variant 1, mRNA	NM_002037	Hs.390567	BX537571
FYN	FYN oncogene related to SRC, FGR, YES (FYN), transcript variant 1, mRNA	NM_002037	Hs.390567	BX537571
FYN	FYN oncogene related to SRC, FGR, YES (FYN), transcript variant 1, mRNA	NM_002037	Hs.390567	BX537571
FYN	FYN oncogene related to SRC, FGR, YES (FYN), transcript variant 1, mRNA	NM_002037	Hs.390567	BX537571
FYN	FYN oncogene related to SRC, FGR, YES (FYN), transcript variant 1, mRNA	NM_002037	Hs.390567	BX537571
FYN	FYN oncogene related to SRC, FGR, YES (FYN), transcript variant 1, mRNA	NM_002037	Hs.390567	BX537571
FYN	FYN oncogene related to SRC, FGR, YES (FYN), transcript variant 1, mRNA	NM_002037	Hs.390567	BX537571
FYN	FYN oncogene related to SRC, FGR, YES (FYN), transcript variant 1, mRNA	NM_002037	Hs.390567	BX537571
FYN	FYN oncogene related to SRC, FGR, YES (FYN), transcript variant 1, mRNA	NM_002037	Hs.390567	BX537571
FYN	FYN oncogene related to SRC, FGR, YES (FYN), transcript variant 1, mRNA	NM_002037	Hs.390567	BX537571
G36726	SHGC-52890 Human STS cDNA, sequence tagged site.	G36726	Unknown	
G6PC	glucose-6-phosphatase, catalytic subunit (G6PC), mRNA	NM_000151	Hs.212293	NM_000151
GAB2	GRB2-associated binding protein 2 (GAB2), transcript variant 2, mRNA	NM_012296	Hs.429434	NM_012296
GAB3	GRB2-associated binding protein 3 (GAB3), mRNA	NM_080612	Hs.496982	NM_001081573
GALK2	galactokinase 2 (GALK2), transcript variant 2, mRNA	NM_001001556	Hs.122006	NM_001001556
GALNACT-2	chondroitin sulfate GalNAcT-2 (GALNACT-2), mRNA	NM_018590	Hs.657569	BX647369
GBE1	glucan (1,4-alpha-), branching enzyme 1 (glycogen branching enzyme, Andersen disease, glycogen storage disease type IV) (GBE1), mRNA	NM_000158	Hs.436062	AK125918
GBP2	guanylate binding protein 2, interferon-inducible (GBP2), mRNA	NM_004120	Hs.386567	NM_004120
GBP3	guanylate binding protein 3 (GBP3), mRNA	NM_018284	Hs.656774	CR936755
GBP3	guanylate binding protein 3 (GBP3), mRNA	NM_018284	Hs.656774	CR936755

GCC1	GRIP and coiled-coil domain containing 1 (GCC1), mRNA	NM_024523	Hs.521168	BC014100
Gcom1	GRINL1A combined protein (Gcom1), transcript variant 1, mRNA	NM_001018090	Hs.437256	NM_001018090
GDF15	growth differentiation factor 15 (GDF15), mRNA	NM_004864	Hs.616962	BQ883534
GDF6	growth differentiation factor 6 (GDF6), mRNA	NM_001001557	Hs.492277	AJ537424
GGCX	gamma-glutamyl carboxylase (GGCX), mRNA	NM_000821	Hs.77719	NM_000821
GLB1L	galactosidase, beta 1-like (GLB1L), mRNA	NM_024506	Hs.181173	BC028370
GLI4	GLI-Kruppel family member GLI4 (GLI4), mRNA	NM_138465	Hs.400533	AB209654
GLIPR1	GLI pathogenesis-related 1 (glioma) (GLIPR1), mRNA	NM_006851	Hs.205558	NM_006851
GLRX2	glutaredoxin 2 (GLRX2), transcript variant 1, mRNA	NM_016066	Hs.458283	BM908128
GLS	glutaminase C mRNA, complete cds.	AF158555	Hs.116448	CR749593
GLTP	glycolipid transfer protein (GLTP), mRNA	NM_016433	Hs.381256	NM_016433
GMFG	glia maturation factor, gamma (GMFG), mRNA	NM_004877	Hs.5210	BU934772
GMPR	guanosine monophosphate reductase (GMPR), mRNA	NM_006877	Hs.484741	BC008281
GNG11	guanine nucleotide binding protein (G protein), gamma 11 (GNG11), mRNA	NM_004126	Hs.83381	BF971151
GNG12	guanine nucleotide binding protein (G protein), gamma 12 (GNG12), mRNA	NM_018841	Hs.700661	NM_018841
GNLY	granulysin (GNLY), transcript variant NKG5, mRNA	NM_006433	Hs.105806	BC063245
GOLT1B	golgi transport 1 homolog B (S. cerevisiae) (GOLT1B), mRNA	NM_016072	Hs.62275	AB097020
GOPC	golgi associated PDZ and coiled-coil motif containing (GOPC), transcript variant 1, mRNA	NM_020399	Hs.191539	AB209385
GOSR2	golgi SNAP receptor complex member 2 (GOSR2), transcript variant B, mRNA	NM_054022	Hs.463278	NM_004287
GPA33	glycoprotein A33 (transmembrane) (GPA33), mRNA	NM_005814	Hs.651244	NM_005814
GPD1L	glycerol-3-phosphate dehydrogenase 1-like (GPD1L), mRNA	NM_015141	Hs.82432	D42047
GPR123	G protein-coupled receptor 123 (GPR123), mRNA	NM_032422	Unknown	
GPR126	G protein-coupled receptor 126 (GPR126), transcript variant a1, mRNA	NM_020455	Hs.318894	NM_020455
GPR126	G protein-coupled receptor 126 (GPR126), transcript variant a1, mRNA	NM_020455	Hs.318894	NM_020455
GPRC5A	G protein-coupled receptor, family C, group 5, member A (GPRC5A), mRNA	NM_003979	Hs.631733	AK122672
GRM5	glutamate receptor, metabotropic 5 (GRM5), mRNA	NM_000842	Hs.147361	D28539
GRPEL1	clone 24706 mRNA sequence.	AF070525	Hs.443723	AK098475
GSG1L	GSG1-like	ENST00000380897	Unknown	
GSTK1	glutathione S-transferase kappa 1 (GSTK1), mRNA	NM_015917	Hs.390667	BM912672
GTF2H1	general transcription factor IIH, polypeptide 1 (62kD subunit) (GTF2H1), mRNA	NM_005316	Hs.577202	NM_005316
GUSBL2	glucuronidase, beta-like 2, mRNA (cDNA clone IMAGE:6047050).	BC065547	Hs.561539	AL831827
H2AFJ	H2A histone family, member J (H2AFJ), transcript variant 2, mRNA	NM_177925	Hs.524280	NM_177925
H2AFJ	H2A histone family, member J (H2AFJ), transcript variant 2, mRNA	NM_177925	Hs.524280	NM_177925
H2AFJ	H2A histone family, member J (H2AFJ), transcript variant 1, mRNA	NM_018267	Unknown	
HARS	histidyl-tRNA synthetase (HARS), mRNA	NM_002109	Hs.528050	AK000498
HARS	histidyl-tRNA synthetase (HARS), mRNA	NM_002109	Hs.528050	AK000498
HBEGF	heparin-binding EGF-like growth factor (HBEGF), mRNA	NM_001945	Hs.799	BC033097
HCFC2	host cell factor C2 (HCFC2), mRNA	NM_013320	Hs.506558	BC033799
HECW2	HECT, C2 and WW domain containing E3 ubiquitin protein ligase 2 (HECW2), mRNA	NM_020760	Hs.654742	AB037722
HECW2	HECT, C2 and WW domain containing E3 ubiquitin protein ligase 2 (HECW2), mRNA	NM_020760	Hs.654742	AB037722
HEG1	mRNA for KIAA1237 protein, partial cds.	AB033063	Hs.477420	NM_020733
HFE	hemochromatosis (HFE), transcript variant 1, mRNA	NM_000410	Hs.233325	NM_000410
HGSNAT	heparan-alpha-glucosaminide N-acetyltransferase, mRNA (cDNA clone IMAGE:3880903), complete cds.	BC012452	Hs.600384	NM_152419
HGSNAT	cDNA FLJ32731 fis, clone TESTI2001134.	AK057293	Hs.600384	NM_152419
HIATL1	hippocampus abundant transcript-like 1 (HIATL1), mRNA	NM_032558	Hs.699244	NM_032558
HIPK3	homeodomain interacting protein kinase 3 (HIPK3), transcript variant 1, mRNA	NM_005734	Hs.201918	NM_005734
HIST1H2AC	histone cluster 1, H2ac (HIST1H2AC), mRNA	NM_003512	Hs.484950	CR608156
HNMT	histamine N-methyltransferase (HNMT), transcript variant 2, mRNA	NM_001024074	Hs.42151	NM_006895
HOP	homeodomain-only protein (HOP), transcript variant 2, mRNA	NM_139211	Hs.654864	BF575818
HPCAL1	hippocalcin-like 1 (HPCAL1), transcript variant 2, mRNA	NM_134421	Hs.580427	CR749837
HRH1	histamine receptor H1 (HRH1), mRNA	NM_000861	Hs.1570	NM_001098213
HRH1	histamine receptor H1 (HRH1), mRNA	NM_000861	Hs.1570	NM_001098213

HTATIP	HIV-1 Tat interacting protein, 60kDa (HTATIP), transcript variant 2, mRNA	NM_006388	Hs.528299	NM_182710
HTF9C	HpaII tiny fragments locus 9C (HTF9C), transcript variant 1, mRNA	NM_022727	Hs.643452	NM_022727
HTR2B	5-hydroxytryptamine (serotonin) receptor 2B (HTR2B), mRNA	NM_000867	Hs.421649	BC063123
HTRA2	HtrA serine peptidase 2 (HTRA2), nuclear gene encoding mitochondrial protein, transcript variant 2, mRNA	NM_145074	Hs.469045	NM_013247
HYAL3	hyaluronoglucosaminidase 3 (HYAL3), mRNA	NM_003549	Hs.129910	BC051750
HYI	hydroxypyruvate isomerase homolog (E. coli) (HYI), mRNA	NM_031207	Hs.643560	AK096244
HYI	hydroxypyruvate isomerase homolog (E. coli) (HYI), mRNA	NM_031207	Hs.643560	AK096244
IAH1	isoamyl acetate-hydrolyzing esterase 1 homolog (S. cerevisiae) (IAH1), mRNA	NM_001039613	Hs.700710	AK092982
IDH3G	isocitrate dehydrogenase 3 (NAD+) gamma (IDH3G), nuclear gene encoding mitochondrial protein, transcript variant 1, mRNA	NM_004135	Hs.410197	AB209206
IFI16	interferon, gamma-inducible protein 16 (IFI16), mRNA	NM_005531	Hs.380250	NM_005531
IFI16	interferon, gamma-inducible protein 16 (IFI16), mRNA	NM_005531	Hs.380250	NM_005531
IFIH1	interferon induced with helicase C domain 1 (IFIH1), mRNA	NM_022168	Hs.163173	NM_022168
IFNGR2	interferon gamma receptor 2 (interferon gamma transducer 1) (IFNGR2), mRNA	NM_005534	Hs.634632	NM_005534
IGFBP4	insulin-like growth factor binding protein 4 (IGFBP4), mRNA	NM_001552	Hs.462998	NM_001552
IGHA1	cDNA FLJ46621 fis, clone TLUNG2001445, highly similar to Ig alpha-1 chain C region.	AK128476	Hs.648398	AK128652
IGKC	immunoglobulin kappa constant, mRNA (cDNA clone MGC:71265 IMAGE:6302855), complete cds.	BC067092	Hs.449621	AK128524
IL10RB	interleukin 10 receptor, beta (IL10RB), mRNA	NM_000628	Hs.654593	AK124057
IL18R1	interleukin 18 receptor 1 (IL18R1), mRNA	NM_003855	Hs.469521	NM_003855
IL18R1	interleukin 18 receptor 1 (IL18R1), mRNA	NM_003855	Hs.469521	NM_003855
IL1R1	interleukin 1 receptor, type I (IL1R1), mRNA	NM_000877	Hs.557403	M27492
ILK	integrin-linked kinase (ILK), transcript variant 3, mRNA	NM_001014795	Hs.655002	CR749220
ILK	integrin-linked kinase (ILK), transcript variant 3, mRNA	NM_001014795	Hs.655002	CR749220
ILKAP	integrin-linked kinase-associated serine/threonine phosphatase 2C (ILKAP), transcript variant 2, mRNA	NM_176799	Unknown	
IMPAD1	inositol monophosphatase domain containing 1 (IMPAD1), mRNA	NM_017813	Hs.591872	BC067814
INPP1	inositol polyphosphate-1-phosphatase (INPP1), mRNA	NM_002194	Hs.32309	AK093560
IQSEC3	IQ motif and Sec7 domain 3, mRNA (cDNA clone MGC:30156 IMAGE:4940063), complete cds.	BC024764	Hs.536319	AB029033
IRAK4	interleukin-1 receptor-associated kinase 4 (IRAK4), mRNA	NM_016123	Hs.138499	AK000528
IRAK4	interleukin-1 receptor-associated kinase 4 (IRAK4), mRNA	NM_016123	Hs.138499	AK000528
IRF2	interferon regulatory factor 2 (IRF2), mRNA	NM_002199	Hs.654566	BX648934
ISCU	IscU iron-sulfur cluster scaffold homolog (E. coli) (ISCU), nuclear gene encoding mitochondrial protein, transcript variant 1, mRNA	NM_014301	Hs.615131	AK057251
ITGA2	integrin, alpha 2 (CD49B, alpha 2 subunit of VLA-2 receptor) (ITGA2), mRNA	NM_002203	Hs.482077	NM_002203
ITGA2	integrin, alpha 2 (CD49B, alpha 2 subunit of VLA-2 receptor) (ITGA2), mRNA	NM_002203	Hs.482077	NM_002203
ITGA4	integrin, alpha 4 (antigen CD49D, alpha 4 subunit of VLA-4 receptor) (ITGA4), mRNA	NM_000885	Hs.694732	NM_000885
ITGB1	integrin, beta 1 (fibronectin receptor, beta polypeptide, antigen CD29 includes MDF2, MSK12) (ITGB1), transcript variant 1E, mRNA	NM_133376	Hs.695946	NM_002211
ITGB1	integrin, beta 1 (fibronectin receptor, beta polypeptide, antigen CD29 includes MDF2, MSK12) (ITGB1), transcript variant 1E, mRNA	NM_133376	Hs.695946	NM_002211
ITGB1	integrin, beta 1 (fibronectin receptor, beta polypeptide, antigen CD29 includes MDF2, MSK12) (ITGB1), transcript variant 1E, mRNA	NM_133376	Hs.695946	NM_002211
ITGB1	integrin, beta 1 (fibronectin receptor, beta polypeptide, antigen CD29 includes MDF2, MSK12) (ITGB1), transcript variant 1E, mRNA	NM_133376	Hs.695946	NM_002211
ITGB1	full length insert cDNA clone ZD39G09.	AF086249	Hs.695946	NM_002211
ITGB1	integrin, beta 1 (fibronectin receptor, beta polypeptide, antigen CD29 includes MDF2, MSK12) (ITGB1), transcript variant 1E, mRNA	NM_133376	Hs.695946	NM_002211
ITGB1	integrin, beta 1 (fibronectin receptor, beta polypeptide, antigen CD29 includes MDF2, MSK12) (ITGB1), transcript variant 1E, mRNA	NM_133376	Hs.695946	NM_002211
ITGB1	integrin, beta 1 (fibronectin receptor, beta polypeptide, antigen CD29 includes MDF2, MSK12) (ITGB1), transcript variant 1E, mRNA	NM_133376	Hs.695946	NM_002211
ITGB1	integrin, beta 1 (fibronectin receptor, beta polypeptide, antigen CD29 includes MDF2, MSK12) (ITGB1), transcript variant 1E, mRNA	NM_133376	Hs.695946	NM_002211
ITGB1	integrin, beta 1 (fibronectin receptor, beta polypeptide, antigen CD29 includes MDF2, MSK12) (ITGB1), transcript variant 1E, mRNA	NM_133376	Hs.695946	NM_002211
ITGB1	integrin, beta 1 (fibronectin receptor, beta polypeptide, antigen CD29 includes MDF2, MSK12) (ITGB1), transcript variant 1E, mRNA	NM_133376	Hs.695946	NM_002211
ITGB1	integrin, beta 1 (fibronectin receptor, beta polypeptide, antigen CD29 includes MDF2, MSK12) (ITGB1), transcript variant 1E, mRNA	NM_133376	Hs.695946	NM_002211
ITGB1	integrin, beta 1 (fibronectin receptor, beta polypeptide, antigen CD29 includes MDF2, MSK12) (ITGB1), transcript variant 1A, mRNA	NM_002211	Hs.695946	NM_002211
ITGB1BP1	integrin beta 1 binding protein 1 (ITGB1BP1), transcript variant 2, mRNA	NM_022334	Hs.467662	AK093179

ITGB3	integrin, beta 3 (platelet glycoprotein IIIa, antigen CD61) (ITGB3), mRNA	NM_000212	Hs.218040	NM_000212
ITGB3	integrin, beta 3 (platelet glycoprotein IIIa, antigen CD61) (ITGB3), mRNA	NM_000212	Hs.218040	NM_000212
ITGB3	integrin, beta 3 (platelet glycoprotein IIIa, antigen CD61) (ITGB3), mRNA	NM_000212	Hs.218040	NM_000212
JAK2	Janus kinase 2 (a protein tyrosine kinase) (JAK2), mRNA	NM_004972	Hs.656213	AF058925
JAK2	Janus kinase 2 (a protein tyrosine kinase) (JAK2), mRNA	NM_004972	Hs.656213	AF058925
JAK2	Janus kinase 2 (a protein tyrosine kinase) (JAK2), mRNA	NM_004972	Hs.656213	AF058925
JAK2	Janus kinase 2 (a protein tyrosine kinase) (JAK2), mRNA	NM_004972	Hs.656213	AF058925
JAK2	Janus kinase 2 (a protein tyrosine kinase) (JAK2), mRNA	NM_004972	Hs.656213	AF058925
JAK2	Janus kinase 2 (a protein tyrosine kinase) (JAK2), mRNA	NM_004972	Hs.656213	AF058925
JAK2	Janus kinase 2 (a protein tyrosine kinase) (JAK2), mRNA	NM_004972	Hs.656213	AF058925
JAK2	Janus kinase 2 (a protein tyrosine kinase) (JAK2), mRNA	NM_004972	Hs.656213	AF058925
JAK2	Janus kinase 2 (a protein tyrosine kinase) (JAK2), mRNA	NM_004972	Hs.656213	AF058925
JAK2	Janus kinase 2 (a protein tyrosine kinase) (JAK2), mRNA	NM_004972	Hs.656213	AF058925
JMJD4	jumonji domain containing 4 (JMJD4), mRNA	NM_023007	Hs.555974	AK022579
JOSD1	Josephin domain containing 1 (JOSD1), mRNA	NM_014876	Hs.3094	CR596979
KATNA1	katanin p60 (ATPase-containing) subunit A 1 (KATNA1), mRNA	NM_007044	Hs.450175	CR600129
KATNAL1	katanin p60 subunit A-like 1 (KATNAL1), transcript variant 1, mRNA	NM_032116	Hs.243596	NM_032116
KCTD9	potassium channel tetramerisation domain containing 9 (KCTD9), mRNA	NM_017634	Hs.72071	AL117436
KCTD9	potassium channel tetramerisation domain containing 9 (KCTD9), mRNA	NM_017634	Hs.72071	AL117436
KDELR2	KDEL (Lys-Asp-Glu-Leu) endoplasmic reticulum protein retention receptor 2 (KDELR2), mRNA	NM_006854	Hs.654552	NM_006854
KDELR2	KDEL (Lys-Asp-Glu-Leu) endoplasmic reticulum protein retention receptor 2 (KDELR2), mRNA	NM_006854	Hs.654552	NM_006854
KIAA0226	KIAA0226, mRNA (cDNA clone IMAGE:5217239), complete cds.	BC033615	Hs.478868	NM_014687
KIAA0256	KIAA0256 gene product (KIAA0256), mRNA	NM_014701	Hs.9997	NM_014701
KIAA0323	KIAA0323 (KIAA0323), mRNA	NM_015299	Hs.700630	NM_015299
KIAA1160	KIAA1160 protein (KIAA1160), mRNA	NM_020701	Hs.512661	BC019849
KIAA1600	KIAA1600 (KIAA1600), mRNA	NM_020940	Hs.192619	AB046820
KIAA1652	mRNA for KIAA1652 protein, partial cds.	AB051439	Hs.443430	AB051439
KIAA1949	KIAA1949, mRNA (cDNA clone MGC:71094 IMAGE:5575643), complete cds.	BC066644	Hs.696054	AK124880
KIAA2002	mRNA for KIAA2002 protein.	AB082533	Hs.9587	NM_024776
KIF3B	kinesin family member 3B (KIF3B), mRNA	NM_004798	Hs.369670	NM_004798
KIF5B	kinesin family member 5B (KIF5B), mRNA	NM_004521	Hs.644646	NM_004521
KIF9	kinesin family member 9 (KIF9), transcript variant 1, mRNA	NM_022342	Hs.373947	NM_182902
KLF2	Kruppel-like factor 2 (lung) (KLF2), mRNA	NM_016270	Hs.107740	AK225820
KLF6	Kruppel-like factor 6 (KLF6), transcript variant 2, mRNA	NM_001300	Hs.4055	U51869
KLF6	Kruppel-like factor 6 (KLF6), transcript variant 2, mRNA	NM_001300	Hs.4055	U51869
KLF9	Kruppel-like factor 9 (KLF9), mRNA	NM_001206	Hs.150557	NM_001206
KLHL3	kelch-like 3 (Drosophila) (KLHL3), mRNA	NM_017415	Hs.655084	AF208070
KRT7	keratin 7 (KRT7), mRNA	NM_005556	Hs.411501	AK128505
KRT80	keratin 80 (KRT80), mRNA	NM_182507	Hs.140978	BC065180
KRT80	keratin 80 (KRT80), mRNA	NM_182507	Hs.140978	BC065180
KRTAP4-5	keratin associated protein 4-5 (KRTAP4-5), mRNA	NM_033188	Hs.514863	AJ406937
KTI12	KTI12 homolog, chromatin associated (S. cerevisiae) (KTI12), mRNA	NM_138417	Hs.655082	NM_138417
LAMA3	laminin, alpha 3 (LAMA3), transcript variant 1, mRNA	NM_198129	Hs.436367	NM_198129
LAMA4	laminin, alpha 4 (LAMA4), mRNA	NM_002290	Hs.654572	BC066552
LAMB3	laminin, beta 3 (LAMB3), transcript variant 2, mRNA	NM_001017402	Hs.497636	NM_001017402
LAMP2	lysosomal-associated membrane protein 2 (LAMP2), transcript variant LAMP2B, mRNA	NM_013995	Hs.496684	BX648255
LAT2	linker for activation of T cells family, member 2 (LAT2), transcript variant 1, mRNA	NM_032464	Hs.647049	AK092904
LATS2	LATS, large tumor suppressor, homolog 2 (Drosophila) (LATS2), mRNA	NM_014572	Hs.78960	NM_014572
LATS2	Serine/threonine-protein kinase LATS2 (EC 2.7.11.1) (Large tumor suppressor homolog 2) (Serine/threonine-protein kinase kpm) (Kinase phosphorylated during mitosis protein) (Warts-like kinase).	ENST00000382592	Unknown	
LCT	lactase (LCT), mRNA	NM_002299	Hs.551506	NM_002299
LDHA	lactate dehydrogenase A (LDHA), mRNA	NM_005566	Hs.2795	BM457440

LDHA	lactate dehydrogenase A (LDHA), mRNA	NM_005566	Hs.2795	BM457440
LGALS3	lectin, galactoside-binding, soluble, 3 (galectin 3) (LGALS3), transcript variant 1, mRNA	NM_002306	Hs.531081	AB209391
LGALS8	lectin, galactoside-binding, soluble, 8 (galectin 8) (LGALS8), transcript variant 1, mRNA	NM_006499	Hs.4082	AB209914
LIG4	ligase IV, DNA, ATP-dependent (LIG4), transcript variant 1, mRNA	NM_002312	Hs.166091	NM_002312
LIMA1	LIM domain and actin binding 1 (LIMA1), mRNA	NM_016357	Hs.525419	BC110815
LIMS3	LIM and senescent cell antigen-like domains 3 (LIMS3), mRNA	NM_033514	Hs.535619	AK096170
LINS1	lines homolog 1 (Drosophila) (LINS1), transcript variant 1, mRNA	NM_018148	Hs.105633	NM_018148
LIPE	lipase, hormone-sensitive (LIPE), mRNA	NM_005357	Hs.656980	BC070041
LIX1L	Lix1 homolog (mouse)-like (LIX1L), mRNA	NM_153713	Hs.632435	AK128733
LMAN1	lectin, mannose-binding, 1 (LMAN1), mRNA	NM_005570	Hs.465295	X71661
LMNA	lamin A/C (LMNA), transcript variant 2, mRNA	NM_005572	Hs.594444	NM_170707
LNPEP	leucyl/cystinyl aminopeptidase (LNPEP), transcript variant 1, mRNA	NM_005575	Hs.656905	AB208883
LOC116236	PPWG6510.	ENST00000307201	Unknown	
LOC116349	hypothetical protein BC014011, mRNA (cDNA clone MGC:35369 IMAGE:5183143), complete cds.	BC029796	Hs.446702	BF972119
LOC126075	hypothetical protein LOC126075, mRNA (cDNA clone IMAGE:5171832).	ENST00000327804	Unknown	
LOC127099	PREDICTED: similar to 60S acidic ribosomal protein P1 (LOC127099), mRNA	XM_060328	Unknown	
LOC134145	hypothetical protein LOC134145 (LOC134145), mRNA	NM_199133	Hs.481569	AK000674
LOC134466	cDNA FLJ39217 fis, clone OCBBF2006639, moderately similar to ZINC FINGER PROTEIN 84.	AK096536	Hs.661876	AK096536
LOC153277	cDNA FLJ20096 fis, clone COL04378.	AK000103	Hs.595953	AK000103
LOC161635	Homo sapiens, clone IMAGE:5166482, mRNA, partial cds.	BC028192	Hs.659822	BC066972
LOC162073	cDNA FLJ39008 fis, clone NT2RI2025255.	AK096327	Hs.530899	NM_001034841
LOC201164	similar to CG12314 gene product (LOC201164), mRNA	NM_178836	Hs.31652	NM_178836
LOC205251	cDNA clone IMAGE:5763979.	BC064430	Hs.128499	BC064430
LOC222103	PREDICTED: similar to Keratin, type II cytoskeletal 8 (Cytokeratin-8) (CK-8) (Keratin-8) (K8) (LOC222103), mRNA	XR_018058	Hs.648116	XR_018058
LOC253981	cDNA: FLJ21778 fis, clone HEP00201.	AK025431	Hs.283378	NM_001085400
LOC255374	similar to hypothetical protein MGC49416 (LOC255374), mRNA	NM_203397	Hs.632319	NM_203397
LOC284441	actin-related protein 2 pseudogene (LOC284441) on chromosome 19	NR_003128	Unknown	
LOC284454	mRNA; cDNA DKFZp686K181 (from clone DKFZp686K181).	BX640708	Hs.436426	AL832183
LOC286260	hypothetical protein LOC286260, mRNA (cDNA clone IMAGE:5115498), with apparent retained intron.	BC032375	Hs.593896	AK023312
LOC338620	hypothetical protein LOC338620, mRNA (cDNA clone IMAGE:6023208), partial cds.	BC043009	Hs.660499	BC043009
LOC340460	PREDICTED: similar to Keratin, type I cytoskeletal 18 (Cytokeratin-18) (CK-18) (Keratin-18) (K18) (LOC340460), mRNA	XR_018344	Hs.647171	XR_018344
LOC341965	PREDICTED: similar to Voltage-dependent anion-selective channel protein 3 (VDAC-3) (hVDAC3) (Outer mitochondrial membrane protein porin 3) (LOC341965), mRNA	XR_019103	Hs.647556	XR_019103
LOC342865	PREDICTED: hypothetical LOC342865 (LOC342865), mRNA	ENST00000335523	Unknown	
LOC344887	mRNA; cDNA DKFZp686B14224 (from clone DKFZp686B14224).	BX640843	Hs.128803	BX640843
LOC345645	PREDICTED: similar to peptidase (prosome, macropain) 26S subunit, ATPase 1 (LOC345645), mRNA	XR_018613	Hs.646945	XR_018613
LOC374395	similar to RIKEN cDNA 1810059G22 (LOC374395), mRNA	NM_199337	Hs.381134	BM913006
LOC387763	hypothetical LOC387763, mRNA (cDNA clone IMAGE:6272440), partial cds.	ENST00000339446	Unknown	
LOC388743	PREDICTED: similar to calpain 8 (LOC388743), mRNA	ENST00000366873	Unknown	
LOC388965	similar to hepatitis C virus core-binding protein 6; cervical cancer oncogene 3 (LOC388965), mRNA	NM_001013648	Unknown	
LOC390482	PREDICTED: similar to Aldose reductase (AR) (Aldehyde reductase) (LOC390482), mRNA	XR_018461	Hs.647557	XR_018461
LOC391160	PREDICTED: similar to Actin-related protein 2	XR_018489	Hs.647765	XR_018489
LOC392226	602077703F1 NIH_MGC_62 cDNA clone IMAGE:4252032 5', mRNA sequence	BF572267	Hs.571430	BF572267
LOC400456	hypothetical gene supported by BC040875, mRNA (cDNA clone IMAGE:5721930).	BC040875	Hs.632160	BC040875
LOC401397	hypothetical LOC401397, mRNA (cDNA clone IMAGE:4244115), complete cds.	BC107860	Hs.117929	CR625886
LOC441131	PREDICTED: similar to Actin-related protein 2	XR_018731	Hs.646993	XR_018731
LOC441177	hypothetical protein LOC441177 (LOC441177), mRNA	NM_001013720	Unknown	
LOC441964	PREDICTED: similar to sperm protein SSP411 (LOC441964), mRNA	XM_497783	Hs.642684	XM_497783
LOC442142	PREDICTED: similar to SRY-box containing gene 30 (LOC442142), mRNA	XR_016528	Hs.634724	AK097647
LOC442272	PREDICTED: similar to YKT6 v-SNARE protein (LOC442272), mRNA	XR_019351	Hs.648080	XR_019351
LOC442325	cDNA FLJ26697 fis, clone PCD00618.	AK130207	Hs.683165	AK130207

LOC51233	hypothetical protein LOC51233 (LOC51233), mRNA	NM_016449	Hs.517466	BC056888
LOC541471	cDNA FLJ10934 fis, clone OVARC1000640.	AK001796	Hs.652426	AK001796
LOC572558	chromosome 2 mRNA sequence.	AY343891	Hs.9015	CF127520
LOC641796	PREDICTED: similar to Protein C20orf45 (LOC641796), mRNA	XM_001128028	Unknown	
LOC642413	PREDICTED: similar to Cathepsin L precursor (Major excreted protein) (MEP) (LOC642413), mRNA	XR_016155	Hs.558776	XR_016155
LOC643560	PREDICTED: similar to Superoxide dismutase (LOC643560), mRNA	XR_018758	Hs.650965	XR_018758
LOC643668	PREDICTED: similar to peptidase (prosome, macropain) 26S subunit, ATPase 1 (LOC643668), mRNA	XR_019339	Hs.576609	XR_019339
LOC643783	PREDICTED: hypothetical LOC643783 (LOC643783), mRNA	XM_931798	Hs.631514	BQ278261
LOC644357	PREDICTED: similar to Elongation factor 1-delta (EF-1-delta) (Antigen NY-CO-4) (LOC644357), mRNA	XM_927514	Unknown	
LOC644387	PREDICTED: similar to myelin protein zero-like 1 isoform a (LOC644387), mRNA	XR_016224	Hs.647057	BG193894
LOC644462	PREDICTED: similar to amyotrophic lateral sclerosis 2 (juvenile) chromosome region, candidate 2 (LOC644462), mRNA	XM_930312	Hs.613059	XM_930312
LOC645132	PREDICTED: similar to vacuolar protein sorting 35 (LOC645132), mRNA	XR_016136	Hs.589436	XR_016136
LOC645332	Q4KMP3_HUMAN (Q4KMP3) FAM86B1 protein, partial (22%)	ENST00000359244	Unknown	
LOC647252	PREDICTED: similar to Charged multivesicular body protein 5 (Chromatin-modifying protein 5) (Vacuolar protein sorting 60) (Vps60) (hVps60) (SNF7 domain-containing protein 2) (LOC647252), mRNA	XR_019210	Hs.647758	XR_019210
LOC647907	PREDICTED: similar to DnaJ homolog subfamily A member 1 (Heat shock 40 kDa protein 4) (DnaJ protein homolog 2) (HSJ-2) (HSDJ) (LOC647907), mRNA	XR_018209	Hs.648331	XR_018209
LOC648895	PREDICTED: similar to Pyruvate kinase isozymes M1	XR_018496	Hs.201430	XR_018496
LOC651053	PREDICTED: similar to ATP-dependent RNA helicase DDX18 (DEAD box protein 18) (Myc-regulated DEAD box protein) (MrDb) (LOC651053), mRNA	XR_019392	Hs.650970	XR_019392
LOC652192	PREDICTED: similar to Keratin, type I cytoskeletal 18 (Cytokeratin-18) (CK-18) (Keratin-18) (K18) (LOC652192), mRNA	XR_019238	Hs.647927	XR_019238
LOC653316	hypothetical protein LOC653316 (LOC653316), mRNA	NM_001079527	Hs.652193	NM_001079527
LOC653769	PREDICTED: similar to Group 10 secretory phospholipase A2 precursor (Group X secretory phospholipase A2) (Phosphatidylcholine 2-acylhydrolase GX) (GX sPLA2) (sPLA2-X) (LOC653769), mRNA	XR_017277	Hs.679109	XR_017277
LOC654342	PREDICTED: lymphocyte-specific protein 1 (LSP1), mRNA	XM_946374	Hs.656760	CR601542
LOC727982	cDNA clone IMAGE:5266866.	BC047589	Hs.444391	BC047589
LOC729717	BX118285 Soares_testis_NHT cDNA clone IMAGp998G173521, mRNA sequence	BX118285	Hs.124007	BX118285
LOC729792	PREDICTED: hypothetical protein LOC729792 (LOC729792), mRNA	XM_001131329	Hs.582967	XM_001131329
LOC730044	similar to hypothetical protein FLJ20897, mRNA (cDNA clone MGC:149669 IMAGE:40117257), complete cds.	ENST00000306722	Unknown	
LOC730556	PREDICTED: similar to Coiled-coil-helix-coiled-coil-helix domain-containing protein 2 (HCV NS2 trans-regulated protein) (NS2TP) (LOC730556), mRNA	XR_015322	Hs.646956	XR_015322
LOC731479	CDNA FLJ43983 fis, clone TESTI4018881.	ENST00000360524	Unknown	
LOC92154	hypothetical protein BC002770 (LOC92154), mRNA	NM_138383	Hs.432387	NM_138383
LOC92482	hypothetical protein LOC92482, mRNA (cDNA clone IMAGE:6063114), partial cds.	BC073157	Hs.651309	AK125829
LRP10	low density lipoprotein receptor-related protein 10 (LRP10), mRNA	NM_014045	Hs.525232	NM_014045
LRP10	low density lipoprotein receptor-related protein 10 (LRP10), mRNA	NM_014045	Hs.525232	NM_014045
LRRC17	leucine rich repeat containing 17 (LRRC17), transcript variant 2, mRNA	NM_005824	Hs.567412	AK225701
LRRC28	leucine rich repeat containing 28 (LRRC28), mRNA	NM_144598	Hs.578684	AL833556
LRRC32	leucine rich repeat containing 32 (LRRC32), mRNA	NM_005512	Hs.151641	BC052210
LRRC57	leucine rich repeat containing 57 (LRRC57), mRNA	NM_153260	Hs.234681	AK094891
LRRC59	leucine rich repeat containing 59 (LRRC59), mRNA	NM_018509	Hs.370927	AK025328
LRRFIP2	leucine rich repeat (in FLII) interacting protein 2 (LRRFIP2), transcript variant 2, mRNA	NM_017724	Hs.475319	AK124432
LSMD1	LSM domain containing 1 (LSMD1), mRNA	NM_032356	Hs.565094	NM_032356
LSS	lanosterol synthase (2,3-oxidosqualene-lanosterol cyclase) (LSS), transcript variant 2, mRNA	NM_001001438	Hs.596543	AK226141
LSS	lanosterol synthase (2,3-oxidosqualene-lanosterol cyclase) (LSS), transcript variant 2, mRNA	NM_001001438	Hs.596543	AK226141
LY96	lymphocyte antigen 96 (LY96), mRNA	NM_015364	Hs.660766	BM918324
LYRM2	LYR motif containing 2 (LYRM2), mRNA	NM_020466	Hs.177275	NM_020466
LYZL2	lysozyme-like 2 (LYZL2), mRNA	NM_183058	Hs.522610	BF979319
MAGEA10	melanoma antigen family A, 10 (MAGEA10), transcript variant 1, mRNA	NM_001011543	Hs.18048	NM_001011543
MAN2C1	mannosidase, alpha, class 2C, member 1, mRNA (cDNA clone IMAGE:4329693), partial cds.	BC010081	Hs.598731	BC010081
MAP3K12	mitogen-activated protein kinase kinase kinase 12 (MAP3K12), mRNA	NM_006301	Hs.699199	AB209453
MAP3K14	mitogen-activated protein kinase kinase kinase 14 (MAP3K14), mRNA	NM_003954	Hs.404183	Y10256

MAPK1	mitogen-activated protein kinase 1 (MAPK1), transcript variant 1, mRNA	NM_002745	Hs.431850	AL157438
MAPK11	mitogen-activated protein kinase 11 (MAPK11), mRNA	NM_002751	Hs.57732	BC027933
MAPK8IP3	mitogen-activated protein kinase 8 interacting protein 3 (MAPK8IP3), transcript variant 1, mRNA	NM_015133	Hs.207763	NM_015133
MARCH9	membrane-associated ring finger (C3HC4) 9 (MARCH9), mRNA	NM_138396	Hs.632709	NM_138396
MBNL1	muscleblind-like (Drosophila) (MBNL1), transcript variant 6, mRNA	NM_207296	Hs.478000	NM_021038
MBNL1	muscleblind-like (Drosophila) (MBNL1), transcript variant 1, mRNA	NM_021038	Hs.478000	NM_021038
MBTPS2	membrane-bound transcription factor peptidase, site 2 (MBTPS2), mRNA	NM_015884	Hs.585245	NM_015884
MCAT	malonyl CoA:ACP acyltransferase (mitochondrial) (MCAT), nuclear gene encoding mitochondrial protein, transcript variant 2, mRNA	NM_014507	Hs.349111	NM_173467
MCAT	malonyl CoA:ACP acyltransferase (mitochondrial) (MCAT), nuclear gene encoding mitochondrial protein, transcript variant 1, mRNA	NM_173467	Hs.349111	NM_173467
MCFD2	multiple coagulation factor deficiency 2 (MCFD2), mRNA	NM_139279	Hs.293689	AL833900
MDFIC	MyoD family inhibitor domain containing (MDFIC), mRNA	NM_199072	Hs.427236	NM_199072
MDM2	Mdm2, transformed 3T3 cell double minute 2, p53 binding protein (mouse) (MDM2), transcript variant MDM2, mRNA	NM_002392	Hs.567303	M92424
MDM2	Mdm2, transformed 3T3 cell double minute 2, p53 binding protein (mouse) (MDM2), transcript variant MDM2, mRNA	NM_002392	Hs.567303	M92424
MDM2	Mdm2, transformed 3T3 cell double minute 2, p53 binding protein (mouse) (MDM2), transcript variant MDM2, mRNA	NM_002392	Hs.567303	M92424
MDM2	Mdm2, transformed 3T3 cell double minute 2, p53 binding protein (mouse) (MDM2), transcript variant MDM2, mRNA	NM_002392	Hs.567303	M92424
MDM2	Mdm2, transformed 3T3 cell double minute 2, p53 binding protein (mouse) (MDM2), transcript variant MDM2, mRNA	NM_002392	Hs.567303	M92424
MDM2	Mdm2, transformed 3T3 cell double minute 2, p53 binding protein (mouse) (MDM2), transcript variant MDM2, mRNA	NM_002392	Hs.567303	M92424
MDS032	uncharacterized hematopoietic stem/progenitor cells protein MDS032 (MDS032), mRNA	NM_018467	Hs.16187	BM547041
ME1	malic enzyme 1, NADP(+)-dependent, cytosolic (ME1), mRNA	NM_002395	Hs.21160	NM_002395
ME3	malic enzyme 3, NADP(+)-dependent, mitochondrial (ME3), nuclear gene encoding mitochondrial protein, transcript variant 2, mRNA	NM_001014811	Hs.199743	NM_001014811
MED10	mediator of RNA polymerase II transcription, subunit 10 homolog (NUT2, S. cerevisiae) (MED10), mRNA	NM_032286	Hs.13885	BG335164
MED8	mediator of RNA polymerase II transcription, subunit 8 homolog (S. cerevisiae) (MED8), transcript variant 4, mRNA	NM_001001653	Unknown	
MFAP3	microfibrillar-associated protein 3 (MFAP3), mRNA	NM_005927	Hs.432818	NM_005927
MFSD5	major facilitator superfamily domain containing 5 (MFSD5), mRNA	NM_032889	Hs.654660	AK074684
MFSD5	major facilitator superfamily domain containing 5 (MFSD5), mRNA	NM_032889	Hs.654660	AK074684
MGAT2	mannosyl (alpha-1,6-)-glycoprotein beta-1,2-N-acetylglucosaminyltransferase (MGAT2), transcript variant 1, mRNA	NM_002408	Hs.93338	NM_002408
MGC102966	similar to Keratin, type I cytoskeletal 16 (Cytokeratin-16) (CK-16) (Keratin-16) (K16), mRNA (cDNA clone MGC:102966 IMAGE:4752428), complete cds.	BC110641	Hs.572477	BC110641
MGC11102	hypothetical protein MGC11102 (MGC11102), mRNA	NM_032325	Hs.425178	NM_032325
MGC11257	hypothetical protein MGC11257 (MGC11257), mRNA	NM_032350	Hs.653258	BC025971
MGC13114	hypothetical protein MGC13114 (MGC13114), transcript variant 8, mRNA	NM_001040166	Unknown	
MGC21874	cDNA FLJ45019 fis, clone BRAWH3015825.	AK126966	Hs.518614	XM_001128049
MGC24125	hypothetical protein MGC24125, mRNA (cDNA clone IMAGE:4687544), complete cds.	BC020886	Hs.673379	AK097110
MGC42157	cDNA clone IMAGE:4799398.	BC030111	Hs.591071	BC037904
MGC50559	hypothetical protein MGC50559 (MGC50559), mRNA	NM_173802	Hs.585084	AL832339
MGC7036	hypothetical protein MGC7036 (MGC7036), mRNA	NM_145058	Hs.488173	AB085763
MGC70863	similar to RPL23AP7 protein (MGC70863), transcript variant 1, mRNA	NM_203477	Hs.406135	BC065556
MGC70863	similar to RPL23AP7 protein (MGC70863), transcript variant 2, mRNA	NM_203302	Hs.406135	BC065556
MGC70863	similar to RPL23AP7 protein (MGC70863), transcript variant 1, mRNA	NM_203477	Hs.406135	BC065556
MGC70863	Novel human gene mapping to chomosome 22.	AL365511	Hs.406135	BC065556
MGC71993	similar to DNA segment, Chr 11, Brigham & Womens Genetics 0434 expressed (MGC71993), mRNA	NM_001004333	Hs.632232	BC040148
MGC87631	similar to hypothetical protein FLJ36492 (MGC87631), mRNA	NM_001004306	Hs.674830	BC068597
MGP	matrix Gla protein (MGP), mRNA	NM_000900	Hs.365706	CR623037
MICA	MHC class I polypeptide-related sequence A (MICA), mRNA	NM_000247	Hs.549053	AK094237
MICAL2	microtubule associated monoxygenase, calponin and LIM domain containing 2 (MICAL2), mRNA	NM_014632	Hs.501928	BX538021
MICALL2	MICAL-like 2 (MICALL2), transcript variant 1, mRNA	NM_182924	Hs.376617	BC037988
MINA	MYC induced nuclear antigen (MINA), transcript variant 3, mRNA	NM_032778	Hs.696280	NM_001042533
MINA	MYC induced nuclear antigen (MINA), transcript variant 1, mRNA	NM_001042533	Hs.696280	NM_001042533

MLLT6	myeloid/lymphoid or mixed-lineage leukemia (trithorax homolog, Drosophila); translocated to, 6 (MLLT6), mRNA	NM_005937	Hs.91531	NM_005937
MLLT7	myeloid/lymphoid or mixed-lineage leukemia (trithorax homolog, Drosophila); translocated to, 7 (MLLT7), mRNA	NM_005938	Hs.584654	NM_005938
MLX	MAX-like protein X (MLX), transcript variant 3, mRNA	NM_170607	Hs.383019	AB209235
MLXIP	MLX interacting protein (MLXIP), mRNA	NM_014938	Hs.437153	NM_014938
MMAA	methylmalonic aciduria (cobalamin deficiency) cblA type (MMAA), mRNA	NM_172250	Hs.452864	AK126662
MMP10	matrix metallopeptidase 10 (stromelysin 2) (MMP10), mRNA	NM_002425	Hs.2258	AK222601
MMP14	matrix metallopeptidase 14 (membrane-inserted) (MMP14), mRNA	NM_004995	Hs.2399	NM_004995
MOBKL2A	MOB1, Mps One Binder kinase activator-like 2A (yeast) (MOBKL2A), mRNA	NM_130807	Hs.86912	AK024373
MORN3	MORN repeat containing 3 (MORN3), mRNA	NM_173855	Hs.434154	NM_173855
MPHOSPH6	M-phase phosphoprotein 6, mRNA (cDNA clone IMAGE:4603429), complete cds.	BC029395	Hs.344400	BX537773
MPPE1	metallophosphoesterase 1 (MPPE1), mRNA	NM_023075	Hs.514713	NM_023075
MPZL1	myelin protein zero-like 1 (MPZL1), transcript variant 1, mRNA	NM_003953	Hs.493919	NM_003953
MPZL1	myelin protein zero-like 1 (MPZL1), transcript variant 1, mRNA	NM_003953	Hs.493919	NM_003953
MR1	major histocompatibility complex, class I-related (MR1), mRNA	NM_001531	Hs.101840	AF031469
MR1	major histocompatibility complex, class I-related (MR1), mRNA	NM_001531	Hs.101840	AF031469
MRPL24	mitochondrial ribosomal protein L24 (MRPL24), nuclear gene encoding mitochondrial protein, transcript variant 1, mRNA	NM_145729	Hs.418233	BQ883105
MRPL27	mitochondrial ribosomal protein L27 (MRPL27), nuclear gene encoding mitochondrial protein, transcript variant 1, mRNA	NM_016504	Hs.7736	NM_148571
MRPL40	mitochondrial ribosomal protein L40 (MRPL40), nuclear gene encoding mitochondrial protein, mRNA	NM_003776	Hs.431307	AK123768
MRPL53	mitochondrial ribosomal protein L53 (MRPL53), nuclear gene encoding mitochondrial protein, mRNA	NM_053050	Hs.534527	BM467462
MRPL54	mitochondrial ribosomal protein L54 (MRPL54), nuclear gene encoding mitochondrial protein, mRNA	NM_172251	Hs.356578	BM920672
MRPS11	mitochondrial ribosomal protein S11 (MRPS11), nuclear gene encoding mitochondrial protein, transcript variant 1, mRNA	NM_022839	Hs.111286	AK027059
MRPS11	mitochondrial ribosomal protein S11 (MRPS11), nuclear gene encoding mitochondrial protein, transcript variant 1, mRNA	NM_022839	Hs.111286	AK027059
MRRF	mitochondrial ribosome recycling factor (MRRF), nuclear gene encoding mitochondrial protein, transcript variant 1, mRNA	NM_138777	Hs.368011	NM_138777
MSL3L1	male-specific lethal 3-like 1 (Drosophila) (MSL3L1), transcript variant 1, mRNA	NM_078629	Hs.655288	NM_078628
MSRB3	methionine sulfoxide reductase B3 (MSRB3), transcript variant 2, mRNA	NM_001031679	Hs.339024	NM_001031679
MTCP1	cDNA FLJ38567 fis, clone HCHON2005166.	AK095886	Hs.657232	AK095886
MTDH	metadherin (MTDH), mRNA	NM_178812	Hs.377155	BC045642
MTDH	metadherin (MTDH), mRNA	NM_178812	Hs.377155	BC045642
MTDH	metadherin (MTDH), mRNA	NM_178812	Hs.377155	BC045642
MTDH	metadherin (MTDH), mRNA	NM_178812	Hs.377155	BC045642
MTDH	metadherin (MTDH), mRNA	NM_178812	Hs.377155	BC045642
MTDH	metadherin (MTDH), mRNA	NM_178812	Hs.377155	BC045642
MTDH	metadherin (MTDH), mRNA	NM_178812	Hs.377155	BC045642
MTDH	metadherin (MTDH), mRNA	NM_178812	Hs.377155	BC045642
MTDH	metadherin (MTDH), mRNA	NM_178812	Hs.377155	BC045642
MTDH	metadherin (MTDH), mRNA	NM_178812	Hs.377155	BC045642
MTMR6	myotubularin related protein 6 (MTMR6), mRNA	NM_004685	Hs.696140	NM_004685
MTPN	myotrophin (MTPN), mRNA	NM_145808	Hs.654894	AL834231
MTPN	myotrophin (MTPN), mRNA	NM_145808	Hs.654894	AL834231
MTUS1	mitochondrial tumor suppressor 1 (MTUS1), nuclear gene encoding mitochondrial protein, transcript variant 3, mRNA	NM_001001927	Unknown	
MUS81	MUS81 endonuclease homolog (S. cerevisiae) (MUS81), mRNA	NM_025128	Unknown	
MVK	mevalonate kinase (mevalonic aciduria), mRNA (cDNA clone MGC:9001 IMAGE:3921543), complete cds.	BC016140	Hs.130607	X75311
MXD1	MAX dimerization protein 1 (MXD1), mRNA	NM_002357	Hs.468908	BC098396
MXD4	MAX dimerization protein 4 (MXD4), mRNA	NM_006454	Hs.655020	AK024501
MXRA7	matrix-remodelling associated 7 (MXRA7), transcript variant 1, mRNA	NM_001008528	Hs.250723	NM_001008528
MXRA7	matrix-remodelling associated 7 (MXRA7), transcript variant 1, mRNA	NM_001008528	Hs.250723	NM_001008528
MXRA7	matrix-remodelling associated 7 (MXRA7), transcript variant 2, mRNA	NM_001008529	Hs.250723	NM_001008528
MYO1G	myosin IG (MYO1G), mRNA	NM_033054	Hs.37617	BC063854

NAB1	NGFI-A binding protein 1 (EGR1 binding protein 1) (NAB1), mRNA	NM_005966	Hs.570078	NM_005966
NAG	neuroblastoma-amplified protein (NAG), mRNA	NM_015909	Hs.467759	NM_015909
NAT5	N-acetyltransferase 5 (NAT5), transcript variant 1, mRNA	NM_016100	Hs.368783	CR607693
NAV1	neuron navigator 1 (NAV1), mRNA	NM_020443	Hs.585374	AY043013
NAV1	neuron navigator 1 (NAV1), mRNA	NM_020443	Hs.585374	AY043013
NAV3	neuron navigator 3 (NAV3), mRNA	NM_014903	Hs.655301	NM_014903
NCOA4	nuclear receptor coactivator 4 (NCOA4), mRNA	NM_005437	Hs.696095	AL162047
NCOA4	nuclear receptor coactivator 4 (NCOA4), mRNA	NM_005437	Hs.696095	AL162047
NCOA7	nuclear receptor coactivator 7 (NCOA7), mRNA	NM_181782	Hs.171426	AL834442
NCOA7	nuclear receptor coactivator 7 (NCOA7), mRNA	NM_181782	Hs.171426	AL834442
NDFIP1	Nedd4 family interacting protein 1 (NDFIP1), mRNA	NM_030571	Hs.9788	AK124884
NDRG1	N-myc downstream regulated gene 1 (NDRG1), mRNA	NM_006096	Hs.372914	AK124709
NDUFA1	NADH dehydrogenase (ubiquinone) 1 alpha subcomplex, 1, 7.5kDa (NDUFA1), nuclear gene encoding mitochondrial protein, mRNA	NM_004541	Hs.534168	BM918631
NDUFA1	NADH dehydrogenase (ubiquinone) 1 alpha subcomplex, 1, 7.5kDa (NDUFA1), nuclear gene encoding mitochondrial protein, mRNA	NM_004541	Hs.534168	BM918631
NDUFA9	NADH dehydrogenase (ubiquinone) 1 alpha subcomplex, 9, 39kDa (NDUFA9), mRNA	NM_005002	Hs.75227	BM546373
NDUFA9	NADH dehydrogenase (ubiquinone) 1 alpha subcomplex, 9, 39kDa (NDUFA9), mRNA	NM_005002	Hs.75227	BM546373
NDUFB5	NADH dehydrogenase (ubiquinone) 1 beta subcomplex, 5, 16kDa (NDUFB5), nuclear gene encoding mitochondrial protein, mRNA	NM_002492	Hs.518424	BX648034
NDUFB7	NADH dehydrogenase (ubiquinone) 1 beta subcomplex, 7, 18kDa (NDUFB7), nuclear gene encoding mitochondrial protein, mRNA	NM_004146	Hs.532853	BQ058291
NDUFB7	NADH dehydrogenase (ubiquinone) 1 beta subcomplex, 7, 18kDa (NDUFB7), nuclear gene encoding mitochondrial protein, mRNA	NM_004146	Hs.532853	BQ058291
NECAP1	NECAP endocytosis associated 1 (NECAP1), mRNA	NM_015509	Hs.555927	AK094805
NEK11	NIMA (never in mitosis gene a)- related kinase 11 (NEK11), transcript variant 2, mRNA	NM_145910	Hs.657336	AB071996
NEK7	NIMA (never in mitosis gene a)-related kinase 7 (NEK7), mRNA	NM_133494	Hs.24119	BC043003
NFATC3	nuclear factor of activated T-cells, cytoplasmic, calcineurin-dependent 3 (NFATC3), transcript variant 4, mRNA	NM_173164	Hs.632209	NM_004555
NFIB	nuclear factor I/B (NFIB), mRNA	NM_005596	Hs.699215	NM_005596
NFIB	nuclear factor I/B (NFIB), mRNA	NM_005596	Hs.699215	NM_005596
NFIC	nuclear factor I/C (CCAAT-binding transcription factor) (NFIC), transcript variant 1, mRNA	NM_005597	Hs.170131	NM_205843
NFIX	nuclear factor I/X (CCAAT-binding transcription factor) (NFIX), mRNA	NM_002501	Hs.257970	NM_002501
NFKB1	nuclear factor of kappa light polypeptide gene enhancer in B-cells 1 (p105) (NFKB1), mRNA	NM_003998	Hs.654408	NM_003998
NFKB1	nuclear factor of kappa light polypeptide gene enhancer in B-cells 1 (p105) (NFKB1), mRNA	NM_003998	Hs.654408	NM_003998
NFKB1	nuclear factor of kappa light polypeptide gene enhancer in B-cells 1 (p105) (NFKB1), mRNA	NM_003998	Hs.654408	NM_003998
NFKB1	nuclear factor of kappa light polypeptide gene enhancer in B-cells 1 (p105) (NFKB1), mRNA	NM_003998	Hs.654408	NM_003998
NFKB1	nuclear factor of kappa light polypeptide gene enhancer in B-cells 1 (p105) (NFKB1), mRNA	NM_003998	Hs.654408	NM_003998
NFKB1	nuclear factor of kappa light polypeptide gene enhancer in B-cells 1 (p105) (NFKB1), mRNA	NM_003998	Hs.654408	NM_003998
NFKB1	nuclear factor of kappa light polypeptide gene enhancer in B-cells 1 (p105) (NFKB1), mRNA	NM_003998	Hs.654408	NM_003998
NFKB1	nuclear factor of kappa light polypeptide gene enhancer in B-cells 1 (p105) (NFKB1), mRNA	NM_003998	Hs.654408	NM_003998
NFKB1	nuclear factor of kappa light polypeptide gene enhancer in B-cells 1 (p105) (NFKB1), mRNA	NM_003998	Hs.654408	NM_003998
NFKB1	nuclear factor of kappa light polypeptide gene enhancer in B-cells 1 (p105) (NFKB1), mRNA	NM_003998	Hs.654408	NM_003998
NFKBIL1	nuclear factor of kappa light polypeptide gene enhancer in B-cells inhibitor-like 1 (NFKBIL1), mRNA	NM_005007	Hs.2764	BM559803
NIBP	NIK and IKK{beta} binding protein (NIBP), mRNA	NM_031466	Hs.654911	BX647286
NID1	nidogen 1 (NID1), mRNA	NM_002508	Hs.356624	NM_002508
NINJ1	ninjurin 1 (NINJ1), mRNA	NM_004148	Hs.494457	BQ073581
NIPSNAP3B	nipsnap homolog 3B (C. elegans) (NIPSNAP3B), mRNA	NM_018376	Hs.567532	BX647652
NISCH	nischarin, mRNA (cDNA clone IMAGE:5761683), partial cds.	BC050552	Hs.435290	NM_007184
NMD3	NMD3 homolog (S. cerevisiae) (NMD3), mRNA	NM_015938	Hs.656143	AK094749
NME6	non-metastatic cells 6, protein expressed in (nucleoside-diphosphate kinase) (NME6), mRNA	NM_005793	Hs.465558	BQ071537
NNMT	nicotinamide N-methyltransferase (NNMT), mRNA	NM_006169	Hs.503911	AK097984
NOD1	nucleotide-binding oligomerization domain containing 1 (NOD1), mRNA	NM_006092	Hs.405153	AK023969
NOSIP	nitric oxide synthase interacting protein (NOSIP), mRNA	NM_015953	Hs.7236	BC047314
NOSIP	nitric oxide synthase interacting protein (NOSIP), mRNA	NM_015953	Hs.7236	BC047314
NR3C2	nuclear receptor subfamily 3, group C, member 2 (NR3C2), mRNA	NM_000901	Hs.163924	M16801

NRAS	neuroblastoma RAS viral (v-ras) oncogene homolog (NRAS), mRNA	NM_002524	Hs.486502	X02751
NRP1	neuropilin 1 (NRP1), transcript variant 3, mRNA	NM_001024629	Hs.131704	CR749333
NRSN2	neurensin 2 (NRSN2), mRNA	NM_024958	Hs.416024	AL136915
NSUN3	NOL1/NOP2/Sun domain family, member 3 (NSUN3), mRNA	NM_022072	Hs.656338	BC020602
NSUN4	NOL1/NOP2/Sun domain family, member 4 (NSUN4), mRNA	NM_199044	Hs.163424	NM_199044
NT5C3L	5'-nucleotidase, cytosolic III-like (NT5C3L), mRNA	NM_052935	Hs.237536	CR602296
NT5E	5'-nucleotidase, ecto (CD73) (NT5E), mRNA	NM_002526	Hs.153952	BC065937
NTAN1	N-terminal asparagine amidase (NTAN1), mRNA	NM_173474	Hs.592045	AK092915
NTN4	netrin 4 (NTN4), mRNA	NM_021229	Hs.201034	NM_021229
NUDT14	nudix (nucleoside diphosphate linked moiety X)-type motif 14 (NUDT14), mRNA	NM_177533	Hs.526432	BQ068667
NUDT18	nudix (nucleoside diphosphate linked moiety X)-type motif 18 (NUDT18), mRNA	NM_024815	Hs.527101	AK124446
NUDT2	nudix (nucleoside diphosphate linked moiety X)-type motif 2 (NUDT2), transcript variant 1, mRNA	NM_001161	Hs.493767	BM802700
NUDT4	nudix (nucleoside diphosphate linked moiety X)-type motif 4 (NUDT4), transcript variant 2, mRNA	NM_199040	Hs.591008	NM_199040
NUDT4	nudix (nucleoside diphosphate linked moiety X)-type motif 4 (NUDT4), transcript variant 2, mRNA	NM_199040	Hs.591008	NM_199040
OAF	OAF homolog (Drosophila) (OAF), mRNA	NM_178507	Hs.445081	BC047726
OAF	OAF homolog (Drosophila) (OAF), mRNA	NM_178507	Hs.445081	BC047726
OAS1	2',5'-oligoadenylate synthetase 1, 40/46kDa (OAS1), transcript variant 2, mRNA	NM_002534	Hs.524760	NM_016816
OAZ3	ornithine decarboxylase antizyme 3 (OAZ3), mRNA	NM_016178	Hs.699818	AK125876
OAZ3	ornithine decarboxylase antizyme 3 (OAZ3), mRNA	NM_016178	Hs.699818	AK125876
OBFC1	oligonucleotide/oligosaccharide-binding fold containing 1 (OBFC1), mRNA	NM_024928	Hs.134491	BM551311
OBFC2A	oligonucleotide/oligosaccharide-binding fold containing 2A (OBFC2A), mRNA	NM_001031716	Hs.591610	AL832659
OPHN1	oligophrenin 1 (OPHN1), mRNA	NM_002547	Hs.128824	AJ001189
OR1L3	olfactory receptor, family 1, subfamily L, member 3 (OR1L3), mRNA	NM_001005234	Hs.626839	NM_001005234
OR2C1	olfactory receptor, family 2, subfamily C, member 1 (OR2C1), mRNA	NM_012368	Hs.258574	BX324671
OR51E2	olfactory receptor, family 51, subfamily E, member 2 (OR51E2), mRNA	NM_030774	Hs.501758	NM_030774
ORAOV1	oral cancer overexpressed 1 (ORAOV1), mRNA	NM_153451	Hs.523854	AK126490
OSAP	ovary-specific acidic protein (OSAP), mRNA	NM_032623	Hs.84549	AK023115
OSBPL5	oxysterol binding protein-like 5 (OSBPL5), transcript variant 1, mRNA	NM_020896	Hs.436166	NM_020896
OSMR	oncostatin M receptor, mRNA (cDNA clone IMAGE:4043935), complete cds.	BC010943	Hs.120658	NM_003999
OTOP3	otopetrin 3 (OTOP3), mRNA	NM_178233	Hs.454407	NM_178233
OTOR	otoraplin (OTOR), mRNA	NM_020157	Hs.41119	NM_020157
OVCA2	candidate tumor suppressor in ovarian cancer 2 (OVCA2), mRNA	NM_080822	Hs.513856	AK090530
P117	hypothetical protein P117 (P117), mRNA	NM_205767	Hs.356626	BM563199
P4HA3	procollagen-proline, 2-oxoglutarate 4-dioxygenase (proline 4-hydroxylase), alpha polypeptide III (P4HA3), mRNA	NM_182904	Hs.660541	AL833965
PAEP	progestagen-associated endometrial protein (placental protein 14, pregnancy-associated endometrial alpha-2-globulin, alpha uterine protein) (PAEP), transcript variant 2, mRNA	NM_002571	Hs.532325	AK094008
PAF1	Paf1, RNA polymerase II associated factor, homolog (S. cerevisiae) (PAF1), mRNA	NM_019088	Hs.466714	NM_019088
PARP3	poly (ADP-ribose) polymerase family, member 3 (PARP3), transcript variant 3, mRNA	NM_001003935	Hs.271742	NM_001003935
PARP3	poly (ADP-ribose) polymerase family, member 3 (PARP3), transcript variant 3, mRNA	NM_001003935	Hs.271742	NM_001003935
PARP9	poly (ADP-ribose) polymerase family, member 9 (PARP9), mRNA	NM_031458	Hs.518200	AB209742
PARVA	parvin, alpha (PARVA), mRNA	NM_018222	Hs.607144	AL832682
PARVA	parvin, alpha (PARVA), mRNA	NM_018222	Hs.607144	AL832682
PCDH12	protocadherin 12 (PCDH12), mRNA	NM_016580	Hs.439474	NM_016580
PCMT1	protein-L-isoaspartate (D-aspartate) O-methyltransferase (PCMT1), mRNA	NM_005389	Hs.279257	CR615194
PDE10A	phosphodiesterase 10A (PDE10A), mRNA	NM_006661	Hs.584856	AB020593
PDE1C	phosphodiesterase 1C, calmodulin-dependent 70kDa (PDE1C), mRNA	NM_005020	Hs.655694	AK091734
PDE4B	phosphodiesterase 4B, cAMP-specific (phosphodiesterase E4 dunce homolog, Drosophila) (PDE4B), transcript variant d, mRNA	NM_001037341	Hs.198072	NM_001037341
PDE4D	phosphodiesterase 4D, cAMP-specific (phosphodiesterase E3 dunce homolog, Drosophila), mRNA (cDNA clone IMAGE:4280941), complete cds.	BC008390	Hs.654358	NM_006203
PDE8A	phosphodiesterase 8A (PDE8A), transcript variant 1, mRNA	NM_002605	Hs.9333	BC060762
PDGFB	platelet-derived growth factor beta polypeptide (simian sarcoma viral (v-sis) oncogene homolog) (PDGFB), transcript variant 1, mRNA	NM_002608	Hs.1976	M12783
PDLIM4	PDZ and LIM domain 4 (PDLIM4), mRNA	NM_003687	Hs.424312	NM_003687

PDLIM7	PDZ and LIM domain 7 (enigma) (PDLIM7), transcript variant 1, mRNA	NM_005451	Hs.533040	AK096826
PDZD11	PDZ domain containing 11 (PDZD11), mRNA	NM_016484	Hs.11042	BX537725
PELO	PRO1770 mRNA, complete cds.	AF118075	Hs.669791	AF118075
PER1	period homolog 1 (Drosophila), mRNA (cDNA clone IMAGE:5215552), complete cds.	BC028207	Hs.445534	NM_002616
PGGT1B	protein geranylgeranyltransferase type I, beta subunit (PGGT1B), mRNA	NM_005023	Hs.254006	NM_005023
PGK1	Phosphoglycerate kinase 1 (EC 2.7.2.3) (Primer recognition protein 2) (PRP 2).	ENST00000373316	Unknown	
PHF11	PHD finger protein 11 (PHF11), transcript variant 1, mRNA	NM_001040443	Hs.369039	NM_001040443
PHF20L1	PHD finger protein 20-like 1 (PHF20L1), transcript variant 2, mRNA	NM_032205	Hs.304362	NM_016018
PHLDA1	pleckstrin homology-like domain, family A, member 1 (PHLDA1), mRNA	NM_007350	Hs.602085	NM_007350
PHLDB2	pleckstrin homology-like domain, family B, member 2 (PHLDB2), mRNA	NM_145753	Hs.477114	AL832205
PICALM	phosphatidylinositol binding clathrin assembly protein (PICALM), transcript variant 1, mRNA	NM_007166	Hs.163893	NM_007166
PICALM	phosphatidylinositol binding clathrin assembly protein (PICALM), transcript variant 1, mRNA	NM_007166	Hs.163893	NM_007166
PIP5K2B	phosphatidylinositol-4-phosphate 5-kinase, type II, beta (PIP5K2B), mRNA	NM_003559	Hs.260603	NM_003559
PIP5K2B	phosphatidylinositol-4-phosphate 5-kinase, type II, beta (PIP5K2B), mRNA	NM_003559	Hs.260603	NM_003559
PLA2G2E	phospholipase A2, group IIE (PLA2G2E), mRNA	NM_014589	Hs.272372	AF189279
PLA2G4A	phospholipase A2, group IVA (cytosolic, calcium-dependent) (PLA2G4A), mRNA	NM_024420	Hs.497200	NM_024420
PLA2G4A	phospholipase A2, group IVA (cytosolic, calcium-dependent) (PLA2G4A), mRNA	NM_024420	Hs.497200	NM_024420
PLA2G4A	phospholipase A2, group IVA (cytosolic, calcium-dependent) (PLA2G4A), mRNA	NM_024420	Hs.497200	NM_024420
PLA2G4A	phospholipase A2, group IVA (cytosolic, calcium-dependent) (PLA2G4A), mRNA	NM_024420	Hs.497200	NM_024420
PLA2G4A	phospholipase A2, group IVA (cytosolic, calcium-dependent) (PLA2G4A), mRNA	NM_024420	Hs.497200	NM_024420
PLA2G4A	phospholipase A2, group IVA (cytosolic, calcium-dependent) (PLA2G4A), mRNA	NM_024420	Hs.497200	NM_024420
PLA2G4A	phospholipase A2, group IVA (cytosolic, calcium-dependent) (PLA2G4A), mRNA	NM_024420	Hs.497200	NM_024420
PLA2G4A	phospholipase A2, group IVA (cytosolic, calcium-dependent) (PLA2G4A), mRNA	NM_024420	Hs.497200	NM_024420
PLA2G4A	phospholipase A2, group IVA (cytosolic, calcium-dependent) (PLA2G4A), mRNA	NM_024420	Hs.497200	NM_024420
PLA2G4A	phospholipase A2, group IVA (cytosolic, calcium-dependent) (PLA2G4A), mRNA	NM_024420	Hs.497200	NM_024420
PLCL1	phospholipase C-like 1 (PLCL1), mRNA	NM_006226	Hs.153322	NM_006226
PLD1	phospholipase D1, phosphatidylcholine-specific (PLD1), mRNA	NM_002662	Hs.382865	AB209907
PLD1	phospholipase D1, phosphatidylcholine-specific (PLD1), mRNA	NM_002662	Hs.382865	AB209907
PLD1	cDNA FLJ34578 fis, clone KIDNE2008404, highly similar to PHOSPHOLIPASE D1 (EC 3.1.4.4).	AK091897	Hs.382865	AB209907
PLDN	pallidin homolog (mouse) (PLDN), mRNA	NM_012388	Hs.7037	AK128626
PLDN	pallidin homolog (mouse) (PLDN), mRNA	NM_012388	Hs.7037	AK128626
PLEK2	pleckstrin 2 (PLEK2), mRNA	NM_016445	Hs.170473	BM913044
PLK3	polo-like kinase 3 (Drosophila) (PLK3), mRNA	NM_004073	Hs.632415	AJ293866
PLOD2	procollagen-lysine, 2-oxoglutarate 5-dioxygenase 2 (PLOD2), transcript variant 1, mRNA	NM_182943	Hs.477866	NM_182943
PLSCR4	phospholipid scramblase 4 (PLSCR4), mRNA	NM_020353	Hs.477869	BC028354
PLXNA4A	mRNA for KIAA1550 protein, partial cds.	AB046770	Hs.511454	AB046770
PMP22	peripheral myelin protein 22 (PMP22), transcript variant 1, mRNA	NM_000304	Hs.372031	NM_000304
PNPLA8	patatin-like phospholipase domain containing 8 (PNPLA8), mRNA	NM_015723	Hs.617340	AL834147
POLE4	polymerase (DNA-directed), epsilon 4 (p12 subunit) (POLE4), mRNA	NM_019896	Hs.469060	BM924454
POLH	cDNA: FLJ21978 fis, clone HEP06046, highly similar to AF158185 xeroderma pigmentosum variant RAD30 (RAD30) mRNA.	AK025631	Hs.655467	AB024313
POLH	polymerase (DNA directed), eta (POLH), mRNA	NM_006502	Hs.655467	AB024313
POLR2J	polymerase (RNA) II (DNA directed) polypeptide J, 13.3kDa (POLR2J), mRNA	NM_006234	Hs.654952	BM910996
POLR2L	polymerase (RNA) II (DNA directed) polypeptide L, 7.6kDa (POLR2L), mRNA	NM_021128	Hs.441072	BM919305
POMZP3	POM (POM121 homolog, rat) and ZP3 fusion (POMZP3), transcript variant 2, mRNA	NM_152992	Hs.488877	CR603033
POP4	processing of precursor 4, ribonuclease P/MRP subunit (S. cerevisiae) (POP4), mRNA	NM_006627	Hs.699350	CR936714
PPFIBP1	PTPRF interacting protein, binding protein 1 (liprin beta 1) (PPFIBP1), transcript variant 1, mRNA	NM_003622	Hs.172445	NM_003622
PPFIBP1	PTPRF interacting protein, binding protein 1 (liprin beta 1) (PPFIBP1), transcript variant 2, mRNA	NM_177444	Hs.172445	NM_003622
PPIB	peptidylprolyl isomerase B (cyclophilin B) (PPIB), mRNA	NM_000942	Hs.434937	BQ049986
PPIE	peptidylprolyl isomerase E (cyclophilin E) (PPIE), transcript variant 2, mRNA	NM_203456	Hs.700626	CR617834
PPP1R11	protein phosphatase 1, regulatory (inhibitor) subunit 11 (PPP1R11), mRNA	NM_021959	Hs.82887	AF070599
PPP1R14D	protein phosphatase 1, regulatory (inhibitor) subunit 14D (PPP1R14D), mRNA	NM_017726	Hs.192927	NM_017726

PPP1R15A	protein phosphatase 1, regulatory (inhibitor) subunit 15A (PPP1R15A), mRNA	NM_014330	Hs.631593	AK225632
PPP1R9B	protein phosphatase 1, regulatory subunit 9B, spinophilin (PPP1R9B), mRNA	NM_032595	Hs.514323	NM_032595
PPP2CB	protein phosphatase 2 (formerly 2A), catalytic subunit, beta isoform (PPP2CB), transcript variant 2, mRNA	NM_001009552	Hs.491440	NM_001009552
PRAMEF8	PRAME family member 8 (PRAMEF8), mRNA	NM_001012276	Hs.631872	NM_001012276
PRDM15	C21orf83 isoform C (C21orf83) mRNA, complete cds.	AY063456	Hs.473893	AB051813
PRDX2	peroxiredoxin 2 (PRDX2), nuclear gene encoding mitochondrial protein, transcript variant 3, mRNA	NM_181738	Hs.695971	BM805899
PRDX5	peroxiredoxin 5 (PRDX5), nuclear gene encoding mitochondrial protein, transcript variant 1, mRNA	NM_012094	Hs.502823	BM462424
PRKAG2	protein kinase, AMP-activated, gamma 2 non-catalytic subunit (PRKAG2), transcript variant a, mRNA	NM_016203	Hs.647072	CR933725
PRKCH	protein kinase C, eta (PRKCH), mRNA	NM_006255	Hs.333907	BC037268
PROSC	proline synthetase co-transcribed homolog (bacterial) (PROSC), mRNA	NM_007198	Hs.304792	NM_007198
PRR14	proline rich 14 (PRR14), mRNA	NM_024031	Hs.293629	BC050677
PRR8	proline rich 8, mRNA (cDNA clone MGC:20460 IMAGE:4542314), complete cds.	BC011923	Hs.591815	NM_001008408
PRRG1	proline rich Gla (G-carboxyglutamic acid) 1 (PRRG1), mRNA	NM_000950	Hs.190341	BC030786
PRSS23	protease, serine, 23 (PRSS23), mRNA	NM_007173	Hs.25338	AL832007
PRSS23	protease, serine, 23 (PRSS23), mRNA	NM_007173	Hs.25338	AL832007
PSCD2	pleckstrin homology, Sec7 and coiled-coil domains 2 (cytohesin-2) (PSCD2), transcript variant 2, mRNA	NM_004228	Hs.144011	AF318380
PSG9	pregnancy specific beta-1-glycoprotein 9 (PSG9), mRNA	NM_002784	Hs.502092	M94890
PSMB7	proteasome (prosome, macropain) subunit, beta type, 7 (PSMB7), mRNA	NM_002799	Hs.213470	BU588892
PSMC2	proteasome (prosome, macropain) 26S subunit, ATPase, 2 (PSMC2), mRNA	NM_002803	Hs.437366	AB075520
PSMD5	proteasome (prosome, macropain) 26S subunit, non-ATPase, 5 (PSMD5), mRNA	NM_005047	Hs.193725	NM_005047
PSMD9	proteasome (prosome, macropain) 26S subunit, non-ATPase, 9 (PSMD9), mRNA	NM_002813	Hs.131151	BC028421
PSME1	proteasome (prosome, macropain) activator subunit 1 (PA28 alpha) (PSME1), transcript variant 1, mRNA	NM_006263	Hs.75348	BQ073751
PSORS1C1	psoriasis susceptibility 1 candidate 1 (PSORS1C1), mRNA	NM_014068	Hs.310958	BX640954
PSTPIP2	proline-serine-threonine phosphatase interacting protein 2 (PSTPIP2), mRNA	NM_024430	Hs.567384	NM_024430
PSTPIP2	proline-serine-threonine phosphatase interacting protein 2 (PSTPIP2), mRNA	NM_024430	Hs.567384	NM_024430
PTGS2	prostaglandin-endoperoxide synthase 2 (prostaglandin G/H synthase and cyclooxygenase) (PTGS2), mRNA	NM_000963	Hs.196384	NM_000963
PTGS2	prostaglandin-endoperoxide synthase 2 (prostaglandin G/H synthase and cyclooxygenase) (PTGS2), mRNA	NM_000963	Hs.196384	NM_000963
PTGS2	prostaglandin-endoperoxide synthase 2 (prostaglandin G/H synthase and cyclooxygenase) (PTGS2), mRNA	NM_000963	Hs.196384	NM_000963
PTGS2	prostaglandin-endoperoxide synthase 2 (prostaglandin G/H synthase and cyclooxygenase) (PTGS2), mRNA	NM_000963	Hs.196384	NM_000963
PTGS2	prostaglandin-endoperoxide synthase 2 (prostaglandin G/H synthase and cyclooxygenase) (PTGS2), mRNA	NM_000963	Hs.196384	NM_000963
PTGS2	prostaglandin-endoperoxide synthase 2 (prostaglandin G/H synthase and cyclooxygenase) (PTGS2), mRNA	NM_000963	Hs.196384	NM_000963
PTGS2	prostaglandin-endoperoxide synthase 2 (prostaglandin G/H synthase and cyclooxygenase) (PTGS2), mRNA	NM_000963	Hs.196384	NM_000963
PTGS2	prostaglandin-endoperoxide synthase 2 (prostaglandin G/H synthase and cyclooxygenase) (PTGS2), mRNA	NM_000963	Hs.196384	NM_000963
PTGS2	prostaglandin-endoperoxide synthase 2 (prostaglandin G/H synthase and cyclooxygenase) (PTGS2), mRNA	NM_000963	Hs.196384	NM_000963
PTGS2	prostaglandin-endoperoxide synthase 2 (prostaglandin G/H synthase and cyclooxygenase) (PTGS2), mRNA	NM_000963	Hs.196384	NM_000963
PTGS2	prostaglandin-endoperoxide synthase 2 (prostaglandin G/H synthase and cyclooxygenase) (PTGS2), mRNA	NM_000963	Hs.196384	NM_000963
PTP4A1	protein tyrosine phosphatase type IVA, member 1 (PTP4A1), mRNA	NM_003463	Hs.227777	NM_003463
PTPRE	protein tyrosine phosphatase, receptor type, E (PTPRE), transcript variant 1, mRNA	NM_006504	Hs.127022	BX648180
PTPRE	protein tyrosine phosphatase, receptor type, E (PTPRE), transcript variant 1, mRNA	NM_006504	Hs.127022	BX648180
PTRF	polymerase I and transcript release factor (PTRF), mRNA	NM_012232	Hs.437191	BC066123
PTS	6-pyruvoyltetrahydropterin synthase (PTS), mRNA	NM_000317	Hs.503860	BM928025
PTTG1IP	pituitary tumor-transforming 1 interacting protein (PTTG1IP), mRNA	NM_004339	Hs.474010	AK095586
PTX3	pentraxin-related gene, rapidly induced by IL-1 beta (PTX3), mRNA	NM_002852	Hs.591286	NM_002852
PTX3	pentraxin-related gene, rapidly induced by IL-1 beta (PTX3), mRNA	NM_002852	Hs.591286	NM_002852
PTX3	pentraxin-related gene, rapidly induced by IL-1 beta (PTX3), mRNA	NM_002852	Hs.591286	NM_002852
PTX3	pentraxin-related gene, rapidly induced by IL-1 beta (PTX3), mRNA	NM_002852	Hs.591286	NM_002852
PTX3	pentraxin-related gene, rapidly induced by IL-1 beta (PTX3), mRNA	NM_002852	Hs.591286	NM_002852

PTX3	pentraxin-related gene, rapidly induced by IL-1 beta (PTX3), mRNA	NM_002852	Hs.591286	NM_002852
PTX3	pentraxin-related gene, rapidly induced by IL-1 beta (PTX3), mRNA	NM_002852	Hs.591286	NM_002852
PTX3	pentraxin-related gene, rapidly induced by IL-1 beta (PTX3), mRNA	NM_002852	Hs.591286	NM_002852
PTX3	pentraxin-related gene, rapidly induced by IL-1 beta (PTX3), mRNA	NM_002852	Hs.591286	NM_002852
PTX3	pentraxin-related gene, rapidly induced by IL-1 beta (PTX3), mRNA	NM_002852	Hs.591286	NM_002852
PUS7L	pseudouridylate synthase 7 homolog (S. cerevisiae)-like (PUS7L), mRNA	NM_031292	Hs.445814	NM_001098615
PVRL4	poliovirus receptor-related 4 (PVRL4), mRNA	NM_030916	Hs.492490	AK027753
QPCT	glutaminyl-peptide cyclotransferase (glutaminyl cyclase) (QPCT), mRNA	NM_012413	Hs.79033	NM_012413
QRICH2	glutamine rich 2 (QRICH2), mRNA	NM_032134	Hs.252739	AL136774
RAB2	RAB2, member RAS oncogene family (RAB2), mRNA	NM_002865	Hs.369017	AL137321
RAB27A	RAB27A, member RAS oncogene family (RAB27A), transcript variant 1, mRNA	NM_004580	Hs.654978	NM_004580
RAB32	RAB32, member RAS oncogene family (RAB32), mRNA	NM_006834	Hs.287714	BM546299
RAB32	RAB32, member RAS oncogene family (RAB32), mRNA	NM_006834	Hs.287714	BM546299
RAB6A	RAB6A, member RAS oncogene family (RAB6A), transcript variant 1, mRNA	NM_002869	Hs.503222	NM_002869
RAB6C	cDNA FLJ30942 fis, clone FEBRA2007551, moderately similar to RAS-RELATED PROTEIN RAB-6.	AK055504	Hs.591552	AL136727
RABIF	RAB interacting factor (RABIF), mRNA	NM_002871	Hs.90875	NM_002871
RABIF	RAB interacting factor (RABIF), mRNA	NM_002871	Hs.90875	NM_002871
RABL3	RAB, member of RAS oncogene family-like 3 (RABL3), mRNA	NM_173825	Hs.444360	NM_173825
RAD51C	RAD51 homolog C (S. cerevisiae) (RAD51C), transcript variant 2, mRNA	NM_002876	Hs.412587	BC073161
RANBP9	RAN binding protein 9 (RANBP9), mRNA	NM_005493	Hs.306242	BC052781
RAP1GDS1	RAP1, GTP-GDP dissociation stimulator 1 (RAP1GDS1), mRNA	NM_021159	Hs.132858	NM_001100426
RAPGEF4	Rap guanine nucleotide exchange factor (GEF) 4 (RAPGEF4), mRNA	NM_007023	Hs.470646	AB209681
RAPGEF4	Rap guanine nucleotide exchange factor (GEF) 4 (RAPGEF4), mRNA	NM_007023	Hs.470646	AB209681
RAPH1	Ras association (RalGDS/AF-6) and pleckstrin homology domains 1 (RAPH1), transcript variant 1, mRNA	NM_213589	Hs.471162	NM_213589
RARRES1	retinoic acid receptor responder (tazarotene induced) 1 (RARRES1), transcript variant 2, mRNA	NM_002888	Hs.131269	NM_206963
RASSF8	clone B4-E11 carcinoma associated protein HOJ-1 mRNA, complete cds, alternatively spliced.	AY665468	Hs.696433	AY665468
RBL2	retinoblastoma-like 2 (p130) (RBL2), mRNA	NM_005611	Hs.513609	BC034490
RBM9	RNA binding motif protein 9 (RBM9), transcript variant 1, mRNA	NM_001031695	Hs.282998	NM_001031695
RBMS3	RNA binding motif, single stranded interacting protein (RBMS3), transcript variant 2, mRNA	NM_014483	Hs.696468	AL831860
RCN1	reticulocalbin 1, EF-hand calcium binding domain (RCN1), mRNA	NM_002901	Hs.97887	AK126419
RELA	v-rel reticuloendotheliosis viral oncogene homolog A, nuclear factor of kappa light polypeptide gene enhancer in B-cells 3, p65 (avian), mRNA (cDNA clone MGC:131774 IMAGE:6019711), complete cds.	BC110830	Hs.502875	BC110830
RELA	v-rel reticuloendotheliosis viral oncogene homolog A, nuclear factor of kappa light polypeptide gene enhancer in B-cells 3, p65 (avian), mRNA (cDNA clone MGC:131774 IMAGE:6019711), complete cds.	BC110830	Hs.502875	BC110830
RELA	v-rel reticuloendotheliosis viral oncogene homolog A, nuclear factor of kappa light polypeptide gene enhancer in B-cells 3, p65 (avian), mRNA (cDNA clone MGC:131774 IMAGE:6019711), complete cds.	BC110830	Hs.502875	BC110830
RELA	v-rel reticuloendotheliosis viral oncogene homolog A, nuclear factor of kappa light polypeptide gene enhancer in B-cells 3, p65 (avian), mRNA (cDNA clone MGC:131774 IMAGE:6019711), complete cds.	BC110830	Hs.502875	BC110830
RELA	v-rel reticuloendotheliosis viral oncogene homolog A, nuclear factor of kappa light polypeptide gene enhancer in B-cells 3, p65 (avian), mRNA (cDNA clone MGC:131774 IMAGE:6019711), complete cds.	BC110830	Hs.502875	BC110830
RELA	v-rel reticuloendotheliosis viral oncogene homolog A, nuclear factor of kappa light polypeptide gene enhancer in B-cells 3, p65 (avian), mRNA (cDNA clone MGC:131774 IMAGE:6019711), complete cds.	BC110830	Hs.502875	BC110830
RELA	v-rel reticuloendotheliosis viral oncogene homolog A, nuclear factor of kappa light polypeptide gene enhancer in B-cells 3, p65 (avian), mRNA (cDNA clone MGC:131774 IMAGE:6019711), complete cds.	BC110830	Hs.502875	BC110830
RELA	v-rel reticuloendotheliosis viral oncogene homolog A, nuclear factor of kappa light polypeptide gene enhancer in B-cells 3, p65 (avian), mRNA (cDNA clone MGC:131774 IMAGE:6019711), complete cds.	BC110830	Hs.502875	BC110830
RELA	v-rel reticuloendotheliosis viral oncogene homolog A, nuclear factor of kappa light polypeptide gene enhancer in B-cells 3, p65 (avian), mRNA (cDNA clone MGC:131774 IMAGE:6019711), complete cds.	BC110830	Hs.502875	BC110830
RELA	v-rel reticuloendotheliosis viral oncogene homolog A, nuclear factor of kappa light polypeptide gene enhancer in B-cells 3, p65 (avian), mRNA (cDNA clone MGC:131774 IMAGE:6019711), complete cds.	BC110830	Hs.502875	BC110830
RFK	riboflavin kinase (RFK), mRNA	NM_018339	Hs.37558	NM_018339
RGMB	RGM domain family, member B (RGMB), transcript variant 1, mRNA	NM_001012761	Hs.526902	NM_001012761

RGS20	regulator of G-protein signalling 20 (RGS20), transcript variant 1, mRNA	NM_170587	Hs.368733	NM_170587
RGS4	regulator of G-protein signalling 4 (RGS4), mRNA	NM_005613	Hs.386726	AB209019
RGS4	regulator of G-protein signalling 4 (RGS4), mRNA	NM_005613	Hs.386726	AB209019
RHOA	ras homolog gene family, member A (RHOA), mRNA	NM_001664	Hs.247077	NM_001664
RHOA	ras homolog gene family, member A (RHOA), mRNA	NM_001664	Hs.247077	NM_001664
RHOA	ras homolog gene family, member A (RHOA), mRNA	NM_001664	Hs.247077	NM_001664
RHOA	ras homolog gene family, member A (RHOA), mRNA	NM_001664	Hs.247077	NM_001664
RHOA	ras homolog gene family, member A (RHOA), mRNA	NM_001664	Hs.247077	NM_001664
RHOA	ras homolog gene family, member A (RHOA), mRNA	NM_001664	Hs.247077	NM_001664
RHOA	ras homolog gene family, member A (RHOA), mRNA	NM_001664	Hs.247077	NM_001664
RHOA	ras homolog gene family, member A (RHOA), mRNA	NM_001664	Hs.247077	NM_001664
RHOA	ras homolog gene family, member A (RHOA), mRNA	NM_001664	Hs.247077	NM_001664
RHOA	ras homolog gene family, member A (RHOA), mRNA	NM_001664	Hs.247077	NM_001664
RHOA	ras homolog gene family, member A (RHOA), mRNA	NM_001664	Hs.247077	NM_001664
RHOA	ras homolog gene family, member A (RHOA), mRNA	NM_001664	Hs.247077	NM_001664
RHOC	ras homolog gene family, member C (RHOC), transcript variant 1, mRNA	NM_175744	Hs.502659	AK094474
RIN3	Ras and Rab interactor 3 (RIN3), mRNA	NM_024832	Hs.326822	AK090451
RIOK3	RIO kinase 3 (yeast) (RIOK3), transcript variant 2, mRNA	NM_145906	Unknown	
RIOK3	RIO kinase 3 (yeast) (RIOK3), transcript variant 2, mRNA	NM_145906	Unknown	
RIPK1	receptor (TNFRSF)-interacting serine-threonine kinase 1 (RIPK1), mRNA	NM_003804	Hs.519842	AB208926
RMND1	required for meiotic nuclear division 1 homolog (S. cerevisiae) (RMND1), mRNA	NM_017909	Hs.486835	BC106065
RNASEH2C	ribonuclease H2, subunit C (RNASEH2C), mRNA	NM_032193	Hs.397010	NM_032193
RNASEH2C	ribonuclease H2, subunit C (RNASEH2C), mRNA	NM_032193	Hs.397010	NM_032193
RNF113B	ring finger protein 113B (RNF113B), mRNA	NM_178861	Hs.296045	BC017585
RNF14	ring finger protein 14 (RNF14), transcript variant 1, mRNA	NM_004290	Hs.483616	NM_004290
RNF14	ring finger protein 14 (RNF14), transcript variant 1, mRNA	NM_004290	Hs.483616	NM_004290
RNF170	ring finger protein 170 (RNF170), mRNA	NM_030954	Hs.696153	AK095625
RNF185	ring finger protein 185 (RNF185), mRNA	NM_152267	Hs.517553	NM_152267
RNF6	ring finger protein (C3H2C3 type) 6 (RNF6), transcript variant 1, mRNA	NM_005977	Hs.136885	NM_005977
ROM1	retinal outer segment membrane protein 1 (ROM1), mRNA	NM_000327	Hs.281564	NM_000327
RP11-217H1.1	implantation-associated protein (DKFZp564K142), mRNA	NM_032121	Hs.323562	BC060842
RP11-217H1.1	implantation-associated protein (DKFZp564K142), mRNA	NM_032121	Hs.323562	BC060842
RP11-262H14.4	cDNA FLJ45890 fis, clone OCBBF3022576.	AK127789	Hs.658041	AK127789
RP11-262H14.4	hypothetical protein MGC21881 (MGC21881), mRNA	NM_203448	Unknown	
RP11-262H14.4	hypothetical protein MGC21881 (MGC21881), mRNA	NM_203448	Unknown	
RP11-262H14.4	hypothetical protein MGC21881 (MGC21881), mRNA	NM_203448	Unknown	
RP11-262H14.4	hypothetical protein MGC21881 (MGC21881), mRNA	NM_203448	Unknown	
RP5-821D11.2	meiosis defective 1 (MEI1), mRNA	NM_152513	Hs.116419	BC070111
RPL10	ribosomal protein L10, mRNA (cDNA clone IMAGE:4499390), with apparent retained intron.	BC021806	Hs.657751	BC065487
RPL10	ribosomal protein L10 (RPL10), mRNA	NM_006013	Hs.534404	NM_006013
RPL23AP7	ribosomal protein L23a pseudogene 7 (RPL23AP7) on chromosome 2	NR_000029	Unknown	
RPS6KA3	ribosomal protein S6 kinase, 90kDa, polypeptide 3 (RPS6KA3), mRNA	NM_004586	Hs.445387	NM_004586
RPS6KA4	ribosomal protein S6 kinase, 90kDa, polypeptide 4 (RPS6KA4), transcript variant 1, mRNA	NM_003942	Hs.105584	AK223561
RSNL2	cDNA FLJ32705 fis, clone TESTI2000600, weakly similar to RESTIN.	AK057267	Hs.122927	AB209042
RTP4	receptor (chemosensory) transporter protein 4 (RTP4), mRNA	NM_022147	Hs.43388	BE889572
RUFY2	RUN and FYVE domain containing 2 (RUFY2), transcript variant 1, mRNA	NM_017987	Hs.653144	NM_017987
RUNDC2B	RUN domain containing 2B (RUNDC2B), mRNA	NM_001012391	Hs.658149	AK023827
S100A10	S100 calcium binding protein A10 (S100A10), mRNA	NM_002966	Hs.143873	CD388106
S100A6	S100 calcium binding protein A6 (S100A6), mRNA	NM_014624	Hs.275243	BM904612
SAC3D1	SAC3 domain containing 1 (SAC3D1), mRNA	NM_013299	Hs.23642	NM_013299
SAMD9	sterile alpha motif domain containing 9 (SAMD9), mRNA	NM_017654	Hs.65641	NM_017654
SAMD9	sterile alpha motif domain containing 9 (SAMD9), mRNA	NM_017654	Hs.65641	NM_017654

SAR1B	SAR1 gene homolog B (S. cerevisiae) (SAR1B), transcript variant 1, mRNA	NM_001033503	Hs.432984	AK056821
SAT1	spermidine/spermine N1-acetyltransferase 1 (SAT1), mRNA	NM_002970	Hs.28491	BF680536
SAT1	spermidine/spermine N1-acetyltransferase 1 (SAT1), mRNA	NM_002970	Hs.28491	BF680536
SCARB2	scavenger receptor class B, member 2 (SCARB2), mRNA	NM_005506	Hs.349656	NM_005506
SCARB2	scavenger receptor class B, member 2 (SCARB2), mRNA	NM_005506	Hs.349656	NM_005506
SCGB3A1	secretoglobin, family 3A, member 1 (SCGB3A1), mRNA	NM_052863	Hs.62492	BM921624
SCNM1	sodium channel modifier 1 (SCNM1), transcript variant 2, mRNA	NM_001002234	Unknown	
SDF2	stromal cell-derived factor 2 (SDF2), mRNA	NM_006923	Hs.514036	BQ420405
SDPR	serum deprivation response (phosphatidylserine binding protein) (SDPR), mRNA	NM_004657	Hs.26530	NM_004657
SEC22A	SEC22 vesicle trafficking protein homolog A (S. cerevisiae) (SEC22A), mRNA	NM_012430	Hs.477361	AK057587
SEC22A	SEC22 vesicle trafficking protein homolog A (S. cerevisiae) (SEC22A), mRNA	NM_012430	Hs.477361	AK057587
SEC23A	Sec23 homolog A (S. cerevisiae) (SEC23A), mRNA	NM_006364	Hs.272927	BC036649
SEC24D	cDNA FLJ20702 fis, clone KAIA2174.	AK000709	Unknown	
SECTM1	secreted and transmembrane 1 (SECTM1), mRNA	NM_003004	Hs.558009	NM_003004
SEL1L	sel-1 suppressor of lin-12-like (C. elegans) (SEL1L), mRNA	NM_005065	Hs.181300	NM_005065
SEL1L	sel-1 suppressor of lin-12-like (C. elegans) (SEL1L), mRNA	NM_005065	Hs.181300	NM_005065
SELP	selectin P (granule membrane protein 140kDa, antigen CD62) (SELP), mRNA	NM_003005	Hs.73800	NM_003005
SELT	selenoprotein T (SELT), mRNA	NM_016275	Hs.369052	NM_016275
SEPT8	septin SEPT8_v2 (KIAA0202) mRNA, partial cds, alternatively spliced.	AF440762	Hs.533017	AF179995
SERPINB2	Plasminogen activator inhibitor 2 precursor (PAI-2) (Placental plasminogen activator inhibitor) (Monocyte Arg-serpin) (Urokinase inhibitor).	ENST00000299502	Unknown	
SERPINB2	serpin peptidase inhibitor, clade B (ovalbumin), member 2 (SERPINB2), mRNA	NM_002575	Hs.594481	BC012609
SERPINB6	serpin peptidase inhibitor, clade B (ovalbumin), member 6 (SERPINB6), mRNA	NM_004568	Hs.519523	AK128637
SERPINB6	serpin peptidase inhibitor, clade B (ovalbumin), member 6 (SERPINB6), mRNA	NM_004568	Hs.519523	AK128637
SERPINB8	serpin peptidase inhibitor, clade B (ovalbumin), member 8 (SERPINB8), transcript variant 3, mRNA	NM_001031848	Hs.368077	NM_198833
SERPINB8	serpin peptidase inhibitor, clade B (ovalbumin), member 8 (SERPINB8), transcript variant 2, mRNA	NM_198833	Hs.368077	NM_198833
SERTAD1	SERTA domain containing 1 (SERTAD1), mRNA	NM_013376	Hs.269898	AK074652
SERTAD2	SERTA domain containing 2 (SERTAD2), mRNA	NM_014755	Hs.693696	NM_014755
SESN1	sestrin 1 (SESN1), mRNA	NM_014454	Hs.591336	AF033122
SETD7	SET domain containing (lysine methyltransferase) 7 (SETD7), mRNA	NM_030648	Hs.480792	NM_030648
SETD8	SET domain containing (lysine methyltransferase) 8 (SETD8), mRNA	NM_020382	Hs.700682	BC050346
SF3B5	splicing factor 3b, subunit 5, 10kDa (SF3B5), mRNA	NM_031287	Hs.110695	BM467616
SFRS2B	splicing factor, arginine/serine-rich 2B (SFRS2B), mRNA	NM_032102	Hs.648465	NM_032102
SFT2D2	SFT2 domain containing 2 (SFT2D2), mRNA	NM_199344	Hs.645435	AL035297
SGCB	sarcoglycan, beta (43kDa dystrophin-associated glycoprotein) (SGCB), mRNA	NM_000232	Hs.438953	NM_000232
SGCE	sarcoglycan, epsilon (SGCE), mRNA	NM_003919	Hs.371199	NM_001099401
SGPL1	sphingosine-1-phosphate lyase 1 (SGPL1), mRNA	NM_003901	Hs.499984	AB033078
SH3BGRL3	SH3 domain binding glutamic acid-rich protein like 3 (SH3BGRL3), mRNA	NM_031286	Hs.109051	AF466367
SIAE	sialic acid acetylesterase (SIAE), mRNA	NM_170601	Hs.10056	BC068450
SIAHBP1	fuse-binding protein-interacting repressor (SIAHBP1), transcript variant 2, mRNA	NM_014281	Hs.521924	AF114818
SIAHBP1	fuse-binding protein-interacting repressor (SIAHBP1), transcript variant 2, mRNA	NM_014281	Hs.521924	AF114818
SIRPB1	signal-regulatory protein beta 1 (SIRPB1), mRNA	NM_006065	Hs.664861	Y10376
SIRT2	sirtuin (silent mating type information regulation 2 homolog) 2 (S. cerevisiae) (SIRT2), transcript variant 1, mRNA	NM_012237	Hs.466693	AK092940
SLA/LP	soluble liver antigen/liver pancreas antigen (SLA/LP), transcript variant 1, mRNA	NM_016955	Hs.253305	BX648976
SLAIN2	SLAIN motif family, member 2, mRNA (cDNA clone IMAGE:4752398), complete cds.	BC031691	Hs.479677	NM_020846
SLC12A2	solute carrier family 12 (sodium/potassium/chloride transporters), member 2 (SLC12A2), mRNA	NM_001046	Hs.162585	NM_001046
SLC15A2	solute carrier family 15 (H+/peptide transporter), member 2 (SLC15A2), mRNA	NM_021082	Hs.518089	BC038445
SLC16A7	solute carrier family 16, member 7 (monocarboxylic acid transporter 2) (SLC16A7), mRNA	NM_004731	Hs.439643	NM_004731
SLC26A4	solute carrier family 26, member 4 (SLC26A4), mRNA	NM_000441	Hs.571246	NM_000441
SLC2A4RG	SLC2A4 regulator (SLC2A4RG), mRNA	NM_020062	Hs.435126	NM_020062
SLC35B1	solute carrier family 35, member B1 (SLC35B1), mRNA	NM_005827	Hs.154073	AK124975

SLC35D1	solute carrier family 35 (UDP-glucuronic acid/UDP-N-acetylgalactosamine dual transporter), member D1 (SLC35D1), mRNA	NM_015139	Hs.213642	D87449
SLC35E3	solute carrier family 35, member E3 (SLC35E3), mRNA	NM_018656	Hs.506011	NM_018656
SLC35E3	solute carrier family 35, member E3 (SLC35E3), mRNA	NM_018656	Hs.506011	NM_018656
SLC35F5	full-length cDNA clone CS0DL006YA16 of B cells (Ramos cell line) Cot 25-normalized of (human).	CR615423	Hs.632527	BC050096
SLC39A9	solute carrier family 39 (zinc transporter), member 9 (SLC39A9), mRNA	NM_018375	Hs.432690	AY358687
SLC41A2	solute carrier family 41, member 2 (SLC41A2), mRNA	NM_032148	Hs.577463	NM_032148
SLC4A1AP	solute carrier family 4 (anion exchanger), member 1, adaptor protein (SLC4A1AP), mRNA	NM_018158	Hs.306000	NM_018158
SLC5A4	solute carrier family 5 (low affinity glucose cotransporter), member 4 (SLC5A4), mRNA	NM_014227	Hs.130101	AJ133127
SLC9A1	solute carrier family 9 (sodium/hydrogen exchanger), member 1 (antiporter, Na+/H+, amiloride sensitive) (SLC9A1), mRNA	NM_003047	Hs.469116	NM_003047
SLC9A8	solute carrier family 9 (sodium/hydrogen exchanger), member 8 (SLC9A8), mRNA	NM_015266	Hs.444202	NM_015266
SMAD3	mad protein homolog (hMAD-3) mRNA, complete cds.	U68019	Hs.36915	NM_005902
SMAP1L	stromal membrane-associated protein 1-like (SMAP1L), mRNA	NM_022733	Hs.15200	BC021133
SMTN	smoothelin (SMTN), transcript variant 2, mRNA	NM_134269	Hs.149098	AB209643
SNIP1	Smad nuclear interacting protein 1 (SNIP1), mRNA	NM_024700	Hs.471951	NM_024700
SNX1	sorting nexin 1 (SNX1), transcript variant 1, mRNA	NM_003099	Hs.188634	NM_003099
SNX12	Sorting nexin-12.	ENST00000374274	Unknown	
SNX17	sorting nexin 17 (SNX17), mRNA	NM_014748	Hs.278569	NM_014748
SNX19	sorting nexin 19 (SNX19), mRNA	NM_014758	Hs.444024	NM_014758
SNX19	sorting nexin 19 (SNX19), mRNA	NM_014758	Hs.444024	NM_014758
SNX3	sorting nexin 3 (SNX3), mRNA	NM_003795	Hs.12102	BC016863
SNX3	sorting nexin 3 (SNX3), mRNA	NM_003795	Hs.12102	BC016863
SNX7	sorting nexin 7 (SNX7), transcript variant 1, mRNA	NM_015976	Hs.197015	NM_015976
SOAT1	sterol O-acyltransferase (acyl-Coenzyme A: cholesterol acyltransferase) 1 (SOAT1), transcript variant 688113, mRNA	NM_003101	Hs.496383	L21934
SP100	SP100 nuclear antigen (SP100), mRNA	NM_003113	Hs.369056	NM_001080391
SP110	SP110 nuclear body protein (SP110), transcript variant b, mRNA	NM_004510	Hs.145150	AK128274
SPAG1	sperm associated antigen 1 (SPAG1), transcript variant 1, mRNA	NM_003114	Hs.591866	NM_003114
SPFH2	SPFH domain family, member 2 (SPFH2), transcript variant 2, mRNA	NM_001003790	Hs.696282	NM_007175
SPHK1	sphingosine kinase 1 (SPHK1), transcript variant 1, mRNA	NM_021972	Hs.68061	AK095578
SPIRE1	spire homolog 1 (Drosophila) (SPIRE1), mRNA	NM_020148	Hs.515283	AB032961
SPOCD1	cDNA FLJ39908 fis, clone SPLEN2017620.	AK097227	Hs.62604	AK131447
SPOCD1	SPOC domain containing 1 (SPOCD1), mRNA	NM_144569	Hs.62604	AK131447
SPOCK1	sparc/osteonectin, cwcv and kazal-like domains proteoglycan (testican) 1 (SPOCK1), mRNA	NM_004598	Hs.654695	NM_004598
SPR	sepiapterin reductase (7,8-dihydrobiopterin:NADP+ oxidoreductase) (SPR), mRNA	NM_003124	Hs.301540	AK222942
SQRDL	sulfide quinone reductase-like (yeast) (SQRDL), mRNA	NM_021199	Hs.511251	AK130140
SRI	sorcin (SRI), transcript variant 1, mRNA	NM_003130	Hs.489040	NM_003130
SRP14P1	signal recognition particle 14kDa (homologous Alu RNA binding protein) pseudogene 1 (SRP14P1) on chromosome 12	NR_003273	Unknown	
SRP54	signal recognition particle 54kDa (SRP54), mRNA	NM_003136	Hs.167535	BC000652
SRPK2	SFRS protein kinase 2 (SRPK2), transcript variant 2, mRNA	NM_182691	Hs.285197	U88666
SRPX	sushi-repeat-containing protein, X-linked (SRPX), mRNA	NM_006307	Hs.15154	BC020684
SSFA2	sperm specific antigen 2 (SSFA2), mRNA	NM_006751	Hs.591602	BX648182
ST3GAL4	cDNA FLJ11867 fis, clone HEMBA1006976, weakly similar to H.sapiens mRNA for Gal-beta(1-3/1-4)GlcNAc alpha-2.3-sialyltransferase.	AK021929	Hs.591947	AK128605
ST7	suppression of tumorigenicity 7 (ST7), transcript variant a, mRNA	NM_018412	Hs.368131	BC075855
STAM2	signal transducing adaptor molecule (SH3 domain and ITAM motif) 2 (STAM2), mRNA	NM_005843	Hs.17200	NM_005843
STARD13	START domain containing 13 (STARD13), transcript variant alpha, mRNA	NM_178006	Hs.507704	BX647695
STARD4	START domain containing 4, sterol regulated (STARD4), mRNA	NM_139164	Hs.93842	AL832599
STARD9	StAR-related lipid transfer protein 9 (StARD9) (START domain- containing protein 9) (Fragment).	ENST00000290607	Unknown	
STAT1	signal transducer and activator of transcription 1, 91kDa (STAT1), transcript variant alpha, mRNA	NM_007315	Hs.699271	NM_007315
STAT6	signal transducer and activator of transcription 6, interleukin-4 induced (STAT6), mRNA	NM_003153	Hs.524518	NM_003153
STK10	serine/threonine kinase 10 (STK10), mRNA	NM_005990	Hs.519756	NM_005990
STK17A	serine/threonine kinase 17a (apoptosis-inducing) (STK17A), mRNA	NM_004760	Hs.699353	NM_004760

STK17A	serine/threonine kinase 17a (apoptosis-inducing) (STK17A), mRNA	NM_004760	Hs.699353	NM_004760
STOML1	stomatin (EPB72)-like 1 (STOML1), mRNA	NM_004809	Hs.194816	AK091531
STS	steroid sulfatase (microsomal), arylsulfatase C, isozyme S (STS), mRNA	NM_000351	Hs.522578	M16505
STT3A	STT3, subunit of the oligosaccharyltransferase complex, homolog A (S. cerevisiae) (STT3A), mRNA	NM_152713	Hs.504237	BX649102
STX18	syntaxin 18 (STX18), mRNA	NM_016930	Hs.584913	BX647083
STX7	syntaxin 7 (STX7), mRNA	NM_003569	Hs.593148	AJ420529
SUMF1	sulfatase modifying factor 1 (SUMF1), mRNA	NM_182760	Hs.588682	AY358092
SURF4	surfeit 4 (SURF4), mRNA	NM_033161	Hs.696048	NM_033161
SUSD1	sushi domain containing 1 (SUSD1), mRNA	NM_022486	Hs.494827	NM_022486
SYT11	synaptotagmin XI (SYT11), mRNA	NM_152280	Hs.32984	NM_152280
SYT11	synaptotagmin XI (SYT11), mRNA	NM_152280	Hs.32984	NM_152280
SYTL4	synaptotagmin-like 4 (granuphilin-a) (SYTL4), mRNA	NM_080737	Hs.592224	AL832596
TACC1	transforming, acidic coiled-coil containing protein 1 (TACC1), mRNA	NM_006283	Hs.279245	CR933618
TAOK3	TAO kinase 3 (TAOK3), mRNA	NM_016281	Hs.644420	NM_016281
TAOK3	TAO kinase 3 (TAOK3), mRNA	NM_016281	Hs.644420	NM_016281
TATDN2	TatD DNase domain containing 2 (TATDN2), mRNA	NM_014760	Hs.475401	D86972
TBC1D12	mRNA for KIAA0608 protein, partial cds.	AB011180	Hs.500598	NM_015188
TBRG1	transforming growth factor beta regulator 1 (TBRG1), mRNA	NM_032811	Hs.436410	AK074140
TCEAL7	transcription elongation factor A (SII)-like 7 (TCEAL7), mRNA	NM_152278	Hs.21861	BF219487
TCEAL7	transcription elongation factor A (SII)-like 7 (TCEAL7), mRNA	NM_152278	Hs.21861	BF219487
TCP11L2	t-complex 11 (mouse)-like 2 (TCP11L2), mRNA	NM_152772	Hs.696047	AF306858
TCTE3	t-complex-associated-testis-expressed 3 (TCTE3), mRNA	NM_174910	Hs.584808	BC063455
TEKT4	tektin 4 (TEKT4), mRNA	NM_144705	Hs.631517	AK097438
TERF2	telomeric repeat binding factor 2 (TERF2), mRNA	NM_005652	Hs.63335	NM_005652
TEX261	testis expressed sequence 261 (TEX261), mRNA	NM_144582	Hs.516087	NM_144582
TEX261	testis expressed sequence 261 (TEX261), mRNA	NM_144582	Hs.516087	NM_144582
TEX264	testis expressed sequence 264 (TEX264), mRNA	NM_015926	Hs.517864	AL832608
TFPI	tissue factor pathway inhibitor (lipoprotein-associated coagulation inhibitor) (TFPI), transcript variant 2, mRNA	NM_001032281	Hs.516578	NM_006287
TFPI	tissue factor pathway inhibitor (lipoprotein-associated coagulation inhibitor) (TFPI), transcript variant 1, mRNA	NM_006287	Hs.516578	NM_006287
TFPT	TCF3 (E2A) fusion partner (in childhood Leukemia) (TFPT), mRNA	NM_013342	Hs.590939	BM810210
TGFBR2	transforming growth factor, beta receptor II (70/80kDa) (TGFBR2), transcript variant 1, mRNA	NM_001024847	Hs.82028	BX648313
TGM1	transglutaminase 1 (K polypeptide epidermal type I, protein-glutamine-gamma-glutamyltransferase) (TGM1), mRNA	NM_000359	Hs.508950	NM_000359
TGOLN2	trans-golgi network protein 2 (TGOLN2), mRNA	NM_006464	Hs.593382	NM_006464
TH1L	TH1-like (Drosophila) (TH1L), transcript variant 1, mRNA	NM_198976	Hs.517148	BX647417
THBS1	thrombospondin 1 (THBS1), mRNA	NM_003246	Hs.164226	NM_003246
THBS1	thrombospondin 1 (THBS1), mRNA	NM_003246	Hs.164226	NM_003246
THBS1	thrombospondin 1 (THBS1), mRNA	NM_003246	Hs.164226	NM_003246
THBS1	thrombospondin 1 (THBS1), mRNA	NM_003246	Hs.164226	NM_003246
THBS1	thrombospondin 1 (THBS1), mRNA	NM_003246	Hs.164226	NM_003246
THBS1	thrombospondin 1 (THBS1), mRNA	NM_003246	Hs.164226	NM_003246
THC2463424	AA348270 EST54713 Hippocampus I cDNA 3' end similar to EST containing Alu repeat, mRNA sequence	THC2463424	Unknown	
THC2493621	AW241739 xn74d12.x1 Soares_NFL_T_GBC_S1 cDNA clone IMAGE:2700215 3', mRNA sequence	THC2493621	Unknown	
THC2500665	BC008589 SENP5 protein {Homo sapiens} (exp=-1; wgp=0; cg=0), partial (10%)	THC2500665	Unknown	
THC2506377	HSU02032 ribosomal protein L23a {Homo sapiens} (exp=-1; wgp=0; cg=0), partial (92%)	THC2506377	Unknown	
THC2508817	Q7PPA2_ANOGA (Q7PPA2) ENSANGP00000004457 (Fragment), partial (3%)	THC2508817	Unknown	
THC2516687	AI401097 th22c12.x1 NCI_CGAP_Pr28 cDNA clone IMAGE:2119030 3', mRNA sequence	THC2516687	Unknown	
THC2522342	Q80YT0_MOUSE (Q80YT0) Whdc1 protein, partial (4%)	THC2522342	Unknown	
THC2525408	AV738929 AV738929 CB cDNA clone CBCCMG04 5', mRNA sequence	THC2525408	Unknown	
THC2525955	Q3T035_BOVIN (Q3T035) Actin related protein 2/3 complex, subunit 3, 21kDa, partial (53%)	THC2525955	Unknown	
THC2529957	THC2529957	THC2529957	Unknown	

THC2531867	AW505189 UI-HF-BN0-alt-a-09-0-UI.r1 NIH_MGC_50 cDNA clone IMAGE:3080728 5', mRNA sequence	THC2531867	Unknown	
THC2532114	BU735310 UI-E-DW0-agk-p-10-0-UI.s1 UI-E-DW0 cDNA clone UI-E-DW0-agk-p-10-0-UI 3', mRNA sequence	THC2532114	Unknown	
THC2535223	BC000698 keratin 18 {Homo sapiens} (exp=-1; wgp=0; cg=0), partial (19%)	THC2535223	Unknown	
THC2536108	ALU1_HUMAN (P39188) Alu subfamily J sequence contamination warning entry, partial (32%)	THC2536108	Unknown	
THC2538568	1AK6 Destrin, Nmr, Minimized Average Structure. {Sus scrofa} (exp=-1; wgp=0; cg=0), partial (30%)	THC2538568	Unknown	
THC2538882	Q5VT28_HUMAN (Q5VT28) Family with sequence similarity 27, member B (Family with sequence similarity 27, member A) (Family with sequence similarity 27, member C), partial (85%)	THC2538882	Unknown	
THC2540361	ALU5_HUMAN (P39192) Alu subfamily SC sequence contamination warning entry, partial (18%)	THC2540361	Unknown	
THC2545097	IBP7_HUMAN (Q16270) Insulin-like growth factor-binding protein 7 precursor (IGFBP-7) (IBP-7) (IGF-binding protein 7) (MAC25 protein) (Prostacyclin-stimulating factor) (PGI2-stimulating factor) (IGFBP-rP1), partial (58%)	THC2545097	Unknown	
THC2545454	THC2545454	THC2545454	Unknown	
THC2546004	THC2546004	THC2546004	Unknown	
THC2548567	THC2548567	THC2548567	Unknown	
THC2551944	XM_814371 protein kinase {Trypanosoma cruzi strain CL Brener} (exp=-1; wgp=0; cg=0), partial (5%)	THC2551944	Unknown	
THC2557134	Q48AJ7_COLP3 (Q48AJ7) Cold-shock DNA-binding domain family protein, partial (12%)	THC2557134	Unknown	
THC2558878	THC2558878	THC2558878	Unknown	
THC2559002	THC2559002	THC2559002	Unknown	
THC2563307	THC2563307	THC2563307	Unknown	
THC2565151	Q5TZZ9_HUMAN (Q5TZZ9) Annexin A1 (ANXA1 protein), partial (21%)	THC2565151	Unknown	
THC2574606	MUSVIM01 vimentin protein {Mus musculus} (exp=-1; wgp=0; cg=0), partial (16%)	THC2574606	Unknown	
THC2577186	AF450266 NUCB2 protein {Homo sapiens} (exp=-1; wgp=0; cg=0), partial (49%)	THC2577186	Unknown	
THC2582291	ALU7_HUMAN (P39194) Alu subfamily SQ sequence contamination warning entry, partial (13%)	THC2582291	Unknown	
THC2585854	1T5A_A Chain A, Human Pyruvate Kinase M2. {Homo sapiens} (exp=-1; wgp=0; cg=0), partial (11%)	THC2585854	Unknown	
THC2586905	BC051814 YWHAZ protein {Homo sapiens} (exp=-1; wgp=0; cg=0), partial (60%)	THC2586905	Unknown	
THC2596442	1W7B_A Chain A, Annexin A2: Does It Induce Membrane Aggregation By A New Multimeric State Of The Protein. {Homo sapiens} (exp=-1; wgp=0; cg=0), partial (24%)	THC2596442	Unknown	
THC2603473	BX338933 BX338933 PLACENTA COT 25-NORMALIZED cDNA clone CS0DI065YH21 3-PRIME, mRNA sequence	THC2603473	Unknown	
THC2608967	THC2608967	THC2608967	Unknown	
THC2614203	Q7KZ66_HUMAN (Q7KZ66) NF2 protein (Fragment), partial (36%)	THC2614203	Unknown	
THC2620182	THC2620182	THC2620182	Unknown	
THC2623335	THC2623335	THC2623335	Unknown	
THC2627335	2F2S_A Chain A, Human Mitochondrial Acetoacetyl-Coa Thiolase. {Homo sapiens} (exp=-1; wgp=0; cg=0), partial (15%)	THC2627335	Unknown	
THC2631475	BC011812 DNAJB12 protein {Homo sapiens} (exp=-1; wgp=0; cg=0), partial (42%)	THC2631475	Unknown	
THC2637492	ACOX3_HUMAN (O15254) Acyl-coenzyme A oxidase 3, peroxisomal (Pristanoyl-CoA oxidase) (Branched-chain acyl-CoA oxidase) (BRCACox) , partial (4%)	THC2637492	Unknown	
THC2640099	THC2640099	THC2640099	Unknown	
THC2643750	Q9NQR4_HUMAN (Q9NQR4) Nit protein 2 (Nitrilase family, member 2) (CUA002), partial (77%)	THC2643750	Unknown	
THC2648791	THC2648791	THC2648791	Unknown	
THC2649152	THC2649152	THC2649152	Unknown	
THC2650022	THC2650022	THC2650022	Unknown	
THC2650074	THC2650074	THC2650074	Unknown	
THC2650457	ALU6_HUMAN (P39193) Alu subfamily SP sequence contamination warning entry, partial (12%)	THC2650457	Unknown	
THC2654000	THC2654000	THC2654000	Unknown	
THC2656077	THC2656077	THC2656077	Unknown	
THC2656875	THC2656875	THC2656875	Unknown	
THC2657989	ALU2_HUMAN (P39189) Alu subfamily SB sequence contamination warning entry, partial (27%)	THC2657989	Unknown	
THC2658069	THC2658069	THC2658069	Unknown	
THC2659814	Q495E5_HUMAN (Q495E5) Nanos homolog 3 (Drosophila), partial (10%)	THC2659814	Unknown	
THC2660784	Q3AUE7_CHLCH (Q3AUE7) Glucosamine-fructose-6-phosphate aminotransferase, isomerising , partial (4%)	THC2660784	Unknown	
THC2669126	THC2669126	THC2669126	Unknown	

THC2680571	THC2680571	THC2680571	Unknown	
THC2688123	Q1WZC5_9FIRM (Q1WZC5) S-adenosylmethionine:tRNA-ribosyltransferase- isomerase, partial (5%)	THC2688123	Unknown	
THC2690931	AF235005 suppression of tumorigenicity 16 protein {Homo sapiens} (exp=-1; wgp=0; cg=0), partial (13%)	THC2690931	Unknown	
THC2694242	THC2694242	THC2694242	Unknown	
THC2697511	THC2697511	THC2697511	Unknown	
THC2705989	AL356953 leucine-rich repeat-containing G protein-coupled receptor 6 {Homo sapiens} (exp=0; wgp=1; cg=0), partial (4%)	THC2705989	Unknown	
THC2717828	THC2717828	THC2717828	Unknown	
THC2721124	Q9BTU8_HUMAN (Q9BTU8) Lysosomal-associated protein transmembrane 4 alpha, partial (38%)	THC2721124	Unknown	
THC2726026	Q3X600_9ACTN (Q3X600) Adenine deaminase , partial (4%)	THC2726026	Unknown	
THC2736113	E41LA_HUMAN (Q9HCS5) Band 4.1-like protein 4A (NBL4 protein), partial (31%)	THC2736113	Unknown	
THC2740030	THC2740030	THC2740030	Unknown	
THC2750590	THC2750590	THC2750590	Unknown	
THC2788875	CA843452 ir51a07.x1 HR85 islet cDNA clone IMAGE:6548462 3' similar to SW:UAP1_HUMAN Q16222 UDP-N-ACETYLGLUCOSAMINE PYROPHOSPHORYLASE ;, mRNA sequence	THC2788875	Unknown	
THSD1	thrombospondin, type I, domain containing 1 (THSD1), transcript variant 1, mRNA	NM_018676	Hs.325667	AK096289
THSD4	thrombospondin, type I, domain containing 4 (THSD4), mRNA	NM_024817	Hs.387057	NM_024817
TICAM1	toll-like receptor adaptor molecule 1 (TICAM1), transcript variant 2, mRNA	NM_182919	Hs.29344	BC035331
TICAM2	toll-like receptor adaptor molecule 2 (TICAM2), mRNA	NM_021649	Hs.696163	NM_181836
TIRAP	toll-interleukin 1 receptor (TIR) domain containing adaptor protein (TIRAP), transcript variant 2, mRNA	NM_148910	Hs.537126	NM_001039661
TITF1	thyroid transcription factor 1 (TITF1), transcript variant 2, mRNA	NM_003317	Hs.700584	NM_003317
TK2	thymidine kinase 2, mitochondrial (TK2), mRNA	NM_004614	Hs.512619	NM_004614
TK2	thymidine kinase 2, mitochondrial (TK2), mRNA	NM_004614	Hs.512619	NM_004614
TLN1	talin 1 (TLN1), mRNA	NM_006289	Hs.471014	AB028950
TM2D3	TM2 domain containing 3 (TM2D3), transcript variant 1, mRNA	NM_078474	Hs.288912	BX641093
TM4SF1	transmembrane 4 L six family member 1 (TM4SF1), mRNA	NM_014220	Hs.696050	AL832780
TM7SF3	transmembrane 7 superfamily member 3 (TM7SF3), mRNA	NM_016551	Hs.438641	AK091115
TM7SF3	transmembrane 7 superfamily member 3 (TM7SF3), mRNA	NM_016551	Hs.438641	AK091115
TMBIM1	transmembrane BAX inhibitor motif containing 1 (TMBIM1), mRNA	NM_022152	Hs.591605	AK090618
TMBIM4	transmembrane BAX inhibitor motif containing 4 (TMBIM4), mRNA	NM_016056	Hs.505934	AL117550
TMCO2	transmembrane and coiled-coil domains 2 (TMCO2), mRNA	NM_001008740	Hs.406265	BI829449
TMED5	transmembrane emp24 protein transport domain containing 5 (TMED5), mRNA	NM_016040	Hs.482873	CR936808
TMED7	transmembrane emp24 protein transport domain containing 7 (TMED7), mRNA	NM_181836	Hs.696163	NM_181836
TMEM109	transmembrane protein 109 (TMEM109), mRNA	NM_024092	Hs.13662	BC001309
TMEM111	transmembrane protein 111 (TMEM111), mRNA	NM_018447	Hs.475392	AK022202
TMEM111	Transmembrane protein 111.	ENST00000383810	Unknown	
TMEM111	transmembrane protein 111 (TMEM111), mRNA	NM_018447	Hs.475392	AK022202
TMEM112	transmembrane protein 112 (TMEM112), mRNA	NM_022773	Hs.71912	AK127296
TMEM113	transmembrane protein 113 (TMEM113), mRNA	NM_025222	Hs.194110	AK123860
TMEM136	transmembrane protein 136 (TMEM136), mRNA	NM_174926	Hs.700698	AK128040
TMEM147	transmembrane protein 147 (TMEM147), mRNA	NM_032635	Hs.9234	CR603882
TMEM163	transmembrane protein 163 (TMEM163), mRNA	NM_030923	Hs.369471	AL833765
TMEM166	transmembrane protein 166 (TMEM166), mRNA	NM_032181	Hs.302346	BC063016
TMEM19	transmembrane protein 19 (TMEM19), mRNA	NM_018279	Hs.688627	NM_018279
TMEM19	transmembrane protein 19 (TMEM19), mRNA	NM_018279	Hs.688627	NM_018279
TMEM22	transmembrane protein 22 (TMEM22), mRNA	NM_025246	Hs.655019	BC050423
TMEM22	transmembrane protein 22 (TMEM22), mRNA	NM_025246	Hs.655019	BC050423
TMEM24	transmembrane protein 24 (TMEM24), mRNA	NM_014807	Hs.587176	NM_014807
TMEM30A	transmembrane protein 30A (TMEM30A), mRNA	NM_018247	Hs.108530	NM_018247
TMEM30A	transmembrane protein 30A (TMEM30A), mRNA	NM_018247	Hs.108530	NM_018247
TMEM41A	transmembrane protein 41A (TMEM41A), mRNA	NM_080652	Hs.634586	BX647928

TMEM43	transmembrane protein 43 (TMEM43), mRNA	NM_024334	Hs.517817	AK074073
TMEM50B	transmembrane protein 50B (TMEM50B), mRNA	NM_006134	Hs.433668	NM_006134
TMEM62	transmembrane protein 62 (TMEM62), mRNA	NM_024956	Hs.511175	NM_024956
TMEM70	transmembrane protein 70 (TMEM70), transcript variant 2, mRNA	NM_001040613	Hs.106650	BC002748
TMEM87B	transmembrane protein 87B (TMEM87B), mRNA	NM_032824	Hs.656298	AK027587
TMEM87B	transmembrane protein 87B (TMEM87B), mRNA	NM_032824	Hs.656298	AK027587
TMEM9B	TMEM9 domain family, member B (TMEM9B), mRNA	NM_020644	Hs.501853	BC040124
TMLHE	trimethyllysine hydroxylase, epsilon (TMLHE), mRNA	NM_018196	Hs.133321	AK001589
TMOD4	tropomodulin 4 (muscle) (TMOD4), mRNA	NM_013353	Hs.250763	AF177173
TMPIT	transmembrane protein induced by tumor necrosis factor alpha (TMPIT), mRNA	NM_031925	Hs.488835	BM805119
TMSB10	thymosin, beta 10 (TMSB10), mRNA	NM_021103	Hs.446574	BQ276852
TncRNA	Human clone 137308 mRNA, partial cds.	U60873	Hs.648467	EF177379
TNFRSF10B	tumor necrosis factor receptor superfamily, member 10b (TNFRSF10B), transcript variant 1, mRNA	NM_003842	Hs.521456	NM_003842
TNFRSF14	tumor necrosis factor receptor superfamily, member 14 (herpesvirus entry mediator) (TNFRSF14), mRNA	NM_003820	Hs.512898	AB208808
TNFRSF1A	tumor necrosis factor receptor superfamily, member 1A (TNFRSF1A), mRNA	NM_001065	Hs.279594	NM_001065
TNFRSF1A	tumor necrosis factor receptor superfamily, member 1A (TNFRSF1A), mRNA	NM_001065	Hs.279594	NM_001065
TNFRSF1A	tumor necrosis factor receptor superfamily, member 1A (TNFRSF1A), mRNA	NM_001065	Hs.279594	NM_001065
TNFRSF1A	tumor necrosis factor receptor superfamily, member 1A (TNFRSF1A), mRNA	NM_001065	Hs.279594	NM_001065
TNFRSF1A	tumor necrosis factor receptor superfamily, member 1A (TNFRSF1A), mRNA	NM_001065	Hs.279594	NM_001065
TNFRSF1A	tumor necrosis factor receptor superfamily, member 1A (TNFRSF1A), mRNA	NM_001065	Hs.279594	NM_001065
TNFRSF1A	tumor necrosis factor receptor superfamily, member 1A (TNFRSF1A), mRNA	NM_001065	Hs.279594	NM_001065
TNFRSF1A	tumor necrosis factor receptor superfamily, member 1A (TNFRSF1A), mRNA	NM_001065	Hs.279594	NM_001065
TNFRSF1A	tumor necrosis factor receptor superfamily, member 1A (TNFRSF1A), mRNA	NM_001065	Hs.279594	NM_001065
TNFRSF1A	tumor necrosis factor receptor superfamily, member 1A (TNFRSF1A), mRNA	NM_001065	Hs.279594	NM_001065
TNFSF4	tumor necrosis factor (ligand) superfamily, member 4 (tax-transcriptionally activated glycoprotein 1, 34kDa) (TNFSF4), mRNA	NM_003326	Hs.181097	NM_003326
TNIP1	TNFAIP3 interacting protein 1 (TNIP1), mRNA	NM_006058	Hs.543850	NM_006058
TNIP1	TNFAIP3 interacting protein 1 (TNIP1), mRNA	NM_006058	Hs.543850	NM_006058
TNIP3	TNFAIP3 interacting protein 3 (TNIP3), mRNA	NM_024873	Hs.208206	AF277289
TNS1	tensin 1 (TNS1), mRNA	NM_022648	Unknown	
TNS1	tensin 1 (TNS1), mRNA	NM_022648	Unknown	
TNS1	tensin 1 (TNS1), mRNA	NM_022648	Unknown	
TOMM34	translocase of outer mitochondrial membrane 34 (TOMM34), nuclear gene encoding mitochondrial protein, mRNA	NM_006809	Hs.517066	AB085681
TOR1AIP2	torsin A interacting protein 2 (TOR1AIP2), mRNA	NM_145034	Hs.693681	BC094724
TP53AP1	TP53 activated protein 1, mRNA (cDNA clone MGC:75059 IMAGE:5736876), complete cds.	BC061927	Unknown	
TP53I3	tumor protein p53 inducible protein 3 (TP53I3), transcript variant 1, mRNA	NM_004881	Hs.50649	AK223382
TPK1	thiamin pyrophosphokinase 1 (TPK1), transcript variant 1, mRNA	NM_022445	Hs.660232	NM_022445
TPM4	tropomyosin 4 (TPM4), mRNA	NM_003290	Hs.631618	AK056565
TPM4	tropomyosin 4 (TPM4), mRNA	NM_003290	Hs.631618	AK056565
TPP1	tripeptidyl peptidase I (TPP1), mRNA	NM_000391	Hs.523454	NM_000391
TRADD	TNFRSF1A-associated via death domain (TRADD), mRNA	NM_003789	Hs.460996	AK090673
TRAF6	TNF receptor-associated factor 6 (TRAF6), transcript variant 1, mRNA	NM_145803	Hs.591983	NM_145803
TRAPPC5	trafficking protein particle complex 5 (TRAPPC5), transcript variant 1, mRNA	NM_174894	Hs.432413	AF461155
TRAPPC5	trafficking protein particle complex 5 (TRAPPC5), transcript variant 1, mRNA	NM_174894	Hs.432413	AF461155
TRIB3	tribbles homolog 3 (Drosophila) (TRIB3), mRNA	NM_021158	Hs.516826	AY247738
TRIM35	tripartite motif-containing 35 (TRIM35), transcript variant 2, mRNA	NM_171982	Hs.104223	NM_171982
TRIM44	tripartite motif-containing 44 (TRIM44), mRNA	NM_017583	Hs.591987	NM_017583
TRIM5	tripartite motif-containing 5 (TRIM5), transcript variant gamma, mRNA	NM_033092	Hs.370515	BX647152
TRIM8	tripartite motif-containing 8 (TRIM8), mRNA	NM_030912	Hs.336810	NM_030912
TRIO	cDNA FLJ16543 fis, clone OCBBF3002654, highly similar to Triple functional domain protein.	AK131423	Hs.130031	NM_007118
TRPM4	transient receptor potential cation channel, subfamily M, member 4 (TRPM4), mRNA	NM_017636	Hs.467101	NM_017636
TSC22D4	TSC22 domain family, member 4 (TSC22D4), mRNA	NM_030935	Hs.469798	NM_030935

TSGA14	testis specific, 14 (TSGA14), mRNA	NM_018718	Hs.368315	AF429308
TSPAN9	tetraspanin 9 (TSPAN9), mRNA	NM_006675	Hs.504517	AK024470
TSPYL5	TSPY-like 5 (TSPYL5), mRNA	NM_033512	Hs.173094	NM_033512
TSSC4	tumor suppressing subtransferable candidate 4 (TSSC4), mRNA	NM_005706	Hs.523424	AK095568
TTC16	tetratricopeptide repeat domain 16 (TTC16), mRNA	NM_144965	Hs.642748	AK057342
TTC26	tetratricopeptide repeat domain 26 (TTC26), mRNA	NM_024926	Hs.659165	BC034466
TTC33	tetratricopeptide repeat domain 33 (TTC33), mRNA	NM_012382	Hs.348915	NM_012382
TTL	tubulin tyrosine ligase (TTL), mRNA	NM_153712	Hs.358997	BX649144
TTLL11	tubulin tyrosine ligase-like family, member 11 (TTLL11), mRNA	NM_194252	Hs.656140	NM_194252
TUSC1	tumor suppressor candidate 1 (TUSC1), mRNA	NM_001004125	Hs.26268	NM_001004125
TUSC1	tumor suppressor candidate 1 (TUSC1), mRNA	NM_001004125	Hs.26268	NM_001004125
TWSG1	twisted gastrulation homolog 1 (Drosophila) (TWSG1), mRNA	NM_020648	Hs.514685	NM_020648
TWSG1	twisted gastrulation homolog 1 (Drosophila) (TWSG1), mRNA	NM_020648	Hs.514685	NM_020648
TXNDC10	thioredoxin domain containing 10 (TXNDC10), mRNA	NM_019022	Hs.440534	BX647846
TXNDC11	thioredoxin domain containing 11 (TXNDC11), mRNA	NM_015914	Hs.313847	BC002856
TXNDC9	thioredoxin domain containing 9 (TXNDC9), mRNA	NM_005783	Hs.536122	BC070183
TXNL4B	thioredoxin-like 4B (TXNL4B), mRNA	NM_017853	Hs.134406	AK000518
TXNL4B	thioredoxin-like 4B (TXNL4B), mRNA	NM_017853	Hs.134406	AK000518
TYK2	tyrosine kinase 2 (TYK2), mRNA	NM_003331	Hs.75516	NM_003331
UBA52	ubiquitin A-52 residue ribosomal protein fusion product 1 (UBA52), transcript variant 1, mRNA	NM_001033930	Hs.5308	NM_001033930
UBA52	ubiquitin A-52 residue ribosomal protein fusion product 1 (UBA52), transcript variant 1, mRNA	NM_001033930	Hs.5308	NM_001033930
UBA52	ubiquitin A-52 residue ribosomal protein fusion product 1 (UBA52), transcript variant 1, mRNA	NM_001033930	Hs.5308	NM_001033930
UBE2B	ubiquitin-conjugating enzyme E2B (RAD6 homolog) (UBE2B), mRNA	NM_003337	Hs.695927	NM_003337
UBE2D4	ubiquitin-conjugating enzyme E2D 4 (putative) (UBE2D4), mRNA	NM_015983	Hs.19196	BM543814
UBE2J1	ubiquitin-conjugating enzyme E2, J1 (UBC6 homolog, yeast) (UBE2J1), mRNA	NM_016021	Hs.163776	NM_016021
UBE3B	ubiquitin protein ligase E3B (UBE3B), transcript variant 3, mRNA	NM_183415	Hs.374067	NM_130466
UBQLN1	ubiquilin 1 (UBQLN1), transcript variant 2, mRNA	NM_053067	Hs.9589	AB209468
UBR1	ubiquitin protein ligase E3 component n-recognin 1 (UBR1), mRNA	NM_174916	Hs.591121	NM_174916
UEVLD	UEV and lactate/malate dehyrogenase domains (UEVLD), transcript variant 1, mRNA	NM_001040697	Hs.407991	NM_001040697
UEVLD	UEV and lactate/malate dehyrogenase domains (UEVLD), transcript variant 2, mRNA	NM_018314	Hs.407991	NM_001040697
UNC84A	unc-84 homolog A (C. elegans) (UNC84A), mRNA	NM_025154	Hs.438072	AB018353
UNQ1887	signal peptide peptidase 3 (SPPL3), mRNA	NM_139015	Hs.507087	NM_139015
UNQ6490	clone DNA147309 YPLR6490 (UNQ6490) mRNA, complete cds.	AY358209	Hs.526966	AY358209
USF2	clone TCCCIA00046 mRNA sequence.	AY007087	Hs.454534	NM_003367
USP30	ubiquitin specific peptidase 30 (USP30), mRNA	NM_032663	Hs.486434	AL834278
USP38	ubiquitin specific peptidase 38 (USP38), mRNA	NM_032557	Hs.480848	NM_032557
USP40	ubiquitin specific peptidase 40 (USP40), mRNA	NM_018218	Hs.96513	NM_018218
USP40	ubiquitin specific peptidase 40 (USP40), mRNA	NM_018218	Hs.96513	NM_018218
VAMP3	vesicle-associated membrane protein 3 (cellubrevin) (VAMP3), mRNA	NM_004781	Hs.66708	NM_004781
VAMP4	vesicle-associated membrane protein 4 (VAMP4), mRNA	NM_003762	Hs.6651	AK123624
VAMP5	vesicle-associated membrane protein 5 (myobrevin) (VAMP5), mRNA	NM_006634	Hs.172684	BG535701
VEGFC	vascular endothelial growth factor C (VEGFC), mRNA	NM_005429	Hs.435215	NM_005429
VHLL	von Hippel-Lindau tumor suppressor-like (VHLL), mRNA	NM_001004319	Hs.532378	AY494836
VIM	vimentin (VIM), mRNA	NM_003380	Hs.642813	AK093924
VPS16	vacuolar protein sorting 16 homolog (S. cerevisiae) (VPS16), transcript variant 2, mRNA	NM_080414	Hs.654871	NM_022575
VPS29	vacuolar protein sorting 29 homolog (S. cerevisiae) (VPS29), transcript variant 1, mRNA	NM_016226	Hs.632735	AL832866
VPS37C	vacuolar protein sorting 37 homolog C (S. cerevisiae) (VPS37C), mRNA	NM_017966	Hs.523715	AL834261
W60781	W60781 zd26f05.r1 Soares_fetal_heart_NbHH19W cDNA clone IMAGE:341793 5' similar to gb:J02874 FATTY ACID-BINDING PROTEIN, ADIPOCYTE (HUMAN);, mRNA sequence	W60781	Hs.391561	CB999901
WBSCR28	Williams-Beuren syndrome chromosome region 28 (WBSCR28), mRNA	NM_182504	Hs.647026	AY372054
WDR13	WD repeat domain 13 (WDR13), mRNA	NM_017883	Hs.521973	L08237
WDR32	WD repeat domain 32 (WDR32), mRNA	NM_024345	Hs.118394	NM_024345
WDR34	WD repeat domain 34 (WDR34), mRNA	NM_052844	Hs.495240	BM479044

WDR41	WD repeat domain 41 (WDR41), mRNA	NM_018268	Hs.482573	AL834138
WDR42A	WD repeat domain 42A, mRNA (cDNA clone IMAGE:5743945), complete cds.	BC111063	Hs.632447	NM_015726
WDR69	WD repeat domain 69 (WDR69), mRNA	NM_178821	Hs.424594	AK127882
WIPF1	WAS/WASL interacting protein family, member 1 (WIPF1), transcript variant 2, mRNA	NM_001077269	Hs.654521	NM_001077269
WIPF2	WAS/WASL interacting protein family, member 2 (WIPF2), mRNA	NM_133264	Hs.421622	NM_133264
WTAP	Wilms tumor 1 associated protein (WTAP), transcript variant 3, mRNA	NM_152858	Hs.446091	CR627456
WWTR1	WW domain containing transcription regulator 1 (WWTR1), mRNA	NM_015472	Hs.699296	AL833852
XPNPEP1	X-prolyl aminopeptidase (aminopeptidase P) 1, soluble (XPNPEP1), mRNA	NM_020383	Hs.390623	AK095447
XPNPEP1	X-prolyl aminopeptidase (aminopeptidase P) 1, soluble (XPNPEP1), mRNA	NM_020383	Hs.390623	AK095447
XPR1	xenotropic and polytropic retrovirus receptor (XPR1), mRNA	NM_004736	Hs.227656	BC028576
XRN1	5'-3' exoribonuclease 1 (XRN1), transcript variant 1, mRNA	NM_019001	Hs.435103	BX640905
YIPF3	Yip1 domain family, member 3 (YIPF3), mRNA	NM_015388	Hs.440950	AK021433
YIPF5	Yip1 domain family, member 5 (YIPF5), transcript variant 1, mRNA	NM_001024947	Hs.372050	AY640926
YKT6	YKT6 v-SNARE homolog (S. cerevisiae) (YKT6), mRNA	NM_006555	Hs.520794	NM_006555
YPEL2	yippee-like 2 (Drosophila) (YPEL2), mRNA	NM_001005404	Hs.463613	NM_001005404
ZBTB38	cDNA clone IMAGE:6168734.	BC072415	Hs.518301	NM_001080412
ZC3H7A	zinc finger CCCH-type containing 7A (ZC3H7A), mRNA	NM_014153	Hs.371856	NM_014153
ZDHHC21	zinc finger, DHHC-type containing 21 (ZDHHC21), mRNA	NM_178566	Hs.649522	AK127313
ZDHHC21	HSPC097 mRNA, partial cds.	AF161360	Hs.649522	AK127313
ZFAND2A	zinc finger, AN1-type domain 2A (ZFAND2A), mRNA	NM_182491	Hs.648111	AK123998
ZFAND2B	zinc finger, AN1-type domain 2B (ZFAND2B), mRNA	NM_138802	Hs.534540	AK091345
ZFAND3	zinc finger, AN1-type domain 3 (ZFAND3), mRNA	NM_021943	Hs.36959	AK023284
ZFAND3	zinc finger, AN1-type domain 3 (ZFAND3), mRNA	NM_021943	Hs.36959	AK023284
ZFP28	zinc finger protein 28 homolog (mouse) (ZFP28), mRNA	NM_020828	Hs.14794	AF507045
ZFP91	zinc finger protein 91 homolog (mouse) (ZFP91), transcript variant 1, mRNA	NM_053023	Hs.524920	NM_053023
ZFX	zinc finger protein, X-linked (ZFX), mRNA	NM_003410	Hs.336681	AB209892
ZMAT5	zinc finger, matrin type 5 (ZMAT5), transcript variant 1, mRNA	NM_019103	Hs.643608	BM547909
ZMYND11	zinc finger, MYND domain containing 11 (ZMYND11), transcript variant 2, mRNA	NM_212479	Hs.292265	NM_006624
ZNF132	zinc finger protein 132 (ZNF132), mRNA	NM_003433	Hs.156169	NM_003433
ZNF169	zinc finger protein 169, mRNA (cDNA clone IMAGE:5259146), complete cds.	BC035060	Hs.387623	AK122832
ZNF229	zinc finger protein 229 (ZNF229), mRNA	NM_014518	Hs.48589	AL833447
ZNF276	zinc finger protein 276 (ZNF276), mRNA	NM_152287	Hs.290154	AK056405
ZNF276	zinc finger protein 276 (ZNF276), mRNA	NM_152287	Hs.290154	AK056405
ZNF333	Zinc finger protein 333.	ENST00000292530	Unknown	
ZNF337	zinc finger protein 337 (ZNF337), mRNA	NM_015655	Hs.661684	CR621860
ZNF343	zinc finger protein 343 (ZNF343), mRNA	NM_024325	Hs.516846	NM_024325
ZNF343	zinc finger protein 343 (ZNF343), mRNA	NM_024325	Hs.516846	NM_024325
ZNF37A	zinc finger protein 37A (ZNF37A), transcript variant 1, mRNA	NM_001007094	Hs.292575	BX647089
ZNF44	zinc finger protein 44 (ZNF44), mRNA	NM_016264	Hs.656794	NM_016264
ZNF471	mRNA for KIAA1396 protein, partial cds.	AB037817	Hs.230188	AB037817
ZNF493	zinc finger protein 493, mRNA (cDNA clone IMAGE:4617832), partial cds.	BC022394	Unknown	
ZNF501	zinc finger protein 501 (ZNF501), mRNA	NM_145044	Hs.401045	BC013762
ZNF524	zinc finger protein 524 (ZNF524), mRNA	NM_153219	Hs.440291	NM_153219
ZNF528	zinc finger protein 528 (ZNF528), mRNA	NM_032423	Hs.662043	AK056552
ZNF542	zinc finger protein 542 (ZNF542) on chromosome 19	NR_003127	Unknown	
ZNF547	zinc finger protein 547 (ZNF547), mRNA	NM_173631	Hs.446620	NM_173631
ZNF619	zinc finger protein 619 (ZNF619), mRNA	NM_173656	Hs.407159	NM_173656
ZNF653	zinc finger protein 653 (ZNF653), mRNA	NM_138783	Hs.465928	BC016816
ZNF658B	zinc finger protein 658B (ZNF658B), mRNA	NM_001032297	Hs.534812	NM_001032297
ZNF70	zinc finger protein 70 (ZNF70), mRNA	NM_021916	Hs.382874	NM_021916
ZNF707	zinc finger protein 707 (ZNF707), mRNA	NM_173831	Hs.521922	AK055198
ZNF720	full-length cDNA clone CS0DN002YO09 of Adult brain of (human).	CR611323	Unknown	

ZNF767	zinc finger family member 767 (ZNF767), mRNA	NM_024910	Hs.696011	AK022762
ZNF772	zinc finger protein 772 (ZNF772), mRNA	NM_001024596	Hs.388810	BX647068
ZNF79	zinc finger protein 79 (ZNF79), mRNA	NM_007135	Hs.522399	AK054606
ZNF79	zinc finger protein 79 (ZNF79), mRNA	NM_007135	Hs.522399	AK054606
ZNF799	ZNF799 protein (Fragment).	ENST00000357979	Unknown	
ZSCAN4	zinc finger and SCAN domain containing 4 (ZSCAN4), mRNA	NM_152677	Hs.469663	AK092424
ZYG11B	zyg-11 homolog B (C. elegans) (ZYG11B), mRNA	NM_024646	Hs.476280	NM_024646
A_23_P143676	A_23_P143676	A_23_P143676	Unknown	
A_23_P145596	A_23_P145596	A_23_P145596	Unknown	
A_23_P17152	A_23_P17152	A_23_P17152	Unknown	
A_23_P205500	A_23_P205500	A_23_P205500	Unknown	
A_23_P206741	A_23_P206741	A_23_P206741	Unknown	
A_23_P247	A_23_P247	A_23_P247	Unknown	
A_23_P57836	A_23_P57836	A_23_P57836	Unknown	
A_23_P91130	A_23_P91130	A_23_P91130	Unknown	
A_24_P152315	A_24_P152315	A_24_P152315	Unknown	
A_24_P152792	A_24_P152792	A_24_P152792	Unknown	
A_24_P187355	A_24_P187355	A_24_P187355	Unknown	
A_24_P213144	A_24_P213144	A_24_P213144	Unknown	
A_24_P221960	A_24_P221960	A_24_P221960	Unknown	
A_24_P272523	A_24_P272523	A_24_P272523	Unknown	
A_24_P272653	A_24_P272653	A_24_P272653	Unknown	
A_24_P279760	A_24_P279760	A_24_P279760	Unknown	
A_24_P290257	A_24_P290257	A_24_P290257	Unknown	
A_24_P298179	A_24_P298179	A_24_P298179	Unknown	
A_24_P32207	A_24_P32207	A_24_P32207	Unknown	
A_24_P370484	A_24_P370484	A_24_P370484	Unknown	
A_24_P384119	A_24_P384119	A_24_P384119	Unknown	
A_24_P392742	A_24_P392742	A_24_P392742	Unknown	
A_24_P399341	A_24_P399341	A_24_P399341	Unknown	
A_24_P408981	A_24_P408981	A_24_P408981	Unknown	
A_24_P409410	A_24_P409410	A_24_P409410	Unknown	
A_24_P41593	A_24_P41593	A_24_P41593	Unknown	
A_24_P534243	A_24_P534243	A_24_P534243	Unknown	
A_24_P565898	A_24_P565898	A_24_P565898	Unknown	
A_24_P599225	A_24_P599225	A_24_P599225	Unknown	
A_24_P600622	A_24_P600622	A_24_P600622	Unknown	
A_24_P622697	A_24_P622697	A_24_P622697	Unknown	
A_24_P657629	A_24_P657629	A_24_P657629	Unknown	
A_24_P669822	A_24_P669822	A_24_P669822	Unknown	
A_24_P681563	A_24_P681563	A_24_P681563	Unknown	
A_24_P692600	A_24_P692600	A_24_P692600	Unknown	
A_24_P701582	A_24_P701582	A_24_P701582	Unknown	
A_24_P7470	A_24_P7470	A_24_P7470	Unknown	
A_24_P832737	A_24_P832737	A_24_P832737	Unknown	
A_24_P834646	A_24_P834646	A_24_P834646	Unknown	
A_24_P872359	A_24_P872359	A_24_P872359	Unknown	
A_24_P900721	A_24_P900721	A_24_P900721	Unknown	
A_24_P910080	A_24_P910080	A_24_P910080	Unknown	
A_24_P910381	A_24_P910381	A_24_P910381	Unknown	
A_24_P912985	A_24_P912985	A_24_P912985	Unknown	
A_24_P917897	A_24_P917897	A_24_P917897	Unknown	

A_24_P925334	A_24_P925334	A_24_P925334	Unknown	
A_24_P932706	A_24_P932706	A_24_P932706	Unknown	
A_24_P938583	A_24_P938583	A_24_P938583	Unknown	
A_24_P941070	A_24_P941070	A_24_P941070	Unknown	
A_32_P117908	A_32_P117908	A_32_P117908	Unknown	
A_32_P121234	A_32_P121234	A_32_P121234	Unknown	
A_32_P127412	A_32_P127412	A_32_P127412	Unknown	
A_32_P140501	A_32_P140501	A_32_P140501	Unknown	
A_32_P157671	A_32_P157671	A_32_P157671	Unknown	
A_32_P163046	A_32_P163046	A_32_P163046	Unknown	
A_32_P168727	A_32_P168727	A_32_P168727	Unknown	
A_32_P170814	A_32_P170814	A_32_P170814	Unknown	
A_32_P17416	A_32_P17416	A_32_P17416	Unknown	
A_32_P174385	A_32_P174385	A_32_P174385	Unknown	
A_32_P190334	A_32_P190334	A_32_P190334	Unknown	
A_32_P192354	A_32_P192354	A_32_P192354	Unknown	
A_32_P210193	A_32_P210193	A_32_P210193	Unknown	
A_32_P210744	A_32_P210744	A_32_P210744	Unknown	
A_32_P21742	A_32_P21742	A_32_P21742	Unknown	
A_32_P223327	A_32_P223327	A_32_P223327	Unknown	
A_32_P24431	A_32_P24431	A_32_P24431	Unknown	
A_32_P36225	A_32_P36225	A_32_P36225	Unknown	
A_32_P39991	A_32_P39991	A_32_P39991	Unknown	
A_32_P43415	A_32_P43415	A_32_P43415	Unknown	
A_32_P46351	A_32_P46351	A_32_P46351	Unknown	
A_32_P59532	A_32_P59532	A_32_P59532	Unknown	
A_32_P64928	A_32_P64928	A_32_P64928	Unknown	
A_32_P687	A_32_P687	A_32_P687	Unknown	
A_32_P78876	A_32_P78876	A_32_P78876	Unknown	
A_32_P8627	A_32_P8627	A_32_P8627	Unknown	
A_32_P94521	A_32_P94521	A_32_P94521	Unknown	
A_32_P96085	A_32_P96085	A_32_P96085	Unknown	
A_32_P99804	A_32_P99804	A_32_P99804	Unknown	

Supplemental Table B2

K-Means Cluster 2 -- 2453 genes

Gene Name	Description	Acc	UGCluster	UGRepAcc
AA076992	AA076992 7B05H11 Chromosome 7 Fetal Brain cDNA Library cDNA clone 7B05H11, mRNA sequence	AA076992	Hs.694689	AL040395
AA132288	AA132288 zo27h09.r1 Stratagene colon (#937204) cDNA clone IMAGE:588161 5', mRNA sequence	AA132288	Unknown	
AA291137	AA291137 zs46b08.s1 NCI_CGAP_GCB1 cDNA clone IMAGE:700503 3', mRNA sequence	AA291137	Hs.471040	BC036456
AA507111	AA507111 nh41e06.s1 NCI_CGAP_Pr5 cDNA clone IMAGE:954946, mRNA sequence	AA507111	Hs.529023	NM_018181
AA516102	AA516102 ni31d10.s1 NCI_CGAP_Ew1 cDNA clone IMAGE:971923, mRNA sequence	AA516102	Hs.532286	NM_016258
AA564209	AA564209 nk42g12.s1 NCI_CGAP_GC2 cDNA clone IMAGE:1016230 3', mRNA sequence	AA564209	Hs.700846	DB313114
AA639753	nq82c12.s1 NCI_CGAP_Co9 cDNA clone IMAGE:1158838 3', mRNA sequence	AA639753	Hs.547580	AA639753
AA719641	AA719641 zg52f01.s1 Soares_pineal_gland_N3HPG cDNA clone IMAGE:396985 3', mRNA sequence	AA719641	Hs.504352	NM_016522
AA725860	AA725860 ai23f08.s1 Soares_testis_NHT cDNA clone 1343655 3', mRNA sequence	AA725860	Hs.301048	AK226075
AA744682	AA744682 ny26b06.s1 NCI_CGAP_GCB1 cDNA clone IMAGE:1272851 3', mRNA sequence	AA744682	Hs.444360	NM_173825
AA807748	AA807748 nv66h01.s1 NCI_CGAP_GCB1 cDNA clone IMAGE:1234801 3', mRNA sequence	AA807748	Hs.99836	AL832122
AADAT	aminoadipate aminotransferase (AADAT), transcript variant 1, mRNA	NM_016228	Hs.529735	AF481738
AB002438	mRNA from chromosome 5q21-22, clone:FBR89.	AB002438	Hs.156967	BC032619
AB011149	mRNA for KIAA0577 protein, partial cds.	AB011149	Unknown	
AB073660	primary neuroblastoma cDNA, clone:Nbla00437, full insert sequence.	AB073660	Hs.657083	AB073660
AB263414	MI related novel mRNA, variant-1.	AB263414	Hs.696021	AB263414
ACAA2	acetyl-Coenzyme A acyltransferase 2 (mitochondrial 3-oxoacyl-Coenzyme A thiolase) (ACAA2), nuclear gene encoding mitochondrial protein, mRNA	NM_006111	Hs.200136	NM_001080467
ACN9	ACN9 homolog (S. cerevisiae) (ACN9), mRNA	NM_020186	Hs.592269	BC028409
ACSL3	acyl-CoA synthetase long-chain family member 3 (ACSL3), transcript variant 1, mRNA	NM_004457	Hs.655772	NM_004457
ACTR3B	ARP3 actin-related protein 3 homolog B (yeast) (ACTR3B), transcript variant 1, mRNA	NM_020445	Hs.647117	AB209174
ACTR6	ARP6 actin-related protein 6 homolog (yeast) (ACTR6), mRNA	NM_022496	Hs.115088	AK124075
ACVR1C	activin A receptor, type IC (ACVR1C), mRNA	NM_145259	Hs.352338	BC022530
ACVR2B	activin A receptor, type IIB (ACVR2B), mRNA	NM_001106	Hs.174273	NM_001106
ACVR2B	activin A receptor, type IIB (ACVR2B), mRNA	NM_001106	Hs.174273	NM_001106
ACVR2B	activin A receptor, type IIB (ACVR2B), mRNA	NM_001106	Hs.174273	NM_001106
ADAMTS13	ADAM metallopeptidase with thrombospondin type 1 motif, 13 (ADAMTS13), transcript variant 1, mRNA	NM_139025	Hs.131433	AB069698
ADAMTS16	ADAM metallopeptidase with thrombospondin type 1 motif, 16 (ADAMTS16), mRNA	NM_139056	Hs.661915	NM_139056
ADAMTS20	cDNA FLJ13166 fis, clone NT2RP3003701, weakly similar to F-SPONDIN PRECURSOR.	AK023228	Hs.287554	AF488804
ADAR	adenosine deaminase, RNA-specific (ADAR), transcript variant 1, mRNA	NM_001111	Hs.12341	X79448
ADARB2	adenosine deaminase, RNA-specific, B2 (RED2 homolog rat) (ADARB2), mRNA	NM_018702	Hs.657984	NM_018702
ADCY2	adenylate cyclase 2 (brain) (ADCY2), mRNA	NM_020546	Hs.481545	CR749634
ADNP	Activity-dependent neuroprotector (Activity-dependent neuroprotective protein).	ENST00000349014	Unknown	
ADNP	activity-dependent neuroprotector (ADNP), transcript variant 1, mRNA	NM_015339	Hs.570355	BC075794
ADRA2A	adrenergic, alpha-2A-, receptor (ADRA2A), mRNA	NM_000681	Hs.249159	NM_000681
ADRA2C	adrenergic, alpha-2C-, receptor (ADRA2C), mRNA	NM_000683	Hs.123022	BC142625
ADRBK2	adrenergic, beta, receptor kinase 2 (ADRBK2), mRNA	NM_005160	Hs.657494	NM_005160
AEBP2	AE binding protein 2 (AEBP2), mRNA	NM_153207	Hs.126497	AB209384
AF037219	PIX1 mRNA sequence.	AF037219	Hs.654383	NM_130839
AF085829	full length insert cDNA clone YI39D03.	AF085829	Hs.447840	AK126727
AF086013	full length insert cDNA clone YW18F01.	AF086013	Hs.684714	AA640840
AF086427	full length insert cDNA clone ZD79D11.	AF086427	Hs.597703	BX106238
AF088004	full length insert cDNA clone YY51E04.	AF088004	Hs.659586	BQ774355
AF111851	PRO0611 mRNA, complete cds.	AF111851	Unknown	
AF113012	PRO0767 mRNA, complete cds.	AF113012	Unknown	
AF116603	PRO0386 mRNA, complete cds.	AF116603	Hs.621374	AF116603
AF118062	PRO1386 mRNA, complete cds.	AF118062	Unknown	
AF118071	PRO1693 mRNA, complete cds.	AF118071	Unknown	
AF130062	clone FLB7715 PRO2051 mRNA, complete cds.	AF130062	Hs.491351	NM_004859

AF132204	PRO2259 mRNA, complete cds.	AF132204	Hs.554262	AF132204
AF150379	AF150379 Human mRNA from cd34+ stem cells cDNA clone CBMAJE07, mRNA sequence	AF150379	Hs.568999	AF150379
AF161353	HSPC090 mRNA, partial cds.	AF161353	Hs.669325	AF150232
AF209507	olfactory receptor-like protein mRNA, complete sequence.	AF209507	Hs.447469	AF209507
AF279773	clone N1 NTera2D1 teratocarcinoma mRNA.	AF279773	Hs.660713	AF279773
AF279782	clone P1 NTera2D1 teratocarcinoma mRNA.	AF279782	Hs.553947	AF279782
AF318318	pp10394 mRNA, complete cds.	AF318318	Hs.684472	AF318318
AF333762	FKSG41 (FKSG41) mRNA, complete cds.	AF333762	Unknown	
AF339813	clone IMAGE:297403, mRNA sequence.	AF339813	Hs.656512	AF339813
AF355802	CYP3A5 mRNA, allele CYP3A5*3, exon 5B and partial cds, alternatively spliced.	AF355802	Unknown	
AF445027	clone 114 tumor rejection antigen mRNA, complete cds.	AF445027	Hs.592426	AF445027
AGTPBP1	ATP/GTP binding protein 1 (AGTPBP1), mRNA	NM_015239	Hs.494321	AB028958
AHCTF1	AT hook containing transcription factor 1 (AHCTF1), mRNA	NM_015446	Hs.300887	NM_015446
AHCTF1	AT hook containing transcription factor 1 (AHCTF1), mRNA	NM_015446	Hs.300887	NM_015446
AHI1	Abelson helper integration site 1 (AHI1), mRNA	NM_017651	Hs.386684	NM_017651
AI090937	AI090937 qa52c01.s1 Soares_NhHMPu_S1 cDNA clone IMAGE:1690368 3', mRNA sequence	AI090937	Hs.677678	BG329526
AI276257	AI276257 ql65f01.x1 Soares_NhHMPu_S1 cDNA clone IMAGE:1877209 3', mRNA sequence	AI276257	Hs.477440	AF392454
AI458424	AI458424 tj82b06.x1 Soares_NSF_F8_9W_OT_PA_P_S1 cDNA clone IMAGE:2147987 3', mRNA sequence	AI458424	Hs.412597	Z26317
AI536613	to81h03.x1 NCI_CGAP_Gas4 cDNA clone IMAGE:2184725 3', mRNA sequence	AI536613	Hs.695182	BF674064
AI676010	wc04b06.x1 NCI_CGAP_Pr28 cDNA clone IMAGE:2314163 3', mRNA sequence	AI676010	Hs.635597	AI676010
AI791143	AI791143 ac17d04.x5 Stratagene ovary (#937217) cDNA clone IMAGE:856711 3' similar to contains Alu repetitive element;, mRNA sequence	AI791143	Unknown	
AI937300	wp75e09.x1 NCI_CGAP_Brn25 cDNA clone IMAGE:2467624 3' similar to TR:O75276 O75276 PKD1 ;, mRNA sequence	AI937300	Hs.578874	AI937300
AIM1L	absent in melanoma 1-like (AIM1L), mRNA	NM_017977	Hs.128738	NM_001039775
AJ420543	mRNA full length insert cDNA clone EUROIMAGE 1090207.	AJ420543	Hs.547712	AJ420543
AJ519285	partial mRNA for IgM immunoglobulin heavy chain variable region (IGHV gene), clone ANBPM204.	AJ519285	Hs.551925	BQ706730
AJAP1	adherens junction associated protein 1 (AJAP1), transcript variant 1, mRNA	NM_018836	Hs.25924	NM_018836
AK021458	cDNA FLJ11396 fis, clone HEMBA1000604.	AK021458	Hs.649599	AK021458
AK021664	cDNA FLJ11602 fis, clone HEMBA1003908.	AK021664	Hs.653123	BX537526
AK021689	cDNA FLJ11627 fis, clone HEMBA1004225.	AK021689	Hs.137567	AK021689
AK021693	cDNA FLJ11631 fis, clone HEMBA1004267.	AK021693	Hs.699348	AK021693
AK021744	cDNA FLJ11682 fis, clone HEMBA1004880.	AK021744	Hs.191828	AK021744
AK021751	cDNA FLJ11689 fis, clone HEMBA1004977.	AK021751	Hs.645624	AK021751
AK021866	cDNA FLJ11804 fis, clone HEMBA1006272, moderately similar to RETROVIRUS-RELATED PROTEASE (EC 3.4.23.-).	AK021866	Unknown	
AK022015	cDNA FLJ11953 fis, clone HEMBB1000883.	AK022015	Hs.677083	AK022015
AK022024	cDNA FLJ11962 fis, clone HEMBB1001047.	AK022024	Hs.677085	AK022024
AK022045	cDNA FLJ11983 fis, clone HEMBB1001337.	AK022045	Hs.655386	AK024201
AK022793	cDNA FLJ12731 fis, clone NT2RP2000108.	AK022793	Hs.162105	BC043213
AK022893	cDNA FLJ12831 fis, clone NT2RP2003099.	AK022893	Hs.288478	BC037533
AK023104	cDNA FLJ13042 fis, clone NT2RP3001318.	AK023104	Hs.234478	AK023104
AK023121	cDNA FLJ13059 fis, clone NT2RP3001589.	AK023121	Unknown	
AK023376	cDNA FLJ13314 fis, clone OVARC1001506, highly similar to POLYCYSTIN PRECURSOR.	AK023376	Hs.648395	AK131084
AK023515	cDNA FLJ13453 fis, clone PLACE1003205.	AK023515	Hs.301715	AK023515
AK023645	cDNA FLJ13583 fis, clone PLACE1009050.	AK023645	Hs.658324	AK023645
AK023682	cDNA FLJ13620 fis, clone PLACE1010947.	AK023682	Hs.593575	AK023682
AK023737	cDNA FLJ13675 fis, clone PLACE1011875, highly similar to mRNA for KIAA0580 protein.	AK023737	Unknown	
AK024092	cDNA FLJ14030 fis, clone HEMBA1004086.	AK024092	Hs.220864	NM_001271
AK024127	cDNA FLJ14065 fis, clone HEMBB1000917.	AK024127	Hs.660822	AK024127
AK024870	cDNA: FLJ21217 fis, clone COL00536.	AK024870	Hs.173135	NM_006482
AK024938	cDNA: FLJ21285 fis, clone COL01912.	AK024938	Hs.677298	AK024938
AK025613	cDNA: FLJ21960 fis, clone HEP05517.	AK025613	Hs.696159	NM_018387
AK026267	cDNA: FLJ22614 fis, clone HSI05089.	AK026267	Hs.632607	AK026267

AK026295	cDNA: FLJ22642 fis, clone HSI06970.	AK026295	Hs.288232	AK026295
AK026659	cDNA: FLJ23006 fis, clone LNG00414.	AK026659	Hs.79881	AK026659
AK026811	cDNA: FLJ23158 fis, clone LNG09623.	AK026811	Unknown	
AK027383	cDNA FLJ14477 fis, clone MAMMA1001522.	AK027383	Hs.632037	AK027383
AK027667	cDNA FLJ14761 fis, clone NT2RP3003302.	AK027667	Unknown	
AK054806	cDNA FLJ30244 fis, clone BRACE2002157.	AK054806	Hs.684446	AK054806
AK054852	cDNA FLJ30290 fis, clone BRACE2002884.	AK054852	Hs.486508	AL833602
AK054946	cDNA FLJ30384 fis, clone BRACE2008114.	AK054946	Hs.661564	AK054946
AK055641	cDNA FLJ31079 fis, clone HSYRA2001595.	AK055641	Hs.349283	AK095108
AK055694	cDNA FLJ31132 fis, clone IMR322000953.	AK055694	Unknown	
AK056135	cDNA FLJ31573 fis, clone NT2RI2001769.	AK056135	Hs.536395	BC041856
AK056973	cDNA FLJ32411 fis, clone SKMUS2000515.	AK056973	Hs.569670	AK056973
AK057015	cDNA FLJ32453 fis, clone SKMUS2001703.	AK057015	Hs.90093	NM_002154
AK057710	cDNA FLJ33148 fis, clone UTERU2000238.	AK057710	Hs.597376	AK057710
AK090670	cDNA FLJ33351 fis, clone BRACE2005063.	AK090670	Hs.657400	AK090670
AK090766	cDNA FLJ33447 fis, clone BRAMY1000098.	AK090766	Hs.655064	AK125793
AK090827	cDNA FLJ33508 fis, clone BRAMY2005094.	AK090827	Hs.595153	CR621577
AK090827	cDNA FLJ33508 fis, clone BRAMY2005094.	AK090827	Hs.595153	CR621577
AK091569	cDNA FLJ34250 fis, clone FCBBF4000529.	AK091569	Hs.594418	AK091569
AK092090	cDNA FLJ34771 fis, clone NT2NE2003150.	AK092090	Hs.433010	AK092090
AK092544	cDNA FLJ35225 fis, clone PROST2001116.	AK092544	Hs.648444	AK092544
AK092810	cDNA FLJ35491 fis, clone SMINT2008625, moderately similar to GLYCINE CLEAVAGE SYSTEM H PROTEIN PRECURSOR.	AK092810	Hs.356055	AK092810
AK092942	cDNA FLJ35623 fis, clone SPLEN2010986.	AK092942	Hs.660700	XR_015431
AK092942	cDNA FLJ35623 fis, clone SPLEN2010986.	AK092942	Hs.660700	XR_015431
AK093617	cDNA FLJ36298 fis, clone THYMU2004344.	AK093617	Hs.536535	AL833073
AK093617	cDNA FLJ36298 fis, clone THYMU2004344.	AK093617	Hs.536535	AL833073
AK093869	cDNA FLJ36550 fis, clone TRACH2008113.	AK093869	Unknown	
AK094447	cDNA FLJ37128 fis, clone BRACE2022928.	AK094447	Hs.635286	AK094447
AK094726	cDNA FLJ37407 fis, clone BRAMY2028472.	AK094726	Hs.654832	AK094726
AK094786	cDNA FLJ37467 fis, clone BRAWH2011920.	AK094786	Hs.616436	AK094786
AK095046	cDNA FLJ37727 fis, clone BRHIP2019972.	AK095046	Hs.49117	NM_006581
AK095046	cDNA FLJ37727 fis, clone BRHIP2019972.	AK095046	Hs.49117	NM_006581
AK095707	cDNA FLJ38388 fis, clone FEBRA2004485.	AK095707	Hs.374278	AK095707
AK097080	cDNA FLJ39761 fis, clone SPLEN1000083.	AK097080	Hs.534942	XR_017612
AK097080	cDNA FLJ39761 fis, clone SPLEN1000083.	AK097080	Hs.534942	XR_017612
AK097497	cDNA FLJ40178 fis, clone TESTI2017932.	AK097497	Hs.683814	AK097497
AK097743	cDNA FLJ40424 fis, clone TESTI2039026.	AK097743	Hs.666576	BC033403
AK097877	cDNA FLJ40558 fis, clone THYMU2002756.	AK097877	Unknown	
AK123096	cDNA FLJ41101 fis, clone BLADE2004670.	AK123096	Unknown	
AK123439	cDNA FLJ41445 fis, clone BRSTN2002105.	AK123439	Hs.701639	AK123439
AK123446	cDNA FLJ41452 fis, clone BRSTN2010363.	AK123446	Hs.520589	XM_499022
AK123649	cDNA FLJ41655 fis, clone FEBRA2025477.	AK123649	Hs.587283	AK123649
AK123649	cDNA FLJ41655 fis, clone FEBRA2025477.	AK123649	Hs.587283	AK123649
AK123765	cDNA FLJ41771 fis, clone IMR322009807.	AK123765	Hs.32333	AK123765
AK124515	cDNA FLJ42524 fis, clone BRACE3001384.	AK124515	Hs.599238	AK124515
AK125648	cDNA FLJ43660 fis, clone SYNOV4004823.	AK125648	Hs.155085	AK125648
AK130071	cDNA FLJ26561 fis, clone LNF03981.	AK130071	Hs.178499	BC040658
AK131472	cDNA FLJ16640 fis, clone TESTI4028938, moderately similar to Zinc finger protein 85.	AK131472	Hs.427120	AK131472
AK2	adenylate kinase 2 (AK2), transcript variant AK2B, mRNA	NM_013411	Hs.470907	NM_013411
AK7	adenylate kinase 7 (AK7), mRNA	NM_152327	Hs.667462	NM_152327
AKAP4	A kinase (PRKA) anchor protein 4 (AKAP4), transcript variant 1, mRNA	NM_003886	Hs.97633	AF072756
AKAP7	A kinase (PRKA) anchor protein 7 (AKAP7), transcript variant gamma, mRNA	NM_016377	Hs.486483	AF152929

AKAP9	A kinase (PRKA) anchor protein (yotiao) 9 (AKAP9), transcript variant 4, mRNA	NM_147166	Hs.651221	NM_147171
AL050204	mRNA; cDNA DKFZp586F1223 (from clone DKFZp586F1223).	AL050204	Hs.28540	AL050204
AL079999	AL079999 DKFZp586P2018_r1 586 (synonym: hute1) cDNA clone DKFZp586P2018 5', mRNA sequence	AL079999	Hs.445036	NM_017759
AL117636	mRNA; cDNA DKFZp434H205 (from clone DKFZp434H205).	AL117636	Hs.592750	AL117636
AL133090	mRNA; cDNA DKFZp434E0528 (from clone DKFZp434E0528).	AL133090	Hs.592567	AL133090
AL133577	mRNA; cDNA DKFZp434G0972 (from clone DKFZp434G0972).	AL133577	Hs.656803	AL133577
AL137342	mRNA; cDNA DKFZp761G1111 (from clone DKFZp761G1111).	AL137342	Hs.144197	NM_003360
AL359055	mRNA full length insert cDNA clone EUROIMAGE 2344436.	AL359055	Hs.547764	AL359055
AL549236	AL549236 AL549236 PLACENTA COT 25-NORMALIZED cDNA clone CS0DI049YP18 3-PRIME, mRNA sequence	AL549236	Hs.654413	CR623537
AL573187	AL573187 AL573187 PLACENTA COT 25-NORMALIZED cDNA clone CS0DI036YC04 3-PRIME, mRNA sequence	AL573187	Hs.259347	NM_173601
AL831999	mRNA; cDNA DKFZp451K063 (from clone DKFZp451K063).	AL831999	Hs.547396	AL831999
ALDH18A1	aldehyde dehydrogenase 18 family, member A1 (ALDH18A1), nuclear gene encoding mitochondrial protein, transcript variant 1, mRNA	NM_002860	Hs.500645	NM_002860
ALDH7A1	aldehyde dehydrogenase 7 family, member A1 (ALDH7A1), mRNA	NM_001182	Hs.483239	NM_001182
ALMS1	Alstrom syndrome 1 (ALMS1), mRNA	NM_015120	Hs.184720	AJ417593
ALMS1	Alstrom syndrome 1 (ALMS1), mRNA	NM_015120	Hs.184720	AJ417593
ALS2CR13	amyotrophic lateral sclerosis 2 (juvenile) chromosome region, candidate 13 (ALS2CR13), mRNA	NM_173511	Hs.471130	NM_173511
AMD1	adenosylmethionine decarboxylase 1 (AMD1), transcript variant 1, mRNA	NM_001634	Hs.159118	BX640599
ANAPC4	anaphase promoting complex subunit 4 (ANAPC4), mRNA	NM_013367	Hs.152173	AL353932
ANGEL2	angel homolog 2 (Drosophila) (ANGEL2), mRNA	NM_144567	Hs.157078	NM_144567
ANKHD1	ankyrin repeat and KH domain containing 1 (ANKHD1), transcript variant 1, mRNA	NM_017747	Hs.653135	NM_020690
ANKRD10	ankyrin repeat domain 10 (ANKRD10), mRNA	NM_017664	Hs.525163	BX641022
ANKRD12	ankyrin repeat domain 12 (ANKRD12), mRNA	NM_015208	Hs.464585	NM_015208
ANKRD21	ankyrin repeat domain 21 (ANKRD21), mRNA	NM_174981	Hs.442712	AY172978
ANKRD21	ankyrin repeat domain 21 (ANKRD21), mRNA	NM_174981	Hs.442712	AY172978
ANKRD32	ankyrin repeat domain 32 (ANKRD32), mRNA	NM_032290	Hs.657315	CR936768
ANKRD32	Ankyrin repeat domain-containing protein 32.	ENST00000255667	Unknown	
ANKRD58	full-length cDNA clone CS0DI054YC18 of Placenta Cot 25-normalized of (human).	ENST00000343905	Unknown	
ANP32E	acidic (leucine-rich) nuclear phosphoprotein 32 family, member E (ANP32E), mRNA	NM_030920	Hs.656466	AL832664
ANUBL1	AN1, ubiquitin-like, homolog (Xenopus laevis) (ANUBL1), mRNA	NM_174890	Hs.89029	BC045587
AP1M2	adaptor-related protein complex 1, mu 2 subunit (AP1M2), mRNA	NM_005498	Hs.18894	BC005021
APOC1	apolipoprotein C-I (APOC1), mRNA	NM_001645	Hs.110675	AJ249921
APOC1	apolipoprotein C-I (APOC1), mRNA	NM_001645	Hs.110675	AJ249921
APPBP1	amyloid beta precursor protein binding protein 1 (APPBP1), transcript variant 2, mRNA	NM_001018159	Hs.460978	NM_001018159
APPL	adaptor protein containing pH domain, PTB domain and leucine zipper motif 1 (APPL), mRNA	NM_012096	Hs.476415	NM_012096
APRIN	androgen-induced proliferation inhibitor (APRIN), mRNA	NM_015032	Hs.699308	AL137201
AQR	cDNA FLJ10311 fis, clone NT2RM2000359, highly similar to mRNA for KIAA0560 protein.	AK001173	Hs.510958	EF553519
ARFIP1	ADP-ribosylation factor interacting protein 1 (arfaptin 1) (ARFIP1), transcript variant 1, mRNA	NM_001025595	Hs.416089	NM_001025595
ARHGAP12	Rho GTPase activating protein 12 (ARHGAP12), mRNA	NM_018287	Hs.499264	AL834250
ARHGAP28	Rho GTPase activating protein 28 (ARHGAP28), transcript variant 2, mRNA	NM_030672	Hs.183114	NM_001010000
ARHGAP9	Rho GTPase activating protein 9 (ARHGAP9), mRNA	NM_032496	Hs.437126	BC006107
ARHGEF16	Rho guanine exchange factor (GEF) 16 (ARHGEF16), mRNA	NM_014448	Hs.87435	CR609468
ARHGEF19	Rho guanine nucleotide exchange factor (GEF) 19 (ARHGEF19), mRNA	NM_153213	Hs.591532	BC040640
ARHGEF5	Rho guanine nucleotide exchange factor (GEF) 5 (ARHGEF5), mRNA	NM_005435	Hs.334	AK160365
ARID1A	AT rich interactive domain 1A (SWI-like) (ARID1A), transcript variant 1, mRNA	NM_006015	Hs.468972	NM_006015
ARID2	AT rich interactive domain 2 (ARID, RFX-like) (ARID2), mRNA	NM_152641	Hs.696080	NM_152641
ARID3A	AT rich interactive domain 3A (BRIGHT-like) (ARID3A), mRNA	NM_005224	Hs.501296	BC060828
ARID3B	AT rich interactive domain 3B (BRIGHT-like) (ARID3B), mRNA	NM_006465	Hs.696207	AB208830
ARL5B	ADP-ribosylation factor-like 5B (ARL5B), mRNA	NM_178815	Hs.25362	BC024163
ARL5B	ADP-ribosylation factor-like protein 5B (ADP-ribosylation factor-like protein 8).	ENST00000377275	Unknown	
ARMC4	armadillo repeat containing 4 (ARMC4), mRNA	NM_018076	Hs.127530	BC085611
ARMC8	armadillo repeat containing 8 (ARMC8), transcript variant 3, mRNA	NM_213654	Hs.266826	AL096748

ARMCX5	armadillo repeat containing, X-linked 5 (ARMCX5), mRNA	NM_022838	Hs.522729	NM_014710
ARSE	arylsulfatase E (chondrodysplasia punctata 1) (ARSE), mRNA	NM_000047	Hs.386975	AK223183
ASCC3L1	activating signal cointegrator 1 complex subunit 3-like 1 (ASCC3L1), mRNA	NM_014014	Hs.246112	NM_014014
ASF1A	ASF1 anti-silencing function 1 homolog A (S. cerevisiae) (ASF1A), mRNA	NM_014034	Hs.292316	NM_014034
ASH2L	ash2 (absent, small, or homeotic)-like (Drosophila) (ASH2L), mRNA	NM_004674	Hs.521530	BC015936
ATAD2B	CDNA FLJ37502 fis, clone BRAWH2016446, moderately similar to TAT- BINDING HOMOLOG 7. (Fragment).	ENST00000238789	Unknown	
ATG10	ATG10 autophagy related 10 homolog (S. cerevisiae) (ATG10), mRNA	NM_031482	Hs.700785	NM_031482
ATG4C	Cysteine protease ATG4C (EC 3.4.22.-) (Autophagy-related protein 4 homolog C) (Autophagin-3) (Autophagy-related cysteine endopeptidase 3) (AUT-like 3 cysteine endopeptidase).	ENST00000317868	Unknown	
ATPAF1	ATP synthase mitochondrial F1 complex assembly factor 1 (ATPAF1), nuclear gene encoding mitochondrial protein, transcript variant 1, mRNA	NM_022745	Hs.100874	AB007963
ATR	ataxia telangiectasia and Rad3 related (ATR), mRNA	NM_001184	Hs.271791	NM_001184
ATRNL1	attractin-like 1 (ATRNL1), mRNA	NM_207303	Hs.501127	NM_207303
ATXN2	ataxin 2 (ATXN2), mRNA	NM_002973	Hs.76253	NM_002973
ATXN7	ataxin 7 (ATXN7), mRNA	NM_000333	Hs.476595	NM_000333
AU157551	AU157551 PLACE1 cDNA clone PLACE1008231 3', mRNA sequence	AU157551	Hs.613725	AU157551
AUH	AU RNA binding protein/enoyl-Coenzyme A hydratase (AUH), nuclear gene encoding mitochondrial protein, mRNA	NM_001698	Hs.175905	AK124142
AUTS2	autism susceptibility candidate 2 (AUTS2), mRNA	NM_015570	Hs.700600	AF326917
AV645774	AV645774 AV645774 GLC cDNA clone GLCAEF02 3', mRNA sequence	AV645774	Unknown	
AV742170	AV742170 AV742170 CB cDNA clone CBFBRD03 5', mRNA sequence	AV742170	Hs.594987	BU616824
AV753543	AV753543 NPD cDNA clone NPDBEC03 5', mRNA sequence	AV753543	Hs.59093	AV753543
AW014022	AW014022 UI-H-BI0-aah-f-09-0-UI.s1 NCI_CGAP_Sub1 cDNA clone IMAGE:2709281 3', mRNA sequence	AW014022	Hs.170953	BG618474
AW090036	AW090036 xd01b06.x1 NCI_CGAP_Brn35 cDNA clone IMAGE:2592467 3', mRNA sequence	AW090036	Hs.104661	NM_015168
AW291149	UI-H-BI2-agb-c-11-0-UI.s1 NCI_CGAP_Sub4 cDNA clone IMAGE:2723709 3', mRNA sequence	AW291149	Hs.609438	AW291149
AW377662	AW377662 PM0-CT0237-141099-001-e02 CT0237 cDNA, mRNA sequence	AW377662	Hs.380164	BX648343
AW771919	hn66e07.x1 NCI_CGAP_Kid11 cDNA clone IMAGE:3032868 3', mRNA sequence	AW771919	Hs.610775	AW771919
AXIN2	axin 2 (conductin, axil) (AXIN2), mRNA	NM_004655	Hs.156527	CR933657
AZIN1	antizyme inhibitor 1 (AZIN1), transcript variant 1, mRNA	NM_015878	Hs.459106	NM_015878
BAIAP2L1	BAI1-associated protein 2-like 1 (BAIAP2L1), mRNA	NM_018842	Hs.656063	AK124269
BAIAP2L2	cDNA: FLJ22582 fis, clone HSI02576.	AK026235	Unknown	
BARD1	BRCA1 associated RING domain 1 (BARD1), mRNA	NM_000465	Hs.591642	AK223409
BAT2D1	BAT2 domain containing 1 (BAT2D1), mRNA	NM_015172	Hs.494614	NM_015172
BAT2D1	BAT2 domain containing 1 (BAT2D1), mRNA	NM_015172	Hs.494614	NM_015172
BAZ1B	bromodomain adjacent to zinc finger domain, 1B (BAZ1B), mRNA	NM_032408	Hs.694847	NM_032408
BC000206	Homo sapiens, clone IMAGE:3350658, mRNA.	BC000206	Unknown	
BC001783	mRNA similar to ribosomal protein L35a (cDNA clone IMAGE:3355533).	BC001783	Unknown	
BC010544	cDNA clone IMAGE:3462401, partial cds.	BC010544	Hs.660769	BQ067694
BC010544	cDNA clone IMAGE:3462401, partial cds.	BC010544	Hs.660769	BQ067694
BC012528	cDNA clone IMAGE:3928921, partial cds.	BC012528	Hs.330741	CX788943
BC014971	Homo sapiens, Similar to tubulin, beta, 2, clone IMAGE:4873024, mRNA.	BC014971	Hs.513833	BC014971
BC017904	Homo sapiens, clone IMAGE:4272979, mRNA, partial cds.	BC017904	Hs.680649	BC017904
BC029452	Homo sapiens, clone IMAGE:3681561, mRNA.	BC029452	Hs.662210	BC051759
BC029927	Homo sapiens, clone IMAGE:5167950, mRNA.	BC029927	Hs.535746	AL117627
BC031013	Homo sapiens, clone IMAGE:4655360, mRNA.	BC031013	Hs.325015	BC031013
BC033250	Homo sapiens, clone IMAGE:5441027, mRNA, partial cds.	BC033250	Hs.679977	BC033250
BC033829	cDNA clone IMAGE:3856003, partial cds.	BC033829	Hs.371240	NM_005100
BC033983	cDNA clone IMAGE:5295564.	BC033983	Hs.568860	BC047404
BC035247	cDNA clone IMAGE:4827879.	BC035247	Hs.387011	BQ070964
BC035377	cDNA clone IMAGE:4826240.	BC035377	Hs.633960	CD244199
BC037535	cDNA clone IMAGE:5274219.	BC037535	Hs.597434	BC037740
BC037740	cDNA clone IMAGE:5263531.	BC037740	Hs.597434	BC037740
BC037838	cDNA clone IMAGE:4813920.	BC037838	Hs.594876	AK025546

BC038245	Homo sapiens, clone IMAGE:5241654, mRNA.	BC038245	Hs.335413	BC038245
BC038355	Homo sapiens, clone IMAGE:3858719, mRNA.	BC038355	Hs.654953	BC038355
BC038512	cDNA clone IMAGE:5262734.	BC038512	Hs.296031	BC038512
BC038747	cDNA clone IMAGE:5268658.	BC038747	Hs.572495	BC038747
BC044608	cDNA clone IMAGE:4827340.	BC044608	Hs.635056	BC044608
BC046476	cDNA clone IMAGE:5276802.	BC046476	Hs.434326	BC017749
BC047111	cDNA clone IMAGE:5314178.	BC047111	Hs.595378	BC047111
BC048201	Homo sapiens, clone IMAGE:3660074, mRNA.	BC048201	Hs.558872	BC070147
BC053353	cDNA clone IMAGE:5246408, partial cds.	BC053353	Hs.604397	BC053353
BC062758	cDNA clone IMAGE:4081583, partial cds.	BC062758	Hs.571424	BC062758
BC063381	cDNA clone IMAGE:4301941, partial cds.	BC063381	Hs.626096	BC063381
BC063542	cDNA clone IMAGE:4525305, **** WARNING: chimeric clone ****.	BC063542	Unknown	
BC063641	cDNA clone IMAGE:4745832, partial cds.	BC063641	Hs.471695	BC063393
BC067244	cDNA clone IMAGE:4807381, partial cds.	BC067244	Hs.153412	BC030623
BC067244	cDNA clone IMAGE:4807381, partial cds.	BC067244	Hs.153412	BC030623
BC067244	cDNA clone IMAGE:4807381, partial cds.	BC067244	Hs.153412	BC030623
BC067244	cDNA clone IMAGE:4807381, partial cds.	BC067244	Hs.153412	BC030623
BC067244	cDNA clone IMAGE:4807381, partial cds.	BC067244	Hs.153412	BC030623
BC067244	cDNA clone IMAGE:4807381, partial cds.	BC067244	Hs.153412	BC030623
BC067244	cDNA clone IMAGE:4807381, partial cds.	BC067244	Hs.153412	BC030623
BC067244	cDNA clone IMAGE:4807381, partial cds.	BC067244	Hs.153412	BC030623
BC067244	cDNA clone IMAGE:4807381, partial cds.	BC067244	Hs.153412	BC030623
BC070363	cDNA clone IMAGE:3960708, partial cds.	BC070363	Hs.465405	BI517643
BC073935	cDNA clone IMAGE:5219247, partial cds.	BC073935	Hs.434403	AK074886
BC082970	cDNA clone IMAGE:6598034.	BC082970	Hs.329266	NM_005338
BC087732	cDNA clone IMAGE:6253289, **** WARNING: chimeric clone ****.	BC087732	Unknown	
BC092421	cDNA clone IMAGE:30378758.	BC092421	Hs.356757	BC062355
BC092421	cDNA clone IMAGE:30378758.	BC092421	Hs.356757	BC062355
BCKDHB	branched chain keto acid dehydrogenase E1, beta polypeptide (maple syrup urine disease) (BCKDHB), nuclear gene encoding mitochondrial protein, transcript variant 1, mRNA	NM_183050	Hs.654441	BC034481
BCKDHB	branched chain keto acid dehydrogenase E1, beta polypeptide (maple syrup urine disease) (BCKDHB), nuclear gene encoding mitochondrial protein, transcript variant 1, mRNA	NM_183050	Hs.654441	BC034481
BCL11A	B-cell CLL/lymphoma 11A (zinc finger protein) (BCL11A), transcript variant 5, mRNA	NM_138553	Unknown	
BCL11B	B-cell CLL/lymphoma 11B (zinc finger protein) (BCL11B), transcript variant 1, mRNA	NM_138576	Hs.699440	NM_138576
BCL9	B-cell CLL/lymphoma 9 (BCL9), mRNA	NM_004326	Hs.415209	NM_004326
BCLAF1	BCL2-associated transcription factor 1 (BCLAF1), transcript variant 1, mRNA	NM_014739	Hs.486542	NM_014739
BCLAF1	BCL2-associated transcription factor 1 (BCLAF1), transcript variant 1, mRNA	NM_014739	Hs.486542	NM_014739
BCLAF1	BCL2-associated transcription factor 1 (BCLAF1), transcript variant 1, mRNA	NM_014739	Hs.486542	NM_014739
BCOR	BCL6 co-repressor (BCOR), transcript variant 1, mRNA	NM_017745	Hs.659681	BC114220
BDP1	B double prime 1, subunit of RNA polymerase III transcription initiation factor IIIB (BDP1), mRNA	NM_018429	Hs.258272	NM_018429
BDP1	B double prime 1, subunit of RNA polymerase III transcription initiation factor IIIB (BDP1), mRNA	NM_018429	Hs.258272	NM_018429
BE044472	BE044472 ho46b03.x1 Soares_NFL_T_GBC_S1 cDNA clone IMAGE:3040397 3', mRNA sequence	BE044472	Hs.592202	BC042980
BE176693	BE176693 RC3-HT0585-010400-013-g03 HT0585 cDNA, mRNA sequence	BE176693	Hs.624002	BX640918
BE184906	MR1-HT0707-100500-001-a08 HT0707 cDNA, mRNA sequence	BE184906	Hs.675966	BE184907
BE826587	BE826587 QV1-EN0042-300500-224-e04 EN0042 cDNA, mRNA sequence	BE826587	Hs.654700	AK126219
BF513730	BF513730 UI-H-BW1-amy-e-05-0-UI.s1 NCI_CGAP_Sub7 cDNA clone IMAGE:3071696 3', mRNA sequence	BF513730	Hs.3068	NM_003071
BF761348	BF761348 RC2-CS0018-041000-015-g01 CS0018 cDNA, mRNA sequence	BF761348	Unknown	
BF983943	602304519F1 NIH_MGC_88 cDNA clone IMAGE:4396012 5', mRNA sequence	BF983943	Hs.614842	BF983943
BF989562	BF989562 QV4-GN0120-231000-451-g04 GN0120 cDNA, mRNA sequence	BF989562	Hs.675877	CN288981
BG216262	RST35951 Athersys RAGE Library cDNA, mRNA sequence	BG216262	Hs.637431	BG216262
BG292169	BG292169 602386485F1 NIH_MGC_93 cDNA clone IMAGE:4515548 5', mRNA sequence	BG292169	Hs.422901	XM_001130279
BG951379	MR1-CT0735-120101-003-h03 CT0735 cDNA, mRNA sequence	BG951379	Hs.99677	BG951379
BG998430	BG998430 RC4-HT1110-170401-012-b08 HT1110 cDNA, mRNA sequence	BG998430	Hs.133020	BM460676

BHLHB5	basic helix-loop-helix domain containing, class B, 5 (BHLHB5), mRNA	NM_152414	Hs.591870	NM_152414
BHLHB9	basic helix-loop-helix domain containing, class B, 9 (BHLHB9), mRNA	NM_030639	Hs.4276	BC041409
BHMT	betaine-homocysteine methyltransferase (BHMT), mRNA	NM_001713	Hs.80756	BC012616
BI759100	603042504F1 NIH_MGC_116 cDNA clone IMAGE:5182897 5', mRNA sequence	BI759100	Hs.59203	BI759100
BM690036	UI-E-CK1-abr-b-07-0-UI.r1 UI-E-CK1 cDNA clone UI-E-CK1-abr-b-07-0-UI 5', mRNA sequence	BM690036	Hs.121667	BM690036
BM928667	AGENCOURT_6726860 NIH_MGC_100 cDNA clone IMAGE:5798808 5', mRNA sequence	BM928667	Hs.113170	BM928667
BM983822	BM983822 UI-CF-DU1-aay-e-18-0-UI.s1 UI-CF-DU1 cDNA clone UI-CF-DU1-aay-e-18-0-UI 3', mRNA sequence	BM983822	Hs.166463	NM_031844
BMS1L	BMS1-like, ribosome assembly protein (yeast) (BMS1L), mRNA	NM_014753	Hs.10848	BC043345
BPTF	bromodomain PHD finger transcription factor (BPTF), transcript variant 2, mRNA	NM_004459	Hs.444200	NM_182641
BPTF	bromodomain PHD finger transcription factor (BPTF), transcript variant 1, mRNA	NM_182641	Hs.444200	NM_182641
BQ000605	BQ000605 UI-H-DP0-avu-h-21-0-UI.s1 NCI_CGAP_Fs1 cDNA clone IMAGE:5884484 3', mRNA sequence	BQ000605	Hs.185677	AB007899
BQ379494	BQ379494 CM0-UT0003-031100-582-b09 UT0003 cDNA, mRNA sequence	BQ379494	Hs.518525	BC051726
BQ881683	AGENCOURT_8228657 Lupski_dorsal_root_ganglion cDNA clone IMAGE:6182093 5', mRNA sequence	BQ881683	Hs.597961	BQ881683
BRCA1	breast cancer 1, early onset (BRCA1), transcript variant BRCA1b, mRNA	NM_007295	Hs.194143	NM_007295
BRCA1	breast cancer 1, early onset (BRCA1), transcript variant BRCA1b, mRNA	NM_007295	Hs.194143	NM_007295
BRCA1	breast cancer 1, early onset (BRCA1), transcript variant BRCA1b, mRNA	NM_007295	Hs.194143	NM_007295
BRCA1	breast cancer 1, early onset (BRCA1), transcript variant BRCA1b, mRNA	NM_007295	Hs.194143	NM_007295
BRCA1	breast cancer 1, early onset (BRCA1), transcript variant BRCA1b, mRNA	NM_007295	Hs.194143	NM_007295
BRCA1	breast cancer 1, early onset (BRCA1), transcript variant BRCA1b, mRNA	NM_007295	Hs.194143	NM_007295
BRCA1	breast cancer 1, early onset (BRCA1), transcript variant BRCA1b, mRNA	NM_007295	Hs.194143	NM_007295
BRCA1	breast cancer 1, early onset (BRCA1), transcript variant BRCA1b, mRNA	NM_007295	Hs.194143	NM_007295
BRCA1	breast cancer 1, early onset (BRCA1), transcript variant BRCA1b, mRNA	NM_007295	Hs.194143	NM_007295
BRCA1	breast cancer 1, early onset (BRCA1), transcript variant BRCA1b, mRNA	NM_007295	Hs.194143	NM_007295
BRD1	bromodomain containing 1 (BRD1), mRNA	NM_014577	Hs.127950	BX640795
BRD2	bromodomain containing 2 (BRD2), mRNA	NM_005104	Hs.75243	NM_005104
BRD7	bromodomain containing 7 (BRD7), mRNA	NM_013263	Hs.437894	BC094706
BRIP1	BRCA1 interacting protein C-terminal helicase 1 (BRIP1), mRNA	NM_032043	Hs.532799	AF360549
BRWD1	bromodomain and WD repeat domain containing 1 (BRWD1), transcript variant 1, mRNA	NM_018963	Hs.654740	NM_033656
BRWD1	bromodomain and WD repeat domain containing 1 (BRWD1), transcript variant 1, mRNA	NM_018963	Hs.654740	NM_033656
BRWD2	bromodomain and WD repeat domain containing 2 (BRWD2), mRNA	NM_018117	Hs.144447	AL137699
BRWD3	bromodomain and WD repeat domain containing 3 (BRWD3), mRNA	NM_153252	Hs.170667	AY497052
BRWD3	bromodomain and WD repeat domain containing 3 (BRWD3), mRNA	NM_153252	Hs.170667	AY497052
BTAF1	BTAF1 RNA polymerase II, B-TFIID transcription factor-associated, 170kDa (Mot1 homolog, S. cerevisiae) (BTAF1), mRNA	NM_003972	Hs.500526	NM_003972
BTBD3	BTB (POZ) domain containing 3 (BTBD3), transcript variant 1, mRNA	NM_014962	Hs.244590	NM_014962
BTF3	basic transcription factor 3 (BTF3), transcript variant 1, mRNA	NM_001037637	Hs.591768	BQ923254
BTNL3	butyrophilin-like 3 (BTNL3), transcript variant 1, mRNA	NM_197975	Hs.225949	AY358385
BU633383	BU633383 UI-H-FL1-bgu-h-03-0-UI.s1 NCI_CGAP_FL1 cDNA clone UI-H-FL1-bgu-h-03-0-UI 3', mRNA sequence	BU633383	Unknown	
BX089701	BX089701 BX089701 NCI_CGAP_Lu24 cDNA clone IMAGp998I115809 ; IMAGE:2341330, mRNA sequence	BX089701	Hs.661515	BG254791
BX098639	BX098639 Soares_testis_NHT cDNA clone IMAGp998N224405, mRNA sequence	BX098639	Hs.586428	BX098639
BX102475	BX102475 Soares_testis_NHT cDNA clone IMAGp998J051786, mRNA sequence	BX102475	Hs.445721	AW967446
BX106115	BX106115 Soares fetal liver spleen 1NFLS cDNA clone IMAGp998E20122, mRNA sequence	BX106115	Hs.624136	BX106115
BX108468	BX108468 Soares_testis_NHT cDNA clone IMAGp998J144405, mRNA sequence	BX108468	Hs.126101	BX108468
BX116997	BX116997 Soares_placenta_8to9weeks_2NbHP8to9W cDNA clone IMAGp998G16558, mRNA sequence	BX116997	Hs.26297	BX116997
BX281073	BX281073 BX281073 NCI_CGAP_Lu24 cDNA clone IMAGp998O175628 ; IMAGE:2273128, mRNA sequence	BX281073	Hs.445885	NM_019590
BX375060	BX375060 NEUROBLASTOMA COT 25-NORMALIZED cDNA clone CS0DC007YF13 5-PRIME, mRNA sequence	BX375060	Hs.597351	BX375060
BX393727	BX393727 NEUROBLASTOMA COT 25-NORMALIZED cDNA clone CS0DC001YP02 5-PRIME, mRNA sequence	BX393727	Hs.440088	BX393727
BX414807	BX414807 BX414807 THYMUS cDNA clone CS0CAP002YP17 3-PRIME, mRNA sequence	BX414807	Hs.517228	NM_003253
BX537819	mRNA; cDNA DKFZp686B06256 (from clone DKFZp686B06256)	BX537819	Hs.157882	BX537819
BX641027	mRNA; cDNA DKFZp686O10247 (from clone DKFZp686O10247).	BX641027	Hs.463375	AB023141

C10orf137	chromosome 10 open reading frame 137 (C10orf137), mRNA	NM_015608	Hs.468688	NM_015608
C10orf137	chromosome 10 open reading frame 137 (C10orf137), mRNA	NM_015608	Hs.468688	NM_015608
C10orf57	chromosome 10 open reading frame 57 (C10orf57), mRNA	NM_025125	Hs.169982	AL832397
C10orf82	cDNA FLJ40268 fis, clone TESTI2026537.	ENST00000388884	Unknown	
C10orf82	chromosome 10 open reading frame 82 (C10orf82), mRNA	NM_144661	Hs.121347	BX537582
C12orf53	chromosome 12 open reading frame 53 (C12orf53), mRNA	NM_153685	Hs.44067	AK091656
C13orf18	chromosome 13 open reading frame 18 (C13orf18), mRNA	NM_025113	Unknown	
C13orf23	chromosome 13 open reading frame 23 (C13orf23), transcript variant 1, mRNA	NM_025138	Hs.318526	NM_025138
C13orf25	C13orf25 v_2 mRNA, complete cds, miR-91-precursor-13 micro RNA, microRNA miR-91, microRNA miR-17, miR-18-precursor-13 micro RNA, microRNA miR-18, miR-19a-precursor-13 micro RNA, microRNA miR-19a, microRNA miR-20, miR-19b-precursor-13...	AB176708	Hs.24115	AB176708
C13orf7	chromosome 13 open reading frame 7 (C13orf7), mRNA	NM_024546	Hs.567576	CR936752
C14orf104	chromosome 14 open reading frame 104 (C14orf104), mRNA	NM_018139	Hs.231761	NM_018139
C14orf106	chromosome 14 open reading frame 106 (C14orf106), mRNA	NM_018353	Hs.437941	BC065544
C14orf112	chromosome 14 open reading frame 112 (C14orf112), mRNA	NM_016468	Hs.137108	CR603105
C14orf129	chromosome 14 open reading frame 129 (C14orf129), mRNA	NM_016472	Hs.592297	AK094654
C14orf130	chromosome 14 open reading frame 130 (C14orf130), transcript variant 1, mRNA	NM_018108	Unknown	
C14orf131	chromosome 14 open reading frame 131 (C14orf131), mRNA	NM_018335	Hs.106005	NM_018335
C14orf166	chromosome 14 open reading frame 166 (C14orf166), mRNA	NM_016039	Hs.534457	AK129516
C14orf32	chromosome 14 open reading frame 32 (C14orf32), mRNA	NM_144578	Hs.594338	NM_144578
C15orf15	chromosome 15 open reading frame 15 (C15orf15), mRNA	NM_016304	Hs.274772	BF244626
C15orf27	chromosome 15 open reading frame 27 (C15orf27), mRNA	NM_152335	Hs.661411	NM_152335
C15orf42	chromosome 15 open reading frame 42 (C15orf42), mRNA	NM_152259	Hs.441708	NM_152259
C16orf53	chromosome 16 open reading frame 53 (C16orf53), mRNA	NM_024516	Hs.676031	CR609250
C16orf59	chromosome 16 open reading frame 59 (C16orf59), mRNA	NM_025108	Hs.534491	CR612076
C16orf75	chromosome 16 open reading frame 75 (C16orf75), mRNA	NM_152308	Hs.347524	BC039361
C17orf41	chromosome 17 open reading frame 41 (C17orf41), mRNA	NM_024857	Hs.528902	AY557611
C17orf80	HLC-8 mRNA, complete cds.	AY163812	Hs.12929	NM_017941
C18orf21	chromosome 18 open reading frame 21 (C18orf21), mRNA	NM_031446	Hs.37883	BC025950
C19orf46	chromosome 19 open reading frame 46 (C19orf46), mRNA	NM_001039876	Hs.436743	NM_001039876
C1orf106	chromosome 1 open reading frame 106 (C1orf106), mRNA	NM_018265	Hs.518997	AK001763
C1orf109	chromosome 1 open reading frame 109 (C1orf109), mRNA	NM_017850	Hs.272673	AK092319
C1orf112	chromosome 1 open reading frame 112 (C1orf112), mRNA	NM_018186	Hs.443551	AK127098
C1orf121	chromosome 1 open reading frame 121 (C1orf121), mRNA	NM_016076	Hs.498317	NM_016076
C1orf135	chromosome 1 open reading frame 135 (C1orf135), mRNA	NM_024037	Hs.149305	AK024326
C1orf172	chromosome 1 open reading frame 172 (C1orf172), mRNA	NM_152365	Hs.188881	AK091952
C1orf210	chromosome 1 open reading frame 210 (C1orf210), mRNA	NM_182517	Hs.158963	NM_182517
C1orf25	chromosome 1 open reading frame 25 (C1orf25), mRNA	NM_030934	Hs.591488	NM_030934
C1QTNF4	C1q and tumor necrosis factor related protein 4 (C1QTNF4), mRNA	NM_031909	Hs.662633	BQ068291
C20orf11	chromosome 20 open reading frame 11 (C20orf11), mRNA	NM_017896	Hs.353013	NM_017896
C20orf119	chromosome 20 open reading frame 119, mRNA (cDNA clone IMAGE:4745538), with apparent retained intron.	BC039151	Hs.641481	AK124047
C20orf12	chromosome 20 open reading frame 12 (C20orf12), mRNA	NM_018152	Unknown	
C20orf12	chromosome 20 open reading frame 12 (C20orf12), mRNA	NM_018152	Unknown	
C20orf19	chromosome 20 open reading frame 19 (C20orf19), mRNA	NM_018474	Hs.187635	BC039296
C20orf19	chromosome 20 open reading frame 19, mRNA (cDNA clone IMAGE:5261585), complete cds.	BC039296	Hs.187635	BC039296
C20orf23	chromosome 20 open reading frame 23 (C20orf23), mRNA	NM_024704	Hs.101774	AY166853
C20orf42	chromosome 20 open reading frame 42 (C20orf42), mRNA	NM_017671	Hs.472054	AB105105
C20orf46	chromosome 20 open reading frame 46 (C20orf46), mRNA	NM_018354	Hs.516834	AK126837
C21orf129	chromosome 21 open reading frame 129 (C21orf129), mRNA	NM_152506	Unknown	
C21orf57	chromosome 21 open reading frame 57 (C21orf57), transcript variant 1, mRNA	NM_058181	Hs.474066	BC068100
C21orf66	cDNA clone IMAGE:5497083, containing frame-shift errors.	BC062992	Hs.700618	BC062992
C21orf88	cDNA clone MGC:99480 IMAGE:4874993, complete cds.	BC080530	Hs.375120	BC080530
C22orf27	chromosome 22 open reading frame 27 (C22orf27), mRNA	NM_153044	Unknown	

C22orf35	cDNA FLJ25887 fis, clone CBR02996.	AK098753	Hs.696021	AB263414
C2orf14	mRNA; cDNA DKFZp434F1719 (from clone DKFZp434F1719).	AL136789	Hs.633271	XR_015755
C2orf15	chromosome 2 open reading frame 15 (C2orf15), mRNA	NM_144706	Hs.352211	BC021264
C2orf33	chromosome 2 open reading frame 33 (C2orf33), mRNA	NM_020194	Hs.471528	NM_020194
C3orf15	chromosome 3 open reading frame 15 (C3orf15), mRNA	NM_033364	Hs.341906	NM_033364
C3orf17	chromosome 3 open reading frame 17 (C3orf17), transcript variant 1, mRNA	NM_015412	Hs.591288	NM_015412
C3orf38	Uncharacterized protein C3orf38.	ENST00000383697	Unknown	
C3orf41	mRNA; cDNA DKFZp434B172 (from clone DKFZp434B172).	AL117530	Hs.697193	AL117530
C3orf63	chromosome 3 open reading frame 63 (C3orf63), mRNA	NM_015224	Hs.116877	AF180425
C6orf111	chromosome 6 open reading frame 111 (C6orf111), mRNA	NM_032870	Hs.520287	NM_032870
C6orf111	chromosome 6 open reading frame 111 (C6orf111), mRNA	NM_032870	Hs.520287	NM_032870
C6orf111	Splicing factor, arginine/serine-rich 130 (Serine-arginine-rich- splicing regulatory protein 130) (SRrp130) (SR-rich protein) (SR- related protein).	ENST00000369239	Unknown	
C6orf113	chromosome 6 open reading frame 113 (C6orf113), mRNA	NM_145062	Hs.29857	BX537872
C6orf130	chromosome 6 open reading frame 130 (C6orf130), mRNA	NM_145063	Hs.227457	BQ065260
C6orf159	chromosome 6 open reading frame 159 (C6orf159), mRNA	NM_001009994	Hs.149454	AK124105
C6orf168	chromosome 6 open reading frame 168 (C6orf168), mRNA	NM_032511	Hs.573245	AK096480
C6orf170	chromosome 6 open reading frame 170 (C6orf170), mRNA	NM_152730	Hs.121396	AK131446
C6orf170	chromosome 6 open reading frame 170 (C6orf170), mRNA	NM_152730	Hs.121396	AK131446
C6orf211	chromosome 6 open reading frame 211 (C6orf211), mRNA	NM_024573	Hs.15929	AK022972
C6orf59	cDNA: FLJ23112 fis, clone LNG07874.	AK026765	Hs.664873	AK026765
C6orf60	chromosome 6 open reading frame 60 (C6orf60), mRNA	NM_024581	Hs.443789	BC060769
C7orf16	chromosome 7 open reading frame 16 (C7orf16), mRNA	NM_006658	Hs.227011	NM_006658
C7orf24	chromosome 7 open reading frame 24 (C7orf24), mRNA	NM_024051	Hs.530024	AK021779
C7orf46	chromosome 7 open reading frame 46 (C7orf46), mRNA	NM_199136	Hs.592178	BC063130
C9orf102	chromosome 9 open reading frame 102 (C9orf102), transcript variant 1, mRNA	NM_020207	Hs.632686	NM_020207
C9orf116	chromosome 9 open reading frame 116 (C9orf116), transcript variant 1, mRNA	NM_001048265	Hs.414028	AK024007
C9orf45	cDNA FLJ41850 fis, clone NT2RI3003738, highly similar to GL012 mRNA.	AK123844	Hs.657064	CR749230
C9orf71	chromosome 9 open reading frame 71 (C9orf71), mRNA	NM_153237	Hs.663056	AK126504
C9orf72	chromosome 9 open reading frame 72 (C9orf72), transcript variant 1, mRNA	NM_018325	Hs.493639	NM_018325
C9orf93	chromosome 9 open reading frame 93 (C9orf93), mRNA	NM_173550	Hs.17267	CR936775
CA14	carbonic anhydrase XIV (CA14), mRNA	NM_012113	Hs.528988	AK074765
CA312250	CA312250 UI-CF-FN0-afm-d-23-0-UI.s1 UI-CF-FN0 cDNA clone UI-CF-FN0-afm-d-23-0-UI 3', mRNA sequence	CA312250	Hs.307836	BQ723235
CA423858	UI-H-FE1-bed-l-01-0-UI.s1 NCI_CGAP_FE1 cDNA clone UI-H-FE1-bed-l-01-0-UI 3', mRNA sequence	CA423858	Hs.541501	CA423858
CACHD1	cache domain containing 1 (CACHD1), mRNA	NM_020925	Hs.443891	BX537603
CACNG2	cDNA FLJ30520 fis, clone BRAWH2000866.	AK055082	Hs.680609	AK055082
CALB1	calbindin 1, 28kDa (CALB1), mRNA	NM_004929	Hs.65425	BX537530
CALCA	calcitonin/calcitonin-related polypeptide, alpha (CALCA), transcript variant 3, mRNA	NM_001033953	Hs.37058	X02330
CAND1	cullin-associated and neddylation-dissociated 1 (CAND1), mRNA	NM_018448	Hs.546407	AB020636
CAPN12	calpain 12 (CAPN12), mRNA	NM_144691	Hs.653110	NM_144691
CAPN12	calpain 12 (CAPN12), mRNA	NM_144691	Hs.653110	NM_144691
CAPN13	calpain 13 (CAPN13), mRNA	NM_144575	Hs.660911	BX647678
CAPN6	calpain 6 (CAPN6), mRNA	NM_014289	Hs.496593	AJ000388
CASC3	cancer susceptibility candidate 3 (CASC3), mRNA	NM_007359	Hs.694800	NM_007359
CASP8AP2	CASP8 associated protein 2 (CASP8AP2), mRNA	NM_012115	Hs.558218	BC150274
CASP9	caspase 9, apoptosis-related cysteine peptidase (CASP9), transcript variant alpha, mRNA	NM_001229	Hs.329502	AB209147
CASP9	caspase 9, apoptosis-related cysteine peptidase (CASP9), transcript variant alpha, mRNA	NM_001229	Hs.329502	AB209147
CB305813	CB305813 UI-CF-EN1-aeg-d-07-0-UI.s1 UI-CF-EN1 cDNA clone UI-CF-EN1-aeg-d-07-0-UI 3', mRNA sequence	CB305813	Hs.473152	NM_003222
CB852269	CB852269 UI-CF-FN0-afm-c-12-0-UI.s1 UI-CF-FN0 cDNA clone UI-CF-FN0-afm-c-12-0-UI 3', mRNA sequence	CB852269	Hs.661088	AK023844
CBX1	chromobox homolog 1 (HP1 beta homolog Drosophila) (CBX1), mRNA	NM_006807	Hs.77254	NM_006807
CBX3	chromobox homolog 3 (HP1 gamma homolog, Drosophila) (CBX3), transcript variant 2, mRNA	NM_016587	Hs.381189	BX647444
CCAR1	cell division cycle and apoptosis regulator 1 (CCAR1), mRNA	NM_018237	Hs.49853	AK128100

CCDC112	coiled-coil domain containing 112 (CCDC112), transcript variant 1, mRNA	NM_001040440	Hs.436121	NM_152549
CCDC113	coiled-coil domain containing 113 (CCDC113), mRNA	NM_014157	Hs.11614	NM_014157
CCDC120	coiled-coil domain containing 120 (CCDC120), mRNA	NM_033626	Hs.522643	BC008769
CCDC131	coiled-coil domain containing 131 (CCDC131), mRNA	NM_144982	Hs.527874	NM_144982
CCDC14	coiled-coil domain containing 14 (CCDC14), mRNA	NM_022757	Hs.656256	BX537652
CCDC24	coiled-coil domain containing 24 (CCDC24), mRNA	NM_152499	Hs.632394	AK092569
CCDC34	coiled-coil domain containing 34 (CCDC34), transcript variant 1, mRNA	NM_030771	Hs.143733	BC008496
CCDC39	mRNA; cDNA DKFZp434A128 (from clone DKFZp434A128).	AL122120	Hs.652356	AL122120
CCDC41	coiled-coil domain containing 41 (CCDC41), transcript variant 1, mRNA	NM_016122	Hs.279209	NM_016122
CCDC45	coiled-coil domain containing 45 (CCDC45), mRNA	NM_138363	Hs.569713	BX641136
CCDC45	coiled-coil domain containing 45 (CCDC45), mRNA	NM_138363	Hs.569713	BX641136
CCDC54	coiled-coil domain containing 54 (CCDC54), mRNA	NM_032600	Hs.164799	NM_032600
CCDC55	coiled-coil domain containing 55 (CCDC55), transcript variant 1, mRNA	NM_032141	Hs.462663	AK094552
CCDC66	coiled-coil domain containing 66 (CCDC66), mRNA	NM_001012506	Hs.476399	AL833326
CCDC77	coiled-coil domain containing 77 (CCDC77), mRNA	NM_032358	Hs.631656	AK027638
CCDC84	coiled-coil domain containing 84 (CCDC84), mRNA	NM_198489	Hs.534613	AB094093
CCNB1IP1	cyclin B1 interacting protein 1 (CCNB1IP1), transcript variant 3, mRNA	NM_182851	Hs.107003	NM_182851
CCNE1	cyclin E1 (CCNE1), transcript variant 1, mRNA	NM_001238	Hs.244723	BC035498
CCT2	chaperonin containing TCP1, subunit 2 (beta) (CCT2), mRNA	NM_006431	Hs.189772	NM_006431
CCT8	chaperonin containing TCP1, subunit 8 (theta) (CCT8), mRNA	NM_006585	Hs.125113	BC012584
CD19	CD19 molecule (CD19), mRNA	NM_001770	Hs.652262	BC052294
CD24	CD24 signal transducer mRNA, complete cds and 3' region.	L33930	Hs.694721	AK125531
CD247	CD247 molecule (CD247), transcript variant 1, mRNA	NM_198053	Hs.156445	AK223325
CD52	CD52 molecule (CD52), mRNA	NM_001803	Hs.276770	BQ276596
CD653123	CD653123 AGENCOURT_14552349 NIA Human H1 Embryonic Stem Cell cDNA Library (Long) cDNA clone IMAGE:30425925 5', mRNA sequence	CD653123	Hs.87889	NM_177438
CD69	CD69 molecule (CD69), mRNA	NM_001781	Hs.208854	NM_001781
CD69	CD69 molecule (CD69), mRNA	NM_001781	Hs.208854	NM_001781
CDC23	cell division cycle 23 homolog (S. cerevisiae) (CDC23), mRNA	NM_004661	Hs.153546	NM_004661
CDC2L6	cell division cycle 2-like 6 (CDK8-like), mRNA (cDNA clone IMAGE:5296862).	BC036637	Unknown	
CDC5L	CDC5 cell division cycle 5-like (S. pombe) (CDC5L), mRNA	NM_001253	Hs.485471	AB007892
CDC7	cell division cycle 7 homolog (S. cerevisiae) (CDC7), mRNA	NM_003503	Hs.533573	AB209337
CDH1	cadherin 1, type 1, E-cadherin (epithelial) (CDH1), mRNA	NM_004360	Hs.461086	NM_004360
CDH1	cadherin 1, type 1, E-cadherin (epithelial) (CDH1), mRNA	NM_004360	Hs.461086	NM_004360
CDH1	cadherin 1, type 1, E-cadherin (epithelial) (CDH1), mRNA	NM_004360	Hs.461086	NM_004360
CDH1	cadherin 1, type 1, E-cadherin (epithelial) (CDH1), mRNA	NM_004360	Hs.461086	NM_004360
CDH1	cadherin 1, type 1, E-cadherin (epithelial) (CDH1), mRNA	NM_004360	Hs.461086	NM_004360
CDH1	cadherin 1, type 1, E-cadherin (epithelial) (CDH1), mRNA	NM_004360	Hs.461086	NM_004360
CDH1	cadherin 1, type 1, E-cadherin (epithelial) (CDH1), mRNA	NM_004360	Hs.461086	NM_004360
CDH1	cadherin 1, type 1, E-cadherin (epithelial) (CDH1), mRNA	NM_004360	Hs.461086	NM_004360
CDH1	cadherin 1, type 1, E-cadherin (epithelial) (CDH1), mRNA	NM_004360	Hs.461086	NM_004360
CDH1	cadherin 1, type 1, E-cadherin (epithelial) (CDH1), mRNA	NM_004360	Hs.461086	NM_004360
CDH3	cadherin 3, type 1, P-cadherin (placental) (CDH3), mRNA	NM_001793	Hs.461074	BC040486
CDH4	cDNA: FLJ22202 fis, clone HRC01333.	AK025855	Hs.598638	AK092092
CDK8	cyclin-dependent kinase 8 (CDK8), mRNA	NM_001260	Hs.382306	BC094784
CDX1	caudal type homeobox transcription factor 1 (CDX1), mRNA	NM_001804	Hs.1545	NM_001804
CECR6	cat eye syndrome chromosome region, candidate 6 (CECR6), mRNA	NM_031890	Hs.209577	AF307451
CEI	coordinated expression to IRXA2 homeobox gene (CEI), mRNA	NM_178569	Hs.668017	NM_178569
CELSR2	cadherin, EGF LAG seven-pass G-type receptor 2 (flamingo homolog, Drosophila) (CELSR2), mRNA	NM_001408	Hs.57652	AF234887
CELSR3	cadherin, EGF LAG seven-pass G-type receptor 3 (flamingo homolog, Drosophila) (CELSR3), mRNA	NM_001407	Hs.631926	NM_001407
CENPJ	centromere protein J (CENPJ), mRNA	NM_018451	Hs.513379	NM_018451
CENPP	Centromere protein P (CENP-P).	ENST00000375587	Unknown	

CENPQ	centromere protein Q (CENPQ), mRNA	NM_018132	Hs.88663	NM_018132
CENPQ	centromere protein Q (CENPQ), mRNA	NM_018132	Hs.88663	NM_018132
CENPQ	centromere protein Q (CENPQ), mRNA	NM_018132	Hs.88663	NM_018132
CENPQ	centromere protein Q (CENPQ), mRNA	NM_018132	Hs.88663	NM_018132
CENPQ	centromere protein Q (CENPQ), mRNA	NM_018132	Hs.88663	NM_018132
CENPQ	centromere protein Q (CENPQ), mRNA	NM_018132	Hs.88663	NM_018132
CENPQ	centromere protein Q (CENPQ), mRNA	NM_018132	Hs.88663	NM_018132
CENPQ	centromere protein Q (CENPQ), mRNA	NM_018132	Hs.88663	NM_018132
CENPQ	centromere protein Q (CENPQ), mRNA	NM_018132	Hs.88663	NM_018132
CENPQ	centromere protein Q (CENPQ), mRNA	NM_018132	Hs.88663	NM_018132
CENTB2	centaurin, beta 2 (CENTB2), mRNA	NM_012287	Hs.654597	BC060767
CENTG2	centaurin, gamma 2 (CENTG2), transcript variant 1, mRNA	NM_001037131	Hs.435039	NM_001037131
CEP135	centrosomal protein 135kDa (CEP135), mRNA	NM_025009	Hs.518767	NM_025009
CEP57	centrosomal protein 57kDa (CEP57), mRNA	NM_014679	Hs.101014	NM_014679
CEP70	centrosomal protein 70kDa (CEP70), mRNA	NM_024491	Hs.531962	NM_024491
CETN3	centrin, EF-hand protein, 3 (CDC31 homolog, yeast) (CETN3), mRNA	NM_004365	Hs.591767	BF218841
CGI-09	CGI-09 protein (CGI-09), mRNA	NM_015939	Hs.128791	AB032979
CGI-115	CGI-115 protein (CGI-115), mRNA	NM_016052	Hs.660109	NM_016052
CGI-115	CGI-115 protein (CGI-115), mRNA	ENST00000366932	Unknown	
CGN	cingulin (CGN), mRNA	NM_020770	Hs.591464	BC146657
CHD1	chromodomain helicase DNA binding protein 1 (CHD1), mRNA	NM_001270	Hs.696018	NM_001270
CHD1L	chromodomain helicase DNA binding protein 1-like (CHD1L), mRNA	NM_004284	Hs.191164	NM_004284
CHD6	chromodomain helicase DNA binding protein 6 (CHD6), mRNA	NM_032221	Hs.371979	NM_032221
CHD7	chromodomain helicase DNA binding protein 7 (CHD7), mRNA	NM_017780	Hs.20395	NM_017780
CHD7	chromodomain helicase DNA binding protein 7 (CHD7), mRNA	NM_017780	Hs.20395	NM_017780
CHDH	cDNA FLJ30840 fis, clone FEBRA2002442, highly similar to partial mRNA for choline dehydrogenase.	AK055402	Hs.126688	AK055402
CHERP	calcium homeostasis endoplasmic reticulum protein (CHERP), mRNA	NM_006387	Hs.631627	NM_006387
CHIC1	mRNA; cDNA DKFZp686F2342 (from clone DKFZp686F2342).	CR936642	Hs.496323	NM_001039840
CHKA	choline kinase alpha (CHKA), transcript variant 1, mRNA	NM_001277	Hs.569019	AK054792
CHML	choroideremia-like (Rab escort protein 2) (CHML), mRNA	NM_001821	Hs.654545	NM_001821
CHML	choroideremia-like (Rab escort protein 2) (CHML), mRNA	NM_001821	Hs.654545	NM_001821
CHMP2B	chromatin modifying protein 2B (CHMP2B), mRNA	NM_014043	Hs.476930	AK002165
CHN1	chimerin (chimaerin) 1 (CHN1), transcript variant 1, mRNA	NM_001822	Hs.654534	NM_001822
CHST8	carbohydrate (N-acetylgalactosamine 4-0) sulfotransferase 8 (CHST8), mRNA	NM_022467	Hs.165724	NM_022467
CHST9	carbohydrate (N-acetylgalactosamine 4-0) sulfotransferase 9 (CHST9), mRNA	NM_031422	Hs.657938	NM_031422
CIB4	calcium and integrin binding family member 4 (CIB4), mRNA	NM_001029881	Hs.591579	BF681360
CLDN12	claudin 12 (CLDN12), mRNA	NM_012129	Hs.258576	CR933597
CLDN23	claudin 23 (CLDN23), mRNA	NM_194284	Hs.183617	NM_194284
CLDN3	claudin 3 (CLDN3), mRNA	NM_001306	Hs.647023	NM_001306
CLEC2D	C-type lectin domain family 2, member D (CLEC2D), transcript variant 2, mRNA	NM_001004419	Hs.268326	NM_001004419
CLEC2D	C-type lectin domain family 2, member D (CLEC2D), transcript variant 1, mRNA	NM_013269	Hs.268326	NM_001004419
CLRN1	clarin 1 (CLRN1), transcript variant 1, mRNA	NM_174878	Hs.380222	NM_174878
CN430008	CN430008 17000600067619 GRN_PRENEU cDNA 5', mRNA sequence	CN430008	Unknown	
CN431194	CN431194 328775669 GRN_ES cDNA 5', mRNA sequence	CN431194	Hs.591205	NM_007129
CN478720	CN478720 UI-CF-FN0-afu-e-07-0-UI.s1 UI-CF-FN0 cDNA clone UI-CF-FN0-afu-e-07-0-UI 3', mRNA sequence	CN478720	Hs.595302	BF792881
CN479126	CN479126 UI-CF-FN0-afv-i-22-0-UI.s1 UI-CF-FN0 cDNA clone UI-CF-FN0-afv-i-22-0-UI 3', mRNA sequence	CN479126	Hs.504609	BG165657
CNIH3	cornichon homolog 3 (Drosophila) (CNIH3), mRNA	NM_152495	Hs.28659	CR595048
CNNM4	cyclin M4 (CNNM4), mRNA	NM_020184	Hs.656229	BC063295
CNOT1	CCR4-NOT transcription complex, subunit 1 (CNOT1), transcript variant 1, mRNA	NM_016284	Hs.460923	NM_016284
CNOT10	CCR4-NOT transcription complex, subunit 10 (CNOT10), mRNA	NM_015442	Hs.444851	AK022952
CNOT7	CCR4-NOT transcription complex, subunit 7 (CNOT7), transcript variant 1, mRNA	NM_013354	Hs.598387	BC060852

COCH	coagulation factor C homolog, cochlin (Limulus polyphemus) (COCH), mRNA	NM_004086	Hs.21016	AK123362
COCH	coagulation factor C homolog, cochlin (Limulus polyphemus) (COCH), mRNA	NM_004086	Hs.21016	AK123362
COL20A1	collagen, type XX, alpha 1 (COL20A1), mRNA	NM_020882	Hs.271285	AB040943
COL4A3BP	Goodpasture antigen-binding protein (EC 2.7.11.9) (GPBP) (Collagen type IV alpha-3-binding protein) (StAR-related lipid transfer protein 11) (StARD11) (START domain-containing protein 11).	ENST00000380494	Unknown	
COL4A6	collagen, type IV, alpha 6 (COL4A6), transcript variant B, mRNA	NM_033641	Hs.145586	NM_033641
COMMD4	COMM domain containing 4 (COMMD4), mRNA	NM_017828	Hs.351327	AK124968
COMMD5	COMM domain containing 5 (COMMD5), mRNA	NM_014066	Hs.631856	AF113540
COPG2	coatomer protein complex, subunit gamma 2 (COPG2), mRNA	NM_012133	Hs.532231	NM_012133
CORO2A	Coronin-2A (WD repeat-containing protein 2) (IR10).	ENST00000375077	Unknown	
COX18	COX18 cytochrome c oxidase assembly homolog (S. cerevisiae) (COX18), mRNA	NM_173827	Hs.356697	NM_173827
COX6B2	cytochrome c oxidase subunit VIb polypeptide 2 (testis) (COX6B2), mRNA	NM_144613	Hs.550544	AK057427
CPSF3	cleavage and polyadenylation specific factor 3, 73kDa (CPSF3), mRNA	NM_016207	Hs.515972	BC014106
CPSF6	cleavage and polyadenylation specific factor 6, 68kDa (CPSF6), mRNA	NM_007007	Hs.369606	NM_007007
CR591264	full-length cDNA clone CS0DC023YK19 of Neuroblastoma Cot 25-normalized of (human).	CR591264	Hs.556108	AK091492
CR594072	full-length cDNA clone CS0DI005YB22 of Placenta Cot 25-normalized of (human).	CR594072	Hs.128702	AK091414
CR594208	full-length cDNA clone CS0DH004YD13 of T cells (Jurkat cell line) of (human).	CR594208	Hs.348514	BC014384
CR595483	full-length cDNA clone CS0CAP008YG08 of Thymus of (human).	CR595483	Hs.646505	XR_015997
CR596214	full-length cDNA clone CS0DC006YB07 of Neuroblastoma Cot 25-normalized of (human).	CR596214	Hs.662029	BG036557
CR597846	full-length cDNA clone CS0DC012YL18 of Neuroblastoma Cot 25-normalized of (human).	CR597846	Hs.628886	CR597846
CR598370	full-length cDNA clone CS0DF003YI18 of Fetal brain of (human).	CR598370	Hs.444291	CR598370
CR600369	full-length cDNA clone CS0DF025YM09 of Fetal brain of (human).	CR600369	Hs.654774	BX537762
CR602210	full-length cDNA clone CS0DA003YL19 of Neuroblastoma of (human).	CR602210	Hs.655150	BC010437
CR605719	full-length cDNA clone CS0DK002YG10 of HeLa cells Cot 25-normalized of (human).	CR605719	Hs.593076	CR605719
CR605947	full-length cDNA clone CS0DD004YC02 of Neuroblastoma Cot 50-normalized of (human).	CR605947	Hs.663861	BX395274
CR607569	full-length cDNA clone CS0DF027YA11 of Fetal brain of (human).	CR607569	Hs.516075	AB209211
CR607939	full-length cDNA clone CS0DB009YI22 of Neuroblastoma Cot 10-normalized of (human).	CR607939	Hs.349705	AK131521
CR608275	full-length cDNA clone CS0CAP007YE04 of Thymus of (human).	CR608275	Hs.594184	CR608275
CR611122	full-length cDNA clone CS0DF022YM06 of Fetal brain of (human).	CR611122	Hs.593461	CR611122
CR611712	full-length cDNA clone CS0DB005YH06 of Neuroblastoma Cot 10-normalized of (human).	CR611712	Hs.596900	CR611712
CR616939	full-length cDNA clone CS0DF026YH11 of Fetal brain of (human).	CR616939	Hs.223770	XM_065166
CR617018	full-length cDNA clone CS0DG001YH13 of B cells (Ramos cell line) of (human).	CR617018	Hs.434075	CR607989
CR617865	full-length cDNA clone CS0DF021YD16 of Fetal brain of (human).	CR617865	Hs.525163	BX641022
CR620293	full-length cDNA clone CS0DF028YD24 of Fetal brain of (human).	CR620293	Hs.696021	AB263414
CR620977	full-length cDNA clone CS0CAP004YK15 of Thymus of (human).	CR620977	Hs.377961	CR620977
CR623787	full-length cDNA clone CS0DI079YL01 of Placenta Cot 25-normalized of (human).	CR623787	Hs.687264	CR623787
CR624679	full-length cDNA clone CS0DF003YC20 of Fetal brain of (human).	CR624679	Hs.399952	CR624679
CR626729	full-length cDNA clone CS0DF009YE11 of Fetal brain of (human).	CR626729	Hs.446336	AK128712
CR627415	mRNA; cDNA DKFZp686G19280 (from clone DKFZp686G19280).	CR627415	Unknown	
CR744556	CR744556 CR744556 NCI_CGAP_Co3 cDNA clone IMAGp998M072299 ; IMAGE:925230 5', mRNA sequence	CR744556	Unknown	
CR936791	mRNA; cDNA DKFZp781C2356 (from clone DKFZp781C2356).	CR936791	Hs.696414	CR936791
CRABP1	cellular retinoic acid binding protein 1 (CRABP1), mRNA	NM_004378	Hs.346950	AK096006
CREB1	cAMP responsive element binding protein 1 (CREB1), transcript variant B, mRNA	NM_134442	Hs.584750	NM_134442
CREB3L4	cAMP responsive element binding protein 3-like 4 (CREB3L4), mRNA	NM_130898	Hs.372924	AY049977
CREBZF	CREB/ATF bZIP transcription factor (CREBZF), mRNA	NM_001039618	Hs.535319	NM_001039618
CRNKL1	Crn, crooked neck-like 1 (Drosophila) (CRNKL1), mRNA	NM_016652	Hs.171342	NM_016652
CROP	cisplatin resistance-associated overexpressed protein (CROP), transcript variant 2, mRNA	NM_006107	Hs.130293	NM_016424
CRYM	crystallin, mu (CRYM), transcript variant 1, mRNA	NM_001888	Hs.924	BX648477
CSAG2	CSAG family, member 2 (CSAG2), mRNA	NM_004909	Hs.522810	AF136715
CSAG3A	CSAG family, member 3A (CSAG3A), mRNA	NM_203311	Hs.522810	AF136715
CSDE1	cold shock domain containing E1, RNA-binding (CSDE1), transcript variant 1, mRNA	NM_001007553	Hs.700602	CR749378
CSDE1	cold shock domain containing E1, RNA-binding (CSDE1), transcript variant 1, mRNA	NM_001007553	Hs.700602	CR749378

CSE1L	CSE1 chromosome segregation 1-like (yeast) (CSE1L), mRNA	NM_001316	Hs.90073	NM_001316
CSNK1G3	casein kinase 1, gamma 3 (CSNK1G3), transcript variant 4, mRNA	NM_001044723	Hs.129206	NM_001044723
CSPP1	centrosome and spindle pole associated protein 1 (CSPP1), transcript variant 1, mRNA	NM_001077204	Hs.370147	NM_001077204
CSRP2BP	CSRP2 binding protein (CSRP2BP), transcript variant 1, mRNA	NM_020536	Hs.488051	NM_020536
CSTF2T	cleavage stimulation factor, 3' pre-RNA, subunit 2, 64kDa, tau variant (CSTF2T), mRNA	NM_015235	Hs.591358	NM_015235
CSTF3	cleavage stimulation factor, 3' pre-RNA, subunit 3, 77kDa (CSTF3), transcript variant 1, mRNA	NM_001326	Hs.44402	NM_001326
CTAGE3	CTAGE-3 protein mRNA, complete cds.	AF338231	Hs.623891	AF338231
CTBP1	C-terminal binding protein 1 (CTBP1), transcript variant 2, mRNA	NM_001012614	Hs.208597	BC064333
CTBP2	C-terminal binding protein 2 (CTBP2), transcript variant 2, mRNA	NM_022802	Hs.501345	NM_022802
CTDSPL2	CTD (carboxy-terminal domain, RNA polymerase II, polypeptide A) small phosphatase like 2 (CTDSPL2), mRNA	NM_016396	Hs.646495	NM_016396
CTLA4	cytotoxic T-lymphocyte-associated protein 4 (CTLA4), transcript variant 1, mRNA	NM_005214	Hs.247824	AF414120
CTPS	CTP synthase (CTPS), mRNA	NM_001905	Hs.473087	NM_001905
CTTNBP2	cortactin binding protein 2 (CTTNBP2), mRNA	NM_033427	Hs.592285	NM_033427
CUL1	cullin 1 (CUL1), mRNA	NM_003592	Hs.146806	NM_003592
CUL5	cullin 5 (CUL5), mRNA	NM_003478	Hs.440320	NM_003478
CXorf23	chromosome X open reading frame 23 (CXorf23), mRNA	NM_198279	Hs.28896	AL833278
CXXC6	CXXC-type zinc finger protein 6 (Leukemia-associated protein with a CXXC domain).	ENST00000373644	Unknown	
CXXC6	CXXC-type zinc finger protein 6 (Leukemia-associated protein with a CXXC domain).	ENST00000373644	Unknown	
CYB5A	cytochrome b5 type A (microsomal) (CYB5A), transcript variant 2, mRNA	NM_001914	Hs.465413	AB209617
CYP26A1	cytochrome P450, family 26, subfamily A, polypeptide 1 (CYP26A1), transcript variant 2, mRNA	NM_057157	Hs.150595	AK027560
D70835	mRNA for Zinc-finger protein, partial cds.	D70835	Hs.264345	AB209601
DA380034	DA380034 BRTHA2 cDNA clone BRTHA2013080 5', mRNA sequence	DA380034	Hs.628261	DA372386
DARS	aspartyl-tRNA synthetase (DARS), mRNA	NM_001349	Hs.503787	NM_001349
DB304731	DB304731 BRAWH3 cDNA clone BRAWH3006941 3', mRNA sequence	DB304731	Hs.593335	DB304731
DB318193	DB318193 HCASM2 cDNA clone HCASM2001866 3', mRNA sequence	DB318193	Hs.701892	DB318193
DBP	D site of albumin promoter (albumin D-box) binding protein (DBP), mRNA	NM_001352	Hs.414480	AB208807
DCC1	defective in sister chromatid cohesion homolog 1 (S. cerevisiae) (DCC1), mRNA	NM_024094	Hs.315167	BC001316
DCC1	defective in sister chromatid cohesion homolog 1 (S. cerevisiae) (DCC1), mRNA	NM_024094	Hs.315167	BC001316
DCC1	defective in sister chromatid cohesion homolog 1 (S. cerevisiae) (DCC1), mRNA	NM_024094	Hs.315167	BC001316
DCC1	defective in sister chromatid cohesion homolog 1 (S. cerevisiae) (DCC1), mRNA	NM_024094	Hs.315167	BC001316
DCC1	defective in sister chromatid cohesion homolog 1 (S. cerevisiae) (DCC1), mRNA	NM_024094	Hs.315167	BC001316
DCC1	defective in sister chromatid cohesion homolog 1 (S. cerevisiae) (DCC1), mRNA	NM_024094	Hs.315167	BC001316
DCC1	defective in sister chromatid cohesion homolog 1 (S. cerevisiae) (DCC1), mRNA	NM_024094	Hs.315167	BC001316
DCC1	defective in sister chromatid cohesion homolog 1 (S. cerevisiae) (DCC1), mRNA	NM_024094	Hs.315167	BC001316
DCC1	defective in sister chromatid cohesion homolog 1 (S. cerevisiae) (DCC1), mRNA	NM_024094	Hs.315167	BC001316
DCC1	defective in sister chromatid cohesion homolog 1 (S. cerevisiae) (DCC1), mRNA	NM_024094	Hs.315167	BC001316
DCLRE1A	DNA cross-link repair 1A (PSO2 homolog, S. cerevisiae) (DCLRE1A), mRNA	NM_014881	Hs.1560	D42045
DCP2	DCP2 decapping enzyme homolog (S. cerevisiae) (DCP2), mRNA	NM_152624	Hs.443875	NM_152624
DCUN1D1	RP42 protein mRNA, complete cds.	AF292100	Unknown	
DCUN1D1	RP42 protein mRNA, complete cds.	AF292100	Unknown	
DDX25	DEAD (Asp-Glu-Ala-Asp) box polypeptide 25 (DDX25), mRNA	NM_013264	Hs.420263	BC050360
DDX27	DEAD (Asp-Glu-Ala-Asp) box polypeptide 27 (DDX27), mRNA	NM_017895	Hs.65234	AL832131
DDX28	DEAD (Asp-Glu-Ala-Asp) box polypeptide 28 (DDX28), nuclear gene encoding mitochondrial protein, mRNA	NM_018380	Hs.458313	NM_018380
DDX3X	DEAD (Asp-Glu-Ala-Asp) box polypeptide 3, X-linked (DDX3X), mRNA	NM_001356	Hs.380774	NM_001356
DDX46	DEAD (Asp-Glu-Ala-Asp) box polypeptide 46 (DDX46), mRNA	NM_014829	Hs.533245	NM_014829
DDX46	DEAD (Asp-Glu-Ala-Asp) box polypeptide 46 (DDX46), mRNA	NM_014829	Hs.533245	NM_014829
DEFA4	defensin, alpha 4, corticostatin (DEFA4), mRNA	NM_001925	Hs.591391	BU616655
DEK	DEK oncogene (DNA binding) (DEK), mRNA	NM_003472	Hs.695957	BX641063
DENND1C	DENN/MADD domain containing 1C (DENND1C), mRNA	NM_024898	Hs.236449	AL713770
DEPDC4	DEP domain containing 4 (DEPDC4), mRNA	NM_152317	Hs.653118	AK090824
DEPDC4	cDNA FLJ33505 fis, clone BRAMY2004542.	AK090824	Hs.653118	AK090824

DGCR8	DiGeorge syndrome critical region gene 8 (DGCR8), mRNA	NM_022720	Hs.700598	BC037564
DHFR	dihydrofolate reductase (DHFR), mRNA	NM_000791	Hs.648635	NM_000791
DHRS13	dehydrogenase/reductase (SDR family) member 13 (DHRS13), mRNA	NM_144683	Hs.631760	BC015582
DHRS13	dehydrogenase/reductase (SDR family) member 13 (DHRS13), mRNA	NM_144683	Hs.631760	BC015582
DHX36	DEAH (Asp-Glu-Ala-His) box polypeptide 36 (DHX36), mRNA	NM_020865	Hs.446270	AF217190
DHX9	DEAH (Asp-Glu-Ala-His) box polypeptide 9 (DHX9), mRNA	NM_001357	Hs.191518	AK226102
DIO3	deiodinase, iodothyronine, type III (DIO3), mRNA	NM_001362	Hs.49322	NM_001362
DKFZp434F142	mRNA; cDNA DKFZp434F142 (from clone DKFZp434F142).	AL136837	Hs.588334	AL136837
DKFZp434M131	mRNA; cDNA DKFZp434M131 (from clone DKFZp434M131); partial cds.	AL080179	Hs.632070	AL080179
DKFZp586I1420	hypothetical protein DKFZp586I1420 (DKFZp586I1420) on chromosome 7	NR_002186	Unknown	
DKFZP686E2158	hypothetical protein LOC643155 (DKFZP686E2158), mRNA	NM_001048249	Hs.508479	NM_001048249
DLEU7	deleted in lymphocytic leukemia 7 (DLEU7) mRNA, complete cds.	AY357595	Hs.673860	BC035481
DLG2	discs, large homolog 2, chapsyn-110 (Drosophila) (DLG2), mRNA	NM_001364	Hs.654862	NM_001364
DLG3	discs, large homolog 3 (neuroendocrine-dlg, Drosophila) (DLG3), transcript variant 1, mRNA	NM_021120	Hs.522680	NM_021120
DLG5	discs, large homolog 5 (Drosophila) (DLG5), mRNA	NM_004747	Hs.652690	BC146794
DLGAP1	discs, large (Drosophila) homolog-associated protein 1 (DLGAP1), transcript variant alpha, mRNA	NM_004746	Hs.654793	NM_004746
DMTF1	cyclin D binding myb-like transcription factor 1 (DMTF1), mRNA	NM_021145	Hs.654981	AK126664
DMXL1	Dmx-like 1 (DMXL1), mRNA	NM_005509	Hs.181042	NM_005509
DNAH5	dynein, axonemal, heavy chain 5 (DNAH5), mRNA	NM_001369	Hs.212360	NM_001369
DNAJB6	DnaJ (Hsp40) homolog, subfamily B, member 6 (DNAJB6), transcript variant 2, mRNA	NM_005494	Hs.490745	AL832124
DNMT3A	DNA (cytosine-5-)-methyltransferase 3 alpha (DNMT3A), transcript variant 1, mRNA	NM_175629	Hs.515840	AB208833
DNTTIP2	deoxynucleotidyltransferase, terminal, interacting protein 2 (DNTTIP2), mRNA	NM_014597	Hs.85769	AK123702
DNTTIP2	deoxynucleotidyltransferase, terminal, interacting protein 2 (DNTTIP2), mRNA	NM_014597	Hs.85769	AK123702
DNTTIP2	deoxynucleotidyltransferase, terminal, interacting protein 2 (DNTTIP2), mRNA	NM_014597	Hs.85769	AK123702
DNTTIP2	deoxynucleotidyltransferase, terminal, interacting protein 2 (DNTTIP2), mRNA	NM_014597	Hs.85769	AK123702
DNTTIP2	deoxynucleotidyltransferase, terminal, interacting protein 2 (DNTTIP2), mRNA	NM_014597	Hs.85769	AK123702
DNTTIP2	deoxynucleotidyltransferase, terminal, interacting protein 2 (DNTTIP2), mRNA	NM_014597	Hs.85769	AK123702
DNTTIP2	deoxynucleotidyltransferase, terminal, interacting protein 2 (DNTTIP2), mRNA	NM_014597	Hs.85769	AK123702
DNTTIP2	deoxynucleotidyltransferase, terminal, interacting protein 2 (DNTTIP2), mRNA	NM_014597	Hs.85769	AK123702
DNTTIP2	deoxynucleotidyltransferase, terminal, interacting protein 2 (DNTTIP2), mRNA	NM_014597	Hs.85769	AK123702
DNTTIP2	deoxynucleotidyltransferase, terminal, interacting protein 2 (DNTTIP2), mRNA	NM_014597	Hs.85769	AK123702
DOCK3	dedicator of cytokinesis 3 (DOCK3), mRNA	NM_004947	Hs.476284	NM_004947
DPM1	dolichyl-phosphate mannosyltransferase polypeptide 1, catalytic subunit (DPM1), mRNA	NM_003859	Hs.654951	BF217743
DPP10	dipeptidyl-peptidase 10 (DPP10), transcript variant 1, mRNA	NM_020868	Hs.591555	BC030832
DPY19L1P1	cDNA: FLJ23115 fis, clone LNG07933.	AK026768	Hs.633705	AK026768
DQ786238	clone HLS_IMAGE_1881469 mRNA sequence.	DQ786238	Hs.654872	BM557567
DQ786246	clone HLS_IMAGE_204740 mRNA sequence.	DQ786246	Hs.374577	DQ786246
DQ786286	clone HLS_IMAGE_470261 mRNA sequence.	DQ786286	Hs.654588	BC035411
DT220604	KB-EST0005688 BPS7 cDNA, mRNA sequence	DT220604	Hs.645915	DT220604
DTL	denticleless homolog (Drosophila) (DTL), mRNA	NM_016448	Hs.656473	NM_016448
DTL	denticleless homolog (Drosophila) (DTL), mRNA	NM_016448	Hs.656473	NM_016448
DTL	denticleless homolog (Drosophila) (DTL), mRNA	NM_016448	Hs.656473	NM_016448
DTL	denticleless homolog (Drosophila) (DTL), mRNA	NM_016448	Hs.656473	NM_016448
DTL	denticleless homolog (Drosophila) (DTL), mRNA	NM_016448	Hs.656473	NM_016448
DTL	denticleless homolog (Drosophila) (DTL), mRNA	NM_016448	Hs.656473	NM_016448
DTL	denticleless homolog (Drosophila) (DTL), mRNA	NM_016448	Hs.656473	NM_016448
DTL	denticleless homolog (Drosophila) (DTL), mRNA	NM_016448	Hs.656473	NM_016448
DTL	denticleless homolog (Drosophila) (DTL), mRNA	NM_016448	Hs.656473	NM_016448
DTL	denticleless homolog (Drosophila) (DTL), mRNA	NM_016448	Hs.656473	NM_016448
DTNB	dystrobrevin, beta (DTNB), transcript variant 4, mRNA	NM_183360	Hs.307720	Y15718
DYDC2	DPY30 domain containing 2 (DYDC2), mRNA	NM_032372	Hs.512782	BC018606
DYNC2H1	G protein interaction factor 1-like mRNA sequence.	AF288405	Hs.503721	NM_001080463

DYNC2H1	dynein, cytoplasmic 2, heavy chain 1, mRNA (cDNA clone IMAGE:5265846), complete cds.	BC037496	Hs.503721	NM_001080463
DZIP3	zinc finger DAZ interacting protein 3 (DZIP3), mRNA	NM_014648	Hs.409210	BC063882
E2F3	E2F transcription factor 3 (E2F3), mRNA	NM_001949	Hs.269408	NM_001949
E2F5	E2F transcription factor 5, p130-binding (E2F5), mRNA	NM_001951	Hs.445758	X86097
EAF1	ELL associated factor 1 (EAF1), mRNA	NM_033083	Hs.474479	NM_033083
ECEL1	endothelin converting enzyme-like 1 (ECEL1), mRNA	NM_004826	Hs.26880	AB030579
ECHDC1	enoyl Coenzyme A hydratase domain containing 1 (ECHDC1), mRNA	NM_018479	Hs.486410	AL834469
ECT2	epithelial cell transforming sequence 2 oncogene (ECT2), mRNA	NM_018098	Hs.518299	AY376439
EDG4	endothelial differentiation, lysophosphatidic acid G-protein-coupled receptor, 4 (EDG4), mRNA	NM_004720	Hs.122575	AK123043
EED	embryonic ectoderm development (EED), transcript variant 2, mRNA	NM_152991	Hs.503510	NM_152991
EIF1AX	eukaryotic translation initiation factor 1A, X-linked (EIF1AX), mRNA	NM_001412	Hs.522590	NM_001412
EIF2A	eukaryotic translation initiation factor 2A, 65kDa (EIF2A), mRNA	NM_032025	Hs.655782	NM_032025
EIF3S10	eukaryotic translation initiation factor 3, subunit 10 theta, 150/170kDa (EIF3S10), mRNA	NM_003750	Hs.523299	NM_003750
EIF3S6	eukaryotic translation initiation factor 3, subunit 6 48kDa (EIF3S6), mRNA	NM_001568	Hs.405590	AK124178
EIF3S6	eukaryotic translation initiation factor 3, subunit 6 48kDa (EIF3S6), mRNA	NM_001568	Hs.405590	AK124178
EIF4A3	eukaryotic translation initiation factor 4A, isoform 3 (EIF4A3), mRNA	NM_014740	Hs.389649	CR749455
EIF4E	eukaryotic translation initiation factor 4E (EIF4E), mRNA	NM_001968	Hs.249718	NM_001968
EIF4EBP2	Eukaryotic translation initiation factor 4E-binding protein 2 (4E-BP2) (eIF4E-binding protein 2).	ENST00000373218	Unknown	
EIF4EBP2	eukaryotic translation initiation factor 4E binding protein 2 (EIF4EBP2), mRNA	NM_004096	Hs.695953	BC050633
ELF2	E74-like factor 2 (ets domain transcription factor) (ELF2), transcript variant 1, mRNA	NM_201999	Hs.699401	U43188
ELMOD2	ELMO domain-containing protein 2.	ENST00000323570	Unknown	
ELOVL7	ELOVL family member 7, elongation of long chain fatty acids (yeast) (ELOVL7), mRNA	NM_024930	Hs.274256	BC094792
EML4	echinoderm microtubule associated protein like 4 (EML4), mRNA	NM_019063	Hs.593614	NM_019063
ENAH	enabled homolog (Drosophila) (ENAH), transcript variant 1, mRNA	NM_001008493	Hs.497893	NM_001008493
ENST000002197	CAGF9 (Fragment).	ENST00000219746	Unknown	
ENST000002428	Zinc finger CCCH domain-containing protein 13.	ENST00000242848	Unknown	
ENST000002518	Kinesin-like motor protein C20orf23 (Sorting nexin-23).	ENST00000251847	Unknown	
ENST000002542	CDNA FLJ46156 fis, clone TESTI4001569.	ENST00000254271	Unknown	
ENST000002643	SLAIN2 protein.	ENST00000264313	Unknown	
ENST000002720	tubulin, beta 8	ENST00000272035	Unknown	
ENST000002936	SYCE2 protein (Fragment).	ENST00000293695	Unknown	
ENST000002993	mRNA for KIAA1786 protein, partial cds.	ENST00000299308	Unknown	
ENST000003039	ENST00000303979	ENST00000303979	Unknown	
ENST000003060	U6 snRNA-associated Sm-like protein LSm3.	ENST00000306024	Unknown	
ENST000003183	ENST00000318301	ENST00000318301	Unknown	
ENST000003206	chromosome 8 open reading frame 56, mRNA (cDNA clone IMAGE:4820412).	ENST00000320662	Unknown	
ENST000003272	Adenylate kinase isoenzyme 4, mitochondrial (EC 2.7.4.3) (Adenylate kinase 3-like 1) (ATP-AMP transphosphorylase).	ENST00000327299	Unknown	
ENST000003314	PREDICTED: zinc finger protein 716 (ZNF716), mRNA	ENST00000331425	Unknown	
ENST000003354	cDNA FLJ46467 fis, clone THYMU3022668.	ENST00000335459	Unknown	
ENST000003403	full-length cDNA clone CS0DF038YM01 of Fetal brain of (human).	ENST00000340301	Unknown	
ENST000003403	Novel protein (Hypothetical protein RP13-15M17.2).	ENST00000340381	Unknown	
ENST000003411	FUS-interacting serine-arginine-rich protein 1 (TLS-associated protein with Ser-Arg repeats) (TLS-associated protein with SR repeats) (TASR) (TLS-associated serine-arginine protein) (TLS-associated SR protein) (40 kDa SR-repressor protein) (SRrp40)...	ENST00000341154	Unknown	
ENST000003422	Arginine/serine-rich-splicing factor 10 (Transformer-2-beta) (HTRA2- beta) (Transformer 2 protein homolog).	ENST00000342294	Unknown	
ENST000003432	sarcoma antigen NY-SAR-41	ENST00000343253	Unknown	
ENST000003435	EF-hand domain-containing family member C2.	ENST00000343571	Unknown	
ENST000003550	protein expressed in prostate, ovary, testis, and placenta 14	ENST00000355077	Unknown	
ENST000003550	Zinc finger protein 492 (Fragment).	ENST00000355095	Unknown	
ENST000003550	Zinc finger protein 492 (Fragment).	ENST00000355095	Unknown	
ENST000003561	Doublesex- and mab-3-related transcription factor C1.	ENST00000356102	Unknown	
ENST000003577	similar to nuclear pore complex interacting protein (LOC440348), mRNA	ENST00000357796	Unknown	

ENST000003588	ENST00000358828	ENST00000358828	Unknown	
ENST000003695	Zinc finger protein 292.	ENST00000369577	Unknown	
ENST000003712	tetratricopeptide repeat domain 22	ENST00000371276	Unknown	
ENST000003713	helicase, lymphoid-specific	ENST00000371327	Unknown	
ENST000003720	Chordin-like protein 1 precursor (Neuralin-1) (Ventroptin) (Neurogenesin-1).	ENST00000372045	Unknown	
ENST000003728	Chromsome 20 open reading frame 119 (Fragment).	ENST00000372821	Unknown	
ENST000003749	Spindlin-3 (Spindlin-like protein 3) (SPIN-3).	ENST00000374919	Unknown	
ENST000003771	cutaneous T-cell lymphoma tumor antigen se70-2	ENST00000377131	Unknown	
ENST000003771	cutaneous T-cell lymphoma tumor antigen se70-2	ENST00000377156	Unknown	
ENST000003777	t-SNARE domain-containing protein 1.	ENST00000377711	Unknown	
ENST000003789	ADP-ribosylation factor-like 6 interacting protein 2	ENST00000378954	Unknown	
ENST000003799	RAS-responsive element-binding protein 1 (RREB-1) (Raf-responsive zinc finger protein LZ321).	ENST00000379938	Unknown	
EP300	E1A binding protein p300 (EP300), mRNA	NM_001429	Hs.517517	U01877
EP300	E1A binding protein p300 (EP300), mRNA	NM_001429	Hs.517517	U01877
EP400	E1A binding protein p400 (EP400), mRNA	NM_015409	Hs.699245	NM_015409
EPB41L4B	cDNA: FLJ21596 fis, clone COL07110.	AK025249	Unknown	
EPB41L4B	erythrocyte membrane protein band 4.1 like 4B (EPB41L4B), transcript variant 1, mRNA	NM_018424	Hs.591901	AF153416
EPHB6	EPH receptor B6 (EPHB6), mRNA	NM_004445	Hs.380089	BC051028
ERBB3	v-erb-b2 erythroblastic leukemia viral oncogene homolog 3 (avian) (ERBB3), transcript variant 1, mRNA	NM_001982	Hs.118681	NM_001982
ERC1	ELKS/RAB6-interacting/CAST family member 1 (ERC1), transcript variant beta, mRNA	NM_178037	Hs.658200	NM_178037
ERH	enhancer of rudimentary homolog (Drosophila) (ERH), mRNA	NM_004450	Hs.509791	BI856529
ERVWE1	endogenous retroviral family W, env(C7), member 1 (syncytin) (ERVWE1), mRNA	NM_014590	Unknown	
ESCO1	establishment of cohesion 1 homolog 1 (S. cerevisiae) (ESCO1), mRNA	NM_052911	Hs.464733	AL832041
ESCO1	establishment of cohesion 1 homolog 1 (S. cerevisiae) (ESCO1), mRNA	NM_052911	Hs.464733	AL832041
ETAA1	Ewing's tumor-associated antigen 1 (ETAA1), mRNA	NM_019002	Hs.353022	AJ242682
EZH2	enhancer of zeste homolog 2 (Drosophila) (EZH2), transcript variant 1, mRNA	NM_004456	Hs.444082	AB208895
FAAH2	fatty acid amide hydrolase 2 (FAAH2), mRNA	NM_174912	Hs.496205	BC073922
FAM102B	family with sequence similarity 102, member B (FAM102B), mRNA	NM_001010883	Hs.200230	CR936815
FAM105A	family with sequence similarity 105, member A (FAM105A), mRNA	NM_019018	Hs.591751	AK001989
FAM117A	family with sequence similarity 117, member A (FAM117A), mRNA	NM_030802	Hs.514308	NM_030802
FAM122B	family with sequence similarity 122B (FAM122B), mRNA	NM_145284	Hs.404706	BC110846
FAM123B	family with sequence similarity 123B (FAM123B), mRNA	NM_152424	Hs.314225	NM_152424
FAM19A4	family with sequence similarity 19 (chemokine (C-C motif)-like), member A4 (FAM19A4), transcript variant 1, mRNA	NM_182522	Hs.187873	AY325117
FAM19A5	family with sequence similarity 19 (chemokine (C-C motif)-like), member A5 (FAM19A5), mRNA	NM_015381	Hs.632788	NM_015381
FAM29A	family with sequence similarity 29, member A (FAM29A), mRNA	NM_017645	Hs.533468	NM_017645
FAM29A	family with sequence similarity 29, member A (FAM29A), mRNA	NM_017645	Hs.533468	NM_017645
FAM29A	family with sequence similarity 29, member A (FAM29A), mRNA	NM_017645	Hs.533468	NM_017645
FAM35A	family with sequence similarity 35, member A (FAM35A), mRNA	NM_019054	Hs.500419	NM_019054
FAM44A	family with sequence similarity 44, member A (FAM44A), mRNA	NM_148894	Hs.444517	NM_148894
FAM44A	family with sequence similarity 44, member A (FAM44A), mRNA	NM_148894	Hs.444517	NM_148894
FAM44B	family with sequence similarity 44, member B (FAM44B), mRNA	NM_138369	Hs.425091	BM908302
FAM45A	family with sequence similarity 45, member A (FAM45A), mRNA	NM_207009	Hs.434241	AK027029
FAM49B	family with sequence similarity 49, member B (FAM49B), mRNA	NM_016623	Hs.126941	CR749628
FAM60A	family with sequence similarity 60, member A (FAM60A), mRNA	NM_021238	Hs.505154	BX648630
FAM76B	family with sequence similarity 76, member B (FAM76B), mRNA	NM_144664	Hs.288304	NM_144664
FAM77C	family with sequence similarity 77, member C (FAM77C), mRNA	NM_024522	Hs.470259	AK022712
FAM83B	family with sequence similarity 83, member B (FAM83B), mRNA	NM_001010872	Hs.657974	BC101628
FAM90A1	family with sequence similarity 90, member A1 (FAM90A1), mRNA	NM_018088	Hs.196086	BC042608
FAM90A10	FAM90A10	ENST00000382591	Unknown	
FAM96A	family with sequence similarity 96, member A (FAM96A), transcript variant 1, mRNA	NM_032231	Hs.439548	BM548195
FANCL	Fanconi anemia, complementation group L (FANCL), mRNA	NM_018062	Hs.699478	BC037570

FBXL12	F-box and leucine-rich repeat protein 12 (FBXL12), mRNA	NM_017703	Hs.12439	AK027004
FBXO30	F-box protein 30 (FBXO30), mRNA	NM_032145	Hs.421095	NM_032145
FBXO5	F-box protein 5 (FBXO5), mRNA	NM_012177	Hs.699374	AK055221
FEZF2	FEZ family zinc finger 2 (FEZF2), mRNA	NM_018008	Hs.241523	NM_018008
FGF19	fibroblast growth factor 19 (FGF19), mRNA	NM_005117	Hs.249200	NM_005117
FGFBP3	fibroblast growth factor binding protein 3 (FGFBP3), mRNA	NM_152429	Hs.591917	AK075410
FGFBP3	fibroblast growth factor binding protein 3 (FGFBP3), mRNA	NM_152429	Hs.591917	AK075410
FGFR1OP	C-C chemokine receptor type 6 (C-C CKR-6) (CC-CKR-6) (CCR-6) (LARC receptor) (GPR-CY4) (GPRCY4) (Chemokine receptor-like 3) (CKR-L3) (DRY6) (G-protein coupled receptor 29) (CD196 antigen).	ENST00000366847	Unknown	
FGFR1OP	FGFR1 oncogene partner (FGFR1OP), transcript variant 1, mRNA	NM_007045	Hs.487175	BC037785
FGFR2	fibroblast growth factor receptor 2 (bacteria-expressed kinase, keratinocyte growth factor receptor, craniofacial dysostosis 1, Crouzon syndrome, Pfeiffer syndrome, Jackson-Weiss syndrome) (FGFR2), transcript variant 2, mRNA	NM_022970	Hs.533683	NM_022970
FGFR3	fibroblast growth factor receptor 3 (achondroplasia, thanatophoric dwarfism) (FGFR3), transcript variant 1, mRNA	NM_000142	Hs.1420	AB209441
FIGN	fidgetin (FIGN), mRNA	NM_018086	Hs.593650	BX649105
FIGN	fidgetin (FIGN), mRNA	NM_018086	Hs.593650	BX649105
FKBP3	FK506 binding protein 3, 25kDa (FKBP3), mRNA	NM_002013	Hs.509226	BG112095
FLJ10213	hypothetical protein FLJ10213 (FLJ10213), mRNA	NM_018029	Hs.658858	BC051316
FLJ11021	similar to splicing factor, arginine/serine-rich 4 (FLJ11021), transcript variant 2, mRNA	NM_198261	Hs.432996	NM_198261
FLJ12716	FLJ12716 protein (FLJ12716), transcript variant 1, mRNA	NM_021942	Hs.443240	BX647127
FLJ12716	FLJ12716 protein (FLJ12716), transcript variant 1, mRNA	NM_021942	Hs.443240	BX647127
FLJ12986	cDNA FLJ12986 fis, clone NT2RP3000055.	AK023048	Hs.54713	XR_016325
FLJ13305	mRNA; cDNA DKFZp686O21143 (from clone DKFZp686O21143).	BX648834	Hs.440466	BX648834
FLJ14803	hypothetical protein FLJ14803 (FLJ14803), mRNA	NM_032842	Hs.267245	NM_032842
FLJ16542	FLJ16542 protein (FLJ16542), mRNA	NM_001004301	Hs.699590	BX647981
FLJ20273	RNA-binding protein (FLJ20273), mRNA	NM_019027	Hs.518727	NM_001098634
FLJ20464	cDNA FLJ20464 fis, clone KAT06158.	AK000471	Unknown	
FLJ21062	hypothetical protein FLJ21062 (FLJ21062), mRNA	NM_001039706	Hs.657403	AL833446
FLJ22795	colon cancer-associated antigen AgSK1-2HT-ECS mRNA, complete cds.	AF316855	Hs.498322	AF316855
FLJ23861	hypothetical protein FLJ23861 (FLJ23861), mRNA	NM_152519	Hs.591638	NM_152519
FLJ25006	hypothetical protein FLJ25006 (FLJ25006), mRNA	NM_144610	Hs.657973	BC045622
FLJ25770	hypothetical protein FLJ25770 (FLJ25770), transcript variant 2, mRNA	NM_178555	Unknown	
FLJ30428	cDNA FLJ30428 fis, clone BRACE2008941.	AK054990	Hs.131740	BC131487
FLJ32745	cDNA FLJ13088 fis, clone NT2RP3002102.	AK023150	Hs.362702	AK023150
FLJ32745	hypothetical protein FLJ32745 (FLJ32745), mRNA	NM_144978	Hs.362702	AK023150
FLJ32784	cDNA FLJ32784 fis, clone TESTI2002245.	AK057346	Unknown	
FLJ34077	weakly similar to zinc finger protein 195, mRNA (cDNA clone IMAGE:3606289), partial cds.	BC003519	Unknown	
FLJ35773	hypothetical protein FLJ35773 (FLJ35773), mRNA	NM_152599	Hs.213603	AY129026
FLJ35934	cDNA FLJ35934 fis, clone TESTI2011315.	AK093253	Hs.375092	AK093253
FLJ37953	hypothetical protein FLJ37953 (FLJ37953), mRNA	NM_001039693	Hs.204619	NM_001039693
FLJ39653	hypothetical protein FLJ39653 (FLJ39653), mRNA	NM_152684	Unknown	
FLJ39660	hypothetical protein FLJ39660, mRNA (cDNA clone IMAGE:4556987), complete cds.	BC062449	Hs.132519	NM_001080539
FLJ39660	mRNA; cDNA DKFZp434P055 (from clone DKFZp434P055).	AL834537	Unknown	
FLJ40473	cDNA FLJ40473 fis, clone TESTI2042806.	AK097792	Hs.603636	AK097792
FLJ40869	hypothetical protein FLJ40869, mRNA (cDNA clone IMAGE:4513298), with apparent retained intron.	BC035863	Hs.467793	CR936783
FLJ40869	hypothetical protein FLJ40869 (FLJ40869), mRNA	NM_182625	Hs.467793	CR936783
FLJ44451	clone Z'3-1 placenta expressed mRNA from chromosome X.	U66047	Hs.496916	U66047
FLJ44894	similar to zinc finger protein 91 (FLJ44894), mRNA	NM_001039884	Hs.631635	AK126842
FLJ45244	FLJ45244 protein (FLJ45244), mRNA	NM_207443	Unknown	
FLJ45445	FLJ45445 protein (FLJ45445), mRNA	NM_001004321	Unknown	
FLJ90709	hypothetical protein FLJ90709 (FLJ90709), mRNA	NM_173514	Hs.649685	BX641065
FLT3	fms-related tyrosine kinase 3 (FLT3), mRNA	NM_004119	Hs.507590	NM_004119

FLVCR	Feline leukemia virus subgroup C receptor-related protein 1 (Feline leukemia virus subgroup C receptor) (hFLVCR).	ENST00000366971	Unknown	
FNBP1L	formin binding protein 1-like (FNBP1L), transcript variant 1, mRNA	NM_001024948	Hs.134060	NM_001024948
FOXA2	forkhead box A2 (FOXA2), transcript variant 1, mRNA	NM_021784	Hs.155651	NM_021784
FOXA2	forkhead box A2 (FOXA2), transcript variant 1, mRNA	NM_021784	Hs.155651	NM_021784
FOXG1B	forkhead box G1B (FOXG1B), mRNA	NM_005249	Hs.695962	NM_005249
FOXG1B	forkhead box G1B (FOXG1B), mRNA	NM_005249	Hs.695962	NM_005249
FRAS1	mRNA; cDNA DKFZp761C1517 (from clone DKFZp761C1517).	AL831853	Hs.369448	NM_025074
FUBP3	far upstream element (FUSE) binding protein 3 (FUBP3), mRNA	NM_003934	Hs.98751	NM_003934
FUSIP1	FUS interacting protein (serine/arginine-rich) 1 (FUSIP1), transcript variant 1, mRNA	NM_006625	Hs.3530	AK125834
FUSIP1	FUS interacting protein (serine/arginine-rich) 1 (FUSIP1), transcript variant 2, mRNA	NM_054016	Hs.3530	AK125834
FZD3	Frizzled-3 precursor (Fz-3) (hFz3).	ENST00000380239	Unknown	
FZD3	frizzled homolog 3 (Drosophila) (FZD3), mRNA	NM_017412	Hs.40735	NM_017412
G36631	SHGC-53577 Human STS cDNA, sequence tagged site.	G36631	Unknown	
G3BP2	GTPase activating protein (SH3 domain) binding protein 2 (G3BP2), transcript variant 1, mRNA	NM_203505	Hs.303676	NM_203505
GALNT3	UDP-N-acetyl-alpha-D-galactosamine:polypeptide N-acetylgalactosaminyltransferase 3 (GalNAc-T3) (GALNT3), mRNA	NM_004482	Hs.170986	NM_004482
GARNL1	GTPase activating Rap/RanGAP domain-like 1 (GARNL1), transcript variant 2, mRNA	NM_194301	Hs.113150	AY596970
GATAD1	GATA zinc finger domain containing 1 (GATAD1), mRNA	NM_021167	Hs.21145	NM_021167
GATAD1	GATA zinc finger domain containing 1 (GATAD1), mRNA	NM_021167	Hs.21145	NM_021167
GCA	grancalcin, EF-hand calcium binding protein (GCA), mRNA	NM_012198	Hs.377894	BC005214
GCC2	GRIP and coiled-coil domain containing 2 (GCC2), transcript variant 1, mRNA	NM_181453	Hs.436505	NM_181453
GCC2	GRIP and coiled-coil domain containing 2 (GCC2), transcript variant 1, mRNA	NM_181453	Hs.436505	NM_181453
GCLC	glutamate-cysteine ligase, catalytic subunit (GCLC), mRNA	NM_001498	Hs.654465	AK094940
GCSH	glycine cleavage system protein H (aminomethyl carrier) (GCSH), mRNA	NM_004483	Hs.546256	BM474983
GHRL	ghrelin/obestatin preprohormone (GHRL), mRNA	NM_016362	Hs.590080	BX389908
GHRL	ghrelin/obestatin preprohormone (GHRL), mRNA	NM_016362	Hs.590080	BX389908
GHRL	ghrelin/obestatin preprohormone (GHRL), mRNA	NM_016362	Hs.590080	BX389908
GLCCI1	glucocorticoid induced transcript 1 (GLCCI1), mRNA	NM_138426	Hs.131673	NM_138426
GLCCI1	glucocorticoid induced transcript 1 (GLCCI1), mRNA	NM_138426	Hs.131673	NM_138426
GLDN	gliomedin (GLDN), mRNA	NM_181789	Hs.526441	NM_181789
GLI1	glioma-associated oncogene homolog 1 (zinc finger protein) (GLI1), mRNA	NM_005269	Hs.632702	NM_005269
GLI2	GLI-Kruppel family member GLI2 (GLI2), mRNA	NM_005270	Hs.111867	NM_005270
GLT1D1	glycosyltransferase 1 domain containing 1 (GLT1D1), mRNA	NM_144669	Hs.655668	BC043528
GLUL	glutamate-ammonia ligase (glutamine synthetase) (GLUL), transcript variant 1, mRNA	NM_002065	Hs.518525	BC051726
GLYATL1	glycine-N-acyltransferase-like 1 (GLYATL1), mRNA	NM_080661	Hs.38085	AK130017
GMNN	geminin, DNA replication inhibitor (GMNN), mRNA	NM_015895	Hs.234896	BQ064691
GNA13	guanine nucleotide binding protein (G protein), alpha 13 (GNA13), mRNA	NM_006572	Hs.515018	NM_006572
GNAL	guanine nucleotide binding protein (G protein), alpha activating activity polypeptide, olfactory type (GNAL), transcript variant 2, mRNA	NM_002071	Hs.136295	AK090868
GNG13	guanine nucleotide binding protein (G protein), gamma 13 (GNG13), mRNA	NM_016541	Hs.247888	AB030207
GNG4	guanine nucleotide binding protein (G protein), gamma 4 (GNG4), mRNA	NM_004485	Hs.159711	NM_001098722
GNGT1	guanine nucleotide binding protein (G protein), gamma transducing activity polypeptide 1 (GNGT1), mRNA	NM_021955	Hs.696174	AL571240
GNL2	guanine nucleotide binding protein-like 2 (nucleolar) (GNL2), mRNA	NM_013285	Hs.75528	AK130697
GOLGA1	cDNA FLJ11758 fis, clone HEMBA1005609, highly similar to mRNA; cDNA DKFZp564K133 (from clone DKFZp564K133).	AK021820	Hs.133469	U51587
GOLT1A	golgi transport 1 homolog A (S. cerevisiae) (GOLT1A), mRNA	NM_198447	Hs.532401	AB075871
GPBP1	mRNA; cDNA DKFZp761C169 (from clone DKFZp761C169).	AL161991	Hs.444279	AL161991
GPBP1	GC-rich promoter binding protein 1 (GPBP1), mRNA	NM_022913	Hs.444279	AL161991
GPC2	glypican 2 (cerebroglycan) (GPC2), mRNA	NM_152742	Hs.211701	BC027972
GPLD1	glycosylphosphatidylinositol specific phospholipase D1 (GPLD1), transcript variant 1, mRNA	NM_001503	Hs.591810	NM_001503
GPLD1	glycosylphosphatidylinositol specific phospholipase D1 (GPLD1), transcript variant 2, mRNA	NM_177483	Hs.591810	NM_001503
GPR125	G protein-coupled receptor 125 (GPR125), mRNA	NM_145290	Hs.99195	NM_145290
GPR19	G protein-coupled receptor 19 (GPR19), mRNA	NM_006143	Hs.657862	AK096388

GPR23	G protein-coupled receptor 23 (GPR23), mRNA	NM_005296	Hs.522701	NM_005296
GPR98	G protein-coupled receptor 98 (GPR98), transcript variant 1, mRNA	NM_032119	Hs.591777	AF435925
GPR98	mRNA; cDNA DKFZp761P0710 (from clone DKFZp761P0710).	AL136541	Hs.591777	AF435925
GPRC5C	cDNA FLJ20242 fis, clone COLF6369.	AK000249	Hs.446438	AK131210
GRHL2	grainyhead-like 2 (Drosophila) (GRHL2), mRNA	NM_024915	Hs.661088	AK023844
GRM3	glutamate receptor, metabotropic 3 (GRM3), mRNA	NM_000840	Hs.590575	BC041407
GRP	gastrin-releasing peptide (GRP), transcript variant 1, mRNA	NM_002091	Hs.153444	NM_002091
GSPT2	G1 to S phase transition 2 (GSPT2), mRNA	NM_018094	Hs.59523	BC036077
GSTA3	glutathione S-transferase A3 (GSTA3), mRNA	NM_000847	Hs.102484	NM_000847
GTF2A1	cDNA FLJ11579 fis, clone HEMBA1003579.	AK021641	Hs.593630	BC037828
GTF2F2	general transcription factor IIF, polypeptide 2 (30kD subunit) (GTF2F2), mRNA	NM_004128	Hs.654582	CR609785
GTF2F2	general transcription factor IIF, polypeptide 2 (30kD subunit) (GTF2F2), mRNA	NM_004128	Hs.654582	CR609785
GTF2I	general transcription factor II, i (GTF2I), transcript variant 1, mRNA	NM_032999	Hs.647041	NM_032999
GTF2I	general transcription factor II, i (GTF2I), transcript variant 1, mRNA	NM_032999	Hs.647041	NM_032999
GTF2I	general transcription factor II, i (GTF2I), transcript variant 1, mRNA	NM_032999	Hs.647041	NM_032999
GTF2I	general transcription factor II, i (GTF2I), transcript variant 1, mRNA	NM_032999	Hs.647041	NM_032999
GTF2IRD1	GTF2I repeat domain containing 1 (GTF2IRD1), transcript variant 2, mRNA	NM_005685	Hs.647056	AB209389
GTF3C2	general transcription factor IIIC, polypeptide 2, beta 110kDa (GTF3C2), transcript variant 1, mRNA	NM_001521	Hs.75782	NM_001521
GTPBP4	Nucleolar GTP-binding protein 1 (Chronic renal failure gene protein) (GTP-binding protein NGB).	ENST00000381391	Unknown	
GTPBP9	GTP-binding protein 9 (putative) (GTPBP9), transcript variant 1, mRNA	NM_013341	Hs.157351	NM_013341
GUCY2C	guanylate cyclase 2C (heat stable enterotoxin receptor) (GUCY2C), mRNA	NM_004963	Hs.524278	S57551
GULP1	GULP, engulfment adaptor PTB domain containing 1 (GULP1), mRNA	NM_016315	Hs.470887	AB209890
GUSBL2	glucuronidase, beta-like 2 (GUSBL2), mRNA	NM_206908	Unknown	
GUSBP1	glucuronidase, beta pseudogene 1 (GUSBP1), mRNA	NM_207331	Unknown	
GUSBP1	glucuronidase, beta pseudogene 1 (GUSBP1), mRNA	NM_207331	Unknown	
GYG2	glycogenin 2 (GYG2), transcript variant 2, mRNA	NM_003918	Hs.567381	NM_003918
H40632	H40632 yp50g01.s1 Soares retina N2b4HR cDNA clone IMAGE:190896 3', mRNA sequence	H40632	Hs.536655	BC041913
HACE1	HECT domain and ankyrin repeat containing, E3 ubiquitin protein ligase 1 (HACE1), mRNA	NM_020771	Hs.434340	AB037741
HAPLN4	hyaluronan and proteoglycan link protein 4 (HAPLN4), mRNA	NM_023002	Hs.367829	BC142698
HAT1	histone acetyltransferase 1 (HAT1), transcript variant 1, mRNA	NM_003642	Hs.632532	AK127840
HCG12	HLA complex group 12 (HCG12) on chromosome 6	NR_002831	Unknown	
HCG18	full-length cDNA clone CS0DI044YA04 of Placenta Cot 25-normalized of (human).	CR606587	Hs.485041	NM_003449
HDAC2	histone deacetylase 2 (HDAC2), mRNA	NM_001527	Hs.3352	NM_001527
HDGF	hepatoma-derived growth factor (high-mobility group protein 1-like) (HDGF), mRNA	NM_004494	Hs.506748	NM_004494
HDHD2	haloacid dehalogenase-like hydrolase domain containing 2 (HDHD2), mRNA	NM_032124	Hs.465041	BX537623
HEATR3	hypothetical protein mRNA, complete cds.	AF462442	Unknown	
HERC2P4	hect domain and RLD 2 pseudogene 4 (HERC2P4) on chromosome 16	NR_002827	Unknown	
HEXIM2	hexamthylene bis-acetamide inducible 2 (HEXIM2), mRNA	NM_144608	Hs.56382	AK056946
HIBCH	3-hydroxyisobutyryl-Coenzyme A hydrolase (HIBCH), transcript variant 1, mRNA	NM_014362	Hs.656685	AK124567
HIBCH	3-hydroxyisobutyryl-Coenzyme A hydrolase (HIBCH), transcript variant 1, mRNA	NM_014362	Hs.656685	AK124567
HIC2	hypermethylated in cancer 2 (HIC2), mRNA	NM_015094	Hs.632767	NM_015094
HIF3A	hypoxia inducible factor 3, alpha subunit (HIF3A), transcript variant 2, mRNA	NM_022462	Hs.420830	NM_022462
HIG2	hypoxia-inducible protein 2 (HIG2), mRNA	NM_013332	Hs.433213	NM_018396
HIP2	huntingtin interacting protein 2 (HIP2), mRNA	NM_005339	Hs.50308	AK093533
HIST1H1D	histone cluster 1, H1d (HIST1H1D), mRNA	NM_005320	Hs.136857	NM_005320
HIST1H3A	histone cluster 1, H3a (HIST1H3A), mRNA	NM_003529	Hs.546315	CD671279
HIST1H3B	histone cluster 1, H3b (HIST1H3B), mRNA	NM_003537	Hs.533292	DB042078
HIST1H3E	histone cluster 1, H3e (HIST1H3E), mRNA	NM_003532	Hs.443021	BE893548
HIST1H3F	histone cluster 1, H3f (HIST1H3F), mRNA	NM_021018	Hs.247814	EL736790
HIST1H3H	histone cluster 1, H3h (HIST1H3H), mRNA	NM_003536	Hs.591778	BQ072669
HIST1H4F	histone cluster 1, H4f (HIST1H4F), mRNA	NM_003540	Hs.247816	DA557005
HIVEP1	human immunodeficiency virus type I enhancer binding protein 1 (HIVEP1), mRNA	NM_002114	Hs.567284	X51435

HK1	hexokinase 1 (HK1), nuclear gene encoding mitochondrial protein, transcript variant 3, mRNA	NM_033497	Hs.695941	AK128226
HK2	hexokinase 2 (HK2), mRNA	NM_000189	Hs.591588	NM_000189
HKR1	GLI-Kruppel family member HKR1 (HKR1), mRNA	NM_181786	Hs.657027	AK128116
HLA-DOA	major histocompatibility complex, class II, DO alpha (HLA-DOA), mRNA	NM_002119	Hs.631991	NM_002119
HLA-DQB1	Human MHC class II HLA-DQ-beta (DR2-DQw1/DR4 DQw3) mRNA, complete cds, clone ROF2D.	M20432	Hs.409934	AK097297
HLA-DQB1	major histocompatibility complex, class II, DQ beta 1 (HLA-DQB1), mRNA	NM_002123	Hs.409934	AK097297
HLTF	helicase-like transcription factor (HLTF), transcript variant 1, mRNA	NM_003071	Hs.3068	NM_003071
HMG20A	high-mobility group 20A (HMG20A), mRNA	NM_018200	Hs.69594	NM_018200
HMG2L1	high-mobility group protein 2-like 1 (HMG2L1), transcript variant 1, mRNA	NM_005487	Hs.588815	NM_005487
HMGCR	3-hydroxy-3-methylglutaryl-Coenzyme A reductase (HMGCR), mRNA	NM_000859	Hs.699171	NM_000859
HMGCS1	3-hydroxy-3-methylglutaryl-Coenzyme A synthase 1 (soluble) (HMGCS1), mRNA	NM_002130	Hs.397729	BX537620
HN1L	hematological and neurological expressed 1-like (HN1L), mRNA	NM_144570	Hs.513261	NM_144570
HNRPA1	heterogeneous nuclear ribonucleoprotein A1 (HNRPA1), transcript variant 2, mRNA	NM_031157	Hs.699190	NM_031157
HNRPA1	heterogeneous nuclear ribonucleoprotein A1 (HNRPA1), transcript variant 2, mRNA	NM_031157	Hs.699190	NM_031157
HNRPA3	heterogeneous nuclear ribonucleoprotein A3 (HNRPA3), mRNA	NM_194247	Hs.516539	NM_194247
HNRPA3	heterogeneous nuclear ribonucleoprotein A3 (HNRPA3), mRNA	NM_194247	Hs.516539	NM_194247
HNRPDL	heterogeneous nuclear ribonucleoprotein D-like (HNRPDL), transcript variant 2, mRNA	NM_031372	Hs.527105	NM_031372
HNRPH1	heterogeneous nuclear ribonucleoprotein H1 (H) (HNRPH1), mRNA	NM_005520	Hs.604001	BX647205
HNRPH3	heterogeneous nuclear ribonucleoprotein H3 (2H9) (HNRPH3), transcript variant 2H9, mRNA	NM_012207	Hs.699337	BC039824
HNRPLL	Heterogeneous nuclear ribonucleoprotein L-like (Stromal RNA-regulating factor) (BLOCK24 protein).	ENST00000378915	Unknown	
HNRPM	heterogeneous nuclear ribonucleoprotein M (HNRPM), transcript variant 1, mRNA	NM_005968	Hs.465808	NM_005968
HNRPU	heterogeneous nuclear ribonucleoprotein U (scaffold attachment factor A) (HNRPU), transcript variant 1, mRNA	NM_031844	Hs.166463	NM_031844
HNRPU	heterogeneous nuclear ribonucleoprotein U (scaffold attachment factor A) (HNRPU), transcript variant 1, mRNA	NM_031844	Hs.166463	NM_031844
HOOK1	hook homolog 1 (Drosophila) (HOOK1), mRNA	NM_015888	Hs.378836	NM_015888
HOOK1	hook homolog 1 (Drosophila) (HOOK1), mRNA	NM_015888	Hs.378836	NM_015888
HOXB13	homeobox B13 (HOXB13), mRNA	NM_006361	Hs.66731	AY937237
HPGD	hydroxyprostaglandin dehydrogenase 15-(NAD) (HPGD), mRNA	NM_000860	Hs.655491	NM_000860
HPGD	hydroxyprostaglandin dehydrogenase 15-(NAD) (HPGD), mRNA	NM_000860	Hs.655491	NM_000860
HPN	hepsin (transmembrane protease, serine 1) (HPN), transcript variant 1, mRNA	NM_182983	Hs.182385	AK125670
HPN	hepsin (transmembrane protease, serine 1) (HPN), transcript variant 1, mRNA	NM_182983	Hs.182385	AK125670
HPRT1	hypoxanthine phosphoribosyltransferase 1 (Lesch-Nyhan syndrome) (HPRT1), mRNA	NM_000194	Hs.412707	NM_000194
HPRT1	hypoxanthine phosphoribosyltransferase 1 (Lesch-Nyhan syndrome) (HPRT1), mRNA	NM_000194	Hs.412707	NM_000194
HPRT1	hypoxanthine phosphoribosyltransferase 1 (Lesch-Nyhan syndrome) (HPRT1), mRNA	NM_000194	Hs.412707	NM_000194
HS3ST5	cDNA FLJ33755 fis, clone BRCAN2000523.	AK091074	Hs.645477	AK126930
HSD11B2	hydroxysteroid (11-beta) dehydrogenase 2 (HSD11B2), mRNA	NM_000196	Hs.1376	BC036780
HSD17B6	hydroxysteroid (17-beta) dehydrogenase 6 homolog (mouse) (HSD17B6), mRNA	NM_003725	Hs.524513	NM_003725
HSP90AA2	heat shock protein 90kDa alpha (cytosolic), class A member 2 (HSP90AA2), mRNA	NM_001040141	Unknown	
HSPA12A	cDNA FLJ13874 fis, clone THYRO1001365.	AK023936	Unknown	
HSPA14	heat shock 70kDa protein 14 (HSPA14), transcript variant 1, mRNA	NM_016299	Hs.534169	AK226065
HTR1E	5-hydroxytryptamine (serotonin) receptor 1E (HTR1E), mRNA	NM_000865	Hs.1611	NM_000865
IARS	isoleucine-tRNA synthetase (IARS), transcript variant long, mRNA	NM_013417	Hs.445403	NM_013417
ID1	inhibitor of DNA binding 1, dominant negative helix-loop-helix protein (ID1), transcript variant 1, mRNA	NM_002165	Hs.504609	BG165657
IDH1	isocitrate dehydrogenase 1 (NADP+), soluble (IDH1), mRNA	NM_005896	Hs.593422	AF020038
IGF1R	insulin-like growth factor 1 receptor (IGF1R), mRNA	NM_000875	Hs.643120	NM_000875
IGF2BP1	insulin-like growth factor 2 mRNA binding protein 1 (IGF2BP1), mRNA	NM_006546	Hs.144936	NM_006546
IGF2BP1	insulin-like growth factor 2 mRNA binding protein 1 (IGF2BP1), mRNA	NM_006546	Hs.144936	NM_006546
IGF2BP1	insulin-like growth factor 2 mRNA binding protein 1 (IGF2BP1), mRNA	NM_006546	Hs.144936	NM_006546
IGF2BP1	insulin-like growth factor 2 mRNA binding protein 1 (IGF2BP1), mRNA	NM_006546	Hs.144936	NM_006546
IGF2BP1	insulin-like growth factor 2 mRNA binding protein 1 (IGF2BP1), mRNA	NM_006546	Hs.144936	NM_006546
IGF2BP1	insulin-like growth factor 2 mRNA binding protein 1 (IGF2BP1), mRNA	NM_006546	Hs.144936	NM_006546
IGF2BP1	insulin-like growth factor 2 mRNA binding protein 1 (IGF2BP1), mRNA	NM_006546	Hs.144936	NM_006546

IGF2BP1	insulin-like growth factor 2 mRNA binding protein 1 (IGF2BP1), mRNA	NM_006546	Hs.144936	NM_006546
IGF2BP1	insulin-like growth factor 2 mRNA binding protein 1 (IGF2BP1), mRNA	NM_006546	Hs.144936	NM_006546
IGF2BP1	insulin-like growth factor 2 mRNA binding protein 1 (IGF2BP1), mRNA	NM_006546	Hs.144936	NM_006546
IGF2BP2	insulin-like growth factor 2 mRNA binding protein 2 (IGF2BP2), transcript variant 1, mRNA	NM_006548	Hs.35354	NM_006548
IGFBPL1	insulin-like growth factor binding protein-like 1 (IGFBPL1), mRNA	NM_001007563	Hs.349705	AK131521
IHPK2	inositol hexaphosphate kinase 2 (IHPK2), transcript variant 3, mRNA	NM_001005910	Hs.595983	BX537544
IHPK2	inositol hexaphosphate kinase 2 (IHPK2), transcript variant 3, mRNA	NM_001005910	Hs.595983	BX537544
IKBKAP	inhibitor of kappa light polypeptide gene enhancer in B-cells, kinase complex-associated protein (IKBKAP), mRNA	NM_003640	Hs.494738	AF153419
IKZF1	PRO0758 mRNA, complete cds.	AF116605	Unknown	
IKZF1	IKAROS family zinc finger 1 (Ikaros) (IKZF1), mRNA	NM_006060	Hs.488251	NM_006060
IL17RB	interleukin 17 receptor B (IL17RB), mRNA	NM_018725	Hs.654970	NM_018725
IL2RB	interleukin 2 receptor, beta (IL2RB), mRNA	NM_000878	Hs.474787	NM_000878
INADL	InaD-like (Drosophila) (INADL), mRNA	NM_176877	Hs.478125	NM_176877
INADL	InaD-like (Drosophila) (INADL), mRNA	NM_176877	Hs.478125	NM_176877
ING1	inhibitor of growth family, member 1 (ING1), transcript variant 1, mRNA	NM_198219	Hs.46700	NM_198219
ING2	inhibitor of growth family, member 2 (ING2), mRNA	NM_001564	Hs.107153	NM_001564
ING5	inhibitor of growth family, member 5 (ING5), mRNA	NM_032329	Hs.645460	NM_032329
INTS3	integrator complex subunit 3 (INTS3), mRNA	NM_023015	Hs.516522	CR749376
INTS6	integrator complex subunit 6 (INTS6), transcript variant 1, mRNA	NM_012141	Hs.439440	NM_012141
INTU	inturned planar cell polarity effector homolog (Drosophila) (INTU), mRNA	NM_015693	Hs.391481	BC051698
IQCB1	IQ motif containing B1 (IQCB1), transcript variant 3, mRNA	NM_001023571	Hs.604110	D25278
IQGAP2	IQ motif containing GTPase activating protein 2 (IQGAP2), mRNA	NM_006633	Hs.291030	NM_006633
IRX2	Iroquois-class homeodomain protein IRX-2 (Iroquois homeobox protein 2) (Homeodomain protein IRXA2).	ENST00000382611	Unknown	
ITLN2	intelectin 2 (ITLN2), mRNA	NM_080878	Hs.385631	AY358905
JARID2	jumonji, AT rich interactive domain 2 (JARID2), mRNA	NM_004973	Hs.696068	NM_004973
JMJD1B	jumonji domain containing 1B (JMJD1B), mRNA	NM_016604	Hs.483486	NM_016604
JMJD1C	jumonji domain containing 1C (JMJD1C), transcript variant 2, mRNA	NM_004241	Hs.413416	NM_032776
JMJD1C	jumonji domain containing 1C (JMJD1C), transcript variant 1, mRNA	NM_032776	Hs.413416	NM_032776
JPH3	junctophilin 3, mRNA (cDNA clone IMAGE:3867947), complete cds.	BC008690	Hs.592068	AK126663
KATNAL2	mRNA; cDNA DKFZp667C165 (from clone DKFZp667C165).	AL512748	Hs.404137	NM_031303
KBTBD7	kelch repeat and BTB (POZ) domain containing 7 (KBTBD7), mRNA	NM_032138	Hs.63841	BC022033
KCNB2	potassium voltage-gated channel, Shab-related subfamily, member 2 (KCNB2), mRNA	NM_004770	Hs.661102	AF338730
KCND2	potassium voltage-gated channel, Shal-related subfamily, member 2 (KCND2), mRNA	NM_012281	Hs.654739	AB028967
KCND2	potassium voltage-gated channel, Shal-related subfamily, member 2 (KCND2), mRNA	NM_012281	Hs.654739	AB028967
KCNE1L	KCNE1-like (KCNE1L), mRNA	NM_012282	Hs.522753	AK223306
KCNE3	potassium voltage-gated channel, Isk-related family, member 3 (KCNE3), mRNA	NM_005472	Hs.523899	NM_005472
KCNG1	potassium voltage-gated channel, subfamily G, member 1 (KCNG1), transcript variant 1, mRNA	NM_002237	Hs.118695	AK128721
KCNH8	potassium voltage-gated channel, subfamily H (eag-related), member 8 (KCNH8), mRNA	NM_144633	Hs.475656	AY053503
KCNK1	potassium channel, subfamily K, member 1 (KCNK1), mRNA	NM_002245	Hs.208544	AK090902
KCNK5	potassium channel, subfamily K, member 5 (KCNK5), mRNA	NM_003740	Hs.444448	NM_003740
KCNN1	potassium intermediate/small conductance calcium-activated channel, subfamily N, member 1 (KCNN1), mRNA	NM_002248	Hs.158173	NM_002248
KCNQ2	potassium voltage-gated channel, KQT-like subfamily, member 2 (KCNQ2), transcript variant 5, mRNA	NM_172109	Hs.161851	NM_172107
KCNS3	potassium voltage-gated channel, delayed-rectifier, subfamily S, member 3 (KCNS3), mRNA	NM_002252	Hs.414489	AK225833
KCTD3	potassium channel tetramerisation domain containing 3 (KCTD3), mRNA	NM_016121	Hs.335139	NM_016121
KCTD6	potassium channel tetramerisation domain containing 6 (KCTD6), mRNA	NM_153331	Hs.13982	AK027572
KHDRBS2	KH domain containing, RNA binding, signal transduction associated 2 (KHDRBS2), mRNA	NM_152688	Hs.519794	BC034043
KIAA0240	KIAA0240 (KIAA0240), mRNA	NM_015349	Hs.658033	NM_015349
KIAA0286	KIAA0286 protein (KIAA0286), mRNA	NM_015257	Hs.591040	AL833311
KIAA0368	Proteasome-associated protein ECM29 homolog (Ecm29).	ENST00000338205	Unknown	
KIAA0460	KIAA0460 (KIAA0460), mRNA	NM_015203	Hs.213666	BX641025
KIAA0484	mRNA, chromosome 1 specific transcript KIAA0484.	AB007953	Unknown	

KIAA0528	KIAA0528 (KIAA0528), mRNA	NM_014802	Hs.271014	BX537485
KIAA0773	KIAA0773 gene product (KIAA0773), mRNA	NM_014690	Hs.648908	AL832579
KIAA0947	mRNA for KIAA0947 protein, partial cds.	AB023164	Hs.449296	NM_015325
KIAA1012	KIAA1012 (KIAA1012), mRNA	NM_014939	Hs.202001	NM_014939
KIAA1086	mRNA for KIAA1086 protein, partial cds.	ENST00000262961	Unknown	
KIAA1155	mRNA for KIAA1155 protein, partial cds.	ENST00000244221	Unknown	
KIAA1166	KIAA1166 (KIAA1166), mRNA	NM_018684	Hs.28249	AB032992
KIAA1212	KIAA1212 (KIAA1212), mRNA	NM_018084	Hs.292925	NM_018084
KIAA1212	KIAA1212 (KIAA1212), mRNA	NM_018084	Hs.292925	NM_018084
KIAA1212	KIAA1212 (KIAA1212), mRNA	NM_018084	Hs.292925	NM_018084
KIAA1212	KIAA1212 (KIAA1212), mRNA	NM_018084	Hs.292925	NM_018084
KIAA1212	KIAA1212 (KIAA1212), mRNA	NM_018084	Hs.292925	NM_018084
KIAA1212	KIAA1212 (KIAA1212), mRNA	NM_018084	Hs.292925	NM_018084
KIAA1212	KIAA1212 (KIAA1212), mRNA	NM_018084	Hs.292925	NM_018084
KIAA1212	KIAA1212 (KIAA1212), mRNA	NM_018084	Hs.292925	NM_018084
KIAA1212	KIAA1212 (KIAA1212), mRNA	NM_018084	Hs.292925	NM_018084
KIAA1212	KIAA1212 (KIAA1212), mRNA	NM_018084	Hs.292925	NM_018084
KIAA1212	KIAA1212 (KIAA1212), mRNA	NM_018084	Hs.292925	NM_018084
KIAA1212	KIAA1212 (KIAA1212), mRNA	NM_018084	Hs.292925	NM_018084
KIAA1217	KIAA1217 (KIAA1217), mRNA	NM_019590	Hs.445885	NM_019590
KIAA1267	KIAA1267 (KIAA1267), mRNA	NM_015443	Hs.648744	BX538006
KIAA1344	KIAA1344 (KIAA1344), mRNA	NM_020784	Hs.532609	BC142650
KIAA1409	KIAA1409 (KIAA1409), mRNA	NM_020818	Hs.126561	NM_020818
KIAA1411	KIAA1411 (KIAA1411), mRNA	NM_020819	Hs.211700	AB037832
KIAA1411	KIAA1411 (KIAA1411), mRNA	NM_020819	Hs.211700	AB037832
KIAA1411	KIAA1411 (KIAA1411), mRNA	NM_020819	Hs.211700	AB037832
KIAA1430	mRNA for KIAA1430 protein, partial cds.	AB037851	Hs.535734	NM_020827
KIAA1468	KIAA1468 (KIAA1468), mRNA	NM_020854	Hs.465323	NM_020854
KIAA1524	mRNA for KIAA1524 protein, partial cds.	AB040957	Hs.591308	AB040957
KIAA1543	KIAA1543, mRNA (cDNA clone IMAGE:5745021), with apparent retained intron.	BC035808	Hs.17686	NM_001080429
KIAA1604	KIAA1604 protein (KIAA1604), mRNA	NM_020943	Hs.311363	NM_020943
KIAA1622	KIAA1622 (KIAA1622), transcript variant 1, mRNA	NM_058237	Hs.259599	NM_058237
KIAA1688	KIAA1688 protein (KIAA1688), mRNA	NM_025251	Hs.148988	NM_025251
KIAA1718	KIAA1718 protein (KIAA1718), mRNA	NM_030647	Hs.308710	NM_030647
KIAA1727	KIAA1727 protein (KIAA1727), mRNA	NM_033393	Hs.132629	NM_033393
KIAA1804	mixed lineage kinase 4 (KIAA1804), mRNA	NM_032435	Hs.547779	NM_032435
KIAA1922	KIAA1922 protein, mRNA (cDNA clone IMAGE:2960715), complete cds.	BC033082	Hs.597881	AB067509
KIAA1958	KIAA1958 (KIAA1958), mRNA	ENST00000337530	Unknown	
KIF13A	cDNA: FLJ21650 fis, clone COL08516.	AK025303	Hs.189915	AY014403
KIF26A	kinesin family member 26A, mRNA (cDNA clone IMAGE:3502885), complete cds.	BC009415	Hs.134970	NM_015656
KIF27	kinesin family member 27 (KIF27), mRNA	NM_017576	Hs.697514	AY237536
KIF2A	kinesin heavy chain member 2A (KIF2A), mRNA	NM_004520	Hs.558351	BC047544
KIF5C	kinesin family member 5C (KIF5C), mRNA	NM_004522	Hs.660699	NM_004522
KIN	KIN, antigenic determinant of recA protein homolog (mouse) (KIN), mRNA	NM_012311	Hs.397918	BC037913
KIRREL2	kin of IRRE like 2 (Drosophila) (KIRREL2), transcript variant 3, mRNA	NM_199180	Hs.145729	NM_199180
KIT	v-kit Hardy-Zuckerman 4 feline sarcoma viral oncogene homolog (KIT), mRNA	NM_000222	Hs.479754	NM_000222
KIT	v-kit Hardy-Zuckerman 4 feline sarcoma viral oncogene homolog (KIT), mRNA	NM_000222	Hs.479754	NM_000222
KIT	v-kit Hardy-Zuckerman 4 feline sarcoma viral oncogene homolog (KIT), mRNA	NM_000222	Hs.479754	NM_000222
KIT	v-kit Hardy-Zuckerman 4 feline sarcoma viral oncogene homolog (KIT), mRNA	NM_000222	Hs.479754	NM_000222
KIT	v-kit Hardy-Zuckerman 4 feline sarcoma viral oncogene homolog (KIT), mRNA	NM_000222	Hs.479754	NM_000222
KIT	v-kit Hardy-Zuckerman 4 feline sarcoma viral oncogene homolog (KIT), mRNA	NM_000222	Hs.479754	NM_000222
KIT	v-kit Hardy-Zuckerman 4 feline sarcoma viral oncogene homolog (KIT), mRNA	NM_000222	Hs.479754	NM_000222

KIT	v-kit Hardy-Zuckerman 4 feline sarcoma viral oncogene homolog (KIT), mRNA	NM_000222	Hs.479754	NM_000222
KIT	v-kit Hardy-Zuckerman 4 feline sarcoma viral oncogene homolog (KIT), mRNA	NM_000222	Hs.479754	NM_000222
KIT	v-kit Hardy-Zuckerman 4 feline sarcoma viral oncogene homolog (KIT), mRNA	NM_000222	Hs.479754	NM_000222
KLF8	Krueppel-like factor 8 (Basic krueppel-like factor 3) (Zinc finger protein 741).	ENST00000374929	Unknown	
KLHL14	kelch-like 14 (Drosophila) (KLHL14), mRNA	NM_020805	Hs.446164	AB037805
KLHL15	Kelch-like protein 15.	ENST00000328046	Unknown	
KLHL15	Kelch-like protein 15.	ENST00000328046	Unknown	
KLK8	kallikrein-related peptidase 8 (KLK8), transcript variant 2, mRNA	NM_144505	Hs.104570	DQ267420
Klkbl4	cDNA FLJ25339 fis, clone TST00959.	AK058068	Hs.411239	BC057843
KNTC1	kinetochore associated 1 (KNTC1), mRNA	NM_014708	Hs.300559	BC150278
KNTC1	kinetochore associated 1 (KNTC1), mRNA	NM_014708	Hs.300559	BC150278
KNTC1	kinetochore associated 1 (KNTC1), mRNA	NM_014708	Hs.300559	BC150278
KNTC1	kinetochore associated 1 (KNTC1), mRNA	NM_014708	Hs.300559	BC150278
KNTC1	kinetochore associated 1 (KNTC1), mRNA	NM_014708	Hs.300559	BC150278
KNTC1	kinetochore associated 1 (KNTC1), mRNA	NM_014708	Hs.300559	BC150278
KNTC1	kinetochore associated 1 (KNTC1), mRNA	NM_014708	Hs.300559	BC150278
KNTC1	kinetochore associated 1 (KNTC1), mRNA	NM_014708	Hs.300559	BC150278
KNTC1	kinetochore associated 1 (KNTC1), mRNA	NM_014708	Hs.300559	BC150278
KNTC1	kinetochore associated 1 (KNTC1), mRNA	NM_014708	Hs.300559	BC150278
KRAS	v-Ki-ras2 Kirsten rat sarcoma viral oncogene homolog (KRAS), transcript variant a, mRNA	NM_033360	Hs.505033	NM_033360
KRAS	v-Ki-ras2 Kirsten rat sarcoma viral oncogene homolog (KRAS), transcript variant a, mRNA	NM_033360	Hs.505033	NM_033360
KREMEN2	kringle containing transmembrane protein 2 (KREMEN2), transcript variant 4, mRNA	NM_172229	Hs.661128	BC009383
KRIT1	KRIT1, ankyrin repeat containing (KRIT1), transcript variant 4, mRNA	NM_194455	Hs.531987	AF388384
KRIT1	KRIT1, ankyrin repeat containing (KRIT1), transcript variant 4, mRNA	NM_194455	Hs.531987	AF388384
KRT25	keratin 25 (KRT25), mRNA	NM_181534	Hs.55412	AJ564204
LAMB4	laminin beta-4 chain precursor (LAMB4) mRNA, alternatively spliced short variant, partial cds.	AF029325	Unknown	
LARP7	La ribonucleoprotein domain family, member 7 (LARP7), transcript variant 1, mRNA	NM_016648	Hs.696461	BC066945
LCORL	ligand dependent nuclear receptor corepressor-like (LCORL), mRNA	NM_153686	Hs.446201	AL133031
LDHB	lactate dehydrogenase B (LDHB), mRNA	NM_002300	Hs.446149	AB209231
LEMD1	LEM domain containing 1 (LEMD1), mRNA	NM_001001552	Hs.655520	BC036636
LEMD3	LEM domain containing 3 (LEMD3), mRNA	NM_014319	Hs.505905	NM_014319
LEO1	Leo1, Paf1/RNA polymerase II complex component, homolog (S. cerevisiae) (LEO1), mRNA	NM_138792	Hs.567662	AY302186
LFNG	LFNG O-fucosylpeptide 3-beta-N-acetylglucosaminyltransferase (LFNG), transcript variant 1, mRNA	NM_001040167	Hs.159142	NM_001040167
LHFPL1	lipoma HMGIC fusion partner-like 1 (LHFPL1), mRNA	NM_178175	Hs.297420	NM_178175
LHPP	phospholysine phosphohistidine inorganic pyrophosphate phosphatase (LHPP), mRNA	NM_022126	Hs.527748	BC110344
LIN28B	lin-28 homolog B (C. elegans) (LIN28B), mRNA	NM_001004317	Hs.23616	NM_001004317
LIN28B	lin-28 homolog B (C. elegans) (LIN28B), mRNA	NM_001004317	Hs.23616	NM_001004317
LMO1	LIM domain only 1 (rhombotin 1) (LMO1), mRNA	NM_002315	Hs.654426	M26682
LOC120376	hypothetical protein LOC120376, mRNA (cDNA clone IMAGE:40030426), partial cds.	BC110079	Hs.31409	AL831866
LOC146429	mRNA; cDNA DKFZp434L1226 (from clone DKFZp434L1226).	AL137382	Hs.447544	XM_370997
LOC147645	Homo sapiens, clone IMAGE:4401841, mRNA.	BC016993	Hs.293236	XM_085831
LOC152217	hypothetical protein BC007882, mRNA (cDNA clone MGC:71256 IMAGE:6730791), complete cds.	BC062368	Hs.118820	AK123020
LOC200420	LOC200420 (LOC200420), mRNA	NM_145300	Unknown	
LOC221362	cDNA FLJ38040 fis, clone CTONG2013985.	AK095359	Hs.7921	AK091117
LOC221442	cDNA FLJ44104 fis, clone TESTI4044123.	AK126092	Hs.374076	BX537595
LOC257407	full length insert cDNA clone YZ88E12.	AF086098	Hs.551993	AF086098
LOC283970	hypothetical protein LOC283970, mRNA (cDNA clone MGC:71024 IMAGE:4403073), complete cds.	BC063633	Hs.513695	BX647358
LOC284033	cDNA FLJ37733 fis, clone BRHIP2020827.	AK095052	Hs.592124	AK095052
LOC284422	HSPC323 mRNA, partial cds.	ENST00000211092	Unknown	
LOC285548	cDNA FLJ25375 fis, clone TST01981.	AK058104	Hs.529284	BC048110
LOC285708	cDNA FLJ34759 fis, clone NT2NE2001874.	AK092078	Hs.533011	AK092078
LOC285908	hypothetical protein LOC285908 (LOC285908), mRNA	NM_181722	Hs.50755	NM_181722

LOC285908	hypothetical protein LOC285908 (LOC285908), mRNA	NM_181722	Hs.50755	NM_181722
LOC342892	mRNA; cDNA DKFZp686J154 (from clone DKFZp686J154).	CR627133	Hs.406307	CR627133
LOC348840	hypothetical protein LOC348840 (LOC348840), mRNA	NM_182631	Unknown	
LOC387787	PREDICTED: similar to CG9804-PA, transcript variant 1 (LOC387787), mRNA	XM_370636	Hs.591971	XM_370636
LOC388494	full-length cDNA clone CS0DF014YD20 of Fetal brain of (human).	CR593500	Hs.65750	NM_015174
LOC389634	cDNA FLJ90405 fis, clone NT2RP2006099.	AK074886	Hs.434403	AK074886
LOC389634	hypothetical LOC389634, mRNA (cDNA clone IMAGE:4157715).	BC037255	Hs.696467	AK124896
LOC389857	clone IMAGE:24166 hypothetical protein mRNA, partial cds.	AY730278	Hs.631771	AY730278
LOC399491	LOC399491 protein, mRNA (cDNA clone IMAGE:4941479), containing frame-shift errors.	BC048794	Hs.648395	AK131084
LOC399744	hypothetical LOC399744 (LOC399744), mRNA	NM_001013665	Unknown	
LOC399744	hypothetical LOC399744 (LOC399744), mRNA	NM_001013665	Unknown	
LOC399744	hypothetical LOC399744 (LOC399744), mRNA	NM_001013665	Unknown	
LOC399851	clone FP17388 unknown mRNA.	AY129010	Hs.652050	AY129010
LOC400590	hypothetical LOC400590, mRNA (cDNA clone MGC:70830 IMAGE:5248762), complete cds.	BC062632	Hs.558901	BC062632
LOC401357	hypothetical LOC401357 (LOC401357), mRNA	NM_001013685	Unknown	
LOC401357	hypothetical LOC401357 (LOC401357), mRNA	NM_001013685	Unknown	
LOC401357	hypothetical LOC401357 (LOC401357), mRNA	NM_001013685	Unknown	
LOC440350	similar to nuclear pore complex interacting protein (LOC440350), mRNA	NM_001018122	Hs.652266	XM_001132780
LOC440353	nuclear pore complex interacting protein pseudogene (LOC440353) on chromosome 16	NR_002603	Unknown	
LOC440353	nuclear pore complex interacting protein pseudogene (LOC440353) on chromosome 16	NR_002603	Unknown	
LOC440353	nuclear pore complex interacting protein pseudogene (LOC440353) on chromosome 16	NR_002603	Unknown	
LOC440353	nuclear pore complex interacting protein pseudogene (LOC440353) on chromosome 16	NR_002603	Unknown	
LOC441687	PREDICTED: similar to testis expressed gene 21 (LOC441687), mRNA	XR_018333	Hs.662369	XR_018333
LOC441748	AGENCOURT_6625160 NIH_MGC_114 cDNA clone IMAGE:5763013 5', mRNA sequence	BM925287	Hs.681787	BM925287
LOC442319	PREDICTED: similar to zinc finger protein 92 (HTF12) isoform 2 (LOC442319), mRNA	XR_016680	Hs.640774	XR_016680
LOC554175	hypothetical LOC554175, mRNA (cDNA clone MGC:11077 IMAGE:3688915), complete cds.	BC006530	Hs.646517	BC006530
LOC642005	PREDICTED: similar to zinc finger protein 682 (LOC642005), mRNA	XM_935785	Unknown	
LOC642123	cDNA FLJ46881 fis, clone UTERU3015647, moderately similar to Embigin precursor.	AK128714	Hs.697682	AK128714
LOC642123	cDNA FLJ46881 fis, clone UTERU3015647, moderately similar to Embigin precursor.	AK128714	Hs.697682	AK128714
LOC642652	PREDICTED: hypothetical LOC642652 (LOC642652), mRNA	XR_016196	Hs.646440	XR_016196
LOC643194	cDNA FLJ14635 fis, clone NT2RP2001196.	AK027541	Hs.660929	AK027541
LOC643313	cDNA clone IMAGE:4129277, partial cds.	BC006361	Hs.676949	BC006361
LOC643684	PREDICTED: hypothetical LOC643684 (LOC643684), mRNA	XM_931745	Hs.567958	XM_931745
LOC644186	PREDICTED: hypothetical protein LOC644186 (LOC644186), mRNA	XM_927380	Hs.535660	BX100292
LOC645249	hypothetical protein LOC645249, mRNA (cDNA clone IMAGE:4634276), partial cds.	BC022831	Hs.224879	XM_928291
LOC645676	cDNA FLJ44595 fis, clone BLADE2004849.	AK126559	Hs.701419	AK126559
LOC646090	PREDICTED: similar to rhophilin-like protein (LOC646090), mRNA	XR_017120	Hs.692717	XR_017120
LOC647057	PREDICTED: similar to carbonic anhydrase VA, mitochondrial precursor (LOC647057), mRNA	XR_017430	Hs.592035	XR_017430
LOC647211	PREDICTED: similar to rhophilin-like protein (LOC647211), mRNA	XR_017499	Hs.568203	XR_017499
LOC648232	mRNA, endogenous retrovirus.	AB128832	Hs.289232	AB128832
LOC648378	PREDICTED: similar to ribosomal protein S14 (LOC648378), mRNA	XR_018303	Hs.646683	XR_018303
LOC650177	PREDICTED: similar to sphingomyelinase, intestinal alkaline (LOC650177), mRNA	XR_018734	Hs.613123	XR_018734
LOC652720	cDNA FLJ44355 fis, clone TRACH3006699.	AK126329	Hs.36190	AK126329
LOC727867	similar to zinc finger protein 43 (HTF6) (LOC729863), mRNA	ENST00000305570	Unknown	
LOC728555	cDNA FLJ40901 fis, clone UTERU2003704.	AK098220	Hs.664334	AK098220
LOC728640	PREDICTED: hypothetical protein LOC728640 (LOC728640), mRNA	XR_015474	Hs.659642	XR_015400
LOC731058	PREDICTED: hypothetical protein LOC731058 (LOC731058), mRNA	XR_015778	Hs.646799	XR_015778
LOC731275	PREDICTED: hypothetical protein LOC731275 (LOC731275), mRNA	XR_015431	Hs.660700	XR_015431
LOC91431	prematurely terminated mRNA decay factor-like (LOC91431), mRNA	NM_138698	Unknown	
LOC91431	prematurely terminated mRNA decay factor-like (LOC91431), mRNA	NM_138698	Unknown	
LPHN1	latrophilin 1 (LPHN1), transcript variant 1, mRNA	NM_001008701	Hs.654658	NM_001008701
LRAT	lecithin retinol acyltransferase (phosphatidylcholine--retinol O-acyltransferase) (LRAT), mRNA	NM_004744	Hs.658427	AY546085

LRAT	lecithin retinol acyltransferase (phosphatidylcholine--retinol O-acyltransferase) (LRAT), mRNA	NM_004744	Hs.658427	AY546085
LRAT	lecithin retinol acyltransferase (phosphatidylcholine--retinol O-acyltransferase) (LRAT), mRNA	NM_004744	Hs.658427	AY546085
LRBA	LPS-responsive vesicle trafficking, beach and anchor containing (LRBA), mRNA	NM_006726	Hs.480938	NM_006726
LRGUK	leucine-rich repeats and guanylate kinase domain containing (LRGUK), mRNA	NM_144648	Hs.149774	AK057348
LRPPRC	leucine-rich PPR-motif containing (LRPPRC), mRNA	NM_133259	Hs.368084	AY289212
LRRC4	leucine rich repeat containing 4 (LRRC4), mRNA	NM_022143	Hs.655003	AF196976
LRRC40	leucine rich repeat containing 40 (LRRC40), mRNA	NM_017768	Hs.147836	AL390149
LRRC8D	leucine rich repeat containing 8 family, member D (LRRC8D), mRNA	NM_018103	Hs.482087	NM_018103
LRRCC1	leucine rich repeat and coiled-coil domain containing 1 (LRRCC1), transcript variant 1, mRNA	NM_033402	Hs.193115	AB051551
LRRCC1	leucine rich repeat and coiled-coil domain containing 1 (LRRCC1), transcript variant 1, mRNA	NM_033402	Hs.193115	AB051551
LRRIQ2	leucine-rich repeats and IQ motif containing 2 (LRRIQ2), mRNA	NM_024548	Hs.444135	AL833269
LSM14A	LSM14A, SCD6 homolog A (S. cerevisiae) (LSM14A), mRNA	NM_015578	Hs.407368	AK127401
LSM14A	LSM14A, SCD6 homolog A (S. cerevisiae) (LSM14A), mRNA	NM_015578	Hs.407368	AK127401
LSM3	LSM3 homolog, U6 small nuclear RNA associated (S. cerevisiae) (LSM3), mRNA	NM_014463	Hs.111632	BG329333
LSM3	LSM3 homolog, U6 small nuclear RNA associated (S. cerevisiae) (LSM3), mRNA	NM_014463	Hs.111632	BG329333
LSM7	LSM7 homolog, U6 small nuclear RNA associated (S. cerevisiae) (LSM7), mRNA	NM_016199	Hs.512610	AK127573
LTBP4	latent transforming growth factor beta binding protein 4 (LTBP4), transcript variant 1, mRNA	NM_001042544	Hs.466766	NM_001042544
LYCAT	lysocardiolipin acyltransferase (LYCAT), transcript variant 1, mRNA	NM_182551	Hs.468048	NM_182551
LYPD3	LY6/PLAUR domain containing 3 (LYPD3), mRNA	NM_014400	Hs.631594	AL832153
LYRM7	Lyrm7 homolog (mouse) (LYRM7), mRNA	NM_181705	Hs.115467	NM_181705
LYRM7	Lyrm7 homolog (mouse) (LYRM7), mRNA	NM_181705	Hs.115467	NM_181705
MAGEA12	melanoma antigen family A, 12 (MAGEA12), mRNA	NM_005367	Hs.169246	NM_005367
MAGEA2B	melanoma antigen family A, 2B (MAGEA2B), mRNA	NM_153488	Hs.670781	NM_175743
MAGEA9	melanoma antigen family A, 9 (MAGEA9), mRNA	NM_005365	Hs.512582	NM_005365
MAGEE1	melanoma antigen family E, 1 (MAGEE1), mRNA	NM_020932	Hs.8453	AB046807
MAGI2	membrane associated guanylate kinase, WW and PDZ domain containing 2 (MAGI2), mRNA	NM_012301	Hs.654788	NM_012301
MAL2	mal, T-cell differentiation protein 2 (MAL2), mRNA	NM_052886	Hs.201083	NM_052886
MALAT1	metastasis associated lung adenocarcinoma transcript 1 (non-coding RNA) (MALAT1) on chromosome 11	NR_002819	Unknown	
MAML1	mastermind-like 1 (Drosophila) (MAML1), mRNA	NM_014757	Hs.631951	AB209135
MAP3K15	mitogen-activated protein kinase kinase kinase 15 (MAP3K15), mRNA	NM_001001671	Hs.656028	NM_001001671
MAP3K7	mitogen-activated protein kinase kinase kinase 7 (MAP3K7), transcript variant A, mRNA	NM_003188	Hs.644143	BX648277
MAP7	microtubule-associated protein 7 (MAP7), mRNA	NM_003980	Hs.486548	NM_003980
MAP7	microtubule-associated protein 7 (MAP7), mRNA	NM_003980	Hs.486548	NM_003980
MAPK6	mitogen-activated protein kinase 6 (MAPK6), mRNA	NM_002748	Hs.411847	NM_002748
MAPK6	mitogen-activated protein kinase 6 (MAPK6), mRNA	NM_002748	Hs.411847	NM_002748
MARVELD3	MARVEL domain containing 3, mRNA (cDNA clone IMAGE:2820819), partial cds.	BC005052	Hs.513706	NM_001017967
MASTL	microtubule associated serine/threonine kinase-like (MASTL), mRNA	NM_032844	Hs.276905	AK123004
MAT2A	methionine adenosyltransferase II, alpha (MAT2A), mRNA	NM_005911	Hs.516157	NM_005911
MAT2A	methionine adenosyltransferase II, alpha (MAT2A), mRNA	NM_005911	Hs.516157	NM_005911
MATR3	matrin 3 (MATR3), transcript variant 1, mRNA	NM_199189	Hs.268939	CR936615
MBNL3	muscleblind-like 3 (Drosophila) (MBNL3), transcript variant R, mRNA	NM_133486	Hs.105134	NM_018388
MBTD1	mbt domain containing 1 (MBTD1), mRNA	NM_017643	Hs.652131	AK000062
MCF2L	full length insert cDNA clone ZD77E02.	AF086413	Hs.597691	CN430781
MCM3	MCM3 minichromosome maintenance deficient 3 (S. cerevisiae) (MCM3), mRNA	NM_002388	Hs.179565	NM_002388
MCM3AP	MCM3 minichromosome maintenance deficient 3 (S. cerevisiae) associated protein (MCM3AP), mRNA	NM_003906	Hs.389037	AJ010089
MCM3APAS	MCM3 minichromosome maintenance deficient 3 (S. cerevisiae) associated protein antisense (MCM3APAS) on chromosome 21	NR_002776	Unknown	
MCM3APAS	MCM3 minichromosome maintenance deficient 3 (S. cerevisiae) associated protein antisense (MCM3APAS) on chromosome 21	NR_002776	Unknown	
MCM6	minichromosome maintenance deficient 6 homolog (S. cerevisiae) (MCM6), mRNA	NM_005915	Hs.444118	NM_005915
MCM8	MCM8 minichromosome maintenance deficient 8 (S. cerevisiae) (MCM8), transcript variant 2, mRNA	NM_182802	Hs.631506	NM_182802
MCM8	MCM8 minichromosome maintenance deficient 8 (S. cerevisiae) (MCM8), transcript variant 2, mRNA	NM_182802	Hs.631506	NM_182802

MDC1	mediator of DNA damage checkpoint 1 (MDC1), mRNA	NM_014641	Hs.519993	NM_014641
MED4	mediator of RNA polymerase II transcription, subunit 4 homolog (S. cerevisiae) (MED4), mRNA	NM_014166	Hs.181112	AK001934
MEGF10	multiple EGF-like-domains 10 (MEGF10), mRNA	NM_032446	Hs.438709	CR749437
MESDC2	mesoderm development candidate 2 (MESDC2), mRNA	NM_015154	Hs.578450	NM_015154
METAP2	methionyl aminopeptidase 2 (METAP2), mRNA	NM_006838	Hs.591005	NM_006838
METTL9	methyltransferase like 9 (METTL9), transcript variant 1, mRNA	NM_016025	Hs.279583	NM_016025
METTL9	methyltransferase like 9 (METTL9), transcript variant 1, mRNA	NM_016025	Hs.279583	NM_016025
MFAP1	microfibrillar-associated protein 1 (MFAP1), mRNA	NM_005926	Hs.61418	NM_005926
MGA	CDNA FLJ13298 fis, clone OVARC1001306, weakly similar to N-MYC PROTO- ONCOGENE PROTEIN. (Fragment).	ENST00000310492	Unknown	
MGC11102	hypothetical protein MGC11102 (MGC11102), mRNA	NM_032325	Hs.425178	NM_032325
MGC12935	hypothetical protein MGC12935, mRNA (cDNA clone IMAGE:4309284), partial cds.	BC004565	Hs.247812	AW976335
MGC13379	HSPC244 (MGC13379), mRNA	NM_016499	Hs.26745	NM_016499
MGC15634	cDNA: FLJ22822 fis, clone KAIA3968.	AK026475	Hs.656372	AK026475
MGC15634	cDNA clone IMAGE:3344302.	BC007286	Hs.30323	BC007286
MGC17403	TFS2-M domain-containing protein 1.	ENST00000314720	Unknown	
MGC17403	cDNA FLJ39713 fis, clone SMINT2013170, weakly similar to TRANSCRIPTION FACTOR S-II-RELATED PROTEIN 4.	AK097032	Unknown	
MGC20983	hypothetical protein MGC20983 (MGC20983), mRNA	NM_145045	Hs.124010	BC036041
MGC21675	hypothetical protein MGC21675 (MGC21675), mRNA	NM_052861	Hs.561314	BC111466
MGC39715	hypothetical protein MGC39715 (MGC39715), mRNA	NM_152628	Hs.567758	AK127517
MGC39900	hypothetical protein MGC39900 (MGC39900), mRNA	NM_194324	Unknown	
MGC40405	hypothetical protein MGC40405 (MGC40405), transcript variant 2, mRNA	NM_001040057	Hs.489105	NM_001040057
MGC40405	hypothetical protein MGC40405 (MGC40405), transcript variant 2, mRNA	NM_001040057	Hs.489105	NM_001040057
MGC40405	hypothetical protein MGC40405 (MGC40405), transcript variant 2, mRNA	NM_001040057	Hs.489105	NM_001040057
MGC4562	hypothetical protein MGC4562 (MGC4562), mRNA	NM_133375	Hs.446251	BC022089
MGC5139	hypothetical protein MGC5139, mRNA (cDNA clone IMAGE:3448346), complete cds.	BC004815	Hs.127610	NM_001080533
MGEA5	meningioma expressed antigen 5 (hyaluronidase) (MGEA5), mRNA	NM_012215	Hs.500842	AF036144
MLF1IP	MLF1 interacting protein (MLF1IP), mRNA	NM_024629	Hs.575032	AF516710
MLLT10	myeloid/lymphoid or mixed-lineage leukemia (trithorax homolog, Drosophila); translocated to, 10 (MLLT10), transcript variant 1, mRNA	NM_004641	Hs.30385	AB209755
MLLT10	myeloid/lymphoid or mixed-lineage leukemia (trithorax homolog, Drosophila); translocated to, 10 (MLLT10), transcript variant 2, mRNA	NM_001009569	Hs.30385	AB209755
MLPH	melanophilin (MLPH), transcript variant 1, mRNA	NM_024101	Hs.102406	NM_024101
MLXIPL	MLX interacting protein-like (MLXIPL), transcript variant 1, mRNA	NM_032951	Hs.647055	NM_032951
MMD	monocyte to macrophage differentiation-associated (MMD), mRNA	NM_012329	Hs.463483	NM_012329
MND1	meiotic nuclear divisions 1 homolog (S. cerevisiae) (MND1), mRNA	NM_032117	Hs.294088	BG496847
MOBKL2B	MOB1, Mps One Binder kinase activator-like 2B (yeast) (MOBKL2B), mRNA	NM_024761	Hs.699322	NM_024761
MOBKL2B	MOB1, Mps One Binder kinase activator-like 2B (yeast) (MOBKL2B), mRNA	NM_024761	Hs.699322	NM_024761
MOGAT1	monoacylglycerol O-acyltransferase 1 (MOGAT1), mRNA	NM_058165	Hs.344090	NM_058165
MOSC1	MOCO sulphurase C-terminal domain containing 1 (MOSC1), mRNA	NM_022746	Hs.497816	AK092439
MPHOSPH10	M-phase phosphoprotein 10 (U3 small nucleolar ribonucleoprotein) (MPHOSPH10), mRNA	NM_005791	Hs.656208	NM_005791
MPV17L	MPV17 mitochondrial membrane protein-like (MPV17L), mRNA	NM_173803	Hs.401798	DQ004255
MRPL3	mitochondrial ribosomal protein L3 (MRPL3), nuclear gene encoding mitochondrial protein, mRNA	NM_007208	Hs.205163	BM541805
MRPL42	mitochondrial ribosomal protein L42 (MRPL42), nuclear gene encoding mitochondrial protein, transcript variant 3, mRNA	NM_172178	Hs.199579	CR749344
MRPL47	mitochondrial ribosomal protein L47 (MRPL47), nuclear gene encoding mitochondrial protein, transcript variant 1, mRNA	NM_020409	Hs.283734	BM455219
MRPL50	mitochondrial ribosomal protein L50	ENST00000374865	Unknown	
MRPL50	mitochondrial ribosomal protein L50 (MRPL50), nuclear gene encoding mitochondrial protein, mRNA	NM_019051	Hs.288224	BE893534
MRPS9	mitochondrial ribosomal protein S9 (MRPS9), nuclear gene encoding mitochondrial protein, mRNA	NM_182640	Hs.590900	NM_182640
MRS2L	MRS2-like, magnesium homeostasis factor (S. cerevisiae) (MRS2L), mRNA	NM_020662	Hs.533291	NM_020662
MSH2	mutS homolog 2, colon cancer, nonpolyposis type 1 (E. coli) (MSH2), mRNA	NM_000251	Hs.597656	AK223284
MSH2	mutS homolog 2, colon cancer, nonpolyposis type 1 (E. coli) (MSH2), mRNA	NM_000251	Hs.597656	AK223284
MSH2	mutS homolog 2, colon cancer, nonpolyposis type 1 (E. coli) (MSH2), mRNA	NM_000251	Hs.597656	AK223284
MSH2	mutS homolog 2, colon cancer, nonpolyposis type 1 (E. coli) (MSH2), mRNA	NM_000251	Hs.597656	AK223284

MSH2	mutS homolog 2, colon cancer, nonpolyposis type 1 (E. coli) (MSH2), mRNA	NM_000251	Hs.597656	AK223284
MSH2	mutS homolog 2, colon cancer, nonpolyposis type 1 (E. coli) (MSH2), mRNA	NM_000251	Hs.597656	AK223284
MSH2	mutS homolog 2, colon cancer, nonpolyposis type 1 (E. coli) (MSH2), mRNA	NM_000251	Hs.597656	AK223284
MSH2	mutS homolog 2, colon cancer, nonpolyposis type 1 (E. coli) (MSH2), mRNA	NM_000251	Hs.597656	AK223284
MSH2	mutS homolog 2, colon cancer, nonpolyposis type 1 (E. coli) (MSH2), mRNA	NM_000251	Hs.597656	AK223284
MSH2	mutS homolog 2, colon cancer, nonpolyposis type 1 (E. coli) (MSH2), mRNA	NM_000251	Hs.597656	AK223284
MSH6	mutS homolog 6 (E. coli) (MSH6), mRNA	NM_000179	Hs.445052	BC071594
MSL2L1	male-specific lethal 2-like 1 (Drosophila) (MSL2L1), mRNA	NM_018133	Hs.18631	NM_018133
MSTP9	macrophage stimulating, pseudogene 9 (MSTP9) on chromosome 1	NR_002729	Unknown	
MTAC2D1	membrane targeting (tandem) C2 domain containing 1 (MTAC2D1), mRNA	NM_152332	Hs.510262	BC040503
MTERF	mitochondrial transcription termination factor (MTERF), nuclear gene encoding mitochondrial protein, mRNA	NM_006980	Hs.532216	AL832861
MTERF	mitochondrial transcription termination factor (MTERF), nuclear gene encoding mitochondrial protein, mRNA	NM_006980	Hs.532216	AL832861
MTF2	metal response element binding transcription factor 2 (MTF2), mRNA	NM_007358	Hs.591449	AJ010014
MTHFD2L	methylenetetrahydrofolate dehydrogenase (NADP+ dependent) 2-like, mRNA (cDNA clone IMAGE:4794959), complete cds.	BC037529	Hs.479954	AK122799
MTMR15	myotubularin related protein 15 (MTMR15), mRNA	NM_014967	Hs.584863	CR936727
MTMR8	myotubularin related protein 8 (MTMR8), mRNA	NM_017677	Hs.442892	NM_017677
MUTYH	mutY homolog (E. coli) (MUTYH), transcript variant alpha1, mRNA	NM_012222	Hs.271353	NM_012222
MYB	v-myb myeloblastosis viral oncogene homolog (avian) (MYB), mRNA	NM_005375	Hs.654446	AJ606319
MYCL1	v-myc myelocytomatosis viral oncogene homolog 1, lung carcinoma derived (avian) (MYCL1), transcript variant 3, mRNA	NM_005376	Hs.437922	NM_001033081
MYCN	v-myc myelocytomatosis viral related oncogene, neuroblastoma derived (avian) (MYCN), mRNA	NM_005378	Hs.25960	NM_005378
MYO5B	myosin 5B (MYO5B) mRNA, complete cds.	AY274809	Hs.200136	NM_001080467
MYSM1	mRNA for KIAA1915 protein, partial cds.	AB067502	Hs.477495	AB067502
MYST4	MYST histone acetyltransferase (monocytic leukemia) 4 (MYST4), mRNA	NM_012330	Hs.35758	NM_012330
N34682	N34682 yy15g08.s1 Soares melanocyte 2NbHM cDNA clone IMAGE:271358 3' similar to contains Alu repetitive element;contains element MER12 repetitive element ;, mRNA sequence	N34682	Unknown	
N4BP2	Nedd4 binding protein 2 (N4BP2), mRNA	NM_018177	Hs.396494	AY267013
N4BP2	Nedd4 binding protein 2 (N4BP2), mRNA	NM_018177	Hs.396494	AY267013
NACA	nascent-polypeptide-associated complex alpha polypeptide (NACA), mRNA	NM_005594	Hs.505735	AK096699
NAG6	hypothetical protein DKFZp434G156 (NAG6), mRNA	NM_022742	Hs.521178	BC150331
NAP1L1	nucleosome assembly protein 1-like 1 (NAP1L1), transcript variant 1, mRNA	NM_139207	Hs.524599	AL162068
NAP1L2	nucleosome assembly protein 1-like 2 (NAP1L2), mRNA	NM_021963	Hs.66180	NM_021963
NARG1	NMDA receptor regulated 1 (NARG1), mRNA	NM_057175	Hs.555985	NM_057175
NARG1L	NMDA receptor regulated 1-like (NARG1L), transcript variant 1, mRNA	NM_024561	Hs.512914	CR627327
NASP	nuclear autoantigenic sperm protein (histone-binding) (NASP), transcript variant 1, mRNA	NM_172164	Hs.319334	AY700118
NASP	nuclear autoantigenic sperm protein (histone-binding) (NASP), transcript variant 1, mRNA	NM_172164	Hs.319334	AY700118
NAT8L	N-acetyltransferase 8-like (NAT8L), mRNA	NM_178557	Hs.318529	NM_178557
NDNL2	necdin-like 2 (NDNL2), mRNA	NM_138704	Hs.656412	AK074138
NEDD4L	neural precursor cell expressed, developmentally down-regulated 4-like (NEDD4L), mRNA	NM_015277	Hs.185677	AB007899
NEK3	NIMA (never in mitosis gene a)-related kinase 3 (NEK3), transcript variant 1, mRNA	NM_002498	Hs.409989	AK131359
NEK4	NIMA (never in mitosis gene a)-related kinase 4 (NEK4), mRNA	NM_003157	Hs.631921	NM_003157
NFE2	nuclear factor (erythroid-derived 2), 45kDa (NFE2), mRNA	NM_006163	Hs.75643	NM_006163
NFYB	nuclear transcription factor Y, beta (NFYB), mRNA	NM_006166	Hs.84928	NM_006166
NFYB	nuclear transcription factor Y, beta (NFYB), mRNA	NM_006166	Hs.84928	NM_006166
NGFRAP1	nerve growth factor receptor (TNFRSF16) associated protein 1 (NGFRAP1), transcript variant 3, mRNA	NM_014380	Hs.448588	CR593909
NGFRAP1L1	NGFRAP1-like 1 (NGFRAP1L1), mRNA	NM_001012978	Hs.47209	BM548306
NIF3L1	NIF3 NGG1 interacting factor 3-like 1 (S. pombe) (NIF3L1), mRNA	NM_021824	Hs.145284	AK127110
NIPBL	Nipped-B homolog (Drosophila) (NIPBL), transcript variant B, mRNA	NM_015384	Hs.481927	AJ627032
NIPBL	Nipped-B homolog (Drosophila) (NIPBL), transcript variant A, mRNA	NM_133433	Hs.481927	AJ627032
NIPBL	Nipped-B homolog (Drosophila) (NIPBL), transcript variant B, mRNA	NM_015384	Hs.481927	AJ627032
NIPSNAP1	nipsnap homolog 1 (C. elegans) (NIPSNAP1), mRNA	NM_003634	Hs.173878	NM_003634
NKTR	natural killer-tumor recognition sequence (NKTR), transcript variant 2, mRNA	NM_001012651	Unknown	

NLGN3	neuroligin 3 (NLGN3), mRNA	NM_018977	Hs.438877	NM_018977
NLGN4X	neuroligin 4, X-linked (NLGN4X), transcript variant 1, mRNA	NM_020742	Hs.21107	NM_020742
NLN	Neurolysin, mitochondrial precursor (EC 3.4.24.16) (Neurotensin endopeptidase) (Mitochondrial oligopeptidase M) (Microsomal endopeptidase) (MEP).	ENST00000380985	Unknown	
NLRP2	NLR family, pyrin domain containing 2 (NLRP2), mRNA	NM_017852	Hs.369279	BC039269
NMT1	N-myristoyltransferase 1 (NMT1), mRNA	NM_021079	Hs.532790	NM_021079
NMU	neuromedin U (NMU), mRNA	NM_006681	Hs.418367	BF034907
NO145	145 kDa nucleolar protein (NO145), mRNA	NM_001040274	Hs.95600	NM_001040274
NOL5A	nucleolar protein 5A (56kDa with KKE/D repeat) (NOL5A), mRNA	NM_006392	Hs.376064	BX641071
NOL8	nucleolar protein 8 (NOL8), mRNA	NM_017948	Hs.442199	CR627363
NOL8	nucleolar protein 8 (NOL8), mRNA	NM_017948	Hs.442199	CR627363
NOP5/NOP58	nucleolar protein NOP5/NOP58 (NOP5/NOP58), mRNA	NM_015934	Hs.471104	CR618609
NOS2A	nitric oxide synthase 2A (inducible, hepatocytes) (NOS2A), transcript variant 1, mRNA	NM_000625	Hs.700571	NM_000625
NOS2A	nitric oxide synthase 2A (inducible, hepatocytes) (NOS2A), transcript variant 1, mRNA	NM_000625	Hs.700571	NM_000625
NOS2A	nitric oxide synthase 2A (inducible, hepatocytes) (NOS2A), transcript variant 1, mRNA	NM_000625	Hs.700571	NM_000625
NOS2A	nitric oxide synthase 2A (inducible, hepatocytes) (NOS2A), transcript variant 1, mRNA	NM_000625	Hs.700571	NM_000625
NOS2A	nitric oxide synthase 2A (inducible, hepatocytes) (NOS2A), transcript variant 1, mRNA	NM_000625	Hs.700571	NM_000625
NOS2A	nitric oxide synthase 2A (inducible, hepatocytes) (NOS2A), transcript variant 1, mRNA	NM_000625	Hs.700571	NM_000625
NOS2A	nitric oxide synthase 2A (inducible, hepatocytes) (NOS2A), transcript variant 1, mRNA	NM_000625	Hs.700571	NM_000625
NOS2A	nitric oxide synthase 2A (inducible, hepatocytes) (NOS2A), transcript variant 1, mRNA	NM_000625	Hs.700571	NM_000625
NOS2A	nitric oxide synthase 2A (inducible, hepatocytes) (NOS2A), transcript variant 1, mRNA	NM_000625	Hs.700571	NM_000625
NOS2A	nitric oxide synthase 2A (inducible, hepatocytes) (NOS2A), transcript variant 1, mRNA	NM_000625	Hs.700571	NM_000625
NOX1	NADPH oxidase 1 (NOX1), transcript variant NOH-1Lv, mRNA	NM_013955	Hs.592227	AF127763
NP109438	GB|S77447.1|AAB34086.1 copper transporting P-type ATPase, ATP7B	NP109438	Unknown	
NPEPL1	cDNA FLJ42065 fis, clone SYNOV2006430.	AK124059	Hs.554211	AK124059
NPIP	nuclear pore complex interacting protein (NPIP), mRNA	NM_006985	Hs.694739	BM467683
NPIP	nuclear pore complex interacting protein (NPIP), mRNA	NM_006985	Hs.694739	BM467683
NPM1	nucleophosmin (nucleolar phosphoprotein B23, numatrin) (NPM1), transcript variant 1, mRNA	NM_002520	Hs.557550	BM541948
NPM1	nucleophosmin (nucleolar phosphoprotein B23, numatrin) (NPM1), transcript variant 1, mRNA	NM_002520	Hs.557550	BM541948
NPM1	nucleophosmin (nucleolar phosphoprotein B23, numatrin) (NPM1), transcript variant 1, mRNA	NM_002520	Hs.557550	BM541948
NPM2	nucleophosmin/nucleoplasmin, 2 (NPM2), mRNA	NM_182795	Hs.131055	AK127602
NPPC	natriuretic peptide precursor C (NPPC), mRNA	NM_024409	Hs.247916	BF976889
NR2C1	nuclear receptor subfamily 2, group C, member 1 (NR2C1), transcript variant 1, mRNA	NM_003297	Hs.108301	BC026074
NSBP1	nucleosomal binding protein 1 (NSBP1), mRNA	NM_030763	Hs.282204	AF250329
NSBP1	nucleosomal binding protein 1 (NSBP1), mRNA	NM_030763	Hs.282204	AF250329
NSBP1	nucleosomal binding protein 1 (NSBP1), mRNA	NM_030763	Hs.282204	AF250329
NSBP1	nucleosomal binding protein 1 (NSBP1), mRNA	NM_030763	Hs.282204	AF250329
NSBP1	nucleosomal binding protein 1 (NSBP1), mRNA	NM_030763	Hs.282204	AF250329
NSBP1	nucleosomal binding protein 1 (NSBP1), mRNA	NM_030763	Hs.282204	AF250329
NSBP1	nucleosomal binding protein 1 (NSBP1), mRNA	NM_030763	Hs.282204	AF250329
NSBP1	nucleosomal binding protein 1 (NSBP1), mRNA	NM_030763	Hs.282204	AF250329
NSBP1	nucleosomal binding protein 1 (NSBP1), mRNA	NM_030763	Hs.282204	AF250329
NSBP1	nucleosomal binding protein 1 (NSBP1), mRNA	NM_030763	Hs.282204	AF250329
NSUN6	NOL1/NOP2/Sun domain family, member 6 (NSUN6), mRNA	NM_182543	Hs.396175	BC033534
NSUN7	NOL1/NOP2/Sun domain family, member 7 (NSUN7), mRNA	NM_024677	Hs.590923	BC042401
NSUN7	NOL1/NOP2/Sun domain family, member 7 (NSUN7), mRNA	NM_024677	Hs.590923	BC042401
NT5C1B	5'-nucleotidase, cytosolic IB (NT5C1B), transcript variant 1, mRNA	NM_001002006	Hs.120319	NM_001002006
NT5DC1	5'-nucleotidase domain containing 1 (NT5DC1), mRNA	NM_152729	Hs.520341	NM_152729
NUCKS1	Nuclear ubiquitous casein and cyclin-dependent kinases substrate (P1).	ENST00000367142	Unknown	
NUDT10	nudix (nucleoside diphosphate linked moiety X)-type motif 10 (NUDT10), mRNA	NM_153183	Hs.375178	AF469196
NUDT11	nudix (nucleoside diphosphate linked moiety X)-type motif 11 (NUDT11), mRNA	NM_018159	Hs.200016	NM_018159
NUDT12	nudix (nucleoside diphosphate linked moiety X)-type motif 12 (NUDT12), mRNA	NM_031438	Hs.434289	NM_031438
NUDT21	nudix (nucleoside diphosphate linked moiety X)-type motif 21 (NUDT21), mRNA	NM_007006	Hs.528834	BX537360

NUFIP1	nuclear fragile X mental retardation protein interacting protein 1 (NUFIP1), mRNA	NM_012345	Hs.525006	NM_012345
NUP107	nucleoporin 107kDa (NUP107), mRNA	NM_020401	Hs.524574	NM_020401
NUP153	nucleoporin 153kDa (NUP153), mRNA	NM_005124	Hs.633334	AB210024
NUP50	nucleoporin 50kDa (NUP50), transcript variant 2, mRNA	NM_007172	Hs.475103	NM_007172
NUP50	nucleoporin 50kDa (NUP50), transcript variant 2, mRNA	NM_007172	Hs.475103	NM_007172
NUP54	nucleoporin 54kDa (NUP54), mRNA	NM_017426	Hs.430435	NM_017426
NUPL1	KIAA0410 mRNA, partial cds.	AB007870	Unknown	
NUS1	nuclear undecaprenyl pyrophosphate synthase 1 homolog (S. cerevisiae) (NUS1), mRNA	NM_138459	Hs.289008	NM_138459
NVL	nuclear VCP-like (NVL), transcript variant 1, mRNA	NM_002533	Hs.497867	NM_002533
NXF2	nuclear RNA export factor 2 (NXF2), transcript variant 1, mRNA	NM_017809	Hs.530358	BX647232
OBSCN	cDNA FLJ14124 fis, clone MAMMA1002498.	AK024186	Unknown	
OBSCN	obscurin, cytoskeletal calmodulin and titin-interacting RhoGEF (OBSCN), mRNA	NM_052843	Hs.656999	NM_001098623
OCLN	occludin (OCLN), mRNA	NM_002538	Hs.592605	NM_002538
OCLN	occludin (OCLN), mRNA	NM_002538	Hs.592605	NM_002538
OFD1	oral-facial-digital syndrome 1 (OFD1), mRNA	NM_003611	Hs.6483	Y16355
OLIG1	oligodendrocyte transcription factor 1 (OLIG1), mRNA	NM_138983	Hs.56663	BC033290
OPRM1	opioid receptor, mu 1 (OPRM1), transcript variant MOR-1A, mRNA	NM_001008504	Hs.2353	AY309009
OR51B2	olfactory receptor, family 51, subfamily B, member 2 (OR51B2), mRNA	NM_033180	Hs.680163	BC117461
ORC3L	origin recognition complex, subunit 3-like (yeast) (ORC3L), transcript variant 1, mRNA	NM_181837	Hs.410228	NM_181837
ORC4L	origin recognition complex, subunit 4-like (yeast) (ORC4L), transcript variant 2, mRNA	NM_002552	Hs.558364	NM_002552
OSBPL11	oxysterol binding protein-like 11 (OSBPL11), mRNA	NM_022776	Hs.477440	AF392454
OSGEPL1	O-sialoglycoprotein endopeptidase-like 1 (OSGEPL1), mRNA	NM_022353	Hs.60772	NM_022353
OSTalpha	organic solute transporter alpha (OSTalpha), mRNA	NM_152672	Hs.651164	AK172837
OSTalpha	organic solute transporter alpha (OSTalpha), mRNA	NM_152672	Hs.651164	AK172837
OSTbeta	organic solute transporter beta (OSTbeta), mRNA	NM_178859	Hs.534533	AK129601
OTUD3	mRNA for KIAA0459 protein, partial cds.	AB007928	Hs.374987	NM_015207
OVOL1	ovo-like 1(Drosophila) (OVOL1), mRNA	NM_004561	Hs.134434	BC059408
OVOL2	ovo-like 2 (Drosophila) (OVOL2), mRNA	NM_021220	Hs.697036	NM_021220
OVOS2	ovostatin 2, mRNA (cDNA clone IMAGE:4827636).	BC039117	Hs.524331	CR749395
OXGR1	oxoglutarate (alpha-ketoglutarate) receptor 1 (OXGR1), mRNA	NM_080818	Hs.352218	AK122639
OXGR1	oxoglutarate (alpha-ketoglutarate) receptor 1 (OXGR1), mRNA	NM_080818	Hs.352218	AK122639
P18SRP	P18SRP protein (P18SRP), mRNA	NM_173829	Hs.656180	NM_173829
P2RY1	purinergic receptor P2Y, G-protein coupled, 1 (P2RY1), mRNA	NM_002563	Hs.654526	NM_002563
P53AIP1	p53-regulated apoptosis-inducing protein 1 (P53AIP1), mRNA	NM_022112	Hs.160953	AB045832
PAGE4	P antigen family, member 4 (prostate associated) (PAGE4), mRNA	NM_007003	Hs.441038	CR601744
PAIP1	poly(A) binding protein interacting protein 1 (PAIP1), transcript variant 1, mRNA	NM_006451	Hs.482038	NM_006451
PAN3	PAN3 polyA specific ribonuclease subunit homolog (S. cerevisiae) (PAN3), mRNA	NM_175854	Hs.656549	NM_175854
PANK1	pantothenate kinase 1 (PANK1), transcript variant alpha, mRNA	NM_148977	Hs.376351	AK127777
PAP2D	phosphatidic acid phosphatase type 2 (PAP2D), transcript variant 2, mRNA	NM_001010861	Hs.483948	NM_001037317
PAPD5	PAP associated domain containing 5 (PAPD5), transcript variant 1, mRNA	NM_001040284	Hs.514342	NM_001040284
PAPOLA	poly(A) polymerase alpha (PAPOLA), mRNA	NM_032632	Hs.253726	NM_032632
PAPOLG	poly(A) polymerase gamma (PAPOLG), mRNA	NM_022894	Hs.387471	AB209304
PAQR3	progestin and adipoQ receptor family member III (PAQR3), mRNA	NM_001040202	Hs.657312	NM_001040202
PARG	poly (ADP-ribose) glycohydrolase (PARG), mRNA	NM_003631	Hs.536158	NM_003631
PARP1	poly (ADP-ribose) polymerase family, member 1 (PARP1), mRNA	NM_001618	Hs.177766	AK225654
PARP1	poly (ADP-ribose) polymerase family, member 1 (PARP1), mRNA	NM_001618	Hs.177766	AK225654
PARP1	poly (ADP-ribose) polymerase family, member 1 (PARP1), mRNA	NM_001618	Hs.177766	AK225654
PARP1	poly (ADP-ribose) polymerase family, member 1 (PARP1), mRNA	NM_001618	Hs.177766	AK225654
PARP1	poly (ADP-ribose) polymerase family, member 1 (PARP1), mRNA	NM_001618	Hs.177766	AK225654
PARP1	poly (ADP-ribose) polymerase family, member 1 (PARP1), mRNA	NM_001618	Hs.177766	AK225654
PARP1	poly (ADP-ribose) polymerase family, member 1 (PARP1), mRNA	NM_001618	Hs.177766	AK225654
PARP1	poly (ADP-ribose) polymerase family, member 1 (PARP1), mRNA	NM_001618	Hs.177766	AK225654

PARP1	poly (ADP-ribose) polymerase family, member 1 (PARP1), mRNA	NM_001618	Hs.177766	AK225654
PARP1	poly (ADP-ribose) polymerase family, member 1 (PARP1), mRNA	NM_001618	Hs.177766	AK225654
PATZ1	POZ (BTB) and AT hook containing zinc finger 1 (PATZ1), transcript variant 1, mRNA	NM_014323	Hs.517557	NM_014323
PAX7	paired box gene 7 (PAX7), transcript variant 2, mRNA	NM_013945	Hs.113253	NM_002584
PAXIP1	PAX interacting (with transcription-activation domain) protein 1 (PAXIP1), mRNA	NM_007349	Hs.443881	NM_007349
PCDH11Y	protocadherin 11 Y-linked (PCDH11Y), transcript variant c, mRNA	NM_032973	Hs.661308	NM_032973
PCDH11Y	protocadherin 11 Y-linked (PCDH11Y), transcript variant c, mRNA	NM_032973	Hs.661308	NM_032973
PCDHB13	protocadherin beta 13 (PCDHB13), mRNA	NM_018933	Hs.283803	AF217745
PCDHB2	protocadherin beta 2 (PCDHB2), mRNA	NM_018936	Hs.533023	BC098575
PCDHB2	protocadherin beta 2 (PCDHB2), mRNA	NM_018936	Hs.533023	BC098575
PCGF6	polycomb group ring finger 6 (PCGF6), transcript variant 1, mRNA	NM_001011663	Hs.335808	BC010235
PCSK4	proprotein convertase subtilisin/kexin type 4 (PCSK4), mRNA	NM_017573	Hs.46884	BC142942
PCSK9	proprotein convertase subtilisin/kexin type 9 (PCSK9), mRNA	NM_174936	Hs.18844	AK124635
PCYT1B	phosphate cytidylyltransferase 1, choline, beta (PCYT1B), mRNA	NM_004845	Hs.660708	AK226121
PCYT1B	phosphate cytidylyltransferase 1, choline, beta (PCYT1B), mRNA	NM_004845	Hs.660708	AK226121
PDCD6IP	programmed cell death 6 interacting protein (PDCD6IP), mRNA	NM_013374	Hs.475896	NM_013374
PDE3B	phosphodiesterase 3B, cGMP-inhibited (PDE3B), mRNA	NM_000922	Hs.656437	AB209326
PDE5A	phosphodiesterase 5A, cGMP-specific (PDE5A), transcript variant 1, mRNA	NM_001083	Hs.647971	NM_001083
PDE7A	phosphodiesterase 7A (PDE7A), transcript variant 1, mRNA	NM_002603	Hs.584788	AF332652
PDE7A	phosphodiesterase 7A (PDE7A), transcript variant 2, mRNA	NM_002604	Hs.584788	AF332652
PDE9A	phosphodiesterase 9A (PDE9A), transcript variant 1, mRNA	NM_002606	Hs.473927	AK127770
PDK1	pyruvate dehydrogenase kinase, isozyme 1 (PDK1), nuclear gene encoding mitochondrial protein, mRNA	NM_002610	Hs.470633	NM_002610
PDZK1	PDZ domain containing 1 (PDZK1), mRNA	NM_002614	Hs.444751	NM_002614
PELI1	pellino homolog 1 (Drosophila) (PELI1), mRNA	NM_020651	Hs.7886	AF302505
PELI2	pellino homolog 2 (Drosophila) (PELI2), mRNA	NM_021255	Hs.657926	NM_021255
PEX1	peroxisome biogenesis factor 1 (PEX1), mRNA	NM_000466	Hs.164682	NM_000466
PFKFB1	6-phosphofructo-2-kinase/fructose-2,6-biphosphatase 1 (PFKFB1), mRNA	NM_002625	Hs.444304	X52638
PGA3	pepsinogen 3, group I (pepsinogen A) (PGA3), mRNA	NM_001079807	Hs.647247	AL832946
PHF21B	PHD finger protein 21B (PHF21B), mRNA	NM_138415	Hs.254097	BC012187
PHF3	PHD finger protein 3 (PHF3), mRNA	NM_015153	Hs.348921	BX648268
PHF6	PHD finger protein 6 (PHF6), transcript variant 2, mRNA	NM_032458	Hs.356501	NM_032458
PHF8	PHD finger protein 8 (PHF8), mRNA	NM_015107	Hs.133352	CR933612
PHIP	pleckstrin homology domain interacting protein (PHIP), mRNA	NM_017934	Hs.511817	NM_017934
PHIP	pleckstrin homology domain interacting protein (PHIP), mRNA	NM_017934	Hs.511817	NM_017934
PHKA2	phosphorylase kinase, alpha 2 (liver) (PHKA2), mRNA	NM_000292	Hs.54941	NM_000292
PHLPPL	PH domain and leucine rich repeat protein phosphatase-like (PHLPPL), mRNA	NM_015020	Hs.531564	AB023148
PHOSPHO2	phosphatase, orphan 2 (PHOSPHO2), mRNA	NM_001008489	Hs.655150	BC010437
PIP5K3	phosphatidylinositol-3-phosphate/phosphatidylinositol 5-kinase, type III (PIP5K3), transcript variant 2, mRNA	NM_015040	Hs.173939	NM_015040
PITPNC1	phosphatidylinositol transfer protein, cytoplasmic 1 (PITPNC1), transcript variant 2, mRNA	NM_181671	Hs.591185	NM_181671
PLA2G2A	phospholipase A2, group IIA (platelets, synovial fluid) (PLA2G2A), mRNA	NM_000300	Hs.466804	CR609269
PLAG1	pleiomorphic adenoma gene 1 (PLAG1), mRNA	NM_002655	Hs.14968	NM_002655
PLCH1	phospholipase C, eta 1 (PLCH1), mRNA	NM_014996	Hs.567423	AY691170
PLCH1	phospholipase C, eta 1 (PLCH1), mRNA	NM_014996	Hs.567423	AY691170
PLCXD1	phosphatidylinositol-specific phospholipase C, X domain containing 1 (PLCXD1), mRNA	NM_018390	Hs.522568	AK091006
PLEKHH1	pleckstrin homology domain containing, family H (with MyTH4 domain) member 1 (PLEKHH1), mRNA	NM_020715	Hs.594236	NM_020715
PLEKHK1	pleckstrin homology domain containing, family K member 1 (PLEKHK1), mRNA	NM_145307	Hs.58559	NM_145307
PLEKHK1	pleckstrin homology domain containing, family K member 1 (PLEKHK1), mRNA	NM_145307	Hs.58559	NM_145307
PMS1	PMS1 postmeiotic segregation increased 1 (S. cerevisiae) (PMS1), mRNA	NM_000534	Hs.111749	CR749432
PNLIPRP2	pancreatic lipase-related protein 2 (PNLIPRP2), mRNA	NM_005396	Hs.423598	NM_005396
PNN	pinin, desmosome associated protein (PNN), mRNA	NM_002687	Hs.409965	NM_002687
POLA1	polymerase (DNA directed), alpha 1 (POLA1), mRNA	NM_016937	Hs.567319	NM_016937

POLD1	polymerase (DNA directed), delta 1, catalytic subunit 125kDa (POLD1), mRNA	NM_002691	Hs.279413	AB209560
POLD3	polymerase (DNA-directed), delta 3, accessory subunit (POLD3), mRNA	NM_006591	Hs.82502	NM_006591
POLI	polymerase (DNA directed) iota (POLI), mRNA	NM_007195	Hs.438533	NM_007195
POM121	POM121 membrane glycoprotein (rat) (POM121), mRNA	NM_172020	Hs.655217	NM_172020
POT1	POT1 protection of telomeres 1 homolog (S. pombe) (POT1), transcript variant 1, mRNA	NM_015450	Hs.31968	NM_015450
POTE14	protein expressed in prostate, ovary, testis, and placenta 14 (POTE14), transcript variant POTE-14A, mRNA	NM_001005356	Hs.684914	NM_001004053
POTE15	protein expressed in prostate, ovary, testis, and placenta 15 (POTE15), mRNA	NM_207355	Hs.558766	NM_207355
POTE2	protein expressed in prostate, ovary, testis, and placenta 2 (POTE2), transcript variant POTE-2D, mRNA	NM_001005359	Unknown	
POTE2	protein expressed in prostate, ovary, testis, and placenta 2 (POTE2), transcript variant POTE-2D, mRNA	NM_001005359	Unknown	
POTE2	protein expressed in prostate, ovary, testis, and placenta 2 (POTE2), transcript variant POTE-2B, mRNA	NM_001005364	Unknown	
POU2F1	POU domain, class 2, transcription factor 1 (Octamer-binding transcription factor 1) (Oct-1) (OTF-1) (NF-A1).	ENST00000367866	Unknown	
POU2F1	POU domain, class 2, transcription factor 1 (POU2F1), mRNA	NM_002697	Hs.493649	AK091438
PPAT	phosphoribosyl pyrophosphate amidotransferase (PPAT), mRNA	NM_002703	Hs.331420	AB209246
PPHLN1	periphilin 1 (PPHLN1), transcript variant 1, mRNA	NM_016488	Hs.444157	NM_016488
PPIG	peptidylprolyl isomerase G (cyclophilin G) (PPIG), mRNA	NM_004792	Hs.470544	U40763
PPIL3	peptidylprolyl isomerase (cyclophilin)-like 3 (PPIL3), transcript variant PPIL3c, mRNA	NM_131916	Hs.121076	NM_131916
PPM1E	protein phosphatase 1E (PP2C domain containing) (PPM1E), mRNA	NM_014906	Hs.245044	NM_014906
PPP1R9A	protein phosphatase 1, regulatory (inhibitor) subunit 9A (PPP1R9A), mRNA	NM_017650	Hs.21816	NM_017650
PPP1R9A	protein phosphatase 1, regulatory (inhibitor) subunit 9A (PPP1R9A), mRNA	NM_017650	Hs.21816	NM_017650
PPP2R5E	protein phosphatase 2, regulatory subunit B (B56), epsilon isoform (PPP2R5E), mRNA	NM_006246	Hs.334868	BC056156
PPP3R1	protein phosphatase 3 (formerly 2B), regulatory subunit B, 19kDa, alpha isoform (calcineurin B, type I) (PPP3R1), mRNA	NM_000945	Hs.280604	BC027913
PPP6C	Serine/threonine-protein phosphatase 6 (EC 3.1.3.16) (PP6).	ENST00000373547	Unknown	
PPWD1	peptidylprolyl isomerase domain and WD repeat containing 1 (PPWD1), mRNA	NM_015342	Hs.121432	AK093675
PRDM13	PR domain containing 13 (PRDM13), mRNA	NM_021620	Hs.287386	AY004253
PRIM1	primase, polypeptide 1, 49kDa (PRIM1), mRNA	NM_000946	Hs.534339	NM_000946
PRIM1	primase, polypeptide 1, 49kDa (PRIM1), mRNA	NM_000946	Hs.534339	NM_000946
PRIM1	primase, polypeptide 1, 49kDa (PRIM1), mRNA	NM_000946	Hs.534339	NM_000946
PRIM1	primase, polypeptide 1, 49kDa (PRIM1), mRNA	NM_000946	Hs.534339	NM_000946
PRIM1	primase, polypeptide 1, 49kDa (PRIM1), mRNA	NM_000946	Hs.534339	NM_000946
PRIM1	primase, polypeptide 1, 49kDa (PRIM1), mRNA	NM_000946	Hs.534339	NM_000946
PRIM1	primase, polypeptide 1, 49kDa (PRIM1), mRNA	NM_000946	Hs.534339	NM_000946
PRIM1	primase, polypeptide 1, 49kDa (PRIM1), mRNA	NM_000946	Hs.534339	NM_000946
PRIM1	primase, polypeptide 1, 49kDa (PRIM1), mRNA	NM_000946	Hs.534339	NM_000946
PRIM1	primase, polypeptide 1, 49kDa (PRIM1), mRNA	NM_000946	Hs.534339	NM_000946
PRKCB1	protein kinase C, beta 1 (PRKCB1), transcript variant 2, mRNA	NM_002738	Hs.460355	AL833252
PRMT6	protein arginine methyltransferase 6 (PRMT6), mRNA	NM_018137	Hs.26006	AY043278
PROCA1	proline-rich cyclin A1-interacting protein (PROCA1), mRNA	NM_152465	Hs.207471	AL137531
PROK2	prokineticin 2 (PROK2), mRNA	NM_021935	Hs.528665	AF333025
PROM1	prominin 1 (PROM1), mRNA	NM_006017	Hs.614734	AF117225
PRPF38A	PRP38 pre-mRNA processing factor 38 (yeast) domain containing A (PRPF38A), transcript variant 2, mRNA	NM_032864	Hs.5301	NM_032864
PRPF38A	PRP38 pre-mRNA processing factor 38 (yeast) domain containing A (PRPF38A), transcript variant 1, mRNA	NM_032284	Unknown	
PRPF38B	PRP38 pre-mRNA processing factor 38 (yeast) domain containing B (PRPF38B), mRNA	NM_018061	Hs.342307	AL833950
PRPF38B	PRP38 pre-mRNA processing factor 38 (yeast) domain containing B (PRPF38B), mRNA	NM_018061	Hs.342307	AL833950
PRPF39	PRP39 pre-mRNA processing factor 39 homolog (S. cerevisiae) (PRPF39), mRNA	NM_017922	Hs.274337	BC028683
PRPF40A	PRP40 pre-mRNA processing factor 40 homolog A (yeast), mRNA (cDNA clone IMAGE:4343048), partial cds.	BC027178	Hs.643580	NM_017892
PRPF4B	PRP4 pre-mRNA processing factor 4 homolog B (yeast) (PRPF4B), mRNA	NM_003913	Hs.159014	NM_003913
PRR15	proline rich 15 (PRR15), mRNA	NM_175887	Hs.91109	NM_175887
PRRT2	proline-rich transmembrane protein 2 (PRRT2), mRNA	NM_145239	Hs.655071	AK092265
PRSS16	protease, serine, 16 (thymus) (PRSS16), mRNA	NM_005865	Hs.274407	AK126160

PRTG	protogenin homolog (Gallus gallus) (PRTG), mRNA	NM_173814	Hs.130957	NM_173814
PSAT1	phosphoserine aminotransferase 1 (PSAT1), transcript variant 1, mRNA	NM_058179	Hs.494261	NM_058179
PSIP1	PC4 and SFRS1 interacting protein 1 (PSIP1), transcript variant 2, mRNA	NM_033222	Hs.658434	BX649155
PSMA2	proteasome (prosome, macropain) subunit, alpha type, 2 (PSMA2), mRNA	NM_002787	Hs.333786	BX641097
PSMA3	proteasome (prosome, macropain) subunit, alpha type, 3 (PSMA3), transcript variant 1, mRNA	NM_002788	Hs.558799	BM918616
PSMC6	proteasome (prosome, macropain) 26S subunit, ATPase, 6 (PSMC6), mRNA	NM_002806	Hs.156171	AK127456
PSMD12	26S proteasome non-ATPase regulatory subunit 12 (26S proteasome regulatory subunit p55).	ENST00000356126	Unknown	
PSPH	phosphoserine phosphatase (PSPH), mRNA	NM_004577	Hs.512656	BX537439
PTAR1	mRNA; cDNA DKFZp313P0917 (from clone DKFZp313P0917).	AL832683	Hs.700761	NM_001099666
PTBP2	polypyrimidine tract binding protein 2 (PTBP2), mRNA	NM_021190	Hs.591430	AB209266
PTCD1	pentatricopeptide repeat domain 1 (PTCD1), mRNA	NM_015545	Hs.632313	AB014532
PTPN13	protein tyrosine phosphatase, non-receptor type 13 (APO-1/CD95 (Fas)-associated phosphatase) (PTPN13), transcript variant 4, mRNA	NM_080685	Hs.436142	U12128
PTPN2	protein tyrosine phosphatase, non-receptor type 2 (PTPN2), transcript variant 2, mRNA	NM_080422	Hs.654527	AB209569
PTPRZ1	protein tyrosine phosphatase, receptor-type, Z polypeptide 1 (PTPRZ1), mRNA	NM_002851	Hs.489824	NM_002851
PUM2	pumilio homolog 2 (Drosophila) (PUM2), mRNA	NM_015317	Hs.467824	AF315591
PUM2	pumilio homolog 2 (Drosophila) (PUM2), mRNA	NM_015317	Hs.467824	AF315591
PVRL3	poliovirus receptor-related 3, mRNA (cDNA clone IMAGE:4066485), complete cds.	BC017572	Hs.293917	BC001336
PVRL3	poliovirus receptor-related 3, mRNA (cDNA clone IMAGE:4066485), complete cds.	BC017572	Hs.293917	BC001336
QRICH1	glutamine-rich 1 (QRICH1), transcript variant 1, mRNA	NM_017730	Hs.297389	NM_017730
R3HDM1	R3H domain containing 1 (R3HDM1), mRNA	NM_015361	Hs.412462	NM_015361
R78584	yi73c12.r1 Soares placenta Nb2HP cDNA clone IMAGE:144886 3', mRNA sequence	R78584	Hs.595453	R78584
RAB11FIP4	RAB11 family interacting protein 4 (class II) (RAB11FIP4), mRNA	NM_032932	Hs.406788	NM_032932
RAB11FIP4	RAB11 family interacting protein 4 (class II) (RAB11FIP4), mRNA	NM_032932	Hs.406788	NM_032932
RAB11FIP4	RAB11 family interacting protein 4 (class II) (RAB11FIP4), mRNA	NM_032932	Hs.406788	NM_032932
RAB25	RAB25, member RAS oncogene family (RAB25), mRNA	NM_020387	Hs.632469	BE612887
RABGAP1L	mRNA for KIAA0471 protein, partial cds.	AB007940	Hs.585378	AB007940
RABGGTB	Rab geranylgeranyltransferase, beta subunit (RABGGTB), mRNA	NM_004582	Hs.78948	AB209023
RABGGTB	Rab geranylgeranyltransferase, beta subunit (RABGGTB), mRNA	NM_004582	Hs.78948	AB209023
RAD17	RAD17 homolog (S. pombe) (RAD17), transcript variant 8, mRNA	NM_002873	Hs.16184	AF076838
RAD18	RAD18 homolog (S. cerevisiae) (RAD18), mRNA	NM_020165	Hs.375684	NM_020165
RAD21	RAD21 homolog (S. pombe) (RAD21), mRNA	NM_006265	Hs.81848	NM_006265
RAD23B	RAD23 homolog B (S. cerevisiae) (RAD23B), mRNA	NM_002874	Hs.521640	NM_002874
RAD50	RAD50 homolog (S. cerevisiae) (RAD50), transcript variant 1, mRNA	NM_005732	Hs.655835	U63139
RAD50	RAD50 homolog (S. cerevisiae) (RAD50), transcript variant 1, mRNA	NM_005732	Hs.655835	U63139
RAD50	RAD50 homolog (S. cerevisiae) (RAD50), transcript variant 1, mRNA	NM_005732	Hs.655835	U63139
RAD50	RAD50 homolog (S. cerevisiae) (RAD50), transcript variant 1, mRNA	NM_005732	Hs.655835	U63139
RAD50	RAD50 homolog (S. cerevisiae) (RAD50), transcript variant 1, mRNA	NM_005732	Hs.655835	U63139
RAD50	RAD50 homolog (S. cerevisiae) (RAD50), transcript variant 1, mRNA	NM_005732	Hs.655835	U63139
RAD50	RAD50 homolog (S. cerevisiae) (RAD50), transcript variant 1, mRNA	NM_005732	Hs.655835	U63139
RAD50	RAD50 homolog (S. cerevisiae) (RAD50), transcript variant 1, mRNA	NM_005732	Hs.655835	U63139
RAD50	RAD50 homolog (S. cerevisiae) (RAD50), transcript variant 1, mRNA	NM_005732	Hs.655835	U63139
RAD50	RAD50 homolog (S. cerevisiae) (RAD50), transcript variant 1, mRNA	NM_005732	Hs.655835	U63139
RAD54B	RAD54 homolog B (S. cerevisiae) (RAD54B), mRNA	NM_012415	Hs.30561	NM_012415
RAD54B	RAD54 homolog B (S. cerevisiae) (RAD54B), mRNA	NM_012415	Hs.30561	NM_012415
RAD54B	RAD54 homolog B (S. cerevisiae) (RAD54B), mRNA	NM_012415	Hs.30561	NM_012415
RAD54B	RAD54 homolog B (S. cerevisiae) (RAD54B), mRNA	NM_012415	Hs.30561	NM_012415
RAD54B	RAD54 homolog B (S. cerevisiae) (RAD54B), mRNA	NM_012415	Hs.30561	NM_012415
RAD54B	RAD54 homolog B (S. cerevisiae) (RAD54B), mRNA	NM_012415	Hs.30561	NM_012415
RAD54B	RAD54 homolog B (S. cerevisiae) (RAD54B), mRNA	NM_012415	Hs.30561	NM_012415
RAD54B	RAD54 homolog B (S. cerevisiae) (RAD54B), mRNA	NM_012415	Hs.30561	NM_012415
RAD54B	RAD54 homolog B (S. cerevisiae) (RAD54B), mRNA	NM_012415	Hs.30561	NM_012415
RAD54B	RAD54 homolog B (S. cerevisiae) (RAD54B), mRNA	NM_012415	Hs.30561	NM_012415

RAD54L	RAD54-like (S. cerevisiae) (RAD54L), mRNA	NM_003579	Hs.696005	NM_003579
RANBP2	RAN binding protein 2 (RANBP2), mRNA	NM_006267	Hs.590897	NM_006267
RANBP2	RAN binding protein 2 (RANBP2), mRNA	NM_006267	Hs.590897	NM_006267
RANBP5	RAN binding protein 5 (RANBP5), mRNA	NM_002271	Hs.699240	NM_002271
RAPGEF6	Rap guanine nucleotide exchange factor (GEF) 6 (RAPGEF6), mRNA	NM_016340	Hs.483329	AF478567
RASEF	cDNA FLJ31614 fis, clone NT2RI2002970.	AK056176	Hs.657750	NM_152573
RASGEF1A	RasGEF domain family, member 1A, mRNA (cDNA clone MGC:26821 IMAGE:4814750), complete cds.	BC022548	Hs.125293	AK127432
RAX	retina and anterior neural fold homeobox (RAX), mRNA	NM_013435	Hs.278957	NM_013435
RB1CC1	RB1-inducible coiled-coil 1 (RB1CC1), mRNA	NM_014781	Hs.196102	NM_014781
RBAK	RB-associated KRAB zinc finger (RBAK), mRNA	NM_021163	Hs.592827	NM_021163
RBAK	RB-associated KRAB zinc finger (RBAK), mRNA	NM_021163	Hs.592827	NM_021163
RBBP6	retinoblastoma binding protein 6 (RBBP6), transcript variant 1, mRNA	NM_006910	Hs.188553	NM_006910
RBBP8	retinoblastoma binding protein 8 (RBBP8), transcript variant 1, mRNA	NM_002894	Hs.546282	NM_002894
RBM11	RNA binding motif protein 11 (RBM11), mRNA	NM_144770	Hs.283828	BC030196
RBM12	RNA binding motif protein 12 (RBM12), transcript variant 1, mRNA	NM_006047	Hs.246413	NM_006047
RBM12B	RNA binding motif protein 12B (RBM12B), mRNA	NM_203390	Hs.192788	NM_203390
RBM16	RNA binding motif protein 16 (RBM16), mRNA	NM_014892	Hs.591329	NM_014892
RBM16	RNA binding motif protein 16 (RBM16), mRNA	NM_014892	Hs.591329	NM_014892
RBM25	RNA binding motif protein 25 (RBM25), mRNA	NM_021239	Hs.531106	BX647116
RBM25	RNA binding motif protein 25 (RBM25), mRNA	NM_021239	Hs.531106	BX647116
RBM35B	RNA binding motif protein 35B (RBM35B), mRNA	NM_024939	Hs.592053	NM_024939
RBM39	RNA binding motif protein 39 (RBM39), transcript variant 1, mRNA	NM_184234	Hs.282901	CR749443
RBMX	RNA binding motif protein, X-linked (RBMX), mRNA	NM_002139	Hs.380118	BX647131
RBMXL1	RNA binding motif protein, X-linked-like 1 (RBMXL1), mRNA	NM_019610	Hs.481898	AL832554
RBPSUH	mRNA for H-2K binding factor-2, complete cds.	D14041	Unknown	
RCBTB1	regulator of chromosome condensation (RCC1) and BTB (POZ) domain containing protein 1 (RCBTB1), mRNA	NM_018191	Hs.508021	AB209222
RCC2	regulator of chromosome condensation 2 (RCC2), mRNA	NM_018715	Hs.380857	AJ421269
RCHY1	ring finger and CHY zinc finger domain containing 1 (RCHY1), transcript variant 1, mRNA	NM_015436	Hs.48297	NM_015436
RCN2	reticulocalbin 2, EF-hand calcium binding domain (RCN2), mRNA	NM_002902	Hs.79088	AK126522
RCOR2	REST corepressor 2 (RCOR2), mRNA	NM_173587	Hs.98788	NM_173587
RDM1	RAD52 motif 1 (RDM1), transcript variant 2, mRNA	NM_001034836	Hs.194411	NM_145654
RECQL4	RecQ protein-like 4 (RECQL4), mRNA	NM_004260	Hs.31442	BC020496
REPS1	RALBP1 associated Eps domain containing 1 (REPS1), mRNA	NM_031922	Hs.334603	AB209143
REST	RE1-silencing transcription factor (REST), mRNA	NM_005612	Hs.631513	NM_005612
RFC3	replication factor C (activator 1) 3, 38kDa (RFC3), transcript variant 1, mRNA	NM_002915	Hs.115474	AK128459
RFC3	replication factor C (activator 1) 3, 38kDa (RFC3), transcript variant 2, mRNA	NM_181558	Hs.115474	AK128459
RFC4	replication factor C (activator 1) 4, 37kDa (RFC4), transcript variant 1, mRNA	NM_002916	Hs.518475	NM_002916
RFWD2	ring finger and WD repeat domain 2 (RFWD2), transcript variant 1, mRNA	NM_022457	Hs.523744	NM_022457
RFX3	mRNA; cDNA DKFZp761E2423 (from clone DKFZp761E2423)	AL157466	Hs.664105	AL157466
RFXDC2	regulatory factor X domain containing 2 (RFXDC2), mRNA	NM_022841	Hs.282855	CR749418
RG9MTD1	RNA (guanine-9-) methyltransferase domain containing 1 (RG9MTD1), mRNA	NM_017819	Hs.700716	AK000439
RG9MTD1	RNA (guanine-9-) methyltransferase domain containing 1 (RG9MTD1), mRNA	NM_017819	Hs.700716	AK000439
RG9MTD1	RNA (guanine-9-) methyltransferase domain containing 1 (RG9MTD1), mRNA	NM_017819	Hs.700716	AK000439
RG9MTD1	RNA (guanine-9-) methyltransferase domain containing 1 (RG9MTD1), mRNA	NM_017819	Hs.700716	AK000439
RG9MTD1	RNA (guanine-9-) methyltransferase domain containing 1 (RG9MTD1), mRNA	NM_017819	Hs.700716	AK000439
RG9MTD1	RNA (guanine-9-) methyltransferase domain containing 1 (RG9MTD1), mRNA	NM_017819	Hs.700716	AK000439
RG9MTD1	RNA (guanine-9-) methyltransferase domain containing 1 (RG9MTD1), mRNA	NM_017819	Hs.700716	AK000439
RG9MTD1	RNA (guanine-9-) methyltransferase domain containing 1 (RG9MTD1), mRNA	NM_017819	Hs.700716	AK000439
RG9MTD1	RNA (guanine-9-) methyltransferase domain containing 1 (RG9MTD1), mRNA	NM_017819	Hs.700716	AK000439
RG9MTD1	RNA (guanine-9-) methyltransferase domain containing 1 (RG9MTD1), mRNA	NM_017819	Hs.700716	AK000439
RGL3	ral guanine nucleotide dissociation stimulator-like 3	ENST00000380456	Unknown	

RGMA	RGM domain family, member A (RGMA), mRNA	NM_020211	Hs.271277	AK125047
RGMA	RGM domain family, member A (RGMA), mRNA	NM_020211	Hs.271277	AK125047
RGPD2	RANBP2-like and GRIP domain containing 2 (RGPD2), mRNA	NM_001078170	Hs.656849	CR749330
RGPD5	RANBP2-like and GRIP domain containing 5 (RGPD5), transcript variant 1, mRNA	NM_005054	Hs.469630	NM_005054
RHBDL3	rhomboid, veinlet-like 3 (Drosophila) (RHBDL3), mRNA	NM_138328	Hs.655027	NM_138328
RHBG	Rh family, B glycoprotein (RHBG), mRNA	NM_020407	Hs.131835	BC065218
RHO	rhodopsin (opsin 2, rod pigment) (retinitis pigmentosa 4, autosomal dominant) (RHO), mRNA	NM_000539	Hs.247565	NM_000539
RHPN2	rhophilin, Rho GTPase binding protein 2 (RHPN2), mRNA	NM_033103	Hs.466435	AK126506
RIC3	RIC3 isoform d (RIC3) mRNA, complete cds.	AY326436	Hs.655660	AL832601
RICS	Rho GTPase-activating protein (RICS), mRNA	NM_014715	Hs.440379	AL833062
RICS	Rho GTPase-activating protein (RICS), mRNA	NM_014715	Hs.440379	AL833062
RIF1	RAP1 interacting factor homolog (yeast) (RIF1), mRNA	NM_018151	Hs.655671	NM_018151
RIF1	RAP1 interacting factor homolog (yeast) (RIF1), mRNA	NM_018151	Hs.655671	NM_018151
RIF1	RAP1 interacting factor homolog (yeast) (RIF1), mRNA	NM_018151	Hs.655671	NM_018151
RIOK2	RIO kinase 2 (yeast) (RIOK2), mRNA	NM_018343	Hs.27021	AK001697
RIOK2	RIO kinase 2 (yeast) (RIOK2), mRNA	NM_018343	Hs.27021	AK001697
RIOK2	RIO kinase 2 (yeast) (RIOK2), mRNA	NM_018343	Hs.27021	AK001697
RIOK2	Serine/threonine-protein kinase RIO2 (EC 2.7.11.1) (RIO kinase 2).	ENST00000379841	Unknown	
RIOK2	RIO kinase 2 (yeast) (RIOK2), mRNA	NM_018343	Hs.27021	AK001697
RIOK2	RIO kinase 2 (yeast) (RIOK2), mRNA	NM_018343	Hs.27021	AK001697
RIOK2	RIO kinase 2 (yeast) (RIOK2), mRNA	NM_018343	Hs.27021	AK001697
RIOK2	RIO kinase 2 (yeast) (RIOK2), mRNA	NM_018343	Hs.27021	AK001697
RIOK2	RIO kinase 2 (yeast) (RIOK2), mRNA	NM_018343	Hs.27021	AK001697
RIOK2	RIO kinase 2 (yeast) (RIOK2), mRNA	NM_018343	Hs.27021	AK001697
RIOK2	RIO kinase 2 (yeast) (RIOK2), mRNA	NM_018343	Hs.27021	AK001697
RKHD2	ring finger and KH domain containing 2 (RKHD2), mRNA	NM_016626	Hs.465144	NM_016626
RMI1	RMI1, RecQ mediated genome instability 1, homolog (S. cerevisiae) (RMI1), mRNA	NM_024945	Hs.284137	NM_024945
RNASEH2B	ribonuclease H2, subunit B (RNASEH2B), mRNA	NM_024570	Hs.306291	AK124228
RNASEN	ribonuclease III, nuclear (RNASEN), mRNA	NM_013235	Hs.97997	NM_013235
RNASET2	ribonuclease T2 (RNASET2), mRNA	NM_003730	Hs.529989	AK001769
RNF157	ring finger protein 157 (RNF157), mRNA	NM_052916	Hs.500643	NM_052916
RNF17	ring finger protein 17 (RNF17), mRNA	NM_031277	Hs.97464	NM_031277
RNF2	ring finger protein 2 (RNF2), mRNA	NM_007212	Hs.591490	NM_007212
RNF44	ring finger protein 44 (RNF44), mRNA	NM_014901	Hs.650059	BC063297
RNGTT	RNA guanylyltransferase and 5'-phosphatase (RNGTT), mRNA	NM_003800	Hs.699450	BX537450
RNMT	RNA (guanine-7-) methyltransferase (RNMT), mRNA	NM_003799	Hs.592347	AF067791
ROBO2	cDNA FLJ90299 fis, clone NT2RP2000514, highly similar to roundabout 2 (robo2) mRNA.	AK074780	Hs.13305	BX648828
RP11-38O23.2	PNPK6288 (LOC389852), mRNA	NM_205856	Hs.319319	NM_205856
RP11-93B10.1	hypothetical protein LOC139886 (LOC139886), mRNA	NM_001012968	Hs.612782	AL833314
RP3-509I19.5	lung specific F-box and DH domain containing protein (LFDH), mRNA	NM_001077706	Hs.660603	NM_001077706
RP4-691N24.1	KIAA0980 protein (KIAA0980), mRNA	NM_025176	Hs.696157	NM_025176
RP6-213H19.1	serine/threonine protein kinase MST4 (MST4), transcript variant 1, mRNA	NM_016542	Hs.444247	NM_016542
RPA3	replication protein A3, 14kDa (RPA3), mRNA	NM_002947	Hs.487540	NM_002947
RPL10A	ribosomal protein L10a (RPL10A), mRNA	NM_007104	Hs.546269	BF972106
RPL24	ribosomal protein L24 (RPL24), mRNA	NM_000986	Hs.477028	CR608385
RPRM	reprimo, TP53 dependent G2 arrest mediator candidate (RPRM), mRNA	NM_019845	Hs.100890	AB043585
RPRML	reprimo-like (RPRML), mRNA	NM_203400	Hs.367999	BC033942
RPS6	ribosomal protein S6 (RPS6), mRNA	NM_001010	Hs.408073	BG403498
RPS6KA6	ribosomal protein S6 kinase, 90kDa, polypeptide 6 (RPS6KA6), mRNA	NM_014496	Hs.368153	AF184965
RPS7	ribosomal protein S7 (RPS7), mRNA	NM_001011	Hs.546287	AB209386
RSF1	remodeling and spacing factor 1 (RSF1), mRNA	NM_016578	Hs.420229	AF227948
RTF1	Rtf1, Paf1/RNA polymerase II complex component, homolog (S. cerevisiae) (RTF1), mRNA	NM_015138	Hs.511096	NM_015138

RUFY3	RUN and FYVE domain containing 3 (RUFY3), transcript variant 1, mRNA	NM_001037442	Hs.7972	BC051716
RUTBC2	RUN and TBC1 domain containing 2 (RUTBC2), transcript variant 1, mRNA	NM_001039948	Hs.474397	AB275761
S100A14	S100 calcium binding protein A14 (S100A14), mRNA	NM_020672	Hs.288998	BG674026
SAE2	SUMO1 activating enzyme subunit 2 (SAE2), mRNA	NM_005499	Hs.631580	AK124730
SALL1	sal-like 1 (Drosophila) (SALL1), mRNA	NM_002968	Hs.135787	NM_002968
SALL2	sal-like 2 (Drosophila) (SALL2), mRNA	NM_005407	Hs.416358	NM_005407
SAMD3	sterile alpha motif domain containing 3 (SAMD3), transcript variant 1, mRNA	NM_001017373	Hs.440508	AK091351
SAP130	Sin3A-associated protein, 130kDa (SAP130), mRNA	NM_024545	Hs.32995	AL136833
SAPS3	SAPS domain family, member 3 (SAPS3), mRNA	NM_018312	Hs.503022	CR936718
SASS6	spindle assembly 6 homolog (C. elegans) (SASS6), mRNA	NM_194292	Hs.591447	BX641109
SATB2	cDNA: FLJ21474 fis, clone COL04941.	AK025127	Unknown	
SATB2	SATB family member 2 (SATB2), mRNA	NM_015265	Hs.516617	AB209376
SAV1	salvador homolog 1 (Drosophila) (SAV1), mRNA	NM_021818	Hs.642842	AL833378
SCAMP1	secretory carrier membrane protein 1 (SCAMP1), mRNA	NM_004866	Hs.482587	NM_004866
SCC-112	SCC-112 protein (SCC-112), mRNA	NM_015200	Hs.331431	NM_001100399
SCG3	secretogranin III (SCG3), mRNA	NM_013243	Hs.232618	NM_013243
SCML2	sex comb on midleg-like 2 (Drosophila) (SCML2), mRNA	NM_006089	Hs.495774	BC040497
SCN4A	sodium channel, voltage-gated, type IV, alpha (SCN4A), mRNA	NM_000334	Hs.46038	M81758
SCRL	full-length cDNA clone CS0DB003YO14 of Neuroblastoma Cot 10-normalized of (human).	ENST00000270642	Unknown	
SDCCAG10	serologically defined colon cancer antigen 10 (SDCCAG10), mRNA	NM_005869	Hs.371372	BX647971
SDR-O	orphan short-chain dehydrogenase / reductase (SDR-O), mRNA	NM_148897	Hs.380178	AK122782
SEC11A	SEC11 homolog A (S. cerevisiae) (SEC11A), mRNA	NM_014300	Hs.9534	CR749656
SEC24A	SEC24 related gene family, member A (S. cerevisiae) (SEC24A), mRNA	NM_021982	Hs.595540	NM_021982
SEC31A	cDNA FLJ46167 fis, clone TESTI4003179.	AK128047	Hs.370024	AK128047
SECISBP2	SECIS binding protein 2 (SECISBP2), mRNA	NM_024077	Hs.59804	NM_024077
SECISBP2	SECIS binding protein 2 (SECISBP2), mRNA	NM_024077	Hs.59804	NM_024077
SEH1L	SEH1-like (S. cerevisiae) (SEH1L), transcript variant 2, mRNA	NM_031216	Hs.301048	AK226075
SEMA3A	sema domain, immunoglobulin domain (Ig), short basic domain, secreted, (semaphorin) 3A (SEMA3A), mRNA	NM_006080	Hs.252451	NM_006080
SEMA3A	sema domain, immunoglobulin domain (Ig), short basic domain, secreted, (semaphorin) 3A (SEMA3A), mRNA	NM_006080	Hs.252451	NM_006080
SEMA4C	sema domain, immunoglobulin domain (Ig), transmembrane domain (TM) and short cytoplasmic domain, (semaphorin) 4C (SEMA4C), mRNA	NM_017789	Hs.516220	AB051526
SEMA6A	sema domain, transmembrane domain (TM), and cytoplasmic domain, (semaphorin) 6A (SEMA6A), mRNA	NM_020796	Hs.156967	BC032619
SENP6	SUMO1/sentrin specific peptidase 6 (SENP6), mRNA	NM_015571	Hs.485784	NM_015571
SEPHS1	Selenide, water dikinase 1 (EC 2.7.9.3) (Selenophosphate synthetase 1) (Selenium donor protein 1).	ENST00000327347	Unknown	
SEPT3	septin 3 (SEPT3), transcript variant A, mRNA	NM_145733	Hs.120483	AL833942
SERBP1	SERPINE1 mRNA binding protein 1 (SERBP1), transcript variant 1, mRNA	NM_001018067	Hs.530412	NM_001018067
SERBP1	SERPINE1 mRNA binding protein 1 (SERBP1), transcript variant 1, mRNA	NM_001018067	Hs.530412	NM_001018067
SERBP1	SERPINE1 mRNA binding protein 1 (SERBP1), transcript variant 1, mRNA	NM_001018067	Hs.530412	NM_001018067
SETD2	SET domain containing 2 (SETD2), mRNA	NM_014159	Hs.517941	BX649110
SETD2	SET domain containing 2 (SETD2), mRNA	NM_014159	Hs.517941	BX649110
SETD6	SET domain containing 6 (SETD6), mRNA	NM_024860	Hs.592060	AK024801
SETX	senataxin (SETX), mRNA	NM_015046	Hs.460317	NM_015046
SF3A3	splicing factor 3a, subunit 3, 60kDa (SF3A3), mRNA	NM_006802	Hs.77897	AK128438
SF3A3	splicing factor 3a, subunit 3, 60kDa (SF3A3), mRNA	NM_006802	Hs.77897	AK128438
SF3B1	splicing factor 3b, subunit 1, 155kDa (SF3B1), transcript variant 1, mRNA	NM_012433	Hs.632554	NM_012433
SFPQ	splicing factor proline/glutamine-rich (polypyrimidine tract binding protein associated) (SFPQ), mRNA	NM_005066	Hs.355934	AK095702
SFRP1	secreted frizzled-related protein 1 (SFRP1), mRNA	NM_003012	Hs.695991	BC036503
SFRP2	secreted frizzled-related protein 2 (SFRP2), mRNA	NM_003013	Hs.481022	NM_003013
SFRP2	secreted frizzled-related protein 2 (SFRP2), mRNA	NM_003013	Hs.481022	NM_003013
SFRP2	secreted frizzled-related protein 2 (SFRP2), mRNA	NM_003013	Hs.481022	NM_003013
SFRS1	splicing factor, arginine/serine-rich 1 (splicing factor 2, alternate splicing factor) (SFRS1), transcript variant 2, mRNA	NM_001078166	Hs.68714	NM_001078166

SFRS2IP	splicing factor, arginine/serine-rich 2, interacting protein (SFRS2IP), mRNA	NM_004719	Hs.210367	NM_004719
SFRS3	splicing factor, arginine/serine-rich 3 (SFRS3), mRNA	NM_003017	Hs.405144	AK091927
SFRS7	splicing factor, arginine/serine-rich 7, 35kDa (SFRS7), mRNA	NM_001031684	Hs.309090	AK091425
SGEF	infant liver cDNA, clone:HMFN1864, full insert sequence.	AB073386	Hs.570682	AK127282
SGEF	Src homology 3 domain-containing guanine nucleotide exchange factor (SGEF), mRNA	NM_015595	Hs.570682	AK127282
SHANK2	SH3 and multiple ankyrin repeat domains 2 (SHANK2), transcript variant 1, mRNA	NM_012309	Hs.268726	NM_012309
SHF	Src homology 2 domain containing F (SHF), mRNA	NM_138356	Hs.310399	NM_138356
SHF	cDNA FLJ41664 fis, clone FEBRA2027352.	AK123658	Hs.310399	NM_138356
SHOX	Human pseudoautosomal homeodomain-containing protein (PHOG) mRNA, complete cds.	U89331	Hs.105932	U89331
SHPRH	SNF2 histone linker PHD RING helicase (SHPRH), transcript variant 2, mRNA	NM_173082	Hs.124537	NM_001042683
SHPRH	SNF2 histone linker PHD RING helicase (SHPRH), transcript variant 1, mRNA	NM_001042683	Hs.124537	NM_001042683
SIN3A	SIN3 homolog A, transcription regulator (yeast) (SIN3A), mRNA	NM_015477	Hs.513039	NM_015477
SIP1	survival of motor neuron protein interacting protein 1 (SIP1), transcript variant alpha, mRNA	NM_003616	Hs.696323	BC028095
SIRT1	sirtuin (silent mating type information regulation 2 homolog) 1 (S. cerevisiae) (SIRT1), mRNA	NM_012238	Hs.369779	NM_012238
SIX4	sine oculis homeobox homolog 4 (Drosophila) (SIX4), mRNA	NM_017420	Hs.97849	NM_017420
SKIV2L2	superkiller viralicidic activity 2-like 2 (S. cerevisiae) (SKIV2L2), mRNA	NM_015360	Hs.274531	BC065258
SLAIN1	SLAIN motif family, member 1 (SLAIN1), transcript variant 1, mRNA	NM_001040153	Hs.349955	NM_001040153
SLC10A4	solute carrier family 10 (sodium/bile acid cotransporter family), member 4 (SLC10A4), mRNA	NM_152679	Hs.132591	BC019066
SLC13A3	solute carrier family 13 (sodium-dependent dicarboxylate transporter), member 3 (SLC13A3), transcript variant 2, mRNA	NM_001011554	Hs.655498	NM_001011554
SLC16A9	solute carrier family 16, member 9 (monocarboxylic acid transporter 9) (SLC16A9), mRNA	NM_194298	Hs.499709	AK126643
SLC18A2	solute carrier family 18 (vesicular monoamine), member 2 (SLC18A2), mRNA	NM_003054	Hs.654476	BC030593
SLC19A2	solute carrier family 19 (thiamine transporter), member 2 (SLC19A2), mRNA	NM_006996	Hs.30246	AJ237724
SLC25A13	solute carrier family 25, member 13 (citrin) (SLC25A13), mRNA	NM_014251	Hs.489190	NM_014251
SLC25A32	solute carrier family 25, member 32 (SLC25A32), mRNA	NM_030780	Hs.654812	NM_030780
SLC25A33	solute carrier family 25, member 33 (SLC25A33), mRNA	NM_032315	Hs.568613	CR595626
SLC27A2	solute carrier family 27 (fatty acid transporter), member 2 (SLC27A2), mRNA	NM_003645	Hs.11729	AK223145
SLC2A14	solute carrier family 2 (facilitated glucose transporter), member 14, mRNA (cDNA clone MGC:71510 IMAGE:5297510), complete cds.	BC060766	Hs.655169	AK126026
SLC2A3	solute carrier family 2 (facilitated glucose transporter), member 3 (SLC2A3), mRNA	NM_006931	Hs.419240	AB209607
SLC30A9	solute carrier family 30 (zinc transporter), member 9 (SLC30A9), mRNA	NM_006345	Hs.479634	NM_006345
SLC34A2	solute carrier family 34 (sodium phosphate), member 2 (SLC34A2), mRNA	NM_006424	Hs.479372	NM_006424
SLC35F3	solute carrier family 35, member F3 (SLC35F3), mRNA	NM_173508	Hs.158748	AK095031
SLC37A3	solute carrier family 37 (glycerol-3-phosphate transporter), member 3 (SLC37A3), transcript variant 1, mRNA	NM_207113	Hs.446021	NM_207113
SLC38A1	solute carrier family 38, member 1 (SLC38A1), transcript variant 1, mRNA	NM_030674	Hs.699239	NM_030674
SLC38A3	solute carrier family 38, member 3 (SLC38A3), mRNA	NM_006841	Hs.76460	BX537382
SLC39A2	solute carrier family 39 (zinc transporter), member 2 (SLC39A2), mRNA	NM_014579	Hs.175783	CR621267
SLC5A12	solute carrier family 5 (sodium/glucose cotransporter), member 12 (SLC5A12), transcript variant 1, mRNA	NM_001042366	Hs.148907	BC049207
SLC5A3	solute carrier family 5 (inositol transporters), member 3 (SLC5A3), mRNA	NM_006933	Hs.302742	NM_006933
SLC6A20	solute carrier family 6 (proline IMINO transporter), member 20 (SLC6A20), transcript variant 1, mRNA	NM_020208	Hs.413095	AJ276207
SLCO1A2	solute carrier organic anion transporter family, member 1A2 (SLCO1A2), transcript variant 3, mRNA	NM_005075	Hs.46440	NM_134431
SLCO1A2	solute carrier organic anion transporter family, member 1A2 (SLCO1A2), transcript variant 1, mRNA	NM_134431	Hs.46440	NM_134431
SLCO4C1	solute carrier organic anion transporter family, member 4C1 (SLCO4C1), mRNA	NM_180991	Hs.127648	NM_180991
SLITRK5	SLIT and NTRK-like family, member 5 (SLITRK5), mRNA	NM_015567	Hs.591208	NM_015567
SLTM	SAFB-like, transcription modulator (SLTM), transcript variant 1, mRNA	NM_024755	Hs.512932	NM_024755
SMA5	SMA5 (SMA5), mRNA	NM_021036	Unknown	
SMARCA5	SWI/SNF related, matrix associated, actin dependent regulator of chromatin, subfamily a, member 5 (SMARCA5), mRNA	NM_003601	Hs.589489	NM_003601
SMARCAD1	SWI/SNF-related, matrix-associated actin-dependent regulator of chromatin, subfamily a, containing DEAD/H box 1 (SMARCAD1), mRNA	NM_020159	Hs.410406	AY008271
SMARCAD1	SWI/SNF-related, matrix-associated actin-dependent regulator of chromatin, subfamily a, containing DEAD/H box 1 (SMARCAD1), mRNA	NM_020159	Hs.410406	AY008271
SMARCAD1	SWI/SNF-related, matrix-associated actin-dependent regulator of chromatin, subfamily a, containing DEAD/H box 1 (SMARCAD1), mRNA	NM_020159	Hs.410406	AY008271
SMARCAD1	SWI/SNF-related, matrix-associated actin-dependent regulator of chromatin, subfamily a, containing DEAD/H box 1 (SMARCAD1), mRNA	NM_020159	Hs.410406	AY008271

SMARCAD1	SWI/SNF-related, matrix-associated actin-dependent regulator of chromatin, subfamily a, containing DEAD/H box 1 (SMARCAD1), mRNA	NM_020159	Hs.410406	AY008271
SMARCAD1	SWI/SNF-related, matrix-associated actin-dependent regulator of chromatin, subfamily a, containing DEAD/H box 1 (SMARCAD1), mRNA	NM_020159	Hs.410406	AY008271
SMARCAD1	SWI/SNF-related, matrix-associated actin-dependent regulator of chromatin, subfamily a, containing DEAD/H box 1 (SMARCAD1), mRNA	NM_020159	Hs.410406	AY008271
SMARCAD1	SWI/SNF-related, matrix-associated actin-dependent regulator of chromatin, subfamily a, containing DEAD/H box 1 (SMARCAD1), mRNA	NM_020159	Hs.410406	AY008271
SMARCAD1	SWI/SNF-related, matrix-associated actin-dependent regulator of chromatin, subfamily a, containing DEAD/H box 1 (SMARCAD1), mRNA	NM_020159	Hs.410406	AY008271
SMARCAD1	SWI/SNF-related, matrix-associated actin-dependent regulator of chromatin, subfamily a, containing DEAD/H box 1 (SMARCAD1), mRNA	NM_020159	Hs.410406	AY008271
SMARCC1	SWI/SNF related, matrix associated, actin dependent regulator of chromatin, subfamily c, member 1 (SMARCC1), mRNA	NM_003074	Hs.476179	NM_003074
SMARCC1	SWI/SNF related, matrix associated, actin dependent regulator of chromatin, subfamily c, member 1 (SMARCC1), mRNA	NM_003074	Hs.476179	NM_003074
SMC1A	structural maintenance of chromosomes 1A (SMC1A), mRNA	NM_006306	Hs.211602	NM_006306
SMC3	structural maintenance of chromosomes 3 (SMC3), mRNA	NM_005445	Hs.24485	NM_005445
SMC4	cDNA FLJ11338 fis, clone PLACE1010720, highly similar to mRNA for chromosome-associated polypeptide-C.	AK002200	Hs.58992	NM_005496
SMG1	PI-3-kinase-related kinase SMG-1 (SMG1), mRNA	NM_015092	Hs.460179	NM_015092
SMG1	PI-3-kinase-related kinase SMG-1 (SMG1), mRNA	NM_015092	Hs.460179	NM_015092
SMG1	PI-3-kinase-related kinase SMG-1 (SMG1), mRNA	NM_015092	Hs.460179	NM_015092
SMG1	PI-3-kinase-related kinase SMG-1 (SMG1), mRNA	NM_015092	Hs.460179	NM_015092
SMNDC1	survival motor neuron domain containing 1 (SMNDC1), mRNA	NM_005871	Hs.632093	BC039110
SMUG1	cDNA FLJ34149 fis, clone FCBBF3012588, highly similar to single-strand selective monofunctional uracil DNA glycosylase mRNA.	AK091468	Hs.632721	AK091468
SMYD4	SET and MYND domain containing 4 (SMYD4), mRNA	NM_052928	Hs.514602	NM_052928
SNAPC4	small nuclear RNA activating complex, polypeptide 4, 190kDa (SNAPC4), mRNA	NM_003086	Hs.113265	AF032387
SNHG1	small nucleolar RNA host gene (non-protein coding) 1 (SNHG1) on chromosome 11	NR_003098	Unknown	
SNN	stannin (SNN), mRNA	NM_003498	Hs.700592	NM_003498
SNRPC	small nuclear ribonucleoprotein polypeptide C (SNRPC), mRNA	NM_003093	Hs.1063	BU603692
SNTG2	syntrophin, gamma 2 (SNTG2), mRNA	NM_018968	Hs.657453	AJ003029
SNX10	sorting nexin 10 (SNX10), mRNA	NM_013322	Hs.571296	BX648363
SNX10	sorting nexin 10 (SNX10), mRNA	NM_013322	Hs.571296	BX648363
SNX2	sorting nexin 2 (SNX2), mRNA	NM_003100	Hs.696016	NM_003100
SNX26	sorting nexin 26 (SNX26), mRNA	NM_052948	Hs.515364	AK127255
SOCS2	suppressor of cytokine signaling 2 (SOCS2), mRNA	NM_003877	Hs.485572	BC070039
SON	SON DNA binding protein (SON), transcript variant f, mRNA	NM_138927	Hs.517262	NM_138927
SORL1	sortilin-related receptor, L(DLR class) A repeats-containing (SORL1), mRNA	NM_003105	Hs.368592	NM_003105
SOX2	SRY (sex determining region Y)-box 2 (SOX2), mRNA	NM_003106	Hs.518438	NM_003106
SOX21	SRY (sex determining region Y)-box 21 (SOX21), mRNA	NM_007084	Hs.187577	NM_007084
SOX3	SRY (sex determining region Y)-box 3 (SOX3), mRNA	NM_005634	Hs.157429	NM_005634
SP3	Sp3 transcription factor (SP3), transcript variant 1, mRNA	NM_003111	Hs.531587	AB209334
SP3	mRNA; cDNA DKFZp686N17231 (from clone DKFZp686N17231).	BX648857	Hs.531587	AB209334
SP5	Sp5 transcription factor (SP5), mRNA	NM_001003845	Hs.368802	AB096175
SP5	Sp5 transcription factor (SP5), mRNA	NM_001003845	Hs.368802	AB096175
SPAST	spastin (SPAST), transcript variant 1, mRNA	NM_014946	Hs.468091	NM_014946
SPATA6	spermatogenesis associated 6	ENST00000371847	Unknown	
SPFH2	SPFH domain family, member 2 (SPFH2), transcript variant 1, mRNA	NM_007175	Hs.696282	NM_007175
SPINT1	serine peptidase inhibitor, Kunitz type 1 (SPINT1), transcript variant 1, mRNA	NM_181642	Hs.233950	BC018702
SPTLC1	serine palmitoyltransferase, long chain base subunit 1 (SPTLC1), transcript variant 1, mRNA	NM_006415	Hs.90458	AB209757
SR140	Human mRNA for KIAA0332 gene, partial cds.	AB002330	Hs.596572	NM_001080415
SR140	SR140 protein.	ENST00000319822	Unknown	
SRC	v-src sarcoma (Schmidt-Ruppin A-2) viral oncogene homolog (avian) (SRC), transcript variant 1, mRNA	NM_005417	Hs.195659	NM_005417
SRCRB4D	scavenger receptor cysteine rich domain containing, group B (4 domains) (SRCRB4D), mRNA	NM_080744	Hs.567684	NM_080744
SRGAP3	SLIT-ROBO Rho GTPase activating protein 3, mRNA (cDNA clone IMAGE:4830856), complete cds.	BC039300	Hs.654743	NM_014850
SRRM1	serine/arginine repetitive matrix 1 (SRRM1), mRNA	NM_005839	Hs.18192	NM_005839
SRRM1	serine/arginine repetitive matrix 1 (SRRM1), mRNA	NM_005839	Hs.18192	NM_005839

SSB	Sjogren syndrome antigen B (autoantigen La) (SSB), mRNA	NM_003142	Hs.632535	AK124811
SSB	Sjogren syndrome antigen B (autoantigen La) (SSB), mRNA	NM_003142	Hs.632535	AK124811
SSTR1	somatostatin receptor 1 (SSTR1), mRNA	NM_001049	Hs.248160	NM_001049
ST14	suppression of tumorigenicity 14 (colon carcinoma) (ST14), mRNA	NM_021978	Hs.504315	NM_021978
STAU1	staufen, RNA binding protein, homolog 1 (Drosophila) (STAU1), transcript variant T3, mRNA	NM_017453	Hs.596704	NM_017453
STAU2	staufen, RNA binding protein, homolog 2 (Drosophila) (STAU2), mRNA	NM_014393	Hs.561815	NM_014393
STK31	serine/threonine kinase 31 (STK31), transcript variant 2, mRNA	NM_032944	Hs.309767	BC036476
STON2	stonin 2 (STON2), mRNA	NM_033104	Hs.14248	BX648249
STOX1	storkhead box 1 (STOX1), mRNA	NM_152709	Hs.37636	AY842014
STOX1	storkhead box 1 (STOX1), mRNA	NM_152709	Hs.37636	AY842014
STRAP	serine/threonine kinase receptor associated protein (STRAP), mRNA	NM_007178	Hs.504895	NM_007178
STRAP	serine/threonine kinase receptor associated protein (STRAP), mRNA	NM_007178	Hs.504895	NM_007178
STRBP	spermatid perinuclear RNA binding protein (STRBP), mRNA	NM_018387	Hs.696159	NM_018387
STRBP	spermatid perinuclear RNA binding protein (STRBP), mRNA	NM_018387	Hs.696159	NM_018387
SUB1	Activated RNA polymerase II transcriptional coactivator p15 (SUB1 homolog) (Positive cofactor 4) (PC4) (p14).	ENST00000265073	Unknown	
SUB1	SUB1 homolog (S. cerevisiae) (SUB1), mRNA	NM_006713	Hs.229641	BX537584
SUB1	SUB1 homolog (S. cerevisiae) (SUB1), mRNA	NM_006713	Hs.229641	BX537584
SUDS3	suppressor of defective silencing 3 homolog (S. cerevisiae) (SUDS3), mRNA	NM_022491	Hs.416630	NM_022491
SUHW2	suppressor of hairy wing homolog 2 (Drosophila) (SUHW2), mRNA	NM_080764	Hs.43834	NM_080764
SUHW3	suppressor of hairy wing homolog 3 (Drosophila) (SUHW3), mRNA	NM_017666	Hs.308418	BC051728
SUPT16H	suppressor of Ty 16 homolog (S. cerevisiae) (SUPT16H), mRNA	NM_007192	Hs.213724	NM_007192
SUPT16H	suppressor of Ty 16 homolog (S. cerevisiae) (SUPT16H), mRNA	NM_007192	Hs.213724	NM_007192
SURF5	surfeit 5 (SURF5), transcript variant b, mRNA	NM_133640	Hs.78354	AK124518
SUSD3	sushi domain containing 3 (SUSD3), mRNA	NM_145006	Hs.88417	AK128289
SUV39H2	suppressor of variegation 3-9 homolog 2 (Drosophila) (SUV39H2), mRNA	NM_024670	Hs.554883	NM_024670
SUV420H2	suppressor of variegation 4-20 homolog 2 (Drosophila) (SUV420H2), mRNA	NM_032701	Hs.590982	BC044889
SUZ12	suppressor of zeste 12 homolog (Drosophila) (SUZ12), mRNA	NM_015355	Hs.462732	D63881
SUZ12	suppressor of zeste 12 homolog (Drosophila) (SUZ12), mRNA	NM_015355	Hs.462732	D63881
SYNE2	spectrin repeat containing, nuclear envelope 2 (SYNE2), transcript variant 5, mRNA	NM_182914	Hs.525392	NM_182914
SYNE2	spectrin repeat containing, nuclear envelope 2 (SYNE2), transcript variant 1, mRNA	NM_015180	Hs.525392	NM_182914
SYNE2	spectrin repeat containing, nuclear envelope 2 (SYNE2), transcript variant 1, mRNA	NM_015180	Hs.525392	NM_182914
SYNE2	spectrin repeat containing, nuclear envelope 2 (SYNE2), transcript variant 5, mRNA	NM_182914	Hs.525392	NM_182914
TACSTD1	tumor-associated calcium signal transducer 1 (TACSTD1), mRNA	NM_002354	Hs.699160	AK026585
TADA1L	transcriptional adaptor 1 (HFI1 homolog, yeast)-like (TADA1L), mRNA	NM_053053	Hs.435967	BC036497
TAF1	Transcription initiation factor TFIID subunit 1 (EC 2.7.11.1) (Transcription initiation factor TFIID 250 kDa subunit) (TAF(II)250) (TAFII-250) (TAFII250) (TBP-associated factor 250 kDa) (p250) (Cell cycle gene 1 protein)....	ENST00000373790	Unknown	
TAF9	TAF9 RNA polymerase II, TATA box binding protein (TBP)-associated factor, 32kDa (TAF9), transcript variant 4, mRNA	NM_001015892	Hs.653163	BC033320
TAF9	TAF9 RNA polymerase II, TATA box binding protein (TBP)-associated factor, 32kDa (TAF9), transcript variant 4, mRNA	NM_001015892	Hs.653163	BC033320
TAF9B	TAF9B RNA polymerase II, TATA box binding protein (TBP)-associated factor, 31kDa (TAF9B), mRNA	NM_015975	Hs.592248	NM_015975
TARBP1	Tar (HIV-1) RNA binding protein 1 (TARBP1), mRNA	NM_005646	Hs.498115	U38847
TARDBP	TAR DNA binding protein (TARDBP), mRNA	NM_007375	Hs.300624	NM_007375
TARDBP	TAR DNA binding protein (TARDBP), mRNA	NM_007375	Hs.300624	NM_007375
TATDN1	TatD DNase domain containing 1 (TATDN1), mRNA	NM_032026	Hs.170568	AK125270
TBC1D14	TBC1 domain family, member 14 (TBC1D14), mRNA	NM_020773	Hs.518611	NM_020773
TBC1D14	TBC1 domain family, member 14 (TBC1D14), mRNA	NM_020773	Hs.518611	NM_020773
TBC1D15	TBC1 domain family, member 15 (TBC1D15), mRNA	NM_022771	Hs.284630	NM_022771
TBCA	tubulin folding cofactor A (TBCA), mRNA	NM_004607	Hs.291212	BC067837
TBCE	tubulin folding cofactor E (TBCE), transcript variant 1, mRNA	NM_001079515	Hs.498143	NM_001079515
TBP	TATA box binding protein (TBP), mRNA	NM_003194	Hs.590872	BC110341
TBP	TATA box binding protein (TBP), mRNA	NM_003194	Hs.590872	BC110341
TBP	TATA box binding protein (TBP), mRNA	NM_003194	Hs.590872	BC110341

TCEAL8	transcription elongation factor A (SII)-like 8 (TCEAL8), transcript variant 1, mRNA	NM_153333	Hs.389734	BF673962
TCF2	transcription factor 2, hepatic; LF-B3; variant hepatic nuclear factor (TCF2), transcript variant b, mRNA	NM_006481	Hs.191144	NM_006481
TCF7	transcription factor 7 (T-cell specific, HMG-box) (TCF7), transcript variant 1, mRNA	NM_003202	Hs.573153	AL834166
TCF7	transcription factor 7 (T-cell specific, HMG-box) (TCF7), transcript variant 1, mRNA	NM_003202	Hs.573153	AL834166
TCF7	transcription factor 7 (T-cell specific, HMG-box) (TCF7), transcript variant 1, mRNA	NM_003202	Hs.573153	AL834166
TCF7	transcription factor 7 (T-cell specific, HMG-box) (TCF7), transcript variant 1, mRNA	NM_003202	Hs.573153	AL834166
TCF7	transcription factor 7 (T-cell specific, HMG-box) (TCF7), transcript variant 1, mRNA	NM_003202	Hs.573153	AL834166
TCF7L2	Transcription factor 7-like 2 (HMG box transcription factor 4) (T- cell-specific transcription factor 4) (TCF-4) (hTCF-4).	ENST00000369397	Unknown	
TDH	cDNA FLJ25033 fis, clone CBL02720.	AK057762	Hs.583896	AK057762
TDRD10	tudor domain containing 10 (TDRD10), mRNA	NM_182499	Hs.387671	NM_001098475
TEDDM1	transmembrane epididymal protein 1 (TEDDM1), mRNA	NM_172000	Hs.156977	NM_172000
TEX10	testis expressed sequence 10 (TEX10), mRNA	NM_017746	Hs.494648	AK000294
TEX14	testis expressed sequence 14 (TEX14), transcript variant 1, mRNA	NM_198393	Hs.390221	AL834143
TFAM	Transcription factor A, mitochondrial precursor (mtTFA) (Mitochondrial transcription factor 1) (MtTF1) (Transcription factor 6-like 2).	ENST00000373899	Unknown	
TFAM	transcription factor A, mitochondrial (TFAM), mRNA	NM_003201	Hs.642966	NM_003201
TFB2M	transcription factor B2, mitochondrial (TFB2M), mRNA	NM_022366	Hs.696427	AK026835
THAP5	THAP domain containing 5 (THAP5), mRNA	NM_182529	Hs.650237	AL833137
THC2493256	Q9P0J7_HUMAN (Q9P0J7) Potassium channel modulatory factor (FIGC1), partial (27%)	THC2493256	Unknown	
THC2495068	TWST1_GORGO (Q8MI06) Twist-related protein 1, partial (8%)	THC2495068	Unknown	
THC2495785	CN284574 17000531534200 GRN_EB cDNA 5', mRNA sequence	THC2495785	Unknown	
THC2498136	Q96316_ARATH (Q96316) Blue-copper binging protein III (Uclacyanin 3), partial (5%)	THC2498136	Unknown	
THC2505541	Q5VV55_HUMAN (Q5VV55) H3 histone, family 3A (H3 histone, family 3B) (H3F3A protein), complete	THC2505541	Unknown	
THC2505640	AJ710595 AJ710595 CMPD01 cDNA clone CMPD07455, mRNA sequence	THC2505640	Unknown	
THC2506656	Q7T2T6_BOTJR (Q7T2T6) Ribosomal protein, partial (70%)	THC2506656	Unknown	
THC2507321	Q7XBG7_PYRPY (Q7XBG7) Polygalacturonase-inhibiting protein, partial (5%)	THC2507321	Unknown	
THC2508355	Q25GJ8_MACFA (Q25GJ8) Brain cDNA, clone: QflA-22015, complete	THC2508355	Unknown	
THC2511513	THC2511513	THC2511513	Unknown	
THC2512199	THC2512199	THC2512199	Unknown	
THC2519808	THC2519808	THC2519808	Unknown	
THC2520542	Q9XDH2_MYCTU (Q9XDH2) Proline-rich mucin homolog, partial (3%)	THC2520542	Unknown	
THC2522381	ALU2_HUMAN (P39189) Alu subfamily SB sequence contamination warning entry, partial (20%)	THC2522381	Unknown	
THC2523685	BQ101125 ij24c06.y1 Melton Normalized Human Islet 4 N4-HIS 1 cDNA clone IMAGE:6135562 5', mRNA sequence	THC2523685	Unknown	
THC2524570	Q86TZ0_HUMAN (Q86TZ0) Full-length cDNA clone CS0DC023YN15 of Neuroblastoma of (human) (Fragment), partial (85%)	THC2524570	Unknown	
THC2526427	Q68EM8_HUMAN (Q68EM8) MAN2C1 protein, partial (13%)	THC2526427	Unknown	
THC2533546	XM_750378 rRNA assembly protein Mis3 {Aspergillus fumigatus Af293} (exp=-1; wgp=0; cg=0), partial (6%)	THC2533546	Unknown	
THC2536674	AA353695 EST62114 Jurkat T-cells V cDNA 5' end, mRNA sequence	THC2536674	Unknown	
THC2537289	UDU1_ARATH (Q9FHD5) DUF26 domain-containing protein 1 precursor, partial (7%)	THC2537289	Unknown	
THC2537371	Q294S6_DROPS (Q294S6) GA11008-PA (Fragment), partial (5%)	THC2537371	Unknown	
THC2537873	AK223183 arylsulfatase E precursor variant {Homo sapiens} (exp=-1; wgp=0; cg=0), partial (24%)	THC2537873	Unknown	
THC2539168	ALU1_HUMAN (P39188) Alu subfamily J sequence contamination warning entry, partial (11%)	THC2539168	Unknown	
THC2539698	Q59GN2_HUMAN (Q59GN2) Ribosomal protein L39 variant (Fragment), partial (77%)	THC2539698	Unknown	
THC2542546	ALU1_HUMAN (P39188) Alu subfamily J sequence contamination warning entry, partial (5%)	THC2542546	Unknown	
THC2543120	THC2543120	THC2543120	Unknown	
THC2545558	Q9F8M7_CARHY (Q9F8M7) DTDP-glucose 4,6-dehydratase (Fragment), partial (11%)	THC2545558	Unknown	
THC2548755	1ZQ9_A Chain A, Crystal Structure Of Human Dimethyladenosine Transferase. {Homo sapiens} (exp=-1; wgp=-1; cg=-1), partial (14%)	THC2548755	Unknown	
THC2548807	Q7Z5Z9_HUMAN (Q7Z5Z9) NPC-A-5, complete	THC2548807	Unknown	
THC2550474	THC2550474	THC2550474	Unknown	
THC2550720	ALU1_HUMAN (P39188) Alu subfamily J sequence contamination warning entry, partial (30%)	THC2550720	Unknown	
THC2553406	THC2553406	THC2553406	Unknown	

THC2554943	HSU77948 Bruton's tyrosine kinase-associated protein-135 {Homo sapiens} (exp=-1; wgp=0; cg=0), partial (18%)	THC2554943	Unknown	
THC2557016	Q3F710_9BURK (Q3F710) Inner-membrane translocator, partial (6%)	THC2557016	Unknown	
THC2559752	EF1B_HUMAN (P24534) Elongation factor 1-beta (EF-1-beta), complete	THC2559752	Unknown	
THC2562932	Q52M62_HUMAN (Q52M62) LOC285908 protein, partial (28%)	THC2562932	Unknown	
THC2565422	ALU1_HUMAN (P39188) Alu subfamily J sequence contamination warning entry, partial (3%)	THC2565422	Unknown	
THC2566704	ALU8_HUMAN (P39195) Alu subfamily SX sequence contamination warning entry, partial (9%)	THC2566704	Unknown	
THC2567672	W18193 IMAGE:20064 Soares infant brain 1NIB cDNA clone IMAGE:20064, mRNA sequence	THC2567672	Unknown	
THC2572360	THC2572360	THC2572360	Unknown	
THC2575678	Q753E4_ASHGO (Q753E4) AFR372Wp, partial (3%)	THC2575678	Unknown	
THC2576067	THC2576067	THC2576067	Unknown	
THC2580884	BM986990 UI-H-CO0-aqe-h-08-0-UI.s1 NCI_CGAP_Sub9 cDNA clone IMAGE:3104077 3', mRNA sequence	THC2580884	Unknown	
THC2581431	Q96AC8_HUMAN (Q96AC8) VIL1 protein, partial (46%)	THC2581431	Unknown	
THC2609466	BG548754 602576375F1 NIH_MGC_77 cDNA clone IMAGE:4704379 5', mRNA sequence	THC2609466	Unknown	
THC2612020	THC2612020	THC2612020	Unknown	
THC2614876	THC2614876	THC2614876	Unknown	
THC2615469	AA987196 or92a11.s1 NCI_CGAP_Lu5 cDNA clone IMAGE:1603292 3', mRNA sequence	THC2615469	Unknown	
THC2618142	THC2618142	THC2618142	Unknown	
THC2618237	Q8N329_HUMAN (Q8N329) C3orf64 protein, partial (18%)	THC2618237	Unknown	
THC2618720	THC2618720	THC2618720	Unknown	
THC2618883	THC2618883	THC2618883	Unknown	
THC2621536	THC2621536	THC2621536	Unknown	
THC2624264	ALU6_HUMAN (P39193) Alu subfamily SP sequence contamination warning entry, partial (9%)	THC2624264	Unknown	
THC2626445	THC2626445	THC2626445	Unknown	
THC2626557	THC2626557	THC2626557	Unknown	
THC2629174	THC2629174	THC2629174	Unknown	
THC2632174	THC2632174	THC2632174	Unknown	
THC2633747	ALU2_HUMAN (P39189) Alu subfamily SB sequence contamination warning entry, partial (3%)	THC2633747	Unknown	
THC2634713	THC2634713	THC2634713	Unknown	
THC2636164	ALU8_HUMAN (P39195) Alu subfamily SX sequence contamination warning entry, partial (6%)	THC2636164	Unknown	
THC2636523	THC2636523	THC2636523	Unknown	
THC2638025	THC2638025	THC2638025	Unknown	
THC2639806	THC2639806	THC2639806	Unknown	
THC2641484	THC2641484	THC2641484	Unknown	
THC2643320	Q306F7_HUMAN (Q306F7) Down syndrome encephalopathy related protein 1, partial (11%)	THC2643320	Unknown	
THC2643352	Q2RZ67_SALRD (Q2RZ67) Glycosyl transferase, group 1 family protein , partial (6%)	THC2643352	Unknown	
THC2644861	ALU1_HUMAN (P39188) Alu subfamily J sequence contamination warning entry, partial (11%)	THC2644861	Unknown	
THC2645690	BX089289 BX089289 Soares fetal liver spleen 1NFLS cDNA clone IMAGp998O17111 ; IMAGE:120808, mRNA sequence	THC2645690	Unknown	
THC2648397	ALU1_HUMAN (P39188) Alu subfamily J sequence contamination warning entry, partial (8%)	THC2648397	Unknown	
THC2648646	THC2648646	THC2648646	Unknown	
THC2649313	THC2649313	THC2649313	Unknown	
THC2649856	O63611_BALCA (O63611) NADH dehydrogenase subunit 2, partial (5%)	THC2649856	Unknown	
THC2650120	ALU6_HUMAN (P39193) Alu subfamily SP sequence contamination warning entry, partial (14%)	THC2650120	Unknown	
THC2650668	Q4RGI5_TETNG (Q4RGI5) Chromosome undetermined SCAF15099, whole genome shotgun sequence. (Fragment), partial (6%)	THC2650668	Unknown	
THC2651140	Q62JH7_BURMA (Q62JH7) PspA/IM30 family protein, partial (5%)	THC2651140	Unknown	
THC2651649	GRIP1_HUMAN (Q9Y3R0) Glutamate receptor-interacting protein 1 (GRIP1 protein), partial (82%)	THC2651649	Unknown	
THC2652075	Q35BU9_9BRAD (Q35BU9) Probable oligopeptide binding protein AppA precursor, partial (4%)	THC2652075	Unknown	
THC2652307	ALU8_HUMAN (P39195) Alu subfamily SX sequence contamination warning entry, partial (10%)	THC2652307	Unknown	
THC2652707	THC2652707	THC2652707	Unknown	
THC2653001	BX098637 BX098637 Soares fetal liver spleen 1NFLS cDNA clone IMAGp998F16386 ; IMAGE:200847, mRNA sequence	THC2653001	Unknown	
THC2654381	THC2654381	THC2654381	Unknown	

THC2654608	Q961E2_DROME (Q961E2) GM14238p, partial (8%)	THC2654608	Unknown	
THC2655094	XM_747355 succinyl-CoA synthetase {Aspergillus fumigatus Af293} (exp=-1; wgp=0; cg=0), partial (3%)	THC2655094	Unknown	
THC2655140	Q640D8_XENLA (Q640D8) LOC494721 protein, partial (5%)	THC2655140	Unknown	
THC2655396	THC2655396	THC2655396	Unknown	
THC2655842	BF476310 naa21a07.x1 NCI_CGAP_Pr28 cDNA clone IMAGE:3255444 3' similar to contains Alu repetitive element;contains element MIR MIR repetitive element ;, mRNA sequence	THC2655842	Unknown	
THC2656240	Q6PAX8_XENLA (Q6PAX8) MGC68553 protein, partial (16%)	THC2656240	Unknown	
THC2656479	KTNA1_MOUSE (Q9WV86) Katanin p60 ATPase-containing subunit A1 (Katanin p60 subunit A1) (p60 katanin) (Lipotransin) , partial (29%)	THC2656479	Unknown	
THC2657612	THC2657612	THC2657612	Unknown	
THC2658030	AF311284 erythroid membrane-associated protein {Homo sapiens} (exp=-1; wgp=0; cg=0), partial (25%)	THC2658030	Unknown	
THC2661968	Q7L4D9_HUMAN (Q7L4D9) RRN3 protein (Fragment), partial (16%)	THC2661968	Unknown	
THC2664480	ALU1_HUMAN (P39188) Alu subfamily J sequence contamination warning entry, partial (6%)	THC2664480	Unknown	
THC2665444	THC2665444	THC2665444	Unknown	
THC2667284	AL563955 AL563955 FETAL LIVER cDNA clone CS0DM001YO03 3-PRIME, mRNA sequence	THC2667284	Unknown	
THC2668629	THC2668629	THC2668629	Unknown	
THC2668907	BC010357 RSRC1 protein {Homo sapiens} (exp=-1; wgp=0; cg=0), partial (38%)	THC2668907	Unknown	
THC2670384	THC2670384	THC2670384	Unknown	
THC2671048	Q3DWD9_CHLAU (Q3DWD9) YLP motif, partial (6%)	THC2671048	Unknown	
THC2671126	M4A4D_MOUSE (Q99N05) Membrane-spanning 4-domains subfamily A member 4D, partial (5%)	THC2671126	Unknown	
THC2671553	THC2671553	THC2671553	Unknown	
THC2671553	THC2671553	THC2671553	Unknown	
THC2672768	THC2672768	THC2672768	Unknown	
THC2673973	THC2673973	THC2673973	Unknown	
THC2674354	Q36LB5_MARHY (Q36LB5) Nucleoside-diphosphate-sugar epimerases, partial (8%)	THC2674354	Unknown	
THC2674900	THC2674900	THC2674900	Unknown	
THC2675062	THC2675062	THC2675062	Unknown	
THC2676284	Q6DN14_HUMAN (Q6DN14) MCTP1L, partial (4%)	THC2676284	Unknown	
THC2677432	THC2677432	THC2677432	Unknown	
THC2677630	ALU7_HUMAN (P39194) Alu subfamily SQ sequence contamination warning entry, partial (10%)	THC2677630	Unknown	
THC2679021	THC2679021	THC2679021	Unknown	
THC2682280	THC2682280	THC2682280	Unknown	
THC2682560	THC2682560	THC2682560	Unknown	
THC2682884	THC2682884	THC2682884	Unknown	
THC2684143	THC2684143	THC2684143	Unknown	
THC2686753	THC2686753	THC2686753	Unknown	
THC2688670	THC2688670	THC2688670	Unknown	
THC2690347	O61886_CAEEL (O61886) Seven tm receptor protein 224, partial (5%)	THC2690347	Unknown	
THC2691517	THC2691517	THC2691517	Unknown	
THC2691929	THC2691929	THC2691929	Unknown	
THC2692269	NM_061085 C49A1.10 {Caenorhabditis elegans} (exp=-1; wgp=0; cg=0), partial (6%)	THC2692269	Unknown	
THC2693238	ALU1_HUMAN (P39188) Alu subfamily J sequence contamination warning entry, partial (9%)	THC2693238	Unknown	
THC2693741	THC2693741	THC2693741	Unknown	
THC2694186	THC2694186	THC2694186	Unknown	
THC2694827	U74612 forkhead box M1A {Homo sapiens} (exp=-1; wgp=0; cg=0), partial (3%)	THC2694827	Unknown	
THC2694873	THC2694873	THC2694873	Unknown	
THC2695400	THC2695400	THC2695400	Unknown	
THC2696143	THC2696143	THC2696143	Unknown	
THC2696614	AK223183 arylsulfatase E precursor variant {Homo sapiens} (exp=-1; wgp=0; cg=0), partial (19%)	THC2696614	Unknown	
THC2696831	THC2696831	THC2696831	Unknown	
THC2698177	Q99PF7_CRIGR (Q99PF7) Ribosomal protein S28 (Fragment), partial (32%)	THC2698177	Unknown	
THC2700133	THC2700133	THC2700133	Unknown	
THC2700191	O41021_PBCV1 (O41021) A312aR protein, partial (26%)	THC2700191	Unknown	

THC2701431	Q25EW1_MACFA (Q25EW1) Brain cDNA, clone: QmoA-11067, partial (63%)	THC2701431	Unknown	
THC2706230	THC2706230	THC2706230	Unknown	
THC2706736	THC2706736	THC2706736	Unknown	
THC2708549	N51961 yz07b02.s1 Soares_multiple_sclerosis_2NbHMSP cDNA clone IMAGE:282315 3', mRNA sequence	THC2708549	Unknown	
THC2708669	Q3IE62_PSEHT (Q3IE62) Dihydropteroate synthase (DHPS) (Dihydropteroate pyrophosphorylase) , partial (5%)	THC2708669	Unknown	
THC2708687	THC2708687	THC2708687	Unknown	
THC2712697	Q6JHZ7_HUMAN (Q6JHZ7) HCV-NS5ATP5 binding protein 1, partial (23%)	THC2712697	Unknown	
THC2713715	BQ188033 UI-E-EJ1-aju-o-13-0-UI.r1 UI-E-EJ1 cDNA clone UI-E-EJ1-aju-o-13-0-UI 5', mRNA sequence	THC2713715	Unknown	
THC2716080	THC2716080	THC2716080	Unknown	
THC2716274	AF195969 rho GTPase activating protein 8 isoform 2 {Homo sapiens} (exp=-1; wgp=0; cg=0), partial (11%)	THC2716274	Unknown	
THC2718499	Q5THX3_HUMAN (Q5THX3) Expressed in hematopoietic cells, heart, liver (HLL), partial (11%)	THC2718499	Unknown	
THC2720788	AF498955 small GTP binding protein RAB28 {Homo sapiens} (exp=-1; wgp=0; cg=0), partial (20%)	THC2720788	Unknown	
THC2721182	THC2721182	THC2721182	Unknown	
THC2724078	Q5U682_HUMAN (Q5U682) Tweety 1, isoform 2, partial (8%)	THC2724078	Unknown	
THC2725167	ALU1_HUMAN (P39188) Alu subfamily J sequence contamination warning entry, partial (13%)	THC2725167	Unknown	
THC2727164	THC2727164	THC2727164	Unknown	
THC2730567	THC2730567	THC2730567	Unknown	
THC2730708	ZNF73_HUMAN (O43830) Zinc finger protein 73 (Zinc finger protein 186) (hZNF2), complete	THC2730708	Unknown	
THC2732721	ARHG5_HUMAN (Q12774) Rho guanine nucleotide exchange factor 5 (Guanine nucleotide regulatory protein TIM) (Oncogene TIM) (p60 TIM) (Transforming immortalized mammary oncogene), partial (8%)	THC2732721	Unknown	
THC2734388	MMFXR1H9 fragile-X-related protein 1 isoform f {Mus musculus} (exp=-1; wgp=0; cg=0), partial (4%)	THC2734388	Unknown	
THC2735742	THC2735742	THC2735742	Unknown	
THC2743491	THC2743491	THC2743491	Unknown	
THC2746571	Q5I0G3_HUMAN (Q5I0G3) MDH1B protein, partial (43%)	THC2746571	Unknown	
THC2749235	THC2749235	THC2749235	Unknown	
THC2751913	THC2751913	THC2751913	Unknown	
THC2752542	THC2752542	THC2752542	Unknown	
THC2752592	THC2752592	THC2752592	Unknown	
THC2757340	THC2757340	THC2757340	Unknown	
THC2757602	THC2757602	THC2757602	Unknown	
THC2757997	THC2757997	THC2757997	Unknown	
THC2761472	Q8D3J9_WIGBR (Q8D3J9) AtpB protein, partial (5%)	THC2761472	Unknown	
THC2762149	BC037545 poly(ADP-ribosyl)transferase {Homo sapiens} (exp=-1; wgp=0; cg=0), partial (24%)	THC2762149	Unknown	
THC2762599	BI523298 603175578T1 NIH_MGC_121 cDNA clone IMAGE:5239842 3', mRNA sequence	THC2762599	Unknown	
THC2766373	THC2766373	THC2766373	Unknown	
THC2769953	THC2769953	THC2769953	Unknown	
THC2778165	THC2778165	THC2778165	Unknown	
THC2783023	Q8IUM9_HUMAN (Q8IUM9) ACSL3 protein, complete	THC2783023	Unknown	
THC2788120	AA258164 zs35b10.s1 NCI_CGAP_GCB1 cDNA clone IMAGE:687163 3', mRNA sequence	THC2788120	Unknown	
THG1L	interphase cytoplasmic foci protein 45	ENST00000231198	Unknown	
THOC2	THO complex 2, mRNA (cDNA clone IMAGE:5556338), partial cds.	BC054050	Hs.592243	NM_001081550
THUMPD1	THUMP domain containing 1 (THUMPD1), mRNA	NM_017736	Hs.460232	NM_017736
THUMPD1	THUMP domain containing 1 (THUMPD1), mRNA	NM_017736	Hs.460232	NM_017736
TIGD2	tigger transposable element derived 2 (TIGD2), mRNA	NM_145715	Hs.58924	NM_145715
TJP1	tight junction protein 1 (zona occludens 1) (TJP1), transcript variant 1, mRNA	NM_003257	Hs.510833	NM_003257
TMEM1	transmembrane protein 1 (TMEM1), transcript variant 1, mRNA	NM_003274	Hs.126221	NM_003274
TMEM106B	transmembrane protein 106B (TMEM106B), mRNA	NM_018374	Hs.396358	NM_018374
TMEM118	transmembrane protein 118 (TMEM118), mRNA	NM_032814	Hs.437195	AK094682
TMEM125	transmembrane protein 125 (TMEM125), mRNA	NM_144626	Hs.104476	BC072393
TMEM132B	transmembrane protein 132B (TMEM132B), mRNA	NM_052907	Hs.524838	NM_052907

TMEM132B	transmembrane protein 132B (TMEM132B), mRNA	NM_052907	Hs.524838	NM_052907
TMEM140	cDNA FLJ32348 fis, clone PROST2007200.	AK056910	Hs.567530	AK056910
TMEM145	transmembrane protein 145 (TMEM145), mRNA	NM_173633	Hs.382075	NM_173633
TMEM161B	transmembrane protein 161B (TMEM161B), mRNA	NM_153354	Hs.379972	BX647345
TMEM170	mRNA; cDNA DKFZp686O1555 (from clone DKFZp686O1555).	BX648484	Hs.487510	BX648484
TMEM18	transmembrane protein 18 (TMEM18), mRNA	NM_152834	Hs.43899	BC032379
TMEM30B	transmembrane protein 30B (TMEM30B), mRNA	NM_001017970	Hs.659339	NM_001017970
TMEM41B	transmembrane protein 41B, mRNA (cDNA clone MGC:33897 IMAGE:5259179), complete cds.	BC035034	Hs.594563	BC035034
TMEM41B	transmembrane protein 41B (TMEM41B), mRNA	NM_015012	Hs.594563	BC035034
TMEM46	transmembrane protein 46 (TMEM46), mRNA	NM_001007538	Hs.433791	NM_001007538
TMEM92	transmembrane protein 92 (TMEM92), mRNA	NM_153229	Hs.224630	AK090637
TMEM97	transmembrane protein 97 (TMEM97), mRNA	NM_014573	Hs.199695	NM_014573
TMSL8	thymosin-like 8 (TMSL8), mRNA	NM_021992	Hs.56145	BG471140
TMSL8	NB thymosin beta (Thymosin-like protein 8).	ENST00000372602	Unknown	
TNFRSF17	tumor necrosis factor receptor superfamily, member 17 (TNFRSF17), mRNA	NM_001192	Hs.2556	Z29575
TNKS	tankyrase, TRF1-interacting ankyrin-related ADP-ribose polymerase (TNKS), mRNA	NM_003747	Hs.370267	NM_003747
TNRC6B	trinucleotide repeat containing 6B (TNRC6B), transcript variant 1, mRNA	NM_015088	Hs.372082	NM_015088
TNRC6C	cDNA FLJ31859 fis, clone NT2RP7001231.	AK056421	Hs.584945	NM_018996
TNRC9	CAGF9 (Fragment).	ENST00000388816	Unknown	
TOP2B	topoisomerase (DNA) II beta 180kDa (TOP2B), mRNA	NM_001068	Hs.475733	NM_001068
TOPORS	topoisomerase I binding, arginine/serine-rich (TOPORS), mRNA	NM_005802	Hs.589962	NM_005802
TPD52	tumor protein D52 (TPD52), transcript variant 1, mRNA	NM_001025252	Hs.368433	NM_001025252
TRAF5	TNF receptor-associated factor 5 (TRAF5), transcript variant 1, mRNA	NM_004619	Hs.523930	AB000509
TREML4	triggering receptor expressed on myeloid cells-like 4 (TREML4), mRNA	NM_198153	Hs.434181	AK090633
TRIM24	tripartite motif-containing 24 (TRIM24), transcript variant 1, mRNA	NM_015905	Hs.490287	NM_015905
TRIM6	tripartite motif-containing 6 (TRIM6), transcript variant 1, mRNA	NM_001003818	Hs.125300	NM_001003819
TRIM6	tripartite motif-containing 6 (TRIM6), transcript variant 1, mRNA	NM_001003818	Hs.125300	NM_001003819
TRIT1	tRNA isopentenyltransferase 1 (TRIT1), mRNA	NM_017646	Hs.356554	AF074918
TRMT11	tRNA methyltransferase 11 homolog (S. cerevisiae) (TRMT11), mRNA	NM_001031712	Hs.404186	NM_001031712
TRMT11	tRNA methyltransferase 11 homolog (S. cerevisiae) (TRMT11), mRNA	NM_001031712	Hs.404186	NM_001031712
TRMT12	tRNA methyltransferase 12 homolog (S. cerevisiae) (TRMT12), mRNA	NM_017956	Hs.9925	AK000779
TRPC1	transient receptor potential cation channel, subfamily C, member 1 (TRPC1), mRNA	NM_003304	Hs.250687	X89066
TRRAP	transformation/transcription domain-associated protein (TRRAP), mRNA	NM_003496	Hs.203952	AF110377
TSGA13	testis specific, 13 (TSGA13), mRNA	NM_052933	Hs.592266	AK093329
TSN	translin (TSN), mRNA	NM_004622	Hs.75066	NM_004622
TTC19	tetratricopeptide repeat domain 19 (TTC19), mRNA	NM_017775	Hs.462316	AK094819
TTC35	tetratricopeptide repeat domain 35 (TTC35), mRNA	NM_014673	Hs.654351	AK057571
TTF1	transcription termination factor, RNA polymerase I (TTF1), mRNA	NM_007344	Hs.54780	NM_007344
TTMB	TTMB protein (TTMB), mRNA	NM_001003682	Hs.531492	AL110282
TUBB2B	tubulin, beta 2B (TUBB2B), mRNA	NM_178012	Hs.300701	CR625172
TUBD1	tubulin, delta 1 (TUBD1), mRNA	NM_016261	Hs.463638	NM_016261
TXNDC3	thioredoxin domain containing 3 (spermatozoa) (TXNDC3), mRNA	NM_016616	Hs.563491	NM_017549
TYRO3	TYRO3 protein tyrosine kinase (TYRO3), mRNA	NM_006293	Hs.381282	NM_006293
U52054	Human S6 H-8 mRNA expressed in chromosome 6-suppressed melanoma cells.	U52054	Hs.561411	BM564371
U69195	U69195 Soares infant brain 1NIB cDNA clone 32996, mRNA sequence	U69195	Hs.697380	U69195
UBE2D2	ubiquitin-conjugating enzyme E2D 2 (UBC4/5 homolog, yeast) (UBE2D2), transcript variant 2, mRNA	NM_181838	Hs.108332	NM_181838
UBE4B	ubiquitination factor E4B (UFD2 homolog, yeast) (UBE4B), mRNA	NM_006048	Hs.632370	NM_006048
UBP1	upstream binding protein 1 (LBP-1a) (UBP1), mRNA	NM_014517	Hs.696578	CR749798
UBXD6	UBX domain containing 6 (UBXD6), mRNA	NM_005671	Hs.153678	D83767
UGT1A8	UDP glucuronosyltransferase 1 family, polypeptide A8 (UGT1A8), mRNA	NM_019076	Hs.654499	NM_001072
UGT2B28	UDP glucuronosyltransferase 2 family, polypeptide B28 (UGT2B28), mRNA	NM_053039	Hs.653154	AF177272
UNC93A	unc-93 homolog A (C. elegans) (UNC93A), mRNA	NM_018974	Hs.567508	AK091987

UNQ467	KIPV467 (UNQ467), mRNA	NM_207392	Hs.112457	AJ293408
UPF3B	UPF3 regulator of nonsense transcripts homolog B (yeast) (UPF3B), transcript variant 1, mRNA	NM_080632	Hs.103832	NM_080632
UPP2	uridine phosphorylase 2 (UPP2), mRNA	NM_173355	Hs.128427	AK122743
USP25	ubiquitin specific peptidase 25 (USP25), mRNA	NM_013396	Hs.473370	AL833500
USP32	ubiquitin specific peptidase 32 (USP32), mRNA	NM_032582	Hs.132868	NM_032582
USP37	ubiquitin specific peptidase 37 (USP37), mRNA	NM_020935	Hs.166068	BX538024
USP37	ubiquitin specific peptidase 37 (USP37), mRNA	NM_020935	Hs.166068	BX538024
USP43	cDNA FLJ33502 fis, clone BRAMY2004492, weakly similar to UBIQUITIN CARBOXYL-TERMINAL HYDROLASE 4 (EC 3.1.2.15).	AK090821	Unknown	
USP46	ubiquitin specific peptidase 46 (USP46), mRNA	NM_022832	Hs.331478	NM_022832
USP46	ubiquitin specific peptidase 46 (USP46), mRNA	NM_022832	Hs.331478	NM_022832
USP48	ubiquitin specific peptidase 48 (USP48), transcript variant 1, mRNA	NM_032236	Hs.467524	NM_032236
USP51	ubiquitin specific peptidase 51 (USP51), mRNA	NM_201286	Hs.134289	NM_201286
USP54	ubiquitin specific peptidase 54, mRNA (cDNA clone IMAGE:6503621), with apparent retained intron.	BC110845	Hs.657355	NM_152586
USP54	cDNA FLJ45138 fis, clone BRAWH3039258.	AK127081	Unknown	
USP6	ubiquitin specific peptidase 6 (Tre-2 oncogene) (USP6), mRNA	NM_004505	Hs.448851	BX647719
USP8	ubiquitin specific peptidase 8 (USP8), mRNA	NM_005154	Hs.644563	D29956
VASH2	vasohibin 2 (VASH2), mRNA	NM_024749	Hs.96885	BC051856
VAV3	vav 3 oncogene (VAV3), transcript variant 1, mRNA	NM_006113	Hs.267659	NM_006113
VCX	variable charge, X-linked (VCX), mRNA	NM_013452	Hs.567503	AF167081
VCX2	variable charge, X-linked 2 (VCX2), mRNA	NM_016378	Hs.279737	AF167079
VCX3A	variable charge, X-linked 3A (VCX3A), mRNA	NM_016379	Hs.278906	AF167078
VEZF1	vascular endothelial zinc finger 1 (VEZF1), mRNA	NM_007146	Hs.463569	NM_007146
VIL1	villin 1 (VIL1), mRNA	NM_007127	Hs.654595	AK223398
VIL2	UG0651E06 mRNA, complete cds.	AF351612	Hs.663419	AF351612
VPS4B	vacuolar protein sorting 4 homolog B (S. cerevisiae) (VPS4B), mRNA	NM_004869	Hs.126550	NM_004869
WAC	WW domain containing adaptor with coiled-coil (WAC), transcript variant 2, mRNA	NM_100264	Hs.435610	AK091453
WAC	WW domain-containing adapter protein with coiled-coil.	ENST00000375664	Unknown	
WAPAL	wings apart-like homolog (Drosophila) (WAPAL), mRNA	NM_015045	Hs.203099	AB065003
WDHD1	WD repeat and HMG-box DNA binding protein 1 (WDHD1), transcript variant 1, mRNA	NM_007086	Hs.385998	NM_007086
WDHD1	WD repeat and HMG-box DNA binding protein 1 (WDHD1), transcript variant 1, mRNA	NM_007086	Hs.385998	NM_007086
WDHD1	WD repeat and HMG-box DNA binding protein 1 (WDHD1), transcript variant 1, mRNA	NM_007086	Hs.385998	NM_007086
WDHD1	WD repeat and HMG-box DNA binding protein 1 (WDHD1), transcript variant 1, mRNA	NM_007086	Hs.385998	NM_007086
WDHD1	WD repeat and HMG-box DNA binding protein 1 (WDHD1), transcript variant 1, mRNA	NM_007086	Hs.385998	NM_007086
WDHD1	WD repeat and HMG-box DNA binding protein 1 (WDHD1), transcript variant 1, mRNA	NM_007086	Hs.385998	NM_007086
WDHD1	WD repeat and HMG-box DNA binding protein 1 (WDHD1), transcript variant 1, mRNA	NM_007086	Hs.385998	NM_007086
WDHD1	WD repeat and HMG-box DNA binding protein 1 (WDHD1), transcript variant 1, mRNA	NM_007086	Hs.385998	NM_007086
WDHD1	WD repeat and HMG-box DNA binding protein 1 (WDHD1), transcript variant 1, mRNA	NM_007086	Hs.385998	NM_007086
WDHD1	WD repeat and HMG-box DNA binding protein 1 (WDHD1), transcript variant 1, mRNA	NM_007086	Hs.385998	NM_007086
WDR21A	WD repeat domain 21A (WDR21A), transcript variant 2, mRNA	NM_181340	Hs.331491	AL080157
WDR21A	WD repeat domain 21A (WDR21A), transcript variant 2, mRNA	NM_181340	Hs.331491	AL080157
WDR59	WD repeat domain 59 (WDR59), mRNA	NM_030581	Hs.280951	NM_030581
WDR67	WD repeat domain 67 (WDR67), mRNA	NM_145647	Hs.492716	AK056434
WDR72	WD repeat domain 72 (WDR72), mRNA	NM_182758	Hs.208067	BX648571
WDR86	cDNA FLJ38667 fis, clone HLUNG2006843.	AK095986	Hs.659231	AK095986
WDSOF1	WD repeats and SOF1 domain containing (WDSOF1), mRNA	NM_015420	Hs.532265	AK001693
WFDC2	WAP four-disulfide core domain 2 (WFDC2), transcript variant 2, mRNA	NM_080736	Unknown	
WFDC2	WAP four-disulfide core domain 2 (WFDC2), transcript variant 4, mRNA	NM_080734	Unknown	
WFIKKN1	WAP, follistatin/kazal, immunoglobulin, kunitz and netrin domain containing 1 (WFIKKN1), mRNA	NM_053284	Hs.345818	AK075356
WIF1	WNT inhibitory factor 1 (WIF1), mRNA	NM_007191	Hs.284122	AY358344
WIPI2	WD repeat domain, phosphoinositide interacting 2 (WIPI2), transcript variant 1, mRNA	NM_015610	Hs.122363	NM_015610
WNK3	Serine/threonine-protein kinase WNK3 (EC 2.7.11.1) (Protein kinase with no lysine 3) (Protein kinase, lysine-deficient 3).	ENST00000375169	Unknown	

WNK3	WNK lysine deficient protein kinase 3 (WNK3), transcript variant 1, mRNA	NM_020922	Hs.92423	NM_020922
WWOX	WW domain containing oxidoreductase (WWOX), transcript variant 1, mRNA	NM_016373	Hs.461453	NM_016373
X15675	Human pTR7 mRNA for repetitive sequence.	X15675	Hs.569018	M85205
XCL1	chemokine (C motif) ligand 1 (XCL1), mRNA	NM_002995	Hs.546295	NM_002995
XCL2	chemokine (C motif) ligand 2 (XCL2), mRNA	NM_003175	Hs.458346	BC070308
XPO1	exportin 1 (CRM1 homolog, yeast) (XPO1), mRNA	NM_003400	Hs.370770	AL833550
YEATS4	YEATS domain containing 4 (YEATS4), mRNA	NM_006530	Hs.4029	BX640958
YME1L1	YME1-like 1 (S. cerevisiae) (YME1L1), nuclear gene encoding mitochondrial protein, transcript variant 2, mRNA	NM_139313	Unknown	
YPEL1	yippee-like 1 (Drosophila) (YPEL1), mRNA	NM_013313	Hs.517436	CR933630
YTHDC1	YTH domain containing 1 (YTHDC1), transcript variant 1, mRNA	NM_001031732	Hs.175955	NM_001031732
YTHDC1	YTH domain containing 1 (YTHDC1), transcript variant 1, mRNA	NM_001031732	Hs.175955	NM_001031732
YTHDF1	YTH domain family, member 1 (YTHDF1), mRNA	NM_017798	Hs.11747	BC050284
YTHDF2	YTH domain family, member 2 (YTHDF2), mRNA	NM_016258	Hs.532286	NM_016258
YWHAZ	tyrosine 3-monooxygenase/tryptophan 5-monooxygenase activation protein, zeta polypeptide (YWHAZ), transcript variant 2, mRNA	NM_145690	Hs.492407	BC051814
ZBED4	zinc finger, BED-type containing 4 (ZBED4), mRNA	NM_014838	Hs.475208	NM_014838
ZBTB8OS	zinc finger and BTB domain containing 8 opposite strand (ZBTB8OS), mRNA	NM_178547	Hs.655921	BM926729
ZBTB8OS	cDNA FLJ31988 fis, clone NT2RP7008863.	AK056550	Hs.16003	AK056550
ZC3H15	zinc finger CCCH-type containing 15 (ZC3H15), mRNA	NM_018471	Hs.368598	NM_018471
ZC3H5	zinc finger CCCH-type containing 5, mRNA (cDNA clone IMAGE:6012927), partial cds.	BC053362	Hs.655493	NM_001080419
ZC3H6	zinc finger CCCH-type containing 6 (ZC3H6), mRNA	NM_198581	Hs.190477	NM_198581
ZCCHC10	zinc finger, CCHC domain containing 10 (ZCCHC10), mRNA	NM_017665	Hs.29700	AK122970
ZCCHC17	zinc finger, CCHC domain containing 17 (ZCCHC17), mRNA	NM_016505	Hs.524094	NM_016505
ZCCHC3	zinc finger, CCHC domain containing 3 (ZCCHC3), mRNA	NM_033089	Hs.28608	BC069238
ZCCHC7	zinc finger, CCHC domain containing 7 (ZCCHC7), mRNA	NM_032226	Hs.654700	AK126219
ZCCHC7	zinc finger, CCHC domain containing 7 (ZCCHC7), mRNA	NM_032226	Hs.654700	AK126219
ZCCHC8	zinc finger, CCHC domain containing 8 (ZCCHC8), mRNA	NM_017612	Hs.37706	NM_017612
ZCCHC9	zinc finger, CCHC domain containing 9 (ZCCHC9), mRNA	NM_032280	Hs.15536	AL512712
ZFAND6	zinc finger, AN1-type domain 6 (ZFAND6), mRNA	NM_019006	Hs.654787	CR749384
ZFHX2	zinc finger homeobox 2 (ZFHX2), mRNA	NM_033400	Unknown	
ZFP37	zinc finger protein 37 homolog (mouse) (ZFP37), mRNA	NM_003408	Hs.150406	AF022158
ZFP62	cDNA FLJ34231 fis, clone FCBBF3025905, highly similar to Mus musculus (clone pMLZ-1) zinc finger protein (Zfp) mRNA.	AK091550	Hs.509227	AL832408
ZIC3	Zic family member 3 heterotaxy 1 (odd-paired homolog, Drosophila) (ZIC3), mRNA	NM_003413	Hs.111227	NM_003413
ZIC5	Zic family member 5 (odd-paired homolog, Drosophila) (ZIC5), mRNA	NM_033132	Hs.508570	NM_033132
ZMAT1	zinc finger, matrin type 1 (ZMAT1), transcript variant 3, mRNA	NM_032441	Hs.496512	NM_032441
ZMYM1	zinc finger, MYM-type 1 (ZMYM1), mRNA	NM_024772	Hs.471243	CR936813
ZMYM1	zinc finger, MYM-type 1 (ZMYM1), mRNA	NM_024772	Hs.471243	CR936813
ZMYM2	mRNA; cDNA DKFZp564B162 (from clone DKFZp564B162).	AL136621	Hs.644041	BX648905
ZMYM2	zinc finger, MYM-type 2 (ZMYM2), mRNA	NM_003453	Hs.644041	BX648905
ZNF10	zinc finger protein 10 (ZNF10), mRNA	NM_015394	Hs.507355	BC024182
ZNF100	zinc finger protein 100 (ZNF100), mRNA	NM_173531	Hs.365142	BC035579
ZNF100	zinc finger protein 100 (ZNF100), mRNA	NM_173531	Hs.365142	BC035579
ZNF114	zinc finger protein 114 (ZNF114), mRNA	NM_153608	Hs.511883	AK075062
ZNF117	zinc finger protein 117 (ZNF117), mRNA	NM_024498	Unknown	
ZNF131	zinc finger protein 131 (ZNF131), mRNA	NM_003432	Hs.535804	NM_003432
ZNF133	zinc finger protein 133 (ZNF133), mRNA	NM_003434	Hs.472221	NM_003434
ZNF138	zinc finger protein 138 (ZNF138), mRNA	NM_006524	Hs.184080	AL834121
ZNF143	zinc finger protein 143 (ZNF143), mRNA	NM_003442	Hs.523471	NM_003442
ZNF146	zinc finger protein 146 (ZNF146), mRNA	NM_007145	Hs.643436	NM_007145
ZNF160	zinc finger protein 160 (ZNF160), transcript variant 2, mRNA	NM_198893	Hs.655967	NM_198893
ZNF165	zinc finger protein 165 (ZNF165), mRNA	NM_003447	Hs.535177	NM_003447
ZNF17	zinc finger protein 17 (ZNF17), mRNA	NM_006959	Hs.657353	BC030788

ZNF180	zinc finger protein 180 (ZNF180), mRNA	NM_013256	Hs.130683	BC051903
ZNF195	zinc finger protein 195 (ZNF195), mRNA	NM_007152	Hs.386294	AL833722
ZNF195	zinc finger protein 195 (ZNF195), mRNA	NM_007152	Hs.386294	AL833722
ZNF195	zinc finger protein 195 (ZNF195), mRNA	NM_007152	Hs.386294	AL833722
ZNF195	zinc finger protein 195 (ZNF195), mRNA	NM_007152	Hs.386294	AL833722
ZNF195	zinc finger protein 195 (ZNF195), mRNA	NM_007152	Hs.386294	AL833722
ZNF195	zinc finger protein 195 (ZNF195), mRNA	NM_007152	Hs.386294	AL833722
ZNF195	zinc finger protein 195 (ZNF195), mRNA	NM_007152	Hs.386294	AL833722
ZNF195	zinc finger protein 195 (ZNF195), mRNA	NM_007152	Hs.386294	AL833722
ZNF195	zinc finger protein 195 (ZNF195), mRNA	NM_007152	Hs.386294	AL833722
ZNF195	zinc finger protein 195 (ZNF195), mRNA	NM_007152	Hs.386294	AL833722
ZNF197	zinc finger protein 197 (ZNF197), transcript variant 1, mRNA	NM_006991	Hs.157035	NM_006991
ZNF20	zinc finger protein 20 (ZNF20), mRNA	NM_021143	Hs.512823	NM_021143
ZNF208	zinc finger protein 208 (ZNF208), mRNA	NM_007153	Hs.660982	NM_007153
ZNF212	zinc finger protein 212 (ZNF212), mRNA	NM_012256	Hs.490510	AK074821
ZNF226	zinc finger protein 226 (ZNF226), transcript variant 1, mRNA	NM_001032372	Hs.145956	BX648775
ZNF232	zinc finger protein 232 (ZNF232), mRNA	NM_014519	Hs.279914	NM_014519
ZNF233	zinc finger protein 233 (ZNF233), mRNA	NM_181756	Hs.466891	AK055310
ZNF254	zinc finger protein 254 (ZNF254), transcript variant 1, mRNA	NM_203282	Hs.434406	BC043147
ZNF254	zinc finger protein 254 (ZNF254), transcript variant 1, mRNA	NM_203282	Hs.434406	BC043147
ZNF254	zinc finger protein 254 (ZNF254), transcript variant 1, mRNA	NM_203282	Hs.434406	BC043147
ZNF256	zinc finger protein 256 (ZNF256), mRNA	NM_005773	Hs.596242	NM_005773
ZNF26	zinc finger protein 26 (ZNF26), mRNA	NM_019591	Hs.489608	AK122941
ZNF268	H.sapiens HZF3 mRNA for zinc finger protein.	X78926	Hs.654533	BC142989
ZNF273	zinc finger protein 273 (ZNF273), transcript variant 1, mRNA	NM_021148	Hs.520889	NM_021148
ZNF281	zinc finger protein 281 (ZNF281), mRNA	NM_012482	Hs.59757	BC060820
ZNF286	zinc finger protein 286 (ZNF286), mRNA	NM_020652	Hs.660199	AF217226
ZNF292	Zinc finger protein 292.	ENST00000339907	Unknown	
ZNF294	zinc finger protein 294 (ZNF294), mRNA	NM_015565	Hs.288773	NM_015565
ZNF295	zinc finger protein 295 (ZNF295), mRNA	NM_020727	Hs.434947	NM_001098402
ZNF3	zinc finger protein 3 (ZNF3), transcript variant 2, mRNA	NM_032924	Hs.435302	BX647182
ZNF304	zinc finger protein 304 (ZNF304), mRNA	NM_020657	Hs.287374	AJ276316
ZNF304	zinc finger protein 304 (ZNF304), mRNA	NM_020657	Hs.287374	AJ276316
ZNF320	zinc finger protein 320 (ZNF320), mRNA	NM_207333	Hs.446907	NM_207333
ZNF334	zinc finger protein 334 (ZNF334), transcript variant 2, mRNA	NM_199441	Hs.584933	BC026321
ZNF397	zinc finger protein (ZNF397) mRNA, complete cds.	AF533250	Unknown	
ZNF423	zinc finger protein 423 (ZNF423), mRNA	NM_015069	Hs.530930	NM_015069
ZNF429	zinc finger protein 429 (ZNF429), mRNA	NM_001001415	Hs.656558	NM_001076678
ZNF43	zinc finger protein 43 (ZNF43), mRNA	NM_003423	Hs.534365	NM_003423
ZNF430	zinc finger protein 430 (ZNF430), mRNA	NM_025189	Hs.466289	AL832422
ZNF430	zinc finger protein 430 (ZNF430), mRNA	NM_025189	Hs.466289	AL832422
ZNF431	zinc finger protein 431 (ZNF431), mRNA	NM_133473	Hs.696753	NM_133473
ZNF443	zinc finger protein 443 (ZNF443), mRNA	NM_005815	Hs.631623	BC032753
ZNF449	zinc finger protein 449 (ZNF449), mRNA	NM_152695	Hs.28780	BX537604
ZNF451	zinc finger protein 451 (ZNF451), transcript variant 1, mRNA	NM_001031623	Hs.485628	NM_001031623
ZNF452	zinc finger protein 452 (ZNF452), mRNA	NM_052923	Hs.176980	AY517631
ZNF462	zinc finger protein 462 (ZNF462), mRNA	NM_021224	Hs.370379	BX648965
ZNF484	zinc finger protein 484 (ZNF484), transcript variant 2, mRNA	NM_001007101	Hs.668378	NM_031486
ZNF486	zinc finger protein 486, mRNA (cDNA clone IMAGE:2819829), partial cds.	BC008936	Hs.590991	AB209550
ZNF493	zinc finger protein 493 (ZNF493), transcript variant 3, mRNA	NM_001076678	Hs.656558	NM_001076678
ZNF493	zinc finger protein 493 (ZNF493), transcript variant 3, mRNA	NM_001076678	Hs.656558	NM_001076678
ZNF493	zinc finger protein 493 (ZNF493), transcript variant 3, mRNA	NM_001076678	Hs.656558	NM_001076678

ZNF508	zinc finger protein 508 (ZNF508), mRNA	NM_014913	Hs.131915	BC071589
ZNF518	zinc finger protein 518 (ZNF518), mRNA	NM_014803	Hs.657337	NM_014803
ZNF518	zinc finger protein 518 (ZNF518), mRNA	NM_014803	Hs.657337	NM_014803
ZNF519	zinc finger protein 519 (ZNF519), mRNA	NM_145287	Hs.352635	AB209417
ZNF545	zinc finger protein 545 (ZNF545), mRNA	NM_133466	Hs.558734	AB075828
ZNF545	mRNA for KIAA1948 protein.	AB075828	Hs.558734	AB075828
ZNF549	zinc finger protein 549 (ZNF549), mRNA	NM_153263	Hs.564295	AL833090
ZNF562	zinc finger protein 562 (ZNF562), mRNA	NM_017656	Hs.655594	AK000086
ZNF567	zinc finger protein 567 (ZNF567), mRNA	NM_152603	Hs.697242	AK126691
ZNF569	zinc finger protein 569 (ZNF569), mRNA	NM_152484	Hs.511848	AL833408
ZNF571	zinc finger protein 571 (ZNF571), mRNA	NM_016536	Hs.590944	BC114479
ZNF588	zinc finger protein 588 (ZNF588), transcript variant 1, mRNA	NM_016220	Hs.50216	NM_016220
ZNF594	mRNA for KIAA1871 protein, partial cds.	AB058774	Hs.699950	AB058774
ZNF608	zinc finger protein 608 (ZNF608), mRNA	NM_020747	Hs.700803	BC151226
ZNF613	cDNA FLJ13590 fis, clone PLACE1009398, moderately similar to ZINC FINGER PROTEIN 135.	AK023652	Hs.183390	BC057776
ZNF616	zinc finger protein 616, mRNA (cDNA clone MGC:45556 IMAGE:4186857), complete cds.	BC032805	Hs.645225	BM544555
ZNF638	zinc finger protein 638 (ZNF638), transcript variant 1, mRNA	NM_014497	Hs.434401	CR749322
ZNF643	zinc finger protein 643 (ZNF643), mRNA	NM_023070	Hs.133034	AK091612
ZNF644	zinc finger protein 644 (ZNF644), transcript variant 1, mRNA	NM_201269	Hs.173001	BC150177
ZNF66	cDNA FLJ16537 fis, clone OCBBF2032274, moderately similar to Zinc finger protein 93.	AK131420	Hs.658748	AK131420
ZNF660	zinc finger protein 660 (ZNF660), mRNA	NM_173658	Hs.669324	BX538171
ZNF675	zinc finger protein 675 (ZNF675), mRNA	NM_138330	Hs.264345	AB209601
ZNF678	zinc finger protein 678 (ZNF678), mRNA	NM_178549	Hs.656372	AK026475
ZNF680	zinc finger protein 680 (ZNF680), mRNA	NM_178558	Hs.520886	BC030700
ZNF681	zinc finger protein 681 (ZNF681), mRNA	NM_138286	Hs.666326	AK122869
ZNF690	zinc finger protein 690 (ZNF690), mRNA	NM_152455	Hs.418287	NM_152455
ZNF691	zinc finger protein 691 (ZNF691), mRNA	NM_015911	Hs.20879	AK095184
ZNF708	zinc finger protein 708 (ZNF708), mRNA	NM_021269	Hs.466296	AK131527
ZNF708	zinc finger protein 708 (ZNF708), mRNA	NM_021269	Hs.466296	AK131527
ZNF714	zinc finger protein 714 (ZNF714), mRNA	NM_182515	Hs.466291	NM_182515
ZNF714	zinc finger protein 714 (ZNF714), mRNA	NM_182515	Hs.466291	NM_182515
ZNF721	zinc finger protein 721 (ZNF721), mRNA	NM_133474	Hs.428360	NM_133474
ZNF738	zinc finger protein 738, mRNA (cDNA clone IMAGE:4838161), complete cds.	BC034499	Hs.359535	BC034499
ZNF738	PREDICTED: hypothetical protein LOC148203 (LOC148203), misc RNA	XR_015756	Unknown	
ZNF761	zinc finger protein 761 (ZNF761), mRNA	NM_001008401	Hs.699590	BX647981
ZNF84	zinc finger protein 84 (ZNF84), mRNA	NM_003428	Hs.654730	BC036656
ZNF85	zinc finger protein 85 (ZNF85), mRNA	NM_003429	Hs.37138	BC036394
ZNF91	zinc finger protein 91 (ZNF91), mRNA	NM_003430	Hs.654471	NM_003430
ZNF92	zinc finger protein 92 (ZNF92), transcript variant 1, mRNA	NM_007139	Hs.9521	NM_152626
ZNF92	zinc finger protein 92 (ZNF92), transcript variant 1, mRNA	NM_007139	Hs.9521	NM_152626
ZNF99	PREDICTED: zinc finger protein 99 (ZNF99), mRNA	XM_001132267	Unknown	
ZNHIT3	zinc finger, HIT type 3 (ZNHIT3), transcript variant 2, mRNA	NM_004773	Hs.2210	CD299213
ZNRF3	mRNA for KIAA1133 protein, partial cds.	AB051436	Hs.655242	AB051436
ZRANB3	zinc finger, RAN-binding domain containing 3, mRNA (cDNA clone IMAGE:5575956), complete cds.	BC064616	Hs.658422	BX647838
ZRANB3	zinc finger, RAN-binding domain containing 3 (ZRANB3), mRNA	NM_032143	Hs.658422	BX647838
ZRF1	zuotin related factor 1 (ZRF1), mRNA	NM_014377	Hs.558476	NM_014377
A_23_P121234	A_23_P121234	A_23_P121234	Unknown	
A_23_P135063	A_23_P135063	A_23_P135063	Unknown	
A_23_P13535	A_23_P13535	A_23_P13535	Unknown	
A_23_P15226	A_23_P15226	A_23_P15226	Unknown	
A_23_P28927	A_23_P28927	A_23_P28927	Unknown	
A_23_P4462	A_23_P4462	A_23_P4462	Unknown	

A_23_P45902	A_23_P45902	A_23_P45902	Unknown	
A_23_P50195	A_23_P50195	A_23_P50195	Unknown	
A_23_P62857	A_23_P62857	A_23_P62857	Unknown	
A_23_P96035	A_23_P96035	A_23_P96035	Unknown	
A_24_P108281	A_24_P108281	A_24_P108281	Unknown	
A_24_P136155	A_24_P136155	A_24_P136155	Unknown	
A_24_P174353	A_24_P174353	A_24_P174353	Unknown	
A_24_P195724	A_24_P195724	A_24_P195724	Unknown	
A_24_P247616	A_24_P247616	A_24_P247616	Unknown	
A_24_P314290	A_24_P314290	A_24_P314290	Unknown	
A_24_P367282	A_24_P367282	A_24_P367282	Unknown	
A_24_P384069	A_24_P384069	A_24_P384069	Unknown	
A_24_P409681	A_24_P409681	A_24_P409681	Unknown	
A_24_P476718	A_24_P476718	A_24_P476718	Unknown	
A_24_P482189	A_24_P482189	A_24_P482189	Unknown	
A_24_P492885	A_24_P492885	A_24_P492885	Unknown	
A_24_P52168	A_24_P52168	A_24_P52168	Unknown	
A_24_P539080	A_24_P539080	A_24_P539080	Unknown	
A_24_P543460	A_24_P543460	A_24_P543460	Unknown	
A_24_P556328	A_24_P556328	A_24_P556328	Unknown	
A_24_P58899	A_24_P58899	A_24_P58899	Unknown	
A_24_P593724	A_24_P593724	A_24_P593724	Unknown	
A_24_P636834	A_24_P636834	A_24_P636834	Unknown	
A_24_P666795	A_24_P666795	A_24_P666795	Unknown	
A_24_P676566	A_24_P676566	A_24_P676566	Unknown	
A_24_P703803	A_24_P703803	A_24_P703803	Unknown	
A_24_P717305	A_24_P717305	A_24_P717305	Unknown	
A_24_P814872	A_24_P814872	A_24_P814872	Unknown	
A_24_P868992	A_24_P868992	A_24_P868992	Unknown	
A_24_P88493	A_24_P88493	A_24_P88493	Unknown	
A_24_P923510	A_24_P923510	A_24_P923510	Unknown	
A_24_P928453	A_24_P928453	A_24_P928453	Unknown	
A_24_P929533	A_24_P929533	A_24_P929533	Unknown	
A_24_P931804	A_24_P931804	A_24_P931804	Unknown	
A_24_P932046	A_24_P932046	A_24_P932046	Unknown	
A_24_P93397	A_24_P93397	A_24_P93397	Unknown	
A_24_P935852	A_24_P935852	A_24_P935852	Unknown	
A_24_P942661	A_24_P942661	A_24_P942661	Unknown	
A_32_P106246	A_32_P106246	A_32_P106246	Unknown	
A_32_P107859	A_32_P107859	A_32_P107859	Unknown	
A_32_P124887	A_32_P124887	A_32_P124887	Unknown	
A_32_P130217	A_32_P130217	A_32_P130217	Unknown	
A_32_P131583	A_32_P131583	A_32_P131583	Unknown	
A_32_P13852	A_32_P13852	A_32_P13852	Unknown	
A_32_P145302	A_32_P145302	A_32_P145302	Unknown	
A_32_P14737	A_32_P14737	A_32_P14737	Unknown	
A_32_P147734	A_32_P147734	A_32_P147734	Unknown	
A_32_P154956	A_32_P154956	A_32_P154956	Unknown	
A_32_P159289	A_32_P159289	A_32_P159289	Unknown	
A_32_P162443	A_32_P162443	A_32_P162443	Unknown	
A_32_P187715	A_32_P187715	A_32_P187715	Unknown	
A_32_P190944	A_32_P190944	A_32_P190944	Unknown	

A_32_P19460	A_32_P19460	A_32_P19460	Unknown	
A_32_P205792	A_32_P205792	A_32_P205792	Unknown	
A_32_P206917	A_32_P206917	A_32_P206917	Unknown	
A_32_P232647	A_32_P232647	A_32_P232647	Unknown	
A_32_P28158	A_32_P28158	A_32_P28158	Unknown	
A_32_P31827	A_32_P31827	A_32_P31827	Unknown	
A_32_P38228	A_32_P38228	A_32_P38228	Unknown	
A_32_P3914	A_32_P3914	A_32_P3914	Unknown	
A_32_P42149	A_32_P42149	A_32_P42149	Unknown	
A_32_P429046	A_32_P429046	A_32_P429046	Unknown	
A_32_P55438	A_32_P55438	A_32_P55438	Unknown	
A_32_P59990	A_32_P59990	A_32_P59990	Unknown	
A_32_P67303	A_32_P67303	A_32_P67303	Unknown	
A_32_P71016	A_32_P71016	A_32_P71016	Unknown	
A_32_P80295	A_32_P80295	A_32_P80295	Unknown	
A_32_P86494	A_32_P86494	A_32_P86494	Unknown	
A_32_P99744	A_32_P99744	A_32_P99744	Unknown	

Supplemental Table B3

K-Means Cluster 3 -- 1009 genes

Gene Name	Description	Acc	UGCluster	UGRepAcc
A1BG	alpha-1-B glycoprotein (A1BG), mRNA	NM_130786	Hs.529161	NM_130786
AA432215	zw75f06.r1 Soares_testis_NHT cDNA clone IMAGE:782051 5', mRNA sequence	AA432215	Hs.98588	AA432215
AA550817	AA550817 nj34c01.s1 NCI_CGAP_AA1 cDNA clone IMAGE:994368 3' similar to contains element MER16 MER16 repetitive element ;, mRNA sequence	AA550817	Hs.659665	AK022035
AB058742	mRNA for KIAA1839 protein, partial cds.	AB058742	Unknown	
ABCD3	ATP-binding cassette, sub-family D (ALD), member 3 (ABCD3), mRNA	NM_002858	Hs.700576	BX648715
ABHD8	abhydrolase domain containing 8 (ABHD8), mRNA	NM_024527	Hs.515664	NM_024527
ABR	active BCR-related gene (ABR), transcript variant 1, mRNA	NM_021962	Hs.159306	AK124547
ACOT11	Acyl-coenzyme A thioesterase 11 (EC 3.1.2.-) (Acyl-CoA thioesterase 11) (Acyl-CoA thioester hydrolase 11) (Brown fat-inducible thioesterase) (BFIT) (Adipose-associated thioesterase).	ENST00000371316	Unknown	
ADA	adenosine deaminase (ADA), mRNA	NM_000022	Hs.654536	BM809993
ADAMDEC1	ADAM-like, decysin 1 (ADAMDEC1), mRNA	NM_014479	Hs.521459	Y13323
ADC	arginine decarboxylase (ADC), mRNA	NM_052998	Hs.101807	AL832640
ADCYAP1	Pituitary adenylate cyclase-activating polypeptide precursor (PACAP) [Contains: PACAP-related peptide (PRP-48); Pituitary adenylate cyclase-activating polypeptide 27 (PACAP-27) (PACAP27); Pituitary adenylate cyclase-activating polypeptide 38...	ENST00000269200	Unknown	
ADRA1B	adrenergic, alpha-1B-, receptor (ADRA1B), mRNA	NM_000679	Hs.368632	NM_000679
AF035290	clone 23556 mRNA sequence.	AF035290	Hs.656663	AF035290
AF052152	clone 24628 mRNA sequence.	AF052152	Hs.593611	AF052152
AF074994	full length insert cDNA YH91C06.	AF074994	Hs.656810	BU754431
AF086011	full length insert cDNA clone YW18A11.	AF086011	Hs.655148	BX089392
AF103312	clone Z117 immunoglobulin heavy chain variable region mRNA, partial cds.	AF103312	Hs.547404	BQ711793
AF131798	clone 25119 mRNA sequence.	AF131798	Hs.448887	AK091326
AF187554	sperm antigen-36 mRNA, complete cds.	AF187554	Unknown	
AFAR3	aflatoxin B1 aldehyde reductase 3 (AFAR3), mRNA	NM_201252	Hs.662342	NM_201252
AFF4	AF4/FMR2 family, member 4 (AFF4), mRNA	NM_014423	Hs.519313	NM_014423
AFM	afamin (AFM), mRNA	NM_001133	Hs.168718	NM_001133
AGRIN	agrin (AGRIN), mRNA	NM_198576	Hs.273330	AB191264
AGRIN	agrin (AGRIN), mRNA	NM_198576	Hs.273330	AB191264
AI078143	AI078143 oz30b05.x1 Soares_total_fetus_Nb2HF8_9w cDNA clone IMAGE:1676817 3' similar to contains Alu repetitive element;contains element PTR7 repetitive element ;, mRNA sequence	AI078143	Hs.656836	NM_005665
AI348443	AI348443 qo24g01.x1 NCI_CGAP_Lu5 cDNA clone IMAGE:1909488 3', mRNA sequence	AI348443	Hs.695994	CR936770
AI523154	ar69c11.x1 Barstead aorta HPLRB6 cDNA clone IMAGE:2127860 3', mRNA sequence	AI523154	Hs.645581	BE878470
AI954795	wq33d03.x1 NCI_CGAP_GC6 cDNA clone IMAGE:2473061 3', mRNA sequence	AI954795	Hs.156135	BE500990
AIFM3	apoptosis-inducing factor, mitochondrion-associated, 3 (AIFM3), transcript variant 2, mRNA	NM_001018060	Hs.163543	AK126917
AJ005814	mRNA for hoxA7 protein.	AJ005814	Unknown	
AJ420487	mRNA full length insert cDNA clone EUROIMAGE 1895238.	AJ420487	Hs.559456	AK057104
AK021595	cDNA FLJ11533 fis, clone HEMBA1002678.	AK021595	Hs.655802	AK021595
AK022110	cDNA FLJ12048 fis, clone HEMBB1001990.	AK022110	Hs.700615	BX537894
AK022337	cDNA FLJ12275 fis, clone MAMMA1001686.	AK022337	Hs.661289	AK022337
AK022892	cDNA FLJ12830 fis, clone NT2RP2003073.	AK022892	Hs.475208	NM_014838
AK027319	cDNA FLJ14413 fis, clone HEMBA1004670.	AK027319	Hs.334832	AK123705
AK055428	cDNA FLJ30866 fis, clone FEBRA2004110, highly similar to PHOSPHOLIPASE ADRAB-B PRECURSOR (EC 3.1.-.-).	AK055428	Unknown	
AK056384	cDNA FLJ31822 fis, clone NT2RP6000043.	AK056384	Hs.657123	BC038361
AK090397	mRNA for FLJ00272 protein.	AK090397	Hs.248746	AK123488
AK090897	cDNA FLJ33578 fis, clone BRAMY2011639.	AK090897	Hs.420757	BC063474
AK092508	cDNA FLJ35189 fis, clone PLACE6016210.	AK092508	Hs.595754	AK092508
AK092577	cDNA FLJ35258 fis, clone PROST2004146.	AK092577	Hs.585907	AK092577
AK093222	cDNA FLJ35903 fis, clone TESTI2009585.	AK093222	Hs.670269	AK093222
AK094175	cDNA FLJ36856 fis, clone ASTRO2014863.	AK094175	Hs.133892	BX648171
AK094424	cDNA FLJ37105 fis, clone BRACE2019510.	AK094424	Hs.638464	AK094424
AK096031	cDNA FLJ38712 fis, clone KIDNE2006733.	AK096031	Hs.638433	AK096031

AK096677	cDNA FLJ39358 fis, clone PEBLM2004015.	AK096677	Unknown	
AK097166	cDNA FLJ39847 fis, clone SPLEN2014645, weakly similar to H.sapiens mRNA for plakophilin 2a and b.	AK097166	Unknown	
AK097637	cDNA FLJ40318 fis, clone TESTI2030556.	AK097637	Hs.181159	AB051442
AK123157	cDNA FLJ41162 fis, clone BRACE2039600, highly similar to VOLTAGE-GATED POTASSIUM CHANNEL PROTEIN KV3.1.	AK123157	Hs.587695	AK123157
AK123168	cDNA FLJ41173 fis, clone BRACE2042394.	AK123168	Hs.515383	BG033631
AK123673	cDNA FLJ41679 fis, clone HCASM2003212.	AK123673	Hs.24040	AK123673
AK123861	cDNA FLJ41867 fis, clone OCBBF2005546.	AK123861	Hs.658919	AK123861
AK124470	cDNA FLJ42479 fis, clone BRACE2031899.	AK124470	Hs.660318	AK124470
AK128103	cDNA FLJ46224 fis, clone TESTI4014262.	AK128103	Hs.640099	AK128103
AK129584	cDNA FLJ26073 fis, clone RCT01314.	AK129584	Hs.643181	CR610566
AK130878	cDNA FLJ27368 fis, clone UBA03195.	AK130878	Hs.593907	AL833212
AK226066	mRNA for lysosomal-associated membrane protein 2 precursor variant, clone: fj10535.	AK226066	Hs.496684	BX648255
AKAP14	A kinase (PRKA) anchor protein 14 (AKAP14), transcript variant 1, mRNA	NM_178813	Hs.592245	BC066357
AKR7A2	aldo-keto reductase family 7, member A2 (aflatoxin aldehyde reductase) (AKR7A2), mRNA	NM_003689	Hs.571886	NM_003689
AL109696	mRNA full length insert cDNA clone EUROIMAGE 21920.	AL109696	Hs.185701	AK094929
ALB	clone FLB9714 PRO2619 mRNA, complete cds.	AF130077	Hs.418167	AF130077
ALDH9A1	aldehyde dehydrogenase 9 family, member A1 (ALDH9A1), mRNA	NM_000696	Hs.2533	U34252
ALDOB	aldolase B, fructose-bisphosphate (ALDOB), mRNA	NM_000035	Hs.530274	NM_000035
ALOX5AP	arachidonate 5-lipoxygenase-activating protein (ALOX5AP), mRNA	NM_001629	Hs.507658	BU198818
ALOX5AP	arachidonate 5-lipoxygenase-activating protein (ALOX5AP), mRNA	NM_001629	Hs.507658	BU198818
ALS2	amyotrophic lateral sclerosis 2 (juvenile) (ALS2), mRNA	NM_020919	Hs.471096	NM_020919
AMN	cDNA FLJ35505 fis, clone SMINT2009363.	AK092824	Unknown	
AMPD2	adenosine monophosphate deaminase 2 (isoform L) (AMPD2), transcript variant 1, mRNA	NM_004037	Hs.82927	NM_004037
AMPH	amphiphysin (Stiff-Man syndrome with breast cancer 128kDa autoantigen) (AMPH), transcript variant 1, mRNA	NM_001635	Hs.592182	NM_001635
AMPH	amphiphysin (Stiff-Man syndrome with breast cancer 128kDa autoantigen) (AMPH), transcript variant 1, mRNA	NM_001635	Hs.592182	NM_001635
ANKFN1	ankyrin-repeat and fibronectin type III domain containing 1 (ANKFN1), mRNA	NM_153228	Hs.673040	AK095654
ANKFY1	ankyrin repeat and FYVE domain containing 1 (ANKFY1), transcript variant 1, mRNA	NM_016376	Hs.696087	CR933717
ANKRD12	ankyrin repeat domain 12 (ANKRD12), mRNA	NM_015208	Hs.464585	NM_015208
ANKRD45	ankyrin repeat domain 45 (ANKRD45), mRNA	NM_198493	Hs.130054	AK127170
ANKRD6	ankyrin repeat domain 6 (ANKRD6), mRNA	NM_014942	Hs.656539	NM_014942
ANKS1B	ankyrin repeat and sterile alpha motif domain containing 1B (ANKS1B), transcript variant 2, mRNA	NM_181670	Hs.506458	NM_152788
AP1S2	adaptor-related protein complex 1, sigma 2 subunit (AP1S2), mRNA	NM_003916	Hs.656471	BX647483
AP3B1	adaptor-related protein complex 3, beta 1 subunit (AP3B1), mRNA	NM_003664	Hs.532091	BC038444
APBB3	amyloid beta (A4) precursor protein-binding, family B, member 3 (APBB3), transcript variant 4, mRNA	NM_006051	Hs.529449	AK125244
APC	adenomatosis polyposis coli (APC), mRNA	NM_000038	Hs.158932	NM_000038
APLP1	amyloid beta (A4) precursor-like protein 1 (APLP1), transcript variant 2, mRNA	NM_005166	Hs.74565	NM_001024807
APOA4	apolipoprotein A-IV (APOA4), mRNA	NM_000482	Hs.591940	NM_000482
AQP10	aquaporin 10 (AQP10), mRNA	NM_080429	Hs.259048	CR614330
ARHGEF11	Rho guanine nucleotide exchange factor (GEF) 11 (ARHGEF11), transcript variant 2, mRNA	NM_198236	Hs.516954	BC057394
ARHGEF7	Rho guanine nucleotide exchange factor (GEF) 7 (ARHGEF7), transcript variant 2, mRNA	NM_145735	Hs.508738	BX648030
ARL3	ADP-ribosylation factor-like 3 (ARL3), mRNA	NM_004311	Hs.182215	NM_004311
ARL4C	ADP-ribosylation factor-like 4C (ARL4C), mRNA	NM_005737	Hs.655990	NM_005737
ARMC3	armadillo repeat containing 3 (ARMC3), mRNA	NM_173081	Hs.659807	BC039312
ARPP-21	cyclic AMP-regulated phosphoprotein, 21 kD (ARPP-21), transcript variant 4, mRNA	NM_001025069	Hs.475902	NM_016300
ARPP-21	cyclic AMP-regulated phosphoprotein, 21 kD (ARPP-21), transcript variant 1, mRNA	NM_016300	Hs.475902	NM_016300
ARV1	ARV1 homolog (S. cerevisiae) (ARV1), mRNA	NM_022786	Hs.700665	AK092987
ATAD3B	mRNA for KIAA1273 protein, partial cds.	AB033099	Hs.23413	AB033099
ATP1B4	ATPase, (Na+)/K+ transporting, beta 4 polypeptide (ATP1B4), mRNA	NM_012069	Hs.662608	AF158383
ATP2B3	ATPase, Ca++ transporting, plasma membrane 3 (ATP2B3), transcript variant 1, mRNA	NM_021949	Hs.533956	NM_021949
ATP6V0A1	ATPase, H+ transporting, lysosomal V0 subunit a1 (ATP6V0A1), mRNA	NM_005177	Hs.463074	AK125927

ATP6V0B	ATPase, H+ transporting, lysosomal 21kDa, V0 subunit b (ATP6V0B), transcript variant 1, mRNA	NM_004047	Hs.632406	CR596289
ATPIF1	ATPase inhibitory factor 1 (ATPIF1), nuclear gene encoding mitochondrial protein, transcript variant 3, mRNA	NM_178191	Hs.590908	NM_178191
AV702101	AV702101 ADB cDNA clone ADBCGB06 5', mRNA sequence	AV702101	Hs.97579	AV702101
AVIL	advillin (AVIL), mRNA	NM_006576	Hs.584854	BX647344
AW188034	AW188034 xj91e12.x1 Soares_NFL_T_GBC_S1 cDNA clone IMAGE:2664622 3' similar to gb:M30496 UBIQUITIN CARBOXYL-TERMINAL HYDROLASE ISOZYME L3 (HUMAN);, mRNA sequence	AW188034	Hs.564550	CA975953
AW949170	AW949170 QV4-FT0006-110500-206-c01 FT0006 cDNA, mRNA sequence	AW949170	Hs.485195	NM_002959
AX721087	Sequence 47 from Patent WO0220754.	AX721087	Unknown	
B3GALT6	UDP-Gal:betaGal beta 1,3-galactosyltransferase polypeptide 6 (B3GALT6), mRNA	NM_080605	Hs.284284	NM_080605
B3GNT6	UDP-GlcNAc:betaGal beta-1,3-N-acetylglucosaminyltransferase 6 (core 3 synthase) (B3GNT6), mRNA	NM_138706	Hs.352622	NM_138706
BAIAP2	BAI1-associated protein 2 (BAIAP2), transcript variant 1, mRNA	NM_017450	Hs.128316	AK127291
BAIAP3	BAI1-associated protein 3 (BAIAP3), mRNA	NM_003933	Hs.458427	AL834321
BBOX1	butyrobetaine (gamma), 2-oxoglutarate dioxygenase (gamma-butyrobetaine hydroxylase) 1 (BBOX1), mRNA	NM_003986	Hs.591996	NM_003986
BBS4	Bardet-Biedl syndrome 4 (BBS4), mRNA	NM_033028	Hs.208681	AY457143
BC009228	cDNA clone IMAGE:3952569, partial cds.	BC009228	Hs.633824	AK124964
BC015449	Homo sapiens, clone IMAGE:4427279, mRNA.	BC015449	Hs.615028	BC015449
BC016291	cDNA clone IMAGE:3910094, partial cds.	BC016291	Hs.638073	BC016291
BC021677	Homo sapiens, clone IMAGE:4045663, mRNA.	BC021677	Hs.211626	BC021677
BC028243	Homo sapiens, clone IMAGE:5415705, mRNA, partial cds.	BC028243	Hs.683428	AK023800
BC030993	Homo sapiens, clone IMAGE:4556434, mRNA, partial cds.	BC030993	Hs.211511	BC033811
BC031957	cDNA clone IMAGE:4838390.	BC031957	Hs.638344	BC031957
BC035146	cDNA clone IMAGE:5264735.	BC035146	Hs.356604	NM_018979
BC037323	cDNA clone IMAGE:5261489.	BC037323	Hs.387982	BC037323
BC039363	cDNA clone IMAGE:5270672.	BC039363	Hs.129435	NM_001099439
BC042469	Homo sapiens, clone IMAGE:5198554, mRNA.	BC042469	Hs.152595	BC042469
BC042589	cDNA clone IMAGE:4819084.	BC042589	Hs.130853	BC042589
BC042853	cDNA clone IMAGE:5271371.	BC042853	Hs.376281	DQ266102
BC043411	Homo sapiens, clone IMAGE:6155889, mRNA.	BC043411	Hs.446446	BC043411
BC045168	cDNA clone IMAGE:4797829.	BC045168	Hs.283928	BC045168
BC104430	cDNA clone IMAGE:40021976.	BC104430	Hs.662784	BC029383
BC108268	cDNA clone IMAGE:4751428.	BC108268	Hs.573343	BC108268
BC128163	serine protease inhibitor, mRNA (cDNA clone MGC:149259 IMAGE:40113027), complete cds.	BC128163	Hs.483771	AK001520
BCAP31	B-cell receptor-associated protein 31 (BCAP31), mRNA	NM_005745	Hs.522817	BM466989
BE502686	BE502686 hz20h04.x1 NCI_CGAP_GC6 cDNA clone IMAGE:3208567 3', mRNA sequence	BE502686	Hs.126357	AK226116
BE766438	BE766438 IL3-NT0104-020800-232-E09 NT0104 cDNA, mRNA sequence	BE766438	Hs.700640	BC030009
BE786351	BE786351 601474522F1 NIH_MGC_68 cDNA clone IMAGE:3877396 5', mRNA sequence	BE786351	Hs.351306	NM_004696
BE893137	601437034F1 NIH_MGC_72 cDNA clone IMAGE:3922112 5', mRNA sequence	BE893137	Hs.633243	BE893137
BG193790	RST12926 Athersys RAGE Library cDNA, mRNA sequence	BG193790	Unknown	
BG623116	602648012F1 NIH_MGC_79 cDNA clone IMAGE:4769690 5', mRNA sequence	BG623116	Hs.496897	BG623116
BLZF1	Golgin 45 (Basic leucine zipper nuclear factor 1) (JEM-1) (p45 basic leucine-zipper nuclear factor).	ENST00000367808	Unknown	
BM461836	AGENCOURT_6418378 NIH_MGC_71 cDNA clone IMAGE:5533889 5', mRNA sequence	BM461836	Hs.547454	BM461836
BM768581	K-EST0051582 S14K402 cDNA clone S14K402-6-G10 5', mRNA sequence	BM768581	Hs.351126	BM768581
BM984396	BM984396 UI-CF-DU1-abf-e-18-0-UI.s1 UI-CF-DU1 cDNA clone UI-CF-DU1-abf-e-18-0-UI 3', mRNA sequence	BM984396	Hs.652928	BM984396
BM986936	BM986936 UI-H-CO0-aqe-a-10-0-UI.s1 NCI_CGAP_Sub9 cDNA clone IMAGE:3103745 3', mRNA sequence	BM986936	Hs.591347	U44429
BPI	bactericidal/permeability-increasing protein (BPI), mRNA	NM_001725	Hs.529019	BC040955
BQ189494	BQ189494 UI-E-EJ1-aka-g-05-0-UI.r1 UI-E-EJ1 cDNA clone UI-E-EJ1-aka-g-05-0-UI 5', mRNA sequence	BQ189494	Hs.664796	BQ189494
BSCL2	Bernardinelli-Seip congenital lipodystrophy 2 (seipin) (BSCL2), mRNA	NM_032667	Hs.533709	DQ470474
BSDC1	BSD domain containing 1 (BSDC1), mRNA	NM_018045	Hs.353454	BX641056
BX105638	BX105638 Soares_testis_NHT cDNA clone IMAGp998I154499, mRNA sequence	BX105638	Hs.147408	BX105638
C10orf126	chromosome 10 open reading frame 126, mRNA (cDNA clone IMAGE:3924461), complete cds.	BC033839	Unknown	

C10orf33	chromosome 10 open reading frame 33 (C10orf33), mRNA	NM_032709	Hs.238303	BC050574
C11orf56	chromosome 11 open reading frame 56 (C11orf56), mRNA	NM_032127	Hs.501793	NM_032127
C12orf22	chromosome 12 open reading frame 22 (C12orf22), mRNA	NM_030809	Hs.524425	AJ298133
C12orf28	chromosome 12 open reading frame 28 (C12orf28), mRNA	NM_182530	Unknown	
C13orf18	chromosome 13 open reading frame 18, mRNA (cDNA clone MGC:40256 IMAGE:5212065), complete cds.	BC032311	Unknown	
C14orf10	cDNA FLJ46718 fis, clone TRACH3018240.	AK128559	Hs.617139	AK128559
C14orf119	chromosome 14 open reading frame 119 (C14orf119), mRNA	NM_017924	Hs.525238	NM_017924
C15orf28	chromosome 15 open reading frame 28 (C15orf28), mRNA	NM_001040150	Unknown	
C16orf57	chromosome 16 open reading frame 57 (C16orf57), mRNA	NM_024598	Hs.588873	AK124443
C17orf76	chromosome 17 open reading frame 76 (C17orf76), mRNA	NM_207387	Hs.25425	AL833260
C19orf4	chromosome 19 open reading frame 4 (C19orf4), mRNA	NM_012109	Hs.329850	NM_012109
C19orf55	chromosome 19 open reading frame 55 (C19orf55), mRNA	NM_001039887	Hs.527982	BC110893
C1orf104	cDNA FLJ43522 fis, clone PLACE5000260.	AK125510	Unknown	
C1orf122	chromosome 1 open reading frame 122 (C1orf122), mRNA	NM_198446	Hs.532749	BC016402
C1orf122	chromosome 1 open reading frame 122 (C1orf122), mRNA	NM_198446	Hs.532749	BC016402
C1orf126	chromosome 1 open reading frame 126 (C1orf126), mRNA	NM_182534	Unknown	
C1orf131	cDNA FLJ30562 fis, clone BRAWH2004731.	AK055124	Hs.556017	AK055124
C1orf142	chromosome 1 open reading frame 142 (C1orf142), mRNA	NM_053052	Hs.325081	AK026908
C1orf149	chromosome 1 open reading frame 149 (C1orf149), mRNA	NM_022756	Hs.17118	BX640719
C1orf151	chromosome 1 open reading frame 151 (C1orf151), mRNA	NM_001032363	Hs.466662	NM_001032363
C1orf158	chromosome 1 open reading frame 158 (C1orf158), mRNA	NM_152290	Hs.98095	BX647383
C1orf192	chromosome 1 open reading frame 192 (C1orf192), mRNA	NM_001013625	Hs.534593	BM800357
C1orf213	chromosome 1 open reading frame 213 (C1orf213), transcript variant 1, mRNA	NM_138479	Hs.61884	BC054115
C1orf63	chromosome 1 open reading frame 63, mRNA (cDNA clone MGC:74698 IMAGE:6147639), complete cds.	BC065040	Hs.259412	AK096351
C1orf63	chromosome 1 open reading frame 63 (C1orf63), mRNA	NM_020317	Hs.259412	AK096351
C1orf87	chromosome 1 open reading frame 87 (C1orf87), mRNA	NM_152377	Hs.47385	AK126061
C1orf88	chromosome 1 open reading frame 88 (C1orf88), mRNA	NM_181643	Hs.172510	BC050319
C1QDC2	C1q domain containing 2, mRNA (cDNA clone MGC:105127 IMAGE:30554756), complete cds.	BC089443	Hs.197613	BC089443
C20orf197	chromosome 20 open reading frame 197 (C20orf197), mRNA	NM_173644	Hs.335319	AY358539
C20orf26	chromosome 20 open reading frame 26 (C20orf26), mRNA	NM_015585	Hs.176013	NM_015585
C21orf29	chromosome 21 open reading frame 29 (C21orf29), mRNA	NM_144991	Hs.660703	NM_144991
C22orf23	cDNA FLJ40020 fis, clone STOMA2006780.	AK097339	Hs.517612	AK097339
C2orf39	chromosome 2 open reading frame 39 (C2orf39), mRNA	NM_145038	Hs.393714	AK128286
C3orf25	chromosome 3 open reading frame 25 (C3orf25), mRNA	NM_207307	Hs.652347	AL133011
C3orf34	chromosome 3 open reading frame 34 (C3orf34), mRNA	NM_032898	Hs.334526	BC007827
C4BPA	complement component 4 binding protein, alpha (C4BPA), mRNA	NM_000715	Hs.1012	BC022312
C4orf26	chromosome 4 open reading frame 26 (C4orf26), mRNA	NM_178497	Hs.24510	BC061701
C6orf165	CMP-sialic acid transporter (CMP-Sia-Tr) (CMP-SA-Tr) (Solute carrier family 35 member A1).	ENST00000369562	Unknown	
C6orf165	chromosome 6 open reading frame 165 (C6orf165), transcript variant 1, mRNA	NM_001031743	Hs.82921	NM_178823
C6orf204	C6orf204 protein (Fragment).	ENST00000368491	Unknown	
C6orf204	chromosome 6 open reading frame 204 (C6orf204), transcript variant 2, mRNA	NM_206921	Hs.656959	BC045657
C6orf206	chromosome 6 open reading frame 206 (C6orf206), mRNA	NM_152732	Hs.534585	BQ676144
C6orf65	chromosome 6 open reading frame 65 (C6orf65), mRNA	NM_152731	Hs.582993	BC036119
C6orf85	chromosome 6 open reading frame 85, mRNA (cDNA clone IMAGE:3846727), complete cds.	BC022217	Hs.700628	NM_021945
C7orf45	chromosome 7 open reading frame 45 (C7orf45), mRNA	NM_145268	Hs.351816	NM_145268
C8B	complement component 8, beta polypeptide (C8B), mRNA	NM_000066	Hs.391835	NM_000066
C8orf12	chromosome 8 open reading frame 12, mRNA (cDNA clone IMAGE:6303594).	BC080558	Hs.583896	AK057762
C8orf22	chromosome 8 open reading frame 22 (C8orf22), mRNA	NM_001007176	Hs.49890	AJ276240
C8orf39	PRO1905 mRNA, complete cds.	AF116672	Hs.192788	NM_203390
C8orf51	cDNA: FLJ22850 fis, clone KAT00518.	AK026503	Hs.245886	BC000203
C9orf4	chromosome 9 open reading frame 4 (C9orf4), mRNA	NM_014334	Hs.347537	NM_014334

CA10	carbonic anhydrase X (CA10), mRNA	NM_020178	Hs.463466	NM_001082533
CA12	cDNA FLJ20151 fis, clone COL08412.	AK000158	Hs.210995	NM_001218
CA3	carbonic anhydrase III, muscle specific (CA3), mRNA	NM_005181	Hs.82129	NM_005181
CABC1	chaperone, ABC1 activity of bc1 complex homolog (S. pombe) (CABC1), nuclear gene encoding mitochondrial protein, mRNA	NM_020247	Hs.118241	NM_020247
CALD1	caldesmon 1 (CALD1), transcript variant 1, mRNA	NM_033138	Hs.490203	NM_033138
CALD1	cDNA FLJ12160 fis, clone MAMMA1000567.	AK022222	Unknown	
CALD1	NAG22 protein mRNA, complete cds.	AF247820	Unknown	
CALM2	calmodulin 2 (phosphorylase kinase, delta) (CALM2), mRNA	NM_001743	Hs.643483	BG110202
CAMK1D	calcium/calmodulin-dependent protein kinase ID (CAMK1D), transcript variant 1, mRNA	NM_020397	Hs.659517	NM_153498
CAPSL	calcyphosine-like (CAPSL), transcript variant 1, mRNA	NM_144647	Hs.55150	NM_144647
CAPZA1	capping protein (actin filament) muscle Z-line, alpha 1 (CAPZA1), mRNA	NM_006135	Hs.514934	BX648738
CASC1	cancer susceptibility candidate 1 (CASC1), mRNA	NM_018272	Hs.407771	NM_001082972
CASC4	cancer susceptibility candidate 4 (CASC4), transcript variant 1, mRNA	NM_138423	Hs.512867	NM_138423
CASC4	cancer susceptibility candidate 4 (CASC4), transcript variant 1, mRNA	NM_138423	Hs.512867	NM_138423
CB240827	UI-CF-FN0-afw-h-15-0-UI.s1 UI-CF-FN0 cDNA clone UI-CF-FN0-afw-h-15-0-UI 3', mRNA sequence	CB240827	Hs.621746	CB240827
CCDC128	coiled-coil domain containing 128 (CCDC128), mRNA	NM_152994	Hs.654619	AK122900
CCDC37	coiled-coil domain containing 37 (CCDC37), mRNA	NM_182628	Hs.591305	NM_182628
CCDC65	coiled-coil domain containing 65 (CCDC65), mRNA	NM_033124	Hs.512805	AF382188
CCDC72	cDNA FLJ44631 fis, clone BRACE2018443.	AK126594	Hs.356440	AK126594
CCL18	chemokine (C-C motif) ligand 18 (pulmonary and activation-regulated) (CCL18), mRNA	NM_002988	Hs.143961	Y13710
CCNL2	cyclin L2 (CCNL2), transcript variant 2, mRNA	NM_001039577	Hs.515704	NM_001039577
CCR3	chemokine (C-C motif) receptor 3 (CCR3), transcript variant 1, mRNA	NM_001837	Hs.506190	NM_001837
CD6	CD6 molecule (CD6), mRNA	NM_006725	Hs.695959	AL832261
CD8A	CD8a molecule (CD8A), transcript variant 1, mRNA	NM_001768	Hs.85258	AK124156
CDC42BPA	CDC42 binding protein kinase alpha (DMPK-like) (CDC42BPA), transcript variant A, mRNA	NM_014826	Hs.35433	NM_003607
CDC42SE1	CDC42 small effector 1 (CDC42SE1), transcript variant 1, mRNA	NM_001038707	Hs.22065	NM_001038707
CDH22	cadherin-like 22 (CDH22), mRNA	NM_021248	Hs.472861	NM_021248
CDH6	cadherin 6, type 2, K-cadherin (fetal kidney) (CDH6), mRNA	NM_004932	Hs.171054	NM_004932
CDH6	Cadherin-6 precursor (Kidney-cadherin) (K-cadherin).	ENST00000382216	Unknown	
CDH6	Cadherin-6 precursor (Kidney-cadherin) (K-cadherin).	ENST00000382216	Unknown	
CDK5	cyclin-dependent kinase 5 (CDK5), mRNA	NM_004935	Hs.647078	BG577212
CDKN2B	cyclin-dependent kinase inhibitor 2B (p15, inhibits CDK4) (CDKN2B), transcript variant 1, mRNA	NM_004936	Hs.72901	NM_078487
CDKN2B	cyclin-dependent kinase inhibitor 2B (p15, inhibits CDK4) (CDKN2B), transcript variant 2, mRNA	NM_078487	Hs.72901	NM_078487
CENTB5	centaurin, beta 5 (CENTB5), mRNA	NM_030649	Hs.535257	AB051503
CENTB5	centaurin, beta 5 (CENTB5), mRNA	NM_030649	Hs.535257	AB051503
CF143262	CF143262 UI-HF-BR0p-aqt-a-11-0-UI.r1 NIH_MGC_52 cDNA clone IMAGE:3101469 5', mRNA sequence	CF143262	Hs.252387	AF231024
CFHR1	complement factor H-related 1 (CFHR1), mRNA	NM_002113	Hs.363396	AK226113
CFHR2	complement factor H-related 2 (CFHR2), mRNA	NM_005666	Hs.632450	BG567998
CHD5	chromodomain helicase DNA binding protein 5 (CHD5), mRNA	NM_015557	Hs.522898	AF425231
CHIA	chitinase, acidic (CHIA), transcript variant 2, mRNA	NM_021797	Hs.128814	NM_201653
CHID1	chitinase domain containing 1 (CHID1), mRNA	NM_023947	Hs.144468	AK124697
CHRD	chordin (CHRD), mRNA	NM_003741	Hs.166186	AF209930
CHRNA1	cholinergic receptor, nicotinic, alpha 1 (muscle), mRNA (cDNA clone MGC:12708 IMAGE:4124038), complete cds.	BC006314	Unknown	
CIRBP	cDNA FLJ46566 fis, clone THYMU3040829, moderately similar to Cold-inducible RNA-binding protein.	AK128423	Hs.634522	AK095781
CIRBP	cDNA FLJ46566 fis, clone THYMU3040829, moderately similar to Cold-inducible RNA-binding protein.	AK128423	Hs.634522	AK095781
CIRBP	cDNA FLJ46566 fis, clone THYMU3040829, moderately similar to Cold-inducible RNA-binding protein.	AK128423	Hs.634522	AK095781
CLCC1	chloride channel CLIC-like 1 (CLCC1), transcript variant 1, mRNA	NM_001048210	Hs.658489	NM_001048210
CLEC1B	C-type lectin domain family 1, member B (CLEC1B), mRNA	NM_016509	Hs.409794	BX647321
CLIC6	chloride intracellular channel 6 (CLIC6), mRNA	NM_053277	Hs.473695	AF448439
CLK1	CDC-like kinase 1 (CLK1), transcript variant 1, mRNA	NM_004071	Hs.433732	NM_004071

CLTA	clathrin, light chain (Lca) (CLTA), transcript variant 2, mRNA	NM_007096	Hs.522114	CR599555
CLTC	clathrin, heavy chain (Hc) (CLTC), mRNA	NM_004859	Hs.491351	NM_004859
CLUL1	clusterin-like 1 (retinal) (CLUL1), transcript variant 1, mRNA	NM_014410	Hs.632357	NM_014410
CNN3	calponin 3, acidic (CNN3), mRNA	NM_001839	Hs.700591	NM_001839
CNTN3	contactin 3 (plasmacytoma associated) (CNTN3), mRNA	NM_020872	Hs.12723	NM_020872
CNTN3	contactin 3 (plasmacytoma associated) (CNTN3), mRNA	NM_020872	Hs.12723	NM_020872
CNTN3	contactin 3 (plasmacytoma associated) (CNTN3), mRNA	NM_020872	Hs.12723	NM_020872
COG3	component of oligomeric golgi complex 3 (COG3), mRNA	NM_031431	Hs.507948	AF349676
COL11A1	collagen, type XI, alpha 1 (COL11A1), transcript variant B, mRNA	NM_080629	Hs.523446	NM_080629
COL18A1	collagen, type XVIII, alpha 1 (COL18A1), transcript variant 1, mRNA	NM_030582	Hs.517356	AF018081
COL4A3	collagen, type IV, alpha 3 (Goodpasture antigen) (COL4A3), transcript variant 1, mRNA	NM_000091	Hs.570065	NM_000091
COL4A3	collagen, type IV, alpha 3 (Goodpasture antigen) (COL4A3), transcript variant 1, mRNA	NM_000091	Hs.570065	NM_000091
COL4A4	collagen, type IV, alpha 4 (COL4A4), mRNA	NM_000092	Hs.591645	NM_000092
COL9A2	collagen, type IX, alpha 2 (COL9A2), mRNA	NM_001852	Hs.418012	BC041479
COPB2	coatomer protein complex, subunit beta 2 (beta prime) (COPB2), mRNA	NM_004766	Hs.75724	AK128561
COX7A2L	cytochrome c oxidase subunit VIIa polypeptide 2 like (COX7A2L), nuclear gene encoding mitochondrial protein, mRNA	NM_004718	Hs.339639	AK130281
COX7B	Cytochrome c oxidase polypeptide VIIb, mitochondrial precursor (EC 1.9.3.1).	ENST00000373335	Unknown	
CP110	CP110 protein (CP110), mRNA	NM_014711	Hs.279912	NM_014711
CR594735	full-length cDNA clone CS0DE011YB11 of Placenta of (human).	CR594735	Hs.153408	AK001432
CR620892	full-length cDNA clone CS0DL007YK20 of B cells (Ramos cell line) Cot 25-normalized of (human).	CR620892	Hs.584916	AK000271
CR622844	full-length cDNA clone CS0DF026YC16 of Fetal brain of (human).	CR622844	Hs.449098	BM906393
CR625594	full-length cDNA clone CS0DF026YM07 of Fetal brain of (human).	CR625594	Hs.125867	AL133642
CRISPLD2	cysteine-rich secretory protein LCCL domain containing 2 (CRISPLD2), mRNA	NM_031476	Hs.513779	AL136861
CRP	C-reactive protein, pentraxin-related (CRP), mRNA	NM_000567	Hs.695960	NM_000567
CRYGC	crystallin, gamma C (CRYGC), mRNA	NM_020989	Hs.72910	BG196322
CRYGS	crystallin, gamma S (CRYGS), mRNA	NM_017541	Hs.376209	BX648612
CRYGS	crystallin, gamma S (CRYGS), mRNA	NM_017541	Hs.376209	BX648612
CSNK1D	mRNA for casein kinase 1, delta isoform 1 variant protein.	AB209463	Hs.631725	AB209463
CTBS	chitobiase, di-N-acetyl-, mRNA (cDNA clone IMAGE:4823479), complete cds.	BC024007	Hs.513557	BC096752
CUGBP2	CUG triplet repeat, RNA binding protein 2 (CUGBP2), transcript variant 3, mRNA	NM_001025077	Hs.309288	NM_001025077
CX3CL1	chemokine (C-X3-C motif) ligand 1 (CX3CL1), mRNA	NM_002996	Hs.531668	AB209037
CX3CL1	chemokine (C-X3-C motif) ligand 1 (CX3CL1), mRNA	NM_002996	Hs.531668	AB209037
CXorf12	chromosome X open reading frame 12 (CXorf12), mRNA	NM_003492	Hs.23119	NM_003492
CXorf27	Huntingtin-interacting protein HYPM (Huntingtin yeast partner M).	ENST00000341016	Unknown	
CYP2C9	cytochrome P450, family 2, subfamily C, polypeptide 9 (CYP2C9), mRNA	NM_000771	Hs.282624	M61855
CYP3A4	cytochrome P450, family 3, subfamily A, polypeptide 4 (CYP3A4), mRNA	NM_017460	Hs.654391	NM_017460
CYP3A5	cytochrome P450, family 3, subfamily A, polypeptide 5 (CYP3A5), mRNA	NM_000777	Hs.695915	BX537676
D4ST1	dermatan 4 sulfotransferase 1 (D4ST1), mRNA	NM_130468	Hs.442449	AB066595
DAP3	death associated protein 3 (DAP3), nuclear gene encoding mitochondrial protein, transcript variant 1, mRNA	NM_033657	Hs.516746	CR615460
DDOST	dolichyl-diphosphooligosaccharide-protein glycosyltransferase (DDOST), mRNA	NM_005216	Hs.523145	NM_005216
DDX24	DEAD (Asp-Glu-Ala-Asp) box polypeptide 24 (DDX24), mRNA	NM_020414	Hs.510328	BX537533
DEC1	Homo sapiens, clone IMAGE:5526534, mRNA, partial cds.	BC030567	Hs.148841	AK056153
DERL3	Der1-like domain family, member 3 (DERL3), transcript variant 1, mRNA	NM_198440	Hs.593679	AK125830
DGAT2	diacylglycerol O-acyltransferase homolog 2 (mouse) (DGAT2), mRNA	NM_032564	Hs.334305	CR749377
DGAT2	diacylglycerol O-acyltransferase homolog 2 (mouse) (DGAT2), mRNA	NM_032564	Hs.334305	CR749377
DHRS1	dehydrogenase/reductase (SDR family) member 1 (DHRS1), mRNA	NM_138452	Hs.348350	AK126383
DHRS1	dehydrogenase/reductase (SDR family) member 1 (DHRS1), mRNA	NM_138452	Hs.348350	AK126383
DIO2	deiodinase, iodothyronine, type II (DIO2), transcript variant 1, mRNA	NM_013989	Hs.202354	NM_013989
DIRAS2	DIRAS family, GTP-binding RAS-like 2 (DIRAS2), mRNA	NM_017594	Hs.165636	NM_017594
DIRAS2	DIRAS family, GTP-binding RAS-like 2 (DIRAS2), mRNA	NM_017594	Hs.165636	NM_017594
DKFZp667E0512	mRNA; cDNA DKFZp667E0512 (from clone DKFZp667E0512).	AL713660	Hs.618463	AL713660

DKFZp761H2121	cDNA FLJ33774 fis, clone BRSSN2000244.	AK091093	Hs.134065	AK091093
DKK2	dickkopf homolog 2 (Xenopus laevis) (DKK2), mRNA	NM_014421	Hs.211869	AF177395
DKK2	dickkopf homolog 2 (Xenopus laevis) (DKK2), mRNA	NM_014421	Hs.211869	AF177395
DNAH7	dynein, axonemal, heavy chain 7 (DNAH7), mRNA	NM_018897	Hs.97403	AB023161
DNAJC8	DnaJ (Hsp40) homolog, subfamily C, member 8 (DNAJC8), mRNA	NM_014280	Hs.433540	CD013879
DNAPTP6	DNA polymerase-transactivated protein 6 (DNAPTP6), mRNA	NM_015535	Hs.120323	NM_001100422
DNASE1L2	deoxyribonuclease I-like 2 (DNASE1L2), mRNA	NM_001374	Hs.103503	AK098028
DNHD2	cDNA FLJ46751 fis, clone TRACH3022960, weakly similar to Dynein beta chain, ciliary.	AK128592	Hs.201378	AK128592
DNM3	dynamin 3 (DNM3), mRNA	NM_015569	Hs.654775	AL136712
DOCK8	dedicator of cytokinesis 8 (DOCK8), mRNA	NM_203447	Hs.132599	AL832270
DQ786199	clone HLS_IMAGE_135010 mRNA sequence.	DQ786199	Hs.523529	XM_209227
DQ786232	clone HLS_IMAGE_1759573 mRNA sequence.	DQ786232	Hs.586392	DQ786232
DQ926472	clone IM2 3u45 immunoglobulin heavy chain variable region mRNA, partial cds.	DQ926472	Unknown	
DRD1IP	dopamine receptor D1 interacting protein (DRD1IP), mRNA	NM_015722	Hs.148680	BM808864
DSC1	desmocollin 1 (DSC1), transcript variant Dsc1b, mRNA	NM_004948	Hs.567260	X72925
DSG3	desmoglein 3 (pemphigus vulgaris antigen) (DSG3), mRNA	NM_001944	Hs.1925	NM_001944
DUOX1	mRNA; cDNA DKFZp434L0610 (from clone DKFZp434L0610); partial cds.	AL137592	Hs.272813	NM_017434
DUOX2	dual oxidase 2 (DUOX2), mRNA	NM_014080	Hs.71377	AB209010
DUSP5P	dual specificity phosphatase 5 pseudogene (DUSP5P) on chromosome 1	NR_002834	Unknown	
DUSP5P	dual specificity phosphatase 5 pseudogene (DUSP5P) on chromosome 1	NR_002834	Unknown	
DVL1	dishevelled, dsh homolog 1 (Drosophila) (DVL1), transcript variant 2, mRNA	NM_181870	Unknown	
DYNC1I1	dynein, cytoplasmic 1, intermediate chain 1 (DYNC1I1), mRNA	NM_004411	Hs.440364	BC053542
DYNC1I1	dynein, cytoplasmic 1, intermediate chain 1 (DYNC1I1), mRNA	NM_004411	Hs.440364	BC053542
DYNC1LI2	dynein, cytoplasmic 1, light intermediate chain 2 (DYNC1LI2), mRNA	NM_006141	Hs.369068	NM_006141
EGFL6	EGF-like-domain, multiple 6 (EGFL6), mRNA	NM_015507	Hs.12844	AJ245671
EIF2C4	mRNA for KIAA1567 protein, partial cds.	AB046787	Hs.471492	AB046787
EIF4A2	eukaryotic translation initiation factor 4A, isoform 2 (EIF4A2), mRNA	NM_001967	Hs.700566	CR612348
EIF4A2	eukaryotic translation initiation factor 4A, isoform 2 (EIF4A2), mRNA	NM_001967	Hs.700566	CR612348
EIF4G2	eukaryotic translation initiation factor 4 gamma, 2 (EIF4G2), transcript variant 1, mRNA	NM_001418	Hs.183684	BC065276
ELAVL3	ELAV (embryonic lethal, abnormal vision, Drosophila)-like 3 (Hu antigen C) (ELAVL3), transcript variant 1, mRNA	NM_001420	Hs.1701	NM_001420
ENO1	enolase 1, (alpha) (ENO1), mRNA	NM_001428	Hs.517145	AL833741
ENST000002191	Nuclear transport factor 2 (NTF-2) (Placental protein 15) (PP15).	ENST00000219169	Unknown	
ENST000002852	Sterile alpha motif domain-containing protein 14.	ENST00000285206	Unknown	
ENST000002999	PREDICTED: hypothetical LOC644353 (LOC644353), mRNA	ENST00000299903	Unknown	
ENST000003157	CDNA FLJ34790 fis, clone NT2NE2005323 (C17orf44 protein) (Hypothetical protein C17orf44).	ENST00000315707	Unknown	
ENST000003179	l(3)mbt-like 4	ENST00000317931	Unknown	
ENST000003202	C11orf64 protein.	ENST00000320202	Unknown	
ENST000003228	Numb-interacting protein	ENST00000322839	Unknown	
ENST000003280	partial mRNA for immunoglobulin kappa light chain variable region (IGKV gene), isolate B-CLL 483.	ENST00000328018	Unknown	
ENST000003303	Alpha-amylase 2B precursor (EC 3.2.1.1) (1,4-alpha-D-glucan glucanohydrolase) (Alpha-amylase carcinoid).	ENST00000330330	Unknown	
ENST000003309	PREDICTED: hypothetical LOC391648 (LOC391648), mRNA	ENST00000330935	Unknown	
ENST000003391	TBC1 domain family, member 3 pseudogene 2, mRNA (cDNA clone MGC:64921 IMAGE:5744726), complete cds.	ENST00000339120	Unknown	
ENST000003401	Exocyst complex component 3 (Exocyst complex component Sec6).	ENST00000340158	Unknown	
ENST000003561	Histone H3.1 (H3/a) (H3/b) (H3/c) (H3/d) (H3/f) (H3/h) (H3/i) (H3/j) (H3/k) (H3/l).	ENST00000356177	Unknown	
ENST000003565	FAM39B protein.	ENST00000356572	Unknown	
ENST000003597	armadillo repeat containing 9	ENST00000359743	Unknown	
ENST000003620	Prostate-specific and androgen-regulated protein 1 (PART-1).	ENST00000362001	Unknown	
ENST000003699	Novel protein.	ENST00000369945	Unknown	
ENST000003829	Zinc finger MYM-type protein 5 (Zinc finger protein 237) (Zinc finger protein 198-like 1).	ENST00000382909	Unknown	
ENST000003834	HLA-B associated transcript 3 (Fragment).	ENST00000383448	Unknown	
ENST000003894	PCLO_HUMAN Isoform 3 of Q9Y6V0 - (Human)	ENST00000389477	Unknown	

ENTPD3	ectonucleoside triphosphate diphosphohydrolase 3 (ENTPD3), mRNA	NM_001248	Hs.441145	AF034840
EPB41L4B	erythrocyte membrane protein band 4.1 like 4B (EPB41L4B), transcript variant 2, mRNA	NM_019114	Hs.591901	AF153416
EPS15	epidermal growth factor receptor pathway substrate 15 (EPS15), mRNA	NM_001981	Hs.83722	NM_001981
EXOC7	exocyst complex component 7 (EXOC7), transcript variant 1, mRNA	NM_001013839	Hs.533985	NM_001013839
EXOC7	exocyst complex component 7 (EXOC7), transcript variant 1, mRNA	NM_001013839	Hs.533985	NM_001013839
F8A1	coagulation factor VIII-associated (intronic transcript) 1 (F8A1), mRNA	NM_012151	Hs.533543	BC039693
F9	coagulation factor IX (plasma thromboplastic component, Christmas disease, hemophilia B) (F9), mRNA	NM_000133	Hs.522798	NM_000133
FAM102A	family with sequence similarity 102, member A (FAM102A), transcript variant 1, mRNA	NM_001035254	Hs.568044	AK074108
FAM14B	family with sequence similarity 14, member B (FAM14B), mRNA	NM_206949	Hs.19414	CR625015
FAM14B	family with sequence similarity 14, member B (FAM14B), mRNA	NM_206949	Hs.19414	CR625015
FAM40A	family with sequence similarity 40, member A (FAM40A), mRNA	NM_033088	Hs.584996	AL834196
FAM46C	family with sequence similarity 46, member C (FAM46C), mRNA	NM_017709	Hs.356216	NM_017709
FAM81B	family with sequence similarity 81, member B (FAM81B), mRNA	NM_152548	Hs.276287	BC034772
FAM82C	family with sequence similarity 82, member C (FAM82C), mRNA	NM_018145	Hs.511067	AK123282
FAM92B	family with sequence similarity 92, member B (FAM92B), mRNA	NM_198491	Hs.125713	AK126284
FANCC	Fanconi anemia, complementation group C, mRNA (cDNA clone IMAGE:4777682), with apparent retained intron.	BC034271	Hs.672362	BC034271
FGF14	fibroblast growth factor 14 (FGF14), transcript variant 2, mRNA	NM_175929	Hs.696392	AY188178
FGFBP2	fibroblast growth factor binding protein 2 (FGFBP2), mRNA	NM_031950	Hs.98785	NM_031950
FLJ10781	hypothetical protein FLJ10781 (FLJ10781), mRNA	NM_018215	Hs.8395	BC032508
FLJ20489	cDNA clone MGC:26667 IMAGE:4798578, complete cds.	BC026344	Hs.438867	BC026344
FLJ21075	hypothetical protein FLJ21075 (FLJ21075), mRNA	NM_025031	Hs.287647	CA420605
FLJ23577	KPL2 protein (FLJ23577), transcript variant 1, mRNA	NM_024867	Hs.298863	AL832607
FLJ25076	mRNA, clone: TH049G03.	AB188494	Hs.126856	XM_059689
FLJ33790	hypothetical protein FLJ33790 (FLJ33790), mRNA	NM_001039548	Hs.292451	AL050370
FLJ33790	hypothetical protein FLJ33790 (FLJ33790), mRNA	NM_001039548	Hs.292451	AL050370
FLJ35348	FLJ35348, mRNA (cDNA clone IMAGE:5398136), partial cds.	BC065204	Hs.699260	BC065204
FLJ35834	hypothetical protein FLJ35834 (FLJ35834), mRNA	NM_178827	Hs.159650	AK127736
FLJ35880	hypothetical protein FLJ35880 (FLJ35880), mRNA	NM_153264	Hs.205403	NM_153264
FLJ36032	cDNA FLJ36032 fis, clone TESTI2017069.	AK093351	Hs.297967	AK093351
FLJ37228	cDNA FLJ37228 fis, clone BRAMY2000411.	AK094547	Hs.596660	AK094547
FLJ39575	hypothetical protein FLJ39575 (FLJ39575), mRNA	NM_182597	Hs.396189	NM_182597
FLJ40172	hypothetical protein FLJ40172 (FLJ40172), mRNA	NM_173649	Hs.531575	NM_173649
FLJ40194	FLJ40194 protein (FLJ40194), mRNA	NM_001007529	Unknown	
FLJ45557	hypothetical protein LOC642938 (FLJ45557), mRNA	NM_001039762	Hs.613882	AK127465
FLJ46266	FLJ46266 protein (FLJ46266), mRNA	NM_207430	Hs.411600	BM473494
FLJ46266	CDNA FLJ46266 fis, clone TESTI4027139.	ENST00000332814	Unknown	
FLJ46385	FLJ46385 protein (FLJ46385), mRNA	NM_001001675	Unknown	
FOXE1	forkhead box E1 (thyroid transcription factor 2) (FOXE1), mRNA	NM_004473	Hs.159234	U89995
FOXL2	forkhead box L2 (FOXL2), mRNA	NM_023067	Hs.289292	DQ089670
FRG2	FSHD region gene 2 protein (FRG2), mRNA	NM_001005217	Hs.626907	AY714545
FRMD7	FERM domain containing 7 (FRMD7), mRNA	NM_194277	Hs.170776	AK125336
FUCA1	fucosidase, alpha-L- 1, tissue (FUCA1), mRNA	NM_000147	Hs.370858	NM_000147
FUT3	fucosyltransferase 3 (galactoside 3(4)-L-fucosyltransferase, Lewis blood group) (FUT3), mRNA	NM_000149	Hs.169238	NM_000149
GABRA2	gamma-aminobutyric acid (GABA) A receptor, alpha 2 (GABRA2), mRNA	NM_000807	Hs.116250	AK125179
GALM	galactose mutarotase (aldose 1-epimerase) (GALM), mRNA	NM_138801	Hs.435012	BC019263
GALNT10	cDNA FLJ11715 fis, clone HEMBA1005223.	AK021777	Hs.651323	AK021777
GALNT11	UDP-N-acetyl-alpha-D-galactosamine:polypeptide N-acetylgalactosaminyltransferase 11 (GalNAc-T11) (GALNT11), mRNA	NM_022087	Hs.647109	AK128545
GALNT13	UDP-N-acetyl-alpha-D-galactosamine:polypeptide N-acetylgalactosaminyltransferase 13 (GalNAc-T13) (GALNT13), mRNA	NM_052917	Hs.470277	NM_052917
GDA	guanine deaminase (GDA), mRNA	NM_004293	Hs.494163	NM_004293
GGA1	golgi associated, gamma adaptin ear containing, ARF binding protein 1 (GGA1), transcript variant 2, mRNA	NM_001001560	Hs.499158	AK122898

GGCX	gamma-glutamyl carboxylase (GGCX), mRNA	NM_000821	Hs.77719	NM_000821
GLB1	galactosidase, beta 1 (GLB1), transcript variant 1, mRNA	NM_000404	Hs.443031	AK126979
GLT8D1	glycosyltransferase 8 domain containing 1 (GLT8D1), transcript variant 3, mRNA	NM_001010983	Hs.297304	NM_001010983
GLULD1	glutamate-ammonia ligase (glutamine synthetase) domain containing 1 (GLULD1), mRNA	NM_016571	Hs.149585	AF242388
GNAO1	guanine nucleotide binding protein (G protein), alpha activating activity polypeptide O (GNAO1), transcript variant 1, mRNA	NM_020988	Hs.695994	CR936770
GNAS	GNAS complex locus (GNAS), transcript variant 7, mRNA	NM_001077489	Hs.125898	NM_001077490
GNB1	guanine nucleotide binding protein (G protein), beta polypeptide 1 (GNB1), mRNA	NM_002074	Hs.700577	NM_002074
GNRH1	gonadotropin-releasing hormone 1 (luteinizing-releasing hormone) (GNRH1), mRNA	NM_000825	Hs.82963	NM_000825
GOLGA6	golgi autoantigen, golgin subfamily a, 6 (GOLGA6), mRNA	NM_001038640	Hs.546408	NM_018652
GPR112	G protein-coupled receptor 112 (GPR112), mRNA	NM_153834	Hs.381354	AY882585
GPR123	G protein-coupled receptor 123 (GPR123), mRNA	NM_032422	Unknown	
GPR26	G protein-coupled receptor 26 (GPR26), mRNA	NM_153442	Hs.12751	AJ505757
GPR35	G protein-coupled receptor 35 (GPR35), mRNA	NM_005301	Hs.239891	NM_005301
GPR37	G protein-coupled receptor 37 (endothelin receptor type B-like) (GPR37), mRNA	NM_005302	Hs.406094	BX649006
GPR87	G protein-coupled receptor 87 (GPR87), mRNA	NM_023915	Hs.591292	NM_023915
GPR88	strg mRNA for striatum-specific G protein-coupled receptor, complete cds.	AB042410	Hs.170053	AB042410
GRIA2	glutamate receptor, ionotropic, AMPA 2 (GRIA2), mRNA	NM_000826	Hs.32763	NM_000826
GRIA2	glutamate receptor, ionotropic, AMPA 2 (GRIA2), mRNA	NM_000826	Hs.32763	NM_000826
GRIA2	glutamate receptor, ionotropic, AMPA 2 (GRIA2), mRNA	NM_000826	Hs.32763	NM_000826
GRM1	glutamate receptor, metabotropic 1 (GRM1), mRNA	NM_000838	Hs.32945	NM_000838
GRM7	glutamate receptor, metabotropic 7 (GRM7), transcript variant 2, mRNA	NM_181874	Hs.660131	NM_181874
GUCY2F	guanylate cyclase 2F, retinal (GUCY2F), mRNA	NM_001522	Hs.123074	L37378
HABP2	hyaluronan binding protein 2 (HABP2), mRNA	NM_004132	Hs.422542	NM_004132
HBLD2	HESB like domain containing 2 (HBLD2), mRNA	NM_030940	Hs.449291	NM_030940
HCFC1R1	host cell factor C1 regulator 1 (XPO1 dependent) (HCFC1R1), transcript variant 1, mRNA	NM_017885	Hs.423103	BM919270
HDAC5	histone deacetylase 5 (HDAC5), transcript variant 3, mRNA	NM_001015053	Hs.438782	NM_001015053
HHLA3	HERV-H LTR-associating 3 (HHLA3), transcript variant 2, mRNA	NM_007071	Hs.142245	NM_001036645
HIST1H2AD	histone cluster 1, H2ad (HIST1H2AD), mRNA	NM_021065	Hs.679229	DA450711
HIST1H2AE	histone cluster 1, H2ae (HIST1H2AE), mRNA	NM_021052	Hs.121017	BE741093
HIST1H2AG	histone cluster 1, H2ag (HIST1H2AG), mRNA	NM_021064	Hs.51011	BC067782
HIST1H2BE	histone cluster 1, H2be (HIST1H2BE), mRNA	NM_003523	Hs.534369	BP362977
HIST1H2BF	histone cluster 1, H2bf (HIST1H2BF), mRNA	NM_003522	Hs.182137	BC056264
HIST1H2BN	histone cluster 1, H2bn (HIST1H2BN), mRNA	NM_003520	Hs.673851	NM_003520
HIST2H2AA4	histone cluster 2, H2aa4 (HIST2H2AA4), mRNA	NM_001040874	Hs.701937	NM_001040874
HIST2H2AB	histone cluster 2, H2ab (HIST2H2AB), mRNA	NM_175065	Hs.664173	BC132811
HIST2H2AC	histone cluster 2, H2ac (HIST2H2AC), mRNA	NM_003517	Hs.408067	EL734895
HIST3H2A	histone cluster 3, H2a (HIST3H2A), mRNA	NM_033445	Hs.700190	BM465264
HIST3H2BB	histone cluster 3, H2bb (HIST3H2BB), mRNA	NM_175055	Hs.376691	DB010344
HIST3H2BB	Histone H2B type 3-B (H2B type 12).	ENST00000354653	Unknown	
HIST4H4	histone cluster 4, H4, mRNA (cDNA clone MGC:24116 IMAGE:4619662), complete cds.	BC020884	Unknown	
HKDC1	hexokinase domain containing 1 (HKDC1), mRNA	NM_025130	Unknown	
HKDC1	hexokinase domain containing 1 (HKDC1), mRNA	NM_025130	Unknown	
HMP19	HMP19 protein (HMP19), mRNA	NM_015980	Hs.559412	AK098398
HOXC13	homeobox C13 (HOXC13), mRNA	NM_017410	Hs.118608	NM_017410
HP	haptoglobin (HP), mRNA	NM_005143	Hs.513711	AK055872
HPR	haptoglobin-related protein (HPR), mRNA	NM_020995	Hs.655361	NM_020995
HPX	hemopexin (HPX), mRNA	NM_000613	Hs.426485	BC005395
HRG	histidine-rich glycoprotein (HRG), mRNA	NM_000412	Hs.1498	CR749302
HS3ST4	heparan sulfate (glucosamine) 3-O-sulfotransferase 4 (HS3ST4), mRNA	NM_006040	Hs.655275	NM_006040
HSD17B2	hydroxysteroid (17-beta) dehydrogenase 2 (HSD17B2), mRNA	NM_002153	Hs.162795	BX648557
HSD3B1	hydroxy-delta-5-steroid dehydrogenase, 3 beta- and steroid delta-isomerase 1 (HSD3B1), mRNA	NM_000862	Hs.364941	CD014103

HSP90AA1	heat shock protein 90kDa alpha (cytosolic), class A member 1 (HSP90AA1), transcript variant 2, mRNA	NM_005348	Hs.525600	NM_001017963
HSP90B1	heat shock protein 90kDa beta (Grp94), member 1 (HSP90B1), mRNA	NM_003299	Hs.192374	AB209534
HTN3	histatin 3 (HTN3), mRNA	NM_000200	Hs.654442	CR749410
HTR5A	5-hydroxytryptamine (serotonin) receptor 5A (HTR5A), mRNA	NM_024012	Hs.65791	AK094255
IDS	iduronate 2-sulfatase (Hunter syndrome) (IDS), transcript variant 1, mRNA	NM_000202	Hs.460960	NM_000202
IDS	Iduronate 2-sulfatase precursor (EC 3.1.6.13) (Alpha-L-iduronate sulfate sulfatase) (Idursulfase) .	ENST00000340855	Unknown	
IDS	iduronate 2-sulfatase (Hunter syndrome) (IDS), transcript variant 2, mRNA	NM_006123	Hs.460960	NM_000202
IFNGR1	interferon gamma receptor 1 (IFNGR1), mRNA	NM_000416	Hs.520414	AK127636
IFT122	intraflagellar transport 122 homolog (Chlamydomonas) (IFT122), transcript variant 3, mRNA	NM_018262	Hs.655284	NM_052985
IFT140	intraflagellar transport 140 homolog (Chlamydomonas) (IFT140), mRNA	NM_014714	Hs.389438	AB209020
IGFBP2	insulin-like growth factor binding protein 2, 36kDa (IGFBP2), mRNA	NM_000597	Hs.438102	AB209509
IGHM	human full-length cDNA clone CS0DD006YL02 of Neuroblastoma of (human).	BX161420	Hs.510635	AK090461
IGKC	immunoglobulin kappa constant, mRNA (cDNA clone MGC:88771 IMAGE:4576136), complete cds.	BC073764	Hs.449621	AK128524
IL10	interleukin 10 (IL10), mRNA	NM_000572	Hs.193717	BC022315
IL13RA1	interleukin 13 receptor, alpha 1 (IL13RA1), mRNA	NM_001560	Hs.496646	Y10659
IL2	interleukin 2 (IL2), mRNA	NM_000586	Hs.89679	S82692
INHA	inhibin, alpha (INHA), mRNA	NM_002191	Hs.407506	BC039076
INPP5F	inositol polyphosphate-5-phosphatase F (INPP5F), transcript variant 1, mRNA	NM_014937	Hs.369755	NM_014937
INPP5F	inositol polyphosphate-5-phosphatase F (INPP5F), transcript variant 1, mRNA	NM_014937	Hs.369755	NM_014937
IPP	intracisternal A particle-promoted polypeptide (IPP), mRNA	NM_005897	Hs.699548	NM_005897
IRF2BP2	interferon regulatory factor 2 binding protein 2 (IRF2BP2), transcript variant 1, mRNA	NM_182972	Hs.350268	NM_182972
ISG15	ISG15 ubiquitin-like modifier (ISG15), mRNA	NM_005101	Hs.458485	BM916335
ISGF3G	Transcriptional regulator ISGF3 subunit gamma (Interferon regulatory factor 9) (IRF-9) (IFN-alpha-responsive transcription factor subunit) (Interferon-stimulated gene factor 3 gamma) (ISGF3 p48 subunit) (ISGF- 3 gamma)....	ENST00000324076	Unknown	
ITK	IL2-inducible T-cell kinase (ITK), mRNA	NM_005546	Hs.558348	AB209622
ITM2B	integral membrane protein 2B (ITM2B), mRNA	NM_021999	Hs.699207	BX537657
ITPKA	inositol 1,4,5-trisphosphate 3-kinase A (ITPKA), mRNA	NM_002220	Hs.2722	BC026331
JTB	jumping translocation breakpoint (JTB), mRNA	NM_006694	Hs.6396	BQ062593
KCNA1	potassium voltage-gated channel, shaker-related subfamily, member 1 (episodic ataxia with myokymia) (KCNA1), mRNA	NM_000217	Hs.416139	NM_000217
KCNA1	potassium voltage-gated channel, shaker-related subfamily, member 1 (episodic ataxia with myokymia) (KCNA1), mRNA	NM_000217	Hs.416139	NM_000217
KCNIP2	Kv channel interacting protein 2 (KCNIP2), transcript variant 7, mRNA	NM_173197	Hs.97044	NM_014591
KCNJ10	potassium inwardly-rectifying channel, subfamily J, member 10 (KCNJ10), mRNA	NM_002241	Hs.408960	NM_002241
KCNMB3	potassium large conductance calcium-activated channel, subfamily M beta member 3 (KCNMB3), transcript variant 1, mRNA	NM_171828	Hs.591285	BC082272
KCTD16	potassium channel tetramerisation domain containing 16 (KCTD16), mRNA	NM_020768	Hs.693927	AB037738
KCTD16	potassium channel tetramerisation domain containing 16 (KCTD16), mRNA	NM_020768	Hs.693927	AB037738
KIAA0494	KIAA0494 (KIAA0494), mRNA	NM_014774	Hs.100874	AB007963
KIAA0513	KIAA0513 (KIAA0513), mRNA	NM_014732	Hs.301658	AB011085
KIAA0892	KIAA0892 (KIAA0892), mRNA	NM_015329	Hs.654939	NM_015329
KIAA0907	KIAA0907 (KIAA0907), mRNA	NM_014949	Hs.24656	AB020714
KIAA1045	KIAA1045, mRNA (cDNA clone IMAGE:5730505), containing frame-shift errors.	BC041169	Hs.7989	NM_015297
KIAA1394	KIAA1394 protein, mRNA (cDNA clone IMAGE:4310128), complete cds.	BC036557	Hs.502982	EF560746
KIAA1432	mRNA for KIAA1432 protein, partial cds.	AB037853	Hs.211520	AB037853
KIAA1446	likely ortholog of rat brain-enriched guanylate kinase-associated protein (KIAA1446), mRNA	NM_020836	Hs.211751	AB040879
KIAA1486	CDNA FLJ30664 fis, clone FCBBF1000604, weakly similar to Myosin heavy chain Myr 8b.	ENST00000272907	Unknown	
KIAA1614	Novel protein.	ENST00000367588	Unknown	
KIAA1683	KIAA1683 (KIAA1683), mRNA	NM_025249	Hs.313471	AB051470
KIAA1715	KIAA1715 (KIAA1715), mRNA	NM_030650	Hs.209561	CR936742
KIAA1797	KIAA1797 (KIAA1797), mRNA	NM_017794	Hs.408652	NM_017794
KIAA1853	KIAA1853 (KIAA1853), mRNA	NM_194286	Hs.112577	NM_194286
KIAA1875	KIAA1875 (KIAA1875), mRNA	NM_032529	Unknown	

KIAA1946	mRNA for KIAA1946 protein.	AB075826	Hs.28872	AB075826
KIAA2013	KIAA2013 (KIAA2013), mRNA	ENST00000376572	Unknown	
KIAA2013	KIAA2013 (KIAA2013), mRNA	ENST00000376572	Unknown	
KIF5A	kinesin family member 5A (KIF5A), mRNA	NM_004984	Hs.151219	AB210045
KITLG	KIT ligand (KITLG), transcript variant b, mRNA	NM_000899	Hs.1048	NM_000899
KLHL1	kelch-like 1 (Drosophila) (KLHL1), mRNA	NM_020866	Hs.508201	AB040923
KLK5	kallikrein-related peptidase 5 (KLK5), transcript variant 1, mRNA	NM_012427	Hs.50915	AY359010
KRT40	keratin 40 (KRT40), mRNA	NM_182497	Hs.567666	NM_182497
KRTCAP2	keratinocyte associated protein 2 (KRTCAP2), mRNA	NM_173852	Hs.516671	AK024167
L1CAM	L1 cell adhesion molecule (L1CAM), transcript variant 1, mRNA	NM_000425	Hs.522818	NM_000425
L3MBTL4	l(3)mbt-like 4 (Drosophila) (L3MBTL4), mRNA	NM_173464	Hs.128279	BC039316
LAMA1	laminin, alpha 1 (LAMA1), mRNA	NM_005559	Hs.270364	NM_005559
LAPTM4A	lysosomal-associated protein transmembrane 4 alpha (LAPTM4A), mRNA	NM_014713	Hs.467807	BF697273
LAYN	cDNA FLJ39606 fis, clone SKNSH2006234.	AK096925	Hs.503831	AK096925
LAYN	layilin (LAYN), mRNA	NM_178834	Hs.503831	AK096925
LBP	lipopolysaccharide binding protein (LBP), mRNA	NM_004139	Hs.154078	NM_004139
LECT2	leukocyte cell-derived chemotaxin 2 (LECT2), mRNA	NM_002302	Hs.512580	NM_002302
LEPRE1	leucine proline-enriched proteoglycan (leprecan) 1 (LEPRE1), mRNA	NM_022356	Hs.437656	NM_022356
LHFPL2	lipoma HMGIC fusion partner-like 2 (LHFPL2), mRNA	NM_005779	Hs.79299	NM_005779
LHX9	LIM homeobox 9 (LHX9), transcript variant 1, mRNA	NM_020204	Hs.442578	NM_020204
LMX1A	LIM homeobox transcription factor 1, alpha (LMX1A), transcript variant 1, mRNA	NM_177398	Hs.667312	AK127724
LOC147650	ALLA2487.	ENST00000331594	Unknown	
LOC151174	chromosome 2 unknown mRNA.	ENST00000334973	Unknown	
LOC151438	cDNA FLJ31315 fis, clone LIVER1000303.	AK055877	Hs.516245	AK055877
LOC153811	cDNA FLJ11672 fis, clone HEMBA1004733.	AK021734	Hs.144515	AK021734
LOC165186	CDNA FLJ43756 fis, clone TESTI2045920.	ENST00000379557	Unknown	
LOC196541	hypothetical protein LOC196541 (LOC196541), mRNA	NM_001010977	Hs.508623	AK092513
LOC201229	hypothetical protein LOC201229 (LOC201229), mRNA	NM_001076680	Hs.700571	NM_000625
LOC255275	similar to myeloid-associated differentiation marker (LOC728889), mRNA	ENST00000330655	Unknown	
LOC255411	cDNA FLJ27495 fis, clone TST03995.	ENST00000380487	Unknown	
LOC257396	cDNA FLJ40574 fis, clone THYMU2007036.	AK097893	Hs.12326	BC041894
LOC374973	LOC374973, mRNA (cDNA clone IMAGE:5269821), partial cds.	ENST00000334122	Unknown	
LOC387856	similar to expressed sequence AI836003 (LOC387856), mRNA	NM_001013635	Hs.251699	BC049387
LOC389517	similar to Williams Beuren syndrome chromosome region 19 (LOC389517), mRNA	NM_001032389	Unknown	
LOC389517	similar to Williams Beuren syndrome chromosome region 19 (LOC389517), mRNA	NM_001032389	Unknown	
LOC400590	hypothetical LOC400590, mRNA (cDNA clone MGC:70830 IMAGE:5248762), complete cds.	BC062632	Hs.558901	BC062632
LOC401431	hypothetical gene LOC401431 (LOC401431), mRNA	NM_001008745	Unknown	
LOC441258	cDNA: FLJ20949 fis, clone ADSE01902.	AK024602	Hs.571275	AK024602
LOC493869	similar to RIKEN cDNA 2310016C16 (LOC493869), mRNA	NM_001008397	Hs.700615	BX537894
LOC493869	similar to RIKEN cDNA 2310016C16 (LOC493869), mRNA	NM_001008397	Hs.700615	BX537894
LOC493869	similar to RIKEN cDNA 2310016C16 (LOC493869), mRNA	NM_001008397	Hs.700615	BX537894
LOC493869	similar to RIKEN cDNA 2310016C16 (LOC493869), mRNA	NM_001008397	Hs.700615	BX537894
LOC493869	similar to RIKEN cDNA 2310016C16 (LOC493869), mRNA	NM_001008397	Hs.700615	BX537894
LOC493869	similar to RIKEN cDNA 2310016C16 (LOC493869), mRNA	NM_001008397	Hs.700615	BX537894
LOC493869	similar to RIKEN cDNA 2310016C16 (LOC493869), mRNA	NM_001008397	Hs.700615	BX537894
LOC493869	similar to RIKEN cDNA 2310016C16 (LOC493869), mRNA	NM_001008397	Hs.700615	BX537894
LOC493869	similar to RIKEN cDNA 2310016C16 (LOC493869), mRNA	NM_001008397	Hs.700615	BX537894
LOC493869	similar to RIKEN cDNA 2310016C16 (LOC493869), mRNA	NM_001008397	Hs.700615	BX537894
LOC493869	similar to RIKEN cDNA 2310016C16 (LOC493869), mRNA	NM_001008397	Hs.700615	BX537894
LOC497190	secretory protein LOC497190 (LOC497190), mRNA	NM_001011880	Hs.556045	AL833339
LOC643037	PREDICTED: hypothetical LOC643037 (LOC643037), mRNA	XM_926406	Hs.97691	XM_926406
LOC643650	hypothetical protein LOC643650, mRNA (cDNA clone IMAGE:5019308), partial cds.	BC033221	Hs.661008	BC033221

LOC643744	PREDICTED: similar to D-PCa-2 protein isoform c (LOC643744), mRNA	XR_017206	Hs.482549	BE907016
LOC644246	hypothetical protein LOC644246, mRNA (cDNA clone IMAGE:4730995), partial cds.	BC020847	Hs.644600	BG620930
LOC646627	clone DNA59613 phospholipase inhibitor (UNQ511) mRNA, complete cds.	AY358469	Hs.632511	NM_001085474
LOC647323	hypothetical protein LOC647323, mRNA (cDNA clone IMAGE:5205242), partial cds.	BC027871	Hs.652830	BC027871
LOC728449	Annexin A8 (Annexin VIII) (Vascular anticoagulant-beta) (VAC-beta).	ENST00000335083	Unknown	
LOC728783	PREDICTED: hypothetical protein LOC728783 (LOC728783), mRNA	XR_015377	Hs.636711	XR_015377
LOC90835	hypothetical protein LOC90835 (LOC90835), mRNA	NM_001014979	Hs.65735	AL122109
LOH11CR2A	loss of heterozygosity, 11, chromosomal region 2, gene A (LOH11CR2A), transcript variant 1, mRNA	NM_014622	Hs.152944	AY366508
LOR	loricrin (LOR), mRNA	NM_000427	Hs.251680	BC108290
LPGAT1	lysophosphatidylglycerol acyltransferase 1 (LPGAT1), mRNA	NM_014873	Hs.654626	NM_014873
LRFN1	leucine rich repeat and fibronectin type III domain containing 1, mRNA (cDNA clone IMAGE:3860672), partial cds.	BC014678	Hs.97860	NM_020862
LRRC42	leucine rich repeat containing 42 (LRRC42), mRNA	NM_052940	Hs.40094	AK090881
LRRC7	leucine rich repeat containing 7 (LRRC7), mRNA	NM_020794	Hs.479658	BX648943
LYPLAL1	lysophospholipase-like 1 (LYPLAL1), mRNA	NM_138794	Hs.657617	CR593519
MAB21L1	mab-21-like 1 (C. elegans) (MAB21L1), mRNA	NM_005584	Hs.584776	AB073388
MACF1	microtubule-actin crosslinking factor 1 (MACF1), transcript variant 2, mRNA	NM_033044	Hs.580782	NM_033044
MAEA	macrophage erythroblast attacher (MAEA), transcript variant 1, mRNA	NM_001017405	Hs.139896	AB208907
MAEA	macrophage erythroblast attacher (MAEA), transcript variant 1, mRNA	NM_001017405	Hs.139896	AB208907
MAF	short form transcription factor C-MAF (c-maf) mRNA, complete cds.	AF055376	Hs.699396	NM_001031804
MAFB	v-maf musculoaponeurotic fibrosarcoma oncogene homolog B (avian) (MAFB), mRNA	NM_005461	Hs.651210	NM_005461
MAGED1	melanoma antigen family D, 1 (MAGED1), transcript variant 1, mRNA	NM_001005333	Hs.5258	NM_001005333
MAK10	MAK10 homolog, amino-acid N-acetyltransferase subunit, (S. cerevisiae) (MAK10), mRNA	NM_024635	Hs.436098	NM_024635
MALAT1	metastasis associated lung adenocarcinoma transcript 1 (non-coding RNA) (MALAT1) on chromosome 11	NR_002819	Unknown	
MAMDC4	MAM domain containing 4 (MAMDC4), mRNA	NM_206920	Hs.376780	AY358419
MAP1LC3C	microtubule-associated protein 1 light chain 3 gamma (MAP1LC3C), mRNA	NM_001004343	Hs.534971	NM_001004343
MAPT	microtubule-associated protein tau (MAPT), transcript variant 1, mRNA	NM_016835	Hs.101174	AK226139
MAPT	microtubule-associated protein tau (MAPT), transcript variant 1, mRNA	NM_016835	Hs.101174	AK226139
MAPT	microtubule-associated protein tau (MAPT), transcript variant 1, mRNA	NM_016835	Hs.101174	AK226139
MAPT	microtubule-associated protein tau (MAPT), transcript variant 1, mRNA	NM_016835	Hs.101174	AK226139
MAPT	microtubule-associated protein tau (MAPT), transcript variant 1, mRNA	NM_016835	Hs.101174	AK226139
MAPT	microtubule-associated protein tau (MAPT), transcript variant 1, mRNA	NM_016835	Hs.101174	AK226139
MAPT	microtubule-associated protein tau (MAPT), transcript variant 1, mRNA	NM_016835	Hs.101174	AK226139
MAPT	microtubule-associated protein tau (MAPT), transcript variant 1, mRNA	NM_016835	Hs.101174	AK226139
MAPT	microtubule-associated protein tau (MAPT), transcript variant 1, mRNA	NM_016835	Hs.101174	AK226139
MCF2L	cDNA FLJ12122 fis, clone MAMMA1000129.	AK022184	Unknown	
MDFI	MyoD family inhibitor (MDFI), mRNA	NM_005586	Hs.520119	CR594049
MEGF6	multiple EGF-like-domains 6 (MEGF6), mRNA	NM_001409	Hs.593645	NM_001409
METRN	meteorin, glial cell differentiation regulator (METRN), mRNA	NM_024042	Hs.533772	BM553584
MFAP2	microfibrillar-associated protein 2 (MFAP2), transcript variant 1, mRNA	NM_017459	Hs.389137	BC028033
MGC15523	hypothetical protein MGC15523 (MGC15523), transcript variant 1, mRNA	NM_001037984	Hs.352240	NM_001037984
MGC16075	hypothetical protein MGC16075, mRNA (cDNA clone MGC:16075 IMAGE:3616854), complete cds.	BC007354	Hs.488236	BC007354
MGC29891	GA repeat binding protein, beta 2	ENST00000368918	Unknown	
MGC29891	hypothetical protein MGC29891 (MGC29891), mRNA	NM_144618	Hs.654699	BC027033
MGC4655	hypothetical protein MGC4655 (MGC4655), mRNA	NM_033309	Hs.592061	NM_033309
MGC88374	similar to CG32662-PA (MGC88374), mRNA	NM_001004331	Unknown	
MME	membrane metallo-endopeptidase (neutral endopeptidase, enkephalinase) (MME), transcript variant 2b, mRNA	NM_007289	Hs.307734	NM_007289
MME	membrane metallo-endopeptidase (neutral endopeptidase, enkephalinase) (MME), transcript variant 2b, mRNA	NM_007289	Hs.307734	NM_007289
MOSPD1	motile sperm domain containing 1 (MOSPD1), mRNA	NM_019556	Hs.590789	BC005700
MPZL1	myelin protein zero-like 1 (MPZL1), transcript variant 1, mRNA	NM_003953	Hs.493919	NM_003953
MS4A8B	membrane-spanning 4-domains, subfamily A, member 8B (MS4A8B), mRNA	NM_031457	Hs.150878	AF237905

MSX1	msh homeobox 1 (MSX1), mRNA	NM_002448	Hs.424414	NM_002448
MSX2	msh homeobox 2 (MSX2), mRNA	NM_002449	Hs.89404	NM_002449
MT3	metallothionein 3 (growth inhibitory factor (neurotrophic)) (MT3), mRNA	NM_005954	Hs.73133	BM544485
MT3	metallothionein 3 (growth inhibitory factor (neurotrophic)) (MT3), mRNA	NM_005954	Hs.73133	BM544485
MTM1	myotubularin 1 (MTM1), mRNA	NM_000252	Hs.655056	NM_000252
MTM1	myotubularin 1 (MTM1), mRNA	NM_000252	Hs.655056	NM_000252
MUC17	mucin 17, cell surface associated (MUC17), mRNA	NM_001040105	Hs.271819	NM_001040105
MXD3	cDNA FLJ35523 fis, clone SPLEN2001503.	AK092842	Hs.699373	AK092842
MYL1	myosin, light chain 1, alkali; skeletal, fast (MYL1), transcript variant 1f, mRNA	NM_079420	Hs.187338	BF790783
MYLC2PL	myosin light chain 2, precursor lymphocyte-specific, mRNA (cDNA clone MGC:3479 IMAGE:3616529), complete cds.	BC002778	Hs.247831	BC002778
MYT1L	myelin transcription factor 1-like (MYT1L), mRNA	NM_015025	Hs.434418	NM_015025
MYT1L	myelin transcription factor 1-like (MYT1L), mRNA	NM_015025	Hs.434418	NM_015025
NAT14	N-acetyltransferase 14 (NAT14), mRNA	NM_020378	Hs.31854	BM805249
NCF2	neutrophil cytosolic factor 2 (65kDa, chronic granulomatous disease, autosomal 2) (NCF2), mRNA	NM_000433	Hs.587558	AB209647
NCSTN	nicastrin (NCSTN), mRNA	NM_015331	Hs.517249	BC047621
NEB	nebulin (NEB), mRNA	NM_004543	Hs.588655	NM_004543
NEURL	neuralized homolog (Drosophila) (NEURL), mRNA	NM_004210	Hs.654845	BX648827
NGEF	neuronal guanine nucleotide exchange factor (NGEF), mRNA	NM_019850	Hs.97316	NM_019850
NGLY1	N-glycanase 1 (NGLY1), mRNA	NM_018297	Hs.368960	AB209549
NISCH	nischarin (NISCH), mRNA	NM_007184	Hs.435290	NM_007184
NISCH	nischarin (NISCH), mRNA	NM_007184	Hs.435290	NM_007184
NOL4	nucleolar protein 4 (NOL4), mRNA	NM_003787	Hs.514795	NM_003787
NP373359	GB|AL445468.8|CAC41956.1 bA74P14.2 (novel protein)	NP373359	Unknown	
NPAL3	NIPA-like domain containing 3 (NPAL3), mRNA	NM_020448	Hs.523442	BC063583
NPVF	neuropeptide VF precursor (NPVF), mRNA	NM_022150	Hs.699493	NM_022150
NR2E1	nuclear receptor subfamily 2, group E, member 1 (NR2E1), mRNA	NM_003269	Hs.157688	NM_003269
NRG2	neuregulin 2 (NRG2), transcript variant 3, mRNA	NM_013982	Hs.408515	NM_013982
NSF	N-ethylmaleimide-sensitive factor (NSF), mRNA	NM_006178	Hs.431279	AF135168
NTRK2	mRNA; cDNA DKFZp686D1394 (from clone DKFZp686D1394).	BX649001	Hs.494312	NM_001018065
NTRK3	neurotrophic tyrosine kinase, receptor, type 3 (NTRK3), transcript variant 1, mRNA	NM_001012338	Hs.410969	NM_001007156
NY-REN-7	PREDICTED: NY-REN-7 antigen (NY-REN-7), mRNA	XM_001132409	Unknown	
OCRL	oculocerebrorenal syndrome of Lowe (OCRL), transcript variant a, mRNA	NM_000276	Hs.126357	AK226116
OCRL	oculocerebrorenal syndrome of Lowe (OCRL), transcript variant a, mRNA	NM_000276	Hs.126357	AK226116
ODF3L1	outer dense fiber of sperm tails 3-like 1 (ODF3L1), mRNA	NM_175881	Hs.144348	BM559646
OR10T2	olfactory receptor, family 10, subfamily T, member 2 (OR10T2), mRNA	NM_001004475	Hs.553588	NM_001004475
OR2B6	olfactory receptor, family 2, subfamily B, member 6 (OR2B6), mRNA	NM_012367	Hs.532145	BC109251
OR7E91P	olfactory receptor, family 7, subfamily E, member 91 pseudogene (OR7E91P) on chromosome 2	NR_002185	Unknown	
ORMDL3	ORM1-like 3 (S. cerevisiae) (ORMDL3), mRNA	NM_139280	Hs.514151	AK093063
OTOA	otoancorin (OTOA), transcript variant 2, mRNA	NM_170664	Hs.408336	NM_144672
OTUD5	OTU domain containing 5 (OTUD5), mRNA	NM_017602	Hs.496098	NM_017602
OTUD7B	OTU domain containing 7B (OTUD7B), mRNA	NM_020205	Hs.98322	NM_020205
P11	26 serine protease (P11), mRNA	NM_006025	Hs.997	AK075446
P4HA1	procollagen-proline, 2-oxoglutarate 4-dioxygenase (proline 4-hydroxylase), alpha polypeptide I (P4HA1), transcript variant 1, mRNA	NM_000917	Hs.500047	BX648829
PACRG	PARK2 co-regulated (PACRG), mRNA	NM_152410	Hs.25791	NM_152410
PADI2	peptidyl arginine deiminase, type II (PADI2), mRNA	NM_007365	Hs.33455	NM_007365
PADI2	Protein-arginine deiminase type-2 (EC 3.5.3.15) (Protein-arginine deiminase type II) (Peptidylarginine deiminase II) (PAD-H19).	ENST00000375486	Unknown	
PADI2	peptidyl arginine deiminase, type II (PADI2), mRNA	NM_007365	Hs.33455	NM_007365
PARP6	poly (ADP-ribose) polymerase family, member 6 (PARP6), mRNA	NM_020214	Hs.270244	AK091172
PCP2	Purkinje cell protein 2 (PCP2), mRNA	NM_174895	Hs.591400	BC025387
PCSK1N	proprotein convertase subtilisin/kexin type 1 inhibitor (PCSK1N), mRNA	NM_013271	Hs.522640	BM805628
PCSK6	proprotein convertase subtilisin/kexin type 6 (PCSK6), transcript variant 1, mRNA	NM_002570	Hs.498494	NM_002570

PCYOX1	prenylcysteine oxidase 1 (PCYOX1), mRNA	NM_016297	Hs.591572	AB020715
PDGFD	platelet derived growth factor D (PDGFD), transcript variant 1, mRNA	NM_025208	Hs.352298	BC030645
PDIA2	protein disulfide isomerase family A, member 2 (PDIA2), mRNA	NM_006849	Hs.66581	NM_006849
PHACTR4	phosphatase and actin regulator 4 (PHACTR4), transcript variant 1, mRNA	NM_001048183	Hs.225641	NM_001048183
PIK4CA	phosphatidylinositol 4-kinase, catalytic, alpha polypeptide (PIK4CA), transcript variant 1, mRNA	NM_002650	Hs.529438	NM_058004
PIK4CA	phosphatidylinositol 4-kinase, catalytic, alpha polypeptide (PIK4CA), transcript variant 1, mRNA	NM_002650	Hs.529438	NM_058004
PIK4CB	phosphatidylinositol 4-kinase, catalytic, beta polypeptide (PIK4CB), mRNA	NM_002651	Hs.632465	BC040300
PITX1	paired-like homeodomain transcription factor 1 (PITX1), mRNA	NM_002653	Hs.84136	NM_002653
PLA2G1B	phospholipase A2, group IB (pancreas) (PLA2G1B), mRNA	NM_000928	Hs.992	BE969664
PLAC2	placenta-specific 2	ENST00000312785	Unknown	
PLAC2	placenta-specific 2 (PLAC2), mRNA	NM_153375	Unknown	
PLB1	phospholipase B1, mRNA (cDNA clone IMAGE:6147102), complete cds.	BC065041	Hs.444933	NM_153021
PLB1	phospholipase B1 (PLB1), mRNA	NM_153021	Hs.444933	NM_153021
PLB1	phospholipase B1 (PLB1), mRNA	NM_153021	Hs.444933	NM_153021
PLCB1	phospholipase C, beta 1 (phosphoinositide-specific) (PLCB1), transcript variant 1, mRNA	NM_015192	Hs.431173	AB011153
PLGLA1	PREDICTED: plasminogen-like A1, transcript variant 6 (PLGLA1), mRNA	XM_934128	Unknown	
PLGLA1	PREDICTED: plasminogen-like A1, transcript variant 6 (PLGLA1), mRNA	XM_934128	Unknown	
PMCH	pro-melanin-concentrating hormone (PMCH), mRNA	NM_002674	Hs.694729	BG216766
PMCHL1	pro-melanin-concentrating hormone-like 1 protein (PMCHL1) mRNA, complete cds, alternatively spliced.	AY008410	Unknown	
PMS2L5	postmeiotic segregation increased 2-like 5 (PMS2L5), mRNA	NM_174930	Hs.647025	D38502
PNKD	paroxysmal nonkinesiogenic dyskinesia (PNKD), transcript variant 3, mRNA	NM_001077399	Hs.98475	NM_015488
POLR2B	polymerase (RNA) II (DNA directed) polypeptide B, 140kDa (POLR2B), mRNA	NM_000938	Hs.602757	BC023503
POMT1	protein-O-mannosyltransferase 1 (POMT1), transcript variant 1, mRNA	NM_007171	Hs.522449	AB209430
POMT2	protein-O-mannosyltransferase 2 (POMT2), mRNA	NM_013382	Hs.132989	AB209114
PPCS	phosphopantothenoylcysteine synthetase (PPCS), transcript variant 1, mRNA	NM_024664	Hs.473495	AL832217
PPCS	phosphopantothenoylcysteine synthetase (PPCS), transcript variant 1, mRNA	NM_024664	Hs.473495	AL832217
PPP1R3A	protein phosphatase 1, regulatory (inhibitor) subunit 3A (glycogen and sarcoplasmic reticulum binding subunit, skeletal muscle) (PPP1R3A), mRNA	NM_002711	Hs.458309	NM_002711
PPP1R3E	mRNA for FLJ00089 protein, partial cds.	AK024489	Unknown	
PPP3CA	protein phosphatase 3 (formerly 2B), catalytic subunit, alpha isoform (calcineurin A alpha) (PPP3CA), mRNA	NM_000944	Hs.435512	AL353950
PPP3CA	protein phosphatase 3 (formerly 2B), catalytic subunit, alpha isoform (calcineurin A alpha) (PPP3CA), mRNA	NM_000944	Hs.435512	AL353950
PPYR1	pancreatic polypeptide receptor 1 (PPYR1), mRNA	NM_005972	Hs.524719	NM_005972
PRDX1	peroxiredoxin 1 (PRDX1), transcript variant 1, mRNA	NM_002574	Hs.180909	BF185567
PRKAB2	protein kinase, AMP-activated, beta 2 non-catalytic subunit (PRKAB2), mRNA	NM_005399	Hs.50732	NM_005399
PRPH	peripherin (PRPH), mRNA	NM_006262	Hs.37044	AK125587
PRUNE	prune homolog (Drosophila) (PRUNE), mRNA	NM_021222	Hs.78524	AF051907
PRUNE	prune homolog (Drosophila) (PRUNE), mRNA	NM_021222	Hs.78524	AF051907
PSEN2	presenilin 2 (Alzheimer disease 4) (PSEN2), transcript variant 1, mRNA	NM_000447	Hs.25363	BC006365
PSEN2	presenilin 2 (Alzheimer disease 4) (PSEN2), transcript variant 1, mRNA	NM_000447	Hs.25363	BC006365
PSMD4	proteasome (prosome, macropain) 26S subunit, non-ATPase, 4 (PSMD4), transcript variant 2, mRNA	NM_153822	Unknown	
PTGDS	prostaglandin D2 synthase 21kDa (brain) (PTGDS), mRNA	NM_000954	Hs.446429	BM805807
PTGER3	prostaglandin E receptor 3 (subtype EP3) (PTGER3), transcript variant 5, mRNA	NM_198715	Hs.445000	NM_198715
PTHR1	parathyroid hormone receptor 1 (PTHR1), mRNA	NM_000316	Hs.1019	AF495723
PTPRC	protein tyrosine phosphatase, receptor type, C (PTPRC), transcript variant 1, mRNA	NM_002838	Hs.654514	NM_002838
PTPRC	protein tyrosine phosphatase, receptor type, C (PTPRC), transcript variant 1, mRNA	NM_002838	Hs.654514	NM_002838
PTPRC	protein tyrosine phosphatase, receptor type, C (PTPRC), transcript variant 1, mRNA	NM_002838	Hs.654514	NM_002838
PTPRC	protein tyrosine phosphatase, receptor type, C (PTPRC), transcript variant 1, mRNA	NM_002838	Hs.654514	NM_002838
PTPRC	protein tyrosine phosphatase, receptor type, C (PTPRC), transcript variant 1, mRNA	NM_002838	Hs.654514	NM_002838
PTPRC	protein tyrosine phosphatase, receptor type, C (PTPRC), transcript variant 1, mRNA	NM_002838	Hs.654514	NM_002838
PTPRC	protein tyrosine phosphatase, receptor type, C (PTPRC), transcript variant 1, mRNA	NM_002838	Hs.654514	NM_002838
PTPRC	protein tyrosine phosphatase, receptor type, C (PTPRC), transcript variant 1, mRNA	NM_002838	Hs.654514	NM_002838

PTPRT	protein tyrosine phosphatase, receptor type, T (PTPRT), transcript variant 1, mRNA	NM_133170	Hs.526879	NM_133170
RAB33B	RAB33B, member RAS oncogene family (RAB33B), mRNA	NM_031296	Hs.591679	AL136904
RAB36	RAB36, member RAS oncogene family (RAB36), mRNA	NM_004914	Hs.369557	AB023061
RAI2	retinoic acid induced 2 (RAI2), mRNA	NM_021785	Hs.701485	NM_021785
RAP2C	RAP2C, member of RAS oncogene family (RAP2C), mRNA	NM_021183	Hs.119889	AY298955
RASGEF1B	RasGEF domain family, member 1B (RASGEF1B), mRNA	NM_152545	Hs.591696	BX648337
RASSF5	Ras association (RalGDS/AF-6) domain family 5 (RASSF5), transcript variant 1, mRNA	NM_182663	Hs.497579	NM_182663
RBM10	RNA binding motif protein 10 (RBM10), transcript variant 1, mRNA	NM_005676	Hs.401509	NM_005676
RBM5	RNA binding motif protein 5 (RBM5), mRNA	NM_005778	Hs.439480	AB208813
RDX	radixin (RDX), mRNA	NM_002906	Hs.263671	NM_002906
REEP5	receptor accessory protein 5 (REEP5), mRNA	NM_005669	Hs.429608	NM_005669
REG3G	regenerating islet-derived 3 gamma (REG3G), transcript variant 1, mRNA	NM_001008387	Hs.447084	NM_001008387
RENBP	renin binding protein (RENBP), mRNA	NM_002910	Hs.158331	NM_002910
RERE	arginine-glutamic acid dipeptide (RE) repeats (RERE), transcript variant 1, mRNA	NM_012102	Hs.463041	NM_012102
RGS7BP	regulator of G-protein signalling 7 binding protein (RGS7BP), mRNA	NM_001029875	Hs.657133	NM_001029875
RGS9BP	regulator of G protein signalling 9 binding protein (RGS9BP), mRNA	NM_207391	Hs.528491	AK124499
RHCE	Rh blood group, CcEe antigens (RHCE), transcript variant 1, mRNA	NM_020485	Hs.449968	NM_020485
RIPK5	receptor interacting protein kinase 5 (RIPK5), transcript variant 1, mRNA	NM_015375	Hs.6874	AY429674
RNF13	ring finger protein 13 (RNF13), transcript variant 1, mRNA	NM_007282	Hs.12333	NM_007282
RNF187	ring finger protein 187, mRNA (cDNA clone IMAGE:3633225), partial cds.	BC012758	Hs.356377	XM_928029
RNF6	ring finger protein (C3H2C3 type) 6 (RNF6), transcript variant 1, mRNA	NM_005977	Hs.136885	NM_005977
RNF7	ring finger protein 7 (RNF7), transcript variant 1, mRNA	NM_014245	Hs.134623	NM_183237
ROPN1L	ropporin 1-like (ROPN1L), mRNA	NM_031916	Hs.381089	BM563234
ROPN1L	ropporin 1-like (ROPN1L), mRNA	NM_031916	Hs.381089	BM563234
RORB	mRNA; cDNA DKFZp686J0395 (from clone DKFZp686J0395).	BX647070	Hs.494178	BX647070
RORB	RAR-related orphan receptor B (RORB), mRNA	NM_006914	Hs.494178	BX647070
RP11-217H1.1	implantation-associated protein (DKFZp564K142), mRNA	NM_032121	Hs.323562	BC060842
RP11-393H10.2	hypothetical protein FLJ14503 (FLJ14503), mRNA	NM_152780	Hs.127951	BC089400
RP13-347D8.3	mRNA for KIAA1210 protein, partial cds.	AB033036	Hs.97594	NM_020721
RP13-36C9.6	cancer/testis antigen CT45-5 (CT45-5), mRNA	NM_001007551	Hs.535081	NM_001007551
RP5-1054A22.3	KIAA1755 protein (KIAA1755), mRNA	NM_001029864	Hs.472690	NM_001029864
RP5-1054A22.3	KIAA1755 protein (KIAA1755), mRNA	NM_001029864	Hs.472690	NM_001029864
RPE65	retinal pigment epithelium-specific protein 65kDa (RPE65), mRNA	NM_000329	Hs.2133	U18991
RPIP8	RaP2 interacting protein 8 (RPIP8), mRNA	NM_006695	Hs.500197	AK123330
RPLP1	ribosomal protein, large, P1 (RPLP1), transcript variant 1, mRNA	NM_001003	Hs.356502	BQ278494
RPLP1	ribosomal protein, large, P1 (RPLP1), transcript variant 1, mRNA	NM_001003	Hs.356502	BQ278494
RTCD1	RNA terminal phosphate cyclase domain 1 (RTCD1), mRNA	NM_003729	Hs.552835	AL080219
RTCD1	RNA 3'-terminal phosphate cyclase (EC 6.5.1.4) (RNA-3'-phosphate cyclase) (RNA cyclase).	ENST00000370128	Unknown	
RTN1	reticulon 1 (RTN1), transcript variant 1, mRNA	NM_021136	Hs.368626	NM_021136
RUFY2	RUN and FYVE domain containing 2 (RUFY2), transcript variant 2, mRNA	NM_001042417	Hs.653144	NM_017987
RUFY3	RUN and FYVE domain containing 3 (RUFY3), transcript variant 2, mRNA	NM_014961	Hs.7972	BC051716
S80491	stem cell factor {alternatively spliced} .	S80491	Hs.661108	BM688124
SAA4	serum amyloid A4, constitutive (SAA4), mRNA	NM_006512	Hs.654493	BG564326
SAA4	serum amyloid A4, constitutive (SAA4), mRNA	NM_006512	Hs.654493	BG564326
SAA4	serum amyloid A4, constitutive (SAA4), mRNA	NM_006512	Hs.654493	BG564326
SAA4	serum amyloid A4, constitutive (SAA4), mRNA	NM_006512	Hs.654493	BG564326
SAA4	serum amyloid A4, constitutive (SAA4), mRNA	NM_006512	Hs.654493	BG564326
SAA4	serum amyloid A4, constitutive (SAA4), mRNA	NM_006512	Hs.654493	BG564326
SAA4	serum amyloid A4, constitutive (SAA4), mRNA	NM_006512	Hs.654493	BG564326
SAA4	serum amyloid A4, constitutive (SAA4), mRNA	NM_006512	Hs.654493	BG564326
SAA4	serum amyloid A4, constitutive (SAA4), mRNA	NM_006512	Hs.654493	BG564326
SAA4	serum amyloid A4, constitutive (SAA4), mRNA	NM_006512	Hs.654493	BG564326

SAG	S-antigen; retina and pineal gland (arrestin) (SAG), mRNA	NM_000541	Hs.32721	BX647827
SCG2	secretogranin II (chromogranin C) (SCG2), mRNA	NM_003469	Hs.516726	BC022509
SCGB1A1	secretoglobin, family 1A, member 1 (uteroglobin) (SCGB1A1), mRNA	NM_003357	Hs.523732	BI819219
SCGB1D1	secretoglobin, family 1D, member 1 (SCGB1D1), mRNA	NM_006552	Hs.202686	CB987582
SCRN3	secernin 3 (SCRN3), mRNA	NM_024583	Hs.470679	NM_024583
SDC4	syndecan 4 (amphiglycan, ryudocan) (SDC4), mRNA	NM_002999	Hs.632267	NM_002999
SERPINA3	serpin peptidase inhibitor, clade A (alpha-1 antiproteinase, antitrypsin), member 3 (SERPINA3), mRNA	NM_001085	Hs.534293	AK093049
SERPINA3	serpin peptidase inhibitor, clade A (alpha-1 antiproteinase, antitrypsin), member 3 (SERPINA3), mRNA	NM_001085	Hs.534293	AK093049
SERPINI2	serpin peptidase inhibitor, clade I (pancpin), member 2 (SERPINI2), mRNA	NM_006217	Hs.445555	BC027859
SEZ6L	seizure related 6 homolog (mouse)-like (SEZ6L), mRNA	NM_021115	Hs.194766	AB023144
SHD	Src homology 2 domain containing transforming protein D (SHD), mRNA	NM_020209	Hs.7423	AK056268
SLC13A4	solute carrier family 13 (sodium/sulfate symporters), member 4 (SLC13A4), mRNA	NM_012450	Hs.490241	AB208809
SLC16A6	solute carrier family 16, member 6 (monocarboxylic acid transporter 7) (SLC16A6), mRNA	NM_004694	Hs.42645	BC064832
SLC22A3	solute carrier family 22 (extraneuronal monoamine transporter), member 3 (SLC22A3), mRNA	NM_021977	Hs.567337	BX640965
SLC25A44	solute carrier family 25, member 44 (SLC25A44), mRNA	NM_014655	Hs.532375	BC039854
SLC26A11	solute carrier family 26, member 11 (SLC26A11), mRNA	NM_173626	Hs.4866	BC035900
SLC2A11	solute carrier family 2 (facilitated glucose transporter), member 11 (SLC2A11), transcript variant 1, mRNA	NM_030807	Hs.632772	AK092466
SLC2A12	solute carrier family 2 (facilitated glucose transporter), member 12 (SLC2A12), mRNA	NM_145176	Hs.486508	AL833602
SLC2A2	solute carrier family 2 (facilitated glucose transporter), member 2 (SLC2A2), mRNA	NM_000340	Hs.167584	NM_000340
SLC2A3P1	Human glucose transporter pseudogene.	M55536	Hs.388400	BX648891
SLC2A5	solute carrier family 2 (facilitated glucose/fructose transporter), member 5 (SLC2A5), mRNA	NM_003039	Hs.530003	BC035878
SLC39A12	solute carrier family 39 (zinc transporter), member 12 (SLC39A12), mRNA	NM_152725	Hs.350895	BC035118
SLC44A5	Choline transporter-like protein 5 (Solute carrier family 44 member 5).	ENST00000370855	Unknown	
SLC6A17	solute carrier family 6, member 17 (SLC6A17), mRNA	NM_001010898	Hs.128382	NM_001010898
SLC6A2	solute carrier family 6 (neurotransmitter transporter, noradrenalin), member 2 (SLC6A2), mRNA	NM_001043	Hs.78036	NM_001043
SLC6A8	solute carrier family 6 (neurotransmitter transporter, creatine), member 8 (SLC6A8), mRNA	NM_005629	Hs.540696	NM_005629
SLC9A6	solute carrier family 9 (sodium/hydrogen exchanger), member 6 (SLC9A6), transcript variant 1, mRNA	NM_001042537	Hs.62185	NM_001042537
SLFNL1	cDNA FLJ23878 fis, clone LNG13675.	AK074458	Hs.194609	BC050353
SLITRK1	SLIT and NTRK-like family, member 1 (SLITRK1), mRNA	NM_052910	Hs.415478	NM_052910
SLITRK1	SLIT and NTRK-like family, member 1 (SLITRK1), mRNA	NM_052910	Hs.415478	NM_052910
SMAD6	SMAD family member 6 (SMAD6), mRNA	NM_005585	Hs.153863	NM_005585
SMG5	Smg-5 homolog, nonsense mediated mRNA decay factor (C. elegans) (SMG5), mRNA	NM_015327	Hs.516837	BC038296
SMYD3	SET and MYND domain containing 3 (SMYD3), mRNA	NM_022743	Hs.567571	AK023594
SNAP25	synaptosomal-associated protein, 25kDa (SNAP25), transcript variant 1, mRNA	NM_003081	Hs.167317	AK090857
SNIP	SNAP25-interacting protein (SNIP), mRNA	NM_025248	Hs.448872	NM_025248
SNX24	sorting nexing 24 (SNX24), mRNA	NM_014035	Hs.483200	NM_014035
SORBS2	clone HQ0618 PRO0618 mRNA, complete cds.	AF090937	Unknown	
SOST	sclerosteosis (SOST), mRNA	NM_025237	Hs.349204	AY358627
SPAG8	sperm associated antigen 8 (SPAG8), transcript variant 2, mRNA	NM_172312	Hs.256747	S83157
SPANXA1	sperm protein associated with the nucleus, X-linked, family member A1 (SPANXA1), mRNA	NM_013453	Hs.334464	BF212177
SPATA13	spermatogenesis associated 13 (SPATA13), mRNA	NM_153023	Hs.657121	BX648244
SPTLC3	Uncharacterized protein C20orf38.	ENST00000359573	Unknown	
SPTLC3	serine palmitoyltransferase, long chain base subunit 3 (SPTLC3), mRNA	NM_018327	Hs.425023	NM_018327
SPTLC3	serine palmitoyltransferase, long chain base subunit 3 (SPTLC3), mRNA	NM_018327	Hs.425023	NM_018327
SSU72	SSU72 RNA polymerase II CTD phosphatase homolog (S. cerevisiae) (SSU72), mRNA	NM_014188	Hs.657061	AK023110
SSX1	synovial sarcoma, X breakpoint 1 (SSX1), mRNA	NM_005635	Hs.434142	NM_005635
STK11IP	serine/threonine kinase 11 interacting protein (STK11IP), mRNA	NM_052902	Hs.22410	BC034051
STMN4	stathmin-like 4 (STMN4), mRNA	NM_030795	Hs.201058	NM_030795
STX6	cDNA FLJ32095 fis, clone OCBBF2000998.	AK056657	Unknown	
SUSD4	sushi domain containing 4 (SUSD4), transcript variant 1, mRNA	NM_017982	Hs.497841	AK096265
SUSD4	sushi domain containing 4 (SUSD4), transcript variant 2, mRNA	NM_001037175	Hs.497841	AK096265

SVOP	SV2 related protein homolog (rat) (SVOP), mRNA	NM_018711	Hs.4221	BC033587
SYF2	SYF2 homolog, RNA splicing factor (S. cerevisiae), mRNA (cDNA clone IMAGE:4042910), partial cds.	BC015824	Unknown	
SYP	synaptophysin (SYP), mRNA	NM_003179	Hs.632804	BC064550
SYT12	synaptotagmin XII (SYT12), mRNA	NM_177963	Hs.287636	AK024381
SYT16	synaptotagmin XVI (SYT16), mRNA	NM_031914	Hs.404139	AJ617628
SYT9	synaptotagmin IX (SYT9), mRNA	NM_175733	Hs.177193	BC046367
TAF11	TAF11 RNA polymerase II, TATA box binding protein (TBP)-associated factor, 28kDa (TAF11), mRNA	NM_005643	Hs.112444	BX647568
TAIP-2	TGF-beta induced apoptosis protein 2 (TAIP-2), mRNA	NM_024969	Hs.470479	AB063300
TAX1BP1	Tax1 (human T-cell leukemia virus type I) binding protein 1 (TAX1BP1), transcript variant 1, mRNA	NM_006024	Hs.34576	NM_006024
TEKT1	tektin 1 (TEKT1), mRNA	NM_053285	Hs.462108	AK094836
TEX11	testis expressed sequence 11 (TEX11), transcript variant 1, mRNA	NM_001003811	Hs.121776	NM_001003811
THBS3	thrombospondin 3 (THBS3), mRNA	NM_007112	Hs.169875	NM_007112
THC2496620	THC2496620	THC2496620	Unknown	
THC2515382	Q8SY50_DROME (Q8SY50) GH17483p (CG6361-PA), partial (5%)	THC2515382	Unknown	
THC2519054	Q5W0G2_HUMAN (Q5W0G2) Ankyrin repeat domain 18B, partial (11%)	THC2519054	Unknown	
THC2520461	Q455W3_9BURK (Q455W3) Aldehyde dehydrogenase, partial (4%)	THC2520461	Unknown	
THC2520858	ALU6_HUMAN (P39193) Alu subfamily SP sequence contamination warning entry, partial (12%)	THC2520858	Unknown	
THC2534144	Q25F20_MACFA (Q25F20) Brain cDNA, clone: QmoA-10668, partial (98%)	THC2534144	Unknown	
THC2539273	THC2539273	THC2539273	Unknown	
THC2540174	ALU6_HUMAN (P39193) Alu subfamily SP sequence contamination warning entry, partial (5%)	THC2540174	Unknown	
THC2544911	MUSEFTU elongation factor Tu {Mus musculus} (exp=-1; wgp=0; cg=0), partial (37%)	THC2544911	Unknown	
THC2550202	Q96HL9_HUMAN (Q96HL9) CHP protein, partial (59%)	THC2550202	Unknown	
THC2553512	THC2553512	THC2553512	Unknown	
THC2573284	THC2573284	THC2573284	Unknown	
THC2586959	1PK0_D Chain D, Crystal Structure Of The Ef3-Cam Complexed With Pmeapp. {Homo sapiens} (exp=-1; wgp=0; cg=0), partial (70%)	THC2586959	Unknown	
THC2608799	THC2608799	THC2608799	Unknown	
THC2611044	Q4THP9_TETNG (Q4THP9) Chromosome undetermined SCAF2771, whole genome shotgun sequence. (Fragment), partial (9%)	THC2611044	Unknown	
THC2622815	THC2622815	THC2622815	Unknown	
THC2626957	THC2626957	THC2626957	Unknown	
THC2634796	THC2634796	THC2634796	Unknown	
THC2641075	THC2641075	THC2641075	Unknown	
THC2643086	Q3IWS5_RHOS4 (Q3IWS5) Transcriptional regulator, GntR family, partial (5%)	THC2643086	Unknown	
THC2644853	Q9VWG7_DROME (Q9VWG7) CG14218-PA, partial (7%)	THC2644853	Unknown	
THC2651501	THC2651501	THC2651501	Unknown	
THC2654929	THC2654929	THC2654929	Unknown	
THC2655314	THC2655314	THC2655314	Unknown	
THC2656690	THC2656690	THC2656690	Unknown	
THC2657259	Q8J2S7_9EURO (Q8J2S7) DNA topoisomerase II (Fragment), partial (3%)	THC2657259	Unknown	
THC2657593	ALU1_HUMAN (P39188) Alu subfamily J sequence contamination warning entry, partial (7%)	THC2657593	Unknown	
THC2657613	Q5NT18_ENTHI (Q5NT18) Small GTPase EhRabM3 (Fragment), partial (7%)	THC2657613	Unknown	
THC2657938	Q77LW4_9NUCL (Q77LW4) Fgf, partial (5%)	THC2657938	Unknown	
THC2660448	THC2660448	THC2660448	Unknown	
THC2664171	THC2664171	THC2664171	Unknown	
THC2664792	ALU1_HUMAN (P39188) Alu subfamily J sequence contamination warning entry, partial (7%)	THC2664792	Unknown	
THC2667726	THC2667726	THC2667726	Unknown	
THC2672319	THC2672319	THC2672319	Unknown	
THC2672629	ALU7_HUMAN (P39194) Alu subfamily SQ sequence contamination warning entry, partial (3%)	THC2672629	Unknown	
THC2673265	Q6IHS4_DROME (Q6IHS4) HDC01261, partial (9%)	THC2673265	Unknown	
THC2674391	ALU8_HUMAN (P39195) Alu subfamily SX sequence contamination warning entry, partial (8%)	THC2674391	Unknown	
THC2675496	THC2675496	THC2675496	Unknown	
THC2681757	THC2681757	THC2681757	Unknown	

THC2681889	THC2681889	THC2681889	Unknown	
THC2683169	THC2683169	THC2683169	Unknown	
THC2685559	Q267U1_MYCVN (Q267U1) CDP-diacylglycerol--serine O-phosphatidyltransferase precursor , partial (5%)	THC2685559	Unknown	
THC2689572	THC2689572	THC2689572	Unknown	
THC2690338	Q9SL08_ARATH (Q9SL08) Expressed protein, partial (5%)	THC2690338	Unknown	
THC2693398	ALU1_HUMAN (P39188) Alu subfamily J sequence contamination warning entry, partial (11%)	THC2693398	Unknown	
THC2694828	Q3SDF2_PARTE (Q3SDF2) EPI21 protein, partial (5%)	THC2694828	Unknown	
THC2698311	HUMKIP2 p57KIP2 {Homo sapiens} (exp=-1; wgp=0; cg=0), partial (13%)	THC2698311	Unknown	
THC2713663	THC2713663	THC2713663	Unknown	
THC2714497	Q4T2S3_TETNG (Q4T2S3) Chromosome undetermined SCAF10199, whole genome shotgun sequence, partial (4%)	THC2714497	Unknown	
THC2715480	THC2715480	THC2715480	Unknown	
THC2718172	THC2718172	THC2718172	Unknown	
THC2719106	THC2719106	THC2719106	Unknown	
THC2720710	Q8IYG9_HUMAN (Q8IYG9) DPP6 protein, partial (23%)	THC2720710	Unknown	
THC2721496	Q4H139_SPDV (Q4H139) Non structural protein 3 (Fragment), partial (11%)	THC2721496	Unknown	
THC2722767	THC2722767	THC2722767	Unknown	
THC2723431	Q93V73_MAIZE (Q93V73) Globulin 1 (Fragment), partial (7%)	THC2723431	Unknown	
THC2723523	THC2723523	THC2723523	Unknown	
THC2730125	THC2730125	THC2730125	Unknown	
THC2744399	THC2744399	THC2744399	Unknown	
THC2770932	THC2770932	THC2770932	Unknown	
THC2779700	Q4RVR0_TETNG (Q4RVR0) Chromosome 9 SCAF14991, whole genome shotgun sequence, partial (8%)	THC2779700	Unknown	
TM9SF2	transmembrane 9 superfamily member 2 (TM9SF2), mRNA	NM_004800	Hs.654824	BC110656
TMCC2	transmembrane and coiled-coil domain family 2 (TMCC2), mRNA	NM_014858	Hs.6360	AY358192
TMCO1	transmembrane and coiled-coil domains 1 (TMCO1), mRNA	NM_019026	Hs.93832	CR614153
TMCO1	transmembrane and coiled-coil domains 1 (TMCO1), mRNA	NM_019026	Hs.93832	CR614153
TMED10	transmembrane emp24-like trafficking protein 10 (yeast) (TMED10), mRNA	NM_006827	Hs.74137	NM_006827
TMEM130	transmembrane protein 130 (TMEM130), mRNA	NM_152913	Hs.270753	AK124634
TMEM132A	transmembrane protein 132A (TMEM132A), transcript variant 1, mRNA	NM_017870	Hs.118552	NM_017870
TMEM2	transmembrane protein 2 (TMEM2), mRNA	NM_013390	Hs.494146	AF137030
TMEM50A	transmembrane protein 50A (TMEM50A), mRNA	NM_014313	Hs.523054	AY071927
TMEM59	transmembrane protein 59 (TMEM59), mRNA	NM_004872	Hs.523262	AK074636
TMEM81	transmembrane protein 81 (TMEM81), mRNA	NM_203376	Hs.146928	NM_203376
TMIE	transmembrane inner ear (TMIE), mRNA	NM_147196	Hs.185777	AY081842
TMPRSS6	transmembrane protease, serine 6 (TMPRSS6), mRNA	NM_153609	Hs.370885	NM_153609
TMPRSS6	transmembrane protease, serine 6 (TMPRSS6), mRNA	NM_153609	Hs.370885	NM_153609
TNC	tenascin C (hexabrachion) (TNC), mRNA	NM_002160	Hs.143250	NM_002160
TNRC4	trinucleotide repeat containing 4 (TNRC4), mRNA	NM_007185	Hs.26047	NM_007185
TOR1AIP2	Torsin-1A-interacting protein 2 (Lumenal domain-like LAP1).	ENST00000367612	Unknown	
TP53BP1	tumor protein p53 binding protein, 1 (TP53BP1), mRNA	NM_005657	Hs.440968	AF078776
TPM1	tropomyosin 1 (alpha) (TPM1), transcript variant 5, mRNA	NM_000366	Hs.133892	BX648171
TPM1	tropomyosin 1 (alpha) (TPM1), transcript variant 3, mRNA	NM_001018004	Hs.133892	BX648171
TPM1	tropomyosin 1 (alpha) (TPM1), transcript variant 3, mRNA	NM_001018004	Hs.133892	BX648171
TPM1	tropomyosin 1 (alpha) (TPM1), transcript variant 3, mRNA	NM_001018004	Hs.133892	BX648171
TRAM1	translocation associated membrane protein 1 (TRAM1), mRNA	NM_014294	Hs.491988	BC032018
TRIM11	tripartite motif-containing 11 (TRIM11), mRNA	NM_145214	Hs.13543	AK226119
TROVE2	TROVE domain family, member 2 (TROVE2), transcript variant 1, mRNA	NM_001042369	Hs.288178	CR936683
TRPA1	transient receptor potential cation channel, subfamily A, member 1 (TRPA1), mRNA	NM_007332	Hs.667156	Y10601
TRPM3	transient receptor potential cation channel, subfamily M, member 3 (TRPM3), transcript variant 7, mRNA	NM_206948	Hs.47288	NM_001007471
TRPM3	mRNA; cDNA DKFZp761A19121 (from clone DKFZp761A19121).	AL136545	Hs.47288	NM_001007471

TRPM3	transient receptor potential cation channel, subfamily M, member 3 (TRPM3), transcript variant 7, mRNA	NM_206948	Hs.47288	NM_001007471
TRPM3	transient receptor potential cation channel, subfamily M, member 3 (TRPM3), transcript variant 9, mRNA	NM_001007471	Hs.47288	NM_001007471
TSC22D1	TSC22 domain family, member 1 (TSC22D1), transcript variant 1, mRNA	NM_183422	Hs.507916	NM_183422
TSGA10	testis specific, 10 (TSGA10), mRNA	NM_025244	Hs.120267	AY014284
TSP50	testes-specific protease 50 (TSP50), mRNA	NM_013270	Hs.120365	AK092598
TSPAN13	tetraspanin 13 (TSPAN13), mRNA	NM_014399	Hs.364544	AK128509
TSPAN2	tetraspanin 2 (TSPAN2), mRNA	NM_005725	Hs.310458	NM_005725
TSPAN7	tetraspanin 7 (TSPAN7), mRNA	NM_004615	Hs.441664	AB062057
TTC3	tetratricopeptide repeat domain 3 (TTC3), transcript variant 1, mRNA	NM_003316	Hs.368214	D84294
TTC9B	cDNA FLJ30373 fis, clone BRACE2007882, weakly similar to Actin-depolymerizing protein N-WASP.	AK054935	Unknown	
TTLL7	Tubulin--tyrosine ligase-like protein 7 (Protein NYD-SP30).	ENST00000370703	Unknown	
TTLL7	tubulin tyrosine ligase-like family, member 7 (TTLL7), mRNA	NM_024686	Hs.445826	NM_024686
TTLL7	tubulin tyrosine ligase-like family, member 7 (TTLL7), mRNA	NM_024686	Hs.445826	NM_024686
TUBA1	tubulin, alpha 1 (TUBA1), mRNA	NM_006000	Hs.75318	AK054731
TUSC4	tumor suppressor candidate 4 (TUSC4), mRNA	NM_006545	Hs.437083	BC050412
TXNDC6	thioredoxin domain containing 6 (TXNDC6), mRNA	NM_178130	Hs.660992	NM_178130
TXNDC6	thioredoxin-like 2 mRNA, complete cds.	AF196568	Hs.660992	NM_178130
TYR	tyrosinase (oculocutaneous albinism IA) (TYR), mRNA	NM_000372	Hs.503555	M27160
TYR	tyrosinase (oculocutaneous albinism IA) (TYR), mRNA	NM_000372	Hs.503555	M27160
TYR	tyrosinase (oculocutaneous albinism IA) (TYR), mRNA	NM_000372	Hs.503555	M27160
U79301	Human clone 23842 mRNA sequence.	U79301	Hs.656184	U79301
UACA	uveal autoantigen with coiled-coil domains and ankyrin repeats (UACA), transcript variant 2, mRNA	NM_001008224	Hs.108049	NM_001008224
UBD	ubiquitin D (UBD), mRNA	NM_006398	Hs.44532	NM_006398
UBE2A	ubiquitin-conjugating enzyme E2A (RAD6 homolog) (UBE2A), transcript variant 1, mRNA	NM_003336	Hs.379466	BC042021
UBL3	ubiquitin-like 3 (UBL3), mRNA	NM_007106	Hs.145575	BC044582
UGT1A6	UDP glucuronosyltransferase 1 family, polypeptide A6 (UGT1A6), transcript variant 1, mRNA	NM_001072	Hs.654499	NM_001072
UGT1A6	UDP glucuronosyltransferase 1 family, polypeptide A6 (UGT1A6), transcript variant 1, mRNA	NM_001072	Hs.654499	NM_001072
UGT2B15	UDP glucuronosyltransferase 2 family, polypeptide B15 (UGT2B15), mRNA	NM_001076	Hs.700565	XM_001127829
UGT2B17	UDP glucuronosyltransferase 2 family, polypeptide B17 (UGT2B17), mRNA	NM_001077	Hs.575083	U59209
UGT2B4	UDP glucuronosyltransferase 2 family, polypeptide B4 (UGT2B4), mRNA	NM_021139	Hs.285887	BC026264
ULK1	unc-51-like kinase 1 (C. elegans) (ULK1), mRNA	NM_003565	Hs.47061	AF045458
ULK1	unc-51-like kinase 1 (C. elegans) (ULK1), mRNA	NM_003565	Hs.47061	AF045458
UNG2	uracil-DNA glycosylase 2 (UNG2), mRNA	NM_021147	Hs.3041	BC004877
UPK1B	uroplakin 1B (UPK1B), mRNA	NM_006952	Hs.271580	NM_006952
USH1C	cDNA: FLJ21290 fis, clone COL01954.	AK024943	Unknown	
USP20	ubiquitin specific peptidase 20 (USP20), transcript variant 1, mRNA	NM_001008563	Hs.5452	NM_006676
VPS45	vacuolar protein sorting 45 homolog (S. cerevisiae) (VPS45), mRNA	NM_007259	Hs.443750	NM_007259
WDR16	WD repeat domain 16 (WDR16), transcript variant 1, mRNA	NM_145054	Hs.232270	NM_153210
WDR26	WD repeat domain 26 (WDR26), mRNA	NM_025160	Hs.497873	AY221751
WDR44	WD repeat domain 44 (WDR44), mRNA	NM_019045	Hs.98510	NM_019045
WDR55	WD repeat domain 55, mRNA (cDNA clone IMAGE:3347441).	BC002482	Unknown	
WDR6	WD repeat domain 6 (WDR6), mRNA	NM_018031	Hs.654815	NM_018031
WDR68	WD repeat domain 68 (WDR68), mRNA	NM_005828	Hs.410596	NM_005828
WNT2B	wingless-type MMTV integration site family, member 2B (WNT2B), transcript variant WNT-2B1, mRNA	NM_004185	Hs.258575	AK127449
WNT4	wingless-type MMTV integration site family, member 4 (WNT4), mRNA	NM_030761	Hs.591521	AY358947
ZBTB40	Zinc finger and BTB domain-containing protein 40.	ENST00000374651	Unknown	
ZBTB41	zinc finger and BTB domain containing 41 (ZBTB41), mRNA	NM_194314	Hs.529439	NM_194314
ZBTB48	zinc finger and BTB domain containing 48 (ZBTB48), mRNA	NM_005341	Hs.502330	BX648395
ZCCHC12	zinc finger, CCHC domain containing 12 (ZCCHC12), mRNA	NM_173798	Hs.21417	BC036572
ZDHHC9	zinc finger, DHHC-type containing 9 (ZDHHC9), transcript variant 1, mRNA	NM_016032	Hs.193566	NM_016032

ZDHHC9	zinc finger, DHHC-type containing 9 (ZDHHC9), transcript variant 1, mRNA	NM_016032	Hs.193566	NM_016032
ZFYVE27	zinc finger, FYVE domain containing 27 (ZFYVE27), transcript variant 1, mRNA	NM_001002261	Hs.523194	BX647836
ZIC4	zinc family member 4 protein HZIC4 (ZIC4) mRNA, complete cds.	AF332509	Unknown	
ZMIZ2	zinc finger, MIZ-type containing 2 (ZMIZ2), transcript variant 1, mRNA	NM_031449	Hs.77978	NM_031449
ZMYND10	zinc finger, MYND-type containing 10 (ZMYND10), mRNA	NM_015896	Hs.526735	AK096525
ZNF264	zinc finger protein 264 (ZNF264), mRNA	NM_003417	Hs.515634	NM_003417
ZNF365	zinc finger protein 365 (ZNF365), transcript variant A, mRNA	NM_014951	Hs.22653	NM_014951
ZNF403	zinc finger protein 403 (ZNF403), mRNA	NM_024835	Hs.514116	AF268387
ZNF500	zinc finger protein 500 (ZNF500), mRNA	NM_021646	Hs.513316	AB011129
ZNF536	zinc finger protein 536 (ZNF536), mRNA	NM_014717	Hs.378901	BC150171
ZNF692	zinc finger protein 692 (ZNF692), mRNA	NM_017865	Hs.377705	AK126079
ZUBR1	zinc finger, UBR1 type 1 (ZUBR1), mRNA	NM_020765	Hs.148078	NM_020765
A_23_P140454	A_23_P140454	A_23_P140454	Unknown	
A_23_P156609	A_23_P156609	A_23_P156609	Unknown	
A_23_P165819	A_23_P165819	A_23_P165819	Unknown	
A_23_P369758	A_23_P369758	A_23_P369758	Unknown	
A_23_P46070	A_23_P46070	A_23_P46070	Unknown	
A_23_P61191	A_23_P61191	A_23_P61191	Unknown	
A_23_P95125	A_23_P95125	A_23_P95125	Unknown	
A_24_P144314	A_24_P144314	A_24_P144314	Unknown	
A_24_P323298	A_24_P323298	A_24_P323298	Unknown	
A_24_P33105	A_24_P33105	A_24_P33105	Unknown	
A_24_P464238	A_24_P464238	A_24_P464238	Unknown	
A_24_P545587	A_24_P545587	A_24_P545587	Unknown	
A_24_P633825	A_24_P633825	A_24_P633825	Unknown	
A_24_P669854	A_24_P669854	A_24_P669854	Unknown	
A_24_P713893	A_24_P713893	A_24_P713893	Unknown	
A_24_P725365	A_24_P725365	A_24_P725365	Unknown	
A_24_P790424	A_24_P790424	A_24_P790424	Unknown	
A_24_P926478	A_24_P926478	A_24_P926478	Unknown	
A_24_P931264	A_24_P931264	A_24_P931264	Unknown	
A_24_P936637	A_24_P936637	A_24_P936637	Unknown	
A_24_P943429	A_24_P943429	A_24_P943429	Unknown	
A_32_P100719	A_32_P100719	A_32_P100719	Unknown	
A_32_P103614	A_32_P103614	A_32_P103614	Unknown	
A_32_P116088	A_32_P116088	A_32_P116088	Unknown	
A_32_P134556	A_32_P134556	A_32_P134556	Unknown	
A_32_P160670	A_32_P160670	A_32_P160670	Unknown	
A_32_P173023	A_32_P173023	A_32_P173023	Unknown	
A_32_P180185	A_32_P180185	A_32_P180185	Unknown	
A_32_P220161	A_32_P220161	A_32_P220161	Unknown	
A_32_P23154	A_32_P23154	A_32_P23154	Unknown	
A_32_P235293	A_32_P235293	A_32_P235293	Unknown	
A_32_P31206	A_32_P31206	A_32_P31206	Unknown	
A_32_P35031	A_32_P35031	A_32_P35031	Unknown	
A_32_P40673	A_32_P40673	A_32_P40673	Unknown	
A_32_P47474	A_32_P47474	A_32_P47474	Unknown	
A_32_P52948	A_32_P52948	A_32_P52948	Unknown	
A_32_P55586	A_32_P55586	A_32_P55586	Unknown	
A_32_P599	A_32_P599	A_32_P599	Unknown	
A_32_P78285	A_32_P78285	A_32_P78285	Unknown	
A_32_P81696	A_32_P81696	A_32_P81696	Unknown	

A_32_P90615	A_32_P90615	A_32_P90615	Unknown	

Supplemental Table B4

K-Means Cluster 4 -- 1484 genes

Gene Name	Description	Acc	UGCluster	UGRepAcc
A2BP1	ataxin 2-binding protein 1 (A2BP1), transcript variant 3, mRNA	NM_145893	Hs.459842	BC026312
A2M	alpha-2-macroglobulin (A2M), mRNA	NM_000014	Hs.212838	CR749334
AA609065	af10g10.s1 Soares_testis_NHT cDNA clone IMAGE:1031298 3' similar to gb:M37721 PEPTIDYL-GLYCINE ALPHA-AMIDATING MONOOXYGENASE PRECURSOR (HUMAN);, mRNA sequence	AA609065	Hs.645723	AW966028
AA807805	nu88h02.s1 NCI_CGAP_Alv1 cDNA clone IMAGE:1217811, mRNA sequence	AA807805	Hs.574158	AA807805
AA972037	op88e01.s1 Soares_NFL_T_GBC_S1 cDNA clone IMAGE:1583928 3' similar to contains MER4.t1 MER4 repetitive element ;, mRNA sequence	AA972037	Hs.633513	AA972037
AARSD1	alanyl-tRNA synthetase domain containing 1 (AARSD1), mRNA	NM_025267	Hs.317403	CR619011
ABAT	4-aminobutyrate aminotransferase (ABAT), nuclear gene encoding mitochondrial protein, transcript variant 2, mRNA	NM_000663	Hs.336768	NM_000663
ABCA12	ATP-binding cassette, sub-family A (ABC1), member 12 (ABCA12), transcript variant 1, mRNA	NM_173076	Hs.134585	NM_173076
ABCC3	ATP-binding cassette, sub-family C (CFTR/MRP), member 3 (ABCC3), mRNA	NM_003786	Hs.463421	AF085692
ABLIM1	actin binding LIM protein 1 (ABLIM1), transcript variant 3, mRNA	NM_001003408	Hs.438236	NM_00100340[illegible]
ABLIM2	actin binding LIM protein family, member 2 (ABLIM2), mRNA	NM_032432	Hs.233404	AK130044
ABRA	actin-binding Rho activating protein (ABRA), mRNA	NM_139166	Hs.374668	AL832152
ACACB	acetyl-Coenzyme A carboxylase beta (ACACB), mRNA	NM_001093	Hs.234898	NM_001093
ACADL	acyl-Coenzyme A dehydrogenase, long chain (ACADL), nuclear gene encoding mitochondrial protein, mRNA	NM_001608	Hs.471277	BC039063
ACADVL	acyl-Coenzyme A dehydrogenase, very long chain (ACADVL), nuclear gene encoding mitochondrial protein, transcript variant 1, mRNA	NM_000018	Hs.437178	NM_000018
ACE2	angiotensin I converting enzyme (peptidyl-dipeptidase A) 2 (ACE2), mRNA	NM_021804	Hs.178098	AB193259
ACOT1	acyl-CoA thioesterase 1 (ACOT1), mRNA	NM_001037161	Hs.700643	BC006335
ACOT11	acyl-CoA thioesterase 11 (ACOT11), transcript variant 2, mRNA	NM_147161	Hs.234786	AB014607
ACOT2	acyl-CoA thioesterase 2 (ACOT2), mRNA	NM_006821	Hs.700643	BC006335
ACOT2	acyl-CoA thioesterase 2 (ACOT2), mRNA	NM_006821	Hs.700643	BC006335
ACOX2	acyl-Coenzyme A oxidase 2, branched chain (ACOX2), mRNA	NM_003500	Hs.444959	BC033517
ACTA2	actin, alpha 2, smooth muscle, aorta (ACTA2), mRNA	NM_001613	Hs.500483	BX647362
ACTC1	actin, alpha, cardiac muscle 1 (ACTC1), mRNA	NM_005159	Hs.696107	NM_005159
ACTC1	actin, alpha, cardiac muscle 1 (ACTC1), mRNA	NM_005159	Hs.696107	NM_005159
ACTG2	actin, gamma 2, smooth muscle, enteric (ACTG2), mRNA	NM_001615	Hs.516105	BF965163
ACTN2	actinin, alpha 2 (ACTN2), mRNA	NM_001103	Hs.498178	NM_001103
ACTRT1	actin-related protein T1 (ACTRT1), mRNA	NM_138289	Hs.680125	BC014597
ACYP2	H.sapiens mRNA for acylphosphatase, muscle type (MT) isoenzyme.	X84195	Unknown	
ACYP2	acylphosphatase 2, muscle type (ACYP2), mRNA	NM_138448	Hs.516173	BX647332
ADAM12	ADAM metallopeptidase domain 12 (meltrin alpha) (ADAM12), transcript variant 2, mRNA	NM_021641	Hs.655388	NM_003474
ADAM12	ADAM metallopeptidase domain 12 (meltrin alpha) (ADAM12), transcript variant 1, mRNA	NM_003474	Hs.655388	NM_003474
ADAM19	ADAM metallopeptidase domain 19 (meltrin beta) (ADAM19), transcript variant 2, mRNA	NM_033274	Hs.483944	Y13786
ADAMTS12	ADAM metallopeptidase with thrombospondin type 1 motif, 12 (ADAMTS12), mRNA	NM_030955	Hs.12680	AJ250725
ADAMTSL2	ADAMTS-like 2 (ADAMTSL2), mRNA	NM_014694	Hs.522543	AB011177
ADAMTSL3	ADAMTS-like 3 (ADAMTSL3), mRNA	NM_207517	Hs.459162	NM_207517
ADAMTSL3	ADAMTS-like 3 (ADAMTSL3), mRNA	NM_207517	Hs.459162	NM_207517
ADAMTSL3	ADAMTS-like 3 (ADAMTSL3), mRNA	NM_207517	Hs.459162	NM_207517
ADAMTSL5	ADAMTS-like 5 (ADAMTSL5), mRNA	NM_213604	Hs.371674	AK131571
ADCY5	adenylate cyclase 5 (ADCY5), mRNA	NM_183357	Hs.655144	AK124691
ADCY5	adenylate cyclase 5 (ADCY5), mRNA	NM_183357	Hs.655144	AK124691
ADCY6	adenylate cyclase 6 (ADCY6), transcript variant 1, mRNA	NM_015270	Hs.525401	NM_015270
ADD1	adducin 1 (alpha), mRNA (cDNA clone MGC:44427 IMAGE:5297337), complete cds.	BC042998	Hs.183706	CR936693
ADHFE1	alcohol dehydrogenase, iron containing, 1 (ADHFE1), transcript variant 2, mRNA	NM_144650	Hs.268869	BC028400
ADORA1	adenosine A1 receptor (ADORA1), transcript variant 1, mRNA	NM_000674	Hs.77867	AK127752
ADPRHL1	ADP-ribosylhydrolase like 1 (ADPRHL1), transcript variant 1, mRNA	NM_138430	Hs.98669	NM_199162
ADPRHL1	ADP-ribosylhydrolase like 1 (ADPRHL1), transcript variant 2, mRNA	NM_199162	Hs.98669	NM_199162
ADRB2	adrenergic, beta-2-, receptor, surface (ADRB2), mRNA	NM_000024	Hs.591251	M15169

ADRB2	adrenergic, beta-2-, receptor, surface (ADRB2), mRNA	NM_000024	Hs.591251	M15169
ADRB2	adrenergic, beta-2-, receptor, surface (ADRB2), mRNA	NM_000024	Hs.591251	M15169
ADRB2	adrenergic, beta-2-, receptor, surface (ADRB2), mRNA	NM_000024	Hs.591251	M15169
ADRB2	adrenergic, beta-2-, receptor, surface (ADRB2), mRNA	NM_000024	Hs.591251	M15169
ADRB2	adrenergic, beta-2-, receptor, surface (ADRB2), mRNA	NM_000024	Hs.591251	M15169
ADRB2	adrenergic, beta-2-, receptor, surface (ADRB2), mRNA	NM_000024	Hs.591251	M15169
ADRB2	adrenergic, beta-2-, receptor, surface (ADRB2), mRNA	NM_000024	Hs.591251	M15169
ADRB2	adrenergic, beta-2-, receptor, surface (ADRB2), mRNA	NM_000024	Hs.591251	M15169
ADRB2	adrenergic, beta-2-, receptor, surface (ADRB2), mRNA	NM_000024	Hs.591251	M15169
ADSSL1	adenylosuccinate synthase like 1 (ADSSL1), transcript variant 1, mRNA	NM_199165	Hs.592327	AK096124
AEBP1	AE binding protein 1 (AEBP1), mRNA	NM_001129	Hs.439463	NM_001129
AF086154	full length insert cDNA clone ZB55F04.	AF086154	Hs.142505	AF086154
AF088076	full length insert cDNA clone ZE01A04.	AF088076	Hs.445045	BG036341
AF090940	clone HQ0644 PRO0644 mRNA, complete cds.	AF090940	Unknown	
AF400500	clone Rt-16 SNURF-SNRPN mRNA, downstream untranslated exons, alternatively spliced.	AF400500	Hs.632166	AF400501
AFF4	AF4/FMR2 family, member 4 (AFF4), mRNA	NM_014423	Hs.519313	NM_014423
AI003989	AI003989 ou03b10.x1 Soares_total_fetus_Nb2HF8_9w cDNA clone IMAGE:1625179 3', mRNA sequence	AI003989	Hs.498586	CR627109
AI379175	AI379175 tc66c08.x1 Soares_NhHMPu_S1 cDNA clone IMAGE:2069582 3', mRNA sequence	AI379175	Hs.493819	AK091288
AI433842	AI433842 ti17b05.x1 NCI_CGAP_Kid11 cDNA clone IMAGE:2130705 3' similar to contains element MER6 repetitive element ;, mRNA sequence	AI433842	Hs.699517	BC056883
AI754733	cr29d06.x1 Human bone marrow stromal cells cDNA clone HBMSC_cr29d06 3', mRNA sequence	AI754733	Hs.645734	AL573456
AIFM2	apoptosis-inducing factor, mitochondrion-associated, 2 (AIFM2), mRNA	NM_032797	Hs.655377	NM_032797
AJ295982	mRNA for hypothetical protein (ORF1), clone Telethon(Italy_B41)_Strait14635_FL303.	AJ295982	Hs.668296	AJ295982
AJ318805	AJ318805 adipose tissue cDNA clone 2040, mRNA sequence	AJ318805	Hs.86538	AJ318805
AK000992	cDNA FLJ10130 fis, clone HEMBA1003035.	AK000992	Hs.658833	AK000992
AK001007	cDNA FLJ10145 fis, clone HEMBA1003322.	AK001007	Hs.138760	AK001007
AK001062	cDNA FLJ10200 fis, clone HEMBA1004863, highly similar to mRNA; cDNA DKFZp586M2022.	AK001062	Hs.522928	AL833529
AK001116	cDNA FLJ10254 fis, clone HEMBB1000848.	AK001116	Unknown	
AK001966	cDNA FLJ11104 fis, clone PLACE1005730.	AK001966	Hs.504540	AK023809
AK021463	cDNA FLJ11401 fis, clone HEMBA1000702.	AK021463	Hs.677034	AK021463
AK023338	cDNA FLJ13276 fis, clone OVARC1001040.	AK023338	Hs.654763	AL833040
AK023391	cDNA FLJ13329 fis, clone OVARC1001795.	AK023391	Hs.592775	AK125888
AK024455	mRNA for FLJ00047 protein, partial cds.	AK024455	Hs.677419	AK024455
AK026372	cDNA: FLJ22719 fis, clone HSI14307.	AK026372	Hs.634333	AK026372
AK026984	cDNA: FLJ23331 fis, clone HEP12664.	AK026984	Hs.33368	AK026984
AK054562	mRNA for FLJ00054 protein, partial cds.	AK054562	Hs.665624	AK054562
AK055214	cDNA FLJ30652 fis, clone DFNES2000011.	AK055214	Hs.592801	BX641108
AK055621	cDNA FLJ31059 fis, clone HSYRA2000832.	AK055621	Hs.583755	AK055621
AK055915	cDNA FLJ31353 fis, clone MESAN2000264.	AK055915	Hs.696089	AK055915
AK055981	cDNA FLJ31419 fis, clone NT2NE2000356.	AK055981	Hs.655249	AK055981
AK057935	cDNA FLJ25206 fis, clone REC05955.	AK057935	Unknown	
AK074635	cDNA FLJ90154 fis, clone HEMBB1002162, weakly similar to genethonin 1 mRNA.	AK074635	Hs.109590	CR627383
AK090499	cDNA FLJ33180 fis, clone ADRGL2003638.	AK090499	Hs.380030	AK095045
AK090499	cDNA FLJ33180 fis, clone ADRGL2003638.	AK090499	Hs.380030	AK095045
AK094088	cDNA FLJ36769 fis, clone ADIPS2000245.	AK094088	Hs.42572	CR627287
AK094860	cDNA FLJ37541 fis, clone BRCAN2026340.	AK094860	Hs.549913	AK094860
AK094995	cDNA FLJ37676 fis, clone BRHIP2012627.	AK094995	Hs.646953	AK094995
AK095108	cDNA FLJ37789 fis, clone BRHIP3000081.	AK095108	Hs.349283	AK095108
AK096020	cDNA FLJ38701 fis, clone KIDNE2002198.	AK096020	Hs.435845	NM_004452
AK123912	cDNA FLJ41918 fis, clone PERIC2002243.	AK123912	Hs.658808	BX648566
AK124390	cDNA FLJ42399 fis, clone ASTRO2003024.	AK124390	Hs.592775	AK125888
AK124778	cDNA FLJ42788 fis, clone BRAWH3007129.	AK124778	Hs.254117	AK124778

AK126415	cDNA FLJ44451 fis, clone UTERU2023039.	AK126415	Hs.496916	U66047
AK130038	cDNA FLJ26528 fis, clone KDN08653.	AK130038	Hs.254699	AK125803
AKAP13	A kinase (PRKA) anchor protein 13 (AKAP13), transcript variant 1, mRNA	NM_006738	Hs.459211	NM_006738
AKAP13	A kinase (PRKA) anchor protein 13 (AKAP13), transcript variant 1, mRNA	NM_006738	Hs.459211	NM_006738
AKAP6	A kinase (PRKA) anchor protein 6 (AKAP6), mRNA	NM_004274	Hs.509083	BC150288
AKR1B10	aldo-keto reductase family 1, member B10 (aldose reductase) (AKR1B10), mRNA	NM_020299	Hs.116724	BC072676
AKR1B10	aldo-keto reductase family 1, member B10 (aldose reductase) (AKR1B10), mRNA	NM_020299	Hs.116724	BC072676
AKR1C1	aldo-keto reductase family 1, member C1 (dihydrodiol dehydrogenase 1; 20-alpha (3-alpha)-hydroxysteroid dehydrogenase) (AKR1C1), mRNA	NM_001353	Hs.460260	AK226067
AKR1C1	aldo-keto reductase family 1, member C1 (dihydrodiol dehydrogenase 1; 20-alpha (3-alpha)-hydroxysteroid dehydrogenase) (AKR1C1), mRNA	NM_001353	Hs.460260	AK226067
AKR1C1	aldo-keto reductase family 1, member C1 (dihydrodiol dehydrogenase 1; 20-alpha (3-alpha)-hydroxysteroid dehydrogenase) (AKR1C1), mRNA	NM_001353	Hs.460260	AK226067
AL096727	mRNA; cDNA DKFZp434B104 (from clone DKFZp434B104).	AL096727	Hs.657260	BX648489
AL109695	mRNA full length insert cDNA clone EUROIMAGE 39820.	AL109695	Hs.311187	AF205074
AL110257	mRNA; cDNA DKFZp566P2346 (from clone DKFZp566P2346).	AL110257	Hs.531168	NM_152450
AL117454	mRNA; cDNA DKFZp586J1717 (from clone DKFZp586J1717).	AL117454	Hs.458733	AL117454
AL571926	AL571926 PLACENTA COT 25-NORMALIZED cDNA clone CS0DI029YJ06 3-PRIME, mRNA sequence	AL571926	Hs.699553	AL571926
AL574249	AL574249 PLACENTA COT 25-NORMALIZED cDNA clone CS0DI057YF21 3-PRIME, mRNA sequence	AL574249	Hs.644846	AL571948
AL833309	mRNA; cDNA DKFZp313A137 (from clone DKFZp313A137).	AL833309	Hs.622596	AL833309
ALAS2	aminolevulinate, delta-, synthase 2 (sideroblastic/hypochromic anemia) (ALAS2), nuclear gene encoding mitochondrial protein, transcript variant 1, mRNA	NM_000032	Hs.522666	BC030230
ALDOA	aldolase A, fructose-bisphosphate (ALDOA), transcript variant 1, mRNA	NM_000034	Hs.513490	AK098778
ALDOC	aldolase C, fructose-bisphosphate (ALDOC), mRNA	NM_005165	Hs.155247	NM_005165
ALS2CR11	amyotrophic lateral sclerosis 2 (juvenile) chromosome region, candidate 11 (ALS2CR11), mRNA	NM_152525	Hs.335788	BX648732
AMFR	autocrine motility factor receptor (AMFR), mRNA	NM_001144	Hs.295137	NM_001144
ANGPTL7	angiopoietin-like 7 (ANGPTL7), mRNA	NM_021146	Hs.146559	NM_021146
ANK1	ankyrin 1, erythrocytic (ANK1), transcript variant 3, mRNA	NM_000037	Hs.654438	NM_000037
ANK3	ankyrin 3, node of Ranvier (ankyrin G) (ANK3), transcript variant 2, mRNA	NM_001149	Hs.499725	NM_020987
ANK3	cDNA FLJ44903 fis, clone BRAMY3005184, highly similar to Mus musculus ankyrin 3, epithelial (Ank3).	AK126851	Hs.499725	NM_020987
ANKRA2	ankyrin repeat, family A (RFXANK-like), 2 (ANKRA2), mRNA	NM_023039	Hs.239154	AF251051
ANKRD25	ankyrin repeat domain 25 (ANKRD25), mRNA	NM_015493	Hs.284208	NM_015493
ANKRD29	ankyrin repeat domain 29 (ANKRD29), mRNA	NM_173505	Hs.374774	NM_173505
ANTXR1	anthrax toxin receptor 1 (ANTXR1), transcript variant 2, mRNA	NM_053034	Hs.165859	AF279145
ANTXR1	anthrax toxin receptor 1 (ANTXR1), transcript variant 2, mRNA	NM_053034	Hs.165859	AF279145
APOBEC2	apolipoprotein B mRNA editing enzyme, catalytic polypeptide-like 2 (APOBEC2), mRNA	NM_006789	Hs.555915	BC047767
AQP3	aquaporin 3 (Gill blood group) (AQP3), mRNA	NM_004925	Hs.234642	NM_004925
AQP3	aquaporin 3 (Gill blood group) (AQP3), mRNA	NM_004925	Hs.234642	NM_004925
AREG	amphiregulin (schwannoma-derived growth factor) (AREG), mRNA	NM_001657	Hs.270833	BC009799
ARHGAP1	Rho GTPase activating protein 1 (ARHGAP1), mRNA	NM_004308	Hs.138860	NM_004308
ARHGEF9	Cdc42 guanine nucleotide exchange factor (GEF) 9 (ARHGEF9), mRNA	NM_015185	Hs.54697	NM_015185
ARID5B	AT rich interactive domain 5B (MRF1-like) (ARID5B), mRNA	NM_032199	Hs.535297	NM_032199
ARL8A	ADP-ribosylation factor-like 8A (ARL8A), mRNA	NM_138795	Hs.497399	NM_138795
ARP11	mRNA for actin-related protein Arp11, complete cds.	AB039791	Hs.664579	BX640643
ASAH1	N-acylsphingosine amidohydrolase (acid ceramidase) 1 (ASAH1), transcript variant 1, mRNA	NM_177924	Hs.527412	NM_177924
ASB1	ankyrin repeat and SOCS box-containing 1 (ASB1), mRNA	NM_001040445	Hs.516788	NM_001040445
ASB2	ankyrin repeat and SOCS box-containing 2 (ASB2), mRNA	NM_016150	Hs.510327	NM_016150
ASPN	asporin (LRR class 1) (ASPN), mRNA	NM_017680	Hs.435655	BC063114
ATF3	activating transcription factor 3 (ATF3), transcript variant 4, mRNA	NM_001040619	Hs.460	NM_00104061[illegible]
ATP13A3	Probable cation-transporting ATPase 13A3 (EC 3.6.3.-) (ATPase family homolog up-regulated in senescence cells 1).	ENST00000256	Unknown	
ATP1A1	ATPase, Na+/K+ transporting, alpha 1 polypeptide (ATP1A1), transcript variant 1, mRNA	NM_000701	Hs.371889	NM_000701
ATP1B1	ATPase, Na+/K+ transporting, beta 1 polypeptide (ATP1B1), transcript variant 1, mRNA	NM_001677	Hs.291196	NM_001677
ATP2A2	ATPase, Ca++ transporting, cardiac muscle, slow twitch 2 (ATP2A2), transcript variant 2, mRNA	NM_001681	Hs.506759	BX648282
ATP2A2	ATPase, Ca++ transporting, cardiac muscle, slow twitch 2 (ATP2A2), transcript variant 2, mRNA	NM_001681	Hs.506759	BX648282

ATP2A2	ATPase, Ca++ transporting, cardiac muscle, slow twitch 2 (ATP2A2), transcript variant 2, mRNA	NM_001681	Hs.506759	BX648282
ATP5A1	ATP synthase, H+ transporting, mitochondrial F1 complex, alpha subunit 1, cardiac muscle (ATP5A1), nuclear gene encoding mitochondrial protein, transcript variant 1, mRNA	NM_001001937	Hs.298280	CR627212
ATP5B	ATP synthase, H+ transporting, mitochondrial F1 complex, beta polypeptide (ATP5B), nuclear gene encoding mitochondrial protein, mRNA	NM_001686	Hs.406510	BG177099
ATP5G3	ATP synthase, H+ transporting, mitochondrial F0 complex, subunit C3 (subunit 9) (ATP5G3), nuclear gene encoding mitochondrial protein, transcript variant 3, mRNA	NM_001002258	Hs.429	NM_00100225[illegible]
ATP5J	ATP synthase, H+ transporting, mitochondrial F0 complex, subunit F6 (ATP5J), nuclear gene encoding mitochondrial protein, transcript variant 1, mRNA	NM_001003703	Hs.246310	CF993935
ATP5L	ATP synthase, H+ transporting, mitochondrial F0 complex, subunit G (ATP5L), nuclear gene encoding mitochondrial protein, mRNA	NM_006476	Hs.486360	BF698866
ATP5O	ATP synthase, H+ transporting, mitochondrial F1 complex, O subunit (oligomycin sensitivity conferring protein) (ATP5O), nuclear gene encoding mitochondrial protein, mRNA	NM_001697	Hs.409140	BF965152
ATP6V1H	ATPase, H+ transporting, lysosomal 50/57kDa, V1 subunit H (ATP6V1H), transcript variant 1, mRNA	NM_015941	Hs.491737	NM_015941
ATPAF1	ATP synthase mitochondrial F1 complex assembly factor 1 (ATPAF1), nuclear gene encoding mitochondrial protein, transcript variant 1, mRNA	NM_022745	Hs.100874	AB007963
AV707343	AV707343 ADB cDNA clone ADBBSH02 5', mRNA sequence	AV707343	Hs.595279	AV707343
AVIL	advillin (AVIL), mRNA	NM_006576	Hs.584854	BX647344
AW444553	AW444553 UI-H-BI3-ajv-c-10-0-UI.s1 NCI_CGAP_Sub5 cDNA clone IMAGE:2732947 3', mRNA sequence	AW444553	Hs.124951	NM_174911
AW804939	AW804939 QV4-UM0094-060400-159-g07 UM0094 cDNA, mRNA sequence	AW804939	Hs.514535	BC015761
AW978428	AW978428 EST390537 MAGE resequences, MAGP cDNA, mRNA sequence	AW978428	Hs.533913	AK126583
AX781433	Sequence 16 from Patent EP1321519.	AX781433	Unknown	
AY358219	clone DNA188137 RRLF9220 (UNQ9220) mRNA, complete cds.	AY358219	Unknown	
B3GALT2	UDP-Gal:betaGlcNAc beta 1,3-galactosyltransferase, polypeptide 2 (B3GALT2), mRNA	NM_003783	Hs.518834	BC022507
B3GALT2	UDP-Gal:betaGlcNAc beta 1,3-galactosyltransferase, polypeptide 2 (B3GALT2), mRNA	NM_003783	Hs.518834	BC022507
B4GALNT3	cDNA FLJ16224 fis, clone CTONG3009287.	AK131277	Hs.504416	AK131277
B4GALT1	UDP-Gal:betaGlcNAc beta 1,4- galactosyltransferase, polypeptide 1 (B4GALT1), mRNA	NM_001497	Hs.651277	NM_001497
BAG2	BCL2-associated athanogene 2 (BAG2), mRNA	NM_004282	Hs.55220	AK023735
BAI2	brain-specific angiogenesis inhibitor 2 (BAI2), mRNA	NM_001703	Hs.524138	NM_001703
BAT3	HLA-B associated transcript 3 (BAT3), transcript variant 1, mRNA	NM_004639	Hs.440900	NM_004639
BC008341	cDNA clone IMAGE:3509098, **** WARNING: chimeric clone ****.	BC008341	Unknown	
BC010055	cDNA clone IMAGE:3845525, **** WARNING: chimeric clone ****.	BC010055	Unknown	
BC015977	Homo sapiens, clone IMAGE:4042121, mRNA, partial cds.	BC015977	Hs.350952	BF130157
BC018589	cDNA clone IMAGE:3863301.	BC018589	Unknown	
BC031250	cDNA clone IMAGE:5268928.	BC031250	Hs.647667	BC031250
BC031274	cDNA clone IMAGE:5294477.	BC031274	Hs.382116	BC047410
BC034233	cDNA clone IMAGE:5277859.	BC034233	Hs.279714	BC034233
BC034812	cDNA clone IMAGE:4825606.	BC034812	Hs.600720	BC034812
BC036424	cDNA clone IMAGE:4798730.	BC036424	Hs.530791	BC036424
BC038556	Homo sapiens, clone IMAGE:3446976, mRNA.	BC038556	Hs.369398	BC038556
BC040195	cDNA clone IMAGE:4816129.	BC040195	Hs.448989	BC040195
BC047230	cDNA clone IMAGE:4817555.	BC047230	Hs.660214	AK054983
BC063570	cDNA clone IMAGE:3865015, **** WARNING: chimeric clone ****.	BC063570	Unknown	
BC065285	cDNA clone IMAGE:6016511, **** WARNING: chimeric clone ****.	BC065285	Unknown	
BCAS1	breast carcinoma amplified sequence 1 (BCAS1), mRNA	NM_003657	Hs.400556	CR749643
BCL6	B-cell CLL/lymphoma 6 (zinc finger protein 51) (BCL6), transcript variant 2, mRNA	NM_138931	Hs.478588	BX649185
BE703462	BE703462 MR2-NN1111-080800-009-d10 NN1111 cDNA, mRNA sequence	BE703462	Hs.291196	NM_001677
BE710618	BE710618 QV3-HT0636-240500-204-e03 HT0636 cDNA, mRNA sequence	BE710618	Hs.450230	NM_00101339[illegible]
BE835321	BE835321 RC5-FN0022-300600-022-G12 FN0022 cDNA, mRNA sequence	BE835321	Hs.676515	BQ448273
BEST1	bestrophin 1 (BEST1), mRNA	NM_004183	Hs.693826	AB209451
BEST3	bestrophin 3 (BEST3), transcript variant 1, mRNA	NM_032735	Hs.280782	NM_032735
BG191465	RST10559 Athersys RAGE Library cDNA, mRNA sequence	BG191465	Hs.600586	BG191465
BG199525	BG199525 RST18816 Athersys RAGE Library cDNA, mRNA sequence	BG199525	Hs.581170	BG204150
BG216229	BG216229 RST35803 Athersys RAGE Library cDNA, mRNA sequence	BG216229	Hs.127535	BX647549
BHLHB3	basic helix-loop-helix domain containing, class B, 3 (BHLHB3), mRNA	NM_030762	Hs.177841	AB044088
BI024548	BI024548 PM3-MT0205-300101-005-f07 MT0205 cDNA, mRNA sequence	BI024548	Hs.352642	NM_021642
BI910665	603070430F1 NIH_MGC_118 cDNA clone IMAGE:5219455 5', mRNA sequence	BI910665	Hs.137007	BI910665

BICC1	cDNA: FLJ22476 fis, clone HRC10682.	AK026129	Hs.158745	BX538102
BLNK	B-cell linker (BLNK), mRNA	NM_013314	Hs.665244	AK225546
BLOC1S2	biogenesis of lysosome-related organelles complex-1, subunit 2 (BLOC1S2), transcript variant 2, mRNA	NM_001001342	Hs.696127	NM_00100134
BM982926	BM982926 UI-CF-EN1-acs-n-01-0-UI.s1 UI-CF-EN1 cDNA clone UI-CF-EN1-acs-n-01-0-UI 3', mRNA sequence	BM982926	Hs.187866	NM_012428
BQ019626	BQ019626 UI-H-ED0-axd-f-18-0-UI.s1 NCI_CGAP_ED0 cDNA clone IMAGE:5827217 3', mRNA sequence	BQ019626	Hs.504687	BM473095
BQ130147	BQ130147 ij85d08.x1 Human insulinoma cDNA clone IMAGE:5778111 3', mRNA sequence	BQ130147	Hs.656261	AK125406
BQ448507	UI-H-EU1-bag-p-08-0-UI.s1 NCI_CGAP_Ct1 cDNA clone UI-H-EU1-bag-p-08-0-UI 3', mRNA sequence	BQ448507	Hs.636120	BQ448507
BRP44L	brain protein 44-like (BRP44L), mRNA	NM_016098	Hs.172755	BF698899
BTC	betacellulin (BTC), mRNA	NM_001729	Hs.591704	BC011618
BTN1A1	butyrophilin, subfamily 1, member A1 (BTN1A1), mRNA	NM_001732	Hs.153058	NM_001732
BU561469	AGENCOURT_10278709 NIH_MGC_82 cDNA clone IMAGE:6592525 5', mRNA sequence	BU561469	Hs.209000	BU561469
BU617889	UI-H-DF0-bet-g-16-0-UI.s1 NCI_CGAP_DF0 cDNA clone UI-H-DF0-bet-g-16-0-UI 3', mRNA sequence	BU617889	Hs.602551	BG179248
BVES	blood vessel epicardial substance (BVES), transcript variant 5, mRNA	NM_147147	Hs.221660	BC040502
BVES	blood vessel epicardial substance (BVES), transcript variant 5, mRNA	NM_147147	Hs.221660	BC040502
BX101252	BX101252 NCI_CGAP_Lu24 cDNA clone IMAGp998I115625, mRNA sequence	BX101252	Hs.121525	BX101252
BX106493	BX106493 Soares_parathyroid_tumor_NbHPA cDNA clone IMAGp998H19725, mRNA sequence	BX106493	Hs.597557	BX106493
BX110903	BX110903 Soares_multiple_sclerosis_2NbHMSP cDNA clone IMAGp998K11615, mRNA sequence	BX110903	Hs.49132	BX110903
BX111600	BX111600 BX111600 Soares_placenta_8to9weeks_2NbHP8to9W cDNA clone IMAGp998D154357 ; IMAGE:1715294, mRNA sequence	BX111600	Hs.391527	CB853491
BX113452	BX113452 Soares infant brain 1NIB cDNA clone IMAGp998L21165, mRNA sequence	BX113452	Hs.594545	BX113452
BX114143	BX114143 Soares_pregnant_uterus_NbHPU cDNA clone IMAGp998G234345, mRNA sequence	BX114143	Hs.151274	BX114143
BX117479	BX117479 NCI_CGAP_Lu19 cDNA clone IMAGp998I075985, mRNA sequence	BX117479	Hs.10263	BX117479
BX427588	BX427588 FETAL LIVER cDNA clone CS0DM011YM01 3-PRIME, mRNA sequence	BX427588	Hs.669724	BX427588
BX444658	BX444658 BX444658 ADULT BRAIN cDNA clone CS0DN005YA23 3-PRIME, mRNA sequence	BX444658	Hs.408767	BC107897
BX647543	mRNA; cDNA DKFZp779F2345 (from clone DKFZp779F2345).	BX647543	Hs.444595	BX647543
BX648566	mRNA; cDNA DKFZp779F0655 (from clone DKFZp779F0655).	BX648566	Hs.658808	BX648566
BX955634	BX955634 DKFZp781L1872_r1 781 (synonym: hlcc4) cDNA clone DKFZp781L1872 5', mRNA sequence	BX955634	Hs.597082	AI492500
BZW2	basic leucine zipper and W2 domains 2 (BZW2), mRNA	NM_014038	Hs.487635	AK027837
C10orf56	chromosome 10 open reading frame 56 (C10orf56), mRNA	NM_153367	Hs.523080	BC028617
C11orf21	C11orf21 mRNA, complete cds.	AB029488	Hs.559181	AB029488
C12orf34	chromosome 12 open reading frame 34 (C12orf34), mRNA	NM_032829	Hs.661785	BC020193
C12orf62	chromosome 12 open reading frame 62 (C12orf62), mRNA	NM_032901	Hs.388645	BI116796
C14orf124	chromosome 14 open reading frame 124 (C14orf124), mRNA	NM_020195	Hs.643552	AF226050
C14orf128	chromosome 14 open reading frame 128, mRNA (cDNA clone MGC:15504 IMAGE:2990071), complete cds.	BC007251	Hs.496755	BC007251
C14orf140	chromosome 14 open reading frame 140 (C14orf140), transcript variant 1, mRNA	NM_024643	Hs.48642	NM_024643
C15orf48	chromosome 15 open reading frame 48 (C15orf48), transcript variant 2, mRNA	NM_032413	Hs.112242	BU175177
C16orf71	chromosome 16 open reading frame 71 (C16orf71), mRNA	NM_139170	Hs.602738	BX648126
C16orf76	chromosome 16 open reading frame 76 (C16orf76), mRNA	NM_152339	Hs.374556	NM_152339
C18orf1	chromosome 18 open reading frame 1 (C18orf1), transcript variant a2, mRNA	NM_181482	Hs.149363	NM_00100367
C18orf25	chromosome 18 open reading frame 25 (C18orf25), transcript variant 1, mRNA	NM_145055	Hs.696077	NM_145055
C19orf30	chromosome 19 open reading frame 30 (C19orf30), mRNA	NM_174947	Unknown	
C19orf36	chromosome 19 open reading frame 36 (C19orf36), transcript variant 1, mRNA	NM_001031735	Hs.424049	BC040964
C19orf55	chromosome 19 open reading frame 55 (C19orf55), mRNA	NM_001039887	Hs.527982	BC110893
C1orf105	chromosome 1 open reading frame 105 (C1orf105), mRNA	NM_139240	Hs.517991	NM_139240
C1orf167	mRNA; cDNA DKFZp434F1313 (from clone DKFZp434F1313).	AL834308	Hs.585415	XM_209234
C1orf175	chromosome 1 open reading frame 175 (C1orf175), mRNA	NM_001039464	Hs.657435	CR749830
C1orf88	chromosome 1 open reading frame 88 (C1orf88), mRNA	NM_181643	Hs.172510	BC050319
C1QTNF1	C1q and tumor necrosis factor related protein 1 (C1QTNF1), mRNA	NM_198594	Hs.201398	AY358424
C1QTNF2	C1q and tumor necrosis factor related protein 2 (C1QTNF2), mRNA	NM_031908	Hs.110062	NM_031908
C1S	complement component 1, s subcomponent (C1S), transcript variant 1, mRNA	NM_001734	Hs.458355	AK126711
C20orf102	chromosome 20 open reading frame 102 (C20orf102), mRNA	NM_080607	Hs.517029	NM_080607
C20orf121	chromosome 20 open reading frame 121 (C20orf121), transcript variant 1, mRNA	NM_024331	Hs.283869	NM_024331

C20orf141	chromosome 20 open reading frame 141 (C20orf141), mRNA	NM_080739	Hs.352187	BI548862
C20orf149	chromosome 20 open reading frame 149 (C20orf149), mRNA	NM_024299	Hs.79625	BG168849
C20orf166	chromosome 20 open reading frame 166 (C20orf166), mRNA	NM_178463	Hs.86507	NM_178463
C20orf30	chromosome 20 open reading frame 30 (C20orf30), transcript variant 2, mRNA	NM_001009924	Hs.472024	NM_00100992[illegible]
C20orf75	chromosome 20 open reading frame 75 (C20orf75), mRNA	NM_152611	Hs.149133	BC027720
C21orf37	BX089409 Soares_multiple_sclerosis_2NbHMSP cDNA clone IMAGp998A02613 ; IMAGE:277513, mRNA sequence	BX089409	Hs.46707	BX089409
C22orf16	chromosome 22 open reading frame 16 (C22orf16), mRNA	NM_213720	Hs.66915	BM913730
C3	complement component 3 (C3), mRNA	NM_000064	Hs.529053	BC063852
C3orf1	chromosome 3 open reading frame 1 (C3orf1), mRNA	NM_016589	Hs.477287	NM_016589
C3orf45	chromosome 3 open reading frame 45 (C3orf45), mRNA	NM_153215	Hs.534543	BC028000
C3orf58	chromosome 3 open reading frame 58 (C3orf58), mRNA	NM_173552	Hs.288954	NM_173552
C4B	complement component 4B (Childo blood group) (C4B), mRNA	NM_001002029	Hs.534847	NM_00100202[illegible]
C4orf22	chromosome 4 open reading frame 22 (C4orf22), mRNA	NM_152770	Hs.527104	AK128642
C4orf29	chromosome 4 open reading frame 29 (C4orf29), mRNA	NM_001039717	Hs.445817	BC075793
C4orf7	chromosome 4 open reading frame 7 (C4orf7), mRNA	NM_152997	Hs.320147	CB958028
C6orf112	mRNA; cDNA DKFZp434F2226 (from clone DKFZp434F2226).	AL137368	Hs.586271	AL137368
C6orf154	chromosome 6 open reading frame 154 (C6orf154), mRNA	NM_001012974	Hs.445552	NM_00101297[illegible]
C7	complement component 7 (C7), mRNA	NM_000587	Hs.78065	NM_000587
C8orf79	chromosome 8 open reading frame 79 (C8orf79), mRNA	NM_001039462	Unknown	
C9orf24	chromosome 9 open reading frame 24 (C9orf24), transcript variant 1, mRNA	NM_032596	Hs.50334	BM805429
C9orf7	chromosome 9 open reading frame 7 (C9orf7), mRNA	NM_017586	Hs.62003	AK074852
CA12	carbonic anhydrase XII (CA12), transcript variant 1, mRNA	NM_001218	Hs.210995	NM_001218
CA12	carbonic anhydrase XII (CA12), transcript variant 1, mRNA	NM_001218	Hs.210995	NM_001218
CA314451	CA314451 UI-CF-FN0-afh-a-09-0-UI.s1 UI-CF-FN0 cDNA clone UI-CF-FN0-afh-a-09-0-UI 3', mRNA sequence	CA314451	Hs.369574	NM_006449
CA840930	CA840930 ip30g05.x1 HR85 islet cDNA clone IMAGE:6219056 3', mRNA sequence	CA840930	Hs.75360	BX419477
CACNB2	calcium channel, voltage-dependent, beta 2 subunit (CACNB2), transcript variant 1, mRNA	NM_000724	Hs.699270	NM_000724
CAMK2B	calcium/calmodulin-dependent protein kinase (CaM kinase) II beta (CAMK2B), transcript variant 6, mRNA	NM_172082	Hs.351887	NM_001220
CAMK2D	calcium/calmodulin-dependent protein kinase (CaM kinase) II delta (CAMK2D), transcript variant 1, mRNA	NM_172127	Hs.144114	BX647363
CANX	calnexin (CANX), transcript variant 1, mRNA	NM_001746	Hs.699155	NM_001746
CAP2	CAP, adenylate cyclase-associated protein, 2 (yeast) (CAP2), mRNA	NM_006366	Hs.132902	NM_006366
CAPS	calcyphosine (CAPS), transcript variant 1, mRNA	NM_004058	Hs.584744	AK090469
CARD9	caspase recruitment domain family, member 9 (CARD9), mRNA	NM_052813	Hs.694071	AK024001
CASQ1	calsequestrin 1 (fast-twitch, skeletal muscle) (CASQ1), nuclear gene encoding mitochondrial protein, mRNA	NM_001231	Hs.632476	NM_001231
CB240572	CB240572 UI-CF-FN0-afv-f-02-0-UI.s1 UI-CF-FN0 cDNA clone UI-CF-FN0-afv-f-02-0-UI 3', mRNA sequence	CB240572	Hs.460109	NM_00104011[illegible]
CBLN2	cerebellin 2 precursor (CBLN2), mRNA	NM_182511	Hs.569851	AK125422
CBX7	chromobox homolog 7 (CBX7), mRNA	NM_175709	Hs.356416	NM_175709
CBX7	chromobox homolog 7 (CBX7), mRNA	NM_175709	Hs.356416	NM_175709
CCDC80	coiled-coil domain containing 80 (CCDC80), transcript variant 1, mRNA	NM_199511	Hs.477128	NM_199511
CCDC81	coiled-coil domain containing 81 (CCDC81), mRNA	NM_021827	Hs.144913	BC126412
CCK	cholecystokinin (CCK), mRNA	NM_000729	Hs.458426	BC111026
CCL20	chemokine (C-C motif) ligand 20 (CCL20), mRNA	NM_004591	Hs.75498	BC020698
CCNDBP1	cyclin D-type binding-protein 1 (CCNDBP1), transcript variant 2, mRNA	NM_037370	Hs.36794	CR614852
CD4	CD4 molecule (CD4), mRNA	NM_000616	Hs.631659	S79267
CDC37L1	cell division cycle 37 homolog (S. cerevisiae)-like 1 (CDC37L1), mRNA	NM_017913	Hs.561954	CR598577
CDC42EP3	CDC42 effector protein (Rho GTPase binding) 3 (CDC42EP3), mRNA	NM_006449	Hs.369574	NM_006449
CDC42SE1	CDC42 small effector 1 (CDC42SE1), transcript variant 1, mRNA	NM_001038707	Hs.22065	NM_00103870[illegible]
CDH16	cadherin 16, KSP-cadherin (CDH16), mRNA	NM_004062	Hs.513660	AK075243
CDH2	cadherin 2, type 1, N-cadherin (neuronal) (CDH2), mRNA	NM_001792	Hs.464829	S42303
CDH8	cadherin 8, type 2 (CDH8), mRNA	NM_001796	Hs.368322	AK124734
CEBPB	CCAAT/enhancer binding protein (C/EBP), beta (CEBPB), mRNA	NM_005194	Hs.693759	BC021931
CEBPB	CCAAT/enhancer binding protein (C/EBP), beta (CEBPB), mRNA	NM_005194	Hs.693759	BC021931

CEBPB	CCAAT/enhancer binding protein (C/EBP), beta (CEBPB), mRNA	NM_005194	Hs.693759	BC021931
CEBPB	CCAAT/enhancer binding protein (C/EBP), beta (CEBPB), mRNA	NM_005194	Hs.693759	BC021931
CEBPB	CCAAT/enhancer binding protein (C/EBP), beta (CEBPB), mRNA	NM_005194	Hs.693759	BC021931
CEBPB	CCAAT/enhancer binding protein (C/EBP), beta (CEBPB), mRNA	NM_005194	Hs.693759	BC021931
CEBPB	CCAAT/enhancer binding protein (C/EBP), beta (CEBPB), mRNA	NM_005194	Hs.693759	BC021931
CEBPB	CCAAT/enhancer binding protein (C/EBP), beta (CEBPB), mRNA	NM_005194	Hs.693759	BC021931
CEBPB	CCAAT/enhancer binding protein (C/EBP), beta (CEBPB), mRNA	NM_005194	Hs.693759	BC021931
CEBPB	CCAAT/enhancer binding protein (C/EBP), beta (CEBPB), mRNA	NM_005194	Hs.693759	BC021931
CEBPD	CCAAT/enhancer binding protein (C/EBP), delta (CEBPD), mRNA	NM_005195	Hs.440829	BM924801
CENTA2	centaurin, alpha 2 (CENTA2), mRNA	NM_018404	Hs.514063	NM_018404
CENTB1	cDNA FLJ44545 fis, clone UTERU3005970.	AK126509	Hs.337242	AK126509
CES1	carboxylesterase 1 (monocyte/macrophage serine esterase 1) (CES1), transcript variant 3, mRNA	NM_001266	Hs.558865	NM_001025195
CFB	complement factor B (CFB), mRNA	NM_001710	Hs.69771	NM_001710
CHI3L1	chitinase 3-like 1 (cartilage glycoprotein-39) (CHI3L1), mRNA	NM_001276	Hs.382202	NM_001276
CHMP4C	chromatin modifying protein 4C (CHMP4C), mRNA	NM_152284	Hs.183861	NM_152284
CHPF	chondroitin polymerizing factor (CHPF), mRNA	NM_024536	Hs.516711	AB095813
CHRDL2	chordin-like 2 (CHRDL2), mRNA	NM_015424	Hs.432379	BC142623
CHRDL2	chordin-like 2 (CHRDL2), mRNA	NM_015424	Hs.432379	BC142623
CHRM2	cholinergic receptor, muscarinic 2 (CHRM2), transcript variant 2, mRNA	NM_001006627	Hs.535891	AL832585
CHRM4	cholinergic receptor, muscarinic 4 (CHRM4), mRNA	NM_000741	Hs.248100	AF385590
CHST3	carbohydrate (chondroitin 6) sulfotransferase 3 (CHST3), mRNA	NM_004273	Hs.158304	AB017915
CITED2	Cbp/p300-interacting transactivator, with Glu/Asp-rich carboxy-terminal domain, 2 (CITED2), mRNA	NM_006079	Hs.82071	NM_006079
CKM	creatine kinase, muscle (CKM), mRNA	NM_001824	Hs.334347	NM_001824
CKMT2	creatine kinase, mitochondrial 2 (sarcomeric) (CKMT2), nuclear gene encoding mitochondrial protein, mRNA	NM_001825	Hs.80691	NM_001825
CLCN3	chloride channel 3 (CLCN3), transcript variant e, mRNA	NM_173872	Hs.481186	NM_173872
CLCNKB	chloride channel Kb (CLCNKB), mRNA	NM_000085	Hs.352243	NM_000085
CLDN1	claudin 1 (CLDN1), mRNA	NM_021101	Hs.439060	NM_021101
CLDN1	claudin 1 (CLDN1), mRNA	NM_021101	Hs.439060	NM_021101
CLEC4C	C-type lectin domain family 4, member C (CLEC4C), transcript variant 1, mRNA	NM_130441	Hs.351812	AK122596
CLIC5	chloride intracellular channel 5 (CLIC5), mRNA	NM_016929	Hs.485489	DQ679794
CLIC5	chloride intracellular channel 5 (CLIC5), mRNA	NM_016929	Hs.485489	DQ679794
CLSTN2	calsyntenin 2 (CLSTN2), mRNA	NM_022131	Hs.158529	AJ278018
CLTB	clathrin, light chain (Lcb) (CLTB), transcript variant 2, mRNA	NM_007097	Hs.484241	BM908253
CMYA1	cardiomyopathy associated 1 (CMYA1), mRNA	NM_194293	Hs.447868	AJ626900
CMYA3	cardiomyopathy associated 3 (CMYA3), transcript variant 1, mRNA	NM_152381	Hs.73680	NM_152381
CMYA5	cardiomyopathy associated 5 (CMYA5), mRNA	NM_153610	Hs.482625	NM_153610
CMYA5	cardiomyopathy associated 5 (CMYA5), mRNA	NM_153610	Hs.482625	NM_153610
CNN1	calponin 1, basic, smooth muscle (CNN1), mRNA	NM_001299	Hs.465929	BC036307
COL14A1	collagen, type XIV, alpha 1 (undulin) (COL14A1), mRNA	NM_021110	Hs.409662	NM_021110
COL15A1	collagen, type XV, alpha 1 (COL15A1), mRNA	NM_001855	Hs.409034	NM_001855
COL15A1	collagen, type XV, alpha 1 (COL15A1), mRNA	NM_001855	Hs.409034	NM_001855
COL16A1	collagen, type XVI, alpha 1 (COL16A1), mRNA	NM_001856	Hs.368921	NM_001856
COL1A1	H.sapiens mRNA for prepro-alpha1(I) collagen.	Z74615	Hs.172928	Z74615
COL1A1	H.sapiens mRNA for prepro-alpha1(I) collagen.	Z74615	Hs.172928	Z74615
COL1A1	H.sapiens mRNA for prepro-alpha1(I) collagen.	Z74615	Hs.172928	Z74615
COL1A1	H.sapiens mRNA for prepro-alpha1(I) collagen.	Z74615	Hs.172928	Z74615
COL1A1	H.sapiens mRNA for prepro-alpha1(I) collagen.	Z74615	Hs.172928	Z74615
COL1A1	H.sapiens mRNA for prepro-alpha1(I) collagen.	Z74615	Hs.172928	Z74615
COL1A1	H.sapiens mRNA for prepro-alpha1(I) collagen.	Z74615	Hs.172928	Z74615
COL1A1	H.sapiens mRNA for prepro-alpha1(I) collagen.	Z74615	Hs.172928	Z74615
COL1A1	H.sapiens mRNA for prepro-alpha1(I) collagen.	Z74615	Hs.172928	Z74615
COL1A1	H.sapiens mRNA for prepro-alpha1(I) collagen.	Z74615	Hs.172928	Z74615

COL1A2	collagen, type I, alpha 2 (COL1A2), mRNA	NM_000089	Hs.489142	J03464
COL1A2	collagen, type I, alpha 2 (COL1A2), mRNA	NM_000089	Hs.489142	J03464
COL1A2	collagen, type I, alpha 2 (COL1A2), mRNA	NM_000089	Hs.489142	J03464
COL1A2	collagen, type I, alpha 2 (COL1A2), mRNA	NM_000089	Hs.489142	J03464
COL1A2	collagen, type I, alpha 2 (COL1A2), mRNA	NM_000089	Hs.489142	J03464
COL1A2	collagen, type I, alpha 2 (COL1A2), mRNA	NM_000089	Hs.489142	J03464
COL1A2	collagen, type I, alpha 2 (COL1A2), mRNA	NM_000089	Hs.489142	J03464
COL1A2	collagen, type I, alpha 2 (COL1A2), mRNA	NM_000089	Hs.489142	J03464
COL1A2	collagen, type I, alpha 2 (COL1A2), mRNA	NM_000089	Hs.489142	J03464
COL1A2	collagen, type I, alpha 2 (COL1A2), mRNA	NM_000089	Hs.489142	J03464
COL1A2	collagen, type I, alpha 2 (COL1A2), mRNA	NM_000089	Hs.489142	J03464
COL3A1	collagen, type III, alpha 1 (Ehlers-Danlos syndrome type IV, autosomal dominant) (COL3A1), mRNA	NM_000090	Hs.443625	NM_000090
COL4A1	collagen, type IV, alpha 1 (COL4A1), mRNA	NM_001845	Hs.17441	NM_001845
COL4A2	collagen, type IV, alpha 2 (COL4A2), mRNA	NM_001846	Hs.508716	NM_001846
COL6A2	collagen, type VI, alpha 2 (COL6A2), transcript variant 2C2, mRNA	NM_001849	Hs.420269	AK128695
COL6A2	collagen, type VI, alpha 2 (COL6A2), transcript variant 2C2a', mRNA	NM_058175	Hs.420269	AK128695
COL7A1	collagen, type VII, alpha 1 (epidermolysis bullosa, dystrophic, dominant and recessive) (COL7A1), mRNA	NM_000094	Hs.476218	L02870
COLQ	collagen-like tail subunit (single strand of homotrimer) of asymmetric acetylcholinesterase (COLQ), transcript variant II, mRNA	NM_080538	Hs.146735	NM_080538
COMP	cartilage oligomeric matrix protein (COMP), mRNA	NM_000095	Hs.1584	BC033676
COQ9	cDNA PSEC0129 fis, clone PLACE1004170.	AK075438	Hs.513632	AK075438
CORIN	corin, serine peptidase (CORIN), mRNA	NM_006587	Hs.518618	BX648285
CORO6	coronin 6 (CORO6), mRNA	NM_032854	Hs.143046	AK094683
COX5B	cytochrome c oxidase subunit Vb (COX5B), mRNA	NM_001862	Hs.1342	BM912880
COX6A1	cytochrome c oxidase subunit VIa polypeptide 1 (COX6A1), nuclear gene encoding mitochondrial protein, mRNA	NM_004373	Hs.497118	BQ433343
COX6B1	cytochrome c oxidase subunit Vib polypeptide 1 (ubiquitous) (COX6B1), mRNA	NM_001863	Hs.431668	CD517959
COX6C	cytochrome c oxidase subunit VIc (COX6C), mRNA	NM_004374	Hs.351875	AK128382
COX7A2	cytochrome c oxidase subunit VIIa polypeptide 2 (liver) (COX7A2), mRNA	NM_001865	Hs.70312	BQ214619
COX7B	cytochrome c oxidase subunit VIIb (COX7B), nuclear gene encoding mitochondrial protein, mRNA	NM_001866	Hs.522699	BG179412
COX8A	cytochrome c oxidase subunit 8A (ubiquitous) (COX8A), mRNA	NM_004074	Hs.433901	CD555987
CPA4	carboxypeptidase A4 (CPA4), mRNA	NM_016352	Hs.93764	NM_016352
CPA4	carboxypeptidase A4 (CPA4), mRNA	NM_016352	Hs.93764	NM_016352
CPEB4	cytoplasmic polyadenylation element binding protein 4 (CPEB4), mRNA	NM_030627	Hs.127126	BX538213
CPEB4	cytoplasmic polyadenylation element binding protein 4 (CPEB4), mRNA	NM_030627	Hs.127126	BX538213
CR593784	full-length cDNA clone CS0DI036YE11 of Placenta Cot 25-normalized of (human).	CR593784	Hs.304253	CR593784
CR595668	full-length cDNA clone CS0DB003YC18 of Neuroblastoma Cot 10-normalized of (human).	CR595668	Hs.42239	XM_927661
CR610759	full-length cDNA clone CS0DI026YO05 of Placenta Cot 25-normalized of (human).	CR610759	Hs.598335	CR610759
CR613736	full-length cDNA clone CS0DI072YA21 of Placenta Cot 25-normalized of (human).	CR613736	Hs.596312	XM_00112890[illegible]
CR618217	full-length cDNA clone CS0DC014YA07 of Neuroblastoma Cot 25-normalized of (human).	CR618217	Hs.136313	AK123792
CR618615	full-length cDNA clone CL0BB018ZH05 of Neuroblastoma of (human).	CR618615	Hs.31917	BX647106
CR618615	full-length cDNA clone CL0BB018ZH05 of Neuroblastoma of (human).	CR618615	Hs.31917	BX647106
CR621698	full-length cDNA clone CS0DF030YF21 of Fetal brain of (human).	CR621698	Hs.613981	CR621698
CR740121	CR740121 library (Ebert L) cDNA clone IMAGp971G1750 ; IMAGE:767753 5', mRNA sequence	CR740121	Hs.85989	CR740121
CR936711	mRNA; cDNA DKFZp781P02163 (from clone DKFZp781P02163).	CR936711	Hs.602301	CR936711
CREG1	cellular repressor of E1A-stimulated genes 1 (CREG1), mRNA	NM_003851	Hs.5710	NM_003851
CRY2	cryptochrome 2 (photolyase-like) (CRY2), mRNA	NM_021117	Hs.532491	BC035161
CRYAB	crystallin, alpha B (CRYAB), mRNA	NM_001885	Hs.408767	BC107897
CRYBA2	crystallin, beta A2 (CRYBA2), transcript variant 1, mRNA	NM_005209	Hs.415790	BM910348
CSDC2	cold shock domain containing C2, RNA binding (CSDC2), mRNA	NM_014460	Hs.310893	NM_014460
CSF1R	colony stimulating factor 1 receptor, formerly McDonough feline sarcoma viral (v-fms) oncogene homolog (CSF1R), mRNA	NM_005211	Hs.654394	X03663
CSMD1	CUB and Sushi multiple domains 1 (CSMD1), mRNA	NM_033225	Hs.571466	AB209502
CSPG4	chondroitin sulfate proteoglycan 4 (melanoma-associated) (CSPG4), mRNA	NM_001897	Hs.513044	NM_001897

CSRP3	cysteine and glycine-rich protein 3 (cardiac LIM protein) (CSRP3), mRNA	NM_003476	Hs.83577	NM_003476
CST6	cystatin E/M (CST6), mRNA	NM_001323	Hs.139389	AK092391
CTNNA3	catenin (cadherin-associated protein), alpha 3, mRNA (cDNA clone IMAGE:4823848), complete cds.	BC022004	Hs.660362	NM_013266
CTNNA3	catenin (cadherin-associated protein), alpha 3 (CTNNA3), mRNA	NM_013266	Hs.660362	NM_013266
CXCL10	chemokine (C-X-C motif) ligand 10 (CXCL10), mRNA	NM_001565	Hs.632586	BC010954
CYFIP2	cytoplasmic FMR1 interacting protein 2 (CYFIP2), transcript variant 2, mRNA	NM_001037332	Hs.519702	NM_00103733
CYP17A1	cytochrome P450, family 17, subfamily A, polypeptide 1 (CYP17A1), mRNA	NM_000102	Hs.438016	AK094106
CYP1B1	cytochrome P450, family 1, subfamily B, polypeptide 1 (CYP1B1), mRNA	NM_000104	Hs.154654	NM_000104
CYP21A2	cytochrome P450, family 21, subfamily A, polypeptide 2 (CYP21A2), mRNA	NM_000500	Hs.654479	CD013987
CYP2J2	cytochrome P450, family 2, subfamily J, polypeptide 2 (CYP2J2), mRNA	NM_000775	Hs.152096	AK055088
CYP4V2	cytochrome P450, family 4, subfamily V, polypeptide 2 (CYP4V2), mRNA	NM_207352	Hs.237642	BX648730
CYP7B1	cytochrome P450, family 7, subfamily B, polypeptide 1 (CYP7B1), mRNA	NM_004820	Hs.667720	NM_004820
DAAM1	dishevelled associated activator of morphogenesis 1 (DAAM1), mRNA	NM_014992	Hs.654934	NM_014992
DAAM1	dishevelled associated activator of morphogenesis 1 (DAAM1), mRNA	NM_014992	Hs.654934	NM_014992
DACT3	dapper, antagonist of beta-catenin, homolog 3 (Xenopus laevis) (DACT3), mRNA	NM_145056	Hs.515490	BC034052
DAND5	DAN domain family, member 5 (DAND5), mRNA	NM_152654	Hs.331981	BC025333
DB325746	DB325746 OCBBF2 cDNA clone OCBBF2004259 3', mRNA sequence	DB325746	Hs.701447	DB325746
DCAKD	dephospho-CoA kinase domain containing (DCAKD), mRNA	NM_024819	Hs.463148	BC006472
DCBLD2	discoidin, CUB and LCCL domain containing 2 (DCBLD2), mRNA	NM_080927	Hs.203691	NM_080927
DDAH1	dimethylarginine dimethylaminohydrolase 1 (DDAH1), mRNA	NM_012137	Hs.379858	NM_012137
DDIT3	DNA-damage-inducible transcript 3 (DDIT3), mRNA	NM_004083	Hs.505777	BC107859
DDR1	discoidin domain receptor family, member 1 (DDR1), transcript variant 1, mRNA	NM_013993	Hs.631988	NM_013993
DDT	D-dopachrome tautomerase (DDT), mRNA	NM_001355	Hs.656723	AK126983
DENND2C	DENN/MADD domain containing 2C (DENND2C), mRNA	NM_198459	Hs.654928	CR749576
DENND2D	DENN/MADD domain containing 2D (DENND2D), mRNA	NM_024901	Hs.557850	AL713773
DES	desmin (DES), mRNA	NM_001927	Hs.594952	AK022087
DGAT1	diacylglycerol O-acyltransferase homolog 1 (mouse) (DGAT1), mRNA	NM_012079	Hs.613075	NM_012079
DGKD	diacylglycerol kinase, delta 130kDa (DGKD), transcript variant 2, mRNA	NM_152879	Hs.471675	NM_152879
DGKI	diacylglycerol kinase, iota (DGKI), mRNA	NM_004717	Hs.242947	AB209167
DHRS2	dehydrogenase/reductase (SDR family) member 2 (DHRS2), transcript variant 1, mRNA	NM_182908	Hs.272499	AB209653
DHRS2	dehydrogenase/reductase (SDR family) member 2 (DHRS2), transcript variant 1, mRNA	NM_182908	Hs.272499	AB209653
DHRS7C	PREDICTED: dehydrogenase	ENST00000330	Unknown	
DHRS9	dehydrogenase/reductase (SDR family) member 9 (DHRS9), transcript variant 1, mRNA	NM_005771	Hs.179608	NM_005771
DIABLO	diablo homolog (Drosophila) (DIABLO), nuclear gene encoding mitochondrial protein, transcript variant 1, mRNA	NM_019887	Hs.169611	AK001399
DIP2C	DIP2 disco-interacting protein 2 homolog C (Drosophila) (DIP2C), mRNA	NM_014974	Hs.432397	AB023151
DISP2	dispatched homolog 2 (Drosophila) (DISP2), mRNA	NM_033510	Hs.355645	AB051529
DKFZP434P21	POM121-like protein, mRNA (cDNA clone IMAGE:40053742).	BC112340	Hs.645245	BC112340
DKFZP586H21	regeneration associated muscle protease (DKFZP586H2123), transcript variant 1, mRNA	NM_015430	Hs.55044	BX640676
DLD	dihydrolipoamide dehydrogenase (DLD), mRNA	NM_000108	Hs.131711	NM_000108
DMN	desmuslin (DMN), transcript variant A, mRNA	NM_145728	Hs.207106	BC151243
DMPK	dystrophia myotonica-protein kinase (DMPK), mRNA	NM_004409	Hs.631596	AB209363
DNAJB1	DnaJ (Hsp40) homolog, subfamily B, member 1 (DNAJB1), mRNA	NM_006145	Hs.515210	BC002352
DNAJB2	DnaJ (Hsp40) homolog, subfamily B, member 2 (DNAJB2), transcript variant 2, mRNA	NM_006736	Hs.77768	NM_006736
DNAJB9	DnaJ (Hsp40) homolog, subfamily B, member 9 (DNAJB9), mRNA	NM_012328	Hs.6790	AK092204
DOK6	docking protein 6 (DOK6), mRNA	NM_152721	Hs.569915	AY599248
DOK7	docking protein 7 (DOK7), mRNA	NM_173660	Hs.122110	NM_173660
DPP4	dipeptidyl-peptidase 4 (CD26, adenosine deaminase complexing protein 2) (DPP4), mRNA	NM_001935	Hs.368912	NM_001935
DQ145726	ARNT-interacting protein 2 (AINP2) mRNA, complete cds.	DQ145726	Hs.655196	BE971190
DRD5	dopamine receptor D5 (DRD5), mRNA	NM_000798	Hs.380681	NM_000798
DSCR1L1	Down syndrome critical region gene 1-like 1 (DSCR1L1), mRNA	NM_005822	Hs.440168	NM_005822
DTNA	dystrobrevin, alpha (DTNA), transcript variant 7, mRNA	NM_001392	Hs.695993	NM_001390
DUOX1	dual oxidase 1 (DUOX1), transcript variant 1, mRNA	NM_017434	Hs.272813	NM_017434

DUOX2	dual oxidase 2 (DUOX2), mRNA	NM_014080	Hs.71377	AB209010
DUSP26	dual specificity phosphatase 26 (putative) (DUSP26), mRNA	NM_024025	Hs.8719	AB158288
DUSP27	Novel protein.	ENST00000361	Unknown	
DUX3	double homeobox, 3 (DUX3), mRNA	NM_012148	Unknown	
DVL3	dishevelled, dsh homolog 3 (Drosophila) (DVL3), mRNA	NM_004423	Hs.388116	D86963
DVL3	dishevelled, dsh homolog 3 (Drosophila) (DVL3), mRNA	NM_004423	Hs.388116	D86963
DYNC1LI1	dynein, cytoplasmic 1, light intermediate chain 1 (DYNC1LI1), mRNA	NM_016141	Hs.529495	BX649189
EBI3	Epstein-Barr virus induced gene 3 (EBI3), mRNA	NM_005755	Hs.501452	BM919170
ECGF1	endothelial cell growth factor 1 (platelet-derived) (ECGF1), mRNA	NM_001953	Hs.592212	AK057214
ECHDC2	enoyl Coenzyme A hydratase domain containing 2 (ECHDC2), mRNA	NM_018281	Hs.476319	BC051887
ECM1	extracellular matrix protein 1 (ECM1), transcript variant 1, mRNA	NM_004425	Hs.81071	AK097205
ECM2	extracellular matrix protein 2, female organ and adipocyte specific (ECM2), mRNA	NM_001393	Hs.117060	AB011792
EDN2	endothelin 2 (EDN2), mRNA	NM_001956	Hs.1407	NM_001956
EEF1A2	eukaryotic translation elongation factor 1 alpha 2 (EEF1A2), mRNA	NM_001958	Hs.433839	AB209064
EFHD1	EF-hand domain family, member D1 (EFHD1), mRNA	NM_025202	Hs.516769	CR599551
EFHD1	EF-hand domain family, member D1 (EFHD1), mRNA	NM_025202	Hs.516769	CR599551
EHD3	EH-domain containing 3 (EHD3), mRNA	NM_014600	Hs.368808	NM_014600
EIF2AK1	Eukaryotic translation initiation factor 2-alpha kinase 1 (EC 2.7.11.1) (Heme-regulated eukaryotic initiation factor eIF-2-alpha kinase) (Heme-regulated inhibitor) (Heme-controlled repressor) (HCR) (Hemin-sensitive initiation factor 2-alpha kinase)....	ENST00000199	Unknown	
EIF5A2	eukaryotic translation initiation factor 5A2 (EIF5A2), mRNA	NM_020390	Hs.164144	AY205261
ELN	cDNA PSEC0254 fis, clone NT2RP3003474, moderately similar to ELASTIN PRECURSOR.	AK075554	Hs.647061	NM_000501
ELN	elastin (supravalvular aortic stenosis, Williams-Beuren syndrome), mRNA (cDNA clone MGC:70763 IMAGE:6153564), complete cds.	BC065566	Hs.647061	NM_000501
EMILIN2	elastin microfibril interfacer 2 (EMILIN2), mRNA	NM_032048	Hs.532815	AF270513
ENO3	enolase 3 (beta, muscle) (ENO3), transcript variant 1, mRNA	NM_001976	Hs.224171	NM_001976
ENPP4	ectonucleotide pyrophosphatase/phosphodiesterase 4 (putative function) (ENPP4), mRNA	NM_014936	Hs.696582	AB020686
ENST00000284	PREDICTED: similar to gamma-aminobutyric acid (GABA-A) receptor, subunit epsilon (LOC651250), mRNA	ENST00000284	Unknown	
ENST00000284	PDZ and LIM domain protein 3 (Actinin-associated LIM protein) (Alpha- actinin-2-associated LIM protein).	ENST00000284	Unknown	
ENST00000310	Probable cation-transporting ATPase 13A3 (EC 3.6.3.-) (ATPase family homolog up-regulated in senescence cells 1).	ENST00000310	Unknown	
ENST00000314	Zinc finger protein 541.	ENST00000314	Unknown	
ENST00000315	MGC45800 protein.	ENST00000315	Unknown	
ENST00000319	serine incorporator 4	ENST00000319	Unknown	
ENST00000324	chromosome 21 open reading frame 94, mRNA (cDNA clone MGC:161504 IMAGE:8991942), complete cds.	ENST00000324	Unknown	
ENST00000326	CDNA FLJ35271 fis, clone PROST2005886 (Hypothetical protein FLJ37078) (CDNA FLJ37078 fis, clone BRACE2016315).	ENST00000326	Unknown	
ENST00000328	IGSF5 protein (Fragment).	ENST00000328	Unknown	
ENST00000332	LMOD2 protein (Fragment).	ENST00000332	Unknown	
ENST00000332	full-length cDNA clone CS0DC017YM12 of Neuroblastoma Cot 25-normalized of (human).	ENST00000332	Unknown	
ENST00000333	PREDICTED: similar to Keratin, type I cytoskeletal 18 (Cytokeratin-18) (CK-18) (Keratin-18) (K18) (LOC344462), mRNA	ENST00000333	Unknown	
ENST00000340	CDNA FLJ46111 fis, clone TESTI2034913, moderately similar to Keratin, type II cytoskeletal 8.	ENST00000340	Unknown	
ENST00000346	Zinc finger protein 275.	ENST00000346	Unknown	
ENST00000358	ENST00000358162	ENST00000358	Unknown	
ENST00000360	clone CD-27-VH immunoglobulin heavy chain variable region mRNA, partial cds.	ENST00000360	Unknown	
ENST00000371	Guanine nucleotide-binding protein G(s) subunit alpha isoforms XLas (Adenylate cyclase-stimulating G alpha protein) (Extra large alphas protein) (XLalphas).	ENST00000371	Unknown	
ENST00000372	LIM domain-binding protein 3 (Z-band alternatively spliced PDZ-motif protein) (Protein cypher).	ENST00000372	Unknown	
ENST00000373	Eukaryotic translation initiation factor 4E type 2 (eIF4E type 2) (eIF-4E type 2) (mRNA cap-binding protein type 3) (Eukaryotic translation initiation factor 4E-like 3) (Eukaryotic translation initiation factor 4E homologous protein) (mRNA cap-binding...	ENST00000373	Unknown	
ENST00000374	myosin, heavy polypeptide 7B, cardiac muscle, beta	ENST00000374	Unknown	
ENST00000375	Serine/threonine-protein kinase WNK2 (EC 2.7.11.1) (Protein kinase with no lysine 2) (Protein kinase, lysine-deficient 2).	ENST00000375	Unknown	
ENST00000375	Uncharacterized protein C20orf112.	ENST00000375	Unknown	
ENST00000382	D21S2091E mRNA sequence.	ENST00000382	Unknown	

ENTPD6	ectonucleoside triphosphate diphosphohydrolase 6 (putative function) (ENTPD6), mRNA	NM_001247	Hs.500375	AK124625
EPDR1	ependymin related protein 1 (zebrafish) (EPDR1), mRNA	NM_017549	Hs.563491	NM_017549
EPN3	epsin 3 (EPN3), mRNA	NM_017957	Hs.670090	AK000785
EPS8	epidermal growth factor receptor pathway substrate 8 (EPS8), mRNA	NM_004447	Hs.591160	NM_004447
EPS8L2	EPS8-like 2 (EPS8L2), mRNA	NM_022772	Hs.55016	AK122903
EPSTI1	epithelial stromal interaction 1 (breast) (EPSTI1), transcript variant 2, mRNA	NM_033255	Hs.546467	AL831953
ERGIC2	ERGIC and golgi 2 (ERGIC2), mRNA	NM_016570	Hs.339453	AL834128
ERO1LB	ERO1-like protein beta precursor (EC 1.8.4.-) (ERO1-Lbeta) (Oxidoreductin-1-Lbeta) (Endoplasmic oxidoreductin-1-like protein B).	ENST00000354	Unknown	
ESPNL	espin-like (ESPNL), mRNA	NM_194312	Hs.127724	NM_194312
ETFB	electron-transfer-flavoprotein, beta polypeptide (ETFB), transcript variant 2, mRNA	NM_001014763	Hs.654553	AL833205
ETFB	electron-transfer-flavoprotein, beta polypeptide (ETFB), transcript variant 2, mRNA	NM_001014763	Hs.654553	AL833205
ETFB	electron-transfer-flavoprotein, beta polypeptide (ETFB), transcript variant 2, mRNA	NM_001014763	Hs.654553	AL833205
ETFB	electron-transfer-flavoprotein, beta polypeptide (ETFB), transcript variant 2, mRNA	NM_001014763	Hs.654553	AL833205
ETFB	electron-transfer-flavoprotein, beta polypeptide (ETFB), transcript variant 2, mRNA	NM_001014763	Hs.654553	AL833205
ETFB	electron-transfer-flavoprotein, beta polypeptide (ETFB), transcript variant 2, mRNA	NM_001014763	Hs.654553	AL833205
ETFB	electron-transfer-flavoprotein, beta polypeptide (ETFB), transcript variant 2, mRNA	NM_001014763	Hs.654553	AL833205
ETFB	electron-transfer-flavoprotein, beta polypeptide (ETFB), transcript variant 2, mRNA	NM_001014763	Hs.654553	AL833205
ETFB	electron-transfer-flavoprotein, beta polypeptide (ETFB), transcript variant 2, mRNA	NM_001014763	Hs.654553	AL833205
ETFB	electron-transfer-flavoprotein, beta polypeptide (ETFB), transcript variant 2, mRNA	NM_001014763	Hs.654553	AL833205
EXTL1	exostoses (multiple)-like 1 (EXTL1), mRNA	NM_004455	Hs.150956	NM_004455
F2RL2	coagulation factor II (thrombin) receptor-like 2 (F2RL2), mRNA	NM_004101	Hs.42502	NM_004101
F8	coagulation factor VIII, procoagulant component (hemophilia A) (F8), transcript variant 1, mRNA	NM_000132	Hs.654450	NM_000132
FABP3	fatty acid binding protein 3, muscle and heart (mammary-derived growth inhibitor) (FABP3), mRNA	NM_004102	Hs.657242	BG336702
FAM129A	family with sequence similarity 129, member A (FAM129A), transcript variant 2, mRNA	NM_052966	Hs.518662	NM_052966
FAM13C1	family with sequence similarity 13, member C1 (FAM13C1), transcript variant 2, mRNA	NM_001001971	Hs.607594	BC036453
FAM21C	family with sequence similarity 21, member C (FAM21C), mRNA	NM_015262	Hs.365286	AB011164
FAM36A	family with sequence similarity 36, member A (FAM36A), mRNA	NM_198076	Hs.411490	AK095297
FAM46A	family with sequence similarity 46, member A (FAM46A), mRNA	NM_017633	Hs.10784	NM_017633
FAM46C	family with sequence similarity 46, member C (FAM46C), mRNA	NM_017709	Hs.356216	NM_017709
FAM5B	family with sequence similarity 5, member B (FAM5B), mRNA	NM_021165	Hs.495918	AB161694
FAM5C	family with sequence similarity 5, member C (FAM5C), mRNA	NM_199051	Hs.65765	AB111893
FBLN2	fibulin 2 (FBLN2), transcript variant 1, mRNA	NM_001004019	Hs.198862	NM_00100401[illegible]
FBLN5	fibulin 5 (FBLN5), mRNA	NM_006329	Hs.332708	BX537531
FBLN5	fibulin 5 (FBLN5), mRNA	NM_006329	Hs.332708	BX537531
FBXL7	F-box and leucine-rich repeat protein 7 (FBXL7), mRNA	NM_012304	Hs.433057	AB020647
FBXO40	F-box protein 40 (FBXO40), mRNA	NM_016298	Hs.272564	AB033021
FBXO9	F-box protein 9 (FBXO9), transcript variant 2, mRNA	NM_033480	Hs.216653	NM_033480
FBXO9	F-box protein 9 (FBXO9), transcript variant 2, mRNA	NM_033480	Hs.216653	NM_033480
FBXO9	F-box protein 9 (FBXO9), transcript variant 2, mRNA	NM_033480	Hs.216653	NM_033480
FCAMR	Fc alpha/mu receptor mRNA, complete cds; alternatively spliced.	AY063125	Hs.145519	AF354295
FETUB	fetuin B (FETUB), mRNA	NM_014375	Hs.81073	NM_014375
FGF1	acid fibroblast growth factor-like protein (GLIO703) mRNA, complete cds.	AF211169	Hs.483635	NM_000800
FGF1	fibroblast growth factor 1 (acidic) (FGF1), transcript variant 1, mRNA	NM_000800	Hs.483635	NM_000800
FGF1	fibroblast growth factor 1 (acidic) (FGF1), transcript variant 1, mRNA	NM_000800	Hs.483635	NM_000800
FGF1	fibroblast growth factor 1 (acidic) (FGF1), transcript variant 1, mRNA	NM_000800	Hs.483635	NM_000800
FGF18	fibroblast growth factor 18 (FGF18), mRNA	NM_003862	Hs.87191	AF075292
FGF18	fibroblast growth factor 18 (FGF18), mRNA	NM_003862	Hs.87191	AF075292
FGFBP2	fibroblast growth factor binding protein 2 (FGFBP2), mRNA	NM_031950	Hs.98785	NM_031950
FHIT	fragile histidine triad gene (FHIT), mRNA	NM_002012	Hs.655995	AK127931
FHOD3	formin homology 2 domain containing 3 (FHOD3), mRNA	NM_025135	Hs.436636	NM_025135
FILIP1	filamin A interacting protein 1 (FILIP1), mRNA	NM_015687	Hs.696158	AL832009
FLJ10081	hypothetical protein FLJ10081 (FLJ10081), mRNA	NM_017991	Hs.516341	NM_017991

FLJ10847	hypothetical protein FLJ10847 (FLJ10847), mRNA	NM_018242	Hs.232054	BC010661
FLJ10916	hypothetical protein FLJ10916 (FLJ10916), mRNA	NM_018271	Hs.516179	AK095303
FLJ20152	hypothetical protein FLJ20152 (FLJ20152), transcript variant 1, mRNA	NM_001034850	Hs.481704	NM_00103485
FLJ20701	hypothetical protein FLJ20701 (FLJ20701), mRNA	NM_017933	Hs.409352	NM_017933
FLJ23152	cDNA: FLJ23152 fis, clone LNG09431.	AK026805	Hs.677399	AK026805
FLJ30092	cDNA FLJ30092 fis, clone BNGH41000029.	AK054654	Hs.695995	AB014514
FLJ31715	cDNA FLJ31715 fis, clone NT2RI2006553.	AK056277	Hs.596176	BC022164
FLJ34651	cDNA FLJ34651 fis, clone KIDNE2018167.	AK091970	Unknown	
FLJ36166	hypothetical protein FLJ36166 (FLJ36166), mRNA	NM_182634	Unknown	
FLJ38377	hypothetical protein FLJ38377 (FLJ38377), mRNA	NM_152698	Hs.376218	AK095696
FLJ39502	cDNA FLJ39502 fis, clone PROST2017098.	AK096821	Hs.324341	BX649042
FLJ40504	hypothetical protein FLJ40504 (FLJ40504), mRNA	NM_173624	Hs.371796	AK097823
FLJ46111	FLJ46111 protein, mRNA (cDNA clone MGC:138211 IMAGE:8327474), complete cds.	BC112006	Hs.689472	AK127993
FMOD	fibromodulin (FMOD), mRNA	NM_002023	Hs.519168	NM_002023
FNBP1	formin binding protein 1 (FNBP1), mRNA	NM_015033	Hs.189409	AL049935
FNDC1	fibronectin type III domain containing 1 (FNDC1), mRNA	NM_032532	Hs.520525	NM_032532
FXYD1	FXYD domain containing ion transport regulator 1 (phospholemman) (FXYD1), transcript variant a, mRNA	NM_005031	Hs.442498	AK124802
FZD1	frizzled homolog 1 (Drosophila) (FZD1), mRNA	NM_003505	Hs.94234	AB017363
GABRA1	gamma-aminobutyric acid (GABA) A receptor, alpha 1 (GABRA1), mRNA	NM_000806	Hs.175934	NM_000806
GABRB1	gamma-aminobutyric acid (GABA) A receptor, beta 1 (GABRB1), mRNA	NM_000812	Hs.27283	AK127001
GADD45G	growth arrest and DNA-damage-inducible, gamma (GADD45G), mRNA	NM_006705	Hs.9701	CR613579
GAGE7	G antigen 7 (GAGE7), mRNA	NM_021123	Hs.460641	BC024914
GALNT5	UDP-N-acetyl-alpha-D-galactosamine:polypeptide N-acetylgalactosaminyltransferase 5 (GalNAc-T5) (GALNT5), mRNA	NM_014568	Hs.269027	BC142703
GALNT9	UDP-N-acetyl-alpha-D-galactosamine:polypeptide N-acetylgalactosaminyltransferase 9 (GalNAc-T9) (GALNT9), mRNA	NM_021808	Hs.301062	AF458594
GALT	galactose-1-phosphate uridylyltransferase (GALT), mRNA	NM_000155	Hs.522090	CR606256
GAPVD1	GTPase activating protein and VPS9 domains 1 (GAPVD1), mRNA	NM_015635	Hs.495134	NM_015635
GAPVD1	GTPase activating protein and VPS9 domains 1 (GAPVD1), mRNA	NM_015635	Hs.495134	NM_015635
GAS7	growth arrest-specific 7 (GAS7), transcript variant c, mRNA	NM_201433	Hs.462214	NM_201433
GAS7	growth arrest-specific 7 (GAS7), transcript variant c, mRNA	NM_201433	Hs.462214	NM_201433
GATA4	pp10443 mRNA, complete cds.	AF318320	Hs.243987	NM_002052
GDF2	growth differentiation factor 2 (GDF2), mRNA	NM_016204	Hs.279463	NM_016204
GEM	GTP binding protein overexpressed in skeletal muscle (GEM), transcript variant 1, mRNA	NM_005261	Hs.654463	NM_005261
GENX-3414	genethonin 1 (GENX-3414), mRNA	NM_003943	Hs.109590	CR627383
GFRA1	GDNF family receptor alpha 1 (GFRA1), transcript variant 2, mRNA	NM_145793	Hs.591913	NM_005264
GFRA3	GDNF family receptor alpha 3 (GFRA3), mRNA	NM_001496	Hs.58042	NM_001496
GGTL3	gamma-glutamyltransferase-like 3 (GGTL3), mRNA	NM_178026	Hs.433738	BC141843
GJA12	gap junction protein, alpha 12, 47kDa (GJA12), mRNA	NM_020435	Hs.100072	BC089439
GJA3	gap junction protein, alpha 3, 46kDa (connexin 46) (GJA3), mRNA	NM_021954	Hs.130313	NM_021954
GOT1	glutamic-oxaloacetic transaminase 1, soluble (aspartate aminotransferase 1) (GOT1), mRNA	NM_002079	Hs.500756	CR620721
GOT1	glutamic-oxaloacetic transaminase 1, soluble (aspartate aminotransferase 1) (GOT1), mRNA	NM_002079	Hs.500756	CR620721
GPC1	glypican 1 (GPC1), mRNA	NM_002081	Hs.328232	AB209122
GPNMB	glycoprotein (transmembrane) nmb (GPNMB), transcript variant 1, mRNA	NM_001005340	Hs.190495	BC032783
GPR1	G protein-coupled receptor 1 (GPR1), mRNA	NM_005279	Hs.700744	NM_005279
GPR135	G protein-coupled receptor 135 (GPR135), mRNA	NM_022571	Hs.647573	NM_022571
GPR171	G protein-coupled receptor 171 (GPR171), mRNA	NM_013308	Hs.549152	NM_013308
GPX3	glutathione peroxidase 3 (plasma) (GPX3), mRNA	NM_002084	Hs.386793	NM_002084
GRAMD3	GRAM domain containing 3 (GRAMD3), mRNA	NM_023927	Hs.363558	AK024966
GRIN2C	glutamate receptor, ionotropic, N-methyl D-aspartate 2C (GRIN2C), mRNA	NM_000835	Hs.436980	AB208799
GSG1	germ cell associated 1 (GSG1), transcript variant 1, mRNA	NM_031289	Hs.240053	AK075322
GSG1L	GSG1-like (GSG1L), mRNA	NM_144675	Hs.91910	AK128775
GSTM4	glutathione S-transferase M4 (GSTM4), transcript variant 2, mRNA	NM_147148	Hs.348387	NM_000850

GSTM4	glutathione S-transferase M4 (GSTM4), transcript variant 2, mRNA	NM_147148	Hs.348387	NM_000850
GUP1	GUP1 glycerol uptake/transporter homolog (S. cerevisiae) (GUP1), mRNA	NM_020707	Hs.476041	NM_020707
GYG1	glycogenin 1 (GYG1), mRNA	NM_004130	Hs.477892	CR601242
GYPC	glycophorin C (Gerbich blood group) (GYPC), transcript variant 1, mRNA	NM_002101	Hs.59138	BC016653
H19	H19, imprinted maternally expressed untranslated mRNA (H19) on chromosome 11	NR_002196	Unknown	
H81180	H81180 yu99a12.s1 Soares fetal liver spleen 1NFLS cDNA clone IMAGE:241342 3' similar to gb:X57138_rna1 HISTONE H2B.2 (HUMAN);, mRNA sequence	H81180	Hs.2178	BC069193
HAVCR2	hepatitis A virus cellular receptor 2 (HAVCR2), mRNA	NM_032782	Hs.616365	AK123613
HBLD2	HESB like domain containing 2 (HBLD2), mRNA	NM_030940	Hs.449291	NM_030940
HDAC9	histone deacetylase 9 (HDAC9), transcript variant 2, mRNA	NM_058177	Hs.196054	AJ459808
HEXIM1	hexamethylene bis-acetamide inducible 1 (HEXIM1), mRNA	NM_006460	Hs.15299	NM_006460
HIGD1A	HIG1 domain family, member 1A (HIGD1A), mRNA	NM_014056	Hs.7917	AL833541
HILS1	histone linker H1 domain, spermatid-specific 1 (HILS1), mRNA	NM_194072	Unknown	
HIST1H1C	histone cluster 1, H1c (HIST1H1C), mRNA	NM_005319	Hs.7644	BQ940876
HIST1H1E	histone cluster 1, H1e (HIST1H1E), mRNA	NM_005321	Hs.248133	BU603483
HIST2H2AA4	histone cluster 2, H2aa4 (HIST2H2AA4), mRNA	NM_001040874	Hs.701937	NM_00104087[illegible]
HIST2H2BE	histone cluster 2, H2be (HIST2H2BE), mRNA	NM_003528	Hs.2178	BC069193
HIST2H2BE	histone cluster 2, H2be (HIST2H2BE), mRNA	NM_003528	Hs.2178	BC069193
HIVEP2	human immunodeficiency virus type I enhancer binding protein 2 (HIVEP2), mRNA	NM_006734	Hs.510172	NM_006734
HNMT	histamine N-methyltransferase (HNMT), transcript variant 1, mRNA	NM_006895	Hs.42151	NM_006895
HRC	histidine rich calcium binding protein (HRC), mRNA	NM_002152	Hs.436885	NM_002152
HSD17B1	hydroxysteroid (17-beta) dehydrogenase 1 (HSD17B1), mRNA	NM_000413	Hs.654385	AK127832
HSD3B1	hydroxy-delta-5-steroid dehydrogenase, 3 beta- and steroid delta-isomerase 1 (HSD3B1), mRNA	NM_000862	Hs.364941	CD014103
HSDL2	hydroxysteroid dehydrogenase like 2 (HSDL2), mRNA	NM_032303	Hs.59486	AY093428
HSPA1A	heat shock 70kDa protein 1A (HSPA1A), mRNA	NM_005345	Hs.520028	BC018740
HSPB2	heat shock 27kDa protein 2 (HSPB2), mRNA	NM_001541	Hs.97013	BC110872
HSPB3	heat shock 27kDa protein 3 (HSPB3), mRNA	NM_006308	Hs.41707	NM_006308
HSPB7	heat shock 27kDa protein family, member 7 (cardiovascular) (HSPB7), mRNA	NM_014424	Hs.502612	AL832181
HSPC171	HSPC171 protein (HSPC171), mRNA	NM_014187	Hs.433203	BF204699
HTR2A	5-hydroxytryptamine (serotonin) receptor 2A (HTR2A), mRNA	NM_000621	Hs.654586	X57830
HTRA3	HtrA serine peptidase 3 (HTRA3), mRNA	NM_053044	Hs.479119	BC035717
IFIT2	interferon-induced protein with tetratricopeptide repeats 2 (IFIT2), mRNA	NM_001547	Hs.437609	NM_001547
IFIT3	interferon-induced protein with tetratricopeptide repeats 3 (IFIT3), mRNA	NM_001549	Hs.47338	AF026939
IGF2R	insulin-like growth factor 2 receptor (IGF2R), mRNA	NM_000876	Hs.487062	NM_000876
IGFBP3	insulin-like growth factor binding protein 3 (IGFBP3), transcript variant 1, mRNA	NM_001013398	Hs.450230	NM_00101339[illegible]
IGFBP3	insulin-like growth factor binding protein 3 (IGFBP3), transcript variant 1, mRNA	NM_001013398	Hs.450230	NM_00101339[illegible]
IGFBP3	insulin-like growth factor binding protein 3 (IGFBP3), transcript variant 1, mRNA	NM_001013398	Hs.450230	NM_00101339[illegible]
IGFBP3	insulin-like growth factor binding protein 3 (IGFBP3), transcript variant 1, mRNA	NM_001013398	Hs.450230	NM_00101339[illegible]
IGFBP3	insulin-like growth factor binding protein 3 (IGFBP3), transcript variant 1, mRNA	NM_001013398	Hs.450230	NM_00101339[illegible]
IGFBP3	insulin-like growth factor binding protein 3 (IGFBP3), transcript variant 1, mRNA	NM_001013398	Hs.450230	NM_00101339[illegible]
IGFBP3	insulin-like growth factor binding protein 3 (IGFBP3), transcript variant 1, mRNA	NM_001013398	Hs.450230	NM_00101339[illegible]
IGFBP3	insulin-like growth factor binding protein 3 (IGFBP3), transcript variant 1, mRNA	NM_001013398	Hs.450230	NM_00101339[illegible]
IGFBP3	insulin-like growth factor binding protein 3 (IGFBP3), transcript variant 1, mRNA	NM_001013398	Hs.450230	NM_00101339[illegible]
IGFBP3	insulin-like growth factor binding protein 3 (IGFBP3), transcript variant 1, mRNA	NM_001013398	Hs.450230	NM_00101339[illegible]
IGFBP3	insulin-like growth factor binding protein 3 (IGFBP3), transcript variant 1, mRNA	NM_001013398	Hs.450230	NM_00101339[illegible]
IGFL2	IGF-like family member 2 (IGFL2), mRNA	NM_001002915	Hs.99376	AK093330
IGHV1-69	immunoglobulin heavy variable 1-69, mRNA (cDNA clone MGC:88338 IMAGE:6557369), complete cds.	BC070333	Hs.634941	BQ706543
IHPK3	inositol hexaphosphate kinase 3 (IHPK3), mRNA	NM_054111	Hs.17253	BC089389
IL10	interleukin 10 (IL10), mRNA	NM_000572	Hs.193717	BC022315
IL10RA	interleukin 10 receptor, alpha (IL10RA), mRNA	NM_001558	Hs.504035	AB209626
IL11	interleukin 11 (IL11), mRNA	NM_000641	Hs.467304	NM_000641
IL11RA	interleukin 11 receptor, alpha (IL11RA), transcript variant 2, mRNA	NM_147162	Hs.591088	BC003110
IL13RA2	interleukin 13 receptor, alpha 2 (IL13RA2), mRNA	NM_000640	Hs.336046	U70981

IL17B	interleukin 17B (IL17B), mRNA	NM_014443	Hs.156979	NM_014443
IL1RAP	interleukin 1 receptor accessory protein (IL1RAP), transcript variant 1, mRNA	NM_002182	Hs.478673	NM_002182
IL1RAP	interleukin 1 receptor accessory protein (IL1RAP), transcript variant 1, mRNA	NM_002182	Hs.478673	NM_002182
IL22RA1	interleukin 22 receptor, alpha 1 (IL22RA1), mRNA	NM_021258	Hs.110915	BC029273
IL22RA1	interleukin 22 receptor, alpha 1 (IL22RA1), mRNA	NM_021258	Hs.110915	BC029273
IL33	interleukin 33 (IL33), mRNA	NM_033439	Hs.348390	BC047085
IL7	interleukin 7 (IL7), mRNA	NM_000880	Hs.591873	BC047698
INPP5A	inositol polyphosphate-5-phosphatase, 40kDa (INPP5A), mRNA	NM_005539	Hs.523360	NM_005539
IQCG	IQ motif containing G (IQCG), mRNA	NM_032263	Hs.591675	AK095094
IQSEC3	IQ motif and Sec7 domain 3 (IQSEC3), mRNA	NM_015232	Hs.536319	AB029033
IRX4	iroquois homeobox protein 4 (IRX4), mRNA	NM_016358	Hs.196927	NM_016358
ISLR	immunoglobulin superfamily containing leucine-rich repeat (ISLR), transcript variant 1, mRNA	NM_005545	Hs.699822	AK074668
ISLR2	immunoglobulin superfamily containing leucine-rich repeat 2 (ISLR2), mRNA	NM_020851	Hs.254775	AB040898
ITGA1	integrin, alpha 1 (ITGA1), mRNA	NM_181501	Hs.696076	BX648284
ITGA11	integrin, alpha 11 (ITGA11), transcript variant 1, mRNA	NM_001004439	Hs.436416	NM_00100443[illegible]
ITGA7	integrin, alpha 7 (ITGA7), mRNA	NM_002206	Hs.524484	BC050280
ITGAV	integrin, alpha V (vitronectin receptor, alpha polypeptide, antigen CD51) (ITGAV), mRNA	NM_002210	Hs.436873	NM_002210
ITGAV	integrin, alpha V (vitronectin receptor, alpha polypeptide, antigen CD51) (ITGAV), mRNA	NM_002210	Hs.436873	NM_002210
ITGAV	integrin, alpha V (vitronectin receptor, alpha polypeptide, antigen CD51) (ITGAV), mRNA	NM_002210	Hs.436873	NM_002210
ITGB1BP2	integrin beta 1 binding protein (melusin) 2 (ITGB1BP2), mRNA	NM_012278	Hs.109999	BM808220
ITGB2	integrin, beta 2 (complement component 3 receptor 3 and 4 subunit) (ITGB2), mRNA	NM_000211	Hs.375957	NM_000211
ITGB2	integrin, beta 2 (complement component 3 receptor 3 and 4 subunit) (ITGB2), mRNA	NM_000211	Hs.375957	NM_000211
ITGB2	integrin, beta 2 (complement component 3 receptor 3 and 4 subunit) (ITGB2), mRNA	NM_000211	Hs.375957	NM_000211
ITGB2	integrin, beta 2 (complement component 3 receptor 3 and 4 subunit) (ITGB2), mRNA	NM_000211	Hs.375957	NM_000211
ITGB2	integrin, beta 2 (complement component 3 receptor 3 and 4 subunit) (ITGB2), mRNA	NM_000211	Hs.375957	NM_000211
ITGB2	integrin, beta 2 (complement component 3 receptor 3 and 4 subunit) (ITGB2), mRNA	NM_000211	Hs.375957	NM_000211
ITGB2	integrin, beta 2 (complement component 3 receptor 3 and 4 subunit) (ITGB2), mRNA	NM_000211	Hs.375957	NM_000211
ITGB2	integrin, beta 2 (complement component 3 receptor 3 and 4 subunit) (ITGB2), mRNA	NM_000211	Hs.375957	NM_000211
ITGB2	integrin, beta 2 (complement component 3 receptor 3 and 4 subunit) (ITGB2), mRNA	NM_000211	Hs.375957	NM_000211
ITGB8	integrin, beta 8 (ITGB8), mRNA	NM_002214	Hs.592171	NM_002214
ITIH5	inter-alpha (globulin) inhibitor H5 (ITIH5), transcript variant 1, mRNA	NM_030569	Hs.498586	CR627109
ITIH5	inter-alpha (globulin) inhibitor H5 (ITIH5), transcript variant 3, mRNA	NM_001001851	Hs.498586	CR627109
ITIH5	inter-alpha (globulin) inhibitor H5 (ITIH5), transcript variant 1, mRNA	NM_030569	Hs.498586	CR627109
ITIH5	inter-alpha (globulin) inhibitor H5 (ITIH5), transcript variant 1, mRNA	NM_030569	Hs.498586	CR627109
ITK	IL2-inducible T-cell kinase (ITK), mRNA	NM_005546	Hs.558348	AB209622
JPH2	junctophilin 2 (JPH2), transcript variant 1, mRNA	NM_020433	Hs.441737	NM_020433
K03200	Human melanoma-associated antigen p97 (melanotransferrin) mRNA, 3' flank.	K03200	Unknown	
KCNA4	potassium voltage-gated channel, shaker-related subfamily, member 4 (KCNA4), mRNA	NM_002233	Hs.592002	NM_002233
KCND3	Potassium voltage-gated channel subfamily D member 3 (Voltage-gated potassium channel subunit Kv4.3).	ENST00000369	Unknown	
KCND3	Potassium voltage-gated channel subfamily D member 3 (Voltage-gated potassium channel subunit Kv4.3).	ENST00000369	Unknown	
KCNE1	potassium voltage-gated channel, Isk-related family, member 1 (KCNE1), mRNA	NM_000219	Hs.121495	NM_000219
KCNE4	potassium voltage-gated channel, Isk-related family, member 4 (KCNE4), mRNA	NM_080671	Hs.348522	NM_080671
KCNF1	potassium voltage-gated channel, subfamily F, member 1 (KCNF1), mRNA	NM_002236	Hs.23735	BC026110
KCNH2	potassium voltage-gated channel, subfamily H (eag-related), member 2 (KCNH2), transcript variant 1, mRNA	NM_000238	Hs.647099	DQ525913
KCNH2	potassium voltage-gated channel, subfamily H (eag-related), member 2 (KCNH2), transcript variant 2, mRNA	NM_172056	Hs.647099	DQ525913
KCNJ12	potassium inwardly-rectifying channel, subfamily J, member 12 (KCNJ12), mRNA	NM_021012	Hs.200629	NM_021012
KCNJ3	potassium inwardly-rectifying channel, subfamily J, member 3 (KCNJ3), mRNA	NM_002239	Hs.591606	NM_002239
KCNJ4	potassium inwardly-rectifying channel, subfamily J, member 4 (KCNJ4), transcript variant 1, mRNA	NM_152868	Hs.32505	NM_152868
KCNJ5	potassium inwardly-rectifying channel, subfamily J, member 5 (KCNJ5), mRNA	NM_000890	Hs.632109	NM_000890
KCNJ5	potassium inwardly-rectifying channel, subfamily J, member 5 (KCNJ5), mRNA	NM_000890	Hs.632109	NM_000890
KCNK3	potassium channel, subfamily K, member 3 (KCNK3), mRNA	NM_002246	Hs.645288	NM_002246

KCNMB1	potassium large conductance calcium-activated channel, subfamily M, beta member 1 (KCNMB1), mRNA	NM_004137	Hs.484099	BC025707
KCNMB2	potassium large conductance calcium-activated channel, subfamily M, beta member 2 (KCNMB2), transcript variant 1, mRNA	NM_181361	Hs.478368	AF209747
KCNMB2	potassium large conductance calcium-activated channel, subfamily M, beta member 2 (KCNMB2), transcript variant 1, mRNA	NM_181361	Hs.478368	AF209747
KCNMB4	potassium large conductance calcium-activated channel, subfamily M, beta member 4 (KCNMB4), mRNA	NM_014505	Hs.525529	NM_014505
KCNQ1	potassium voltage-gated channel, KQT-like subfamily, member 1 (KCNQ1), transcript variant 1, mRNA	NM_000218	Hs.95162	BX640740
KCTD11	potassium channel tetramerisation domain containing 11 (KCTD11), mRNA	NM_001002914	Hs.592112	NM_00100291[illegible]
KCTD4	potassium channel tetramerisation domain containing 4 (KCTD4), mRNA	NM_198404	Hs.23406	NM_198404
KIAA0143	KIAA0143 protein (KIAA0143), mRNA	NM_015137	Hs.204564	NM_015137
KIAA0363	Human mRNA for KIAA0363 gene, partial cds.	ENST00000258	Unknown	
KIAA0774	KIAA0774 (KIAA0774), transcript variant 1, mRNA	NM_001033602	Hs.22287	NM_00103360[illegible]
KIAA0774	KIAA0774 (KIAA0774), transcript variant 1, mRNA	NM_001033602	Hs.22287	NM_00103360[illegible]
KIAA1199	KIAA1199 (KIAA1199), mRNA	NM_018689	Hs.459088	AY581148
KIAA1199	KIAA1199 (KIAA1199), mRNA	NM_018689	Hs.459088	AY581148
KIAA1908	mRNA for KIAA1908 protein, partial cds.	AB067495	Hs.436146	AB067495
KIDINS220	kinase D-interacting substance of 220 kDa (KIDINS220), mRNA	NM_020738	Hs.9873	AB033076
KIRREL	kin of IRRE like (Drosophila) (KIRREL), mRNA	NM_018240	Hs.657006	AY302131
KLF5	Kruppel-like factor 5 (intestinal) (KLF5), mRNA	NM_001730	Hs.508234	AF132818
KLHDC3	kelch domain containing 3 (KLHDC3), mRNA	NM_057161	Hs.412468	BC021546
KLHL21	kelch-like 21 (Drosophila) (KLHL21), mRNA	NM_014851	Hs.7764	NM_014851
KREMEN1	kringle containing transmembrane protein 1 (KREMEN1), transcript variant 3, mRNA	NM_001039570	Hs.229335	NM_00103957[illegible]
KRT18	keratin 18 (KRT18), transcript variant 1, mRNA	NM_000224	Hs.406013	CR616919
KRT18	keratin 18 (KRT18), transcript variant 1, mRNA	NM_000224	Hs.406013	CR616919
KRT18	keratin 18 (KRT18), transcript variant 1, mRNA	NM_000224	Hs.406013	CR616919
KRT72	keratin 72 (KRT72), mRNA	NM_080747	Hs.662013	NM_080747
KRT8	keratin 8 (KRT8), mRNA	NM_002273	Hs.533782	CR607281
KRT8	keratin 8 (KRT8), mRNA	NM_002273	Hs.533782	CR607281
KRTHB5	mRNA; cDNA DKFZp547P055 (from clone DKFZp547P055).	AL359596	Hs.42547	AL359596
LAMA2	laminin, alpha 2 (merosin, congenital muscular dystrophy) (LAMA2), transcript variant 1, mRNA	NM_000426	Hs.200841	NM_000426
LARP6	La ribonucleoprotein domain family, member 6 (LARP6), transcript variant 1, mRNA	NM_018357	Hs.416755	NM_018357
LGALS3BP	lectin, galactoside-binding, soluble, 3 binding protein (LGALS3BP), mRNA	NM_005567	Hs.514535	BC015761
LIF	leukemia inhibitory factor (cholinergic differentiation factor) (LIF), mRNA	NM_002309	Hs.2250	NM_002309
LIFR	leukemia inhibitory factor receptor alpha (LIFR), mRNA	NM_002310	Hs.133421	NM_002310
LILRB3	leukocyte immunoglobulin-like receptor, subfamily B (with TM and ITIM domains), member 3 (LILRB3), mRNA	NM_006864	Hs.631592	NM_024318
LIMS2	LIM and senescent cell antigen-like domains 2 (LIMS2), mRNA	NM_017980	Hs.469881	AK123014
LIN7B	lin-7 homolog B (C. elegans) (LIN7B), mRNA	NM_022165	Hs.221737	BG749971
LINCR	likely ortholog of mouse lung-inducible Neutralized-related C3HC4 RING domain protein, mRNA (cDNA clone MGC:15646 IMAGE:3346442), complete cds.	BC012317	Hs.149219	AL389981
LMCD1	LIM and cysteine-rich domains 1 (LMCD1), mRNA	NM_014583	Hs.475353	AK074448
LMO7	LIM domain 7 (LMO7), mRNA	NM_005358	Hs.207631	AF330045
LMO7	LIM domain 7 (LMO7), mRNA	NM_005358	Hs.207631	AF330045
LMOD1	leiomodin 1 (smooth muscle) (LMOD1), mRNA	NM_012134	Hs.519075	AK127212
LMOD3	leiomodin 3 (fetal) (LMOD3), mRNA	NM_198271	Hs.350621	AL832033
LOC121054	PREDICTED: similar to Keratin, type I cytoskeletal 18 (Cytokeratin-18) (CK-18) (Keratin-18) (K18) (LOC121054), mRNA	XR_019191	Hs.646407	XR_019191
LOC132205	cDNA FLJ33859 fis, clone CTONG2006223, moderately similar to KERATIN, TYPE II CYTOSKELETAL 8.	AK091178	Hs.590045	AK091178
LOC132391	PREDICTED: similar to Keratin, type I cytoskeletal 18 (Cytokeratin-18) (CK-18) (Keratin-18) (K18) (LOC132391), mRNA	XR_018938	Hs.648019	XR_018938
LOC139060	PREDICTED: similar to Keratin, type I cytoskeletal 18 (Cytokeratin-18) (CK-18) (Keratin-18) (K18) (LOC139060), mRNA	XR_018311	Hs.648308	XR_018311
LOC150166	cDNA FLJ32274 fis, clone PROST2000036.	AK056836	Hs.48353	AK056836
LOC161247	similar to CG10671-like (LOC161247), mRNA	NM_203402	Hs.128060	BC139911
LOC202134	hypothetical protein LOC202134 (LOC202134), mRNA	NM_001079529	Hs.646912	NM_00107952[illegible]
LOC219731	cDNA FLJ32141 fis, clone PLACE5000067.	AK056703	Hs.585473	AK056703
LOC253012	hypothetical protein LOC253012 (LOC253012), transcript variant 1, mRNA	NM_001039372	Hs.443169	BC046365

LOC254848	hypothetical protein LOC254848, mRNA (cDNA clone IMAGE:4544270), with apparent retained intron.	BC014113	Hs.663029	BC043614
LOC255783	hypothetical protein LOC255783 (LOC255783) on chromosome 19	NR_002797	Unknown	
LOC283278	mRNA; cDNA DKFZp686B1134 (from clone DKFZp686B1134).	AL833334	Hs.12332	NM_175058
LOC283701	mRNA for FLJ00278 protein.	AK090401	Hs.546844	AK090401
LOC283861	cDNA FLJ40824 fis, clone TRACH2011113.	AK098143	Hs.192155	AK098143
LOC285813	cDNA FLJ36950 fis, clone BRACE2005742.	AK094269	Hs.594133	AK094269
LOC286016	hypothetical protein LOC286016 (LOC286016) on chromosome 7	NR_002187	Unknown	
LOC339781	PREDICTED: similar to Keratin, type I cytoskeletal 18 (Cytokeratin-18) (CK-18) (Keratin-18) (K18) (LOC339781), mRNA	XR_019026	Hs.647837	XR_019026
LOC342419	PREDICTED: similar to Keratin, type II cytoskeletal 8 (Cytokeratin-8) (CK-8) (Keratin-8) (K8) (Cytokeratin endo A) (LOC342419), mRNA	XR_017139	Hs.647373	XR_017139
LOC342732	PREDICTED: similar to keratin 8 (LOC342732), mRNA	XR_017372	Hs.464986	BC051727
LOC343326	PREDICTED: similar to Keratin, type I cytoskeletal 18 (Cytokeratin-18) (CK-18) (Keratin-18) (K18) (LOC343326), mRNA	XR_019568	Hs.647720	XR_019568
LOC344320	PREDICTED: similar to Keratin, type II cytoskeletal 8 (Cytokeratin-8) (CK-8) (Keratin-8) (K8) (LOC344320), mRNA	XR_018991	Hs.647887	XR_018991
LOC345430	PREDICTED: similar to Keratin, type I cytoskeletal 18 (Cytokeratin-18) (CK-18) (Keratin-18) (K18) (LOC345430), mRNA	XR_016695	Hs.693383	XR_016695
LOC347333	PREDICTED: similar to Keratin, type II cytoskeletal 8 (Cytokeratin-8) (CK-8) (Keratin-8) (K8) (LOC347333), mRNA	XR_018299	Hs.650946	XR_018299
LOC375449	similar to microtubule associated testis specific serine/threonine protein kinase (LOC375449), mRNA	NM_198828	Hs.595458	AY830839
LOC388135	similar to RIKEN cDNA 6030419C18 gene (LOC388135), mRNA	NM_001039614	Hs.40794	BC111368
LOC389517	similar to Williams Beuren syndrome chromosome region 19 (LOC389517), mRNA	NM_001032389	Unknown	
LOC390472	PREDICTED: similar to Keratin, type II cytoskeletal 8 (Cytokeratin-8) (CK-8) (Keratin-8) (K8) (LOC390472), mRNA	XR_019203	Hs.647551	XR_019203
LOC390610	PREDICTED: similar to Keratin, type II cytoskeletal 8 (Cytokeratin-8) (CK-8) (Keratin-8) (K8) (LOC390610), mRNA	XR_017341	Hs.699134	XR_017341
LOC390904	PREDICTED: similar to Keratin, type I cytoskeletal 18 (Cytokeratin-18) (CK-18) (Keratin-18) (K18) (LOC390904), mRNA	XR_017288	Hs.611474	BM017017
LOC391081	PREDICTED: similar to 3 beta-hydroxysteroid dehydrogenase	XR_019377	Hs.660905	XR_019377
LOC391179	PREDICTED: similar to Keratin, type I cytoskeletal 18 (Cytokeratin-18) (CK-18) (Keratin-18) (K18) (LOC391179), mRNA	XR_017012	Hs.693418	XR_018953
LOC391271	PREDICTED: hypothetical LOC391271 (LOC391271), mRNA	XR_019037	Hs.647453	XR_019037
LOC391485	PREDICTED: similar to Keratin, type II cytoskeletal 8 (Cytokeratin-8) (CK-8) (Keratin-8) (K8) (Cytokeratin endo A) (LOC391485), mRNA	XR_018532	Hs.693407	XR_018532
LOC391589	PREDICTED: similar to Keratin, type I cytoskeletal 18 (Cytokeratin-18) (CK-18) (Keratin-18) (K18) (LOC391589), mRNA	XR_019198	Hs.647934	XR_019198
LOC391803	PREDICTED: similar to Keratin, type I cytoskeletal 18 (Cytokeratin-18) (CK-18) (Keratin-18) (K18) (LOC391803), mRNA	XR_018462	Hs.646957	XR_018462
LOC391819	PREDICTED: similar to Keratin, type I cytoskeletal 18 (Cytokeratin-18) (CK-18) (Keratin-18) (K18) (LOC391819), mRNA	XR_016386	Hs.693265	XR_016386
LOC391819	PREDICTED: similar to Keratin, type I cytoskeletal 18 (Cytokeratin-18) (CK-18) (Keratin-18) (K18) (LOC391819), mRNA	XR_016386	Hs.693265	XR_016386
LOC391827	PREDICTED: similar to Keratin, type I cytoskeletal 18 (Cytokeratin-18) (CK-18) (Keratin-18) (K18) (LOC391827), mRNA	XR_018420	Hs.646672	XR_018420
LOC392479	PREDICTED: similar to Keratin, type II cytoskeletal 8 (Cytokeratin-8) (CK-8) (Keratin-8) (K8) (LOC392479), mRNA	XR_018698	Hs.650943	XR_018698
LOC400794	hypothetical gene supported by BC030596, mRNA (cDNA clone IMAGE:4823265).	BC033551	Hs.374847	BC030596
LOC401317	cDNA clone IMAGE:30398108.	ENST00000381	Unknown	
LOC401317	cDNA clone IMAGE:30398108.	ENST00000381	Unknown	
LOC402429	PREDICTED: similar to Keratin, type II cytoskeletal 8 (Cytokeratin-8) (CK-8) (Keratin-8) (K8) (LOC402429), mRNA	XR_018415	Hs.648362	XR_018415
LOC440799	purinergic receptor P2X-like 1, orphan receptor pseudogene (LOC440799) on chromosome 22	NR_002829	Unknown	
LOC442249	PREDICTED: hypothetical LOC442249 (LOC442249), mRNA	XR_019231	Hs.651269	XR_019231
LOC442405	PREDICTED: similar to Keratin, type I cytoskeletal 18 (Cytokeratin-18) (CK-18) (Keratin-18) (K18) (LOC442405), mRNA	XR_018749	Hs.648143	XR_018749
LOC554208	cDNA FLJ25893 fis, clone CBR03492.	AK098759	Hs.406964	AK098759
LOC642448	PREDICTED: similar to Keratin, type I cytoskeletal 18 (Cytokeratin-18) (CK-18) (Keratin-18) (K18) (LOC642448), mRNA	XR_016161	Hs.648272	XR_016161
LOC643143	PREDICTED: hypothetical LOC643143 (LOC643143), mRNA	XM_931358	Hs.632493	DA898365
LOC643447	PREDICTED: similar to Keratin, type II cytoskeletal 8 (Cytokeratin-8) (CK-8) (Keratin-8) (K8) (LOC643447), mRNA	XR_016392	Hs.647309	XR_016392
LOC643471	PREDICTED: similar to Keratin, type I cytoskeletal 18 (Cytokeratin-18) (CK-18) (Keratin-18) (K18) (LOC643471), mRNA	XR_016765	Hs.647770	XR_018670
LOC643594	PREDICTED: similar to CG13731-PA (LOC643594), mRNA	XM_926898	Unknown	
LOC643950	AF150420 Human mRNA from cd34+ stem cells cDNA clone CBNAZD12, mRNA sequence	AF150420	Hs.560372	AF150420
LOC644030	PREDICTED: similar to Keratin, type I cytoskeletal 18 (Cytokeratin-18) (CK-18) (Keratin-18) (K18) (LOC644030), mRNA	XR_019060	Hs.650993	XR_019060

LOC644998	PREDICTED: similar to Keratin, type II cytoskeletal 8 (Cytokeratin-8) (CK-8) (Keratin-8) (K8) (LOC644998), mRNA	XR_016847	Hs.648222	XR_016847
LOC645212	cDNA clone IMAGE:5314207.	ENST00000313	Unknown	
LOC646723	PREDICTED: similar to Keratin, type I cytoskeletal 18 (Cytokeratin-18) (CK-18) (Keratin-18) (K18) (LOC646723), mRNA	XR_017241	Hs.663868	XR_018339
LOC647162	PREDICTED: similar to Keratin, type I cytoskeletal 18 (Cytokeratin-18) (CK-18) (Keratin-18) (K18) (LOC647162), mRNA	XR_017456	Hs.683581	XR_019330
LOC647162	PREDICTED: similar to Keratin, type I cytoskeletal 18 (Cytokeratin-18) (CK-18) (Keratin-18) (K18) (LOC647162), mRNA	XR_017456	Hs.683581	XR_019330
LOC647913	PREDICTED: similar to Keratin, type I cytoskeletal 18 (Cytokeratin-18) (CK-18) (Keratin-18) (K18) (LOC647913), mRNA	XR_018216	Hs.648340	XR_018216
LOC649233	PREDICTED: similar to Keratin, type I cytoskeletal 18 (Cytokeratin-18) (CK-18) (Keratin-18) (K18) (LOC649233), mRNA	XR_018843	Hs.616869	XR_018843
LOC649344	PREDICTED: similar to Keratin, type II cytoskeletal 8 (Cytokeratin-8) (CK-8) (Keratin-8) (K8) (LOC649344), mRNA	XR_018597	Hs.647727	XR_018597
LOC649375	PREDICTED: similar to Keratin, type I cytoskeletal 18 (Cytokeratin-18) (CK-18) (Keratin-18) (K18) (LOC649375), mRNA	XR_018559	Hs.647487	XR_018559
LOC649545	PREDICTED: similar to Keratin, type II cytoskeletal 8 (Cytokeratin-8) (CK-8) (Keratin-8) (K8) (LOC649545), mRNA	XR_018641	Hs.693340	XR_018641
LOC649928	PREDICTED: similar to Keratin, type I cytoskeletal 18 (Cytokeratin-18) (CK-18) (Keratin-18) (K18) (LOC649928), mRNA	XR_018959	Hs.648052	XR_018959
LOC651154	PREDICTED: similar to actinin, alpha 4 (LOC651154), mRNA	XR_018943	Hs.648017	XR_018943
LOC651439	PREDICTED: similar to Keratin, type I cytoskeletal 18 (Cytokeratin-18) (CK-18) (Keratin-18) (K18) (LOC651439), mRNA	XR_019146	Hs.647572	XR_019146
LOC651696	PREDICTED: similar to Keratin, type I cytoskeletal 18 (Cytokeratin-18) (CK-18) (Keratin-18) (K18) (LOC651696), mRNA	XR_019148	Hs.646788	BM547144
LOC651721	hypothetical protein LOC651721, mRNA (cDNA clone IMAGE:4430430), partial cds.	BC026225	Hs.598754	BC026225
LOC651758	PREDICTED: similar to ciliary rootlet coiled-coil, rootletin (LOC651758), mRNA	XM_940977	Unknown	
LOC651900	hypothetical protein LOC651900, mRNA (cDNA clone IMAGE:3874689), partial cds.	BC008248	Hs.559249	BC008248
LOC651929	PREDICTED: similar to Keratin, type I cytoskeletal 18 (Cytokeratin-18) (CK-18) (Keratin-18) (K18) (LOC651929), mRNA	XR_019186	Hs.646986	XR_019186
LOC653284	mRNA; cDNA DKFZp667O0524 (from clone DKFZp667O0524).	AL832834	Hs.404220	BX640681
LOC728371	PREDICTED: similar to ankyrin repeat domain 20A (LOC728371), mRNA	XR_015273	Hs.675938	XR_015273
LOC728586	PREDICTED: hypothetical protein LOC728586 (LOC728586), mRNA	XM_001130569	Hs.591271	XM_00113056[illegible]
LOC729344	PREDICTED: similar to Keratin, type II cytoskeletal 8 (Cytokeratin-8) (CK-8) (Keratin-8) (K8) (Cytokeratin endo A) (LOC729344), mRNA	XR_015516	Hs.646513	XR_015516
LOC729970	cDNA FLJ37477 fis, clone BRAWH2013047.	AK094796	Hs.297988	AK094796
LOC730227	cDNA FLJ14231 fis, clone NT2RP3004470.	AK024293	Hs.293928	AK024293
LOC730683	PREDICTED: similar to notch1-induced protein (LOC730683), mRNA	XM_001126777	Unknown	
LOC730957	PREDICTED: similar to filaggrin (LOC730957), mRNA	XM_001127881	Unknown	
LOC731274	PREDICTED: similar to tau tubulin kinase 2 (LOC731274), mRNA	XR_015430	Hs.646971	XR_015430
LOC731794	PREDICTED: similar to Keratin, type I cytoskeletal 18 (Cytokeratin-18) (CK-18) (Keratin-18) (K18) (LOC731794), mRNA	XR_015605	Hs.647846	XR_015605
LOC81691	exonuclease NEF-sp (LOC81691), mRNA	NM_030941	Hs.177926	AK057254
LONP2	lon peptidase 2, peroxisomal (LONP2), mRNA	NM_031490	Hs.295923	AK090452
LOXL1	lysyl oxidase-like 1 (LOXL1), mRNA	NM_005576	Hs.65436	BC068542
LOXL4	lysyl oxidase-like 4 (LOXL4), mRNA	NM_032211	Hs.306814	BC013153
LPL	lipoprotein lipase (LPL), mRNA	NM_000237	Hs.180878	NM_000237
LPP	LIM domain containing preferred translocation partner in lipoma (LPP), mRNA	NM_005578	Hs.444362	NM_005578
LRCH1	leucine-rich repeats and calponin homology (CH) domain containing 1 (LRCH1), mRNA	NM_015116	Hs.656722	AY050632
LRP1	low density lipoprotein-related protein 1 (alpha-2-macroglobulin receptor) (LRP1), mRNA	NM_002332	Hs.162757	NM_002332
LRP1	low density lipoprotein-related protein 1 (alpha-2-macroglobulin receptor) (LRP1), mRNA	NM_002332	Hs.162757	NM_002332
LRRC55	leucine rich repeat containing 55 (LRRC55), mRNA	NM_001005210	Hs.199853	AK127591
LRRTM2	leucine rich repeat transmembrane neuronal 2 (LRRTM2), mRNA	NM_015564	Hs.656653	NM_015564
LRTM2	leucine-rich repeats and transmembrane domains 2 (LRTM2), mRNA	NM_001039029	Hs.585579	AK126866
LSM1	LSM1 homolog, U6 small nuclear RNA associated (S. cerevisiae) (LSM1), mRNA	NM_014462	Hs.425311	CR593275
LTK	leukocyte tyrosine kinase (LTK), transcript variant 1, mRNA	NM_002344	Hs.434481	BC045607
LYSMD1	LysM, putative peptidoglycan-binding, domain containing 1 (LYSMD1), mRNA	NM_212551	Hs.591482	BX647911
LYSMD4	mRNA; cDNA DKFZp779J2370 (from clone DKFZp779J2370).	BX640928	Hs.562568	AK124852
LYZL4	lysozyme-like 4 (LYZL4), mRNA	NM_144634	Hs.234895	BG208285
MAGI3	membrane associated guanylate kinase, WW and PDZ domain containing 3 (MAGI3), transcript variant 1, mRNA	NM_020965	Unknown	
MAN1C1	mannosidase, alpha, class 1C, member 1 (MAN1C1), mRNA	NM_020379	Hs.197043	AB209275

MAN1C1	Mannosyl-oligosaccharide 1,2-alpha-mannosidase IC (EC 3.2.1.113) (Processing alpha-1,2-mannosidase IC) (Alpha-1,2-mannosidase IC) (Mannosidase alpha class 1C member 1) (HMIC).	ENST00000374	Unknown	
MAOB	monoamine oxidase B (MAOB), nuclear gene encoding mitochondrial protein, mRNA	NM_000898	Hs.654473	NM_000898
MAOB	monoamine oxidase B (MAOB), nuclear gene encoding mitochondrial protein, mRNA	NM_000898	Hs.654473	NM_000898
MAP3K2	mitogen-activated protein kinase kinase kinase 2 (MAP3K2), mRNA	NM_006609	Hs.145605	NM_006609
MAP3K2	mitogen-activated protein kinase kinase kinase 2 (MAP3K2), mRNA	NM_006609	Hs.145605	NM_006609
MAP6	microtubule-associated protein 6 (MAP6), transcript variant 1, mRNA	NM_033063	Hs.585540	BC150254
MAPRE3	microtubule-associated protein, RP/EB family, member 3 (MAPRE3), mRNA	NM_012326	Hs.515860	AB025186
MARCH6	membrane-associated ring finger (C3HC4) 6 (MARCH6), mRNA	NM_005885	Hs.432862	BC111494
MASP1	mannan-binding lectin serine peptidase 1 (C4/C2 activating component of Ra-reactive factor) (MASP1), transcript variant 3, mRNA	NM_001031849	Hs.89983	BX641029
MASP1	mannan-binding lectin serine peptidase 1 (C4/C2 activating component of Ra-reactive factor) (MASP1), transcript variant 2, mRNA	NM_139125	Hs.89983	BX641029
MATN2	matrilin 2 (MATN2), transcript variant 2, mRNA	NM_030583	Hs.189445	BX648291
MB	myoglobin (MB), transcript variant 2, mRNA	NM_203377	Hs.517586	BF670653
MCEE	methylmalonyl CoA epimerase (MCEE), mRNA	NM_032601	Hs.94949	AB209469
MDGA1	MAM domain containing glycosylphosphatidylinositol anchor 1 (MDGA1), mRNA	NM_153487	Hs.437993	AF478693
MDH1	malate dehydrogenase 1, NAD (soluble) (MDH1), mRNA	NM_005917	Hs.526521	AL832067
MEF2A	MADS box transcription enhancer factor 2, polypeptide A (myocyte enhancer factor 2A) (MEF2A), mRNA	NM_005587	Hs.268675	AL831995
MEF2B	MADS box transcription enhancer factor 2, polypeptide B (myocyte enhancer factor 2B) (MEF2B), mRNA	NM_005919	Hs.153629	AK128256
MFAP5	microfibrillar associated protein 5 (MFAP5), mRNA	NM_003480	Hs.512842	NM_003480
MFI2	antigen p97 (melanoma associated) identified by monoclonal antibodies 133.2 and 96.5 (MFI2), transcript variant 2, mRNA	NM_033316	Hs.184727	NM_005929
MFN2	mitofusin 2 (MFN2), nuclear gene encoding mitochondrial protein, mRNA	NM_014874	Hs.695980	D86987
MGC13057	hypothetical protein MGC13057 (MGC13057), transcript variant 1, mRNA	NM_001042519	Hs.389311	NM_00104251
MGC23985	similar to AVLV472 (MGC23985), mRNA	NM_206966	Hs.660038	CD556191
MGC99813	similar to RIKEN cDNA A230078I05 gene (MGC99813), mRNA	NM_001005209	Hs.446664	BC068567
MID1	Midline-1 (EC 6.3.2.-) (Tripartite motif-containing protein 18) (Putative transcription factor XPRF) (Midin) (RING finger protein 59) (Midline 1 RING finger protein).	ENST00000380	Unknown	
MITF	microphthalmia-associated transcription factor (MITF), transcript variant 1, mRNA	NM_198159	Hs.166017	NM_198159
MLCK	MLCK protein (MLCK), mRNA	NM_182493	Hs.130465	NM_182493
MMP15	matrix metallopeptidase 15 (membrane-inserted) (MMP15), mRNA	NM_002428	Hs.80343	NM_002428
MMP15	matrix metallopeptidase 15 (membrane-inserted) (MMP15), mRNA	NM_002428	Hs.80343	NM_002428
MOAP1	modulator of apoptosis 1 (MOAP1), mRNA	NM_022151	Hs.24719	NM_022151
MOG	myelin oligodendrocyte glycoprotein (MOG), transcript variant alpha4, mRNA	NM_206814	Unknown	
MPP3	membrane protein, palmitoylated 3 (MAGUK p55 subfamily member 3) (MPP3), mRNA	NM_001932	Hs.396566	U37707
MPP3	membrane protein, palmitoylated 3 (MAGUK p55 subfamily member 3) (MPP3), mRNA	NM_001932	Hs.396566	U37707
MPP7	membrane protein, palmitoylated 7 (MAGUK p55 subfamily member 7) (MPP7), mRNA	NM_173496	Hs.499159	NM_173496
MRAS	muscle RAS oncogene homolog (MRAS), mRNA	NM_012219	Hs.527021	NM_012219
MRAS	muscle RAS oncogene homolog (MRAS), mRNA	NM_012219	Hs.527021	NM_012219
MRC2	mannose receptor, C type 2 (MRC2), mRNA	NM_006039	Hs.7835	NM_006039
MS4A4A	membrane-spanning 4-domains, subfamily A, member 4 (MS4A4A), transcript variant 1, mRNA	NM_024021	Hs.325960	BX641064
MSRB2	methionine sulfoxide reductase B2 (MSRB2), mRNA	NM_012228	Hs.461420	NM_012228
MTCP1	mature T-cell proliferation 1 (MTCP1), nuclear gene encoding mitochondrial protein, transcript variant B1, mRNA	NM_001018025	Hs.6917	NM_014221
MUC20	mucin 20, cell surface associated (MUC20), mRNA	NM_152673	Hs.599259	NM_00109851
MYADM	myeloid-associated differentiation marker (MYADM), transcript variant 1, mRNA	NM_001020818	Hs.380906	NM_00102081
MYBPC3	myosin binding protein C, cardiac (MYBPC3), mRNA	NM_000256	Hs.524906	BC142685
MYH11	myosin, heavy chain 11, smooth muscle (MYH11), transcript variant SM1B, mRNA	NM_001040114	Hs.460109	NM_00104011
MYH11	myosin, heavy chain 11, smooth muscle (MYH11), transcript variant SM2B, mRNA	NM_001040113	Hs.460109	NM_00104011
MYH7	myosin, heavy chain 7, cardiac muscle, beta (MYH7), mRNA	NM_000257	Hs.278432	NM_000257
MYH7B	myosin, heavy chain 7B, cardiac muscle, beta (MYH7B), mRNA	NM_020884	Hs.414122	BC151242
MYH8	myosin, heavy chain 8, skeletal muscle, perinatal (MYH8), mRNA	NM_002472	Hs.440895	NM_017534
MYL3	myosin, light chain 3, alkali; ventricular, skeletal, slow (MYL3), mRNA	NM_000258	Hs.517939	BC009790
MYL3	myosin, light chain 3, alkali; ventricular, skeletal, slow (MYL3), mRNA	NM_000258	Hs.517939	BC009790
MYL4	myosin, light chain 4, alkali; atrial, embryonic (MYL4), transcript variant 2, mRNA	NM_002476	Hs.463300	BM919855

MYL7	myosin, light chain 7, regulatory (MYL7), mRNA	NM_021223	Hs.75636	BI836837
MYL9	myosin, light chain 9, regulatory (MYL9), transcript variant 2, mRNA	NM_181526	Hs.504687	BM473095
MYLK	myosin, light chain kinase (MYLK), transcript variant 1, mRNA	NM_053025	Hs.556600	NM_053025
MYLK	myosin, light chain kinase (MYLK), transcript variant 1, mRNA	NM_053025	Hs.556600	NM_053025
MYLPF	fast skeletal myosin light chain 2 (MYLPF), mRNA	NM_013292	Hs.50889	BF575830
MYO15B	cDNA FLJ90199 fis, clone MAMMA1001609.	AK074680	Hs.390817	AF418290
MYO18A	myosin XVIIIA (MYO18A), transcript variant 1, mRNA	NM_078471	Hs.699630	D86970
MYO18B	myosin XVIIIB (MYO18B), mRNA	NM_032608	Hs.417959	NM_032608
MYOCD	myocardin (MYOCD), mRNA	NM_153604	Hs.567641	AK128608
MYOM1	myomesin 1 (skelemin) 185kDa (MYOM1), mRNA	NM_003803	Hs.464469	NM_003803
MYOM1	myomesin 1 (skelemin) 185kDa (MYOM1), mRNA	NM_003803	Hs.464469	NM_003803
MYOM3	myomesin family, member 3 (MYOM3), mRNA	NM_152372	Hs.523413	NM_152372
MYOT	myotilin (MYOT), mRNA	NM_006790	Hs.84665	AF133820
MYOZ2	myozenin 2 (MYOZ2), mRNA	NM_016599	Hs.381047	NM_016599
MYOZ2	myozenin 2 (MYOZ2), mRNA	NM_016599	Hs.381047	NM_016599
MYPN	myopalladin (MYPN), mRNA	NM_032578	Hs.55205	NM_032578
NANOS1	nanos homolog 1 (Drosophila) (NANOS1), transcript variant 1, mRNA	NM_199461	Hs.591918	NM_199461
NCAM1	neural cell adhesion molecule 1 (NCAM1), transcript variant 3, mRNA	NM_001076682	Hs.503878	NM_181351
NCAM1	neural cell adhesion molecule 1 (NCAM1), transcript variant 3, mRNA	NM_001076682	Hs.503878	NM_181351
NCOA1	nuclear receptor coactivator 1 (NCOA1), transcript variant 3, mRNA	NM_147233	Hs.699183	NM_147223
NDFIP1	Nedd4 family interacting protein 1 (NDFIP1), mRNA	NM_030571	Hs.9788	AK124884
NDFIP2	Nedd4 family interacting protein 2 (NDFIP2), mRNA	NM_019080	Hs.525093	AB032991
NDFIP2	Nedd4 family interacting protein 2 (NDFIP2), mRNA	NM_019080	Hs.525093	AB032991
NDRG2	NDRG family member 2 (NDRG2), transcript variant 1, mRNA	NM_201535	Hs.525205	AK096999
NDUFA12	NADH dehydrogenase (ubiquinone) 1 alpha subcomplex, 12 (NDUFA12), mRNA	NM_018838	Hs.506374	BM543134
NDUFA6	NADH dehydrogenase (ubiquinone) 1 alpha subcomplex, 6, 14kDa (NDUFA6), nuclear gene encoding mitochondrial protein, mRNA	NM_002490	Hs.274416	NM_002490
NDUFB2	NADH dehydrogenase (ubiquinone) 1 beta subcomplex, 2, 8kDa (NDUFB2), nuclear gene encoding mitochondrial protein, mRNA	NM_004546	Hs.655788	CR610711
NDUFB9	NADH dehydrogenase (ubiquinone) 1 beta subcomplex, 9, 22kDa (NDUFB9), mRNA	NM_005005	Hs.15977	BU180150
NDUFB9	NADH dehydrogenase (ubiquinone) 1 beta subcomplex, 9, 22kDa (NDUFB9), mRNA	NM_005005	Hs.15977	BU180150
NDUFS1	NADH dehydrogenase (ubiquinone) Fe-S protein 1, 75kDa (NADH-coenzyme Q reductase) (NDUFS1), nuclear gene encoding mitochondrial protein, mRNA	NM_005006	Hs.471207	BC030833
NEBL	nebulette (NEBL), transcript variant 1, mRNA	NM_006393	Hs.5025	Y16241
NENF	neuron derived neurotrophic factor (NENF), mRNA	NM_013349	Hs.461787	BU502624
NEURL2	neuralized homolog 2 (Drosophila) (NEURL2), mRNA	NM_080749	Hs.517094	NM_080749
NEXN	nexilin (F actin binding protein) (NEXN), mRNA	NM_144573	Hs.632387	NM_144573
NFKBIZ	nuclear factor of kappa light polypeptide gene enhancer in B-cells inhibitor, zeta (NFKBIZ), transcript variant 1, mRNA	NM_031419	Hs.319171	NM_031419
NFYC	nuclear transcription factor Y, gamma (NFYC), mRNA	NM_014223	Hs.233458	AK055329
NGFR	nerve growth factor receptor (TNFR superfamily, member 16) (NGFR), mRNA	NM_002507	Hs.415768	BC050309
NHP2L1	NHP2 non-histone chromosome protein 2-like 1 (S. cerevisiae) (NHP2L1), transcript variant 1, mRNA	NM_005008	Hs.182255	AK124020
NIPSNAP3B	nipsnap homolog 3B (C. elegans) (NIPSNAP3B), mRNA	NM_018376	Hs.567532	BX647652
NKX2-5	NK2 transcription factor related, locus 5 (Drosophila) (NKX2-5), mRNA	NM_004387	Hs.54473	BC025711
NOV	nephroblastoma overexpressed gene (NOV), mRNA	NM_002514	Hs.235935	NM_002514
NOV	nephroblastoma overexpressed gene (NOV), mRNA	NM_002514	Hs.235935	NM_002514
NOXA1	NADPH oxidase activator 1 (NOXA1), mRNA	NM_006647	Hs.495554	BC110840
NP102468	GB|M27751.1|AAA58913.1 immunoglobulin kappa-chain A14 V-region precursor	NP102468	Unknown	
NPC2	Niemann-Pick disease, type C2 (NPC2), mRNA	NM_006432	Hs.433222	BQ896617
NPPA	natriuretic peptide precursor A (NPPA), mRNA	NM_006172	Hs.75640	BI832557
NPPB	natriuretic peptide precursor B (NPPB), mRNA	NM_002521	Hs.219140	BI833353
NPW	Neuropeptide W precursor (Preproprotein L8) (hPPL8) .	ENST00000329	Unknown	
NR4A3	nuclear receptor subfamily 4, group A, member 3 (NR4A3), transcript variant 2, mRNA	NM_173198	Hs.279522	NM_173198
NUDT16L1	nudix (nucleoside diphosphate linked moiety X)-type motif 16-like 1 (NUDT16L1), mRNA	NM_032349	Hs.592084	BQ679635

NUDT9P1	nudix (nucleoside diphosphate linked moiety X)-type motif 9 pseudogene 1 (NUDT9P1) on chromosome 10	NR_002779	Unknown	
NUPR1	nuclear protein 1 (NUPR1), transcript variant 1, mRNA	NM_001042483	Hs.513463	BG284742
OBSL1	obscurin-like 1, mRNA (cDNA clone IMAGE:4413820), complete cds.	BC061909	Hs.526594	EF063638
OCLM	oculomedin (OCLM), mRNA	NM_022375	Hs.679230	BC069096
OGN	osteoglycin (osteoinductive factor, mimecan) (OGN), transcript variant 1, mRNA	NM_033014	Hs.109439	NM_033014
OLFML2B	olfactomedin-like 2B (OLFML2B), mRNA	NM_015441	Hs.507515	BC067274
OLFML2B	olfactomedin-like 2B (OLFML2B), mRNA	NM_015441	Hs.507515	BC067274
OR11H12	olfactory receptor, family 11, subfamily H, member 12 (OR11H12), mRNA	NM_001013354	Hs.534880	NM_00101335[illegible]
OR51E2	olfactory receptor, family 51, subfamily E, member 2 (OR51E2), mRNA	NM_030774	Hs.501758	NM_030774
OSTM1	osteopetrosis associated transmembrane protein 1 (OSTM1), mRNA	NM_014028	Hs.226780	NM_014028
OXCT1	3-oxoacid CoA transferase 1 (OXCT1), nuclear gene encoding mitochondrial protein, mRNA	NM_000436	Hs.278277	NM_000436
OXT	oxytocin, prepro- (neurophysin I) (OXT), mRNA	NM_000915	Hs.113216	NM_000915
OXTR	oxytocin receptor (OXTR), mRNA	NM_000916	Hs.2820	NM_000916
P2RX1	purinergic receptor P2X, ligand-gated ion channel, 1 (P2RX1), mRNA	NM_002558	Hs.41735	BC027949
PA2G4	cDNA FLJ31839 fis, clone NT2RP7000086.	AK056401	Hs.659905	AK056401
PALLD	palladin, cytoskeletal associated protein (PALLD), mRNA	NM_016081	Hs.151220	NM_016081
PALM2	paralemmin 2 (PALM2), transcript variant 1, mRNA	NM_053016	Hs.591908	NM_053016
PAPPA	pregnancy-associated plasma protein A, pappalysin 1 (PAPPA), mRNA	NM_002581	Hs.694735	NM_002581
PAPPA	pregnancy-associated plasma protein A, pappalysin 1 (PAPPA), mRNA	NM_002581	Hs.694735	NM_002581
PARP15	poly (ADP-ribose) polymerase family, member 15 (PARP15), mRNA	NM_152615	Hs.120250	BX647656
PCBP3	poly(rC) binding protein 3 (PCBP3), mRNA	NM_020528	Hs.474049	AK126016
PCDHGA8	protocadherin gamma subfamily A, 8 (PCDHGA8), transcript variant 1, mRNA	NM_032088	Hs.368160	AB002325
PDE4DIP	phosphodiesterase 4D interacting protein (myomegalin) (PDE4DIP), transcript variant 3, mRNA	NM_022359	Hs.654651	NM_014644
PDE4DIP	mRNA, similar to rat myomegalin, complete cds.	AB042555	Hs.613082	AB042555
PDE4DIP	phosphodiesterase 4D interacting protein (myomegalin) (PDE4DIP), transcript variant 5, mRNA	NM_001002811	Hs.654651	NM_014644
PDE4DIP	phosphodiesterase 4D interacting protein (myomegalin) (PDE4DIP), transcript variant 1, mRNA	NM_014644	Hs.654651	NM_014644
PDE6A	phosphodiesterase 6A, cGMP-specific, rod, alpha (PDE6A), mRNA	NM_000440	Hs.567314	M26061
PDE6B	phosphodiesterase 6B, cGMP-specific, rod, beta (congenital stationary night blindness 3, autosomal dominant) (PDE6B), mRNA	NM_000283	Hs.654544	NM_000283
PDK2	pyruvate dehydrogenase kinase, isozyme 2 (PDK2), mRNA	NM_002611	Hs.256667	AK055119
PDLIM3	PDZ and LIM domain 3 (PDLIM3), mRNA	NM_014476	Hs.85862	BX647263
PDLIM5	PDZ and LIM domain 5 (PDLIM5), transcript variant 4, mRNA	NM_001011515	Hs.480311	NM_006457
PDLIM5	PDZ and LIM domain 5 (PDLIM5), transcript variant 1, mRNA	NM_006457	Hs.480311	NM_006457
PDRG1	p53 and DNA damage regulated 1 (PDRG1), mRNA	NM_030815	Hs.435755	BM913118
PDXK	pyridoxal (pyridoxine, vitamin B6) kinase (PDXK), mRNA	NM_003681	Hs.284491	NM_003681
PDXK	pyridoxal (pyridoxine, vitamin B6) kinase (PDXK), mRNA	NM_003681	Hs.284491	NM_003681
PELO	pelota homolog (Drosophila) (PELO), mRNA	NM_015946	Hs.696076	BX648284
PENK	proenkephalin (PENK), mRNA	NM_006211	Hs.339831	NM_006211
PFKM	phosphofructokinase, muscle (PFKM), mRNA	NM_000289	Hs.75160	AK126020
PGAM2	phosphoglycerate mutase 2 (muscle) (PGAM2), mRNA	NM_000290	Hs.632642	BM553200
PGK1	phosphoglycerate kinase 1 (PGK1), mRNA	NM_000291	Hs.78771	NM_000291
PGK1	phosphoglycerate kinase 1 (PGK1), mRNA	NM_000291	Hs.78771	NM_000291
PGK1	phosphoglycerate kinase 1 (PGK1), mRNA	NM_000291	Hs.78771	NM_000291
PGK1	phosphoglycerate kinase 1 (PGK1), mRNA	NM_000291	Hs.78771	NM_000291
PGK1	phosphoglycerate kinase 1 (PGK1), mRNA	NM_000291	Hs.78771	NM_000291
PGK1	phosphoglycerate kinase 1 (PGK1), mRNA	NM_000291	Hs.78771	NM_000291
PGK1	phosphoglycerate kinase 1 (PGK1), mRNA	NM_000291	Hs.78771	NM_000291
PGK1	phosphoglycerate kinase 1 (PGK1), mRNA	NM_000291	Hs.78771	NM_000291
PGK1	phosphoglycerate kinase 1 (PGK1), mRNA	NM_000291	Hs.78771	NM_000291
PGK1	phosphoglycerate kinase 1 (PGK1), mRNA	NM_000291	Hs.78771	NM_000291
PGM1	phosphoglucomutase 1 (PGM1), mRNA	NM_002633	Hs.1869	NM_002633
PGM3	phosphoglucomutase 3 (PGM3), mRNA	NM_015599	Hs.700611	AL117443

PGM5	phosphoglucomutase 5 (PGM5), mRNA	NM_021965	Hs.307835	AL137698
PGM5	phosphoglucomutase 5 (PGM5), mRNA	NM_021965	Hs.307835	AL137698
PGM5	phosphoglucomutase 5 (PGM5), mRNA	NM_021965	Hs.307835	AL137698
PGPEP1	pyroglutamyl-peptidase I (PGPEP1), mRNA	NM_017712	Hs.131776	NM_017712
PGRMC2	progesterone receptor membrane component 2 (PGRMC2), mRNA	NM_006320	Hs.507910	AK094949
PH-4	hypoxia-inducible factor prolyl 4-hydroxylase (PH-4), transcript variant 2, mRNA	NM_017732	Unknown	
PIK3R1	phosphoinositide-3-kinase, regulatory subunit 1 (p85 alpha) (PIK3R1), transcript variant 1, mRNA	NM_181523	Hs.132225	NM_181523
PIK3R2	phosphoinositide-3-kinase, regulatory subunit 2 (p85 beta) (PIK3R2), mRNA	NM_005027	Hs.371344	NM_005027
PINK1	PTEN induced putative kinase 1 (PINK1), nuclear gene encoding mitochondrial protein, mRNA	NM_032409	Hs.389171	AB053323
PKP2	H.sapiens mRNA for plakophilin 2a and b.	X97675	Hs.164384	NM_004572
PKP2	plakophilin 2 (PKP2), transcript variant 2b, mRNA	NM_004572	Hs.164384	NM_004572
PLA2G5	phospholipase A2, group V (PLA2G5), mRNA	NM_000929	Hs.319438	NM_000929
PLCD1	phospholipase C, delta 1 (PLCD1), mRNA	NM_006225	Hs.80776	BX647927
PLCXD3	phosphatidylinositol-specific phospholipase C, X domain containing 3 (PLCXD3), mRNA	NM_001005473	Hs.145404	NM_001005473
PLEKHA4	pleckstrin homology domain containing, family A (phosphoinositide binding specific) member 4 (PLEKHA4), mRNA	NM_020904	Hs.9469	AB208908
PLEKHA4	pleckstrin homology domain containing, family A (phosphoinositide binding specific) member 4 (PLEKHA4), mRNA	NM_020904	Hs.9469	AB208908
PLEKHA7	pleckstrin homology domain containing, family A member 7 (PLEKHA7), mRNA	NM_175058	Hs.12332	NM_175058
PLEKHH3	pleckstrin homology domain containing, family H (with MyTH4 domain) member 3 (PLEKHH3), mRNA	NM_024927	Hs.632251	AB208939
PLN	phospholamban (PLN), mRNA	NM_002667	Hs.170839	AK129844
PLN	phospholamban (PLN), mRNA	NM_002667	Hs.170839	AK129844
PLXDC1	plexin domain containing 1 (PLXDC1), mRNA	NM_020405	Hs.125036	NM_020405
PLXNB2	Human mRNA for KIAA0315 gene, partial cds.	AB002313	Hs.3989	NM_012401
PLXNB2	Human mRNA for KIAA0315 gene, partial cds.	AB002313	Hs.3989	NM_012401
PLXNC1	mRNA for plexin C1 variant protein.	AB208934	Hs.584845	AB208934
PLXNC1	plexin C1 (PLXNC1), mRNA	NM_005761	Hs.584845	AB208934
PMM1	phosphomannomutase 1 (PMM1), mRNA	NM_002676	Hs.75835	AK094811
PNPLA8	patatin-like phospholipase domain containing 8 (PNPLA8), mRNA	NM_015723	Hs.617340	AL834147
POMZP3	POM (POM121 homolog, rat) and ZP3 fusion (POMZP3), transcript variant 2, mRNA	NM_152992	Hs.488877	CR603033
POPDC2	popeye domain containing 2 (POPDC2), mRNA	NM_022135	Hs.656031	AK124602
POPDC2	popeye domain containing 2 (POPDC2), mRNA	NM_022135	Hs.656031	AK124602
POPDC3	popeye domain containing 3 (POPDC3), mRNA	NM_022361	Hs.458336	NM_022361
PPAPDC1B	phosphatidic acid phosphatase type 2 domain containing 1B, mRNA (cDNA clone MGC:32924 IMAGE:5267610), complete cds.	BC033025	Hs.567619	BC033025
PPAPDC3	phosphatidic acid phosphatase type 2 domain containing 3 (PPAPDC3), mRNA	NM_032728	Hs.134292	AK075207
PPARA	peroxisome proliferator-activated receptor alpha (PPARA), transcript variant 5, mRNA	NM_005036	Hs.103110	NM_005036
PPARA	peroxisome proliferator-activated receptor alpha (PPARA), transcript variant 5, mRNA	NM_005036	Hs.103110	NM_005036
PPIC	peptidylprolyl isomerase C (cyclophilin C) (PPIC), mRNA	NM_000943	Hs.110364	BG761203
PPM1K	protein phosphatase 1K (PP2C domain containing) (PPM1K), mRNA	NM_152542	Hs.291000	BC041350
PPME1	protein phosphatase methylesterase 1 (PPME1), mRNA	NM_016147	Hs.503251	AK095005
PPP1R12B	protein phosphatase 1, regulatory (inhibitor) subunit 12B (PPP1R12B), transcript variant 2, mRNA	NM_032105	Hs.444403	NM_032105
PPP1R14A	protein phosphatase 1, regulatory (inhibitor) subunit 14A (PPP1R14A), mRNA	NM_033256	Hs.631569	BM811652
PPP1R16A	protein phosphatase 1, regulatory (inhibitor) subunit 16A (PPP1R16A), mRNA	NM_032902	Hs.521937	AK090471
PPP1R3C	protein phosphatase 1, regulatory (inhibitor) subunit 3C (PPP1R3C), mRNA	NM_005398	Hs.303090	BX537399
PPP2R5A	protein phosphatase 2, regulatory subunit B (B56), alpha isoform (PPP2R5A), mRNA	NM_006243	Hs.497684	NM_006243
PRELP	proline/arginine-rich end leucine-rich repeat protein (PRELP), transcript variant 1, mRNA	NM_002725	Hs.632481	NM_002725
PRG4	proteoglycan 4 (PRG4), mRNA	NM_005807	Hs.647723	NM_005807
PRKAA2	protein kinase, AMP-activated, alpha 2 catalytic subunit (PRKAA2), mRNA	NM_006252	Hs.591439	NM_006252
PRKAB2	protein kinase, AMP-activated, beta 2 non-catalytic subunit (PRKAB2), mRNA	NM_005399	Hs.50732	NM_005399
PROX1	prospero-related homeobox 1 (PROX1), mRNA	NM_002763	Hs.585369	NM_002763
PRRX1	paired related homeobox 1 (PRRX1), transcript variant pmx-1a, mRNA	NM_006902	Hs.283416	NM_006902
PSD3	pleckstrin and Sec7 domain containing 3 (PSD3), transcript variant 1, mRNA	NM_015310	Hs.434255	NM_015310
PSEN1	presenilin 1 (Alzheimer disease 3) (PSEN1), mRNA	NM_000021	Hs.592324	AK122722

PSEN1	presenilin 1 (Alzheimer disease 3) (PSEN1), mRNA	NM_000021	Hs.592324	AK122722
PSEN1	presenilin 1 (Alzheimer disease 3) (PSEN1), mRNA	NM_000021	Hs.592324	AK122722
PSEN1	presenilin 1 (Alzheimer disease 3) (PSEN1), mRNA	NM_000021	Hs.592324	AK122722
PSEN1	presenilin 1 (Alzheimer disease 3) (PSEN1), mRNA	NM_000021	Hs.592324	AK122722
PSEN1	presenilin 1 (Alzheimer disease 3) (PSEN1), mRNA	NM_000021	Hs.592324	AK122722
PSEN1	presenilin 1 (Alzheimer disease 3) (PSEN1), mRNA	NM_000021	Hs.592324	AK122722
PSG6	pregnancy specific beta-1-glycoprotein 6 (PSG6), transcript variant 1, mRNA	NM_002782	Hs.654414	BC020652
PTGER2	prostaglandin E receptor 2 (subtype EP2), 53kDa (PTGER2), mRNA	NM_000956	Hs.2090	NM_000956
PTGES	prostaglandin E synthase (PTGES), mRNA	NM_004878	Hs.146688	AK127663
PTHB1	parathyroid hormone-responsive B1 (PTHB1), transcript variant 2, mRNA	NM_198428	Hs.372360	NM_198428
PTP4A1	protein tyrosine phosphatase type IVA, member 1 (PTP4A1), mRNA	NM_003463	Hs.227777	NM_003463
PTP4A3	protein tyrosine phosphatase type IVA, member 3 (PTP4A3), transcript variant 1, mRNA	NM_032611	Hs.43666	AK128380
PTPLA	protein tyrosine phosphatase-like (proline instead of catalytic arginine), member A (PTPLA), mRNA	NM_014241	Hs.114062	AY455942
PTPN3	protein tyrosine phosphatase, non-receptor type 3 (PTPN3), mRNA	NM_002829	Hs.436429	BX648253
PTPRC	protein tyrosine phosphatase, receptor type, C (PTPRC), transcript variant 1, mRNA	NM_002838	Hs.654514	NM_002838
PTPRH	protein tyrosine phosphatase, receptor type, H (PTPRH), mRNA	NM_002842	Hs.179770	D15049
PTPRR	protein tyrosine phosphatase, receptor type, R (PTPRR), transcript variant 1, mRNA	NM_002849	Hs.506076	NM_002849
PXDNL	peroxidasin homolog-like (Drosophila) (PXDNL), mRNA	NM_144651	Hs.444882	AY877349
RAB21	RAB21, member RAS oncogene family (RAB21), mRNA	NM_014999	Hs.524590	BC009109
RAB27B	RAB27B, member RAS oncogene family (RAB27B), mRNA	NM_004163	Hs.25318	AF131784
RAB31	RAB31, member RAS oncogene family (RAB31), mRNA	NM_006868	Hs.99528	NM_006868
RAB6B	RAB6B, member RAS oncogene family (RAB6B), mRNA	NM_016577	Hs.12152	NM_016577
RAB6B	RAB6B, member RAS oncogene family (RAB6B), mRNA	NM_016577	Hs.12152	NM_016577
RAB6IP1	RAB6 interacting protein 1 (RAB6IP1), mRNA	NM_015213	Hs.501857	NM_015213
RAB7B	RAB7B, member RAS oncogene family (RAB7B), mRNA	NM_177403	Hs.534612	NM_177403
RAB7B	RAB7B, member RAS oncogene family (RAB7B), mRNA	NM_177403	Hs.534612	NM_177403
RAB9B	Ras-related protein Rab-9B (Rab-9L) (RAB9-like protein).	ENST00000243	Unknown	
RAB9B	RAB9B, member RAS oncogene family (RAB9B), mRNA	NM_016370	Hs.522736	BC041336
RAMP1	receptor (G protein-coupled) activity modifying protein 1 (RAMP1), mRNA	NM_005855	Hs.471783	BG036385
RAP2A	RAP2A, member of RAS oncogene family (RAP2A), mRNA	NM_021033	Hs.508480	BC041333
RARS	arginyl-tRNA synthetase (RARS), mRNA	NM_002887	Hs.654907	NM_002887
RASAL2	RAS protein activator like 2 (RASAL2), transcript variant 2, mRNA	NM_170692	Hs.656823	NM_170692
RASD1	RAS, dexamethasone-induced 1 (RASD1), mRNA	NM_016084	Hs.25829	BC042688
RASD2	RASD family, member 2 (RASD2), mRNA	NM_014310	Hs.474711	BC013419
RBM24	RNA binding motif protein 24 (RBM24), mRNA	NM_153020	Hs.519904	AL832199
RBM24	RNA binding motif protein 24 (RBM24), mRNA	NM_153020	Hs.519904	AL832199
RBM38	RNA binding motif protein 38 (RBM38), transcript variant 1, mRNA	NM_017495	Hs.236361	CR627021
RBMS1	RNA binding motif, single stranded interacting protein 1 (RBMS1), transcript variant 1, mRNA	NM_016836	Hs.470412	NM_016839
RDH5	retinol dehydrogenase 5 (11-cis/9-cis) (RDH5), mRNA	NM_002905	Hs.632719	BC028298
REEP2	receptor accessory protein 2 (REEP2), mRNA	NM_016606	Hs.416090	AK223611
REXO1L1	REX1, RNA exonuclease 1 homolog (S. cerevisiae)-like 1 (REXO1L1), mRNA	NM_172239	Hs.373854	NM_172239
RHCG	Rh family, C glycoprotein (RHCG), mRNA	NM_016321	Hs.459284	BC030965
RHOQ	ras homolog gene family, member Q (RHOQ), mRNA	NM_012249	Hs.695931	NM_012249
RNF8	ring finger protein 8 (RNF8), transcript variant 1, mRNA	NM_003958	Hs.485278	NM_003958
RP13-401N8.2	hypothetical gene supported by BC042812, mRNA (cDNA clone IMAGE:5269806), complete cds.	BC042812	Unknown	
RPH3A	mRNA for KIAA0985 protein, partial cds.	AB023202	Hs.21239	BX647281
RPL28	ribosomal protein L28 (RPL28), mRNA	NM_000991	Hs.652114	NM_000991
RPSA	cDNA clone IMAGE:3845335, **** WARNING: chimeric clone ****.	BC010054	Unknown	
RRAD	Ras-related associated with diabetes (RRAD), mRNA	NM_004165	Hs.1027	BC057815
RRAD	Ras-related associated with diabetes (RRAD), mRNA	NM_004165	Hs.1027	BC057815
RUNDC1	RUN domain containing 1 (RUNDC1), mRNA	NM_173079	Hs.632255	BC039247
RYR3	ryanodine receptor 3 (RYR3), mRNA	NM_001036	Hs.369250	AJ001515

S100A4	S100 calcium binding protein A4 (S100A4), transcript variant 1, mRNA	NM_002961	Hs.654444	BQ945534
SAA1	serum amyloid A1 (SAA1), transcript variant 1, mRNA	NM_000331	Hs.632144	BG564669
SAA2	serum amyloid A2 (SAA2), mRNA	NM_030754	Hs.654517	BC058008
SBDS	Shwachman-Bodian-Diamond syndrome (SBDS), mRNA	NM_016038	Hs.110445	AY169963
SCAMP1	secretory carrier membrane protein 1 (SCAMP1), mRNA	NM_004866	Hs.482587	NM_004866
SCAND2	SCAN domain containing 2, mRNA (cDNA clone IMAGE:6503167), complete cds.	BC063836	Unknown	
SCN2A2	sodium channel, voltage-gated, type II, alpha 2 (SCN2A2), transcript variant 1, mRNA	NM_021007	Hs.93485	NM_021007
SCN2B	sodium channel, voltage-gated, type II, beta (SCN2B), mRNA	NM_004588	Hs.129783	NM_004588
SCN2B	sodium channel, voltage-gated, type II, beta (SCN2B), mRNA	NM_004588	Hs.129783	NM_004588
SCN3B	sodium channel, voltage-gated, type III, beta (SCN3B), transcript variant 1, mRNA	NM_018400	Hs.4865	NM_018400
SCNN1D	sodium channel, nonvoltage-gated 1, delta (SCNN1D), mRNA	NM_002978	Hs.512681	AK127357
SDC2	syndecan 2 (heparan sulfate proteoglycan 1, cell surface-associated, fibroglycan) (SDC2), mRNA	NM_002998	Hs.1501	BC030133
SDC3	mRNA for KIAA0468 protein, partial cds.	AB007937	Hs.158287	AB007937
SEC61A2	Sec61 alpha 2 subunit (S. cerevisiae) (SEC61A2), mRNA	NM_018144	Hs.112955	AK057532
SELK	selenoprotein K (SELK), mRNA	NM_021237	Hs.58471	BG259147
SEMA3B	sema domain, immunoglobulin domain (Ig), short basic domain, secreted, (semaphorin) 3B (SEMA3B), transcript variant 1, mRNA	NM_004636	Hs.82222	AB209322
SEMA3B	sema domain, immunoglobulin domain (Ig), short basic domain, secreted, (semaphorin) 3B (SEMA3B), transcript variant 1, mRNA	NM_004636	Hs.82222	AB209322
SEMA5A	sema domain, seven thrombospondin repeats (type 1 and type 1-like), transmembrane domain (TM) and short cytoplasmic domain, (semaphorin) 5A (SEMA5A), mRNA	NM_003966	Hs.27621	NM_003966
SEPT5	septin 5 (SEPT5), mRNA	NM_002688	Hs.283743	NM_002688
SEPT5	septin 5 (SEPT5), mRNA	NM_002688	Hs.283743	NM_002688
SERPINI1	serpin peptidase inhibitor, clade I (neuroserpin), member 1 (SERPINI1), mRNA	NM_005025	Hs.478153	CR627434
SESN2	sestrin 2 (SESN2), mRNA	NM_031459	Hs.469543	AY123223
SFRP4	secreted frizzled-related protein 4 (SFRP4), mRNA	NM_003014	Hs.658169	AF026692
SGCA	sarcoglycan, alpha (50kDa dystrophin-associated glycoprotein) (SGCA), mRNA	NM_000023	Hs.463412	BM544123
SGCD	sarcoglycan, delta (35kDa dystrophin-associated glycoprotein) (SGCD), transcript variant 1, mRNA	NM_000337	Hs.591727	NM_000337
SGCD	sarcoglycan, delta (35kDa dystrophin-associated glycoprotein) (SGCD), transcript variant 2, mRNA	NM_172244	Hs.591727	NM_000337
SH2D4A	SH2 domain containing 4A (SH2D4A), mRNA	NM_022071	Hs.303208	AK024799
SH3BGR	SH3 domain binding glutamic acid-rich protein (SH3BGR), transcript variant 1, mRNA	NM_007341	Hs.473847	NM_007341
SH3GL1	SH3-domain GRB2-like 1 (SH3GL1), mRNA	NM_003025	Hs.97616	BC098565
SH3RF2	SH3 domain containing ring finger 2 (SH3RF2), mRNA	NM_152550	Hs.443728	AL833297
SH3TC2	SH3 domain and tetratricopeptide repeats 2 (SH3TC2), mRNA	NM_024577	Hs.483784	NM_024577
SHC4	SHC (Src homology 2 domain containing) family, member 4 (SHC4), mRNA	NM_203349	Hs.642615	NM_203349
SIRPA	signal-regulatory protein alpha (SIRPA), transcript variant 1, mRNA	NM_001040022	Hs.581021	NM_001040022
SIRT4	sirtuin (silent mating type information regulation 2 homolog) 4 (S. cerevisiae) (SIRT4), mRNA	NM_012240	Hs.50861	BC034736
SKIP	SPHK1 (sphingosine kinase type 1) interacting protein (SKIP), mRNA	NM_030623	Hs.436306	CR749494
SLC12A7	solute carrier family 12 (potassium/chloride transporters), member 7 (SLC12A7), mRNA	NM_006598	Hs.172613	NM_006598
SLC16A7	solute carrier family 16, member 7 (monocarboxylic acid transporter 2) (SLC16A7), mRNA	NM_004731	Hs.439643	NM_004731
SLC20A2	solute carrier family 20 (phosphate transporter), member 2 (SLC20A2), mRNA	NM_006749	Hs.653173	NM_006749
SLC22A17	solute carrier family 22 (organic cation transporter), member 17 (SLC22A17), transcript variant 2, mRNA	NM_016609	Hs.373498	BX161416
SLC22A18	solute carrier family 22 (organic cation transporter), member 18 (SLC22A18), transcript variant 2, mRNA	NM_183233	Hs.50868	AF030302
SLC25A3	solute carrier family 25 (mitochondrial carrier; phosphate carrier), member 3 (SLC25A3), nuclear gene encoding mitochondrial protein, transcript variant 3, mRNA	NM_213611	Hs.290404	AK057575
SLC25A4	solute carrier family 25 (mitochondrial carrier; adenine nucleotide translocator), member 4 (SLC25A4), nuclear gene encoding mitochondrial protein, mRNA	NM_001151	Hs.246506	AB209764
SLC25A41	solute carrier family 25, member 41 (SLC25A41), mRNA	NM_173637	Hs.375135	AK097761
SLC26A9	solute carrier family 26, member 9 (SLC26A9), transcript variant 1, mRNA	NM_052934	Hs.164073	AF331525
SLC27A6	solute carrier family 27 (fatty acid transporter), member 6 (SLC27A6), transcript variant 2, mRNA	NM_001017372	Hs.49765	BC041945
SLC28A3	solute carrier family 28 (sodium-coupled nucleoside transporter), member 3 (SLC28A3), mRNA	NM_022127	Hs.591877	AF305210
SLC28A3	concentrative Na+-nucleoside cotransporter	ENST00000376	Unknown	
SLC30A1	solute carrier family 30 (zinc transporter), member 1 (SLC30A1), mRNA	NM_021194	Hs.519469	BX647120
SLC30A3	solute carrier family 30 (zinc transporter), member 3 (SLC30A3), mRNA	NM_003459	Hs.467981	AK127300
SLC39A4	solute carrier family 39 (zinc transporter), member 4 (SLC39A4), transcript variant 1, mRNA	NM_017767	Hs.521934	AK056900

SLC41A1	solute carrier family 41, member 1 (SLC41A1), mRNA	NM_173854	Hs.20274	BX648979
SLC4A3	solute carrier family 4, anion exchanger, member 3 (SLC4A3), transcript variant 1, mRNA	NM_005070	Hs.1176	AY142112
SLC4A4	solute carrier family 4, sodium bicarbonate cotransporter, member 4 (SLC4A4), mRNA	NM_003759	Hs.5462	NM_00109848
SLC6A20	solute carrier family 6 (proline IMINO transporter), member 20 (SLC6A20), transcript variant 1, mRNA	NM_020208	Hs.413095	AJ276207
SLC6A4	solute carrier family 6 (neurotransmitter transporter, serotonin), member 4 (SLC6A4), mRNA	NM_001045	Hs.591192	NM_001045
SLC6A4	solute carrier family 6 (neurotransmitter transporter, serotonin), member 4 (SLC6A4), mRNA	NM_001045	Hs.591192	NM_001045
SLC6A4	solute carrier family 6 (neurotransmitter transporter, serotonin), member 4 (SLC6A4), mRNA	NM_001045	Hs.591192	NM_001045
SLC6A4	solute carrier family 6 (neurotransmitter transporter, serotonin), member 4 (SLC6A4), mRNA	NM_001045	Hs.591192	NM_001045
SLC6A4	solute carrier family 6 (neurotransmitter transporter, serotonin), member 4 (SLC6A4), mRNA	NM_001045	Hs.591192	NM_001045
SLC6A4	solute carrier family 6 (neurotransmitter transporter, serotonin), member 4 (SLC6A4), mRNA	NM_001045	Hs.591192	NM_001045
SLC6A4	solute carrier family 6 (neurotransmitter transporter, serotonin), member 4 (SLC6A4), mRNA	NM_001045	Hs.591192	NM_001045
SLC6A4	solute carrier family 6 (neurotransmitter transporter, serotonin), member 4 (SLC6A4), mRNA	NM_001045	Hs.591192	NM_001045
SLC6A4	solute carrier family 6 (neurotransmitter transporter, serotonin), member 4 (SLC6A4), mRNA	NM_001045	Hs.591192	NM_001045
SLC6A4	solute carrier family 6 (neurotransmitter transporter, serotonin), member 4 (SLC6A4), mRNA	NM_001045	Hs.591192	NM_001045
SLC8A1	solute carrier family 8 (sodium/calcium exchanger), member 1 (SLC8A1), mRNA	NM_021097	Hs.468274	NM_021097
SLC8A1	solute carrier family 8 (sodium/calcium exchanger), member 1 (SLC8A1), mRNA	NM_021097	Hs.468274	NM_021097
SLCO3A1	PREDICTED: solute carrier organic anion transporter family, member 3A1 (SLCO3A1), mRNA	XM_001132480	Unknown	
SLCO3A1	solute carrier organic anion transporter family, member 3A1 (SLCO3A1), mRNA	NM_013272	Hs.311187	AF205074
SMAP1	stromal membrane-associated protein 1 (SMAP1), transcript variant 1, mRNA	NM_001044305	Hs.485717	NM_00104430
SMAP1	stromal membrane-associated protein 1 (SMAP1), transcript variant 1, mRNA	NM_001044305	Hs.485717	NM_00104430
SMCR7	Smith-Magenis syndrome chromosome region, candidate 7 (SMCR7), transcript variant 1, mRNA	NM_139162	Hs.655555	AK128310
SMPX	small muscle protein, X-linked (SMPX), mRNA	NM_014332	Hs.86492	BF693607
SMYD1	SET and MYND domain containing 1 (SMYD1), mRNA	NM_198274	Hs.516176	NM_198274
SNAP91	synaptosomal-associated protein, 91kDa homolog (mouse) (SNAP91), mRNA	NM_014841	Hs.368046	CR749348
SNF1LK	SNF1-like kinase (SNF1LK), mRNA	NM_173354	Hs.282113	BC038504
SNTA1	syntrophin, alpha 1 (dystrophin-associated protein A1, 59kDa, acidic component) (SNTA1), mRNA	NM_003098	Hs.31121	NM_003098
SNTA1	syntrophin, alpha 1 (dystrophin-associated protein A1, 59kDa, acidic component) (SNTA1), mRNA	NM_003098	Hs.31121	NM_003098
SNTB1	syntrophin, beta 1 (dystrophin-associated protein A1, 59kDa, basic component 1) (SNTB1), mRNA	NM_021021	Hs.655236	AK026095
SOCS3	suppressor of cytokine signaling 3 (SOCS3), mRNA	NM_003955	Hs.527973	NM_003955
SOD1	superoxide dismutase 1, soluble (amyotrophic lateral sclerosis 1 (adult)) (SOD1), mRNA	NM_000454	Hs.443914	BM913065
SOD1	superoxide dismutase 1, soluble (amyotrophic lateral sclerosis 1 (adult)) (SOD1), mRNA	NM_000454	Hs.443914	BM913065
SOD3	superoxide dismutase 3, extracellular (SOD3), mRNA	NM_003102	Hs.2420	BM906283
SORBS2	sorbin and SH3 domain containing 2 (SORBS2), transcript variant 2, mRNA	NM_021069	Hs.655143	NM_021069
SORT1	sortilin 1 (SORT1), mRNA	NM_002959	Hs.485195	NM_002959
SORT1	sortilin 1 (SORT1), mRNA	NM_002959	Hs.485195	NM_002959
SPATA19	spermatogenesis associated 19 (SPATA19), mRNA	NM_174927	Hs.97541	BC058039
SPATA22	spermatogenesis associated 22 (SPATA22), mRNA	NM_032598	Hs.351068	AK057485
SPG3A	spastic paraplegia 3A (autosomal dominant) (SPG3A), transcript variant 2, mRNA	NM_181598	Hs.584905	AF444143
SPIC	Spi-C transcription factor (Spi-1/PU.1 related) (SPIC), mRNA	NM_152323	Hs.577097	AF518404
SPINK4	serine peptidase inhibitor, Kazal type 4 (SPINK4), mRNA	NM_014471	Hs.555934	AA502919
SPOCK2	sparc/osteonectin, cwcv and kazal-like domains proteoglycan (testican) 2 (SPOCK2), mRNA	NM_014767	Hs.523009	NM_014767
SPON2	spondin 2, extracellular matrix protein (SPON2), mRNA	NM_012445	Hs.302963	AK024499
SPPL2A	signal peptide peptidase-like 2A (SPPL2A), mRNA	NM_032802	Hs.401537	BC025740
SPTB	spectrin, beta, erythrocytic (includes spherocytosis, clinical type I) (SPTB), transcript variant 2, mRNA	NM_000347	Hs.417303	NM_00102485
SPTB	spectrin, beta, erythrocytic (includes spherocytosis, clinical type I) (SPTB), transcript variant 1, mRNA	NM_001024858	Hs.417303	NM_00102485
SPTB	spectrin, beta, erythrocytic (includes spherocytosis, clinical type I) (SPTB), transcript variant 1, mRNA	NM_001024858	Hs.417303	NM_00102485
SRF	serum response factor (c-fos serum response element-binding transcription factor) (SRF), mRNA	NM_003131	Hs.520140	AB209128
SSPN	sarcospan (Kras oncogene-associated gene) (SSPN), mRNA	NM_005086	Hs.183428	NM_005086
ST6GALNAC2	ST6 (alpha-N-acetyl-neuraminyl-2,3-beta-galactosyl-1,3)-N-acetylgalactosaminide alpha-2,6-sialyltransferase 2 (ST6GALNAC2), mRNA	NM_006456	Hs.592105	BC038114
ST8SIA5	ST8 alpha-N-acetyl-neuraminide alpha-2,8-sialyltransferase 5 (ST8SIA5), mRNA	NM_013305	Hs.465025	NM_013305
STAM	signal transducing adaptor molecule (SH3 domain and ITAM motif) 1 (STAM), mRNA	NM_003473	Hs.441498	NM_003473
STAT4	signal transducer and activator of transcription 4 (STAT4), mRNA	NM_003151	Hs.80642	NM_003151

STATH	statherin (STATH), transcript variant 1, mRNA	NM_003154	Hs.654495	CB986651
STC2	stanniocalcin 2 (STC2), mRNA	NM_003714	Hs.233160	NM_003714
STK38L	serine/threonine kinase 38 like (STK38L), mRNA	NM_015000	Hs.184523	AB023182
SULT1A1	sulfotransferase family, cytosolic, 1A, phenol-preferring, member 1 (SULT1A1), transcript variant 2, mRNA	NM_177529	Hs.567342	AB209149
SULT1A4	sulfotransferase family, cytosolic, 1A, phenol-preferring, member 4 (SULT1A4), transcript variant 1, mRNA	NM_001017389	Hs.460558	NM_00101738[illegible]
SUPT6H	suppressor of Ty 6 homolog (S. cerevisiae) (SUPT6H), mRNA	NM_003170	Hs.250429	BC150268
SURF1	surfeit 1 (SURF1), nuclear gene encoding mitochondrial protein, mRNA	NM_003172	Hs.512464	BM923055
SV2C	mRNA for KIAA1054 protein, partial cds.	AB028977	Hs.663229	AB028977
SYNGR1	synaptogyrin 1 (SYNGR1), transcript variant 1c, mRNA	NM_145738	Hs.216226	NM_004711
SYNJ2BP	synaptojanin 2 binding protein (SYNJ2BP), mRNA	NM_018373	Hs.443661	AK123967
SYNPO	mRNA; cDNA DKFZp451G172 (from clone DKFZp451G172).	AL831818	Hs.654723	AB028952
SYNPO2L	synaptopodin 2-like (SYNPO2L), mRNA	NM_024875	Hs.645273	AB188489
SYT15	synaptotagmin XV (SYT15), transcript variant b, mRNA	NM_181519	Hs.696346	NM_031912
T66139	yc77b03.s1 Soares infant brain 1NIB cDNA clone IMAGE:21948 3', mRNA sequence	T66139	Hs.633014	T66139
TAGLN	transgelin (TAGLN), transcript variant 1, mRNA	NM_001001522	Hs.632099	AB209555
TAGLN	transgelin (TAGLN), transcript variant 1, mRNA	NM_001001522	Hs.632099	AB209555
TBC1D10A	TBC1 domain family, member 10A (TBC1D10A), mRNA	NM_031937	Hs.655273	AK131086
TBX21	T-box 21 (TBX21), mRNA	NM_013351	Hs.272409	NM_013351
TCAP	titin-cap (telethonin) (TCAP), mRNA	NM_003673	Hs.514146	AK096328
TCEA3	transcription elongation factor A (SII), 3 (TCEA3), mRNA	NM_003196	Hs.446354	NM_003196
TCEA3	transcription elongation factor A (SII), 3 (TCEA3), mRNA	NM_003196	Hs.446354	NM_003196
TDRD9	tudor domain containing 9 (TDRD9), mRNA	NM_153046	Hs.21454	AL833915
TEAD3	TEA domain family member 3 (TEAD3), mRNA	NM_003214	Hs.485205	AK226144
TEAD3	TEA domain family member 3 (TEAD3), mRNA	NM_003214	Hs.485205	AK226144
TES	testis derived transcript (3 LIM domains) (TES), transcript variant 2, mRNA	NM_152829	Hs.592286	NM_015641
TES	testis derived transcript (3 LIM domains) (TES), transcript variant 2, mRNA	NM_152829	Hs.592286	NM_015641
TFRC	transferrin receptor (p90, CD71) (TFRC), mRNA	NM_003234	Hs.529618	BC001188
TFRC	transferrin receptor (p90, CD71) (TFRC), mRNA	NM_003234	Hs.529618	BC001188
TFRC	transferrin receptor (p90, CD71) (TFRC), mRNA	NM_003234	Hs.529618	BC001188
TFRC	transferrin receptor (p90, CD71) (TFRC), mRNA	NM_003234	Hs.529618	BC001188
TFRC	transferrin receptor (p90, CD71) (TFRC), mRNA	NM_003234	Hs.529618	BC001188
TFRC	transferrin receptor (p90, CD71) (TFRC), mRNA	NM_003234	Hs.529618	BC001188
TFRC	transferrin receptor (p90, CD71) (TFRC), mRNA	NM_003234	Hs.529618	BC001188
TFRC	transferrin receptor (p90, CD71) (TFRC), mRNA	NM_003234	Hs.529618	BC001188
TFRC	transferrin receptor (p90, CD71) (TFRC), mRNA	NM_003234	Hs.529618	BC001188
TFRC	transferrin receptor (p90, CD71) (TFRC), mRNA	NM_003234	Hs.529618	BC001188
TG	thyroglobulin (TG), mRNA	NM_003235	Hs.654591	NM_003235
TGFB3	transforming growth factor, beta 3 (TGFB3), mRNA	NM_003239	Hs.592317	AK122902
TGFBI	transforming growth factor, beta-induced, 68kDa (TGFBI), mRNA	NM_000358	Hs.369397	AB209598
TGFBR1	transforming growth factor, beta receptor I (activin A receptor type II-like kinase, 53kDa) (TGFBR1), mRNA	NM_004612	Hs.494622	NM_004612
THAP6	THAP domain containing 6 (THAP6), mRNA	NM_144721	Hs.479971	AL833350
THAP8	THAP domain containing 8 (THAP8), mRNA	NM_152658	Hs.350209	AK093048
THC2478115	THC2478115	THC2478115	Unknown	
THC2515368	THC2515368	THC2515368	Unknown	
THC2524582	Q5U0N8_HUMAN (Q5U0N8) Keratin 18 (Cell proliferation-inducing protein 46), partial (46%)	THC2524582	Unknown	
THC2527772	HUMC4AA2 complement component C4A {Homo sapiens} (exp=-1; wgp=0; cg=0), partial (6%)	THC2527772	Unknown	
THC2536579	THC2536579	THC2536579	Unknown	
THC2538067	Q86XA0_HUMAN (Q86XA0) LOC124512 protein (Fragment), partial (47%)	THC2538067	Unknown	
THC2550463	Q2KAS9_RHIEC (Q2KAS9) Probable two-component sensor histidine kinase protein, partial (4%)	THC2550463	Unknown	
THC2560976	THC2560976	THC2560976	Unknown	
THC2569153	Q804E3_GASAC (Q804E3) Neuronal 22 protein (Fragment), partial (18%)	THC2569153	Unknown	
THC2574008	Q475C5_RALEJ (Q475C5) Phosphate butyryltransferase , partial (5%)	THC2574008	Unknown	

THC2594845	AF246221 transmembrane protein BRI {Homo sapiens} (exp=-1; wgp=0; cg=0), partial (43%)	THC2594845	Unknown	
THC2603259	Q96IM5_HUMAN (Q96IM5) RAB7B protein, complete	THC2603259	Unknown	
THC2604598	AF154107 UDP-GalNAc:polypeptide N-acetylgalactosaminyltransferase 5 {Homo sapiens} (exp=-1; wgp=0; cg=0), partial (25%)	THC2604598	Unknown	
THC2609288	Q6YNM9_9LACO (Q6YNM9) Glycerol dehydratase medium subunit GldD, partial (7%)	THC2609288	Unknown	
THC2611894	THC2611894	THC2611894	Unknown	
THC2634329	THC2634329	THC2634329	Unknown	
THC2637707	ALU8_HUMAN (P39195) Alu subfamily SX sequence contamination warning entry, partial (8%)	THC2637707	Unknown	
THC2638097	Q3MHD6_HUMAN (Q3MHD6) COX17 homolog, cytochrome c oxidase assembly protein, complete	THC2638097	Unknown	
THC2642612	ALU1_HUMAN (P39188) Alu subfamily J sequence contamination warning entry, partial (7%)	THC2642612	Unknown	
THC2642694	Q6TDT1_HUMAN (Q6TDT1) Protein transactivated by hepatitis B virus E antigen, partial (11%)	THC2642694	Unknown	
THC2644672	XM_760139 citrate synthase {Theileria parva strain Muguga} (exp=-1; wgp=0; cg=0) , partial (5%)	THC2644672	Unknown	
THC2647388	Q4TD86_TETNG (Q4TD86) Chromosome undetermined SCAF6431, whole genome shotgun sequence. (Fragment), partial (9%)	THC2647388	Unknown	
THC2647689	THC2647689	THC2647689	Unknown	
THC2655298	THC2655298	THC2655298	Unknown	
THC2657567	THC2657567	THC2657567	Unknown	
THC2657647	NM_075999 F48B9.7 {Caenorhabditis elegans} (exp=-1; wgp=0; cg=0), partial (9%)	THC2657647	Unknown	
THC2658269	THC2658269	THC2658269	Unknown	
THC2659198	THC2659198	THC2659198	Unknown	
THC2659236	Q3W1V4_9ACTO (Q3W1V4) Protein-tyrosine kinase , partial (3%)	THC2659236	Unknown	
THC2661428	Q8IP73_DROME (Q8IP73) CG31855-PA (RE70574p), partial (12%)	THC2661428	Unknown	
THC2662262	ALU2_HUMAN (P39189) Alu subfamily SB sequence contamination warning entry, partial (32%)	THC2662262	Unknown	
THC2664371	ALU5_HUMAN (P39192) Alu subfamily SC sequence contamination warning entry, partial (7%)	THC2664371	Unknown	
THC2664573	THC2664573	THC2664573	Unknown	
THC2664989	Q40J89_EHRCH (Q40J89) Cation efflux protein, partial (6%)	THC2664989	Unknown	
THC2668815	Q4TBH3_TETNG (Q4TBH3) Chromosome 13 SCAF7124, whole genome shotgun sequence, partial (3%)	THC2668815	Unknown	
THC2676737	Q6FYH6_BARQU (Q6FYH6) Succinate dehydrogenase cytochrome b560 subunit, partial (10%)	THC2676737	Unknown	
THC2676797	THC2676797	THC2676797	Unknown	
THC2678509	THC2678509	THC2678509	Unknown	
THC2681410	THC2681410	THC2681410	Unknown	
THC2682885	Q6BEA3_RAT (Q6BEA3) WDNM1 homolog, partial (33%)	THC2682885	Unknown	
THC2684060	THC2684060	THC2684060	Unknown	
THC2684874	Q4FM57_PELUB (Q4FM57) Lipid-A-disaccharide synthase (LpxB) , partial (6%)	THC2684874	Unknown	
THC2685393	THC2685393	THC2685393	Unknown	
THC2688736	THC2688736	THC2688736	Unknown	
THC2689579	BPAP_BOVIN (P84291) Pregnancy-associated protein bPAP (Fragments), partial (10%)	THC2689579	Unknown	
THC2692434	BC018029 6-pyruvoyltetrahydropterin synthase {Homo sapiens} (exp=-1; wgp=0; cg=0), partial (25%)	THC2692434	Unknown	
THC2706386	Q9T6M0_GLOPA (Q9T6M0) NADH-ubiquinone oxidoreductase subunit 1, partial (6%)	THC2706386	Unknown	
THC2709754	Q6YSY6_ORYSA (Q6YSY6) Mucin-like protein, partial (6%)	THC2709754	Unknown	
THC2711870	O60448_HUMAN (O60448) Neuronal thread protein AD7c-NTP, partial (5%)	THC2711870	Unknown	
THC2712372	Q9RQ81_9ENTR (Q9RQ81) Alpha subunit of membrane-bound ATP synthase (Fragment), partial (5%)	THC2712372	Unknown	
THC2713266	THC2713266	THC2713266	Unknown	
THC2716965	THC2716965	THC2716965	Unknown	
THC2719076	THC2719076	THC2719076	Unknown	
THC2722577	Q40PK4_DESAC (Q40PK4) IMP dehydrogenase/GMP reductase:Histidine kinase, HAMP region:Bacterial chemotaxis sensory transducer precursor, partial (3%)	THC2722577	Unknown	
THC2730631	ARL9_HUMAN (Q6T311) ADP-ribosylation factor-like protein 9, partial (39%)	THC2730631	Unknown	
THC2733597	Q40J89_EHRCH (Q40J89) Cation efflux protein, partial (6%)	THC2733597	Unknown	
THC2738359	THC2738359	THC2738359	Unknown	
THC2754547	ALU1_HUMAN (P39188) Alu subfamily J sequence contamination warning entry, partial (3%)	THC2754547	Unknown	
THC2755341	Q2JAS1_FRASC (Q2JAS1) Binding-protein-dependent transport systems inner membrane component precursor, partial (8%)	THC2755341	Unknown	
THC2755576	ALU1_HUMAN (P39188) Alu subfamily J sequence contamination warning entry, partial (13%)	THC2755576	Unknown	

THC2785765	COX7B_HUMAN (P24311) Cytochrome c oxidase polypeptide VIIb, mitochondrial precursor , partial (78%)	THC2785765	Unknown	
TIMP1	TIMP metallopeptidase inhibitor 1 (TIMP1), mRNA	NM_003254	Hs.522632	BM913048
TIMP3	TIMP metallopeptidase inhibitor 3 (Sorsby fundus dystrophy, pseudoinflammatory) (TIMP3), mRNA	NM_000362	Hs.644633	NM_000362
TLE6	transducin-like enhancer of split 6 (E(sp1) homolog, Drosophila), mRNA (cDNA clone IMAGE:3687767).	BC007329	Hs.334507	BC020206
TLL2	tolloid-like 2 (TLL2), mRNA	NM_012465	Hs.154296	NM_012465
TMEFF2	transmembrane protein with EGF-like and two follistatin-like domains 2 (TMEFF2), mRNA	NM_016192	Hs.144513	DQ133599
TMEM127	transmembrane protein 127 (TMEM127), mRNA	NM_017849	Hs.699231	AK000514
TMEM134	transmembrane protein 134 (TMEM134), transcript variant 1, mRNA	NM_025124	Hs.288761	AY007143
TMEM139	transmembrane protein 139 (TMEM139), mRNA	NM_153345	Hs.17558	BC035517
TMEM142C	transmembrane protein 142C (TMEM142C), mRNA	NM_152288	Hs.460617	BC015555
TMEM14B	transmembrane protein 14B (TMEM14B), mRNA	NM_030969	Hs.273077	BM916878
TMEM176A	transmembrane protein 176A (TMEM176A), mRNA	NM_018487	Hs.647116	AK123468
TMEM176B	transmembrane protein 176B (TMEM176B), mRNA	NM_014020	Hs.647090	AK097304
TMEM38A	transmembrane protein 38A (TMEM38A), mRNA	NM_024074	Hs.436068	AK025981
TMEM47	transmembrane protein 47 (TMEM47), mRNA	NM_031442	Hs.8769	BC039242
TMOD1	tropomodulin 1 (TMOD1), mRNA	NM_003275	Hs.494595	AK096156
TMOD2	tropomodulin 2 (neuronal) (TMOD2), mRNA	NM_014548	Hs.659839	BC036184
TMTC1	transmembrane and tetratricopeptide repeat containing 1 (TMTC1), mRNA	NM_175861	Hs.401954	NM_175861
TMTC1	transmembrane and tetratricopeptide repeat containing 1 (TMTC1), mRNA	NM_175861	Hs.401954	NM_175861
TNFAIP2	tumor necrosis factor, alpha-induced protein 2 (TNFAIP2), mRNA	NM_006291	Hs.525607	NM_006291
TNFRSF11B	tumor necrosis factor receptor superfamily, member 11b (osteoprotegerin) (TNFRSF11B), mRNA	NM_002546	Hs.81791	NM_002546
TNFRSF11B	tumor necrosis factor receptor superfamily, member 11b (osteoprotegerin) (TNFRSF11B), mRNA	NM_002546	Hs.81791	NM_002546
TNFRSF11B	tumor necrosis factor receptor superfamily, member 11b (osteoprotegerin) (TNFRSF11B), mRNA	NM_002546	Hs.81791	NM_002546
TNFRSF11B	tumor necrosis factor receptor superfamily, member 11b (osteoprotegerin) (TNFRSF11B), mRNA	NM_002546	Hs.81791	NM_002546
TNFRSF11B	tumor necrosis factor receptor superfamily, member 11b (osteoprotegerin) (TNFRSF11B), mRNA	NM_002546	Hs.81791	NM_002546
TNFRSF11B	tumor necrosis factor receptor superfamily, member 11b (osteoprotegerin) (TNFRSF11B), mRNA	NM_002546	Hs.81791	NM_002546
TNFRSF11B	tumor necrosis factor receptor superfamily, member 11b (osteoprotegerin) (TNFRSF11B), mRNA	NM_002546	Hs.81791	NM_002546
TNFRSF11B	tumor necrosis factor receptor superfamily, member 11b (osteoprotegerin) (TNFRSF11B), mRNA	NM_002546	Hs.81791	NM_002546
TNFRSF11B	tumor necrosis factor receptor superfamily, member 11b (osteoprotegerin) (TNFRSF11B), mRNA	NM_002546	Hs.81791	NM_002546
TNFRSF11B	tumor necrosis factor receptor superfamily, member 11b (osteoprotegerin) (TNFRSF11B), mRNA	NM_002546	Hs.81791	NM_002546
TNFRSF11B	tumor necrosis factor receptor superfamily, member 11b (osteoprotegerin) (TNFRSF11B), mRNA	NM_002546	Hs.81791	NM_002546
TNNC1	troponin C type 1 (slow) (TNNC1), mRNA	NM_003280	Hs.118845	CF553054
TNNI1	troponin I type 1 (skeletal, slow) (TNNI1), mRNA	NM_003281	Hs.320890	NM_003281
TNNI3	troponin I type 3 (cardiac) (TNNI3), mRNA	NM_000363	Hs.351582	BC063449
TNNI3K	TNNI3 interacting kinase (TNNI3K), mRNA	NM_015978	Hs.480085	BX640903
TNNI3K	TNNI3 interacting kinase (TNNI3K), mRNA	NM_015978	Hs.480085	BX640903
TNNT2	troponin T {exons 14-15} .	S71126	Unknown	
TNS1	tensin 1 (TNS1), mRNA	NM_022648	Unknown	
TNS3	tensin 3 (TNS3), mRNA	NM_022748	Hs.520814	NM_022748
TPI1	triosephosphate isomerase 1 (TPI1), mRNA	NM_000365	Hs.524219	BM913099
TPM1	tropomyosin 1 (alpha) (TPM1), transcript variant 5, mRNA	NM_000366	Hs.133892	BX648171
TPM2	tropomyosin 2 (beta) (TPM2), transcript variant 2, mRNA	NM_213674	Hs.300772	CR590682
TPM2	tropomyosin 2 (beta) (TPM2), transcript variant 2, mRNA	NM_213674	Hs.300772	CR590682
TREM2	triggering receptor expressed on myeloid cells 2 (TREM2), mRNA	NM_018965	Hs.435295	BM548441
TRIM50	tripartite motif-containing 50 (TRIM50), mRNA	NM_178125	Hs.647053	AY081948
TRIM54	tripartite motif-containing 54 (TRIM54), transcript variant 2, mRNA	NM_187841	Hs.516036	NM_032546
TRIM55	tripartite motif-containing 55 (TRIM55), transcript variant 3, mRNA	NM_184086	Hs.85524	NM_033058
TRIM63	tripartite motif-containing 63 (TRIM63), mRNA	NM_032588	Hs.279709	AF353673
TRIM69	tripartite motif-containing 69 (TRIM69), transcript variant a, mRNA	NM_182985	Hs.489254	AK226115
TRIM9	tripartite motif-containing 9 (TRIM9), transcript variant 1, mRNA	NM_015163	Hs.654750	BC063872
TRIM9	tripartite motif-containing 9 (TRIM9), transcript variant 2, mRNA	NM_052978	Hs.654750	BC063872
TRPT1	tRNA phosphotransferase 1 (TRPT1), transcript variant 1, mRNA	NM_001033678	Hs.326586	CR591349

TSC22D2	TSC22 domain family, member 2 (TSC22D2), mRNA	NM_014779	Hs.699382	AB014569
TSG101	tumor susceptibility gene 101 (TSG101), mRNA	NM_006292	Hs.523512	BM542397
TSPAN16	tetraspanin 16 (TSPAN16), mRNA	NM_012466	Hs.579784	BM552773
TSPAN32	tetraspanin 32 (TSPAN32), transcript variant 1, mRNA	NM_139022	Hs.271954	CR606621
TTN	titin (TTN), transcript variant novex-3, mRNA	NM_133379	Hs.654592	NM_133378
TTYH2	tweety homolog 2 (Drosophila) (TTYH2), transcript variant 1, mRNA	NM_032646	Hs.27935	AK126955
TUBB	tubulin, beta (TUBB), mRNA	NM_178014	Hs.699200	AK098772
TXLNB	taxilin beta (TXLNB), mRNA	NM_153235	Hs.535820	BX647477
TXNDC13	thioredoxin domain containing 13 (TXNDC13), mRNA	NM_021156	Hs.169358	BC044777
UBB	ubiquitin B (UBB), mRNA	NM_018955	Hs.356190	BF572309
UBE2E2	ubiquitin-conjugating enzyme E2E 2 (UBC4/5 homolog, yeast) (UBE2E2), mRNA	NM_152653	Hs.475688	CR592620
UBR1	ubiquitin protein ligase E3 component n-recognin 1 (UBR1), mRNA	NM_174916	Hs.591121	NM_174916
UCRC	ubiquinol-cytochrome c reductase complex (7.2 kD) (UCRC), transcript variant 2, mRNA	NM_001003684	Hs.284292	BF965131
UHMK1	Serine/threonine-protein kinase Kist (EC 2.7.11.1) (Kinase interacting with stathmin) (U2AF homology motif kinase 1).	ENST00000282	Unknown	
UNC45B	unc-45 homolog B (C. elegans) (UNC45B), transcript variant 1, mRNA	NM_173167	Hs.379636	NM_173167
UNC45B	unc-45 homolog B (C. elegans) (UNC45B), transcript variant 1, mRNA	NM_173167	Hs.379636	NM_173167
UNQ501	MBC3205 (UNQ501), mRNA	NM_198536	Hs.8036	BC071616
UQCRC1	ubiquinol-cytochrome c reductase core protein I (UQCRC1), mRNA	NM_003365	Hs.119251	L16842
UQCRQ	ubiquinol-cytochrome c reductase, complex III subunit VII, 9.5kDa (UQCRQ), nuclear gene encoding mitochondrial protein, mRNA	NM_014402	Hs.146602	NM_014402
UROC1	urocanase domain containing 1 (UROC1), mRNA	NM_144639	Hs.331148	AK055862
USMG5	upregulated during skeletal muscle growth 5 homolog (mouse) (USMG5), mRNA	NM_032747	Hs.500921	BE889962
USP15	ubiquitin specific peptidase 15 (USP15), mRNA	NM_006313	Hs.434951	AF106069
VDR	vitamin D (1,25- dihydroxyvitamin D3) receptor (VDR), transcript variant 2, mRNA	NM_001017535	Hs.524368	NM_001017535
VDR	vitamin D (1,25- dihydroxyvitamin D3) receptor (VDR), transcript variant 2, mRNA	NM_001017535	Hs.524368	NM_001017535
VDR	vitamin D (1,25- dihydroxyvitamin D3) receptor (VDR), transcript variant 2, mRNA	NM_001017535	Hs.524368	NM_001017535
VDR	vitamin D (1,25- dihydroxyvitamin D3) receptor (VDR), transcript variant 2, mRNA	NM_001017535	Hs.524368	NM_001017535
VDR	vitamin D (1,25- dihydroxyvitamin D3) receptor (VDR), transcript variant 2, mRNA	NM_001017535	Hs.524368	NM_001017535
VDR	vitamin D (1,25- dihydroxyvitamin D3) receptor (VDR), transcript variant 2, mRNA	NM_001017535	Hs.524368	NM_001017535
VDR	vitamin D (1,25- dihydroxyvitamin D3) receptor (VDR), transcript variant 2, mRNA	NM_001017535	Hs.524368	NM_001017535
VDR	vitamin D (1,25- dihydroxyvitamin D3) receptor (VDR), transcript variant 2, mRNA	NM_001017535	Hs.524368	NM_001017535
VDR	vitamin D (1,25- dihydroxyvitamin D3) receptor (VDR), transcript variant 2, mRNA	NM_001017535	Hs.524368	NM_001017535
VDR	vitamin D (1,25- dihydroxyvitamin D3) receptor (VDR), transcript variant 2, mRNA	NM_001017535	Hs.524368	NM_001017535
VGLL3	vestigial like 3 (Drosophila) (VGLL3), mRNA	NM_016206	Hs.435013	NM_016206
VIPR2	vasoactive intestinal peptide receptor 2 (VIPR2), mRNA	NM_003382	Hs.654505	X95097
VIPR2	vasoactive intestinal peptide receptor 2 (VIPR2), mRNA	NM_003382	Hs.654505	X95097
VLDLR	very low density lipoprotein receptor (VLDLR), transcript variant 1, mRNA	NM_003383	Hs.370422	L20470
VPS37A	vacuolar protein sorting 37 homolog A (S. cerevisiae) (VPS37A), mRNA	NM_152415	Hs.343873	AL834189
VSIG4	V-set and immunoglobulin domain containing 4 (VSIG4), mRNA	NM_007268	Hs.8904	AY358341
VSX1	visual system homeobox 1 homolog, CHX10-like (zebrafish) (VSX1), transcript variant 1, mRNA	NM_014588	Hs.274264	DQ854807
WDR22	WD repeat domain 22 (WDR22), mRNA	NM_003861	Hs.509780	BC150267
WDR45	WD repeat domain 45 (WDR45), transcript variant 1, mRNA	NM_007075	Hs.632807	BC035979
WDR63	WD repeat domain 63 (WDR63), mRNA	NM_145172	Hs.97933	BX648851
WDR81	WD repeat domain 81 (WDR81), mRNA	NM_152348	Hs.234572	AK074111
WEE1	WEE1 homolog (S. pombe) (WEE1), mRNA	NM_003390	Hs.249441	BX641032
WNT11	wingless-type MMTV integration site family, member 11 (WNT11), mRNA	NM_004626	Hs.108219	Y12692
X01147	Human mRNA for immunoglobulin lambda variable region corresponding to NEW protein of V lambda subgroup I.	X01147	Hs.654512	BM922717
XBP1	X-box binding protein 1 (XBP1), transcript variant 1, mRNA	NM_005080	Hs.437638	AK093842
XPO4	exportin 4 (XPO4), mRNA	NM_022459	Hs.507452	NM_022459
XRCC4	X-ray repair complementing defective repair in Chinese hamster cells 4 (XRCC4), transcript variant 3, mRNA	NM_022550	Hs.567359	NM_022550
YIPF5	Yip1 domain family, member 5 (YIPF5), transcript variant 2, mRNA	NM_030799	Hs.372050	AY640926
YIPF7	Yip1 domain family, member 7 (YIPF7), mRNA	NM_182592	Hs.596000	AK096895

ZADH1	zinc binding alcohol dehydrogenase, domain containing 1 (ZADH1), mRNA	NM_152444	Hs.632344	AK096410
ZAK	sterile alpha motif and leucine zipper containing kinase AZK (ZAK), transcript variant 2, mRNA	NM_133646	Hs.444451	AF480462
ZBTB43	zinc finger and BTB domain containing 43 (ZBTB43), mRNA	NM_014007	Hs.591903	AF049907
ZBTB43	Zinc finger and BTB domain-containing protein 43 (Zinc finger protein 297B) (ZnF-x) (Zinc finger and BTB domain-containing protein 22B).	ENST00000373	Unknown	
ZBTB46	zinc finger and BTB domain containing 46 (ZBTB46), mRNA	NM_025224	Hs.585028	AK131482
ZFP106	zinc finger protein 106 homolog (mouse) (ZFP106), mRNA	NM_022473	Hs.511143	AF205632
ZIM2	zinc finger, imprinted 2 (ZIM2), mRNA	NM_015363	Hs.201776	NM_006210
ZNF160	zinc finger protein 160, mRNA (cDNA clone IMAGE:3452857), complete cds.	BC000807	Hs.655967	NM_198893
ZNF211	zinc finger protein 211 (ZNF211), transcript variant 2, mRNA	NM_198855	Hs.590977	AB209847
ZNF257	zinc finger protein 257 (ZNF257), mRNA	NM_033468	Hs.283900	BC036446
ZNF302	zinc finger protein 302 (ZNF302), transcript variant 1, mRNA	NM_018443	Hs.436350	AK122855
ZNF318	zinc finger protein 318 (ZNF318), mRNA	NM_014345	Hs.509718	AF090114
ZNF364	Zinc finger protein 364 (Rabring 7) (RING finger protein 115).	ENST00000369	Unknown	
ZNF385	zinc finger protein 385 (ZNF385), mRNA	NM_015481	Hs.505653	AY461717
ZNF396	zinc finger protein (ZNF396) mRNA, complete cds.	AF533251	Hs.351005	AK055775
ZNF447	zinc finger protein 447 (ZNF447), mRNA	NM_023926	Hs.235390	NM_023926
ZNF454	zinc finger protein 454 (ZNF454), mRNA	NM_182594	Hs.259441	NM_182594
ZNF585A	zinc finger protein 585A (ZNF585A), transcript variant 1, mRNA	NM_152655	Hs.390568	NM_152655
ZNF629	DNA-binding protein (Fragment).	ENST00000262	Unknown	
ZNF672	zinc finger protein 672 (ZNF672), mRNA	NM_024836	Hs.521151	BC035140
ZNF773	zinc finger protein 773 (ZNF773), mRNA	NM_198542	Hs.579576	AK160372
ZP1	zona pellucida glycoprotein 1 (sperm receptor) (ZP1), mRNA	NM_207341	Hs.172130	NM_207341
ZYG11BL	zyg-11 homolog B (C. elegans)-like (ZYG11BL), mRNA	NM_006336	Hs.147950	BC052563
A_23_P113453	A_23_P113453	A_23_P113453	Unknown	
A_23_P154006	A_23_P154006	A_23_P154006	Unknown	
A_23_P170713	A_23_P170713	A_23_P170713	Unknown	
A_23_P21882	A_23_P21882	A_23_P21882	Unknown	
A_23_P251002	A_23_P251002	A_23_P251002	Unknown	
A_23_P33773	A_23_P33773	A_23_P33773	Unknown	
A_23_P72014	A_23_P72014	A_23_P72014	Unknown	
A_24_P15973	A_24_P15973	A_24_P15973	Unknown	
A_24_P161733	A_24_P161733	A_24_P161733	Unknown	
A_24_P177634	A_24_P177634	A_24_P177634	Unknown	
A_24_P229911	A_24_P229911	A_24_P229911	Unknown	
A_24_P230486	A_24_P230486	A_24_P230486	Unknown	
A_24_P247303	A_24_P247303	A_24_P247303	Unknown	
A_24_P281605	A_24_P281605	A_24_P281605	Unknown	
A_24_P290046	A_24_P290046	A_24_P290046	Unknown	
A_24_P290114	A_24_P290114	A_24_P290114	Unknown	
A_24_P315256	A_24_P315256	A_24_P315256	Unknown	
A_24_P33055	A_24_P33055	A_24_P33055	Unknown	
A_24_P340886	A_24_P340886	A_24_P340886	Unknown	
A_24_P341626	A_24_P341626	A_24_P341626	Unknown	
A_24_P358406	A_24_P358406	A_24_P358406	Unknown	
A_24_P409420	A_24_P409420	A_24_P409420	Unknown	
A_24_P452024	A_24_P452024	A_24_P452024	Unknown	
A_24_P452293	A_24_P452293	A_24_P452293	Unknown	
A_24_P471242	A_24_P471242	A_24_P471242	Unknown	
A_24_P587993	A_24_P587993	A_24_P587993	Unknown	
A_24_P591119	A_24_P591119	A_24_P591119	Unknown	
A_24_P6850	A_24_P6850	A_24_P6850	Unknown	
A_24_P7750	A_24_P7750	A_24_P7750	Unknown	

A_24_P780319	A_24_P780319	A_24_P780319	Unknown	
A_24_P792988	A_24_P792988	A_24_P792988	Unknown	
A_24_P844100	A_24_P844100	A_24_P844100	Unknown	
A_24_P890995	A_24_P890995	A_24_P890995	Unknown	
A_24_P913190	A_24_P913190	A_24_P913190	Unknown	
A_24_P93111	A_24_P93111	A_24_P93111	Unknown	
A_32_P121674	A_32_P121674	A_32_P121674	Unknown	
A_32_P127454	A_32_P127454	A_32_P127454	Unknown	
A_32_P162958	A_32_P162958	A_32_P162958	Unknown	
A_32_P163558	A_32_P163558	A_32_P163558	Unknown	
A_32_P214860	A_32_P214860	A_32_P214860	Unknown	
A_32_P223245	A_32_P223245	A_32_P223245	Unknown	
A_32_P24295	A_32_P24295	A_32_P24295	Unknown	
A_32_P24685	A_32_P24685	A_32_P24685	Unknown	
A_32_P35603	A_32_P35603	A_32_P35603	Unknown	
A_32_P36709	A_32_P36709	A_32_P36709	Unknown	
A_32_P441530	A_32_P441530	A_32_P441530	Unknown	
A_32_P47874	A_32_P47874	A_32_P47874	Unknown	
A_32_P4882	A_32_P4882	A_32_P4882	Unknown	
A_32_P66025	A_32_P66025	A_32_P66025	Unknown	
A_32_P93807	A_32_P93807	A_32_P93807	Unknown	
