# Supplementary material for: Transcriptional and Functional Profiling of Human Embryonic Stem Cell-Derived Cardiomyocytes
Source: PLoS One. 2008 Oct 22;3(10):e3474. doi: 10.1371/journal.pone.0003474 (PMC2565131; doi:10.1371/journal.pone.0003474)
Supplement: Table S4 — Gene Ontology analysis of K-means clustering data. (0.20 MB PDF) [file pone.0003474.s013.pdf]

Supplemental Table B5

GO Biological Processes Overrepresented in K-Means Cluster 1

GO CATEGORY	TOTAL GENES	CHANGED GENES	LOG10(p)	FALSE DISCOVERY RATE
GO:0048193_Golgi_vesicle_transport	172	31	-7.699894	0
GO:0016192_vesicle-mediated_transport	664	75	-7.516581	0
GO:0006888_ER_to_Golgi_vesicle-mediated_transport	102	20	-5.777739	0
GO:0043123_positive_regulation_of_I-kappaB_kinase_NF-kappaB_cascade	87	17	-4.981717	0
GO:0043122_regulation_of_I-kappaB_kinase_NF-kappaB_cascade	97	18	-4.911681	0
GO:0046907_intracellular_transport	968	87	-4.394667	0.01
GO:0007243_protein_kinase_cascade	486	51	-4.391138	0.008571
GO:0007249_I-kappaB_kinase_NF-kappaB_cascade	136	21	-4.353143	0.00875
GO:0009967_positive_regulation_of_signal_transduction	169	24	-4.276798	0.007778
GO:0007044_cell-substrate_junction_assembly	9	5	-4.141362	0.009
GO:0030334_regulation_of_cell_migration	85	15	-3.932812	0.01
GO:0045184_establishment_of_protein_localization	886	79	-3.917577	0.009167
GO:0045045_secretory_pathway	432	45	-3.86643	0.010769
GO:0015031_protein_transport	820	74	-3.860564	0.010714
GO:0032940_secretion_by_cell	495	49	-3.621444	0.024667
GO:0048522_positive_regulation_of_cellular_process	1280	105	-3.606594	0.025
GO:0008104_protein_localization	963	83	-3.602009	0.023529
GO:0030335_positive_regulation_of_cell_migration	30	8	-3.592037	0.022778
GO:0006892_post-Golgi_vesicle-mediated_transport	54	11	-3.591567	0.021579
GO:0016197_endosome_transport	74	13	-3.464542	0.0265
GO:0033036_macromolecule_localization	1030	87	-3.462443	0.025238
GO:0051649_establishment_of_cellular_localization	1243	100	-3.159878	0.044545
GO:0051641_cellular_localization	1274	102	-3.142608	0.044348
GO:0001666_response_to_hypoxia	101	15	-3.094621	0.0572
GO:0051270_regulation_of_cell_motility	101	15	-3.094621	0.0572
GO:0009605_response_to_external_stimulus	889	74	-2.84196	0.093077
GO:0042060_wound_healing	187	22	-2.825235	0.094444
GO:0048518_positive_regulation_of_biological_process	1424	110	-2.806236	0.091429
GO:0016044_membrane_organization_and_biogenesis	417	40	-2.800933	0.088276
GO:0040012_regulation_of_locomotion	108	15	-2.791599	0.086
GO:0051272_positive_regulation_of_cell_motility	39	8	-2.774545	0.085161
GO:0006950_response_to_stress	1371	106	-2.731408	0.0925
GO:0007117_budding_cell_bud_growth	5	3	-2.730477	0.102941
GO:0048590_non-developmental_growth	5	3	-2.730477	0.102941
GO:0040017_positive_regulation_of_locomotion	40	8	-2.700225	0.109714
GO:0006916_anti-apoptosis	218	24	-2.635534	0.113889
GO:0046903_secretion	577	51	-2.631709	0.111081
GO:0007242_intracellular_signaling_cascade	1637	122	-2.510782	0.133158
GO:0009612_response_to_mechanical_stimulus	43	8	-2.492437	0.137436
GO:0030866_cortical_actin_cytoskeleton_organization_and_biogenesis	26	6	-2.467224	0.13925
GO:0006663_platelet_activating_factor_biosynthetic_process	2	2	-2.460437	0.214524
GO:0046469_platelet_activating_factor_metabolic_process	2	2	-2.460437	0.214524
GO:0030522_intracellular_receptor-mediated_signaling_pathway	63	10	-2.451866	0.211163
GO:0040011_locomotion	117	15	-2.447145	0.223409
GO:0032496_response_to_lipopolysaccharide	19	5	-2.390632	0.240222
GO:0030447_filamentous_growth	45	8	-2.365276	0.240652
GO:0009611_response_to_wounding	541	47	-2.325752	0.242766
GO:0009725_response_to_hormone_stimulus	193	21	-2.316289	0.23875

GO:0006897_endocytosis	270	27	-2.30449	0.2312
GO:0010324_membrane_invagination	270	27	-2.30449	0.2312
GO:0006893_Golgi_to_plasma_membrane_transport	13	4	-2.254798	0.247647
GO:0006928_cell_motility	618	52	-2.239102	0.241698
GO:0051674_localization_of_cell	618	52	-2.239102	0.241698
GO:0040007_growth	491	43	-2.233168	0.238148
GO:0007262_STAT_protein_nuclear_translocation	7	3	-2.225196	0.251607
GO:0016203_muscle_attachment	7	3	-2.225196	0.251607
GO:0030865_cortical_cytoskeleton_organization_and_biogenesis	29	6	-2.217996	0.250175
GO:0030448_hyphal_growth	21	5	-2.190057	0.256552
GO:0045429_positive_regulation_of_nitric_oxide_biosynthetic_process	14	4	-2.129125	0.277458
GO:0030036_actin_cytoskeleton_organization_and_biogenesis	320	30	-2.100871	0.2785
GO:0007041_lysosomal_transport	22	5	-2.09926	0.276557
GO:0019221_cytokine_and_chemokine_mediated_signaling_pathway	60	9	-2.093821	0.273387
GO:0006915_apoptosis	924	72	-2.078912	0.275714
GO:0007476_imaginal_disc-derived_wing_morphogenesis	31	6	-2.071064	0.275938
GO:0016310_phosphorylation	851	67	-2.064418	0.273538
GO:0031325_positive_regulation_of_cellular_metabolic_process	532	45	-2.046915	0.272273
GO:0007443_Malpighian_tubule_morphogenesis	8	3	-2.040378	0.28806
GO:0042981_regulation_of_apoptosis	693	56	-2.019139	0.290441
GO:0009737_response_to_abscisic_acid_stimulus	15	4	-2.014835	0.297826
GO:0007472_wing_disc_morphogenesis	32	6	-2.002542	0.302429
GO:0006361_transcription_initiation_from_RNA_polymerase_I_promoter	3	2	-2.000667	0.344872
GO:0009738_abscisic_acid_mediated_signaling	3	2	-2.000667	0.344872
GO:0009787_regulation_of_abscisic_acid_mediated_signaling	3	2	-2.000667	0.344872
GO:0010119_regulation_of_stomatal_movement	3	2	-2.000667	0.344872
GO:0035317_imaginal_disc-derived_wing_hair_organization_and_biogenesis	3	2	-2.000667	0.344872
GO:0042684_cardioblast_cell_fate_commitment	3	2	-2.000667	0.344872
GO:0046664_dorsal_closure__amnioserosa_morphology_change	3	2	-2.000667	0.344872
GO:0048170_positive_regulation_of_long-term_neuronal_synaptic_plasticity	3	2	-2.000667	0.344872
GO:0006793_phosphorus_metabolic_process	1037	79	-1.997478	0.338
GO:0006796_phosphate_metabolic_process	1037	79	-1.997478	0.338
GO:0016477_cell_migration	451	39	-1.989016	0.335062
GO:0050817_coagulation	96	12	-1.977501	0.331951
GO:0012501_programmed_cell_death	952	73	-1.944217	0.335904
GO:0006468_protein_amino_acid_phosphorylation	700	56	-1.936065	0.334881
GO:0002237_response_to_molecule_of_bacterial_origin	24	5	-1.93359	0.334118
GO:0030518_steroid_hormone_receptor_signaling_pathway	53	8	-1.930388	0.331395
GO:0043627_response_to_estrogen_stimulus	43	7	-1.919452	0.330575
GO:0009615_response_to_virus	98	12	-1.909587	0.335455
GO:0042090_interleukin-12_biosynthetic_process	9	3	-1.883525	0.339355
GO:0043330_response_to_exogenous_dsRNA	9	3	-1.883525	0.339355
GO:0045075_regulation_of_interleukin-12_biosynthetic_process	9	3	-1.883525	0.339355
GO:0048146_positive_regulation_of_fibroblast_proliferation	9	3	-1.883525	0.339355
GO:0051482_elevation_of_cytosolic_calcium_ion_concentration_during_G-protein_signaling__coupled_to_IP3_second_messenger_(phospholipase_C_activating)	9	3	-1.883525	0.339355
GO:0018108_peptidyl-tyrosine_phosphorylation	76	10	-1.875603	0.339255
GO:0050896_response_to_stimulus	2926	198	-1.873369	0.336737
GO:0043067_regulation_of_programmed_cell_death	706	56	-1.866915	0.333542
GO:0006886_intracellular_protein_transport	618	50	-1.866592	0.330309
GO:0045941_positive_regulation_of_transcription	418	36	-1.851133	0.333061
GO:0008219_cell_death	993	75	-1.838419	0.3298

Supplemental Table B6

GO Biological Processes Overrepresented in K-Means Cluster 2

GO CATEGORY	TOTAL GENES	CHANGED GENES	LOG10(p)	FALSE DISCOVERY RATE
GO:0006139_nucleobase__nucleoside__nucleotide_and_nucleic_acid_metabolic_process	3801	455	-26.685015	0
GO:0016070_RNA_metabolic_process	2861	361	-23.773166	0
GO:0043283_biopolymer_metabolic_process	4991	524	-17.73327	0
GO:0019219_regulation_of_nucleobase__nucleoside__nucleotide_and_nucleic_acid_metabolic_process	2504	302	-16.100626	0
GO:0006259_DNA_metabolic_process	876	139	-15.778723	0
GO:0016071_mRNA_metabolic_process	339	74	-15.603326	0
GO:0032774_RNA_biosynthetic_process	2325	282	-15.175014	0
GO:0006355_regulation_of_transcription__DNA-dependent	2246	274	-15.004023	0
GO:0006397_mRNA_processing	289	66	-14.979766	0
GO:0045449_regulation_of_transcription	2411	289	-14.912589	0
GO:0006350_transcription	2513	298	-14.808725	0
GO:0006351_transcription__DNA-dependent	2320	280	-14.76125	0
GO:0031323_regulation_of_cellular_metabolic_process	2755	315	-13.548918	0
GO:0006396_RNA_processing	510	90	-12.893232	0
GO:0019222_regulation_of_metabolic_process	2894	321	-12.037613	0
GO:0008380_RNA_splicing	228	52	-11.887018	0
GO:0007001_chromosome_organization_and_biogenesis_(sensu_Eukaryota)	547	89	-10.727621	0
GO:0051276_chromosome_organization_and_biogenesis	567	91	-10.626799	0
GO:0006325_establishment_and_or_maintenance_of_chromatin_architecture	383	69	-10.393266	0
GO:0006323_DNA_packaging	389	69	-10.080352	0
GO:0016568_chromatin_modification	250	49	-8.792126	0
GO:0043170_macromolecule_metabolic_process	6555	603	-8.295701	0
GO:0006260_DNA_replication	246	45	-7.179247	0
GO:0006974_response_to_DNA_damage_stimulus	352	57	-6.975268	0
GO:0044238_primary_metabolic_process	7473	661	-6.295809	0
GO:0044237_cellular_metabolic_process	7508	661	-5.910124	0
GO:0006261_DNA-dependent_DNA_replication	140	28	-5.472251	0.001111
GO:0006281_DNA_repair	273	44	-5.466653	0.001071
GO:0050794_regulation_of_cellular_process	4523	420	-5.196397	0.001379
GO:0006338_chromatin_remodeling	108	22	-4.562414	0.003667
GO:0008152_metabolic_process	8100	696	-4.56205	0.003548
GO:0006333_chromatin_assembly_or_disassembly	203	33	-4.330407	0.004062
GO:0042770_DNA_damage_response__signal_transduction	82	18	-4.287805	0.003939
GO:0000375_RNA_splicing__via_transesterification_reactions	113	22	-4.246554	0.004444
GO:0000377_RNA_splicing__via_transesterification_reactions_with_bulged_adenosine_as_nucleophile	113	22	-4.246554	0.004444
GO:0000398_nuclear_mRNA_splicing__via_spliceosome	113	22	-4.246554	0.004444
GO:0009719_response_to_endogenous_stimulus	558	69	-4.020559	0.005135
GO:0000245_spliceosome_assembly	45	12	-3.889584	0.005789
GO:0050789_regulation_of_biological_process	5045	451	-3.838704	0.007692
GO:0006390_transcription_from_mitochondrial_promoter	5	4	-3.753141	0.009524
GO:0006564_L-serine_biosynthetic_process	5	4	-3.753141	0.009524
GO:0045740_positive_regulation_of_DNA_replication	5	4	-3.753141	0.009524
GO:0007064_mitotic_sister_chromatid_cohesion	6	4	-3.303985	0.023256
GO:0051052_regulation_of_DNA_metabolic_process	82	16	-3.260154	0.025682
GO:0016569_covalent_chromatin_modification	90	17	-3.2564	0.025111
GO:0006357_regulation_of_transcription_from_RNA_polymerase_II_promoter	670	76	-3.193408	0.027826
GO:0006366_transcription_from_RNA_polymerase_II_promoter	865	94	-3.184918	0.027234

GO:0045934_negative_regulation_of_nucleobase__nucleoside__nucleotide_and_nucleic_acid_metabolic_process	463	56	-3.1337	0.0275
GO:0006352_transcription_initiation	77	15	-3.083988	0.029796
GO:0016570_histone_modification	87	16	-2.967804	0.035
GO:0000077_DNA_damage_checkpoint	49	11	-2.923082	0.038824
GO:0007062_sister_chromatid_cohesion	23	7	-2.843215	0.043269
GO:0065004_protein-DNA_complex_assembly	188	27	-2.801285	0.046981
GO:0022618_protein-RNA_complex_assembly	143	22	-2.765603	0.047593
GO:0007418_ventral_midline_development	4	3	-2.741671	0.057544
GO:0050686_negative_regulation_of_mRNA_processing	4	3	-2.741671	0.057544
GO:0051253_negative_regulation_of_RNA_metabolic_process	4	3	-2.741671	0.057544
GO:0043009_chordate_embryonic_development	210	29	-2.700125	0.060517
GO:0006310_DNA_recombination	127	20	-2.687896	0.062373
GO:0045005_maintenance_of_fidelity_during_DNA-dependent_DNA_replication	31	8	-2.666253	0.063667
GO:0009070_serine_family_amino_acid_biosynthetic_process	13	5	-2.653601	0.065968
GO:0051054_positive_regulation_of_DNA_metabolic_process	13	5	-2.653601	0.065968
GO:0007131_meiotic_recombination	39	9	-2.571611	0.073333
GO:0021915_neural_tube_development	54	11	-2.562947	0.074531
GO:0031497_chromatin_assembly	150	22	-2.499957	0.078
GO:0006473_protein_amino_acid_acetylation	40	9	-2.491628	0.077879
GO:0006311_meiotic_gene_conversion	14	5	-2.490452	0.078529
GO:0031399_regulation_of_protein_modification	14	5	-2.490452	0.078529
GO:0000075_cell_cycle_checkpoint	97	16	-2.462228	0.087681
GO:0065007_biological_regulation	5486	472	-2.453564	0.087429
GO:0001840_neural_plate_development	48	10	-2.450634	0.087042
GO:0006402_mRNA_catabolic_process	49	10	-2.382442	0.093056
GO:0032446_protein_modification_by_small_protein_conjugation	135	20	-2.37299	0.092877
GO:0031570_DNA_integrity_checkpoint	57	11	-2.371513	0.092162
GO:0043570_maintenance_of_DNA_repeat_elements	5	3	-2.369881	0.105867
GO:0000819_sister_chromatid_segregation	66	12	-2.318839	0.115395
GO:0007059_chromosome_segregation	137	20	-2.299879	0.117013
GO:0022403_cell_cycle_phase	516	57	-2.285223	0.11641
GO:0016567_protein_ubiquitination	119	18	-2.285032	0.11519
GO:0000731_DNA_synthesis_during_DNA_repair	10	4	-2.268907	0.123457
GO:0006563_L-serine_metabolic_process	10	4	-2.268907	0.123457
GO:0043543_protein_amino_acid_acylation	51	10	-2.252537	0.123537
GO:0019941_modification-dependent_protein_catabolic_process	226	29	-2.23415	0.125952
GO:0043632_modification-dependent_macromolecule_catabolic_process	226	29	-2.23415	0.125952
GO:0022607_cellular_component_assembly	795	82	-2.230021	0.126235
GO:0005985_sucrose_metabolic_process	2	2	-2.211259	0.17
GO:0021508_floor_plate_formation	2	2	-2.211259	0.17
GO:0021754_facial_nucleus_development	2	2	-2.211259	0.17
GO:0021990_neural_plate_formation	2	2	-2.211259	0.17
GO:0042796_snRNA_transcription_from_RNA_polymerase_III_promoter	2	2	-2.211259	0.17
GO:0060032_notochord_regression	2	2	-2.211259	0.17
GO:0000184_mRNA_catabolic_process__nonsense-mediated_decay	23	6	-2.138907	0.178172
GO:0007098_centrosome_cycle	23	6	-2.138907	0.178172
GO:0009792_embryonic_development_ending_in_birth_or_egg_hatching	260	32	-2.138024	0.176489
GO:0051252_regulation_of_RNA_metabolic_process	45	9	-2.134047	0.175158
GO:0006298_mismatch_repair	30	7	-2.13346	0.174062
GO:0007127_meiosis_I	70	12	-2.109262	0.174227
GO:0006511_ubiquitin-dependent_protein_catabolic_process	221	28	-2.102128	0.172653
GO:0031573_intra-S_DNA_damage_checkpoint	11	4	-2.100166	0.177778

Supplemental Table B7

GO Biological Processes Overrepresented in K-Means Cluster 3

GO CATEGORY	TOTAL GENES	CHANGED GENES	LOG10(p)	FALSE DISCOVERY RATE
GO:0000904_cellular_morphogenesis_during_differentiation	320	30	-5.863364	0
GO:0007268_synaptic_transmission	455	36	-5.18176	0.005
GO:0001505_regulation_of_neurotransmitter_levels	168	19	-5.057014	0.003333
GO:0048666_neuron_development	344	28	-4.367348	0.0075
GO:0019226_transmission_of_nerve_impulse	518	37	-4.330562	0.012
GO:0031175_neurite_development	310	26	-4.312416	0.01
GO:0048667_neuron_morphogenesis_during_differentiation	275	24	-4.296515	0.0075
GO:0048812_neurite_morphogenesis	275	24	-4.296515	0.0075
GO:0045055_regulated_secretory_pathway	131	15	-4.177113	0.007778
GO:0007269_neurotransmitter_secretion	119	14	-4.064971	0.01
GO:0030030_cell_projection_organization_and_biogenesis	398	30	-4.022761	0.007692
GO:0032990_cell_part_morphogenesis	398	30	-4.022761	0.007692
GO:0048858_cell_projection_morphogenesis	398	30	-4.022761	0.007692
GO:0050877_neurological_process	1027	60	-4.01554	0.007143
GO:0007409_axonogenesis	252	22	-3.988182	0.009333
GO:0007267_cell-cell_signaling	876	53	-3.976279	0.00875
GO:0006953_acute-phase_response	36	7	-3.619939	0.022941
GO:0048488_synaptic_vesicle_endocytosis	26	6	-3.600357	0.021667
GO:0006493_protein_amino_acid_O-linked_glycosylation	27	6	-3.504372	0.029474
GO:0007528_neuromuscular_junction_development	28	6	-3.412787	0.0315
GO:0048935_peripheral_nervous_system_neuron_development	5	3	-3.368144	0.03619
GO:0000902_cell_morphogenesis	820	48	-3.3187	0.036087
GO:0032989_cellular_structure_morphogenesis	820	48	-3.3187	0.036087
GO:0032501_multicellular_organismal_process	4096	179	-3.198549	0.040833
GO:0048934_peripheral_nervous_system_neuron_differentiation	6	3	-3.078784	0.0524
GO:0045045_secretory_pathway	432	29	-3.075026	0.050385
GO:0006805_xenobiotic_metabolic_process	44	7	-3.064979	0.047143
GO:0048675_axon_extension	44	7	-3.064979	0.047143
GO:0032940_secretion_by_cell	495	32	-3.059823	0.046897
GO:0006801_superoxide_metabolic_process	22	5	-3.044342	0.048667
GO:0030182_neuron_differentiation	442	29	-2.92088	0.055484
GO:0006906_vesicle_fusion	14	4	-2.918456	0.056875
GO:0051239_regulation_of_multicellular_organismal_process	465	30	-2.888232	0.093939
GO:0048468_cell_development	1714	84	-2.862605	0.092941
GO:0045161_neuronal_ion_channel_clustering	7	3	-2.847396	0.104857
GO:0009410_response_to_xenobiotic_stimulus	48	7	-2.834633	0.103889
GO:0042445_hormone_metabolic_process	124	12	-2.799712	0.103514
GO:0003001_generation_of_a_signal_involved_in_cell-cell_signaling	176	15	-2.793343	0.102632
GO:0046530_photoreceptor_cell_differentiation	63	8	-2.765426	0.104359
GO:0002526_acute_inflammatory_response	93	10	-2.759035	0.10175
GO:0006334_nucleosome_assembly	109	11	-2.756105	0.099512
GO:0001654_eye_development	179	15	-2.720804	0.100714
GO:0048731_system_development	2443	112	-2.667766	0.103023
GO:0006032_chitin_catabolic_process	8	3	-2.654904	0.105111
GO:0006046_N-acetylglucosamine_catabolic_process	8	3	-2.654904	0.105111
GO:0048489_synaptic_vesicle_transport	68	8	-2.551946	0.115435
GO:0007423_sensory_organ_development	265	19	-2.505993	0.118085
GO:0006043_glucosamine_catabolic_process	9	3	-2.490419	0.1184

GO:0006590_thyroid_hormone_generation	9	3	-2.490419	0.1184
GO:0046348_amino_sugar_catabolic_process	9	3	-2.490419	0.1184
GO:0001754_eye_photoreceptor_cell_differentiation	56	7	-2.442514	0.126471
GO:0043062_extracellular_structure_organization_and_biogenesis	155	13	-2.432445	0.124615
GO:0015755_fructose_transport	3	2	-2.428897	0.158772
GO:0045065_cytotoxic_T_cell_differentiation	3	2	-2.428897	0.158772
GO:0045162_clustering_of_voltage-gated_sodium_channels	3	2	-2.428897	0.158772
GO:0045329_carnitine_biosynthetic_process	3	2	-2.428897	0.158772
GO:0050665_hydrogen_peroxide_biosynthetic_process	3	2	-2.428897	0.158772
GO:0048699_generation_of_neurons	505	30	-2.354102	0.166207
GO:0000272_polysaccharide_catabolic_process	20	4	-2.307331	0.178333
GO:0044247_cellular_polysaccharide_catabolic_process	20	4	-2.307331	0.178333
GO:0046903_secretion	577	33	-2.286489	0.180656
GO:0009100_glycoprotein_metabolic_process	180	14	-2.284586	0.177742
GO:0018193_peptidyl-amino_acid_modification	162	13	-2.270335	0.176508
GO:0006030_chitin_metabolic_process	11	3	-2.220363	0.199219
GO:0022008_neurogenesis	540	31	-2.201798	0.199231
GO:0009437_carnitine_metabolic_process	4	2	-2.138253	0.234366
GO:0019430_removal_of_superoxide_radicals	4	2	-2.138253	0.234366
GO:0031155_regulation_of_fruiting_body_development	4	2	-2.138253	0.234366
GO:0031156_regulation_of_fruiting_body_development_(sensu_Dictyosteliida)	4	2	-2.138253	0.234366
GO:0031157_regulation_of_aggregate_size	4	2	-2.138253	0.234366
GO:0046548_retinal_rod_cell_development	4	2	-2.138253	0.234366
GO:0007399_nervous_system_development	1083	54	-2.127483	0.231528
GO:0031497_chromatin_assembly	150	12	-2.124965	0.228767
GO:0065004_protein-DNA_complex_assembly	188	14	-2.12178	0.225676
GO:0008210_estrogen_metabolic_process	12	3	-2.106966	0.234667
GO:0060047_heart_contraction	82	8	-2.056118	0.242763
GO:0048856_anatomical_structure_development	2893	125	-2.044543	0.24039
GO:0006893_Golgi_to_plasma_membrane_transport	13	3	-2.004542	0.259
GO:0042308_negative_regulation_of_protein_import_into_nucleus	13	3	-2.004542	0.259
GO:0042992_negative_regulation_of_transcription_factor_import_into_nucleus	13	3	-2.004542	0.259
GO:0048741_skeletal_muscle_fiber_development	101	9	-2.000084	0.255244
GO:0048747_muscle_fiber_development	101	9	-2.000084	0.255244
GO:0051179_localization	3673	154	-1.985636	0.254217
GO:0051641_cellular_localization	1274	61	-1.976207	0.252857
GO:0016050_vesicle_organization_and_biogenesis	25	4	-1.951537	0.260824
GO:0048637_skeletal_muscle_development	121	10	-1.946209	0.25907
GO:0006620_posttranslational_protein_targeting_to_membrane	5	2	-1.926759	0.292472
GO:0035269_protein_amino_acid_O-linked_mannosylation	5	2	-1.926759	0.292472
GO:0045077_negative_regulation_of_interferon-gamma_biosynthetic_process	5	2	-1.926759	0.292472
GO:0007519_striated_muscle_development	179	13	-1.921436	0.289667
GO:0048869_cellular_developmental_process	2346	103	-1.91741	0.288462
GO:0006892_post-Golgi_vesicle-mediated_transport	54	6	-1.915296	0.287391
GO:0042744_hydrogen_peroxide_catabolic_process	14	3	-1.911304	0.297742
GO:0009790_embryonic_development	614	33	-1.902211	0.295638
GO:0006936_muscle_contraction	200	14	-1.899037	0.293053
GO:0042990_regulation_of_transcription_factor_import_into_nucleus	26	4	-1.891138	0.292396
GO:0050896_response_to_stimulus	2926	125	-1.888026	0.289485
GO:0051649_establishment_of_cellular_localization	1243	59	-1.8531	0.292959
GO:0008016_regulation_of_heart_contraction	72	7	-1.850333	0.291313

Supplemental Table B8

GO Biological Processes Overrepresented in K-Means Cluster 4

GO CATEGORY	TOTAL GENES	CHANGED GENES	LOG10(p)	FALSE DISCOVERY RATE
GO:0006936_muscle_contraction	200	57	-25.001994	0
GO:0007517_muscle_development	277	54	-15.553823	0
GO:0006941_striated_muscle_contraction	54	22	-13.590545	0
GO:0060047_heart_contraction	82	26	-12.892495	0
GO:0008015_circulation	219	41	-11.327156	0
GO:0032501_multicellular_organismal_process	4096	311	-10.823565	0
GO:0008016_regulation_of_heart_contraction	72	22	-10.64348	0
GO:0006937_regulation_of_muscle_contraction	53	17	-8.729106	0
GO:0048856_anatomical_structure_development	2893	226	-8.303841	0
GO:0051239_regulation_of_multicellular_organismal_process	465	58	-8.294965	0
GO:0048513_organ_development	1901	161	-8.025915	0
GO:0030239_myofibril_assembly	29	12	-7.766961	0
GO:0055002_striated_muscle_cell_development	29	12	-7.766961	0
GO:0055001_muscle_cell_development	30	12	-7.567508	0
GO:0006811_ion_transport	1067	102	-7.565098	0
GO:0048644_muscle_morphogenesis	9	7	-7.28526	0
GO:0031032_actomyosin_structure_organization_and_biogenesis	44	14	-7.243535	0
GO:0007507_heart_development	216	33	-6.963636	0
GO:0048731_system_development	2443	191	-6.878927	0
GO:0060048_cardiac_muscle_contraction	14	8	-6.710725	0
GO:0051146_striated_muscle_cell_differentiation	43	13	-6.475218	0
GO:0007519_striated_muscle_development	179	28	-6.213881	0
GO:0055008_cardiac_muscle_morphogensis	8	6	-6.134915	0
GO:0048628_myoblast_maturation	46	13	-6.098139	0
GO:0048627_myoblast_development	48	13	-5.865195	0
GO:0032502_developmental_process	3960	276	-5.504207	0.000385
GO:0007275_multicellular_organismal_development	2996	218	-5.473374	0.00037
GO:0007010_cytoskeleton_organization_and_biogenesis	738	71	-5.46126	0.000357
GO:0045445_myoblast_differentiation	68	15	-5.442684	0.000345
GO:0048741_skeletal_muscle_fiber_development	101	18	-5.003561	0.000323
GO:0048747_muscle_fiber_development	101	18	-5.003561	0.000323
GO:0055010_ventricular_cardiac_muscle_morphogenesis	7	5	-5.000625	0.000312
GO:0048637_skeletal_muscle_development	121	20	-4.977459	0.000303
GO:0006820_anion_transport	209	28	-4.873448	0.000294
GO:0015698_inorganic_anion_transport	178	25	-4.769007	0.000857
GO:0009887_organ_morphogenesis	758	69	-4.521028	0.001111
GO:0006939_smooth_muscle_contraction	46	11	-4.492155	0.001081
GO:0007155_cell_adhesion	834	74	-4.43977	0.001316
GO:0022610_biological_adhesion	836	74	-4.405317	0.001282
GO:0042692_muscle_cell_differentiation	102	17	-4.368554	0.00125
GO:0030036_actin_cytoskeleton_organization_and_biogenesis	320	36	-4.366481	0.00122
GO:0030029_actin_filament-based_process	339	37	-4.189544	0.002381
GO:0006091_generation_of_precursor_metabolites_and_energy	652	60	-4.128282	0.002326
GO:0015672_monovalent_inorganic_cation_transport	342	37	-4.109001	0.002273
GO:0045214_sarcomere_organization	15	6	-4.029071	0.003111
GO:0002026_cardiac_inotropy	10	5	-3.982545	0.003191
GO:0048738_cardiac_muscle_development	10	5	-3.982545	0.003191
GO:0050665_hydrogen_peroxide_biosynthetic_process	3	3	-3.767498	0.005417

GO:0002526_acute_inflammatory_response	93	15	-3.75905	0.005306
GO:0006817_phosphate_transport	94	15	-3.705955	0.0066
GO:0006812_cation_transport	807	69	-3.687895	0.006471
GO:0006118_electron_transport	442	43	-3.618797	0.007115
GO:0050880_regulation_of_blood_vessel_size	50	10	-3.447384	0.011321
GO:0002250_adaptive_immune_response	102	15	-3.310601	0.013273
GO:0002460_adaptive_immune_response_based_on_somatic_recombination_of_immune_receptors_built_from_immunoglobulin_superfamily_domains	102	15	-3.310601	0.013273
GO:0019724_B_cell_mediated_immunity	72	12	-3.251905	0.014464
GO:0008207_C21-steroid_hormone_metabolic_process	20	6	-3.243992	0.014737
GO:0000302_response_to_reactive_oxygen_species	53	10	-3.234425	0.014655
GO:0002449_lymphocyte_mediated_immunity	94	14	-3.183696	0.019492
GO:0001944_vasculature_development	276	29	-3.140118	0.022333
GO:0021700_developmental_maturation	140	18	-3.128933	0.022131
GO:0006813_potassium_ion_transport	176	21	-3.11627	0.022581
GO:0048514_blood_vessel_morphogenesis	240	26	-3.068823	0.024127
GO:0009653_anatomical_structure_morphogenesis	1708	124	-3.066722	0.02375
GO:0065008_regulation_of_biological_quality	896	72	-3.0479	0.023846
GO:0019725_cell_homeostasis	443	41	-3.040968	0.023485
GO:0006940_regulation_of_smooth_muscle_contraction	22	6	-3.000958	0.027761
GO:0048469_cell_maturation	121	16	-2.974961	0.028382
GO:0042446_hormone_biosynthetic_process	58	10	-2.915674	0.028986
GO:0042592_homeostatic_process	580	50	-2.890933	0.029
GO:0001568_blood_vessel_development	273	28	-2.890851	0.028592
GO:0006096_glycolysis	59	10	-2.856728	0.030972
GO:0006006_glucose_metabolic_process	137	17	-2.814685	0.034932
GO:0016064_immunoglobulin_mediated_immune_response	70	11	-2.809477	0.034865
GO:0002443_leukocyte_mediated_immunity	104	14	-2.74658	0.044267
GO:0032787_monocarboxylic_acid_metabolic_process	307	30	-2.725161	0.046053
GO:0007160_cell-matrix_adhesion	105	14	-2.706597	0.046753
GO:0030855_epithelial_cell_differentiation	62	10	-2.688448	0.046538
GO:0019318_hexose_metabolic_process	191	21	-2.665573	0.047089
GO:0002438_acute_inflammatory_response_to_antigenic_stimulus	11	4	-2.642618	0.048795
GO:0002524_hypersensitivity	11	4	-2.642618	0.048795
GO:0006942_regulation_of_striated_muscle_contraction	11	4	-2.642618	0.048795
GO:0042312_regulation_of_vasodilation	11	4	-2.642618	0.048795
GO:0006119_oxidative_phosphorylation	107	14	-2.628482	0.049405
GO:0030001_metal_ion_transport	705	57	-2.575828	0.052235
GO:0009266_response_to_temperature_stimulus	97	13	-2.571803	0.05186
GO:0042542_response_to_hydrogen_peroxide	35	7	-2.564738	0.052759
GO:0006631_fatty_acid_metabolic_process	208	22	-2.553902	0.052159
GO:0031033_myosin_filament_assembly_or_disassembly	6	3	-2.521539	0.058352
GO:0055003_cardiac_myofibril_assembly	6	3	-2.521539	0.058352
GO:0055013_cardiac_muscle_cell_development	6	3	-2.521539	0.058352
GO:0030241_muscle_thick_filament_assembly	2	2	-2.511105	0.0829
GO:0032781_positive_regulation_of_ATPase_activity	2	2	-2.511105	0.0829
GO:0046314_phosphocreatine_biosynthetic_process	2	2	-2.511105	0.0829
GO:0060046_regulation_of_acrosome_reaction	2	2	-2.511105	0.0829
GO:0060054_positive_regulation_of_epithelial_cell_proliferation_involved_in_wound_healing	2	2	-2.511105	0.0829
GO:0060055_angiogenesis_involved_in_wound_healing	2	2	-2.511105	0.0829
GO:0060056_mammary_gland_involution	2	2	-2.511105	0.0829
GO:0060057_apotosis_involved_in_mammary_gland_involution	2	2	-2.511105	0.0829
